# Strategy to Develop and Evaluate a Multiplex RT-ddPCR in Response to SARS-CoV-2 Genomic Evolution

**DOI:** 10.3390/cimb43030134

**Published:** 2021-11-06

**Authors:** Laura A. E. Van Poelvoorde, Mathieu Gand, Marie-Alice Fraiture, Sigrid C. J. De Keersmaecker, Bavo Verhaegen, Koenraad Van Hoorde, Ann Brigitte Cay, Nadège Balmelle, Philippe Herman, Nancy Roosens

**Affiliations:** 1 Transversal Activities in Applied Genomics, Sciensano, 1050 Brussels, Belgium; Laura.VanPoelvoorde@sciensano.be (L.A.E.V.P.); Mathieu.Gand@sciensano.be (M.G.); MarieAlice.Fraiture@sciensano.be (M.-A.F.); Sigrid.DeKeersmaecker@sciensano.be (S.C.J.D.K.); 2 Food Pathogens, Sciensano, 1050 Brussels, Belgium; Bavo.Verhaegen@sciensano.be (B.V.); Koenraad.VanHoorde@sciensano.be (K.V.H.); 3 Enzootic, Vector-Borne and Bee Diseases, Sciensano, 1180 Brussels, Belgium; AnnBrigitte.Cay@sciensano.be (A.B.C.); Nadege.Balmelle@sciensano.be (N.B.); 4 Expertise and Service Provision, Sciensano, 1050 Brussels, Belgium; Philippe.Herman@sciensano.be

**Keywords:** droplet digital PCR, COVID-19, SARS-CoV-2, wastewater, respiratory samples, monitoring, next-generation sequencing data

## Abstract

The worldwide emergence and spread of severe acute respiratory syndrome coronavirus 2 (SARS-CoV-2) since 2019 has highlighted the importance of rapid and reliable diagnostic testing to prevent and control the viral transmission. However, inaccurate results may occur due to false negatives (FN) caused by polymorphisms or point mutations related to the virus evolution and compromise the accuracy of the diagnostic tests. Therefore, PCR-based SARS-CoV-2 diagnostics should be evaluated and evolve together with the rapidly increasing number of new variants appearing around the world. However, even by using a large collection of samples, laboratories are not able to test a representative collection of samples that deals with the same level of diversity that is continuously evolving worldwide. In the present study, we proposed a methodology based on an in silico and in vitro analysis. First, we used all information offered by available whole-genome sequencing data for SARS-CoV-2 for the selection of the two PCR assays targeting two different regions in the genome, and to monitor the possible impact of virus evolution on the specificity of the primers and probes of the PCR assays during and after the development of the assays. Besides this first essential in silico evaluation, a minimal set of testing was proposed to generate experimental evidence on the method performance, such as specificity, sensitivity and applicability. Therefore, a duplex reverse-transcription droplet digital PCR (RT-ddPCR) method was evaluated in silico by using 154 489 whole-genome sequences of SARS-CoV-2 strains that were representative for the circulating strains around the world. The RT-ddPCR platform was selected as it presented several advantages to detect and quantify SARS-CoV-2 RNA in clinical samples and wastewater. Next, the assays were successfully experimentally evaluated for their sensitivity and specificity. A preliminary evaluation of the applicability of the developed method was performed using both clinical and wastewater samples.

## 1. Introduction

The ongoing coronavirus disease 2019 (COVID-19) pandemic is caused by the severe acute respiratory syndrome coronavirus 2 (SARS-CoV-2), a positive-sense single-stranded RNA virus. The symptoms of COVID-19 include cough, respiratory problems, fever, aches and pains, fatigue, diarrhea and taste and smell disorders [1]. SARS-CoV-2 can also cause severe complications, including death, mostly in the elderly or in people suffering from comorbidities [2,3]. To monitor the spread of the COVID-19 pandemic and to reduce transmission, many governments have implemented intensive contact tracing, testing and isolation [4,5,6,7].

The gold standard for the detection of SARS-CoV-2 is reverse-transcription quantitative polymerase chain reaction (RT-qPCR) on extracted RNA from nasopharyngeal swabs for individual diagnostics. In order to rationalize the monitoring of the virus spread at the level of a country or region regarding the number of samples, the monitoring of wastewater was also proposed for the surveillance of SARS-CoV-2 [8,9,10]. However, there are several limitations associated with wastewater surveillance; generally, a low virus concentration is observed in such samples, which makes detection challenging. Furthermore, the virus detection and quantification can be limited due to the instability of the genome in wastewater, the low efficiency of virus concentration methods and the lack of sensitive detection assays [8].

Although the estimation of the mutation rate of SARS-CoV-2 is lower compared to other RNA viruses [11], the virus is continuously evolving, leading to the emergence of new variants carrying multiple mutations. Current and potential future variants have the potential to be more transmissible, causing more infections and/or leading to vaccine escape [12,13,14,15]. Therefore, it is important to monitor these variants in order to control the epidemic. Furthermore, the emergence of variants can potentially lead to false negative results. The impact of false negative results due to viral mutations in the target region can be reduced by using multiple targets for the detection of the virus genome, as well as a constant monitoring of the effect of mutations on the performance of the PCR method [16]. In the case of a false negative result, the sample should be sequenced to pinpoint what mutation is causing it, and the primers and probe of the PCR assay need to be adapted. The importance of using an in silico analysis using publicly available sequences to identify potential false negative results has already been stated previously [16]. Of course, as mentioned by Gand et al., an in silico study should be backed up by an in vitro study that validates the design using actual samples [16]. Although RT-qPCR methods are the standard for clinical diagnostics and consequently are often used in wastewater samples due to the availability of these methods, many drawbacks were reported related to the use of this technology. First, the tests are expressed in cycle quantification (Cq). The Cq represents the PCR cycle at which the sample produced a fluorescent signal above the background. These Cq values are laboratory- and instrument-specific and a calibration to a quantitative standard is necessary to determine the absolute virus concentration. Furthermore, Cq values are not directly comparable across assays or technology platforms due to differences in nucleic acid extraction methods, viral targets and other parameters [17], thereby affecting inter-laboratory harmonization in the interpretation of the test results. Finally, RT-qPCR is not adapted for wastewater samples, which often contain inhibitors that might influence the Cq values. This could affect the accuracy of viral quantification [18], which was shown for multiple sample matrices by Whale et al. [19].

Reverse-transcriptase droplet digital PCR (RT-ddPCR), may offer an interesting alternative for the detection and quantification of SARS-CoV-2 RNA [20,21]. Comparable with the RT-qPCR method, a target-specific fluorescent probe coupled with primers is used, which makes the adaptation of the existing RT-qPCR assays straightforward. In a ddPCR, a reaction is emulsified into thousands of nanodroplets, of which a proportion does not contain the template molecule [22]. The nanodroplets are used as unique and small bioreactors to amplify the template [23,24,25,26]. At the end-point, the number of positive droplets are digitally counted relative to the total number of droplets. Furthermore, their known volume while flowing through microfluidic devices allows absolute target quantification using Poisson statistics [27,28], which enables an easier comparison between different laboratories and tests compared to RT-qPCR. To the best of our knowledge, eight RT-ddPCR methods designed to detect SARS-CoV-2 were published, of which two are commercial kits designed by BioRad [21,29,30,31,32,33,34,35]. The performance of these methods was tested using reference standards, and four of the methods were tested on clinical samples of infected patient’s throat and nasopharyngeal samples. Three of these methods were tested on wastewater samples [32,33,34]. Moreover, four of these RT-ddPCR methods were tested on respiratory samples [21,30,31,35], and in some cases were found to be positive compared to the negative RT-qPCR results [21,30]. Additionally, the sensitivity of the RT-ddPCR methods for the detection of SARS-CoV-2 has been described previously as comparable or even higher compared to RT-qPCR methods [21,29,30,35]. Therefore, in the case of a low virus concentration, this technology can be interesting to use. Furthermore, inhibition can be encountered in some matrices, such as wastewater. RT-ddPCR separates DNA, inhibitors and reagents in droplets and is an end-point measurement, only measuring after the PCR amplification. Consequently, a reduction in the biases linked to the inhibitors are often observed in RT-ddPCR [36], which makes RT-ddPCR an interesting method for wastewater surveillance. In this study, we propose a methodology using an in silico and in vitro analysis. First, available whole-genome sequencing data for SARS-CoV-2 was used to select primers and probes for PCR assays, as well as to evaluate and monitor the possible impact of virus evolution on the developed PCR assays. Second, a minimal set of in vitro testing was proposed to validate in-house a new duplex RT-ddPCR method specific for the detection of SARS-CoV-2, including specificity and sensitivity assessments. Additionally, the applicability of the proposed RT-ddPCR method was investigated using clinical and wastewater samples. The duplex RT-ddPCR method was developed based on the RT-qPCR methods previously developed by Institute Pasteur [37] and Lu et al. [38].

## 2. Materials and Methods

### 2.1. Selection and Evaluation of Key Target for PCR Detection of SARS-CoV-2 Using WGS Data

For the development of the RT-ddPCR method, two sets of primers and probe were selected from publicly available RT-qPCR assays, namely RdRp_IP4 assay from Institut Pasteur (Paris) [37], and the ORF1a assay from Lu et al., 2020 [38], that target two separate locations specific to the SARS-CoV-2 genome (Table 1). These assays were evaluated in silico [16] for their inclusivity and exclusivity in a previous study in May 2020, which determined the RdRp_IP4 assay [37], S assay from Chan et al., 2020 [39] and ORF1a assay [38] as the most specific and stable assays over time. However, due to the emergence of the B.1.351 lineage in South Africa, a mismatch located in the probe sequence of the S assay was identified, which could lead to a lower sensitivity [40]. Therefore, from the three previously described, only the ORF1a and RdRp_IP4 assays were retained in this study.

The in silico inclusivity of ORF1a and RdRp_IP4 assays was evaluated using the bioinformatics tool SCREENED v1.0 [41], previously used for in silico SARS-CoV-2 assay assessment [16,40], and recent whole-genome SARS-CoV-2 sequences. A total of 296 187 SARS-CoV-2 genomes, obtained from samples collected between 1 November 2020 and 28 February 2021, were obtained from the GISAID database [42] on 7 March 2021. Only complete genomes with high coverage for which the collection date was available were selected, and genomes with low coverage were excluded. Additionally, genomes containing undetermined nucleotides “N” and degenerate nucleotides were excluded from the dataset to retain only high-quality genomes (154 489 genomes) (Supplementary Files S1 and S2). These genomes were divided per month according to their collection date (November: 13 678 genomes; December: 41 128 genomes; January: 58 484 genomes; February: 41 199 genomes). From these datasets, SCREENED performed a two-step BLAST approach to find in each genome the complete amplicon sequence targeted by the ORF1a and RdRp_IP4 primers and probe sets, and subsequently produced mismatch statistics from the hybridization between the nucleotides of the primers and probes and their corresponding annealing sites in the amplicon. Based on these mismatch scores, SCREENED considered that a theoretical positive RT-ddPCR signal was produced if no mismatch in the first five nucleotides of the 3′ end of the primers was reported, if the total number of reported mismatches did not exceed 10% of the oligonucleotide length and if at least 90% of the oligonucleotide sequence aligned correctly to their targets. For the primers and probes evaluated here, this resulted in no more than one or two mismatches being tolerated. These criteria were selected because it has been previously reported that two or more mismatches can lead to potential total test failure, especially if located at the 3′ end [43,44]. Two mismatches or less can result in potential loss of sensitivity but is less likely to lead to total test failure. For each analyzed SARS-CoV-2 genome, a negative SCREENED detection signal was considered as a theoretical FN result, which was used for the in silico inclusivity evaluation (Equation (1)):

Inclusivity (%) = (1 − (Number of FN/Total Number of high quality SARS-CoV-2 genomes)) × 100(1)

FASTA files for November, December, January and February containing 13 678, 41 128, 58 484 and 41 199 SARS-CoV-2 genomes, respectively (Accession ID: Supplementary File S1), and a tab-delimited text file (Supplementary File S3), containing the primer and probe sequences and their corresponding amplicon sequence to be mined in the genomes, were used as input for SCREENED.

### 2.2. Development of RT-ddPCR Method for the Detection of SARS-CoV-2

The RT-ddPCR assay was evaluated using purified RNA from the SARS-CoV-2 virus (Vircell, Granada, Spain–MBC137-R). The RT-ddPCR was performed using the One-Step RT-ddPCR Advanced Kit for Probes (Bio-Rad, Hercules, CA, USA). All the components from the kit were thawed on ice for 30 min and thoroughly mixed by vortexing each tube at maximum speed for 30 s. The reagents were made into larger master mixes and then aliquoted into individual reactions. Each reaction had a total volume of 22 µL that was set up on ice, including 0.99 µL of each primer with an initial concentration of 20 µM and 0.55 µL of each probe with an initial concentration of 10 µM, 1.1 µL of 300 mM DTT, 0.14 µL of dH_2_O, 2.2 µL Reverse Transcriptase, 5.5 µL One-Step Supermix and 8 µL of sample. The primers were obtained from Eurogentec (Seraing, Belgium), while the ZEN probes were supplied by Integrated DNA Technologies (Coralville, IA, USA). According to the manufacturer’s instructions, 20 µL of the reaction mix and 70 µL of Droplet Generation Oil for Probes were loaded into a QX200TM droplet generator (Bio-Rad) and to increase the number of droplets, the cartridge was kept for two minutes at room temperature. After the droplet generation, 40 µL of droplets were recovered per reaction. The amplification was performed in a T100TM Thermal Cycler (Bio-Rad) with the following conditions: one cycle at 25 °C for 3 min, one cycle at 50 °C for 60 min (RT), one cycle at 95 °C for 10 min (Taq polymerase activation); 40 cycles at 95 °C for 30 s (denaturation), 55 °C for 60 s (annealing); one cycle at 98 °C for 10 min (enzyme inactivation); and finally one cycle at 4 °C for 30 min (stabilization). Next, the plate was transferred to the QX200 reader (Bio-Rad) and the results were acquired using the HEX and FAM channel, according to the manufacturer’s instructions. The QuantaSoft software v1.7.4.0917 (Bio-Rad) was used for the interpretation of the results and the threshold was set manually.

### 2.3. Validation of the Specificity of the RT-ddPCR Assay for SARS-CoV-2

The specificity of the method was experimentally established using a set of DNA and RNA controls from Bacillus subtilis Si0005 (Sciensano collection, Brussels, Belgium), Escherichia coli LMG 2092T (BCCM collection, Brussels, Belgium), Aspergillus acidus IHEM 26285 (BCCM collection), Candida cylindracea MUCL 041387 (BCCM collection) and Zea mays (ERM-BF413ak). These were extracted as described in Fraiture et al., 2020 [45]. Additionally, Homo sapiens (Promega, G3041) and viruses including SARS-CoV (Vircell, MBC136-R), MERS-CoV (Vircell, MBC132), influenza H1N1 (Vircell, MBC082), influenza H3 (Vircell, MBC029), influenza B (Vircell, MBC030), adenovirus (Vircell, MBC001), enterovirus D68 (Vircell, MBC125), norovirus (Vircell, MBC111), respiratory syncytial virus A (RSV A) (Vircell, MBC041), rhinovirus (Vircell, MBC091), rotavirus (Vircell, MBC026), coronavirus OC43 (Vircell, MBC135-R) and coronavirus 229E (Vircell, MBC090) were used. The SARS-CoV-2 RNA (Vircell, MBC137-R) was used as a positive control. Each material was tested in duplicate and included 200 copies/µL for the viruses, while the bacterial, fungal, plant and human DNA contained 2 ng/µL.

### 2.4. Validation of Sensitivity of the RT-ddPCR Assay for SARS-CoV-2

The evaluation of the sensitivity was carried out using serial dilutions of purified RNA from the SARS-CoV-2 virus. Seven serial dilutions were prepared, ranging from 0.5 to 200 copies/µL, and each dilution was tested in 12 replicates. The limit of detection (LOD95%) was calculated using the web application Quodata with the number of copies of the target that is required to ensure a probability of detection (POD) of 95% [46].

### 2.5. Applicability Assessment

To assess the applicability of this RT-ddPCR assay on non-artificial samples, five samples collected from patients showing clinical signs of COVID-19 were collected. From these five samples, three samples (clinical samples 1, 2, 3) previously tested positive for SARS-CoV-2 with RT-qPCR, with a high, moderate and low Cq, while two tested negative for SARS-CoV-2 (clinical samples 4, 5) (Supplementary File S4). The clinical samples were obtained from a biobank (allowed by the Biobank compendium of the Federaal Agentschap voor Geneesmiddelen en Gezondheidsproducten [47]). All experiments were performed in accordance with relevant guidelines and regulations. In addition, three wastewater samples (wastewater samples 1, 2, 3) were included that also previously tested positive for the SARS-CoV-2 virus with RT-qPCR, with a high, moderate and low Cq (see Supplementary File S4). Due to the high concentration of clinical sample 3, the sample was diluted 80 times. Consequently, 0.1 µL of sample and 7.9 µL of dH_2_O were used in the reaction (dilution: 80×).

## 3. Results

### 3.1. In Silico Inclusivity Evaluation for the ORF1a and RdRp_IP4 Assays Using SCREENED

The ORF1a and RdRp_IP4 assays were evaluated for their inclusivity with four datasets corresponding to the months November 2020, December 2020, January 2021 and February 2021 (Table 2) using 13 678, 41 128, 58 484 and 41 199 SARS-CoV-2 genomes, respectively. Both for the ORF1a and RdRp_IP4 assays, excellent inclusivity was obtained for the four datasets, because all assays had an inclusivity of more than 99.5%. The little variation observed between the months can mainly be attributed to random and rare mutation events that did not spread in the viral population.

In addition, it was verified that when an FN result was obtained for a given genome, this was limited to either only the forward or reverse primer or the probe. Moreover, if an FN result was obtained for a genome for one of the assays, a positive signal was obtained for the other assay. Consequently, the inclusivity of the multiplex method using the combination of the ORF1a assay and RdRp_IP4 assay is 100%.

Finally, the dataset included 33 611 and 293 genomes belonging to the B.1.1.7 and B.1.351 lineage, respectively. The number of FN that were attributed to these lineages was limited to 0, 1, 6 and 7 for ORF1a assay and 0, 1, 22 and 21 for the RdRP_IP4 assay on a total of 18, 103, 7291 and 26 199 genomes belonging to the B.1.1.7 lineage in the months November 2020, December 2020, January 2021 and February 2021, respectively. For the B.1.351 lineage, the number of FN was limited to 0 for the ORF1a assay and 0, 1 and 0 for the RdRP_IP4 assay on a total of 21, 138 and 134 genomes belonging to B.1.1.7 in the months December 2020, January 2021 and February 2021. No genomes belonging to the B.1.351 lineage were included from the month November 2020.

### 3.2. Specificity Assessment

The specificity of the RT-ddPCR method was experimentally tested for each positive and negative material (Table 3). SARS-CoV-2 RNA was used as a positive control, while four closely related coronaviruses, 10 other viruses, human DNA, plant (Zea mays), two bacteria and two fungi were used as negative controls. Excellent exclusivity was observed because no amplification was observed for all negative controls, while the positive control presented an amplification (Table 3).

### 3.3. Sensitivity Assessment

The sensitivity of the designed RT-ddPCR method was tested using SARS-CoV-2 RNA with different estimated target copy numbers, namely 200, 50, 25, 10, 5, 1, 0.5 and 0 copies/µL. An amplification for all 12 replicates was observed until five estimated target copies/µL (Table 4). The LOD_95%_ for the ORF1a assay was determined at 4.57 [2.74,7.61] estimated target copies/µL, while the RdRp_IP4 assay proved to be more sensitive with a LOD_95%_ of 1.59 [0.95,2.67] estimated target copies/µL. Notably, in 4/12 and 9/12 replicates for the ORF1a assay and RdRp_IP4 assay, respectively, it also tested positive for samples with an estimation of 0.5 and 1 copies/µL (Table 4, Supplementary Files S5 and S6).

### 3.4. Applicability Assessment

The presence and quantity of SARS-CoV-2 was investigated in five clinical (nasopharyngeal swabs) and three wastewater samples. Among the five clinical samples, three samples tested positive for both the ORF1a and RdRp_IP4 assay (Table 5). The three wastewater samples also tested positive for SARS-CoV-2 (Table 5). These detection results corresponded to their previous results obtained with RT-qPCR, where wastewater sample 1 and clinical sample 1 had the lowest concentration, while wastewater sample 3 and clinical sample 3 had the highest concentration. The detailed results of the RT-ddPCR method on the clinical and wastewater samples are presented in Table 5 and Supplementary File S7.

## 4. Discussion

Using a total of 154 489 SARS-CoV-2 high-quality genomes, two simplex RT-qPCR assays that were designed previously to target the conserved regions of ORF1a and RdRp genes were selected for the development of a novel RT-ddPCR multiplex assay for the detection and quantification of SARS-CoV-2. The main advantage of targeting two regions is to anticipate FN results that could occur due to mutations that lead to possible mispriming of the primer and/or probe, and consequently to a lack of viral detection. Indeed, FN results have been reported previously in clinical samples due to the genetic evolution of the virus [40,48,49]. The use of multiple targets for the detection of the viral genome [50,51,52] can reduce the impact of FN results related to viral mutations in the region of the annealing of the primers and/or probe. The failure of one region can be compensated for by the detection of the other, as was shown in this study for the in silico evaluation. Evidently, in the case of a false negative result for one of the targets, further investigation is necessary to identify the mutation causing the false negative result by sequencing the sample. Furthermore, the primers and probe should then be adapted to minimize the impact on the test.

During the development of any new method for pathogen detection, it is of utmost importance to carefully assess its specificity, i.e., inclusivity and exclusivity. For inclusivity, a large number of various strains belonging to the targeted organism should ideally be tested. However, in the case of SARS-CoV-2, it is difficult to obtain a representative collection of all the circulating strains, and to test it experimentally. To overcome this issue, the specificity evaluation can be carried out in silico using bioinformatics and the large number of SARS-CoV-2 high-quality sequences publicly available, as previously performed for ORF1a and RdRp_IP4 assays [37,38]. Moreover, after development, the detection assays need to be under constant monitoring over time, because the virus evolves and a mutation could be introduced within these targets. Currently, several new SARS-CoV-2 variants have emerged, carrying an unusually high number of mutations, and assessing all assays for FN is important. Therefore, in the present study, the latest WGS published data of SARS-CoV-2 (154 489 high-quality whole-genome sequences) were used to perform an in silico analysis of ORF1a and RdRp_IP4 assays, which both showed excellent results, i.e., an inclusivity of more than 99.5% from the beginning of November 2020 to the end of February 2021. Hence, no new mutations impacted the inclusivity, including the mutations linked to the variants of concern that emerged at the end of 2020. Most of the primers and probe sets used in other multi-target RT-ddPCR assays developed for SARS-CoV-2 detection [21,29,31,33,35] have also been previously analyzed for their inclusivity using the same in silico approach [16,40]. Most of these sets showed excellent inclusivity results (>99%), except for the primers and probe set targeting the gene N (June-December 2020: 63.89% inclusivity) used in Kinloch et al. and Suo et al., and initially designed by the China CDC [16,40]. Therefore, the N target used in these assays should preferably not be chosen for developing SARS-CoV-2 detection methods. Concerning the exclusivity, this one has also been previously evaluated in silico for ORF1a and RdRp_IP4 assays successfully, with thousands of non-SARS-CoV-2 genomes [16]. Additionally, following the earlier in silico specificity assessment, a minimal experimental set-up was designed to evaluate the performance of the developed method. First, using a set of DNA and RNA references, the exclusivity of ORF1a and RdRp_IP4 assays was successfully confirmed, with no false positives detected for other viral, bacterial, plant and human RNA and DNA, including closely related viruses such as SARS-CoV, MERS-CoV and coronavirus OC43. This result was expected based on the in silico analysis using the primer and probes selected in the present study performed by Gand et al. [16], where a 100% exclusivity was observed for these two assays, including closely related viruses. In contrast, the specificity of most other RT-ddPCR methods currently published were not experimentally evaluated using non-target DNA, such as that from bacteria [21,29,31,33,35].

Secondly, the sensitivity of our method was estimated at 4.6 and 1.6 estimated target copies/µL (LOD95%) for the ORF1a and RdRp_IP4 assays, respectively. This means that false negative results can possibly occur in the case of samples with a lower viral load than the LOD95%; however, positive results are still possible, as observed in both the sensitivity and applicability assessment. Although other targets were used by most other previously published RT-ddPCR methods, similar LODs were observed [53]. When comparing the LOD to RT-qPCR methods, the RdRp_IP4 assay using RT-ddPCR was found to be more sensitive compared to using RT-qPCR for the same target, with LOD95% of 7.9 estimated copies/µL [53]. Information on the LOD of RT-qPCR could not be found in the literature for the ORF1a assay. In Suo et al. [21], it was demonstrated that negative RT-qPCR results could be identified as positive when repeating the analysis with the optimized RT-ddPCR targeting the ORF1ab and N gene. In Alteri et al. [30], Deiana et al. [31], de Kock et al. [29] and Kinloch et al. [35], targeting the RdRP gene, ORF gene, E gene and N gene, the RT-ddPCR assay was found to be more sensitive than the RT-qPCR assay. Therefore, we expect that this RT-ddPCR assay would be at least as sensitive or even more sensitive [21,29,35] compared to RT-qPCR. In this study, no comparison could be made between the RT-qPCR methods used to characterize the clinical and wastewater samples (Supplementary File S4) and the developed ddPCR method, because different primers and probes were used.

In addition, a preliminary assessment of the applicability of the method was performed on RNA extracted from nasopharyngeal swabs and wastewater samples. The samples were selected on the basis of their different target concentrations, according to Cq values (low, medium and high) previously obtained by RT-qPCR (reflecting, respectively, high, medium and low contamination levels), and their different origins. The main goal of this experimental design was to evaluate a potential matrices effect on the PCR results using a minimum number of samples. The positive results obtained in low Cq samples using our newly developed RT-ddPCR method suggest a sensitivity of at least as high as the RT-qPCR assays used for these samples. Although the price of the RT-ddPCR method was calculated at approximately EUR 6.5 per sample, which is indeed more expensive compared to most RT-qPCR methods, RT-ddPCR reduces the work in the case of absolute quantification. One of the advantages of using RT-ddPCR instead of RT-qPCR is also the absolute quantification of the viral RNA without calibration, which enables comparison between different assays and laboratories without the necessity of a standard curve. Additionally, the accuracy of the RT-ddPCR methods should be less influenced by the inhibitors that are often present in wastewater samples. However, there are some drawbacks to RT-ddPCR, such as the longer turnaround time of the RT-ddPCR compared to RT-qPCR. Moreover, clinical samples may contain a high virus concentration that would need to be diluted in the RT-ddPCR method. The possible repetition of the detection of the samples that need to be diluted takes more time and makes the RT-ddPCR method a less appropriate method for routine surveillance. However, the virus concentration in wastewater samples is often low, making dilution often unnecessary. Moreover, the lower impact of inhibition on the RT-ddPCR method makes it an appropriate method for wastewater surveillance. Due to its absolute quantification, the RT-ddPCR method can also be used to evaluate the performances in different laboratories for the inter-laboratory reproducibility and cross-validation of the methods. Because of its potential higher sensitivity, it could also complement the current RT-qPCR diagnostics to improve the rapid identification of SARS-CoV-2 infections, by detecting the virus before the virus concentration peak is reached and antibodies appear in a diagnostic sample.

In addition to the successful development and validation of the proposed multiplex RT-ddPCR method, a methodology to systematically evaluate and monitor PCR-based methods targeting evolving viruses such as SARS-CoV-2 is provided in this manuscript. This methodology includes a method performance assessment in terms of specificity (*in silico* and experimentally tested), sensitivity and applicability. The main added value of this methodology is related to the first in silico inclusivity assessment step, using a large set of SARS-CoV-2 strain with a high level of diversity, which is not experimentally achievable by collecting samples and testing them. Indeed, even by testing a large collection of samples, laboratories are not able to test a representative collection of samples that deals with this diversity that is continuously evolving and that needs to be seen not only locally but worldwide. Therefore, we believe that at the present time, this first in silico inclusivity assessment step is essential for the development and validation of PCR-based methods targeting the virus, as well as for its continuous evaluation using the newest available WGS data, which are generated over time. Moreover, an additional added value of this methodology is related to the essential experimental testing. Indeed, for the sake of efficiency and simplicity, it should be designed to use a minimal number of critical samples (as proposed in the present study) to assess the performance of the methods (specificity, sensitivity, applicability).

## Figures and Tables

**Table 1:** Primer and probe sets included in the multiplex RT-ddPCR assay.

Primer/Probe	5′→ 3′ Sequence	Target	Nucleotide Position	Concentration	Ref.
ORF1a-F	AGAAGATTGGTTAGATGATGATAGT	ORF1a	3193–3217	0.9 µM	[37]
ORF1a-R	TTCCATCTCTAATTGAGGTTGAACC		3286–3310	0.9 µM	
ORF1a-P	5′6-FAM/TCCTCACTG-ZEN-CCGTCTTGTTGACCA-3′IABkFQ		3229–3252	0.25 µM	
RdRp_IP4-F	GGTAACTGGTATGATTTCG	RdRp gene	14,080–14,098	0.9 µM	[38]
RdRp_IP4-R	CTGGTCAAGGTTAATATAGG		14,167–14,186	0.9 µM	
RdRp_IP4-P	5′HEX-TCATACAAA-ZEN-CCACGCCAGG-3′IABkFQ		14,105–14,123	0.25 µM	

A second, internal ZEN quencher was added to the probes to obtain greater overall dye quenching in addition to the Iowa Black FQ (IABkFQ) quencher. The indicated positions refer to the reference sequence NC_045512.

**Table 2:** Inclusivity in silico evaluation of ORF1a and RdRp_IP4 assays obtained with SCREENED.

Month	Number of Genomes	Assay	FN	Inclusivity
November	13 678	RdRp_IP4	20	99.85%
		ORF1a	17	99.88%
December	41 128	RdRp_IP4	21	99.95%
		ORF1a	95	99.77%
January	58 484	RdRp_IP4	52	99.91%
		ORF1a	67	99.89%
February	41 199	RdRp_IP4	31	99.92%
		ORF1a	28	99.93%

The number of genomes that were used in SCREENED are indicated per month. Additionally, the number of false negative results and the inclusivity are included per assay per month. FN = false negative.

**Table 3:** Specificity assessment of the developed RT-ddPCR method.

**Kingdom**	**Genus**	**Species**	**Strain Number**	**RT-ddPCR**
Animalia	Homo	sapiens	/	-
Plantae	Zea	mays	/	-
Bacteria	Bacillus	subtilis	SI0005	-
	Escherichia	coli	MB1068	-
Fungi	Aspergillus	acidus	26,285	-
	Candida	cylindracea	041387	-
	**Family**		**Species**	**RT-ddPCR**
Viruses	Picornaviridae		Rhinovirus B	-
	Reoviridae		Rotavirus	-
	Orthomyxoviridae		Influenza A (H1N1)	-
	Orthomyxoviridae		Influenza A (H3)	-
	Orthomyxoviridae		Influenza B	-
	Adenoviridae		Adenovirus	-
	Picornaviridae		Enterovirus D68	-
	Caliciviridae		Norovirus	-
	Pneumoviridae		RSV A	-
	Coronaviridae		SARS-CoV	-
	Coronaviridae		MERS-CoV	-
	Coronaviridae		Corona OC43	-
	Coronaviridae		Coronavirus control	-
	Coronaviridae		SARS-CoV-2	+

The absence and presence of amplification is symbolized by a - or +, respectively. The RT-ddPCR method was performed in duplicate on each sample. SARS-CoV-2 RNA was included as a positive control.

**Table 4:** Sensitivity assessments of the developed RT-ddPCR method.

Estimated Target Copy Number	Sensitivity Assessment (ORF1a)	Sensitivity Assessment (RdRp_IP4)
200 copies/µL	+ 12/12 117.59 ± 7.68 copies/µL	+ 12/12 138.46 ± 8.44 copies/µL
50 copies/µL	+ 12/12 25.53 ± 8.02 copies/µL	+ 12/12 27.98 ± 7.82 copies/µL
25 copies/µL	+ 12/12 10.95 ± 2.37 copies/µL	+ 12/12 12.54 ± 1.95 copies/µL
10 copies/µL	+ 12/12 4.45 ± 0.82 copies/µL	+ 12/12 4.70 ± 1.06 copies/µL
5 copies/µL	+ 12/12 1.82 ± 0.66 copies/µL	+ 12/12 2.20 ± 0.90 copies/µL
1 copies/µL	+ 4/12 0.11 ± 0.16 copies/µL	+ 9/12 0.37 ± 0.29 copies/µL
0.5 copies/µL	+ 4/12 0.19 ± 0.31 copies/µL	+ 9/12 0.48 ± 0.44 copies/µL
0 copies/µL	- 0/12	- 0/12

The absence and presence of amplification are indicated by - or +, respectively. For each estimated target copy number, 12 replicates were tested and the number of positive replicates is indicated at the middle line of each box. In addition, the average of the observed copies/µL (± the standard deviation, as obtained with the RT-ddPCR measurement, is indicated at the lower line.

**Table 5:** SARS-CoV-2 investigation in clinical samples and wastewater samples.

Sample	SARS-CoV-2 (ORF1a)	SARS-CoV-2 (RdRp_IP4)	RT-qPCR
Wastewater sample 1	+ 2.48 copies/µL	+ 1.93 copies/µL	+
Wastewater sample 2	+ 6.33 copies/µL	+ 2.20 copies/µL	+
Wastewater sample 3	+ 29.43 copies/µL	+ 36.29 copies/µL	+
Clinical sample 1	+ 2.75 copies/µL	+ 2.75 copies/µL	+
Clinical sample 2	+ 26.13 copies/µL	+ 32.18 copies/µL	+
Clinical sample 3	+ 88,440 copies/µL	+ 91,080 copies/µL	+
Clinical sample 4	-	-	-
Clinical sample 5	-	-	-

The sample name and the kind of sample are given in addition to the results of the detection of SARS-CoV-2 using the ORF1a assay and the RdRp_IP4 assay. The presence or absence of PCR amplification is symbolized by + or -, respectively. For each RT-ddPCR, the observed copies/µL is given. Detailed results from the RT-qPCR can be found in Supplementary File S4.

## Data Availability

The GISAID database (https://www.gisaid.org/) was used for this study on 7 of March 2021. The accession numbers of the used data are available in Supplementary File S1.

## Supplementary Materials

The following are available online at https://www.mdpi.com/article/10.3390/cimb43030134/s1, Supplementary File S1: GISAID metadata, Supplementary File S2: Acknowledgments from the GISAID database, Supplementary File S3: SCREENED input, Supplementary File S4: Table with SARS-CoV-2 investigation in clinical samples and wastewater samples, including Cq values from a previous test and the materials and methods used to determine the Cq, Supplementary File S5: Calculation of LOD95% on the POD curve for the sensitivity of the developed RT-ddPCR method for the ORF1a assay and RdRp_IP4 assay, Supplementary File S6: RT-ddPCR plots of the sensitivity assessment, Supplementary File S7: RT-ddPCR plots of the applicability assessment for the wastewater samples and clinical samples.
