# Supplementary material for: Strategy to Develop and Evaluate a Multiplex RT-ddPCR in Response to SARS-CoV-2 Genomic Evolution
Source: Curr Issues Mol Biol. 2021 Nov 6;43(3):1937–49. doi: 10.3390/cimb43030134 (PMC8928932; doi:10.3390/cimb43030134)
Supplement: Supplementary file 1 [file Supplementary File S2 - Acknowledgment Table.pdf › Supplementary File S2 - Acknowledgment Table.pdf]

We gratefully acknowledge the following Authors from the Originating laboratories responsible for obtaining the specimens, as well as the Submitting laboratories where the genome data were generated and shared via GISAID, on which this research is based.

All Submitters of data may be contacted directly via www.gisaid.org

Authors are sorted alphabetically.

Accession ID	Originating Laboratory	Submitting Laboratory	Authors
EPI_ISL_1005008, EPI_ISL_1005011, EPI_ISL_1005107	Viollier AG	Department of Biosystems Science and Engineering, ETH Zürich	Chaoran Chen, Sarah Nadeau, Catharine Aquino, Ivan Topolsky, Philipp Jablonski, Lara Fuhrmann, David Dreifuss, Katharina Jahn, Andreia Cabral de Gouvea, Maria Domenica Moccia, Simon Grüter, Timothy Sykes, Lennart Opitz, Griffin White, Laura Neff, Doris Popovic, Andrea Patrignani, Jay Tracy, Ralph Schlapbach, Christiane Beckmann, Maurice Redondo, Olivier Kobel, Christoph Noppen, Sophie Seidel, Noemie Santamaria de Souza, Niko Beerenwinkel, Tanja Stadler
EPI_ISL_1005559, EPI_ISL_1005568, EPI_ISL_1005569, EPI_ISL_1005658, EPI_ISL_1005660	Instituto Nacional de Medicina Genomica	Instituto Nacional de Medicina Genomica	Hidalgo-Miranda A, Mendoza-Vargas A, Reyes-Grajeda JP, Cisneros-Villanueva M, Cedro-Tanda A,Peñaloza-Figueroa F, Herrera-Montalvo LA
EPI_ISL_1007678, EPI_ISL_1007679	Department of Internal Medicine, College of Medicine, Chosun University	Department of Internal Medicine, College of Medicine, Chosun University	Dong-Min Kim
EPI_ISL_1007700, EPI_ISL_1007702, EPI_ISL_1007705, EPI_ISL_1007706, EPI_ISL_1007708, EPI_ISL_1007711	Hospital Universitario Marqués de Valdecilla - IDIVAL (Santander, Cantabria)	SeqCOVID-SPAIN consortium/IBV(CSIC)	Mónica Gozalo Margüello, María Eliecer Cano García, Jose Manuel Méndez Legaza, Daniel Pablo Marcos, Jesús Rodríguez Rodríguez, María Siller Ruiz and SeqCOVID-SPAIN consortium
EPI_ISL_1008426, EPI_ISL_1008427, EPI_ISL_1008428, EPI_ISL_1008429, EPI_ISL_1008430, EPI_ISL_1008431	Klinisk mikrobiologi	The Public Health Agency of Sweden	Anna-Malin Linde, Maria Lind Karlberg, Carlo Berg, Oskar Karlsson Lindsjo, Sofia Stamouli, Reza Advani, Mattias Haukland, Petra Holmstrom, Noura Walai, Petra Edquist, Mia Brytting, Anna Risberg, Karin Tegmark-Wisell
EPI_ISL_1008552	Klinisk Mikrobiologi	The Public Health Agency of Sweden	Anna-Malin Linde, Maria Lind Karlberg, Carlo Berg, Oskar Karlsson Lindsjo, Sofia Stamouli, Reza Advani, Mattias Haukland, Petra Holmstrom, Noura Walai, Petra Edquist, Mia Brytting, Anna Risberg, Karin Tegmark-Wisell
EPI_ISL_1009168, EPI_ISL_1009177, EPI_ISL_1009179, EPI_ISL_1009199	American Esoteric Laboratories	Colleen B. Jonsson	Mariah K. Taylor, Walter Reichard, Jyothi Parvathareddy, Colleen B. Jonsson
EPI_ISL_1010735	University of Iowa Hospitals & Clinics, Microbiology Laboratory	University or Iowa, Lung Biology and Cystic Fibrosis Research Center, Pezzulo Lab	Miguel E Ortiz, Alejandro A Pezzulo
EPI_ISL_1011534	Massachusetts General Hospital	Infectious Disease Program, Broad Institute of Harvard and MIT	Lemieux,J.E., Siddle,K.J., Shaw,B., Adams,G., Pierce,V., Turbett,S., Anahtar,M., Branda,J., Slater,D., Harris,J., Lin,A.E., Gladden-Young,A., Lagerborg,K., Rudy,M., DeRuff,K., Carter,A., Normandin,E., Bauer,M., Reilly,S., Tomkins-Tinch,C., Loreth,C., Chaluvadi,S., Neumann,A., Cusick,C., Chapman,S.B., Gnirke,A., Flowers,K., Cerrato,F., Birren,B.W., Gallagher,G., Smole,S., Park,D.J., MacInnis,B.L., Ryan,E., LaRocque,R., Rosenberg,E. and Sabeti,P.C.
EPI_ISL_1014451, EPI_ISL_1014453, EPI_ISL_1014454, EPI_ISL_1014455, EPI_ISL_1014456, EPI_ISL_1014458, EPI_ISL_1014459, EPI_ISL_1014465, EPI_ISL_1014466, EPI_ISL_1014467, EPI_ISL_1014468, EPI_ISL_1014469, EPI_ISL_1014572, EPI_ISL_1014573, EPI_ISL_1014574, EPI_ISL_1014575, EPI_ISL_1014576, EPI_ISL_1014577			
see above	Dutch COVID-19 response team	National Institute for Public Health and the Environment (RIVM)	Adam Meijer, Harry Vennema, Dirk Eggink, Jeroen Cremer, Sharon van den Brink, Bas van der Veer, AnneMarie van den Brandt, Florian Zwagemaker, Dennis Schmitz, Chantal Reusken, on behalf of the national COVID-19 response team
EPI_ISL_1015203, EPI_ISL_1015312, EPI_ISL_1015313, EPI_ISL_1015315, EPI_ISL_1015317, EPI_ISL_1015323	Department of Virology, Pitié-Salpêtrière hospital	Department of Virology, Pitié-Salpêtrière hospital	Valentin Leducq, Aude Jary, Karen Zafilaza, Stéphane Marot, Vincent Calvez, Anne-Geneviève Marcelin
EPI_ISL_1015754	Seattle Flu Study	Seattle Flu Study	Deborah A. Nickerson, Chris D. Frazar, Jover Lee, Benjamin Pelle, Erica Ryke, Matthew Richardson, Amanda Adler, Elisabeth Brandstetter, Peter D. Han, Kairsten Fay, Misja Ilcisin, Kirsten Lacombe, Thomas R. Sibley, Melissa Truong, Caitlin R. Wolf, Karen Cowgill, Stephanie Schrag, Jeff Duchin, Michael Boeckh, Janet A. Englund, Michael Famulare, Barry R. Lutz, Mark J. Rieder, Lea M. Starita, Matthew Thompson, Helen Y. Chu, Trevor Bedford, Jay Shendure
EPI_ISL_1016507	University of Miami Immunology and Histocompatibility Laboratory	University of Miami Immunology and Histocompatibility Laboratory	Emilio Margolles-Clark, PhD and Phillip Ruiz, MD, PhD
EPI_ISL_1016954, EPI_ISL_1016955, EPI_ISL_1016956, EPI_ISL_1016962	UNMC Emerging Pathogens Laboratory	UNMC COVID-19 Response Team	UNMC COVID-19 Response Team
EPI_ISL_1017053	Providence Oregon Regional Laboratories	Providence St. Joseph Health Molecular Genomics Laboratory	Alexa K Dowdell, Brian D Piening, Fred L Robinson, Carlo B Bifulco, Mary Campbell
EPI_ISL_1017066	Providence Alaska Medical Center	Providence St. Joseph Health Molecular Genomics Laboratory	Alexa K Dowdell, Brian D Piening, Fred L Robinson, Carlo B Bifulco, Mary Campbell
EPI_ISL_1017069, EPI_ISL_1017173, EPI_ISL_1017174, EPI_ISL_1017175, EPI_ISL_1017176, EPI_ISL_1017177, EPI_ISL_1017178, EPI_ISL_1017179	Providence Oregon Regional Laboratories	Providence St. Joseph Health Molecular Genomics Laboratory	Alexa K Dowdell, Brian D Piening, Fred L Robinson, Carlo B Bifulco, Mary Campbell
EPI_ISL_1017180	Providence Regional Medical Center Everett	Providence St. Joseph Health Molecular Genomics Laboratory	Alexa K Dowdell, Brian D Piening, Fred L Robinson, Carlo B Bifulco, Mary Campbell
EPI_ISL_1017181, EPI_ISL_1017182, EPI_ISL_1017183, EPI_ISL_1017184, EPI_ISL_1017185, EPI_ISL_1017186	Providence Oregon Regional Laboratories	Providence St. Joseph Health Molecular Genomics Laboratory	Alexa K Dowdell, Brian D Piening, Fred L Robinson, Carlo B Bifulco, Mary Campbell
EPI_ISL_1017187, EPI_ISL_1017188, EPI_ISL_1017189, EPI_ISL_1017190, EPI_ISL_1017191, EPI_ISL_1017192, EPI_ISL_1017193, EPI_ISL_1017194, EPI_ISL_1017195, EPI_ISL_1017196, EPI_ISL_1017197, EPI_ISL_1017198, EPI_ISL_1017199, EPI_ISL_1017200, EPI_ISL_1017201, EPI_ISL_1017202, EPI_ISL_1017203, EPI_ISL_1017204, EPI_ISL_1017205, EPI_ISL_1017206, EPI_ISL_1017207, EPI_ISL_1017208, EPI_ISL_1017209, EPI_ISL_1017210, EPI_ISL_1017211, EPI_ISL_1017212, EPI_ISL_1017213, EPI_ISL_1017214, EPI_ISL_1017215			
see above	Providence Alaska Medical Center	Providence St. Joseph Health Molecular Genomics Laboratory	Alexa K Dowdell, Brian D Piening, Fred L Robinson, Carlo B Bifulco, Mary Campbell
EPI_ISL_1018099, EPI_ISL_1018100, EPI_ISL_1018101	Immunology, Noguchi Memorial Institute for Medical Research	Immunology, Noguchi Memorial Institute for Medical Research	Adu,B., Egyir,B., Kumordjie,S., Agbodzi,B., Yeboah,C., Mohktar,Q., Oteng,F., Owusu-Nyantakyi,C., Asare,K.M., Appiah-Kubi,J., Adusei-Poku,M.A., Odoom,J.K., Ampofo,W.K., Bonney,J.K.
EPI_ISL_1019655, EPI_ISL_1019699, EPI_ISL_1019705, EPI_ISL_1019726, EPI_ISL_1019737, EPI_ISL_1019742, EPI_ISL_1019750, EPI_ISL_1019778, EPI_ISL_1019783, EPI_ISL_1019813, EPI_ISL_1019846, EPI_ISL_1019853, EPI_ISL_1019855, EPI_ISL_1019858, EPI_ISL_1019863, EPI_ISL_1019872, EPI_ISL_1019875, EPI_ISL_1019877, EPI_ISL_1019880, EPI_ISL_1019882, EPI_ISL_1019888, EPI_ISL_1019893, EPI_ISL_1020022, EPI_ISL_1020025, EPI_ISL_1020027, EPI_ISL_1020028, EPI_ISL_1020029, EPI_ISL_1020030, EPI_ISL_1020031, EPI_ISL_1020032, EPI_ISL_1020034, EPI_ISL_1020036, EPI_ISL_1020037, EPI_ISL_1020041, EPI_ISL_1020043, EPI_ISL_1020045, EPI_ISL_1020046, EPI_ISL_1020049, EPI_ISL_1020050, EPI_ISL_1020051, EPI_ISL_1020053, EPI_ISL_1020054, EPI_ISL_1020055, EPI_ISL_1020059, EPI_ISL_1020062, EPI_ISL_1020063			
see above	Department of Health Technology and Informatics, The Hong Kong Polytechnic University	Department of Health Technology and Informatics, The Hong Kong Polytechnic University	Gilman Kit-Hang Siu, Lam-Kwong Lee, Kenneth Siu-Sing Leung, Jake Siu-Lun Leung, Timothy Ting-Leung Ng, Chloe Toi-Mei Chan, Kingsley King-Gee Tam, Hiu-Yin Lao, Denise Sze-Hang Wong, Alan Ka-Lun Wu, Miranda Chong-Yee Yau, Yvette Wai-Man Lai, Kitty Sau-Chun Fung, Sandy Ka-Yee Chau, Barry Kin-Chung Wong, Wing-Kin To, Kristine Luk, Alex Yat-Man Ho, Tak-Lun Que, Kam-Tong Yip, Wing Cheong Yam, David Ho-Keung Shum, Shea Ping Yip
EPI_ISL_1020196	RS PMI, Bogor, West Java	Biosafety Level-3 Laboratory, Indonesian Institute of Sciences (LIPI)	Anggia Prasetyoputri, Isa Nuryana, Sri Swasthikawati, Sugiyono Saputra, Syam Budi Iryanto, Eko Wahyu Putro, Ratih Asmana Ningrum

EPI_ISL_1020287, EPI_ISL_1020289, EPI_ISL_1020290, EPI_ISL_1020293	AZ Klina	AZ Klina	Dr. C. Vael
EPI_ISL_1025908, EPI_ISL_1025909, EPI_ISL_1025911, EPI_ISL_1025914, EPI_ISL_1025915, EPI_ISL_1025916, EPI_ISL_1025919, EPI_ISL_1025920, EPI_ISL_1025921, EPI_ISL_1025922, EPI_ISL_1025923, EPI_ISL_1025925, EPI_ISL_1025927, EPI_ISL_1025929, EPI_ISL_1025930, EPI_ISL_1025931, EPI_ISL_1025932, EPI_ISL_1025936, EPI_ISL_1025938, EPI_ISL_1025939, EPI_ISL_1025941, EPI_ISL_1025942, EPI_ISL_1025943, EPI_ISL_1025944, EPI_ISL_1025945, EPI_ISL_1025947, EPI_ISL_1025948, EPI_ISL_1025950, EPI_ISL_1025951, EPI_ISL_1025952, EPI_ISL_1025953, EPI_ISL_1025954, EPI_ISL_1025955, EPI_ISL_1025956, EPI_ISL_1025957, EPI_ISL_1025959, EPI_ISL_1025960			
see above	Department of Virus and Microbiological Special Diagnostics, Statens Serum Institut, Copenhagen, Denmark	Aalborg University	Danish Covid-19 Genome Consortium
EPI_ISL_1027472	Alameda County Public Health Lab	Chan-Zuckerberg Biohub	CZB Cliahub Consortium
EPI_ISL_1027535, EPI_ISL_1027539	Humboldt County Public Health Laboratory	Chan-Zuckerberg Biohub	CZB Cliahub Consortium
EPI_ISL_1032931, EPI_ISL_1032932	Illinois Department of Public Health	Gagnon Lab, Southern Illinois University	Keith Gagnon
EPI_ISL_1034612, EPI_ISL_1034613, EPI_ISL_1034614, EPI_ISL_1034621, EPI_ISL_1034622, EPI_ISL_1034623, EPI_ISL_1034624, EPI_ISL_1034625, EPI_ISL_1034626, EPI_ISL_1034627, EPI_ISL_1034628, EPI_ISL_1034629, EPI_ISL_1034630, EPI_ISL_1034631, EPI_ISL_1034632, EPI_ISL_1034633, EPI_ISL_1034635, EPI_ISL_1034636, EPI_ISL_1034666, EPI_ISL_1034721, EPI_ISL_1034726, EPI_ISL_1034728, EPI_ISL_1034729, EPI_ISL_1034732, EPI_ISL_1034733, EPI_ISL_1034734, EPI_ISL_1034735			
see above	Department of Microbiology, The University of Hong Kong	Department of Microbiology, The University of Hong Kong	Kelvin K.W. To, Kwok-Yung Yuen
EPI_ISL_1035248, EPI_ISL_1035249, EPI_ISL_1035663, EPI_ISL_1035697	Dutch COVID-19 response team	National Institute for Public Health and the Environment (RIVM)	Adam Meijer, Harry Vennema, Dirk Eggink, Jeroen Cremer, Sharon van den Brink, Bas van der Veer, AnneMarie van den Brandt, Florian Zwagemaker, Dennis Schmitz, Chantal Reusken, on behalf of the national COVID-19 response team
EPI_ISL_1036235, EPI_ISL_1036236	Center of Advanced Studies and Technology, Molecular Genetics Laboratory	Center of Advanced Studies and Technology, Molecular Genetics Laboratory	Ferrante Rossella, Mandatori Domitilla, De Fabritiis Simone, Damiani Verena, Anaclerio Federico
EPI_ISL_1036324, EPI_ISL_1036325, EPI_ISL_1036327, EPI_ISL_1036329, EPI_ISL_1036330, EPI_ISL_1036333	Johns Hopkins Hospital Department of Pathology	Johns Hopkins Hospital Department of Pathology	C. Paul Morris, Chun Huai Luo, Adannaya Amadi, Matthew Schwartz, Nicholas Gallagher, Heba H. Mostafa
EPI_ISL_1038791	US Air Force School of Aerospace Medicine	US Air Force School of Aerospace Medicine	Anthony Fries, Jennifer Meyer, William Gruner, William Buggele, Amanda Javorina, Sarah Purves, Clarise Starr, Elizabeth Macias
EPI_ISL_1038852, EPI_ISL_1038853, EPI_ISL_1038854	Colorado Department of Public Health and Environment	Colorado Department of Puplic Health and Environment	Laura Bankers, Molly C. Hetherington-Rauth, Diana Ir, Shannon Ely, Shannon R. Matzinger, Sarah Elizabeth Totten, Emily A. Travanty
EPI_ISL_1038945, EPI_ISL_1038952, EPI_ISL_1038956, EPI_ISL_1038961, EPI_ISL_1038962, EPI_ISL_1038963, EPI_ISL_1038964	New Mexico Department of Health Scientific Laboratory	New Mexico Department of Health Scientific Laboratory	Ellie Johnson, Anastacia Griego-Fisher, D'eldra Malone, Jennifer Benoit
EPI_ISL_1039058, EPI_ISL_1039070, EPI_ISL_1039073, EPI_ISL_1039079, EPI_ISL_1039082, EPI_ISL_1039086, EPI_ISL_1039089, EPI_ISL_1039095, EPI_ISL_1039097, EPI_ISL_1039099, EPI_ISL_1039101, EPI_ISL_1039102, EPI_ISL_1039103, EPI_ISL_1039104, EPI_ISL_1039113, EPI_ISL_1039118, EPI_ISL_1039119, EPI_ISL_1039122, EPI_ISL_1039123			
see above	Center of Hygiene and Epidemiology in Belgorod Region	WHO National Influenza Centre Russian Federation	Andrey Komissarov, Artem Fadeev, Lyudmila Berdinskikh, Anna Ivanova, Kseniya Komissarova, Dmitry Bazhenov, Tamila Musaeva, Maria Timofeeva, Veronika Eder, Maria Pisareva, Daria Danilenko, Ksenia Safina, Elena Nabieva, Georgii Bazykin, Dmitry Lioznov
EPI_ISL_1039141	Pathogenic Microorganisms Variability Laboratory	WHO National Influenza Centre Russian Federation	Andrey Komissarov, Artem Fadeev, Anna Ivanova, Kseniya Komissarova, Dmitry Bazhenov, Daria Danilenko, Ksenia Safina, Elena Nabieva, Georgii Bazykin, Nadezhda Kuznetsova, Elena Shidlovskaya, Sergey Alkhovsky, Tatyana Vishnevskaya, Elizaveta Divisenko, Alexey Shchetinin, Maria Nikiforova, Andrey Pochtovyy, Evgeny Usachev, Elena Vokalova, Maxim Rubalsky, Oleg Rubalsky, Artem Tkachuk, Vladimir Gushchin, Alexander Gintsburg, Dmitry Lioznov
EPI_ISL_1040682	False Bay Hospital wc FBH	NHLS/UCT	Arash Iranzadeh, Deelan Doolabh, Lynn Tyers, Bruna Galvao, Innocent Mudau, Marvin Hsiao, Kruger Marais, Diana Hardie, Stephen Korsman, Carolyn Williamson
EPI_ISL_1040766	Mitchells Plain Hospital wc MPH	NHLS/UCT	Arash Iranzadeh, Deelan Doolabh, Lynn Tyers, Bruna Galvao, Innocent Mudau, Marvin Hsiao, Kruger Marais, Diana Hardie, Stephen Korsman, Carolyn Williamson
EPI_ISL_1040768	Groote Schuur Hospital wc GSH	NHLS/UCT	Arash Iranzadeh, Deelan Doolabh, Lynn Tyers, Bruna Galvao, Innocent Mudau, Marvin Hsiao, Kruger Marais, Diana Hardie, Stephen Korsman, Carolyn Williamson
EPI_ISL_1040769, EPI_ISL_1040770, EPI_ISL_1040771, EPI_ISL_1040772, EPI_ISL_1040773	2 Military Hospital wc MAA	NHLS/UCT	Arash Iranzadeh, Deelan Doolabh, Lynn Tyers, Bruna Galvao, Innocent Mudau, Marvin Hsiao, Kruger Marais, Diana Hardie, Stephen Korsman, Carolyn Williamson
EPI_ISL_1040774	Victoria Hospital wc VHW	NHLS/UCT	Arash Iranzadeh, Deelan Doolabh, Lynn Tyers, Bruna Galvao, Innocent Mudau, Marvin Hsiao, Kruger Marais, Diana Hardie, Stephen Korsman, Carolyn Williamson
EPI_ISL_1040775	False Bay Hospital wc FBH	NHLS/UCT	Arash Iranzadeh, Deelan Doolabh, Lynn Tyers, Bruna Galvao, Innocent Mudau, Marvin Hsiao, Kruger Marais, Diana Hardie, Stephen Korsman, Carolyn Williamson
EPI_ISL_1048608, EPI_ISL_1048614, EPI_ISL_1048619, EPI_ISL_1048620, EPI_ISL_1048628, EPI_ISL_1048629, EPI_ISL_1048631, EPI_ISL_1048632, EPI_ISL_1048633, EPI_ISL_1048634, EPI_ISL_1048635, EPI_ISL_1048636, EPI_ISL_1048637, EPI_ISL_1048638, EPI_ISL_1048639, EPI_ISL_1048641, EPI_ISL_1048642, EPI_ISL_1048643, EPI_ISL_1048644, EPI_ISL_1048645, EPI_ISL_1048646, EPI_ISL_1048647, EPI_ISL_1048648, EPI_ISL_1048649, EPI_ISL_1048650, EPI_ISL_1048651			
see above	Maine HETL	Tewhey Lab, The Jackson Laboratory	Matluk,N., Dewey,H., Iosue,F., Barter,M., Lynch,R., Munger,H. and Tewhey,R.
EPI_ISL_1049337	Innovative Genomics Institute, UC Berkeley	Innovative Genomics Institute, UC Berkeley	Stacia Wyman, Alison Ciling, Netravathi Krishnappa, Haridha Shivram, Phil Frankino, Liana Lareau
EPI_ISL_1049517	Seattle Flu Study	Seattle Flu Study	Deborah A. Nickerson, Chris D. Frazar, Jover Lee, Benjamin Pelle, Erica Ryke, Matthew Richardson, Amanda Adler, Elisabeth Brandstetter, Peter D. Han, Kairsten Fay, Misja Ilcisin, Kirsten Lacombe, Thomas R. Sibley, Melissa Truong, Caitlin R. Wolf, Karen Cowgill, Stephanie Schrag, Jeff Duchin, Michael Boeckh, Janet A. Englund, Michael Famulare, Barry R. Lutz, Mark J. Rieder, Lea M. Starita, Matthew Thompson, Helen Y. Chu, Trevor Bedford, Jay Shendure
EPI_ISL_1055114, EPI_ISL_1055135, EPI_ISL_1055136, EPI_ISL_1055142, EPI_ISL_1055145	National Virus Reference Laboratory	National Virus Reference Laboratory	Guerrino Macori, Gabriel Gonzalez, Michael Carr, Zoe Yandle, Jonathan Dean, Seamus Fanning, Cillian F De Gascun
EPI_ISL_1055417, EPI_ISL_1055419, EPI_ISL_1055420, EPI_ISL_1055421, EPI_ISL_1055422, EPI_ISL_1055423, EPI_ISL_1055424, EPI_ISL_1055426	Dr. Leonard A. Miller Centre for Health Services	National Microbiology Laboratory (NML)	Anna Majer, Shari Tyson, Grace Seo, Philip Mabon, Elsie Grudeski, Rhiannon Huzarewich, Russell Mandes, Anneliese Landgraff, Jennifer Tanner, Natalie Knox, Morag Graham, Gary Van Domselaar, Robert Needle, Yang Yu, Adel Malek, Laura Gilbert, George Zahariadis, Nathalie Bastien, Yan Li, Timothy Booth, Darian Hole, Madison Chapel, Kirsten Biggar, Kerri Smith, CanCOGeN's metadata curation team, Public Health Agency of Canada CanCOGeN team
EPI_ISL_1055579, EPI_ISL_1055612, EPI_ISL_1055623, EPI_ISL_1055630	QEII Health Sciences Centre	National Microbiology Laboratory (NML)	Anna Majer, Shari Tyson, Grace Seo, Philip Mabon, Elsie Grudeski, Rhiannon Huzarewich, Russell Mandes, Anneliese Landgraff, Jennifer Tanner, Natalie Knox, Morag Graham, Gary Van Domselaar, Todd Hatchette, Jason LeBlanc, Janice Pettipas, Dan Gaston, Nathalie Bastien, Yan Li, Timothy Booth, Darian Hole, Madison Chapel, Kirsten Biggar, CanCOGeN's metadata curation team, Public Health Agency of Canada CanCOGeN team
EPI_ISL_1059902	DNA Laboratories Sdn. Bhd.	Malaysia Genome Institute	Mohd Noor Mat Isa, Irni Suhayu Sapian, Yusuf Muhammad Noor, Nurhezreen Md Iqbal, Mohd Faizal Abu Bakar, Enizza Kasim, Shamsidar Sopie, Siti Noraini Othman, Azrin Ahmad, Wong Yong Wee, Nor Azfa Johari.
EPI_ISL_1060509, EPI_ISL_1060510	Servicio de Microbiología. Hospital Clínico Universitario de Valencia	SeqCOVID-SPAIN consortium/IBV(CSIC)	David Navarro Ortega, Eliseo Albert Vicent, Ignacio Torres and SeqCOVID-SPAIN consortium
EPI_ISL_1060871	Providence Oregon Regional Laboratories	Providence St. Joseph Health Molecular Genomics Laboratory	Alexa K Dowdell, Brian D Piening, Fred L Robinson, Carlo B Bifulco, Mary Campbell

EPI_ISL_1061405	Queen Elizabeth Hospital	Hong Kong Children's Hospital	Herman Tse, Garnet Kwan-Yue Choi, Sally Cheuk-Ying Wong, David Christopher Lung
EPI_ISL_1061408	Kwong Wah Hospital	Hong Kong Children's Hospital	Herman Tse, Garnet Kwan-Yue Choi, Sally Cheuk-Ying Wong, David Christopher Lung
EPI_ISL_1061409, EPI_ISL_1061410, EPI_ISL_1061411	Queen Elizabeth Hospital	Hong Kong Children's Hospital	Herman Tse, Garnet Kwan-Yue Choi, Sally Cheuk-Ying Wong, David Christopher Lung
EPI_ISL_1063636, EPI_ISL_1063637, EPI_ISL_1063638, EPI_ISL_1063678, EPI_ISL_1063688	Division of Emerging Infectious Diseases, Bureau of Infectious Diseases Diagnosis Control, Korea Disease Control and Prevention Agency	Division of Emerging Infectious Diseases, Bureau of Infectious Diseases Diagnosis Control, Korea Disease Control and Prevention Agency	Ae Kyung Park, Il-Hwan Kim, Heui Man Kim, Jeong-Min Kim, Namjoo Lee, Chaeyoung Lee, Sang Hee Woo, Eun-Jin Kim
EPI_ISL_1063808, EPI_ISL_1063862, EPI_ISL_1063864, EPI_ISL_1063866, EPI_ISL_1063867, EPI_ISL_1063868, EPI_ISL_1063869, EPI_ISL_1063870, EPI_ISL_1063871, EPI_ISL_1063872, EPI_ISL_1063873, EPI_ISL_1063874, EPI_ISL_1063875, EPI_ISL_1063876, EPI_ISL_1063878, EPI_ISL_1063879, EPI_ISL_1063880, EPI_ISL_1063881, EPI_ISL_1063882, EPI_ISL_1063884, EPI_ISL_1063885, EPI_ISL_1063886, EPI_ISL_1063887, EPI_ISL_1063888, EPI_ISL_1063889, EPI_ISL_1063890, EPI_ISL_1063893, EPI_ISL_1063894, EPI_ISL_1063895			
see above	Immunogenomics lab, Institute of Life Sciences, Bhubaneswar	Immunogenomics lab, Institute of Life Sciences, Bhubaneswar	Sunil K. Raghav, Safal Walia, Arup Ghosh, Atimukta Jha, Amol M. Kanampalliwar, Shifu Aggarwal, Kirtal Hansdah, Rupesh Dash, Rajeeb Swain, Punit Prasad, INSACOG Consortium, Ajay Parida
EPI_ISL_1064694	A05	The Public Health Agency of Sweden	Oskar Karlsson Lindsjo, Maria Lind Karlberg, Carlo Berg, Anna-Malin Linde, Sofia Stamouli, Samuel Ohman, Reza Advani, Mattias Haukland, Petra Holmstrom, Noura Walai, Petra Edquist, Mia Brytting, Anna Risberg, Karin Tegmark-Wisell
EPI_ISL_1065103, EPI_ISL_1065275, EPI_ISL_1065294, EPI_ISL_1065312, EPI_ISL_1065370, EPI_ISL_1065437, EPI_ISL_1065587, EPI_ISL_1065603, EPI_ISL_1065614, EPI_ISL_1065662, EPI_ISL_1065764, EPI_ISL_1065886, EPI_ISL_1065939, EPI_ISL_1066147, EPI_ISL_1066181, EPI_ISL_1066231, EPI_ISL_1066241, EPI_ISL_1066255, EPI_ISL_1066303, EPI_ISL_1066427, EPI_ISL_1066450, EPI_ISL_1066560, EPI_ISL_1066577, EPI_ISL_1066678, EPI_ISL_1066715, EPI_ISL_1066882, EPI_ISL_1066933, EPI_ISL_1067182, EPI_ISL_1067191, EPI_ISL_1067215, EPI_ISL_1067288, EPI_ISL_1067293, EPI_ISL_1067364, EPI_ISL_1067409, EPI_ISL_1067414, EPI_ISL_1067433, EPI_ISL_1067448, EPI_ISL_1067487, EPI_ISL_1067489, EPI_ISL_1067504, EPI_ISL_1067524, EPI_ISL_1067535			
see above	Department of Virus and Microbiological Special Diagnostics, Statens Serum Institut, Copenhagen, Denmark	Aalborg University	Danish Covid-19 Genome Consortium
EPI_ISL_1067917, EPI_ISL_1067918, EPI_ISL_1067919, EPI_ISL_1067920, EPI_ISL_1067922, EPI_ISL_1067926, EPI_ISL_1067932, EPI_ISL_1067933, EPI_ISL_1067934, EPI_ISL_1067935, EPI_ISL_1067936, EPI_ISL_1067937, EPI_ISL_1067939, EPI_ISL_1067940, EPI_ISL_1067941, EPI_ISL_1067942			
see above	Wisconsin State Laboratory of Hygiene Communicable Disease Division	Wisconsin State Laboratory of Hygiene Communicable Disease Division	Kelsey R. Florek, Abigail C. Shockey
EPI_ISL_1068105, EPI_ISL_1068106, EPI_ISL_1068126, EPI_ISL_1068127, EPI_ISL_1068128, EPI_ISL_1068129, EPI_ISL_1068130, EPI_ISL_1068131, EPI_ISL_1068132, EPI_ISL_1068133, EPI_ISL_1068134, EPI_ISL_1068135, EPI_ISL_1068136, EPI_ISL_1068137, EPI_ISL_1068138, EPI_ISL_1068139, EPI_ISL_1068140, EPI_ISL_1068141			
see above	Laboratorio de Ecologia de Doencas Transmissiveis na Amazonia, Instituto Leonidas e Maria Deane - Fiocruz Amazonia	Laboratorio de Ecologia de Doencas Transmissiveis na Amazonia, Instituto Leonidas e Maria Deane - Fiocruz Amazonia	Valdinete Nascimento, Victor Souza, André Corado, Fernanda Nascimento, George Silva, Ágatha Costa, Debora Duarte, Karina Pessoa, Matilde Mejía, Luciana Gonçalves, Maria Júlia Brandão, Michele Jesus, Felipe Naveca on behalf of the Fiocruz COVID-19 Genomic Surveillance Network
EPI_ISL_1068351, EPI_ISL_1068358, EPI_ISL_1068360, EPI_ISL_1068361	Central Public Health Laboratory - LACEN -Bahia, Salvador, Brazil	Central Public Health Laboratory - LACEN -Bahia, Salvador, Brazil	Stephane Tosta, Luciana Oliveira, Vanessa Nardy,Patrícia Cajado,Marcela Gómez, Breno Dominguez, Jaqueline Gomes, Vagner Fonseca,Marta Giovanetti,Luiz Alcantara, Felicidade Pereira, Arabela Leal
EPI_ISL_1073815, EPI_ISL_1073816	Kansas Health and Environmental Lab	Kansas Health and Environmental Lab	Mike Grose, Carissa Robertson, Ben Olsen, and Phil Adam
EPI_ISL_1076566, EPI_ISL_1076567, EPI_ISL_1076569	Houston Methodist Hospital	Houston Methodist Hospital	S. Wesley Long, Randall J. Olsen, Paul A. Christensen, Sishir Subedi, Robert Olson, James J. Davis, Matthew Ojeda Saavedra, Prasanti Yerramilli, Layne Pruitt, Kristina Reppond, Madison N. Shyer, Jessica Cambric, Ilya J. Finkelstein, Jimmy Gollihar, and James M. Musser
EPI_ISL_1081959	"Stefan S. Nicolau"Institute of Virology	"Stefan S. Nicolau"Institute of Virology	Anca Botezatu, Iulia Virginia Iancu, Coralia Bleotu, Mihaela Economescu, Laura Necula, Denisa Dragu, Lilia Matei, Ioana Pitica, Marinela Bostan, Mirela Mihaila, Laura Grecu, Camelia Sultana, Simona Ruta, Ana Iulia Neagu, Adriana Plesa, Saviana Nedeianu, Alina Nastasie, Cristina Mambet,Gabriela Anton, Carmen Cristina Diaconu
EPI_ISL_1085013	Laboratorio Biologia Molecolare Sars Cov2 - UOC Laboratorio Analisi - Servizio Medicina di Laboratorio, Ospedale "San Francesco" - ATS-ASSL Nuoro	Laboratorio Biologia Molecolare Sars Cov2 - UOC Laboratorio Analisi - Servizio Medicina di Laboratorio, Ospedale "San Francesco" - ATS-ASSL Nuoro	Piras Giovanna, Asproni Rosanna, Monne Maria Itria, Fiamma Maura, , Palmas Angelo Domenico, Lo Maglio Iana, Mameli Giuseppe
EPI_ISL_1085675	Hôpital Henri Mondor	Department of Virology, Henri Mondor University Hospital, Assistance Publique Hôpitaux de Paris, Université Paris-Est Créteil, INSERM U955	Christophe Rodriguez, Slim Fourati, Vanessa Demontant, Guillaume Gricourt, Melissa N'Debi, Alexandre Soulier, Elisabeth Trawinski, Jean-Michel Pawlotsky
EPI_ISL_1092247, EPI_ISL_1092316	Protzer Lab	Protzer Lab, Laboratory for Functional Genome Analysis, Dept. Genomics, Gene Center of the LMU Munich	Ulrike Protzer, Dieter Hoffmann, Till Bunse, Eva C.Schulte, Elisabeth Esser, Stefan Krebs, Alexander Graf, Helmut Blum
EPI_ISL_1094648, EPI_ISL_1094651, EPI_ISL_1094652	KS Health and Environmental Laboratories	Respiratory Viruses Branch, Division of Viral Diseases, Centers for Disease Control and Prevention	Krista Queen, Yan Li, Ying Tao, Jing Zhang, Anna Uehara, Anna Montmayeur, Clinton R. Paden, Peter W. Cook, Rachel Marine, Mili Sheth, Jasmine Padilla, Sarah Nobles, Mark Burroughs, Lori Rowe, Haibin Wang, Ben L. Rambo-Martin, Dhwani Batra, Justin Lee, Suxiang Tong
EPI_ISL_1095516	Instituto Nacional de Medicina Genomica	Instituto Nacional de Medicina Genomica	Hidalgo-Miranda A, Mendoza-Vargas A, Reyes-Grajeda JP, Cedro-Tanda A, Alcaraz N, Gonzalez-Barrera D, Rangel-DeLeon D, Ramirez-Vega O, Sifuentes-Rojas C, Cisneros-Villanueva M, Herrera-Montalvo LA
EPI_ISL_1098647	South Eastern Area Laboratory Services (SEALS)	NSW Health Pathology - Institute of Clinical Pathology and Medical Research; Westmead Hospital; University of Sydney	CIDM-PH et al.
EPI_ISL_1098691, EPI_ISL_1098692, EPI_ISL_1098693, EPI_ISL_1098694, EPI_ISL_1098695, EPI_ISL_1098696, EPI_ISL_1098697, EPI_ISL_1098698, EPI_ISL_1098699, EPI_ISL_1098700, EPI_ISL_1098701, EPI_ISL_1098702, EPI_ISL_1098703, EPI_ISL_1098704, EPI_ISL_1098705, EPI_ISL_1098706, EPI_ISL_1098707, EPI_ISL_1098708, EPI_ISL_1098709, EPI_ISL_1098710, EPI_ISL_1098711, EPI_ISL_1098712, EPI_ISL_1098713, EPI_ISL_1098714, EPI_ISL_1098715, EPI_ISL_1098716, EPI_ISL_1098717, EPI_ISL_1098718, EPI_ISL_1098719, EPI_ISL_1098720, EPI_ISL_1098721, EPI_ISL_1098722, EPI_ISL_1098723, EPI_ISL_1098724, EPI_ISL_1098725, EPI_ISL_1098726, EPI_ISL_1098727, EPI_ISL_1098728, EPI_ISL_1098729, EPI_ISL_1098730, EPI_ISL_1098731, EPI_ISL_1098732, EPI_ISL_1098733, EPI_ISL_1098734, EPI_ISL_1098735, EPI_ISL_1098736, EPI_ISL_1098737, EPI_ISL_1098738, EPI_ISL_1098739, EPI_ISL_1098740, EPI_ISL_1098741, EPI_ISL_1098742, EPI_ISL_1098743, EPI_ISL_1098744, EPI_ISL_1098745, EPI_ISL_1098746, EPI_ISL_1098747, EPI_ISL_1098748, EPI_ISL_1098749, EPI_ISL_1098750, EPI_ISL_1098751, EPI_ISL_1098752, EPI_ISL_1098753			
see above	Fukuoka Institute of Health and Environmental Sciences	Fukuoka Institute of Health and Environmental Sciences	Asako Nakamura, Yoshiki Etoh, Hiroaki Shigemura, Akira Ohishi, Takayuki Kobayashi, Saori Ueda, Chiharu Katamune, Mitsuhiro Hamasaki, Yuki Ashizuka, Tsuyoshi Sekizuka, Kentaro Itokawa, Rina Tanaka, Masanori Hashino, Makoto Kuroda
EPI_ISL_1104842, EPI_ISL_1104854, EPI_ISL_1104868, EPI_ISL_1104869, EPI_ISL_1104878, EPI_ISL_1104883, EPI_ISL_1104895, EPI_ISL_1104896, EPI_ISL_1104897, EPI_ISL_1104905, EPI_ISL_1104914, EPI_ISL_1104923, EPI_ISL_1104929, EPI_ISL_1104935, EPI_ISL_1104941, EPI_ISL_1104947, EPI_ISL_1104968			
see above	University College London Hospital	COVID-19 Genomics UK (COG-UK) Consortium	Judith Heaney, Matthew Byott, Catherine Houlihan, Dan Frampton, Stuart Kirk, Moira Spyer and Eleni Nastouli
EPI_ISL_1109484, EPI_ISL_1109485	Medical Ain Shams Research Institute (MASRI), Ain Shams University	Medical Ain Shams Research Institute (MASRI), Ain Shams University	Hesham Elghazaly, Sara Hassan Agwa, Hala Hafez , Fatma Ebied, Manal Hamdy Elsaid, Mohamed Elhadidi, Aya Mohamed , Shimaa Moustafa, Reham Kassab, Iman Foda , Samia Abdou Girgis, Hoda Ezz Elarab, Hagar Elshora, Osama Mansour.
EPI_ISL_1109980, EPI_ISL_1110009	MEPHI, Aix Marseille University	MEPHI, Aix Marseille University	Anthony LEVASSEUR
EPI_ISL_1110560, EPI_ISL_1110562, EPI_ISL_1110565, EPI_ISL_1110566, EPI_ISL_1110567, EPI_ISL_1110569, EPI_ISL_1110570, EPI_ISL_1110571, EPI_ISL_1110572, EPI_ISL_1110573, EPI_ISL_1110574, EPI_ISL_1110575, EPI_ISL_1110577, EPI_ISL_1110578, EPI_ISL_1110579			
see above	Kansas Health and Environmental Lab	Kansas Health and Environmental Lab	Mike Grose, Carissa Robertson, Ben Olsen, and Phil Adam
EPI_ISL_1111309, EPI_ISL_1111312	Laboratorio de Referencia Nacional de Virus Respiratorio. Instituto Nacional de Salud Perú	Laboratorio de Referencia Nacional de Enteropatógenos. Instituto Nacional de Salud del Perú	Ronnie Gavilan Chavez, Junior Caro Castro, Willi Quino Sifuentes, Veronica Hurtado Vela, Iris Silva Molina, Fiorella Orellana Peralta
EPI_ISL_1111594, EPI_ISL_1111595	Virginia Division of Consolidated Laboratory Services	Virginia Division of Consolidated Laboratory Services	Virginia DCLS
EPI_ISL_1113719, EPI_ISL_1113722, EPI_ISL_1113724, EPI_ISL_1113725, EPI_ISL_1113726, EPI_ISL_1113730, EPI_ISL_1113734, EPI_ISL_1113735, EPI_ISL_1113739, EPI_ISL_1113742, EPI_ISL_1113743, EPI_ISL_1113748, EPI_ISL_1113749, EPI_ISL_1113753			
see above	New Mexico Department of Health Scientific Laboratory	New Mexico Department of Health Scientific Laboratory	Ellie Johnson, Anastacia Griego-Fisher, D'eldra Malone, Jennifer Benoit
EPI_ISL_1114726	Institute for Medical Research, Infectious Disease Research Centre, National Institutes of Health, Ministry of Health Malaysia	Institute for Medical Research, Infectious Disease Research Centre, National Institutes of Health, Ministry of Health Malaysia	Suppiah J, Kamel K, Azizan MA, Thayan R
EPI_ISL_1117451	RS PMI, Bogor, West Java	Biosafety Level-3 Laboratory, Indonesian Institute of Sciences	Anik Budhi Dharmayanthi, Ade Andriani, Hariyatun, Asep M. Ridwanuloh, Syam Budi Iryanto, Eko Wahyu Putro, Gita Syahputra, Susanti, Inswasti Cahyani,

		(LIPI)	Matthew W. Loose, Mohammad Ilyas, Wien Kusharyoto
EPI_ISL_1118719, EPI_ISL_1118722, EPI_ISL_1118724, EPI_ISL_1118725, EPI_ISL_1118726, EPI_ISL_1118730, EPI_ISL_1118731, EPI_ISL_1118732, EPI_ISL_1118735, EPI_ISL_1118736, EPI_ISL_1118737, EPI_ISL_1118749, EPI_ISL_1118751, EPI_ISL_1118753, EPI_ISL_1118755, EPI_ISL_1118756, EPI_ISL_1118759, EPI_ISL_1118764, EPI_ISL_1118768, EPI_ISL_1118771, EPI_ISL_1118773, EPI_ISL_1118774, EPI_ISL_1118777, EPI_ISL_1118780, EPI_ISL_1118781, EPI_ISL_1118782, EPI_ISL_1118783, EPI_ISL_1118787, EPI_ISL_1118789, EPI_ISL_1118790, EPI_ISL_1118791, EPI_ISL_1118793, EPI_ISL_1118794, EPI_ISL_1118795, EPI_ISL_1118796, EPI_ISL_1118797, EPI_ISL_1118800, EPI_ISL_1118804, EPI_ISL_1118806, EPI_ISL_1118811, EPI_ISL_1118812, EPI_ISL_1118814, EPI_ISL_1118815, EPI_ISL_1118816, EPI_ISL_1118817			
see above	University of Michigan Clinical Microbiology Laboratory	Lauring Lab, University of Michigan, Department of Microbiology and Immunology	Valesano
EPI_ISL_1119042, EPI_ISL_1119043, EPI_ISL_1119044, EPI_ISL_1119045, EPI_ISL_1119046, EPI_ISL_1119047, EPI_ISL_1119049, EPI_ISL_1119050, EPI_ISL_1119202, EPI_ISL_1119222	Viollier AG	Department of Biosystems Science and Engineering, ETH Zürich	Chaoran Chen, Sarah Nadeau, Ivan Topolsky, Emmanouil Dermitzakis, Keith Harshman, Ioannis Xenarios, Henri Pegeot, Lorenzo Cerutti, Deborah Penet, Philipp Jablonski, Lara Fuhrmann, David Dreifuss, Katharina Jahn, Christiane Beckmann, Maurice Redondo, Olivier Kobel, Christoph Noppen, Sophie Seidel, Noemie Santamaria de Souza, Niko Beerenwinkel, Tanja Stadler
EPI_ISL_1119234, EPI_ISL_1119235, EPI_ISL_1119236	Viollier AG	Department of Biosystems Science and Engineering, ETH Zürich	Chaoran Chen, Sarah Nadeau, Catharine Aquino, Ivan Topolsky, Philipp Jablonski, Lara Fuhrmann, David Dreifuss, Katharina Jahn, Andreia Cabral de Gouvea, Maria Domenica Moccia, Simon Grüter, Timothy Sykes, Lennart Opitz, Griffin White, Laura Neff, Doris Popovic, Andrea Patrignani, Jay Tracy, Ralph Schlapbach, Christiane Beckmann, Maurice Redondo, Olivier Kobel, Christoph Noppen, Sophie Seidel, Noemie Santamaria de Souza, Niko Beerenwinkel, Tanja Stadler
EPI_ISL_1119238, EPI_ISL_1119244	Viollier AG	Department of Biosystems Science and Engineering, ETH Zürich	Chaoran Chen, Sarah Nadeau, Ivan Topolsky, Emmanouil Dermitzakis, Keith Harshman, Ioannis Xenarios, Henri Pegeot, Lorenzo Cerutti, Deborah Penet, Philipp Jablonski, Lara Fuhrmann, David Dreifuss, Katharina Jahn, Christiane Beckmann, Maurice Redondo, Olivier Kobel, Christoph Noppen, Sophie Seidel, Noemie Santamaria de Souza, Niko Beerenwinkel, Tanja Stadler
EPI_ISL_1119340, EPI_ISL_1119348	Viollier AG	Department of Biosystems Science and Engineering, ETH Zürich	Chaoran Chen, Sarah Nadeau, Catharine Aquino, Ivan Topolsky, Philipp Jablonski, Lara Fuhrmann, David Dreifuss, Katharina Jahn, Andreia Cabral de Gouvea, Maria Domenica Moccia, Simon Grüter, Timothy Sykes, Lennart Opitz, Griffin White, Laura Neff, Doris Popovic, Andrea Patrignani, Jay Tracy, Ralph Schlapbach, Christiane Beckmann, Maurice Redondo, Olivier Kobel, Christoph Noppen, Sophie Seidel, Noemie Santamaria de Souza, Niko Beerenwinkel, Tanja Stadler
EPI_ISL_1119351	Viollier AG	Department of Biosystems Science and Engineering, ETH Zürich	Chaoran Chen, Sarah Nadeau, Ivan Topolsky, Emmanouil Dermitzakis, Keith Harshman, Ioannis Xenarios, Henri Pegeot, Lorenzo Cerutti, Deborah Penet, Philipp Jablonski, Lara Fuhrmann, David Dreifuss, Katharina Jahn, Christiane Beckmann, Maurice Redondo, Olivier Kobel, Christoph Noppen, Sophie Seidel, Noemie Santamaria de Souza, Niko Beerenwinkel, Tanja Stadler
EPI_ISL_1119367	Viollier AG	Department of Biosystems Science and Engineering, ETH Zürich	Chaoran Chen, Sarah Nadeau, Catharine Aquino, Ivan Topolsky, Philipp Jablonski, Lara Fuhrmann, David Dreifuss, Katharina Jahn, Andreia Cabral de Gouvea, Maria Domenica Moccia, Simon Grüter, Timothy Sykes, Lennart Opitz, Griffin White, Laura Neff, Doris Popovic, Andrea Patrignani, Jay Tracy, Ralph Schlapbach, Christiane Beckmann, Maurice Redondo, Olivier Kobel, Christoph Noppen, Sophie Seidel, Noemie Santamaria de Souza, Niko Beerenwinkel, Tanja Stadler
EPI_ISL_1119370	Viollier AG	Department of Biosystems Science and Engineering, ETH Zürich	Chaoran Chen, Sarah Nadeau, Ivan Topolsky, Emmanouil Dermitzakis, Keith Harshman, Ioannis Xenarios, Henri Pegeot, Lorenzo Cerutti, Deborah Penet, Philipp Jablonski, Lara Fuhrmann, David Dreifuss, Katharina Jahn, Christiane Beckmann, Maurice Redondo, Olivier Kobel, Christoph Noppen, Sophie Seidel, Noemie Santamaria de Souza, Niko Beerenwinkel, Tanja Stadler
EPI_ISL_1119371, EPI_ISL_1119372	Viollier AG	Department of Biosystems Science and Engineering, ETH Zürich	Chaoran Chen, Sarah Nadeau, Catharine Aquino, Ivan Topolsky, Philipp Jablonski, Lara Fuhrmann, David Dreifuss, Katharina Jahn, Andreia Cabral de Gouvea, Maria Domenica Moccia, Simon Grüter, Timothy Sykes, Lennart Opitz, Griffin White, Laura Neff, Doris Popovic, Andrea Patrignani, Jay Tracy, Ralph Schlapbach, Christiane Beckmann, Maurice Redondo, Olivier Kobel, Christoph Noppen, Sophie Seidel, Noemie Santamaria de Souza, Niko Beerenwinkel, Tanja Stadler
EPI_ISL_1119374	Viollier AG	Department of Biosystems Science and Engineering, ETH Zürich	Chaoran Chen, Sarah Nadeau, Ivan Topolsky, Emmanouil Dermitzakis, Keith Harshman, Ioannis Xenarios, Henri Pegeot, Lorenzo Cerutti, Deborah Penet, Philipp Jablonski, Lara Fuhrmann, David Dreifuss, Katharina Jahn, Christiane Beckmann, Maurice Redondo, Olivier Kobel, Christoph Noppen, Sophie Seidel, Noemie Santamaria de Souza, Niko Beerenwinkel, Tanja Stadler
EPI_ISL_1119377, EPI_ISL_1119379, EPI_ISL_1119380, EPI_ISL_1119381, EPI_ISL_1119402, EPI_ISL_1119404, EPI_ISL_1119412, EPI_ISL_1119427, EPI_ISL_1119451, EPI_ISL_1119453	Viollier AG	Department of Biosystems Science and Engineering, ETH Zürich	Chaoran Chen, Sarah Nadeau, Catharine Aquino, Ivan Topolsky, Philipp Jablonski, Lara Fuhrmann, David Dreifuss, Katharina Jahn, Andreia Cabral de Gouvea, Maria Domenica Moccia, Simon Grüter, Timothy Sykes, Lennart Opitz, Griffin White, Laura Neff, Doris Popovic, Andrea Patrignani, Jay Tracy, Ralph Schlapbach, Christiane Beckmann, Maurice Redondo, Olivier Kobel, Christoph Noppen, Sophie Seidel, Noemie Santamaria de Souza, Niko Beerenwinkel, Tanja Stadler
EPI_ISL_1119456	Viollier AG	Department of Biosystems Science and Engineering, ETH Zürich	Chaoran Chen, Sarah Nadeau, Ivan Topolsky, Emmanouil Dermitzakis, Keith Harshman, Ioannis Xenarios, Henri Pegeot, Lorenzo Cerutti, Deborah Penet, Philipp Jablonski, Lara Fuhrmann, David Dreifuss, Katharina Jahn, Christiane Beckmann, Maurice Redondo, Olivier Kobel, Christoph Noppen, Sophie Seidel, Noemie Santamaria de Souza, Niko Beerenwinkel, Tanja Stadler
EPI_ISL_1119457, EPI_ISL_1119458, EPI_ISL_1119459, EPI_ISL_1119461	Viollier AG	Department of Biosystems Science and Engineering, ETH Zürich	Chaoran Chen, Sarah Nadeau, Catharine Aquino, Ivan Topolsky, Philipp Jablonski, Lara Fuhrmann, David Dreifuss, Katharina Jahn, Andreia Cabral de Gouvea, Maria Domenica Moccia, Simon Grüter, Timothy Sykes, Lennart Opitz, Griffin White, Laura Neff, Doris Popovic, Andrea Patrignani, Jay Tracy, Ralph Schlapbach, Christiane Beckmann, Maurice Redondo, Olivier Kobel, Christoph Noppen, Sophie Seidel, Noemie Santamaria de Souza, Niko Beerenwinkel, Tanja Stadler
EPI_ISL_1119462, EPI_ISL_1119463, EPI_ISL_1119464	Viollier AG	Department of Biosystems Science and Engineering, ETH Zürich	Chaoran Chen, Sarah Nadeau, Ivan Topolsky, Emmanouil Dermitzakis, Keith Harshman, Ioannis Xenarios, Henri Pegeot, Lorenzo Cerutti, Deborah Penet, Philipp Jablonski, Lara Fuhrmann, David Dreifuss, Katharina Jahn, Christiane Beckmann, Maurice Redondo, Olivier Kobel, Christoph Noppen, Sophie Seidel, Noemie Santamaria de Souza, Niko Beerenwinkel, Tanja Stadler
EPI_ISL_1119466, EPI_ISL_1119467	Viollier AG	Department of Biosystems Science and Engineering, ETH Zürich	Chaoran Chen, Sarah Nadeau, Catharine Aquino, Ivan Topolsky, Philipp Jablonski, Lara Fuhrmann, David Dreifuss, Katharina Jahn, Andreia Cabral de Gouvea, Maria Domenica Moccia, Simon Grüter, Timothy Sykes, Lennart Opitz, Griffin White, Laura Neff, Doris Popovic, Andrea Patrignani, Jay Tracy, Ralph Schlapbach, Christiane Beckmann, Maurice Redondo, Olivier Kobel, Christoph Noppen, Sophie Seidel, Noemie Santamaria de Souza, Niko Beerenwinkel, Tanja Stadler
EPI_ISL_1119468	Viollier AG	Department of Biosystems Science and Engineering, ETH Zürich	Chaoran Chen, Sarah Nadeau, Ivan Topolsky, Emmanouil Dermitzakis, Keith Harshman, Ioannis Xenarios, Henri Pegeot, Lorenzo Cerutti, Deborah Penet, Philipp Jablonski, Lara Fuhrmann, David Dreifuss, Katharina Jahn, Christiane Beckmann, Maurice Redondo, Olivier Kobel, Christoph Noppen, Sophie Seidel, Noemie Santamaria de Souza, Niko Beerenwinkel, Tanja Stadler
EPI_ISL_1119473, EPI_ISL_1119474, EPI_ISL_1119475	Viollier AG	Department of Biosystems Science and Engineering, ETH Zürich	Chaoran Chen, Sarah Nadeau, Catharine Aquino, Ivan Topolsky, Philipp Jablonski, Lara Fuhrmann, David Dreifuss, Katharina Jahn, Andreia Cabral de Gouvea, Maria Domenica Moccia, Simon Grüter, Timothy Sykes, Lennart Opitz, Griffin White, Laura Neff, Doris Popovic, Andrea Patrignani, Jay Tracy, Ralph Schlapbach, Christiane Beckmann, Maurice Redondo, Olivier Kobel, Christoph Noppen, Sophie Seidel, Noemie Santamaria de Souza, Niko Beerenwinkel, Tanja Stadler
EPI_ISL_1119476, EPI_ISL_1119478	Viollier AG	Department of Biosystems Science and Engineering, ETH Zürich	Chaoran Chen, Sarah Nadeau, Ivan Topolsky, Emmanouil Dermitzakis, Keith Harshman, Ioannis Xenarios, Henri Pegeot, Lorenzo Cerutti, Deborah Penet, Philipp Jablonski, Lara Fuhrmann, David Dreifuss, Katharina Jahn, Christiane Beckmann, Maurice Redondo, Olivier Kobel, Christoph Noppen, Sophie Seidel, Noemie Santamaria de Souza, Niko Beerenwinkel, Tanja Stadler
EPI_ISL_1119480	Viollier AG	Department of Biosystems Science and Engineering, ETH Zürich	Chaoran Chen, Sarah Nadeau, Catharine Aquino, Ivan Topolsky, Philipp Jablonski, Lara Fuhrmann, David Dreifuss, Katharina Jahn, Andreia Cabral de Gouvea, Maria Domenica Moccia, Simon Grüter, Timothy Sykes, Lennart Opitz, Griffin White, Laura Neff, Doris Popovic, Andrea Patrignani, Jay Tracy, Ralph Schlapbach, Christiane Beckmann, Maurice Redondo, Olivier Kobel, Christoph Noppen, Sophie Seidel, Noemie Santamaria de Souza, Niko Beerenwinkel, Tanja Stadler
EPI_ISL_1119481	Viollier AG	Department of Biosystems Science and Engineering, ETH Zürich	Chaoran Chen, Sarah Nadeau, Ivan Topolsky, Emmanouil Dermitzakis, Keith Harshman, Ioannis Xenarios, Henri Pegeot, Lorenzo Cerutti, Deborah Penet, Philipp Jablonski, Lara Fuhrmann, David Dreifuss, Katharina Jahn, Christiane Beckmann, Maurice Redondo, Olivier Kobel, Christoph Noppen, Sophie Seidel, Noemie Santamaria de Souza, Niko Beerenwinkel, Tanja Stadler
EPI_ISL_1119482, EPI_ISL_1119483,	Viollier AG	Department of Biosystems Science and Engineering, ETH	Chaoran Chen, Sarah Nadeau, Catharine Aquino, Ivan Topolsky, Philipp Jablonski, Lara Fuhrmann, David Dreifuss, Katharina Jahn, Andreia Cabral de

EPI_ISL_1119484, EPI_ISL_1119486		Zürich	Gouvea, Maria Domenica Moccia, Simon Grüter, Timothy Sykes, Lennart Opitz, Griffin White, Laura Neff, Doris Popovic, Andrea Patrignani, Jay Tracy, Ralph Schlapbach, Christiane Beckmann, Maurice Redondo, Olivier Kobel, Christoph Noppen, Sophie Seidel, Noemie Santamaria de Souza, Niko Beerenwinkel, Tanja Stadler
EPI_ISL_1119487	Viollier AG	Department of Biosystems Science and Engineering, ETH Zürich	Chaoran Chen, Sarah Nadeau, Ivan Topolsky, Emmanouil Dermitzakis, Keith Harshman, Ioannis Xenarios, Henri Pegeot, Lorenzo Cerutti, Deborah Penet, Philipp Jablonski, Lara Fuhrmann, David Dreifuss, Katharina Jahn, Christiane Beckmann, Maurice Redondo, Olivier Kobel, Christoph Noppen, Sophie Seidel, Noemie Santamaria de Souza, Niko Beerenwinkel, Tanja Stadler
EPI_ISL_1119488, EPI_ISL_1119490, EPI_ISL_1119491, EPI_ISL_1119493, EPI_ISL_1119494	Viollier AG	Department of Biosystems Science and Engineering, ETH Zürich	Chaoran Chen, Sarah Nadeau, Catharine Aquino, Ivan Topolsky, Philipp Jablonski, Lara Fuhrmann, David Dreifuss, Katharina Jahn, Andreia Cabral de Gouvea, Maria Domenica Moccia, Simon Grüter, Timothy Sykes, Lennart Opitz, Griffin White, Laura Neff, Doris Popovic, Andrea Patrignani, Jay Tracy, Ralph Schlapbach, Christiane Beckmann, Maurice Redondo, Olivier Kobel, Christoph Noppen, Sophie Seidel, Noemie Santamaria de Souza, Niko Beerenwinkel, Tanja Stadler
EPI_ISL_1119497, EPI_ISL_1119498, EPI_ISL_1119499	Viollier AG	Department of Biosystems Science and Engineering, ETH Zürich	Chaoran Chen, Sarah Nadeau, Ivan Topolsky, Emmanouil Dermitzakis, Keith Harshman, Ioannis Xenarios, Henri Pegeot, Lorenzo Cerutti, Deborah Penet, Philipp Jablonski, Lara Fuhrmann, David Dreifuss, Katharina Jahn, Christiane Beckmann, Maurice Redondo, Olivier Kobel, Christoph Noppen, Sophie Seidel, Noemie Santamaria de Souza, Niko Beerenwinkel, Tanja Stadler
EPI_ISL_1119501	Viollier AG	Department of Biosystems Science and Engineering, ETH Zürich	Chaoran Chen, Sarah Nadeau, Catharine Aquino, Ivan Topolsky, Philipp Jablonski, Lara Fuhrmann, David Dreifuss, Katharina Jahn, Andreia Cabral de Gouvea, Maria Domenica Moccia, Simon Grüter, Timothy Sykes, Lennart Opitz, Griffin White, Laura Neff, Doris Popovic, Andrea Patrignani, Jay Tracy, Ralph Schlapbach, Christiane Beckmann, Maurice Redondo, Olivier Kobel, Christoph Noppen, Sophie Seidel, Noemie Santamaria de Souza, Niko Beerenwinkel, Tanja Stadler
EPI_ISL_1119503	Viollier AG	Department of Biosystems Science and Engineering, ETH Zürich	Chaoran Chen, Sarah Nadeau, Ivan Topolsky, Emmanouil Dermitzakis, Keith Harshman, Ioannis Xenarios, Henri Pegeot, Lorenzo Cerutti, Deborah Penet, Philipp Jablonski, Lara Fuhrmann, David Dreifuss, Katharina Jahn, Christiane Beckmann, Maurice Redondo, Olivier Kobel, Christoph Noppen, Sophie Seidel, Noemie Santamaria de Souza, Niko Beerenwinkel, Tanja Stadler
EPI_ISL_1119506, EPI_ISL_1119507, EPI_ISL_1119508	Viollier AG	Department of Biosystems Science and Engineering, ETH Zürich	Chaoran Chen, Sarah Nadeau, Catharine Aquino, Ivan Topolsky, Philipp Jablonski, Lara Fuhrmann, David Dreifuss, Katharina Jahn, Andreia Cabral de Gouvea, Maria Domenica Moccia, Simon Grüter, Timothy Sykes, Lennart Opitz, Griffin White, Laura Neff, Doris Popovic, Andrea Patrignani, Jay Tracy, Ralph Schlapbach, Christiane Beckmann, Maurice Redondo, Olivier Kobel, Christoph Noppen, Sophie Seidel, Noemie Santamaria de Souza, Niko Beerenwinkel, Tanja Stadler
EPI_ISL_1119509	Viollier AG	Department of Biosystems Science and Engineering, ETH Zürich	Chaoran Chen, Sarah Nadeau, Ivan Topolsky, Emmanouil Dermitzakis, Keith Harshman, Ioannis Xenarios, Henri Pegeot, Lorenzo Cerutti, Deborah Penet, Philipp Jablonski, Lara Fuhrmann, David Dreifuss, Katharina Jahn, Christiane Beckmann, Maurice Redondo, Olivier Kobel, Christoph Noppen, Sophie Seidel, Noemie Santamaria de Souza, Niko Beerenwinkel, Tanja Stadler
EPI_ISL_1119511, EPI_ISL_1119512	Viollier AG	Department of Biosystems Science and Engineering, ETH Zürich	Chaoran Chen, Sarah Nadeau, Catharine Aquino, Ivan Topolsky, Philipp Jablonski, Lara Fuhrmann, David Dreifuss, Katharina Jahn, Andreia Cabral de Gouvea, Maria Domenica Moccia, Simon Grüter, Timothy Sykes, Lennart Opitz, Griffin White, Laura Neff, Doris Popovic, Andrea Patrignani, Jay Tracy, Ralph Schlapbach, Christiane Beckmann, Maurice Redondo, Olivier Kobel, Christoph Noppen, Sophie Seidel, Noemie Santamaria de Souza, Niko Beerenwinkel, Tanja Stadler
EPI_ISL_1119513	Viollier AG	Department of Biosystems Science and Engineering, ETH Zürich	Chaoran Chen, Sarah Nadeau, Ivan Topolsky, Emmanouil Dermitzakis, Keith Harshman, Ioannis Xenarios, Henri Pegeot, Lorenzo Cerutti, Deborah Penet, Philipp Jablonski, Lara Fuhrmann, David Dreifuss, Katharina Jahn, Christiane Beckmann, Maurice Redondo, Olivier Kobel, Christoph Noppen, Sophie Seidel, Noemie Santamaria de Souza, Niko Beerenwinkel, Tanja Stadler
EPI_ISL_1119514	Viollier AG	Department of Biosystems Science and Engineering, ETH Zürich	Chaoran Chen, Sarah Nadeau, Catharine Aquino, Ivan Topolsky, Philipp Jablonski, Lara Fuhrmann, David Dreifuss, Katharina Jahn, Andreia Cabral de Gouvea, Maria Domenica Moccia, Simon Grüter, Timothy Sykes, Lennart Opitz, Griffin White, Laura Neff, Doris Popovic, Andrea Patrignani, Jay Tracy, Ralph Schlapbach, Christiane Beckmann, Maurice Redondo, Olivier Kobel, Christoph Noppen, Sophie Seidel, Noemie Santamaria de Souza, Niko Beerenwinkel, Tanja Stadler
EPI_ISL_1119515, EPI_ISL_1119516, EPI_ISL_1119517, EPI_ISL_1119519, EPI_ISL_1119523	Viollier AG	Department of Biosystems Science and Engineering, ETH Zürich	Chaoran Chen, Sarah Nadeau, Ivan Topolsky, Emmanouil Dermitzakis, Keith Harshman, Ioannis Xenarios, Henri Pegeot, Lorenzo Cerutti, Deborah Penet, Philipp Jablonski, Lara Fuhrmann, David Dreifuss, Katharina Jahn, Christiane Beckmann, Maurice Redondo, Olivier Kobel, Christoph Noppen, Sophie Seidel, Noemie Santamaria de Souza, Niko Beerenwinkel, Tanja Stadler
EPI_ISL_1119525, EPI_ISL_1119526, EPI_ISL_1119528, EPI_ISL_1119530, EPI_ISL_1119531, EPI_ISL_1119532, EPI_ISL_1119538, EPI_ISL_1119539	Viollier AG	Department of Biosystems Science and Engineering, ETH Zürich	Chaoran Chen, Sarah Nadeau, Catharine Aquino, Ivan Topolsky, Philipp Jablonski, Lara Fuhrmann, David Dreifuss, Katharina Jahn, Andreia Cabral de Gouvea, Maria Domenica Moccia, Simon Grüter, Timothy Sykes, Lennart Opitz, Griffin White, Laura Neff, Doris Popovic, Andrea Patrignani, Jay Tracy, Ralph Schlapbach, Christiane Beckmann, Maurice Redondo, Olivier Kobel, Christoph Noppen, Sophie Seidel, Noemie Santamaria de Souza, Niko Beerenwinkel, Tanja Stadler
EPI_ISL_1119540, EPI_ISL_1119541, EPI_ISL_1119542	Viollier AG	Department of Biosystems Science and Engineering, ETH Zürich	Chaoran Chen, Sarah Nadeau, Ivan Topolsky, Emmanouil Dermitzakis, Keith Harshman, Ioannis Xenarios, Henri Pegeot, Lorenzo Cerutti, Deborah Penet, Philipp Jablonski, Lara Fuhrmann, David Dreifuss, Katharina Jahn, Christiane Beckmann, Maurice Redondo, Olivier Kobel, Christoph Noppen, Sophie Seidel, Noemie Santamaria de Souza, Niko Beerenwinkel, Tanja Stadler
EPI_ISL_1119543	Viollier AG	Department of Biosystems Science and Engineering, ETH Zürich	Chaoran Chen, Sarah Nadeau, Catharine Aquino, Ivan Topolsky, Philipp Jablonski, Lara Fuhrmann, David Dreifuss, Katharina Jahn, Andreia Cabral de Gouvea, Maria Domenica Moccia, Simon Grüter, Timothy Sykes, Lennart Opitz, Griffin White, Laura Neff, Doris Popovic, Andrea Patrignani, Jay Tracy, Ralph Schlapbach, Christiane Beckmann, Maurice Redondo, Olivier Kobel, Christoph Noppen, Sophie Seidel, Noemie Santamaria de Souza, Niko Beerenwinkel, Tanja Stadler
EPI_ISL_1119545	Viollier AG	Department of Biosystems Science and Engineering, ETH Zürich	Chaoran Chen, Sarah Nadeau, Ivan Topolsky, Emmanouil Dermitzakis, Keith Harshman, Ioannis Xenarios, Henri Pegeot, Lorenzo Cerutti, Deborah Penet, Philipp Jablonski, Lara Fuhrmann, David Dreifuss, Katharina Jahn, Christiane Beckmann, Maurice Redondo, Olivier Kobel, Christoph Noppen, Sophie Seidel, Noemie Santamaria de Souza, Niko Beerenwinkel, Tanja Stadler
EPI_ISL_1119546	Viollier AG	Department of Biosystems Science and Engineering, ETH Zürich	Chaoran Chen, Sarah Nadeau, Catharine Aquino, Ivan Topolsky, Philipp Jablonski, Lara Fuhrmann, David Dreifuss, Katharina Jahn, Andreia Cabral de Gouvea, Maria Domenica Moccia, Simon Grüter, Timothy Sykes, Lennart Opitz, Griffin White, Laura Neff, Doris Popovic, Andrea Patrignani, Jay Tracy, Ralph Schlapbach, Christiane Beckmann, Maurice Redondo, Olivier Kobel, Christoph Noppen, Sophie Seidel, Noemie Santamaria de Souza, Niko Beerenwinkel, Tanja Stadler
EPI_ISL_1119547	Viollier AG	Department of Biosystems Science and Engineering, ETH Zürich	Chaoran Chen, Sarah Nadeau, Ivan Topolsky, Emmanouil Dermitzakis, Keith Harshman, Ioannis Xenarios, Henri Pegeot, Lorenzo Cerutti, Deborah Penet, Philipp Jablonski, Lara Fuhrmann, David Dreifuss, Katharina Jahn, Christiane Beckmann, Maurice Redondo, Olivier Kobel, Christoph Noppen, Sophie Seidel, Noemie Santamaria de Souza, Niko Beerenwinkel, Tanja Stadler
EPI_ISL_1119549	Viollier AG	Department of Biosystems Science and Engineering, ETH Zürich	Chaoran Chen, Sarah Nadeau, Catharine Aquino, Ivan Topolsky, Philipp Jablonski, Lara Fuhrmann, David Dreifuss, Katharina Jahn, Andreia Cabral de Gouvea, Maria Domenica Moccia, Simon Grüter, Timothy Sykes, Lennart Opitz, Griffin White, Laura Neff, Doris Popovic, Andrea Patrignani, Jay Tracy, Ralph Schlapbach, Christiane Beckmann, Maurice Redondo, Olivier Kobel, Christoph Noppen, Sophie Seidel, Noemie Santamaria de Souza, Niko Beerenwinkel, Tanja Stadler
EPI_ISL_1119550, EPI_ISL_1119551, EPI_ISL_1119552, EPI_ISL_1119553, EPI_ISL_1119554, EPI_ISL_1119555	Viollier AG	Department of Biosystems Science and Engineering, ETH Zürich	Chaoran Chen, Sarah Nadeau, Ivan Topolsky, Emmanouil Dermitzakis, Keith Harshman, Ioannis Xenarios, Henri Pegeot, Lorenzo Cerutti, Deborah Penet, Philipp Jablonski, Lara Fuhrmann, David Dreifuss, Katharina Jahn, Christiane Beckmann, Maurice Redondo, Olivier Kobel, Christoph Noppen, Sophie Seidel, Noemie Santamaria de Souza, Niko Beerenwinkel, Tanja Stadler
EPI_ISL_1119556, EPI_ISL_1119560, EPI_ISL_1119561, EPI_ISL_1119562, EPI_ISL_1119563, EPI_ISL_1119564	Viollier AG	Department of Biosystems Science and Engineering, ETH Zürich	Chaoran Chen, Sarah Nadeau, Catharine Aquino, Ivan Topolsky, Philipp Jablonski, Lara Fuhrmann, David Dreifuss, Katharina Jahn, Andreia Cabral de Gouvea, Maria Domenica Moccia, Simon Grüter, Timothy Sykes, Lennart Opitz, Griffin White, Laura Neff, Doris Popovic, Andrea Patrignani, Jay Tracy, Ralph Schlapbach, Christiane Beckmann, Maurice Redondo, Olivier Kobel, Christoph Noppen, Sophie Seidel, Noemie Santamaria de Souza, Niko Beerenwinkel, Tanja Stadler
EPI_ISL_1119566	Viollier AG	Department of Biosystems Science and Engineering, ETH Zürich	Chaoran Chen, Sarah Nadeau, Ivan Topolsky, Emmanouil Dermitzakis, Keith Harshman, Ioannis Xenarios, Henri Pegeot, Lorenzo Cerutti, Deborah Penet, Philipp Jablonski, Lara Fuhrmann, David Dreifuss, Katharina Jahn, Christiane Beckmann, Maurice Redondo, Olivier Kobel, Christoph Noppen, Sophie Seidel, Noemie Santamaria de Souza, Niko Beerenwinkel, Tanja Stadler

EPI_ISL_1119568	Viollier AG	Department of Biosystems Science and Engineering, ETH Zürich	Chaoran Chen, Sarah Nadeau, Catharine Aquino, Ivan Topolsky, Philipp Jablonski, Lara Fuhrmann, David Dreifuss, Katharina Jahn, Andreia Cabral de Gouvea, Maria Domenica Moccia, Simon Grüter, Timothy Sykes, Lennart Opitz, Griffin White, Laura Neff, Doris Popovic, Andrea Patrignani, Jay Tracy, Ralph Schlapbach, Christiane Beckmann, Maurice Redondo, Olivier Kobel, Christoph Noppen, Sophie Seidel, Noemie Santamaria de Souza, Niko Beerenwinkel, Tanja Stadler
EPI_ISL_1119571	Viollier AG	Department of Biosystems Science and Engineering, ETH Zürich	Chaoran Chen, Sarah Nadeau, Ivan Topolsky, Emmanouil Dermitzakis, Keith Harshman, Ioannis Xenarios, Henri Pegeot, Lorenzo Cerutti, Deborah Penet, Philipp Jablonski, Lara Fuhrmann, David Dreifuss, Katharina Jahn, Christiane Beckmann, Maurice Redondo, Olivier Kobel, Christoph Noppen, Sophie Seidel, Noemie Santamaria de Souza, Niko Beerenwinkel, Tanja Stadler
EPI_ISL_1119572, EPI_ISL_1119573, EPI_ISL_1119575, EPI_ISL_1119576, EPI_ISL_1119578, EPI_ISL_1119579, EPI_ISL_1119580, EPI_ISL_1119582, EPI_ISL_1119583, EPI_ISL_1119585, EPI_ISL_1119586, EPI_ISL_1119587, EPI_ISL_1119588, EPI_ISL_1119590, EPI_ISL_1119592, EPI_ISL_1119593, EPI_ISL_1119594, EPI_ISL_1119600, EPI_ISL_1119601, EPI_ISL_1119602, EPI_ISL_1119603, EPI_ISL_1119604, EPI_ISL_1119605, EPI_ISL_1119607, EPI_ISL_1119608, EPI_ISL_1119610, EPI_ISL_1119611, EPI_ISL_1119612, EPI_ISL_1119613, EPI_ISL_1119614, EPI_ISL_1119615, EPI_ISL_1119617, EPI_ISL_1119618, EPI_ISL_1119619, EPI_ISL_1119620, EPI_ISL_1119621, EPI_ISL_1119622, EPI_ISL_1119623, EPI_ISL_1119625, EPI_ISL_1119626, EPI_ISL_1119627, EPI_ISL_1119629, EPI_ISL_1119631, EPI_ISL_1119633, EPI_ISL_1119634, EPI_ISL_1119635, EPI_ISL_1119636, EPI_ISL_1119640, EPI_ISL_1119641, EPI_ISL_1119643, EPI_ISL_1119645, EPI_ISL_1119646, EPI_ISL_1119651, EPI_ISL_1119654, EPI_ISL_1119655, EPI_ISL_1119657, EPI_ISL_1119659, EPI_ISL_1119661, EPI_ISL_1119662, EPI_ISL_1119663, EPI_ISL_1119664, EPI_ISL_1119665, EPI_ISL_1119666, EPI_ISL_1119670, EPI_ISL_1119675, EPI_ISL_1119677, EPI_ISL_1119678, EPI_ISL_1119679, EPI_ISL_1119681, EPI_ISL_1119682, EPI_ISL_1119683, EPI_ISL_1119684, EPI_ISL_1119685, EPI_ISL_1119686, EPI_ISL_1119687, EPI_ISL_1119688, EPI_ISL_1119689, EPI_ISL_1119690, EPI_ISL_1119693, EPI_ISL_1119694, EPI_ISL_1119695, EPI_ISL_1119696, EPI_ISL_1119697, EPI_ISL_1119699, EPI_ISL_1119700, EPI_ISL_1119701, EPI_ISL_1119702, EPI_ISL_1119704, EPI_ISL_1119706, EPI_ISL_1119707, EPI_ISL_1119708, EPI_ISL_1119709, EPI_ISL_1119710, EPI_ISL_1119711, EPI_ISL_1119712, EPI_ISL_1119713, EPI_ISL_1119714, EPI_ISL_1119715, EPI_ISL_1119716, EPI_ISL_1119717, EPI_ISL_1119718, EPI_ISL_1119720, EPI_ISL_1119722, EPI_ISL_1119723, EPI_ISL_1119724, EPI_ISL_1119726, EPI_ISL_1119727, EPI_ISL_1119728, EPI_ISL_1119729, EPI_ISL_1119730, EPI_ISL_1119732, EPI_ISL_1119734, EPI_ISL_1119739, EPI_ISL_1119740, EPI_ISL_1119741, EPI_ISL_1119742, EPI_ISL_1119745, EPI_ISL_1119746, EPI_ISL_1119747, EPI_ISL_1119748, EPI_ISL_1119749, EPI_ISL_1119751, EPI_ISL_1119752, EPI_ISL_1119753, EPI_ISL_1119754, EPI_ISL_1119755, EPI_ISL_1119756, EPI_ISL_1119757, EPI_ISL_1119759, EPI_ISL_1119760, EPI_ISL_1119762, EPI_ISL_1119764, EPI_ISL_1119765, EPI_ISL_1119766, EPI_ISL_1119767, EPI_ISL_1119768, EPI_ISL_1119770, EPI_ISL_1119771, EPI_ISL_1119773, EPI_ISL_1119775, EPI_ISL_1119776, EPI_ISL_1119777, EPI_ISL_1119778, EPI_ISL_1119781, EPI_ISL_1119782, EPI_ISL_1119784, EPI_ISL_1119786, EPI_ISL_1119787, EPI_ISL_1119789, EPI_ISL_1119790, EPI_ISL_1119792, EPI_ISL_1119793, EPI_ISL_1119794, EPI_ISL_1119795, EPI_ISL_1119796, EPI_ISL_1119798, EPI_ISL_1119799, EPI_ISL_1119800, EPI_ISL_1119801, EPI_ISL_1119802, EPI_ISL_1119804, EPI_ISL_1119805, EPI_ISL_1119807, EPI_ISL_1119808, EPI_ISL_1119809, EPI_ISL_1119810, EPI_ISL_1119811			
see above	Viollier AG	Department of Biosystems Science and Engineering, ETH Zürich	Chaoran Chen, Sarah Nadeau, Catharine Aquino, Ivan Topolsky, Philipp Jablonski, Lara Fuhrmann, David Dreifuss, Katharina Jahn, Andreia Cabral de Gouvea, Maria Domenica Moccia, Simon Grüter, Timothy Sykes, Lennart Opitz, Griffin White, Laura Neff, Doris Popovic, Andrea Patrignani, Jay Tracy, Ralph Schlapbach, Christiane Beckmann, Maurice Redondo, Olivier Kobel, Christoph Noppen, Sophie Seidel, Noemie Santamaria de Souza, Niko Beerenwinkel, Tanja Stadler
EPI_ISL_1119812	Viollier AG	Department of Biosystems Science and Engineering, ETH Zürich	Chaoran Chen, Sarah Nadeau, Ivan Topolsky, Emmanouil Dermitzakis, Keith Harshman, Ioannis Xenarios, Henri Pegeot, Lorenzo Cerutti, Deborah Penet, Philipp Jablonski, Lara Fuhrmann, David Dreifuss, Katharina Jahn, Christiane Beckmann, Maurice Redondo, Olivier Kobel, Christoph Noppen, Sophie Seidel, Noemie Santamaria de Souza, Niko Beerenwinkel, Tanja Stadler
EPI_ISL_1119813, EPI_ISL_1119814	Viollier AG	Department of Biosystems Science and Engineering, ETH Zürich	Chaoran Chen, Sarah Nadeau, Catharine Aquino, Ivan Topolsky, Philipp Jablonski, Lara Fuhrmann, David Dreifuss, Katharina Jahn, Andreia Cabral de Gouvea, Maria Domenica Moccia, Simon Grüter, Timothy Sykes, Lennart Opitz, Griffin White, Laura Neff, Doris Popovic, Andrea Patrignani, Jay Tracy, Ralph Schlapbach, Christiane Beckmann, Maurice Redondo, Olivier Kobel, Christoph Noppen, Sophie Seidel, Noemie Santamaria de Souza, Niko Beerenwinkel, Tanja Stadler
EPI_ISL_1119815, EPI_ISL_1119817, EPI_ISL_1119818	Viollier AG	Department of Biosystems Science and Engineering, ETH Zürich	Chaoran Chen, Sarah Nadeau, Ivan Topolsky, Emmanouil Dermitzakis, Keith Harshman, Ioannis Xenarios, Henri Pegeot, Lorenzo Cerutti, Deborah Penet, Philipp Jablonski, Lara Fuhrmann, David Dreifuss, Katharina Jahn, Christiane Beckmann, Maurice Redondo, Olivier Kobel, Christoph Noppen, Sophie Seidel, Noemie Santamaria de Souza, Niko Beerenwinkel, Tanja Stadler
EPI_ISL_1119819, EPI_ISL_1119822, EPI_ISL_1119823, EPI_ISL_1119825, EPI_ISL_1119826, EPI_ISL_1119828, EPI_ISL_1119829	Viollier AG	Department of Biosystems Science and Engineering, ETH Zürich	Chaoran Chen, Sarah Nadeau, Catharine Aquino, Ivan Topolsky, Philipp Jablonski, Lara Fuhrmann, David Dreifuss, Katharina Jahn, Andreia Cabral de Gouvea, Maria Domenica Moccia, Simon Grüter, Timothy Sykes, Lennart Opitz, Griffin White, Laura Neff, Doris Popovic, Andrea Patrignani, Jay Tracy, Ralph Schlapbach, Christiane Beckmann, Maurice Redondo, Olivier Kobel, Christoph Noppen, Sophie Seidel, Noemie Santamaria de Souza, Niko Beerenwinkel, Tanja Stadler
EPI_ISL_1119830	Viollier AG	Department of Biosystems Science and Engineering, ETH Zürich	Chaoran Chen, Sarah Nadeau, Ivan Topolsky, Emmanouil Dermitzakis, Keith Harshman, Ioannis Xenarios, Henri Pegeot, Lorenzo Cerutti, Deborah Penet, Philipp Jablonski, Lara Fuhrmann, David Dreifuss, Katharina Jahn, Christiane Beckmann, Maurice Redondo, Olivier Kobel, Christoph Noppen, Sophie Seidel, Noemie Santamaria de Souza, Niko Beerenwinkel, Tanja Stadler
EPI_ISL_1119833, EPI_ISL_1119834, EPI_ISL_1119835, EPI_ISL_1119838, EPI_ISL_1119839, EPI_ISL_1119842	Viollier AG	Department of Biosystems Science and Engineering, ETH Zürich	Chaoran Chen, Sarah Nadeau, Catharine Aquino, Ivan Topolsky, Philipp Jablonski, Lara Fuhrmann, David Dreifuss, Katharina Jahn, Andreia Cabral de Gouvea, Maria Domenica Moccia, Simon Grüter, Timothy Sykes, Lennart Opitz, Griffin White, Laura Neff, Doris Popovic, Andrea Patrignani, Jay Tracy, Ralph Schlapbach, Christiane Beckmann, Maurice Redondo, Olivier Kobel, Christoph Noppen, Sophie Seidel, Noemie Santamaria de Souza, Niko Beerenwinkel, Tanja Stadler
EPI_ISL_1119843, EPI_ISL_1119844	Viollier AG	Department of Biosystems Science and Engineering, ETH Zürich	Chaoran Chen, Sarah Nadeau, Ivan Topolsky, Emmanouil Dermitzakis, Keith Harshman, Ioannis Xenarios, Henri Pegeot, Lorenzo Cerutti, Deborah Penet, Philipp Jablonski, Lara Fuhrmann, David Dreifuss, Katharina Jahn, Christiane Beckmann, Maurice Redondo, Olivier Kobel, Christoph Noppen, Sophie Seidel, Noemie Santamaria de Souza, Niko Beerenwinkel, Tanja Stadler
EPI_ISL_1119845	Viollier AG	Department of Biosystems Science and Engineering, ETH Zürich	Chaoran Chen, Sarah Nadeau, Catharine Aquino, Ivan Topolsky, Philipp Jablonski, Lara Fuhrmann, David Dreifuss, Katharina Jahn, Andreia Cabral de Gouvea, Maria Domenica Moccia, Simon Grüter, Timothy Sykes, Lennart Opitz, Griffin White, Laura Neff, Doris Popovic, Andrea Patrignani, Jay Tracy, Ralph Schlapbach, Christiane Beckmann, Maurice Redondo, Olivier Kobel, Christoph Noppen, Sophie Seidel, Noemie Santamaria de Souza, Niko Beerenwinkel, Tanja Stadler
EPI_ISL_1119846, EPI_ISL_1119848, EPI_ISL_1119849	Viollier AG	Department of Biosystems Science and Engineering, ETH Zürich	Chaoran Chen, Sarah Nadeau, Ivan Topolsky, Emmanouil Dermitzakis, Keith Harshman, Ioannis Xenarios, Henri Pegeot, Lorenzo Cerutti, Deborah Penet, Philipp Jablonski, Lara Fuhrmann, David Dreifuss, Katharina Jahn, Christiane Beckmann, Maurice Redondo, Olivier Kobel, Christoph Noppen, Sophie Seidel, Noemie Santamaria de Souza, Niko Beerenwinkel, Tanja Stadler
EPI_ISL_1119850	Viollier AG	Department of Biosystems Science and Engineering, ETH Zürich	Chaoran Chen, Sarah Nadeau, Catharine Aquino, Ivan Topolsky, Philipp Jablonski, Lara Fuhrmann, David Dreifuss, Katharina Jahn, Andreia Cabral de Gouvea, Maria Domenica Moccia, Simon Grüter, Timothy Sykes, Lennart Opitz, Griffin White, Laura Neff, Doris Popovic, Andrea Patrignani, Jay Tracy, Ralph Schlapbach, Christiane Beckmann, Maurice Redondo, Olivier Kobel, Christoph Noppen, Sophie Seidel, Noemie Santamaria de Souza, Niko Beerenwinkel, Tanja Stadler
EPI_ISL_1119851, EPI_ISL_1119853	Viollier AG	Department of Biosystems Science and Engineering, ETH Zürich	Chaoran Chen, Sarah Nadeau, Ivan Topolsky, Emmanouil Dermitzakis, Keith Harshman, Ioannis Xenarios, Henri Pegeot, Lorenzo Cerutti, Deborah Penet, Philipp Jablonski, Lara Fuhrmann, David Dreifuss, Katharina Jahn, Christiane Beckmann, Maurice Redondo, Olivier Kobel, Christoph Noppen, Sophie Seidel, Noemie Santamaria de Souza, Niko Beerenwinkel, Tanja Stadler
EPI_ISL_1119857, EPI_ISL_1119858, EPI_ISL_1119859, EPI_ISL_1119860, EPI_ISL_1119861, EPI_ISL_1119862, EPI_ISL_1119863, EPI_ISL_1119864, EPI_ISL_1119865, EPI_ISL_1119866, EPI_ISL_1119867, EPI_ISL_1119869, EPI_ISL_1119870, EPI_ISL_1119872, EPI_ISL_1119873, EPI_ISL_1119874, EPI_ISL_1119875, EPI_ISL_1119876, EPI_ISL_1119879, EPI_ISL_1119880, EPI_ISL_1119881, EPI_ISL_1119882, EPI_ISL_1119884, EPI_ISL_1119885			
see above	Viollier AG	Department of Biosystems Science and Engineering, ETH Zürich	Chaoran Chen, Sarah Nadeau, Catharine Aquino, Ivan Topolsky, Philipp Jablonski, Lara Fuhrmann, David Dreifuss, Katharina Jahn, Andreia Cabral de Gouvea, Maria Domenica Moccia, Simon Grüter, Timothy Sykes, Lennart Opitz, Griffin White, Laura Neff, Doris Popovic, Andrea Patrignani, Jay Tracy, Ralph Schlapbach, Christiane Beckmann, Maurice Redondo, Olivier Kobel, Christoph Noppen, Sophie Seidel, Noemie Santamaria de Souza, Niko Beerenwinkel, Tanja Stadler
EPI_ISL_1119887	Viollier AG	Department of Biosystems Science and Engineering, ETH Zürich	Chaoran Chen, Sarah Nadeau, Ivan Topolsky, Emmanouil Dermitzakis, Keith Harshman, Ioannis Xenarios, Henri Pegeot, Lorenzo Cerutti, Deborah Penet, Philipp Jablonski, Lara Fuhrmann, David Dreifuss, Katharina Jahn, Christiane Beckmann, Maurice Redondo, Olivier Kobel, Christoph Noppen, Sophie Seidel, Noemie Santamaria de Souza, Niko Beerenwinkel, Tanja Stadler
EPI_ISL_1119889, EPI_ISL_1119891, EPI_ISL_1119892, EPI_ISL_1119896, EPI_ISL_1119898	Viollier AG	Department of Biosystems Science and Engineering, ETH Zürich	Chaoran Chen, Sarah Nadeau, Catharine Aquino, Ivan Topolsky, Philipp Jablonski, Lara Fuhrmann, David Dreifuss, Katharina Jahn, Andreia Cabral de Gouvea, Maria Domenica Moccia, Simon Grüter, Timothy Sykes, Lennart Opitz, Griffin White, Laura Neff, Doris Popovic, Andrea Patrignani, Jay Tracy, Ralph Schlapbach, Christiane Beckmann, Maurice Redondo, Olivier Kobel, Christoph Noppen, Sophie Seidel, Noemie Santamaria de Souza, Niko Beerenwinkel, Tanja Stadler
EPI_ISL_1119903, EPI_ISL_1119904, EPI_ISL_1119905, EPI_ISL_1119908, EPI_ISL_1119909, EPI_ISL_1119910, EPI_ISL_1119911, EPI_ISL_1119912, EPI_ISL_1119913, EPI_ISL_1119914, EPI_ISL_1119916, EPI_ISL_1119917, EPI_ISL_1119919, EPI_ISL_1119920, EPI_ISL_1119921, EPI_ISL_1119922, EPI_ISL_1119925, EPI_ISL_1119926, EPI_ISL_1119927, EPI_ISL_1119930, EPI_ISL_1119931, EPI_ISL_1119932, EPI_ISL_1119934, EPI_ISL_1119935, EPI_ISL_1119936, EPI_ISL_1119937, EPI_ISL_1119940, EPI_ISL_1119941, EPI_ISL_1119943, EPI_ISL_1119944, EPI_ISL_1119946, EPI_ISL_1119947, EPI_ISL_1119949, EPI_ISL_1119950, EPI_ISL_1119951, EPI_ISL_1119952			
see above	Viollier AG	Department of Biosystems Science and Engineering, ETH Zürich	Chaoran Chen, Sarah Nadeau, Ivan Topolsky, Emmanouil Dermitzakis, Keith Harshman, Ioannis Xenarios, Henri Pegeot, Lorenzo Cerutti, Deborah Penet, Philipp Jablonski, Lara Fuhrmann, David Dreifuss, Katharina Jahn, Christiane Beckmann, Maurice Redondo, Olivier Kobel, Christoph Noppen, Sophie

			Seidel, Noemie Santamaria de Souza, Niko Beerenwinkel, Tanja Stadler
EPI_ISL_1122011, EPI_ISL_1122012	Kentucky State Public Health Lab	Kentucky State Public Health Lab	Stephanie Lunn, Karim George, Joshua Tobias, William Grooms, Vaneet Arora, Matthew Johnson, Rachel Zinner, Rhonda Lucas
EPI_ISL_1122349, EPI_ISL_1122350, EPI_ISL_1122351, EPI_ISL_1122352, EPI_ISL_1122353, EPI_ISL_1122354	Ibaraki Prefectural Institute of Public Health	Ibaraki Prefectural Institute of Public Health	Keiko Goto, Tsuyoshi Sekizuka, Kentaro Itokawa, Rina Tanaka, Masanori Hashino, Makoto Kuroda
EPI_ISL_1123264	Rangamati General Hospital RT-PCR lab	Central Biological Research Laboratory and Department of Biochemistry and Molecular Biology	Mohammad Omar Faruque, H. M. Abdullah Al Masud, Imam Hossen, Md. Khondakar Raziur Rahman, Sajib Rudra, Md. Arif Hossain, Shanta Paul, Md. Omer Faruq, Robiul Hasan Bhuiyan, Md. Imranul Hoq
EPI_ISL_1123266	Cox's Bazar Medical College	Central Biological Research Laboratory and Department of Biochemistry and Molecular Biology	Mohammad Omar Faruque, H. M. Abdullah Al Masud, Sajib Rudra, Md. Omer Faruq, Md. Khondakar Raziur Rahman, Imam Hossen, Md. Arif Hossain, Shanta Paul, Robiul Hasan Bhuiyan, Md. Imranul Hoq
EPI_ISL_1123267	Cox's Bazar Medical College	Central Biological Research Laboratory and Department of Biochemistry and Molecular Biology	H. M. Abdullah Al Masud, Mohammad Omar Faruque, Sajib Rudra, Md. Khondakar Raziur Rahman, Imam Hossen, Md. Arif Hossain, Shanta Paul, Md. Omer Faruq, Md. Imranul Hoq, Robiul Hasan Bhuiyan
EPI_ISL_1123268	Chattogram Veterinary and Animal Sciences University	Central Biological Research Laboratory and Department of Biochemistry and Molecular Biology	H. M. Abdullah Al Masud, Mohammad Omar Faruque, Md. Arif Hossain, Shanta Paul, Md. Khondakar Raziur Rahman, Imam Hossen, Sajib Rudra, Md. Omer Faruq, Md. Imranul Hoq, Robiul Hasan Bhuiyan
EPI_ISL_1123677, EPI_ISL_1123816, EPI_ISL_1123862	Department of Virus and Microbiological Special Diagnostics, Statens Serum Institut, Copenhagen, Denmark	Aalborg University	Danish Covid-19 Genome Consortium
EPI_ISL_1123895, EPI_ISL_1123897, EPI_ISL_1123898	Pathogen Genomics Center, National Institute of Infectious Diseases	Pathogen Genomics Center, National Institute of Infectious Diseases	Tsuyoshi Sekizuka, Kentaro Itokawa, Rina Tanaka, Masanori Hashino, Makoto Kuroda
EPI_ISL_1123899	Department of Virus and Microbiological Special Diagnostics, Statens Serum Institut, Copenhagen, Denmark	Aalborg University	Danish Covid-19 Genome Consortium
EPI_ISL_1123900, EPI_ISL_1123902, EPI_ISL_1123904, EPI_ISL_1123905, EPI_ISL_1123907	Pathogen Genomics Center, National Institute of Infectious Diseases	Pathogen Genomics Center, National Institute of Infectious Diseases	Tsuyoshi Sekizuka, Kentaro Itokawa, Rina Tanaka, Masanori Hashino, Makoto Kuroda
EPI_ISL_1123930, EPI_ISL_1123939, EPI_ISL_1124132, EPI_ISL_1124134, EPI_ISL_1124136, EPI_ISL_1124169	Department of Virus and Microbiological Special Diagnostics, Statens Serum Institut, Copenhagen, Denmark	Aalborg University	Danish Covid-19 Genome Consortium
EPI_ISL_1124199	Toyama Institute of Health	Pathogen Genomics Center, National Institute of Infectious Diseases	Tsuyoshi Sekizuka, Kentaro Itokawa, Rina Tanaka, Masanori Hashino, Makoto Kuroda
EPI_ISL_1124249, EPI_ISL_1124326	Department of Virus and Microbiological Special Diagnostics, Statens Serum Institut, Copenhagen, Denmark	Aalborg University	Danish Covid-19 Genome Consortium
EPI_ISL_1124337, EPI_ISL_1124339, EPI_ISL_1124341, EPI_ISL_1124342, EPI_ISL_1124344, EPI_ISL_1124346, EPI_ISL_1124348, EPI_ISL_1124349, EPI_ISL_1124351, EPI_ISL_1124353, EPI_ISL_1124355, EPI_ISL_1124356			
see above	Osaka Institute of Public Health, Morinomiya Center	Pathogen Genomics Center, National Institute of Infectious Diseases	Tsuyoshi Sekizuka, Kentaro Itokawa, Rina Tanaka, Masanori Hashino, Makoto Kuroda
EPI_ISL_1124425, EPI_ISL_1124466, EPI_ISL_1124473	Department of Virus and Microbiological Special Diagnostics, Statens Serum Institut, Copenhagen, Denmark	Aalborg University	Danish Covid-19 Genome Consortium
EPI_ISL_1124508, EPI_ISL_1124510, EPI_ISL_1124512, EPI_ISL_1124514, EPI_ISL_1124516	Pathogen Genomics Center, National Institute of Infectious Diseases	Pathogen Genomics Center, National Institute of Infectious Diseases	Tsuyoshi Sekizuka, Kentaro Itokawa, Rina Tanaka, Masanori Hashino, Makoto Kuroda
EPI_ISL_1124649, EPI_ISL_1124780, EPI_ISL_1124790, EPI_ISL_1125022, EPI_ISL_1125024, EPI_ISL_1125048, EPI_ISL_1125075, EPI_ISL_1125080, EPI_ISL_1125121, EPI_ISL_1125199, EPI_ISL_1125202, EPI_ISL_1125251, EPI_ISL_1125292, EPI_ISL_1125333, EPI_ISL_1125452, EPI_ISL_1125459, EPI_ISL_1125464, EPI_ISL_1125487, EPI_ISL_1125505, EPI_ISL_1125521, EPI_ISL_1125548, EPI_ISL_1125564, EPI_ISL_1125584, EPI_ISL_1125618, EPI_ISL_1125657, EPI_ISL_1125663, EPI_ISL_1125683, EPI_ISL_1125702, EPI_ISL_1125744, EPI_ISL_1125795, EPI_ISL_1125814, EPI_ISL_1125857, EPI_ISL_1125887, EPI_ISL_1125891, EPI_ISL_1125908, EPI_ISL_1125937, EPI_ISL_1126007, EPI_ISL_1126017, EPI_ISL_1126033, EPI_ISL_1126037, EPI_ISL_1126058, EPI_ISL_1126153, EPI_ISL_1126171, EPI_ISL_1126176, EPI_ISL_1126190, EPI_ISL_1126201, EPI_ISL_1126218, EPI_ISL_1126313, EPI_ISL_1126372, EPI_ISL_1126377, EPI_ISL_1126560, EPI_ISL_1126582, EPI_ISL_1126656, EPI_ISL_1126675, EPI_ISL_1126699, EPI_ISL_1126753, EPI_ISL_1126760, EPI_ISL_1126790, EPI_ISL_1126953, EPI_ISL_1126961, EPI_ISL_1126962, EPI_ISL_1126981, EPI_ISL_1127014, EPI_ISL_1127015			
see above	Department of Virus and Microbiological Special Diagnostics, Statens Serum Institut, Copenhagen, Denmark	Aalborg University	Danish Covid-19 Genome Consortium
EPI_ISL_1127119, EPI_ISL_1127120, EPI_ISL_1127155	Pathogen Genomics Center, National Institute of Infectious Diseases	Pathogen Genomics Center, National Institute of Infectious Diseases	Tsuyoshi Sekizuka, Kentaro Itokawa, Rina Tanaka, Masanori Hashino, Makoto Kuroda
EPI_ISL_1127157	Osaka Institute of Public Health, Morinomiya Center	Pathogen Genomics Center, National Institute of Infectious Diseases	Tsuyoshi Sekizuka, Kentaro Itokawa, Rina Tanaka, Masanori Hashino, Makoto Kuroda
EPI_ISL_1127396, EPI_ISL_1127397, EPI_ISL_1127398, EPI_ISL_1127399, EPI_ISL_1127400, EPI_ISL_1127401, EPI_ISL_1127402, EPI_ISL_1127403, EPI_ISL_1127404, EPI_ISL_1127405, EPI_ISL_1127406, EPI_ISL_1127407, EPI_ISL_1127408, EPI_ISL_1127409, EPI_ISL_1127410, EPI_ISL_1127411, EPI_ISL_1127412, EPI_ISL_1127413, EPI_ISL_1127414, EPI_ISL_1127415, EPI_ISL_1127416, EPI_ISL_1127417, EPI_ISL_1127418, EPI_ISL_1127419, EPI_ISL_1127420, EPI_ISL_1127421, EPI_ISL_1127422, EPI_ISL_1127423, EPI_ISL_1127424, EPI_ISL_1127425, EPI_ISL_1127426, EPI_ISL_1127427, EPI_ISL_1127428, EPI_ISL_1127429, EPI_ISL_1127430, EPI_ISL_1127431, EPI_ISL_1127432, EPI_ISL_1127433, EPI_ISL_1127434, EPI_ISL_1127435, EPI_ISL_1127436, EPI_ISL_1127437, EPI_ISL_1127438, EPI_ISL_1127439, EPI_ISL_1127440, EPI_ISL_1127441, EPI_ISL_1127442, EPI_ISL_1127443, EPI_ISL_1127444, EPI_ISL_1127445, EPI_ISL_1127446, EPI_ISL_1127447, EPI_ISL_1127448, EPI_ISL_1127449, EPI_ISL_1127450, EPI_ISL_1127451, EPI_ISL_1127681, EPI_ISL_1127682, EPI_ISL_1127683, EPI_ISL_1127684, EPI_ISL_1127685, EPI_ISL_1127686, EPI_ISL_1127687, EPI_ISL_1127688, EPI_ISL_1127689, EPI_ISL_1127690, EPI_ISL_1127691, EPI_ISL_1127692, EPI_ISL_1127693, EPI_ISL_1127694, EPI_ISL_1127695, EPI_ISL_1127696, EPI_ISL_1127697, EPI_ISL_1127698, EPI_ISL_1127699, EPI_ISL_1127700, EPI_ISL_1127701, EPI_ISL_1127702, EPI_ISL_1127703, EPI_ISL_1127704, EPI_ISL_1127705, EPI_ISL_1127706, EPI_ISL_1127707, EPI_ISL_1127708, EPI_ISL_1127709, EPI_ISL_1127710, EPI_ISL_1127711, EPI_ISL_1127712, EPI_ISL_1127713, EPI_ISL_1127714, EPI_ISL_1127715, EPI_ISL_1127716, EPI_ISL_1127717, EPI_ISL_1127718, EPI_ISL_1127719, EPI_ISL_1127720, EPI_ISL_1127721, EPI_ISL_1127722, EPI_ISL_1127723, EPI_ISL_1127724, EPI_ISL_1127725, EPI_ISL_1127726, EPI_ISL_1127727, EPI_ISL_1127728, EPI_ISL_1127729, EPI_ISL_1127730, EPI_ISL_1127731, EPI_ISL_1127732, EPI_ISL_1127733, EPI_ISL_1127734, EPI_ISL_1127735, EPI_ISL_1127736, EPI_ISL_1127737, EPI_ISL_1127738			
see above	Pathogen Genomics Center, National Institute of Infectious Diseases	Pathogen Genomics Center, National Institute of Infectious Diseases	Tsuyoshi Sekizuka, Kentaro Itokawa, Rina Tanaka, Masanori Hashino, Makoto Kuroda
EPI_ISL_1127947, EPI_ISL_1127948, EPI_ISL_1127949, EPI_ISL_1127950, EPI_ISL_1127951, EPI_ISL_1127952, EPI_ISL_1127953	Osaka Institute of Public Health, Morinomiya Center	Pathogen Genomics Center, National Institute of Infectious Diseases	Tsuyoshi Sekizuka, Kentaro Itokawa, Rina Tanaka, Masanori Hashino, Makoto Kuroda
EPI_ISL_1127999	Kanagawa Prefectural Institute of Public Health	Pathogen Genomics Center, National Institute of Infectious Diseases	Tsuyoshi Sekizuka, Kentaro Itokawa, Rina Tanaka, Masanori Hashino, Makoto Kuroda
EPI_ISL_1128176, EPI_ISL_1128273, EPI_ISL_1128274, EPI_ISL_1128275, EPI_ISL_1128276, EPI_ISL_1128277, EPI_ISL_1128278, EPI_ISL_1128279, EPI_ISL_1128280, EPI_ISL_1128281, EPI_ISL_1128599, EPI_ISL_1128600, EPI_ISL_1128601, EPI_ISL_1128602, EPI_ISL_1128761, EPI_ISL_1128762, EPI_ISL_1128763, EPI_ISL_1128764, EPI_ISL_1128765, EPI_ISL_1128766, EPI_ISL_1128767, EPI_ISL_1128768, EPI_ISL_1129034, EPI_ISL_1129035, EPI_ISL_1129036, EPI_ISL_1129037, EPI_ISL_1129038, EPI_ISL_1129039, EPI_ISL_1129040, EPI_ISL_1129041, EPI_ISL_1129042, EPI_ISL_1129043, EPI_ISL_1129044, EPI_ISL_1129045, EPI_ISL_1129046, EPI_ISL_1129047, EPI_ISL_1129048, EPI_ISL_1129049, EPI_ISL_1129050, EPI_ISL_1129051, EPI_ISL_1129052, EPI_ISL_1129053, EPI_ISL_1129054, EPI_ISL_1129055, EPI_ISL_1129056, EPI_ISL_1129057, EPI_ISL_1129073, EPI_ISL_1129074, EPI_ISL_1129075, EPI_ISL_1129076, EPI_ISL_1129077, EPI_ISL_1129078, EPI_ISL_1129079, EPI_ISL_1129080, EPI_ISL_1129081, EPI_ISL_1129082, EPI_ISL_1129083, EPI_ISL_1129084, EPI_ISL_1129085, EPI_ISL_1129086, EPI_ISL_1129087, EPI_ISL_1129088, EPI_ISL_1129094, EPI_ISL_1129095, EPI_ISL_1129096, EPI_ISL_1129097, EPI_ISL_1129098, EPI_ISL_1129099, EPI_ISL_1129104, EPI_ISL_1129105, EPI_ISL_1129106, EPI_ISL_1129107, EPI_ISL_1129108, EPI_ISL_1129109, EPI_ISL_1129127, EPI_ISL_1129128, EPI_ISL_1129129, EPI_ISL_1129151, EPI_ISL_1129152, EPI_ISL_1129153, EPI_ISL_1129154, EPI_ISL_1129155, EPI_ISL_1129156, EPI_ISL_1129157, EPI_ISL_1129158, EPI_ISL_1129159, EPI_ISL_1129160, EPI_ISL_1129161, EPI_ISL_1129162, EPI_ISL_1129163, EPI_ISL_1129164, EPI_ISL_1129177, EPI_ISL_1129178, EPI_ISL_1129179, EPI_ISL_1129180, EPI_ISL_1129181, EPI_ISL_1129182, EPI_ISL_1129183, EPI_ISL_1129184, EPI_ISL_1129185, EPI_ISL_1129198, EPI_ISL_1129199, EPI_ISL_1129200, EPI_ISL_1129201, EPI_ISL_1129202, EPI_ISL_1129203, EPI_ISL_1129212, EPI_ISL_1129213, EPI_ISL_1129214			
see above	Pathogen Genomics Center, National Institute of Infectious Diseases	Pathogen Genomics Center, National Institute of Infectious Diseases	Tsuyoshi Sekizuka, Kentaro Itokawa, Rina Tanaka, Masanori Hashino, Makoto Kuroda
EPI_ISL_1129310	Viollier AG	Department of Biosystems Science and Engineering, ETH Zürich	Christian Beisel, Sarah Nadeau, Chaoran Chen, Ivan Topolsky, Philipp Jablonski, Lara Fuhrmann, David Dreifuss, Katharina Jahn, Rebecca Denes, Mirjam Feldkamp, Ina Nissen, Natascha Santacroce, Elodie Burcklen, Christiane Beckmann, Maurice Redondo, Olivier Kobel, Christoph Noppen, Sophie Seidel,

			Noemie Santamaria de Souza, Niko Beerenwinkel, Tanja Stadler
EPI_ISL_1129587	Viollier AG	Department of Biosystems Science and Engineering, ETH Zürich	Chaoran Chen, Sarah Nadeau, Catharine Aquino, Ivan Topolsky, Philipp Jablonski, Lara Fuhrmann, David Dreifuss, Katharina Jahn, Andreia Cabral de Gouvea, Maria Domenica Moccia, Simon Grüter, Timothy Sykes, Lennart Opitz, Griffin White, Laura Neff, Doris Popovic, Andrea Patrignani, Jay Tracy, Ralph Schlapbach, Christiane Beckmann, Maurice Redondo, Olivier Kobel, Christoph Noppen, Sophie Seidel, Noemie Santamaria de Souza, Niko Beerenwinkel, Tanja Stadler
EPI_ISL_1129936, EPI_ISL_1129938, EPI_ISL_1130045, EPI_ISL_1130111, EPI_ISL_1130203	Viollier AG	Department of Biosystems Science and Engineering, ETH Zürich	Christian Beisel, Sarah Nadeau, Chaoran Chen, Ivan Topolsky, Philipp Jablonski, Lara Fuhrmann, David Dreifuss, Katharina Jahn, Rebecca Denes, Mirjam Feldkamp, Ina Nissen, Natascha Santacroce, Elodie Burcklen, Christiane Beckmann, Maurice Redondo, Olivier Kobel, Christoph Noppen, Sophie Seidel, Noemie Santamaria de Souza, Niko Beerenwinkel, Tanja Stadler
EPI_ISL_1130220	Viollier AG	Department of Biosystems Science and Engineering, ETH Zürich	Chaoran Chen, Sarah Nadeau, Catharine Aquino, Ivan Topolsky, Philipp Jablonski, Lara Fuhrmann, David Dreifuss, Katharina Jahn, Andreia Cabral de Gouvea, Maria Domenica Moccia, Simon Grüter, Timothy Sykes, Lennart Opitz, Griffin White, Laura Neff, Doris Popovic, Andrea Patrignani, Jay Tracy, Ralph Schlapbach, Christiane Beckmann, Maurice Redondo, Olivier Kobel, Christoph Noppen, Sophie Seidel, Noemie Santamaria de Souza, Niko Beerenwinkel, Tanja Stadler
EPI_ISL_1130724	Viollier AG	Department of Biosystems Science and Engineering, ETH Zürich	Christian Beisel, Sarah Nadeau, Chaoran Chen, Ivan Topolsky, Philipp Jablonski, Lara Fuhrmann, David Dreifuss, Katharina Jahn, Rebecca Denes, Mirjam Feldkamp, Ina Nissen, Natascha Santacroce, Elodie Burcklen, Christiane Beckmann, Maurice Redondo, Olivier Kobel, Christoph Noppen, Sophie Seidel, Noemie Santamaria de Souza, Niko Beerenwinkel, Tanja Stadler
EPI_ISL_1130791, EPI_ISL_1130804, EPI_ISL_1131003	Viollier AG	Department of Biosystems Science and Engineering, ETH Zürich	Chaoran Chen, Sarah Nadeau, Catharine Aquino, Ivan Topolsky, Philipp Jablonski, Lara Fuhrmann, David Dreifuss, Katharina Jahn, Andreia Cabral de Gouvea, Maria Domenica Moccia, Simon Grüter, Timothy Sykes, Lennart Opitz, Griffin White, Laura Neff, Doris Popovic, Andrea Patrignani, Jay Tracy, Ralph Schlapbach, Christiane Beckmann, Maurice Redondo, Olivier Kobel, Christoph Noppen, Sophie Seidel, Noemie Santamaria de Souza, Niko Beerenwinkel, Tanja Stadler
EPI_ISL_1131145, EPI_ISL_1131155, EPI_ISL_1131156, EPI_ISL_1131159, EPI_ISL_1131160, EPI_ISL_1131178, EPI_ISL_1131179, EPI_ISL_1131180, EPI_ISL_1131181, EPI_ISL_1131182, EPI_ISL_1131183, EPI_ISL_1131184, EPI_ISL_1131185, EPI_ISL_1131190, EPI_ISL_1131191, EPI_ISL_1131192, EPI_ISL_1131193, EPI_ISL_1131194, EPI_ISL_1131195, EPI_ISL_1131196, EPI_ISL_1131197, EPI_ISL_1131198, EPI_ISL_1131199, EPI_ISL_1131200, EPI_ISL_1131201, EPI_ISL_1131202, EPI_ISL_1131203, EPI_ISL_1131204, EPI_ISL_1131205, EPI_ISL_1131206, EPI_ISL_1131207, EPI_ISL_1131208, EPI_ISL_1131209, EPI_ISL_1131210, EPI_ISL_1131211, EPI_ISL_1131212, EPI_ISL_1131213, EPI_ISL_1131214, EPI_ISL_1131215, EPI_ISL_1131225, EPI_ISL_1131226, EPI_ISL_1131227, EPI_ISL_1131228, EPI_ISL_1131229, EPI_ISL_1131230, EPI_ISL_1131231, EPI_ISL_1131232, EPI_ISL_1131233, EPI_ISL_1131234, EPI_ISL_1131235, EPI_ISL_1131236, EPI_ISL_1131237, EPI_ISL_1131238, EPI_ISL_1131239, EPI_ISL_1131240, EPI_ISL_1131241, EPI_ISL_1131242, EPI_ISL_1131243, EPI_ISL_1131244, EPI_ISL_1131245, EPI_ISL_1131246, EPI_ISL_1131247, EPI_ISL_1131248, EPI_ISL_1131249, EPI_ISL_1131380, EPI_ISL_1131612, EPI_ISL_1131613, EPI_ISL_1131614, EPI_ISL_1131615, EPI_ISL_1131616, EPI_ISL_1131617, EPI_ISL_1131618, EPI_ISL_1131619, EPI_ISL_1131620, EPI_ISL_1131621, EPI_ISL_1131622, EPI_ISL_1131623, EPI_ISL_1131624, EPI_ISL_1131625, EPI_ISL_1131626, EPI_ISL_1131627, EPI_ISL_1131628, EPI_ISL_1131629, EPI_ISL_1131630, EPI_ISL_1131631, EPI_ISL_1131632, EPI_ISL_1131633, EPI_ISL_1131634, EPI_ISL_1131635, EPI_ISL_1131636, EPI_ISL_1131637, EPI_ISL_1131638, EPI_ISL_1131639, EPI_ISL_1131640, EPI_ISL_1131641, EPI_ISL_1131642, EPI_ISL_1131643, EPI_ISL_1131644, EPI_ISL_1131645, EPI_ISL_1131646, EPI_ISL_1131647, EPI_ISL_1131648, EPI_ISL_1131649, EPI_ISL_1131650, EPI_ISL_1131651, EPI_ISL_1131652, EPI_ISL_1131653, EPI_ISL_1131654, EPI_ISL_1131655, EPI_ISL_1131656, EPI_ISL_1131657, EPI_ISL_1131658, EPI_ISL_1131659, EPI_ISL_1131660, EPI_ISL_1131661, EPI_ISL_1131662, EPI_ISL_1131663, EPI_ISL_1131664, EPI_ISL_1131665, EPI_ISL_1131666, EPI_ISL_1131667, EPI_ISL_1131668, EPI_ISL_1131669, EPI_ISL_1131670, EPI_ISL_1131671, EPI_ISL_1131672, EPI_ISL_1131673, EPI_ISL_1131674, EPI_ISL_1131675, EPI_ISL_1131676, EPI_ISL_1131677, EPI_ISL_1131678, EPI_ISL_1131679, EPI_ISL_1131680, EPI_ISL_1131681, EPI_ISL_1131682, EPI_ISL_1131683, EPI_ISL_1131684, EPI_ISL_1131685, EPI_ISL_1131686, EPI_ISL_1131687, EPI_ISL_1131688, EPI_ISL_1131689, EPI_ISL_1131690, EPI_ISL_1131691, EPI_ISL_1131692, EPI_ISL_1131693, EPI_ISL_1131694, EPI_ISL_1131695, EPI_ISL_1131696, EPI_ISL_1131697, EPI_ISL_1131698, EPI_ISL_1131699, EPI_ISL_1131700, EPI_ISL_1131701, EPI_ISL_1131702, EPI_ISL_1131703, EPI_ISL_1131704, EPI_ISL_1131705, EPI_ISL_1131706, EPI_ISL_1131707, EPI_ISL_1131708, EPI_ISL_1131709, EPI_ISL_1131710, EPI_ISL_1131711, EPI_ISL_1131712, EPI_ISL_1131713, EPI_ISL_1131714			
see above	Pathogen Genomics Center, National Institute of Infectious Diseases	Pathogen Genomics Center, National Institute of Infectious Diseases	Tsuyoshi Sekizuka, Kentaro Itokawa, Rina Tanaka, Masanori Hashino, Makoto Kuroda
EPI_ISL_1132648	Arizona State Public Health Laboratory	Arizona State Public Health Laboratory	Trung Huynh, Jessica Escobar, Katherine Fullerton, Nobuko Fukushima, Stacy White, Linda Getsinger, Victor Waddell
EPI_ISL_1132971, EPI_ISL_1132973, EPI_ISL_1132975, EPI_ISL_1132978, EPI_ISL_1132987, EPI_ISL_1132988, EPI_ISL_1132990, EPI_ISL_1132992, EPI_ISL_1132998, EPI_ISL_1133001, EPI_ISL_1133003, EPI_ISL_1133004, EPI_ISL_1133005, EPI_ISL_1133006, EPI_ISL_1133014, EPI_ISL_1133017, EPI_ISL_1133018, EPI_ISL_1133019, EPI_ISL_1133020, EPI_ISL_1133021, EPI_ISL_1133022, EPI_ISL_1133023, EPI_ISL_1133027, EPI_ISL_1133028, EPI_ISL_1133030, EPI_ISL_1133033, EPI_ISL_1133035, EPI_ISL_1133038, EPI_ISL_1133039, EPI_ISL_1133040, EPI_ISL_1133041, EPI_ISL_1133042, EPI_ISL_1133044, EPI_ISL_1133045, EPI_ISL_1133047, EPI_ISL_1133048, EPI_ISL_1133049, EPI_ISL_1133051, EPI_ISL_1133052, EPI_ISL_1133053, EPI_ISL_1133057, EPI_ISL_1133058, EPI_ISL_1133059, EPI_ISL_1133062, EPI_ISL_1133063, EPI_ISL_1133067, EPI_ISL_1133068, EPI_ISL_1133069, EPI_ISL_1133070, EPI_ISL_1133071, EPI_ISL_1133072, EPI_ISL_1133073, EPI_ISL_1133074, EPI_ISL_1133076, EPI_ISL_1133077, EPI_ISL_1133078, EPI_ISL_1133079, EPI_ISL_1133080, EPI_ISL_1133082, EPI_ISL_1133083, EPI_ISL_1133084, EPI_ISL_1133085, EPI_ISL_1133086, EPI_ISL_1133087, EPI_ISL_1133089, EPI_ISL_1133090, EPI_ISL_1133091, EPI_ISL_1133092, EPI_ISL_1133093, EPI_ISL_1133094, EPI_ISL_1133095, EPI_ISL_1133096, EPI_ISL_1133097, EPI_ISL_1133098, EPI_ISL_1133099			
see above	University of Michigan Clinical Microbiology Laboratory	Lauring Lab, University of Michigan, Department of Microbiology and Immunology	Valesano
EPI_ISL_1133222	Arizona State Public Health Laboratory	Arizona State Public Health Laboratory	Trung Huynh, Jessica Escobar, Katherine Fullerton, Nobuko Fukushima, Stacy White, Linda Getsinger, Victor Waddell
EPI_ISL_1137039, EPI_ISL_1137040, EPI_ISL_1137044, EPI_ISL_1137047	Delaware Public Health Lab	Delaware Public Health Lab	Gregory Hovan
EPI_ISL_1137173, EPI_ISL_1137209, EPI_ISL_1137210, EPI_ISL_1137211, EPI_ISL_1137212, EPI_ISL_1137213, EPI_ISL_1137214, EPI_ISL_1137215, EPI_ISL_1137216, EPI_ISL_1137217, EPI_ISL_1137218, EPI_ISL_1137219, EPI_ISL_1137221, EPI_ISL_1137222, EPI_ISL_1137223, EPI_ISL_1137224, EPI_ISL_1137225, EPI_ISL_1137226, EPI_ISL_1137227, EPI_ISL_1137229, EPI_ISL_1137230, EPI_ISL_1137231, EPI_ISL_1137232, EPI_ISL_1137233, EPI_ISL_1137234, EPI_ISL_1137235, EPI_ISL_1137236, EPI_ISL_1137237, EPI_ISL_1137238			
see above	UPMC Clinical Microbiology Laboratory	Microbial Genome Sequencing Center; Microbial Genomic Epidemiology Laboratory, University of Pittsburgh	Lee H. Harrison, Jane W. Marsh, Marissa P. Griffith, Stephanie L. Mitchell, Vatsala R. Srinivasa, Kady D. Waggle, Daniel J. Snyder, Vaughn S. Cooper
EPI_ISL_1137479, EPI_ISL_1137480, EPI_ISL_1137481, EPI_ISL_1137482, EPI_ISL_1137483, EPI_ISL_1137484, EPI_ISL_1137485, EPI_ISL_1137486, EPI_ISL_1137487, EPI_ISL_1137488, EPI_ISL_1137489, EPI_ISL_1137490, EPI_ISL_1137491			
see above	Laboratorio de Referencia Nacional de Virus Respiratorio. Instituto Nacional de Salud Perú	Laboratorio de Referencia Nacional de Biotecnología y Biología Molecular. Instituto Nacional de Salud Perú	Carlos Padilla Rojas, Karolyn Vega Chozo, Luis Barcena, Priscila Lope Pari, Omar Caceres Rey, Marco Galarza Perez, Maribel Huaringa Nuñez, Johanna Balbuena Torrez, Henri Bailon Calderon, Nancy Rojas Serrano
EPI_ISL_1138510	Illinois Department of Public Health	Gagnon Lab, Southern Illinois University	Keith Gagnon
EPI_ISL_1138600, EPI_ISL_1138601, EPI_ISL_1138602, EPI_ISL_1138603, EPI_ISL_1138604, EPI_ISL_1138607, EPI_ISL_1138608, EPI_ISL_1138609, EPI_ISL_1138610, EPI_ISL_1138611, EPI_ISL_1138615, EPI_ISL_1138616, EPI_ISL_1138617, EPI_ISL_1138621, EPI_ISL_1138622, EPI_ISL_1138623, EPI_ISL_1138624, EPI_ISL_1138625, EPI_ISL_1138628, EPI_ISL_1138629, EPI_ISL_1138630, EPI_ISL_1138632, EPI_ISL_1138635, EPI_ISL_1138636, EPI_ISL_1138637, EPI_ISL_1138638, EPI_ISL_1138639, EPI_ISL_1138640, EPI_ISL_1138643, EPI_ISL_1138644, EPI_ISL_1138645, EPI_ISL_1138646, EPI_ISL_1138647, EPI_ISL_1138651, EPI_ISL_1138653, EPI_ISL_1138654, EPI_ISL_1138657, EPI_ISL_1138658, EPI_ISL_1138659, EPI_ISL_1138660, EPI_ISL_1138664, EPI_ISL_1138665, EPI_ISL_1138666, EPI_ISL_1138667, EPI_ISL_1138671, EPI_ISL_1138672, EPI_ISL_1138673, EPI_ISL_1138674, EPI_ISL_1138677, EPI_ISL_1138678, EPI_ISL_1138679, EPI_ISL_1138681			
see above	Institut für Medizinische Virologie, Universitätsklinikum Frankfurt	Institut für Medizinische Virologie, Universitätsklinikum Frankfurt	Barbara Muehlemann et al
EPI_ISL_1140353, EPI_ISL_1140355, EPI_ISL_1140356, EPI_ISL_1140359, EPI_ISL_1140362, EPI_ISL_1140364, EPI_ISL_1140365	Labor ZOTZ|KLIMAS; MVZ Düsseldorf-Centrum	Robert Koch Institute	unknown
EPI_ISL_1141013, EPI_ISL_1141015	Labor Kneißler GmbH & Co. KG	Robert Koch Institute	unknown
EPI_ISL_1143958	Bayerisches Landesamt für Gesundheit und Lebensmittelsicherheit (LGL)	Robert Koch Institute	unknown
EPI_ISL_1144913, EPI_ISL_1144914, EPI_ISL_1144915, EPI_ISL_1144917, EPI_ISL_1144918, EPI_ISL_1144919, EPI_ISL_1144920, EPI_ISL_1144921, EPI_ISL_1144922	Medizinische Hochschule Hannover Institut für Virologie	Robert Koch Institute	unknown
EPI_ISL_1159071, EPI_ISL_1159072, EPI_ISL_1159073, EPI_ISL_1159074, EPI_ISL_1159075, EPI_ISL_1159076, EPI_ISL_1159077, EPI_ISL_1159078, EPI_ISL_1159079, EPI_ISL_1159080, EPI_ISL_1159083, EPI_ISL_1159085, EPI_ISL_1159087, EPI_ISL_1159089, EPI_ISL_1159091, EPI_ISL_1159092, EPI_ISL_1159096, EPI_ISL_1159102			
see above	Department of Health Technology and Informatics, The Hong Kong Polytechnic University	Department of Health Technology and Informatics, The Hong Kong Polytechnic University	Gilman Kit-Hang Siu, Lam-Kwong Lee, Kenneth Siu-Sing Leung, Jake Siu-Lun Leung, Timothy Ting-Leung Ng, Chloe Toi-Mei Chan, Kingsley King-Gee Tam, Hiu-Yin Lao, Denise Sze-Hang Wong, Alan Ka-Lun Wu, Miranda Chong-Yee Yau, Yvette Wai-Man Lai, Kitty Sau-Chun Fung, Sandy Ka-Yee Chau, Barry Kin-Chung Wong, Wing-Kin To, Kristine Luk, Alex Yat-Man Ho, Tak-Lun Que, Kam-Tong Yip, Wing Cheong Yam, David Ho-Keung Shum, Shea Ping Yip

EPI_ISL_1159175	HOSPITAL CLINIC	Instituto de Salud Carlos III	Sandonís,V. Vázquez-Morón, S. Iglesias-Caballero, M. Camarero, S. Pozo, F. Casas, I. Jiménez, P. Zaballos, A. Monzón, S. Varona, S. Cuesta, I. VILA ESTAPE, JORDI
EPI_ISL_1159386	Siti Khodijah Hospital	Institute of Tropical Disease, Universitas Airlangga	Krisnoadi Rahardjo, Aldise M Nastri, Jezzy R Dewantari, Rima R Prasetya, Muhammad Hamdan, Gatot Soegiarto, Laksmi Wulandari, Resti Yudhawati, Yasuko Mori, Soetjipto, Kazufumi Shimizu, Maria I Lusida
EPI_ISL_1159387	Adi Husada Kapasari Hospital	Institute of Tropical Disease, Universitas Airlangga	Maria I Lusida, Krisnoadi Rahardjo, Aldise M Nastri, Jezzy R Dewantari, Rima R Prasetya, Hermanto Wijaya, Gatot Soegiarto, Laksmi Wulandari, Resti Yudhawati, Soetjipto, Yasuko Mori, Kazufumi Shimizu
EPI_ISL_1159388	Paru Hospital	Institute of Tropical Disease, Universitas Airlangga	Aldise M Nastri, Jezzy R Dewantari, Rima R Prasetya, Krisnoadi Rahardjo, Dyah Retno, Gatot Soegiarto, Laksmi Wulandari, Resti Yudhawati, Soetjipto, Yasuko Mori, Maria I Lusida, Kazufumi Shimizu
EPI_ISL_1159389	Darmo Hospital	Institute of Tropical Disease, Universitas Airlangga	Jezzy R Dewantari, Rima R Prasetya, Krisnoadi Rahardjo, Aldise M Nastri, Sulung Budianto, Gatot Soegiarto, Laksmi Wulandari, Resti Yudhawati, Soetjipto, Yasuko Mori, Maria I Lusida, Kazufumi Shimizu
EPI_ISL_1160138, EPI_ISL_1160148, EPI_ISL_1160152, EPI_ISL_1160155, EPI_ISL_1160157, EPI_ISL_1160161, EPI_ISL_1160174, EPI_ISL_1160182	Center of Medical Microbiology, Virology, and Hospital Hygiene, University of Duesseldorf	Center of Medical Microbiology, Virology, and Hospital Hygiene, University of Duesseldorf	Maximilian Damagnez;Alexander Dilthey;Torsten Houwaart;Lisanna Hülse;Malte Kohns Vasconcelos;Nadine Lübke;Jessica Nicolai;Klaus Pfeffer;Daniel Strelow;Jörg Timm;Andreas Walker;Tobias Wienemann
EPI_ISL_1163832, EPI_ISL_1163833, EPI_ISL_1163834, EPI_ISL_1163838, EPI_ISL_1163842, EPI_ISL_1163843, EPI_ISL_1163845	Microbiology and Virology Unit, Azienda Ospedale Padova, Padova, Italy	Department of Molecular Medicine, Computational Medicine Group, Univeresity of Padova, Padova, Italy	Elisa Franchin, Claudia Del Vecchio, Francesco Onelia, Stefano Toppo, Enrico Lavezzo, Laura Manuto, Federico Bianca , Marco Grazioli, Andrea Crisanti
EPI_ISL_1164686, EPI_ISL_1164687, EPI_ISL_1164688, EPI_ISL_1164689, EPI_ISL_1164691, EPI_ISL_1164692, EPI_ISL_1164693, EPI_ISL_1164694, EPI_ISL_1164695, EPI_ISL_1164696, EPI_ISL_1164697, EPI_ISL_1164698			
see above	Department of Molecular Virology, Cyprus Institute of Neurology and Genetics	Department of Molecular Virology, Cyprus Institute of Neurology and Genetics	Jan Richter, Pavlos Fanis, Christina Tryfonos, Dana Koptides, George Krashias, Stavros Bashiardes, Andreas Hadjisavvas, Maria Loizidou, Anastasis Oulas, Denise Alexandrou, Olga Kalakouta, Mihalis Panayiotidis, George Spyrou, Christina Christodoulou
EPI_ISL_1164931	Ohio Department of Health Laboratory	Ohio Department of Health Laboratory	Holmes, Jennifer; Eric Brandt, Keoni Omura, Glen McGillivary, Caitlin McDonnell, Jade Mowery, Stephanie Mccracken, Tyler Payne, Kirtana Ramadugu, Erica Leasure, Brent Lee, Kelsey Florek, Heather Blankenship, Allison Black, Quanta Brown, and Tammy Bannerman
EPI_ISL_1165079, EPI_ISL_1165080	Medical Ain Shams Research Institute (MASRI), Ain Shams University	Medical Ain Shams Research Institute (MASRI), Ain Shams University	Hesham Elghazaly, Sara Hassan Agwa, Hala Hafez , Fatma Ebied, Manal Hamdy Elsaid, Mohamed Elhadidi, Aya Mohamed , Shimaa Moustafa, Reham Kassab, Iman Foda , Samia Abdou Girgis, Hoda Ezz Elarab, Hagar Elshora, Osama Mansour.
EPI_ISL_1165442	Ohio Department of Health Laboratory	Ohio Department of Health Laboratory	Holmes, Jennifer; Eric Brandt, Keoni Omura, Glen McGillivary, Caitlin McDonnell, Jade Mowery, Stephanie Mccracken, Tyler Payne, Kirtana Ramadugu, Erica Leasure, Brent Lee, Kelsey Florek, Heather Blankenship, Allison Black, Quanta Brown, and Tammy Bannerman
EPI_ISL_1167040, EPI_ISL_1167041, EPI_ISL_1167042, EPI_ISL_1167043, EPI_ISL_1167044, EPI_ISL_1167045, EPI_ISL_1167050, EPI_ISL_1167052, EPI_ISL_1167056, EPI_ISL_1167058, EPI_ISL_1167061, EPI_ISL_1167063			
see above	"Dr. Andrija Stampar" Teaching Institute of Public Health, Department of Clinical Microbiology	Istituto di Genomica Applicata	Jasmina Vranes, Slobodanka Radovic, Fedrica Cattonaro, Irena Jurman, Vera Vendramin, Gabriele Magris, Eleonora Paparelli, Davide Scaglione, Michele Morgante
EPI_ISL_1167709, EPI_ISL_1167710	Genetica Molecular and Subdepartamento de Virologia ISP Chile	Instituto de Salud Publica de Chile	Javier Tognarelli, Karen Orostica, Barbara Parra, Loredana Arata, Jaime Lagos, Gisselle Barra, Patricia Bustos, Rodrigo Fasce, Andres Castillo, Jorge Fernandez
EPI_ISL_1168662, EPI_ISL_1168663, EPI_ISL_1168669, EPI_ISL_1168679	Centers for Disease Control and Prevention, Dengue Branch	Centers for Disease Control and Prevention, Dengue Branch	Gilberto A. Santiago, Glenda Gonzalez, Betzabel Flores, Keyla Charriez, Gabriela Paz-Bailey, Jorge L. Munoz-Jordan
EPI_ISL_1169534, EPI_ISL_1169535, EPI_ISL_1169537	Commonwealth Healthcare Center	Genomics and Discovery, Respiratory Viruses Branch, Division of Viral Diseases, Centers for Disease Control and Prevention	Krista Queen, Yan Li, Ying Tao, Jing Zhang, Anna Uehara, Anna Montmayeur, Clinton R. Paden, Peter W. Cook, Rachel Marine, Mili Sheth, Jasmine Padilla, Sarah Nobles, Mark Burroughs, Lori Rowe, Haibin Wang, Ben L. Rambo-Martin, Dhwani Batra, Justin Lee, Suxiang Tong
EPI_ISL_1169697, EPI_ISL_1169699, EPI_ISL_1169701, EPI_ISL_1169702, EPI_ISL_1169703, EPI_ISL_1169704, EPI_ISL_1169705, EPI_ISL_1169706, EPI_ISL_1169707, EPI_ISL_1169710, EPI_ISL_1169711, EPI_ISL_1169714, EPI_ISL_1169715			
see above	Colorado Department of Public Health and Environment	Colorado Department of Public Health and Environment	Laura Bankers, Molly C. Hetherington-Rauth, Diana Ir, Shannon Ely, Shannon R. Matzinger, Sarah Elizabeth Totten, Emily A. Travanty
EPI_ISL_626124, EPI_ISL_626127, EPI_ISL_626129, EPI_ISL_626132, EPI_ISL_626133, EPI_ISL_626134, EPI_ISL_626138, EPI_ISL_626139, EPI_ISL_626146, EPI_ISL_626148, EPI_ISL_626149, EPI_ISL_626157, EPI_ISL_626164			
see above	Department of Virus and Microbiological Special Diagnostics, Statens Serum Institut, Denmark	Albertsen lab, Department of Chemistry and Bioscience, Aalborg University, Denmark	Danish Covid-19 Genome Consortia
EPI_ISL_651107, EPI_ISL_652401, EPI_ISL_652402, EPI_ISL_652403, EPI_ISL_652404, EPI_ISL_652405, EPI_ISL_652408, EPI_ISL_652409, EPI_ISL_652410, EPI_ISL_652411, EPI_ISL_652412			
see above	Queens Medical Centre, Clinical Microbiology Department / DeepSeq Nottingham	COVID-19 Genomics UK (COG-UK) Consortium	Gemma Clark, Wendy Smith, Manjinder Khakh, Vicki M Fleming, Michelle M Lister, Hannah Howson-Wells, Jonathan Ball, Patrick McClure, Joseph Chappell, Theocharis Tsoleridis, Nadine Holmes, Matthew Carlisle, Christopher Moore, Fei Sang, Johnny Debebe, Victoria Wright, Matthew Loose
EPI_ISL_654799, EPI_ISL_654800, EPI_ISL_654801, EPI_ISL_654802, EPI_ISL_654803, EPI_ISL_654804, EPI_ISL_654805, EPI_ISL_654806, EPI_ISL_654808	SA Pathology	SA Pathology	Lex Leong, Julien Soubrier, Chuan Kok Lim, Song Gao, Mark Turra, Karin Kassahn, Ivan Bastian, Geoff Higgins
EPI_ISL_660070	Zurita & Zurita Laboratorios	Zurita & Zurita Laboratorios	Gabriela Sevillano Camilo Zurita-Salinas Karen Loaiza David Ortega-Paredes Jeannete Zurita
EPI_ISL_660229, EPI_ISL_660231, EPI_ISL_660232, EPI_ISL_660256, EPI_ISL_660257	KRISP, KZN Research Innovation and Sequencing Platform	KRISP, KZN Research Innovation and Sequencing Platform	Giandhari J, Pillay S, Lessells R, Mdlalose K, York D, Khan S, Tegally H, Wilkinson E, de Oliveira T
EPI_ISL_661218, EPI_ISL_661219, EPI_ISL_661220, EPI_ISL_661222, EPI_ISL_661224, EPI_ISL_661225, EPI_ISL_661226, EPI_ISL_661228, EPI_ISL_661230, EPI_ISL_661231, EPI_ISL_661232, EPI_ISL_661233, EPI_ISL_661234, EPI_ISL_661236, EPI_ISL_661238, EPI_ISL_661239, EPI_ISL_661240, EPI_ISL_661241			
see above	Department of Clinical Microbiology	GIGA Medical Genomics	Keith Durkin, Maria Artesi, Sébastien Bontems, Raphaël Boreux, Bouchra Boujemla, Cécile Meex, Pierrette Melin, Marie-Pierre Hayette, Vincent Bours
EPI_ISL_661258	LabPLUS	Institute of Environmental Science and Research (ESR)	Xiaoyun Ren, Matt Storey, Nikki Freed, Muhammad Faisal, Jing Wang, Hermes Perez, Anja Werno, Antje van der Linden, Arlo Upton, Chris Mansell, David Hammer, Dragana Drinkovic, Gary McAuliffe, Hana Sofia Andersson, James Ussher, Jill Sherwood, Josh Freeman, Julia Howard, Juliet Elvy, Mary DeAlmeida, Matt Blakiston, Matthew Rogers, Max Bloomfield, Michael Addidle, Michelle Balm, Sally Roberts, Sarah Jefferies, Sharmini Muttaiyah, Susan Morpeth, Susan Taylor, Timothy Blackmore, Vani Sathyendran, Veronica Playle, Virginia Hope, Erasmus Smit, Lauren Jelly, Olin Silander, Joep de Ligt
EPI_ISL_665005, EPI_ISL_665013	Department of Pathology, University of Cambridge	COVID-19 Genomics UK (COG-UK) Consortium	Aminu S. Jahun, Yasmin Chaudhry, Grant Hall, Iliana Georgana, Myra Hosmillo, Martin D. Curran, Malte Pinckert, Surendra Parmar, Ian Goodfellow
EPI_ISL_665636	University College London Hospital	COVID-19 Genomics UK (COG-UK) Consortium	Judith Heaney, Matthew Byott, Catherine Houlihan, Dan Frampton, Stuart Kirk, Moira Spyer and Eleni Nastouli
EPI_ISL_665641	West of Scotland Specialist Virology Centre, NHSGGC / MRC-University of Glasgow Centre for Virus Research	COVID-19 Genomics UK (COG-UK) Consortium	Ana da Silva Filipe, Natasha Johnson, Kathy Smollett, Daniel Mair, Stephen Carmichael, Alice Broos, Lily Tong, Jenna Nichols, Kyriaki Nomikou; Sarah McDonald; Richard Orton, Joseph Hughes, Sreenu Vattipally, David L Robertson; Alasdair MacLean, Rory Gunson; Sharif Shaaban, Matthew Holden; Rachel Blacow, Guy Mollett, Kathy Li, James Shepherd, Antonia Ho, Emma Thomson
EPI_ISL_665840, EPI_ISL_665841, EPI_ISL_665848	University College London Hospital	COVID-19 Genomics UK (COG-UK) Consortium	Judith Heaney, Matthew Byott, Catherine Houlihan, Dan Frampton, Stuart Kirk, Moira Spyer and Eleni Nastouli
EPI_ISL_665908, EPI_ISL_665910, EPI_ISL_665911, EPI_ISL_665914, EPI_ISL_665916, EPI_ISL_665918, EPI_ISL_665923, EPI_ISL_665924, EPI_ISL_665925, EPI_ISL_665926, EPI_ISL_665927, EPI_ISL_665932, EPI_ISL_665933, EPI_ISL_665936, EPI_ISL_665937			
see above	Queens Medical Centre, Clinical Microbiology Department / DeepSeq Nottingham	COVID-19 Genomics UK (COG-UK) Consortium	Gemma Clark, Wendy Smith, Manjinder Khakh, Vicki M Fleming, Michelle M Lister, Hannah Howson-Wells, Jonathan Ball, Patrick McClure, Joseph Chappell, Theocharis Tsoleridis, Nadine Holmes, Matthew Carlisle, Christopher Moore, Fei Sang, Johnny Debebe, Victoria Wright, Matthew Loose

EPI_ISL_666046, EPI_ISL_666050, EPI_ISL_666057	West of Scotland Specialist Virology Centre, NHSGGC / MRC-University of Glasgow Centre for Virus Research	COVID-19 Genomics UK (COG-UK) Consortium	Ana da Silva Filipe, Natasha Johnson, Kathy Smollett, Daniel Mair, Stephen Carmichael, Alice Broos, Lily Tong, Jenna Nichols, Kyriaki Nomikou; Sarah McDonald; Richard Orton, Joseph Hughes, Sreenu Vattipally, David L Robertson; Alasdair MacLean, Rory Gunson; Sharif Shaaban, Matthew Holden; Rachel Blacow, Guy Mollett, Kathy Li, James Shepherd, Antonia Ho, Emma Thomson
EPI_ISL_668601, EPI_ISL_668606, EPI_ISL_668635, EPI_ISL_668636, EPI_ISL_668663, EPI_ISL_668667, EPI_ISL_668668, EPI_ISL_668669, EPI_ISL_668671, EPI_ISL_668672, EPI_ISL_668718, EPI_ISL_668719, EPI_ISL_668853, EPI_ISL_668855, EPI_ISL_668858, EPI_ISL_668859, EPI_ISL_668861, EPI_ISL_668863, EPI_ISL_668867, EPI_ISL_668868, EPI_ISL_668869, EPI_ISL_668870, EPI_ISL_668871, EPI_ISL_668872, EPI_ISL_668873, EPI_ISL_668874, EPI_ISL_668875, EPI_ISL_668877, EPI_ISL_668878, EPI_ISL_668880, EPI_ISL_668881, EPI_ISL_668883, EPI_ISL_668884, EPI_ISL_668885, EPI_ISL_668887, EPI_ISL_668889, EPI_ISL_668893, EPI_ISL_668894, EPI_ISL_668896, EPI_ISL_668900, EPI_ISL_668903, EPI_ISL_668904, EPI_ISL_668905, EPI_ISL_668907, EPI_ISL_668908, EPI_ISL_668909, EPI_ISL_668910, EPI_ISL_668911, EPI_ISL_668912, EPI_ISL_668913, EPI_ISL_668914, EPI_ISL_668915, EPI_ISL_668918, EPI_ISL_668923, EPI_ISL_668924, EPI_ISL_668925, EPI_ISL_668927, EPI_ISL_668928, EPI_ISL_668930, EPI_ISL_668931, EPI_ISL_668932, EPI_ISL_668933, EPI_ISL_668936, EPI_ISL_668937, EPI_ISL_668940, EPI_ISL_668943, EPI_ISL_668944, EPI_ISL_668946, EPI_ISL_668947, EPI_ISL_668948, EPI_ISL_668949, EPI_ISL_668950, EPI_ISL_668951, EPI_ISL_668952, EPI_ISL_668954, EPI_ISL_668956			
see above	Department of Virus and Microbiological Special Diagnostics, Statens Serum Institut, Copenhagen, Denmark	Albertsen Lab, Department of Chemistry and Bioscience, Aalborg University, Denmark	Danish Covid-19 Genome Consortium
EPI_ISL_671883	National Virus Reference Laboratory	National Virus Reference Laboratory	Michael Carr, Gabriel Gonzalez, Jonathan Dean, Daniel Hare, Cillian F De Gascun
EPI_ISL_672758, EPI_ISL_672764, EPI_ISL_672782, EPI_ISL_672799, EPI_ISL_672837, EPI_ISL_672850, EPI_ISL_672853, EPI_ISL_672857, EPI_ISL_672864, EPI_ISL_672894	Lighthouse Lab in Glasgow	Wellcome Sanger Institute for the COVID-19 Genomics UK (COG-UK) Consortium	Harper VanSteenhouse, Yumi Kasai, David Gray, Carol Clugston, Anna Dominiczak and Alex Alderton, Roberto Amato, Sonia Goncalves, Ewan Harrison, David K. Jackson, Ian Johnston, Dominic Kwiatkowski, Cordelia Langford, John Sillitoe on behalf of the Wellcome Sanger Institute COVID-19 Surveillance Team
EPI_ISL_673288, EPI_ISL_673443	Lighthouse Lab in Cambridge	Wellcome Sanger Institute for the COVID-19 Genomics UK (COG-UK) Consortium	Rob Howes, The Lighthouse Lab in Cambridge and Alex Alderton, Roberto Amato, Sonia Goncalves, Ewan Harrison, David K. Jackson, Ian Johnston, Dominic Kwiatkowski, Cordelia Langford, John Sillitoe on behalf of the Wellcome Sanger Institute COVID-19 Surveillance Team
EPI_ISL_674863	Lighthouse Lab in Milton Keynes	Wellcome Sanger Institute for the COVID-19 Genomics UK (COG-UK) Consortium	The Lighthouse Lab in Milton Keynes and Alex Alderton, Roberto Amato, Sonia Goncalves, Ewan Harrison, David K. Jackson, Ian Johnston, Dominic Kwiatkowski, Cordelia Langford, John Sillitoe on behalf of the Wellcome Sanger Institute COVID-19 Surveillance Team
EPI_ISL_676491, EPI_ISL_676502, EPI_ISL_676503, EPI_ISL_676504	Klinisk mikrobiologi	The Public Health Agency of Sweden	Department of Microbiology, The Public Health Agency of Sweden
EPI_ISL_678261, EPI_ISL_678264, EPI_ISL_678265, EPI_ISL_678266, EPI_ISL_678267	Communicable Disease Laboratory, Public Health Directorate	Communicable Disease Laboratory, Public Health Directorate	Alwasti,H., Altaif,Z., AlHujairi,Z., AlAbbas,Z.
EPI_ISL_678456, EPI_ISL_678457, EPI_ISL_678458, EPI_ISL_678459, EPI_ISL_678461, EPI_ISL_678464, EPI_ISL_678465, EPI_ISL_678466, EPI_ISL_678469, EPI_ISL_678470, EPI_ISL_678472, EPI_ISL_678473, EPI_ISL_678475, EPI_ISL_678478, EPI_ISL_678479, EPI_ISL_678480, EPI_ISL_678483, EPI_ISL_678484, EPI_ISL_678485			
see above	Department of Clinical Microbiology	GIGA Medical Genomics	Keith Durkin, Maria Artesi, Sébastien Bontems, Raphaël Boreux, Bouchra Boujemla, Cécile Meex, Pierrette Melin, Marie-Pierre Hayette, Vincent Bours
EPI_ISL_678706, EPI_ISL_678719	Respiratory Virus Unit, Microbiology Services Colindale, Public Health England	COVID-19 Genomics UK (COG-UK) Consortium	PHE Covid Sequencing Team
EPI_ISL_679161, EPI_ISL_679165, EPI_ISL_679166, EPI_ISL_679167, EPI_ISL_679168, EPI_ISL_679179, EPI_ISL_679191, EPI_ISL_679193	Department of Pathology, University of Cambridge	COVID-19 Genomics UK (COG-UK) Consortium	Aminu S. Jahun, Yasmin Chaudhry, Grant Hall, Iliana Georgana, Myra Hosmillo, Martin D. Curran, Malte Pinckert, Surendra Parmar, Ian Goodfellow
EPI_ISL_679256, EPI_ISL_679258, EPI_ISL_679263, EPI_ISL_679278, EPI_ISL_679308, EPI_ISL_679313	Queens Medical Centre, Clinical Microbiology Department / DeepSeq Nottingham	COVID-19 Genomics UK (COG-UK) Consortium	Gemma Clark, Wendy Smith, Manjinder Khakh, Vicki M Fleming, Michelle M Lister, Hannah Howson-Wells, Jonathan Ball, Patrick McClure, Joseph Chappell, Theocharis Tsoleridis, Nadine Holmes, Matthew Carlisle, Christopher Moore, Fei Sang, Johnny Debebe, Victoria Wright, Matthew Loose
EPI_ISL_679407, EPI_ISL_679416, EPI_ISL_679417, EPI_ISL_679420, EPI_ISL_679421, EPI_ISL_679422, EPI_ISL_679423, EPI_ISL_679424, EPI_ISL_679425, EPI_ISL_679426, EPI_ISL_679432, EPI_ISL_679433, EPI_ISL_679434, EPI_ISL_679437, EPI_ISL_679438, EPI_ISL_679441, EPI_ISL_679442, EPI_ISL_679446			
see above	University College London Hospital	COVID-19 Genomics UK (COG-UK) Consortium	Judith Heaney, Matthew Byott, Catherine Houlihan, Dan Frampton, Stuart Kirk, Moira Spyer and Eleni Nastouli
EPI_ISL_679944	Queens Medical Centre, Clinical Microbiology Department / DeepSeq Nottingham	COVID-19 Genomics UK (COG-UK) Consortium	Gemma Clark, Wendy Smith, Manjinder Khakh, Vicki M Fleming, Michelle M Lister, Hannah Howson-Wells, Jonathan Ball, Patrick McClure, Joseph Chappell, Theocharis Tsoleridis, Nadine Holmes, Matthew Carlisle, Christopher Moore, Fei Sang, Johnny Debebe, Victoria Wright, Matthew Loose
EPI_ISL_679951, EPI_ISL_679954	Lincolnshire Hospitals and DeepSeq Nottingham	COVID-19 Genomics UK (COG-UK) Consortium	Nichola Duckworth, Tim Sloan, Sarah Walsh, Jonathan Ball, Patrick McClure, Joeseph Chappell, Nadine Holmes, Matthew Carlisle, Christopher Moore, Fei Sang, Johnny Debebe, Victoria Wright, Matthew Loose
EPI_ISL_680144	Virology Department, Sheffield Teaching Hospitals NHS Foundation Trust/Department of Infection, Immunity and Cardiovascular Disease, The Medical School, University of Sheffield	COVID-19 Genomics UK (COG-UK) Consortium	Thushan de Silva, Matthew Parker, Nikki Smith, Adri Angyal, Rebecca Brown, Luke Green, Rachel Tucker, Paul Parsons, Danielle Groves, Katie Johnson, Laura Carrilero, Alex Keeley, Dave Partridge, Matthew Wyles, Benjamin Lindsey, Mehmet Yavuz, Mohammad Raza, Cariad Evans
EPI_ISL_680461, EPI_ISL_680467, EPI_ISL_680486, EPI_ISL_680490, EPI_ISL_680491, EPI_ISL_680493, EPI_ISL_680541, EPI_ISL_680542, EPI_ISL_680549, EPI_ISL_680550, EPI_ISL_680552, EPI_ISL_680553, EPI_ISL_680555			
see above	Virology Department, Royal Infirmary of Edinburgh, NHS Lothian / School of Biological Sciences, University of Edinburgh / Institute of Genetics and Molecular Medicine, University of Edinburgh	COVID-19 Genomics UK (COG-UK) Consortium	McHugh M, Dewar R, Rooke S, Gallagher M, Balcaza C, O'Toole Á, Scher E, Hill V, McCrone JT, Colquhoun R, Yu X, Jackson B, Rambaut A, Williams TC, Templeton K
EPI_ISL_680610, EPI_ISL_680662, EPI_ISL_680665, EPI_ISL_680666, EPI_ISL_680670	Wales Specialist Virology Centre Sequencing lab: Pathogen Genomics Unit	COVID-19 Genomics UK (COG-UK) Consortium	Catherine Moore, Johnathan Evans, Laura Gifford, Malorie Perry, Simon Cottrell, Angela Marchbank, Alec Birchley, Alexander Adams, Amy Gaskin, Bree Gatica-Wilcox, Jason Coombes, Joel Southgate, Lauren Gilbert, Lee Graham, Nicole Pacchiarini, Sara Kumziene-Summerhayes, Sarah Taylor, Sophie Jones, Sara Rey, Matthew Bull, Joanne Watkins, Sally Corden, Tom Connor
EPI_ISL_681325, EPI_ISL_681329, EPI_ISL_681331, EPI_ISL_681332, EPI_ISL_681333, EPI_ISL_681336, EPI_ISL_681337, EPI_ISL_681338, EPI_ISL_681341, EPI_ISL_681343, EPI_ISL_681344, EPI_ISL_681346, EPI_ISL_681350, EPI_ISL_681352, EPI_ISL_681354, EPI_ISL_681359, EPI_ISL_681363, EPI_ISL_681364, EPI_ISL_681367, EPI_ISL_681371, EPI_ISL_681373, EPI_ISL_681379, EPI_ISL_681387, EPI_ISL_681397, EPI_ISL_681401, EPI_ISL_681406, EPI_ISL_681407, EPI_ISL_681408, EPI_ISL_681409, EPI_ISL_681411, EPI_ISL_681414, EPI_ISL_681415, EPI_ISL_681417, EPI_ISL_681420, EPI_ISL_681422, EPI_ISL_681423, EPI_ISL_681426, EPI_ISL_681428, EPI_ISL_681431, EPI_ISL_681433, EPI_ISL_681437, EPI_ISL_681438, EPI_ISL_681440, EPI_ISL_681443, EPI_ISL_681444, EPI_ISL_681449, EPI_ISL_681450, EPI_ISL_681451, EPI_ISL_681452, EPI_ISL_681454, EPI_ISL_681455, EPI_ISL_681456, EPI_ISL_681467, EPI_ISL_681470, EPI_ISL_681474, EPI_ISL_681475, EPI_ISL_681476, EPI_ISL_681480, EPI_ISL_681481, EPI_ISL_681485, EPI_ISL_681489, EPI_ISL_681490, EPI_ISL_681495, EPI_ISL_681498, EPI_ISL_681504, EPI_ISL_681508, EPI_ISL_681516, EPI_ISL_681517, EPI_ISL_681518, EPI_ISL_681520, EPI_ISL_681521, EPI_ISL_681524, EPI_ISL_681525, EPI_ISL_681526, EPI_ISL_681529, EPI_ISL_681531, EPI_ISL_681532, EPI_ISL_681538, EPI_ISL_681546, EPI_ISL_681548, EPI_ISL_681549, EPI_ISL_681553, EPI_ISL_681555, EPI_ISL_681557, EPI_ISL_681558, EPI_ISL_681559, EPI_ISL_681560, EPI_ISL_681572, EPI_ISL_681574, EPI_ISL_681575, EPI_ISL_681577, EPI_ISL_681580, EPI_ISL_681582, EPI_ISL_681583, EPI_ISL_681586, EPI_ISL_681589, EPI_ISL_681590, EPI_ISL_681591, EPI_ISL_681593, EPI_ISL_681594, EPI_ISL_681595, EPI_ISL_681602, EPI_ISL_681608, EPI_ISL_681609, EPI_ISL_681613, EPI_ISL_681614, EPI_ISL_681615, EPI_ISL_681619, EPI_ISL_681621, EPI_ISL_681623, EPI_ISL_681625, EPI_ISL_681627, EPI_ISL_681629, EPI_ISL_681633, EPI_ISL_681635, EPI_ISL_681636, EPI_ISL_681639, EPI_ISL_681640, EPI_ISL_681641, EPI_ISL_681642, EPI_ISL_681646, EPI_ISL_681647, EPI_ISL_681649, EPI_ISL_681650, EPI_ISL_681653, EPI_ISL_681655			
see above	Lighthouse Lab in Milton Keynes	Wellcome Sanger Institute for the COVID-19 Genomics UK (COG-UK) Consortium	The Lighthouse Lab in Milton Keynes and Alex Alderton, Roberto Amato, Sonia Goncalves, Ewan Harrison, David K. Jackson, Ian Johnston, Dominic Kwiatkowski, Cordelia Langford, John Sillitoe on behalf of the Wellcome Sanger Institute COVID-19 Surveillance Team
EPI_ISL_682279, EPI_ISL_682282, EPI_ISL_682287, EPI_ISL_682288	Middlemore Hospital	Institute of Environmental Science and Research (ESR)	Xiaoyun Ren, Matt Storey, Nikki Freed, Muhammad Faisal, Jing Wang, Hermes Perez, Anja Werno, Antje van der Linden, Arlo Upton, Chris Mansell, David Hammer, Dragana Drinkovic, Gary McAuliffe, Hana Sofia Andersson, James Ussher, Jill Sherwood, Josh Freeman, Julia Howard, Juliet Elvy, Mary DeAlmeida, Matt Blakiston, Matthew Rogers, Max Bloomfield, Michael Addidle, Michelle Balm, Sally Roberts, Sarah Jefferies, Sharmini Muttaiyah, Susan Morpeth, Susan Taylor, Timothy Blackmore, Vani Sathyendran, Veronica Playle, Virginia Hope, Erasmus Smit, Lauren Jelly, Olin Silander, Joep de Ligt
EPI_ISL_682292	LabPLUS	Institute of Environmental Science and Research (ESR)	Xiaoyun Ren, Matt Storey, Nikki Freed, Muhammad Faisal, Jing Wang, Hermes Perez, Anja Werno, Antje van der Linden, Arlo Upton, Chris Mansell, David Hammer, Dragana Drinkovic, Gary McAuliffe, Hana Sofia Andersson, James Ussher, Jill Sherwood, Josh Freeman, Julia Howard, Juliet Elvy, Mary DeAlmeida, Matt Blakiston, Matthew Rogers, Max Bloomfield, Michael Addidle, Michelle Balm, Sally Roberts, Sarah Jefferies, Sharmini Muttaiyah, Susan Morpeth, Susan Taylor, Timothy Blackmore, Vani Sathyendran, Veronica Playle, Virginia Hope, Erasmus Smit, Lauren Jelly, Olin Silander, Joep de Ligt
EPI_ISL_682296	Canterbury Health Laboratories	Institute of Environmental Science and Research (ESR)	Xiaoyun Ren, Matt Storey, Nikki Freed, Muhammad Faisal, Jing Wang, Hermes Perez, Anja Werno, Antje van der Linden, Arlo Upton, Chris Mansell, David Hammer, Dragana Drinkovic, Gary McAuliffe, Hana Sofia Andersson, James Ussher, Jill Sherwood, Josh Freeman, Julia Howard, Juliet Elvy, Mary

			DeAlmeida, Matt Blakiston, Matthew Rogers, Max Bloomfield, Michael Addidle, Michelle Balm, Sally Roberts, Sarah Jefferies, Sharmini Muttaiyah, Susan Morpeth, Susan Taylor, Timothy Blackmore, Vani Sathyendran, Veronica Playle, Virginia Hope, Erasmus Smit, Lauren Jelly, Olin Silander, Joep de Ligt
EPI_ISL_682353, EPI_ISL_682354, EPI_ISL_682357, EPI_ISL_682358, EPI_ISL_682361, EPI_ISL_682362, EPI_ISL_682363, EPI_ISL_682364, EPI_ISL_682365, EPI_ISL_682366, EPI_ISL_682369, EPI_ISL_682371, EPI_ISL_682374, EPI_ISL_682375, EPI_ISL_682378, EPI_ISL_682379, EPI_ISL_682380, EPI_ISL_682381, EPI_ISL_682382, EPI_ISL_682383, EPI_ISL_682384, EPI_ISL_682385, EPI_ISL_682386, EPI_ISL_682387, EPI_ISL_682389, EPI_ISL_682391, EPI_ISL_682395, EPI_ISL_682397, EPI_ISL_682398, EPI_ISL_682404, EPI_ISL_682409, EPI_ISL_682410, EPI_ISL_682412, EPI_ISL_682413, EPI_ISL_682416, EPI_ISL_682418, EPI_ISL_682420, EPI_ISL_682422, EPI_ISL_682423, EPI_ISL_682424, EPI_ISL_682425, EPI_ISL_682427, EPI_ISL_682429, EPI_ISL_682431, EPI_ISL_682432, EPI_ISL_682433, EPI_ISL_682434, EPI_ISL_682435, EPI_ISL_682436, EPI_ISL_682437, EPI_ISL_682438, EPI_ISL_682440, EPI_ISL_682441, EPI_ISL_682445, EPI_ISL_682447, EPI_ISL_682448, EPI_ISL_682449, EPI_ISL_682450, EPI_ISL_682452, EPI_ISL_682456, EPI_ISL_682457, EPI_ISL_682458, EPI_ISL_682459, EPI_ISL_682460, EPI_ISL_682462, EPI_ISL_682463, EPI_ISL_682464, EPI_ISL_682468, EPI_ISL_682470, EPI_ISL_682474, EPI_ISL_682475, EPI_ISL_682476, EPI_ISL_682477, EPI_ISL_682478, EPI_ISL_682479, EPI_ISL_682481, EPI_ISL_682482, EPI_ISL_682486, EPI_ISL_682487, EPI_ISL_682488, EPI_ISL_682489, EPI_ISL_682491, EPI_ISL_682492, EPI_ISL_682494, EPI_ISL_682495, EPI_ISL_682496, EPI_ISL_682500, EPI_ISL_682501, EPI_ISL_682502, EPI_ISL_682503, EPI_ISL_682504, EPI_ISL_682505, EPI_ISL_682510, EPI_ISL_682512, EPI_ISL_682514, EPI_ISL_682515, EPI_ISL_682517, EPI_ISL_682519, EPI_ISL_682520, EPI_ISL_682522, EPI_ISL_682523, EPI_ISL_682524, EPI_ISL_682525, EPI_ISL_682527, EPI_ISL_682528, EPI_ISL_682529, EPI_ISL_682531, EPI_ISL_682532, EPI_ISL_682533, EPI_ISL_682534, EPI_ISL_682535, EPI_ISL_682538, EPI_ISL_682539, EPI_ISL_682540, EPI_ISL_682541, EPI_ISL_682543, EPI_ISL_682546, EPI_ISL_682548, EPI_ISL_682552, EPI_ISL_682553, EPI_ISL_682554, EPI_ISL_682555, EPI_ISL_682556, EPI_ISL_682557, EPI_ISL_682562, EPI_ISL_682564, EPI_ISL_682566, EPI_ISL_682567, EPI_ISL_682568, EPI_ISL_682571, EPI_ISL_682576, EPI_ISL_682577, EPI_ISL_682578, EPI_ISL_682581, EPI_ISL_682583, EPI_ISL_682584, EPI_ISL_682585, EPI_ISL_682586, EPI_ISL_682587, EPI_ISL_682589, EPI_ISL_682591, EPI_ISL_682592, EPI_ISL_682593, EPI_ISL_682594, EPI_ISL_682595, EPI_ISL_682596, EPI_ISL_682597, EPI_ISL_682598, EPI_ISL_682599, EPI_ISL_682600, EPI_ISL_682601, EPI_ISL_682604, EPI_ISL_682605, EPI_ISL_682606, EPI_ISL_682607, EPI_ISL_682608, EPI_ISL_682609, EPI_ISL_682610, EPI_ISL_682613, EPI_ISL_682615, EPI_ISL_682616, EPI_ISL_682617, EPI_ISL_682618, EPI_ISL_682620, EPI_ISL_682621, EPI_ISL_682622, EPI_ISL_682623, EPI_ISL_682624, EPI_ISL_682625, EPI_ISL_682627, EPI_ISL_682628, EPI_ISL_682632, EPI_ISL_682634, EPI_ISL_682635, EPI_ISL_682637, EPI_ISL_682638, EPI_ISL_682641, EPI_ISL_682642, EPI_ISL_682644, EPI_ISL_682646, EPI_ISL_682647, EPI_ISL_682648, EPI_ISL_682649, EPI_ISL_682651, EPI_ISL_682654, EPI_ISL_682655, EPI_ISL_682656, EPI_ISL_682657, EPI_ISL_682661, EPI_ISL_682662, EPI_ISL_682663, EPI_ISL_682664, EPI_ISL_682666, EPI_ISL_682667, EPI_ISL_682670, EPI_ISL_682672, EPI_ISL_682673, EPI_ISL_682674, EPI_ISL_682679, EPI_ISL_682680, EPI_ISL_682681, EPI_ISL_682682, EPI_ISL_682683, EPI_ISL_682685, EPI_ISL_682687, EPI_ISL_682689, EPI_ISL_682690, EPI_ISL_682692, EPI_ISL_682694, EPI_ISL_682696, EPI_ISL_682697, EPI_ISL_682698, EPI_ISL_682699, EPI_ISL_682702, EPI_ISL_682705, EPI_ISL_682706, EPI_ISL_682707, EPI_ISL_682708, EPI_ISL_682709, EPI_ISL_682710, EPI_ISL_682711, EPI_ISL_682712, EPI_ISL_682717, EPI_ISL_682718, EPI_ISL_682719, EPI_ISL_682720, EPI_ISL_682723, EPI_ISL_682724, EPI_ISL_682726, EPI_ISL_682727, EPI_ISL_682728, EPI_ISL_682730, EPI_ISL_682731, EPI_ISL_682732, EPI_ISL_682733, EPI_ISL_682734, EPI_ISL_682736, EPI_ISL_682742, EPI_ISL_682744, EPI_ISL_682746, EPI_ISL_682747, EPI_ISL_682748, EPI_ISL_682749, EPI_ISL_682750, EPI_ISL_682752, EPI_ISL_682753, EPI_ISL_682754, EPI_ISL_682757, EPI_ISL_682758, EPI_ISL_682761, EPI_ISL_682763, EPI_ISL_682764, EPI_ISL_682766, EPI_ISL_682768, EPI_ISL_682769, EPI_ISL_682772, EPI_ISL_682773, EPI_ISL_682774, EPI_ISL_682775, EPI_ISL_682782, EPI_ISL_682784, EPI_ISL_682785, EPI_ISL_682786, EPI_ISL_682787, EPI_ISL_682788, EPI_ISL_682789, EPI_ISL_682792, EPI_ISL_682793, EPI_ISL_682795, EPI_ISL_682796, EPI_ISL_682798, EPI_ISL_682800, EPI_ISL_682801, EPI_ISL_682802, EPI_ISL_682803, EPI_ISL_682804, EPI_ISL_682805, EPI_ISL_682806, EPI_ISL_682809, EPI_ISL_682810, EPI_ISL_682811, EPI_ISL_682812, EPI_ISL_682813, EPI_ISL_682817, EPI_ISL_682819, EPI_ISL_682820, EPI_ISL_682822, EPI_ISL_682823, EPI_ISL_682824, EPI_ISL_682827, EPI_ISL_682828, EPI_ISL_682831, EPI_ISL_682833, EPI_ISL_682835, EPI_ISL_682836, EPI_ISL_682838, EPI_ISL_682840, EPI_ISL_682841, EPI_ISL_682844, EPI_ISL_682845, EPI_ISL_682846, EPI_ISL_682848, EPI_ISL_682849, EPI_ISL_682850, EPI_ISL_682851, EPI_ISL_682854, EPI_ISL_682855, EPI_ISL_682856, EPI_ISL_682857, EPI_ISL_682858, EPI_ISL_682859, EPI_ISL_682862, EPI_ISL_682864, EPI_ISL_682866, EPI_ISL_682867, EPI_ISL_682868, EPI_ISL_682869, EPI_ISL_682870, EPI_ISL_682871, EPI_ISL_682874, EPI_ISL_682876, EPI_ISL_682878, EPI_ISL_682880, EPI_ISL_682881, EPI_ISL_682883, EPI_ISL_682885, EPI_ISL_682886, EPI_ISL_682892, EPI_ISL_682893, EPI_ISL_682894, EPI_ISL_682895, EPI_ISL_682897, EPI_ISL_682898, EPI_ISL_682900, EPI_ISL_682902, EPI_ISL_682903, EPI_ISL_682904, EPI_ISL_682905, EPI_ISL_682907, EPI_ISL_682908, EPI_ISL_682910, EPI_ISL_682913, EPI_ISL_682914, EPI_ISL_682916, EPI_ISL_682919, EPI_ISL_682920, EPI_ISL_682921, EPI_ISL_682925, EPI_ISL_682927, EPI_ISL_682928			
see above	Department of Virus and Microbiological Special Diagnostics, Statens Serum Institut, Copenhagen, Denmark	Albertsen Lab, Department of Chemistry and Bioscience, Aalborg University, Denmark	Danish Covid-19 Genome Consortium
EPI_ISL_683482, EPI_ISL_683489, EPI_ISL_683541, EPI_ISL_683542, EPI_ISL_683548, EPI_ISL_683552, EPI_ISL_683553, EPI_ISL_683554, EPI_ISL_683557, EPI_ISL_683560, EPI_ISL_683572, EPI_ISL_683576, EPI_ISL_683577, EPI_ISL_683578, EPI_ISL_683579, EPI_ISL_683580, EPI_ISL_683581, EPI_ISL_683583, EPI_ISL_683586, EPI_ISL_683587, EPI_ISL_683588, EPI_ISL_683590, EPI_ISL_683591			
see above	Respiratory Virus Unit, Microbiology Services Colindale, Public Health England	COVID-19 Genomics UK (COG-UK) Consortium	PHE Covid Sequencing Team
EPI_ISL_683689	Hennepin County Medical Center	Minnesota Department of Health, Public Health Laboratory	Alexandra Lorentz, Jacob Garfin, Matt Plumb, and Xiong Wang
EPI_ISL_683729	Minnesota Department of Health, Public Health Laboratory	Minnesota Department of Health, Public Health Laboratory	Alexandra Lorentz, Jacob Garfin, Matt Plumb, and Xiong Wang
EPI_ISL_683766, EPI_ISL_683779	DOHMH Corona	New York City Public Health Laboratory	Jade Wang, et al.
EPI_ISL_683780	DOHMH Chelsea	New York City Public Health Laboratory	Jade Wang, et al.
EPI_ISL_683784	DOHMH Riverside	New York City Public Health Laboratory	Jade Wang, et al.
EPI_ISL_683786	DOHMH Jamaica	New York City Public Health Laboratory	Jade Wang, et al.
EPI_ISL_683789	DOHMH PHL	New York City Public Health Laboratory	Jade Wang, et al.
EPI_ISL_683790	DOHMH Morrisania	New York City Public Health Laboratory	Jade Wang, et al.
EPI_ISL_683791	DOHMH Jamaica	New York City Public Health Laboratory	Jade Wang, et al.
EPI_ISL_683792	DOHMH Chelsea	New York City Public Health Laboratory	Jade Wang, et al.
EPI_ISL_683793, EPI_ISL_683794	DOHMH Corona	New York City Public Health Laboratory	Jade Wang, et al.
EPI_ISL_683795	DOHMH Central Harlem	New York City Public Health Laboratory	Jade Wang, et al.
EPI_ISL_683796	DOHMH Corona	New York City Public Health Laboratory	Jade Wang, et al.
EPI_ISL_683797	DOHMH PHL	New York City Public Health Laboratory	Jade Wang, et al.
EPI_ISL_683798, EPI_ISL_683799, EPI_ISL_683800	DOHMH Corona	New York City Public Health Laboratory	Jade Wang, et al.
EPI_ISL_683801, EPI_ISL_683803, EPI_ISL_683805, EPI_ISL_683806	DOHMH Morrisania	New York City Public Health Laboratory	Jade Wang, et al.
EPI_ISL_683808	DOHMH Chelsea	New York City Public Health Laboratory	Jade Wang, et al.
EPI_ISL_683811, EPI_ISL_683812	DOHMH Corona	New York City Public Health Laboratory	Jade Wang, et al.
EPI_ISL_683813	DOHMH Central Harlem	New York City Public Health Laboratory	Jade Wang, et al.
EPI_ISL_683814	DOHMH Morrisania	New York City Public Health Laboratory	Jade Wang, et al.
EPI_ISL_683815	DOHMH Corona	New York City Public Health Laboratory	Jade Wang, et al.
EPI_ISL_683816	DOHMH Fort Greene	New York City Public Health Laboratory	Jade Wang, et al.
EPI_ISL_683817, EPI_ISL_683818	DOHMH Corona	New York City Public Health Laboratory	Jade Wang, et al.
EPI_ISL_683819, EPI_ISL_683820	DOHMH Riverside	New York City Public Health Laboratory	Jade Wang, et al.
EPI_ISL_683822	DOHMH Jamaica	New York City Public Health Laboratory	Jade Wang, et al.
EPI_ISL_683824	DOHMH Corona	New York City Public Health Laboratory	Jade Wang, et al.
EPI_ISL_683825, EPI_ISL_683826	DOHMH Morrisania	New York City Public Health Laboratory	Jade Wang, et al.
EPI_ISL_683829	DOHMH Central Harlem	New York City Public Health Laboratory	Jade Wang, et al.
EPI_ISL_683830	DOHMH Chelsea	New York City Public Health Laboratory	Jade Wang, et al.
EPI_ISL_683831	DOHMH Corona	New York City Public Health Laboratory	Jade Wang, et al.
EPI_ISL_683832	DOHMH Fort Greene	New York City Public Health Laboratory	Jade Wang, et al.
EPI_ISL_683834	DOHMH Central Harlem	New York City Public Health Laboratory	Jade Wang, et al.
EPI_ISL_683875	DOHMH PHL	New York City Public Health Laboratory	Jade Wang, et al.
EPI_ISL_683927, EPI_ISL_683928	DOHMH Central Harlem	New York City Public Health Laboratory	Jade Wang, et al.
EPI_ISL_683930	DOHMH Jamaica	New York City Public Health Laboratory	Jade Wang, et al.
EPI_ISL_683931	DOHMH Chelsea	New York City Public Health Laboratory	Jade Wang, et al.
EPI_ISL_683932, EPI_ISL_683934	DOHMH Corona	New York City Public Health Laboratory	Jade Wang, et al.

EPI_ISL_683936	DOHMH Chelsea	New York City Public Health Laboratory	Jade Wang, et al.
EPI_ISL_683978, EPI_ISL_683979	DOHMH Corona	New York City Public Health Laboratory	Jade Wang, et al.
EPI_ISL_683981	DOHMH Chelsea	New York City Public Health Laboratory	Jade Wang, et al.
EPI_ISL_683982, EPI_ISL_683983	DOHMH Central Harlem	New York City Public Health Laboratory	Jade Wang, et al.
EPI_ISL_683986, EPI_ISL_683990	DOHMH Jamaica	New York City Public Health Laboratory	Jade Wang, et al.
EPI_ISL_683993	DOHMH PHL	New York City Public Health Laboratory	Jade Wang, et al.
EPI_ISL_683996	DOHMH Fort Greene	New York City Public Health Laboratory	Jade Wang, et al.
EPI_ISL_686602, EPI_ISL_686606, EPI_ISL_686608	Respiratory Virus Unit, Microbiology Services Colindale, Public Health England	COVID-19 Genomics UK (COG-UK) Consortium	PHE Covid Sequencing Team
EPI_ISL_692776, EPI_ISL_692778	Massachusetts State Public Health Laboratory	Massachusetts State Public Health Laboratory	Andrew Lang, Timelia Fink, Glen Gallagher, Sandra Smole
EPI_ISL_693303, EPI_ISL_693304, EPI_ISL_693305, EPI_ISL_693306	Department of Laboratory Medicine, National Taiwan University Hospital	Microbial Genomics Core Lab, National Taiwan University Centers of Genomic and Precision Medicine	Shiou-Hwei Yeh, You-Yu Lin, Ya-Yun Lai, Chiao-Ling Li, Shan-Chwen Chang, Pei-Jer Chen, Sui-Yuan Chang
EPI_ISL_693307, EPI_ISL_693318	National Public Health Laboratory, National Centre for Infectious Diseases	National Public Health Laboratory, National Centre for Infectious Diseases	Tze Minn Mak, Sophie Octavia, Zhenyang Zhou, Lin Cui, Raymond Tzer Pin Lin
EPI_ISL_693332, EPI_ISL_693333	Lighthouse Lab in Alderley Park	Wellcome Sanger Institute for the COVID-19 Genomics UK (COG-UK) Consortium	Jacquelyn Wynn, Mairead Hyland, The Lighthouse Lab in Alderley Park and Alex Alderton, Roberto Amato, Sonia Goncalves, Ewan Harrison, David K. Jackson, Ian Johnston, Dominic Kwiatkowski, Cordelia Langford, John Sillitoe on behalf of the Wellcome Sanger Institute COVID-19 Surveillance Team
EPI_ISL_693406, EPI_ISL_693466	Respiratory Virus Unit, Microbiology Services Colindale, Public Health England	COVID-19 Genomics UK (COG-UK) Consortium	PHE Covid Sequencing Team
EPI_ISL_693766, EPI_ISL_693770, EPI_ISL_693774	General practitioner	National Reference Center for Viruses of Respiratory Infections, Institut Pasteur, Paris	Marion Barbet, Sylvie Behillil, Méline Bizard, Angela Brisebarre, Camille Capel, Etienne Simon-Lorière, Vincent Enouf, Maud Vanpeene, Sylvie van der Werf
EPI_ISL_693958, EPI_ISL_693963, EPI_ISL_693973, EPI_ISL_693975, EPI_ISL_694007	Viollier AG	Department of Biosystems Science and Engineering, ETH Zürich	Christian Beisel, Sarah Nadeau, Chaoran Chen, Ivan Topolsky, Pedro Ferreira, Philipp Jablonski, Susana Posada-Céspedes, Tobias Schär, Ina Nissen, Natascha Santacroce, Elodie Burcklen, Christiane Beckmann, Maurice Redondo, Olivier Kobel, Christoph Noppen, Sophie Seidel, Noemie Santamaria de Souza, Niko Beerenwinkel, Tanja Stadler
EPI_ISL_696462	Sedgefield Clinic wc SGE & NHLS/UCT	KRISP, KZN Research Innovation and Sequencing Platform	Arash Iranzadeh, Deelan Doolabh, Lynn Tyers, Bruna Galvao, Innocent Mudau, Marvin Hsiao, Kruger Marais, Jennifer Giandhari, Sureshnee Pillay, Houriiyah Tegally, Emanuel James San, Tulio de Oliveira, Diana Hardie, Stephen Korsman, Carolyn Williamson
EPI_ISL_698079, EPI_ISL_698080, EPI_ISL_698086, EPI_ISL_698091, EPI_ISL_698100	Unity Health Toronto	Ontario Institute for Cancer Research	Ramzi Fattouh, Larissa M. Matukas, Yan Chen,Mark Downing, Trina Otterman, Karel Boissinot, Le Luu, Samira Mubareka, TIBDN, Ilinca Lungu, Bernard Lam, Jeremy Johns, Paul Krzyzanowski, Richard de Borja, Felicia Vincelli, Philip Zuzarte, Jared T. Simpson
EPI_ISL_699655	1-Laboratory of Microbiology, National Reference Lab, Charles Nicolle Hospital; 2-University of Tunis ElManar, Faculty of Medicine of Tunis, LR99ES09, Tunis, Tunisia	1-Clinical and Experimental Pharmacology Lab, LR16SP02, National Center of Pharmacovigilance, University of Tunis El Manar, Tunis, Tunisia. 2-Neurodegenerative diseases and psychiatric troubles, LR18SP03, Razi Hospital, University of Tunis El Manar, Tunis, Tunisia. 3- Ministry of Health, National Observatory of New and Emerging Diseases, 1006, Tunis, Tunisia	Ilhem Boutiba-Ben Boubaker, Sameh Trabelsi, Nissaf Ben Alaya, Maher Kharrat, Alia Ben Kahla, Jalila Ben Khelil, Salma Abid, Sana Ferjani, Asma Ferjani, Mouna Ben Sassi, Mouna Safer, Guedi Berrabeh,Salwa Mrabet, Hanen ElJebari, Gaies Emna, Riadh Daghfous, Riadh Gouider.
EPI_ISL_701673, EPI_ISL_701674, EPI_ISL_701675, EPI_ISL_701676, EPI_ISL_701677, EPI_ISL_701678, EPI_ISL_701680, EPI_ISL_701681, EPI_ISL_701682, EPI_ISL_701684, EPI_ISL_701685, EPI_ISL_701690, EPI_ISL_701691, EPI_ISL_701692, EPI_ISL_701693, EPI_ISL_701695, EPI_ISL_701696, EPI_ISL_701698, EPI_ISL_701699, EPI_ISL_701701, EPI_ISL_701704, EPI_ISL_701706, EPI_ISL_701707, EPI_ISL_701709, EPI_ISL_701711, EPI_ISL_701714, EPI_ISL_701715, EPI_ISL_701716, EPI_ISL_701718, EPI_ISL_701719, EPI_ISL_701723, EPI_ISL_701725, EPI_ISL_701727, EPI_ISL_701728, EPI_ISL_701729, EPI_ISL_701730, EPI_ISL_701732, EPI_ISL_701733, EPI_ISL_701736, EPI_ISL_701738, EPI_ISL_701739, EPI_ISL_701741, EPI_ISL_701742, EPI_ISL_701746, EPI_ISL_701747, EPI_ISL_701748, EPI_ISL_701749, EPI_ISL_701750, EPI_ISL_701752, EPI_ISL_701753, EPI_ISL_701754, EPI_ISL_701756, EPI_ISL_701758, EPI_ISL_701760, EPI_ISL_701761, EPI_ISL_701762, EPI_ISL_701764, EPI_ISL_701770, EPI_ISL_701773, EPI_ISL_701776, EPI_ISL_701778, EPI_ISL_701781, EPI_ISL_701782, EPI_ISL_701783, EPI_ISL_701785, EPI_ISL_701787, EPI_ISL_701788, EPI_ISL_701789, EPI_ISL_701790, EPI_ISL_701791, EPI_ISL_701795, EPI_ISL_701796, EPI_ISL_701798, EPI_ISL_701799, EPI_ISL_701800, EPI_ISL_701802, EPI_ISL_701804, EPI_ISL_701805, EPI_ISL_701806, EPI_ISL_701807, EPI_ISL_701809, EPI_ISL_701810, EPI_ISL_701814, EPI_ISL_701815, EPI_ISL_701817, EPI_ISL_701818, EPI_ISL_701820, EPI_ISL_701821, EPI_ISL_701823, EPI_ISL_701824, EPI_ISL_701825, EPI_ISL_701826, EPI_ISL_701827, EPI_ISL_701829, EPI_ISL_701831, EPI_ISL_701832, EPI_ISL_701833, EPI_ISL_701834, EPI_ISL_701835, EPI_ISL_701836, EPI_ISL_701838, EPI_ISL_701841, EPI_ISL_701843, EPI_ISL_701845, EPI_ISL_701847, EPI_ISL_701850, EPI_ISL_701853, EPI_ISL_701854, EPI_ISL_701855, EPI_ISL_701856, EPI_ISL_701859, EPI_ISL_701860, EPI_ISL_701861, EPI_ISL_701862, EPI_ISL_701866, EPI_ISL_701867, EPI_ISL_701869, EPI_ISL_701871, EPI_ISL_701872, EPI_ISL_701873, EPI_ISL_701877, EPI_ISL_701879, EPI_ISL_701880, EPI_ISL_701883, EPI_ISL_701885, EPI_ISL_701888, EPI_ISL_701891, EPI_ISL_701893, EPI_ISL_701894, EPI_ISL_701896, EPI_ISL_701897, EPI_ISL_701899, EPI_ISL_701900, EPI_ISL_701901, EPI_ISL_701902, EPI_ISL_701903, EPI_ISL_701904, EPI_ISL_701908, EPI_ISL_701910, EPI_ISL_701911, EPI_ISL_701913, EPI_ISL_701914, EPI_ISL_701918, EPI_ISL_701919, EPI_ISL_701920, EPI_ISL_701922, EPI_ISL_701923, EPI_ISL_701924, EPI_ISL_701927, EPI_ISL_701928, EPI_ISL_701931, EPI_ISL_701933, EPI_ISL_701934, EPI_ISL_701938, EPI_ISL_701939, EPI_ISL_701940, EPI_ISL_701942, EPI_ISL_701944, EPI_ISL_701945, EPI_ISL_701946, EPI_ISL_701947, EPI_ISL_701948, EPI_ISL_701950, EPI_ISL_701952, EPI_ISL_701954, EPI_ISL_701956, EPI_ISL_701960, EPI_ISL_701965, EPI_ISL_701968, EPI_ISL_701970, EPI_ISL_701971, EPI_ISL_701972, EPI_ISL_701974, EPI_ISL_701975, EPI_ISL_701976, EPI_ISL_701977, EPI_ISL_701979, EPI_ISL_701981, EPI_ISL_701988, EPI_ISL_701990, EPI_ISL_701991, EPI_ISL_701993, EPI_ISL_701994, EPI_ISL_701998, EPI_ISL_701999, EPI_ISL_702001, EPI_ISL_702002, EPI_ISL_702003, EPI_ISL_702006, EPI_ISL_702007, EPI_ISL_702008, EPI_ISL_702009, EPI_ISL_702010, EPI_ISL_702016, EPI_ISL_702017, EPI_ISL_702021			
see above	Lighthouse Lab in Alderley Park	Wellcome Sanger Institute for the COVID-19 Genomics UK (COG-UK) Consortium	Jacquelyn Wynn, Mairead Hyland, The Lighthouse Lab in Alderley Park and Alex Alderton, Roberto Amato, Sonia Goncalves, Ewan Harrison, David K. Jackson, Ian Johnston, Dominic Kwiatkowski, Cordelia Langford, John Sillitoe on behalf of the Wellcome Sanger Institute COVID-19 Surveillance Team
EPI_ISL_702028, EPI_ISL_702030, EPI_ISL_702031, EPI_ISL_702032, EPI_ISL_702033, EPI_ISL_702035, EPI_ISL_702036, EPI_ISL_702037, EPI_ISL_702039, EPI_ISL_702043, EPI_ISL_702044, EPI_ISL_702046, EPI_ISL_702048, EPI_ISL_702051, EPI_ISL_702055, EPI_ISL_702056, EPI_ISL_702058, EPI_ISL_702060, EPI_ISL_702061, EPI_ISL_702062, EPI_ISL_702063, EPI_ISL_702065, EPI_ISL_702067, EPI_ISL_702068, EPI_ISL_702070, EPI_ISL_702071, EPI_ISL_702072, EPI_ISL_702073, EPI_ISL_702075, EPI_ISL_702076, EPI_ISL_702077, EPI_ISL_702078, EPI_ISL_702079, EPI_ISL_702081, EPI_ISL_702085, EPI_ISL_702087, EPI_ISL_702088, EPI_ISL_702090, EPI_ISL_702092, EPI_ISL_702098, EPI_ISL_702099, EPI_ISL_702100, EPI_ISL_702104, EPI_ISL_702105, EPI_ISL_702106, EPI_ISL_702108, EPI_ISL_702111, EPI_ISL_702114, EPI_ISL_702116, EPI_ISL_702117, EPI_ISL_702118, EPI_ISL_702122, EPI_ISL_702124, EPI_ISL_702125, EPI_ISL_702126, EPI_ISL_702127, EPI_ISL_702128, EPI_ISL_702129, EPI_ISL_702130, EPI_ISL_702131, EPI_ISL_702135, EPI_ISL_702136, EPI_ISL_702138, EPI_ISL_702140, EPI_ISL_702144, EPI_ISL_702145, EPI_ISL_702146, EPI_ISL_702147, EPI_ISL_702149, EPI_ISL_702152, EPI_ISL_702153, EPI_ISL_702155, EPI_ISL_702157, EPI_ISL_702158, EPI_ISL_702159, EPI_ISL_702160, EPI_ISL_702161, EPI_ISL_702162, EPI_ISL_702163, EPI_ISL_702166, EPI_ISL_702167, EPI_ISL_702168, EPI_ISL_702170, EPI_ISL_702171, EPI_ISL_702172, EPI_ISL_702177, EPI_ISL_702179, EPI_ISL_702181, EPI_ISL_702182, EPI_ISL_702183, EPI_ISL_702184, EPI_ISL_702192, EPI_ISL_702193, EPI_ISL_702198, EPI_ISL_702200, EPI_ISL_702201, EPI_ISL_702202, EPI_ISL_702203, EPI_ISL_702205, EPI_ISL_702206, EPI_ISL_702207, EPI_ISL_702209, EPI_ISL_702210, EPI_ISL_702211, EPI_ISL_702212, EPI_ISL_702213, EPI_ISL_702214, EPI_ISL_702215, EPI_ISL_702216, EPI_ISL_702218, EPI_ISL_702219, EPI_ISL_702220, EPI_ISL_702221, EPI_ISL_702223, EPI_ISL_702224, EPI_ISL_702225, EPI_ISL_702226, EPI_ISL_702228, EPI_ISL_702229, EPI_ISL_702230, EPI_ISL_702231, EPI_ISL_702232, EPI_ISL_702233, EPI_ISL_702235, EPI_ISL_702237, EPI_ISL_702239, EPI_ISL_702240, EPI_ISL_702242, EPI_ISL_702243, EPI_ISL_702245, EPI_ISL_702247, EPI_ISL_702248, EPI_ISL_702249, EPI_ISL_702250, EPI_ISL_702251, EPI_ISL_702252, EPI_ISL_702254, EPI_ISL_702257, EPI_ISL_702258, EPI_ISL_702259, EPI_ISL_702261, EPI_ISL_702262, EPI_ISL_702263, EPI_ISL_702264, EPI_ISL_702266, EPI_ISL_702267, EPI_ISL_702268, EPI_ISL_702271, EPI_ISL_702272, EPI_ISL_702273, EPI_ISL_702274, EPI_ISL_702276, EPI_ISL_702277, EPI_ISL_702281, EPI_ISL_702284, EPI_ISL_702285, EPI_ISL_702286, EPI_ISL_702288, EPI_ISL_702289, EPI_ISL_702291, EPI_ISL_702292, EPI_ISL_702293, EPI_ISL_702294, EPI_ISL_702296, EPI_ISL_702297, EPI_ISL_702298, EPI_ISL_702299, EPI_ISL_702302, EPI_ISL_702303, EPI_ISL_702305, EPI_ISL_702306, EPI_ISL_702308, EPI_ISL_702309, EPI_ISL_702311, EPI_ISL_702312, EPI_ISL_702314, EPI_ISL_702315, EPI_ISL_702316, EPI_ISL_702317, EPI_ISL_702320, EPI_ISL_702322, EPI_ISL_702323, EPI_ISL_702324, EPI_ISL_702329, EPI_ISL_702330, EPI_ISL_702333, EPI_ISL_702335, EPI_ISL_702336, EPI_ISL_702337, EPI_ISL_702338, EPI_ISL_702339, EPI_ISL_702340, EPI_ISL_702341, EPI_ISL_702344, EPI_ISL_702345, EPI_ISL_702346, EPI_ISL_702350, EPI_ISL_702352, EPI_ISL_702353, EPI_ISL_702356, EPI_ISL_702358, EPI_ISL_702359, EPI_ISL_702360, EPI_ISL_702361, EPI_ISL_702364, EPI_ISL_702365, EPI_ISL_702367, EPI_ISL_702368			
see above	Lighthouse Lab in Cambridge	Wellcome Sanger Institute for the COVID-19 Genomics UK (COG-UK) Consortium	Rob Howes, The Lighthouse Lab in Cambridge and Alex Alderton, Roberto Amato, Sonia Goncalves, Ewan Harrison, David K. Jackson, Ian Johnston, Dominic Kwiatkowski, Cordelia Langford, John Sillitoe on behalf of the Wellcome Sanger Institute COVID-19 Surveillance Team
EPI_ISL_702370, EPI_ISL_702371, EPI_ISL_702372, EPI_ISL_702373, EPI_ISL_702374, EPI_ISL_702375, EPI_ISL_702376, EPI_ISL_702377, EPI_ISL_702379, EPI_ISL_702382, EPI_ISL_702384, EPI_ISL_702388, EPI_ISL_702389, EPI_ISL_702390, EPI_ISL_702394, EPI_ISL_702396, EPI_ISL_702397, EPI_ISL_702398, EPI_ISL_702399, EPI_ISL_702401, EPI_ISL_702405, EPI_ISL_702406, EPI_ISL_702407, EPI_ISL_702408, EPI_ISL_702410, EPI_ISL_702412, EPI_ISL_702413, EPI_ISL_702414, EPI_ISL_702415, EPI_ISL_702417, EPI_ISL_702418, EPI_ISL_702424, EPI_ISL_702425, EPI_ISL_702427, EPI_ISL_702428, EPI_ISL_702429, EPI_ISL_702430, EPI_ISL_702431, EPI_ISL_702437, EPI_ISL_702439, EPI_ISL_702440, EPI_ISL_702441, EPI_ISL_702442, EPI_ISL_702444, EPI_ISL_702445, EPI_ISL_702447, EPI_ISL_702448, EPI_ISL_702450, EPI_ISL_702451, EPI_ISL_702452, EPI_ISL_702454, EPI_ISL_702456, EPI_ISL_702457, EPI_ISL_702458, EPI_ISL_702461, EPI_ISL_702462, EPI_ISL_702463, EPI_ISL_702465, EPI_ISL_702475, EPI_ISL_702479, EPI_ISL_702480, EPI_ISL_702485, EPI_ISL_702491, EPI_ISL_702494, EPI_ISL_702497, EPI_ISL_702499, EPI_ISL_702501, EPI_ISL_702502, EPI_ISL_702506, EPI_ISL_702509, EPI_ISL_702514, EPI_ISL_702516, EPI_ISL_702517, EPI_ISL_702522, EPI_ISL_702524, EPI_ISL_702527, EPI_ISL_702529, EPI_ISL_702530, EPI_ISL_702532, EPI_ISL_702534, EPI_ISL_702535, EPI_ISL_702538, EPI_ISL_702542, EPI_ISL_702546, EPI_ISL_702548, EPI_ISL_702555, EPI_ISL_702557, EPI_ISL_702563, EPI_ISL_702565, EPI_ISL_702570, EPI_ISL_702572, EPI_ISL_702573, EPI_ISL_702575, EPI_ISL_702576, EPI_ISL_702578, EPI_ISL_702583, EPI_ISL_702589, EPI_ISL_702593, EPI_ISL_702595, EPI_ISL_702596, EPI_ISL_702598, EPI_ISL_702599, EPI_ISL_702601, EPI_ISL_702606, EPI_ISL_702608, EPI_ISL_702611, EPI_ISL_702613, EPI_ISL_702614, EPI_ISL_702616, EPI_ISL_702617, EPI_ISL_702619, EPI_ISL_702622, EPI_ISL_702624, EPI_ISL_702627, EPI_ISL_702629, EPI_ISL_702630, EPI_ISL_702634, EPI_ISL_702635, EPI_ISL_702637, EPI_ISL_702640, EPI_ISL_702643, EPI_ISL_702645, EPI_ISL_702646, EPI_ISL_702650, EPI_ISL_702651, EPI_ISL_702653, EPI_ISL_702654, EPI_ISL_702658, EPI_ISL_702660, EPI_ISL_702662, EPI_ISL_702668, EPI_ISL_702670, EPI_ISL_702671, EPI_ISL_702673, EPI_ISL_702678, EPI_ISL_702680, EPI_ISL_702683, EPI_ISL_702686, EPI_ISL_702688, EPI_ISL_702689, EPI_ISL_702691, EPI_ISL_702696, EPI_ISL_702701, EPI_ISL_702702, EPI_ISL_702704, EPI_ISL_702706, EPI_ISL_702711, EPI_ISL_702712, EPI_ISL_702714, EPI_ISL_702717, EPI_ISL_702719, EPI_ISL_702724, EPI_ISL_702725, EPI_ISL_702730, EPI_ISL_702732, EPI_ISL_702733, EPI_ISL_702735, EPI_ISL_702737, EPI_ISL_702745, EPI_ISL_702748, EPI_ISL_702750, EPI_ISL_702752, EPI_ISL_702757, EPI_ISL_702760, EPI_ISL_702762, EPI_ISL_702765, EPI_ISL_702767, EPI_ISL_702768, EPI_ISL_702772, EPI_ISL_702773, EPI_ISL_702775, EPI_ISL_702777, EPI_ISL_702778, EPI_ISL_702781, EPI_ISL_702783, EPI_ISL_702786, EPI_ISL_702788, EPI_ISL_702791, EPI_ISL_702793, EPI_ISL_702796, EPI_ISL_702797, EPI_ISL_702799, EPI_ISL_702800, EPI_ISL_702802, EPI_ISL_702804, EPI_ISL_702807, EPI_ISL_702810, EPI_ISL_702811, EPI_ISL_702816, EPI_ISL_702818, EPI_ISL_702821, EPI_ISL_702822, EPI_ISL_702906, EPI_ISL_702909, EPI_ISL_702912, EPI_ISL_702914, EPI_ISL_702916, EPI_ISL_702918, EPI_ISL_702921, EPI_ISL_702923, EPI_ISL_702924, EPI_ISL_702927, EPI_ISL_702931, EPI_ISL_702933, EPI_ISL_702936, EPI_ISL_702938, EPI_ISL_702940, EPI_ISL_702942, EPI_ISL_702943, EPI_ISL_702945, EPI_ISL_702954, EPI_ISL_702955, EPI_ISL_702957, EPI_ISL_702958, EPI_ISL_702960, EPI_ISL_702963, EPI_ISL_702965, EPI_ISL_702967, EPI_ISL_702968, EPI_ISL_702973, EPI_ISL_702975, EPI_ISL_702976, EPI_ISL_702978, EPI_ISL_702979, EPI_ISL_702981, EPI_ISL_702983, EPI_ISL_702986, EPI_ISL_702988, EPI_ISL_702989, EPI_ISL_702991, EPI_ISL_702993, EPI_ISL_702995, EPI_ISL_702998, EPI_ISL_703000, EPI_ISL_703003, EPI_ISL_703004, EPI_ISL_703009, EPI_ISL_703012, EPI_ISL_703014, EPI_ISL_703017, EPI_ISL_703019, EPI_ISL_703022, EPI_ISL_703027, EPI_ISL_703028, EPI_ISL_703030, EPI_ISL_703031, EPI_ISL_703033, EPI_ISL_703035, EPI_ISL_703037, EPI_ISL_703039, EPI_ISL_703044, EPI_ISL_703050,			

EPI_ISL_703052, EPI_ISL_703054, EPI_ISL_703055, EPI_ISL_703058, EPI_ISL_703063, EPI_ISL_703065, EPI_ISL_703068, EPI_ISL_703070, EPI_ISL_703075, EPI_ISL_703078, EPI_ISL_703079, EPI_ISL_703081, EPI_ISL_703083, EPI_ISL_703084, EPI_ISL_703091, EPI_ISL_703092, EPI_ISL_703094, EPI_ISL_703095, EPI_ISL_703098, EPI_ISL_703100, EPI_ISL_703101, EPI_ISL_703103, EPI_ISL_703106, EPI_ISL_703108, EPI_ISL_703109, EPI_ISL_703112, EPI_ISL_703116, EPI_ISL_703117, EPI_ISL_703120, EPI_ISL_703122, EPI_ISL_703123, EPI_ISL_703125, EPI_ISL_703128, EPI_ISL_703131, EPI_ISL_703134, EPI_ISL_703135, EPI_ISL_703140, EPI_ISL_703142, EPI_ISL_703143, EPI_ISL_703145, EPI_ISL_703146, EPI_ISL_703151, EPI_ISL_703153, EPI_ISL_703154, EPI_ISL_703156, EPI_ISL_703157, EPI_ISL_703159, EPI_ISL_703160, EPI_ISL_703161, EPI_ISL_703163, EPI_ISL_703164, EPI_ISL_703166, EPI_ISL_703168, EPI_ISL_703169, EPI_ISL_703171, EPI_ISL_703173, EPI_ISL_703180, EPI_ISL_703183, EPI_ISL_703185, EPI_ISL_703188, EPI_ISL_703189, EPI_ISL_703191, EPI_ISL_703193, EPI_ISL_703194, EPI_ISL_703196, EPI_ISL_703199, EPI_ISL_703201, EPI_ISL_703202, EPI_ISL_703204, EPI_ISL_703209, EPI_ISL_703212, EPI_ISL_703214, EPI_ISL_703215, EPI_ISL_703217, EPI_ISL_703218, EPI_ISL_703220, EPI_ISL_703221, EPI_ISL_703224, EPI_ISL_703226, EPI_ISL_703231, EPI_ISL_703232, EPI_ISL_703234, EPI_ISL_703236, EPI_ISL_703237, EPI_ISL_703239, EPI_ISL_703242, EPI_ISL_703244, EPI_ISL_703247, EPI_ISL_703250, EPI_ISL_703252, EPI_ISL_703257

see above	Lighthouse Lab in Alderley Park	Wellcome Sanger Institute for the COVID-19 Genomics UK (COG-UK) Consortium	Jacquelyn Wynn, Mairead Hyland, The Lighthouse Lab in Alderley Park and Alex Alderton, Roberto Amato, Sonia Goncalves, Ewan Harrison, David K. Jackson, Ian Johnston, Dominic Kwiatkowski, Cordelia Langford, John Sillitoe on behalf of the Wellcome Sanger Institute COVID-19 Surveillance Team
EPI_ISL_703262, EPI_ISL_703263	Lighthouse Lab in Glasgow	Wellcome Sanger Institute for the COVID-19 Genomics UK (COG-UK) Consortium	Harper VanSteenhouse, Yumi Kasai, David Gray, Carol Clugston, Anna Dominiczak and Alex Alderton, Roberto Amato, Sonia Goncalves, Ewan Harrison, David K. Jackson, Ian Johnston, Dominic Kwiatkowski, Cordelia Langford, John Sillitoe on behalf of the Wellcome Sanger Institute COVID-19 Surveillance Team
EPI_ISL_703265, EPI_ISL_703266, EPI_ISL_703268	Lighthouse Lab in Alderley Park	Wellcome Sanger Institute for the COVID-19 Genomics UK (COG-UK) Consortium	Jacquelyn Wynn, Mairead Hyland, The Lighthouse Lab in Alderley Park and Alex Alderton, Roberto Amato, Sonia Goncalves, Ewan Harrison, David K. Jackson, Ian Johnston, Dominic Kwiatkowski, Cordelia Langford, John Sillitoe on behalf of the Wellcome Sanger Institute COVID-19 Surveillance Team
EPI_ISL_703270	Lighthouse Lab in Glasgow	Wellcome Sanger Institute for the COVID-19 Genomics UK (COG-UK) Consortium	Harper VanSteenhouse, Yumi Kasai, David Gray, Carol Clugston, Anna Dominiczak and Alex Alderton, Roberto Amato, Sonia Goncalves, Ewan Harrison, David K. Jackson, Ian Johnston, Dominic Kwiatkowski, Cordelia Langford, John Sillitoe on behalf of the Wellcome Sanger Institute COVID-19 Surveillance Team
EPI_ISL_703274, EPI_ISL_703277	Lighthouse Lab in Alderley Park	Wellcome Sanger Institute for the COVID-19 Genomics UK (COG-UK) Consortium	Jacquelyn Wynn, Mairead Hyland, The Lighthouse Lab in Alderley Park and Alex Alderton, Roberto Amato, Sonia Goncalves, Ewan Harrison, David K. Jackson, Ian Johnston, Dominic Kwiatkowski, Cordelia Langford, John Sillitoe on behalf of the Wellcome Sanger Institute COVID-19 Surveillance Team
EPI_ISL_703280, EPI_ISL_703285, EPI_ISL_703286, EPI_ISL_703290, EPI_ISL_703291	Lighthouse Lab in Glasgow	Wellcome Sanger Institute for the COVID-19 Genomics UK (COG-UK) Consortium	Harper VanSteenhouse, Yumi Kasai, David Gray, Carol Clugston, Anna Dominiczak and Alex Alderton, Roberto Amato, Sonia Goncalves, Ewan Harrison, David K. Jackson, Ian Johnston, Dominic Kwiatkowski, Cordelia Langford, John Sillitoe on behalf of the Wellcome Sanger Institute COVID-19 Surveillance Team
EPI_ISL_703294	Lighthouse Lab in Alderley Park	Wellcome Sanger Institute for the COVID-19 Genomics UK (COG-UK) Consortium	Jacquelyn Wynn, Mairead Hyland, The Lighthouse Lab in Alderley Park and Alex Alderton, Roberto Amato, Sonia Goncalves, Ewan Harrison, David K. Jackson, Ian Johnston, Dominic Kwiatkowski, Cordelia Langford, John Sillitoe on behalf of the Wellcome Sanger Institute COVID-19 Surveillance Team
EPI_ISL_703296	Lighthouse Lab in Glasgow	Wellcome Sanger Institute for the COVID-19 Genomics UK (COG-UK) Consortium	Harper VanSteenhouse, Yumi Kasai, David Gray, Carol Clugston, Anna Dominiczak and Alex Alderton, Roberto Amato, Sonia Goncalves, Ewan Harrison, David K. Jackson, Ian Johnston, Dominic Kwiatkowski, Cordelia Langford, John Sillitoe on behalf of the Wellcome Sanger Institute COVID-19 Surveillance Team
EPI_ISL_703298	Lighthouse Lab in Alderley Park	Wellcome Sanger Institute for the COVID-19 Genomics UK (COG-UK) Consortium	Jacquelyn Wynn, Mairead Hyland, The Lighthouse Lab in Alderley Park and Alex Alderton, Roberto Amato, Sonia Goncalves, Ewan Harrison, David K. Jackson, Ian Johnston, Dominic Kwiatkowski, Cordelia Langford, John Sillitoe on behalf of the Wellcome Sanger Institute COVID-19 Surveillance Team
EPI_ISL_703300	Lighthouse Lab in Glasgow	Wellcome Sanger Institute for the COVID-19 Genomics UK (COG-UK) Consortium	Harper VanSteenhouse, Yumi Kasai, David Gray, Carol Clugston, Anna Dominiczak and Alex Alderton, Roberto Amato, Sonia Goncalves, Ewan Harrison, David K. Jackson, Ian Johnston, Dominic Kwiatkowski, Cordelia Langford, John Sillitoe on behalf of the Wellcome Sanger Institute COVID-19 Surveillance Team
EPI_ISL_703301	Lighthouse Lab in Alderley Park	Wellcome Sanger Institute for the COVID-19 Genomics UK (COG-UK) Consortium	Jacquelyn Wynn, Mairead Hyland, The Lighthouse Lab in Alderley Park and Alex Alderton, Roberto Amato, Sonia Goncalves, Ewan Harrison, David K. Jackson, Ian Johnston, Dominic Kwiatkowski, Cordelia Langford, John Sillitoe on behalf of the Wellcome Sanger Institute COVID-19 Surveillance Team
EPI_ISL_703304	Lighthouse Lab in Glasgow	Wellcome Sanger Institute for the COVID-19 Genomics UK (COG-UK) Consortium	Harper VanSteenhouse, Yumi Kasai, David Gray, Carol Clugston, Anna Dominiczak and Alex Alderton, Roberto Amato, Sonia Goncalves, Ewan Harrison, David K. Jackson, Ian Johnston, Dominic Kwiatkowski, Cordelia Langford, John Sillitoe on behalf of the Wellcome Sanger Institute COVID-19 Surveillance Team
EPI_ISL_703306, EPI_ISL_703307	Lighthouse Lab in Alderley Park	Wellcome Sanger Institute for the COVID-19 Genomics UK (COG-UK) Consortium	Jacquelyn Wynn, Mairead Hyland, The Lighthouse Lab in Alderley Park and Alex Alderton, Roberto Amato, Sonia Goncalves, Ewan Harrison, David K. Jackson, Ian Johnston, Dominic Kwiatkowski, Cordelia Langford, John Sillitoe on behalf of the Wellcome Sanger Institute COVID-19 Surveillance Team
EPI_ISL_703309	Lighthouse Lab in Glasgow	Wellcome Sanger Institute for the COVID-19 Genomics UK (COG-UK) Consortium	Harper VanSteenhouse, Yumi Kasai, David Gray, Carol Clugston, Anna Dominiczak and Alex Alderton, Roberto Amato, Sonia Goncalves, Ewan Harrison, David K. Jackson, Ian Johnston, Dominic Kwiatkowski, Cordelia Langford, John Sillitoe on behalf of the Wellcome Sanger Institute COVID-19 Surveillance Team
EPI_ISL_703311, EPI_ISL_703312, EPI_ISL_703314, EPI_ISL_703317, EPI_ISL_703318	Lighthouse Lab in Alderley Park	Wellcome Sanger Institute for the COVID-19 Genomics UK (COG-UK) Consortium	Jacquelyn Wynn, Mairead Hyland, The Lighthouse Lab in Alderley Park and Alex Alderton, Roberto Amato, Sonia Goncalves, Ewan Harrison, David K. Jackson, Ian Johnston, Dominic Kwiatkowski, Cordelia Langford, John Sillitoe on behalf of the Wellcome Sanger Institute COVID-19 Surveillance Team
EPI_ISL_703320	Lighthouse Lab in Glasgow	Wellcome Sanger Institute for the COVID-19 Genomics UK (COG-UK) Consortium	Harper VanSteenhouse, Yumi Kasai, David Gray, Carol Clugston, Anna Dominiczak and Alex Alderton, Roberto Amato, Sonia Goncalves, Ewan Harrison, David K. Jackson, Ian Johnston, Dominic Kwiatkowski, Cordelia Langford, John Sillitoe on behalf of the Wellcome Sanger Institute COVID-19 Surveillance Team
EPI_ISL_703322	Lighthouse Lab in Alderley Park	Wellcome Sanger Institute for the COVID-19 Genomics UK (COG-UK) Consortium	Jacquelyn Wynn, Mairead Hyland, The Lighthouse Lab in Alderley Park and Alex Alderton, Roberto Amato, Sonia Goncalves, Ewan Harrison, David K. Jackson, Ian Johnston, Dominic Kwiatkowski, Cordelia Langford, John Sillitoe on behalf of the Wellcome Sanger Institute COVID-19 Surveillance Team
EPI_ISL_703323, EPI_ISL_703325	Lighthouse Lab in Glasgow	Wellcome Sanger Institute for the COVID-19 Genomics UK (COG-UK) Consortium	Harper VanSteenhouse, Yumi Kasai, David Gray, Carol Clugston, Anna Dominiczak and Alex Alderton, Roberto Amato, Sonia Goncalves, Ewan Harrison, David K. Jackson, Ian Johnston, Dominic Kwiatkowski, Cordelia Langford, John Sillitoe on behalf of the Wellcome Sanger Institute COVID-19 Surveillance Team
EPI_ISL_703328, EPI_ISL_703329	Lighthouse Lab in Alderley Park	Wellcome Sanger Institute for the COVID-19 Genomics UK (COG-UK) Consortium	Jacquelyn Wynn, Mairead Hyland, The Lighthouse Lab in Alderley Park and Alex Alderton, Roberto Amato, Sonia Goncalves, Ewan Harrison, David K. Jackson, Ian Johnston, Dominic Kwiatkowski, Cordelia Langford, John Sillitoe on behalf of the Wellcome Sanger Institute COVID-19 Surveillance Team
EPI_ISL_703331	Lighthouse Lab in Glasgow	Wellcome Sanger Institute for the COVID-19 Genomics UK (COG-UK) Consortium	Harper VanSteenhouse, Yumi Kasai, David Gray, Carol Clugston, Anna Dominiczak and Alex Alderton, Roberto Amato, Sonia Goncalves, Ewan Harrison, David K. Jackson, Ian Johnston, Dominic Kwiatkowski, Cordelia Langford, John Sillitoe on behalf of the Wellcome Sanger Institute COVID-19 Surveillance Team
EPI_ISL_703333	Lighthouse Lab in Alderley Park	Wellcome Sanger Institute for the COVID-19 Genomics UK (COG-UK) Consortium	Jacquelyn Wynn, Mairead Hyland, The Lighthouse Lab in Alderley Park and Alex Alderton, Roberto Amato, Sonia Goncalves, Ewan Harrison, David K. Jackson, Ian Johnston, Dominic Kwiatkowski, Cordelia Langford, John Sillitoe on behalf of the Wellcome Sanger Institute COVID-19 Surveillance Team
EPI_ISL_703337	Lighthouse Lab in Glasgow	Wellcome Sanger Institute for the COVID-19 Genomics UK (COG-UK) Consortium	Harper VanSteenhouse, Yumi Kasai, David Gray, Carol Clugston, Anna Dominiczak and Alex Alderton, Roberto Amato, Sonia Goncalves, Ewan Harrison, David K. Jackson, Ian Johnston, Dominic Kwiatkowski, Cordelia Langford, John Sillitoe on behalf of the Wellcome Sanger Institute COVID-19 Surveillance Team
EPI_ISL_703339, EPI_ISL_703341	Lighthouse Lab in Alderley Park	Wellcome Sanger Institute for the COVID-19 Genomics UK (COG-UK) Consortium	Jacquelyn Wynn, Mairead Hyland, The Lighthouse Lab in Alderley Park and Alex Alderton, Roberto Amato, Sonia Goncalves, Ewan Harrison, David K. Jackson, Ian Johnston, Dominic Kwiatkowski, Cordelia Langford, John Sillitoe on behalf of the Wellcome Sanger Institute COVID-19 Surveillance Team
EPI_ISL_703345	Lighthouse Lab in Glasgow	Wellcome Sanger Institute for the COVID-19 Genomics UK (COG-UK) Consortium	Harper VanSteenhouse, Yumi Kasai, David Gray, Carol Clugston, Anna Dominiczak and Alex Alderton, Roberto Amato, Sonia Goncalves, Ewan Harrison, David K. Jackson, Ian Johnston, Dominic Kwiatkowski, Cordelia Langford, John Sillitoe on behalf of the Wellcome Sanger Institute COVID-19 Surveillance Team
EPI_ISL_703347	Lighthouse Lab in Alderley Park	Wellcome Sanger Institute for the COVID-19 Genomics UK (COG-UK) Consortium	Jacquelyn Wynn, Mairead Hyland, The Lighthouse Lab in Alderley Park and Alex Alderton, Roberto Amato, Sonia Goncalves, Ewan Harrison, David K. Jackson, Ian Johnston, Dominic Kwiatkowski, Cordelia Langford, John Sillitoe on behalf of the Wellcome Sanger Institute COVID-19 Surveillance Team
EPI_ISL_703355, EPI_ISL_703358	Lighthouse Lab in Glasgow	Wellcome Sanger Institute for the COVID-19 Genomics UK (COG-UK) Consortium	Harper VanSteenhouse, Yumi Kasai, David Gray, Carol Clugston, Anna Dominiczak and Alex Alderton, Roberto Amato, Sonia Goncalves, Ewan Harrison, David K. Jackson, Ian Johnston, Dominic Kwiatkowski, Cordelia Langford, John Sillitoe on behalf of the Wellcome Sanger Institute COVID-19 Surveillance Team
EPI_ISL_703365, EPI_ISL_703368	Lighthouse Lab in Alderley Park	Wellcome Sanger Institute for the COVID-19 Genomics UK (COG-UK) Consortium	Jacquelyn Wynn, Mairead Hyland, The Lighthouse Lab in Alderley Park and Alex Alderton, Roberto Amato, Sonia Goncalves, Ewan Harrison, David K. Jackson, Ian Johnston, Dominic Kwiatkowski, Cordelia Langford, John Sillitoe on behalf of the Wellcome Sanger Institute COVID-19 Surveillance Team

EPI_ISL_703370	Lighthouse Lab in Glasgow	Wellcome Sanger Institute for the COVID-19 Genomics UK (COG-UK) Consortium	Harper VanSteenhouse, Yumi Kasai, David Gray, Carol Clugston, Anna Dominiczak and Alex Alderton, Roberto Amato, Sonia Goncalves, Ewan Harrison, David K. Jackson, Ian Johnston, Dominic Kwiatkowski, Cordelia Langford, John Sillitoe on behalf of the Wellcome Sanger Institute COVID-19 Surveillance Team
EPI_ISL_703374, EPI_ISL_703376	Lighthouse Lab in Alderley Park	Wellcome Sanger Institute for the COVID-19 Genomics UK (COG-UK) Consortium	Jacquelyn Wynn, Mairead Hyland, The Lighthouse Lab in Alderley Park and Alex Alderton, Roberto Amato, Sonia Goncalves, Ewan Harrison, David K. Jackson, Ian Johnston, Dominic Kwiatkowski, Cordelia Langford, John Sillitoe on behalf of the Wellcome Sanger Institute COVID-19 Surveillance Team
EPI_ISL_703381, EPI_ISL_703382, EPI_ISL_703384	Lighthouse Lab in Glasgow	Wellcome Sanger Institute for the COVID-19 Genomics UK (COG-UK) Consortium	Harper VanSteenhouse, Yumi Kasai, David Gray, Carol Clugston, Anna Dominiczak and Alex Alderton, Roberto Amato, Sonia Goncalves, Ewan Harrison, David K. Jackson, Ian Johnston, Dominic Kwiatkowski, Cordelia Langford, John Sillitoe on behalf of the Wellcome Sanger Institute COVID-19 Surveillance Team
EPI_ISL_703388	Lighthouse Lab in Alderley Park	Wellcome Sanger Institute for the COVID-19 Genomics UK (COG-UK) Consortium	Jacquelyn Wynn, Mairead Hyland, The Lighthouse Lab in Alderley Park and Alex Alderton, Roberto Amato, Sonia Goncalves, Ewan Harrison, David K. Jackson, Ian Johnston, Dominic Kwiatkowski, Cordelia Langford, John Sillitoe on behalf of the Wellcome Sanger Institute COVID-19 Surveillance Team
EPI_ISL_703393	Lighthouse Lab in Glasgow	Wellcome Sanger Institute for the COVID-19 Genomics UK (COG-UK) Consortium	Harper VanSteenhouse, Yumi Kasai, David Gray, Carol Clugston, Anna Dominiczak and Alex Alderton, Roberto Amato, Sonia Goncalves, Ewan Harrison, David K. Jackson, Ian Johnston, Dominic Kwiatkowski, Cordelia Langford, John Sillitoe on behalf of the Wellcome Sanger Institute COVID-19 Surveillance Team
EPI_ISL_703394, EPI_ISL_703396, EPI_ISL_703397	Lighthouse Lab in Alderley Park	Wellcome Sanger Institute for the COVID-19 Genomics UK (COG-UK) Consortium	Jacquelyn Wynn, Mairead Hyland, The Lighthouse Lab in Alderley Park and Alex Alderton, Roberto Amato, Sonia Goncalves, Ewan Harrison, David K. Jackson, Ian Johnston, Dominic Kwiatkowski, Cordelia Langford, John Sillitoe on behalf of the Wellcome Sanger Institute COVID-19 Surveillance Team
EPI_ISL_703401	Lighthouse Lab in Glasgow	Wellcome Sanger Institute for the COVID-19 Genomics UK (COG-UK) Consortium	Harper VanSteenhouse, Yumi Kasai, David Gray, Carol Clugston, Anna Dominiczak and Alex Alderton, Roberto Amato, Sonia Goncalves, Ewan Harrison, David K. Jackson, Ian Johnston, Dominic Kwiatkowski, Cordelia Langford, John Sillitoe on behalf of the Wellcome Sanger Institute COVID-19 Surveillance Team
EPI_ISL_703402, EPI_ISL_703404	Lighthouse Lab in Alderley Park	Wellcome Sanger Institute for the COVID-19 Genomics UK (COG-UK) Consortium	Jacquelyn Wynn, Mairead Hyland, The Lighthouse Lab in Alderley Park and Alex Alderton, Roberto Amato, Sonia Goncalves, Ewan Harrison, David K. Jackson, Ian Johnston, Dominic Kwiatkowski, Cordelia Langford, John Sillitoe on behalf of the Wellcome Sanger Institute COVID-19 Surveillance Team
EPI_ISL_703408, EPI_ISL_703410	Lighthouse Lab in Glasgow	Wellcome Sanger Institute for the COVID-19 Genomics UK (COG-UK) Consortium	Harper VanSteenhouse, Yumi Kasai, David Gray, Carol Clugston, Anna Dominiczak and Alex Alderton, Roberto Amato, Sonia Goncalves, Ewan Harrison, David K. Jackson, Ian Johnston, Dominic Kwiatkowski, Cordelia Langford, John Sillitoe on behalf of the Wellcome Sanger Institute COVID-19 Surveillance Team
EPI_ISL_703418, EPI_ISL_703420, EPI_ISL_703425	Lighthouse Lab in Alderley Park	Wellcome Sanger Institute for the COVID-19 Genomics UK (COG-UK) Consortium	Jacquelyn Wynn, Mairead Hyland, The Lighthouse Lab in Alderley Park and Alex Alderton, Roberto Amato, Sonia Goncalves, Ewan Harrison, David K. Jackson, Ian Johnston, Dominic Kwiatkowski, Cordelia Langford, John Sillitoe on behalf of the Wellcome Sanger Institute COVID-19 Surveillance Team
EPI_ISL_703429, EPI_ISL_703431	Lighthouse Lab in Glasgow	Wellcome Sanger Institute for the COVID-19 Genomics UK (COG-UK) Consortium	Harper VanSteenhouse, Yumi Kasai, David Gray, Carol Clugston, Anna Dominiczak and Alex Alderton, Roberto Amato, Sonia Goncalves, Ewan Harrison, David K. Jackson, Ian Johnston, Dominic Kwiatkowski, Cordelia Langford, John Sillitoe on behalf of the Wellcome Sanger Institute COVID-19 Surveillance Team
EPI_ISL_703433, EPI_ISL_703436, EPI_ISL_703437	Lighthouse Lab in Alderley Park	Wellcome Sanger Institute for the COVID-19 Genomics UK (COG-UK) Consortium	Jacquelyn Wynn, Mairead Hyland, The Lighthouse Lab in Alderley Park and Alex Alderton, Roberto Amato, Sonia Goncalves, Ewan Harrison, David K. Jackson, Ian Johnston, Dominic Kwiatkowski, Cordelia Langford, John Sillitoe on behalf of the Wellcome Sanger Institute COVID-19 Surveillance Team
EPI_ISL_703439	Lighthouse Lab in Glasgow	Wellcome Sanger Institute for the COVID-19 Genomics UK (COG-UK) Consortium	Harper VanSteenhouse, Yumi Kasai, David Gray, Carol Clugston, Anna Dominiczak and Alex Alderton, Roberto Amato, Sonia Goncalves, Ewan Harrison, David K. Jackson, Ian Johnston, Dominic Kwiatkowski, Cordelia Langford, John Sillitoe on behalf of the Wellcome Sanger Institute COVID-19 Surveillance Team
EPI_ISL_703441	Lighthouse Lab in Alderley Park	Wellcome Sanger Institute for the COVID-19 Genomics UK (COG-UK) Consortium	Jacquelyn Wynn, Mairead Hyland, The Lighthouse Lab in Alderley Park and Alex Alderton, Roberto Amato, Sonia Goncalves, Ewan Harrison, David K. Jackson, Ian Johnston, Dominic Kwiatkowski, Cordelia Langford, John Sillitoe on behalf of the Wellcome Sanger Institute COVID-19 Surveillance Team
EPI_ISL_703442, EPI_ISL_703444, EPI_ISL_703445, EPI_ISL_703447	Lighthouse Lab in Glasgow	Wellcome Sanger Institute for the COVID-19 Genomics UK (COG-UK) Consortium	Harper VanSteenhouse, Yumi Kasai, David Gray, Carol Clugston, Anna Dominiczak and Alex Alderton, Roberto Amato, Sonia Goncalves, Ewan Harrison, David K. Jackson, Ian Johnston, Dominic Kwiatkowski, Cordelia Langford, John Sillitoe on behalf of the Wellcome Sanger Institute COVID-19 Surveillance Team
EPI_ISL_703448, EPI_ISL_703450	Lighthouse Lab in Alderley Park	Wellcome Sanger Institute for the COVID-19 Genomics UK (COG-UK) Consortium	Jacquelyn Wynn, Mairead Hyland, The Lighthouse Lab in Alderley Park and Alex Alderton, Roberto Amato, Sonia Goncalves, Ewan Harrison, David K. Jackson, Ian Johnston, Dominic Kwiatkowski, Cordelia Langford, John Sillitoe on behalf of the Wellcome Sanger Institute COVID-19 Surveillance Team
EPI_ISL_703453, EPI_ISL_703456, EPI_ISL_703463	Lighthouse Lab in Glasgow	Wellcome Sanger Institute for the COVID-19 Genomics UK (COG-UK) Consortium	Harper VanSteenhouse, Yumi Kasai, David Gray, Carol Clugston, Anna Dominiczak and Alex Alderton, Roberto Amato, Sonia Goncalves, Ewan Harrison, David K. Jackson, Ian Johnston, Dominic Kwiatkowski, Cordelia Langford, John Sillitoe on behalf of the Wellcome Sanger Institute COVID-19 Surveillance Team
EPI_ISL_703464, EPI_ISL_703467	Lighthouse Lab in Alderley Park	Wellcome Sanger Institute for the COVID-19 Genomics UK (COG-UK) Consortium	Jacquelyn Wynn, Mairead Hyland, The Lighthouse Lab in Alderley Park and Alex Alderton, Roberto Amato, Sonia Goncalves, Ewan Harrison, David K. Jackson, Ian Johnston, Dominic Kwiatkowski, Cordelia Langford, John Sillitoe on behalf of the Wellcome Sanger Institute COVID-19 Surveillance Team
EPI_ISL_703474	Lighthouse Lab in Glasgow	Wellcome Sanger Institute for the COVID-19 Genomics UK (COG-UK) Consortium	Harper VanSteenhouse, Yumi Kasai, David Gray, Carol Clugston, Anna Dominiczak and Alex Alderton, Roberto Amato, Sonia Goncalves, Ewan Harrison, David K. Jackson, Ian Johnston, Dominic Kwiatkowski, Cordelia Langford, John Sillitoe on behalf of the Wellcome Sanger Institute COVID-19 Surveillance Team
EPI_ISL_703475, EPI_ISL_703478, EPI_ISL_703483, EPI_ISL_703490	Lighthouse Lab in Alderley Park	Wellcome Sanger Institute for the COVID-19 Genomics UK (COG-UK) Consortium	Jacquelyn Wynn, Mairead Hyland, The Lighthouse Lab in Alderley Park and Alex Alderton, Roberto Amato, Sonia Goncalves, Ewan Harrison, David K. Jackson, Ian Johnston, Dominic Kwiatkowski, Cordelia Langford, John Sillitoe on behalf of the Wellcome Sanger Institute COVID-19 Surveillance Team
EPI_ISL_703491	Lighthouse Lab in Glasgow	Wellcome Sanger Institute for the COVID-19 Genomics UK (COG-UK) Consortium	Harper VanSteenhouse, Yumi Kasai, David Gray, Carol Clugston, Anna Dominiczak and Alex Alderton, Roberto Amato, Sonia Goncalves, Ewan Harrison, David K. Jackson, Ian Johnston, Dominic Kwiatkowski, Cordelia Langford, John Sillitoe on behalf of the Wellcome Sanger Institute COVID-19 Surveillance Team
EPI_ISL_703493	Lighthouse Lab in Alderley Park	Wellcome Sanger Institute for the COVID-19 Genomics UK (COG-UK) Consortium	Jacquelyn Wynn, Mairead Hyland, The Lighthouse Lab in Alderley Park and Alex Alderton, Roberto Amato, Sonia Goncalves, Ewan Harrison, David K. Jackson, Ian Johnston, Dominic Kwiatkowski, Cordelia Langford, John Sillitoe on behalf of the Wellcome Sanger Institute COVID-19 Surveillance Team
EPI_ISL_703496	Lighthouse Lab in Glasgow	Wellcome Sanger Institute for the COVID-19 Genomics UK (COG-UK) Consortium	Harper VanSteenhouse, Yumi Kasai, David Gray, Carol Clugston, Anna Dominiczak and Alex Alderton, Roberto Amato, Sonia Goncalves, Ewan Harrison, David K. Jackson, Ian Johnston, Dominic Kwiatkowski, Cordelia Langford, John Sillitoe on behalf of the Wellcome Sanger Institute COVID-19 Surveillance Team
EPI_ISL_703498	Lighthouse Lab in Alderley Park	Wellcome Sanger Institute for the COVID-19 Genomics UK (COG-UK) Consortium	Jacquelyn Wynn, Mairead Hyland, The Lighthouse Lab in Alderley Park and Alex Alderton, Roberto Amato, Sonia Goncalves, Ewan Harrison, David K. Jackson, Ian Johnston, Dominic Kwiatkowski, Cordelia Langford, John Sillitoe on behalf of the Wellcome Sanger Institute COVID-19 Surveillance Team
EPI_ISL_703501	Lighthouse Lab in Glasgow	Wellcome Sanger Institute for the COVID-19 Genomics UK (COG-UK) Consortium	Harper VanSteenhouse, Yumi Kasai, David Gray, Carol Clugston, Anna Dominiczak and Alex Alderton, Roberto Amato, Sonia Goncalves, Ewan Harrison, David K. Jackson, Ian Johnston, Dominic Kwiatkowski, Cordelia Langford, John Sillitoe on behalf of the Wellcome Sanger Institute COVID-19 Surveillance Team
EPI_ISL_703503	Lighthouse Lab in Alderley Park	Wellcome Sanger Institute for the COVID-19 Genomics UK (COG-UK) Consortium	Jacquelyn Wynn, Mairead Hyland, The Lighthouse Lab in Alderley Park and Alex Alderton, Roberto Amato, Sonia Goncalves, Ewan Harrison, David K. Jackson, Ian Johnston, Dominic Kwiatkowski, Cordelia Langford, John Sillitoe on behalf of the Wellcome Sanger Institute COVID-19 Surveillance Team
EPI_ISL_703506, EPI_ISL_703514, EPI_ISL_703515	Lighthouse Lab in Glasgow	Wellcome Sanger Institute for the COVID-19 Genomics UK (COG-UK) Consortium	Harper VanSteenhouse, Yumi Kasai, David Gray, Carol Clugston, Anna Dominiczak and Alex Alderton, Roberto Amato, Sonia Goncalves, Ewan Harrison, David K. Jackson, Ian Johnston, Dominic Kwiatkowski, Cordelia Langford, John Sillitoe on behalf of the Wellcome Sanger Institute COVID-19 Surveillance Team
EPI_ISL_703518, EPI_ISL_703520	Lighthouse Lab in Alderley Park	Wellcome Sanger Institute for the COVID-19 Genomics UK (COG-UK) Consortium	Jacquelyn Wynn, Mairead Hyland, The Lighthouse Lab in Alderley Park and Alex Alderton, Roberto Amato, Sonia Goncalves, Ewan Harrison, David K. Jackson, Ian Johnston, Dominic Kwiatkowski, Cordelia Langford, John Sillitoe on behalf of the Wellcome Sanger Institute COVID-19 Surveillance Team
EPI_ISL_703522, EPI_ISL_703525	Lighthouse Lab in Glasgow	Wellcome Sanger Institute for the COVID-19 Genomics UK (COG-UK) Consortium	Harper VanSteenhouse, Yumi Kasai, David Gray, Carol Clugston, Anna Dominiczak and Alex Alderton, Roberto Amato, Sonia Goncalves, Ewan Harrison, David K. Jackson, Ian Johnston, Dominic Kwiatkowski, Cordelia Langford, John Sillitoe on behalf of the Wellcome Sanger Institute COVID-19 Surveillance Team

EPI_ISL_703526	Lighthouse Lab in Alderley Park	Wellcome Sanger Institute for the COVID-19 Genomics UK (COG-UK) Consortium	Jacquelyn Wynn, Mairead Hyland, The Lighthouse Lab in Alderley Park and Alex Alderton, Roberto Amato, Sonia Goncalves, Ewan Harrison, David K. Jackson, Ian Johnston, Dominic Kwiatkowski, Cordelia Langford, John Sillitoe on behalf of the Wellcome Sanger Institute COVID-19 Surveillance Team
EPI_ISL_703529	Lighthouse Lab in Glasgow	Wellcome Sanger Institute for the COVID-19 Genomics UK (COG-UK) Consortium	Harper VanSteenhouse, Yumi Kasai, David Gray, Carol Clugston, Anna Dominiczak and Alex Alderton, Roberto Amato, Sonia Goncalves, Ewan Harrison, David K. Jackson, Ian Johnston, Dominic Kwiatkowski, Cordelia Langford, John Sillitoe on behalf of the Wellcome Sanger Institute COVID-19 Surveillance Team
EPI_ISL_703531, EPI_ISL_703532	Lighthouse Lab in Alderley Park	Wellcome Sanger Institute for the COVID-19 Genomics UK (COG-UK) Consortium	Jacquelyn Wynn, Mairead Hyland, The Lighthouse Lab in Alderley Park and Alex Alderton, Roberto Amato, Sonia Goncalves, Ewan Harrison, David K. Jackson, Ian Johnston, Dominic Kwiatkowski, Cordelia Langford, John Sillitoe on behalf of the Wellcome Sanger Institute COVID-19 Surveillance Team
EPI_ISL_703534	Lighthouse Lab in Glasgow	Wellcome Sanger Institute for the COVID-19 Genomics UK (COG-UK) Consortium	Harper VanSteenhouse, Yumi Kasai, David Gray, Carol Clugston, Anna Dominiczak and Alex Alderton, Roberto Amato, Sonia Goncalves, Ewan Harrison, David K. Jackson, Ian Johnston, Dominic Kwiatkowski, Cordelia Langford, John Sillitoe on behalf of the Wellcome Sanger Institute COVID-19 Surveillance Team
EPI_ISL_703536	Lighthouse Lab in Alderley Park	Wellcome Sanger Institute for the COVID-19 Genomics UK (COG-UK) Consortium	Jacquelyn Wynn, Mairead Hyland, The Lighthouse Lab in Alderley Park and Alex Alderton, Roberto Amato, Sonia Goncalves, Ewan Harrison, David K. Jackson, Ian Johnston, Dominic Kwiatkowski, Cordelia Langford, John Sillitoe on behalf of the Wellcome Sanger Institute COVID-19 Surveillance Team
EPI_ISL_703538	Lighthouse Lab in Glasgow	Wellcome Sanger Institute for the COVID-19 Genomics UK (COG-UK) Consortium	Harper VanSteenhouse, Yumi Kasai, David Gray, Carol Clugston, Anna Dominiczak and Alex Alderton, Roberto Amato, Sonia Goncalves, Ewan Harrison, David K. Jackson, Ian Johnston, Dominic Kwiatkowski, Cordelia Langford, John Sillitoe on behalf of the Wellcome Sanger Institute COVID-19 Surveillance Team
EPI_ISL_703539	Lighthouse Lab in Alderley Park	Wellcome Sanger Institute for the COVID-19 Genomics UK (COG-UK) Consortium	Jacquelyn Wynn, Mairead Hyland, The Lighthouse Lab in Alderley Park and Alex Alderton, Roberto Amato, Sonia Goncalves, Ewan Harrison, David K. Jackson, Ian Johnston, Dominic Kwiatkowski, Cordelia Langford, John Sillitoe on behalf of the Wellcome Sanger Institute COVID-19 Surveillance Team
EPI_ISL_703541, EPI_ISL_703546	Lighthouse Lab in Glasgow	Wellcome Sanger Institute for the COVID-19 Genomics UK (COG-UK) Consortium	Harper VanSteenhouse, Yumi Kasai, David Gray, Carol Clugston, Anna Dominiczak and Alex Alderton, Roberto Amato, Sonia Goncalves, Ewan Harrison, David K. Jackson, Ian Johnston, Dominic Kwiatkowski, Cordelia Langford, John Sillitoe on behalf of the Wellcome Sanger Institute COVID-19 Surveillance Team
EPI_ISL_703547, EPI_ISL_703549, EPI_ISL_703552	Lighthouse Lab in Alderley Park	Wellcome Sanger Institute for the COVID-19 Genomics UK (COG-UK) Consortium	Jacquelyn Wynn, Mairead Hyland, The Lighthouse Lab in Alderley Park and Alex Alderton, Roberto Amato, Sonia Goncalves, Ewan Harrison, David K. Jackson, Ian Johnston, Dominic Kwiatkowski, Cordelia Langford, John Sillitoe on behalf of the Wellcome Sanger Institute COVID-19 Surveillance Team
EPI_ISL_703553, EPI_ISL_703557	Lighthouse Lab in Glasgow	Wellcome Sanger Institute for the COVID-19 Genomics UK (COG-UK) Consortium	Harper VanSteenhouse, Yumi Kasai, David Gray, Carol Clugston, Anna Dominiczak and Alex Alderton, Roberto Amato, Sonia Goncalves, Ewan Harrison, David K. Jackson, Ian Johnston, Dominic Kwiatkowski, Cordelia Langford, John Sillitoe on behalf of the Wellcome Sanger Institute COVID-19 Surveillance Team
EPI_ISL_703558	Lighthouse Lab in Alderley Park	Wellcome Sanger Institute for the COVID-19 Genomics UK (COG-UK) Consortium	Jacquelyn Wynn, Mairead Hyland, The Lighthouse Lab in Alderley Park and Alex Alderton, Roberto Amato, Sonia Goncalves, Ewan Harrison, David K. Jackson, Ian Johnston, Dominic Kwiatkowski, Cordelia Langford, John Sillitoe on behalf of the Wellcome Sanger Institute COVID-19 Surveillance Team
EPI_ISL_703560	Lighthouse Lab in Glasgow	Wellcome Sanger Institute for the COVID-19 Genomics UK (COG-UK) Consortium	Harper VanSteenhouse, Yumi Kasai, David Gray, Carol Clugston, Anna Dominiczak and Alex Alderton, Roberto Amato, Sonia Goncalves, Ewan Harrison, David K. Jackson, Ian Johnston, Dominic Kwiatkowski, Cordelia Langford, John Sillitoe on behalf of the Wellcome Sanger Institute COVID-19 Surveillance Team
EPI_ISL_703563, EPI_ISL_703566, EPI_ISL_703568, EPI_ISL_703571, EPI_ISL_703573, EPI_ISL_703576, EPI_ISL_703581	Lighthouse Lab in Alderley Park	Wellcome Sanger Institute for the COVID-19 Genomics UK (COG-UK) Consortium	Jacquelyn Wynn, Mairead Hyland, The Lighthouse Lab in Alderley Park and Alex Alderton, Roberto Amato, Sonia Goncalves, Ewan Harrison, David K. Jackson, Ian Johnston, Dominic Kwiatkowski, Cordelia Langford, John Sillitoe on behalf of the Wellcome Sanger Institute COVID-19 Surveillance Team
EPI_ISL_703583	Lighthouse Lab in Glasgow	Wellcome Sanger Institute for the COVID-19 Genomics UK (COG-UK) Consortium	Harper VanSteenhouse, Yumi Kasai, David Gray, Carol Clugston, Anna Dominiczak and Alex Alderton, Roberto Amato, Sonia Goncalves, Ewan Harrison, David K. Jackson, Ian Johnston, Dominic Kwiatkowski, Cordelia Langford, John Sillitoe on behalf of the Wellcome Sanger Institute COVID-19 Surveillance Team
EPI_ISL_703586, EPI_ISL_703591, EPI_ISL_703595, EPI_ISL_703602, EPI_ISL_703605, EPI_ISL_703618, EPI_ISL_703620, EPI_ISL_703630	Lighthouse Lab in Alderley Park	Wellcome Sanger Institute for the COVID-19 Genomics UK (COG-UK) Consortium	Jacquelyn Wynn, Mairead Hyland, The Lighthouse Lab in Alderley Park and Alex Alderton, Roberto Amato, Sonia Goncalves, Ewan Harrison, David K. Jackson, Ian Johnston, Dominic Kwiatkowski, Cordelia Langford, John Sillitoe on behalf of the Wellcome Sanger Institute COVID-19 Surveillance Team
EPI_ISL_703633, EPI_ISL_703634, EPI_ISL_703640, EPI_ISL_703641	Lighthouse Lab in Glasgow	Wellcome Sanger Institute for the COVID-19 Genomics UK (COG-UK) Consortium	Harper VanSteenhouse, Yumi Kasai, David Gray, Carol Clugston, Anna Dominiczak and Alex Alderton, Roberto Amato, Sonia Goncalves, Ewan Harrison, David K. Jackson, Ian Johnston, Dominic Kwiatkowski, Cordelia Langford, John Sillitoe on behalf of the Wellcome Sanger Institute COVID-19 Surveillance Team
EPI_ISL_703646, EPI_ISL_703648	Lighthouse Lab in Alderley Park	Wellcome Sanger Institute for the COVID-19 Genomics UK (COG-UK) Consortium	Jacquelyn Wynn, Mairead Hyland, The Lighthouse Lab in Alderley Park and Alex Alderton, Roberto Amato, Sonia Goncalves, Ewan Harrison, David K. Jackson, Ian Johnston, Dominic Kwiatkowski, Cordelia Langford, John Sillitoe on behalf of the Wellcome Sanger Institute COVID-19 Surveillance Team
EPI_ISL_703650, EPI_ISL_703651, EPI_ISL_703657, EPI_ISL_703659	Lighthouse Lab in Glasgow	Wellcome Sanger Institute for the COVID-19 Genomics UK (COG-UK) Consortium	Harper VanSteenhouse, Yumi Kasai, David Gray, Carol Clugston, Anna Dominiczak and Alex Alderton, Roberto Amato, Sonia Goncalves, Ewan Harrison, David K. Jackson, Ian Johnston, Dominic Kwiatkowski, Cordelia Langford, John Sillitoe on behalf of the Wellcome Sanger Institute COVID-19 Surveillance Team
EPI_ISL_703664, EPI_ISL_703665	Lighthouse Lab in Alderley Park	Wellcome Sanger Institute for the COVID-19 Genomics UK (COG-UK) Consortium	Jacquelyn Wynn, Mairead Hyland, The Lighthouse Lab in Alderley Park and Alex Alderton, Roberto Amato, Sonia Goncalves, Ewan Harrison, David K. Jackson, Ian Johnston, Dominic Kwiatkowski, Cordelia Langford, John Sillitoe on behalf of the Wellcome Sanger Institute COVID-19 Surveillance Team
EPI_ISL_703672	Lighthouse Lab in Glasgow	Wellcome Sanger Institute for the COVID-19 Genomics UK (COG-UK) Consortium	Harper VanSteenhouse, Yumi Kasai, David Gray, Carol Clugston, Anna Dominiczak and Alex Alderton, Roberto Amato, Sonia Goncalves, Ewan Harrison, David K. Jackson, Ian Johnston, Dominic Kwiatkowski, Cordelia Langford, John Sillitoe on behalf of the Wellcome Sanger Institute COVID-19 Surveillance Team
EPI_ISL_703675	Lighthouse Lab in Alderley Park	Wellcome Sanger Institute for the COVID-19 Genomics UK (COG-UK) Consortium	Jacquelyn Wynn, Mairead Hyland, The Lighthouse Lab in Alderley Park and Alex Alderton, Roberto Amato, Sonia Goncalves, Ewan Harrison, David K. Jackson, Ian Johnston, Dominic Kwiatkowski, Cordelia Langford, John Sillitoe on behalf of the Wellcome Sanger Institute COVID-19 Surveillance Team
EPI_ISL_703677	Lighthouse Lab in Glasgow	Wellcome Sanger Institute for the COVID-19 Genomics UK (COG-UK) Consortium	Harper VanSteenhouse, Yumi Kasai, David Gray, Carol Clugston, Anna Dominiczak and Alex Alderton, Roberto Amato, Sonia Goncalves, Ewan Harrison, David K. Jackson, Ian Johnston, Dominic Kwiatkowski, Cordelia Langford, John Sillitoe on behalf of the Wellcome Sanger Institute COVID-19 Surveillance Team
EPI_ISL_703681	Lighthouse Lab in Alderley Park	Wellcome Sanger Institute for the COVID-19 Genomics UK (COG-UK) Consortium	Jacquelyn Wynn, Mairead Hyland, The Lighthouse Lab in Alderley Park and Alex Alderton, Roberto Amato, Sonia Goncalves, Ewan Harrison, David K. Jackson, Ian Johnston, Dominic Kwiatkowski, Cordelia Langford, John Sillitoe on behalf of the Wellcome Sanger Institute COVID-19 Surveillance Team
EPI_ISL_703685	Lighthouse Lab in Glasgow	Wellcome Sanger Institute for the COVID-19 Genomics UK (COG-UK) Consortium	Harper VanSteenhouse, Yumi Kasai, David Gray, Carol Clugston, Anna Dominiczak and Alex Alderton, Roberto Amato, Sonia Goncalves, Ewan Harrison, David K. Jackson, Ian Johnston, Dominic Kwiatkowski, Cordelia Langford, John Sillitoe on behalf of the Wellcome Sanger Institute COVID-19 Surveillance Team
EPI_ISL_703686, EPI_ISL_703688, EPI_ISL_703691, EPI_ISL_703692, EPI_ISL_703696	Lighthouse Lab in Alderley Park	Wellcome Sanger Institute for the COVID-19 Genomics UK (COG-UK) Consortium	Jacquelyn Wynn, Mairead Hyland, The Lighthouse Lab in Alderley Park and Alex Alderton, Roberto Amato, Sonia Goncalves, Ewan Harrison, David K. Jackson, Ian Johnston, Dominic Kwiatkowski, Cordelia Langford, John Sillitoe on behalf of the Wellcome Sanger Institute COVID-19 Surveillance Team
EPI_ISL_703697	Lighthouse Lab in Glasgow	Wellcome Sanger Institute for the COVID-19 Genomics UK (COG-UK) Consortium	Harper VanSteenhouse, Yumi Kasai, David Gray, Carol Clugston, Anna Dominiczak and Alex Alderton, Roberto Amato, Sonia Goncalves, Ewan Harrison, David K. Jackson, Ian Johnston, Dominic Kwiatkowski, Cordelia Langford, John Sillitoe on behalf of the Wellcome Sanger Institute COVID-19 Surveillance Team
EPI_ISL_703699, EPI_ISL_703702	Lighthouse Lab in Alderley Park	Wellcome Sanger Institute for the COVID-19 Genomics UK (COG-UK) Consortium	Jacquelyn Wynn, Mairead Hyland, The Lighthouse Lab in Alderley Park and Alex Alderton, Roberto Amato, Sonia Goncalves, Ewan Harrison, David K. Jackson, Ian Johnston, Dominic Kwiatkowski, Cordelia Langford, John Sillitoe on behalf of the Wellcome Sanger Institute COVID-19 Surveillance Team
EPI_ISL_703703	Lighthouse Lab in Glasgow	Wellcome Sanger Institute for the COVID-19 Genomics UK (COG-UK) Consortium	Harper VanSteenhouse, Yumi Kasai, David Gray, Carol Clugston, Anna Dominiczak and Alex Alderton, Roberto Amato, Sonia Goncalves, Ewan Harrison, David K. Jackson, Ian Johnston, Dominic Kwiatkowski, Cordelia Langford, John Sillitoe on behalf of the Wellcome Sanger Institute COVID-19 Surveillance

			Team
EPI_ISL_703705, EPI_ISL_703777, EPI_ISL_703778, EPI_ISL_703780, EPI_ISL_703783	Lighthouse Lab in Alderley Park	Wellcome Sanger Institute for the COVID-19 Genomics UK (COG-UK) Consortium	Jacquelyn Wynn, Mairead Hyland, The Lighthouse Lab in Alderley Park and Alex Alderton, Roberto Amato, Sonia Goncalves, Ewan Harrison, David K. Jackson, Ian Johnston, Dominic Kwiatkowski, Cordelia Langford, John Sillitoe on behalf of the Wellcome Sanger Institute COVID-19 Surveillance Team
EPI_ISL_703786	Lighthouse Lab in Glasgow	Wellcome Sanger Institute for the COVID-19 Genomics UK (COG-UK) Consortium	Harper VanSteenhouse, Yumi Kasai, David Gray, Carol Clugston, Anna Dominiczak and Alex Alderton, Roberto Amato, Sonia Goncalves, Ewan Harrison, David K. Jackson, Ian Johnston, Dominic Kwiatkowski, Cordelia Langford, John Sillitoe on behalf of the Wellcome Sanger Institute COVID-19 Surveillance Team
EPI_ISL_703788	Lighthouse Lab in Alderley Park	Wellcome Sanger Institute for the COVID-19 Genomics UK (COG-UK) Consortium	Jacquelyn Wynn, Mairead Hyland, The Lighthouse Lab in Alderley Park and Alex Alderton, Roberto Amato, Sonia Goncalves, Ewan Harrison, David K. Jackson, Ian Johnston, Dominic Kwiatkowski, Cordelia Langford, John Sillitoe on behalf of the Wellcome Sanger Institute COVID-19 Surveillance Team
EPI_ISL_703790	Lighthouse Lab in Glasgow	Wellcome Sanger Institute for the COVID-19 Genomics UK (COG-UK) Consortium	Harper VanSteenhouse, Yumi Kasai, David Gray, Carol Clugston, Anna Dominiczak and Alex Alderton, Roberto Amato, Sonia Goncalves, Ewan Harrison, David K. Jackson, Ian Johnston, Dominic Kwiatkowski, Cordelia Langford, John Sillitoe on behalf of the Wellcome Sanger Institute COVID-19 Surveillance Team
EPI_ISL_703791, EPI_ISL_703793, EPI_ISL_703795, EPI_ISL_703796, EPI_ISL_703798	Lighthouse Lab in Alderley Park	Wellcome Sanger Institute for the COVID-19 Genomics UK (COG-UK) Consortium	Jacquelyn Wynn, Mairead Hyland, The Lighthouse Lab in Alderley Park and Alex Alderton, Roberto Amato, Sonia Goncalves, Ewan Harrison, David K. Jackson, Ian Johnston, Dominic Kwiatkowski, Cordelia Langford, John Sillitoe on behalf of the Wellcome Sanger Institute COVID-19 Surveillance Team
EPI_ISL_703801	Lighthouse Lab in Glasgow	Wellcome Sanger Institute for the COVID-19 Genomics UK (COG-UK) Consortium	Harper VanSteenhouse, Yumi Kasai, David Gray, Carol Clugston, Anna Dominiczak and Alex Alderton, Roberto Amato, Sonia Goncalves, Ewan Harrison, David K. Jackson, Ian Johnston, Dominic Kwiatkowski, Cordelia Langford, John Sillitoe on behalf of the Wellcome Sanger Institute COVID-19 Surveillance Team
EPI_ISL_703803, EPI_ISL_703804	Lighthouse Lab in Alderley Park	Wellcome Sanger Institute for the COVID-19 Genomics UK (COG-UK) Consortium	Jacquelyn Wynn, Mairead Hyland, The Lighthouse Lab in Alderley Park and Alex Alderton, Roberto Amato, Sonia Goncalves, Ewan Harrison, David K. Jackson, Ian Johnston, Dominic Kwiatkowski, Cordelia Langford, John Sillitoe on behalf of the Wellcome Sanger Institute COVID-19 Surveillance Team
EPI_ISL_703808	Lighthouse Lab in Glasgow	Wellcome Sanger Institute for the COVID-19 Genomics UK (COG-UK) Consortium	Harper VanSteenhouse, Yumi Kasai, David Gray, Carol Clugston, Anna Dominiczak and Alex Alderton, Roberto Amato, Sonia Goncalves, Ewan Harrison, David K. Jackson, Ian Johnston, Dominic Kwiatkowski, Cordelia Langford, John Sillitoe on behalf of the Wellcome Sanger Institute COVID-19 Surveillance Team
EPI_ISL_703809, EPI_ISL_703812, EPI_ISL_703814	Lighthouse Lab in Alderley Park	Wellcome Sanger Institute for the COVID-19 Genomics UK (COG-UK) Consortium	Jacquelyn Wynn, Mairead Hyland, The Lighthouse Lab in Alderley Park and Alex Alderton, Roberto Amato, Sonia Goncalves, Ewan Harrison, David K. Jackson, Ian Johnston, Dominic Kwiatkowski, Cordelia Langford, John Sillitoe on behalf of the Wellcome Sanger Institute COVID-19 Surveillance Team
EPI_ISL_703816, EPI_ISL_703819, EPI_ISL_703820, EPI_ISL_703822, EPI_ISL_703824, EPI_ISL_703825, EPI_ISL_703830, EPI_ISL_703836, EPI_ISL_703840, EPI_ISL_703841, EPI_ISL_703843, EPI_ISL_703851, EPI_ISL_703852, EPI_ISL_703854, EPI_ISL_703861, EPI_ISL_703862, EPI_ISL_703866, EPI_ISL_703869, EPI_ISL_703878, EPI_ISL_703880, EPI_ISL_703882, EPI_ISL_703883, EPI_ISL_703887, EPI_ISL_703889, EPI_ISL_703890, EPI_ISL_703895, EPI_ISL_703900, EPI_ISL_703901, EPI_ISL_703906, EPI_ISL_703909, EPI_ISL_703910, EPI_ISL_703912, EPI_ISL_703917, EPI_ISL_703919, EPI_ISL_703925, EPI_ISL_703928, EPI_ISL_703930, EPI_ISL_703933, EPI_ISL_703934, EPI_ISL_703938, EPI_ISL_703943, EPI_ISL_703944, EPI_ISL_703949, EPI_ISL_703951, EPI_ISL_703956, EPI_ISL_703962, EPI_ISL_703967, EPI_ISL_703972, EPI_ISL_703976, EPI_ISL_703980, EPI_ISL_703981, EPI_ISL_703983, EPI_ISL_703984, EPI_ISL_703986, EPI_ISL_703988, EPI_ISL_703989, EPI_ISL_703991, EPI_ISL_703994, EPI_ISL_703997, EPI_ISL_703999, EPI_ISL_704000, EPI_ISL_704002, EPI_ISL_704004, EPI_ISL_704005, EPI_ISL_704007, EPI_ISL_704008, EPI_ISL_704010, EPI_ISL_704013, EPI_ISL_704016, EPI_ISL_704018, EPI_ISL_704023, EPI_ISL_704024, EPI_ISL_704026, EPI_ISL_704029, EPI_ISL_704031, EPI_ISL_704034, EPI_ISL_704035, EPI_ISL_704037, EPI_ISL_704040, EPI_ISL_704042, EPI_ISL_704045, EPI_ISL_704048, EPI_ISL_704051, EPI_ISL_704053, EPI_ISL_704055, EPI_ISL_704056, EPI_ISL_704058, EPI_ISL_704059, EPI_ISL_704063, EPI_ISL_704064, EPI_ISL_704066, EPI_ISL_704071, EPI_ISL_704072, EPI_ISL_704077, EPI_ISL_704078, EPI_ISL_704080, EPI_ISL_704082, EPI_ISL_704083, EPI_ISL_704085, EPI_ISL_704088, EPI_ISL_704090, EPI_ISL_704091, EPI_ISL_704093, EPI_ISL_704094, EPI_ISL_704096, EPI_ISL_704099, EPI_ISL_704104, EPI_ISL_704106, EPI_ISL_704107, EPI_ISL_704109, EPI_ISL_704110, EPI_ISL_704114, EPI_ISL_704116, EPI_ISL_704121, EPI_ISL_704122, EPI_ISL_704124, EPI_ISL_704125, EPI_ISL_704130, EPI_ISL_704133, EPI_ISL_704136, EPI_ISL_704140, EPI_ISL_704143, EPI_ISL_704144, EPI_ISL_704146, EPI_ISL_704152, EPI_ISL_704154, EPI_ISL_704157, EPI_ISL_704162, EPI_ISL_704164, EPI_ISL_704165, EPI_ISL_704169, EPI_ISL_704170, EPI_ISL_704172, EPI_ISL_704173, EPI_ISL_704175, EPI_ISL_704182, EPI_ISL_704183, EPI_ISL_704185, EPI_ISL_704186, EPI_ISL_704189, EPI_ISL_704192, EPI_ISL_704197, EPI_ISL_704200, EPI_ISL_704202, EPI_ISL_704204, EPI_ISL_704205, EPI_ISL_704208, EPI_ISL_704211, EPI_ISL_704213, EPI_ISL_704214, EPI_ISL_704215, EPI_ISL_704220, EPI_ISL_704221, EPI_ISL_704224, EPI_ISL_704226, EPI_ISL_704227, EPI_ISL_704229, EPI_ISL_704230, EPI_ISL_704234, EPI_ISL_704235, EPI_ISL_704237, EPI_ISL_704240, EPI_ISL_704242, EPI_ISL_704245, EPI_ISL_704246, EPI_ISL_704248, EPI_ISL_704251, EPI_ISL_704253, EPI_ISL_704254, EPI_ISL_704258, EPI_ISL_704261, EPI_ISL_704263, EPI_ISL_704264, EPI_ISL_704268, EPI_ISL_704274, EPI_ISL_704275, EPI_ISL_704278, EPI_ISL_704281, EPI_ISL_704282, EPI_ISL_704284, EPI_ISL_704287, EPI_ISL_704288, EPI_ISL_704290, EPI_ISL_704292, EPI_ISL_704293, EPI_ISL_704295, EPI_ISL_704296, EPI_ISL_704298, EPI_ISL_704300, EPI_ISL_704303, EPI_ISL_704306, EPI_ISL_704313, EPI_ISL_704314, EPI_ISL_704316, EPI_ISL_704318, EPI_ISL_704319, EPI_ISL_704323, EPI_ISL_704326, EPI_ISL_704327, EPI_ISL_704330, EPI_ISL_704332, EPI_ISL_704335, EPI_ISL_704337, EPI_ISL_704338, EPI_ISL_704342, EPI_ISL_704343, EPI_ISL_704345, EPI_ISL_704346, EPI_ISL_704348, EPI_ISL_704349, EPI_ISL_704351, EPI_ISL_704352, EPI_ISL_704357, EPI_ISL_704358, EPI_ISL_704360, EPI_ISL_704363, EPI_ISL_704368, EPI_ISL_704370, EPI_ISL_704376, EPI_ISL_704380, EPI_ISL_704381, EPI_ISL_704383, EPI_ISL_704388, EPI_ISL_704391, EPI_ISL_704394, EPI_ISL_704396, EPI_ISL_704400, EPI_ISL_704402, EPI_ISL_704404, EPI_ISL_704405, EPI_ISL_704407, EPI_ISL_704412, EPI_ISL_704415, EPI_ISL_704421, EPI_ISL_704423, EPI_ISL_704426, EPI_ISL_704428, EPI_ISL_704429, EPI_ISL_704431, EPI_ISL_704434, EPI_ISL_704442, EPI_ISL_704444, EPI_ISL_704445, EPI_ISL_704447, EPI_ISL_704450, EPI_ISL_704451, EPI_ISL_704453, EPI_ISL_704455, EPI_ISL_704456, EPI_ISL_704463, EPI_ISL_704468, EPI_ISL_704469, EPI_ISL_704471, EPI_ISL_704476, EPI_ISL_704477, EPI_ISL_704479, EPI_ISL_704485, EPI_ISL_704487, EPI_ISL_704493, EPI_ISL_704496, EPI_ISL_704498, EPI_ISL_704500, EPI_ISL_704501, EPI_ISL_704504, EPI_ISL_704506, EPI_ISL_704514, EPI_ISL_704523, EPI_ISL_704525, EPI_ISL_704530, EPI_ISL_704531, EPI_ISL_704536, EPI_ISL_704539, EPI_ISL_704541, EPI_ISL_704547, EPI_ISL_704549, EPI_ISL_704550, EPI_ISL_704552, EPI_ISL_704556, EPI_ISL_704558, EPI_ISL_704560, EPI_ISL_704561, EPI_ISL_704566, EPI_ISL_704568, EPI_ISL_704569, EPI_ISL_704571			
see above	Lighthouse Lab in Glasgow	Wellcome Sanger Institute for the COVID-19 Genomics UK (COG-UK) Consortium	Harper VanSteenhouse, Yumi Kasai, David Gray, Carol Clugston, Anna Dominiczak and Alex Alderton, Roberto Amato, Sonia Goncalves, Ewan Harrison, David K. Jackson, Ian Johnston, Dominic Kwiatkowski, Cordelia Langford, John Sillitoe on behalf of the Wellcome Sanger Institute COVID-19 Surveillance Team
EPI_ISL_704606	Department of Pathology, University of Cambridge	COVID-19 Genomics UK (COG-UK) Consortium	Aminu S. Jahun, Yasmin Chaudhry, Grant Hall, Iliana Georgana, Myra Hosmillo, Martin D. Curran, Malte Pinckert, Surendra Parmar, Ian Goodfellow
EPI_ISL_704607	University College London Hospital	COVID-19 Genomics UK (COG-UK) Consortium	Judith Heaney, Matthew Byott, Catherine Houlihan, Dan Frampton, Stuart Kirk, Moira Spyer and Eleni Nastouli
EPI_ISL_704611, EPI_ISL_704613, EPI_ISL_704616, EPI_ISL_704618	Department of Pathology, University of Cambridge	COVID-19 Genomics UK (COG-UK) Consortium	Aminu S. Jahun, Yasmin Chaudhry, Grant Hall, Iliana Georgana, Myra Hosmillo, Martin D. Curran, Malte Pinckert, Surendra Parmar, Ian Goodfellow
EPI_ISL_704638	Virology Department, Sheffield Teaching Hospitals NHS Foundation Trust/Department of Infection, Immunity and Cardiovascular Disease, The Medical School, University of Sheffield	COVID-19 Genomics UK (COG-UK) Consortium	Thushan de Silva, Matthew Parker, Nikki Smith, Adri Angyal, Rebecca Brown, Luke Green, Rachel Tucker, Paul Parsons, Danielle Groves, Katie Johnson, Laura Carrilero, Alex Keeley, Dave Partridge, Matthew Wyles, Benjamin Lindsey, Mehmet Yavuz, Mohammad Raza, Cariad Evans
EPI_ISL_704642, EPI_ISL_704644, EPI_ISL_704645	Lighthouse Lab in Glasgow	Wellcome Sanger Institute for the COVID-19 Genomics UK (COG-UK) Consortium	Harper VanSteenhouse, Yumi Kasai, David Gray, Carol Clugston, Anna Dominiczak and Alex Alderton, Roberto Amato, Sonia Goncalves, Ewan Harrison, David K. Jackson, Ian Johnston, Dominic Kwiatkowski, Cordelia Langford, John Sillitoe on behalf of the Wellcome Sanger Institute COVID-19 Surveillance Team
EPI_ISL_704648	Department of Pathology, University of Cambridge	COVID-19 Genomics UK (COG-UK) Consortium	Aminu S. Jahun, Yasmin Chaudhry, Grant Hall, Iliana Georgana, Myra Hosmillo, Martin D. Curran, Malte Pinckert, Surendra Parmar, Ian Goodfellow
EPI_ISL_704649, EPI_ISL_704652, EPI_ISL_704655	Lighthouse Lab in Glasgow	Wellcome Sanger Institute for the COVID-19 Genomics UK (COG-UK) Consortium	Harper VanSteenhouse, Yumi Kasai, David Gray, Carol Clugston, Anna Dominiczak and Alex Alderton, Roberto Amato, Sonia Goncalves, Ewan Harrison, David K. Jackson, Ian Johnston, Dominic Kwiatkowski, Cordelia Langford, John Sillitoe on behalf of the Wellcome Sanger Institute COVID-19 Surveillance Team
EPI_ISL_704656	Department of Pathology, University of Cambridge	COVID-19 Genomics UK (COG-UK) Consortium	Aminu S. Jahun, Yasmin Chaudhry, Grant Hall, Iliana Georgana, Myra Hosmillo, Martin D. Curran, Malte Pinckert, Surendra Parmar, Ian Goodfellow
EPI_ISL_704657, EPI_ISL_704658	Lighthouse Lab in Glasgow	Wellcome Sanger Institute for the COVID-19 Genomics UK (COG-UK) Consortium	Harper VanSteenhouse, Yumi Kasai, David Gray, Carol Clugston, Anna Dominiczak and Alex Alderton, Roberto Amato, Sonia Goncalves, Ewan Harrison, David K. Jackson, Ian Johnston, Dominic Kwiatkowski, Cordelia Langford, John Sillitoe on behalf of the Wellcome Sanger Institute COVID-19 Surveillance Team
EPI_ISL_704662	Department of Pathology, University of Cambridge	COVID-19 Genomics UK (COG-UK) Consortium	Aminu S. Jahun, Yasmin Chaudhry, Grant Hall, Iliana Georgana, Myra Hosmillo, Martin D. Curran, Malte Pinckert, Surendra Parmar, Ian Goodfellow
EPI_ISL_704663	Lighthouse Lab in Glasgow	Wellcome Sanger Institute for the COVID-19 Genomics UK (COG-UK) Consortium	Harper VanSteenhouse, Yumi Kasai, David Gray, Carol Clugston, Anna Dominiczak and Alex Alderton, Roberto Amato, Sonia Goncalves, Ewan Harrison, David K. Jackson, Ian Johnston, Dominic Kwiatkowski, Cordelia Langford, John Sillitoe on behalf of the Wellcome Sanger Institute COVID-19 Surveillance Team
EPI_ISL_704664	Department of Pathology, University of Cambridge	COVID-19 Genomics UK (COG-UK) Consortium	Aminu S. Jahun, Yasmin Chaudhry, Grant Hall, Iliana Georgana, Myra Hosmillo, Martin D. Curran, Malte Pinckert, Surendra Parmar, Ian Goodfellow
EPI_ISL_704665	Lighthouse Lab in Glasgow	Wellcome Sanger Institute for the COVID-19 Genomics UK (COG-UK) Consortium	Harper VanSteenhouse, Yumi Kasai, David Gray, Carol Clugston, Anna Dominiczak and Alex Alderton, Roberto Amato, Sonia Goncalves, Ewan Harrison, David K. Jackson, Ian Johnston, Dominic Kwiatkowski, Cordelia Langford, John Sillitoe on behalf of the Wellcome Sanger Institute COVID-19 Surveillance Team
EPI_ISL_704666	University College London Hospital	COVID-19 Genomics UK (COG-UK) Consortium	Judith Heaney, Matthew Byott, Catherine Houlihan, Dan Frampton, Stuart Kirk, Moira Spyer and Eleni Nastouli
EPI_ISL_704667, EPI_ISL_704670, EPI_ISL_704675, EPI_ISL_704680,	Lighthouse Lab in Glasgow	Wellcome Sanger Institute for the COVID-19 Genomics UK (COG-UK) Consortium	Harper VanSteenhouse, Yumi Kasai, David Gray, Carol Clugston, Anna Dominiczak and Alex Alderton, Roberto Amato, Sonia Goncalves, Ewan Harrison, David K. Jackson, Ian Johnston, Dominic Kwiatkowski, Cordelia Langford, John Sillitoe on behalf of the Wellcome Sanger Institute COVID-19 Surveillance

EPI_ISL_704681, EPI_ISL_704683, EPI_ISL_704684, EPI_ISL_704686			Team
EPI_ISL_704688, EPI_ISL_704691, EPI_ISL_704693, EPI_ISL_704696, EPI_ISL_704697, EPI_ISL_704699, EPI_ISL_704702, EPI_ISL_704705, EPI_ISL_704707, EPI_ISL_704708, EPI_ISL_704713			
see above	Lighthouse Lab in Cambridge	Wellcome Sanger Institute for the COVID-19 Genomics UK (COG-UK) Consortium	Rob Howes, The Lighthouse Lab in Cambridge and Alex Alderton, Roberto Amato, Sonia Goncalves, Ewan Harrison, David K. Jackson, Ian Johnston, Dominic Kwiatkowski, Cordelia Langford, John Sillitoe on behalf of the Wellcome Sanger Institute COVID-19 Surveillance Team
EPI_ISL_704714	Quadram Institute Bioscience	COVID-19 Genomics UK (COG-UK) Consortium	Dave J. Baker, Gemma L. Kay, Alp Aydin, Thanh Le-Viet, Steven Rudder, Ana P. Tedim, Anastasia Kolyva, Maria Diaz, Leonardo de Oliveira Martins, Nabil-Fareed Alikhan, Lizzie Meadows, Rachael Stanley, Ngozi Elumogo, Muhammed Yasir, Nicholas M. Thomson, Alexander J Trotter, Rachel Gilroy, Samuel Bloomfield, Claire Stuart, Andrew Bell, Reenesh Prakash, Samir Dervisevic, Alison E. Mather, John Wain, Mark Webber, Andrew J. Page, Justin O'Grady
EPI_ISL_704715, EPI_ISL_704724, EPI_ISL_704726, EPI_ISL_704727, EPI_ISL_704730, EPI_ISL_704732, EPI_ISL_704735, EPI_ISL_704738, EPI_ISL_704740, EPI_ISL_704743, EPI_ISL_704745, EPI_ISL_704753, EPI_ISL_704763, EPI_ISL_704766, EPI_ISL_704767, EPI_ISL_704769, EPI_ISL_704771, EPI_ISL_704773, EPI_ISL_704774, EPI_ISL_704776, EPI_ISL_704779, EPI_ISL_704781, EPI_ISL_704782, EPI_ISL_704784, EPI_ISL_704785, EPI_ISL_704790, EPI_ISL_704792, EPI_ISL_704795, EPI_ISL_704796, EPI_ISL_704801, EPI_ISL_704803, EPI_ISL_704805, EPI_ISL_704806, EPI_ISL_704808, EPI_ISL_704811, EPI_ISL_704813, EPI_ISL_704814, EPI_ISL_704816, EPI_ISL_704818, EPI_ISL_704819, EPI_ISL_704821, EPI_ISL_704822, EPI_ISL_704824, EPI_ISL_704827, EPI_ISL_704830, EPI_ISL_704832, EPI_ISL_704833			
see above	Lighthouse Lab in Cambridge	Wellcome Sanger Institute for the COVID-19 Genomics UK (COG-UK) Consortium	Rob Howes, The Lighthouse Lab in Cambridge and Alex Alderton, Roberto Amato, Sonia Goncalves, Ewan Harrison, David K. Jackson, Ian Johnston, Dominic Kwiatkowski, Cordelia Langford, John Sillitoe on behalf of the Wellcome Sanger Institute COVID-19 Surveillance Team
EPI_ISL_704834	Department of Pathology, University of Cambridge	COVID-19 Genomics UK (COG-UK) Consortium	Aminu S. Jahun, Yasmin Chaudhry, Grant Hall, Iliana Georgana, Myra Hosmillo, Martin D. Curran, Malte Pinckert, Surendra Parmar, Ian Goodfellow
EPI_ISL_704835, EPI_ISL_704840, EPI_ISL_704841, EPI_ISL_704843, EPI_ISL_704844, EPI_ISL_704849	Lighthouse Lab in Cambridge	Wellcome Sanger Institute for the COVID-19 Genomics UK (COG-UK) Consortium	Rob Howes, The Lighthouse Lab in Cambridge and Alex Alderton, Roberto Amato, Sonia Goncalves, Ewan Harrison, David K. Jackson, Ian Johnston, Dominic Kwiatkowski, Cordelia Langford, John Sillitoe on behalf of the Wellcome Sanger Institute COVID-19 Surveillance Team
EPI_ISL_704850	Quadram Institute Bioscience	COVID-19 Genomics UK (COG-UK) Consortium	Dave J. Baker, Gemma L. Kay, Alp Aydin, Thanh Le-Viet, Steven Rudder, Ana P. Tedim, Anastasia Kolyva, Maria Diaz, Leonardo de Oliveira Martins, Nabil-Fareed Alikhan, Lizzie Meadows, Rachael Stanley, Ngozi Elumogo, Muhammed Yasir, Nicholas M. Thomson, Alexander J Trotter, Rachel Gilroy, Samuel Bloomfield, Claire Stuart, Andrew Bell, Reenesh Prakash, Samir Dervisevic, Alison E. Mather, John Wain, Mark Webber, Andrew J. Page, Justin O'Grady
EPI_ISL_704851, EPI_ISL_704856, EPI_ISL_704857, EPI_ISL_704859, EPI_ISL_704860, EPI_ISL_704862, EPI_ISL_704865, EPI_ISL_704867, EPI_ISL_704876, EPI_ISL_704880, EPI_ISL_704881, EPI_ISL_704884, EPI_ISL_704886, EPI_ISL_704888, EPI_ISL_704889, EPI_ISL_704893, EPI_ISL_704894, EPI_ISL_704898, EPI_ISL_704899, EPI_ISL_704902, EPI_ISL_704904			
see above	Lighthouse Lab in Cambridge	Wellcome Sanger Institute for the COVID-19 Genomics UK (COG-UK) Consortium	Rob Howes, The Lighthouse Lab in Cambridge and Alex Alderton, Roberto Amato, Sonia Goncalves, Ewan Harrison, David K. Jackson, Ian Johnston, Dominic Kwiatkowski, Cordelia Langford, John Sillitoe on behalf of the Wellcome Sanger Institute COVID-19 Surveillance Team
EPI_ISL_704905	Liverpool Clinical Laboratories	COVID-19 Genomics UK (COG-UK) Consortium	Sam Haldenby, Anita Lucaci, Steve Paterson, Julian Hiscox, Alistair Darby, M Almsaud, A Alrezaihi, Muhannad Alruwaili, Stuart D Armstrong, Jones Benjamin, Eleanor G Bentley, Anu Chawla, Jordan J Clark, Angela Cowell, Richard Eccles, Isabel García-Dorival, Matthew Gemmell, Alessandro Gerada, PKF Gilmore, Richard Gregory, Ximeng Han, Catherine Hartley, Margaret Hughes, Miren Iturriza-Gomara, James Johnson, L Luu, Jenifer Manson, Charlotte Nelson, Elaine O'Toole, Cassie Olateju, Rebekah Penrice-Randal , Lucille Rainbow, N.P Randle, Trevor Ian Robinson, Parul Sharma, Ghada T Shawli, James P Stewart, Neil Swainston, Ecaterina Vamos, Joanne Watts, Mark Whitehead
EPI_ISL_704906, EPI_ISL_704907, EPI_ISL_704912, EPI_ISL_704913	Lighthouse Lab in Cambridge	Wellcome Sanger Institute for the COVID-19 Genomics UK (COG-UK) Consortium	Rob Howes, The Lighthouse Lab in Cambridge and Alex Alderton, Roberto Amato, Sonia Goncalves, Ewan Harrison, David K. Jackson, Ian Johnston, Dominic Kwiatkowski, Cordelia Langford, John Sillitoe on behalf of the Wellcome Sanger Institute COVID-19 Surveillance Team
EPI_ISL_704914	Quadram Institute Bioscience	COVID-19 Genomics UK (COG-UK) Consortium	Dave J. Baker, Gemma L. Kay, Alp Aydin, Thanh Le-Viet, Steven Rudder, Ana P. Tedim, Anastasia Kolyva, Maria Diaz, Leonardo de Oliveira Martins, Nabil-Fareed Alikhan, Lizzie Meadows, Rachael Stanley, Ngozi Elumogo, Muhammed Yasir, Nicholas M. Thomson, Alexander J Trotter, Rachel Gilroy, Samuel Bloomfield, Claire Stuart, Andrew Bell, Reenesh Prakash, Samir Dervisevic, Alison E. Mather, John Wain, Mark Webber, Andrew J. Page, Justin O'Grady
EPI_ISL_704919, EPI_ISL_704920, EPI_ISL_704922, EPI_ISL_704924, EPI_ISL_704925, EPI_ISL_704927, EPI_ISL_704932, EPI_ISL_704933, EPI_ISL_704936, EPI_ISL_704940, EPI_ISL_704941, EPI_ISL_704943, EPI_ISL_704949, EPI_ISL_704953, EPI_ISL_704956, EPI_ISL_704958, EPI_ISL_704959, EPI_ISL_704961, EPI_ISL_704962, EPI_ISL_704964, EPI_ISL_704967, EPI_ISL_704969, EPI_ISL_704971, EPI_ISL_704975, EPI_ISL_704979, EPI_ISL_704980, EPI_ISL_704985, EPI_ISL_704987, EPI_ISL_704988, EPI_ISL_704990, EPI_ISL_704992, EPI_ISL_704993, EPI_ISL_704995, EPI_ISL_704996, EPI_ISL_704998, EPI_ISL_705000, EPI_ISL_705003, EPI_ISL_705006, EPI_ISL_705009, EPI_ISL_705011, EPI_ISL_705012, EPI_ISL_705016, EPI_ISL_705017, EPI_ISL_705019, EPI_ISL_705020, EPI_ISL_705022, EPI_ISL_705025, EPI_ISL_705027, EPI_ISL_705028, EPI_ISL_705029, EPI_ISL_705031, EPI_ISL_705033, EPI_ISL_705034, EPI_ISL_705036, EPI_ISL_705037, EPI_ISL_705039, EPI_ISL_705041, EPI_ISL_705042, EPI_ISL_705044, EPI_ISL_705045, EPI_ISL_705047, EPI_ISL_705049, EPI_ISL_705050, EPI_ISL_705052, EPI_ISL_705053, EPI_ISL_705055, EPI_ISL_705057, EPI_ISL_705058, EPI_ISL_705060, EPI_ISL_705063, EPI_ISL_705065, EPI_ISL_705066, EPI_ISL_705068, EPI_ISL_705069, EPI_ISL_705071, EPI_ISL_705073, EPI_ISL_705076, EPI_ISL_705079, EPI_ISL_705080, EPI_ISL_705082, EPI_ISL_705085, EPI_ISL_705087, EPI_ISL_705090, EPI_ISL_705092, EPI_ISL_705093, EPI_ISL_705095, EPI_ISL_705097, EPI_ISL_705100, EPI_ISL_705103, EPI_ISL_705107, EPI_ISL_705109, EPI_ISL_705111, EPI_ISL_705112, EPI_ISL_705114, EPI_ISL_705117, EPI_ISL_705122, EPI_ISL_705123, EPI_ISL_705127, EPI_ISL_705128, EPI_ISL_705130, EPI_ISL_705135, EPI_ISL_705136, EPI_ISL_705138, EPI_ISL_705139, EPI_ISL_705141, EPI_ISL_705142, EPI_ISL_705146, EPI_ISL_705147, EPI_ISL_705151, EPI_ISL_705152, EPI_ISL_705157, EPI_ISL_705159, EPI_ISL_705162, EPI_ISL_705164, EPI_ISL_705165, EPI_ISL_705167, EPI_ISL_705173, EPI_ISL_705175, EPI_ISL_705177, EPI_ISL_705178, EPI_ISL_705181, EPI_ISL_705185, EPI_ISL_705186, EPI_ISL_705188			
see above	Lighthouse Lab in Cambridge	Wellcome Sanger Institute for the COVID-19 Genomics UK (COG-UK) Consortium	Rob Howes, The Lighthouse Lab in Cambridge and Alex Alderton, Roberto Amato, Sonia Goncalves, Ewan Harrison, David K. Jackson, Ian Johnston, Dominic Kwiatkowski, Cordelia Langford, John Sillitoe on behalf of the Wellcome Sanger Institute COVID-19 Surveillance Team
EPI_ISL_705191, EPI_ISL_705192, EPI_ISL_705200	Lighthouse Lab in Milton Keynes	Wellcome Sanger Institute for the COVID-19 Genomics UK (COG-UK) Consortium	The Lighthouse Lab in Milton Keynes and Alex Alderton, Roberto Amato, Sonia Goncalves, Ewan Harrison, David K. Jackson, Ian Johnston, Dominic Kwiatkowski, Cordelia Langford, John Sillitoe on behalf of the Wellcome Sanger Institute COVID-19 Surveillance Team
EPI_ISL_705472	University College London Hospital	COVID-19 Genomics UK (COG-UK) Consortium	Judith Heaney, Matthew Byott, Catherine Houlihan, Dan Frampton, Stuart Kirk, Moira Spyer and Eleni Nastouli
EPI_ISL_705486	Northumbria University / South Tees Hospitals NHS Foundation Trust / North Cumbria Integrated Care NHS Foundation Trust / North Tees and Hartlepool NHS Foundation Trust / Newcastle Hospitals NHS Foundation Trust	COVID-19 Genomics UK (COG-UK) Consortium	Darren L Smith,Andrew Nelson,Matthew Bashton,Greg R Young,Joshua Loh,John Allan,Mohammad A Tariq,Giles S Holt,Gary Black,Wen C Yew,Lynn Dover,Paul Baker,Steve Liggett,Sarah Essex,Jane Greenaway,Debra Padgett,Clive Graham,Garren Scott,Edward Barton,Emma Swindells,Brendan Payne,Jennifer Collins,Yusri Taha,Gary Eltringham
EPI_ISL_705536, EPI_ISL_705551, EPI_ISL_705555, EPI_ISL_705557, EPI_ISL_705562	Department of Pathology, University of Cambridge	COVID-19 Genomics UK (COG-UK) Consortium	Aminu S. Jahun, Yasmin Chaudhry, Grant Hall, Iliana Georgana, Myra Hosmillo, Martin D. Curran, Malte Pinckert, Surendra Parmar, Ian Goodfellow
EPI_ISL_705645, EPI_ISL_705652, EPI_ISL_705658, EPI_ISL_705659, EPI_ISL_705663, EPI_ISL_705664, EPI_ISL_705674, EPI_ISL_705684, EPI_ISL_705699, EPI_ISL_705701, EPI_ISL_705702, EPI_ISL_705706, EPI_ISL_705707, EPI_ISL_705708, EPI_ISL_705712, EPI_ISL_705726, EPI_ISL_705730			
see above	West of Scotland Specialist Virology Centre, NHSGGC / MRC-University of Glasgow Centre for Virus Research	COVID-19 Genomics UK (COG-UK) Consortium	Ana da Silva Filipe, Natasha Johnson, Kathy Smollett, Daniel Mair, Stephen Carmichael, Alice Broos, Lily Tong, Jenna Nichols, Kyriaki Nomikou; Sarah McDonald; Richard Orton, Joseph Hughes, Sreenu Vattipally, David L Robertson; Alasdair MacLean, Rory Gunson; Sharif Shaaban, Matthew Holden; Rachel Blacow, Guy Mollett, Kathy Li, James Shepherd, Antonia Ho, Emma Thomson
EPI_ISL_705735, EPI_ISL_705736, EPI_ISL_705739, EPI_ISL_705740, EPI_ISL_705743, EPI_ISL_705744, EPI_ISL_705745, EPI_ISL_705746, EPI_ISL_705748, EPI_ISL_705749, EPI_ISL_705775, EPI_ISL_705777, EPI_ISL_705780, EPI_ISL_705781, EPI_ISL_705782, EPI_ISL_705784			
see above	Virology Department, Royal Infirmary of Edinburgh, NHS Lothian / School of Biological Sciences, University of Edinburgh / Institute of Genetics and Molecular Medicine, University of Edinburgh	COVID-19 Genomics UK (COG-UK) Consortium	McHugh M, Dewar R, Rooke S, Gallagher M, Balcaza C, O'Toole Á, Scher E, Hill V, McCrone JT, Colquhoun R, Yu X, Jackson B, Rambaut A, Williams TC, Templeton K
EPI_ISL_705929, EPI_ISL_705931	Liverpool Clinical Laboratories	COVID-19 Genomics UK (COG-UK) Consortium	Sam Haldenby, Anita Lucaci, Steve Paterson, Julian Hiscox, Alistair Darby, M Almsaud, A Alrezaihi, Muhannad Alruwaili, Stuart D Armstrong, Jones Benjamin, Eleanor G Bentley, Anu Chawla, Jordan J Clark, Angela Cowell, Richard Eccles, Isabel García-Dorival, Matthew Gemmell, Alessandro Gerada, PKF Gilmore, Richard Gregory, Ximeng Han, Catherine Hartley, Margaret Hughes, Miren Iturriza-Gomara, James Johnson, L Luu, Jenifer Manson, Charlotte Nelson, Elaine O'Toole, Cassie Olateju, Rebekah Penrice-Randal , Lucille Rainbow, N.P Randle, Trevor Ian Robinson, Parul Sharma, Ghada T Shawli, James P Stewart, Neil Swainston, Ecaterina Vamos, Joanne Watts, Mark Whitehead
EPI_ISL_705942, EPI_ISL_705943, EPI_ISL_705944, EPI_ISL_705946, EPI_ISL_705947, EPI_ISL_705948, EPI_ISL_705949, EPI_ISL_705950, EPI_ISL_705952, EPI_ISL_705953, EPI_ISL_705954, EPI_ISL_705955			
see above	University College London Hospital	COVID-19 Genomics UK (COG-UK) Consortium	Judith Heaney, Matthew Byott, Catherine Houlihan, Dan Frampton, Stuart Kirk, Moira Spyer and Eleni Nastouli
EPI_ISL_706284, EPI_ISL_706312, EPI_ISL_706320, EPI_ISL_706321, EPI_ISL_706328, EPI_ISL_706331, EPI_ISL_706334, EPI_ISL_706335, EPI_ISL_706337, EPI_ISL_706338, EPI_ISL_706341, EPI_ISL_706343, EPI_ISL_706344, EPI_ISL_706347, EPI_ISL_706349, EPI_ISL_706350			

see above	Northumbria University / South Tees Hospitals NHS Foundation Trust / North Cumbria Integrated Care NHS Foundation Trust / North Tees and Hartlepool NHS Foundation Trust / Newcastle Hospitals NHS Foundation Trust	COVID-19 Genomics UK (COG-UK) Consortium	Darren L Smith,Andrew Nelson,Matthew Bashton,Greg R Young,Joshua Loh,John Allan,Mohammad A Tariq,Giles S Holt,Gary Black,Wen C Yew,Lynn Dover,Paul Baker,Steve Liggett,Sarah Essex,Jane Greenaway,Debra Padgett,Clive Graham,Garren Scott,Edward Barton,Emma Swindells,Brendan Payne,Jennifer Collins,Yusri Taha,Gary Eltringham
EPI_ISL_706364, EPI_ISL_706367	Quadram Institute Bioscience	COVID-19 Genomics UK (COG-UK) Consortium	Dave J. Baker, Gemma L. Kay, Alp Aydin, Thanh Le-Viet, Steven Rudder, Ana P. Tedim, Anastasia Kolyva, Maria Diaz, Leonardo de Oliveira Martins, Nabil-Fareed Alikhan, Lizzie Meadows, Rachael Stanley, Ngozi Elumogo, Muhammed Yasir, Nicholas M. Thomson, Alexander J Trotter, Rachel Gilroy, Samuel Bloomfield, Claire Stuart, Andrew Bell, Reenesh Prakash, Samir Dervisevic, Alison E. Mather, John Wain, Mark Webber, Andrew J. Page, Justin O'Grady
EPI_ISL_706428	Queens Medical Centre, Clinical Microbiology Department / DeepSeq Nottingham	COVID-19 Genomics UK (COG-UK) Consortium	Gemma Clark, Wendy Smith, Manjinder Khakh, Vicki M Fleming, Michelle M Lister, Hannah Howson-Wells, Jonathan Ball, Patrick McClure, Joseph Chappell, Theocharis Tsoleridis, Nadine Holmes, Matthew Carlisle, Christopher Moore, Fei Sang, Johnny Debebe, Victoria Wright, Matthew Loose
EPI_ISL_706582, EPI_ISL_706583, EPI_ISL_706596, EPI_ISL_706602, EPI_ISL_706622, EPI_ISL_706633, EPI_ISL_706639, EPI_ISL_706641, EPI_ISL_706644, EPI_ISL_706646, EPI_ISL_706776, EPI_ISL_706786, EPI_ISL_706789, EPI_ISL_706807, EPI_ISL_706809, EPI_ISL_706813, EPI_ISL_706816, EPI_ISL_706818, EPI_ISL_706822, EPI_ISL_706827, EPI_ISL_706833, EPI_ISL_706834, EPI_ISL_706837, EPI_ISL_706848, EPI_ISL_706851, EPI_ISL_706861, EPI_ISL_706862			
see above	Wales Specialist Virology Centre Sequencing lab: Pathogen Genomics Unit	COVID-19 Genomics UK (COG-UK) Consortium	Catherine Moore, Johnathan Evans, Laura Gifford, Malorie Perry, Simon Cottrell, Angela Marchbank, Alec Birchley, Alexander Adams, Amy Gaskin, Bree Gatica-Wilcox, Jason Coombes, Joel Southgate, Lauren Gilbert, Lee Graham, Nicole Pacchiarini, Sara Kumziene-Summerhayes, Sarah Taylor, Sophie Jones, Sara Rey, Matthew Bull, Joanne Watkins, Sally Corden, Tom Connor
EPI_ISL_706988, EPI_ISL_706999, EPI_ISL_707006	Virology Department, Sheffield Teaching Hospitals NHS Foundation Trust/Department of Infection, Immunity and Cardiovascular Disease, The Medical School, University of Sheffield	COVID-19 Genomics UK (COG-UK) Consortium	Thushan de Silva, Matthew Parker, Nikki Smith, Adri Angyal, Rebecca Brown, Luke Green, Rachel Tucker, Paul Parsons, Danielle Groves, Katie Johnson, Laura Carrilero, Alex Keeley, Dave Partridge, Matthew Wyles, Benjamin Lindsey, Mehmet Yavuz, Mohammad Raza, Cariad Evans
EPI_ISL_707697, EPI_ISL_707698, EPI_ISL_707699, EPI_ISL_707700	1-Laboratory of Microbiology, National Reference Lab, Charles Nicolle Hospital; 2-University of Tunis ElManar, Faculty of Medicine of Tunis, LR99ES09, Tunis, Tunisia	1-Clinical and Experimental Pharmacology Lab, LR16SP02, National Center of Pharmacovigilance, University of Tunis El Manar, Tunis, Tunisia. 2-Neurodegenerative diseases and psychiatric troubles, LR18SP03, Razi Hospital, University of Tunis El Manar, Tunis, Tunisia. 3- Ministry of Health, National Observatory of New and Emerging Diseases, 1006, Tunis, Tunisia	Ilhem Boutiba-Ben Boubaker, Sameh Trabelsi, Nissaf Ben Alaya, Maher Kharrat, Alia Ben Kahla, Jalila Ben Khelil, Salma Abid, Sana Ferjani, Mouna Ben Sassi, Mouna Safer, Zaineb Hamzaoui, Habiba Ben Romdhane, Souissi Amira, Rouaa Ben Othman, Hanen El Jebari, Asma Ferjani, Gaies Emna, Riadh Daghfous, Riadh Gouider.
EPI_ISL_708136, EPI_ISL_708137, EPI_ISL_708138, EPI_ISL_708139	Medical Microbiology Unit, Department for Laboratory Medicine, Drammen Hospital, Vestre Viken Health Trust,	Norwegian Institute of Public Health, Department of Virology	Kathrine Stene-Johansen, Kamilla Heddeland Instefjord, Hilde Elshaug, Marie Paulsen Madsen, Rasmus Riis Kopperud, Hilde Vollan, Karoline Bragstad, Olav Hungnes
EPI_ISL_708169, EPI_ISL_708170	Norwegian Institute of Public Health, Department of Virology	Norwegian Institute of Public Health, Department of Virology	Kathrine Stene-Johansen, Kamilla Heddeland Instefjord, Hilde Elshaug, Marie Paulsen Madsen, Rasmus Riis Kopperud, Hilde Vollan, Karoline Bragstad, Olav Hungnes
EPI_ISL_708172, EPI_ISL_708173, EPI_ISL_708174, EPI_ISL_708175	Oslo University Hospital, Department of Medical Microbiology	Norwegian Institute of Public Health, Department of Virology	Kathrine Stene-Johansen, Kamilla Heddeland Instefjord, Hilde Elshaug, Marie Paulsen Madsen, Rasmus Riis Kopperud, Hilde Vollan, Karoline Bragstad, Olav Hungnes
EPI_ISL_708181	Norwegian Institute of Public Health, Department of Virology	Norwegian Institute of Public Health, Department of Virology	Kathrine Stene-Johansen, Kamilla Heddeland Instefjord, Hilde Elshaug, Marie Paulsen Madsen, Rasmus Riis Kopperud, Hilde Vollan, Karoline Bragstad, Olav Hungnes
EPI_ISL_708214	Michigan Department of Health and Human Services, Bureau of Laboratories	Michigan Department of Health and Human Services, Bureau of Laboratories	Blankenship HM, Riner D, Soehnlen MK
EPI_ISL_708458, EPI_ISL_708459	Department of Clinical Microbiology	GIGA Medical Genomics	Keith Durkin, Maria Artesi, Justine Defêche, Gilles Darcis, Michel Moutschen, Sébastien Bontems, Raphaël Boreux, Bouchra Boujemla, Cécile Meex, Pierrette Melin, Marie-Pierre Hayette, Vincent Bours
EPI_ISL_708485, EPI_ISL_708486, EPI_ISL_708487, EPI_ISL_708488, EPI_ISL_708489, EPI_ISL_708491, EPI_ISL_708492, EPI_ISL_708493, EPI_ISL_708494, EPI_ISL_708495	Minnesota Department of Health, Public Health Laboratory	Minnesota Department of Health, Public Health Laboratory	Alexandra Lorentz, Jacob Garfin, Matt Plumb, and Xiong Wang
EPI_ISL_708530	Secretaria Municipal de Saude de Fernandópolis	Instituto Adolfo Lutz, Interdiciplinary Procedures Center, Strategic Laboratory	Claudio Tavares Sacchi, Claudia Regina Gonçalves, Erica Valessa Ramos Gomes, Carlos Henrique Camargo, Karoline Rodrigues Campos, Fernanda Modesto Tolentino Binhardi, Maricelia Navarro Pinheiro Flores, Marcia Maria Costa Nunes Soares, Janaina Other Martins Montanha
EPI_ISL_708844, EPI_ISL_708848, EPI_ISL_708849, EPI_ISL_708856, EPI_ISL_708857, EPI_ISL_708858, EPI_ISL_708862, EPI_ISL_708869, EPI_ISL_708871, EPI_ISL_708879, EPI_ISL_708889, EPI_ISL_708896, EPI_ISL_708897, EPI_ISL_708898, EPI_ISL_708900, EPI_ISL_708908, EPI_ISL_708913, EPI_ISL_708916, EPI_ISL_708920, EPI_ISL_708963, EPI_ISL_708969, EPI_ISL_708975, EPI_ISL_708979, EPI_ISL_708983, EPI_ISL_708984, EPI_ISL_708986			
see above	Lighthouse Lab in Milton Keynes	Wellcome Sanger Institute for the COVID-19 Genomics UK (COG-UK) Consortium	The Lighthouse Lab in Milton Keynes and Alex Alderton, Roberto Amato, Sonia Goncalves, Ewan Harrison, David K. Jackson, Ian Johnston, Dominic Kwiatkowski, Cordelia Langford, John Sillitoe on behalf of the Wellcome Sanger Institute COVID-19 Surveillance Team
EPI_ISL_709048	Lighthouse Lab in Glasgow	Wellcome Sanger Institute for the COVID-19 Genomics UK (COG-UK) Consortium	Harper VanSteenhouse, Yumi Kasai, David Gray, Carol Clugston, Anna Dominiczak and Alex Alderton, Roberto Amato, Sonia Goncalves, Ewan Harrison, David K. Jackson, Ian Johnston, Dominic Kwiatkowski, Cordelia Langford, John Sillitoe on behalf of the Wellcome Sanger Institute COVID-19 Surveillance Team
EPI_ISL_709569, EPI_ISL_709571, EPI_ISL_709572, EPI_ISL_709573, EPI_ISL_709574, EPI_ISL_709575, EPI_ISL_709576, EPI_ISL_709577, EPI_ISL_709578, EPI_ISL_709579, EPI_ISL_709582, EPI_ISL_709583, EPI_ISL_709584, EPI_ISL_709585, EPI_ISL_709586, EPI_ISL_709588, EPI_ISL_709589, EPI_ISL_709590, EPI_ISL_709591, EPI_ISL_709592, EPI_ISL_709594, EPI_ISL_709596, EPI_ISL_709597, EPI_ISL_709598, EPI_ISL_709600, EPI_ISL_709602, EPI_ISL_709603, EPI_ISL_709605, EPI_ISL_709607, EPI_ISL_709608, EPI_ISL_709609, EPI_ISL_709610, EPI_ISL_709613, EPI_ISL_709614, EPI_ISL_709615, EPI_ISL_709617, EPI_ISL_709618, EPI_ISL_709619, EPI_ISL_709620, EPI_ISL_709621, EPI_ISL_709626, EPI_ISL_709629, EPI_ISL_709632, EPI_ISL_709633, EPI_ISL_709637, EPI_ISL_709640, EPI_ISL_709643, EPI_ISL_709644, EPI_ISL_709645, EPI_ISL_709646, EPI_ISL_709647, EPI_ISL_709649, EPI_ISL_709650, EPI_ISL_709653, EPI_ISL_709654, EPI_ISL_709655, EPI_ISL_709657, EPI_ISL_709658, EPI_ISL_709659, EPI_ISL_709660, EPI_ISL_709661, EPI_ISL_709662, EPI_ISL_709663, EPI_ISL_709664, EPI_ISL_709665, EPI_ISL_709666, EPI_ISL_709671, EPI_ISL_709673, EPI_ISL_709674, EPI_ISL_709675, EPI_ISL_709676, EPI_ISL_709678, EPI_ISL_709679, EPI_ISL_709680, EPI_ISL_709681, EPI_ISL_709683, EPI_ISL_709684, EPI_ISL_709686, EPI_ISL_709688, EPI_ISL_709689, EPI_ISL_709690, EPI_ISL_709691, EPI_ISL_709693, EPI_ISL_709694, EPI_ISL_709696, EPI_ISL_709697, EPI_ISL_709698, EPI_ISL_709700, EPI_ISL_709702, EPI_ISL_709703, EPI_ISL_709705, EPI_ISL_709709, EPI_ISL_709711, EPI_ISL_709713, EPI_ISL_709714, EPI_ISL_709716, EPI_ISL_709717, EPI_ISL_709719, EPI_ISL_709720, EPI_ISL_709721, EPI_ISL_709723, EPI_ISL_709724, EPI_ISL_709728, EPI_ISL_709730, EPI_ISL_709731, EPI_ISL_709733, EPI_ISL_709734, EPI_ISL_709738, EPI_ISL_709739, EPI_ISL_709742, EPI_ISL_709743, EPI_ISL_709744, EPI_ISL_709747, EPI_ISL_709748, EPI_ISL_709749, EPI_ISL_709750, EPI_ISL_709751, EPI_ISL_709753, EPI_ISL_709754, EPI_ISL_709756, EPI_ISL_709757, EPI_ISL_709758, EPI_ISL_709759, EPI_ISL_709762, EPI_ISL_709763, EPI_ISL_709764, EPI_ISL_709769, EPI_ISL_709770, EPI_ISL_709771, EPI_ISL_709774, EPI_ISL_709775, EPI_ISL_709777, EPI_ISL_709779, EPI_ISL_709780, EPI_ISL_709781, EPI_ISL_709782, EPI_ISL_709783, EPI_ISL_709784, EPI_ISL_709785, EPI_ISL_709786, EPI_ISL_709787, EPI_ISL_709788, EPI_ISL_709790, EPI_ISL_709791, EPI_ISL_709792, EPI_ISL_709793, EPI_ISL_709794, EPI_ISL_709795, EPI_ISL_709796			
see above	Lighthouse Lab in Milton Keynes	Wellcome Sanger Institute for the COVID-19 Genomics UK (COG-UK) Consortium	The Lighthouse Lab in Milton Keynes and Alex Alderton, Roberto Amato, Sonia Goncalves, Ewan Harrison, David K. Jackson, Ian Johnston, Dominic Kwiatkowski, Cordelia Langford, John Sillitoe on behalf of the Wellcome Sanger Institute COVID-19 Surveillance Team
EPI_ISL_709800	Lighthouse Lab in Glasgow	Wellcome Sanger Institute for the COVID-19 Genomics UK (COG-UK) Consortium	Harper VanSteenhouse, Yumi Kasai, David Gray, Carol Clugston, Anna Dominiczak and Alex Alderton, Roberto Amato, Sonia Goncalves, Ewan Harrison, David K. Jackson, Ian Johnston, Dominic Kwiatkowski, Cordelia Langford, John Sillitoe on behalf of the Wellcome Sanger Institute COVID-19 Surveillance Team
EPI_ISL_709802	Lighthouse Lab in Milton Keynes	Wellcome Sanger Institute for the COVID-19 Genomics UK (COG-UK) Consortium	The Lighthouse Lab in Milton Keynes and Alex Alderton, Roberto Amato, Sonia Goncalves, Ewan Harrison, David K. Jackson, Ian Johnston, Dominic Kwiatkowski, Cordelia Langford, John Sillitoe on behalf of the Wellcome Sanger Institute COVID-19 Surveillance Team
EPI_ISL_709805	Lighthouse Lab in Cambridge	Wellcome Sanger Institute for the COVID-19 Genomics UK (COG-UK) Consortium	Rob Howes, The Lighthouse Lab in Cambridge and Alex Alderton, Roberto Amato, Sonia Goncalves, Ewan Harrison, David K. Jackson, Ian Johnston, Dominic Kwiatkowski, Cordelia Langford, John Sillitoe on behalf of the Wellcome Sanger Institute COVID-19 Surveillance Team
EPI_ISL_709806	Lighthouse Lab in Glasgow	Wellcome Sanger Institute for the COVID-19 Genomics UK (COG-UK) Consortium	Harper VanSteenhouse, Yumi Kasai, David Gray, Carol Clugston, Anna Dominiczak and Alex Alderton, Roberto Amato, Sonia Goncalves, Ewan Harrison, David K. Jackson, Ian Johnston, Dominic Kwiatkowski, Cordelia Langford, John Sillitoe on behalf of the Wellcome Sanger Institute COVID-19 Surveillance Team
EPI_ISL_709807, EPI_ISL_709808	Lighthouse Lab in Cambridge	Wellcome Sanger Institute for the COVID-19 Genomics UK (COG-UK) Consortium	Rob Howes, The Lighthouse Lab in Cambridge and Alex Alderton, Roberto Amato, Sonia Goncalves, Ewan Harrison, David K. Jackson, Ian Johnston, Dominic Kwiatkowski, Cordelia Langford, John Sillitoe on behalf of the Wellcome Sanger Institute COVID-19 Surveillance Team

EPI_ISL_709809, EPI_ISL_709812	Lighthouse Lab in Glasgow	Wellcome Sanger Institute for the COVID-19 Genomics UK (COG-UK) Consortium	Harper VanSteenhouse, Yumi Kasai, David Gray, Carol Clugston, Anna Dominiczak and Alex Alderton, Roberto Amato, Sonia Goncalves, Ewan Harrison, David K. Jackson, Ian Johnston, Dominic Kwiatkowski, Cordelia Langford, John Sillitoe on behalf of the Wellcome Sanger Institute COVID-19 Surveillance Team
EPI_ISL_709813, EPI_ISL_709814	Lighthouse Lab in Cambridge	Wellcome Sanger Institute for the COVID-19 Genomics UK (COG-UK) Consortium	Rob Howes, The Lighthouse Lab in Cambridge and Alex Alderton, Roberto Amato, Sonia Goncalves, Ewan Harrison, David K. Jackson, Ian Johnston, Dominic Kwiatkowski, Cordelia Langford, John Sillitoe on behalf of the Wellcome Sanger Institute COVID-19 Surveillance Team
EPI_ISL_709815	Lighthouse Lab in Milton Keynes	Wellcome Sanger Institute for the COVID-19 Genomics UK (COG-UK) Consortium	The Lighthouse Lab in Milton Keynes and Alex Alderton, Roberto Amato, Sonia Goncalves, Ewan Harrison, David K. Jackson, Ian Johnston, Dominic Kwiatkowski, Cordelia Langford, John Sillitoe on behalf of the Wellcome Sanger Institute COVID-19 Surveillance Team
EPI_ISL_709816, EPI_ISL_709818, EPI_ISL_709820	Lighthouse Lab in Glasgow	Wellcome Sanger Institute for the COVID-19 Genomics UK (COG-UK) Consortium	Harper VanSteenhouse, Yumi Kasai, David Gray, Carol Clugston, Anna Dominiczak and Alex Alderton, Roberto Amato, Sonia Goncalves, Ewan Harrison, David K. Jackson, Ian Johnston, Dominic Kwiatkowski, Cordelia Langford, John Sillitoe on behalf of the Wellcome Sanger Institute COVID-19 Surveillance Team
EPI_ISL_709822	Lighthouse Lab in Cambridge	Wellcome Sanger Institute for the COVID-19 Genomics UK (COG-UK) Consortium	Rob Howes, The Lighthouse Lab in Cambridge and Alex Alderton, Roberto Amato, Sonia Goncalves, Ewan Harrison, David K. Jackson, Ian Johnston, Dominic Kwiatkowski, Cordelia Langford, John Sillitoe on behalf of the Wellcome Sanger Institute COVID-19 Surveillance Team
EPI_ISL_709824	Lighthouse Lab in Milton Keynes	Wellcome Sanger Institute for the COVID-19 Genomics UK (COG-UK) Consortium	The Lighthouse Lab in Milton Keynes and Alex Alderton, Roberto Amato, Sonia Goncalves, Ewan Harrison, David K. Jackson, Ian Johnston, Dominic Kwiatkowski, Cordelia Langford, John Sillitoe on behalf of the Wellcome Sanger Institute COVID-19 Surveillance Team
EPI_ISL_709827, EPI_ISL_709829	Lighthouse Lab in Cambridge	Wellcome Sanger Institute for the COVID-19 Genomics UK (COG-UK) Consortium	Rob Howes, The Lighthouse Lab in Cambridge and Alex Alderton, Roberto Amato, Sonia Goncalves, Ewan Harrison, David K. Jackson, Ian Johnston, Dominic Kwiatkowski, Cordelia Langford, John Sillitoe on behalf of the Wellcome Sanger Institute COVID-19 Surveillance Team
EPI_ISL_709830	Lighthouse Lab in Milton Keynes	Wellcome Sanger Institute for the COVID-19 Genomics UK (COG-UK) Consortium	The Lighthouse Lab in Milton Keynes and Alex Alderton, Roberto Amato, Sonia Goncalves, Ewan Harrison, David K. Jackson, Ian Johnston, Dominic Kwiatkowski, Cordelia Langford, John Sillitoe on behalf of the Wellcome Sanger Institute COVID-19 Surveillance Team
EPI_ISL_709831	Lighthouse Lab in Glasgow	Wellcome Sanger Institute for the COVID-19 Genomics UK (COG-UK) Consortium	Harper VanSteenhouse, Yumi Kasai, David Gray, Carol Clugston, Anna Dominiczak and Alex Alderton, Roberto Amato, Sonia Goncalves, Ewan Harrison, David K. Jackson, Ian Johnston, Dominic Kwiatkowski, Cordelia Langford, John Sillitoe on behalf of the Wellcome Sanger Institute COVID-19 Surveillance Team
EPI_ISL_709833	Lighthouse Lab in Milton Keynes	Wellcome Sanger Institute for the COVID-19 Genomics UK (COG-UK) Consortium	The Lighthouse Lab in Milton Keynes and Alex Alderton, Roberto Amato, Sonia Goncalves, Ewan Harrison, David K. Jackson, Ian Johnston, Dominic Kwiatkowski, Cordelia Langford, John Sillitoe on behalf of the Wellcome Sanger Institute COVID-19 Surveillance Team
EPI_ISL_709834, EPI_ISL_709835	Lighthouse Lab in Glasgow	Wellcome Sanger Institute for the COVID-19 Genomics UK (COG-UK) Consortium	Harper VanSteenhouse, Yumi Kasai, David Gray, Carol Clugston, Anna Dominiczak and Alex Alderton, Roberto Amato, Sonia Goncalves, Ewan Harrison, David K. Jackson, Ian Johnston, Dominic Kwiatkowski, Cordelia Langford, John Sillitoe on behalf of the Wellcome Sanger Institute COVID-19 Surveillance Team
EPI_ISL_709836	Lighthouse Lab in Cambridge	Wellcome Sanger Institute for the COVID-19 Genomics UK (COG-UK) Consortium	Rob Howes, The Lighthouse Lab in Cambridge and Alex Alderton, Roberto Amato, Sonia Goncalves, Ewan Harrison, David K. Jackson, Ian Johnston, Dominic Kwiatkowski, Cordelia Langford, John Sillitoe on behalf of the Wellcome Sanger Institute COVID-19 Surveillance Team
EPI_ISL_709838, EPI_ISL_709839, EPI_ISL_709841	Lighthouse Lab in Milton Keynes	Wellcome Sanger Institute for the COVID-19 Genomics UK (COG-UK) Consortium	The Lighthouse Lab in Milton Keynes and Alex Alderton, Roberto Amato, Sonia Goncalves, Ewan Harrison, David K. Jackson, Ian Johnston, Dominic Kwiatkowski, Cordelia Langford, John Sillitoe on behalf of the Wellcome Sanger Institute COVID-19 Surveillance Team
EPI_ISL_709842, EPI_ISL_709844, EPI_ISL_709846	Lighthouse Lab in Cambridge	Wellcome Sanger Institute for the COVID-19 Genomics UK (COG-UK) Consortium	Rob Howes, The Lighthouse Lab in Cambridge and Alex Alderton, Roberto Amato, Sonia Goncalves, Ewan Harrison, David K. Jackson, Ian Johnston, Dominic Kwiatkowski, Cordelia Langford, John Sillitoe on behalf of the Wellcome Sanger Institute COVID-19 Surveillance Team
EPI_ISL_709847	Lighthouse Lab in Milton Keynes	Wellcome Sanger Institute for the COVID-19 Genomics UK (COG-UK) Consortium	The Lighthouse Lab in Milton Keynes and Alex Alderton, Roberto Amato, Sonia Goncalves, Ewan Harrison, David K. Jackson, Ian Johnston, Dominic Kwiatkowski, Cordelia Langford, John Sillitoe on behalf of the Wellcome Sanger Institute COVID-19 Surveillance Team
EPI_ISL_709848	Lighthouse Lab in Glasgow	Wellcome Sanger Institute for the COVID-19 Genomics UK (COG-UK) Consortium	Harper VanSteenhouse, Yumi Kasai, David Gray, Carol Clugston, Anna Dominiczak and Alex Alderton, Roberto Amato, Sonia Goncalves, Ewan Harrison, David K. Jackson, Ian Johnston, Dominic Kwiatkowski, Cordelia Langford, John Sillitoe on behalf of the Wellcome Sanger Institute COVID-19 Surveillance Team
EPI_ISL_709852	Lighthouse Lab in Milton Keynes	Wellcome Sanger Institute for the COVID-19 Genomics UK (COG-UK) Consortium	The Lighthouse Lab in Milton Keynes and Alex Alderton, Roberto Amato, Sonia Goncalves, Ewan Harrison, David K. Jackson, Ian Johnston, Dominic Kwiatkowski, Cordelia Langford, John Sillitoe on behalf of the Wellcome Sanger Institute COVID-19 Surveillance Team
EPI_ISL_709854	Lighthouse Lab in Cambridge	Wellcome Sanger Institute for the COVID-19 Genomics UK (COG-UK) Consortium	Rob Howes, The Lighthouse Lab in Cambridge and Alex Alderton, Roberto Amato, Sonia Goncalves, Ewan Harrison, David K. Jackson, Ian Johnston, Dominic Kwiatkowski, Cordelia Langford, John Sillitoe on behalf of the Wellcome Sanger Institute COVID-19 Surveillance Team
EPI_ISL_709856	Lighthouse Lab in Milton Keynes	Wellcome Sanger Institute for the COVID-19 Genomics UK (COG-UK) Consortium	The Lighthouse Lab in Milton Keynes and Alex Alderton, Roberto Amato, Sonia Goncalves, Ewan Harrison, David K. Jackson, Ian Johnston, Dominic Kwiatkowski, Cordelia Langford, John Sillitoe on behalf of the Wellcome Sanger Institute COVID-19 Surveillance Team
EPI_ISL_709857, EPI_ISL_709858, EPI_ISL_709859	Lighthouse Lab in Glasgow	Wellcome Sanger Institute for the COVID-19 Genomics UK (COG-UK) Consortium	Harper VanSteenhouse, Yumi Kasai, David Gray, Carol Clugston, Anna Dominiczak and Alex Alderton, Roberto Amato, Sonia Goncalves, Ewan Harrison, David K. Jackson, Ian Johnston, Dominic Kwiatkowski, Cordelia Langford, John Sillitoe on behalf of the Wellcome Sanger Institute COVID-19 Surveillance Team
EPI_ISL_709860	Lighthouse Lab in Milton Keynes	Wellcome Sanger Institute for the COVID-19 Genomics UK (COG-UK) Consortium	The Lighthouse Lab in Milton Keynes and Alex Alderton, Roberto Amato, Sonia Goncalves, Ewan Harrison, David K. Jackson, Ian Johnston, Dominic Kwiatkowski, Cordelia Langford, John Sillitoe on behalf of the Wellcome Sanger Institute COVID-19 Surveillance Team
EPI_ISL_709861, EPI_ISL_709862	Lighthouse Lab in Glasgow	Wellcome Sanger Institute for the COVID-19 Genomics UK (COG-UK) Consortium	Harper VanSteenhouse, Yumi Kasai, David Gray, Carol Clugston, Anna Dominiczak and Alex Alderton, Roberto Amato, Sonia Goncalves, Ewan Harrison, David K. Jackson, Ian Johnston, Dominic Kwiatkowski, Cordelia Langford, John Sillitoe on behalf of the Wellcome Sanger Institute COVID-19 Surveillance Team
EPI_ISL_709863, EPI_ISL_709866	Lighthouse Lab in Milton Keynes	Wellcome Sanger Institute for the COVID-19 Genomics UK (COG-UK) Consortium	The Lighthouse Lab in Milton Keynes and Alex Alderton, Roberto Amato, Sonia Goncalves, Ewan Harrison, David K. Jackson, Ian Johnston, Dominic Kwiatkowski, Cordelia Langford, John Sillitoe on behalf of the Wellcome Sanger Institute COVID-19 Surveillance Team
EPI_ISL_709867, EPI_ISL_709869, EPI_ISL_709870	Lighthouse Lab in Cambridge	Wellcome Sanger Institute for the COVID-19 Genomics UK (COG-UK) Consortium	Rob Howes, The Lighthouse Lab in Cambridge and Alex Alderton, Roberto Amato, Sonia Goncalves, Ewan Harrison, David K. Jackson, Ian Johnston, Dominic Kwiatkowski, Cordelia Langford, John Sillitoe on behalf of the Wellcome Sanger Institute COVID-19 Surveillance Team
EPI_ISL_709871, EPI_ISL_709872	Lighthouse Lab in Milton Keynes	Wellcome Sanger Institute for the COVID-19 Genomics UK (COG-UK) Consortium	The Lighthouse Lab in Milton Keynes and Alex Alderton, Roberto Amato, Sonia Goncalves, Ewan Harrison, David K. Jackson, Ian Johnston, Dominic Kwiatkowski, Cordelia Langford, John Sillitoe on behalf of the Wellcome Sanger Institute COVID-19 Surveillance Team
EPI_ISL_709873	Lighthouse Lab in Cambridge	Wellcome Sanger Institute for the COVID-19 Genomics UK (COG-UK) Consortium	Rob Howes, The Lighthouse Lab in Cambridge and Alex Alderton, Roberto Amato, Sonia Goncalves, Ewan Harrison, David K. Jackson, Ian Johnston, Dominic Kwiatkowski, Cordelia Langford, John Sillitoe on behalf of the Wellcome Sanger Institute COVID-19 Surveillance Team
EPI_ISL_709874	Lighthouse Lab in Milton Keynes	Wellcome Sanger Institute for the COVID-19 Genomics UK (COG-UK) Consortium	The Lighthouse Lab in Milton Keynes and Alex Alderton, Roberto Amato, Sonia Goncalves, Ewan Harrison, David K. Jackson, Ian Johnston, Dominic Kwiatkowski, Cordelia Langford, John Sillitoe on behalf of the Wellcome Sanger Institute COVID-19 Surveillance Team
EPI_ISL_709875	Lighthouse Lab in Glasgow	Wellcome Sanger Institute for the COVID-19 Genomics UK (COG-UK) Consortium	Harper VanSteenhouse, Yumi Kasai, David Gray, Carol Clugston, Anna Dominiczak and Alex Alderton, Roberto Amato, Sonia Goncalves, Ewan Harrison, David K. Jackson, Ian Johnston, Dominic Kwiatkowski, Cordelia Langford, John Sillitoe on behalf of the Wellcome Sanger Institute COVID-19 Surveillance Team
EPI_ISL_709876, EPI_ISL_709878	Lighthouse Lab in Cambridge	Wellcome Sanger Institute for the COVID-19 Genomics UK (COG-UK) Consortium	Rob Howes, The Lighthouse Lab in Cambridge and Alex Alderton, Roberto Amato, Sonia Goncalves, Ewan Harrison, David K. Jackson, Ian Johnston, Dominic Kwiatkowski, Cordelia Langford, John Sillitoe on behalf of the Wellcome Sanger Institute COVID-19 Surveillance Team
EPI_ISL_709879	Lighthouse Lab in Milton Keynes	Wellcome Sanger Institute for the COVID-19 Genomics UK (COG-UK) Consortium	The Lighthouse Lab in Milton Keynes and Alex Alderton, Roberto Amato, Sonia Goncalves, Ewan Harrison, David K. Jackson, Ian Johnston, Dominic Kwiatkowski, Cordelia Langford, John Sillitoe on behalf of the Wellcome Sanger Institute COVID-19 Surveillance Team
EPI_ISL_709880	Lighthouse Lab in Cambridge	Wellcome Sanger Institute for the COVID-19 Genomics UK (COG-UK) Consortium	Rob Howes, The Lighthouse Lab in Cambridge and Alex Alderton, Roberto Amato, Sonia Goncalves, Ewan Harrison, David K. Jackson, Ian Johnston, Dominic Kwiatkowski, Cordelia Langford, John Sillitoe on behalf of the Wellcome Sanger Institute COVID-19 Surveillance Team
EPI_ISL_709881	Lighthouse Lab in Milton Keynes	Wellcome Sanger Institute for the COVID-19 Genomics UK (COG-UK) Consortium	The Lighthouse Lab in Milton Keynes and Alex Alderton, Roberto Amato, Sonia Goncalves, Ewan Harrison, David K. Jackson, Ian Johnston, Dominic Kwiatkowski, Cordelia Langford, John Sillitoe on behalf of the Wellcome Sanger Institute COVID-19 Surveillance Team

EPI_ISL_710576	VC Sorgenfrimottagningen	The Public Health Agency of Sweden	Department of Microbiology, The Public Health Agency of Sweden
EPI_ISL_710577, EPI_ISL_710578	Vardcentralen Brinken	The Public Health Agency of Sweden	Department of Microbiology, The Public Health Agency of Sweden
EPI_ISL_710581, EPI_ISL_710582, EPI_ISL_710583	The Public Health Agency of Sweden	The Public Health Agency of Sweden	Department of Microbiology, The Public Health Agency of Sweden
EPI_ISL_710585, EPI_ISL_710586	Orsa VC	The Public Health Agency of Sweden	Department of Microbiology, The Public Health Agency of Sweden
EPI_ISL_710588	Omtanken Grimmered	The Public Health Agency of Sweden	Department of Microbiology, The Public Health Agency of Sweden
EPI_ISL_710589, EPI_ISL_710590	Narhalsan Fjallbacka VC	The Public Health Agency of Sweden	Department of Microbiology, The Public Health Agency of Sweden
EPI_ISL_710604	Klinisk mikrobiologi	The Public Health Agency of Sweden	Department of Microbiology, The Public Health Agency of Sweden
EPI_ISL_710607	Halsomedicinskt Center	The Public Health Agency of Sweden	Department of Microbiology, The Public Health Agency of Sweden
EPI_ISL_710612	Capio Bro VC	The Public Health Agency of Sweden	Department of Microbiology, The Public Health Agency of Sweden
EPI_ISL_710699, EPI_ISL_710700, EPI_ISL_710701, EPI_ISL_710702, EPI_ISL_710703, EPI_ISL_710706, EPI_ISL_710707, EPI_ISL_710709, EPI_ISL_710711, EPI_ISL_710715, EPI_ISL_710716, EPI_ISL_710717, EPI_ISL_710718, EPI_ISL_710719, EPI_ISL_710720, EPI_ISL_710721, EPI_ISL_710722, EPI_ISL_710723, EPI_ISL_710724, EPI_ISL_710726, EPI_ISL_710728, EPI_ISL_710729, EPI_ISL_710732, EPI_ISL_710733, EPI_ISL_710735, EPI_ISL_710736, EPI_ISL_710737, EPI_ISL_710739, EPI_ISL_710741, EPI_ISL_710742, EPI_ISL_710744, EPI_ISL_710745			
see above	Lighthouse Lab in Alderley Park	Wellcome Sanger Institute for the COVID-19 Genomics UK (COG-UK) Consortium	Jacquelyn Wynn, Mairead Hyland, The Lighthouse Lab in Alderley Park and Alex Alderton, Roberto Amato, Sonia Goncalves, Ewan Harrison, David K. Jackson, Ian Johnston, Dominic Kwiatkowski, Cordelia Langford, John Sillitoe on behalf of the Wellcome Sanger Institute COVID-19 Surveillance Team
EPI_ISL_710748	Lighthouse Lab in Glasgow	Wellcome Sanger Institute for the COVID-19 Genomics UK (COG-UK) Consortium	Harper VanSteenhouse, Yumi Kasai, David Gray, Carol Clugston, Anna Dominiczak and Alex Alderton, Roberto Amato, Sonia Goncalves, Ewan Harrison, David K. Jackson, Ian Johnston, Dominic Kwiatkowski, Cordelia Langford, John Sillitoe on behalf of the Wellcome Sanger Institute COVID-19 Surveillance Team
EPI_ISL_710749, EPI_ISL_710750, EPI_ISL_710751	Lighthouse Lab in Alderley Park	Wellcome Sanger Institute for the COVID-19 Genomics UK (COG-UK) Consortium	Jacquelyn Wynn, Mairead Hyland, The Lighthouse Lab in Alderley Park and Alex Alderton, Roberto Amato, Sonia Goncalves, Ewan Harrison, David K. Jackson, Ian Johnston, Dominic Kwiatkowski, Cordelia Langford, John Sillitoe on behalf of the Wellcome Sanger Institute COVID-19 Surveillance Team
EPI_ISL_710753, EPI_ISL_710754, EPI_ISL_710755, EPI_ISL_710758, EPI_ISL_710759, EPI_ISL_710760, EPI_ISL_710761, EPI_ISL_710762, EPI_ISL_710763, EPI_ISL_710764, EPI_ISL_710765, EPI_ISL_710766, EPI_ISL_710768, EPI_ISL_710771, EPI_ISL_710772, EPI_ISL_710774, EPI_ISL_710775, EPI_ISL_710776, EPI_ISL_710777, EPI_ISL_710779, EPI_ISL_710780, EPI_ISL_710781, EPI_ISL_710783, EPI_ISL_710785, EPI_ISL_710786, EPI_ISL_710787, EPI_ISL_710789, EPI_ISL_710790, EPI_ISL_710793, EPI_ISL_710794, EPI_ISL_710795, EPI_ISL_710797, EPI_ISL_710799, EPI_ISL_710801, EPI_ISL_710802, EPI_ISL_710803, EPI_ISL_710804, EPI_ISL_710805, EPI_ISL_710807, EPI_ISL_710808, EPI_ISL_710809, EPI_ISL_710810, EPI_ISL_710811, EPI_ISL_710812, EPI_ISL_710813, EPI_ISL_710814, EPI_ISL_710815, EPI_ISL_710817, EPI_ISL_710818, EPI_ISL_710819, EPI_ISL_710820			
see above	Lighthouse Lab in Glasgow	Wellcome Sanger Institute for the COVID-19 Genomics UK (COG-UK) Consortium	Harper VanSteenhouse, Yumi Kasai, David Gray, Carol Clugston, Anna Dominiczak and Alex Alderton, Roberto Amato, Sonia Goncalves, Ewan Harrison, David K. Jackson, Ian Johnston, Dominic Kwiatkowski, Cordelia Langford, John Sillitoe on behalf of the Wellcome Sanger Institute COVID-19 Surveillance Team
EPI_ISL_710823, EPI_ISL_710825, EPI_ISL_710828, EPI_ISL_710829	Lighthouse Lab in Cambridge	Wellcome Sanger Institute for the COVID-19 Genomics UK (COG-UK) Consortium	Rob Howes, The Lighthouse Lab in Cambridge and Alex Alderton, Roberto Amato, Sonia Goncalves, Ewan Harrison, David K. Jackson, Ian Johnston, Dominic Kwiatkowski, Cordelia Langford, John Sillitoe on behalf of the Wellcome Sanger Institute COVID-19 Surveillance Team
EPI_ISL_711229, EPI_ISL_711230, EPI_ISL_711231, EPI_ISL_711232, EPI_ISL_711233, EPI_ISL_711234, EPI_ISL_711235, EPI_ISL_711236, EPI_ISL_711237, EPI_ISL_711238, EPI_ISL_711239, EPI_ISL_711240, EPI_ISL_711241, EPI_ISL_711242, EPI_ISL_711243, EPI_ISL_711244, EPI_ISL_711245, EPI_ISL_711246, EPI_ISL_711247, EPI_ISL_711248, EPI_ISL_711249, EPI_ISL_711250, EPI_ISL_711251, EPI_ISL_711252, EPI_ISL_711253, EPI_ISL_711254, EPI_ISL_711255, EPI_ISL_711256, EPI_ISL_711257, EPI_ISL_711258, EPI_ISL_711259, EPI_ISL_711260, EPI_ISL_711261, EPI_ISL_711262, EPI_ISL_711263, EPI_ISL_711264, EPI_ISL_711265, EPI_ISL_711266, EPI_ISL_711268, EPI_ISL_711269, EPI_ISL_711270, EPI_ISL_711271, EPI_ISL_711272, EPI_ISL_711286, EPI_ISL_711287, EPI_ISL_711288, EPI_ISL_711289, EPI_ISL_711290, EPI_ISL_711291, EPI_ISL_711292, EPI_ISL_711293, EPI_ISL_711295, EPI_ISL_711297, EPI_ISL_711298, EPI_ISL_711301, EPI_ISL_711302, EPI_ISL_711304, EPI_ISL_711305, EPI_ISL_711306, EPI_ISL_711307, EPI_ISL_711308, EPI_ISL_711309, EPI_ISL_711310, EPI_ISL_711311, EPI_ISL_711312, EPI_ISL_711313, EPI_ISL_711314, EPI_ISL_711315, EPI_ISL_711316, EPI_ISL_711317, EPI_ISL_711319, EPI_ISL_711321, EPI_ISL_711322, EPI_ISL_711323, EPI_ISL_711324, EPI_ISL_711325, EPI_ISL_711326, EPI_ISL_711327, EPI_ISL_711328, EPI_ISL_711329, EPI_ISL_711330, EPI_ISL_711331, EPI_ISL_711332, EPI_ISL_711333, EPI_ISL_711334, EPI_ISL_711335, EPI_ISL_711336, EPI_ISL_711337, EPI_ISL_711338, EPI_ISL_711339, EPI_ISL_711340, EPI_ISL_711341, EPI_ISL_711342, EPI_ISL_711343, EPI_ISL_711344, EPI_ISL_711345, EPI_ISL_711346, EPI_ISL_711347, EPI_ISL_711348, EPI_ISL_711349, EPI_ISL_711350, EPI_ISL_711351, EPI_ISL_711352, EPI_ISL_711353, EPI_ISL_711354, EPI_ISL_711355, EPI_ISL_711356, EPI_ISL_711357, EPI_ISL_711358, EPI_ISL_711359, EPI_ISL_711360, EPI_ISL_711361, EPI_ISL_711362, EPI_ISL_711363, EPI_ISL_711365, EPI_ISL_711366, EPI_ISL_711367, EPI_ISL_711368, EPI_ISL_711369, EPI_ISL_711370, EPI_ISL_711372, EPI_ISL_711373, EPI_ISL_711374, EPI_ISL_711375, EPI_ISL_711376, EPI_ISL_711377, EPI_ISL_711378, EPI_ISL_711379, EPI_ISL_711380, EPI_ISL_711381, EPI_ISL_711382, EPI_ISL_711383, EPI_ISL_711388, EPI_ISL_711389, EPI_ISL_711390, EPI_ISL_711394, EPI_ISL_711445, EPI_ISL_711446, EPI_ISL_711448, EPI_ISL_711449, EPI_ISL_711450, EPI_ISL_711451, EPI_ISL_711452, EPI_ISL_711453, EPI_ISL_711454, EPI_ISL_711455, EPI_ISL_711456, EPI_ISL_711457, EPI_ISL_711458, EPI_ISL_711459, EPI_ISL_711460, EPI_ISL_711461, EPI_ISL_711463, EPI_ISL_711464, EPI_ISL_711465, EPI_ISL_711466, EPI_ISL_711467, EPI_ISL_711468, EPI_ISL_711469, EPI_ISL_711470, EPI_ISL_711471, EPI_ISL_711472, EPI_ISL_711473, EPI_ISL_711474, EPI_ISL_711475, EPI_ISL_711476, EPI_ISL_711478, EPI_ISL_711479, EPI_ISL_711480, EPI_ISL_711481, EPI_ISL_711482, EPI_ISL_711483, EPI_ISL_711484, EPI_ISL_711485, EPI_ISL_711486, EPI_ISL_711487, EPI_ISL_711488, EPI_ISL_711489, EPI_ISL_711490, EPI_ISL_711491, EPI_ISL_711492, EPI_ISL_711493, EPI_ISL_711494, EPI_ISL_711495, EPI_ISL_711496, EPI_ISL_711497, EPI_ISL_711498, EPI_ISL_711499, EPI_ISL_711500, EPI_ISL_711502, EPI_ISL_711503, EPI_ISL_711504, EPI_ISL_711505, EPI_ISL_711506, EPI_ISL_711507, EPI_ISL_711508, EPI_ISL_711509, EPI_ISL_711510, EPI_ISL_711511, EPI_ISL_711512, EPI_ISL_711513, EPI_ISL_711514, EPI_ISL_711515, EPI_ISL_711516, EPI_ISL_711517, EPI_ISL_711518, EPI_ISL_711520, EPI_ISL_711521, EPI_ISL_711522, EPI_ISL_711523, EPI_ISL_711524, EPI_ISL_711525, EPI_ISL_711526, EPI_ISL_711527, EPI_ISL_711528, EPI_ISL_711529, EPI_ISL_711530, EPI_ISL_711531, EPI_ISL_711532, EPI_ISL_711533, EPI_ISL_711534, EPI_ISL_711536, EPI_ISL_711537, EPI_ISL_711538, EPI_ISL_711539, EPI_ISL_711540, EPI_ISL_711541, EPI_ISL_711542, EPI_ISL_711544, EPI_ISL_711545, EPI_ISL_711547, EPI_ISL_711548, EPI_ISL_711549, EPI_ISL_711550, EPI_ISL_711551, EPI_ISL_711552, EPI_ISL_711553, EPI_ISL_711554, EPI_ISL_711555, EPI_ISL_711556, EPI_ISL_711557, EPI_ISL_711558, EPI_ISL_711559, EPI_ISL_711560, EPI_ISL_711561, EPI_ISL_711562, EPI_ISL_711563, EPI_ISL_711564, EPI_ISL_711565, EPI_ISL_711566, EPI_ISL_711567, EPI_ISL_711568, EPI_ISL_711569, EPI_ISL_711570, EPI_ISL_711571, EPI_ISL_711572, EPI_ISL_711573, EPI_ISL_711574, EPI_ISL_711575, EPI_ISL_711576, EPI_ISL_711577, EPI_ISL_711579, EPI_ISL_711580, EPI_ISL_711581, EPI_ISL_711582, EPI_ISL_711583, EPI_ISL_711584, EPI_ISL_711585, EPI_ISL_711586, EPI_ISL_711587, EPI_ISL_711588, EPI_ISL_711589, EPI_ISL_711590, EPI_ISL_711591, EPI_ISL_711592, EPI_ISL_711593, EPI_ISL_711594, EPI_ISL_711595, EPI_ISL_711596, EPI_ISL_711597, EPI_ISL_711598, EPI_ISL_711599, EPI_ISL_711600, EPI_ISL_711601, EPI_ISL_711602, EPI_ISL_711603, EPI_ISL_711604, EPI_ISL_711605, EPI_ISL_711606, EPI_ISL_711607, EPI_ISL_711609, EPI_ISL_711610, EPI_ISL_711611, EPI_ISL_711612, EPI_ISL_711613, EPI_ISL_711614, EPI_ISL_711615, EPI_ISL_711616, EPI_ISL_711617, EPI_ISL_711618, EPI_ISL_711619, EPI_ISL_711620, EPI_ISL_711621, EPI_ISL_711622, EPI_ISL_711623, EPI_ISL_711624, EPI_ISL_711625, EPI_ISL_711626, EPI_ISL_711627, EPI_ISL_711628, EPI_ISL_711629, EPI_ISL_711630, EPI_ISL_711631, EPI_ISL_711632, EPI_ISL_711633, EPI_ISL_711634, EPI_ISL_711635, EPI_ISL_711636, EPI_ISL_711637, EPI_ISL_711638, EPI_ISL_711639, EPI_ISL_711640, EPI_ISL_711641, EPI_ISL_711642, EPI_ISL_711644, EPI_ISL_711645, EPI_ISL_711646, EPI_ISL_711647, EPI_ISL_711648, EPI_ISL_711649, EPI_ISL_711650, EPI_ISL_711652, EPI_ISL_711653, EPI_ISL_711654, EPI_ISL_711655, EPI_ISL_711656, EPI_ISL_711657, EPI_ISL_711658, EPI_ISL_711659, EPI_ISL_711660, EPI_ISL_711661, EPI_ISL_711662, EPI_ISL_711664, EPI_ISL_711665, EPI_ISL_711666, EPI_ISL_711667, EPI_ISL_711668, EPI_ISL_711669, EPI_ISL_711670, EPI_ISL_711671, EPI_ISL_711672, EPI_ISL_711673, EPI_ISL_711674, EPI_ISL_711675, EPI_ISL_711676, EPI_ISL_711677, EPI_ISL_711678, EPI_ISL_711680, EPI_ISL_711681, EPI_ISL_711682, EPI_ISL_711683, EPI_ISL_711684, EPI_ISL_711685, EPI_ISL_711686, EPI_ISL_711687, EPI_ISL_711688, EPI_ISL_711689, EPI_ISL_711690, EPI_ISL_711691, EPI_ISL_711692, EPI_ISL_711693, EPI_ISL_711695, EPI_ISL_711696, EPI_ISL_711697, EPI_ISL_711699, EPI_ISL_711700, EPI_ISL_711701, EPI_ISL_711703, EPI_ISL_711704, EPI_ISL_711705, EPI_ISL_711707, EPI_ISL_711708, EPI_ISL_711709, EPI_ISL_711710, EPI_ISL_711711, EPI_ISL_711712, EPI_ISL_711713, EPI_ISL_711714, EPI_ISL_711715, EPI_ISL_711716, EPI_ISL_711717, EPI_ISL_711718, EPI_ISL_711719, EPI_ISL_711720, EPI_ISL_711721, EPI_ISL_711722, EPI_ISL_711723, EPI_ISL_711724, EPI_ISL_711725, EPI_ISL_711726, EPI_ISL_711727, EPI_ISL_711728, EPI_ISL_711729, EPI_ISL_711730, EPI_ISL_711731, EPI_ISL_711732, EPI_ISL_711733, EPI_ISL_711734, EPI_ISL_711735, EPI_ISL_711736, EPI_ISL_711737, EPI_ISL_711738, EPI_ISL_711739, EPI_ISL_711740, EPI_ISL_711741, EPI_ISL_711742, EPI_ISL_711743, EPI_ISL_711745, EPI_ISL_711746, EPI_ISL_711747, EPI_ISL_711748, EPI_ISL_711749, EPI_ISL_711750, EPI_ISL_711751, EPI_ISL_711752, EPI_ISL_711754, EPI_ISL_711755, EPI_ISL_711756, EPI_ISL_711757, EPI_ISL_711758, EPI_ISL_711760, EPI_ISL_711761, EPI_ISL_711763, EPI_ISL_711764, EPI_ISL_711765, EPI_ISL_711766, EPI_ISL_711767, EPI_ISL_711768, EPI_ISL_711769, EPI_ISL_711770, EPI_ISL_711771, EPI_ISL_711772, EPI_ISL_711773, EPI_ISL_711774, EPI_ISL_711775, EPI_ISL_711776, EPI_ISL_711777, EPI_ISL_711780, EPI_ISL_711782, EPI_ISL_711783, EPI_ISL_711784, EPI_ISL_711785, EPI_ISL_711786, EPI_ISL_711787, EPI_ISL_711788, EPI_ISL_711789, EPI_ISL_711790, EPI_ISL_711791, EPI_ISL_711792, EPI_ISL_711793, EPI_ISL_711794, EPI_ISL_711795, EPI_ISL_711796, EPI_ISL_711797, EPI_ISL_711799, EPI_ISL_711800, EPI_ISL_711801, EPI_ISL_711802, EPI_ISL_711803, EPI_ISL_711804, EPI_ISL_711805, EPI_ISL_711806, EPI_ISL_711807, EPI_ISL_711808, EPI_ISL_711809, EPI_ISL_711810, EPI_ISL_711811, EPI_ISL_711812, EPI_ISL_711813, EPI_ISL_711814, EPI_ISL_711816, EPI_ISL_711817, EPI_ISL_711818, EPI_ISL_711819, EPI_ISL_711820, EPI_ISL_711821, EPI_ISL_711822, EPI_ISL_711823, EPI_ISL_711824, EPI_ISL_711826, EPI_ISL_711827, EPI_ISL_711828, EPI_ISL_711829, EPI_ISL_711830, EPI_ISL_711831, EPI_ISL_711832, EPI_ISL_711833, EPI_ISL_711834, EPI_ISL_711835, EPI_ISL_711836, EPI_ISL_711837, EPI_ISL_711838, EPI_ISL_711839, EPI_ISL_711840, EPI_ISL_711842, EPI_ISL_711843, EPI_ISL_711844, EPI_ISL_711845, EPI_ISL_711846, EPI_ISL_711847, EPI_ISL_711848, EPI_ISL_711849, EPI_ISL_711850, EPI_ISL_711851, EPI_ISL_711852, EPI_ISL_711853, EPI_ISL_711854, EPI_ISL_711855, EPI_ISL_711856, EPI_ISL_711858, EPI_ISL_711859, EPI_ISL_711860, EPI_ISL_711861, EPI_ISL_711862, EPI_ISL_711863, EPI_ISL_711864, EPI_ISL_711865, EPI_ISL_711867, EPI_ISL_711868, EPI_ISL_711869, EPI_ISL_711870, EPI_ISL_711871, EPI_ISL_711872, EPI_ISL_711873, EPI_ISL_711874, EPI_ISL_711875, EPI_ISL_711876, EPI_ISL_711877, EPI_ISL_711878, EPI_ISL_711879, EPI_ISL_711880, EPI_ISL_711881, EPI_ISL_711882, EPI_ISL_711883, EPI_ISL_711884, EPI_ISL_711885, EPI_ISL_711886, EPI_ISL_711887, EPI_ISL_711888, EPI_ISL_711889, EPI_ISL_711890, EPI_ISL_711891, EPI_ISL_711892, EPI_ISL_711893, EPI_ISL_711894, EPI_ISL_711895, EPI_ISL_711896, EPI_ISL_711897, EPI_ISL_711898, EPI_ISL_711899, EPI_ISL_711900, EPI_ISL_711901, EPI_ISL_711902, EPI_ISL_711903, EPI_ISL_711904, EPI_ISL_711905, EPI_ISL_711906, EPI_ISL_711907, EPI_ISL_711908, EPI_ISL_711909, EPI_ISL_711910, EPI_ISL_711911, EPI_ISL_711912, EPI_ISL_711913, EPI_ISL_711914, EPI_ISL_711915, EPI_ISL_711916, EPI_ISL_711918, EPI_ISL_711919, EPI_ISL_711920, EPI_ISL_711921, EPI_ISL_711922, EPI_ISL_711923, EPI_ISL_711924, EPI_ISL_711925, EPI_ISL_711926, EPI_ISL_711927, EPI_ISL_711928, EPI_ISL_711929, EPI_ISL_711930, EPI_ISL_711931, EPI_ISL_711932, EPI_ISL_711933, EPI_ISL_711934, EPI_ISL_711935, EPI_ISL_711936, EPI_ISL_711937, EPI_ISL_711938, EPI_ISL_711939, EPI_ISL_711940, EPI_ISL_711943, EPI_ISL_711944, EPI_ISL_711945, EPI_ISL_711947, EPI_ISL_711948, EPI_ISL_711949, EPI_ISL_711950, EPI_ISL_711951, EPI_ISL_711952, EPI_ISL_711953, EPI_ISL_711954, EPI_ISL_711955, EPI_ISL_711956, EPI_ISL_711958, EPI_ISL_711959, EPI_ISL_711960, EPI_ISL_711961, EPI_ISL_711962, EPI_ISL_711963, EPI_ISL_711964, EPI_ISL_711965, EPI_ISL_711966, EPI_ISL_711967, EPI_ISL_711968, EPI_ISL_711969, EPI_ISL_711970, EPI_ISL_711971, EPI_ISL_711972, EPI_ISL_711973, EPI_ISL_711974, EPI_ISL_711975, EPI_ISL_711976, EPI_ISL_711979, EPI_ISL_711980, EPI_ISL_711981, EPI_ISL_711983, EPI_ISL_711984, EPI_ISL_711985, EPI_ISL_711986, EPI_ISL_711987, EPI_ISL_711988, EPI_ISL_711989, EPI_ISL_711991, EPI_ISL_711992, EPI_ISL_711993, EPI_ISL_711994, EPI_ISL_711995, EPI_ISL_711996, EPI_ISL_711997, EPI_ISL_711998, EPI_ISL_711999, EPI_ISL_712000, EPI_ISL_712001, EPI_ISL_712002, EPI_ISL_712003, EPI_ISL_712004, EPI_ISL_712006, EPI_ISL_712007, EPI_ISL_712008, EPI_ISL_712009, EPI_ISL_712010, EPI_ISL_712011, EPI_ISL_712012, EPI_ISL_712013, EPI_ISL_712014, EPI_ISL_712015, EPI_ISL_712016, EPI_ISL_712018, EPI_ISL_712020, EPI_ISL_712021, EPI_ISL_712022, EPI_ISL_712023, EPI_ISL_712024, EPI_ISL_712025, EPI_ISL_712026, EPI_ISL_712027, EPI_ISL_712028, EPI_ISL_712029, EPI_ISL_712030, EPI_ISL_712031, EPI_ISL_712032, EPI_ISL_712033, EPI_ISL_712034, EPI_ISL_712036, EPI_ISL_712038, EPI_ISL_712039, EPI_ISL_712040, EPI_ISL_712041, EPI_ISL_712042, EPI_ISL_712043, EPI_ISL_712044, EPI_ISL_712047, EPI_ISL_712048, EPI_ISL_712049, EPI_ISL_712051, EPI_ISL_712052, EPI_ISL_712053, EPI_ISL_712055, EPI_ISL_712056, EPI_ISL_712057, EPI_ISL_712058, EPI_ISL_712059, EPI_ISL_712249, EPI_ISL_712254, EPI_ISL_712255, EPI_ISL_712256, EPI_ISL_712257, EPI_ISL_712258, EPI_ISL_712259, EPI_ISL_712260, EPI_ISL_712261, EPI_ISL_712262, EPI_ISL_712263, EPI_ISL_712264, EPI_ISL_712265, EPI_ISL_712266, EPI_ISL_712267, EPI_ISL_712268, EPI_ISL_712269, EPI_ISL_712271, EPI_ISL_712272, EPI_ISL_712273, EPI_ISL_712274, EPI_ISL_712275, EPI_ISL_712276, EPI_ISL_712277, EPI_ISL_712278, EPI_ISL_712279, EPI_ISL_712280, EPI_ISL_712281, EPI_ISL_712282, EPI_ISL_712283, EPI_ISL_712284, EPI_ISL_712285, EPI_ISL_712286, EPI_ISL_712287, EPI_ISL_712288, EPI_ISL_712289, EPI_ISL_712290, EPI_ISL_712291, EPI_ISL_712292, EPI_ISL_712293, EPI_ISL_712294, EPI_ISL_712296, EPI_ISL_712297, EPI_ISL_712299, EPI_ISL_712300, EPI_ISL_712301, EPI_ISL_712302, EPI_ISL_712303, EPI_ISL_712304, EPI_ISL_712322, EPI_ISL_712323, EPI_ISL_712324, EPI_ISL_712325, EPI_ISL_712326, EPI_ISL_712327, EPI_ISL_712328, EPI_ISL_712329, EPI_ISL_712330, EPI_ISL_712331, EPI_ISL_712332, EPI_ISL_712333, EPI_ISL_712334, EPI_ISL_712335, EPI_ISL_712336, EPI_ISL_712347, EPI_ISL_712348, EPI_ISL_712349, EPI_ISL_712350, EPI_ISL_712351, EPI_ISL_712352, EPI_ISL_712353, EPI_ISL_712354, EPI_ISL_712355, EPI_ISL_712356, EPI_ISL_712357, EPI_ISL_712358, EPI_ISL_712359, EPI_ISL_712360, EPI_ISL_712361, EPI_ISL_712362, EPI_ISL_712363, EPI_ISL_712364, EPI_ISL_712366, EPI_ISL_712367, EPI_ISL_712368, EPI_ISL_712369, EPI_ISL_712370, EPI_ISL_712371, EPI_ISL_712372, EPI_ISL_712374, EPI_ISL_712375, EPI_ISL_712377, EPI_ISL_712378, EPI_ISL_712380, EPI_ISL_712381, EPI_ISL_712382, EPI_ISL_712383, EPI_ISL_712384, EPI_ISL_712385, EPI_ISL_712387, EPI_ISL_712388, EPI_ISL_712389, EPI_ISL_712390, EPI_ISL_712391, EPI_ISL_712392, EPI_ISL_712393, EPI_ISL_712394, EPI_ISL_712395, EPI_ISL_712396, EPI_ISL_712397, EPI_ISL_712398, EPI_ISL_712399, EPI_ISL_712400, EPI_ISL_712401, EPI_ISL_712402, EPI_ISL_712403, EPI_ISL_712404, EPI_ISL_712405, EPI_ISL_712407, EPI_ISL_712408, EPI_ISL_712409, EPI_ISL_712410, EPI_ISL_712411, EPI_ISL_712412, EPI_ISL_712413, EPI_ISL_712414, EPI_ISL_712415, EPI_ISL_712416, EPI_ISL_712420, EPI_ISL_712422, EPI_ISL_712423, EPI_ISL_712424, EPI_ISL_712425, EPI_ISL_712426, EPI_ISL_712531, EPI_ISL_712532, EPI_ISL_712533, EPI_ISL_712534, EPI_ISL_712535, EPI_ISL_712536, EPI_ISL_712537, EPI_ISL_712538, EPI_ISL_712539, EPI_ISL_712540, EPI_ISL_712541, EPI_ISL_712542, EPI_ISL_712543, EPI_ISL_712544, EPI_ISL_712545, EPI_ISL_712546, EPI_ISL_712547, EPI_ISL_712548, EPI_ISL_712549, EPI_ISL_712551, EPI_ISL_712552, EPI_ISL_712553, EPI_ISL_712554, EPI_ISL_712555, EPI_ISL_712556, EPI_ISL_712557, EPI_ISL_712558, EPI_ISL_712559, EPI_ISL_712560, EPI_ISL_712561, EPI_ISL_712562, EPI_ISL_712563, EPI_ISL_712564, EPI_ISL_712566, EPI_ISL_712567, EPI_ISL_712569, EPI_ISL_712570, EPI_ISL_712571, EPI_ISL_712572, EPI_ISL_712573, EPI_ISL_712574, EPI_ISL_712575, EPI_ISL_712578, EPI_ISL_712579, EPI_ISL_712580, EPI_ISL_712581, EPI_ISL_712582, EPI_ISL_712583, EPI_ISL_712584, EPI_ISL_712585, EPI_ISL_712586, EPI_ISL_712587, EPI_ISL_712588, EPI_ISL_712589, EPI_ISL_712590, EPI_ISL_712591, EPI_ISL_712592, EPI_ISL_712593, EPI_ISL_712594, EPI_ISL_712595, EPI_ISL_712596, EPI_ISL_712597, EPI_ISL_712598, EPI_ISL_712599, EPI_ISL_712601, EPI_ISL_712602, EPI_ISL_712603, EPI_ISL_712604, EPI_ISL_712605, EPI_ISL_712606, EPI_ISL_712607, EPI_ISL_712608, EPI_ISL_712609, EPI_ISL_712610, EPI_ISL_712611, EPI_ISL_712612, EPI_ISL_712613, EPI_ISL_712614, EPI_ISL_712615, EPI_ISL_712616, EPI_ISL_712617, EPI_ISL_712618, EPI_ISL_712619, EPI_ISL_712620, EPI_ISL_712621, EPI_ISL_712622, EPI_ISL_712623, EPI_ISL_712625, EPI_ISL_712626, EPI_ISL_712627, EPI_ISL_712629, EPI_ISL_712630, EPI_ISL_712631, EPI_ISL_712632, EPI_ISL_712633, EPI_ISL_712634, EPI_ISL_712635, EPI_ISL_712636, EPI_ISL_712637, EPI_ISL_712638,			

EPI_ISL_712639, EPI_ISL_712640, EPI_ISL_712641, EPI_ISL_712642, EPI_ISL_712643, EPI_ISL_712644, EPI_ISL_712645, EPI_ISL_712646, EPI_ISL_712647, EPI_ISL_712648, EPI_ISL_712649, EPI_ISL_712650, EPI_ISL_712651, EPI_ISL_712652, EPI_ISL_712653, EPI_ISL_712654, EPI_ISL_712655, EPI_ISL_712656,
EPI_ISL_712657, EPI_ISL_712658, EPI_ISL_712659, EPI_ISL_712660, EPI_ISL_712661, EPI_ISL_712662, EPI_ISL_712663, EPI_ISL_712664, EPI_ISL_712665, EPI_ISL_712666, EPI_ISL_712667, EPI_ISL_712669, EPI_ISL_712670, EPI_ISL_712671, EPI_ISL_712673, EPI_ISL_712674, EPI_ISL_712675, EPI_ISL_712676,
EPI_ISL_712677, EPI_ISL_712678, EPI_ISL_712679, EPI_ISL_712680, EPI_ISL_712681, EPI_ISL_712682, EPI_ISL_712683, EPI_ISL_712684, EPI_ISL_712685, EPI_ISL_712686, EPI_ISL_712687, EPI_ISL_712688, EPI_ISL_712689, EPI_ISL_712690, EPI_ISL_712692, EPI_ISL_712693, EPI_ISL_712694, EPI_ISL_712695,
EPI_ISL_712696, EPI_ISL_712697, EPI_ISL_712698, EPI_ISL_712699, EPI_ISL_712700, EPI_ISL_712701, EPI_ISL_712702, EPI_ISL_712703, EPI_ISL_712704, EPI_ISL_712705, EPI_ISL_712706, EPI_ISL_712707, EPI_ISL_712708, EPI_ISL_712709, EPI_ISL_712710, EPI_ISL_712711, EPI_ISL_712712, EPI_ISL_712713,
EPI_ISL_712715, EPI_ISL_712716, EPI_ISL_712717, EPI_ISL_712718, EPI_ISL_712719, EPI_ISL_712720, EPI_ISL_712721, EPI_ISL_712722, EPI_ISL_712723, EPI_ISL_712724, EPI_ISL_712725, EPI_ISL_712726, EPI_ISL_712727, EPI_ISL_712728, EPI_ISL_712729, EPI_ISL_712731, EPI_ISL_712732, EPI_ISL_712734,
EPI_ISL_712735, EPI_ISL_712736, EPI_ISL_712737, EPI_ISL_712738, EPI_ISL_712739, EPI_ISL_712740, EPI_ISL_712741, EPI_ISL_712743, EPI_ISL_712744, EPI_ISL_712745, EPI_ISL_712746, EPI_ISL_712747, EPI_ISL_712748, EPI_ISL_712749, EPI_ISL_712750, EPI_ISL_712751, EPI_ISL_712752, EPI_ISL_712753,
EPI_ISL_712754, EPI_ISL_712755, EPI_ISL_712756, EPI_ISL_712757, EPI_ISL_712758, EPI_ISL_712759, EPI_ISL_712760, EPI_ISL_712761, EPI_ISL_712762, EPI_ISL_712763, EPI_ISL_712764, EPI_ISL_712765, EPI_ISL_712766, EPI_ISL_712767, EPI_ISL_712768, EPI_ISL_712769, EPI_ISL_712770, EPI_ISL_712771,
EPI_ISL_712772, EPI_ISL_712773, EPI_ISL_712774, EPI_ISL_712775, EPI_ISL_712776, EPI_ISL_712777, EPI_ISL_712778, EPI_ISL_712779, EPI_ISL_712780, EPI_ISL_712781, EPI_ISL_712782, EPI_ISL_712784, EPI_ISL_712785, EPI_ISL_712786, EPI_ISL_712787, EPI_ISL_712788, EPI_ISL_712789, EPI_ISL_712790,
EPI_ISL_712791, EPI_ISL_712792, EPI_ISL_712793, EPI_ISL_712794, EPI_ISL_712795, EPI_ISL_712796, EPI_ISL_712797, EPI_ISL_712798, EPI_ISL_712799, EPI_ISL_712801, EPI_ISL_712802, EPI_ISL_712803, EPI_ISL_712804, EPI_ISL_712805, EPI_ISL_712807, EPI_ISL_712808, EPI_ISL_712809, EPI_ISL_712811,
EPI_ISL_712812, EPI_ISL_712813, EPI_ISL_712814, EPI_ISL_712815, EPI_ISL_712816, EPI_ISL_712817, EPI_ISL_712818, EPI_ISL_712819, EPI_ISL_712820, EPI_ISL_712823, EPI_ISL_712824, EPI_ISL_712825, EPI_ISL_712826, EPI_ISL_712827, EPI_ISL_712828, EPI_ISL_712829, EPI_ISL_712830, EPI_ISL_712831,
EPI_ISL_712832, EPI_ISL_712833, EPI_ISL_712834, EPI_ISL_712835, EPI_ISL_712836, EPI_ISL_712837, EPI_ISL_712838, EPI_ISL_712839, EPI_ISL_712840, EPI_ISL_712841, EPI_ISL_712842, EPI_ISL_712843, EPI_ISL_712844, EPI_ISL_712845, EPI_ISL_712846, EPI_ISL_712847, EPI_ISL_712848, EPI_ISL_712849,
EPI_ISL_712850, EPI_ISL_712851, EPI_ISL_712852, EPI_ISL_712853, EPI_ISL_712854, EPI_ISL_712855, EPI_ISL_712856, EPI_ISL_712857, EPI_ISL_712858, EPI_ISL_712859, EPI_ISL_712860, EPI_ISL_712861, EPI_ISL_712862, EPI_ISL_712863, EPI_ISL_712864, EPI_ISL_712865, EPI_ISL_712866, EPI_ISL_712867,
EPI_ISL_712868, EPI_ISL_712869, EPI_ISL_712870, EPI_ISL_712871, EPI_ISL_712872, EPI_ISL_712873, EPI_ISL_712874, EPI_ISL_712875, EPI_ISL_712876, EPI_ISL_712877, EPI_ISL_712878, EPI_ISL_712879, EPI_ISL_712880, EPI_ISL_712881, EPI_ISL_712882, EPI_ISL_712883, EPI_ISL_712884, EPI_ISL_712885,
EPI_ISL_712886, EPI_ISL_712887, EPI_ISL_712888, EPI_ISL_712889, EPI_ISL_712890, EPI_ISL_712891, EPI_ISL_712892, EPI_ISL_712893, EPI_ISL_712894, EPI_ISL_712895, EPI_ISL_712896, EPI_ISL_712897, EPI_ISL_712899, EPI_ISL_712900, EPI_ISL_712901, EPI_ISL_712902, EPI_ISL_712903, EPI_ISL_712904,
EPI_ISL_712905, EPI_ISL_712906, EPI_ISL_712907, EPI_ISL_712908, EPI_ISL_712909, EPI_ISL_712910, EPI_ISL_712911, EPI_ISL_712912, EPI_ISL_712913, EPI_ISL_712914, EPI_ISL_712915, EPI_ISL_712916, EPI_ISL_712917, EPI_ISL_712918, EPI_ISL_712919, EPI_ISL_712920, EPI_ISL_712922, EPI_ISL_712923,
EPI_ISL_712924, EPI_ISL_712925, EPI_ISL_712926, EPI_ISL_712927, EPI_ISL_712928, EPI_ISL_712929, EPI_ISL_712930, EPI_ISL_712931, EPI_ISL_712932, EPI_ISL_712933, EPI_ISL_712934, EPI_ISL_712935, EPI_ISL_712936, EPI_ISL_712937, EPI_ISL_712938, EPI_ISL_712939, EPI_ISL_712940, EPI_ISL_712941,
EPI_ISL_712942, EPI_ISL_712943, EPI_ISL_712944, EPI_ISL_712945, EPI_ISL_712946, EPI_ISL_712947, EPI_ISL_712948, EPI_ISL_712949, EPI_ISL_712950, EPI_ISL_712951, EPI_ISL_712953, EPI_ISL_712954, EPI_ISL_712955, EPI_ISL_712956, EPI_ISL_712957, EPI_ISL_712958, EPI_ISL_712959, EPI_ISL_712960,
EPI_ISL_712961, EPI_ISL_712962, EPI_ISL_712964, EPI_ISL_712965, EPI_ISL_712966, EPI_ISL_712967, EPI_ISL_712968, EPI_ISL_712969, EPI_ISL_712970, EPI_ISL_712971, EPI_ISL_712972, EPI_ISL_712974, EPI_ISL_712975, EPI_ISL_712976, EPI_ISL_712977, EPI_ISL_712978, EPI_ISL_712979, EPI_ISL_712980,
EPI_ISL_712981, EPI_ISL_712982, EPI_ISL_712983, EPI_ISL_712984, EPI_ISL_712985, EPI_ISL_712986, EPI_ISL_712987, EPI_ISL_712988, EPI_ISL_712989, EPI_ISL_712990, EPI_ISL_712991, EPI_ISL_712992, EPI_ISL_712993, EPI_ISL_712994, EPI_ISL_712995, EPI_ISL_712996, EPI_ISL_712997, EPI_ISL_712998,
EPI_ISL_712999, EPI_ISL_713000, EPI_ISL_713001, EPI_ISL_713002, EPI_ISL_713003, EPI_ISL_713004, EPI_ISL_713005, EPI_ISL_713006, EPI_ISL_713007, EPI_ISL_713008, EPI_ISL_713009, EPI_ISL_713010, EPI_ISL_713011, EPI_ISL_713012, EPI_ISL_713013, EPI_ISL_713014, EPI_ISL_713015, EPI_ISL_713016,
EPI_ISL_713017, EPI_ISL_713018, EPI_ISL_713019, EPI_ISL_713020, EPI_ISL_713021, EPI_ISL_713022, EPI_ISL_713023, EPI_ISL_713024, EPI_ISL_713025, EPI_ISL_713026, EPI_ISL_713027, EPI_ISL_713028, EPI_ISL_713029, EPI_ISL_713030, EPI_ISL_713031, EPI_ISL_713032, EPI_ISL_713033, EPI_ISL_713034,
EPI_ISL_713035, EPI_ISL_713036, EPI_ISL_713037, EPI_ISL_713038, EPI_ISL_713039, EPI_ISL_713040, EPI_ISL_713041, EPI_ISL_713042, EPI_ISL_713043, EPI_ISL_713045, EPI_ISL_713046, EPI_ISL_713047, EPI_ISL_713049, EPI_ISL_713050, EPI_ISL_713051, EPI_ISL_713052, EPI_ISL_713053, EPI_ISL_713054,
EPI_ISL_713055, EPI_ISL_713056, EPI_ISL_713057, EPI_ISL_713058, EPI_ISL_713059, EPI_ISL_713060, EPI_ISL_713061, EPI_ISL_713062, EPI_ISL_713063, EPI_ISL_713064, EPI_ISL_713065, EPI_ISL_713066, EPI_ISL_713067, EPI_ISL_713068, EPI_ISL_713069, EPI_ISL_713070, EPI_ISL_713071, EPI_ISL_713072,
EPI_ISL_713073, EPI_ISL_713074, EPI_ISL_713075, EPI_ISL_713076, EPI_ISL_713077, EPI_ISL_713078, EPI_ISL_713079, EPI_ISL_713081, EPI_ISL_713082, EPI_ISL_713083, EPI_ISL_713084, EPI_ISL_713085, EPI_ISL_713086, EPI_ISL_713087, EPI_ISL_713088, EPI_ISL_713089, EPI_ISL_713090, EPI_ISL_713091,
EPI_ISL_713092, EPI_ISL_713093, EPI_ISL_713095, EPI_ISL_713096, EPI_ISL_713097, EPI_ISL_713269, EPI_ISL_713270, EPI_ISL_713271, EPI_ISL_713272, EPI_ISL_713273, EPI_ISL_713274, EPI_ISL_713275, EPI_ISL_713276, EPI_ISL_713277, EPI_ISL_713278, EPI_ISL_713279, EPI_ISL_713280, EPI_ISL_713281,
EPI_ISL_713282, EPI_ISL_713283, EPI_ISL_713284, EPI_ISL_713285, EPI_ISL_713286, EPI_ISL_713287, EPI_ISL_713288, EPI_ISL_713289, EPI_ISL_713291, EPI_ISL_713292, EPI_ISL_713293, EPI_ISL_713294, EPI_ISL_713315, EPI_ISL_713317, EPI_ISL_713318, EPI_ISL_713319, EPI_ISL_713320, EPI_ISL_713321,
EPI_ISL_713322, EPI_ISL_713323, EPI_ISL_713324, EPI_ISL_713325, EPI_ISL_713326, EPI_ISL_713327, EPI_ISL_713332, EPI_ISL_713333, EPI_ISL_713334, EPI_ISL_713335, EPI_ISL_713336, EPI_ISL_713337, EPI_ISL_713338, EPI_ISL_713339, EPI_ISL_713340, EPI_ISL_713341, EPI_ISL_713342, EPI_ISL_713343,
EPI_ISL_713344, EPI_ISL_713345, EPI_ISL_713347, EPI_ISL_713348, EPI_ISL_713350, EPI_ISL_713351, EPI_ISL_713352, EPI_ISL_713353, EPI_ISL_713354, EPI_ISL_713355, EPI_ISL_713356, EPI_ISL_713357, EPI_ISL_713358, EPI_ISL_713360, EPI_ISL_713361, EPI_ISL_713362, EPI_ISL_713363, EPI_ISL_713364,
EPI_ISL_713365, EPI_ISL_713366, EPI_ISL_713367, EPI_ISL_713368, EPI_ISL_713369, EPI_ISL_713370, EPI_ISL_713371, EPI_ISL_713372, EPI_ISL_713373, EPI_ISL_713375, EPI_ISL_713376, EPI_ISL_713377, EPI_ISL_713378, EPI_ISL_713379, EPI_ISL_713380, EPI_ISL_713381, EPI_ISL_713383, EPI_ISL_713384,
EPI_ISL_713385, EPI_ISL_713386, EPI_ISL_713387, EPI_ISL_713388, EPI_ISL_713389, EPI_ISL_713390, EPI_ISL_713391, EPI_ISL_713392, EPI_ISL_713393, EPI_ISL_713394, EPI_ISL_713395, EPI_ISL_713396, EPI_ISL_713397, EPI_ISL_713398, EPI_ISL_713399, EPI_ISL_713400, EPI_ISL_713401, EPI_ISL_713402,
EPI_ISL_713403, EPI_ISL_713405, EPI_ISL_713407, EPI_ISL_713408, EPI_ISL_713409, EPI_ISL_713410, EPI_ISL_713411, EPI_ISL_713412, EPI_ISL_713414, EPI_ISL_713415, EPI_ISL_713416, EPI_ISL_713417, EPI_ISL_713418, EPI_ISL_713419, EPI_ISL_713420, EPI_ISL_713421, EPI_ISL_713422, EPI_ISL_713425,
EPI_ISL_713426, EPI_ISL_713427, EPI_ISL_713428, EPI_ISL_713429, EPI_ISL_713513, EPI_ISL_713514, EPI_ISL_713515, EPI_ISL_713516, EPI_ISL_713517, EPI_ISL_713518, EPI_ISL_713519, EPI_ISL_713520, EPI_ISL_713521, EPI_ISL_713522, EPI_ISL_713524, EPI_ISL_713525, EPI_ISL_713526, EPI_ISL_713528,
EPI_ISL_713529, EPI_ISL_713530, EPI_ISL_713531, EPI_ISL_713532, EPI_ISL_713533, EPI_ISL_713534, EPI_ISL_713535, EPI_ISL_713536, EPI_ISL_713537, EPI_ISL_713539, EPI_ISL_713540, EPI_ISL_713541, EPI_ISL_713542, EPI_ISL_713543, EPI_ISL_713544, EPI_ISL_713545, EPI_ISL_713546, EPI_ISL_713547,
EPI_ISL_713548, EPI_ISL_713549, EPI_ISL_713550, EPI_ISL_713551, EPI_ISL_713552, EPI_ISL_713553, EPI_ISL_713554, EPI_ISL_713555, EPI_ISL_713556, EPI_ISL_713557, EPI_ISL_713558, EPI_ISL_713559, EPI_ISL_713561, EPI_ISL_713562, EPI_ISL_713563, EPI_ISL_713565, EPI_ISL_713566, EPI_ISL_713567,
EPI_ISL_713568, EPI_ISL_713569, EPI_ISL_713570, EPI_ISL_713573, EPI_ISL_713574, EPI_ISL_713575, EPI_ISL_713576, EPI_ISL_713577, EPI_ISL_713578, EPI_ISL_713579, EPI_ISL_713580, EPI_ISL_713581, EPI_ISL_713582, EPI_ISL_713583, EPI_ISL_713585, EPI_ISL_713586, EPI_ISL_713588, EPI_ISL_713589,
EPI_ISL_713590, EPI_ISL_713591, EPI_ISL_713592, EPI_ISL_713593, EPI_ISL_713594, EPI_ISL_713595, EPI_ISL_713596, EPI_ISL_713597, EPI_ISL_713598, EPI_ISL_713599, EPI_ISL_713600, EPI_ISL_713601, EPI_ISL_713602, EPI_ISL_713603, EPI_ISL_713604, EPI_ISL_713606, EPI_ISL_713607, EPI_ISL_713608,
EPI_ISL_713609, EPI_ISL_713610, EPI_ISL_713611, EPI_ISL_713612, EPI_ISL_713613, EPI_ISL_713614, EPI_ISL_713616, EPI_ISL_713617, EPI_ISL_713618, EPI_ISL_713619, EPI_ISL_713620, EPI_ISL_713621, EPI_ISL_713622, EPI_ISL_713624, EPI_ISL_713625, EPI_ISL_713626, EPI_ISL_713627, EPI_ISL_713628,
EPI_ISL_713630, EPI_ISL_713631, EPI_ISL_713632, EPI_ISL_713633, EPI_ISL_713634, EPI_ISL_713635, EPI_ISL_713636, EPI_ISL_713637, EPI_ISL_713638, EPI_ISL_713639, EPI_ISL_713640, EPI_ISL_713641, EPI_ISL_713642, EPI_ISL_713643, EPI_ISL_713645, EPI_ISL_713646, EPI_ISL_713647, EPI_ISL_713648,
EPI_ISL_713649, EPI_ISL_713650, EPI_ISL_713651, EPI_ISL_713652, EPI_ISL_713653, EPI_ISL_713654, EPI_ISL_713655, EPI_ISL_713656, EPI_ISL_713657, EPI_ISL_713658, EPI_ISL_713659, EPI_ISL_713660, EPI_ISL_713661, EPI_ISL_713662, EPI_ISL_713663, EPI_ISL_713664, EPI_ISL_713665, EPI_ISL_713667,
EPI_ISL_713668, EPI_ISL_713669, EPI_ISL_713670, EPI_ISL_713671, EPI_ISL_713672, EPI_ISL_713673, EPI_ISL_713674, EPI_ISL_713675, EPI_ISL_713676, EPI_ISL_713677, EPI_ISL_713678, EPI_ISL_713679, EPI_ISL_713680, EPI_ISL_713682, EPI_ISL_713683, EPI_ISL_713684, EPI_ISL_713685, EPI_ISL_713686,
EPI_ISL_713687, EPI_ISL_713688, EPI_ISL_713689, EPI_ISL_713691, EPI_ISL_713692, EPI_ISL_713693, EPI_ISL_713694, EPI_ISL_713695, EPI_ISL_713696, EPI_ISL_713697, EPI_ISL_713698, EPI_ISL_713699, EPI_ISL_713700, EPI_ISL_713701, EPI_ISL_713702, EPI_ISL_713703, EPI_ISL_713704, EPI_ISL_713705,
EPI_ISL_713706, EPI_ISL_713707, EPI_ISL_713708, EPI_ISL_713709, EPI_ISL_713710, EPI_ISL_713711, EPI_ISL_713712, EPI_ISL_713713, EPI_ISL_713714, EPI_ISL_713715, EPI_ISL_713716, EPI_ISL_713717, EPI_ISL_713718, EPI_ISL_713719, EPI_ISL_713720, EPI_ISL_713721, EPI_ISL_713722, EPI_ISL_713723,
EPI_ISL_713724, EPI_ISL_713725, EPI_ISL_713726, EPI_ISL_713727, EPI_ISL_713729, EPI_ISL_713730, EPI_ISL_713731, EPI_ISL_713732, EPI_ISL_713733, EPI_ISL_713734, EPI_ISL_713735, EPI_ISL_713736, EPI_ISL_713737, EPI_ISL_713738, EPI_ISL_713739, EPI_ISL_713740, EPI_ISL_713743, EPI_ISL_713744,
EPI_ISL_713745, EPI_ISL_713746, EPI_ISL_713747, EPI_ISL_713748, EPI_ISL_713749, EPI_ISL_713750, EPI_ISL_713751, EPI_ISL_713752, EPI_ISL_713753, EPI_ISL_713754, EPI_ISL_713755, EPI_ISL_713756, EPI_ISL_713757, EPI_ISL_713758, EPI_ISL_713759, EPI_ISL_713760, EPI_ISL_713761, EPI_ISL_713762,
EPI_ISL_713763, EPI_ISL_713764, EPI_ISL_713765, EPI_ISL_713766, EPI_ISL_713767, EPI_ISL_713768, EPI_ISL_713769, EPI_ISL_713771, EPI_ISL_713772, EPI_ISL_713773, EPI_ISL_713775, EPI_ISL_713776, EPI_ISL_713777, EPI_ISL_713778, EPI_ISL_713779, EPI_ISL_713780, EPI_ISL_713781, EPI_ISL_713782,
EPI_ISL_713783, EPI_ISL_713784, EPI_ISL_713785, EPI_ISL_713786, EPI_ISL_713787, EPI_ISL_713788, EPI_ISL_713790, EPI_ISL_713791, EPI_ISL_713793, EPI_ISL_713794, EPI_ISL_713795, EPI_ISL_713796, EPI_ISL_713797, EPI_ISL_713798, EPI_ISL_713799, EPI_ISL_713800, EPI_ISL_713801, EPI_ISL_713802,
EPI_ISL_713803, EPI_ISL_713804, EPI_ISL_713805, EPI_ISL_713806, EPI_ISL_713807, EPI_ISL_713808, EPI_ISL_713809, EPI_ISL_713810, EPI_ISL_713811, EPI_ISL_713812, EPI_ISL_713813, EPI_ISL_713814, EPI_ISL_713815, EPI_ISL_713816, EPI_ISL_713817, EPI_ISL_713818, EPI_ISL_713819, EPI_ISL_713820,
EPI_ISL_713822, EPI_ISL_713823, EPI_ISL_713824, EPI_ISL_713825, EPI_ISL_713826, EPI_ISL_713828, EPI_ISL_713829, EPI_ISL_713830, EPI_ISL_713831, EPI_ISL_713832, EPI_ISL_713833, EPI_ISL_713834, EPI_ISL_713835, EPI_ISL_713836, EPI_ISL_713837, EPI_ISL_713838, EPI_ISL_713839, EPI_ISL_713840,
EPI_ISL_713841, EPI_ISL_713842, EPI_ISL_713843, EPI_ISL_713844, EPI_ISL_713847, EPI_ISL_713848, EPI_ISL_713849, EPI_ISL_713850, EPI_ISL_713851, EPI_ISL_713852, EPI_ISL_713854, EPI_ISL_713855, EPI_ISL_713856, EPI_ISL_713857, EPI_ISL_713858, EPI_ISL_713859, EPI_ISL_713860, EPI_ISL_713861,
EPI_ISL_713862, EPI_ISL_713863, EPI_ISL_713864, EPI_ISL_713865, EPI_ISL_713866, EPI_ISL_713867, EPI_ISL_713869, EPI_ISL_713870, EPI_ISL_713871, EPI_ISL_713874, EPI_ISL_713875, EPI_ISL_713876, EPI_ISL_713877, EPI_ISL_713879, EPI_ISL_713880, EPI_ISL_713881, EPI_ISL_713882, EPI_ISL_713883,
EPI_ISL_713884, EPI_ISL_713885, EPI_ISL_713887, EPI_ISL_713888, EPI_ISL_713889, EPI_ISL_713890, EPI_ISL_713892, EPI_ISL_713893, EPI_ISL_713894, EPI_ISL_713895, EPI_ISL_713896, EPI_ISL_713897, EPI_ISL_713898, EPI_ISL_713899, EPI_ISL_713900, EPI_ISL_713901, EPI_ISL_713902, EPI_ISL_713903,
EPI_ISL_713904, EPI_ISL_713905, EPI_ISL_713906, EPI_ISL_713907, EPI_ISL_713908, EPI_ISL_713909, EPI_ISL_713910, EPI_ISL_713911, EPI_ISL_713912, EPI_ISL_713913, EPI_ISL_713914, EPI_ISL_713915, EPI_ISL_713916, EPI_ISL_713917, EPI_ISL_713918, EPI_ISL_713919, EPI_ISL_713920, EPI_ISL_713921,
EPI_ISL_713922, EPI_ISL_713924, EPI_ISL_713925, EPI_ISL_713926, EPI_ISL_713927, EPI_ISL_713928, EPI_ISL_713929, EPI_ISL_713930, EPI_ISL_713932, EPI_ISL_713933, EPI_ISL_713934, EPI_ISL_713935, EPI_ISL_713936, EPI_ISL_713937, EPI_ISL_713938, EPI_ISL_713940, EPI_ISL_713941, EPI_ISL_713942,
EPI_ISL_713943, EPI_ISL_713944, EPI_ISL_713945, EPI_ISL_713946, EPI_ISL_713948, EPI_ISL_713950, EPI_ISL_713951, EPI_ISL_713952, EPI_ISL_713953, EPI_ISL_713954, EPI_ISL_713955, EPI_ISL_713956, EPI_ISL_713957, EPI_ISL_713958, EPI_ISL_713959, EPI_ISL_713960, EPI_ISL_713961, EPI_ISL_713962,
EPI_ISL_713963, EPI_ISL_713964, EPI_ISL_713965, EPI_ISL_713966, EPI_ISL_713967, EPI_ISL_713969, EPI_ISL_713970, EPI_ISL_713971, EPI_ISL_713972, EPI_ISL_713973, EPI_ISL_713974, EPI_ISL_713975, EPI_ISL_713976, EPI_ISL_713978, EPI_ISL_713979, EPI_ISL_713980, EPI_ISL_713983, EPI_ISL_713985,
EPI_ISL_713986, EPI_ISL_713987, EPI_ISL_713988, EPI_ISL_713989, EPI_ISL_713990, EPI_ISL_713991, EPI_ISL_713992, EPI_ISL_713994, EPI_ISL_713995, EPI_ISL_713996, EPI_ISL_713997, EPI_ISL_713999, EPI_ISL_714000, EPI_ISL_714001, EPI_ISL_714002, EPI_ISL_714003, EPI_ISL_714005, EPI_ISL_714006,
EPI_ISL_714007, EPI_ISL_714008, EPI_ISL_714009, EPI_ISL_714010, EPI_ISL_714011, EPI_ISL_714013, EPI_ISL_714014, EPI_ISL_714015, EPI_ISL_714016, EPI_ISL_714017, EPI_ISL_714018, EPI_ISL_714019, EPI_ISL_714020, EPI_ISL_714022, EPI_ISL_714023, EPI_ISL_714024, EPI_ISL_714025, EPI_ISL_714026,
EPI_ISL_714027, EPI_ISL_714028, EPI_ISL_714029, EPI_ISL_714031, EPI_ISL_714032, EPI_ISL_714033, EPI_ISL_714034, EPI_ISL_714035, EPI_ISL_714037, EPI_ISL_714038, EPI_ISL_714039, EPI_ISL_714040, EPI_ISL_714042, EPI_ISL_714043, EPI_ISL_714044, EPI_ISL_714045, EPI_ISL_714046, EPI_ISL_714047,
EPI_ISL_714048, EPI_ISL_714050, EPI_ISL_714051, EPI_ISL_714052, EPI_ISL_714053, EPI_ISL_714054, EPI_ISL_714055, EPI_ISL_714056, EPI_ISL_714057, EPI_ISL_714058, EPI_ISL_714059, EPI_ISL_714060, EPI_ISL_714061, EPI_ISL_714062, EPI_ISL_714063, EPI_ISL_714064, EPI_ISL_714065, EPI_ISL_714066,
EPI_ISL_714067, EPI_ISL_714068, EPI_ISL_714069, EPI_ISL_714070, EPI_ISL_714071, EPI_ISL_714074, EPI_ISL_714075, EPI_ISL_714077, EPI_ISL_714078, EPI_ISL_714079, EPI_ISL_714080, EPI_ISL_714081, EPI_ISL_714082, EPI_ISL_714083, EPI_ISL_714084, EPI_ISL_714085, EPI_ISL_714086, EPI_ISL_714087,
EPI_ISL_714088, EPI_ISL_714089, EPI_ISL_714090, EPI_ISL_714091, EPI_ISL_714093, EPI_ISL_714094, EPI_ISL_714095, EPI_ISL_714096, EPI_ISL_714097, EPI_ISL_714259, EPI_ISL_714260, EPI_ISL_714261, EPI_ISL_714262, EPI_ISL_714263, EPI_ISL_714264, EPI_ISL_714266, EPI_ISL_714267, EPI_ISL_714268,
EPI_ISL_714270, EPI_ISL_714271, EPI_ISL_714291, EPI_ISL_714292, EPI_ISL_714294, EPI_ISL_714295, EPI_ISL_714296, EPI_ISL_714297, EPI_ISL_714298, EPI_ISL_714300, EPI_ISL_714301, EPI_ISL_714302, EPI_ISL_714303, EPI_ISL_714304, EPI_ISL_714305, EPI_ISL_714306, EPI_ISL_714307, EPI_ISL_714308,
EPI_ISL_714310, EPI_ISL_714311, EPI_ISL_714312, EPI_ISL_714313, EPI_ISL_714314, EPI_ISL_714315, EPI_ISL_714316, EPI_ISL_714317, EPI_ISL_714319, EPI_ISL_714321, EPI_ISL_714322, EPI_ISL_714323, EPI_ISL_714324, EPI_ISL_714326, EPI_ISL_714327, EPI_ISL_714328, EPI_ISL_714329, EPI_ISL_714330,
EPI_ISL_714331, EPI_ISL_714332, EPI_ISL_714333, EPI_ISL_714334, EPI_ISL_714335, EPI_ISL_714337, EPI_ISL_714338, EPI_ISL_714339, EPI_ISL_714340, EPI_ISL_714341, EPI_ISL_714342, EPI_ISL_714343, EPI_ISL_714344, EPI_ISL_714345, EPI_ISL_714347, EPI_ISL_714348, EPI_ISL_714349, EPI_ISL_714350,
EPI_ISL_714351, EPI_ISL_714352, EPI_ISL_714353, EPI_ISL_714354, EPI_ISL_714355, EPI_ISL_714356, EPI_ISL_714357, EPI_ISL_714358, EPI_ISL_714359, EPI_ISL_714360, EPI_ISL_714361, EPI_ISL_714362, EPI_ISL_714372, EPI_ISL_714373, EPI_ISL_714374, EPI_ISL_714375, EPI_ISL_714376, EPI_ISL_714379,
EPI_ISL_714380, EPI_ISL_714478, EPI_ISL_714479, EPI_ISL_714481, EPI_ISL_714482, EPI_ISL_714483, EPI_ISL_714484, EPI_ISL_714485, EPI_ISL_714486, EPI_ISL_714487, EPI_ISL_714488, EPI_ISL_714489, EPI_ISL_714490, EPI_ISL_714491, EPI_ISL_714492, EPI_ISL_714493, EPI_ISL_714495, EPI_ISL_714496,
EPI_ISL_714497, EPI_ISL_714498, EPI_ISL_714499, EPI_ISL_714500, EPI_ISL_714501, EPI_ISL_714502, EPI_ISL_714503, EPI_ISL_714504, EPI_ISL_714505, EPI_ISL_714506, EPI_ISL_714508, EPI_ISL_714509, EPI_ISL_714510, EPI_ISL_714511, EPI_ISL_714512, EPI_ISL_714513, EPI_ISL_714514, EPI_ISL_714515,
EPI_ISL_714516, EPI_ISL_714517, EPI_ISL_714518, EPI_ISL_714519, EPI_ISL_714520, EPI_ISL_714521, EPI_ISL_714522, EPI_ISL_714523, EPI_ISL_714524, EPI_ISL_714525, EPI_ISL_714526, EPI_ISL_714527, EPI_ISL_714528, EPI_ISL_714529, EPI_ISL_714530, EPI_ISL_714531, EPI_ISL_714532, EPI_ISL_714534,
EPI_ISL_714535, EPI_ISL_714536, EPI_ISL_714537, EPI_ISL_714538, EPI_ISL_714539, EPI_ISL_714540, EPI_ISL_714541, EPI_ISL_714542, EPI_ISL_714543, EPI_ISL_714544, EPI_ISL_714545, EPI_ISL_714546, EPI_ISL_714547, EPI_ISL_714548, EPI_ISL_714549, EPI_ISL_714550, EPI_ISL_714551, EPI_ISL_714552,
EPI_ISL_714553, EPI_ISL_714554, EPI_ISL_714555, EPI_ISL_714556, EPI_ISL_714557, EPI_ISL_714558, EPI_ISL_714559, EPI_ISL_714560, EPI_ISL_714561, EPI_ISL_714562, EPI_ISL_714563, EPI_ISL_714564, EPI_ISL_714566, EPI_ISL_714567, EPI_ISL_714568, EPI_ISL_714569, EPI_ISL_714570, EPI_ISL_714571,
EPI_ISL_714572, EPI_ISL_714573, EPI_ISL_714574, EPI_ISL_714576, EPI_ISL_714577, EPI_ISL_714578, EPI_ISL_714579, EPI_ISL_714580, EPI_ISL_714581, EPI_ISL_714582, EPI_ISL_714583, EPI_ISL_714584, EPI_ISL_714585, EPI_ISL_714586, EPI_ISL_714587, EPI_ISL_714588, EPI_ISL_714589, EPI_ISL_714590,
EPI_ISL_714591, EPI_ISL_714592, EPI_ISL_714593, EPI_ISL_714594, EPI_ISL_714595, EPI_ISL_714596, EPI_ISL_714597, EPI_ISL_714599, EPI_ISL_714600, EPI_ISL_714601, EPI_ISL_714602, EPI_ISL_714603, EPI_ISL_714604, EPI_ISL_714605, EPI_ISL_714606, EPI_ISL_714607, EPI_ISL_714608, EPI_ISL_714609,
EPI_ISL_714611, EPI_ISL_714612, EPI_ISL_714613, EPI_ISL_714614, EPI_ISL_714615, EPI_ISL_714616, EPI_ISL_714617, EPI_ISL_714618, EPI_ISL_714619, EPI_ISL_714620, EPI_ISL_714621, EPI_ISL_714622, EPI_ISL_714623, EPI_ISL_714624, EPI_ISL_714625, EPI_ISL_714626, EPI_ISL_714627, EPI_ISL_714628,
EPI_ISL_714629, EPI_ISL_714630, EPI_ISL_714631, EPI_ISL_714632, EPI_ISL_714633, EPI_ISL_714634, EPI_ISL_714635, EPI_ISL_714636, EPI_ISL_714637, EPI_ISL_714638, EPI_ISL_714640, EPI_ISL_714641, EPI_ISL_714642, EPI_ISL_714643, EPI_ISL_714644, EPI_ISL_714645, EPI_ISL_714646, EPI_ISL_714647,
EPI_ISL_714648, EPI_ISL_714649, EPI_ISL_714650, EPI_ISL_714651, EPI_ISL_714652, EPI_ISL_714653, EPI_ISL_714654, EPI_ISL_714655, EPI_ISL_714656, EPI_ISL_714657, EPI_ISL_714658, EPI_ISL_714659, EPI_ISL_714660, EPI_ISL_714661, EPI_ISL_714662, EPI_ISL_714663, EPI_ISL_714664, EPI_ISL_714665,
EPI_ISL_714670, EPI_ISL_714671, EPI_ISL_714672, EPI_ISL_714673, EPI_ISL_714674, EPI_ISL_714675, EPI_ISL_714676, EPI_ISL_714677, EPI_ISL_714678, EPI_ISL_714679, EPI_ISL_714680, EPI_ISL_714681, EPI_ISL_714682, EPI_ISL_714683, EPI_ISL_714684, EPI_ISL_714685, EPI_ISL_714686, EPI_ISL_714688,
EPI_ISL_714689, EPI_ISL_714690, EPI_ISL_714691, EPI_ISL_714692, EPI_ISL_714693, EPI_ISL_714694, EPI_ISL_714695, EPI_ISL_714696, EPI_ISL_714697, EPI_ISL_714698, EPI_ISL_714699, EPI_ISL_714700, EPI_ISL_714701, EPI_ISL_714702, EPI_ISL_714703, EPI_ISL_714704, EPI_ISL_714705, EPI_ISL_714706,
EPI_ISL_714707, EPI_ISL_714708, EPI_ISL_714709, EPI_ISL_714710, EPI_ISL_714711, EPI_ISL_714712, EPI_ISL_714713, EPI_ISL_714715, EPI_ISL_714716, EPI_ISL_714717, EPI_ISL_714718, EPI_ISL_714719, EPI_ISL_714721, EPI_ISL_714722, EPI_ISL_714723, EPI_ISL_714724, EPI_ISL_714725, EPI_ISL_714726,
EPI_ISL_714727, EPI_ISL_714728, EPI_ISL_714729, EPI_ISL_714731, EPI_ISL_714732, EPI_ISL_714733, EPI_ISL_714734, EPI_ISL_714735, EPI_ISL_714736, EPI_ISL_714737, EPI_ISL_714738, EPI_ISL_714739, EPI_ISL_714740, EPI_ISL_714741, EPI_ISL_714742, EPI_ISL_714743, EPI_ISL_714744, EPI_ISL_714745,
EPI_ISL_714746, EPI_ISL_714747, EPI_ISL_714748, EPI_ISL_714749, EPI_ISL_714750, EPI_ISL_714751, EPI_ISL_714752, EPI_ISL_714753, EPI_ISL_714754, EPI_ISL_714757, EPI_ISL_714758, EPI_ISL_714759, EPI_ISL_714760, EPI_ISL_714761, EPI_ISL_714762, EPI_ISL_714763, EPI_ISL_714764, EPI_ISL_714765,
EPI_ISL_714766, EPI_ISL_714768, EPI_ISL_714769, EPI_ISL_714771, EPI_ISL_714772, EPI_ISL_714773, EPI_ISL_714774, EPI_ISL_714775, EPI_ISL_714776, EPI_ISL_714777, EPI_ISL_714778, EPI_ISL_714780, EPI_ISL_714781, EPI_ISL_714782, EPI_ISL_714784, EPI_ISL_714785, EPI_ISL_714786, EPI_ISL_714787,
EPI_ISL_714788, EPI_ISL_714789, EPI_ISL_714790, EPI_ISL_714791, EPI_ISL_714793, EPI_ISL_714794, EPI_ISL_714795, EPI_ISL_714796, EPI_ISL_714797, EPI_ISL_714798, EPI_ISL_714799, EPI_ISL_714801, EPI_ISL_714802, EPI_ISL_714803, EPI_ISL_714804, EPI_ISL_714805, EPI_ISL_714806, EPI_ISL_714807,
EPI_ISL_714809, EPI_ISL_714810, EPI_ISL_714811, EPI_ISL_714812, EPI_ISL_714813, EPI_ISL_714817, EPI_ISL_714818, EPI_ISL_714819, EPI_ISL_714820, EPI_ISL_714821, EPI_ISL_714822, EPI_ISL_714823, EPI_ISL_714824, EPI_ISL_714825, EPI_ISL_714826, EPI_ISL_714827, EPI_ISL_714828, EPI_ISL_714829,
EPI_ISL_714830, EPI_ISL_714831, EPI_ISL_714833, EPI_ISL_714834, EPI_ISL_714837, EPI_ISL_714838, EPI_ISL_714839, EPI_ISL_714840, EPI_ISL_714841, EPI_ISL_714842, EPI_ISL_714843, EPI_ISL_714844, EPI_ISL_714845, EPI_ISL_714846, EPI_ISL_714848, EPI_ISL_714849, EPI_ISL_714850, EPI_ISL_714851,
EPI_ISL_714852, EPI_ISL_714853, EPI_ISL_714854, EPI_ISL_714855, EPI_ISL_714856, EPI_ISL_714857, EPI_ISL_714858, EPI_ISL_714859, EPI_ISL_714860, EPI_ISL_714861, EPI_ISL_714862, EPI_ISL_714863, EPI_ISL_714864, EPI_ISL_714866, EPI_ISL_714867, EPI_ISL_714868, EPI_ISL_714869, EPI_ISL_714870,
EPI_ISL_714871, EPI_ISL_714872, EPI_ISL_714873, EPI_ISL_714874, EPI_ISL_714875, EPI_ISL_714876, EPI_ISL_714877, EPI_ISL_714878, EPI_ISL_714879, EPI_ISL_714880, EPI_ISL_714881, EPI_ISL_714883, EPI_ISL_714884, EPI_ISL_714885, EPI_ISL_714886, EPI_ISL_714888, EPI_ISL_714890, EPI_ISL_714891,
EPI_ISL_714892, EPI_ISL_714893, EPI_ISL_714894, EPI_ISL_714895, EPI_ISL_714897, EPI_ISL_714898, EPI_ISL_714899, EPI_ISL_714900, EPI_ISL_714901, EPI_ISL_714902, EPI_ISL_714903

see above	Department of Virus and Microbiological Special Diagnostics, Statens Serum Institut, Copenhagen, Denmark	Albertsen Lab, Department of Chemistry and Bioscience, Aalborg University, Denmark	Danish Covid-19 Genome Consortium
EPI_ISL_717620, EPI_ISL_717621	Lab voor klinische biologie	Onderzoeksgroep Virologie	Nick Vereecke, Laurens Lambrechts, Marthe Pauwels, Bruno Verhasselt, Linos Vandekerckhove, Hans Nauwynck, Sebastiaan Theuns
EPI_ISL_717734, EPI_ISL_717741, EPI_ISL_717752	Kingston Health Sciences Centre and Queen's University	Ontario Institute for Cancer Research	Prameet M. Sheth,Calvin Sjaarda,Robert Colautti,Katya Douchant,Ilinca Lungu,Bernard Lam,Paul Krzyzanowski,Michael Laszloffy,Lawrence E. Heisler,Richard de Borja,Jared T. Simpson
EPI_ISL_718009, EPI_ISL_718011, EPI_ISL_718012, EPI_ISL_718015	Lab voor klinische biologie	Onderzoeksgroep Virologie	Laurens Lambrechts, Nick Vereecke, Marthe Pauwels, Bruno Verhasselt, Linos Vandekerckhove, Hans Nauwynck, Sebastiaan Theuns
EPI_ISL_718016, EPI_ISL_718017, EPI_ISL_718018, EPI_ISL_718020, EPI_ISL_718021	Lab voor klinische biologie	Onderzoeksgroep Virologie	Nick Vereecke, Laurens Lambrechts, Marthe Pauwels, Bruno Verhasselt, Linos Vandekerckhove, Hans Nauwynck, Sebastiaan Theuns
EPI_ISL_718023	Lab voor klinische biologie	Onderzoeksgroep Virologie	Laurens Lambrechts, Nick Vereecke, Marthe Pauwels, Bruno Verhasselt, Linos Vandekerckhove, Hans Nauwynck, Sebastiaan Theuns
EPI_ISL_718025	Lab voor klinische biologie	Onderzoeksgroep Virologie	Nick Vereecke, Laurens Lambrechts, Marthe Pauwels, Bruno Verhasselt, Linos Vandekerckhove, Hans Nauwynck, Sebastiaan Theuns
EPI_ISL_718172, EPI_ISL_718200, EPI_ISL_718202	Ministry of Health Hospitals	Institute of Health and Community Medicine	David Perera, Ooi Mong How, Chua Hock Hin, Tonnii Sia Loong Loong, Wong Jyn Shan, Wong Kiing Aik, Chan Chia Jui
EPI_ISL_719605, EPI_ISL_719606, EPI_ISL_719608, EPI_ISL_719609, EPI_ISL_719611, EPI_ISL_719612, EPI_ISL_719613, EPI_ISL_719619, EPI_ISL_719621, EPI_ISL_719622, EPI_ISL_719623, EPI_ISL_719625, EPI_ISL_719629, EPI_ISL_719631, EPI_ISL_719635, EPI_ISL_719636, EPI_ISL_719640, EPI_ISL_719641, EPI_ISL_719643, EPI_ISL_719644, EPI_ISL_719646, EPI_ISL_719648, EPI_ISL_719651, EPI_ISL_719652, EPI_ISL_719655, EPI_ISL_719656, EPI_ISL_719657, EPI_ISL_719663, EPI_ISL_719664, EPI_ISL_719668, EPI_ISL_719670, EPI_ISL_719671, EPI_ISL_719673, EPI_ISL_719680, EPI_ISL_719681, EPI_ISL_719683, EPI_ISL_719685, EPI_ISL_719686, EPI_ISL_719687, EPI_ISL_719688, EPI_ISL_719691, EPI_ISL_719692, EPI_ISL_719693, EPI_ISL_719694, EPI_ISL_719698, EPI_ISL_719700, EPI_ISL_719701, EPI_ISL_719702, EPI_ISL_719703, EPI_ISL_719705, EPI_ISL_719707, EPI_ISL_719708, EPI_ISL_719709, EPI_ISL_719710, EPI_ISL_719712, EPI_ISL_719713, EPI_ISL_719716, EPI_ISL_719717, EPI_ISL_719720, EPI_ISL_719723, EPI_ISL_719724, EPI_ISL_719725, EPI_ISL_719728, EPI_ISL_719729, EPI_ISL_719731, EPI_ISL_719733, EPI_ISL_719735, EPI_ISL_719736, EPI_ISL_719738, EPI_ISL_719739, EPI_ISL_719740, EPI_ISL_719742, EPI_ISL_719744, EPI_ISL_719748, EPI_ISL_719749, EPI_ISL_719750, EPI_ISL_719755, EPI_ISL_719758, EPI_ISL_719760, EPI_ISL_719761, EPI_ISL_719764, EPI_ISL_719766, EPI_ISL_719768, EPI_ISL_719770, EPI_ISL_719771, EPI_ISL_719772, EPI_ISL_719773, EPI_ISL_719774, EPI_ISL_719775, EPI_ISL_719780, EPI_ISL_719786, EPI_ISL_719787, EPI_ISL_719788, EPI_ISL_719789, EPI_ISL_719790, EPI_ISL_719791, EPI_ISL_719792, EPI_ISL_719793, EPI_ISL_719796, EPI_ISL_719798, EPI_ISL_719800, EPI_ISL_719802, EPI_ISL_719803, EPI_ISL_719804, EPI_ISL_719805, EPI_ISL_719807, EPI_ISL_719809, EPI_ISL_719810, EPI_ISL_719811, EPI_ISL_719812, EPI_ISL_719815, EPI_ISL_719817, EPI_ISL_719819, EPI_ISL_719821, EPI_ISL_719823, EPI_ISL_719829, EPI_ISL_719834, EPI_ISL_719839, EPI_ISL_719841, EPI_ISL_719846, EPI_ISL_719848, EPI_ISL_719856, EPI_ISL_719857, EPI_ISL_719861, EPI_ISL_719862, EPI_ISL_719864, EPI_ISL_719865, EPI_ISL_719866, EPI_ISL_719867, EPI_ISL_719868, EPI_ISL_719871, EPI_ISL_719872, EPI_ISL_719873, EPI_ISL_719875, EPI_ISL_719876, EPI_ISL_719877, EPI_ISL_719880, EPI_ISL_719883, EPI_ISL_719884, EPI_ISL_719885, EPI_ISL_719889, EPI_ISL_719891, EPI_ISL_719892, EPI_ISL_719898, EPI_ISL_719900, EPI_ISL_719901, EPI_ISL_719902, EPI_ISL_719907, EPI_ISL_719908, EPI_ISL_719909, EPI_ISL_719910, EPI_ISL_719911, EPI_ISL_719912, EPI_ISL_719916, EPI_ISL_719918, EPI_ISL_719921, EPI_ISL_719922, EPI_ISL_719923, EPI_ISL_719925, EPI_ISL_719929, EPI_ISL_719930, EPI_ISL_719938, EPI_ISL_719940, EPI_ISL_719944, EPI_ISL_719951, EPI_ISL_719953, EPI_ISL_719958, EPI_ISL_719959, EPI_ISL_719961, EPI_ISL_719965, EPI_ISL_719972, EPI_ISL_719974, EPI_ISL_719978, EPI_ISL_719980, EPI_ISL_719983, EPI_ISL_719984, EPI_ISL_719985, EPI_ISL_719986			
see above	Lighthouse Lab in Glasgow	Wellcome Sanger Institute for the COVID-19 Genomics UK (COG-UK) Consortium	Harper VanSteenhouse, Yumi Kasai, David Gray, Carol Clugston, Anna Dominiczak and Alex Alderton, Roberto Amato, Sonia Goncalves, Ewan Harrison, David K. Jackson, Ian Johnston, Dominic Kwiatkowski, Cordelia Langford, John Sillitoe on behalf of the Wellcome Sanger Institute COVID-19 Surveillance Team
EPI_ISL_719992, EPI_ISL_719994, EPI_ISL_719995, EPI_ISL_719996, EPI_ISL_719998, EPI_ISL_720002, EPI_ISL_720004, EPI_ISL_720006, EPI_ISL_720008, EPI_ISL_720009, EPI_ISL_720010, EPI_ISL_720013, EPI_ISL_720017, EPI_ISL_720019, EPI_ISL_720020, EPI_ISL_720021, EPI_ISL_720024, EPI_ISL_720026, EPI_ISL_720030, EPI_ISL_720033, EPI_ISL_720036, EPI_ISL_720038, EPI_ISL_720039, EPI_ISL_720042, EPI_ISL_720043, EPI_ISL_720044, EPI_ISL_720046, EPI_ISL_720047, EPI_ISL_720049, EPI_ISL_720050, EPI_ISL_720051, EPI_ISL_720053, EPI_ISL_720054, EPI_ISL_720060, EPI_ISL_720063, EPI_ISL_720064, EPI_ISL_720068, EPI_ISL_720069, EPI_ISL_720070, EPI_ISL_720072, EPI_ISL_720073, EPI_ISL_720074, EPI_ISL_720077, EPI_ISL_720078, EPI_ISL_720082, EPI_ISL_720084, EPI_ISL_720086, EPI_ISL_720087, EPI_ISL_720089, EPI_ISL_720093, EPI_ISL_720094, EPI_ISL_720098, EPI_ISL_720099, EPI_ISL_720101, EPI_ISL_720102, EPI_ISL_720104, EPI_ISL_720111, EPI_ISL_720113, EPI_ISL_720114, EPI_ISL_720116, EPI_ISL_720118, EPI_ISL_720119, EPI_ISL_720120, EPI_ISL_720124, EPI_ISL_720126, EPI_ISL_720127, EPI_ISL_720128, EPI_ISL_720129, EPI_ISL_720130, EPI_ISL_720131, EPI_ISL_720132, EPI_ISL_720134, EPI_ISL_720135, EPI_ISL_720136, EPI_ISL_720140, EPI_ISL_720141, EPI_ISL_720142, EPI_ISL_720143, EPI_ISL_720145, EPI_ISL_720147, EPI_ISL_720148, EPI_ISL_720152, EPI_ISL_720153, EPI_ISL_720155, EPI_ISL_720157, EPI_ISL_720158, EPI_ISL_720159, EPI_ISL_720162, EPI_ISL_720164, EPI_ISL_720165, EPI_ISL_720167, EPI_ISL_720168, EPI_ISL_720169, EPI_ISL_720170, EPI_ISL_720171, EPI_ISL_720176, EPI_ISL_720180, EPI_ISL_720184, EPI_ISL_720185, EPI_ISL_720188, EPI_ISL_720191, EPI_ISL_720192, EPI_ISL_720194, EPI_ISL_720197, EPI_ISL_720200, EPI_ISL_720201, EPI_ISL_720203, EPI_ISL_720205, EPI_ISL_720206, EPI_ISL_720207, EPI_ISL_720208, EPI_ISL_720211, EPI_ISL_720213, EPI_ISL_720214, EPI_ISL_720215, EPI_ISL_720217, EPI_ISL_720218, EPI_ISL_720220, EPI_ISL_720222, EPI_ISL_720223, EPI_ISL_720230, EPI_ISL_720233, EPI_ISL_720235, EPI_ISL_720238, EPI_ISL_720239, EPI_ISL_720240, EPI_ISL_720241, EPI_ISL_720242, EPI_ISL_720243, EPI_ISL_720244, EPI_ISL_720246, EPI_ISL_720247, EPI_ISL_720248, EPI_ISL_720251, EPI_ISL_720253, EPI_ISL_720254, EPI_ISL_720255, EPI_ISL_720256, EPI_ISL_720259, EPI_ISL_720262, EPI_ISL_720263, EPI_ISL_720265, EPI_ISL_720266, EPI_ISL_720269, EPI_ISL_720274, EPI_ISL_720275, EPI_ISL_720276, EPI_ISL_720278, EPI_ISL_720281, EPI_ISL_720283, EPI_ISL_720285, EPI_ISL_720286, EPI_ISL_720289, EPI_ISL_720295, EPI_ISL_720296, EPI_ISL_720297, EPI_ISL_720300, EPI_ISL_720307, EPI_ISL_720309, EPI_ISL_720313, EPI_ISL_720314, EPI_ISL_720315, EPI_ISL_720316, EPI_ISL_720317, EPI_ISL_720320, EPI_ISL_720322, EPI_ISL_720324, EPI_ISL_720325, EPI_ISL_720326, EPI_ISL_720328, EPI_ISL_720330, EPI_ISL_720334, EPI_ISL_720336, EPI_ISL_720337, EPI_ISL_720338, EPI_ISL_720339, EPI_ISL_720340, EPI_ISL_720342, EPI_ISL_720344, EPI_ISL_720347, EPI_ISL_720348, EPI_ISL_720349, EPI_ISL_720350, EPI_ISL_720351, EPI_ISL_720352, EPI_ISL_720353, EPI_ISL_720355, EPI_ISL_720356, EPI_ISL_720360, EPI_ISL_720361, EPI_ISL_720362, EPI_ISL_720364, EPI_ISL_720365, EPI_ISL_720366, EPI_ISL_720367, EPI_ISL_720368, EPI_ISL_720371, EPI_ISL_720372, EPI_ISL_720374, EPI_ISL_720375, EPI_ISL_720376, EPI_ISL_720378, EPI_ISL_720379, EPI_ISL_720380, EPI_ISL_720382, EPI_ISL_720383, EPI_ISL_720384, EPI_ISL_720387, EPI_ISL_720389, EPI_ISL_720390, EPI_ISL_720391, EPI_ISL_720392, EPI_ISL_720393, EPI_ISL_720395, EPI_ISL_720397, EPI_ISL_720398, EPI_ISL_720399, EPI_ISL_720401, EPI_ISL_720404, EPI_ISL_720405, EPI_ISL_720406, EPI_ISL_720407, EPI_ISL_720411, EPI_ISL_720412, EPI_ISL_720413, EPI_ISL_720421, EPI_ISL_720423, EPI_ISL_720424, EPI_ISL_720428, EPI_ISL_720429, EPI_ISL_720432, EPI_ISL_720433, EPI_ISL_720434, EPI_ISL_720435, EPI_ISL_720440, EPI_ISL_720441, EPI_ISL_720442, EPI_ISL_720443, EPI_ISL_720444, EPI_ISL_720447, EPI_ISL_720448, EPI_ISL_720451, EPI_ISL_720454, EPI_ISL_720456, EPI_ISL_720459, EPI_ISL_720460, EPI_ISL_720461, EPI_ISL_720463, EPI_ISL_720464, EPI_ISL_720465, EPI_ISL_720467, EPI_ISL_720469, EPI_ISL_720470, EPI_ISL_720471, EPI_ISL_720472, EPI_ISL_720473, EPI_ISL_720474, EPI_ISL_720475, EPI_ISL_720476, EPI_ISL_720479, EPI_ISL_720481, EPI_ISL_720482, EPI_ISL_720484, EPI_ISL_720486, EPI_ISL_720487, EPI_ISL_720489, EPI_ISL_720490, EPI_ISL_720491, EPI_ISL_720493, EPI_ISL_720496, EPI_ISL_720498, EPI_ISL_720500, EPI_ISL_720501, EPI_ISL_720504, EPI_ISL_720506, EPI_ISL_720507, EPI_ISL_720510, EPI_ISL_720511, EPI_ISL_720512, EPI_ISL_720513, EPI_ISL_720514, EPI_ISL_720518, EPI_ISL_720520, EPI_ISL_720521, EPI_ISL_720522, EPI_ISL_720523, EPI_ISL_720524, EPI_ISL_720525, EPI_ISL_720527, EPI_ISL_720528, EPI_ISL_720530, EPI_ISL_720531, EPI_ISL_720533, EPI_ISL_720534, EPI_ISL_720535, EPI_ISL_720537, EPI_ISL_720538, EPI_ISL_720540, EPI_ISL_720541, EPI_ISL_720542, EPI_ISL_720545, EPI_ISL_720547, EPI_ISL_720548, EPI_ISL_720549, EPI_ISL_720550, EPI_ISL_720552, EPI_ISL_720553, EPI_ISL_720554, EPI_ISL_720555, EPI_ISL_720556, EPI_ISL_720557, EPI_ISL_720558, EPI_ISL_720559, EPI_ISL_720560, EPI_ISL_720561, EPI_ISL_720562, EPI_ISL_720564, EPI_ISL_720567, EPI_ISL_720568, EPI_ISL_720570, EPI_ISL_720571, EPI_ISL_720572, EPI_ISL_720573, EPI_ISL_720574, EPI_ISL_720575, EPI_ISL_720577, EPI_ISL_720578, EPI_ISL_720581, EPI_ISL_720582, EPI_ISL_720583, EPI_ISL_720584, EPI_ISL_720585, EPI_ISL_720586, EPI_ISL_720587, EPI_ISL_720589, EPI_ISL_720590, EPI_ISL_720591, EPI_ISL_720592, EPI_ISL_720593, EPI_ISL_720594, EPI_ISL_720596, EPI_ISL_720599, EPI_ISL_720600, EPI_ISL_720605, EPI_ISL_720607, EPI_ISL_720609, EPI_ISL_720610, EPI_ISL_720615, EPI_ISL_720616, EPI_ISL_720618, EPI_ISL_720620, EPI_ISL_720621, EPI_ISL_720623, EPI_ISL_720624, EPI_ISL_720625, EPI_ISL_720627, EPI_ISL_720628, EPI_ISL_720630, EPI_ISL_720632, EPI_ISL_720633, EPI_ISL_720635, EPI_ISL_720636, EPI_ISL_720637, EPI_ISL_720640, EPI_ISL_720641, EPI_ISL_720642, EPI_ISL_720643, EPI_ISL_720644, EPI_ISL_720645, EPI_ISL_720646, EPI_ISL_720647, EPI_ISL_720648, EPI_ISL_720650, EPI_ISL_720651, EPI_ISL_720653, EPI_ISL_720655, EPI_ISL_720658, EPI_ISL_720659, EPI_ISL_720661, EPI_ISL_720663, EPI_ISL_720665, EPI_ISL_720666, EPI_ISL_720667, EPI_ISL_720673			
see above	Lighthouse Lab in Milton Keynes	Wellcome Sanger Institute for the COVID-19 Genomics UK (COG-UK) Consortium	The Lighthouse Lab in Milton Keynes and Alex Alderton, Roberto Amato, Sonia Goncalves, Ewan Harrison, David K. Jackson, Ian Johnston, Dominic Kwiatkowski, Cordelia Langford, John Sillitoe on behalf of the Wellcome Sanger Institute COVID-19 Surveillance Team
EPI_ISL_720732, EPI_ISL_720910, EPI_ISL_720911, EPI_ISL_720912, EPI_ISL_720913, EPI_ISL_720914, EPI_ISL_720915, EPI_ISL_720917, EPI_ISL_720918, EPI_ISL_720919, EPI_ISL_720920, EPI_ISL_720922, EPI_ISL_720926, EPI_ISL_720927, EPI_ISL_720929, EPI_ISL_720930, EPI_ISL_720931, EPI_ISL_720932, EPI_ISL_720933, EPI_ISL_720934, EPI_ISL_720936, EPI_ISL_720939, EPI_ISL_720940, EPI_ISL_720941, EPI_ISL_720942, EPI_ISL_720944, EPI_ISL_720946, EPI_ISL_720948, EPI_ISL_720949, EPI_ISL_720950, EPI_ISL_720952, EPI_ISL_720953, EPI_ISL_720955, EPI_ISL_720957, EPI_ISL_720958, EPI_ISL_720960, EPI_ISL_720962, EPI_ISL_720963, EPI_ISL_720964, EPI_ISL_720965, EPI_ISL_720966, EPI_ISL_720967, EPI_ISL_720969, EPI_ISL_720970, EPI_ISL_720971, EPI_ISL_720974, EPI_ISL_720975, EPI_ISL_720976, EPI_ISL_720977, EPI_ISL_720978, EPI_ISL_720981, EPI_ISL_720982, EPI_ISL_720984, EPI_ISL_720985, EPI_ISL_720986, EPI_ISL_720988, EPI_ISL_720990, EPI_ISL_720991, EPI_ISL_720993, EPI_ISL_720995, EPI_ISL_720996, EPI_ISL_721000, EPI_ISL_721001, EPI_ISL_721003, EPI_ISL_721004, EPI_ISL_721006, EPI_ISL_721007, EPI_ISL_721008, EPI_ISL_721010, EPI_ISL_721011, EPI_ISL_721012, EPI_ISL_721013, EPI_ISL_721015, EPI_ISL_721016, EPI_ISL_721018, EPI_ISL_721020, EPI_ISL_721021, EPI_ISL_721025, EPI_ISL_721026, EPI_ISL_721028, EPI_ISL_721029, EPI_ISL_721031, EPI_ISL_721033, EPI_ISL_721034, EPI_ISL_721036, EPI_ISL_721039, EPI_ISL_721040, EPI_ISL_721041, EPI_ISL_721042, EPI_ISL_721043, EPI_ISL_721045, EPI_ISL_721046, EPI_ISL_721047, EPI_ISL_721048, EPI_ISL_721051, EPI_ISL_721052, EPI_ISL_721053, EPI_ISL_721054, EPI_ISL_721055, EPI_ISL_721056, EPI_ISL_721057, EPI_ISL_721058, EPI_ISL_721059, EPI_ISL_721063, EPI_ISL_721064, EPI_ISL_721066, EPI_ISL_721067, EPI_ISL_721068, EPI_ISL_721070, EPI_ISL_721073, EPI_ISL_721074, EPI_ISL_721075, EPI_ISL_721076, EPI_ISL_721077, EPI_ISL_721078, EPI_ISL_721083, EPI_ISL_721086, EPI_ISL_721087, EPI_ISL_721088, EPI_ISL_721091, EPI_ISL_721093, EPI_ISL_721094, EPI_ISL_721095, EPI_ISL_721096, EPI_ISL_721098, EPI_ISL_721100, EPI_ISL_721102, EPI_ISL_721104, EPI_ISL_721105, EPI_ISL_721106, EPI_ISL_721107, EPI_ISL_721109, EPI_ISL_721110, EPI_ISL_721112, EPI_ISL_721113, EPI_ISL_721120, EPI_ISL_721121, EPI_ISL_721125, EPI_ISL_721126, EPI_ISL_721127, EPI_ISL_721130, EPI_ISL_721131, EPI_ISL_721132, EPI_ISL_721133, EPI_ISL_721134, EPI_ISL_721136, EPI_ISL_721137, EPI_ISL_721140, EPI_ISL_721141, EPI_ISL_721144, EPI_ISL_721145, EPI_ISL_721146, EPI_ISL_721147, EPI_ISL_721148, EPI_ISL_721149, EPI_ISL_721150, EPI_ISL_721152, EPI_ISL_721153, EPI_ISL_721154, EPI_ISL_721155, EPI_ISL_721157, EPI_ISL_721159, EPI_ISL_721161, EPI_ISL_721163, EPI_ISL_721164, EPI_ISL_721165, EPI_ISL_721169, EPI_ISL_721172, EPI_ISL_721174, EPI_ISL_721175, EPI_ISL_721176, EPI_ISL_721177, EPI_ISL_721180, EPI_ISL_721182, EPI_ISL_721184			
see above	Lighthouse Lab in Glasgow	Wellcome Sanger Institute for the COVID-19 Genomics UK (COG-UK) Consortium	Harper VanSteenhouse, Yumi Kasai, David Gray, Carol Clugston, Anna Dominiczak and Alex Alderton, Roberto Amato, Sonia Goncalves, Ewan Harrison, David K. Jackson, Ian Johnston, Dominic Kwiatkowski, Cordelia Langford, John Sillitoe on behalf of the Wellcome Sanger Institute COVID-19 Surveillance Team
EPI_ISL_721185, EPI_ISL_721190, EPI_ISL_721191, EPI_ISL_721194, EPI_ISL_721195, EPI_ISL_721196, EPI_ISL_721197, EPI_ISL_721198, EPI_ISL_721200, EPI_ISL_721201, EPI_ISL_721202, EPI_ISL_721203, EPI_ISL_721204, EPI_ISL_721205, EPI_ISL_721206, EPI_ISL_721207, EPI_ISL_721209, EPI_ISL_721210, EPI_ISL_721211, EPI_ISL_721214, EPI_ISL_721215, EPI_ISL_721216, EPI_ISL_721217, EPI_ISL_721219, EPI_ISL_721220, EPI_ISL_721221, EPI_ISL_721222, EPI_ISL_721225, EPI_ISL_721226, EPI_ISL_721227, EPI_ISL_721229, EPI_ISL_721230, EPI_ISL_721231, EPI_ISL_721233, EPI_ISL_721236, EPI_ISL_721238, EPI_ISL_721239, EPI_ISL_721241, EPI_ISL_721242, EPI_ISL_721243, EPI_ISL_721244, EPI_ISL_721245, EPI_ISL_721246, EPI_ISL_721247, EPI_ISL_721248, EPI_ISL_721249, EPI_ISL_721251, EPI_ISL_721252, EPI_ISL_721253, EPI_ISL_721255, EPI_ISL_721256, EPI_ISL_721258, EPI_ISL_721259, EPI_ISL_721260, EPI_ISL_721261, EPI_ISL_721264, EPI_ISL_721265, EPI_ISL_721267, EPI_ISL_721268, EPI_ISL_721269, EPI_ISL_721272, EPI_ISL_721273, EPI_ISL_721275, EPI_ISL_721277, EPI_ISL_721278, EPI_ISL_721279, EPI_ISL_721280, EPI_ISL_721281, EPI_ISL_721282, EPI_ISL_721284, EPI_ISL_721287, EPI_ISL_721288, EPI_ISL_721290, EPI_ISL_721291, EPI_ISL_721292, EPI_ISL_721294, EPI_ISL_721295, EPI_ISL_721296, EPI_ISL_721297, EPI_ISL_721298, EPI_ISL_721299, EPI_ISL_721300, EPI_ISL_721301, EPI_ISL_721302, EPI_ISL_721303, EPI_ISL_721305, EPI_ISL_721306, EPI_ISL_721308, EPI_ISL_721309, EPI_ISL_721310, EPI_ISL_721311, EPI_ISL_721312, EPI_ISL_721313, EPI_ISL_721314, EPI_ISL_721315, EPI_ISL_721316, EPI_ISL_721318, EPI_ISL_721319, EPI_ISL_721320, EPI_ISL_721323, EPI_ISL_721324, EPI_ISL_721325, EPI_ISL_721326, EPI_ISL_721327, EPI_ISL_721328, EPI_ISL_721329, EPI_ISL_721331, EPI_ISL_721332, EPI_ISL_721336, EPI_ISL_721337, EPI_ISL_721339, EPI_ISL_721340, EPI_ISL_721342, EPI_ISL_721343, EPI_ISL_721344, EPI_ISL_721348, EPI_ISL_721349, EPI_ISL_721351, EPI_ISL_721352, EPI_ISL_721355, EPI_ISL_721357, EPI_ISL_721358, EPI_ISL_721360, EPI_ISL_721361, EPI_ISL_721364, EPI_ISL_721365, EPI_ISL_721367, EPI_ISL_721369, EPI_ISL_721370, EPI_ISL_721371, EPI_ISL_721373, EPI_ISL_721376, EPI_ISL_721377, EPI_ISL_721378, EPI_ISL_721380, EPI_ISL_721382, EPI_ISL_721384, EPI_ISL_721385, EPI_ISL_721387, EPI_ISL_721391, EPI_ISL_721392, EPI_ISL_721393, EPI_ISL_721394, EPI_ISL_721396, EPI_ISL_721397, EPI_ISL_721398, EPI_ISL_721400, EPI_ISL_721401, EPI_ISL_721402, EPI_ISL_721403, EPI_ISL_721404, EPI_ISL_721405, EPI_ISL_721406, EPI_ISL_721409, EPI_ISL_721412, EPI_ISL_721413, EPI_ISL_721415, EPI_ISL_721416, EPI_ISL_721418, EPI_ISL_721419, EPI_ISL_721420, EPI_ISL_721423, EPI_ISL_721425, EPI_ISL_721426, EPI_ISL_721427, EPI_ISL_721428, EPI_ISL_721429, EPI_ISL_721431, EPI_ISL_721433, EPI_ISL_721434, EPI_ISL_721435, EPI_ISL_721436, EPI_ISL_721437, EPI_ISL_721438, EPI_ISL_721439, EPI_ISL_721440, EPI_ISL_721441, EPI_ISL_721444, EPI_ISL_721445, EPI_ISL_721446, EPI_ISL_721447, EPI_ISL_721448, EPI_ISL_721449, EPI_ISL_721450, EPI_ISL_721451, EPI_ISL_721452, EPI_ISL_721454, EPI_ISL_721458, EPI_ISL_721459, EPI_ISL_721461, EPI_ISL_721462, EPI_ISL_721463, EPI_ISL_721467, EPI_ISL_721468, EPI_ISL_721469, EPI_ISL_721470, EPI_ISL_721471, EPI_ISL_721475, EPI_ISL_721477, EPI_ISL_721479, EPI_ISL_721480, EPI_ISL_721481, EPI_ISL_721482, EPI_ISL_721483, EPI_ISL_721485, EPI_ISL_721488, EPI_ISL_721490, EPI_ISL_721491, EPI_ISL_721492, EPI_ISL_721494, EPI_ISL_721495, EPI_ISL_721496, EPI_ISL_721497, EPI_ISL_721498, EPI_ISL_721499, EPI_ISL_721500, EPI_ISL_721501, EPI_ISL_721502, EPI_ISL_721504, EPI_ISL_721505, EPI_ISL_721506, EPI_ISL_721507, EPI_ISL_721508, EPI_ISL_721509, EPI_ISL_721512, EPI_ISL_721513, EPI_ISL_721514, EPI_ISL_721515, EPI_ISL_721516			
see above	Lighthouse Lab in Alderley Park	Wellcome Sanger Institute for the COVID-19 Genomics UK (COG-UK) Consortium	Jacquelyn Wynn, Mairead Hyland, The Lighthouse Lab in Alderley Park and Alex Alderton, Roberto Amato, Sonia Goncalves, Ewan Harrison, David K. Jackson, Ian Johnston, Dominic Kwiatkowski, Cordelia Langford, John Sillitoe on behalf of the Wellcome Sanger Institute COVID-19 Surveillance Team

EPI_ISL_721526, EPI_ISL_721529, EPI_ISL_721531, EPI_ISL_721532	Lighthouse Lab in Milton Keynes	Wellcome Sanger Institute for the COVID-19 Genomics UK (COG-UK) Consortium	The Lighthouse Lab in Milton Keynes and Alex Alderton, Roberto Amato, Sonia Goncalves, Ewan Harrison, David K. Jackson, Ian Johnston, Dominic Kwiatkowski, Cordelia Langford, John Sillitoe on behalf of the Wellcome Sanger Institute COVID-19 Surveillance Team
EPI_ISL_721533	Lighthouse Lab in Glasgow	Wellcome Sanger Institute for the COVID-19 Genomics UK (COG-UK) Consortium	Harper VanSteenhouse, Yumi Kasai, David Gray, Carol Clugston, Anna Dominiczak and Alex Alderton, Roberto Amato, Sonia Goncalves, Ewan Harrison, David K. Jackson, Ian Johnston, Dominic Kwiatkowski, Cordelia Langford, John Sillitoe on behalf of the Wellcome Sanger Institute COVID-19 Surveillance Team
EPI_ISL_721694, EPI_ISL_721771, EPI_ISL_721794, EPI_ISL_721839, EPI_ISL_721869, EPI_ISL_721878, EPI_ISL_721891, EPI_ISL_721910, EPI_ISL_721943, EPI_ISL_721953	Viollier AG	Department of Biosystems Science and Engineering, ETH Zürich	Christian Beisel
EPI_ISL_722201	Vitalis Mostar	Alea Genetic Center	Salihefendic L., Pecar D., Konjhodzic R.
EPI_ISL_722209	Dom Zdravlja Sarajevo	Alea Genetic Center	Salihefendic L., Pecar D., Konjhodzic R.
EPI_ISL_722419, EPI_ISL_722648, EPI_ISL_722649, EPI_ISL_722650, EPI_ISL_722651, EPI_ISL_722652, EPI_ISL_722653, EPI_ISL_722654, EPI_ISL_722655, EPI_ISL_722656, EPI_ISL_722657, EPI_ISL_722658, EPI_ISL_722659, EPI_ISL_722660, EPI_ISL_722661, EPI_ISL_722662, EPI_ISL_722663, EPI_ISL_722664, EPI_ISL_722665, EPI_ISL_722666, EPI_ISL_722667, EPI_ISL_722669, EPI_ISL_722670, EPI_ISL_722671, EPI_ISL_722672, EPI_ISL_722673, EPI_ISL_722674, EPI_ISL_722675, EPI_ISL_722676, EPI_ISL_722677, EPI_ISL_722678, EPI_ISL_722682, EPI_ISL_722683, EPI_ISL_722684, EPI_ISL_722685, EPI_ISL_722686, EPI_ISL_722688, EPI_ISL_722689, EPI_ISL_722690, EPI_ISL_722692, EPI_ISL_722693, EPI_ISL_722695, EPI_ISL_722696, EPI_ISL_722697, EPI_ISL_722711, EPI_ISL_722712			
see above	Dutch COVID-19 response team	Erasmus Medical Center	Bas Oude Munnink, Reina Sikkema, David Nieuwenhuijse, Irina Chestakova, Anne van der Linden, Marjan Boter, Emmanuelle Munger, Corine GeurtsvanKessel, Annemiek van der Eijk, Richard Molenkamp, Marion Koopmans, on behalf of the Dutch national COVID-19 response team.
EPI_ISL_722910	Istituto Zooprofilattico Sperimentale della Puglia e della Basilicata	Istituto Zooprofilattico Sperimentale della Puglia e della Basilicata	Parisi A., Bianco A., Capozzi L., Del Sambro L., Manzulli V, Rondinone V., Pace L., Cipolletta D., Galante D.
EPI_ISL_722936, EPI_ISL_722938, EPI_ISL_722939, EPI_ISL_722940, EPI_ISL_722942, EPI_ISL_722943, EPI_ISL_722946, EPI_ISL_722949, EPI_ISL_722950, EPI_ISL_722952, EPI_ISL_722954, EPI_ISL_722955, EPI_ISL_722956			
see above	Department of Clinical Microbiology	GIGA Medical Genomics	Keith Durkin, Maria Artesi, Sébastien Bontems, Raphaël Boreux, Bouchra Boujemla, Cécile Meex, Pierrette Melin, Marie-Pierre Hayette, Vincent Bours
EPI_ISL_723123, EPI_ISL_723124, EPI_ISL_723125, EPI_ISL_723126, EPI_ISL_723128, EPI_ISL_723130, EPI_ISL_723132, EPI_ISL_723133, EPI_ISL_723134, EPI_ISL_723135, EPI_ISL_723136			
see above	Minnesota Department of Health, Public Health Laboratory	Minnesota Department of Health, Public Health Laboratory	Alexandra Lorentz, Jacob Garfin, Matt Plumb, and Xiong Wang
EPI_ISL_723147, EPI_ISL_723148, EPI_ISL_723149, EPI_ISL_723150, EPI_ISL_723151, EPI_ISL_723153	Vault Health	Minnesota Department of Health, Public Health Laboratory	Alexandra Lorentz, Jacob Garfin, Matt Plumb, and Xiong Wang
EPI_ISL_723155, EPI_ISL_723156, EPI_ISL_723157, EPI_ISL_723160, EPI_ISL_723169, EPI_ISL_723179, EPI_ISL_723202, EPI_ISL_723203, EPI_ISL_723204, EPI_ISL_723205, EPI_ISL_723206, EPI_ISL_723207, EPI_ISL_723208, EPI_ISL_723209, EPI_ISL_723210, EPI_ISL_723211, EPI_ISL_723213, EPI_ISL_723214, EPI_ISL_723215, EPI_ISL_723216, EPI_ISL_723217, EPI_ISL_723218, EPI_ISL_723220, EPI_ISL_723221, EPI_ISL_723222, EPI_ISL_723225, EPI_ISL_723226, EPI_ISL_723227, EPI_ISL_723229, EPI_ISL_723230, EPI_ISL_723231, EPI_ISL_723242, EPI_ISL_723243, EPI_ISL_723244, EPI_ISL_723245, EPI_ISL_723246, EPI_ISL_723247, EPI_ISL_723248, EPI_ISL_723249, EPI_ISL_723250, EPI_ISL_723251, EPI_ISL_723263, EPI_ISL_723264, EPI_ISL_723265, EPI_ISL_723266, EPI_ISL_723267, EPI_ISL_723268, EPI_ISL_723269, EPI_ISL_723270, EPI_ISL_723272, EPI_ISL_723273, EPI_ISL_723274, EPI_ISL_723288, EPI_ISL_723289, EPI_ISL_723290, EPI_ISL_723291, EPI_ISL_723292, EPI_ISL_723293, EPI_ISL_723294, EPI_ISL_723295, EPI_ISL_723296, EPI_ISL_723297, EPI_ISL_723306, EPI_ISL_723307, EPI_ISL_723308, EPI_ISL_723309, EPI_ISL_723310, EPI_ISL_723311, EPI_ISL_723312, EPI_ISL_723313, EPI_ISL_723314, EPI_ISL_723315, EPI_ISL_723316, EPI_ISL_723317, EPI_ISL_723318, EPI_ISL_723319, EPI_ISL_723320, EPI_ISL_723321, EPI_ISL_723322, EPI_ISL_723323, EPI_ISL_723324, EPI_ISL_723325, EPI_ISL_723327, EPI_ISL_723328, EPI_ISL_723329, EPI_ISL_723330, EPI_ISL_723331, EPI_ISL_723332, EPI_ISL_723333, EPI_ISL_723334, EPI_ISL_723335, EPI_ISL_723336, EPI_ISL_723337, EPI_ISL_723338, EPI_ISL_723339, EPI_ISL_723341, EPI_ISL_723342, EPI_ISL_723343, EPI_ISL_723344, EPI_ISL_723345, EPI_ISL_723346, EPI_ISL_723347, EPI_ISL_723348, EPI_ISL_723349, EPI_ISL_723350, EPI_ISL_723351, EPI_ISL_723352, EPI_ISL_723353, EPI_ISL_723354, EPI_ISL_723355, EPI_ISL_723356, EPI_ISL_723357, EPI_ISL_723358, EPI_ISL_723359, EPI_ISL_723360, EPI_ISL_723361, EPI_ISL_723362, EPI_ISL_723363, EPI_ISL_723364, EPI_ISL_723365, EPI_ISL_723366, EPI_ISL_723368, EPI_ISL_723381, EPI_ISL_723383, EPI_ISL_723386, EPI_ISL_723394, EPI_ISL_723395, EPI_ISL_723396, EPI_ISL_723397, EPI_ISL_723398, EPI_ISL_723399, EPI_ISL_723400, EPI_ISL_723401, EPI_ISL_723402, EPI_ISL_723403, EPI_ISL_723404, EPI_ISL_723405, EPI_ISL_723406, EPI_ISL_723407, EPI_ISL_723408, EPI_ISL_723409, EPI_ISL_723410, EPI_ISL_723411, EPI_ISL_723412, EPI_ISL_723413, EPI_ISL_723414, EPI_ISL_723415, EPI_ISL_723416, EPI_ISL_723417, EPI_ISL_723418, EPI_ISL_723419, EPI_ISL_723420, EPI_ISL_723421, EPI_ISL_723422, EPI_ISL_723423, EPI_ISL_723424, EPI_ISL_723428, EPI_ISL_723429, EPI_ISL_723430, EPI_ISL_723431, EPI_ISL_723432, EPI_ISL_723433, EPI_ISL_723434, EPI_ISL_723435, EPI_ISL_723436, EPI_ISL_723440, EPI_ISL_723441, EPI_ISL_723442, EPI_ISL_723443, EPI_ISL_723444, EPI_ISL_723445, EPI_ISL_723447, EPI_ISL_723449, EPI_ISL_723450, EPI_ISL_723451, EPI_ISL_723459, EPI_ISL_723466			
see above	Dutch COVID-19 response team	National Institute for Public Health and the Environment (RIVM)	Adam Meijer, Harry Vennema, Jeroen Cremer, Sharon van den Brink, Bas van der Veer, AnneMarie van den Brandt, Florian Zwagemaker, Dennis Schmitz, Chantal Reusken, on behalf of the national COVID-19 response team
EPI_ISL_723473, EPI_ISL_723477, EPI_ISL_723479, EPI_ISL_723482, EPI_ISL_723483, EPI_ISL_723486, EPI_ISL_723489, EPI_ISL_723490	Virginia DCLS	Virginia DCLS	Virginia DCLS
EPI_ISL_723864, EPI_ISL_723876, EPI_ISL_723885, EPI_ISL_723886, EPI_ISL_723895	Department of Pathology, University of Cambridge	COVID-19 Genomics UK (COG-UK) Consortium	Aminu S. Jahun, Yasmin Chaudhry, Grant Hall, Iliana Georgana, Myra Hosmillo, Martin D. Curran, Malte Pinckert, Surendra Parmar, Ian Goodfellow
EPI_ISL_724063, EPI_ISL_724065, EPI_ISL_724071, EPI_ISL_724087, EPI_ISL_724093, EPI_ISL_724096, EPI_ISL_724104, EPI_ISL_724107, EPI_ISL_724109, EPI_ISL_724110, EPI_ISL_724111, EPI_ISL_724112, EPI_ISL_724115, EPI_ISL_724120, EPI_ISL_724123			
see above	Northumbria University / South Tees Hospitals NHS Foundation Trust / North Cumbria Integrated Care NHS Foundation Trust / North Tees and Hartlepool NHS Foundation Trust / Newcastle Hospitals NHS Foundation Trust	COVID-19 Genomics UK (COG-UK) Consortium	Darren L Smith,Andrew Nelson,Matthew Bashton,Greg R Young,Joshua Loh,John Allan,Mohammad A Tariq,Giles S Holt,Gary Black,Wen C Yew,Lynn Dover,Paul Baker,Steve Liggett,Sarah Essex,Jane Greenaway,Debra Padgett,Clive Graham,Garren Scott,Edward Barton,Emma Swindells,Brendan Payne,Jennifer Collins,Yusri Taha,Gary Eltringham
EPI_ISL_724223, EPI_ISL_724224, EPI_ISL_724226	Virology Department, Royal Infirmary of Edinburgh, NHS Lothian / School of Biological Sciences, University of Edinburgh / Institute of Genetics and Molecular Medicine, University of Edinburgh	COVID-19 Genomics UK (COG-UK) Consortium	McHugh M, Dewar R, Rooke S, Gallagher M, Balcaza C, O'Toole Á, Scher E, Hill V, McCrone JT, Colquhoun R, Yu X, Jackson B, Rambaut A, Williams TC, Templeton K
EPI_ISL_724436, EPI_ISL_724442, EPI_ISL_724448, EPI_ISL_724458, EPI_ISL_724464, EPI_ISL_724468, EPI_ISL_724487, EPI_ISL_724499, EPI_ISL_724501, EPI_ISL_724503, EPI_ISL_724517, EPI_ISL_724522			
see above	Liverpool Clinical Laboratories	COVID-19 Genomics UK (COG-UK) Consortium	Sam Haldenby, Anita Lucaci, Steve Paterson, Julian Hiscox, Alistair Darby, M Almsaud, A Alrezaihi, Muhannad Alruwaili, Stuart D Armstrong, Jones Benjamin, Eleanor G Bentley, Anu Chawla, Jordan J Clark, Angela Cowell, Richard Eccles, Isabel García-Dorival, Matthew Gemmell, Alessandro Gerada, PKF Gilmore, Richard Gregory, Ximeng Han, Catherine Hartley, Margaret Hughes, Miren Iturriza-Gomara, James Johnson, L Luu, Jenifer Manson, Charlotte Nelson, Elaine O'Toole, Cassie Olateju, Rebekah Penrice-Randal , Lucille Rainbow, N.P Randle, Trevor Ian Robinson, Parul Sharma, Ghada T Shawli, James P Stewart, Neil Swainston, Ecaterina Vamos, Joanne Watts, Mark Whitehead
EPI_ISL_724857, EPI_ISL_724859, EPI_ISL_724874, EPI_ISL_724878, EPI_ISL_724880, EPI_ISL_724882, EPI_ISL_724883, EPI_ISL_724888, EPI_ISL_724889, EPI_ISL_724891, EPI_ISL_724924, EPI_ISL_724936, EPI_ISL_724938, EPI_ISL_724941, EPI_ISL_724942, EPI_ISL_724954, EPI_ISL_724959, EPI_ISL_725008, EPI_ISL_725016			
see above	Northumbria University / South Tees Hospitals NHS Foundation Trust / North Cumbria Integrated Care NHS Foundation Trust / North Tees and Hartlepool NHS Foundation Trust / Newcastle Hospitals NHS Foundation Trust	COVID-19 Genomics UK (COG-UK) Consortium	Darren L Smith,Andrew Nelson,Matthew Bashton,Greg R Young,Joshua Loh,John Allan,Mohammad A Tariq,Giles S Holt,Gary Black,Wen C Yew,Lynn Dover,Paul Baker,Steve Liggett,Sarah Essex,Jane Greenaway,Debra Padgett,Clive Graham,Garren Scott,Edward Barton,Emma Swindells,Brendan Payne,Jennifer Collins,Yusri Taha,Gary Eltringham
EPI_ISL_725184, EPI_ISL_725226, EPI_ISL_725248, EPI_ISL_725260	Quadram Institute Bioscience	COVID-19 Genomics UK (COG-UK) Consortium	Dave J. Baker, Gemma L. Kay, Alp Aydin, Thanh Le-Viet, Steven Rudder, Ana P. Tedim, Anastasia Kolyva, Maria Diaz, Leonardo de Oliveira Martins, Nabil-Fareed Alikhan, Lizzie Meadows, Rachael Stanley, Ngozi Elumogo, Muhammed Yasir, Nicholas M. Thomson, Alexander J Trotter, Rachel Gilroy, Samuel Bloomfield, Claire Stuart, Andrew Bell, Reenesh Prakash, Samir Dervisevic, Alison E. Mather, John Wain, Mark Webber, Andrew J. Page, Justin O'Grady
EPI_ISL_725534	Queens Medical Centre, Clinical Microbiology Department / DeepSeq Nottingham	COVID-19 Genomics UK (COG-UK) Consortium	Gemma Clark, Wendy Smith, Manjinder Khakh, Vicki M Fleming, Michelle M Lister, Hannah Howson-Wells, Jonathan Ball, Patrick McClure, Joseph Chappell, Theocharis Tsoleridis, Nadine Holmes, Matthew Carlisle, Christopher Moore, Fei Sang, Johnny Debebe, Victoria Wright, Matthew Loose
EPI_ISL_725556, EPI_ISL_725558, EPI_ISL_725573, EPI_ISL_725581,	Lincolnshire Hospitals and DeepSeq Nottingham	COVID-19 Genomics UK (COG-UK) Consortium	Nichola Duckworth, Tim Sloan, Sarah Walsh, Jonathan Ball, Patrick McClure, Joeseph Chappell, Nadine Holmes, Matthew Carlisle, Christopher Moore, Fei Sang, Johnny Debebe, Victoria Wright, Matthew Loose

EPI_ISL_725586			

EPI_ISL_725677, EPI_ISL_725696, EPI_ISL_725700, EPI_ISL_725705, EPI_ISL_725744, EPI_ISL_725765, EPI_ISL_725767, EPI_ISL_725768, EPI_ISL_725769, EPI_ISL_725777, EPI_ISL_725778, EPI_ISL_725784, EPI_ISL_725786, EPI_ISL_725789, EPI_ISL_725796, EPI_ISL_725798, EPI_ISL_725800, EPI_ISL_725801, EPI_ISL_725805, EPI_ISL_725806, EPI_ISL_725810, EPI_ISL_725814, EPI_ISL_725816, EPI_ISL_725817, EPI_ISL_725818, EPI_ISL_725821, EPI_ISL_725822, EPI_ISL_725825, EPI_ISL_725827, EPI_ISL_725828, EPI_ISL_725830, EPI_ISL_725831, EPI_ISL_725832, EPI_ISL_725833, EPI_ISL_725834, EPI_ISL_725836, EPI_ISL_725837, EPI_ISL_725841, EPI_ISL_725846, EPI_ISL_725847, EPI_ISL_725851, EPI_ISL_725852, EPI_ISL_725864, EPI_ISL_725870, EPI_ISL_725884, EPI_ISL_725886, EPI_ISL_725887, EPI_ISL_725889, EPI_ISL_725890, EPI_ISL_725891, EPI_ISL_725898, EPI_ISL_725899, EPI_ISL_725901, EPI_ISL_725911, EPI_ISL_725915, EPI_ISL_725916, EPI_ISL_725917, EPI_ISL_725918, EPI_ISL_725919, EPI_ISL_725920, EPI_ISL_725921, EPI_ISL_725923, EPI_ISL_725924, EPI_ISL_725926, EPI_ISL_725927, EPI_ISL_725933, EPI_ISL_725936, EPI_ISL_725937, EPI_ISL_725938, EPI_ISL_725940, EPI_ISL_725941, EPI_ISL_725942, EPI_ISL_725943, EPI_ISL_725944, EPI_ISL_725949, EPI_ISL_725953, EPI_ISL_725955, EPI_ISL_725965, EPI_ISL_725969, EPI_ISL_725977, EPI_ISL_725984, EPI_ISL_725996, EPI_ISL_726000, EPI_ISL_726006, EPI_ISL_726011, EPI_ISL_726022, EPI_ISL_726023, EPI_ISL_726028, EPI_ISL_726029, EPI_ISL_726031, EPI_ISL_726066, EPI_ISL_726071, EPI_ISL_726076, EPI_ISL_726085, EPI_ISL_726086, EPI_ISL_726089, EPI_ISL_726091, EPI_ISL_726097, EPI_ISL_726098, EPI_ISL_726101, EPI_ISL_726102, EPI_ISL_726104, EPI_ISL_726105, EPI_ISL_726106, EPI_ISL_726113, EPI_ISL_726115, EPI_ISL_726118, EPI_ISL_726120, EPI_ISL_726123, EPI_ISL_726124, EPI_ISL_726126, EPI_ISL_726133, EPI_ISL_726146, EPI_ISL_726148, EPI_ISL_726149, EPI_ISL_726155, EPI_ISL_726157, EPI_ISL_726158, EPI_ISL_726159, EPI_ISL_726165, EPI_ISL_726166, EPI_ISL_726167, EPI_ISL_726171, EPI_ISL_726172, EPI_ISL_726174, EPI_ISL_726175, EPI_ISL_726177, EPI_ISL_726178, EPI_ISL_726180, EPI_ISL_726182, EPI_ISL_726183, EPI_ISL_726185, EPI_ISL_726186, EPI_ISL_726187, EPI_ISL_726188, EPI_ISL_726192, EPI_ISL_726194, EPI_ISL_726196, EPI_ISL_726198, EPI_ISL_726203, EPI_ISL_726205, EPI_ISL_726209, EPI_ISL_726214, EPI_ISL_726216, EPI_ISL_726220, EPI_ISL_726247, EPI_ISL_726251, EPI_ISL_726256, EPI_ISL_726263, EPI_ISL_726272, EPI_ISL_726275, EPI_ISL_726284, EPI_ISL_726287, EPI_ISL_726297, EPI_ISL_726299, EPI_ISL_726302, EPI_ISL_726304, EPI_ISL_726321, EPI_ISL_726324, EPI_ISL_726348, EPI_ISL_726352, EPI_ISL_726353, EPI_ISL_726363, EPI_ISL_726370, EPI_ISL_726373, EPI_ISL_726374, EPI_ISL_726377, EPI_ISL_726390, EPI_ISL_726391, EPI_ISL_726392, EPI_ISL_726398, EPI_ISL_726404, EPI_ISL_726413, EPI_ISL_726417, EPI_ISL_726418, EPI_ISL_726447, EPI_ISL_726448, EPI_ISL_726449, EPI_ISL_726453, EPI_ISL_726454, EPI_ISL_726460, EPI_ISL_726461, EPI_ISL_726462, EPI_ISL_726463, EPI_ISL_726464, EPI_ISL_726469, EPI_ISL_726471, EPI_ISL_726472, EPI_ISL_726474, EPI_ISL_726477, EPI_ISL_726478, EPI_ISL_726479, EPI_ISL_726480, EPI_ISL_726482, EPI_ISL_726485, EPI_ISL_726487, EPI_ISL_726488, EPI_ISL_726489, EPI_ISL_726492, EPI_ISL_726496, EPI_ISL_726497, EPI_ISL_726498, EPI_ISL_726504, EPI_ISL_726507, EPI_ISL_726513, EPI_ISL_726516, EPI_ISL_726531, EPI_ISL_726533, EPI_ISL_726536, EPI_ISL_726537, EPI_ISL_726539, EPI_ISL_726541, EPI_ISL_726545, EPI_ISL_726547, EPI_ISL_726549, EPI_ISL_726553, EPI_ISL_726554, EPI_ISL_726555, EPI_ISL_726558, EPI_ISL_726560, EPI_ISL_726565, EPI_ISL_726567, EPI_ISL_726568, EPI_ISL_726569, EPI_ISL_726570, EPI_ISL_726572, EPI_ISL_726573, EPI_ISL_726574, EPI_ISL_726575, EPI_ISL_726577, EPI_ISL_726578, EPI_ISL_726579, EPI_ISL_726580, EPI_ISL_726581, EPI_ISL_726584, EPI_ISL_726586, EPI_ISL_726587, EPI_ISL_726592, EPI_ISL_726597, EPI_ISL_726602, EPI_ISL_726603, EPI_ISL_726606, EPI_ISL_726607, EPI_ISL_726608, EPI_ISL_726609, EPI_ISL_726610, EPI_ISL_726613, EPI_ISL_726614, EPI_ISL_726615, EPI_ISL_726616, EPI_ISL_726617, EPI_ISL_726618

see above	Wales Specialist Virology Centre Sequencing lab: Pathogen Genomics Unit	COVID-19 Genomics UK (COG-UK) Consortium	Catherine Moore, Johnathan Evans, Laura Gifford, Malorie Perry, Simon Cottrell, Angela Marchbank, Alec Birchley, Alexander Adams, Amy Gaskin, Bree Gatica-Wilcox, Jason Coombes, Joel Southgate, Lauren Gilbert, Lee Graham, Nicole Pacchiarini, Sara Kumziene-Summerhayes, Sarah Taylor, Sophie Jones, Sara Rey, Matthew Bull, Joanne Watkins, Sally Corden, Tom Connor

We gratefully acknowledge the following Authors from the Originating laboratories responsible for obtaining the specimens, as well as the Submitting laboratories where the genome data were generated and shared via GISAID, on which this research is based.

All Submitters of data may be contacted directly via www.gisaid.org

Authors are sorted alphabetically.

Accession ID	Originating Laboratory	Submitting Laboratory	Authors
EPI_ISL_726619, EPI_ISL_726620, EPI_ISL_726622, EPI_ISL_726623, EPI_ISL_726624, EPI_ISL_726626, EPI_ISL_726627, EPI_ISL_726628, EPI_ISL_726631, EPI_ISL_726635, EPI_ISL_726640, EPI_ISL_726664, EPI_ISL_726672, EPI_ISL_726684, EPI_ISL_726688, EPI_ISL_726697, EPI_ISL_726706, EPI_ISL_726708, EPI_ISL_726714, EPI_ISL_726715, EPI_ISL_726718, EPI_ISL_726721, EPI_ISL_726724, EPI_ISL_726728, EPI_ISL_726729, EPI_ISL_726735, EPI_ISL_726736, EPI_ISL_726737, EPI_ISL_726740, EPI_ISL_726748, EPI_ISL_726749, EPI_ISL_726751, EPI_ISL_726753, EPI_ISL_726754, EPI_ISL_726755, EPI_ISL_726760, EPI_ISL_726761, EPI_ISL_726768, EPI_ISL_726771, EPI_ISL_726772, EPI_ISL_726780, EPI_ISL_726782, EPI_ISL_726787, EPI_ISL_726788, EPI_ISL_726789, EPI_ISL_726792, EPI_ISL_726794, EPI_ISL_726796, EPI_ISL_726801, EPI_ISL_726802, EPI_ISL_726806, EPI_ISL_726808, EPI_ISL_726815, EPI_ISL_726817, EPI_ISL_726819, EPI_ISL_726820, EPI_ISL_726823, EPI_ISL_726824, EPI_ISL_726828, EPI_ISL_726830, EPI_ISL_726831, EPI_ISL_726833, EPI_ISL_726842, EPI_ISL_726845, EPI_ISL_726848, EPI_ISL_726870, EPI_ISL_726883, EPI_ISL_726891, EPI_ISL_726908, EPI_ISL_726954, EPI_ISL_726964, EPI_ISL_726967, EPI_ISL_726970, EPI_ISL_726974, EPI_ISL_726975, EPI_ISL_726977, EPI_ISL_726979, EPI_ISL_726980, EPI_ISL_726983, EPI_ISL_726985, EPI_ISL_726989, EPI_ISL_726990, EPI_ISL_726991, EPI_ISL_726992, EPI_ISL_726993, EPI_ISL_726995, EPI_ISL_726996, EPI_ISL_726997, EPI_ISL_727001, EPI_ISL_727004, EPI_ISL_727005, EPI_ISL_727006, EPI_ISL_727008, EPI_ISL_727009, EPI_ISL_727011, EPI_ISL_727015, EPI_ISL_727016, EPI_ISL_727017, EPI_ISL_727019, EPI_ISL_727020, EPI_ISL_727021, EPI_ISL_727023, EPI_ISL_727025, EPI_ISL_727027, EPI_ISL_727030, EPI_ISL_727218, EPI_ISL_727226, EPI_ISL_727416, EPI_ISL_727417, EPI_ISL_727442, EPI_ISL_727445, EPI_ISL_727446, EPI_ISL_727448, EPI_ISL_727461, EPI_ISL_727466, EPI_ISL_727474, EPI_ISL_727477, EPI_ISL_727480, EPI_ISL_727483, EPI_ISL_727486, EPI_ISL_727495, EPI_ISL_727496, EPI_ISL_727499			
see above	Wales Specialist Virology Centre Sequencing lab: Pathogen Genomics Unit	COVID-19 Genomics UK (COG-UK) Consortium	Catherine Moore, Johnathan Evans, Laura Gifford, Malorie Perry, Simon Cottrell, Angela Marchbank, Alec Birchley, Alexander Adams, Amy Gaskin, Bree Gatica-Wilcox, Jason Coombes, Joel Southgate, Lauren Gilbert, Lee Graham, Nicole Pacchiarini, Sara Kumziene-Summerhayes, Sarah Taylor, Sophie Jones, Sara Rey, Matthew Bull, Joanne Watkins, Sally Corden, Tom Connor
EPI_ISL_728090, EPI_ISL_728094, EPI_ISL_728095, EPI_ISL_728098, EPI_ISL_728108, EPI_ISL_728113, EPI_ISL_728116, EPI_ISL_728120, EPI_ISL_728125, EPI_ISL_728126, EPI_ISL_728133			
see above	University of Wisconsin-Madison AIDS Vaccine Research Laboratories	University of Wisconsin-Madison AIDS Vaccine Research Laboratories	Gage Moreno, Katarina Braun, et al. AIDS Vaccine Research Laboratories
EPI_ISL_728249, EPI_ISL_728250, EPI_ISL_728251, EPI_ISL_728252	Institute for Medical Research, Infectious Disease Research Centre, National Institutes of Health, Ministry of Health Malaysia	Institute for Medical Research, Infectious Disease Research Centre, National Institutes of Health, Ministry of Health Malaysia	Suppiah J, Kamel K, Mohd-Zawawi Z, Thayan R
EPI_ISL_728300	Lighthouse Lab in Glasgow	Wellcome Sanger Institute for the COVID-19 Genomics UK (COG-UK) Consortium	Harper VanSteenhouse, Yumi Kasai, David Gray, Carol Clugston, Anna Dominiczak and Alex Alderton, Roberto Amato, Sonia Goncalves, Ewan Harrison, David K. Jackson, Ian Johnston, Dominic Kwiatkowski, Cordelia Langford, John Sillitoe on behalf of the Wellcome Sanger Institute COVID-19 Surveillance Team
EPI_ISL_728311, EPI_ISL_728315, EPI_ISL_728317, EPI_ISL_728319, EPI_ISL_728320	CNR Virus des Infections Respiratoires - France SUD	CNR Virus des Infections Respiratoires - France SUD	Antonin Bal, Gregory Destras, Claudia Gonzalez, Gwendolyne Burfin, Quentin Semanas, Martine Valette, Bruno Lina, Laurence Josset
EPI_ISL_728564, EPI_ISL_728638, EPI_ISL_728639, EPI_ISL_728640, EPI_ISL_728641, EPI_ISL_728643, EPI_ISL_728674, EPI_ISL_728675, EPI_ISL_728676, EPI_ISL_728677, EPI_ISL_728678, EPI_ISL_728679, EPI_ISL_728680, EPI_ISL_728681, EPI_ISL_728684, EPI_ISL_728685, EPI_ISL_728687, EPI_ISL_728688, EPI_ISL_728696, EPI_ISL_728708, EPI_ISL_728709, EPI_ISL_728710, EPI_ISL_728711, EPI_ISL_728712, EPI_ISL_728713, EPI_ISL_728714, EPI_ISL_728715, EPI_ISL_728716, EPI_ISL_728717, EPI_ISL_728718, EPI_ISL_728719, EPI_ISL_728720, EPI_ISL_728723, EPI_ISL_728724, EPI_ISL_728727, EPI_ISL_728733, EPI_ISL_728735, EPI_ISL_728736, EPI_ISL_728737, EPI_ISL_728748, EPI_ISL_728749, EPI_ISL_728750, EPI_ISL_728751, EPI_ISL_728752, EPI_ISL_728753, EPI_ISL_728754, EPI_ISL_728755, EPI_ISL_728757, EPI_ISL_728758, EPI_ISL_728759, EPI_ISL_728760, EPI_ISL_728761, EPI_ISL_728762			
see above	Dutch COVID-19 response team	National Institute for Public Health and the Environment (RIVM)	Adam Meijer, Harry Vennema, Jeroen Cremer, Sharon van den Brink, Bas van der Veer, AnneMarie van den Brandt, Florian Zwagemaker, Dennis Schmitz, Chantal Reusken, on behalf of the national COVID-19 response team
EPI_ISL_729265, EPI_ISL_729266, EPI_ISL_729271, EPI_ISL_729273, EPI_ISL_729274, EPI_ISL_729277, EPI_ISL_729278, EPI_ISL_729280, EPI_ISL_729284, EPI_ISL_729285, EPI_ISL_729290, EPI_ISL_729291, EPI_ISL_729292, EPI_ISL_729293, EPI_ISL_729294, EPI_ISL_729295, EPI_ISL_729296, EPI_ISL_729297, EPI_ISL_729298, EPI_ISL_729300, EPI_ISL_729301, EPI_ISL_729302, EPI_ISL_729304, EPI_ISL_729305, EPI_ISL_729306, EPI_ISL_729308, EPI_ISL_729310, EPI_ISL_729311, EPI_ISL_729312, EPI_ISL_729313, EPI_ISL_729315, EPI_ISL_729316, EPI_ISL_729317, EPI_ISL_729319, EPI_ISL_729321, EPI_ISL_729322, EPI_ISL_729323, EPI_ISL_729324, EPI_ISL_729325, EPI_ISL_729326, EPI_ISL_729327, EPI_ISL_729329, EPI_ISL_729331, EPI_ISL_729332, EPI_ISL_729333, EPI_ISL_729334			
see above	Toronto Invasive Bacterial Diseases Network	McMaster University	Allison McGeer, Patryk Aftanas, Hooman Derakhshani, Angel Li, Kuganya Nirmalarajah, Emily Panousis, Ahmed Draia, Jalees Nasir, Michael Surette, Samira Mubareka, Andrew G. McArthur
EPI_ISL_729420, EPI_ISL_729443, EPI_ISL_729445, EPI_ISL_729446, EPI_ISL_729447, EPI_ISL_729448, EPI_ISL_729452, EPI_ISL_729459, EPI_ISL_729645, EPI_ISL_729646, EPI_ISL_729648, EPI_ISL_729649, EPI_ISL_729650, EPI_ISL_729651, EPI_ISL_729652, EPI_ISL_729671, EPI_ISL_729672, EPI_ISL_729674, EPI_ISL_729675, EPI_ISL_729676, EPI_ISL_729677, EPI_ISL_729678, EPI_ISL_729679, EPI_ISL_729680, EPI_ISL_729681, EPI_ISL_729682, EPI_ISL_729684, EPI_ISL_729685, EPI_ISL_729687, EPI_ISL_729689, EPI_ISL_729690, EPI_ISL_729691, EPI_ISL_729692, EPI_ISL_729693, EPI_ISL_729694, EPI_ISL_729695, EPI_ISL_729696, EPI_ISL_729704, EPI_ISL_729715, EPI_ISL_729720, EPI_ISL_729722, EPI_ISL_729723, EPI_ISL_729728, EPI_ISL_729729, EPI_ISL_729730, EPI_ISL_729732, EPI_ISL_729733, EPI_ISL_729734			
see above	A. Krumbholz, Labor Dr. Krause und Kollegen MVZ GmbH, Kiel	Charité Universitätsmedizin Berlin, Institut für Virologie	Victor M Corman, Barbara Mühlemann, Jörn Beheim-Schwarzbach, Talitha Veith, Julia Schneider, Terry Jones, Christian Drosten
EPI_ISL_730322	San Diego County Public Health Laboratory	Andersen lab at Scripps Research	SEARCH Alliance San Diego with Tracy Basler, Jovan Shephard, Brett Austin
EPI_ISL_731251, EPI_ISL_731253, EPI_ISL_731892	Lighthouse Lab in Milton Keynes	Wellcome Sanger Institute for the COVID-19 Genomics UK (COG-UK) Consortium	The Lighthouse Lab in Milton Keynes and Alex Alderton, Roberto Amato, Sonia Goncalves, Ewan Harrison, David K. Jackson, Ian Johnston, Dominic Kwiatkowski, Cordelia Langford, John Sillitoe on behalf of the Wellcome Sanger Institute COVID-19 Surveillance Team
EPI_ISL_731894	Lighthouse Lab in Glasgow	Wellcome Sanger Institute for the COVID-19 Genomics UK (COG-UK) Consortium	Harper VanSteenhouse, Yumi Kasai, David Gray, Carol Clugston, Anna Dominiczak and Alex Alderton, Roberto Amato, Sonia Goncalves, Ewan Harrison, David K. Jackson, Ian Johnston, Dominic Kwiatkowski, Cordelia Langford, John Sillitoe on behalf of the Wellcome Sanger Institute COVID-19 Surveillance Team
EPI_ISL_731920	Instituto Nacional de Saude (INSA)	Instituto Nacional de Saude (INSA)	Borges et al
EPI_ISL_731925, EPI_ISL_731927, EPI_ISL_731928, EPI_ISL_731929, EPI_ISL_731930	Instituto Nacional de Saude (INSA) and Instituto Gulbenkian de Ciencia (IGC)	Instituto Nacional de Saude (INSA) and Instituto Gulbenkian de Ciencia (IGC)	Borges et al
EPI_ISL_731951, EPI_ISL_731998, EPI_ISL_731999, EPI_ISL_732008, EPI_ISL_732009, EPI_ISL_732010, EPI_ISL_732011, EPI_ISL_732111, EPI_ISL_732112, EPI_ISL_732113	Instituto Nacional de Saude (INSA)	Instituto Nacional de Saude (INSA)	Borges et al
EPI_ISL_732188, EPI_ISL_732189, EPI_ISL_732190, EPI_ISL_732191, EPI_ISL_732192, EPI_ISL_732193, EPI_ISL_732194, EPI_ISL_732195, EPI_ISL_732219, EPI_ISL_732220, EPI_ISL_732232, EPI_ISL_732233, EPI_ISL_732234, EPI_ISL_732235, EPI_ISL_732236, EPI_ISL_732237, EPI_ISL_732238, EPI_ISL_732239, EPI_ISL_732240, EPI_ISL_732241, EPI_ISL_732242, EPI_ISL_732243, EPI_ISL_732244, EPI_ISL_732245			
see above	Instituto Nacional de Saude (INSA) and Instituto Gulbenkian de Ciencia (IGC)	Instituto Nacional de Saude (INSA) and Instituto Gulbenkian de Ciencia (IGC)	Borges et al
EPI_ISL_732472, EPI_ISL_732493, EPI_ISL_732497, EPI_ISL_732498, EPI_ISL_732499, EPI_ISL_732500, EPI_ISL_732503, EPI_ISL_732505, EPI_ISL_732506, EPI_ISL_732508, EPI_ISL_732509, EPI_ISL_732512, EPI_ISL_732513, EPI_ISL_732514, EPI_ISL_732515, EPI_ISL_732522			
see above	National Virus Reference Laboratory	National Virus Reference Laboratory	Michael Carr, Gabriel Gonzalez, Jonathan Dean, Daniel Hare, Cillian F De Gascun
EPI_ISL_732654	Klinisk mikrobiologi	The Public Health Agency of Sweden	Department of Microbiology, The Public Health Agency of Sweden
EPI_ISL_732655	Narhalsan Fjallbacka VC	The Public Health Agency of Sweden	Department of Microbiology, The Public Health Agency of Sweden
EPI_ISL_732686, EPI_ISL_732687, EPI_ISL_732688, EPI_ISL_732689, EPI_ISL_732690, EPI_ISL_732691,	CNR Virus des Infections Respiratoires - France SUD	CNR Virus des Infections Respiratoires - France SUD	Antonin Bal, Gregory Destras, Claudia Gonzalez, Gwendolyne Burfin, Quentin Semanas, Martine Valette, Bruno Lina, Laurence Josset

EPI_ISL_732692			
EPI_ISL_732949, EPI_ISL_732951, EPI_ISL_732952, EPI_ISL_732954, EPI_ISL_732955, EPI_ISL_732956, EPI_ISL_732957, EPI_ISL_732958, EPI_ISL_732959	Department of Tropical Parasitology	Laboratory of Recombinant Vaccines	Lukasz Rabalski, Maciej Kosinski, Teemu Smura, Kirsi Aaltonen, Ravi Kant, Tarja Sironen, Boguslaw Szewczyk, Maciej Grzybek
EPI_ISL_732975, EPI_ISL_732980, EPI_ISL_732982, EPI_ISL_732987, EPI_ISL_732988, EPI_ISL_733466	WHO National Influenza Centre Russian Federation	WHO National Influenza Centre Russian Federation	Andrey Komissarov, Artem Fadeev, Anna Ivanova, Kseniya Komissarova, Dmitry Bazhenov, Daria Danilenko, Ksenia Safina, Elena Nabieva, Georgii Bazykin, Dmitry Lioznov
EPI_ISL_733493	Omsk Research Institute of Natural Focal Infections	WHO National Influenza Centre Russian Federation	Andrey Komissarov, Artem Fadeev, Anna Ivanova, Kseniya Komissarova, Dmitry Bazhenov, Daria Danilenko, Ekaterina Gradoboeva, Elena Poleshchuk, Aleksei Vasilenko, Valery Yakimenko, Ksenia Safina, Elena Nabieva, Georgii Bazykin, Dmitry Lioznov
EPI_ISL_734170, EPI_ISL_734172, EPI_ISL_734174, EPI_ISL_734180, EPI_ISL_734181, EPI_ISL_734187, EPI_ISL_734189, EPI_ISL_734191, EPI_ISL_734192, EPI_ISL_734193, EPI_ISL_734195, EPI_ISL_734196, EPI_ISL_734198, EPI_ISL_734200, EPI_ISL_734225			
see above	Virginia Division of Consolidated Laboratory Services (DCLS)	Virginia Division of Consolidated Laboratory Services (DCLS)	Virginia DCLS
EPI_ISL_734481	Masonic Medical Research Institute	Wadsworth Center, New York State Department.of Health	Kirsten St. George, Nathan Tucker, Ryan D. Pfeiffer, Daryl M. Lamson, Alexis Russel, Jonathan Plitnick, Navjot Singh, John Kelly, Sara Griesemer, Erasmus Schneider, Erica Lasek-Nesselquist
EPI_ISL_735221, EPI_ISL_735222, EPI_ISL_735224, EPI_ISL_735225, EPI_ISL_735226, EPI_ISL_735227, EPI_ISL_735228, EPI_ISL_735229, EPI_ISL_735230, EPI_ISL_735231, EPI_ISL_735232, EPI_ISL_735233, EPI_ISL_735234, EPI_ISL_735235, EPI_ISL_735236, EPI_ISL_735237, EPI_ISL_735238			
see above	UZ Leuven, National Reference Laboratory for Coronaviruses, Laboratory Medicine, Leuven, Belgium	KU Leuven, Rega Institute, Clinical and Epidemiological Virology	Tony Wawina-Bokalanga, Joan Marti-Carerras, Bert Vanmechelen, Piet Maes
EPI_ISL_735490	Rangamati General Hospital RT-PCR lab,	Central Biological Research Laboratory and Department of Biochemistry and Molecular Biology	Robiul Hasan Bhuiyan, Md. Imranul Hoq, Md. Khondakar Raziur Rahman, Imam Hossen, Sajib Rudra, Md. Arif Hossain, Shanta Paul, Md. Omer Faruq, H. M. Abdullah Al Masud, Mohammad Omar Faruque
EPI_ISL_735493	Cox's Bazar Medicila College	Central Biological Research Laboratory and Department of Biochemistry and Molecular Biology Central Biological Research Laboratory and Department of Biochemistry and Molecular Biology	Robiul Hasan Bhuiyan, Md. Imranul Hoq, Md. Khondakar Raziur Rahman, Imam Hossen, Sajib Rudra, Md. Arif Hossain, Shanta Paul, Md. Omer Faruq, H. M. Abdullah Al Masud, Mohammad Omar Faruque
EPI_ISL_735494	Cox's Bazar Medical College	Central Biological Research Laboratory and Department of Biochemistry and Molecular Biology	Md. Imranul Hoq, Robiul Hasan Bhuiyan, Md. Khondakar Raziur Rahman, Imam Hossen, Sajib Rudra, Md. Arif Hossain, Shanta Paul, Md. Omer Faruq, Mohammad Omar Faruque, H. M. Abdullah Al Masud
EPI_ISL_735496	Chattogram Veterinary and Animal Sciences University	Central Biological Research Laboratory and Department of Biochemistry and Molecular Biology	Md. Imranul Hoq, Robiul Hasan Bhuiyan, Md. Khondakar Raziur Rahman, Imam Hossen, Sajib Rudra, Md. Arif Hossain, Shanta Paul, Md. Omer Faruq, Mohammad Omar Faruque, H. M. Abdullah Al Masud
EPI_ISL_735497	Chattogram Veterinary and Animal Sciences University	Central Biological Research Laboratory and Department of Biochemistry and Molecular Biology	Mohammad Omar Faruque, H. M. Abdullah Al Masud, Imam Hossen, Md. Khondakar Raziur Rahman, Sajib Rudra, Md. Arif Hossain, Shanta Paul, Md. Omer Faruq, Robiul Hasan Bhuiyan, Md. Imranul Hoq
EPI_ISL_736926, EPI_ISL_736994	NHLS-IALCH	KRISP, KZN Research Innovation and Sequencing Platform	Giandhari J, Pillay S, Lessells R, ChimukangaraB, Mdlalose K, York D, Khan S, Tegally H, Wilkinson E, de Oliveira T
EPI_ISL_737182	Michigan Department of Health and Human Services, Bureau of Laboratories	Michigan Department of Health and Human Services, Bureau of Laboratories	Blankenship HM, Riner D, Soehnlen MK
EPI_ISL_737236	Los Angeles County PHL	Los Angeles County PHL	P. Hemarajata et al.
EPI_ISL_737853	Viollier AG	Department of Biosystems Science and Engineering, ETH Zürich	Chaoran Chen, Sarah Nadeau, Catharine Aquino, Ivan Topolsky, Philipp Jablonski, Lara Fuhrmann, David Dreifuss, Katharina Jahn, Andreia Cabral de Gouvea, Maria Domenica Moccia, Simon Grüter, Timothy Sykes, Lennart Opitz, Griffin White, Laura Neff, Doris Popovic, Andrea Patrignani, Jay Tracy, Ralph Schlapbach, Christiane Beckmann, Maurice Redondo, Olivier Kobel, Christoph Noppen, Sophie Seidel, Noemie Santamaria de Souza, Niko Beerenwinkel, Tanja Stadler
EPI_ISL_738064, EPI_ISL_738065	Department of Laboratory Medicine, National Taiwan University Hospital	Microbial Genomics Core Lab, National Taiwan University Centers of Genomic and Precision Medicine	Shiou-Hwei Yeh, You-Yu Lin, Ya-Yun Lai, Chiao-Ling Li, Shan-Chwen Chang, Pei-Jer Chen, Sui-Yuan Chang
EPI_ISL_738326, EPI_ISL_738327, EPI_ISL_738328, EPI_ISL_738329, EPI_ISL_738330, EPI_ISL_738331, EPI_ISL_738332, EPI_ISL_738333, EPI_ISL_738335, EPI_ISL_738336, EPI_ISL_738337, EPI_ISL_738338, EPI_ISL_738339, EPI_ISL_738340, EPI_ISL_738341, EPI_ISL_738342, EPI_ISL_738343, EPI_ISL_738344, EPI_ISL_738345, EPI_ISL_738346, EPI_ISL_738347, EPI_ISL_738348, EPI_ISL_738349			
see above	Landstuhl Regional Medical Center	United States Air Force School of Aerospace Medicine	Anthony Fries, Jennifer Meyer, Amanda Javorina, Sarah Purves, William Gruner, Clarise Starr, Elizabeth Macias, Fritz Castillo, Cole Anderson
EPI_ISL_738714, EPI_ISL_738723	Renegade	Chan-Zuckerberg Biohub	CZB Cliahub Consortium
EPI_ISL_738729, EPI_ISL_738755, EPI_ISL_738761	Orange County Public Health Lab	Chan-Zuckerberg Biohub	CZB Cliahub Consortium
EPI_ISL_738770	Renegade	Chan-Zuckerberg Biohub	CZB Cliahub Consortium
EPI_ISL_738771, EPI_ISL_738805	Santa Clara County Public Health Laboratory	Chan-Zuckerberg Biohub	CZB Cliahub Consortium
EPI_ISL_738811, EPI_ISL_738818, EPI_ISL_738823, EPI_ISL_738840	Orange County Public Health Lab	Chan-Zuckerberg Biohub	CZB Cliahub Consortium
EPI_ISL_738842	Santa Clara County Public Health Laboratory	Chan-Zuckerberg Biohub	CZB Cliahub Consortium
EPI_ISL_738860	Orange County Public Health Lab	Chan-Zuckerberg Biohub	CZB Cliahub Consortium
EPI_ISL_738864	Santa Clara County Public Health Laboratory	Chan-Zuckerberg Biohub	CZB Cliahub Consortium
EPI_ISL_738877	Renegade	Chan-Zuckerberg Biohub	CZB Cliahub Consortium
EPI_ISL_738893, EPI_ISL_738899	Orange County Public Health Lab	Chan-Zuckerberg Biohub	CZB Cliahub Consortium
EPI_ISL_738903	Santa Clara County Public Health Laboratory	Chan-Zuckerberg Biohub	CZB Cliahub Consortium
EPI_ISL_738905, EPI_ISL_738908, EPI_ISL_738928	Orange County Public Health Lab	Chan-Zuckerberg Biohub	CZB Cliahub Consortium
EPI_ISL_738941, EPI_ISL_738955	Santa Clara County Public Health Laboratory	Chan-Zuckerberg Biohub	CZB Cliahub Consortium
EPI_ISL_738968	Orange County Public Health Lab	Chan-Zuckerberg Biohub	CZB Cliahub Consortium
EPI_ISL_738984	Santa Clara County Public Health Laboratory	Chan-Zuckerberg Biohub	CZB Cliahub Consortium
EPI_ISL_738991	Orange County Public Health Lab	Chan-Zuckerberg Biohub	CZB Cliahub Consortium
EPI_ISL_738994, EPI_ISL_739008	Santa Clara County Public Health Laboratory	Chan-Zuckerberg Biohub	CZB Cliahub Consortium
EPI_ISL_739014	Renegade	Chan-Zuckerberg Biohub	CZB Cliahub Consortium
EPI_ISL_739034	Orange County Public Health Lab	Chan-Zuckerberg Biohub	CZB Cliahub Consortium
EPI_ISL_739053	Santa Clara County Public Health Laboratory	Chan-Zuckerberg Biohub	CZB Cliahub Consortium
EPI_ISL_739063	Orange County Public Health Lab	Chan-Zuckerberg Biohub	CZB Cliahub Consortium

EPI_ISL_739066	Santa Clara County Public Health Laboratory	Chan-Zuckerberg Biohub	CZB Cliahub Consortium
EPI_ISL_739069, EPI_ISL_739071	Orange County Public Health Lab	Chan-Zuckerberg Biohub	CZB Cliahub Consortium
EPI_ISL_739076	Santa Clara County Public Health Laboratory	Chan-Zuckerberg Biohub	CZB Cliahub Consortium
EPI_ISL_739094, EPI_ISL_739097	Orange County Public Health Lab	Chan-Zuckerberg Biohub	CZB Cliahub Consortium
EPI_ISL_739123	Humboldt County Public Health Laboratory	Chan-Zuckerberg Biohub	CZB Cliahub Consortium
EPI_ISL_739125	Orange County Public Health Lab	Chan-Zuckerberg Biohub	CZB Cliahub Consortium
EPI_ISL_739146, EPI_ISL_739153	Santa Clara County Public Health Laboratory	Chan-Zuckerberg Biohub	CZB Cliahub Consortium
EPI_ISL_739154, EPI_ISL_739191	Orange County Public Health Lab	Chan-Zuckerberg Biohub	CZB Cliahub Consortium
EPI_ISL_739204	Santa Clara County Public Health Laboratory	Chan-Zuckerberg Biohub	CZB Cliahub Consortium
EPI_ISL_739211, EPI_ISL_739222, EPI_ISL_739230, EPI_ISL_739238	Orange County Public Health Lab	Chan-Zuckerberg Biohub	CZB Cliahub Consortium
EPI_ISL_739252, EPI_ISL_739253	Santa Clara County Public Health Laboratory	Chan-Zuckerberg Biohub	CZB Cliahub Consortium
EPI_ISL_739266	Orange County Public Health Lab	Chan-Zuckerberg Biohub	CZB Cliahub Consortium
EPI_ISL_739269	Humboldt County Public Health Laboratory	Chan-Zuckerberg Biohub	CZB Cliahub Consortium
EPI_ISL_739276	Orange County Public Health Lab	Chan-Zuckerberg Biohub	CZB Cliahub Consortium
EPI_ISL_739277	Santa Clara County Public Health Laboratory	Chan-Zuckerberg Biohub	CZB Cliahub Consortium
EPI_ISL_739289	Orange County Public Health Lab	Chan-Zuckerberg Biohub	CZB Cliahub Consortium
EPI_ISL_739313	Humboldt County Public Health Laboratory	Chan-Zuckerberg Biohub	CZB Cliahub Consortium
EPI_ISL_739316	Renegade	Chan-Zuckerberg Biohub	CZB Cliahub Consortium
EPI_ISL_739320, EPI_ISL_739327	Santa Clara County Public Health Laboratory	Chan-Zuckerberg Biohub	CZB Cliahub Consortium
EPI_ISL_739379	Orange County Public Health Lab	Chan-Zuckerberg Biohub	CZB Cliahub Consortium
EPI_ISL_739380, EPI_ISL_739381	Santa Clara County Public Health Laboratory	Chan-Zuckerberg Biohub	CZB Cliahub Consortium
EPI_ISL_739403	Humboldt County Public Health Laboratory	Chan-Zuckerberg Biohub	CZB Cliahub Consortium
EPI_ISL_739410	Orange County Public Health Lab	Chan-Zuckerberg Biohub	CZB Cliahub Consortium
EPI_ISL_739416	Santa Clara County Public Health Laboratory	Chan-Zuckerberg Biohub	CZB Cliahub Consortium
EPI_ISL_739441	Orange County Public Health Lab	Chan-Zuckerberg Biohub	CZB Cliahub Consortium
EPI_ISL_739457	Santa Clara County Public Health Laboratory	Chan-Zuckerberg Biohub	CZB Cliahub Consortium
EPI_ISL_739466	Renegade	Chan-Zuckerberg Biohub	CZB Cliahub Consortium
EPI_ISL_739476	Santa Clara County Public Health Laboratory	Chan-Zuckerberg Biohub	CZB Cliahub Consortium
EPI_ISL_739478	Renegade	Chan-Zuckerberg Biohub	CZB Cliahub Consortium
EPI_ISL_739482	Orange County Public Health Lab	Chan-Zuckerberg Biohub	CZB Cliahub Consortium
EPI_ISL_739487, EPI_ISL_739491	Santa Clara County Public Health Laboratory	Chan-Zuckerberg Biohub	CZB Cliahub Consortium
EPI_ISL_739513, EPI_ISL_739524	Orange County Public Health Lab	Chan-Zuckerberg Biohub	CZB Cliahub Consortium
EPI_ISL_739543	Santa Clara County Public Health Laboratory	Chan-Zuckerberg Biohub	CZB Cliahub Consortium
EPI_ISL_739547, EPI_ISL_739559	Orange County Public Health Lab	Chan-Zuckerberg Biohub	CZB Cliahub Consortium
EPI_ISL_739562	Santa Clara County Public Health Laboratory	Chan-Zuckerberg Biohub	CZB Cliahub Consortium
EPI_ISL_739564	Renegade	Chan-Zuckerberg Biohub	CZB Cliahub Consortium
EPI_ISL_739568, EPI_ISL_739570, EPI_ISL_739573, EPI_ISL_739577	Orange County Public Health Lab	Chan-Zuckerberg Biohub	CZB Cliahub Consortium
EPI_ISL_739579	Santa Clara County Public Health Laboratory	Chan-Zuckerberg Biohub	CZB Cliahub Consortium
EPI_ISL_739582	Orange County Public Health Lab	Chan-Zuckerberg Biohub	CZB Cliahub Consortium
EPI_ISL_739584, EPI_ISL_739596	Santa Clara County Public Health Laboratory	Chan-Zuckerberg Biohub	CZB Cliahub Consortium
EPI_ISL_739607	Orange County Public Health Lab	Chan-Zuckerberg Biohub	CZB Cliahub Consortium
EPI_ISL_739612	Santa Clara County Public Health Laboratory	Chan-Zuckerberg Biohub	CZB Cliahub Consortium
EPI_ISL_739625, EPI_ISL_739627, EPI_ISL_739636	Orange County Public Health Lab	Chan-Zuckerberg Biohub	CZB Cliahub Consortium
EPI_ISL_739650	Santa Clara County Public Health Laboratory	Chan-Zuckerberg Biohub	CZB Cliahub Consortium
EPI_ISL_739655	Renegade	Chan-Zuckerberg Biohub	CZB Cliahub Consortium
EPI_ISL_739660, EPI_ISL_739661	Al-Quds Nutrition and Health Research Institute, Al-Quds University	Al-Quds Nutrition and Health Research Institute, Al-Quds University	Nasereddin,A., Ereqat,S., Al-Jawabreh,A., Rishmawi,C.
EPI_ISL_739694, EPI_ISL_739697, EPI_ISL_739706, EPI_ISL_739707, EPI_ISL_739708, EPI_ISL_739710, EPI_ISL_739719, EPI_ISL_739721, EPI_ISL_739723, EPI_ISL_739730, EPI_ISL_739740, EPI_ISL_739741, EPI_ISL_739742, EPI_ISL_739752, EPI_ISL_739759, EPI_ISL_739763, EPI_ISL_739764, EPI_ISL_739771, EPI_ISL_739772, EPI_ISL_739775, EPI_ISL_739776, EPI_ISL_739793, EPI_ISL_739797, EPI_ISL_739801, EPI_ISL_739805, EPI_ISL_739811, EPI_ISL_739821, EPI_ISL_739823, EPI_ISL_739842, EPI_ISL_739846, EPI_ISL_739847, EPI_ISL_739849, EPI_ISL_739857, EPI_ISL_739858, EPI_ISL_739865, EPI_ISL_739872, EPI_ISL_739876, EPI_ISL_739879, EPI_ISL_739885, EPI_ISL_739887, EPI_ISL_739888, EPI_ISL_739890, EPI_ISL_739893, EPI_ISL_739899, EPI_ISL_739901, EPI_ISL_739907, EPI_ISL_739921, EPI_ISL_739928, EPI_ISL_739932, EPI_ISL_739935, EPI_ISL_739938, EPI_ISL_739942, EPI_ISL_739950, EPI_ISL_739960, EPI_ISL_739966, EPI_ISL_739972, EPI_ISL_739979, EPI_ISL_739984, EPI_ISL_739986, EPI_ISL_739989, EPI_ISL_739991, EPI_ISL_740011, EPI_ISL_740012, EPI_ISL_740029, EPI_ISL_740035, EPI_ISL_740059, EPI_ISL_740065, EPI_ISL_740072, EPI_ISL_740075, EPI_ISL_740083, EPI_ISL_740088, EPI_ISL_740089, EPI_ISL_740094, EPI_ISL_740102, EPI_ISL_740112, EPI_ISL_740119, EPI_ISL_740128, EPI_ISL_740140, EPI_ISL_740143, EPI_ISL_740148, EPI_ISL_740151, EPI_ISL_740152, EPI_ISL_740158, EPI_ISL_740163, EPI_ISL_740164, EPI_ISL_740167, EPI_ISL_740174, EPI_ISL_740177, EPI_ISL_740211, EPI_ISL_740212, EPI_ISL_740213, EPI_ISL_740216, EPI_ISL_740223, EPI_ISL_740228, EPI_ISL_740235, EPI_ISL_740236, EPI_ISL_740241, EPI_ISL_740253, EPI_ISL_740255, EPI_ISL_740257, EPI_ISL_740284, EPI_ISL_740285, EPI_ISL_740297, EPI_ISL_740299, EPI_ISL_740301, EPI_ISL_740303, EPI_ISL_740309, EPI_ISL_740311, EPI_ISL_740312, EPI_ISL_740315, EPI_ISL_740325, EPI_ISL_740335, EPI_ISL_740339, EPI_ISL_740341, EPI_ISL_740350, EPI_ISL_740351, EPI_ISL_740363, EPI_ISL_740367, EPI_ISL_740371, EPI_ISL_740374, EPI_ISL_740376, EPI_ISL_740378, EPI_ISL_740379, EPI_ISL_740380, EPI_ISL_740385, EPI_ISL_740389, EPI_ISL_740392, EPI_ISL_740393, EPI_ISL_740395, EPI_ISL_740400, EPI_ISL_740411, EPI_ISL_740413, EPI_ISL_740416, EPI_ISL_740420, EPI_ISL_740422, EPI_ISL_740426, EPI_ISL_740428, EPI_ISL_740437, EPI_ISL_740446, EPI_ISL_740452, EPI_ISL_740454, EPI_ISL_740458, EPI_ISL_740459, EPI_ISL_740463, EPI_ISL_740472, EPI_ISL_740476, EPI_ISL_740483, EPI_ISL_740484, EPI_ISL_740494, EPI_ISL_740505, EPI_ISL_740517, EPI_ISL_740523, EPI_ISL_740524, EPI_ISL_740529, EPI_ISL_740541			
see above	Laboratoire national de santé, Microbiology, Virology	Laboratoire national de santé, Microbiology, Microbial Genomics Platform	Anke Wienecke-Baldacchino, Catherine Ragimbeau,Jessica Tapp, Fatu Djabi, Lise Pignon, Raoul Salmon, Tamir Abdelrahman
EPI_ISL_740547	Department of Laboratory Medicine, National Taiwan University Hospital	Microbial Genomics Core Lab, National Taiwan University Centers of Genomic and Precision Medicine	Shiou-Hwei Yeh, You-Yu Lin, Ya-Yun Lai, Chiao-Ling Li, Shan-Chwen Chang, Pei-Jer Chen, Sui-Yuan Chang

EPI_ISL_741436, EPI_ISL_741441, EPI_ISL_741442, EPI_ISL_741447, EPI_ISL_741448	Northumbria University / South Tees Hospitals NHS Foundation Trust / North Cumbria Integrated Care NHS Foundation Trust / North Tees and Hartlepool NHS Foundation Trust / Newcastle Hospitals NHS Foundation Trust	COVID-19 Genomics UK (COG-UK) Consortium	Darren L Smith,Andrew Nelson,Matthew Bashton,Greg R Young,Joshua Loh,John Allan,Mohammad A Tariq,Giles S Holt,Gary Black,Wen C Yew,Lynn Dover,Paul Baker,Steve Liggett,Sarah Essex,Jane Greenaway,Debra Padgett,Clive Graham,Garren Scott,Edward Barton,Emma Swindells,Brendan Payne,Jennifer Collins,Yusri Taha,Gary Eltringham
EPI_ISL_741611	Queens Medical Centre, Clinical Microbiology Department / DeepSeq Nottingham	COVID-19 Genomics UK (COG-UK) Consortium	Gemma Clark, Wendy Smith, Manjinder Khakh, Vicki M Fleming, Michelle M Lister, Hannah Howson-Wells, Jonathan Ball, Patrick McClure, Joseph Chappell, Theocharis Tsoleridis, Nadine Holmes, Matthew Carlisle, Christopher Moore, Fei Sang, Johnny Debebe, Victoria Wright, Matthew Loose
EPI_ISL_741717, EPI_ISL_741740, EPI_ISL_741758	Centre for Enzyme Innovation, University of Portsmouth / Translational Research Laboratory, Portsmouth Hospitals NHS Trust	COVID-19 Genomics UK (COG-UK) Consortium	Angela Beckett,Yann Bourgeois,Garry Scarlett,Sharon Glaysher,Scott Elliott,Kelly Bicknell,Robert Impey,Allyson Lloyd,Sarah Wyllie,Ethan Butcher,Anoop Chauhan,Samuel Robson
EPI_ISL_742542, EPI_ISL_742547, EPI_ISL_742585, EPI_ISL_742588, EPI_ISL_742592, EPI_ISL_742594, EPI_ISL_742606, EPI_ISL_742607, EPI_ISL_742611, EPI_ISL_742612, EPI_ISL_742617, EPI_ISL_742843, EPI_ISL_742880			
see above	Wales Specialist Virology Centre Sequencing lab: Pathogen Genomics Unit	COVID-19 Genomics UK (COG-UK) Consortium	Catherine Moore, Johnathan Evans, Laura Gifford, Malorie Perry, Simon Cottrell, Angela Marchbank, Alec Birchley, Alexander Adams, Amy Gaskin, Bree Gatica-Wilcox, Jason Coombes, Joel Southgate, Lauren Gilbert, Lee Graham, Nicole Pacchiarini, Sara Kumziene-Summerhayes, Sarah Taylor, Sophie Jones, Sara Rey, Matthew Bull, Joanne Watkins, Sally Corden, Tom Connor
EPI_ISL_744137, EPI_ISL_744143, EPI_ISL_744148, EPI_ISL_744162, EPI_ISL_744176, EPI_ISL_744177, EPI_ISL_744179, EPI_ISL_744183, EPI_ISL_744186, EPI_ISL_744199, EPI_ISL_744210, EPI_ISL_744225, EPI_ISL_744230, EPI_ISL_744239, EPI_ISL_744244, EPI_ISL_744247, EPI_ISL_744248, EPI_ISL_744250, EPI_ISL_744251, EPI_ISL_744254, EPI_ISL_744255, EPI_ISL_744256, EPI_ISL_744263, EPI_ISL_744267, EPI_ISL_744268, EPI_ISL_744271, EPI_ISL_744273, EPI_ISL_744276, EPI_ISL_744277, EPI_ISL_744286, EPI_ISL_744293, EPI_ISL_744299, EPI_ISL_744310, EPI_ISL_744314, EPI_ISL_744318, EPI_ISL_744319, EPI_ISL_744322, EPI_ISL_744328, EPI_ISL_744331, EPI_ISL_744333, EPI_ISL_744335, EPI_ISL_744338, EPI_ISL_744342, EPI_ISL_744345, EPI_ISL_744346, EPI_ISL_744349, EPI_ISL_744355, EPI_ISL_744356, EPI_ISL_744360, EPI_ISL_744390, EPI_ISL_744404, EPI_ISL_744407, EPI_ISL_744415, EPI_ISL_744424, EPI_ISL_744426, EPI_ISL_744427, EPI_ISL_744431, EPI_ISL_744432, EPI_ISL_744449, EPI_ISL_744450, EPI_ISL_744453, EPI_ISL_744462, EPI_ISL_744468, EPI_ISL_744476, EPI_ISL_744479, EPI_ISL_744494, EPI_ISL_744499, EPI_ISL_744504, EPI_ISL_744508, EPI_ISL_744518, EPI_ISL_744525, EPI_ISL_744526, EPI_ISL_744540, EPI_ISL_744542, EPI_ISL_744544, EPI_ISL_744545, EPI_ISL_744554, EPI_ISL_744558, EPI_ISL_744564, EPI_ISL_744573, EPI_ISL_744576, EPI_ISL_744580, EPI_ISL_744592, EPI_ISL_744593, EPI_ISL_744603, EPI_ISL_744606, EPI_ISL_744608, EPI_ISL_744614, EPI_ISL_744629, EPI_ISL_744634, EPI_ISL_744635, EPI_ISL_744648, EPI_ISL_744657, EPI_ISL_744665, EPI_ISL_744667, EPI_ISL_744689, EPI_ISL_744692, EPI_ISL_744698, EPI_ISL_744704, EPI_ISL_744707, EPI_ISL_744708, EPI_ISL_744714, EPI_ISL_744715, EPI_ISL_744717, EPI_ISL_744728, EPI_ISL_744730, EPI_ISL_744737, EPI_ISL_744743, EPI_ISL_744747, EPI_ISL_744765, EPI_ISL_744768, EPI_ISL_744771, EPI_ISL_744776, EPI_ISL_744795, EPI_ISL_744797, EPI_ISL_744798, EPI_ISL_744802, EPI_ISL_744803, EPI_ISL_744807, EPI_ISL_744817, EPI_ISL_744827, EPI_ISL_744834, EPI_ISL_744838, EPI_ISL_744839, EPI_ISL_744840, EPI_ISL_744844, EPI_ISL_744845, EPI_ISL_744850, EPI_ISL_744853, EPI_ISL_744856, EPI_ISL_744871, EPI_ISL_744878, EPI_ISL_744880, EPI_ISL_744889, EPI_ISL_744898, EPI_ISL_744900, EPI_ISL_744915, EPI_ISL_744921, EPI_ISL_744923, EPI_ISL_744927, EPI_ISL_744930, EPI_ISL_744933, EPI_ISL_744940, EPI_ISL_744944, EPI_ISL_744950, EPI_ISL_744953, EPI_ISL_744959, EPI_ISL_744965, EPI_ISL_744966, EPI_ISL_744972, EPI_ISL_744975, EPI_ISL_744976, EPI_ISL_744977, EPI_ISL_744980, EPI_ISL_744982, EPI_ISL_744983, EPI_ISL_744986, EPI_ISL_744996, EPI_ISL_745000, EPI_ISL_745008, EPI_ISL_745009, EPI_ISL_745021, EPI_ISL_745026			
see above	Laboratoire national de santé, Microbiology, Virology	Laboratoire national de santé, Microbiology, Microbial Genomics Platform	Anke Wienecke-Baldacchino, Catherine Ragimbeau,Jessica Tapp, Fatu Djabi, Lise Pignon, Raoul Salmon, Tamir Abdelrahman
EPI_ISL_745326	CNR Virus des Infections Respiratoires - France SUD	CNR Virus des Infections Respiratoires - France SUD	Antonin Bal, Gregory Destras, Claudia Gonzalez, Gwendolyne Burfin, Quentin Semanas, Martine Valette, Bruno Lina, Laurence Josset
EPI_ISL_745335	CHU Clermont-Ferrand	CNR Virus des Infections Respiratoires - France SUD	Antonin Bal, Gregory Destras, Gwendolyne Burfin, Hadrien Règue, Quentin Semanas, Martine Valette, Bruno Lina, Christine Archimbaud, Amélie Brebion, Hélène Chabrolles, Martine Chambon, Audrey Mirand, Christel Regagnon, Maxime Bisseux, Patricia Combes, Cécile Henquell, Laurence Josset
EPI_ISL_745342, EPI_ISL_745344	CNR Virus des Infections Respiratoires - France SUD	CNR Virus des Infections Respiratoires - France SUD	Antonin Bal, Gregory Destras, Claudia Gonzalez, Gwendolyne Burfin, Quentin Semanas, Martine Valette, Bruno Lina, Laurence Josset
EPI_ISL_745409	DOHMH Corona	New York City Public Health Laboratory	Jade Wang, et al.
EPI_ISL_745412	DOHMH Morrisania	New York City Public Health Laboratory	Jade Wang, et al.
EPI_ISL_745413	DOHMH Central Harlem	New York City Public Health Laboratory	Jade Wang, et al.
EPI_ISL_745414, EPI_ISL_745415	DOHMH Jamaica	New York City Public Health Laboratory	Jade Wang, et al.
EPI_ISL_745419, EPI_ISL_745420	DOHMH Crown Heights	New York City Public Health Laboratory	Jade Wang, et al.
EPI_ISL_745421, EPI_ISL_745422	DOHMH Chelsea	New York City Public Health Laboratory	Jade Wang, et al.
EPI_ISL_745424	DOHMH Corona	New York City Public Health Laboratory	Jade Wang, et al.
EPI_ISL_745425	DOHMH Central Harlem	New York City Public Health Laboratory	Jade Wang, et al.
EPI_ISL_745426	DOHMH Corona	New York City Public Health Laboratory	Jade Wang, et al.
EPI_ISL_745430, EPI_ISL_745431, EPI_ISL_745433	DOHMH Morrisania	New York City Public Health Laboratory	Jade Wang, et al.
EPI_ISL_745434, EPI_ISL_745435	DOHMH Corona	New York City Public Health Laboratory	Jade Wang, et al.
EPI_ISL_745436	DOHMH Chelsea	New York City Public Health Laboratory	Jade Wang, et al.
EPI_ISL_745437	DOHMH PHL	New York City Public Health Laboratory	Jade Wang, et al.
EPI_ISL_745440	DOHMH Jamaica	New York City Public Health Laboratory	Jade Wang, et al.
EPI_ISL_745445	DOHMH Crown Heights	New York City Public Health Laboratory	Jade Wang, et al.
EPI_ISL_746428, EPI_ISL_746961, EPI_ISL_746965, EPI_ISL_746980, EPI_ISL_746995, EPI_ISL_747014	Utah Public Health Laboratory	Utah Public Health Laboratory	Erin Young, Kelly Oakeson, Tara Gallagher
EPI_ISL_747157, EPI_ISL_747161, EPI_ISL_747162, EPI_ISL_747163, EPI_ISL_747168, EPI_ISL_747170, EPI_ISL_747171, EPI_ISL_747172, EPI_ISL_747182, EPI_ISL_747183, EPI_ISL_747184, EPI_ISL_747187, EPI_ISL_747193			
see above	Respiratory Viruses Branch, Centers for Disease Control and Prevention	Respiratory Viruses Branch, Centers for Disease Control and Prevention	Queen,K., Li,Y., Tao,Y., Uehara,A., Montmayeur,A., Paden,C.R., Cook,P.W., Marine,R., Sheth,M., Wang,H., Lee,J., Tong,S.
EPI_ISL_747398, EPI_ISL_747425, EPI_ISL_747427, EPI_ISL_747429, EPI_ISL_747436, EPI_ISL_747437, EPI_ISL_747438, EPI_ISL_747439, EPI_ISL_747442, EPI_ISL_747443, EPI_ISL_747444, EPI_ISL_747445, EPI_ISL_747446, EPI_ISL_747452, EPI_ISL_747453, EPI_ISL_747454, EPI_ISL_747458			
see above	Division of Emerging Infectious Diseases, Bureau of Infectious Diseases Diagnosis Control, Korea Disease Control and Prevention Agency	Division of Emerging Infectious Diseases, Bureau of Infectious Diseases Diagnosis Control, Korea Disease Control and Prevention Agency	Ae Kyung Park, Il-Hwan Kim, Heui Man Kim, Jeong-Min Kim, Namjoo Lee, Chaeyoung Lee, Sang Hee Woo, Eun-Jin Kim
EPI_ISL_747459, EPI_ISL_747460, EPI_ISL_747461, EPI_ISL_747462, EPI_ISL_747464	Ospedale San Bonifacio	Istituto Zooprofilattico Sperimentale delle Venezie	Adelaide Milani, Alessia Schivo, Annalisa Salviato, Erika Giorgia Quaranta, Ambra Pastori, Bianca Zecchin, Alice Fusaro, Isabella Monne, Calogero Terregino, Antonia Ricci
EPI_ISL_747471, EPI_ISL_747472, EPI_ISL_747473, EPI_ISL_747474, EPI_ISL_747475, EPI_ISL_747476	ULSS9 Distretto di Bussolengo	Istituto Zooprofilattico Sperimentale delle Venezie	Adelaide Milani, Alessia Schivo, Annalisa Salviato, Erika Giorgia Quaranta, Ambra Pastori, Bianca Zecchin, Alice Fusaro, Isabella Monne, Calogero Terregino, Antonia Ricci
EPI_ISL_747478, EPI_ISL_747479, EPI_ISL_747480	ULSS 5 Polesana	Istituto Zooprofilattico Sperimentale delle Venezie	Adelaide Milani, Alessia Schivo, Annalisa Salviato, Erika Giorgia Quaranta, Ambra Pastori, Bianca Zecchin, Alice Fusaro, Isabella Monne, Calogero Terregino, Antonia Ricci
EPI_ISL_747482, EPI_ISL_747483	Ospedale San Bonifacio	Istituto Zooprofilattico Sperimentale delle Venezie	Adelaide Milani, Alessia Schivo, Annalisa Salviato, Erika Giorgia Quaranta, Ambra Pastori, Bianca Zecchin, Alice Fusaro, Isabella Monne, Calogero Terregino, Antonia Ricci
EPI_ISL_747486, EPI_ISL_747487, EPI_ISL_747488, EPI_ISL_747489	ULSS 5 Polesana	Istituto Zooprofilattico Sperimentale delle Venezie	Adelaide Milani, Alessia Schivo, Annalisa Salviato, Erika Giorgia Quaranta, Ambra Pastori, Bianca Zecchin, Alice Fusaro, Isabella Monne, Calogero Terregino, Antonia Ricci
EPI_ISL_747490, EPI_ISL_747493	Respiratory Virus Unit, National Infection Service, Public Health England	COVID-19 Genomics UK (COG-UK) Consortium	PHE Covid Sequencing Team
EPI_ISL_747624, EPI_ISL_747625, EPI_ISL_747626, EPI_ISL_747627, EPI_ISL_747628, EPI_ISL_747629, EPI_ISL_747631, EPI_ISL_747633, EPI_ISL_747634, EPI_ISL_747635, EPI_ISL_747675, EPI_ISL_747676, EPI_ISL_747677, EPI_ISL_747678, EPI_ISL_747679, EPI_ISL_747680, EPI_ISL_747709, EPI_ISL_747710,			

EPI_ISL_747711, EPI_ISL_747712, EPI_ISL_747713, EPI_ISL_747714, EPI_ISL_747716, EPI_ISL_747717, EPI_ISL_747718, EPI_ISL_747719, EPI_ISL_747720, EPI_ISL_748021, EPI_ISL_748022, EPI_ISL_748023, EPI_ISL_748025, EPI_ISL_748026, EPI_ISL_748027, EPI_ISL_748028, EPI_ISL_748030, EPI_ISL_748031, EPI_ISL_748032, EPI_ISL_748034, EPI_ISL_748035, EPI_ISL_748036, EPI_ISL_748037, EPI_ISL_748038, EPI_ISL_748039, EPI_ISL_748040, EPI_ISL_748041, EPI_ISL_748042, EPI_ISL_748043, EPI_ISL_748044, EPI_ISL_748045, EPI_ISL_748046, EPI_ISL_748047, EPI_ISL_748048, EPI_ISL_748049, EPI_ISL_748050, EPI_ISL_748051, EPI_ISL_748052, EPI_ISL_748054, EPI_ISL_748055, EPI_ISL_748056, EPI_ISL_748057, EPI_ISL_748058, EPI_ISL_748059, EPI_ISL_748060, EPI_ISL_748061, EPI_ISL_748062, EPI_ISL_748063, EPI_ISL_748064, EPI_ISL_748065, EPI_ISL_748066, EPI_ISL_748068, EPI_ISL_748070, EPI_ISL_748071, EPI_ISL_748072, EPI_ISL_748073, EPI_ISL_748074, EPI_ISL_748075, EPI_ISL_748076, EPI_ISL_748077, EPI_ISL_748078, EPI_ISL_748079, EPI_ISL_748080, EPI_ISL_748081, EPI_ISL_748082, EPI_ISL_748083, EPI_ISL_748084, EPI_ISL_748085, EPI_ISL_748086, EPI_ISL_748087, EPI_ISL_748088, EPI_ISL_748089, EPI_ISL_748090, EPI_ISL_748092, EPI_ISL_748094, EPI_ISL_748095, EPI_ISL_748096, EPI_ISL_748097, EPI_ISL_748099, EPI_ISL_748100, EPI_ISL_748101, EPI_ISL_748102, EPI_ISL_748103, EPI_ISL_748104, EPI_ISL_748105, EPI_ISL_748107, EPI_ISL_748108, EPI_ISL_748109, EPI_ISL_748110, EPI_ISL_748112, EPI_ISL_748113, EPI_ISL_748114, EPI_ISL_748115, EPI_ISL_748116, EPI_ISL_748117			
see above	Department of Virus and Microbiological Special Diagnostics, Statens Serum Institut, Copenhagen, Denmark	Albertsen Lab, Department of Chemistry and Bioscience, Aalborg University, Denmark	Danish Covid-19 Genome Consortium
EPI_ISL_751454, EPI_ISL_751455, EPI_ISL_751456, EPI_ISL_751457, EPI_ISL_751458, EPI_ISL_751460, EPI_ISL_751463, EPI_ISL_751464, EPI_ISL_751465, EPI_ISL_751472, EPI_ISL_751473, EPI_ISL_751474, EPI_ISL_751475, EPI_ISL_751476			
see above	CHU Purpan - Laboratoire de Virologie - Institut Fédératif de Biologie	CHU Purpan - Laboratoire de Virologie - Institut Fédératif de Biologie	Latour J., Ranger N., Dubois M., Carcenac R., Harter A., Boyer P., Tremeaux P., Izopet J.
EPI_ISL_751558	NH Dept. of Health and Human Services Public Health Labs	Genomics and Discovery, Respiratory Viruses Branch, Division of Viral Diseases, Centers for Disease Control and Prevention	Krista Queen, Yan Li, Ying Tao, Jing Zhang, Anna Uehara, Anna Montmayeur, Clinton R. Paden, Peter W. Cook,Rachel Marine, Mili Sheth, Haibin Wang, Justin Lee, Suxiang Tong
EPI_ISL_751565	IA State Hygienic Laboratory	Genomics and Discovery, Respiratory Viruses Branch, Division of Viral Diseases, Centers for Disease Control and Prevention	Krista Queen, Yan Li, Ying Tao, Jing Zhang, Anna Uehara, Anna Montmayeur, Clinton R. Paden, Peter W. Cook,Rachel Marine, Mili Sheth, Haibin Wang, Justin Lee, Suxiang Tong
EPI_ISL_751576	MN PHL Division, Minnesota Department of Health	Genomics and Discovery, Respiratory Viruses Branch, Division of Viral Diseases, Centers for Disease Control and Prevention	Krista Queen, Yan Li, Ying Tao, Jing Zhang, Anna Uehara, Anna Montmayeur, Clinton R. Paden, Peter W. Cook,Rachel Marine, Mili Sheth, Haibin Wang, Justin Lee, Suxiang Tong
EPI_ISL_751577	MD DOH Laboratories Administration	Genomics and Discovery, Respiratory Viruses Branch, Division of Viral Diseases, Centers for Disease Control and Prevention	Krista Queen, Yan Li, Ying Tao, Jing Zhang, Anna Uehara, Anna Montmayeur, Clinton R. Paden, Peter W. Cook,Rachel Marine, Mili Sheth, Haibin Wang, Justin Lee, Suxiang Tong
EPI_ISL_751578	HI Dept. of Health, State Laboratories Division	Genomics and Discovery, Respiratory Viruses Branch, Division of Viral Diseases, Centers for Disease Control and Prevention	Krista Queen, Yan Li, Ying Tao, Jing Zhang, Anna Uehara, Anna Montmayeur, Clinton R. Paden, Peter W. Cook,Rachel Marine, Mili Sheth, Haibin Wang, Justin Lee, Suxiang Tong
EPI_ISL_751581	NYSDOH Wadsworth Center, Virology Lab	Genomics and Discovery, Respiratory Viruses Branch, Division of Viral Diseases, Centers for Disease Control and Prevention	Krista Queen, Yan Li, Ying Tao, Jing Zhang, Anna Uehara, Anna Montmayeur, Clinton R. Paden, Peter W. Cook,Rachel Marine, Mili Sheth, Haibin Wang, Justin Lee, Suxiang Tong
EPI_ISL_751585	NE Public Health Laboratory	Genomics and Discovery, Respiratory Viruses Branch, Division of Viral Diseases, Centers for Disease Control and Prevention	Krista Queen, Yan Li, Ying Tao, Jing Zhang, Anna Uehara, Anna Montmayeur, Clinton R. Paden, Peter W. Cook,Rachel Marine, Mili Sheth, Haibin Wang, Justin Lee, Suxiang Tong
EPI_ISL_751598	AK State Public Health Lab, State Health Department	Genomics and Discovery, Respiratory Viruses Branch, Division of Viral Diseases, Centers for Disease Control and Prevention	Krista Queen, Yan Li, Ying Tao, Jing Zhang, Anna Uehara, Anna Montmayeur, Clinton R. Paden, Peter W. Cook,Rachel Marine, Mili Sheth, Haibin Wang, Justin Lee, Suxiang Tong
EPI_ISL_751602	DE Public Health Laboratory	Genomics and Discovery, Respiratory Viruses Branch, Division of Viral Diseases, Centers for Disease Control and Prevention	Krista Queen, Yan Li, Ying Tao, Jing Zhang, Anna Uehara, Anna Montmayeur, Clinton R. Paden, Peter W. Cook,Rachel Marine, Mili Sheth, Haibin Wang, Justin Lee, Suxiang Tong
EPI_ISL_751606, EPI_ISL_751607	MD DOH Laboratories Administration	Genomics and Discovery, Respiratory Viruses Branch, Division of Viral Diseases, Centers for Disease Control and Prevention	Krista Queen, Yan Li, Ying Tao, Jing Zhang, Anna Uehara, Anna Montmayeur, Clinton R. Paden, Peter W. Cook,Rachel Marine, Mili Sheth, Haibin Wang, Justin Lee, Suxiang Tong
EPI_ISL_751613	OH Department of Health Laboratory	Genomics and Discovery, Respiratory Viruses Branch, Division of Viral Diseases, Centers for Disease Control and Prevention	Krista Queen, Yan Li, Ying Tao, Jing Zhang, Anna Uehara, Anna Montmayeur, Clinton R. Paden, Peter W. Cook,Rachel Marine, Mili Sheth, Haibin Wang, Justin Lee, Suxiang Tong
EPI_ISL_751614, EPI_ISL_751618	ID Bureau of Laboratories	Genomics and Discovery, Respiratory Viruses Branch, Division of Viral Diseases, Centers for Disease Control and Prevention	Krista Queen, Yan Li, Ying Tao, Jing Zhang, Anna Uehara, Anna Montmayeur, Clinton R. Paden, Peter W. Cook,Rachel Marine, Mili Sheth, Haibin Wang, Justin Lee, Suxiang Tong
EPI_ISL_751638	NE Public Health Laboratory	Genomics and Discovery, Respiratory Viruses Branch, Division of Viral Diseases, Centers for Disease Control and Prevention	Krista Queen, Yan Li, Ying Tao, Jing Zhang, Anna Uehara, Anna Montmayeur, Clinton R. Paden, Peter W. Cook,Rachel Marine, Mili Sheth, Haibin Wang, Justin Lee, Suxiang Tong
EPI_ISL_751641	MN PHL Division, Minnesota Department of Health	Genomics and Discovery, Respiratory Viruses Branch, Division of Viral Diseases, Centers for Disease Control and Prevention	Krista Queen, Yan Li, Ying Tao, Jing Zhang, Anna Uehara, Anna Montmayeur, Clinton R. Paden, Peter W. Cook,Rachel Marine, Mili Sheth, Haibin Wang, Justin Lee, Suxiang Tong
EPI_ISL_751649, EPI_ISL_751650	CT-Dr. Katherine A. Kelley State Public Health Lab	Genomics and Discovery, Respiratory Viruses Branch, Division of Viral Diseases, Centers for Disease Control and Prevention	Krista Queen, Yan Li, Ying Tao, Jing Zhang, Anna Uehara, Anna Montmayeur, Clinton R. Paden, Peter W. Cook,Rachel Marine, Mili Sheth, Haibin Wang, Justin Lee, Suxiang Tong
EPI_ISL_751656	MO State Public Health Laboratory	Genomics and Discovery, Respiratory Viruses Branch, Division of Viral Diseases, Centers for Disease Control and Prevention	Krista Queen, Yan Li, Ying Tao, Jing Zhang, Anna Uehara, Anna Montmayeur, Clinton R. Paden, Peter W. Cook,Rachel Marine, Mili Sheth, Haibin Wang, Justin Lee, Suxiang Tong
EPI_ISL_751659	NJ Public Health and Environmental Laboratories	Genomics and Discovery, Respiratory Viruses Branch, Division of Viral Diseases, Centers for Disease Control and Prevention	Krista Queen, Yan Li, Ying Tao, Jing Zhang, Anna Uehara, Anna Montmayeur, Clinton R. Paden, Peter W. Cook,Rachel Marine, Mili Sheth, Haibin Wang, Justin Lee, Suxiang Tong
EPI_ISL_751661	MS Public Health Laboratory	Genomics and Discovery, Respiratory Viruses Branch, Division of Viral Diseases, Centers for Disease Control and Prevention	Krista Queen, Yan Li, Ying Tao, Jing Zhang, Anna Uehara, Anna Montmayeur, Clinton R. Paden, Peter W. Cook,Rachel Marine, Mili Sheth, Haibin Wang, Justin Lee, Suxiang Tong
EPI_ISL_751666	IA State Hygienic Laboratory	Genomics and Discovery, Respiratory Viruses Branch, Division of Viral Diseases, Centers for Disease Control and Prevention	Krista Queen, Yan Li, Ying Tao, Jing Zhang, Anna Uehara, Anna Montmayeur, Clinton R. Paden, Peter W. Cook,Rachel Marine, Mili Sheth, Haibin Wang, Justin Lee, Suxiang Tong
EPI_ISL_751683	MS Public Health Laboratory	Genomics and Discovery, Respiratory Viruses Branch, Division of Viral Diseases, Centers for Disease Control and Prevention	Krista Queen, Yan Li, Ying Tao, Jing Zhang, Anna Uehara, Anna Montmayeur, Clinton R. Paden, Peter W. Cook,Rachel Marine, Mili Sheth, Haibin Wang, Justin Lee, Suxiang Tong
EPI_ISL_751689	OR State PHL-Virology/Immunology Section	Genomics and Discovery, Respiratory Viruses Branch, Division of Viral Diseases, Centers for Disease Control and Prevention	Krista Queen, Yan Li, Ying Tao, Jing Zhang, Anna Uehara, Anna Montmayeur, Clinton R. Paden, Peter W. Cook,Rachel Marine, Mili Sheth, Haibin Wang, Justin Lee, Suxiang Tong
EPI_ISL_751694	AK State Public Health Lab, State Health Department	Genomics and Discovery, Respiratory Viruses Branch,	Krista Queen, Yan Li, Ying Tao, Jing Zhang, Anna Uehara, Anna Montmayeur, Clinton R. Paden, Peter W. Cook,Rachel Marine, Mili Sheth, Haibin Wang,

		Division of Viral Diseases, Centers for Disease Control and Prevention	Justin Lee, Suxiang Tong
EPI_ISL_751715	MO State Public Health Laboratory	Genomics and Discovery, Respiratory Viruses Branch, Division of Viral Diseases, Centers for Disease Control and Prevention	Krista Queen, Yan Li, Ying Tao, Jing Zhang, Anna Uehara, Anna Montmayeur, Clinton R. Paden, Peter W. Cook,Rachel Marine, Mili Sheth, Haibin Wang, Justin Lee, Suxiang Tong
EPI_ISL_751716	MN PHL Division, Minnesota Department of Health	Genomics and Discovery, Respiratory Viruses Branch, Division of Viral Diseases, Centers for Disease Control and Prevention	Krista Queen, Yan Li, Ying Tao, Jing Zhang, Anna Uehara, Anna Montmayeur, Clinton R. Paden, Peter W. Cook,Rachel Marine, Mili Sheth, Haibin Wang, Justin Lee, Suxiang Tong
EPI_ISL_751729	CT-Dr. Katherine A. Kelley State Public Health Lab	Genomics and Discovery, Respiratory Viruses Branch, Division of Viral Diseases, Centers for Disease Control and Prevention	Krista Queen, Yan Li, Ying Tao, Jing Zhang, Anna Uehara, Anna Montmayeur, Clinton R. Paden, Peter W. Cook,Rachel Marine, Mili Sheth, Haibin Wang, Justin Lee, Suxiang Tong
EPI_ISL_751733	MN PHL Division, Minnesota Department of Health	Genomics and Discovery, Respiratory Viruses Branch, Division of Viral Diseases, Centers for Disease Control and Prevention	Krista Queen, Yan Li, Ying Tao, Jing Zhang, Anna Uehara, Anna Montmayeur, Clinton R. Paden, Peter W. Cook,Rachel Marine, Mili Sheth, Haibin Wang, Justin Lee, Suxiang Tong
EPI_ISL_751734	NE Public Health Laboratory	Genomics and Discovery, Respiratory Viruses Branch, Division of Viral Diseases, Centers for Disease Control and Prevention	Krista Queen, Yan Li, Ying Tao, Jing Zhang, Anna Uehara, Anna Montmayeur, Clinton R. Paden, Peter W. Cook,Rachel Marine, Mili Sheth, Haibin Wang, Justin Lee, Suxiang Tong
EPI_ISL_751739	MN PHL Division, Minnesota Department of Health	Genomics and Discovery, Respiratory Viruses Branch, Division of Viral Diseases, Centers for Disease Control and Prevention	Krista Queen, Yan Li, Ying Tao, Jing Zhang, Anna Uehara, Anna Montmayeur, Clinton R. Paden, Peter W. Cook,Rachel Marine, Mili Sheth, Haibin Wang, Justin Lee, Suxiang Tong
EPI_ISL_751741	OH Department of Health Laboratory	Genomics and Discovery, Respiratory Viruses Branch, Division of Viral Diseases, Centers for Disease Control and Prevention	Krista Queen, Yan Li, Ying Tao, Jing Zhang, Anna Uehara, Anna Montmayeur, Clinton R. Paden, Peter W. Cook,Rachel Marine, Mili Sheth, Haibin Wang, Justin Lee, Suxiang Tong
EPI_ISL_751742	IA State Hygienic Laboratory	Genomics and Discovery, Respiratory Viruses Branch, Division of Viral Diseases, Centers for Disease Control and Prevention	Krista Queen, Yan Li, Ying Tao, Jing Zhang, Anna Uehara, Anna Montmayeur, Clinton R. Paden, Peter W. Cook,Rachel Marine, Mili Sheth, Haibin Wang, Justin Lee, Suxiang Tong
EPI_ISL_751748	ID Bureau of Laboratories	Genomics and Discovery, Respiratory Viruses Branch, Division of Viral Diseases, Centers for Disease Control and Prevention	Krista Queen, Yan Li, Ying Tao, Jing Zhang, Anna Uehara, Anna Montmayeur, Clinton R. Paden, Peter W. Cook,Rachel Marine, Mili Sheth, Haibin Wang, Justin Lee, Suxiang Tong
EPI_ISL_751752	LA Office of Public Health Laboratories	Genomics and Discovery, Respiratory Viruses Branch, Division of Viral Diseases, Centers for Disease Control and Prevention	Krista Queen, Yan Li, Ying Tao, Jing Zhang, Anna Uehara, Anna Montmayeur, Clinton R. Paden, Peter W. Cook,Rachel Marine, Mili Sheth, Haibin Wang, Justin Lee, Suxiang Tong
EPI_ISL_751758, EPI_ISL_751767	HI Dept. of Health, State Laboratories Division	Genomics and Discovery, Respiratory Viruses Branch, Division of Viral Diseases, Centers for Disease Control and Prevention	Krista Queen, Yan Li, Ying Tao, Jing Zhang, Anna Uehara, Anna Montmayeur, Clinton R. Paden, Peter W. Cook,Rachel Marine, Mili Sheth, Haibin Wang, Justin Lee, Suxiang Tong
EPI_ISL_751779	IA State Hygienic Laboratory	Genomics and Discovery, Respiratory Viruses Branch, Division of Viral Diseases, Centers for Disease Control and Prevention	Krista Queen, Yan Li, Ying Tao, Jing Zhang, Anna Uehara, Anna Montmayeur, Clinton R. Paden, Peter W. Cook,Rachel Marine, Mili Sheth, Haibin Wang, Justin Lee, Suxiang Tong
EPI_ISL_751784	VA-Division of Consolidated Laboratory Services	Genomics and Discovery, Respiratory Viruses Branch, Division of Viral Diseases, Centers for Disease Control and Prevention	Krista Queen, Yan Li, Ying Tao, Jing Zhang, Anna Uehara, Anna Montmayeur, Clinton R. Paden, Peter W. Cook,Rachel Marine, Mili Sheth, Haibin Wang, Justin Lee, Suxiang Tong
EPI_ISL_751787	TX DSHS, Lab Services Section MC 1947	Genomics and Discovery, Respiratory Viruses Branch, Division of Viral Diseases, Centers for Disease Control and Prevention	Krista Queen, Yan Li, Ying Tao, Jing Zhang, Anna Uehara, Anna Montmayeur, Clinton R. Paden, Peter W. Cook,Rachel Marine, Mili Sheth, Haibin Wang, Justin Lee, Suxiang Tong
EPI_ISL_751793	AK State Public Health Lab, State Health Department	Genomics and Discovery, Respiratory Viruses Branch, Division of Viral Diseases, Centers for Disease Control and Prevention	Krista Queen, Yan Li, Ying Tao, Jing Zhang, Anna Uehara, Anna Montmayeur, Clinton R. Paden, Peter W. Cook,Rachel Marine, Mili Sheth, Haibin Wang, Justin Lee, Suxiang Tong
EPI_ISL_752531, EPI_ISL_752532, EPI_ISL_752533, EPI_ISL_752535, EPI_ISL_752538, EPI_ISL_752539, EPI_ISL_752540, EPI_ISL_752541, EPI_ISL_752542, EPI_ISL_752546, EPI_ISL_752547, EPI_ISL_752548, EPI_ISL_752549, EPI_ISL_752550, EPI_ISL_752551, EPI_ISL_752553, EPI_ISL_752554, EPI_ISL_752555			
see above	National Virus Reference Laboratory	National Virus Reference Laboratory	Michael Carr, Gabriel Gonzalez, Jonathan Dean, Daniel Hare, Cillian F De Gascun
EPI_ISL_752605	Faculty of Medicine, Al-Quds University	Faculty of Medicine, Al-Quds University	Ereqat,S., Nasereddin,A. and Al-Jawabreh,A.
EPI_ISL_753114, EPI_ISL_753116, EPI_ISL_753117, EPI_ISL_753118, EPI_ISL_753119, EPI_ISL_753120, EPI_ISL_753129, EPI_ISL_753130, EPI_ISL_753131, EPI_ISL_753132, EPI_ISL_753133, EPI_ISL_753134, EPI_ISL_753135, EPI_ISL_753136, EPI_ISL_753137, EPI_ISL_753138, EPI_ISL_753139, EPI_ISL_753140, EPI_ISL_753141, EPI_ISL_753142, EPI_ISL_753143, EPI_ISL_753144, EPI_ISL_753145, EPI_ISL_753146, EPI_ISL_753154, EPI_ISL_753156, EPI_ISL_753157, EPI_ISL_753158, EPI_ISL_753160, EPI_ISL_753161, EPI_ISL_753163, EPI_ISL_753164, EPI_ISL_753165, EPI_ISL_753166, EPI_ISL_753167, EPI_ISL_753170, EPI_ISL_753171, EPI_ISL_753173, EPI_ISL_753174, EPI_ISL_753176, EPI_ISL_753177, EPI_ISL_753198, EPI_ISL_753200, EPI_ISL_753222			
see above	State Laboratories Division, Hawaii State Department of Health	State Laboratories Division, Hawaii State Department of Health	Pamela O'Brien, Sabrina Diemert, Drew Kuwazaki, Razvan Sultana, Edward Desmond
EPI_ISL_753494, EPI_ISL_753526, EPI_ISL_753541, EPI_ISL_753542, EPI_ISL_753543, EPI_ISL_753544, EPI_ISL_753545, EPI_ISL_753546, EPI_ISL_753547, EPI_ISL_753548, EPI_ISL_753549, EPI_ISL_753550, EPI_ISL_753551, EPI_ISL_753552, EPI_ISL_753553, EPI_ISL_753554, EPI_ISL_753555, EPI_ISL_753556, EPI_ISL_753557, EPI_ISL_753558, EPI_ISL_753564, EPI_ISL_753627, EPI_ISL_753634, EPI_ISL_753643, EPI_ISL_753644, EPI_ISL_753645, EPI_ISL_753646, EPI_ISL_753647, EPI_ISL_753648, EPI_ISL_753649, EPI_ISL_753650, EPI_ISL_753651, EPI_ISL_753652			
see above	Clinical virology Laboratory, Children's Hospital Los Angeles	Center for Personalized Medicine, Children's Hospital Los Angeles	Gai et al
EPI_ISL_753827, EPI_ISL_753830, EPI_ISL_753831, EPI_ISL_753833	Charité Universitätsmedizin Berlin, Institut für Virologie/Labor Berlin	Charité Universitätsmedizin Berlin, Institut für Virologie	Victor M Corman, Jörn Beheim-Schwarzbach, Barbara Mühlemann, Julia Schneider, Talitha Veith, Terry Jones, Christian Drosten
EPI_ISL_754141, EPI_ISL_754143, EPI_ISL_754144, EPI_ISL_754145, EPI_ISL_754152, EPI_ISL_754153, EPI_ISL_754154, EPI_ISL_754155, EPI_ISL_754156, EPI_ISL_754157, EPI_ISL_754161, EPI_ISL_754162			
see above	Laboratoire de Microbiologie	National Reference Center for Viruses of Respiratory Infections, Institut Pasteur, Paris	Marion Barbet, Sylvie Behillil, Méline Bizard, Angela Brisebarre, Camille Capel, Etienne Simon-Lorière, Vincent Enouf, Maud Vanpeene, Sylvie van der Werf, Marie-Sarah Fangous
EPI_ISL_754173	Specialized Lab for COVID-19 Detection, Department of Genetic Engineering and Biotechnology	Specialized Lab for COVID-19 Detection, Department of Genetic Engineering and Biotechnology	Md. Nazmul Hasan, Shamsul H. Prodhan, Hammadul Hoque , Nurnabi Azad Jewel, Mumtahin Ahammed, Mahedi Hasan Ridoy, Avishek Sarkar
EPI_ISL_754176	Specialized Lab for COVID-19 Detection, Department of Genetic Engineering and Biotechnology	Specialized Lab for COVID-19 Detection, Department of Genetic Engineering and Biotechnology	Shamsul H. Prodhan, Hammadul Hoque, Nurnabi Azad Jewel, Md. Nazmul Hasan, Md. Mainul Hossain Bakul, Md. Rabiul Awal
EPI_ISL_754177	Specialized Lab for COVID-19 Detection, Department of Genetic Engineering and Biotechnology	Specialized Lab for COVID-19 Detection, Department of Genetic Engineering and Biotechnology	Nurnabi Azad Jewel, Md. Nazmul Hasan, Shamsul H. Prodhan, Hammadul Hoque, Asim Debnath, Sudipto Sakib Duranto
EPI_ISL_754257, EPI_ISL_754287, EPI_ISL_754294, EPI_ISL_754296, EPI_ISL_754297, EPI_ISL_754333,	Respiratory Virus Unit, National Infection Service, Public Health England	COVID-19 Genomics UK (COG-UK) Consortium	PHE Covid Sequencing Team

EPI_ISL_754334, EPI_ISL_754336, EPI_ISL_754354			
EPI_ISL_754402, EPI_ISL_754417, EPI_ISL_754421, EPI_ISL_754477, EPI_ISL_754482, EPI_ISL_754483, EPI_ISL_754488, EPI_ISL_754490, EPI_ISL_754491, EPI_ISL_754493, EPI_ISL_754496, EPI_ISL_754497, EPI_ISL_754499, EPI_ISL_754501, EPI_ISL_754503, EPI_ISL_754506, EPI_ISL_754580, EPI_ISL_754582, EPI_ISL_754583, EPI_ISL_754585, EPI_ISL_754588, EPI_ISL_754589, EPI_ISL_754592, EPI_ISL_754594, EPI_ISL_754595, EPI_ISL_754596, EPI_ISL_754598, EPI_ISL_754605, EPI_ISL_754606, EPI_ISL_754608, EPI_ISL_754610, EPI_ISL_754611, EPI_ISL_754614, EPI_ISL_754618			
see above	Wadsworth Center, New York State Department.of Health	Wadsworth Center, New York State Department.of Health	Kirsten St. George, Daryl M. Lamson, Alexis Russel, Matthew Shudt, Melissa A Leisner, Jonathan Plitnick, Navjot Singh, John Kelly, Sara Griesemer, Erasmus Schneider, Erica Lasek-Nesselquist
EPI_ISL_755111	California Department of Public Health	California Department of Public Health	CDPH IDLB COVIDNet
EPI_ISL_755216, EPI_ISL_755217, EPI_ISL_755220, EPI_ISL_755224	UCSD EXCITE lab	Andersen lab at Scripps Research	SEARCH Alliance San Diego
EPI_ISL_755270, EPI_ISL_755271, EPI_ISL_755274, EPI_ISL_755275, EPI_ISL_755278, EPI_ISL_755290, EPI_ISL_755295	San Diego County Public Health Laboratory	Andersen lab at Scripps Research	SEARCH Alliance San Diego with Tracy Basler, Jovan Shephard, Brett Austin
EPI_ISL_755434, EPI_ISL_755436, EPI_ISL_755437, EPI_ISL_755439, EPI_ISL_755440, EPI_ISL_755444, EPI_ISL_755446, EPI_ISL_755448, EPI_ISL_755449, EPI_ISL_755450, EPI_ISL_755451, EPI_ISL_755452, EPI_ISL_755457, EPI_ISL_755458, EPI_ISL_755462, EPI_ISL_755463, EPI_ISL_755465, EPI_ISL_755467, EPI_ISL_755469, EPI_ISL_755470, EPI_ISL_755471, EPI_ISL_755473, EPI_ISL_755474, EPI_ISL_755476, EPI_ISL_755477, EPI_ISL_755478, EPI_ISL_755479, EPI_ISL_755480, EPI_ISL_755481, EPI_ISL_755483, EPI_ISL_755484, EPI_ISL_755485, EPI_ISL_755486, EPI_ISL_755488			
see above	Maine Health and Environmental Testing Laboratory	Tewhey Lab, The Jackson Laboratory	Matluk,N., Dewey,H., Iosue,F., Barter,M., Lynch,R., Munger,H. and Tewhey,R.
EPI_ISL_755656, EPI_ISL_755658, EPI_ISL_755661, EPI_ISL_755662, EPI_ISL_755663, EPI_ISL_755664, EPI_ISL_755665, EPI_ISL_755666, EPI_ISL_755667, EPI_ISL_755671, EPI_ISL_755675, EPI_ISL_755678, EPI_ISL_755679, EPI_ISL_755683, EPI_ISL_755684, EPI_ISL_755687, EPI_ISL_755688, EPI_ISL_755689, EPI_ISL_755690, EPI_ISL_755691, EPI_ISL_755693, EPI_ISL_755694, EPI_ISL_755695, EPI_ISL_755699, EPI_ISL_755703, EPI_ISL_755706, EPI_ISL_755707, EPI_ISL_755709, EPI_ISL_755711			
see above	Toronto Invasive Bacterial Diseases Network	McMaster University	Allison McGeer, Patryk Aftanas, Hooman Derakhshani, Angel Li, Kuganya Nirmalarajah, Emily Panousis, Ahmed Draia, Jalees Nasir, Michael Surette, Samira Mubareka, Andrew G. McArthur
EPI_ISL_755714, EPI_ISL_755716, EPI_ISL_755717, EPI_ISL_755718, EPI_ISL_755719, EPI_ISL_755720, EPI_ISL_755721, EPI_ISL_755723, EPI_ISL_755724, EPI_ISL_755726, EPI_ISL_755728, EPI_ISL_755733, EPI_ISL_755735			
see above	Toronto Invasive Bacterial Diseases Network	McMaster University	David Richardson, Allison McGeer, Patryk Aftanas, Hooman Derakhshani, Angel Li, Kuganya Nirmalarajah, Emily Panousis, Ahmed Draia, Jalees Nasir, Michael Surette, Samira Mubareka, Andrew G. McArthur
EPI_ISL_755802, EPI_ISL_755805, EPI_ISL_755806, EPI_ISL_755807	Toronto Invasive Bacterial Diseases Network	McMaster University	Allison McGeer, Patryk Aftanas, Hooman Derakhshani, Angel Li, Kuganya Nirmalarajah, Emily Panousis, Ahmed Draia, Jalees Nasir, Michael Surette, Samira Mubareka, Andrew G. McArthur
EPI_ISL_757187, EPI_ISL_757188, EPI_ISL_757190, EPI_ISL_757192, EPI_ISL_757193, EPI_ISL_757196, EPI_ISL_757200, EPI_ISL_757201, EPI_ISL_757202, EPI_ISL_757204, EPI_ISL_757207, EPI_ISL_757208, EPI_ISL_757211, EPI_ISL_757212, EPI_ISL_757213, EPI_ISL_757214, EPI_ISL_757217, EPI_ISL_757218, EPI_ISL_757219, EPI_ISL_757220, EPI_ISL_757223, EPI_ISL_757224, EPI_ISL_757273, EPI_ISL_757274, EPI_ISL_757275, EPI_ISL_757276, EPI_ISL_757278, EPI_ISL_757279, EPI_ISL_757280, EPI_ISL_757281, EPI_ISL_757283, EPI_ISL_757284			
see above	Lighthouse Lab in Glasgow	Wellcome Sanger Institute for the COVID-19 Genomics UK (COG-UK) Consortium	Harper VanSteenhouse, Yumi Kasai, David Gray, Carol Clugston, Anna Dominiczak and Alex Alderton, Roberto Amato, Sonia Goncalves, Ewan Harrison, David K. Jackson, Ian Johnston, Dominic Kwiatkowski, Cordelia Langford, John Sillitoe on behalf of the Wellcome Sanger Institute COVID-19 Surveillance Team
EPI_ISL_757296, EPI_ISL_757297, EPI_ISL_757298, EPI_ISL_757299, EPI_ISL_757300, EPI_ISL_757301, EPI_ISL_757302, EPI_ISL_757303, EPI_ISL_757305, EPI_ISL_757306, EPI_ISL_757307, EPI_ISL_757308, EPI_ISL_757309, EPI_ISL_757310, EPI_ISL_757311, EPI_ISL_757312, EPI_ISL_757313, EPI_ISL_757314, EPI_ISL_757315, EPI_ISL_757317, EPI_ISL_757318, EPI_ISL_757319, EPI_ISL_757320, EPI_ISL_757321, EPI_ISL_757322, EPI_ISL_757324, EPI_ISL_757325, EPI_ISL_757326, EPI_ISL_757327, EPI_ISL_757328, EPI_ISL_757329, EPI_ISL_757330, EPI_ISL_757331, EPI_ISL_757332, EPI_ISL_757333, EPI_ISL_757334, EPI_ISL_757335, EPI_ISL_757336, EPI_ISL_757337, EPI_ISL_757338, EPI_ISL_757339, EPI_ISL_757340, EPI_ISL_757341			
see above	Department of Virology and Immunology, University of Helsinki and Helsinki University Hospital, Huslab Finland	Department of Virology, Faculty of Medicine, University of Helsinki, Helsinki, Finland	Teemu Smura, Ravi Kant, Phuoc Truong, Hussein Alburkat, Hannimari Kallio-Kokko, Jenni Virtanen, Maija Suvanto, Sari Hannula, Harri Kangas, Pekka Ellonen, Olli Vapalahti
EPI_ISL_757699	Department of Virus and Microbiological Special Diagnostics, Statens Serum Institut, Copenhagen, Denmark	Albertsen Lab, Department of Chemistry and Bioscience, Aalborg University, Denmark	Danish Covid-19 Genome Consortium
EPI_ISL_760230, EPI_ISL_760232, EPI_ISL_760233, EPI_ISL_760234, EPI_ISL_760235, EPI_ISL_760236, EPI_ISL_760237, EPI_ISL_760238, EPI_ISL_760239, EPI_ISL_760240, EPI_ISL_760241, EPI_ISL_760242, EPI_ISL_760243, EPI_ISL_760244, EPI_ISL_760245			
see above	Division of Emerging Infectious Diseases, Bureau of Infectious Diseases Diagnosis Control, Korea Disease Control and Prevention Agency	Division of Emerging Infectious Diseases, Bureau of Infectious Diseases Diagnosis Control, Korea Disease Control and Prevention Agency	Ae Kyung Park, Il-Hwan Kim, Heui Man Kim, Jeong-Min Kim, Namjoo Lee, Chaeyoung Lee, Sang Hee Woo, Eun-Jin Kim
EPI_ISL_760490	Lighthouse Lab in Glasgow	Wellcome Sanger Institute for the COVID-19 Genomics UK (COG-UK) Consortium	Harper VanSteenhouse, Yumi Kasai, David Gray, Carol Clugston, Anna Dominiczak and Alex Alderton, Roberto Amato, Sonia Goncalves, Ewan Harrison, David K. Jackson, Ian Johnston, Dominic Kwiatkowski, Cordelia Langford, John Sillitoe on behalf of the Wellcome Sanger Institute COVID-19 Surveillance Team
EPI_ISL_763099, EPI_ISL_763100, EPI_ISL_763101, EPI_ISL_763102, EPI_ISL_763103, EPI_ISL_763111, EPI_ISL_763112, EPI_ISL_763113, EPI_ISL_763114, EPI_ISL_763115, EPI_ISL_763116, EPI_ISL_763118, EPI_ISL_763119, EPI_ISL_763120, EPI_ISL_763121, EPI_ISL_763124, EPI_ISL_763125, EPI_ISL_763163, EPI_ISL_763165, EPI_ISL_763166, EPI_ISL_763167, EPI_ISL_763168, EPI_ISL_763169, EPI_ISL_763170, EPI_ISL_763171, EPI_ISL_763172, EPI_ISL_763235, EPI_ISL_763236, EPI_ISL_763237, EPI_ISL_763275, EPI_ISL_763276, EPI_ISL_763277, EPI_ISL_763305, EPI_ISL_763313			
see above	Dutch COVID-19 response team	Erasmus Medical Center	Bas Oude Munnink, Reina Sikkema, David Nieuwenhuijse, Irina Chestakova, Anne van der Linden, Marjan Boter, Emmanuelle Munger, Corine GeurtsvanKessel, Annemiek van der Eijk, Richard Molenkamp, Marion Koopmans, on behalf of the Dutch national COVID-19 response team.
EPI_ISL_763342	Florida Bureau of Public Health Laboratories	Florida Bureau of Public Health Laboratories	Sarah Schmedes, Jason Blanton
EPI_ISL_763873, EPI_ISL_764307, EPI_ISL_764309, EPI_ISL_764313, EPI_ISL_764325, EPI_ISL_764326, EPI_ISL_764327	Department of Pathology, University of Cambridge	COVID-19 Genomics UK (COG-UK) Consortium	Aminu S. Jahun, Yasmin Chaudhry, Grant Hall, Iliana Georgana, Myra Hosmillo, Martin D. Curran, Malte Pinckert, Surendra Parmar, Ian Goodfellow
EPI_ISL_764360	Virology Department, Royal Infirmary of Edinburgh, NHS Lothian / School of Biological Sciences, University of Edinburgh / Institute of Genetics and Molecular Medicine, University of Edinburgh	COVID-19 Genomics UK (COG-UK) Consortium	McHugh M, Dewar R, Rooke S, Gallagher M, Balcaza C, O'Toole Á, Scher E, Hill V, McCrone JT, Colquhoun R, Yu X, Jackson B, Rambaut A, Williams TC, Templeton K
EPI_ISL_765217	Instituto Nacional de Saude (INSA) and Instituto Gulbenkian de Ciencia (IGC)	Instituto Nacional de Saude (INSA) and Instituto Gulbenkian de Ciencia (IGC)	Borges et al
EPI_ISL_765748, EPI_ISL_765749, EPI_ISL_765780, EPI_ISL_765844, EPI_ISL_765845, EPI_ISL_765846, EPI_ISL_765847, EPI_ISL_765848, EPI_ISL_765849, EPI_ISL_765852, EPI_ISL_765853, EPI_ISL_765854, EPI_ISL_765855, EPI_ISL_765856, EPI_ISL_765857, EPI_ISL_765860, EPI_ISL_765861, EPI_ISL_765864, EPI_ISL_765867, EPI_ISL_765868, EPI_ISL_765871, EPI_ISL_765872, EPI_ISL_765873, EPI_ISL_765874, EPI_ISL_765878, EPI_ISL_765879, EPI_ISL_765880, EPI_ISL_765881, EPI_ISL_765882, EPI_ISL_765886			
see above	Massachusetts General Hospital	Infectious Disease Program, Broad Institute of Harvard and MIT	Lemieux,J.E., Siddle,K.J., Shaw,B., Adams,G., Pierce,V., Turbett,S., Anahtar,M., Branda,J., Slater,D., Harris,J., Lin,A.E., Gladden-Young,A., Lagerborg,K., Rudy,M., DeRuff,K., Carter,A., Normandin,E., Bauer,M., Reilly,S., Tomkins-Tinch,C., Loreth,C., Chaluvadi,S., Neumann,A., Cusick,C., Chapman,S.B., Gnirke,A., Flowers,K., Cerrato,F., Birren,B.W., Gallagher,G., Smole,S., Park,D.J., MacInnis,B.L., Ryan,E., LaRocque,R., Rosenberg,E. and Sabeti,P.C.
EPI_ISL_766277	Respiratory Virus Unit, National Infection Service, Public Health England	COVID-19 Genomics UK (COG-UK) Consortium	PHE Covid Sequencing Team
EPI_ISL_766571, EPI_ISL_766572, EPI_ISL_766573	ULSS 7 Pedemontana - Distretto 1	Istituto Zooprofilattico Sperimentale delle Venezie	Adelaide Milani, Alessia Schivo, Annalisa Salviato, Erika Giorgia Quaranta, Ambra Pastori, Bianca Zecchin, Alice Fusaro, Isabella Monne, Calogero Terregino, Antonia Ricci
EPI_ISL_766614	unknown	The Public Health Agency of Sweden	Department of Microbiology, The Public Health Agency of Sweden
EPI_ISL_766617, EPI_ISL_766618, EPI_ISL_766620, EPI_ISL_766621	Klinisk mikrobiologi	The Public Health Agency of Sweden	Department of Microbiology, The Public Health Agency of Sweden

EPI_ISL_766642, EPI_ISL_766643	Vardcentralen Brinken	The Public Health Agency of Sweden	Department of Microbiology, The Public Health Agency of Sweden
EPI_ISL_766645	Barnakuten	The Public Health Agency of Sweden	Department of Microbiology, The Public Health Agency of Sweden
EPI_ISL_766879, EPI_ISL_766880, EPI_ISL_766881	Wyoming Public Health Laboratory	Wyoming Public Health Laboratory	Noah Hull, Taylor Fearing, Lynette Gumbleton, Channing Weber, Ashley Norberg, Bailey Bowcutt, and Wanda Manley
EPI_ISL_766999, EPI_ISL_767003, EPI_ISL_767006	Delaware Public Health Laboratory	Delaware Public Health Laboratory	Gregory Hovan
EPI_ISL_767239	Lighthouse Lab in Milton Keynes	Wellcome Sanger Institute for the COVID-19 Genomics UK (COG-UK) Consortium	The Lighthouse Lab in Milton Keynes and Alex Alderton, Roberto Amato, Sonia Goncalves, Ewan Harrison, David K. Jackson, Ian Johnston, Dominic Kwiatkowski, Cordelia Langford, John Sillitoe on behalf of the Wellcome Sanger Institute COVID-19 Surveillance Team
EPI_ISL_767319, EPI_ISL_767320, EPI_ISL_767322, EPI_ISL_767323, EPI_ISL_767324, EPI_ISL_767327, EPI_ISL_767328, EPI_ISL_767329, EPI_ISL_767330, EPI_ISL_767333	Lighthouse Lab in Alderley Park	Wellcome Sanger Institute for the COVID-19 Genomics UK (COG-UK) Consortium	Jacquelyn Wynn, Mairead Hyland, The Lighthouse Lab in Alderley Park and Alex Alderton, Roberto Amato, Sonia Goncalves, Ewan Harrison, David K. Jackson, Ian Johnston, Dominic Kwiatkowski, Cordelia Langford, John Sillitoe on behalf of the Wellcome Sanger Institute COVID-19 Surveillance Team
EPI_ISL_768325, EPI_ISL_768326, EPI_ISL_768327, EPI_ISL_768328, EPI_ISL_768329, EPI_ISL_768330, EPI_ISL_768331, EPI_ISL_768332, EPI_ISL_768333, EPI_ISL_768334, EPI_ISL_768335, EPI_ISL_768336, EPI_ISL_768337, EPI_ISL_768338, EPI_ISL_768339, EPI_ISL_768340, EPI_ISL_768341, EPI_ISL_768342, EPI_ISL_768343, EPI_ISL_768344, EPI_ISL_768345, EPI_ISL_768346, EPI_ISL_768347, EPI_ISL_768348, EPI_ISL_768349, EPI_ISL_768350, EPI_ISL_768351, EPI_ISL_768352, EPI_ISL_768353, EPI_ISL_768354, EPI_ISL_768355, EPI_ISL_768356, EPI_ISL_768357, EPI_ISL_768358, EPI_ISL_768359, EPI_ISL_768360			
see above	LSUHS Emerging Viral Threat Laboratory	Microbial Genome Sequencing Center	Jeremy P. Kamil, Jennifer L. Carroll, Camille F. Abshire, Maarten Van Diest, Andrew D. Yurochko, Martin J. Sapp, Rona S. Scott, Christopher G. Kevil, Daniel J. Snyder, Vaughn S. Cooper, John A. Vanchiere
EPI_ISL_768522	Synphaet Hospital	National Institute of Health, Department of Medical Sciences, Ministry of Public Health, Thailand	Pilailuk Okada; Siripaporn Phuygun; Sittiporn Parnmen; Ratana Tacharoenmuang; Pakorn Piromtong; Natchaya Khiadsang; Thanutsapa Thanadachakul; Warawan Wongboot; sirikanda wimol; Sunthareeya Waicharoen;
EPI_ISL_768611	Regional medical sciences center 6 Chonburi	National Institute of Health, Department of Medical Sciences, Ministry of Public Health, Thailand	Pilailuk Okada; Siripaporn Phuygun; Sittiporn Parnmen; Ratana Tacharoenmuang; Pakorn Piromtong; Natchaya Khiadsang; Thanutsapa Thanadachakul; Warawan Wongboot; sirikanda wimol; Sunthareeya Waicharoen;
EPI_ISL_769988	Area De Salud San Francisco-San Antonio (Coopesana)	Inciensa, Instituto Costarricense de Investigación y Enseñanza en Nutrición y Salud	Francisco Duarte, Hebleen Porras, Claudio Soto-Garita, Estela Cordero, Adriana Godínez, Melany Calderón & Mariel López
EPI_ISL_769999	Area De Salud Paraiso-Cervantes	Inciensa, Instituto Costarricense de Investigación y Enseñanza en Nutrición y Salud	Francisco Duarte, Hebleen Porras, Claudio Soto-Garita, Estela Cordero, Adriana Godínez, Melany Calderón & Mariel López
EPI_ISL_770005	Area De Salud Moravia	Inciensa, Instituto Costarricense de Investigación y Enseñanza en Nutrición y Salud	Francisco Duarte, Hebleen Porras, Claudio Soto-Garita, Estela Cordero, Adriana Godínez, Melany Calderón & Mariel López
EPI_ISL_770023	Area De Salud San Francisco-San Antonio (Coopesana)	Inciensa, Instituto Costarricense de Investigación y Enseñanza en Nutrición y Salud	Francisco Duarte, Hebleen Porras, Claudio Soto-Garita, Estela Cordero, Adriana Godínez, Melany Calderón & Mariel López
EPI_ISL_770069, EPI_ISL_770086, EPI_ISL_770087, EPI_ISL_770092, EPI_ISL_770093, EPI_ISL_770095, EPI_ISL_770096, EPI_ISL_770097, EPI_ISL_770102, EPI_ISL_770103, EPI_ISL_770105, EPI_ISL_770106, EPI_ISL_770107, EPI_ISL_770108, EPI_ISL_770109, EPI_ISL_770115, EPI_ISL_770116, EPI_ISL_770117, EPI_ISL_770118, EPI_ISL_770119, EPI_ISL_770120			
see above	Wyoming Public Health Laboratory	Wyoming Public Health Laboratory	Noah Hull, Taylor Fearing, Lynette Gumbleton, Channing Weber, Ashley Norberg, Bailey Bowcutt, and Wanda Manley
EPI_ISL_770551, EPI_ISL_770553, EPI_ISL_770555, EPI_ISL_770557, EPI_ISL_770558, EPI_ISL_770559, EPI_ISL_770560, EPI_ISL_770561, EPI_ISL_770562, EPI_ISL_770563, EPI_ISL_770565, EPI_ISL_770567, EPI_ISL_770568, EPI_ISL_770569, EPI_ISL_770570, EPI_ISL_770575, EPI_ISL_770576, EPI_ISL_770578, EPI_ISL_770579, EPI_ISL_770580, EPI_ISL_770581, EPI_ISL_770585, EPI_ISL_770586, EPI_ISL_770587, EPI_ISL_770591, EPI_ISL_770592, EPI_ISL_770595, EPI_ISL_770596, EPI_ISL_770597, EPI_ISL_770598, EPI_ISL_770599, EPI_ISL_770601, EPI_ISL_770602, EPI_ISL_770603, EPI_ISL_770604, EPI_ISL_770605, EPI_ISL_770606, EPI_ISL_770607, EPI_ISL_770608, EPI_ISL_770609, EPI_ISL_770610, EPI_ISL_770611, EPI_ISL_770612, EPI_ISL_770614, EPI_ISL_770616, EPI_ISL_770618, EPI_ISL_770621, EPI_ISL_770622, EPI_ISL_770623, EPI_ISL_770624, EPI_ISL_770625, EPI_ISL_770627, EPI_ISL_770630			
see above	Laboratório de Microbiologia Molecular - Universidade FEEVALE	Bioinformatics Laboratory / LNCC	Felipe Benites, Fernando Rosado Spilki, Alana Witt Hansen, Juliane Deise Fleck, Juliana Schons, Meriane Demoliner, Ana Karolina Eisen Antunes, Fagner Henrique Heldt, Larissa Mallmann, Bruna Hermann, Ana Luiza Ziulkoski, Vyctoria Goes, Karoline Schallenberger, Matheus Nunes Weber, Paula Rodrigues de Almeida, Alessandra Pavan Lamarca da Silva, Ronaldo da Silva F Jr , Luiz G P de Almeida, Alexandra L Gerber , Ana Paula de C Guimarães,Ana Tereza R de Vasconcelos
EPI_ISL_770674	WHO National Influenza Centre Russian Federation	WHO National Influenza Centre Russian Federation	Andrey Komissarov, Artem Fadeev, Anna Ivanova, Kseniya Komissarova, Dmitry Bazhenov, Daria Danilenko, Ksenia Safina, Elena Nabieva, Georgii Bazykin, Dmitry Lioznov
EPI_ISL_770717, EPI_ISL_770718	ZOTZ KLIMAS MVZ Düsseldorf-Centrum GbR ÜBAG für Labormedizin, Genetik, Zytologie, Pathologie	Center of Medical Microbiology, Virology, and Hospital Hygiene, University of Duesseldorf	Maximilian Damagnez, Alexander Dilthey, Ashley-Jane Duplessis, Patrick Finzer, Katrin Hoffmann, Torsten Houwaart, Lisanna Hülse, Malte Kohns Vasconcelos, Marek Korencak, Nadine Lübke, Jessica Nicolai, Klaus Pfeffer, Daniel Strelow, Jörg Timm, Andreas Walker, Tobias Wienemann, Rainer Zotz
EPI_ISL_770780, EPI_ISL_770781, EPI_ISL_770782	Minnesota Department of Health, Public Health Laboratory	Minnesota Department of Health, Public Health Laboratory	Alexandra Lorentz, Jacob Garfin, Matt Plumb, and Xiong Wang
EPI_ISL_770809, EPI_ISL_770812	Essentia Health-St. Mary's Medical Center	Minnesota Department of Health, Public Health Laboratory	Alexandra Lorentz, Jacob Garfin, Matt Plumb, and Xiong Wang
EPI_ISL_770814	Vault Health	Minnesota Department of Health, Public Health Laboratory	Alexandra Lorentz, Jacob Garfin, Matt Plumb, and Xiong Wang
EPI_ISL_770890, EPI_ISL_770891, EPI_ISL_770893, EPI_ISL_770896, EPI_ISL_770908, EPI_ISL_770910, EPI_ISL_770911, EPI_ISL_770912, EPI_ISL_770913, EPI_ISL_770914, EPI_ISL_770915, EPI_ISL_770916, EPI_ISL_770918, EPI_ISL_770919, EPI_ISL_770920, EPI_ISL_770921, EPI_ISL_770922, EPI_ISL_770923, EPI_ISL_770924, EPI_ISL_770925, EPI_ISL_770926, EPI_ISL_770927, EPI_ISL_770928, EPI_ISL_770929, EPI_ISL_770930, EPI_ISL_770931, EPI_ISL_770938, EPI_ISL_770939, EPI_ISL_770940, EPI_ISL_770942, EPI_ISL_770950, EPI_ISL_770956, EPI_ISL_770957, EPI_ISL_770958, EPI_ISL_770959, EPI_ISL_770960, EPI_ISL_770961, EPI_ISL_770964, EPI_ISL_770965, EPI_ISL_770966, EPI_ISL_770967, EPI_ISL_770968, EPI_ISL_770969, EPI_ISL_770970, EPI_ISL_770973			
see above	Laboratoire national de santé, Microbiology, Virology	Laboratoire national de santé, Microbiology, Microbial Genomics Platform	Anke Wienecke-Baldacchino, Catherine Ragimbeau,Jessica Tapp, Fatu Djabi, Lise Pignon, Raoul Salmon, Tamir Abdelrahman
EPI_ISL_771189, EPI_ISL_771191, EPI_ISL_771222, EPI_ISL_771223, EPI_ISL_771247, EPI_ISL_771248, EPI_ISL_771249, EPI_ISL_771250, EPI_ISL_771253, EPI_ISL_771254, EPI_ISL_771255, EPI_ISL_771256, EPI_ISL_771257, EPI_ISL_771258, EPI_ISL_771260, EPI_ISL_771261, EPI_ISL_771262, EPI_ISL_771263, EPI_ISL_771265, EPI_ISL_771266, EPI_ISL_771267, EPI_ISL_771268, EPI_ISL_771269, EPI_ISL_771271, EPI_ISL_771272, EPI_ISL_771273, EPI_ISL_771274, EPI_ISL_771275, EPI_ISL_771276, EPI_ISL_771277, EPI_ISL_771278, EPI_ISL_771279, EPI_ISL_771280, EPI_ISL_771281, EPI_ISL_771282, EPI_ISL_771284, EPI_ISL_771285, EPI_ISL_771287, EPI_ISL_771289, EPI_ISL_771290, EPI_ISL_771291, EPI_ISL_771292, EPI_ISL_771294, EPI_ISL_771295, EPI_ISL_771296, EPI_ISL_771297, EPI_ISL_771298, EPI_ISL_771302, EPI_ISL_771303, EPI_ISL_771304, EPI_ISL_771305, EPI_ISL_771306, EPI_ISL_771308, EPI_ISL_771309, EPI_ISL_771310, EPI_ISL_771311, EPI_ISL_771312, EPI_ISL_771313, EPI_ISL_771315, EPI_ISL_771316, EPI_ISL_771318, EPI_ISL_771319, EPI_ISL_771320, EPI_ISL_771321, EPI_ISL_771322, EPI_ISL_771325, EPI_ISL_771328, EPI_ISL_771329, EPI_ISL_771334, EPI_ISL_771336, EPI_ISL_771339, EPI_ISL_771340, EPI_ISL_771341, EPI_ISL_771343, EPI_ISL_771344, EPI_ISL_771350, EPI_ISL_771351, EPI_ISL_771352, EPI_ISL_771354, EPI_ISL_771357, EPI_ISL_771360			
see above	Colorado Department of Public Health and Environment	Colorado Department of Puplic Health and Environment	Laura Bankers, Molly C. Hetherington-Rauth, Diana Ir, Shannon Ely, Shannon R. Matzinger, Sarah Elizabeth Totten, Emily A. Travanty
EPI_ISL_775276	Haukeland University Hospital, Dept. of Microbiology	Norwegian Institute of Public Health, Department of Virology	Kathrine Stene-Johansen, Kamilla Heddeland Instefjord, Hilde Elshaug, Atiya R Ali,Marie Paulsen Madsen, Rasmus Riis Kopperud, Hilde Vollan, Karoline Bragstad, Olav Hungnes
EPI_ISL_775284	Oslo University Hospital, Department of Medical Microbiology	Norwegian Institute of Public Health, Department of Virology	Kathrine Stene-Johansen, Kamilla Heddeland Instefjord, Hilde Elshaug, Atiya R Ali,Marie Paulsen Madsen, Rasmus Riis Kopperud, Hilde Vollan, Karoline Bragstad, Olav Hungnes
EPI_ISL_775285	Hospital of Southern Norway - Kristiansand, Department of Medical Microbiology	Norwegian Institute of Public Health, Department of Virology	Kathrine Stene-Johansen, Kamilla Heddeland Instefjord, Hilde Elshaug, Atiya R Ali,Marie Paulsen Madsen, Rasmus Riis Kopperud, Hilde Vollan, Karoline Bragstad, Olav Hungnes
EPI_ISL_775286, EPI_ISL_775287, EPI_ISL_775288, EPI_ISL_775289, EPI_ISL_775290	Nordland Hospital - Bodo, Laboratory Department, Molecular Biology Unit	Norwegian Institute of Public Health, Department of Virology	Kathrine Stene-Johansen, Kamilla Heddeland Instefjord, Hilde Elshaug, Atiya R Ali,Marie Paulsen Madsen, Rasmus Riis Kopperud, Hilde Vollan, Karoline Bragstad, Olav Hungnes
EPI_ISL_775294	Furst Medical Laboratory	Norwegian Institute of Public Health, Department of Virology	Kathrine Stene-Johansen, Kamilla Heddeland Instefjord, Hilde Elshaug, Atiya R Ali,Marie Paulsen Madsen, Rasmus Riis Kopperud, Hilde Vollan, Karoline Bragstad, Olav Hungnes
EPI_ISL_775297	Nordland Hospital - Bodo, Laboratory Department, Molecular Biology Unit	Norwegian Institute of Public Health, Department of Virology	Kathrine Stene-Johansen, Kamilla Heddeland Instefjord, Hilde Elshaug, Atiya R Ali,Marie Paulsen Madsen, Rasmus Riis Kopperud, Hilde Vollan, Karoline Bragstad, Olav Hungnes

EPI_ISL_775335, EPI_ISL_775336	Haukeland University Hospital, Dept. of Microbiology	Norwegian Institute of Public Health, Department of Virology	Kathrine Stene-Johansen, Kamilla Heddeland Instefjord, Hilde Elshaug, Atiya R Ali,Marie Paulsen Madsen, Rasmus Riis Kopperud, Hilde Vollan, Karoline Bragstad, Olav Hungnes
EPI_ISL_775348	Medical Microbiology Unit, Department for Laboratory Medicine, Drammen Hospital, Vestre Viken Health Trust,	Norwegian Institute of Public Health, Department of Virology	Kathrine Stene-Johansen, Kamilla Heddeland Instefjord, Hilde Elshaug, Atiya R Ali,Marie Paulsen Madsen, Rasmus Riis Kopperud, Hilde Vollan, Karoline Bragstad, Olav Hungnes
EPI_ISL_775349, EPI_ISL_775350, EPI_ISL_775351, EPI_ISL_775352	Unilabs Laboratory Medicine	Norwegian Institute of Public Health, Department of Virology	Kathrine Stene-Johansen, Kamilla Heddeland Instefjord, Hilde Elshaug, Atiya R Ali,Marie Paulsen Madsen, Rasmus Riis Kopperud, Hilde Vollan, Karoline Bragstad, Olav Hungnes
EPI_ISL_775354	Dept. of Medical Microbiology, Stavanger University Hospital, Helse Stavanger HF	Norwegian Institute of Public Health, Department of Virology	Kathrine Stene-Johansen, Kamilla Heddeland Instefjord, Hilde Elshaug, Atiya R Ali,Marie Paulsen Madsen, Rasmus Riis Kopperud, Hilde Vollan, Karoline Bragstad, Olav Hungnes
EPI_ISL_775356	Norwegian Institute of Public Health, Department of Virology	Norwegian Institute of Public Health, Department of Virology	Kathrine Stene-Johansen, Kamilla Heddeland Instefjord, Hilde Elshaug, Atiya R Ali,Marie Paulsen Madsen, Rasmus Riis Kopperud, Hilde Vollan, Karoline Bragstad, Olav Hungnes
EPI_ISL_775357	Haukeland University Hospital, Dept. of Microbiology	Norwegian Institute of Public Health, Department of Virology	Kathrine Stene-Johansen, Kamilla Heddeland Instefjord, Hilde Elshaug, Atiya R Ali,Marie Paulsen Madsen, Rasmus Riis Kopperud, Hilde Vollan, Karoline Bragstad, Olav Hungnes
EPI_ISL_775358, EPI_ISL_775359, EPI_ISL_775360, EPI_ISL_775361	Unilabs Laboratory Medicine	Norwegian Institute of Public Health, Department of Virology	Kathrine Stene-Johansen, Kamilla Heddeland Instefjord, Hilde Elshaug, Atiya R Ali,Marie Paulsen Madsen, Rasmus Riis Kopperud, Hilde Vollan, Karoline Bragstad, Olav Hungnes
EPI_ISL_775362, EPI_ISL_775363, EPI_ISL_775364, EPI_ISL_775365, EPI_ISL_775366, EPI_ISL_775367, EPI_ISL_775368, EPI_ISL_775369, EPI_ISL_775370	Foerde Hospital, Department of Microbiology	Norwegian Institute of Public Health, Department of Virology	Kathrine Stene-Johansen, Kamilla Heddeland Instefjord, Hilde Elshaug, Atiya R Ali,Marie Paulsen Madsen, Rasmus Riis Kopperud, Hilde Vollan, Karoline Bragstad, Olav Hungnes
EPI_ISL_775371	Hospital of Southern Norway - Kristiansand, Department of Medical Microbiology	Norwegian Institute of Public Health, Department of Virology	Kathrine Stene-Johansen, Kamilla Heddeland Instefjord, Hilde Elshaug, Atiya R Ali,Marie Paulsen Madsen, Rasmus Riis Kopperud, Hilde Vollan, Karoline Bragstad, Olav Hungnes
EPI_ISL_775373, EPI_ISL_775374, EPI_ISL_775375, EPI_ISL_775376, EPI_ISL_775377, EPI_ISL_775383	Nordland Hospital - Bodo, Laboratory Department, Molecular Biology Unit	Norwegian Institute of Public Health, Department of Virology	Kathrine Stene-Johansen, Kamilla Heddeland Instefjord, Hilde Elshaug, Atiya R Ali,Marie Paulsen Madsen, Rasmus Riis Kopperud, Hilde Vollan, Karoline Bragstad, Olav Hungnes
EPI_ISL_775384	Unilabs Laboratory Medicine	Norwegian Institute of Public Health, Department of Virology	Kathrine Stene-Johansen, Kamilla Heddeland Instefjord, Hilde Elshaug, Atiya R Ali,Marie Paulsen Madsen, Rasmus Riis Kopperud, Hilde Vollan, Karoline Bragstad, Olav Hungnes
EPI_ISL_775387, EPI_ISL_775388, EPI_ISL_775389, EPI_ISL_775390	Akershus University Hospital, Department for Microbiology and Infectious Disease Control	Norwegian Institute of Public Health, Department of Virology	Kathrine Stene-Johansen, Kamilla Heddeland Instefjord, Hilde Elshaug, Atiya R Ali,Marie Paulsen Madsen, Rasmus Riis Kopperud, Hilde Vollan, Karoline Bragstad, Olav Hungnes
EPI_ISL_775398	Nordland Hospital - Bodo, Laboratory Department, Molecular Biology Unit	Norwegian Institute of Public Health, Department of Virology	Kathrine Stene-Johansen, Kamilla Heddeland Instefjord, Hilde Elshaug, Atiya R Ali,Marie Paulsen Madsen, Rasmus Riis Kopperud, Hilde Vollan, Karoline Bragstad, Olav Hungnes
EPI_ISL_775415, EPI_ISL_775417, EPI_ISL_775418, EPI_ISL_775419, EPI_ISL_775420, EPI_ISL_775457	Oslo University Hospital, Department of Medical Microbiology	Norwegian Institute of Public Health, Department of Virology	Kathrine Stene-Johansen, Kamilla Heddeland Instefjord, Hilde Elshaug, Atiya R Ali,Marie Paulsen Madsen, Rasmus Riis Kopperud, Hilde Vollan, Karoline Bragstad, Olav Hungnes
EPI_ISL_775484, EPI_ISL_775488, EPI_ISL_775489, EPI_ISL_775490	Furst Medical Laboratory	Norwegian Institute of Public Health, Department of Virology	Kathrine Stene-Johansen, Kamilla Heddeland Instefjord, Hilde Elshaug, Atiya R Ali,Marie Paulsen Madsen, Rasmus Riis Kopperud, Hilde Vollan, Karoline Bragstad, Olav Hungnes
EPI_ISL_775491, EPI_ISL_775492, EPI_ISL_775493, EPI_ISL_775494	Department of Medical Microbiology, St. Olavs hospital	Norwegian Institute of Public Health, Department of Virology	Kathrine Stene-Johansen, Kamilla Heddeland Instefjord, Hilde Elshaug, Atiya R Ali,Marie Paulsen Madsen, Rasmus Riis Kopperud, Hilde Vollan, Karoline Bragstad, Olav Hungnes
EPI_ISL_775495	Unilabs Laboratory Medicine	Norwegian Institute of Public Health, Department of Virology	Kathrine Stene-Johansen, Kamilla Heddeland Instefjord, Hilde Elshaug, Atiya R Ali,Marie Paulsen Madsen, Rasmus Riis Kopperud, Hilde Vollan, Karoline Bragstad, Olav Hungnes
EPI_ISL_775499	Vestfold Hospital, Toensberg Department of Microbiology	Norwegian Institute of Public Health, Department of Virology	Kathrine Stene-Johansen, Kamilla Heddeland Instefjord, Hilde Elshaug, Atiya R Ali,Marie Paulsen Madsen, Rasmus Riis Kopperud, Hilde Vollan, Karoline Bragstad, Olav Hungnes
EPI_ISL_775511, EPI_ISL_775513, EPI_ISL_775514	Norwegian Institute of Public Health, Department of Virology	Norwegian Institute of Public Health, Department of Virology	Kathrine Stene-Johansen, Kamilla Heddeland Instefjord, Hilde Elshaug, Atiya R Ali,Marie Paulsen Madsen, Rasmus Riis Kopperud, Hilde Vollan, Karoline Bragstad, Olav Hungnes
EPI_ISL_776769	Instituto Adolfo Lutz - Regional de Santo Andre	Instituto Adolfo Lutz, Interdiciplinary Procedures Center, Strategic Laboratory	Claudio Tavares Sacchi, Claudia Regina Gonçalves, Erica Valessa Ramos Gomes, Karoline Rodrigues Campos
EPI_ISL_778871, EPI_ISL_778872, EPI_ISL_778891, EPI_ISL_778892, EPI_ISL_778893, EPI_ISL_778895, EPI_ISL_778896, EPI_ISL_778898, EPI_ISL_778899, EPI_ISL_778900, EPI_ISL_778908, EPI_ISL_778909, EPI_ISL_778911, EPI_ISL_778912, EPI_ISL_778913, EPI_ISL_778916, EPI_ISL_778917			
see above	Maryland Public Health Laboratory	Maryland Public Health Laboratory	Maryland Department of Health Laboratories Administration
EPI_ISL_779156, EPI_ISL_779159, EPI_ISL_779161, EPI_ISL_779163, EPI_ISL_779164, EPI_ISL_779167	Laboratório de Microbiologia Molecular - Universidade FEEVALE	Bioinformatics Laboratory / LNCC	Felipe Benites, Fernando Rosado Spilki, Alana Witt Hansen, Juliane Deise Fleck, Juliana Schons, Meriane Demoliner, Ana Karolina Eisen Antunes, Fagner Henrique Heldt, Larissa Mallmann, Bruna Hermann, Ana Luiza Ziulkoski, Vyctoria Goes, Karoline Schallenberger, Matheus Nunes Weber, Paula Rodrigues de Almeida, Alessandra Pavan Lamarca da Silva, Ronaldo da Silva F Jr , Luiz G P de Almeida, Alexandra L Gerber , Ana Paula de C Guimarães,Ana Tereza R de Vasconcelos
EPI_ISL_779968, EPI_ISL_779975, EPI_ISL_779981, EPI_ISL_779982, EPI_ISL_779985, EPI_ISL_779994, EPI_ISL_779996, EPI_ISL_780002, EPI_ISL_780007, EPI_ISL_780008, EPI_ISL_780012, EPI_ISL_780075, EPI_ISL_780076, EPI_ISL_780079, EPI_ISL_780086, EPI_ISL_780088			
see above	Hospital General Universitario Gregorio Marañón	SeqCOVID-SPAIN consortium/IBV(CSIC)	Darío García de Viedma, Laura Pérez-Lago, Marta Herranz, Jon Sicilia, Julia Suárez, Pilar Catalán, Patricia Muñoz and SeqCOVID-SPAIN consortium
EPI_ISL_780397, EPI_ISL_780398, EPI_ISL_780401	Bermuda Government Molecular Diagnostics Laboratory (MDL)	Respiratory Virus Unit, National Infection Service, Public Health England	PHE Covid Sequencing Team, Dr Carika Weldon (Bermuda), Dr Ayoola Oyinloye (Bermuda)
EPI_ISL_783069, EPI_ISL_783071, EPI_ISL_783073, EPI_ISL_783076, EPI_ISL_783080, EPI_ISL_783081, EPI_ISL_783083, EPI_ISL_783084, EPI_ISL_783085, EPI_ISL_783089, EPI_ISL_783090, EPI_ISL_783092, EPI_ISL_783093, EPI_ISL_783095, EPI_ISL_783097, EPI_ISL_783098, EPI_ISL_783099, EPI_ISL_783100, EPI_ISL_783103, EPI_ISL_783104, EPI_ISL_783105, EPI_ISL_783106, EPI_ISL_783108, EPI_ISL_783109, EPI_ISL_783110, EPI_ISL_783114, EPI_ISL_783115, EPI_ISL_783117, EPI_ISL_783118, EPI_ISL_783119, EPI_ISL_783120, EPI_ISL_783121, EPI_ISL_783123, EPI_ISL_783124, EPI_ISL_783127, EPI_ISL_783128, EPI_ISL_783131, EPI_ISL_783132, EPI_ISL_783133, EPI_ISL_783134, EPI_ISL_783137, EPI_ISL_783138, EPI_ISL_783139, EPI_ISL_783141, EPI_ISL_783142, EPI_ISL_783144, EPI_ISL_783145, EPI_ISL_783146, EPI_ISL_783147, EPI_ISL_783148, EPI_ISL_783149, EPI_ISL_783150, EPI_ISL_783152, EPI_ISL_783156, EPI_ISL_783157, EPI_ISL_783158, EPI_ISL_783162, EPI_ISL_783164, EPI_ISL_783166, EPI_ISL_783168, EPI_ISL_783170, EPI_ISL_783171, EPI_ISL_783172, EPI_ISL_783175, EPI_ISL_783177, EPI_ISL_783178, EPI_ISL_783179, EPI_ISL_783180, EPI_ISL_783181, EPI_ISL_783182, EPI_ISL_783183, EPI_ISL_783186, EPI_ISL_783187, EPI_ISL_783189, EPI_ISL_783190, EPI_ISL_783191, EPI_ISL_783193, EPI_ISL_783196, EPI_ISL_783197, EPI_ISL_783198, EPI_ISL_783199, EPI_ISL_783202, EPI_ISL_783203, EPI_ISL_783204, EPI_ISL_783206, EPI_ISL_783207, EPI_ISL_783210, EPI_ISL_783212, EPI_ISL_783213, EPI_ISL_783214, EPI_ISL_783220, EPI_ISL_783221, EPI_ISL_783225, EPI_ISL_783226, EPI_ISL_783228, EPI_ISL_783230, EPI_ISL_783231, EPI_ISL_783232, EPI_ISL_783233, EPI_ISL_783235, EPI_ISL_783237, EPI_ISL_783238, EPI_ISL_783241, EPI_ISL_783243, EPI_ISL_783244, EPI_ISL_783246, EPI_ISL_783247, EPI_ISL_783248, EPI_ISL_783249, EPI_ISL_783250, EPI_ISL_783251, EPI_ISL_783253, EPI_ISL_783254, EPI_ISL_783255, EPI_ISL_783256, EPI_ISL_783262, EPI_ISL_783265, EPI_ISL_783268, EPI_ISL_783269, EPI_ISL_783271, EPI_ISL_783272, EPI_ISL_783275, EPI_ISL_783276, EPI_ISL_783277, EPI_ISL_783278, EPI_ISL_783280, EPI_ISL_783282, EPI_ISL_783287, EPI_ISL_783289, EPI_ISL_783291, EPI_ISL_783292, EPI_ISL_783293, EPI_ISL_783295, EPI_ISL_783297, EPI_ISL_783300, EPI_ISL_783304, EPI_ISL_783305, EPI_ISL_783310, EPI_ISL_783311, EPI_ISL_783314, EPI_ISL_783316, EPI_ISL_783317, EPI_ISL_783320, EPI_ISL_783321, EPI_ISL_783322, EPI_ISL_783327, EPI_ISL_783328, EPI_ISL_783329, EPI_ISL_783331, EPI_ISL_783332, EPI_ISL_783333, EPI_ISL_783335, EPI_ISL_783339, EPI_ISL_783340, EPI_ISL_783341, EPI_ISL_783342, EPI_ISL_783343, EPI_ISL_783344, EPI_ISL_783345, EPI_ISL_783346, EPI_ISL_783347, EPI_ISL_783348, EPI_ISL_783349, EPI_ISL_783350, EPI_ISL_783351, EPI_ISL_783352, EPI_ISL_783354, EPI_ISL_783355, EPI_ISL_783357, EPI_ISL_783358, EPI_ISL_783360, EPI_ISL_783362, EPI_ISL_783363, EPI_ISL_783364, EPI_ISL_783366			
see above	Lighthouse Lab in Milton Keynes	Wellcome Sanger Institute for the COVID-19 Genomics UK (COG-UK) Consortium	The Lighthouse Lab in Milton Keynes and Alex Alderton, Roberto Amato, Sonia Goncalves, Ewan Harrison, David K. Jackson, Ian Johnston, Dominic Kwiatkowski, Cordelia Langford, John Sillitoe on behalf of the Wellcome Sanger Institute COVID-19 Surveillance Team
EPI_ISL_783569, EPI_ISL_783594, EPI_ISL_783595, EPI_ISL_783596, EPI_ISL_783597, EPI_ISL_783598, EPI_ISL_783600, EPI_ISL_783602, EPI_ISL_783603, EPI_ISL_783606, EPI_ISL_783608, EPI_ISL_783609, EPI_ISL_783610, EPI_ISL_783613, EPI_ISL_783614, EPI_ISL_783617, EPI_ISL_783620, EPI_ISL_783622, EPI_ISL_783630, EPI_ISL_783635, EPI_ISL_783636, EPI_ISL_783638, EPI_ISL_783640, EPI_ISL_783645, EPI_ISL_783649, EPI_ISL_783658, EPI_ISL_783660, EPI_ISL_783662, EPI_ISL_783663, EPI_ISL_783666, EPI_ISL_783676, EPI_ISL_783686, EPI_ISL_783695, EPI_ISL_783699, EPI_ISL_783704, EPI_ISL_783708, EPI_ISL_783721, EPI_ISL_783722, EPI_ISL_783725, EPI_ISL_783726, EPI_ISL_783727, EPI_ISL_783729, EPI_ISL_783733, EPI_ISL_783737, EPI_ISL_783742, EPI_ISL_783744, EPI_ISL_783758, EPI_ISL_783765, EPI_ISL_783798, EPI_ISL_783801, EPI_ISL_783803, EPI_ISL_783810, EPI_ISL_783822, EPI_ISL_783827, EPI_ISL_783828, EPI_ISL_783830, EPI_ISL_783841, EPI_ISL_783843, EPI_ISL_783848, EPI_ISL_783849, EPI_ISL_783853, EPI_ISL_783857, EPI_ISL_783860, EPI_ISL_783862, EPI_ISL_783865, EPI_ISL_783867, EPI_ISL_783877, EPI_ISL_783879, EPI_ISL_783881, EPI_ISL_783888, EPI_ISL_783891, EPI_ISL_783895, EPI_ISL_783896, EPI_ISL_783908, EPI_ISL_783913, EPI_ISL_783915, EPI_ISL_783927, EPI_ISL_783939, EPI_ISL_783940, EPI_ISL_783942, EPI_ISL_783947, EPI_ISL_783950, EPI_ISL_783969, EPI_ISL_783976, EPI_ISL_783981, EPI_ISL_783986, EPI_ISL_783991, EPI_ISL_783995, EPI_ISL_784010, EPI_ISL_784022, EPI_ISL_784029, EPI_ISL_784039, EPI_ISL_784040, EPI_ISL_784051, EPI_ISL_784058, EPI_ISL_784067, EPI_ISL_784070, EPI_ISL_784075, EPI_ISL_784078, EPI_ISL_784082, EPI_ISL_784088, EPI_ISL_784091, EPI_ISL_784094, EPI_ISL_784103, EPI_ISL_784107, EPI_ISL_784108, EPI_ISL_784110, EPI_ISL_784112,			

EPI_ISL_784113, EPI_ISL_784114, EPI_ISL_784115, EPI_ISL_784120, EPI_ISL_784132, EPI_ISL_784133, EPI_ISL_784134, EPI_ISL_784139, EPI_ISL_784140, EPI_ISL_784152, EPI_ISL_784153, EPI_ISL_784158, EPI_ISL_784168, EPI_ISL_784174, EPI_ISL_784177, EPI_ISL_784179, EPI_ISL_784180, EPI_ISL_784400, EPI_ISL_784410, EPI_ISL_784435, EPI_ISL_784449, EPI_ISL_784450, EPI_ISL_784459, EPI_ISL_784461, EPI_ISL_784600, EPI_ISL_784632, EPI_ISL_784634, EPI_ISL_784638, EPI_ISL_784643, EPI_ISL_784658, EPI_ISL_784701, EPI_ISL_784702, EPI_ISL_784710, EPI_ISL_784712, EPI_ISL_784715, EPI_ISL_784717, EPI_ISL_784718, EPI_ISL_784727, EPI_ISL_784734, EPI_ISL_784746, EPI_ISL_784747, EPI_ISL_784749, EPI_ISL_784751, EPI_ISL_784760, EPI_ISL_784763, EPI_ISL_784768, EPI_ISL_784769, EPI_ISL_784778, EPI_ISL_784784, EPI_ISL_784785, EPI_ISL_784787, EPI_ISL_784791, EPI_ISL_784799, EPI_ISL_784800, EPI_ISL_784801, EPI_ISL_784803, EPI_ISL_784804, EPI_ISL_784807, EPI_ISL_784808, EPI_ISL_784810, EPI_ISL_784813, EPI_ISL_784814, EPI_ISL_784817, EPI_ISL_784827, EPI_ISL_784829, EPI_ISL_784831, EPI_ISL_784832, EPI_ISL_784835, EPI_ISL_784851, EPI_ISL_784854, EPI_ISL_784863, EPI_ISL_784870, EPI_ISL_784871, EPI_ISL_784875, EPI_ISL_784883, EPI_ISL_784887, EPI_ISL_784890, EPI_ISL_784892, EPI_ISL_784897, EPI_ISL_784921, EPI_ISL_784923, EPI_ISL_784925, EPI_ISL_784929, EPI_ISL_784931, EPI_ISL_784944, EPI_ISL_784946, EPI_ISL_784950, EPI_ISL_784953, EPI_ISL_784954, EPI_ISL_784955, EPI_ISL_784957, EPI_ISL_784958, EPI_ISL_784960, EPI_ISL_784961, EPI_ISL_784962, EPI_ISL_784969, EPI_ISL_784976, EPI_ISL_784984, EPI_ISL_784986, EPI_ISL_784992, EPI_ISL_785006, EPI_ISL_785013, EPI_ISL_785014, EPI_ISL_785015, EPI_ISL_785040, EPI_ISL_785045, EPI_ISL_785051, EPI_ISL_785060, EPI_ISL_785063, EPI_ISL_785068, EPI_ISL_785073, EPI_ISL_785075, EPI_ISL_785076, EPI_ISL_785077, EPI_ISL_785078, EPI_ISL_785087, EPI_ISL_785088, EPI_ISL_785092, EPI_ISL_785096, EPI_ISL_785097, EPI_ISL_785098, EPI_ISL_785101, EPI_ISL_785103, EPI_ISL_785105, EPI_ISL_785106, EPI_ISL_785108, EPI_ISL_785110, EPI_ISL_785111, EPI_ISL_785112, EPI_ISL_785116, EPI_ISL_785120, EPI_ISL_785125, EPI_ISL_785129, EPI_ISL_785130, EPI_ISL_785135, EPI_ISL_785137, EPI_ISL_785138, EPI_ISL_785139, EPI_ISL_785141, EPI_ISL_785143, EPI_ISL_785149, EPI_ISL_785150, EPI_ISL_785153, EPI_ISL_785158, EPI_ISL_785161, EPI_ISL_785162, EPI_ISL_785167, EPI_ISL_785170, EPI_ISL_785173, EPI_ISL_785174, EPI_ISL_785176, EPI_ISL_785180, EPI_ISL_785183, EPI_ISL_785185, EPI_ISL_785186, EPI_ISL_785187, EPI_ISL_785192, EPI_ISL_785195, EPI_ISL_785197, EPI_ISL_785204, EPI_ISL_785205, EPI_ISL_785206, EPI_ISL_785211, EPI_ISL_785212, EPI_ISL_785214, EPI_ISL_785216, EPI_ISL_785217, EPI_ISL_785218, EPI_ISL_785220, EPI_ISL_785222, EPI_ISL_785225, EPI_ISL_785226, EPI_ISL_785228, EPI_ISL_785230, EPI_ISL_785236, EPI_ISL_785241, EPI_ISL_785244, EPI_ISL_785249, EPI_ISL_785252, EPI_ISL_785254, EPI_ISL_785255, EPI_ISL_785257, EPI_ISL_785258, EPI_ISL_785260, EPI_ISL_785263, EPI_ISL_785264, EPI_ISL_785265, EPI_ISL_785266, EPI_ISL_785270, EPI_ISL_785272, EPI_ISL_785275, EPI_ISL_785279, EPI_ISL_785282, EPI_ISL_785285, EPI_ISL_785287, EPI_ISL_785292, EPI_ISL_785293, EPI_ISL_785295, EPI_ISL_785296, EPI_ISL_786094, EPI_ISL_786095, EPI_ISL_786097, EPI_ISL_786102, EPI_ISL_786107, EPI_ISL_786111, EPI_ISL_786113, EPI_ISL_786114, EPI_ISL_786118, EPI_ISL_786120, EPI_ISL_786122, EPI_ISL_786134, EPI_ISL_786136, EPI_ISL_786140, EPI_ISL_786142, EPI_ISL_786143, EPI_ISL_786147, EPI_ISL_786149, EPI_ISL_786161, EPI_ISL_786164, EPI_ISL_786169, EPI_ISL_786170, EPI_ISL_786173, EPI_ISL_786182, EPI_ISL_786184, EPI_ISL_786186, EPI_ISL_786190			
see above	Houston Methodist Hospital	Houston Methodist Hospital	S. Wesley Long, Randall J. Olsen, Paul A. Christensen, David W. Bernard, James J. Davis, Maulik Shukla, Marcus Nguyen, Matthew Ojeda Saavedra, Prasanti Yerramilli, Layne Pruitt, Sishir Subedi, Heather Hendrickson, and James M. Musser
EPI_ISL_790595, EPI_ISL_790674, EPI_ISL_790695, EPI_ISL_790697	Dutch COVID-19 response team	National Institute for Public Health and the Environment (RIVM)	Adam Meijer, Harry Vennema, Jeroen Cremer, Sharon van den Brink, Bas van der Veer, AnneMarie van den Brandt, Florian Zwagemaker, Dennis Schmitz, Chantal Reusken, on behalf of the national COVID-19 response team
EPI_ISL_791298	National Virus Reference Laboratory	Irish Coronavirus Sequencing Consortium - Teagasc Moorepark	Alejandro Abner Garcia Leon, Paul Cotter, Fiona Crispie, John Kenny, Paddy Mallon, Calum Walsh
EPI_ISL_791343, EPI_ISL_791349, EPI_ISL_791351, EPI_ISL_791366, EPI_ISL_791369, EPI_ISL_791386, EPI_ISL_791387, EPI_ISL_791389, EPI_ISL_791392, EPI_ISL_791394, EPI_ISL_791395, EPI_ISL_791397, EPI_ISL_791399, EPI_ISL_791402, EPI_ISL_791404, EPI_ISL_791405, EPI_ISL_791406, EPI_ISL_791407, EPI_ISL_791408, EPI_ISL_791409, EPI_ISL_791410, EPI_ISL_791412, EPI_ISL_791414			
see above	Johns Hopkins Hospital Department of Pathology	Johns Hopkins Hospital Department of Pathology	C. Paul Morris, Chun Huai Luo, Heba H. Mostafa
EPI_ISL_791490, EPI_ISL_791502, EPI_ISL_791503, EPI_ISL_791509, EPI_ISL_791511	The Hosptial for Sick Children	The Hospital for Sick Children	Aaron Campigotto, Christian Marshall, Lynette Lau
EPI_ISL_791992	CHU - Hôpital Cavale Blanche	National Reference Center for Viruses of Respiratory Infections, Institut Pasteur, Paris	Marion Barbet, Sylvie Behillil, Méline Bizard, Angela Brisebarre, Camille Capel, Etienne Simon-Lorière, Vincent Enouf, Maud Vanpeene, Sylvie van der Werf, Léa Pilorgé
EPI_ISL_792059, EPI_ISL_792060, EPI_ISL_792061, EPI_ISL_792066, EPI_ISL_792067, EPI_ISL_792068, EPI_ISL_792069, EPI_ISL_792071, EPI_ISL_792073, EPI_ISL_792076, EPI_ISL_792079, EPI_ISL_792080			
see above	Toronto Invasive Bacterial Diseases Network	McMaster University	David Richardson, Allison McGeer, Patryk Aftanas, Hooman Derakhshani, Angel Li, Kuganya Nirmalarajah, Emily Panousis, Ahmed Draia, Jalees Nasir, Michael Surette, Samira Mubareka, Andrew G. McArthur
EPI_ISL_792603	Laboratório Central de Saúde Pública do Estado da Paraíba (LACEN-PB)	Laboratory of Respiratory Viruses and Measles, Oswaldo Cruz Institute, FIOCRUZ	Paola Resende, Luciana Appolinario, Fernando Motta, Anna Carolina Paixao, Ana Carolina Mendonca, João Felipe Bezerra, Romero Henrique Teixeira de Vasconcelos, Dalane Loudal Florentino Teixeira, Thiago Franco de Oliveira Carneiro, Marilda Siqueira on behalf of the Fiocruz COVID-19 Genomic Surveillance Network
EPI_ISL_792639, EPI_ISL_792641	Laboratório Central de Saúde Pública do Estado de Alagoas (LACEN-AL)	Laboratory of Respiratory Viruses and Measles, Oswaldo Cruz Institute, FIOCRUZ	Paola Resende, Luciana Appolinario, Fernando Motta, Anna Carolina Paixao, Ana Carolina Mendonca, Anderson Brandao Leite, Marilda Siqueira on behalf of the Fiocruz COVID-19 Genomic Surveillance Network
EPI_ISL_792649, EPI_ISL_792650, EPI_ISL_792651, EPI_ISL_792652, EPI_ISL_792654	Laboratório Central de Saúde Pública do Estado do Paraná (LACEN-PR)	Laboratory of Respiratory Viruses and Measles, Oswaldo Cruz Institute, FIOCRUZ	Paola Resende, Luciana Appolinario, Fernando Motta, Anna Carolina Paixao, Ana Carolina Mendonca, Maria do Carmo Debur, Irina Nastassja Riediger, Marilda Siqueira on behalf of the Fiocruz COVID-19 Genomic Surveillance Network
EPI_ISL_792686, EPI_ISL_792693, EPI_ISL_792695, EPI_ISL_792696, EPI_ISL_792705, EPI_ISL_792706, EPI_ISL_792707	The National Institute of Public Health	State Veterinary Institute Prague	Nagy,A;Jirincova,H;Trnka,D;Vecerova,J
EPI_ISL_793011, EPI_ISL_793012	Department of Virus and Microbiological Special Diagnostics, Statens Serum Institut, Copenhagen, Denmark	Albertsen Lab, Department of Chemistry and Bioscience, Aalborg University, Denmark	Danish Covid-19 Genome Consortium
EPI_ISL_794321, EPI_ISL_794322, EPI_ISL_794323	UZ Leuven, National Reference Laboratory for Coronaviruses, Laboratory Medicine, Leuven, Belgium	KU Leuven, Rega Institute, Clinical and Epidemiological Virology	Tony Wawina-Bokalanga, Joan Marti-Carerras, Bert Vanmechelen, Piet Maes
EPI_ISL_794412, EPI_ISL_794413, EPI_ISL_794414, EPI_ISL_794415, EPI_ISL_794416, EPI_ISL_794417, EPI_ISL_794418, EPI_ISL_794419, EPI_ISL_794555, EPI_ISL_794556, EPI_ISL_794557, EPI_ISL_794558, EPI_ISL_794560, EPI_ISL_794561, EPI_ISL_794562, EPI_ISL_794563, EPI_ISL_794564, EPI_ISL_794565, EPI_ISL_794566, EPI_ISL_794567, EPI_ISL_794568, EPI_ISL_794569, EPI_ISL_794570, EPI_ISL_794571, EPI_ISL_794572, EPI_ISL_794573, EPI_ISL_794574, EPI_ISL_794575, EPI_ISL_794576, EPI_ISL_794577, EPI_ISL_794579, EPI_ISL_794580, EPI_ISL_794581, EPI_ISL_794582, EPI_ISL_794583, EPI_ISL_794584, EPI_ISL_794586, EPI_ISL_794587, EPI_ISL_794588, EPI_ISL_794590			
see above	Department of Virus and Microbiological Special Diagnostics, Statens Serum Institut, Copenhagen, Denmark	Albertsen Lab, Department of Chemistry and Bioscience, Aalborg University, Denmark	Danish Covid-19 Genome Consortium
EPI_ISL_794749, EPI_ISL_794755	Istituto Zooprofilattico Sperimentale della Puglia e della Basilicata	Istituto Zooprofilattico Sperimentale della Puglia e della Basilicata	Parisi A., Bianco A., Capozzi L., Del Sambro L., Manzulli V, Rondinone V., Pace L., Cipolletta D., Galante D.
EPI_ISL_794902, EPI_ISL_795008, EPI_ISL_795009, EPI_ISL_795010	Department of Virus and Microbiological Special Diagnostics, Statens Serum Institut, Copenhagen, Denmark	Albertsen Lab, Department of Chemistry and Bioscience, Aalborg University, Denmark	Danish Covid-19 Genome Consortium
EPI_ISL_800885, EPI_ISL_800889, EPI_ISL_800890, EPI_ISL_800894, EPI_ISL_800895, EPI_ISL_800896, EPI_ISL_800897, EPI_ISL_800898, EPI_ISL_800901, EPI_ISL_800903, EPI_ISL_800908, EPI_ISL_800910, EPI_ISL_800912, EPI_ISL_800914, EPI_ISL_800916, EPI_ISL_800917, EPI_ISL_800919, EPI_ISL_800920, EPI_ISL_800921, EPI_ISL_800922, EPI_ISL_800923, EPI_ISL_800924, EPI_ISL_800926, EPI_ISL_800927, EPI_ISL_800930, EPI_ISL_800932, EPI_ISL_800934, EPI_ISL_800936, EPI_ISL_800938, EPI_ISL_800939, EPI_ISL_800940, EPI_ISL_800941, EPI_ISL_800943, EPI_ISL_800944, EPI_ISL_800950, EPI_ISL_800951, EPI_ISL_800952, EPI_ISL_800953, EPI_ISL_800955, EPI_ISL_800957, EPI_ISL_800958, EPI_ISL_800960, EPI_ISL_800963, EPI_ISL_800964, EPI_ISL_800965, EPI_ISL_800966, EPI_ISL_800967, EPI_ISL_800969, EPI_ISL_800973, EPI_ISL_800974, EPI_ISL_800975, EPI_ISL_800976, EPI_ISL_800977, EPI_ISL_800979, EPI_ISL_800980, EPI_ISL_800981, EPI_ISL_800989, EPI_ISL_800990, EPI_ISL_800991, EPI_ISL_800993, EPI_ISL_801001, EPI_ISL_801002, EPI_ISL_801004, EPI_ISL_801005, EPI_ISL_801006, EPI_ISL_801008, EPI_ISL_801010, EPI_ISL_801011, EPI_ISL_801013, EPI_ISL_801014, EPI_ISL_801016, EPI_ISL_801017, EPI_ISL_801018, EPI_ISL_801023, EPI_ISL_801024, EPI_ISL_801025, EPI_ISL_801026, EPI_ISL_801027, EPI_ISL_801028, EPI_ISL_801031, EPI_ISL_801032, EPI_ISL_801033, EPI_ISL_801035, EPI_ISL_801036, EPI_ISL_801037, EPI_ISL_801039, EPI_ISL_801041, EPI_ISL_801042, EPI_ISL_801044, EPI_ISL_801045, EPI_ISL_801047, EPI_ISL_801048, EPI_ISL_801049, EPI_ISL_801050, EPI_ISL_801053, EPI_ISL_801056, EPI_ISL_801057, EPI_ISL_801059, EPI_ISL_801060, EPI_ISL_801061, EPI_ISL_801064, EPI_ISL_801065, EPI_ISL_801066, EPI_ISL_801068, EPI_ISL_801069, EPI_ISL_801070, EPI_ISL_801072, EPI_ISL_801075, EPI_ISL_801078, EPI_ISL_801080, EPI_ISL_801081, EPI_ISL_801084, EPI_ISL_801085, EPI_ISL_801086, EPI_ISL_801089, EPI_ISL_801091, EPI_ISL_801092, EPI_ISL_801093, EPI_ISL_801095, EPI_ISL_801099, EPI_ISL_801100, EPI_ISL_801101, EPI_ISL_801102, EPI_ISL_801103, EPI_ISL_801105, EPI_ISL_801106, EPI_ISL_801107, EPI_ISL_801108, EPI_ISL_801109, EPI_ISL_801110, EPI_ISL_801111, EPI_ISL_801113, EPI_ISL_801116, EPI_ISL_801118, EPI_ISL_801120, EPI_ISL_801121, EPI_ISL_801122, EPI_ISL_801123, EPI_ISL_801124, EPI_ISL_801126, EPI_ISL_801128, EPI_ISL_801129, EPI_ISL_801130, EPI_ISL_801132, EPI_ISL_801133, EPI_ISL_801134, EPI_ISL_801135, EPI_ISL_801136, EPI_ISL_801137, EPI_ISL_801138, EPI_ISL_801139, EPI_ISL_801142, EPI_ISL_801143, EPI_ISL_801144, EPI_ISL_801145, EPI_ISL_801147, EPI_ISL_801148, EPI_ISL_801154, EPI_ISL_801158, EPI_ISL_801159, EPI_ISL_801160, EPI_ISL_801161, EPI_ISL_801163, EPI_ISL_801164, EPI_ISL_801166, EPI_ISL_801168, EPI_ISL_801169, EPI_ISL_801171, EPI_ISL_801172, EPI_ISL_801173, EPI_ISL_801174, EPI_ISL_801175, EPI_ISL_801176, EPI_ISL_801177, EPI_ISL_801178			
see above	Lighthouse Lab in Cambridge	Wellcome Sanger Institute for the COVID-19 Genomics UK (COG-UK) Consortium	Rob Howes, The Lighthouse Lab in Cambridge and Alex Alderton, Roberto Amato, Sonia Goncalves, Ewan Harrison, David K. Jackson, Ian Johnston, Dominic Kwiatkowski, Cordelia Langford, John Sillitoe on behalf of the Wellcome Sanger Institute COVID-19 Surveillance Team
EPI_ISL_801183, EPI_ISL_801185, EPI_ISL_801187, EPI_ISL_801188, EPI_ISL_801192, EPI_ISL_801193, EPI_ISL_801194, EPI_ISL_801195, EPI_ISL_801196, EPI_ISL_801198, EPI_ISL_801200, EPI_ISL_801201, EPI_ISL_801204, EPI_ISL_801205, EPI_ISL_801209, EPI_ISL_801210, EPI_ISL_801211, EPI_ISL_801213, EPI_ISL_801214, EPI_ISL_801215, EPI_ISL_801216, EPI_ISL_801223, EPI_ISL_801224, EPI_ISL_801226, EPI_ISL_801229, EPI_ISL_801230			
see above	Lighthouse Lab in Milton Keynes	Wellcome Sanger Institute for the COVID-19 Genomics UK (COG-UK) Consortium	The Lighthouse Lab in Milton Keynes and Alex Alderton, Roberto Amato, Sonia Goncalves, Ewan Harrison, David K. Jackson, Ian Johnston, Dominic Kwiatkowski, Cordelia Langford, John Sillitoe on behalf of the Wellcome Sanger Institute COVID-19 Surveillance Team
EPI_ISL_801450, EPI_ISL_801460, EPI_ISL_801461, EPI_ISL_801462, EPI_ISL_801463, EPI_ISL_801464, EPI_ISL_801465, EPI_ISL_801467, EPI_ISL_801468, EPI_ISL_801469, EPI_ISL_801470, EPI_ISL_801490			
see above	Dutch COVID-19 response team	Erasmus Medical Center	Bas Oude Munnink, Reina Sikkema, David Nieuwenhuijse, Irina Chestakova, Anne van der Linden, Marjan Boter, Emmanuelle Munger, Corine GeurtsvanKessel, Annemiek van der Eijk, Richard Molenkamp, Marion Koopmans, on behalf of the Dutch national COVID-19 response team.

EPI_ISL_802567	Essentia Health-St. Mary's Medical Center	Minnesota Department of Health, Public Health Laboratory	Alexandra Lorentz, Jacob Garfin, Matt Plumb, and Xiong Wang
EPI_ISL_802856, EPI_ISL_802857, EPI_ISL_802858	Vilnius University Hospital Santaros Klinikos, Vilnius University	Institute of Biotechnology, Life Sciences Center, Vilnius University	Emilija Vasiliunaite, Milda Norkiene, Albertas Timinskas, Alma Gedvilaite, Aurelija Zvirbliene, Daniel Naumovas, Laimonas Griskevicius
EPI_ISL_803100	Respiratory Viruses Branch, Centers for Disease Control and Prevention	Respiratory Viruses Branch, Centers for Disease Control and Prevention	Queen,K., Li,Y., Tao,Y., Uehara,A., Montmayeur,A., Paden,C.R., Cook,P.W., Marine,R., Sheth,M., Wang,H., Lee,J., Tong,S.
EPI_ISL_804226	Respiratory Virus Unit, National Infection Service, Public Health England	COVID-19 Genomics UK (COG-UK) Consortium	PHE Covid Sequencing Team
EPI_ISL_804582	MEPHI, Aix Marseille University	MEPHI, Aix Marseille University	Anthony LEVASSEUR
EPI_ISL_804791, EPI_ISL_804795, EPI_ISL_804799	Michigan Department of Health and Human Services, Bureau of Laboratories	Michigan Department of Health and Human Services, Bureau of Laboratories	Blankenship HM, Riner D, Soehnlen MK
EPI_ISL_804855	DC Public Health Lab/ Dept. of Forensic Sciences	DC Public Health Lab/ Dept. of Forensic Sciences	Scott Nguyen, Elizabeth Zelaya, Connie Maza, Monica Mann, Brittany Hamilton, David Payne, Jocelyn Hauser
EPI_ISL_807100, EPI_ISL_807121	Washington State Department of Health	Seattle Flu Study	Deborah A. Nickerson, Chris D. Frazar, Jover Lee, Benjamin Pelle, Matthew Richardson, Amanda Adler, Elisabeth Brandstetter, Peter D. Han, Kairsten Fay, Misja Ilcisin, Kirsten Lacombe, Thomas R. Sibley, Melissa Truong, Caitlin R. Wolf, Romesh Gautom, Geoff Melly, Brian Hiatt, Philip Dykema, Scott Lindquist, Michael Boeckh, Janet A. Englund, Michael Famulare, Barry R. Lutz, Mark J. Rieder, Lea M. Starita, Matthew Thompson, Helen Y. Chu, Jay Shendure, Trevor Bedford
EPI_ISL_812152, EPI_ISL_812153, EPI_ISL_812154, EPI_ISL_812155	GA Department of Public Health Laboratory	Pathogen Discovery, Respiratory Viruses Branch, Division of Viral Diseases, Centers for Disease Control and Prevention	Yan Li, Ying Tao, Anna Montmayeur, Jing Zhang, Brian Lynch, Krista Queen, Anna Uehara, Rachel Marine, Peter Cook, Clinton R. Paden, Haibin Wang, Suxiang Tong
EPI_ISL_812204, EPI_ISL_812205, EPI_ISL_812206, EPI_ISL_812210, EPI_ISL_812211, EPI_ISL_812212, EPI_ISL_812213, EPI_ISL_812214, EPI_ISL_812215, EPI_ISL_812217, EPI_ISL_812218, EPI_ISL_812221, EPI_ISL_812222, EPI_ISL_812223, EPI_ISL_812224, EPI_ISL_812225, EPI_ISL_812228, EPI_ISL_812229, EPI_ISL_812230, EPI_ISL_812231			
see above	TN Division of Laboratory Services	Pathogen Discovery, Respiratory Viruses Branch, Division of Viral Diseases, Centers for Disease Control and Prevention	Yan Li, Ying Tao, Anna Montmayeur, Jing Zhang, Brian Lynch, Krista Queen, Anna Uehara, Rachel Marine, Peter Cook, Clinton R. Paden, Haibin Wang, Suxiang Tong
EPI_ISL_813045, EPI_ISL_813047, EPI_ISL_813050, EPI_ISL_813051, EPI_ISL_813053, EPI_ISL_813054, EPI_ISL_813055, EPI_ISL_813059	University of Birmingham	COVID-19 Genomics UK (COG-UK) Consortium	Institute of Microbiology, University of Birmingham: Claire McMurray, Joanne Stockton, Samuel Nicholls, Radoslaw Poplawski, Will Rowe, Josh Quick, Nicholas Loman. University of Birmingham Testing Laboratory: Celina M Whalley, Andrew Bosworth, Charlotte Poxon, Kasun Wanigasooriya, Oliver Pickles, Mike Kidd, Alex Richter, Andrew D Beggs PHE Heartlands Lab: Husam Osman, Andrew Bosworth. Queen Elizabeth Hospital: Anna Casey
EPI_ISL_814571, EPI_ISL_814665, EPI_ISL_814680	Virology Department, Royal Infirmary of Edinburgh, NHS Lothian / School of Biological Sciences, University of Edinburgh / Institute of Genetics and Molecular Medicine, University of Edinburgh	COVID-19 Genomics UK (COG-UK) Consortium	McHugh M, Dewar R, Rooke S, Gallagher M, Balcaza C, O'Toole Á, Scher E, Hill V, McCrone JT, Colquhoun R, Yu X, Jackson B, Rambaut A, Williams TC, Templeton K
EPI_ISL_814771, EPI_ISL_814781, EPI_ISL_814783, EPI_ISL_814794, EPI_ISL_814797	Wales Specialist Virology Centre Sequencing lab: Pathogen Genomics Unit	COVID-19 Genomics UK (COG-UK) Consortium	Catherine Moore, Johnathan Evans, Laura Gifford, Malorie Perry, Simon Cottrell, Angela Marchbank, Alec Birchley, Alexander Adams, Amy Gaskin, Bree Gatica-Wilcox, Jason Coombes, Joel Southgate, Lauren Gilbert, Lee Graham, Nicole Pacchiarini, Sara Kumziene-Summerhayes, Sarah Taylor, Sophie Jones, Sara Rey, Matthew Bull, Joanne Watkins, Sally Corden, Tom Connor
EPI_ISL_815525, EPI_ISL_815558, EPI_ISL_815597, EPI_ISL_815598, EPI_ISL_815599, EPI_ISL_815600, EPI_ISL_815654, EPI_ISL_816068, EPI_ISL_816069, EPI_ISL_816070, EPI_ISL_816071, EPI_ISL_816072, EPI_ISL_816073, EPI_ISL_816074, EPI_ISL_816075, EPI_ISL_816076, EPI_ISL_816077, EPI_ISL_816078, EPI_ISL_816079, EPI_ISL_816080, EPI_ISL_816081, EPI_ISL_816082, EPI_ISL_816084, EPI_ISL_816085, EPI_ISL_816086, EPI_ISL_816087, EPI_ISL_816089, EPI_ISL_816090, EPI_ISL_816091, EPI_ISL_816092, EPI_ISL_816093, EPI_ISL_816094, EPI_ISL_816095, EPI_ISL_816096, EPI_ISL_816097, EPI_ISL_816098, EPI_ISL_816100, EPI_ISL_816101, EPI_ISL_816102, EPI_ISL_816103, EPI_ISL_816104, EPI_ISL_816106, EPI_ISL_816107, EPI_ISL_816108, EPI_ISL_816109, EPI_ISL_816110			
see above	Department of Virus and Microbiological Special Diagnostics, Statens Serum Institut, Copenhagen, Denmark	Albertsen Lab, Department of Chemistry and Bioscience, Aalborg University, Denmark	Danish Covid-19 Genome Consortium
EPI_ISL_819479, EPI_ISL_819493, EPI_ISL_819503, EPI_ISL_819504, EPI_ISL_819521, EPI_ISL_819522	Lincolnshire Hospitals and DeepSeq Nottingham	COVID-19 Genomics UK (COG-UK) Consortium	Nichola Duckworth, Tim Sloan, Sarah Walsh, Jonathan Ball, Patrick McClure, Joeseph Chappell, Nadine Holmes, Matthew Carlisle, Christopher Moore, Fei Sang, Johnny Debebe, Victoria Wright, Matthew Loose
EPI_ISL_823971	OCME Office Of Chief Medical Examiner	New York City Public Health Laboratory	Jade Wang, et al.
EPI_ISL_824000, EPI_ISL_824001, EPI_ISL_824008, EPI_ISL_824012, EPI_ISL_824017, EPI_ISL_824050, EPI_ISL_824126, EPI_ISL_824127, EPI_ISL_824131	Dutch COVID-19 response team	National Institute for Public Health and the Environment (RIVM)	Adam Meijer, Harry Vennema, Jeroen Cremer, Sharon van den Brink, Bas van der Veer, AnneMarie van den Brandt, Florian Zwagemaker, Dennis Schmitz, Chantal Reusken, on behalf of the national COVID-19 response team
EPI_ISL_824393, EPI_ISL_824395	California Department of Public Health	California Department of Public Health	CDPH IDLB COVIDNet
EPI_ISL_824518	New Mexico Department of Health Scientific Laboratory	New Mexico Department of Health Scientific Laboratory	Ellie Johnson, Anastacia Griego-Fisher, D'eldra Malone
EPI_ISL_824659, EPI_ISL_824670, EPI_ISL_824682, EPI_ISL_824694, EPI_ISL_824705, EPI_ISL_824706, EPI_ISL_824717, EPI_ISL_824718, EPI_ISL_824729, EPI_ISL_824740, EPI_ISL_824741			
see above	Cedars-Sinai Medical Center, Department of Pathology & Laboratory Medicine, Molecular Pathology Laboratory	Cedars-Sinai Medical Center, Molecular Pathology Laboratory of Department of Pathology & Laboratory Medicine and Genomic Core	Wenjuan Zhang, Brian Davis, Stephanie Chen, Jorge Mario Sincuir Martinez, Jasmine T Plummer, Eric Vail
EPI_ISL_825042	B.J. Medical College and Civil hospital, Ahmedabad	Gujarat Biotechnology Research Centre	Apurvasinh Puvar, Ramesh Pandit, Janvi Raval, Zarna Patel, Nitin Savaliya, Dinesh Kumar, Zuber Saiyed, Labdhi Pandya, Afzal Ansari, Nikha Trivedi, Pranay Shah, Kamlesh J Upadhyay, Sanjay Kapadia, Dipa Kinariwala, Chaitanya Joshi, Madhvi Joshi
EPI_ISL_825327, EPI_ISL_825335, EPI_ISL_825358, EPI_ISL_825360, EPI_ISL_825361, EPI_ISL_825362, EPI_ISL_825363, EPI_ISL_825364, EPI_ISL_825365, EPI_ISL_825366, EPI_ISL_825384			
see above	Hospital Universitari Vall d'Hebron - Vall d'Hebron Institut de Rercerca	Hospital Universitari Vall d'Hebron	Cristina Andrés, Maria Piñana, Josep F Abril, Damir Garcia-Cehic, Ariadna Rando, Juliana Esperalba, Maria Gema Codina, Carla Castillo, Maria Carmen Martin, Tomàs Pumarola, Josep Quer, Andrés Antón
EPI_ISL_825604	Respiratory Virus Unit, National Infection Service, Public Health England	COVID-19 Genomics UK (COG-UK) Consortium	PHE Covid Sequencing Team
EPI_ISL_826673, EPI_ISL_826723, EPI_ISL_826879, EPI_ISL_826914, EPI_ISL_826932, EPI_ISL_826995, EPI_ISL_826998, EPI_ISL_827068, EPI_ISL_827152, EPI_ISL_827267	deCODE genetics	deCODE genetics	Daniel F Gudbjartsson; Agnar Helgason; Hakon Jonsson; Olafur T Magnusson; Pall Melsted; Gudmundur L Norddahl; Jona Saemundsdottir; Asgeir Sigurdsson; Patrick Sulem; Arna B Agustsdottir; Hannes Eggertsson; Berglind Eiriksdottir; Run Fridriksdottir; Elisabet E Gardarsdottir; Gudmundur Georgsson; Olafia S Gretarsdottir; Kjartan R Gudmundsson; Thora R Gunnarsdottir; Arnaldur Gylfason; Hilma Holm; Brynjar O Jensson; Aslaug Jonasdottir; Kamilla S Josefsdottir; Thordur Kristjansson; Droplaug N Magnusdottir; Solvi Rognvaldsson; Louise le Roux; Gudrun Sigmundsdottir; Gardar Sveinbjornsson; Kristin E Sveinsdottir; Maney Sveinsdottir; Emil A Thorarensen; Bjarni Thorbjornsson; Gisli Masson; Ingileif Jonsdottir; Alma Moller; Thorolfur Gudnason; Karl G Kristinsson; Unnur Thorsteinsdottir; Kari Stefansson
EPI_ISL_827395	The National University Hospital of Iceland	deCODE genetics	Daniel F Gudbjartsson; Agnar Helgason; Hakon Jonsson; Olafur T Magnusson; Pall Melsted; Gudmundur L Norddahl; Jona Saemundsdottir; Asgeir Sigurdsson; Patrick Sulem; Arna B Agustsdottir; Hannes Eggertsson; Berglind Eiriksdottir; Run Fridriksdottir; Elisabet E Gardarsdottir; Gudmundur Georgsson; Olafia S Gretarsdottir; Kjartan R Gudmundsson; Thora R Gunnarsdottir; Arnaldur Gylfason; Hilma Holm; Brynjar O Jensson; Aslaug Jonasdottir; Kamilla S Josefsdottir; Thordur Kristjansson; Droplaug N Magnusdottir; Solvi Rognvaldsson; Louise le Roux; Gudrun Sigmundsdottir; Gardar Sveinbjornsson; Kristin E Sveinsdottir; Maney Sveinsdottir; Emil A Thorarensen; Bjarni Thorbjornsson; Gisli Masson; Ingileif Jonsdottir; Alma Moller; Thorolfur Gudnason; Karl G Kristinsson; Unnur Thorsteinsdottir; Kari Stefansson
EPI_ISL_827399, EPI_ISL_827400, EPI_ISL_827402, EPI_ISL_827442, EPI_ISL_827458, EPI_ISL_827826, EPI_ISL_827830, EPI_ISL_827861, EPI_ISL_827894, EPI_ISL_827906, EPI_ISL_827965, EPI_ISL_828026, EPI_ISL_828090, EPI_ISL_828093, EPI_ISL_828097, EPI_ISL_828110, EPI_ISL_828139, EPI_ISL_828148, EPI_ISL_828151, EPI_ISL_828152, EPI_ISL_828173			

see above	deCODE genetics	deCODE genetics	Daniel F Gudbjartsson; Agnar Helgason; Hakon Jonsson; Olafur T Magnusson; Pall Melsted; Gudmundur L Norddahl; Jona Saemundsdottir; Asgeir Sigurdsson; Patrick Sulem; Arna B Agustsdottir; Hannes Eggertsson; Berglind Eiriksdottir; Run Fridriksdottir; Elisabet E Gardarsdottir; Gudmundur Georgsson; Olafia S Gretarsdottir; Kjartan R Gudmundsson; Thora R Gunnarsdottir; Arnaldur Gylfason; Hilma Holm; Brynjar O Jensson; Aslaug Jonasdottir; Kamilla S Josefsdottir; Thordur Kristjansson; Droplaug N Magnusdottir; Solvi Rognvaldsson; Louise le Roux; Gudrun Sigmundsdottir; Gardar Sveinbjornsson; Kristin E Sveinsdottir; Maney Sveinsdottir; Emil A Thorarensen; Bjarni Thorbjornsson; Gisli Masson; Ingileif Jonsdottir; Alma Moller; Thorolfur Gudnason; Karl G Kristinsson; Unnur Thorsteinsdottir; Kari Stefansson
EPI_ISL_828175	The National University Hospital of Iceland	deCODE genetics	Daniel F Gudbjartsson; Agnar Helgason; Hakon Jonsson; Olafur T Magnusson; Pall Melsted; Gudmundur L Norddahl; Jona Saemundsdottir; Asgeir Sigurdsson; Patrick Sulem; Arna B Agustsdottir; Hannes Eggertsson; Berglind Eiriksdottir; Run Fridriksdottir; Elisabet E Gardarsdottir; Gudmundur Georgsson; Olafia S Gretarsdottir; Kjartan R Gudmundsson; Thora R Gunnarsdottir; Arnaldur Gylfason; Hilma Holm; Brynjar O Jensson; Aslaug Jonasdottir; Kamilla S Josefsdottir; Thordur Kristjansson; Droplaug N Magnusdottir; Solvi Rognvaldsson; Louise le Roux; Gudrun Sigmundsdottir; Gardar Sveinbjornsson; Kristin E Sveinsdottir; Maney Sveinsdottir; Emil A Thorarensen; Bjarni Thorbjornsson; Gisli Masson; Ingileif Jonsdottir; Alma Moller; Thorolfur Gudnason; Karl G Kristinsson; Unnur Thorsteinsdottir; Kari Stefansson
EPI_ISL_828179, EPI_ISL_828208, EPI_ISL_828233, EPI_ISL_828249, EPI_ISL_828409, EPI_ISL_828520, EPI_ISL_828920, EPI_ISL_828947, EPI_ISL_828964, EPI_ISL_829052, EPI_ISL_829137, EPI_ISL_829145, EPI_ISL_829150, EPI_ISL_829151, EPI_ISL_829176, EPI_ISL_829177, EPI_ISL_829182, EPI_ISL_829185, EPI_ISL_829198, EPI_ISL_829211, EPI_ISL_829343, EPI_ISL_829344, EPI_ISL_829345, EPI_ISL_829346, EPI_ISL_829347, EPI_ISL_829368, EPI_ISL_829380, EPI_ISL_829390, EPI_ISL_829437, EPI_ISL_829587, EPI_ISL_829588, EPI_ISL_829589, EPI_ISL_829591, EPI_ISL_829684, EPI_ISL_829728, EPI_ISL_829729, EPI_ISL_829730, EPI_ISL_829731, EPI_ISL_829852, EPI_ISL_829853, EPI_ISL_829854			
see above	deCODE genetics	deCODE genetics	Daniel F Gudbjartsson; Agnar Helgason; Hakon Jonsson; Olafur T Magnusson; Pall Melsted; Gudmundur L Norddahl; Jona Saemundsdottir; Asgeir Sigurdsson; Patrick Sulem; Arna B Agustsdottir; Hannes Eggertsson; Berglind Eiriksdottir; Run Fridriksdottir; Elisabet E Gardarsdottir; Gudmundur Georgsson; Olafia S Gretarsdottir; Kjartan R Gudmundsson; Thora R Gunnarsdottir; Arnaldur Gylfason; Hilma Holm; Brynjar O Jensson; Aslaug Jonasdottir; Kamilla S Josefsdottir; Thordur Kristjansson; Droplaug N Magnusdottir; Solvi Rognvaldsson; Louise le Roux; Gudrun Sigmundsdottir; Gardar Sveinbjornsson; Kristin E Sveinsdottir; Maney Sveinsdottir; Emil A Thorarensen; Bjarni Thorbjornsson; Gisli Masson; Ingileif Jonsdottir; Alma Moller; Thorolfur Gudnason; Karl G Kristinsson; Unnur Thorsteinsdottir; Kari Stefansson
EPI_ISL_829860	The National University Hospital of Iceland	deCODE genetics	Daniel F Gudbjartsson; Agnar Helgason; Hakon Jonsson; Olafur T Magnusson; Pall Melsted; Gudmundur L Norddahl; Jona Saemundsdottir; Asgeir Sigurdsson; Patrick Sulem; Arna B Agustsdottir; Hannes Eggertsson; Berglind Eiriksdottir; Run Fridriksdottir; Elisabet E Gardarsdottir; Gudmundur Georgsson; Olafia S Gretarsdottir; Kjartan R Gudmundsson; Thora R Gunnarsdottir; Arnaldur Gylfason; Hilma Holm; Brynjar O Jensson; Aslaug Jonasdottir; Kamilla S Josefsdottir; Thordur Kristjansson; Droplaug N Magnusdottir; Solvi Rognvaldsson; Louise le Roux; Gudrun Sigmundsdottir; Gardar Sveinbjornsson; Kristin E Sveinsdottir; Maney Sveinsdottir; Emil A Thorarensen; Bjarni Thorbjornsson; Gisli Masson; Ingileif Jonsdottir; Alma Moller; Thorolfur Gudnason; Karl G Kristinsson; Unnur Thorsteinsdottir; Kari Stefansson
EPI_ISL_829905, EPI_ISL_829906, EPI_ISL_829908, EPI_ISL_829976, EPI_ISL_829980, EPI_ISL_829983	deCODE genetics	deCODE genetics	Daniel F Gudbjartsson; Agnar Helgason; Hakon Jonsson; Olafur T Magnusson; Pall Melsted; Gudmundur L Norddahl; Jona Saemundsdottir; Asgeir Sigurdsson; Patrick Sulem; Arna B Agustsdottir; Hannes Eggertsson; Berglind Eiriksdottir; Run Fridriksdottir; Elisabet E Gardarsdottir; Gudmundur Georgsson; Olafia S Gretarsdottir; Kjartan R Gudmundsson; Thora R Gunnarsdottir; Arnaldur Gylfason; Hilma Holm; Brynjar O Jensson; Aslaug Jonasdottir; Kamilla S Josefsdottir; Thordur Kristjansson; Droplaug N Magnusdottir; Solvi Rognvaldsson; Louise le Roux; Gudrun Sigmundsdottir; Gardar Sveinbjornsson; Kristin E Sveinsdottir; Maney Sveinsdottir; Emil A Thorarensen; Bjarni Thorbjornsson; Gisli Masson; Ingileif Jonsdottir; Alma Moller; Thorolfur Gudnason; Karl G Kristinsson; Unnur Thorsteinsdottir; Kari Stefansson
EPI_ISL_830102, EPI_ISL_830103	The National University Hospital of Iceland	deCODE genetics	Daniel F Gudbjartsson; Agnar Helgason; Hakon Jonsson; Olafur T Magnusson; Pall Melsted; Gudmundur L Norddahl; Jona Saemundsdottir; Asgeir Sigurdsson; Patrick Sulem; Arna B Agustsdottir; Hannes Eggertsson; Berglind Eiriksdottir; Run Fridriksdottir; Elisabet E Gardarsdottir; Gudmundur Georgsson; Olafia S Gretarsdottir; Kjartan R Gudmundsson; Thora R Gunnarsdottir; Arnaldur Gylfason; Hilma Holm; Brynjar O Jensson; Aslaug Jonasdottir; Kamilla S Josefsdottir; Thordur Kristjansson; Droplaug N Magnusdottir; Solvi Rognvaldsson; Louise le Roux; Gudrun Sigmundsdottir; Gardar Sveinbjornsson; Kristin E Sveinsdottir; Maney Sveinsdottir; Emil A Thorarensen; Bjarni Thorbjornsson; Gisli Masson; Ingileif Jonsdottir; Alma Moller; Thorolfur Gudnason; Karl G Kristinsson; Unnur Thorsteinsdottir; Kari Stefansson
EPI_ISL_830157, EPI_ISL_830160, EPI_ISL_830174, EPI_ISL_830175, EPI_ISL_830197	deCODE genetics	deCODE genetics	Daniel F Gudbjartsson; Agnar Helgason; Hakon Jonsson; Olafur T Magnusson; Pall Melsted; Gudmundur L Norddahl; Jona Saemundsdottir; Asgeir Sigurdsson; Patrick Sulem; Arna B Agustsdottir; Hannes Eggertsson; Berglind Eiriksdottir; Run Fridriksdottir; Elisabet E Gardarsdottir; Gudmundur Georgsson; Olafia S Gretarsdottir; Kjartan R Gudmundsson; Thora R Gunnarsdottir; Arnaldur Gylfason; Hilma Holm; Brynjar O Jensson; Aslaug Jonasdottir; Kamilla S Josefsdottir; Thordur Kristjansson; Droplaug N Magnusdottir; Solvi Rognvaldsson; Louise le Roux; Gudrun Sigmundsdottir; Gardar Sveinbjornsson; Kristin E Sveinsdottir; Maney Sveinsdottir; Emil A Thorarensen; Bjarni Thorbjornsson; Gisli Masson; Ingileif Jonsdottir; Alma Moller; Thorolfur Gudnason; Karl G Kristinsson; Unnur Thorsteinsdottir; Kari Stefansson
EPI_ISL_830235, EPI_ISL_830237	The National University Hospital of Iceland	deCODE genetics	Daniel F Gudbjartsson; Agnar Helgason; Hakon Jonsson; Olafur T Magnusson; Pall Melsted; Gudmundur L Norddahl; Jona Saemundsdottir; Asgeir Sigurdsson; Patrick Sulem; Arna B Agustsdottir; Hannes Eggertsson; Berglind Eiriksdottir; Run Fridriksdottir; Elisabet E Gardarsdottir; Gudmundur Georgsson; Olafia S Gretarsdottir; Kjartan R Gudmundsson; Thora R Gunnarsdottir; Arnaldur Gylfason; Hilma Holm; Brynjar O Jensson; Aslaug Jonasdottir; Kamilla S Josefsdottir; Thordur Kristjansson; Droplaug N Magnusdottir; Solvi Rognvaldsson; Louise le Roux; Gudrun Sigmundsdottir; Gardar Sveinbjornsson; Kristin E Sveinsdottir; Maney Sveinsdottir; Emil A Thorarensen; Bjarni Thorbjornsson; Gisli Masson; Ingileif Jonsdottir; Alma Moller; Thorolfur Gudnason; Karl G Kristinsson; Unnur Thorsteinsdottir; Kari Stefansson
EPI_ISL_831032, EPI_ISL_831202, EPI_ISL_831203, EPI_ISL_831204, EPI_ISL_831205, EPI_ISL_831206, EPI_ISL_831207, EPI_ISL_831208, EPI_ISL_831209, EPI_ISL_831210, EPI_ISL_831211, EPI_ISL_831212, EPI_ISL_831245, EPI_ISL_831246			
see above	Hospital Universitario La Paz (Madrid)	SeqCOVID-SPAIN consortium/IBV(CSIC)	María Rodríguez-Tejedor, Elias Dahdouh, Fernando Lázaro-Perona, Jesús Mingorance and SeqCOVID-SPAIN consortium
EPI_ISL_831753, EPI_ISL_831756, EPI_ISL_831803, EPI_ISL_831804, EPI_ISL_831807, EPI_ISL_831808, EPI_ISL_831809, EPI_ISL_831810, EPI_ISL_831811, EPI_ISL_831823, EPI_ISL_831824, EPI_ISL_831825, EPI_ISL_831826, EPI_ISL_831827, EPI_ISL_831828, EPI_ISL_831829, EPI_ISL_831830, EPI_ISL_831831, EPI_ISL_831832, EPI_ISL_831833, EPI_ISL_831834, EPI_ISL_831835, EPI_ISL_831843, EPI_ISL_831856, EPI_ISL_831858			
see above	United States Air Force School of Aerospace Medicine	United States Air Force School of Aerospace Medicine	Anthony Fries, Jennifer Meyer, William Gruner, Amanda Javorina, Sarah Purves, Clarise Starr, Elizabeth Macias
EPI_ISL_832038	Wyoming Public Health Laboratory	Wyoming Public Health Laboratory	Noah Hull, Taylor Fearing, Lynette Gumbleton, Channing Weber, Ashley Norberg, Bailey Bowcutt, and Wanda Manley
EPI_ISL_832146, EPI_ISL_832147, EPI_ISL_832148, EPI_ISL_832149, EPI_ISL_832150, EPI_ISL_832151, EPI_ISL_832152, EPI_ISL_832153, EPI_ISL_832154, EPI_ISL_832155, EPI_ISL_832156, EPI_ISL_832157, EPI_ISL_832158, EPI_ISL_832159			
see above	Hospital	National Reference Center for Viruses of Respiratory Infections, Institut Pasteur, Paris	Marion Barbet, Sylvie Behillil, Méline Bizard, Angela Brisebarre, Camille Capel, Etienne Simon-Lorière, Vincent Enouf, Maud Vanpeene, Sylvie van der Werf, Léa Pilorge
EPI_ISL_833162	Lab LOC - Itapecerica da Serra	Instituto Adolfo Lutz, Interdiciplinary Procedures Center, Strategic Laboratory	Claudio Tavares Sacchi, Claudia Regina Gonçalves, Erica Valessa Ramos Gomes, Karoline Rodrigues Campos
EPI_ISL_833163	Instituto Adolfo Lutz - Regional de Marilia	Instituto Adolfo Lutz, Interdiciplinary Procedures Center, Strategic Laboratory	Claudio Tavares Sacchi, Claudia Regina Gonçalves, Erica Valessa Ramos Gomes, Karoline Rodrigues Campos
EPI_ISL_833458, EPI_ISL_833463, EPI_ISL_833471, EPI_ISL_833475, EPI_ISL_833476, EPI_ISL_833479	CHU Purpan - Laboratoire de Virologie - Institut Fédératif de Biologie	CHU Purpan - Laboratoire de Virologie - Institut Fédératif de Biologie	Latour J., Ranger N., Dubois M., Carcenac R., Harter A., Boyer P., Tremeaux P., Izopet J.
EPI_ISL_838055, EPI_ISL_838058, EPI_ISL_838059	West of Scotland Specialist Virology Centre, NHSGGC / MRC-University of Glasgow Centre for Virus Research	COVID-19 Genomics UK (COG-UK) Consortium	Ana da Silva Filipe, Natasha Johnson, Kathy Smollett, Daniel Mair, Stephen Carmichael, Alice Broos, Lily Tong, Jenna Nichols, Kyriaki Nomikou; Sarah McDonald; Richard Orton, Joseph Hughes, Sreenu Vattipally, David L Robertson; Alasdair MacLean, Rory Gunson; Sharif Shaaban, Matthew Holden; Rachel Blacow, Guy Mollett, Kathy Li, James Shepherd, Antonia Ho, Emma Thomson
EPI_ISL_838282	Virology Department, Royal Infirmary of Edinburgh, NHS Lothian / School of Biological Sciences, University of Edinburgh / Institute of Genetics and Molecular Medicine, University of Edinburgh	COVID-19 Genomics UK (COG-UK) Consortium	McHugh M, Dewar R, Rooke S, Gallagher M, Balcaza C, O'Toole Á, Scher E, Hill V, McCrone JT, Colquhoun R, Yu X, Jackson B, Rambaut A, Williams TC, Templeton K
EPI_ISL_840192, EPI_ISL_840193, EPI_ISL_840195, EPI_ISL_840198, EPI_ISL_840199, EPI_ISL_840204, EPI_ISL_840205, EPI_ISL_840210, EPI_ISL_840214, EPI_ISL_840215, EPI_ISL_840216			
see above	Oxford Viromics, NDM, University of Oxford; Oxford University	COVID-19 Genomics UK (COG-UK) Consortium	Tanya Golubchik, David Bonsall, George Macintyre, Amy Trebes, Mariateresa de Cesare, Catrin Moore, Alex Mobbs, Anita Justice, Robert Shaw, Monique

	Hospitals; Basingstoke and North Hampshire Hospital		Andersson, Timothy Peto, Emma Wise, Nathan Moore, Jessica Lynch, Nick Cortes, Matilde Mori, Stephen Kidd, David Buck, John Todd, Christophe Fraser
EPI_ISL_842931, EPI_ISL_842935, EPI_ISL_842941, EPI_ISL_842949, EPI_ISL_842953, EPI_ISL_842966, EPI_ISL_842968, EPI_ISL_842970, EPI_ISL_842975, EPI_ISL_842981, EPI_ISL_842992, EPI_ISL_842993, EPI_ISL_843005, EPI_ISL_843015, EPI_ISL_843017, EPI_ISL_843027, EPI_ISL_843041, EPI_ISL_843043			
see above	Barts Health NHS Trust	COVID-19 Genomics UK (COG-UK) Consortium	CUTINO-MOGUEL, Maria-Teresa; HARRINGTON, David; OWOYEMI, Dola; SHYLINI, Raghavendran; BROAD, Claire; KELE, Beatrix
EPI_ISL_844252, EPI_ISL_844287, EPI_ISL_844288, EPI_ISL_844289, EPI_ISL_844290, EPI_ISL_844291, EPI_ISL_844292, EPI_ISL_844293, EPI_ISL_844294, EPI_ISL_844462, EPI_ISL_844463, EPI_ISL_844464, EPI_ISL_844465, EPI_ISL_844466, EPI_ISL_844467, EPI_ISL_844468, EPI_ISL_844469, EPI_ISL_844470, EPI_ISL_844471, EPI_ISL_844472, EPI_ISL_844473, EPI_ISL_844474, EPI_ISL_844475, EPI_ISL_844476, EPI_ISL_844477, EPI_ISL_844479, EPI_ISL_844480, EPI_ISL_844481, EPI_ISL_844482, EPI_ISL_844483, EPI_ISL_844484, EPI_ISL_844485, EPI_ISL_844486, EPI_ISL_844489, EPI_ISL_844490, EPI_ISL_844491, EPI_ISL_844492, EPI_ISL_844494, EPI_ISL_844495, EPI_ISL_844496, EPI_ISL_844497, EPI_ISL_844498, EPI_ISL_844502, EPI_ISL_844503, EPI_ISL_844504, EPI_ISL_844505, EPI_ISL_844506, EPI_ISL_844507, EPI_ISL_844508, EPI_ISL_844509, EPI_ISL_844511, EPI_ISL_844512, EPI_ISL_844513, EPI_ISL_844514, EPI_ISL_844515, EPI_ISL_844516, EPI_ISL_844517, EPI_ISL_844518, EPI_ISL_844519, EPI_ISL_844520, EPI_ISL_844521, EPI_ISL_844522, EPI_ISL_844524, EPI_ISL_844525, EPI_ISL_844526, EPI_ISL_844527, EPI_ISL_844529, EPI_ISL_844530, EPI_ISL_844531, EPI_ISL_844532, EPI_ISL_844533, EPI_ISL_844534, EPI_ISL_844535, EPI_ISL_844536, EPI_ISL_844537, EPI_ISL_844538, EPI_ISL_844539, EPI_ISL_844540			
see above	Department of Virus and Microbiological Special Diagnostics, Statens Serum Institut, Copenhagen, Denmark	Albertsen Lab, Department of Chemistry and Bioscience, Aalborg University, Denmark	Danish Covid-19 Genome Consortium
EPI_ISL_846771	University of Michigan Clinical Microbiology Laboratory	Lauring Lab, University of Michigan, Department of Microbiology and Immunology	Valesano
EPI_ISL_847803, EPI_ISL_847808, EPI_ISL_847809, EPI_ISL_847810, EPI_ISL_847815	California Department of Public Health	Chiu Laboratory, University of California, San Francisco	Charles Chiu, Xianding (Wayne) Deng, Candace Wang, Brian Bushnell, Scot Federman, Jill Hacker, Debra Wadford
EPI_ISL_848439, EPI_ISL_848527, EPI_ISL_848528, EPI_ISL_848529, EPI_ISL_848530, EPI_ISL_848531, EPI_ISL_848532	Illinois Department of Public Health	Gagnon Lab, Southern Illinois University	Keith Gagnon
EPI_ISL_848715, EPI_ISL_848716, EPI_ISL_848727, EPI_ISL_848755, EPI_ISL_848757, EPI_ISL_848772, EPI_ISL_848773, EPI_ISL_848774, EPI_ISL_848782, EPI_ISL_848805, EPI_ISL_848807, EPI_ISL_848809			
see above	Florida Bureau of Public Health Laboratories	Florida Bureau of Public Health Laboratories	Sarah Schmedes, Jason Blanton
EPI_ISL_849558	Washington State Department of Health	Seattle Flu Study	Deborah A. Nickerson, Chris D. Frazar, Jover Lee, Benjamin Pelle, Matthew Richardson, Amanda Adler, Elisabeth Brandstetter, Peter D. Han, Kairsten Fay, Misja Ilcisin, Kirsten Lacombe, Thomas R. Sibley, Melissa Truong, Caitlin R. Wolf, Romesh Gautom, Geoff Melly, Brian Hiatt, Philip Dykema, Scott Lindquist, Michael Boeckh, Janet A. Englund, Michael Famulare, Barry R. Lutz, Mark J. Rieder, Lea M. Starita, Matthew Thompson, Helen Y. Chu, Jay Shendure, Trevor Bedford
EPI_ISL_850041, EPI_ISL_850047, EPI_ISL_850068, EPI_ISL_850088, EPI_ISL_850098, EPI_ISL_850100, EPI_ISL_850102, EPI_ISL_850110, EPI_ISL_850114, EPI_ISL_850127, EPI_ISL_850130, EPI_ISL_850176			
see above	Renegade	Chan-Zuckerberg Biohub	CZB Cliahub Consortium
EPI_ISL_852633, EPI_ISL_852634, EPI_ISL_852635, EPI_ISL_852636, EPI_ISL_852637, EPI_ISL_852638, EPI_ISL_852639, EPI_ISL_852640, EPI_ISL_852641, EPI_ISL_852642, EPI_ISL_852643, EPI_ISL_852644, EPI_ISL_852645, EPI_ISL_852646, EPI_ISL_852647, EPI_ISL_852648, EPI_ISL_852649			
see above	Institute of Virology, Medical Center, University of Freiburg, Freiburg, Germany	Institute of Virology, Clinial Virus Genomics, Medical Center, University of Freiburg, Freiburg, Germany	Jonas Fuchs, Lisa Kern, Sandra Reuter, Hajo Grundmann, Marcus Panning
EPI_ISL_852845, EPI_ISL_852846, EPI_ISL_852847, EPI_ISL_852848, EPI_ISL_852850, EPI_ISL_852856, EPI_ISL_852864, EPI_ISL_852865	Toronto Invasive Bacterial Diseases Network	Ontario Institute for Cancer Research	Allison McGeer, Patryk Aftanas, Angel Li, Kuganya Nirmalarajah, Samira Mubareka, Ilinca Lungu, Lubaina Kothari, Bernard Lam, Paul Krzyzanowski, Michael Laszloffy, Lawrence E. Heisler, Richard de Borja, Jared T. Simpson
EPI_ISL_857496, EPI_ISL_857497, EPI_ISL_857498, EPI_ISL_857499, EPI_ISL_857501, EPI_ISL_857502, EPI_ISL_857503, EPI_ISL_857513	Swiss National Reference Centre for Influenza	Swiss National Reference Centre for Influenza	Ana Rita Goncalves,Samuel Cordey, Laurent Kaiser, Lorenzo Cerutti, Henri Pegeot, Melyssa Elies, Keith Harshman, Ioannis Xenarios, Emmanouil Dermitzakis
EPI_ISL_857520, EPI_ISL_857521, EPI_ISL_857522, EPI_ISL_857523, EPI_ISL_857524, EPI_ISL_857525, EPI_ISL_857526, EPI_ISL_857527, EPI_ISL_857540	Swiss National Reference Centre for Influenza	Swiss National Reference Centre for Influenza	Tim Roloff, Ana Rita Gonçalves, Madlen Stange, Helena MB Seth-Smith, Alfredo Mari, Karoline Leuzinger, Julia Bielicki, Manuel Battegay, Hans Hirsch, Laurent Kaiser, Adrian Egli
EPI_ISL_859554, EPI_ISL_859556, EPI_ISL_859557, EPI_ISL_859558, EPI_ISL_859559, EPI_ISL_859560	Lighthouse Lab in Cambridge	Wellcome Sanger Institute for the COVID-19 Genomics UK (COG-UK) Consortium	Rob Howes, The Lighthouse Lab in Cambridge and Alex Alderton, Roberto Amato, Sonia Goncalves, Ewan Harrison, David K. Jackson, Ian Johnston, Dominic Kwiatkowski, Cordelia Langford, John Sillitoe on behalf of the Wellcome Sanger Institute COVID-19 Surveillance Team
EPI_ISL_859846, EPI_ISL_859847, EPI_ISL_859848, EPI_ISL_859849, EPI_ISL_859850, EPI_ISL_859851, EPI_ISL_859853, EPI_ISL_859854, EPI_ISL_859855, EPI_ISL_859856, EPI_ISL_859857, EPI_ISL_859858, EPI_ISL_859859, EPI_ISL_859860, EPI_ISL_859861, EPI_ISL_859862, EPI_ISL_859864, EPI_ISL_859866, EPI_ISL_859867, EPI_ISL_859868, EPI_ISL_859869, EPI_ISL_859870, EPI_ISL_859871, EPI_ISL_859872, EPI_ISL_859873, EPI_ISL_859874, EPI_ISL_859875, EPI_ISL_859876, EPI_ISL_859877, EPI_ISL_859879, EPI_ISL_859880, EPI_ISL_859881, EPI_ISL_859882, EPI_ISL_859883, EPI_ISL_859884, EPI_ISL_859885, EPI_ISL_859886, EPI_ISL_859887, EPI_ISL_859895, EPI_ISL_859896, EPI_ISL_859897, EPI_ISL_859898, EPI_ISL_859899, EPI_ISL_859900, EPI_ISL_859901, EPI_ISL_859902, EPI_ISL_859903, EPI_ISL_859904, EPI_ISL_859905, EPI_ISL_859907, EPI_ISL_859908, EPI_ISL_859909, EPI_ISL_859910, EPI_ISL_859911, EPI_ISL_859912, EPI_ISL_859913, EPI_ISL_859916, EPI_ISL_859917, EPI_ISL_859918, EPI_ISL_859919, EPI_ISL_859920, EPI_ISL_859921, EPI_ISL_859922, EPI_ISL_859923, EPI_ISL_859924, EPI_ISL_859925, EPI_ISL_859926, EPI_ISL_859927, EPI_ISL_859928, EPI_ISL_859929, EPI_ISL_859930, EPI_ISL_859931, EPI_ISL_859932, EPI_ISL_859933, EPI_ISL_859934, EPI_ISL_859935			
see above	BTC, Khalifa University	BTC, Khalifa University	Al Safar et al
EPI_ISL_860123, EPI_ISL_860134, EPI_ISL_860152, EPI_ISL_860153, EPI_ISL_860154, EPI_ISL_860156, EPI_ISL_860161, EPI_ISL_860162, EPI_ISL_860163, EPI_ISL_860164, EPI_ISL_860165, EPI_ISL_860166, EPI_ISL_860167, EPI_ISL_860169, EPI_ISL_860171, EPI_ISL_860173			
see above	Keio University School of Medicine	Keio University School of Medicine	Kenjiro Kosaki, Yuka Iwasaki, Hirotsugu Ishizu, Haruhiko Siomi, Kodai Abe
EPI_ISL_860804	WHO/Minsk	Charité Universitätsmedizin Berlin, Institut für Virologie	Victor M Corman, Barbara Mühlemann, Jörn Beheim-Schwarzbach, Talitha Veith, Julia Tesch, Tobias Bleicker, Julia Schneider, Shmialiova Natallia, Sivets Natallia, Terry Jones, Christian Drosten
EPI_ISL_861881, EPI_ISL_861882, EPI_ISL_861883, EPI_ISL_861886, EPI_ISL_861887, EPI_ISL_861888, EPI_ISL_861894, EPI_ISL_861903, EPI_ISL_861904, EPI_ISL_861908, EPI_ISL_861909			
see above	LATE - Laboratório de Técnicas Especiais - Hospital Israelita Albert Einstein	LATE - Laboratório de Técnicas Especiais - Hospital Israelita Albert Einstein	Deyvid Amgarten, Fernanda de Mello Malta, Raquel Riyuzo, Ana Paula Moreira Salles, Pedro Henrique Sebe Rodrigues, João Renato Rebello Pinho
EPI_ISL_862076	National Influenza Center, Virology Department	National Influenza Center	NZ Shafiei Jandaghi, V Salimi, A Nejati, K Sadeghi, J Yavarian, N Ghavvami,F Ajaminejad and T Mokhtari Azad
EPI_ISL_862080	National Influenza Center, Virology Department	National Influenza Center	J Yavarian, NZ Shafiei Jandaghi, V Salimi, A Nejati, K Sadeghi, F Ajaminejad,N Ghavvami and T Mokhtari Azad
EPI_ISL_862081	National Influenza Center, Virology Department	National Influenza Center	NZ Shafiei Jandaghi, V Salimi, A Nejati, K Sadeghi, J Yavarian, F Ajaminejad,N Ghavvami and T Mokhtari Azad
EPI_ISL_866822	Quadram Institute Bioscience	COVID-19 Genomics UK (COG-UK) Consortium	Dave J. Baker, Gemma L. Kay, Alp Aydin, Thanh Le-Viet, Steven Rudder, Ana P. Tedim, Anastasia Kolyva, Maria Diaz, Leonardo de Oliveira Martins, Nabil-Fareed Alikhan, Lizzie Meadows, Rachael Stanley, Ngozi Elumogo, Muhammed Yasir, Nicholas M. Thomson, Alexander J Trotter, Rachel Gilroy, Samuel Bloomfield, Claire Stuart, Andrew Bell, Reenesh Prakash, Samir Dervisevic, Alison E. Mather, John Wain, Mark Webber, Andrew J. Page, Justin O'Grady
EPI_ISL_868766, EPI_ISL_868781, EPI_ISL_868882, EPI_ISL_868883, EPI_ISL_868885, EPI_ISL_868886, EPI_ISL_868894, EPI_ISL_868895, EPI_ISL_868897, EPI_ISL_868984, EPI_ISL_868986, EPI_ISL_868987, EPI_ISL_868988, EPI_ISL_868999, EPI_ISL_869003, EPI_ISL_869005, EPI_ISL_869006, EPI_ISL_869013			
see above	Bioinformatics and Biostatistics Lab, Advanced Sequencing Facility	COVID-19 Genomics UK (COG-UK) Consortium	Aengus Stewart,Jerome Nicod,Chelsea Sawyer,Laura Cubitt,Harshil Patel,Margaret Crawford
EPI_ISL_869263	Krasnoyarsk Regional Clinical Hospital	WHO National Influenza Centre Russian Federation	Andrey Komissarov, Artem Fadeev, Anna Ivanova, Kseniya Komissarova, Dmitry Bazhenov, Mikhail Bakaev, Daria Danilenko, Ksenia Safina, Elena Nabieva, Georgii Bazykin, Dmitry Lioznov

EPI_ISL_871781, EPI_ISL_871782, EPI_ISL_871783, EPI_ISL_871784, EPI_ISL_871785	Department of Virus and Microbiological Special Diagnostics, Statens Serum Institut, Copenhagen, Denmark	Aalborg University	Danish Covid-19 Genome Consortium
EPI_ISL_872608, EPI_ISL_872609, EPI_ISL_872611	Nigeria Centre for Disease Control (NCDC)	African Centre of Excellence for Genomics of Infectious Diseases (ACEGID), Redeemer's University	Oluniyi P.E. et al
EPI_ISL_872700	Rhode Island Department of Health	Infectious Disease Program, Broad Institute of Harvard and MIT	Lemieux,J.E., Siddle,K.J., Huard,R., King,E., Azevedo,K., Miller,A., Adams,G., Gladden-Young,A., Lagerborg,K., Rudy,M., DeRuff,K., Carter,A., Normandin,E., Bauer,M., Reilly,S., Tomkins-Tinch,C., Loreth,C., Chaluvadi,S., Birren,B.W., Gallagher,G., Smole,S., Park,D.J., MacInnis,B.L., and Sabeti,P.C.
EPI_ISL_873126, EPI_ISL_873127, EPI_ISL_873135, EPI_ISL_873137, EPI_ISL_873142, EPI_ISL_873145, EPI_ISL_873154	University of Michigan Clinical Microbiology Laboratory	Lauring Lab, University of Michigan, Department of Microbiology and Immunology	Valesano
EPI_ISL_875553, EPI_ISL_875554	ULSS 2 Treviso	Istituto Zooprofilattico Sperimentale delle Venezie	Adelaide Milani, Alessia Schivo, Annalisa Salviato, Erika Giorgia Quaranta, Ambra Pastori, Bianca Zecchin, Alice Fusaro, Isabella Monne, Calogero Terregino, Antonia Ricci
EPI_ISL_876318, EPI_ISL_876326	Massachusetts State Public Health Laboratory	Massachusetts State Public Health Laboratory	Andrew Lang, Timelia Fink, Glen Gallagher, Sandra Smole
EPI_ISL_876552, EPI_ISL_876554, EPI_ISL_876555, EPI_ISL_876556, EPI_ISL_876559, EPI_ISL_876560, EPI_ISL_876561, EPI_ISL_876562, EPI_ISL_876563, EPI_ISL_876566, EPI_ISL_876567			
see above	Florida Bureau of Public Health Laboratories	Florida Bureau of Public Health Laboratories	Sarah Schmedes, Jason Blanton
EPI_ISL_877331, EPI_ISL_877332, EPI_ISL_877333, EPI_ISL_877334, EPI_ISL_877335, EPI_ISL_877336, EPI_ISL_877337, EPI_ISL_877338, EPI_ISL_877339, EPI_ISL_877340, EPI_ISL_877341, EPI_ISL_877342, EPI_ISL_877343, EPI_ISL_877344, EPI_ISL_877345, EPI_ISL_877346, EPI_ISL_877347, EPI_ISL_877348, EPI_ISL_877349, EPI_ISL_877351, EPI_ISL_877352, EPI_ISL_877353, EPI_ISL_877354, EPI_ISL_877355, EPI_ISL_877356, EPI_ISL_877357, EPI_ISL_877358, EPI_ISL_877359, EPI_ISL_877360, EPI_ISL_877361, EPI_ISL_877362, EPI_ISL_877363, EPI_ISL_877364, EPI_ISL_877365, EPI_ISL_877366, EPI_ISL_877367, EPI_ISL_877368, EPI_ISL_877369, EPI_ISL_877370, EPI_ISL_877371, EPI_ISL_877372, EPI_ISL_877373, EPI_ISL_877374, EPI_ISL_877375, EPI_ISL_877376, EPI_ISL_877377, EPI_ISL_877378, EPI_ISL_877379, EPI_ISL_877380, EPI_ISL_877381, EPI_ISL_877382, EPI_ISL_877383, EPI_ISL_877384, EPI_ISL_877385, EPI_ISL_877386, EPI_ISL_877387, EPI_ISL_877388, EPI_ISL_877389, EPI_ISL_877390, EPI_ISL_877391, EPI_ISL_877392, EPI_ISL_877393, EPI_ISL_877394, EPI_ISL_877395, EPI_ISL_877396, EPI_ISL_877397, EPI_ISL_877398, EPI_ISL_877399, EPI_ISL_877400, EPI_ISL_877401, EPI_ISL_877402, EPI_ISL_877403, EPI_ISL_877404, EPI_ISL_877405, EPI_ISL_877406, EPI_ISL_877407, EPI_ISL_877408, EPI_ISL_877409, EPI_ISL_877410, EPI_ISL_877461, EPI_ISL_877462, EPI_ISL_877463, EPI_ISL_877464, EPI_ISL_877466, EPI_ISL_877467, EPI_ISL_877469, EPI_ISL_877470, EPI_ISL_877471, EPI_ISL_877472, EPI_ISL_877473, EPI_ISL_877474, EPI_ISL_877475, EPI_ISL_877476, EPI_ISL_877477, EPI_ISL_877478, EPI_ISL_877479, EPI_ISL_877480, EPI_ISL_877481, EPI_ISL_877482, EPI_ISL_877483, EPI_ISL_877484, EPI_ISL_877485, EPI_ISL_877486, EPI_ISL_877487, EPI_ISL_877488, EPI_ISL_877489, EPI_ISL_877490, EPI_ISL_877491, EPI_ISL_877492, EPI_ISL_877493, EPI_ISL_877494, EPI_ISL_877495, EPI_ISL_877496, EPI_ISL_877497, EPI_ISL_877498, EPI_ISL_877500, EPI_ISL_877501, EPI_ISL_877502, EPI_ISL_877503, EPI_ISL_877504, EPI_ISL_877505, EPI_ISL_877506, EPI_ISL_877507, EPI_ISL_877508, EPI_ISL_877509, EPI_ISL_877510, EPI_ISL_877511, EPI_ISL_877512, EPI_ISL_877513, EPI_ISL_877514, EPI_ISL_877515, EPI_ISL_877516, EPI_ISL_877517, EPI_ISL_877518, EPI_ISL_877519, EPI_ISL_877520, EPI_ISL_877521, EPI_ISL_877522, EPI_ISL_877523, EPI_ISL_877524, EPI_ISL_877526, EPI_ISL_877527, EPI_ISL_877528, EPI_ISL_877529, EPI_ISL_877530, EPI_ISL_877531, EPI_ISL_877533, EPI_ISL_877534, EPI_ISL_877535, EPI_ISL_877536, EPI_ISL_877537, EPI_ISL_877539, EPI_ISL_877540, EPI_ISL_877541, EPI_ISL_877542, EPI_ISL_877543, EPI_ISL_877544, EPI_ISL_877545, EPI_ISL_877546, EPI_ISL_877547, EPI_ISL_877548, EPI_ISL_877549, EPI_ISL_877550			
see above	Eurofins Diatherix	Hudsonalpha Genome Sequencing Center	Jane Grimwood, Melissa Williams, Lori H. Handley, Joshua Stough, Leslie Malone, Stefan Brzezinski, Ada Stewart, Teresa Jones, Jenell Webber, John Lovell, Jennifer Cart, and Jeremy Schmutz
EPI_ISL_878279, EPI_ISL_878284, EPI_ISL_878287, EPI_ISL_878290, EPI_ISL_878292, EPI_ISL_878300, EPI_ISL_878303, EPI_ISL_878305, EPI_ISL_878311, EPI_ISL_878314, EPI_ISL_878316, EPI_ISL_878319, EPI_ISL_878325, EPI_ISL_878327, EPI_ISL_878330, EPI_ISL_878333, EPI_ISL_878341, EPI_ISL_878342, EPI_ISL_878345, EPI_ISL_878346, EPI_ISL_878348, EPI_ISL_878349, EPI_ISL_878351, EPI_ISL_878352, EPI_ISL_878353, EPI_ISL_878354, EPI_ISL_878355, EPI_ISL_878356, EPI_ISL_878357, EPI_ISL_878358, EPI_ISL_878359, EPI_ISL_878360, EPI_ISL_878361, EPI_ISL_878362, EPI_ISL_878363, EPI_ISL_878364, EPI_ISL_878365, EPI_ISL_878366, EPI_ISL_878367, EPI_ISL_878368, EPI_ISL_878369, EPI_ISL_878370, EPI_ISL_878372, EPI_ISL_878373, EPI_ISL_878374, EPI_ISL_878375, EPI_ISL_878376, EPI_ISL_878377, EPI_ISL_878378, EPI_ISL_878379, EPI_ISL_878381, EPI_ISL_878382, EPI_ISL_878385, EPI_ISL_878386, EPI_ISL_878387, EPI_ISL_878388, EPI_ISL_878390, EPI_ISL_878391, EPI_ISL_878392, EPI_ISL_878393, EPI_ISL_878394, EPI_ISL_878395, EPI_ISL_878396, EPI_ISL_878398, EPI_ISL_878399, EPI_ISL_878400, EPI_ISL_878406, EPI_ISL_878408, EPI_ISL_878411, EPI_ISL_878413, EPI_ISL_878418, EPI_ISL_878421, EPI_ISL_878426, EPI_ISL_878429			
see above	San Diego County Public Health Laboratory	Andersen lab at Scripps Research	SEARCH Alliance San Diego with Tracy Basler, Jovan Shephard, Brett Austin
EPI_ISL_878648, EPI_ISL_878661, EPI_ISL_878666, EPI_ISL_878682, EPI_ISL_878684, EPI_ISL_878705, EPI_ISL_878713, EPI_ISL_878716, EPI_ISL_878719, EPI_ISL_878726, EPI_ISL_878729			
see above	Rady's Childrens Hospital	Andersen lab at Scripps Research	SEARCH Alliance San Diego with Nanda Radamchar, David Dimmock, Linda Luo, Christina Clarke, Kathryn Bouic, Teresa Mueller, Denise Malicki
EPI_ISL_879924, EPI_ISL_879926, EPI_ISL_879932, EPI_ISL_879934, EPI_ISL_879937, EPI_ISL_879942, EPI_ISL_879947, EPI_ISL_879950, EPI_ISL_879953, EPI_ISL_879956, EPI_ISL_879959, EPI_ISL_879964, EPI_ISL_879969, EPI_ISL_879972, EPI_ISL_879975, EPI_ISL_879977, EPI_ISL_879980, EPI_ISL_879982, EPI_ISL_879985, EPI_ISL_879987, EPI_ISL_879990, EPI_ISL_879993, EPI_ISL_879995, EPI_ISL_879998, EPI_ISL_880000, EPI_ISL_880003, EPI_ISL_880009, EPI_ISL_880011, EPI_ISL_880014, EPI_ISL_880019, EPI_ISL_880027, EPI_ISL_880029, EPI_ISL_880032, EPI_ISL_880034			
see above	San Diego County Public Health Laboratory	Andersen lab at Scripps Research	SEARCH Alliance San Diego with Tracy Basler, Jovan Shephard, Brett Austin
EPI_ISL_882697, EPI_ISL_882699, EPI_ISL_882704, EPI_ISL_882705, EPI_ISL_882706, EPI_ISL_882707, EPI_ISL_882708, EPI_ISL_882764	1.AO Universitaria 'S. Giovanni di Dio e Ruggi D'Aragona, Scuola Medica Salernitana' Hospital / 2.UOC di Virologia e Microbiologia, Università della Campania 'L. Vanvitelli' / 3.AO Universitaria 'Federico II' Napoli Hospital / 4.AORN 'San Giuseppe Moscati' Avellino Hospital / 5.AO 'San Pio - presidio G. Rummo' Benevento Hospital / 6.AO 'Sant'Anna e San Sebastiano' Caserta Hospital / 7.PO 'Maria Santissima Addolorata' Eboli Hospital / 8.Biogem Istituto di Ricerche Genetiche	1. Genome Research Center for Health (CRGS) / 2. Laboratory of Molecular Medicine and Genomics(LMMGe) / 3. Center for Research in Pure and Applied Mathematics (CRMPA)	Giorgio Giurato, Francesca Rizzo, Alessandro Weisz, Gianluigi Franci, Giovanni Nassa, Pasquale Pagliano, Roberta Tarallo, Elena Alexandrova, Ylenia D'Agostino, Carlo Ferravante, Jessica Lamberti, Viola Melone, Domenico Memoli, Valeria Mirici Cappa, Domenico Palumbo, Giovanni Pecoraro, Assunta Sellitto, Oriana Strianese, Ilaria Terenzi, Giuseppe Fenza, Aniello Gentile, Antonello Saccomanno, Sonia Amabile, Teresa Rocco, Annamaria Salvati, Emilia Vaccaro, Massimiliano Galdiero, Michele Cennamo, Giuseppe Portella, Maria Grazia Foti, Mariarosaria Ingino, Maria Landi, Maurizio Fumi, Vincenzo Rocco, Rita Greco, Vittoria Letizia, Arnolfo Petruzziello, Maddalena Schioppa, Gregorio Goffredi, Francesca Marciano, Michele Caraglia, Alessia Cossu, Marianna Scrima
EPI_ISL_882957	Specjalistyczny Szpital im. Dra A. Sokoowskiego	National Institute of Public Health - National Institute of Hygiene	Wokowicz Tomasz, Zacharczuk Katarzyna, Gawor Jan
EPI_ISL_883067, EPI_ISL_883068, EPI_ISL_883069, EPI_ISL_883070, EPI_ISL_883071, EPI_ISL_883072, EPI_ISL_883073, EPI_ISL_883074, EPI_ISL_883075, EPI_ISL_883076, EPI_ISL_883077, EPI_ISL_883078, EPI_ISL_883079, EPI_ISL_883080, EPI_ISL_883081, EPI_ISL_883082, EPI_ISL_883083, EPI_ISL_883084, EPI_ISL_883085, EPI_ISL_883086, EPI_ISL_883088, EPI_ISL_883089, EPI_ISL_883090, EPI_ISL_883091, EPI_ISL_883092, EPI_ISL_883093, EPI_ISL_883094, EPI_ISL_883095, EPI_ISL_883096, EPI_ISL_883097, EPI_ISL_883098, EPI_ISL_883099, EPI_ISL_883100, EPI_ISL_883101, EPI_ISL_883102, EPI_ISL_883103, EPI_ISL_883104, EPI_ISL_883105, EPI_ISL_883106, EPI_ISL_883107, EPI_ISL_883108, EPI_ISL_883109, EPI_ISL_883110, EPI_ISL_883111, EPI_ISL_883112, EPI_ISL_883113, EPI_ISL_883114, EPI_ISL_883115, EPI_ISL_883116, EPI_ISL_883117, EPI_ISL_883118, EPI_ISL_883119, EPI_ISL_883120, EPI_ISL_883121, EPI_ISL_883122, EPI_ISL_883123, EPI_ISL_883124, EPI_ISL_883125, EPI_ISL_883126, EPI_ISL_883127, EPI_ISL_883128, EPI_ISL_883129, EPI_ISL_883130, EPI_ISL_883131, EPI_ISL_883132, EPI_ISL_883133, EPI_ISL_883134, EPI_ISL_883135, EPI_ISL_883136, EPI_ISL_883137, EPI_ISL_883138, EPI_ISL_883139, EPI_ISL_883140, EPI_ISL_883141, EPI_ISL_883142, EPI_ISL_883143, EPI_ISL_883144, EPI_ISL_883145, EPI_ISL_883146, EPI_ISL_883147, EPI_ISL_883148, EPI_ISL_883149, EPI_ISL_883150, EPI_ISL_883151, EPI_ISL_883152, EPI_ISL_883153, EPI_ISL_883154, EPI_ISL_883197, EPI_ISL_883199, EPI_ISL_883208, EPI_ISL_883209, EPI_ISL_883212, EPI_ISL_883213, EPI_ISL_883214, EPI_ISL_883215, EPI_ISL_883226, EPI_ISL_883229, EPI_ISL_883230, EPI_ISL_883231, EPI_ISL_883232, EPI_ISL_883233, EPI_ISL_883234, EPI_ISL_883248, EPI_ISL_883249, EPI_ISL_883251, EPI_ISL_883252, EPI_ISL_883253, EPI_ISL_883254, EPI_ISL_883255, EPI_ISL_883256, EPI_ISL_883268, EPI_ISL_883269, EPI_ISL_883271, EPI_ISL_883272, EPI_ISL_883282			
see above	Eurofins Diatherix	Hudsonalpha Genome Sequencing Center	Jane Grimwood, Melissa Williams, Lori H. Handley, Joshua Stough, Leslie Malone, Stefan Brzezinski, Ada Stewart, Teresa Jones, Jenell Webber, John Lovell, Jennifer Cart, and Jeremy Schmutz
EPI_ISL_887589	SC Dept of Health and Env. Control-Bureau of Laboratories	Centers for Disease Control and Prevention Division of Viral Diseases, Pathogen Discovery	Yan Li, Anna Montmayeur, Ying Tao, Jing Zhang, Krista Queen, Anna Uehara, Rachel Marine, Peter Cook, Clinton R. Paden, Haibin Wang, Suxiang Tong
EPI_ISL_888757, EPI_ISL_888758	KU Leuven, Rega Institute, Clinical and Epidemiological Virology	KU Leuven, Rega Institute, Clinical and Epidemiological Virology	Tony Wawina-Bokalanga, Bert Vanmechelen, Joan Marti-Carerras, Piet Maes
EPI_ISL_888988	RS Qadr	Eijkman Institute for Molecular Biology, Ministry of Research and Technology/National Agency for Research and Innovation	Frilasita A Yudhaputri, Hidayat Trimarsanto, Iskandar Adnan, Lydia V. Panggalo, Sukma Oktavianthi, Willy Agustine, Edison Johar, Safarina G Malik, Khin Saw Myint, Amin Soebandrio
EPI_ISL_888989, EPI_ISL_888990	RS Pondok Indah Bintaro Jaya	Eijkman Institute for Molecular Biology, Ministry of Research and Technology/National Agency for Research and Innovation	Frilasita A Yudhaputri, Hidayat Trimarsanto, Iskandar Adnan, Lydia V. Panggalo, Sukma Oktavianthi, Willy Agustine, Edison Johar, Safarina G Malik, Khin Saw Myint, Amin Soebandrio
EPI_ISL_888991	RS Mitra Keluarga Gading Serpong	Eijkman Institute for Molecular Biology, Ministry of Research and Technology/National Agency for Research and Innovation	Frilasita A Yudhaputri, Hidayat Trimarsanto, Iskandar Adnan, Lydia V. Panggalo, Sukma Oktavianthi, Willy Agustine, Edison Johar, Safarina G Malik, Khin Saw Myint, Amin Soebandrio
EPI_ISL_889010	RSU Medika Dramaga	Eijkman Institute for Molecular Biology, Ministry of Research and Technology/National Agency for Research and Innovation	Frilasita A Yudhaputri, Hidayat Trimarsanto, Iskandar Adnan, Lydia V. Panggalo, Sukma Oktavianthi, Willy Agustine, Edison Johar, Safarina G Malik, Khin Saw Myint, Amin Soebandrio
EPI_ISL_889011	RSU Permata Cibubur	Eijkman Institute for Molecular Biology, Ministry of Research and Technology/National Agency for Research and Innovation	Hidayat Trimarsanto, Iskandar Adnan, Lydia V. Panggalo, Sukma Oktavianthi, Willy Agustine, Edison Johar, Frilasita A Yudhaputri, Safarina G Malik, Khin Saw Myint, Amin Soebandrio

EPI_ISL_889443, EPI_ISL_889542, EPI_ISL_890175, EPI_ISL_890176	Ohio Department of Health Laboratory	Ohio Department of Health Laboratory	Holmes, Jennifer; Eric Brandt, Keoni Omura, Glen McGillivary, Caitlin McDonnell, Jade Mowery, Stephanie Mccracken, Tyler Payne, Kirtana Ramadugu, Erica Leasure, Brent Lee, Kelsey Florek, Heather Blankenship, Quanta Brown, and Tammy Bannerman
EPI_ISL_890301	KU Leuven, Rega Institute, Clinical and Epidemiological Virology	KU Leuven, Rega Institute, Clinical and Epidemiological Virology	Tony Wawina-Bokalanga, Bert Vanmechelen, Joan Marti-Carerras, Piet Maes
EPI_ISL_891221, EPI_ISL_891228	University of Medicine and Pharmacy of Craiova	"Stefan cel Mare" University Metagenomics Lab	Lobiuc Andrei, Gheorghita Roxana
EPI_ISL_891229	Suceava County Emergency Hospital	"Stefan cel Mare" University Metagenomics Lab	Lobiuc Andrei, Gheorghita Roxana
EPI_ISL_896107	Viollier AG	University Hospital Basel, Clinical Bacteriology	Tim Roloff, Madlen Stange, Helena MB Seth-Smith, Alfredo Mari, Karoline Leuzinger, Julia Bielicki, Christiane Beckmann, Manuel Battegay, Hans Hirsch, Adrian Egli
EPI_ISL_896186, EPI_ISL_896206	MEPHI, Aix Marseille University	MEPHI, Aix Marseille University	Anthony LEVASSEUR
EPI_ISL_899515	Viollier AG	Department of Biosystems Science and Engineering, ETH Zürich	Christian Beisel, Sarah Nadeau, Chaoran Chen, Ivan Topolsky, Philipp Jablonski, Lara Fuhrmann, David Dreifuss, Katharina Jahn, Tobias Schär, Ina Nissen, Natascha Santacroce, Elodie Burcklen, Christiane Beckmann, Maurice Redondo, Olivier Kobel, Christoph Noppen, Sophie Seidel, Noemie Santamaria de Souza, Niko Beerenwinkel, Tanja Stadler
EPI_ISL_902768, EPI_ISL_902769, EPI_ISL_902770, EPI_ISL_902771, EPI_ISL_902772, EPI_ISL_902773, EPI_ISL_902774, EPI_ISL_902775, EPI_ISL_902776, EPI_ISL_902777, EPI_ISL_902778, EPI_ISL_902779, EPI_ISL_902780, EPI_ISL_902781, EPI_ISL_902782, EPI_ISL_902783, EPI_ISL_902784, EPI_ISL_902785, EPI_ISL_902786, EPI_ISL_902787, EPI_ISL_902788, EPI_ISL_902789, EPI_ISL_902790, EPI_ISL_902791, EPI_ISL_902792, EPI_ISL_902793, EPI_ISL_902794, EPI_ISL_902795, EPI_ISL_902796, EPI_ISL_902797, EPI_ISL_902798, EPI_ISL_902799, EPI_ISL_902800, EPI_ISL_902801, EPI_ISL_902802, EPI_ISL_902803, EPI_ISL_902804, EPI_ISL_902805, EPI_ISL_902806, EPI_ISL_902807, EPI_ISL_902808, EPI_ISL_902809, EPI_ISL_902810, EPI_ISL_902811, EPI_ISL_902812, EPI_ISL_902813, EPI_ISL_902814, EPI_ISL_902815, EPI_ISL_902816, EPI_ISL_902817, EPI_ISL_902818, EPI_ISL_902819, EPI_ISL_902820, EPI_ISL_902821, EPI_ISL_902822, EPI_ISL_902823, EPI_ISL_902824, EPI_ISL_902825, EPI_ISL_902826, EPI_ISL_902827, EPI_ISL_902828, EPI_ISL_902829, EPI_ISL_902830, EPI_ISL_902831, EPI_ISL_902832, EPI_ISL_902833, EPI_ISL_902835, EPI_ISL_902836, EPI_ISL_902837, EPI_ISL_902838, EPI_ISL_902839, EPI_ISL_902840, EPI_ISL_902841, EPI_ISL_902842, EPI_ISL_902843, EPI_ISL_902844, EPI_ISL_902845, EPI_ISL_902846, EPI_ISL_902847, EPI_ISL_902848, EPI_ISL_902849, EPI_ISL_902850, EPI_ISL_902851, EPI_ISL_902852, EPI_ISL_902853, EPI_ISL_902854, EPI_ISL_902855			
see above	Department of Virology and Immunology, University of Helsinki and Helsinki University Hospital, Huslab Finland	Department of Virology, Faculty of Medicine, University of Helsinki, Helsinki, Finland	Teemu Smura, Ravi Kant, Phuoc Truong, Hussein Alburkat, Hannimari Kallio-Kokko, Jenni Virtanen, Maija Suvanto, Essi Korhonen, Sari Hannula, Harri Kangas, Hanna Liimatainen, Satu Kurkela, Hanna Jarva, Maija Lappalainen, Pekka Ellonen, Olli Vapalahti
EPI_ISL_902934, EPI_ISL_902949	Maryland Public Health Laboratory	Maryland Public Health Laboratory	Maryland Department of Health Laboratories Administration
EPI_ISL_903003	Seattle Flu Study	Seattle Flu Study	Deborah A. Nickerson, Chris D. Frazar, Jover Lee, Benjamin Pelle, Erica Ryke, Matthew Richardson, Amanda Adler, Elisabeth Brandstetter, Peter D. Han, Kairsten Fay, Misja Ilcisin, Kirsten Lacombe, Thomas R. Sibley, Melissa Truong, Caitlin R. Wolf, Michael Boeckh, Janet A. Englund, Michael Famulare, Barry R. Lutz, Mark J. Rieder, Lea M. Starita, Matthew Thompson, Jay Shendure, Trevor Bedford, Helen Y. Chu
EPI_ISL_903650, EPI_ISL_903718	VT Dept. of Health Laboratory	Genomics and Discovery, Respiratory Viruses Branch, Division of Viral Diseases, Centers for Disease Control and Prevention	Krista Queen, Yan Li, Ying Tao, Jing Zhang, Anna Uehara, Anna Montmayeur, Clinton R. Paden, Peter W. Cook, Rachel Marine, Mili Sheth, Jasmine Padilla, Sarah Nobles, Mark Burroughs, Lori Rowe, Haibin Wang, Ben L. Rambo-Martin, Dhwani Batra, Justin Lee, Suxiang Tong
EPI_ISL_903820	PA Department of Health, Bureau of Laboratories	Genomics and Discovery, Respiratory Viruses Branch, Division of Viral Diseases, Centers for Disease Control and Prevention	Krista Queen, Yan Li, Ying Tao, Jing Zhang, Anna Uehara, Anna Montmayeur, Clinton R. Paden, Peter W. Cook, Rachel Marine, Mili Sheth, Jasmine Padilla, Sarah Nobles, Mark Burroughs, Lori Rowe, Haibin Wang, Ben L. Rambo-Martin, Dhwani Batra, Justin Lee, Suxiang Tong
EPI_ISL_904005, EPI_ISL_904006	Scientific Veterinary Institute Novi Sad	Veterinary Specialized Institute "Kraljevo", Serbia	Vidanovic,D., Tesovic,B., Knezevic,A., Jovanovic,T., Jankovic,M., Sekler,M., Banovic Djeri,B., Petrovic,T., Volkening,J., Afonso,C.
EPI_ISL_904017	NCSLPH	NCSLPH	Kitty Chase, Michael C Miller, Shermalyn Greene, William Glover
EPI_ISL_904469, EPI_ISL_904471, EPI_ISL_904472, EPI_ISL_904474	Dutch COVID-19 response team	Erasmus Medical Center	Bas Oude Munnink, Reina Sikkema, David Nieuwenhuijse, Irina Chestakova, Anne van der Linden, Marjan Boter, Emmanuelle Munger, Corine GeurtsvanKessel, Annemiek van der Eijk, Richard Molenkamp, Marion Koopmans, on behalf of the Dutch national COVID-19 response team.
EPI_ISL_904735, EPI_ISL_904736, EPI_ISL_904737, EPI_ISL_904738, EPI_ISL_904797, EPI_ISL_904798	Dutch COVID-19 response team	National Institute for Public Health and the Environment (RIVM)	Adam Meijer, Harry Vennema, Dirk Eggink, Jeroen Cremer, Sharon van den Brink, Bas van der Veer, AnneMarie van den Brandt, Florian Zwagemaker, Dennis Schmitz, Chantal Reusken, on behalf of the national COVID-19 response team
EPI_ISL_904935, EPI_ISL_904940, EPI_ISL_904949, EPI_ISL_904950, EPI_ISL_904951, EPI_ISL_904952, EPI_ISL_904953, EPI_ISL_904955	Vilnius University Hospital Santaros Klinikos, Vilnius University	Institute of Biotechnology, Life Sciences Center, Vilnius University	Emilija Vasiliunaite, Milda Norkiene, Albertas Timinskas, Alma Gedvilaite, Aurelija Zvirbliene, Daniel Naumovas, Laimonas Griskevicius
EPI_ISL_905080	Dutch COVID-19 response team	National Institute for Public Health and the Environment (RIVM)	Adam Meijer, Harry Vennema, Dirk Eggink, Jeroen Cremer, Sharon van den Brink, Bas van der Veer, AnneMarie van den Brandt, Florian Zwagemaker, Dennis Schmitz, Chantal Reusken, on behalf of the national COVID-19 response team
EPI_ISL_906263	University of Wisconsin-Madison AIDS Vaccine Research Laboratories	University of Wisconsin-Madison AIDS Vaccine Research Laboratories	Gage Moreno, Katarina Braun, et al. AIDS Vaccine Research Laboratories
EPI_ISL_907078, EPI_ISL_907079, EPI_ISL_907080, EPI_ISL_907081, EPI_ISL_907082, EPI_ISL_907083	Biology, MCL	Biology, MCL	Seadawy,M.G., Shamel,M.D., Gad,A.F., Elhoseiny,M.F., Zekri,A.N.
EPI_ISL_910386, EPI_ISL_910387, EPI_ISL_910388, EPI_ISL_910389, EPI_ISL_910458, EPI_ISL_910459, EPI_ISL_910460, EPI_ISL_910461, EPI_ISL_910462, EPI_ISL_910647, EPI_ISL_910700, EPI_ISL_910701, EPI_ISL_910703, EPI_ISL_910705, EPI_ISL_910714, EPI_ISL_910715, EPI_ISL_910716, EPI_ISL_910717, EPI_ISL_910720, EPI_ISL_910775, EPI_ISL_910776, EPI_ISL_911240			
see above	Laboratoire national de sante, Microbiology, Virology	Laboratoire national de sante, Microbiology, Microbial Genomics Platform	Anke Wienecke-Baldacchino, Catherine Ragimbeau,Jessica Tapp, Fatu Djabi, Lise Pignon, Raoul Salmon, Tamir Abdelrahman
EPI_ISL_911677, EPI_ISL_911679, EPI_ISL_911680, EPI_ISL_911683, EPI_ISL_911687, EPI_ISL_911697	Alaska State Virology Laboratory	Alaska State Virology Laboratory	Stephanie DeRonde, Lisa Smith, Ph.D., Jack Chen, Ph.D.
EPI_ISL_911708	Tanjungpura University Hospital	Tanjungpura University Hospital	Puji Astuti; Andriani; Mahyarudin; Delima Fajar Liana; Virhan Novianry ; Sofi Siti Shofiyah
EPI_ISL_911750	Tanjungpura University Hospital	Tanjungpura University Hospital	Virhan Novianry; Delima Fajar Liana; Mahyarudin; Andriani; Sofi Siti Shofiyah; Puji Astuti; Muhammad Kahtan; Ambar Rialita; Eka Ardiani Putri; Wiwik Windarti; Helmi Sastriawan, Rini Andriani
EPI_ISL_913157, EPI_ISL_913160, EPI_ISL_913201, EPI_ISL_913202, EPI_ISL_913203, EPI_ISL_913204, EPI_ISL_913210, EPI_ISL_913211, EPI_ISL_913212, EPI_ISL_913213, EPI_ISL_913214, EPI_ISL_913215, EPI_ISL_913217, EPI_ISL_913218, EPI_ISL_913219, EPI_ISL_913220, EPI_ISL_913221, EPI_ISL_913222, EPI_ISL_913223, EPI_ISL_913224, EPI_ISL_913226, EPI_ISL_913227, EPI_ISL_913228, EPI_ISL_913229, EPI_ISL_913230, EPI_ISL_913231, EPI_ISL_913236, EPI_ISL_913237, EPI_ISL_913239, EPI_ISL_913241, EPI_ISL_913243, EPI_ISL_913247, EPI_ISL_913249, EPI_ISL_913250, EPI_ISL_913252, EPI_ISL_913254, EPI_ISL_913255			
see above	University of Michigan Clinical Microbiology Laboratory	Lauring Lab, University of Michigan, Department of Microbiology and Immunology	Valesano
EPI_ISL_913278, EPI_ISL_913279, EPI_ISL_913285, EPI_ISL_913313	Klinisk mikrobiologi	The Public Health Agency of Sweden	Anna-Malin Linde, Maria Lind Karlberg, Carlo Berg, Oskar Karlsson Lindsjo, Sofia Stamouli, Reza Advani, Mattias Haukland, Petra Holmstrom, Noura Walai, Petra Edquist, Mia Brytting, Anna Risberg, Karin Tegmark-Wisell
EPI_ISL_913343	Synlab Medilab, Mikrobiologi	The Public Health Agency of Sweden	Anna-Malin Linde, Maria Lind Karlberg, Carlo Berg, Oskar Karlsson Lindsjo, Sofia Stamouli, Reza Advani, Mattias Haukland, Petra Holmstrom, Noura Walai, Petra Edquist, Mia Brytting, Anna Risberg, Karin Tegmark-Wisell
EPI_ISL_913344, EPI_ISL_913345, EPI_ISL_913346, EPI_ISL_913347, EPI_ISL_913354, EPI_ISL_913355, EPI_ISL_913356, EPI_ISL_913461, EPI_ISL_913480	Klinisk mikrobiologi	The Public Health Agency of Sweden	Anna-Malin Linde, Maria Lind Karlberg, Carlo Berg, Oskar Karlsson Lindsjo, Sofia Stamouli, Reza Advani, Mattias Haukland, Petra Holmstrom, Noura Walai, Petra Edquist, Mia Brytting, Anna Risberg, Karin Tegmark-Wisell
EPI_ISL_913933, EPI_ISL_913934,	Instituto de Diagnostico y Referencia Epidemiologicos	Instituto de Diagnostico y Referencia Epidemiologicos	Claudia Wong-Arambula, Abril Rodriguez-Maldonado, Fabiola Garces-Ayala, Adnan Araiza-Rodriguez, David Fragoso-Fonseca, Sergio Rangel-Guerrero,

EPI_ISL_913935, EPI_ISL_913936, EPI_ISL_913937, EPI_ISL_913938, EPI_ISL_913939	INDRE_RNLSP	(INDRE)	Mayra Jimenez-Morales, Nancy Munoz-Hernandez, Natividad Cruz-Ortiz, Tatiana Nunez-Garcia, Gisela Barrera-Badillo, Lucia Hernandez-Rivas, Irma Lopez-Martinez, Ernesto Ramirez-Gonzalez.
EPI_ISL_915366, EPI_ISL_915367, EPI_ISL_915370, EPI_ISL_915371, EPI_ISL_915372, EPI_ISL_915373, EPI_ISL_915374, EPI_ISL_915375, EPI_ISL_915377, EPI_ISL_915378, EPI_ISL_915412, EPI_ISL_915414			
see above	Keio University School of Medicine	Keio University School of Medicine	Kenjiro Kosaki, Yuka Iwasaki, Hirotsugu Ishizu, Haruhiko Siomi, Kodai Abe
EPI_ISL_915440, EPI_ISL_915441, EPI_ISL_915442, EPI_ISL_915443, EPI_ISL_915445, EPI_ISL_915446, EPI_ISL_915447, EPI_ISL_915452, EPI_ISL_915455, EPI_ISL_915459, EPI_ISL_915462, EPI_ISL_915463, EPI_ISL_915464, EPI_ISL_915466, EPI_ISL_915468, EPI_ISL_915469, EPI_ISL_915471, EPI_ISL_915472, EPI_ISL_915473, EPI_ISL_915474, EPI_ISL_915477, EPI_ISL_915479, EPI_ISL_915482, EPI_ISL_915483, EPI_ISL_915484, EPI_ISL_915485, EPI_ISL_915486, EPI_ISL_915487, EPI_ISL_915489, EPI_ISL_915490, EPI_ISL_915491, EPI_ISL_915492, EPI_ISL_915493, EPI_ISL_915494, EPI_ISL_915498, EPI_ISL_915499, EPI_ISL_915500, EPI_ISL_915501, EPI_ISL_915504, EPI_ISL_915506, EPI_ISL_915509, EPI_ISL_915511, EPI_ISL_915513, EPI_ISL_915514, EPI_ISL_915516, EPI_ISL_915518, EPI_ISL_915519, EPI_ISL_915521, EPI_ISL_915522, EPI_ISL_915523, EPI_ISL_915528, EPI_ISL_915529, EPI_ISL_915530, EPI_ISL_915532, EPI_ISL_915533, EPI_ISL_915534, EPI_ISL_915535, EPI_ISL_915537, EPI_ISL_915538, EPI_ISL_915539, EPI_ISL_915540, EPI_ISL_915541, EPI_ISL_915543, EPI_ISL_915544, EPI_ISL_915545, EPI_ISL_915547, EPI_ISL_915548, EPI_ISL_915552, EPI_ISL_915553, EPI_ISL_915554, EPI_ISL_915561, EPI_ISL_915562, EPI_ISL_915563, EPI_ISL_915568, EPI_ISL_915569, EPI_ISL_915570, EPI_ISL_915572, EPI_ISL_915573, EPI_ISL_915575, EPI_ISL_915576, EPI_ISL_915577, EPI_ISL_915578, EPI_ISL_915579, EPI_ISL_915580, EPI_ISL_915581, EPI_ISL_915582, EPI_ISL_915583, EPI_ISL_915584, EPI_ISL_915588, EPI_ISL_915593, EPI_ISL_915594, EPI_ISL_915596, EPI_ISL_915597, EPI_ISL_915598, EPI_ISL_915599, EPI_ISL_915600, EPI_ISL_915601, EPI_ISL_915603, EPI_ISL_915604, EPI_ISL_915605, EPI_ISL_915606, EPI_ISL_915608, EPI_ISL_915610, EPI_ISL_915612, EPI_ISL_915613, EPI_ISL_915615, EPI_ISL_915618, EPI_ISL_915620, EPI_ISL_915621, EPI_ISL_915622, EPI_ISL_915624, EPI_ISL_915625, EPI_ISL_915626, EPI_ISL_915627, EPI_ISL_915628, EPI_ISL_915629, EPI_ISL_915630, EPI_ISL_915631, EPI_ISL_915632, EPI_ISL_915634, EPI_ISL_915635, EPI_ISL_915636, EPI_ISL_915637, EPI_ISL_915638, EPI_ISL_915640, EPI_ISL_915642, EPI_ISL_915643, EPI_ISL_915644, EPI_ISL_915645, EPI_ISL_915646, EPI_ISL_915647, EPI_ISL_915648, EPI_ISL_915649, EPI_ISL_915650, EPI_ISL_915652, EPI_ISL_915653, EPI_ISL_915654, EPI_ISL_915655, EPI_ISL_915657, EPI_ISL_915658, EPI_ISL_915659, EPI_ISL_915665, EPI_ISL_915667, EPI_ISL_915668, EPI_ISL_915669, EPI_ISL_915670, EPI_ISL_915672, EPI_ISL_915673, EPI_ISL_915674, EPI_ISL_915675, EPI_ISL_915676, EPI_ISL_915677, EPI_ISL_915678, EPI_ISL_915679, EPI_ISL_915680, EPI_ISL_915681, EPI_ISL_915682, EPI_ISL_915684, EPI_ISL_915686, EPI_ISL_915687, EPI_ISL_915688, EPI_ISL_915691, EPI_ISL_915692, EPI_ISL_915694, EPI_ISL_915695, EPI_ISL_915696, EPI_ISL_915699, EPI_ISL_915701, EPI_ISL_915703, EPI_ISL_915704, EPI_ISL_915707, EPI_ISL_915708, EPI_ISL_915709, EPI_ISL_915710, EPI_ISL_915712, EPI_ISL_915714, EPI_ISL_915715, EPI_ISL_915716, EPI_ISL_915717, EPI_ISL_915718, EPI_ISL_915719, EPI_ISL_915720, EPI_ISL_915721, EPI_ISL_915722, EPI_ISL_915723, EPI_ISL_915724, EPI_ISL_915725, EPI_ISL_915728, EPI_ISL_915731, EPI_ISL_915732, EPI_ISL_915733, EPI_ISL_915734, EPI_ISL_915735, EPI_ISL_915736, EPI_ISL_915737, EPI_ISL_915738, EPI_ISL_915739, EPI_ISL_915741, EPI_ISL_915742, EPI_ISL_915743, EPI_ISL_915746, EPI_ISL_915747, EPI_ISL_915749, EPI_ISL_915751, EPI_ISL_915752, EPI_ISL_915753, EPI_ISL_915754, EPI_ISL_915755, EPI_ISL_915756, EPI_ISL_915757, EPI_ISL_915758, EPI_ISL_915759, EPI_ISL_915760, EPI_ISL_915761, EPI_ISL_915762, EPI_ISL_915763, EPI_ISL_915764, EPI_ISL_915765, EPI_ISL_915766, EPI_ISL_915767, EPI_ISL_915768, EPI_ISL_915769, EPI_ISL_915770, EPI_ISL_915772, EPI_ISL_915773, EPI_ISL_915774, EPI_ISL_915775, EPI_ISL_915777, EPI_ISL_915779, EPI_ISL_915780, EPI_ISL_915782, EPI_ISL_915783, EPI_ISL_915784, EPI_ISL_915785, EPI_ISL_915786, EPI_ISL_915788, EPI_ISL_915789, EPI_ISL_915793, EPI_ISL_915794, EPI_ISL_915797, EPI_ISL_915799, EPI_ISL_915800, EPI_ISL_915802, EPI_ISL_915804, EPI_ISL_915805, EPI_ISL_915806, EPI_ISL_915808, EPI_ISL_915810, EPI_ISL_915811, EPI_ISL_915813, EPI_ISL_915814, EPI_ISL_915816, EPI_ISL_915817, EPI_ISL_915818, EPI_ISL_915820, EPI_ISL_915822, EPI_ISL_915823, EPI_ISL_915824, EPI_ISL_915829, EPI_ISL_915830, EPI_ISL_915833, EPI_ISL_915835, EPI_ISL_915836, EPI_ISL_915838, EPI_ISL_915841, EPI_ISL_915843, EPI_ISL_915845, EPI_ISL_915847, EPI_ISL_915848, EPI_ISL_915849, EPI_ISL_915852, EPI_ISL_915853, EPI_ISL_915854, EPI_ISL_915855, EPI_ISL_915856, EPI_ISL_915857, EPI_ISL_915859, EPI_ISL_915860, EPI_ISL_915861, EPI_ISL_915862, EPI_ISL_915863, EPI_ISL_915864, EPI_ISL_915865, EPI_ISL_915867, EPI_ISL_915868, EPI_ISL_915870, EPI_ISL_915872, EPI_ISL_915873, EPI_ISL_915874, EPI_ISL_915875, EPI_ISL_915877, EPI_ISL_915881, EPI_ISL_915883, EPI_ISL_915886, EPI_ISL_915887, EPI_ISL_915889, EPI_ISL_915890, EPI_ISL_915892, EPI_ISL_915893, EPI_ISL_915894, EPI_ISL_915896, EPI_ISL_915899, EPI_ISL_915901, EPI_ISL_915902, EPI_ISL_915903, EPI_ISL_915904, EPI_ISL_915905, EPI_ISL_915906, EPI_ISL_915907, EPI_ISL_915908, EPI_ISL_915909, EPI_ISL_915910, EPI_ISL_915913, EPI_ISL_915914, EPI_ISL_915916, EPI_ISL_915917, EPI_ISL_915918, EPI_ISL_915922, EPI_ISL_915924, EPI_ISL_915925, EPI_ISL_915926, EPI_ISL_915927, EPI_ISL_915928, EPI_ISL_915929, EPI_ISL_915930, EPI_ISL_915931, EPI_ISL_915932, EPI_ISL_915933, EPI_ISL_915934, EPI_ISL_915935, EPI_ISL_915937, EPI_ISL_915941, EPI_ISL_915942, EPI_ISL_915943, EPI_ISL_915945, EPI_ISL_915947, EPI_ISL_915949, EPI_ISL_915952, EPI_ISL_915953, EPI_ISL_915955, EPI_ISL_915958, EPI_ISL_915960, EPI_ISL_915961, EPI_ISL_915962, EPI_ISL_915963, EPI_ISL_915965, EPI_ISL_915966, EPI_ISL_915967, EPI_ISL_915968, EPI_ISL_915969, EPI_ISL_915970, EPI_ISL_915971, EPI_ISL_915973, EPI_ISL_915974, EPI_ISL_915975, EPI_ISL_915978, EPI_ISL_915980, EPI_ISL_915981, EPI_ISL_915984, EPI_ISL_915985, EPI_ISL_915986, EPI_ISL_915987, EPI_ISL_915988, EPI_ISL_915989, EPI_ISL_915992, EPI_ISL_915993, EPI_ISL_915994, EPI_ISL_915995, EPI_ISL_915997, EPI_ISL_915998, EPI_ISL_915999, EPI_ISL_916000, EPI_ISL_916002, EPI_ISL_916003, EPI_ISL_916005, EPI_ISL_916011, EPI_ISL_916012, EPI_ISL_916013, EPI_ISL_916014, EPI_ISL_916016, EPI_ISL_916017, EPI_ISL_916019, EPI_ISL_916020, EPI_ISL_916021, EPI_ISL_916023, EPI_ISL_916024, EPI_ISL_916025, EPI_ISL_916026, EPI_ISL_916027, EPI_ISL_916028, EPI_ISL_916029, EPI_ISL_916030, EPI_ISL_916031, EPI_ISL_916033, EPI_ISL_916036, EPI_ISL_916038, EPI_ISL_916040, EPI_ISL_916041, EPI_ISL_916043, EPI_ISL_916044, EPI_ISL_916045, EPI_ISL_916046, EPI_ISL_916050, EPI_ISL_916051, EPI_ISL_916053, EPI_ISL_916054, EPI_ISL_916055, EPI_ISL_916056, EPI_ISL_916059, EPI_ISL_916064, EPI_ISL_916066			
see above	Lighthouse Lab in Alderley Park	Wellcome Sanger Institute for the COVID-19 Genomics UK (COG-UK) Consortium	Jacquelyn Wynn, Mairead Hyland, The Lighthouse Lab in Alderley Park and Alex Alderton, Roberto Amato, Sonia Goncalves, Ewan Harrison, David K. Jackson, Ian Johnston, Dominic Kwiatkowski, Cordelia Langford, John Sillitoe on behalf of the Wellcome Sanger Institute COVID-19 Surveillance Team
EPI_ISL_918205, EPI_ISL_918207, EPI_ISL_918209, EPI_ISL_918211, EPI_ISL_918214, EPI_ISL_918216	Innovative Genomics Institute, UC Berkeley	Innovative Genomics Institute, UC Berkeley	Stacia Wyman, Haridha Shivram, Phil Frankino, Liana Lareau, Shana McDevitt, Justin Choi
EPI_ISL_918366, EPI_ISL_918367, EPI_ISL_918368, EPI_ISL_918369, EPI_ISL_918370, EPI_ISL_918371, EPI_ISL_918372	Virology Unit, Institut Pasteur du Cambodge	Virology Unit, Institut Pasteur du Cambodge	Sokhoun Yann, Ly Sovann, Kraing Sidonn, Yi Sengdoeurn, Chin Savuth, Chau Darapheak, Etienne Simon-Loriere, Veasna Duong, Erik A Karlsson
EPI_ISL_918515, EPI_ISL_918516, EPI_ISL_918523	LACEN - Laboratório Central de Saúde Pública do Para	Evandro Chagas Institute	Santos, M.C.; Silva, A.M.; Junior, W.D.C.; Barbagelata, L.S.; Ferreira, J.A.; Sousa, E.M.A.; da Silva, P.S.; Pinheiro, K.C.; L.C.; Sousa Junior, E.C.
EPI_ISL_918570, EPI_ISL_918577, EPI_ISL_918665, EPI_ISL_918667, EPI_ISL_918679, EPI_ISL_918681	University of Birmingham	COVID-19 Genomics UK (COG-UK) Consortium	Institute of Microbiology, University of Birmingham: Claire McMurray, Joanne Stockton, Samuel Nicholls, Radoslaw Poplawski, Will Rowe, Josh Quick, Nicholas Loman. University of Birmingham Testing Laboratory: Celina M Whalley, Andrew Bosworth, Charlotte Poxon, Kasun Wanigasooriya, Oliver Pickles, Mike Kidd, Alex Richter, Andrew D Beggs PHE Heartlands Lab: Husam Osman, Andrew Bosworth. Queen Elizabeth Hospital: Anna Casey
EPI_ISL_919239, EPI_ISL_919270, EPI_ISL_919273	West of Scotland Specialist Virology Centre, NHSGGC / MRC-University of Glasgow Centre for Virus Research	COVID-19 Genomics UK (COG-UK) Consortium	Ana da Silva Filipe, Natasha Johnson, Kathy Smollett, Daniel Mair, Stephen Carmichael, Alice Broos, Lily Tong, Jenna Nichols, Kyriaki Nomikou; Sarah McDonald; Richard Orton, Joseph Hughes, Sreenu Vattipally, David L Robertson; Alasdair MacLean, Rory Gunson; Sharif Shaaban, Matthew Holden; Rachel Blacow, Guy Mollett, Kathy Li, James Shepherd, Antonia Ho, Emma Thomson
EPI_ISL_919347, EPI_ISL_919348	Virology Department, Royal Infirmary of Edinburgh, NHS Lothian / School of Biological Sciences, University of Edinburgh / Institute of Genetics and Molecular Medicine, University of Edinburgh	COVID-19 Genomics UK (COG-UK) Consortium	McHugh M, Dewar R, Rooke S, Gallagher M, Balcaza C, O'Toole Á, Scher E, Hill V, McCrone JT, Colquhoun R, Yu X, Jackson B, Rambaut A, Williams TC, Templeton K
EPI_ISL_920278, EPI_ISL_920355, EPI_ISL_920376, EPI_ISL_920482, EPI_ISL_920491, EPI_ISL_920509	University College London Hospital	COVID-19 Genomics UK (COG-UK) Consortium	Judith Heaney, Matthew Byott, Catherine Houlihan, Dan Frampton, Stuart Kirk, Moira Spyer and Eleni Nastouli
EPI_ISL_924789, EPI_ISL_924790, EPI_ISL_924792	Bioinformatics and Biostatistics Lab, Advanced Sequencing Facility	COVID-19 Genomics UK (COG-UK) Consortium	Aengus Stewart,Jerome Nicod,Chelsea Sawyer,Laura Cubitt,Harshil Patel,Margaret Crawford
EPI_ISL_925191, EPI_ISL_925193, EPI_ISL_925203	Virginia DCLS	Virginia DCLS	Virginia DCLS
EPI_ISL_925361	NCSLPH	NCSLPH	Chase K, Miller MC, Greene S, Glover W
EPI_ISL_925377	Department of Clinical Microbiology	GIGA Medical Genomics	Keith Durkin, Maria Artesi, Sébastien Bontems, Raphaël Boreux, Bouchra Boujemla, Cécile Meex, Pierrette Melin, Marie-Pierre Hayette, Vincent Bours
EPI_ISL_925979, EPI_ISL_925992, EPI_ISL_926137, EPI_ISL_926166, EPI_ISL_926193, EPI_ISL_926252, EPI_ISL_926323, EPI_ISL_926334, EPI_ISL_926388, EPI_ISL_926432, EPI_ISL_926494, EPI_ISL_926504, EPI_ISL_926515, EPI_ISL_926776, EPI_ISL_926787, EPI_ISL_926811, EPI_ISL_926878, EPI_ISL_926995, EPI_ISL_927118, EPI_ISL_927223, EPI_ISL_927239, EPI_ISL_927252, EPI_ISL_927295, EPI_ISL_927325, EPI_ISL_927352, EPI_ISL_927431, EPI_ISL_927491, EPI_ISL_927502, EPI_ISL_927662, EPI_ISL_927808, EPI_ISL_927843, EPI_ISL_927872, EPI_ISL_928073, EPI_ISL_928081, EPI_ISL_928106, EPI_ISL_928118, EPI_ISL_928148, EPI_ISL_928208, EPI_ISL_928230, EPI_ISL_928240, EPI_ISL_928282, EPI_ISL_928285, EPI_ISL_928297, EPI_ISL_928316, EPI_ISL_928335, EPI_ISL_928380, EPI_ISL_928422, EPI_ISL_928513, EPI_ISL_928606, EPI_ISL_928630, EPI_ISL_928670, EPI_ISL_928687, EPI_ISL_928696, EPI_ISL_928718, EPI_ISL_928722, EPI_ISL_928884, EPI_ISL_928888, EPI_ISL_928939, EPI_ISL_928988, EPI_ISL_929006, EPI_ISL_929022, EPI_ISL_929027, EPI_ISL_929069, EPI_ISL_929213, EPI_ISL_929286, EPI_ISL_929299, EPI_ISL_929357, EPI_ISL_929467, EPI_ISL_929513, EPI_ISL_929657, EPI_ISL_929662, EPI_ISL_929678, EPI_ISL_929695, EPI_ISL_929738			
see above	Department of Virus and Microbiological Special Diagnostics, Statens Serum Institut, Copenhagen, Denmark	Aalborg University	Danish Covid-19 Genome Consortium
EPI_ISL_929763	NCSLPH	NCSLPH	Chase K, Miller MC, Greene S, Glover W
EPI_ISL_929781, EPI_ISL_929790, EPI_ISL_929797, EPI_ISL_929830, EPI_ISL_929900, EPI_ISL_929916, EPI_ISL_929937, EPI_ISL_929995, EPI_ISL_930065, EPI_ISL_930088, EPI_ISL_930111, EPI_ISL_930158, EPI_ISL_930178, EPI_ISL_930180, EPI_ISL_930187, EPI_ISL_930202, EPI_ISL_930212, EPI_ISL_930347, EPI_ISL_930376, EPI_ISL_930403, EPI_ISL_930431, EPI_ISL_930434, EPI_ISL_930497, EPI_ISL_930518, EPI_ISL_930523, EPI_ISL_930562			
see above	Department of Virus and Microbiological Special Diagnostics, Statens Serum Institut, Copenhagen, Denmark	Aalborg University	Danish Covid-19 Genome Consortium
EPI_ISL_931362, EPI_ISL_931364, EPI_ISL_931370	University Hospital Basel, Clinical Virology	University Hospital Basel, Clinical Bacteriology	Tim Roloff, Madlen Stange, Helena MB Seth-Smith, Alfredo Mari, Karoline Leuzinger, Julia Bielicki, Manuel Battegay, Hans Hirsch, Adrian Egli
EPI_ISL_933495, EPI_ISL_933504, EPI_ISL_933506	Lighthouse Lab in Milton Keynes	Wellcome Sanger Institute for the COVID-19 Genomics UK (COG-UK) Consortium	The Lighthouse Lab in Milton Keynes and Alex Alderton, Roberto Amato, Sonia Goncalves, Ewan Harrison, David K. Jackson, Ian Johnston, Dominic Kwiatkowski, Cordelia Langford, John Sillitoe on behalf of the Wellcome Sanger Institute COVID-19 Surveillance Team
EPI_ISL_933512, EPI_ISL_933513, EPI_ISL_933514, EPI_ISL_933515, EPI_ISL_933516, EPI_ISL_933517, EPI_ISL_933518, EPI_ISL_933519, EPI_ISL_933520, EPI_ISL_933521, EPI_ISL_933522, EPI_ISL_933523, EPI_ISL_933524, EPI_ISL_933526, EPI_ISL_933529, EPI_ISL_933530, EPI_ISL_933531			

see above	Instituto Nacional de Medicina Genomica	Instituto Nacional de Medicina Genomica	Hidalgo-Miranda A, Mendoza-Vargas A, Reyes-Grajeda JP, Cisneros-Villanueva M, Cedro-Tanda A,Peñaloza-Figueroa F, Herrera-Montalvo LA
EPI_ISL_933707	Instituto de Diagnostico y Referencia Epidemiologicos INDRE_RNLSP	Instituto de Diagnostico y Referencia Epidemiologicos (INDRE)	Claudia Wong-Arambula, Abril Rodriguez-Maldonado, Fabiola Garces-Ayala, Adnan Araiza-Rodriguez, David Fragoso-Fonseca, Sergio Rangel-Guerrero, Mayra Jimenez-Morales, Nancy Munoz-Hernandez, Natividad Cruz-Ortiz, Tatiana Nunez-Garcia, Gisela Barrera-Badillo, Lucia Hernandez-Rivas, Irma Lopez-Martinez, Ernesto Ramirez-Gonzalez.
EPI_ISL_934337	Klinisk mikrobiologi	The Public Health Agency of Sweden	Anna-Malin Linde, Maria Lind Karlberg, Carlo Berg, Oskar Karlsson Lindsjo, Sofia Stamouli, Reza Advani, Mattias Haukland, Petra Holmstrom, Noura Walai, Petra Edquist, Mia Brytting, Anna Risberg, Karin Tegmark-Wisell
EPI_ISL_934544	Institut für Virologie am Department für Hygiene, Mikrobiologie und Public Health	Bergthaler laboratory, CeMM Research Center for Molecular Medicine of the Austrian Academy of Sciences	Lukas Endler, Anna Schedl, Thomas Penz, Benedikt Agerer, Maelle Le Moing, Michael Schuster, Bekir Erguner, Jan Laine, Martin Senekowitsch, Christoph Bock, Andreas Bergthaler
EPI_ISL_935215	KU Leuven, Rega Institute, Clinical and Epidemiological Virology	KU Leuven, Rega Institute, Clinical and Epidemiological Virology	Tony Wawina-Bokalanga, Bert Vanmechelen, Joan Marti-Carerras, Piet Maes
EPI_ISL_935423, EPI_ISL_935424, EPI_ISL_935425, EPI_ISL_935430, EPI_ISL_935431, EPI_ISL_935433, EPI_ISL_935436, EPI_ISL_935438, EPI_ISL_935447, EPI_ISL_935450, EPI_ISL_935451, EPI_ISL_935454, EPI_ISL_935456, EPI_ISL_935457, EPI_ISL_935458, EPI_ISL_935460, EPI_ISL_935461, EPI_ISL_935462, EPI_ISL_935466, EPI_ISL_935467, EPI_ISL_935474			
see above	Florida Bureau of Public Health Laboratories	Florida Bureau of Public Health Laboratories	Sarah Schmedes, Jason Blanton
EPI_ISL_935878, EPI_ISL_935879, EPI_ISL_935880, EPI_ISL_935881, EPI_ISL_935882, EPI_ISL_935883, EPI_ISL_935884, EPI_ISL_935885, EPI_ISL_935886, EPI_ISL_935888, EPI_ISL_935889, EPI_ISL_935893, EPI_ISL_935894, EPI_ISL_935895, EPI_ISL_935898, EPI_ISL_935899, EPI_ISL_935900, EPI_ISL_935901, EPI_ISL_935902, EPI_ISL_935904, EPI_ISL_935907, EPI_ISL_935908, EPI_ISL_935910, EPI_ISL_935911, EPI_ISL_935912, EPI_ISL_935913, EPI_ISL_935915, EPI_ISL_935916, EPI_ISL_935917, EPI_ISL_935919, EPI_ISL_935920, EPI_ISL_935921, EPI_ISL_935922, EPI_ISL_935924, EPI_ISL_935925, EPI_ISL_935926, EPI_ISL_935927, EPI_ISL_935928, EPI_ISL_935929, EPI_ISL_935930, EPI_ISL_935931, EPI_ISL_935932, EPI_ISL_935933, EPI_ISL_935934, EPI_ISL_935935, EPI_ISL_935936, EPI_ISL_935937, EPI_ISL_935938, EPI_ISL_935939, EPI_ISL_935940, EPI_ISL_935941, EPI_ISL_935942, EPI_ISL_935943, EPI_ISL_935944, EPI_ISL_935945, EPI_ISL_935946, EPI_ISL_935947, EPI_ISL_935949, EPI_ISL_935950, EPI_ISL_935951			
see above	Cadham Provincial laboratory	National Microbiology Laboratory (NML)	Anna Majer, Shari Tyson, Grace Seo, Philip Mabon, Elsie Grudeski, Rhiannon Huzarewich, Russell Mandes, Anneliese Landgraff, Jennifer Tanner, Natalie Knox, Morag Graham, Gary Van Domselaar, Paul Van Caeseele, Jared Bullard, David Alexander, Kerry Dust, Nathalie Bastien, Yan Li, Timothy Booth, Darian Hole, Madison Chapel, Kirsten Biggar, CanCOGeN's metadata curation team, Public Health Agency of Canada CanCOGeN team
EPI_ISL_936489	Hospital Raja Perempuan Zainab II	Institute for Medical Research, Infectious Disease Research Centre, National Institutes of Health, Ministry of Health Malaysia	Suppiah J, Kamel K, Azizan MA, Thayan R
EPI_ISL_936490	Makmal Kesihatan Awam Kota Bharu	Institute for Medical Research, Infectious Disease Research Centre, National Institutes of Health, Ministry of Health Malaysia	Suppiah J, Kamel K, Azizan MA, Thayan R
EPI_ISL_936617, EPI_ISL_936618, EPI_ISL_936620, EPI_ISL_936622, EPI_ISL_936626, EPI_ISL_936629, EPI_ISL_936630, EPI_ISL_937015	Northwestern Memorial Hospital	Ozer Lab	Ramon Lorenzo-Redondo, Lacy M. Simons, Chad J. Achenbach, Lawrence J. Jennings, Michael G. Ison, Judd F. Hultquist, Egon A. Ozer
EPI_ISL_941275, EPI_ISL_941276, EPI_ISL_941277, EPI_ISL_941278, EPI_ISL_941279	Nigeria Centre for Disease Control (NCDC)	African Centre of Excellence for Genomics of Infectious Diseases (ACEGID), Redeemer's University	Oluniyi P.E. et al
EPI_ISL_941906	Virginia DCLS	Virginia DCLS	Virginia DCLS
EPI_ISL_941930	Florida Bureau of Public Health Laboratories	Florida Bureau of Public Health Laboratories	Sarah Schmedes, Jason Blanton
EPI_ISL_942402, EPI_ISL_942403	Virginia DCLS	Virginia DCLS	Virginia DCLS
EPI_ISL_942456, EPI_ISL_942457, EPI_ISL_942460, EPI_ISL_942461, EPI_ISL_942463, EPI_ISL_942465, EPI_ISL_942468, EPI_ISL_942469, EPI_ISL_942472, EPI_ISL_942473, EPI_ISL_942474, EPI_ISL_942476, EPI_ISL_942477, EPI_ISL_942481			
see above	Gundersen Molecular Diagnostics Laboratory	Kabara Cancer Research Institute	Craig S. Richmond, Paraic A. Kenny
EPI_ISL_942485, EPI_ISL_942490, EPI_ISL_942491, EPI_ISL_942492, EPI_ISL_942493	Gundersen Clinical Microbiology Laboratory	Kabara Cancer Research Institute	Craig S. Richmond, Paraic A. Kenny
EPI_ISL_942495, EPI_ISL_942497, EPI_ISL_942500, EPI_ISL_942501	Gundersen Molecular Diagnostics Laboratory	Kabara Cancer Research Institute	Craig S. Richmond, Paraic A. Kenny
EPI_ISL_942506	Gundersen Clinical Microbiology Laboratory	Kabara Cancer Research Institute	Craig S. Richmond, Paraic A. Kenny
EPI_ISL_942514, EPI_ISL_942518, EPI_ISL_942519, EPI_ISL_942521, EPI_ISL_942522, EPI_ISL_942523, EPI_ISL_942531, EPI_ISL_942533, EPI_ISL_942541, EPI_ISL_942542, EPI_ISL_942543, EPI_ISL_942545, EPI_ISL_942546, EPI_ISL_942548			
see above	Gundersen Molecular Diagnostics Laboratory	Kabara Cancer Research Institute	Craig S. Richmond, Paraic A. Kenny
EPI_ISL_943607, EPI_ISL_943609	Central Laboratory of Public Health of Rio Grande do Sul (Lacen-RS)	State Center for Health Surveillance of the Health Department of the State of Rio Grande do Sul (CEVS/SES-RS)	Aline Campos, Amanda da Silva, Anelise Schaurich, Claudia Dornelles, Cynthia Molina, Fernanda Godinho, Lara Crescente, Leticia Garay, Regina Barcellos, Richard Salvato, Tatiana Gregianini, Vagner Fonseca
EPI_ISL_947265	RS Sari Asih Ciledug	Eijkman Institute for Molecular Biology, Ministry of Research and Technology/National Agency for Research and Innovation	Frilasita A Yudhaputri, Hidayat Trimarsanto, Iskandar Adnan, Lydia V. Panggalo, Sukma Oktavianthi, Willy Agustine, Edison Johar, Safarina G Malik, Khin Saw Myint, Amin Soebandrio
EPI_ISL_947268	RSU Anggrek Mas	Eijkman Institute for Molecular Biology, Ministry of Research and Technology/National Agency for Research and Innovation	Frilasita A Yudhaputri, Hidayat Trimarsanto, Iskandar Adnan, Lydia V. Panggalo, Sukma Oktavianthi, Willy Agustine, Edison Johar, Safarina G Malik, Khin Saw Myint, Amin Soebandrio
EPI_ISL_947270	RS Mitra Keluarga Gading Serpong	Eijkman Institute for Molecular Biology, Ministry of Research and Technology/National Agency for Research and Innovation	Frilasita A Yudhaputri, Hidayat Trimarsanto, Iskandar Adnan, Lydia V. Panggalo, Sukma Oktavianthi, Willy Agustine, Edison Johar, Safarina G Malik, Khin Saw Myint, Amin Soebandrio
EPI_ISL_947273	RSIA Sayyidah	Eijkman Institute for Molecular Biology, Ministry of Research and Technology/National Agency for Research and Innovation	Frilasita A Yudhaputri, Hidayat Trimarsanto, Iskandar Adnan, Lydia V. Panggalo, Sukma Oktavianthi, Willy Agustine, Edison Johar, Safarina G Malik, Khin Saw Myint, Amin Soebandrio
EPI_ISL_949353, EPI_ISL_949355, EPI_ISL_949364, EPI_ISL_949369, EPI_ISL_949374, EPI_ISL_949377	University of Birmingham	COVID-19 Genomics UK (COG-UK) Consortium	Institute of Microbiology, University of Birmingham: Claire McMurray, Joanne Stockton, Samuel Nicholls, Radoslaw Poplawski, Will Rowe, Josh Quick, Nicholas Loman. University of Birmingham Testing Laboratory: Celina M Whalley, Andrew Bosworth, Charlotte Poxon, Kasun Wanigasooriya, Oliver Pickles, Mike Kidd, Alex Richter, Andrew D Beggs PHE Heartlands Lab: Husam Osman, Andrew Bosworth. Queen Elizabeth Hospital: Anna Casey
EPI_ISL_950620, EPI_ISL_950624, EPI_ISL_950627, EPI_ISL_950628, EPI_ISL_950635	Queens Medical Centre, Clinical Microbiology Department / DeepSeq Nottingham	COVID-19 Genomics UK (COG-UK) Consortium	Gemma Clark, Wendy Smith, Manjinder Khakh, Vicki M Fleming, Michelle M Lister, Hannah Howson-Wells, Jonathan Ball, Patrick McClure, Joseph Chappell, Theocharis Tsoleridis, Nadine Holmes, Matthew Carlisle, Christopher Moore, Fei Sang, Johnny Debebe, Victoria Wright, Matthew Loose
EPI_ISL_955156	University of Sarajevo, Veterinary Faculty, Laboratory for Molecular Diagnostic and Research Laboratory	University of Sarajevo, Veterinary Faculty, Laboratory for Molecular Diagnostic and Research Laboratory	Goleti T., Goleti Š., Softi A., Ali-Šeho A., Jaži A., Šabi E., Nicevi M., Hodži A., Terzi I.
EPI_ISL_955165, EPI_ISL_955166, EPI_ISL_955167, EPI_ISL_955168	Institute for Biocides and Medical Ecology	Institute of microbiology and Immunology, Faculty of Medicine, University of Belgrade	Knezevic,A., Jankovic,M., Vidanovic,D., Milicevic,O., Tesovic,B., Sekler,M., Jovanovic,T.
EPI_ISL_955192, EPI_ISL_955194, EPI_ISL_955195, EPI_ISL_955196, EPI_ISL_955197, EPI_ISL_955199, EPI_ISL_955201, EPI_ISL_955202, EPI_ISL_955203, EPI_ISL_955204, EPI_ISL_955208			
see above	Instituto Nacional de Medicina Genomica	Instituto Nacional de Medicina Genomica	Hidalgo-Miranda A, Mendoza-Vargas A, Reyes-Grajeda JP, Cisneros-Villanueva M, Cedro-Tanda A,Peñaloza-Figueroa F, Herrera-Montalvo LA
EPI_ISL_955337, EPI_ISL_955338, EPI_ISL_955339, EPI_ISL_955341, EPI_ISL_955342, EPI_ISL_955344,	UC Davis- Department of Pathology and Laboratory Medicine	Chan-Zuckerberg Biohub	CZB Cliahub Consortium

EPI_ISL_955346, EPI_ISL_955348, EPI_ISL_955351, EPI_ISL_955352			
EPI_ISL_955497, EPI_ISL_955500, EPI_ISL_955503, EPI_ISL_955505, EPI_ISL_955508, EPI_ISL_955510, EPI_ISL_955511, EPI_ISL_955512, EPI_ISL_955514, EPI_ISL_955515, EPI_ISL_955516, EPI_ISL_955519, EPI_ISL_955520, EPI_ISL_955523, EPI_ISL_955525, EPI_ISL_955527, EPI_ISL_955528, EPI_ISL_955531, EPI_ISL_955536			
see above	Humboldt County Public Health Laboratory	Chan-Zuckerberg Biohub	CZB Cliahub Consortium
EPI_ISL_955567, EPI_ISL_955568	Santa Clara County Public Health Laboratory	Chan-Zuckerberg Biohub	CZB Cliahub Consortium
EPI_ISL_955632	Orange County Public Health Lab	Chan-Zuckerberg Biohub	CZB Cliahub Consortium
EPI_ISL_955939, EPI_ISL_955945, EPI_ISL_955946, EPI_ISL_955947, EPI_ISL_955948, EPI_ISL_955950, EPI_ISL_955954, EPI_ISL_955955, EPI_ISL_955956, EPI_ISL_955957, EPI_ISL_955958, EPI_ISL_955959, EPI_ISL_955960, EPI_ISL_955961, EPI_ISL_955962, EPI_ISL_955963, EPI_ISL_955964, EPI_ISL_955965, EPI_ISL_955966, EPI_ISL_955967, EPI_ISL_955968, EPI_ISL_955969, EPI_ISL_955970, EPI_ISL_955971, EPI_ISL_955976, EPI_ISL_955977, EPI_ISL_955978, EPI_ISL_955979, EPI_ISL_955980, EPI_ISL_959480, EPI_ISL_959481, EPI_ISL_959482, EPI_ISL_959483, EPI_ISL_959484, EPI_ISL_959485, EPI_ISL_959487, EPI_ISL_959490, EPI_ISL_959491, EPI_ISL_959492, EPI_ISL_959493, EPI_ISL_959494, EPI_ISL_959495, EPI_ISL_959496, EPI_ISL_959500, EPI_ISL_959506, EPI_ISL_959508, EPI_ISL_959509, EPI_ISL_959510, EPI_ISL_959511, EPI_ISL_959514, EPI_ISL_959515, EPI_ISL_959516, EPI_ISL_959517, EPI_ISL_959518, EPI_ISL_959519, EPI_ISL_959520, EPI_ISL_959521, EPI_ISL_959522, EPI_ISL_959523, EPI_ISL_959524, EPI_ISL_959525, EPI_ISL_959527, EPI_ISL_959528, EPI_ISL_959529			
see above	Division of Emerging Infectious Diseases, Bureau of Infectious Diseases Diagnosis Control, Korea Disease Control and Prevention Agency	Division of Emerging Infectious Diseases, Bureau of Infectious Diseases Diagnosis Control, Korea Disease Control and Prevention Agency	Ae Kyung Park, Il-Hwan Kim, Heui Man Kim, Jeong-Min Kim, Namjoo Lee, Chae Young Lee, Sang Hee Woo, Eun-Jin Kim
EPI_ISL_962107	Illinois Department of Public Health	Gagnon Lab, Southern Illinois University	Keith Gagnon
EPI_ISL_962609, EPI_ISL_962614, EPI_ISL_962616, EPI_ISL_962771, EPI_ISL_962775, EPI_ISL_962781, EPI_ISL_962789, EPI_ISL_962794, EPI_ISL_962798, EPI_ISL_962804	San Diego County Public Health Laboratory	Andersen lab at Scripps Research	SEARCH Alliance San Diego with Tracy Basler, Jovan Shephard, Brett Austin
EPI_ISL_962892	ULSS 03 Venezia	Istituto Zooprofilattico Sperimentale delle Venezie	Natale Alda, Mazzotta Elisa, Mason Nicoletta, Ceglie Letizia, Lucchese L, Stefani Annalisa, Bonfante Francesco, Bortolami Alessio, Fincato Alice, Monne Isabella, Bellinati Laura, Mion Monica, Guadagno Carmine, Quaranta Erika, Pastori Ambra, Terregino Calogero
EPI_ISL_978522, EPI_ISL_978529, EPI_ISL_978531, EPI_ISL_978532	Central Public Health Laboratory - LACEN -Bahia, Salvador, Brazil	Central Public Health Laboratory - LACEN -Bahia, Salvador, Brazil	Stephane Tosta, Luciana Oliveira, Vanessa Nardy,Patrícia Cajado,Marcela Gómez, Breno Dominguez, Jaqueline Gomes, Vagner Fonseca,Marta Giovanetti,Luiz Alcantara, Felicidade Pereira, Arabela Leal
EPI_ISL_981176	Johns Hopkins Hospital Department of Pathology	Johns Hopkins Hospital Department of Pathology	C. Paul Morris, Chun Huai Luo, Adannaya Amadi, Matthew Schwartz, Nicholas Gallagher, Heba H. Mostafa
EPI_ISL_982903	MEPHI Aix Marseille University (AMU)	MEPHI Aix Marseille University (AMU)	Anthony LEVASSEUR
EPI_ISL_983699, EPI_ISL_983700, EPI_ISL_983701	Essentia Health-St. Mary's Medical Center	Minnesota Department of Health, Public Health Laboratory	Alexandra Lorentz, Jacob Garfin, Matt Plumb, and Xiong Wang
EPI_ISL_984563, EPI_ISL_984564, EPI_ISL_984570, EPI_ISL_984571, EPI_ISL_984574, EPI_ISL_984575, EPI_ISL_984579, EPI_ISL_984581	California Department of Public Health	Chiu Laboratory, University of California, San Francisco	Charles Chiu, Xianding (Wayne) Deng, Candace Wang, Venice Servellita, Jill Hacker, Debra Wadford
EPI_ISL_984737, EPI_ISL_984739, EPI_ISL_984749, EPI_ISL_984751	Gazi University Faculty of Medicine, Medical Virology Laboratory	Gazi University Faculty of Medicine, Medical Virology Laboratory	Erdem ahin, Gülendam Bozday, Hager Muftah, Selin Yiit, Shaknoza Sarzhanova, Özlem Güzel Tunçcan, Murat Dizbay, Il Fidan, Kayhan Çalar
EPI_ISL_984756	Pandemic Response Lab - NYC	Pandemic Response Lab, R&D	Henry Lee, Michael Hammerling, Melissa Hopkins, Cybill del Castillo, William Ward, Pradeep Bugga, Haiping Hao, Jon Laurent
EPI_ISL_989990, EPI_ISL_989993	Lighthouse Lab in Glasgow	Wellcome Sanger Institute for the COVID-19 Genomics UK (COG-UK) Consortium	Harper VanSteenhouse, Yumi Kasai, David Gray, Carol Clugston, Anna Dominiczak and Alex Alderton, Roberto Amato, Sonia Goncalves, Ewan Harrison, David K. Jackson, Ian Johnston, Dominic Kwiatkowski, Cordelia Langford, John Sillitoe on behalf of the Wellcome Sanger Institute COVID-19 Surveillance Team
EPI_ISL_991309, EPI_ISL_991310, EPI_ISL_991311, EPI_ISL_991312, EPI_ISL_991313, EPI_ISL_991314, EPI_ISL_991315, EPI_ISL_991320, EPI_ISL_991321, EPI_ISL_991323, EPI_ISL_991325, EPI_ISL_991326, EPI_ISL_991327, EPI_ISL_991328, EPI_ISL_991329, EPI_ISL_991330, EPI_ISL_991331, EPI_ISL_991332, EPI_ISL_991333, EPI_ISL_991334, EPI_ISL_991336, EPI_ISL_991339, EPI_ISL_991340, EPI_ISL_991343, EPI_ISL_991344, EPI_ISL_991345, EPI_ISL_991347, EPI_ISL_991348, EPI_ISL_991349, EPI_ISL_991350			
see above	Lighthouse Lab in Alderley Park	Wellcome Sanger Institute for the COVID-19 Genomics UK (COG-UK) Consortium	Jacquelyn Wynn, Mairead Hyland, The Lighthouse Lab in Alderley Park and Alex Alderton, Roberto Amato, Sonia Goncalves, Ewan Harrison, David K. Jackson, Ian Johnston, Dominic Kwiatkowski, Cordelia Langford, John Sillitoe on behalf of the Wellcome Sanger Institute COVID-19 Surveillance Team
EPI_ISL_994668, EPI_ISL_994674, EPI_ISL_994676, EPI_ISL_994677, EPI_ISL_994678, EPI_ISL_994679, EPI_ISL_994680, EPI_ISL_994682, EPI_ISL_994695, EPI_ISL_994704, EPI_ISL_994711, EPI_ISL_994735, EPI_ISL_994736, EPI_ISL_994737, EPI_ISL_994738			
see above	Division of Emerging Infectious Diseases, Bureau of Infectious Diseases Diagnosis Control, Korea Disease Control and Prevention Agency	Division of Emerging Infectious Diseases, Bureau of Infectious Diseases Diagnosis Control, Korea Disease Control and Prevention Agency	Ae Kyung Park, Il-Hwan Kim, Heui Man Kim, Jeong-Min Kim, Namjoo Lee, Chae Young Lee, Sang Hee Woo, Eun-Jin Kim
EPI_ISL_997121	Virology Department, Royal Infirmary of Edinburgh, NHS Lothian / School of Biological Sciences, University of Edinburgh	COVID-19 Genomics UK (COG-UK) Consortium	McHugh M, Dewar R, Cotton S, Rooke S, O'Toole Á, Scher E, Hill V, McCrone JT, Colquhoun R, Yu X, Jackson B, Rambaut A, Templeton K
EPI_ISL_997315, EPI_ISL_997317, EPI_ISL_997321	University of Exeter	COVID-19 Genomics UK (COG-UK) Consortium	Ben Temperton,Aaron Jeffries,Michelle Michelsen,Joanna Warwick-Dugdale,Audrey Farbos,Robyn Manley,Stephen Michell,Jane Masoli
EPI_ISL_998822	Quadram Institute Bioscience	COVID-19 Genomics UK (COG-UK) Consortium	Dave J. Baker, Gemma L. Kay, Alp Aydin, Thanh Le-Viet, Steven Rudder, Ana P. Tedim, Anastasia Kolyva, Maria Diaz, Leonardo de Oliveira Martins, Nabil-Fareed Alikhan, Lizzie Meadows, Rachael Stanley, Ngozi Elumogo, Muhammed Yasir, Nicholas M. Thomson, Alexander J Trotter, Rachel Gilroy, Samuel Bloomfield, Claire Stuart, Andrew Bell, Reenesh Prakash, Samir Dervisevic, Alison E. Mather, John Wain, Mark Webber, Andrew J. Page, Justin O'Grady

We gratefully acknowledge the following Authors from the Originating laboratories responsible for obtaining the specimens, as well as the Submitting laboratories where the genome data were generated and shared via GISAID, on which this research is based.

All Submitters of data may be contacted directly via www.gisaid.org

Authors are sorted alphabetically.

Accession ID	Originating Laboratory	Submitting Laboratory	Authors
EPI_ISL_1001236, EPI_ISL_1001242, EPI_ISL_1001249, EPI_ISL_1001250, EPI_ISL_1001251, EPI_ISL_1001253, EPI_ISL_1001254, EPI_ISL_1001255, EPI_ISL_1001256, EPI_ISL_1001257, EPI_ISL_1001258, EPI_ISL_1001259			
see above	National Virus Reference Laboratory	National Virus Reference Laboratory	Michael Carr, Gabriel Gonzalez, Zoe Yandle, Jonathan Dean, Cillian F De Gascun
EPI_ISL_1002357	Viollier AG	Department of Biosystems Science and Engineering, ETH Zürich	Chaoran Chen, Sarah Nadeau, Catharine Aquino, Ivan Topolsky, Philipp Jablonski, Lara Fuhrmann, David Dreifuss, Katharina Jahn, Andreia Cabral de Gouvea, Maria Domenica Moccia, Simon Grüter, Timothy Sykes, Lennart Opitz, Griffin White, Laura Neff, Doris Popovic, Andrea Patrignani, Jay Tracy, Ralph Schlapbach, Christiane Beckmann, Maurice Redondo, Olivier Kobel, Christoph Noppen, Sophie Seidel, Noemie Santamaria de Souza, Niko Beerenwinkel, Tanja Stadler
EPI_ISL_1004713, EPI_ISL_1004714, EPI_ISL_1004716, EPI_ISL_1004717, EPI_ISL_1004718, EPI_ISL_1004719, EPI_ISL_1004720, EPI_ISL_1004722, EPI_ISL_1004723, EPI_ISL_1004724, EPI_ISL_1004725, EPI_ISL_1004726, EPI_ISL_1004727, EPI_ISL_1004729, EPI_ISL_1004730, EPI_ISL_1004734, EPI_ISL_1004735, EPI_ISL_1004739, EPI_ISL_1004740, EPI_ISL_1004741, EPI_ISL_1004742, EPI_ISL_1004743, EPI_ISL_1004744, EPI_ISL_1004747, EPI_ISL_1004757, EPI_ISL_1004758, EPI_ISL_1004759, EPI_ISL_1004760, EPI_ISL_1004761, EPI_ISL_1004762, EPI_ISL_1004764, EPI_ISL_1004765, EPI_ISL_1004766, EPI_ISL_1004767, EPI_ISL_1004768, EPI_ISL_1004771, EPI_ISL_1004772, EPI_ISL_1004773, EPI_ISL_1004775, EPI_ISL_1004777, EPI_ISL_1004778, EPI_ISL_1004779, EPI_ISL_1004780, EPI_ISL_1004781, EPI_ISL_1004782, EPI_ISL_1004783, EPI_ISL_1004784, EPI_ISL_1004788, EPI_ISL_1004789, EPI_ISL_1004792, EPI_ISL_1004793, EPI_ISL_1004794, EPI_ISL_1004795, EPI_ISL_1004797, EPI_ISL_1004798, EPI_ISL_1004799, EPI_ISL_1004800, EPI_ISL_1004801, EPI_ISL_1004802, EPI_ISL_1004804, EPI_ISL_1004816, EPI_ISL_1004818, EPI_ISL_1004823, EPI_ISL_1004824, EPI_ISL_1004825, EPI_ISL_1004826, EPI_ISL_1004827, EPI_ISL_1004829, EPI_ISL_1004830, EPI_ISL_1004832, EPI_ISL_1004833, EPI_ISL_1004834, EPI_ISL_1004836, EPI_ISL_1004838, EPI_ISL_1004839, EPI_ISL_1004840, EPI_ISL_1004841, EPI_ISL_1004844, EPI_ISL_1004847, EPI_ISL_1004849, EPI_ISL_1004853, EPI_ISL_1004854, EPI_ISL_1004855, EPI_ISL_1004856, EPI_ISL_1004859, EPI_ISL_1004860, EPI_ISL_1004863, EPI_ISL_1004864, EPI_ISL_1004865, EPI_ISL_1004870, EPI_ISL_1004871, EPI_ISL_1004874, EPI_ISL_1004901, EPI_ISL_1004905, EPI_ISL_1004906, EPI_ISL_1004907, EPI_ISL_1004909, EPI_ISL_1004911, EPI_ISL_1004917, EPI_ISL_1004918, EPI_ISL_1004919, EPI_ISL_1004924, EPI_ISL_1004927, EPI_ISL_1004928, EPI_ISL_1004932, EPI_ISL_1004934, EPI_ISL_1004936, EPI_ISL_1004938, EPI_ISL_1004941, EPI_ISL_1004942, EPI_ISL_1004944, EPI_ISL_1004945, EPI_ISL_1004946, EPI_ISL_1004947, EPI_ISL_1004948, EPI_ISL_1004949, EPI_ISL_1004950, EPI_ISL_1004952, EPI_ISL_1004954, EPI_ISL_1004955, EPI_ISL_1004956, EPI_ISL_1004961, EPI_ISL_1004964, EPI_ISL_1004966, EPI_ISL_1004969, EPI_ISL_1004973, EPI_ISL_1004975			
see above	Viollier AG	Department of Biosystems Science and Engineering, ETH Zürich	Chaoran Chen, Sarah Nadeau, Ivan Topolsky, Emmanouil Dermitzakis, Keith Harshman, Ioannis Xenarios, Henri Pegeot, Lorenzo Cerutti, Deborah Penet, Philipp Jablonski, Lara Fuhrmann, David Dreifuss, Katharina Jahn, Christiane Beckmann, Maurice Redondo, Olivier Kobel, Christoph Noppen, Sophie Seidel, Noemie Santamaria de Souza, Niko Beerenwinkel, Tanja Stadler
EPI_ISL_1005560, EPI_ISL_1005561, EPI_ISL_1005562, EPI_ISL_1005563, EPI_ISL_1005564, EPI_ISL_1005566, EPI_ISL_1005571, EPI_ISL_1005573, EPI_ISL_1005574, EPI_ISL_1005575, EPI_ISL_1005577, EPI_ISL_1005578, EPI_ISL_1005579, EPI_ISL_1005580, EPI_ISL_1005581, EPI_ISL_1005582, EPI_ISL_1005656, EPI_ISL_1005659, EPI_ISL_1005661, EPI_ISL_1005662, EPI_ISL_1005663, EPI_ISL_1005665, EPI_ISL_1005666, EPI_ISL_1005667, EPI_ISL_1005668, EPI_ISL_1005669, EPI_ISL_1005671, EPI_ISL_1005673			
see above	Instituto Nacional de Medicina Genomica	Instituto Nacional de Medicina Genomica	Hidalgo-Miranda A, Mendoza-Vargas A, Reyes-Grajeda JP, Cisneros-Villanueva M, Cedro-Tanda A,Peñaloza-Figueroa F, Herrera-Montalvo LA
EPI_ISL_1007680, EPI_ISL_1007688, EPI_ISL_1007695	Hospital Clínico Universitario de Valladolid-Hospital Universitario Río Hortega	SeqCOVID-SPAIN consortium/IBV(CSIC)	Antonio Orduña-Domingo, Marta Hernández, Silvia Rojo, Marta Domínguez-Gil, Carmen Aldea-Mansilla, Juan Luis Muñoz-Bellido, Mª Fe Brezmes-Valdivieso, M. Isabel Fernandez-Natal, Jose María Eiros and SeqCOVID-SPAIN consortium
EPI_ISL_1007751, EPI_ISL_1007753, EPI_ISL_1007754, EPI_ISL_1007755, EPI_ISL_1007757, EPI_ISL_1007758, EPI_ISL_1007760, EPI_ISL_1007761	Hospital Universitario Virgen de las Nieves de Granada-SAS	SeqCOVID-SPAIN consortium/IBV(CSIC)	Sara Sanbonmatsu Gámez, Mercedes Pérez Ruiz, Irene Pedrosa Corral, José M. Navarro-Marí and SeqCOVID-SPAIN consortium
EPI_ISL_1007833	Servicio de Microbiología, Hospital Universitario Central de Asturias	SeqCOVID-SPAIN consortium/IBV(CSIC)	Cristián Castelló Abietar, Jose A. Boga, Susana Rojo-Alba, Marta Elena Álvarez-Argüelles, Santiago Melón and SeqCOVID-SPAIN consortium
EPI_ISL_1007995, EPI_ISL_1007996, EPI_ISL_1007998, EPI_ISL_1007999, EPI_ISL_1008000, EPI_ISL_1008001, EPI_ISL_1008003	Klinikum Wels-Grieskirchen	Bergthaler laboratory, CeMM Research Center for Molecular Medicine of the Austrian Academy of Sciences	Lukas Endler, Anna Schedl, Thomas Penz, Benedikt Agerer, Maelle Le Moing, Michael Schuster, Bekir Erguner, Jan Laine, Martin Senekowitsch, Christoph Bock, Andreas Bergthaler
EPI_ISL_1008013, EPI_ISL_1008014, EPI_ISL_1008016	Institut für Pathologie, Salzkammergut Klinikum Vöcklabruck	Bergthaler laboratory, CeMM Research Center for Molecular Medicine of the Austrian Academy of Sciences	Lukas Endler, Anna Schedl, Thomas Penz, Benedikt Agerer, Maelle Le Moing, Michael Schuster, Bekir Erguner, Jan Laine, Martin Senekowitsch, Christoph Bock, Andreas Bergthaler
EPI_ISL_1008109, EPI_ISL_1008112, EPI_ISL_1008113, EPI_ISL_1008114, EPI_ISL_1008115, EPI_ISL_1008116, EPI_ISL_1008117, EPI_ISL_1008118, EPI_ISL_1008120, EPI_ISL_1008121, EPI_ISL_1008123, EPI_ISL_1008124, EPI_ISL_1008125, EPI_ISL_1008129, EPI_ISL_1008130, EPI_ISL_1008131, EPI_ISL_1008133, EPI_ISL_1008134, EPI_ISL_1008136, EPI_ISL_1008137, EPI_ISL_1008138, EPI_ISL_1008139, EPI_ISL_1008140, EPI_ISL_1008141, EPI_ISL_1008142, EPI_ISL_1008143, EPI_ISL_1008144, EPI_ISL_1008145, EPI_ISL_1008146, EPI_ISL_1008148, EPI_ISL_1008151, EPI_ISL_1008153, EPI_ISL_1008157, EPI_ISL_1008158, EPI_ISL_1008160, EPI_ISL_1008161, EPI_ISL_1008162, EPI_ISL_1008163, EPI_ISL_1008164, EPI_ISL_1008165, EPI_ISL_1008170, EPI_ISL_1008171, EPI_ISL_1008172, EPI_ISL_1008173, EPI_ISL_1008174, EPI_ISL_1008175, EPI_ISL_1008176			
see above	Austrian Agency for Health and Food Safety (AGES)	Bergthaler laboratory, CeMM Research Center for Molecular Medicine of the Austrian Academy of Sciences	Lukas Endler, Anna Schedl, Thomas Penz, Benedikt Agerer, Maelle Le Moing, Michael Schuster, Bekir Erguner, Jan Laine, Martin Senekowitsch, Christoph Bock, Andreas Bergthaler
EPI_ISL_1008250, EPI_ISL_1008251, EPI_ISL_1008252, EPI_ISL_1008254, EPI_ISL_1008257, EPI_ISL_1008258, EPI_ISL_1008261, EPI_ISL_1008263, EPI_ISL_1008264, EPI_ISL_1008265, EPI_ISL_1008357, EPI_ISL_1008358, EPI_ISL_1008359, EPI_ISL_1008360, EPI_ISL_1008361, EPI_ISL_1008362, EPI_ISL_1008363, EPI_ISL_1008364, EPI_ISL_1008366, EPI_ISL_1008367, EPI_ISL_1008370, EPI_ISL_1008371, EPI_ISL_1008375			
see above	Department of Laboratory Medicine, Division of Clinical Virology, University of Medicine, Vienna	Bergthaler laboratory, CeMM Research Center for Molecular Medicine of the Austrian Academy of Sciences	Lukas Endler, Anna Schedl, Thomas Penz, Benedikt Agerer, Maelle Le Moing, Michael Schuster, Bekir Erguner, Jan Laine, Martin Senekowitsch, Christoph Bock, Andreas Bergthaler
EPI_ISL_1008410	A05 Diagnostics	The Public Health Agency of Sweden	Anna-Malin Linde, Maria Lind Karlberg, Carlo Berg, Oskar Karlsson Lindsjo, Sofia Stamouli, Reza Advani, Mattias Haukland, Petra Holmstrom, Noura Walai, Petra Edquist, Mia Brytting, Anna Risberg, Karin Tegmark-Wisell
EPI_ISL_1008433, EPI_ISL_1008435, EPI_ISL_1008437	Klinisk mikrobiologi	The Public Health Agency of Sweden	Anna-Malin Linde, Maria Lind Karlberg, Carlo Berg, Oskar Karlsson Lindsjo, Sofia Stamouli, Reza Advani, Mattias Haukland, Petra Holmstrom, Noura Walai, Petra Edquist, Mia Brytting, Anna Risberg, Karin Tegmark-Wisell
EPI_ISL_1008562	Klinisk Mikrobiologi	The Public Health Agency of Sweden	Anna-Malin Linde, Maria Lind Karlberg, Carlo Berg, Oskar Karlsson Lindsjo, Sofia Stamouli, Reza Advani, Mattias Haukland, Petra Holmstrom, Noura Walai, Petra Edquist, Mia Brytting, Anna Risberg, Karin Tegmark-Wisell
EPI_ISL_1008919, EPI_ISL_1008920	IL Department of Public Health Chicago Laboratory	Pathogen Discovery, Respiratory Viruses Branch, Division of Viral Diseases, Centers for Disease Control and Prevention	Ying Tao, Jing Zhang, Yan Li, Krista Queen, Anna Uehara, Peter Cook, Clinton R. Paden, Haibin Wang, Suxiang Tong
EPI_ISL_1008935, EPI_ISL_1008938, EPI_ISL_1008940, EPI_ISL_1008944, EPI_ISL_1008945, EPI_ISL_1008946, EPI_ISL_1008947	Servicio de Microbiología, Laboratori Clínic Metropolitana Nord. Hospital Universitari Germans Trias i Pujol. Institut d'Investigació en Ciències de la Salut Germans Trias i Pujol (IGTP)	SeqCOVID-SPAIN consortium/IBV(CSIC)	Elisa Martró, Antoni E. Bordoy, Anna Not, Adrián Antuori, Anabel Fernández, Nona Romaní, Verónica Saludes, Cristina Casañ and SeqCOVID-SPAIN consortium
EPI_ISL_1010728	Department of Laboratory Medicine, National Taiwan University Hospital	Microbial Genomics Core Lab, National Taiwan University Centers of Genomic and Precision Medicine	Shiou-Hwei Yeh, You-Yu Lin, Ya-Yun Lai, Chiao-Ling Li, Shan-Chwen Chang, Pei-Jer Chen, Sui-Yuan Chang
EPI_ISL_1011512, EPI_ISL_1011528	Massachusetts General Hospital	Infectious Disease Program, Broad Institute of Harvard and MIT	Lemieux,J.E., Siddle,K.J., Shaw,B., Adams,G., Pierce,V., Turbett,S., Anahtar,M., Branda,J., Slater,D., Harris,J., Lin,A.E., Gladden-Young,A., Lagerborg,K., Rudy,M., DeRuff,K., Carter,A., Normandin,E., Bauer,M., Reilly,S., Tomkins-Tinch,C., Loreth,C., Chaluvadi,S., Neumann,A., Cusick,C., Chapman,S.B., Gnirke,A., Flowers,K., Cerrato,F., Birren,B.W., Gallagher,G., Smole,S., Park,D.J., MacInnis,B.L., Ryan,E., LaRocque,R., Rosenberg,E. and Sabeti,P.C.
EPI_ISL_1011741, EPI_ISL_1011744, EPI_ISL_1011746	Rhode Island Department of Health	Infectious Disease Program, Broad Institute of Harvard and MIT	Lemieux,J.E., Siddle,K.J., Huard,R., King,E., Azevedo,K., Miller,A., Adams,G., Gladden-Young,A., Lagerborg,K., Rudy,M., DeRuff,K., Carter,A., Normandin,E., Bauer,M., Reilly,S., Tomkins-Tinch,C., Loreth,C., Chaluvadi,S., Birren,B.W., Gallagher,G., Smole,S., Park,D.J., MacInnis,B.L., and Sabeti,P.C.

EPI_ISL_1013533, EPI_ISL_1013553	1.AO Universitaria 'S. Giovanni di Dio e Ruggi D'Aragona, Scuola Medica Salernitana' Hospital / 2.UOC di Virologia e Microbiologia, Università della Campania 'L. Vanvitelli' / 3.AO Universitaria 'Federico II' Napoli Hospital / 4.AORN 'San Giuseppe Moscati' Avellino Hospital / 5.AO 'San Pio - presidio G. Rummo' Benevento Hospital / 6.AO 'Sant'Anna e San Sebastiano' Caserta Hospital / 7.PO 'Maria Santissima Addolorata' Eboli Hospital / 8.Biogem Istituto di Ricerche Genetiche	1. Genome Research Center for Health (CRGS) / 2. Laboratory of Molecular Medicine and Genomics(LMMGe) / 3. Center for Research in Pure and Applied Mathematics (CRMPA)	Giorgio Giurato, Francesca Rizzo, Alessandro Weisz, Gianluigi Franci, Giovanni Nassa, Pasquale Pagliano, Roberta Tarallo, Elena Alexandrova, Ylenia D'Agostino, Carlo Ferravante, Jessica Lamberti, Viola Melone, Domenico Memoli, Valeria Mirici Cappa, Domenico Palumbo, Giovanni Pecoraro, Assunta Sellitto, Oriana Strianese, Ilaria Terenzi, Giuseppe Fenza, Aniello Gentile, Antonello Saccomanno, Sonia Amabile, Teresa Rocco, Annamaria Salvati, Emilia Vaccaro, Massimiliano Galdiero, Michele Cennamo, Giuseppe Portella, Maria Grazia Foti, Mariarosaria Ingino, Maria Landi, Maurizio Fumi, Vincenzo Rocco, Rita Greco, Vittoria Letizia, Arnolfo Petruzziello, Maddalena Schioppa, Gregorio Goffredi, Francesca Marciano, Michele Caraglia, Alessia Cossu, Marianna Scrima, Edmondo Adorisio, Morena D'Avenia, Michela Iacobellis, Rosanna Piluscio, Giorgio Dirani, Vittorio Sambri, Simona Semprini, Silvia Zanoli, Francesco Curcio, Stefania Marzinotto, Andreina Baj, Fausto Sessa.
EPI_ISL_1014437, EPI_ISL_1014452, EPI_ISL_1014464, EPI_ISL_1014549, EPI_ISL_1014550, EPI_ISL_1014551, EPI_ISL_1014552, EPI_ISL_1014553, EPI_ISL_1014554, EPI_ISL_1014555, EPI_ISL_1014556, EPI_ISL_1014565, EPI_ISL_1014566, EPI_ISL_1014567, EPI_ISL_1014568, EPI_ISL_1014569, EPI_ISL_1014570, EPI_ISL_1014571			
see above	Dutch COVID-19 response team	National Institute for Public Health and the Environment (RIVM)	Adam Meijer, Harry Vennema, Dirk Eggink, Jeroen Cremer, Sharon van den Brink, Bas van der Veer, AnneMarie van den Brandt, Florian Zwagemaker, Dennis Schmitz, Chantal Reusken, on behalf of the national COVID-19 response team
EPI_ISL_1014685	National Influenza Center, Virology Department	National Influenza Center	K Sadeghi, A Nejati, J Yavarian, NZ Shafiei Jandaghi, V Salimi, F Ajaminejad,N Ghavvami and T Mokhtari Azad
EPI_ISL_1014686	National Influenza Center, Virology Department	National Influenza Center	NZ Shafiei Jandaghi, V Salimi, A Nejati, K Sadeghi, J Yavarian, N Ghavvami,F Ajaminejad and T Mokhtari Azad
EPI_ISL_1015205, EPI_ISL_1015206, EPI_ISL_1015208	Department of Virology, Pitié-Salpêtrière hospital	Department of Virology, Pitié-Salpêtrière hospital	Valentin Leducq, Aude Jary, Karen Zafilaza, Stéphane Marot, Vincent Calvez, Anne-Geneviève Marcelin
EPI_ISL_1015363	Kingston Health Sciences Centre and Queen's University	Ontario Institute for Cancer Research	Prameet M. Sheth,Calvin Sjaarda,Robert Colautti,Katya Douchant,Ilinca Lungu,Bernard Lam,Jeremy Johns,Paul Krzyzanowski,Richard de Borja,Felicia Vincelli,Philip Zuzarte,Jared T. Simpson
EPI_ISL_1015379	Biology Lab, HIA STE-ANNE	IRBA, 2MI	GORGE O., NOLENT F., SARILAR V., JANVIER F.
EPI_ISL_1015726	Arizona State Public Health Laboratory	Arizona State Public Health Laboratory	Trung Huynh, Jessica Escobar, Katherine Fullerton, Nobuko Fukushima, Stacy White, Linda Getsinger, Victor Waddell
EPI_ISL_1016405, EPI_ISL_1016406, EPI_ISL_1016410, EPI_ISL_1016411	MEMORIAL SLOAN KETTERING CANCER CENTER	Wadsworth Center, New York State Department of Health	Kirsten St. George, Daryl M. Lamson, Alexis Russel, Matthew Shudt, Melissa A Leisner, Jonathan Plitnick, Navjot Singh, John Kelly, Erasmus Schneider, Erica Lasek-Nesselquist
EPI_ISL_1016690	University of Sarajevo, Veterinary Faculty, Laboratory for Molecular Diagnostic and Research Laboratory	University of Sarajevo, Veterinary Faculty, Laboratory for Molecular Diagnostic and Research Laboratory	Goletic T., Goletic S., Softic A., Alic-Seho A., Sabic E., Terzic I., Jazic A., Nicevic M., Hodzic A.
EPI_ISL_1016891, EPI_ISL_1016892, EPI_ISL_1016893, EPI_ISL_1016894, EPI_ISL_1016897, EPI_ISL_1016898, EPI_ISL_1016901, EPI_ISL_1016902, EPI_ISL_1016903, EPI_ISL_1016904, EPI_ISL_1016905, EPI_ISL_1016906, EPI_ISL_1016907, EPI_ISL_1016909, EPI_ISL_1016910, EPI_ISL_1016911, EPI_ISL_1016912			
see above	Arizona State Public Health Laboratory	Arizona State Public Health Laboratory	Trung Huynh, Jessica Escobar, Katherine Fullerton, Nobuko Fukushima, Stacy White, Linda Getsinger, Victor Waddell
EPI_ISL_1016963, EPI_ISL_1016965	UNMC Emerging Pathogens Laboratory	UNMC COVID-19 Response Team	UNMC COVID-19 Response Team
EPI_ISL_1017052, EPI_ISL_1017058	Providence St. Joseph Health Orange - Chapman	Providence St. Joseph Health Molecular Genomics Laboratory	Alexa K Dowdell, Brian D Piening, Fred L Robinson, Carlo B Bifulco, Mary Campbell
EPI_ISL_1017059, EPI_ISL_1017060, EPI_ISL_1017061, EPI_ISL_1017062, EPI_ISL_1017063	Providence Saint Joseph Medical Center - Burbank	Providence St. Joseph Health Molecular Genomics Laboratory	Alexa K Dowdell, Brian D Piening, Fred L Robinson, Carlo B Bifulco, Mary Campbell
EPI_ISL_1017075, EPI_ISL_1017076, EPI_ISL_1017077, EPI_ISL_1017078, EPI_ISL_1017079, EPI_ISL_1017080, EPI_ISL_1017081, EPI_ISL_1017082, EPI_ISL_1017083, EPI_ISL_1017084, EPI_ISL_1017085, EPI_ISL_1017086, EPI_ISL_1017087, EPI_ISL_1017088, EPI_ISL_1017089			
see above	Providence St. Joseph Health Orange - Chapman	Providence St. Joseph Health Molecular Genomics Laboratory	Alexa K Dowdell, Brian D Piening, Fred L Robinson, Carlo B Bifulco, Mary Campbell
EPI_ISL_1017090	Providence Oregon Regional Laboratories	Providence St. Joseph Health Molecular Genomics Laboratory	Alexa K Dowdell, Brian D Piening, Fred L Robinson, Carlo B Bifulco, Mary Campbell
EPI_ISL_1017091, EPI_ISL_1017092, EPI_ISL_1017093, EPI_ISL_1017094, EPI_ISL_1017095	Providence St. Joseph Health Orange - Chapman	Providence St. Joseph Health Molecular Genomics Laboratory	Alexa K Dowdell, Brian D Piening, Fred L Robinson, Carlo B Bifulco, Mary Campbell
EPI_ISL_1017096	Providence Oregon Regional Laboratories	Providence St. Joseph Health Molecular Genomics Laboratory	Alexa K Dowdell, Brian D Piening, Fred L Robinson, Carlo B Bifulco, Mary Campbell
EPI_ISL_1017097, EPI_ISL_1017098, EPI_ISL_1017099	Providence Saint Joseph Medical Center - Burbank	Providence St. Joseph Health Molecular Genomics Laboratory	Alexa K Dowdell, Brian D Piening, Fred L Robinson, Carlo B Bifulco, Mary Campbell
EPI_ISL_1017100, EPI_ISL_1017101, EPI_ISL_1017102, EPI_ISL_1017103, EPI_ISL_1017104, EPI_ISL_1017105	Providence Oregon Regional Laboratories	Providence St. Joseph Health Molecular Genomics Laboratory	Alexa K Dowdell, Brian D Piening, Fred L Robinson, Carlo B Bifulco, Mary Campbell
EPI_ISL_1017106	Providence St. Joseph Health Orange - Chapman	Providence St. Joseph Health Molecular Genomics Laboratory	Alexa K Dowdell, Brian D Piening, Fred L Robinson, Carlo B Bifulco, Mary Campbell
EPI_ISL_1017107	Providence Oregon Regional Laboratories	Providence St. Joseph Health Molecular Genomics Laboratory	Alexa K Dowdell, Brian D Piening, Fred L Robinson, Carlo B Bifulco, Mary Campbell
EPI_ISL_1017108, EPI_ISL_1017109, EPI_ISL_1017110, EPI_ISL_1017111	Providence Saint Joseph Medical Center - Burbank	Providence St. Joseph Health Molecular Genomics Laboratory	Alexa K Dowdell, Brian D Piening, Fred L Robinson, Carlo B Bifulco, Mary Campbell
EPI_ISL_1017112, EPI_ISL_1017113, EPI_ISL_1017114, EPI_ISL_1017115	Providence Oregon Regional Laboratories	Providence St. Joseph Health Molecular Genomics Laboratory	Alexa K Dowdell, Brian D Piening, Fred L Robinson, Carlo B Bifulco, Mary Campbell
EPI_ISL_1017116, EPI_ISL_1017117	Providence Saint Joseph Medical Center - Burbank	Providence St. Joseph Health Molecular Genomics Laboratory	Alexa K Dowdell, Brian D Piening, Fred L Robinson, Carlo B Bifulco, Mary Campbell
EPI_ISL_1017118, EPI_ISL_1017119	Providence St. Joseph Health Orange - Chapman	Providence St. Joseph Health Molecular Genomics Laboratory	Alexa K Dowdell, Brian D Piening, Fred L Robinson, Carlo B Bifulco, Mary Campbell
EPI_ISL_1017120, EPI_ISL_1017121, EPI_ISL_1017122, EPI_ISL_1017123, EPI_ISL_1017124, EPI_ISL_1017125	Providence Oregon Regional Laboratories	Providence St. Joseph Health Molecular Genomics Laboratory	Alexa K Dowdell, Brian D Piening, Fred L Robinson, Carlo B Bifulco, Mary Campbell
EPI_ISL_1017126, EPI_ISL_1017127, EPI_ISL_1017128, EPI_ISL_1017129, EPI_ISL_1017130, EPI_ISL_1017131, EPI_ISL_1017132	Providence Saint Joseph Medical Center - Burbank	Providence St. Joseph Health Molecular Genomics Laboratory	Alexa K Dowdell, Brian D Piening, Fred L Robinson, Carlo B Bifulco, Mary Campbell
EPI_ISL_1017133, EPI_ISL_1017134, EPI_ISL_1017135, EPI_ISL_1017136, EPI_ISL_1017137, EPI_ISL_1017138, EPI_ISL_1017139, EPI_ISL_1017140, EPI_ISL_1017141, EPI_ISL_1017142, EPI_ISL_1017143, EPI_ISL_1017144, EPI_ISL_1017145, EPI_ISL_1017146			
see above	Providence St. Joseph Health Orange - Chapman	Providence St. Joseph Health Molecular Genomics Laboratory	Alexa K Dowdell, Brian D Piening, Fred L Robinson, Carlo B Bifulco, Mary Campbell
EPI_ISL_1017147, EPI_ISL_1017148, EPI_ISL_1017149, EPI_ISL_1017150, EPI_ISL_1017151, EPI_ISL_1017152, EPI_ISL_1017153, EPI_ISL_1017154, EPI_ISL_1017155, EPI_ISL_1017156, EPI_ISL_1017157, EPI_ISL_1017158, EPI_ISL_1017159, EPI_ISL_1017160			
see above	Providence Saint Joseph Medical Center - Burbank	Providence St. Joseph Health Molecular Genomics Laboratory	Alexa K Dowdell, Brian D Piening, Fred L Robinson, Carlo B Bifulco, Mary Campbell
EPI_ISL_1017161, EPI_ISL_1017162, EPI_ISL_1017163, EPI_ISL_1017164, EPI_ISL_1017165, EPI_ISL_1017166, EPI_ISL_1017167, EPI_ISL_1017168, EPI_ISL_1017169, EPI_ISL_1017170, EPI_ISL_1017171			
see above	Providence Regional Medical Center Everett	Providence St. Joseph Health Molecular Genomics Laboratory	Alexa K Dowdell, Brian D Piening, Fred L Robinson, Carlo B Bifulco, Mary Campbell
EPI_ISL_1017172	Providence Oregon Regional Laboratories	Providence St. Joseph Health Molecular Genomics Laboratory	Alexa K Dowdell, Brian D Piening, Fred L Robinson, Carlo B Bifulco, Mary Campbell
EPI_ISL_1017506, EPI_ISL_1017522, EPI_ISL_1017526, EPI_ISL_1017527, EPI_ISL_1017530, EPI_ISL_1017539, EPI_ISL_1017544, EPI_ISL_1017549, EPI_ISL_1017556, EPI_ISL_1017558, EPI_ISL_1017560			
see above	Murphy Medical Associates	Grubaugh Lab - Yale School of Public Health	Mary Petrone, Joseph Fauver, Caleb Neal, Steven Murphy, Chantal Vogels, Mallery Breban, Annie Watkins, Tara Alpert, Nathan Grubaugh

EPI_ISL_1017685	Institute of Virology, Vaccines and Sera "Torlak"	Institute of microbiology and Immunology, Faculty of Medicine, University of Belgrade	Knezevic,A., Jankovic,M., Vidanovic,D., Milicevic,O., Tesovic,B., Sekler,M., Jovanovic,T.
EPI_ISL_1018088, EPI_ISL_1018089, EPI_ISL_1018090, EPI_ISL_1018091, EPI_ISL_1018092, EPI_ISL_1018093, EPI_ISL_1018094, EPI_ISL_1018095, EPI_ISL_1018096, EPI_ISL_1018097, EPI_ISL_1018098			
see above	Immunology, Noguchi Memorial Institute for Medical Research	Immunology, Noguchi Memorial Institute for Medical Research	Adu,B., Egyir,B., Kumordjie,S., Agbodzi,B., Yeboah,C., Mohktar,Q., Oteng,F., Owusu-Nyantakyi,C., Asare,K.M., Appiah-Kubi,J., Adusei-Poku,M.A., Odoom,J.K., Ampofo,W.K., Bonney,J.K.
EPI_ISL_1020064, EPI_ISL_1020066, EPI_ISL_1020067, EPI_ISL_1020069, EPI_ISL_1020070, EPI_ISL_1020071, EPI_ISL_1020074, EPI_ISL_1020075, EPI_ISL_1020076, EPI_ISL_1020079, EPI_ISL_1020084, EPI_ISL_1020088, EPI_ISL_1020089, EPI_ISL_1020093, EPI_ISL_1020095, EPI_ISL_1020096, EPI_ISL_1020099, EPI_ISL_1020101, EPI_ISL_1020102, EPI_ISL_1020104, EPI_ISL_1020105, EPI_ISL_1020108, EPI_ISL_1020109, EPI_ISL_1020110, EPI_ISL_1020111, EPI_ISL_1020114, EPI_ISL_1020115, EPI_ISL_1020116, EPI_ISL_1020119, EPI_ISL_1020214, EPI_ISL_1020216, EPI_ISL_1020219, EPI_ISL_1020220, EPI_ISL_1020222, EPI_ISL_1020223, EPI_ISL_1020226, EPI_ISL_1020227, EPI_ISL_1020232, EPI_ISL_1020233, EPI_ISL_1020234, EPI_ISL_1020236, EPI_ISL_1020237, EPI_ISL_1020239, EPI_ISL_1020241, EPI_ISL_1020242, EPI_ISL_1020243, EPI_ISL_1020246, EPI_ISL_1020247, EPI_ISL_1020248, EPI_ISL_1020249, EPI_ISL_1020252, EPI_ISL_1020253, EPI_ISL_1020255, EPI_ISL_1020257, EPI_ISL_1020258, EPI_ISL_1020259, EPI_ISL_1020262, EPI_ISL_1020265, EPI_ISL_1020266, EPI_ISL_1020267, EPI_ISL_1020269, EPI_ISL_1020270, EPI_ISL_1020272, EPI_ISL_1020273, EPI_ISL_1020274, EPI_ISL_1020275, EPI_ISL_1020277, EPI_ISL_1020278, EPI_ISL_1020279, EPI_ISL_1020280, EPI_ISL_1020281			
see above	Department of Health Technology and Informatics, The Hong Kong Polytechnic University	Department of Health Technology and Informatics, The Hong Kong Polytechnic University	Gilman Kit-Hang Siu, Lam-Kwong Lee, Kenneth Siu-Sing Leung, Jake Siu-Lun Leung, Timothy Ting-Leung Ng, Chloe Toi-Mei Chan, Kingsley King-Gee Tam, Hiu-Yin Lao, Denise Sze-Hang Wong, Alan Ka-Lun Wu, Miranda Chong-Yee Yau, Yvette Wai-Man Lai, Kitty Sau-Chun Fung, Sandy Ka-Yee Chau, Barry Kin-Chung Wong, Wing-Kin To, Kristine Luk, Alex Yat-Man Ho, Tak-Lun Que, Kam-Tong Yip, Wing Cheong Yam, David Ho-Keung Shum, Shea Ping Yip
EPI_ISL_1020307, EPI_ISL_1020308, EPI_ISL_1020309	Istituto Zooprofilattico Sperimentale dell' Umbria e delle Marche -Togo Rosati Università degli Studi di Perugia	Department of Food safety, Nutrition and Veterinary public health - Istituto Superiore di Sanità	Massimo Biagetti , Monica Giammarioli, Luca De Sabato, Gabriele Vaccari, Ilaria Di Bartolo, Giovanni Ianiro, Antonella Mencacci, Barbara Camilloni
EPI_ISL_1020317, EPI_ISL_1020318, EPI_ISL_1020319	Department of Infectious Diseases and Immunology, National Hospital Organization Nagoya Medical Center	Clinical Research Center, National Hospital Organization Nagoya Medical Center	Yoshihiro Nakata, Hirotaka Ode, Mai Kubota, Masakazu Matsuda, Kazuhiro Matsuoka, Miho Nakasuji, Mikiko Mori, Mayumi Imahashi, Yoshiyuki Yokomaku, Yasumasa Iwatani
EPI_ISL_1020488, EPI_ISL_1020495, EPI_ISL_1020496, EPI_ISL_1020514, EPI_ISL_1020517, EPI_ISL_1020536, EPI_ISL_1020538, EPI_ISL_1020545, EPI_ISL_1020548, EPI_ISL_1020549	Columbia University Irving Medical Center	Wadsworth Center, New York State Department of Health	Kirsten St. George, Daryl M. Lamson, Alexis Russel, Matthew Shudt, Melissa A Leisner, Jonathan Plitnick, Navjot Singh, John Kelly, Erasmus Schneider, Erica Lasek-Nesselquist
EPI_ISL_1023686, EPI_ISL_1023688, EPI_ISL_1023690, EPI_ISL_1023692, EPI_ISL_1023708	Wyoming Public Health Laboratory	Wyoming Public Health Laboratory	Noah Hull, Taylor Fearing, Lynette Gumbleton, Channing Weber, Ashley Norberg, Bailey Bowcutt, and Wanda Manley
EPI_ISL_1025476, EPI_ISL_1025477, EPI_ISL_1025480, EPI_ISL_1025483, EPI_ISL_1025484, EPI_ISL_1025487, EPI_ISL_1025488, EPI_ISL_1025490, EPI_ISL_1025491, EPI_ISL_1025492, EPI_ISL_1025495, EPI_ISL_1025496, EPI_ISL_1025497, EPI_ISL_1025499, EPI_ISL_1025501, EPI_ISL_1025503, EPI_ISL_1025504, EPI_ISL_1025505, EPI_ISL_1025507, EPI_ISL_1025508, EPI_ISL_1025509, EPI_ISL_1025510, EPI_ISL_1025516, EPI_ISL_1025517, EPI_ISL_1025518, EPI_ISL_1025519, EPI_ISL_1025520, EPI_ISL_1025521, EPI_ISL_1025522, EPI_ISL_1025523, EPI_ISL_1025526, EPI_ISL_1025528, EPI_ISL_1025529, EPI_ISL_1025532, EPI_ISL_1025535, EPI_ISL_1025536, EPI_ISL_1025539, EPI_ISL_1025541, EPI_ISL_1025542, EPI_ISL_1025543, EPI_ISL_1025544, EPI_ISL_1025546, EPI_ISL_1025548, EPI_ISL_1025549, EPI_ISL_1025551, EPI_ISL_1025552, EPI_ISL_1025553, EPI_ISL_1025554, EPI_ISL_1025558, EPI_ISL_1025559, EPI_ISL_1025560, EPI_ISL_1025561, EPI_ISL_1025562, EPI_ISL_1025563, EPI_ISL_1025566, EPI_ISL_1025568, EPI_ISL_1025570, EPI_ISL_1025573, EPI_ISL_1025574, EPI_ISL_1025576, EPI_ISL_1025577, EPI_ISL_1025580, EPI_ISL_1025582, EPI_ISL_1025584, EPI_ISL_1025585, EPI_ISL_1025586, EPI_ISL_1025587, EPI_ISL_1025590, EPI_ISL_1025591, EPI_ISL_1025592, EPI_ISL_1025593, EPI_ISL_1025594, EPI_ISL_1025596, EPI_ISL_1025597, EPI_ISL_1025599, EPI_ISL_1025601, EPI_ISL_1025604, EPI_ISL_1025605, EPI_ISL_1025608, EPI_ISL_1025609, EPI_ISL_1025611, EPI_ISL_1025612, EPI_ISL_1025613, EPI_ISL_1025614, EPI_ISL_1025615, EPI_ISL_1025616, EPI_ISL_1025617, EPI_ISL_1025620, EPI_ISL_1025622, EPI_ISL_1025625, EPI_ISL_1025626, EPI_ISL_1025627, EPI_ISL_1025628, EPI_ISL_1025633, EPI_ISL_1025634, EPI_ISL_1025635, EPI_ISL_1025636, EPI_ISL_1025638, EPI_ISL_1025639, EPI_ISL_1025641, EPI_ISL_1025644, EPI_ISL_1025645, EPI_ISL_1025648, EPI_ISL_1025650, EPI_ISL_1025653, EPI_ISL_1025654, EPI_ISL_1025655, EPI_ISL_1025656, EPI_ISL_1025658, EPI_ISL_1025659, EPI_ISL_1025660, EPI_ISL_1025663, EPI_ISL_1025664, EPI_ISL_1025666, EPI_ISL_1025669, EPI_ISL_1025670, EPI_ISL_1025675, EPI_ISL_1025678, EPI_ISL_1025680, EPI_ISL_1025681, EPI_ISL_1025682, EPI_ISL_1025683, EPI_ISL_1025685, EPI_ISL_1025689, EPI_ISL_1025692, EPI_ISL_1025694, EPI_ISL_1025696, EPI_ISL_1025697, EPI_ISL_1025699, EPI_ISL_1025702, EPI_ISL_1025703, EPI_ISL_1025705, EPI_ISL_1025706, EPI_ISL_1025707, EPI_ISL_1025708, EPI_ISL_1025711, EPI_ISL_1025712, EPI_ISL_1025714, EPI_ISL_1025716, EPI_ISL_1025718, EPI_ISL_1025719, EPI_ISL_1025720, EPI_ISL_1025723, EPI_ISL_1025725, EPI_ISL_1025726, EPI_ISL_1025727, EPI_ISL_1025728, EPI_ISL_1025729, EPI_ISL_1025730, EPI_ISL_1025731, EPI_ISL_1025732, EPI_ISL_1025733, EPI_ISL_1025734, EPI_ISL_1025735, EPI_ISL_1025737, EPI_ISL_1025738, EPI_ISL_1025740, EPI_ISL_1025741, EPI_ISL_1025742, EPI_ISL_1025743, EPI_ISL_1025744, EPI_ISL_1025745, EPI_ISL_1025747, EPI_ISL_1025748, EPI_ISL_1025749, EPI_ISL_1025750, EPI_ISL_1025751, EPI_ISL_1025755, EPI_ISL_1025758, EPI_ISL_1025761, EPI_ISL_1025764, EPI_ISL_1025769, EPI_ISL_1025770, EPI_ISL_1025771, EPI_ISL_1025773, EPI_ISL_1025774, EPI_ISL_1025776, EPI_ISL_1025778, EPI_ISL_1025780, EPI_ISL_1025782, EPI_ISL_1025784, EPI_ISL_1025786, EPI_ISL_1025788, EPI_ISL_1025789, EPI_ISL_1025790, EPI_ISL_1025793, EPI_ISL_1025794, EPI_ISL_1025795, EPI_ISL_1025796, EPI_ISL_1025798, EPI_ISL_1025800, EPI_ISL_1025803, EPI_ISL_1025804, EPI_ISL_1025805, EPI_ISL_1025812, EPI_ISL_1025815, EPI_ISL_1025816, EPI_ISL_1025817, EPI_ISL_1025818, EPI_ISL_1025819, EPI_ISL_1025821, EPI_ISL_1025822, EPI_ISL_1025823, EPI_ISL_1025825, EPI_ISL_1025826, EPI_ISL_1025829, EPI_ISL_1025830, EPI_ISL_1025831, EPI_ISL_1025832, EPI_ISL_1025835, EPI_ISL_1025837, EPI_ISL_1025838, EPI_ISL_1025839, EPI_ISL_1025842, EPI_ISL_1025844, EPI_ISL_1025846, EPI_ISL_1025848, EPI_ISL_1025849, EPI_ISL_1025850, EPI_ISL_1025852, EPI_ISL_1025853, EPI_ISL_1025854, EPI_ISL_1025855, EPI_ISL_1025856, EPI_ISL_1025857, EPI_ISL_1025860, EPI_ISL_1025861, EPI_ISL_1025862, EPI_ISL_1025863, EPI_ISL_1025866, EPI_ISL_1025868, EPI_ISL_1025874, EPI_ISL_1025877, EPI_ISL_1025878, EPI_ISL_1025879, EPI_ISL_1025880, EPI_ISL_1025881, EPI_ISL_1025882, EPI_ISL_1025883, EPI_ISL_1025885, EPI_ISL_1025886, EPI_ISL_1025887, EPI_ISL_1025888, EPI_ISL_1025891, EPI_ISL_1025894, EPI_ISL_1025896, EPI_ISL_1025897, EPI_ISL_1025898, EPI_ISL_1025899, EPI_ISL_1025900, EPI_ISL_1025902, EPI_ISL_1025903, EPI_ISL_1025904, EPI_ISL_1025907			
see above	Department of Virus and Microbiological Special Diagnostics, Statens Serum Institut, Copenhagen, Denmark	Aalborg University	Danish Covid-19 Genome Consortium
EPI_ISL_1027378, EPI_ISL_1027380, EPI_ISL_1027396, EPI_ISL_1027397, EPI_ISL_1027402, EPI_ISL_1027408, EPI_ISL_1027410, EPI_ISL_1027430	Orange County Public Health Lab	Chan-Zuckerberg Biohub	CZB Cliahub Consortium
EPI_ISL_1027438, EPI_ISL_1027440	Santa Clara County Public Health Laboratory	Chan-Zuckerberg Biohub	CZB Cliahub Consortium
EPI_ISL_1027445, EPI_ISL_1027449, EPI_ISL_1027450, EPI_ISL_1027456	Alameda County Public Health Lab	Chan-Zuckerberg Biohub	CZB Cliahub Consortium
EPI_ISL_1027540	Humboldt County Public Health Laboratory	Chan-Zuckerberg Biohub	CZB Cliahub Consortium
EPI_ISL_1027546	Santa Clara County Public Health Laboratory	Chan-Zuckerberg Biohub	CZB Cliahub Consortium
EPI_ISL_1027561	Humboldt County Public Health Laboratory	Chan-Zuckerberg Biohub	CZB Cliahub Consortium
EPI_ISL_1027637, EPI_ISL_1027639, EPI_ISL_1027641, EPI_ISL_1027642, EPI_ISL_1027643	Department of Microbiology, National Institute for Public Health of Kosova	Charité Universitätsmedizin Berlin, Institut für Virologie	Victor M Corman, Julia Schneider, Donjeta Hajdari, Zana Deva, Xhevat Jakupi, Barbara Mühlemann, Jörn Beheim-Schwarzbach, Talitha Veith, Terry Jones, Christian Drosten
EPI_ISL_1029054, EPI_ISL_1029055, EPI_ISL_1029056, EPI_ISL_1029057, EPI_ISL_1029058, EPI_ISL_1029059, EPI_ISL_1029060, EPI_ISL_1029061, EPI_ISL_1029062, EPI_ISL_1029063, EPI_ISL_1029064, EPI_ISL_1029065, EPI_ISL_1029066, EPI_ISL_1029067, EPI_ISL_1029068, EPI_ISL_1029069, EPI_ISL_1029070, EPI_ISL_1029071, EPI_ISL_1029074, EPI_ISL_1029075, EPI_ISL_1029076, EPI_ISL_1029077, EPI_ISL_1029078, EPI_ISL_1029079, EPI_ISL_1029080, EPI_ISL_1029081, EPI_ISL_1029082, EPI_ISL_1029083, EPI_ISL_1029084, EPI_ISL_1029085, EPI_ISL_1029086, EPI_ISL_1029087, EPI_ISL_1029088, EPI_ISL_1029089, EPI_ISL_1029090, EPI_ISL_1029092, EPI_ISL_1029093, EPI_ISL_1029094, EPI_ISL_1029096, EPI_ISL_1029097, EPI_ISL_1029098, EPI_ISL_1029099, EPI_ISL_1029100, EPI_ISL_1029101, EPI_ISL_1029102, EPI_ISL_1029103, EPI_ISL_1029104, EPI_ISL_1029105, EPI_ISL_1029106, EPI_ISL_1029107, EPI_ISL_1029108, EPI_ISL_1029109, EPI_ISL_1029110, EPI_ISL_1029111, EPI_ISL_1029113, EPI_ISL_1029114, EPI_ISL_1029115, EPI_ISL_1029116, EPI_ISL_1029117, EPI_ISL_1029118, EPI_ISL_1029119, EPI_ISL_1029120, EPI_ISL_1029121, EPI_ISL_1029122, EPI_ISL_1029123, EPI_ISL_1029124, EPI_ISL_1029125, EPI_ISL_1029126, EPI_ISL_1029127, EPI_ISL_1029128, EPI_ISL_1029129, EPI_ISL_1029130, EPI_ISL_1029131, EPI_ISL_1029132, EPI_ISL_1029133, EPI_ISL_1029134, EPI_ISL_1029135, EPI_ISL_1029136, EPI_ISL_1029137, EPI_ISL_1029138, EPI_ISL_1029139, EPI_ISL_1029140, EPI_ISL_1029141, EPI_ISL_1029142, EPI_ISL_1029143, EPI_ISL_1029144, EPI_ISL_1029145, EPI_ISL_1029146, EPI_ISL_1029147, EPI_ISL_1029148, EPI_ISL_1029149, EPI_ISL_1029150, EPI_ISL_1029151, EPI_ISL_1029152, EPI_ISL_1029154, EPI_ISL_1029155, EPI_ISL_1029156, EPI_ISL_1029157, EPI_ISL_1029158, EPI_ISL_1029159, EPI_ISL_1029160, EPI_ISL_1029161, EPI_ISL_1029162, EPI_ISL_1029164, EPI_ISL_1029165, EPI_ISL_1029166, EPI_ISL_1029167, EPI_ISL_1029168, EPI_ISL_1029169, EPI_ISL_1029170, EPI_ISL_1029171, EPI_ISL_1029172, EPI_ISL_1029173, EPI_ISL_1029174, EPI_ISL_1029175, EPI_ISL_1029176, EPI_ISL_1029177, EPI_ISL_1029178, EPI_ISL_1029179, EPI_ISL_1029180, EPI_ISL_1029181, EPI_ISL_1029182, EPI_ISL_1029183, EPI_ISL_1029184, EPI_ISL_1029185, EPI_ISL_1029186, EPI_ISL_1029187, EPI_ISL_1029188, EPI_ISL_1029189, EPI_ISL_1029190, EPI_ISL_1029191, EPI_ISL_1029192, EPI_ISL_1029193, EPI_ISL_1029194, EPI_ISL_1029195, EPI_ISL_1029196, EPI_ISL_1029197, EPI_ISL_1029198, EPI_ISL_1029199, EPI_ISL_1029200, EPI_ISL_1029201, EPI_ISL_1029202, EPI_ISL_1029203, EPI_ISL_1029204, EPI_ISL_1029205, EPI_ISL_1029206, EPI_ISL_1029207, EPI_ISL_1029208, EPI_ISL_1029209, EPI_ISL_1029211, EPI_ISL_1029212, EPI_ISL_1029213, EPI_ISL_1029214, EPI_ISL_1029215, EPI_ISL_1029216, EPI_ISL_1029217, EPI_ISL_1029218, EPI_ISL_1029219, EPI_ISL_1029221, EPI_ISL_1029222, EPI_ISL_1029224, EPI_ISL_1029225, EPI_ISL_1029226, EPI_ISL_1029227, EPI_ISL_1029228, EPI_ISL_1029229, EPI_ISL_1029230, EPI_ISL_1029231, EPI_ISL_1029233, EPI_ISL_1029234, EPI_ISL_1029235, EPI_ISL_1029236, EPI_ISL_1029237, EPI_ISL_1029238, EPI_ISL_1029239, EPI_ISL_1029240, EPI_ISL_1029242, EPI_ISL_1029244, EPI_ISL_1029245, EPI_ISL_1029246, EPI_ISL_1029247, EPI_ISL_1029248, EPI_ISL_1029249, EPI_ISL_1029250, EPI_ISL_1029251, EPI_ISL_1029252, EPI_ISL_1029253, EPI_ISL_1029254, EPI_ISL_1029255, EPI_ISL_1029256, EPI_ISL_1029258, EPI_ISL_1029259, EPI_ISL_1029260, EPI_ISL_1029261, EPI_ISL_1029262, EPI_ISL_1029263, EPI_ISL_1029264, EPI_ISL_1029265, EPI_ISL_1029266, EPI_ISL_1029267, EPI_ISL_1029268, EPI_ISL_1029269, EPI_ISL_1029270, EPI_ISL_1029271, EPI_ISL_1029272, EPI_ISL_1029273, EPI_ISL_1029274, EPI_ISL_1029275, EPI_ISL_1029276, EPI_ISL_1029277, EPI_ISL_1029278, EPI_ISL_1029279, EPI_ISL_1029280, EPI_ISL_1029281, EPI_ISL_1029282, EPI_ISL_1029283, EPI_ISL_1029284, EPI_ISL_1029285, EPI_ISL_1029287, EPI_ISL_1029288, EPI_ISL_1029289, EPI_ISL_1029290, EPI_ISL_1029291, EPI_ISL_1029292, EPI_ISL_1029293, EPI_ISL_1029294, EPI_ISL_1029295, EPI_ISL_1029296, EPI_ISL_1029297, EPI_ISL_1029299, EPI_ISL_1029300, EPI_ISL_1029301, EPI_ISL_1029303, EPI_ISL_1029304, EPI_ISL_1029305, EPI_ISL_1029306, EPI_ISL_1029307, EPI_ISL_1029308, EPI_ISL_1029309, EPI_ISL_1029310, EPI_ISL_1029311, EPI_ISL_1029312, EPI_ISL_1029313, EPI_ISL_1029314, EPI_ISL_1029315, EPI_ISL_1029316, EPI_ISL_1029317, EPI_ISL_1029318, EPI_ISL_1029319, EPI_ISL_1029320, EPI_ISL_1029321, EPI_ISL_1029322, EPI_ISL_1029323, EPI_ISL_1029324, EPI_ISL_1029325, EPI_ISL_1029326, EPI_ISL_1029327, EPI_ISL_1029328, EPI_ISL_1029329, EPI_ISL_1029330, EPI_ISL_1029331, EPI_ISL_1029332, EPI_ISL_1029333, EPI_ISL_1029334, EPI_ISL_1029336, EPI_ISL_1029337, EPI_ISL_1029338, EPI_ISL_1029339, EPI_ISL_1029340, EPI_ISL_1029341, EPI_ISL_1029342, EPI_ISL_1029343, EPI_ISL_1029345, EPI_ISL_1029346, EPI_ISL_1029347, EPI_ISL_1029348, EPI_ISL_1029349, EPI_ISL_1029350, EPI_ISL_1029351, EPI_ISL_1029352, EPI_ISL_1029353, EPI_ISL_1029354, EPI_ISL_1029355, EPI_ISL_1029356, EPI_ISL_1029357, EPI_ISL_1029358, EPI_ISL_1029359, EPI_ISL_1029360, EPI_ISL_1029361, EPI_ISL_1029362, EPI_ISL_1029363, EPI_ISL_1029364, EPI_ISL_1029366, EPI_ISL_1029367, EPI_ISL_1029368, EPI_ISL_1029369, EPI_ISL_1029370, EPI_ISL_1029371, EPI_ISL_1029372, EPI_ISL_1029374, EPI_ISL_1029375, EPI_ISL_1029376, EPI_ISL_1029377, EPI_ISL_1029378, EPI_ISL_1029379, EPI_ISL_1029380, EPI_ISL_1029381, EPI_ISL_1029382, EPI_ISL_1029383, EPI_ISL_1029384, EPI_ISL_1029385, EPI_ISL_1029386, EPI_ISL_1029387, EPI_ISL_1029388, EPI_ISL_1029389, EPI_ISL_1029390, EPI_ISL_1029391, EPI_ISL_1029392, EPI_ISL_1029393, EPI_ISL_1029394, EPI_ISL_1029395, EPI_ISL_1029396, EPI_ISL_1029397, EPI_ISL_1029398, EPI_ISL_1029399, EPI_ISL_1029400, EPI_ISL_1029401, EPI_ISL_1029402, EPI_ISL_1029403, EPI_ISL_1029404, EPI_ISL_1029405, EPI_ISL_1029406, EPI_ISL_1029407, EPI_ISL_1029408, EPI_ISL_1029409, EPI_ISL_1029410, EPI_ISL_1029411, EPI_ISL_1029412, EPI_ISL_1029413, EPI_ISL_1029414, EPI_ISL_1029415, EPI_ISL_1029416, EPI_ISL_1029417, EPI_ISL_1029418, EPI_ISL_1029419, EPI_ISL_1029420, EPI_ISL_1029422, EPI_ISL_1029423, EPI_ISL_1029424, EPI_ISL_1029425, EPI_ISL_1029426, EPI_ISL_1029427, EPI_ISL_1029428, EPI_ISL_1029430, EPI_ISL_1029431, EPI_ISL_1029432, EPI_ISL_1029433, EPI_ISL_1029434, EPI_ISL_1029435, EPI_ISL_1029436, EPI_ISL_1029437, EPI_ISL_1029438, EPI_ISL_1029439, EPI_ISL_1029440, EPI_ISL_1029441, EPI_ISL_1029442, EPI_ISL_1029443, EPI_ISL_1029444, EPI_ISL_1029445, EPI_ISL_1029447, EPI_ISL_1029448, EPI_ISL_1029449, EPI_ISL_1029450, EPI_ISL_1029451,			

EPI_ISL_1029452, EPI_ISL_1029453, EPI_ISL_1029454, EPI_ISL_1029455, EPI_ISL_1029456, EPI_ISL_1029457, EPI_ISL_1029458, EPI_ISL_1029459, EPI_ISL_1029460, EPI_ISL_1029461, EPI_ISL_1029462, EPI_ISL_1029463, EPI_ISL_1029464, EPI_ISL_1029465, EPI_ISL_1029466, EPI_ISL_1029467, EPI_ISL_1029468, EPI_ISL_1029469, EPI_ISL_1029470, EPI_ISL_1029471, EPI_ISL_1029472, EPI_ISL_1029473, EPI_ISL_1029474, EPI_ISL_1029475, EPI_ISL_1029476, EPI_ISL_1029477, EPI_ISL_1029478, EPI_ISL_1029479, EPI_ISL_1029480, EPI_ISL_1029481, EPI_ISL_1029482, EPI_ISL_1029483, EPI_ISL_1029485, EPI_ISL_1029486, EPI_ISL_1029487, EPI_ISL_1029488, EPI_ISL_1029489, EPI_ISL_1029490, EPI_ISL_1029491, EPI_ISL_1029492, EPI_ISL_1029493, EPI_ISL_1029494, EPI_ISL_1029495, EPI_ISL_1029496, EPI_ISL_1029497, EPI_ISL_1029498, EPI_ISL_1029499, EPI_ISL_1029500, EPI_ISL_1029501, EPI_ISL_1029502, EPI_ISL_1029503, EPI_ISL_1029504, EPI_ISL_1029505, EPI_ISL_1029506, EPI_ISL_1029507, EPI_ISL_1029508, EPI_ISL_1029509, EPI_ISL_1029510, EPI_ISL_1029511, EPI_ISL_1029512, EPI_ISL_1029513, EPI_ISL_1029514, EPI_ISL_1029515, EPI_ISL_1029516, EPI_ISL_1029517, EPI_ISL_1029518, EPI_ISL_1029519, EPI_ISL_1029520, EPI_ISL_1029521, EPI_ISL_1029522, EPI_ISL_1029523, EPI_ISL_1029524, EPI_ISL_1029525, EPI_ISL_1029526, EPI_ISL_1029527, EPI_ISL_1029528, EPI_ISL_1029529, EPI_ISL_1029530, EPI_ISL_1029531, EPI_ISL_1029532, EPI_ISL_1029533, EPI_ISL_1029534, EPI_ISL_1029535, EPI_ISL_1029536, EPI_ISL_1029537, EPI_ISL_1029538, EPI_ISL_1029539, EPI_ISL_1029540, EPI_ISL_1029541, EPI_ISL_1029542, EPI_ISL_1029543, EPI_ISL_1029544, EPI_ISL_1029545, EPI_ISL_1029546, EPI_ISL_1029547, EPI_ISL_1029548, EPI_ISL_1029549, EPI_ISL_1029550, EPI_ISL_1029551, EPI_ISL_1029552, EPI_ISL_1029553, EPI_ISL_1029554, EPI_ISL_1029555, EPI_ISL_1029556, EPI_ISL_1029557, EPI_ISL_1029558, EPI_ISL_1029559, EPI_ISL_1029560, EPI_ISL_1029561, EPI_ISL_1029562, EPI_ISL_1029563, EPI_ISL_1029572, EPI_ISL_1029573, EPI_ISL_1029579, EPI_ISL_1029580, EPI_ISL_1029581, EPI_ISL_1029582, EPI_ISL_1029583, EPI_ISL_1029584, EPI_ISL_1029585, EPI_ISL_1029586, EPI_ISL_1029587, EPI_ISL_1029588, EPI_ISL_1029589, EPI_ISL_1029590, EPI_ISL_1029591, EPI_ISL_1029592, EPI_ISL_1029593, EPI_ISL_1029594, EPI_ISL_1029596, EPI_ISL_1029598, EPI_ISL_1029599, EPI_ISL_1029600, EPI_ISL_1029601, EPI_ISL_1029602, EPI_ISL_1029603, EPI_ISL_1029604, EPI_ISL_1029605, EPI_ISL_1029606, EPI_ISL_1029607, EPI_ISL_1029608, EPI_ISL_1029609, EPI_ISL_1029610, EPI_ISL_1029612, EPI_ISL_1029613, EPI_ISL_1029614, EPI_ISL_1029616, EPI_ISL_1029617, EPI_ISL_1029618, EPI_ISL_1029619, EPI_ISL_1029620, EPI_ISL_1029621, EPI_ISL_1029622, EPI_ISL_1029623, EPI_ISL_1029624, EPI_ISL_1029625, EPI_ISL_1029626, EPI_ISL_1029627, EPI_ISL_1029628, EPI_ISL_1029629, EPI_ISL_1029630, EPI_ISL_1029631, EPI_ISL_1029632, EPI_ISL_1029633, EPI_ISL_1029634, EPI_ISL_1029635, EPI_ISL_1029636, EPI_ISL_1029637, EPI_ISL_1029638, EPI_ISL_1029639, EPI_ISL_1029640, EPI_ISL_1029641, EPI_ISL_1029642, EPI_ISL_1029643, EPI_ISL_1029644, EPI_ISL_1029645, EPI_ISL_1029646, EPI_ISL_1029647, EPI_ISL_1029648, EPI_ISL_1029649, EPI_ISL_1029650, EPI_ISL_1029651, EPI_ISL_1029652, EPI_ISL_1029653, EPI_ISL_1029654, EPI_ISL_1029655, EPI_ISL_1029656, EPI_ISL_1029657, EPI_ISL_1029658, EPI_ISL_1029660, EPI_ISL_1029661, EPI_ISL_1029662, EPI_ISL_1029663, EPI_ISL_1029664, EPI_ISL_1029665, EPI_ISL_1029666, EPI_ISL_1029667, EPI_ISL_1029668, EPI_ISL_1029669, EPI_ISL_1029670, EPI_ISL_1029671, EPI_ISL_1029672, EPI_ISL_1029673, EPI_ISL_1029674, EPI_ISL_1029675, EPI_ISL_1029676, EPI_ISL_1029677, EPI_ISL_1029678, EPI_ISL_1029679, EPI_ISL_1029680, EPI_ISL_1029681, EPI_ISL_1029682, EPI_ISL_1029683, EPI_ISL_1029684, EPI_ISL_1029685, EPI_ISL_1029686, EPI_ISL_1029687, EPI_ISL_1029688, EPI_ISL_1029689, EPI_ISL_1029690, EPI_ISL_1029691, EPI_ISL_1029692, EPI_ISL_1029693, EPI_ISL_1029694, EPI_ISL_1029695, EPI_ISL_1029696, EPI_ISL_1029697, EPI_ISL_1029699, EPI_ISL_1029700, EPI_ISL_1029701, EPI_ISL_1029702, EPI_ISL_1029703, EPI_ISL_1029704, EPI_ISL_1029705, EPI_ISL_1029706, EPI_ISL_1029707, EPI_ISL_1029708, EPI_ISL_1029709, EPI_ISL_1029710, EPI_ISL_1029711, EPI_ISL_1029712, EPI_ISL_1029713, EPI_ISL_1029714, EPI_ISL_1029715, EPI_ISL_1029716, EPI_ISL_1029717, EPI_ISL_1029718, EPI_ISL_1029719, EPI_ISL_1029720, EPI_ISL_1029721, EPI_ISL_1029722, EPI_ISL_1029724, EPI_ISL_1029725, EPI_ISL_1029726, EPI_ISL_1029727, EPI_ISL_1029728, EPI_ISL_1029729, EPI_ISL_1029730, EPI_ISL_1029731, EPI_ISL_1029732, EPI_ISL_1029733, EPI_ISL_1029734, EPI_ISL_1029735, EPI_ISL_1029737, EPI_ISL_1029738, EPI_ISL_1029739, EPI_ISL_1029740, EPI_ISL_1029741, EPI_ISL_1029742, EPI_ISL_1029743, EPI_ISL_1029744, EPI_ISL_1029745, EPI_ISL_1029746, EPI_ISL_1029747, EPI_ISL_1029748, EPI_ISL_1029749, EPI_ISL_1029750, EPI_ISL_1029751, EPI_ISL_1029752, EPI_ISL_1029753, EPI_ISL_1029754, EPI_ISL_1029755, EPI_ISL_1029756, EPI_ISL_1029757, EPI_ISL_1029758, EPI_ISL_1029759, EPI_ISL_1029760, EPI_ISL_1029761, EPI_ISL_1029762, EPI_ISL_1029763, EPI_ISL_1029764, EPI_ISL_1029765, EPI_ISL_1029769, EPI_ISL_1029770, EPI_ISL_1029772, EPI_ISL_1029774, EPI_ISL_1029792, EPI_ISL_1029815, EPI_ISL_1029816, EPI_ISL_1029817, EPI_ISL_1029818, EPI_ISL_1029819, EPI_ISL_1029820, EPI_ISL_1029821, EPI_ISL_1029822, EPI_ISL_1029823, EPI_ISL_1029824, EPI_ISL_1029825, EPI_ISL_1029826, EPI_ISL_1029827, EPI_ISL_1029828, EPI_ISL_1029829, EPI_ISL_1029866, EPI_ISL_1029890, EPI_ISL_1029891, EPI_ISL_1029896, EPI_ISL_1029910, EPI_ISL_1029911, EPI_ISL_1029913, EPI_ISL_1029914, EPI_ISL_1029915, EPI_ISL_1029916, EPI_ISL_1029918, EPI_ISL_1029919, EPI_ISL_1029920, EPI_ISL_1029923, EPI_ISL_1029924, EPI_ISL_1029925, EPI_ISL_1029928, EPI_ISL_1029929, EPI_ISL_1029930, EPI_ISL_1029931, EPI_ISL_1029932, EPI_ISL_1029933, EPI_ISL_1029934, EPI_ISL_1029935, EPI_ISL_1029936, EPI_ISL_1029937, EPI_ISL_1029938, EPI_ISL_1029939, EPI_ISL_1029940, EPI_ISL_1029941, EPI_ISL_1029942, EPI_ISL_1029943, EPI_ISL_1029944, EPI_ISL_1029945, EPI_ISL_1029946, EPI_ISL_1029947, EPI_ISL_1029948, EPI_ISL_1029949, EPI_ISL_1029950, EPI_ISL_1029951, EPI_ISL_1029952, EPI_ISL_1029953, EPI_ISL_1029954, EPI_ISL_1029955			
see above	Laboratory Corporation of America	Respiratory Viruses Branch, Division of Viral Diseases, Centers for Disease Control and Prevention	Peter W. Cook, Dakota Howard, Dhwani Batra, Ben L. Rambo-Martin, Clinton R. Paden, Suxiang Tong, Duncan MacCannell
EPI_ISL_1030308, EPI_ISL_1030312	Orange County Public Health Lab	Chan-Zuckerberg Biohub	CZB Cliahub Consortium
EPI_ISL_1030409, EPI_ISL_1030422, EPI_ISL_1030430, EPI_ISL_1030434, EPI_ISL_1030439	Santa Clara County Public Health Laboratory	Chan-Zuckerberg Biohub	CZB Cliahub Consortium
EPI_ISL_1030445, EPI_ISL_1030446, EPI_ISL_1030448, EPI_ISL_1030449, EPI_ISL_1030450, EPI_ISL_1030452, EPI_ISL_1030454, EPI_ISL_1030459, EPI_ISL_1030461, EPI_ISL_1030464, EPI_ISL_1030466, EPI_ISL_1030467, EPI_ISL_1030468, EPI_ISL_1030470, EPI_ISL_1030471, EPI_ISL_1030472, EPI_ISL_1030474, EPI_ISL_1030477, EPI_ISL_1030481, EPI_ISL_1030482, EPI_ISL_1030485, EPI_ISL_1030486, EPI_ISL_1030490, EPI_ISL_1030491, EPI_ISL_1030492, EPI_ISL_1030493, EPI_ISL_1030494			
see above	Orange County Public Health Lab	Chan-Zuckerberg Biohub	CZB Cliahub Consortium
EPI_ISL_1030500, EPI_ISL_1030502	Santa Clara County Public Health Laboratory	Chan-Zuckerberg Biohub	CZB Cliahub Consortium
EPI_ISL_1032591, EPI_ISL_1032595, EPI_ISL_1032597, EPI_ISL_1032598, EPI_ISL_1032600, EPI_ISL_1032602, EPI_ISL_1032604, EPI_ISL_1032609, EPI_ISL_1032611, EPI_ISL_1032612, EPI_ISL_1032614			
see above	Wyoming Public Health Laboratory	Wyoming Public Health Laboratory	Noah Hull, Taylor Fearing, Lynette Gumbleton, Channing Weber, Ashley Norberg, Bailey Bowcutt, and Wanda Manley
EPI_ISL_1034317	Akershus University Hospital, Department for Microbiology and Infectious Disease Control	Norwegian Institute of Public Health, Department of Virology	Kathrine Stene-Johansen, Kamilla Heddeland Instefjord, Hilde Elshaug, Atiya R Ali,Marie Paulsen Madsen, Ignacio Garcia Llorente, Serina B Engebretsen, Rasmus Riis Kopperud, Hilde Vollan, Karoline Bragstad, Olav Hungnes
EPI_ISL_1034323	Oslo University Hospital, Department of Medical Microbiology	Norwegian Institute of Public Health, Department of Virology	Kathrine Stene-Johansen, Kamilla Heddeland Instefjord, Hilde Elshaug, Atiya R Ali,Marie Paulsen Madsen, Ignacio Garcia Llorente, Serina B Engebretsen, Rasmus Riis Kopperud, Hilde Vollan, Karoline Bragstad, Olav Hungnes
EPI_ISL_1034324	Department of Medical Microbiology - section Molde, Molde Hospital	Norwegian Institute of Public Health, Department of Virology	Kathrine Stene-Johansen, Kamilla Heddeland Instefjord, Hilde Elshaug, Atiya R Ali,Marie Paulsen Madsen, Ignacio Garcia Llorente, Serina B Engebretsen, Rasmus Riis Kopperud, Hilde Vollan, Karoline Bragstad, Olav Hungnes
EPI_ISL_1034325	Akershus University Hospital, Department for Microbiology and Infectious Disease Control	Norwegian Institute of Public Health, Department of Virology	Kathrine Stene-Johansen, Kamilla Heddeland Instefjord, Hilde Elshaug, Atiya R Ali,Marie Paulsen Madsen, Ignacio Garcia Llorente, Serina B Engebretsen, Rasmus Riis Kopperud, Hilde Vollan, Karoline Bragstad, Olav Hungnes
EPI_ISL_1034347, EPI_ISL_1034348, EPI_ISL_1034350	Department of Medical Microbiology, St. Olavs hospital	Norwegian Institute of Public Health, Department of Virology	Kathrine Stene-Johansen, Kamilla Heddeland Instefjord, Hilde Elshaug, Atiya R Ali,Marie Paulsen Madsen, Ignacio Garcia Llorente, Serina B Engebretsen, Rasmus Riis Kopperud, Hilde Vollan, Karoline Bragstad, Olav Hungnes
EPI_ISL_1034737, EPI_ISL_1034738, EPI_ISL_1034739	Center of Advanced Studies and Technology, Molecular Genetics Laboratory	Center of Advanced Studies and Technology, Molecular Genetics Laboratory	Ferrante Rossella, Mandatori Domitilla, De Fabritiis Simone
EPI_ISL_1034794	National Institute of Laboratory Medicine and Referral Center	Genomic Research Lab, BCSIR	Shahina Akter, Mohammad Samir Uzzaman, Eshrar Osman, Md. Ahashan Habib, Tanjina Akhtar Banu, Abu Sayeed Mohammad Mahmud, Md. Murshed Hasan Sarkar, Barna Goswami, Iffat Jahan, Md. Saddam Hossain, Tasnim Nafisa, Md. Maruf Ahmed Molla, Mahmuda Yeasmin, Asish Kumar Ghosh, Arifa Akram, A. K. M.Shamsuzzaman, Md. Salim Khan
EPI_ISL_1035042, EPI_ISL_1035269, EPI_ISL_1035560, EPI_ISL_1035590, EPI_ISL_1035592	Dutch COVID-19 response team	National Institute for Public Health and the Environment (RIVM)	Adam Meijer, Harry Vennema, Dirk Eggink, Jeroen Cremer, Sharon van den Brink, Bas van der Veer, AnneMarie van den Brandt, Florian Zwagemaker, Dennis Schmitz, Chantal Reusken, on behalf of the national COVID-19 response team
EPI_ISL_1035809	National Institute of Laboratory Medicine and Referral Center	Genomic Research Lab, BCSIR	Tanjina Akhtar Banu, Mohammad Samir Uzzaman, Eshrar Osman, Md. Ahashan Habib, Shahina Akter, Abu Sayeed Mohammad Mahmud, Md. Murshed Hasan Sarkar, Barna Goswami, Iffat Jahan, Md. Saddam Hossain, Tasnim Nafisa, Md. Maruf Ahmed Molla, Mahmuda Yeasmin, Asish Kumar Ghosh, Arifa Akram, A. K. M.Shamsuzzaman, Md. Salim Khan
EPI_ISL_1035812, EPI_ISL_1035813, EPI_ISL_1035815, EPI_ISL_1035818, EPI_ISL_1035820, EPI_ISL_1035824, EPI_ISL_1035826, EPI_ISL_1035827	Nigerian Centre for Disease Control (NCDC)	African Centre of Excellence for Genomics of Infectious Diseases (ACEGID), Redeemer's University, Ede	Olawoye, I.B., et al
EPI_ISL_1035872	National Institute of Laboratory Medicine and Referral Center	Genomic Research Lab, BCSIR, Dr. Qudrat-E-Khuda Road, Dhaka-1205, Bangladesh	Md. Saddam Hossain, Mohammad Samir Uzzaman, Eshrar Osman, Md. Ahashan Habib, Shahina Akter, Tanjina Akhtar Banu, Abu Sayeed Mohammad Mahmud, Md. Murshed Hasan Sarkar, Barna Goswami, Iffat Jahan, Tasnim Nafisa, Md. Maruf Ahmed Molla, Mahmuda Yeasmin, Asish Kumar Ghosh, Arifa Akram, A. K. M.Shamsuzzaman, Md. Salim Khan
EPI_ISL_1036107, EPI_ISL_1036111, EPI_ISL_1036122, EPI_ISL_1036124, EPI_ISL_1036126, EPI_ISL_1036127, EPI_ISL_1036134	Labormedizinisches Zentrum Dr Risch	University Hospital Basel, Clinical Bacteriology	Tim Roloff, Madlen Stange, Helena MB Seth-Smith, Alfredo Mari, Karoline Leuzinger, Julia Bielicki, Nadia Wohlwend,Martin Risch, Lorenz Risch, Manuel Battegay, Hans Hirsch, Adrian Egli
EPI_ISL_1036233	National Institute of Laboratory Medicine and Referral Center	Genomic Research Lab, BCSIR	Md. Ahashan Habib, Mohammad Samir Uzzaman, Eshrar Osman, Shahina Akter, Tanjina Akhtar Banu, Abu Sayeed Mohammad Mahmud, Md. Murshed Hasan Sarkar, Barna Goswami, Iffat Jahan, Md. Saddam Hossain, Tasnim Nafisa, Md. Maruf Ahmed Molla, Mahmuda Yeasmin, Asish Kumar Ghosh, Arifa Akram, A. K. M.Shamsuzzaman, Md. Salim Khan
EPI_ISL_1036237	Center of Advanced Studies and Technology, Molecular Genetics Laboratory	Center of Advanced Studies and Technology, Molecular Genetics Laboratory	Ferrante Rossella, Mandatori Domitilla, De Fabritiis Simone, Damiani Verena, Anaclerio Federico
EPI_ISL_1036316, EPI_ISL_1036317, EPI_ISL_1036318, EPI_ISL_1036319, EPI_ISL_1036323, EPI_ISL_1036326, EPI_ISL_1036328, EPI_ISL_1036331, EPI_ISL_1036332, EPI_ISL_1036334, EPI_ISL_1036335, EPI_ISL_1036336, EPI_ISL_1036337, EPI_ISL_1036339, EPI_ISL_1036352, EPI_ISL_1036356, EPI_ISL_1036357, EPI_ISL_1036369			
see above	Johns Hopkins Hospital Department of Pathology	Johns Hopkins Hospital Department of Pathology	C. Paul Morris, Chun Huai Luo, Adannaya Amadi, Matthew Schwartz, Nicholas Gallagher, Heba H. Mostafa
EPI_ISL_1036622	National Institute of Laboratory Medicine and Referral Center	Genomic Research Lab, BCSIR	Md. Murshed Hasan Sarkar, Mohammad Samir Uzzaman, Eshrar Osman, Md. Ahashan Habib, Shahina Akter, Tanjina Akhtar Banu, Abu Sayeed Mohammad Mahmud, Barna Goswami, Iffat Jahan, Md. Saddam Hossain, Tasnim Nafisa, Md. Maruf Ahmed Molla, Mahmuda Yeasmin, Asish Kumar

			Ghosh, Arifa Akram, A. K. M. Shamsuzzaman, Md. Salim Khan
EPI_ISL_1036658	Tyler Radniecki	Center for Genome Research and Biocomputing	Oregon State University TRACE Project
EPI_ISL_1036665, EPI_ISL_1036666, EPI_ISL_1036668, EPI_ISL_1036676, EPI_ISL_1036680	Wyoming Public Health Laboratory	Wyoming Public Health Laboratory	Noah Hull, Taylor Fearing, Lynette Gumbleton, Channing Weber, Ashley Norberg, Bailey Bowcutt, and Wanda Manley
EPI_ISL_1036751	National Institute of Laboratory Medicine and Referral Center	Genomic Research Lab, BCSIR	Shahina Akter, Mohammad Samir Uzzaman, Eshrar Osman, Md. Ahashan Habib, Tanjina Akhtar Banu, Abu Sayeed Mohammad Mahmud, Md. Murshed Hasan Sarkar, Barna Goswami, Iffat Jahan, Md. Saddam Hossain, Tasnim Nafisa, Md. Maruf Ahmed Molla, Mahmuda Yeasmin, Asish Kumar Ghosh, Arifa Akram, A. K. M.Shamsuzzaman, Md. Salim Khan
EPI_ISL_1036752	National Institute of Laboratory Medicine and Referral Center	Genomic Research Lab, BCSIR, Dr. Qudrat-E-Khuda Road, Dhaka 1205, Bangladesh	Tanjina Akhtar Banu, Mohammad Samir Uzzaman, Eshrar Osman, Md. Ahashan Habib, Shahina Akter, Abu Sayeed Mohammad Mahmud, Md. Murshed Hasan Sarkar, Barna Goswami, Iffat Jahan, Md. Saddam Hossain, Tasnim Nafisa, Md. Maruf Ahmed Molla, Mahmuda Yeasmin, Asish Kumar Ghosh, Arifa Akram, A. K. M.Shamsuzzaman, Md. Salim Khan,
EPI_ISL_1038792	US Air Force School of Aerospace Medicine	US Air Force School of Aerospace Medicine	Anthony Fries, Jennifer Meyer, William Gruner, William Buggele, Amanda Javorina, Sarah Purves, Clarise Starr, Elizabeth Macias
EPI_ISL_1039009, EPI_ISL_1039010, EPI_ISL_1039011	WHO National Influenza Centre Russian Federation	WHO National Influenza Centre Russian Federation	Andrey Komissarov, Artem Fadeev, Anna Ivanova, Kseniya Komissarova, Dmitry Bazhenov, Tamila Musaeva, Maria Timofeeva, Veronika Eder, Maria Pisareva, Daria Danilenko, Ksenia Safina, Elena Nabieva, Georgii Bazykin, Dmitry Lioznov
EPI_ISL_1039012, EPI_ISL_1039014, EPI_ISL_1039015, EPI_ISL_1039016, EPI_ISL_1039020, EPI_ISL_1039026, EPI_ISL_1039029, EPI_ISL_1039031, EPI_ISL_1039033, EPI_ISL_1039034, EPI_ISL_1039043, EPI_ISL_1039044, EPI_ISL_1039053, EPI_ISL_1039054, EPI_ISL_1039055			
see above	Kamchatka Regional Children's Infectious Diseases Hospital	WHO National Influenza Centre Russian Federation	Andrey Komissarov, Artem Fadeev, Anna Ivanova, Kseniya Komissarova, Dmitry Bazhenov, Tamila Musaeva, Maria Timofeeva, Veronika Eder, Maria Pisareva, Daria Danilenko, Ksenia Safina, Elena Nabieva, Georgii Bazykin, Dmitry Lioznov
EPI_ISL_1039068	Center of Hygiene and Epidemiology in Belgorod Region	WHO National Influenza Centre Russian Federation	Andrey Komissarov, Artem Fadeev, Lyudmila Berdinskikh, Anna Ivanova, Kseniya Komissarova, Dmitry Bazhenov, Tamila Musaeva, Maria Timofeeva, Veronika Eder, Maria Pisareva, Daria Danilenko, Ksenia Safina, Elena Nabieva, Georgii Bazykin, Dmitry Lioznov
EPI_ISL_1039126, EPI_ISL_1039128, EPI_ISL_1039129, EPI_ISL_1039130, EPI_ISL_1039131, EPI_ISL_1039133, EPI_ISL_1039136	HELIX LLC	WHO National Influenza Centre Russian Federation	Andrey Komissarov, Artem Fadeev, Anna Ivanova, Kseniya Komissarova, Dmitry Bazhenov, Tamila Musaeva, Maria Timofeeva, Veronika Eder, Maria Pisareva, Daria Danilenko, Ksenia Safina, Elena Nabieva, Georgii Bazykin, Dmitry Lioznov
EPI_ISL_1039152	Pathogenic Microorganisms Variability Laboratory	WHO National Influenza Centre Russian Federation	Andrey Komissarov, Artem Fadeev, Anna Ivanova, Kseniya Komissarova, Dmitry Bazhenov, Daria Danilenko, Ksenia Safina, Elena Nabieva, Georgii Bazykin, Nadezhda Kuznetsova, Elena Shidlovskaya, Sergey Alkhovsky, Tatyana Vishnevskaya, Elizaveta Divisenko, Alexey Shchetinin, Maria Nikiforova, Andrey Pochtovyy, Evgeny Usachev, Elena Vokalova, Maxim Rubalsky, Oleg Rubalsky, Artem Tkachuk, Vladimir Gushchin, Alexander Gintsburg, Dmitry Lioznov
EPI_ISL_1039724	Alaska State Virology Laboratory	Alaska State Virology Laboratory	Stephanie DeRonde, Lisa Smith, Ph.D., Jack Chen, Ph.D.
EPI_ISL_1040484	National Institute of Laboratory Medicine and Referral Center	Genomic Research Lab, BCSIR	Shahina Akter, Mohammad Samir Uzzaman, Eshrar Osman, Md. Ahashan Habib, Tanjina Akhtar Banu, Abu Sayeed Mohammad Mahmud, Md. Murshed Hasan Sarkar, Barna Goswami, Iffat Jahan, Md. Saddam Hossain, Tasnim Nafisa, Md. Maruf Ahmed Molla, Mahmuda Yeasmin, Asish Kumar Ghosh, Arifa Akram, A. K. M.Shamsuzzaman, Md. Salim Khan
EPI_ISL_1040485	National Institute of Laboratory Medicine and Referral Center	Genomic Research Lab, BCSIR	Md. Saddam Hossain, Mohammad Samir Uzzaman, Eshrar Osman, Md. Ahashan Habib, Shahina Akter, Tanjina Akhtar Banu, Abu Sayeed Mohammad Mahmud, Md. Murshed Hasan Sarkar, Barna Goswami, Iffat Jahan, Tasnim Nafisa, Md. Maruf Ahmed Molla, Mahmuda Yeasmin, Asish Kumar Ghosh, Arifa Akram, A. K. M.Shamsuzzaman, Md. Salim Khan
EPI_ISL_1040706, EPI_ISL_1040747, EPI_ISL_1040750, EPI_ISL_1040751, EPI_ISL_1040753	Groote Schuur Hospital wc GSH	NHLS/UCT	Arash Iranzadeh, Deelan Doolabh, Lynn Tyers, Bruna Galvao, Innocent Mudau, Marvin Hsiao, Kruger Marais, Diana Hardie, Stephen Korsman, Carolyn Williamson
EPI_ISL_1040762	Bredasdorp clinic	NHLS/UCT	Arash Iranzadeh, Deelan Doolabh, Lynn Tyers, Bruna Galvao, Innocent Mudau, Marvin Hsiao, Kruger Marais, Diana Hardie, Stephen Korsman, Carolyn Williamson
EPI_ISL_1040763	Groote Schuur Hospital wc GSH	NHLS/UCT	Arash Iranzadeh, Deelan Doolabh, Lynn Tyers, Bruna Galvao, Innocent Mudau, Marvin Hsiao, Kruger Marais, Diana Hardie, Stephen Korsman, Carolyn Williamson
EPI_ISL_1047914, EPI_ISL_1047918, EPI_ISL_1047921, EPI_ISL_1047929, EPI_ISL_1047941, EPI_ISL_1047942, EPI_ISL_1047956	University Hospitals Of Leicester NHS Trust and DeepSeq Nottingham	COVID-19 Genomics UK (COG-UK) Consortium	Christopher Holmes, Paul Bird, Thomas Helmer, Karlie Fallon, Julian Tang, Jonathan Ball, Patrick McClure, Joeseph Chappell, Nadine Holmes, Matthew Carlisle, Christopher Moore, Fei Sang, Johnny Debebe, Victoria Wright, Matthew Loose
EPI_ISL_1048652, EPI_ISL_1048653, EPI_ISL_1048654, EPI_ISL_1048655, EPI_ISL_1048656, EPI_ISL_1048657, EPI_ISL_1048658, EPI_ISL_1048660, EPI_ISL_1048661, EPI_ISL_1048662, EPI_ISL_1048663, EPI_ISL_1048664			
see above	Maine HETL	Tewhey Lab, The Jackson Laboratory	Matluk,N., Dewey,H., Iosue,F., Barter,M., Lynch,R., Munger,H. and Tewhey,R.
EPI_ISL_1048807, EPI_ISL_1048810, EPI_ISL_1048811, EPI_ISL_1048813	Laboratory of Clinical Microbiology, Virology and Bioemergencies - Fatebenefratelli Sacco Hospital	Virology Laboratory, Scientific Department, Army Medical Center	Valeria Micheli, Alessandro Mancon, Alberto Rizzo, Maria Rita Gismondo, Silvia Fillo, Anella Monte, Anna Anselmo, Vanessa Vera Fain, Giovanni Faggioni, Riccardo De Sanctis, Florigio Lista
EPI_ISL_1049281, EPI_ISL_1049296, EPI_ISL_1049298, EPI_ISL_1049299, EPI_ISL_1049300, EPI_ISL_1049301, EPI_ISL_1049308, EPI_ISL_1049309, EPI_ISL_1049311, EPI_ISL_1049313, EPI_ISL_1049314, EPI_ISL_1049316, EPI_ISL_1049318, EPI_ISL_1049320, EPI_ISL_1049324, EPI_ISL_1049327, EPI_ISL_1049329, EPI_ISL_1049330, EPI_ISL_1049331, EPI_ISL_1049333, EPI_ISL_1049335, EPI_ISL_1049336			
see above	Innovative Genomics Institute, UC Berkeley	Innovative Genomics Institute, UC Berkeley	Stacia Wyman, Alison Ciling, Netravathi Krishnappa, Haridha Shivram, Phil Frankino, Liana Lareau
EPI_ISL_1049338, EPI_ISL_1049339	Wadsworth Center, New York State Department.of Health	Wadsworth Center, New York State Department.of Health	Kirsten St. George, Daryl M. Lamson, Alexis Russel, Matthew Shudt, Melissa A Leisner, Jonathan Plitnick, Navjot Singh, John Kelly, Erasmus Schneider, Erica Lasek-Nesselquist
EPI_ISL_1049508, EPI_ISL_1049509, EPI_ISL_1049510	Alaska State Virology Laboratory	Alaska State Virology Laboratory	Stephanie DeRonde, Lisa Smith, Ph.D., Jack Chen, Ph.D.
EPI_ISL_1051505, EPI_ISL_1051517	Quadram Institute Bioscience	COVID-19 Genomics UK (COG-UK) Consortium	Dave J. Baker, Gemma L. Kay, Alp Aydin, Thanh Le-Viet, Steven Rudder, Ana P. Tedim, Anastasia Kolyva, Maria Diaz, Leonardo de Oliveira Martins, Nabil-Fareed Alikhan, Lizzie Meadows, Rachael Stanley, Ngozi Elumogo, Muhammed Yasir, Nicholas M. Thomson, Alexander J Trotter, Rachel Gilroy, Samuel Bloomfield, Claire Stuart, Andrew Bell, Reenesh Prakash, Samir Dervisevic, Alison E. Mather, John Wain, Mark Webber, Andrew J. Page, Justin O'Grady
EPI_ISL_1055124, EPI_ISL_1055163, EPI_ISL_1055167	National Virus Reference Laboratory	National Virus Reference Laboratory	Guerrino Macori, Gabriel Gonzalez, Michael Carr, Zoe Yandle, Jonathan Dean, Seamus Fanning, Cillian F De Gascun
EPI_ISL_1055428, EPI_ISL_1055429, EPI_ISL_1055430, EPI_ISL_1055432, EPI_ISL_1055433, EPI_ISL_1055434, EPI_ISL_1055435, EPI_ISL_1055436, EPI_ISL_1055437, EPI_ISL_1055440, EPI_ISL_1055442, EPI_ISL_1055443, EPI_ISL_1055445, EPI_ISL_1055446, EPI_ISL_1055447, EPI_ISL_1055448			
see above	Dr. Leonard A. Miller Centre for Health Services	National Microbiology Laboratory (NML)	Anna Majer, Shari Tyson, Grace Seo, Philip Mabon, Elsie Grudeski, Rhiannon Huzarewich, Russell Mandes, Anneliese Landgraff, Jennifer Tanner, Natalie Knox, Morag Graham, Gary Van Domselaar, Robert Needle, Yang Yu, Adel Malek, Laura Gilbert, George Zahariadis, Nathalie Bastien, Yan Li, Timothy Booth, Darian Hole, Madison Chapel, Kirsten Biggar, Kerri Smith, CanCOGeN's metadata curation team, Public Health Agency of Canada CanCOGeN team
EPI_ISL_1055475, EPI_ISL_1055476, EPI_ISL_1055477, EPI_ISL_1055478, EPI_ISL_1055480, EPI_ISL_1055481, EPI_ISL_1055482, EPI_ISL_1055483, EPI_ISL_1055484, EPI_ISL_1055485, EPI_ISL_1055486, EPI_ISL_1055487, EPI_ISL_1055488, EPI_ISL_1055489, EPI_ISL_1055490, EPI_ISL_1055491, EPI_ISL_1055492, EPI_ISL_1055493, EPI_ISL_1055494, EPI_ISL_1055495, EPI_ISL_1055496, EPI_ISL_1055497, EPI_ISL_1055498, EPI_ISL_1055499, EPI_ISL_1055500, EPI_ISL_1055501, EPI_ISL_1055502, EPI_ISL_1055503, EPI_ISL_1055505, EPI_ISL_1055506, EPI_ISL_1055507, EPI_ISL_1055508, EPI_ISL_1055509, EPI_ISL_1055510, EPI_ISL_1055511, EPI_ISL_1055512, EPI_ISL_1055513, EPI_ISL_1055514, EPI_ISL_1055515, EPI_ISL_1055516, EPI_ISL_1055517, EPI_ISL_1055519, EPI_ISL_1055520, EPI_ISL_1055521, EPI_ISL_1055522, EPI_ISL_1055525, EPI_ISL_1055526, EPI_ISL_1055527, EPI_ISL_1055528, EPI_ISL_1055530, EPI_ISL_1055533, EPI_ISL_1055534, EPI_ISL_1055535, EPI_ISL_1055536, EPI_ISL_1055540, EPI_ISL_1055545, EPI_ISL_1055694, EPI_ISL_1055697, EPI_ISL_1055719, EPI_ISL_1055720, EPI_ISL_1055721, EPI_ISL_1055722			
see above	QEII Health Sciences Centre	National Microbiology Laboratory (NML)	Anna Majer, Shari Tyson, Grace Seo, Philip Mabon, Elsie Grudeski, Rhiannon Huzarewich, Russell Mandes, Anneliese Landgraff, Jennifer Tanner, Natalie

			Knox, Morag Graham, Gary Van Domselaar, Todd Hatchette, Jason LeBlanc, Janice Pettipas, Dan Gaston, Nathalie Bastien, Yan Li, Timothy Booth, Darian Hole, Madison Chapel, Kirsten Biggar, CanCOGeN's metadata curation team, Public Health Agency of Canada CanCOGeN team
EPI_ISL_1055816	Ospedale SS Annunziata Chieti	Istituto Zooprofilattico Sperimentale dell'Abruzzo e Molise "G. Caporale"	Lorusso A, Marcacci M, Di Domenico M, Ancora M, Curini V, Mangone I, Rinaldi A, Delli Compagni E, Scialabba S, Di Pasquale A, Cammà C, Puglia I, Calistri P, Savini G
EPI_ISL_1055819	SIESP DIPARTIMENTO DI PREVENZIONE SULMONA	Istituto Zooprofilattico Sperimentale dell'Abruzzo e Molise "G. Caporale"	Lorusso A, Marcacci M, Di Domenico M, Ancora M, Curini V, Mangone I, Rinaldi A, Delli Compagni E, Scialabba S, Di Pasquale A, Cammà C, Puglia I, Calistri P, Savini G
EPI_ISL_1059921	Dutch COVID-19 response team	National Institute for Public Health and the Environment (RIVM)	Adam Meijer, Harry Vennema, Dirk Eggink, Jeroen Cremer, Sharon van den Brink, Bas van der Veer, AnneMarie van den Brandt, Florian Zwagemaker, Dennis Schmitz, Chantal Reusken, on behalf of the national COVID-19 response team
EPI_ISL_1059946	Wyoming Public Health Laboratory	Wyoming Public Health Laboratory	Jim Mildenberger, Wanda Manley, Noah Hull, Taylor Fearing, Lynette Gumbleton, Channing Weber, Ashley Norberg, Chayse Rowley, Marley Goetz, Brian Dominguez, Elliot Thomasson, Cari Sloma, and Rob Christensen
EPI_ISL_1059984	CONSEJERIA DE SANIDAD Y ASUNTOS SOCIALES	Instituto de Salud Carlos III	Vázquez-Morón,S. Iglesias-Caballero, M. Sandonís,V. Camarero, S. Pozo, F. Casas, I. Jiménez, P. Zaballos, A. Monzón, S. Varona, S. Cuesta, I. Gutiérrez, G.
EPI_ISL_1060031, EPI_ISL_1060202, EPI_ISL_1060205, EPI_ISL_1060213	Wyoming Public Health Laboratory	Wyoming Public Health Laboratory	Jim Mildenberger, Wanda Manley, Noah Hull, Taylor Fearing, Lynette Gumbleton, Channing Weber, Ashley Norberg, Chayse Rowley, Marley Goetz, Brian Dominguez, Elliot Thomasson, Cari Sloma, and Rob Christensen
EPI_ISL_1060612, EPI_ISL_1060614, EPI_ISL_1060615	Instituto Nacional de Medicina Genomica	Instituto Nacional de Medicina Genomica	Hidalgo-Miranda A, Mendoza-Vargas A, Reyes-Grajeda JP, Cedro-Tanda A, Alcaraz N, Gonzalez-Barrera D, Rangel-DeLeon D, Ramirez-Vega O, Sifuentes-Rojas C, Cisneros-Villanueva M, Herrera-Montalvo LA
EPI_ISL_1061304	Alaska State Virology Laboratory	Alaska State Virology Laboratory	Stephanie DeRonde, Lisa Smith, Ph.D., Jack Chen, Ph.D.
EPI_ISL_1061402	Microbiological Diagnostic Unit - Public Health Laboratory (MDU-PHL)	MDU-PHL	Seemann T., Sait, M.L., Sherry, N.L.
EPI_ISL_1063676, EPI_ISL_1063687, EPI_ISL_1063754	Division of Emerging Infectious Diseases, Bureau of Infectious Diseases Diagnosis Control, Korea Disease Control and Prevention Agency	Division of Emerging Infectious Diseases, Bureau of Infectious Diseases Diagnosis Control, Korea Disease Control and Prevention Agency	Ae Kyung Park, Il-Hwan Kim, Heui Man Kim, Jeong-Min Kim, Namjoo Lee, Chaeyoung Lee, Sang Hee Woo, Eun-Jin Kim
EPI_ISL_1063807, EPI_ISL_1063809, EPI_ISL_1063840, EPI_ISL_1063841, EPI_ISL_1063842, EPI_ISL_1063843, EPI_ISL_1063844, EPI_ISL_1063845, EPI_ISL_1063849, EPI_ISL_1063850	REGIONAL VRDL, ICMR-RMRC BBSR	Immunogenomics lab, Institute of Life Sciences, Bhubaneswar	Sunil K. Raghav, Safal Walia, Arup Ghosh, Atimukta Jha, Amol M. Kanampalliwar, Shifu Aggarwal, Kirtal Hansdah, Rupesh Dash, Rajeeb Swain, Punit Prasad, INSACOG Consortium, Ajay Parida
EPI_ISL_1063855	Molecular Laboratory, Vikash Multispeciality Hospital, Bargarh	Immunogenomics lab, Institute of Life Sciences, Bhubaneswar	Sunil K. Raghav, Safal Walia, Arup Ghosh, Atimukta Jha, Amol M. Kanampalliwar, Shifu Aggarwal, Kirtal Hansdah, Rupesh Dash, Rajeeb Swain, Punit Prasad, INSACOG Consortium, Ajay Parida
EPI_ISL_1063891, EPI_ISL_1063892	Immunogenomics lab, Institute of Life Sciences, Bhubaneswar	Immunogenomics lab, Institute of Life Sciences, Bhubaneswar	Sunil K. Raghav, Safal Walia, Arup Ghosh, Atimukta Jha, Amol M. Kanampalliwar, Shifu Aggarwal, Kirtal Hansdah, Rupesh Dash, Rajeeb Swain, Punit Prasad, INSACOG Consortium, Ajay Parida
EPI_ISL_1063897, EPI_ISL_1063898	REGIONAL VRDL, ICMR-RMRC BBSR	Immunogenomics lab, Institute of Life Sciences, Bhubaneswar	Sunil K. Raghav, Safal Walia, Arup Ghosh, Atimukta Jha, Amol M. Kanampalliwar, Shifu Aggarwal, Kirtal Hansdah, Rupesh Dash, Rajeeb Swain, Punit Prasad, INSACOG Consortium, Ajay Parida
EPI_ISL_1064190, EPI_ISL_1064193	Arizona State Public Health Laboratory	Arizona State Public Health Laboratory	Trung Huynh, Jessica Escobar, Katherine Fullerton, Nobuko Fukushima, Stacy White, Linda Getsinger, Victor Waddell
EPI_ISL_1065100, EPI_ISL_1065101, EPI_ISL_1065111, EPI_ISL_1065115, EPI_ISL_1065119, EPI_ISL_1065126, EPI_ISL_1065145, EPI_ISL_1065147, EPI_ISL_1065156, EPI_ISL_1065171, EPI_ISL_1065187, EPI_ISL_1065194, EPI_ISL_1065202, EPI_ISL_1065206, EPI_ISL_1065208, EPI_ISL_1065213, EPI_ISL_1065217, EPI_ISL_1065218, EPI_ISL_1065232, EPI_ISL_1065233, EPI_ISL_1065234, EPI_ISL_1065240, EPI_ISL_1065242, EPI_ISL_1065245, EPI_ISL_1065248, EPI_ISL_1065250, EPI_ISL_1065251, EPI_ISL_1065265, EPI_ISL_1065272, EPI_ISL_1065273, EPI_ISL_1065279, EPI_ISL_1065282, EPI_ISL_1065286, EPI_ISL_1065290, EPI_ISL_1065291, EPI_ISL_1065296, EPI_ISL_1065304, EPI_ISL_1065313, EPI_ISL_1065314, EPI_ISL_1065326, EPI_ISL_1065327, EPI_ISL_1065330, EPI_ISL_1065340, EPI_ISL_1065343, EPI_ISL_1065352, EPI_ISL_1065359, EPI_ISL_1065371, EPI_ISL_1065376, EPI_ISL_1065382, EPI_ISL_1065388, EPI_ISL_1065393, EPI_ISL_1065395, EPI_ISL_1065407, EPI_ISL_1065417, EPI_ISL_1065428, EPI_ISL_1065452, EPI_ISL_1065458, EPI_ISL_1065460, EPI_ISL_1065464, EPI_ISL_1065465, EPI_ISL_1065466, EPI_ISL_1065468, EPI_ISL_1065474, EPI_ISL_1065475, EPI_ISL_1065488, EPI_ISL_1065491, EPI_ISL_1065497, EPI_ISL_1065509, EPI_ISL_1065515, EPI_ISL_1065517, EPI_ISL_1065521, EPI_ISL_1065522, EPI_ISL_1065528, EPI_ISL_1065531, EPI_ISL_1065533, EPI_ISL_1065534, EPI_ISL_1065535, EPI_ISL_1065542, EPI_ISL_1065545, EPI_ISL_1065553, EPI_ISL_1065556, EPI_ISL_1065562, EPI_ISL_1065564, EPI_ISL_1065566, EPI_ISL_1065567, EPI_ISL_1065581, EPI_ISL_1065582, EPI_ISL_1065593, EPI_ISL_1065601, EPI_ISL_1065604, EPI_ISL_1065611, EPI_ISL_1065613, EPI_ISL_1065616, EPI_ISL_1065620, EPI_ISL_1065621, EPI_ISL_1065626, EPI_ISL_1065636, EPI_ISL_1065652, EPI_ISL_1065655, EPI_ISL_1065658, EPI_ISL_1065660, EPI_ISL_1065674, EPI_ISL_1065677, EPI_ISL_1065679, EPI_ISL_1065681, EPI_ISL_1065687, EPI_ISL_1065696, EPI_ISL_1065699, EPI_ISL_1065702, EPI_ISL_1065717, EPI_ISL_1065720, EPI_ISL_1065723, EPI_ISL_1065729, EPI_ISL_1065733, EPI_ISL_1065738, EPI_ISL_1065754, EPI_ISL_1065760, EPI_ISL_1065761, EPI_ISL_1065762, EPI_ISL_1065769, EPI_ISL_1065770, EPI_ISL_1065774, EPI_ISL_1065777, EPI_ISL_1065778, EPI_ISL_1065780, EPI_ISL_1065781, EPI_ISL_1065783, EPI_ISL_1065787, EPI_ISL_1065788, EPI_ISL_1065789, EPI_ISL_1065795, EPI_ISL_1065799, EPI_ISL_1065803, EPI_ISL_1065804, EPI_ISL_1065810, EPI_ISL_1065814, EPI_ISL_1065820, EPI_ISL_1065829, EPI_ISL_1065831, EPI_ISL_1065847, EPI_ISL_1065851, EPI_ISL_1065852, EPI_ISL_1065866, EPI_ISL_1065868, EPI_ISL_1065870, EPI_ISL_1065883, EPI_ISL_1065902, EPI_ISL_1065906, EPI_ISL_1065915, EPI_ISL_1065921, EPI_ISL_1065925, EPI_ISL_1065930, EPI_ISL_1065931, EPI_ISL_1065941, EPI_ISL_1065942, EPI_ISL_1065947, EPI_ISL_1065952, EPI_ISL_1065956, EPI_ISL_1065969, EPI_ISL_1065971, EPI_ISL_1065972, EPI_ISL_1065985, EPI_ISL_1065992, EPI_ISL_1065994, EPI_ISL_1065998, EPI_ISL_1066003, EPI_ISL_1066008, EPI_ISL_1066017, EPI_ISL_1066026, EPI_ISL_1066028, EPI_ISL_1066029, EPI_ISL_1066032, EPI_ISL_1066033, EPI_ISL_1066034, EPI_ISL_1066038, EPI_ISL_1066043, EPI_ISL_1066052, EPI_ISL_1066060, EPI_ISL_1066061, EPI_ISL_1066069, EPI_ISL_1066072, EPI_ISL_1066073, EPI_ISL_1066075, EPI_ISL_1066078, EPI_ISL_1066085, EPI_ISL_1066089, EPI_ISL_1066090, EPI_ISL_1066091, EPI_ISL_1066101, EPI_ISL_1066103, EPI_ISL_1066107, EPI_ISL_1066109, EPI_ISL_1066111, EPI_ISL_1066120, EPI_ISL_1066126, EPI_ISL_1066139, EPI_ISL_1066172, EPI_ISL_1066180, EPI_ISL_1066187, EPI_ISL_1066202, EPI_ISL_1066203, EPI_ISL_1066211, EPI_ISL_1066213, EPI_ISL_1066217, EPI_ISL_1066219, EPI_ISL_1066225, EPI_ISL_1066240, EPI_ISL_1066244, EPI_ISL_1066251, EPI_ISL_1066266, EPI_ISL_1066268, EPI_ISL_1066274, EPI_ISL_1066276, EPI_ISL_1066300, EPI_ISL_1066306, EPI_ISL_1066319, EPI_ISL_1066333, EPI_ISL_1066334, EPI_ISL_1066341, EPI_ISL_1066348, EPI_ISL_1066373, EPI_ISL_1066378, EPI_ISL_1066382, EPI_ISL_1066387, EPI_ISL_1066389, EPI_ISL_1066393, EPI_ISL_1066406, EPI_ISL_1066419, EPI_ISL_1066424, EPI_ISL_1066429, EPI_ISL_1066430, EPI_ISL_1066433, EPI_ISL_1066435, EPI_ISL_1066448, EPI_ISL_1066451, EPI_ISL_1066452, EPI_ISL_1066456, EPI_ISL_1066459, EPI_ISL_1066461, EPI_ISL_1066462, EPI_ISL_1066463, EPI_ISL_1066481, EPI_ISL_1066482, EPI_ISL_1066485, EPI_ISL_1066486, EPI_ISL_1066492, EPI_ISL_1066493, EPI_ISL_1066499, EPI_ISL_1066511, EPI_ISL_1066512, EPI_ISL_1066513, EPI_ISL_1066520, EPI_ISL_1066521, EPI_ISL_1066527, EPI_ISL_1066531, EPI_ISL_1066533, EPI_ISL_1066535, EPI_ISL_1066548, EPI_ISL_1066549, EPI_ISL_1066554, EPI_ISL_1066555, EPI_ISL_1066556, EPI_ISL_1066557, EPI_ISL_1066561, EPI_ISL_1066562, EPI_ISL_1066564, EPI_ISL_1066566, EPI_ISL_1066569, EPI_ISL_1066573, EPI_ISL_1066574, EPI_ISL_1066589, EPI_ISL_1066598, EPI_ISL_1066601, EPI_ISL_1066609, EPI_ISL_1066615, EPI_ISL_1066618, EPI_ISL_1066623, EPI_ISL_1066624, EPI_ISL_1066626, EPI_ISL_1066627, EPI_ISL_1066631, EPI_ISL_1066632, EPI_ISL_1066634, EPI_ISL_1066636, EPI_ISL_1066644, EPI_ISL_1066650, EPI_ISL_1066652, EPI_ISL_1066653, EPI_ISL_1066663, EPI_ISL_1066682, EPI_ISL_1066690, EPI_ISL_1066695, EPI_ISL_1066702, EPI_ISL_1066704, EPI_ISL_1066706, EPI_ISL_1066709, EPI_ISL_1066711, EPI_ISL_1066712, EPI_ISL_1066713, EPI_ISL_1066716, EPI_ISL_1066720, EPI_ISL_1066725, EPI_ISL_1066735, EPI_ISL_1066738, EPI_ISL_1066751, EPI_ISL_1066752			
see above	Department of Virus and Microbiological Special Diagnostics, Statens Serum Institut, Copenhagen, Denmark	Aalborg University	Danish Covid-19 Genome Consortium
EPI_ISL_1066769	Arizona State Public Health Laboratory	Arizona State Public Health Laboratory	Trung Huynh, Jessica Escobar, Katherine Fullerton, Nobuko Fukushima, Stacy White, Linda Getsinger, Victor Waddell
EPI_ISL_1066779, EPI_ISL_1066785, EPI_ISL_1066791, EPI_ISL_1066800, EPI_ISL_1066803, EPI_ISL_1066809, EPI_ISL_1066821, EPI_ISL_1066825, EPI_ISL_1066826, EPI_ISL_1066827, EPI_ISL_1066839, EPI_ISL_1066840, EPI_ISL_1066841, EPI_ISL_1066843, EPI_ISL_1066844, EPI_ISL_1066848, EPI_ISL_1066850, EPI_ISL_1066853, EPI_ISL_1066854, EPI_ISL_1066858, EPI_ISL_1066859, EPI_ISL_1066860, EPI_ISL_1066862, EPI_ISL_1066864, EPI_ISL_1066873, EPI_ISL_1066887, EPI_ISL_1066891, EPI_ISL_1066897, EPI_ISL_1066903, EPI_ISL_1066906, EPI_ISL_1066912, EPI_ISL_1066921, EPI_ISL_1066930, EPI_ISL_1066935, EPI_ISL_1066941, EPI_ISL_1066943, EPI_ISL_1066948, EPI_ISL_1066950, EPI_ISL_1066952, EPI_ISL_1066965, EPI_ISL_1066968, EPI_ISL_1066969, EPI_ISL_1066974, EPI_ISL_1066975, EPI_ISL_1066980, EPI_ISL_1066985, EPI_ISL_1066987, EPI_ISL_1066995, EPI_ISL_1066999, EPI_ISL_1067003, EPI_ISL_1067007, EPI_ISL_1067015, EPI_ISL_1067016, EPI_ISL_1067018, EPI_ISL_1067029, EPI_ISL_1067030, EPI_ISL_1067038, EPI_ISL_1067044, EPI_ISL_1067046, EPI_ISL_1067052, EPI_ISL_1067058, EPI_ISL_1067063, EPI_ISL_1067066, EPI_ISL_1067068, EPI_ISL_1067072, EPI_ISL_1067075, EPI_ISL_1067080, EPI_ISL_1067088, EPI_ISL_1067110, EPI_ISL_1067115, EPI_ISL_1067117, EPI_ISL_1067119, EPI_ISL_1067132, EPI_ISL_1067133, EPI_ISL_1067137, EPI_ISL_1067138, EPI_ISL_1067141, EPI_ISL_1067143, EPI_ISL_1067145, EPI_ISL_1067148, EPI_ISL_1067158, EPI_ISL_1067159, EPI_ISL_1067161, EPI_ISL_1067162, EPI_ISL_1067163, EPI_ISL_1067170, EPI_ISL_1067175, EPI_ISL_1067176, EPI_ISL_1067177, EPI_ISL_1067179, EPI_ISL_1067184, EPI_ISL_1067188, EPI_ISL_1067190, EPI_ISL_1067192, EPI_ISL_1067193, EPI_ISL_1067194, EPI_ISL_1067199, EPI_ISL_1067201, EPI_ISL_1067204, EPI_ISL_1067205, EPI_ISL_1067210, EPI_ISL_1067211, EPI_ISL_1067212, EPI_ISL_1067216, EPI_ISL_1067217, EPI_ISL_1067230, EPI_ISL_1067235, EPI_ISL_1067237, EPI_ISL_1067241, EPI_ISL_1067243, EPI_ISL_1067246, EPI_ISL_1067249, EPI_ISL_1067253, EPI_ISL_1067256, EPI_ISL_1067257, EPI_ISL_1067258, EPI_ISL_1067264, EPI_ISL_1067267, EPI_ISL_1067271, EPI_ISL_1067279, EPI_ISL_1067281, EPI_ISL_1067286, EPI_ISL_1067289, EPI_ISL_1067294, EPI_ISL_1067307, EPI_ISL_1067309, EPI_ISL_1067313, EPI_ISL_1067318, EPI_ISL_1067321, EPI_ISL_1067323, EPI_ISL_1067325, EPI_ISL_1067335, EPI_ISL_1067342, EPI_ISL_1067352, EPI_ISL_1067355, EPI_ISL_1067357, EPI_ISL_1067365, EPI_ISL_1067380, EPI_ISL_1067382, EPI_ISL_1067383, EPI_ISL_1067384, EPI_ISL_1067385, EPI_ISL_1067394, EPI_ISL_1067395, EPI_ISL_1067396, EPI_ISL_1067398, EPI_ISL_1067401, EPI_ISL_1067405, EPI_ISL_1067407, EPI_ISL_1067410, EPI_ISL_1067411, EPI_ISL_1067425, EPI_ISL_1067434, EPI_ISL_1067437, EPI_ISL_1067438, EPI_ISL_1067440, EPI_ISL_1067441, EPI_ISL_1067444, EPI_ISL_1067445, EPI_ISL_1067446, EPI_ISL_1067453, EPI_ISL_1067469, EPI_ISL_1067473, EPI_ISL_1067483, EPI_ISL_1067485, EPI_ISL_1067490, EPI_ISL_1067500, EPI_ISL_1067501, EPI_ISL_1067510, EPI_ISL_1067512, EPI_ISL_1067519, EPI_ISL_1067530, EPI_ISL_1067537, EPI_ISL_1067555, EPI_ISL_1067558, EPI_ISL_1067559, EPI_ISL_1067561, EPI_ISL_1067562, EPI_ISL_1067569, EPI_ISL_1067579			
see above	Department of Virus and Microbiological Special Diagnostics, Statens Serum Institut, Copenhagen, Denmark	Aalborg University	Danish Covid-19 Genome Consortium
EPI_ISL_1067704	Arizona State Public Health Laboratory	Arizona State Public Health Laboratory	Trung Huynh, Jessica Escobar, Katherine Fullerton, Nobuko Fukushima, Stacy White, Linda Getsinger, Victor Waddell
EPI_ISL_1067894, EPI_ISL_1067897, EPI_ISL_1067898, EPI_ISL_1067900, EPI_ISL_1067906, EPI_ISL_1067908, EPI_ISL_1067909, EPI_ISL_1067910, EPI_ISL_1067911, EPI_ISL_1067916	Wisconsin State Laboratory of Hygiene Communicable Disease Division	Wisconsin State Laboratory of Hygiene Communicable Disease Division	Kelsey R. Florek, Abigail C. Shockey
EPI_ISL_1067988, EPI_ISL_1067989, EPI_ISL_1067990, EPI_ISL_1067993, EPI_ISL_1068001, EPI_ISL_1068002, EPI_ISL_1068003, EPI_ISL_1068006, EPI_ISL_1068007, EPI_ISL_1068008, EPI_ISL_1068009, EPI_ISL_1068012, EPI_ISL_1068014, EPI_ISL_1068016			

see above	Clinical Molecular Microbiology Laboratory, UNC Hospital	Dirk Dittmer	Justin T. Landis , Razia Moorad , Brent A. Eason, Melissa B. Miller, Linda Pluta, Dirk Dittmer, Angelica Juarez, Cecilia Thompson, Shawn Hawken, Cameroon Grant, Evelyn Hoffman, Patricio Cano, Jason Wong, Carolina Caro-Vegas, Ryan McNamara, Blossom Damania.
EPI_ISL_1068108, EPI_ISL_1068109, EPI_ISL_1068113, EPI_ISL_1068115, EPI_ISL_1068142, EPI_ISL_1068143, EPI_ISL_1068144, EPI_ISL_1068145, EPI_ISL_1068146, EPI_ISL_1068147, EPI_ISL_1068148, EPI_ISL_1068152, EPI_ISL_1068153, EPI_ISL_1068155, EPI_ISL_1068161, EPI_ISL_1068196, EPI_ISL_1068223, EPI_ISL_1068255, EPI_ISL_1068257			
see above	Laboratorio de Ecologia de Doencas Transmissiveis na Amazonia, Instituto Leonidas e Maria Deane - Fiocruz Amazonia	Laboratorio de Ecologia de Doencas Transmissiveis na Amazonia, Instituto Leonidas e Maria Deane - Fiocruz Amazonia	Valdinete Nascimento, Victor Souza, André Corado, Fernanda Nascimento, George Silva, Ágatha Costa, Debora Duarte, Karina Pessoa, Matilde Mejía, Luciana Gonçalves, Maria Júlia Brandão, Michele Jesus, Felipe Naveca on behalf of the Fiocruz COVID-19 Genomic Surveillance Network
EPI_ISL_1072965	Department of Microbiology, Faculty of Medicine, Universitas Sumatera Utara	Institute of Tropical Disease, Universitas Airlangga; Faculty of Medicine, Universitas Sumatera Utara	Kazufumi Shimizu, Krisnoadi Rahardjo, Aldise M Nastri, Jezzy R Dewantari, Rima R Prasetya, Inke N D Lubis, R Lia Kusumawati, Muhammad Ichwan, Franciscus Ginting, Meliani, Ramadhan Bestari, Mirzan Hasibuan, Irbah R Nainggolan, R Andika D Cahyadi, Yasuko Mori, Soetjipto, Maria I Lusida
EPI_ISL_1072986	Department of Microbiology, Faculty of Medicine, Universitas Sumatera Utara	Institute of Tropical Disease, Universitas Airlangga; Faculty of Medicine, Universitas Sumatera Utara	Krisnoadi Rahardjo, Aldise M Nastri, Jezzy R Dewantari, Rima R Prasetya, Inke N D Lubis, R Lia Kusumawati, Muhammad Ichwan, Franciscus Ginting, Meliani, Ramadhan Bestari, Mirzan Hasibuan, Irbah R Nainggolan, R Andika D Cahyadi, Yasuko Mori, Soetjipto, Kazufumi Shimizu, Maria I Lusida
EPI_ISL_1072988	National Institute of Laboratory Medicine and Referral Center	Genomic Research Lab, BCSIR	Iffat Jahan, Mohammad Samir Uzzaman, Eshrar Osman, Md. Ahashan Habib, Shahina Akter, Tanjina Akhtar Banu, Abu Sayeed Mohammad Mahmud, Md. Murshed Hasan Sarkar, Barna Goswami, Md. Saddam Hossain, Tasnim Nafisa, Md. Maruf Ahmed Molla, Mahmuda Yeasmin, Asish Kumar Ghosh, Arifa Akram, A. K. M.Shamsuzzaman, Md. Salim Khan
EPI_ISL_1074398, EPI_ISL_1075063, EPI_ISL_1075459, EPI_ISL_1075460, EPI_ISL_1075463, EPI_ISL_1075464, EPI_ISL_1075471, EPI_ISL_1075474, EPI_ISL_1075480, EPI_ISL_1075481, EPI_ISL_1075482, EPI_ISL_1075484, EPI_ISL_1075489, EPI_ISL_1075492, EPI_ISL_1075493, EPI_ISL_1075505, EPI_ISL_1075506, EPI_ISL_1075511, EPI_ISL_1075517, EPI_ISL_1075525, EPI_ISL_1075526, EPI_ISL_1075533, EPI_ISL_1075536, EPI_ISL_1075543, EPI_ISL_1075548, EPI_ISL_1075550, EPI_ISL_1075551, EPI_ISL_1075552, EPI_ISL_1075553, EPI_ISL_1075557, EPI_ISL_1075560, EPI_ISL_1075561, EPI_ISL_1075564, EPI_ISL_1075565, EPI_ISL_1075566, EPI_ISL_1075590, EPI_ISL_1075591, EPI_ISL_1075592, EPI_ISL_1075593, EPI_ISL_1075595, EPI_ISL_1075602, EPI_ISL_1075604, EPI_ISL_1075607, EPI_ISL_1075610, EPI_ISL_1075611, EPI_ISL_1075612, EPI_ISL_1075614, EPI_ISL_1075618, EPI_ISL_1075627, EPI_ISL_1075628, EPI_ISL_1075629, EPI_ISL_1075632, EPI_ISL_1075633, EPI_ISL_1075637, EPI_ISL_1075639, EPI_ISL_1075652, EPI_ISL_1075653			
see above	Houston Methodist Hospital	Houston Methodist Hospital	S. Wesley Long, Randall J. Olsen, Paul A. Christensen, Sishir Subedi, Robert Olson, James J. Davis, Matthew Ojeda Saavedra, Prasanti Yerramilli, Layne Pruitt, Kristina Reppond, Madison N. Shyer, Jessica Cambric, Ilya J. Finkelstein, Jimmy Gollihar, and James M. Musser
EPI_ISL_1075657	Instituto Nacional de Medicina Genomica	Instituto Nacional de Medicina Genomica	Hidalgo-Miranda A, Mendoza-Vargas A, Reyes-Grajeda JP, Cedro-Tanda A, Alcaraz N, Gonzalez-Barrera D, Rangel-DeLeon D, Ramirez-Vega O, Sifuentes-Rojas C, Cisneros-Villanueva M, Herrera-Montalvo LA.
EPI_ISL_1075658, EPI_ISL_1075659, EPI_ISL_1075660, EPI_ISL_1075661, EPI_ISL_1075662, EPI_ISL_1075663, EPI_ISL_1075667, EPI_ISL_1075668, EPI_ISL_1075672, EPI_ISL_1075675, EPI_ISL_1075676, EPI_ISL_1075678, EPI_ISL_1075679, EPI_ISL_1075681, EPI_ISL_1075683, EPI_ISL_1075684, EPI_ISL_1075685, EPI_ISL_1075687, EPI_ISL_1075692, EPI_ISL_1075701, EPI_ISL_1075706, EPI_ISL_1075707, EPI_ISL_1075708, EPI_ISL_1075709, EPI_ISL_1075710, EPI_ISL_1075713, EPI_ISL_1075719, EPI_ISL_1075721, EPI_ISL_1075723, EPI_ISL_1075727, EPI_ISL_1075730, EPI_ISL_1075731, EPI_ISL_1075745, EPI_ISL_1075746, EPI_ISL_1075750, EPI_ISL_1075751, EPI_ISL_1075753, EPI_ISL_1075756, EPI_ISL_1075758, EPI_ISL_1075761, EPI_ISL_1075765, EPI_ISL_1075766, EPI_ISL_1075767, EPI_ISL_1075768, EPI_ISL_1075769, EPI_ISL_1075771, EPI_ISL_1075773, EPI_ISL_1075774, EPI_ISL_1075776, EPI_ISL_1075778, EPI_ISL_1075783, EPI_ISL_1075787, EPI_ISL_1075788, EPI_ISL_1075790, EPI_ISL_1075793, EPI_ISL_1075794, EPI_ISL_1075795, EPI_ISL_1075804, EPI_ISL_1075805, EPI_ISL_1075811, EPI_ISL_1075812, EPI_ISL_1075813, EPI_ISL_1075814, EPI_ISL_1075816, EPI_ISL_1075824, EPI_ISL_1075825, EPI_ISL_1075826, EPI_ISL_1075830, EPI_ISL_1075834, EPI_ISL_1075836, EPI_ISL_1075837, EPI_ISL_1075839, EPI_ISL_1075840, EPI_ISL_1075841, EPI_ISL_1075844, EPI_ISL_1077080, EPI_ISL_1077084, EPI_ISL_1077091, EPI_ISL_1077092, EPI_ISL_1077093, EPI_ISL_1077095, EPI_ISL_1077101, EPI_ISL_1077105, EPI_ISL_1077109, EPI_ISL_1077114, EPI_ISL_1077118, EPI_ISL_1077124, EPI_ISL_1077135, EPI_ISL_1077137, EPI_ISL_1077142, EPI_ISL_1077143, EPI_ISL_1077144, EPI_ISL_1077146, EPI_ISL_1077147, EPI_ISL_1077149, EPI_ISL_1077150, EPI_ISL_1077153, EPI_ISL_1077156, EPI_ISL_1077165, EPI_ISL_1077166, EPI_ISL_1077170, EPI_ISL_1077175, EPI_ISL_1077177, EPI_ISL_1077178, EPI_ISL_1077179, EPI_ISL_1077180, EPI_ISL_1077184, EPI_ISL_1077186, EPI_ISL_1077189, EPI_ISL_1077190, EPI_ISL_1077192, EPI_ISL_1077195, EPI_ISL_1077196, EPI_ISL_1077199, EPI_ISL_1077201, EPI_ISL_1077202, EPI_ISL_1077203, EPI_ISL_1077205, EPI_ISL_1077207, EPI_ISL_1077209, EPI_ISL_1077210, EPI_ISL_1077211, EPI_ISL_1077216, EPI_ISL_1077217, EPI_ISL_1077221, EPI_ISL_1077223, EPI_ISL_1077224, EPI_ISL_1077225, EPI_ISL_1077230, EPI_ISL_1077231, EPI_ISL_1077235, EPI_ISL_1077238, EPI_ISL_1077240, EPI_ISL_1077244, EPI_ISL_1077246, EPI_ISL_1077250, EPI_ISL_1077254, EPI_ISL_1077255, EPI_ISL_1077256, EPI_ISL_1077257, EPI_ISL_1077258, EPI_ISL_1077259, EPI_ISL_1077262, EPI_ISL_1077263, EPI_ISL_1077264, EPI_ISL_1077265, EPI_ISL_1077267, EPI_ISL_1077268, EPI_ISL_1077269, EPI_ISL_1077270, EPI_ISL_1077275, EPI_ISL_1077290, EPI_ISL_1077297, EPI_ISL_1077306, EPI_ISL_1077311, EPI_ISL_1077315, EPI_ISL_1077319, EPI_ISL_1077323, EPI_ISL_1077333, EPI_ISL_1077339, EPI_ISL_1077340, EPI_ISL_1077342, EPI_ISL_1077343, EPI_ISL_1077344, EPI_ISL_1077358, EPI_ISL_1077360, EPI_ISL_1077369, EPI_ISL_1077376, EPI_ISL_1077378, EPI_ISL_1077381, EPI_ISL_1077385, EPI_ISL_1077396, EPI_ISL_1077401, EPI_ISL_1077403, EPI_ISL_1077419, EPI_ISL_1077421, EPI_ISL_1077428, EPI_ISL_1077440, EPI_ISL_1077442, EPI_ISL_1077447, EPI_ISL_1077455, EPI_ISL_1077463			
see above	Houston Methodist Hospital	Houston Methodist Hospital	S. Wesley Long, Randall J. Olsen, Paul A. Christensen, Sishir Subedi, Robert Olson, James J. Davis, Matthew Ojeda Saavedra, Prasanti Yerramilli, Layne Pruitt, Kristina Reppond, Madison N. Shyer, Jessica Cambric, Ilya J. Finkelstein, Jimmy Gollihar, and James M. Musser
EPI_ISL_1081151	Baylor Scott & White-Temple	Baylor Scott & White-Temple	Ari Rao, Linden Morales, Kimberly Walker, Marcus Volz, Shelby Hendrickson
EPI_ISL_1081170	IEH Laboratories & Consulting Group	ID Genomics	Veronika Tchesnokova, Kirthi Kutumbaka, Kai Deng, Hemantha Kulakesara, Lydia Larson, Hao Geng, Curtis Fowler, Mansour Samadpour, Evgeni Sokurenko
EPI_ISL_1081185, EPI_ISL_1081187, EPI_ISL_1081188	Baylor Scott & White-Temple	Baylor Scott & White-Temple	Ari Rao, Linden Morales, Kimberly Walker, Marcus Volz, Shelby Hendrickson
EPI_ISL_1081201	Laboratory Corporation of America	Respiratory Viruses Branch, Division of Viral Diseases, Centers for Disease Control and Prevention	Peter W. Cook, Dakota Howard, Dhwani Batra, Ben L. Rambo-Martin, Eileen de Feo, Jan Antico, Christine Tran, Matthew Tolentino, Shannon Wickline, Kim Gietzen, Brad Sickler, Jingtao Liu, Eric Allen, Phil Febbo, Summer Galloway, Nicole L. Washington, Simon White, Geraint Levan, Kelly Schiabor Barrett, Elizabeth Cirulli, Alexandre Bolze, Ary Ascencio, Charlotte Rivera-Garcia, Ryan Cho, Jason Nguyen, Sherry Wang, Jimmy Ramirez, Tyler Cassens, Efren Sandoval, Magnus Isaksson, William Lee, David Becker, Marc Laurent, James Lu, Clinton R. Paden, Suxiang Tong, Duncan MacCannell
EPI_ISL_1081424, EPI_ISL_1081425, EPI_ISL_1081426, EPI_ISL_1081429, EPI_ISL_1081431, EPI_ISL_1081435, EPI_ISL_1081436, EPI_ISL_1081437, EPI_ISL_1081439, EPI_ISL_1081441, EPI_ISL_1081444, EPI_ISL_1081451, EPI_ISL_1081453, EPI_ISL_1081454			
see above	Genomica Lab Molecular, M©xico	Andersen lab at Scripps Research	SEARCH Alliance San Diego with Jonathan Gonzalez Garcia, Jose Roman Chavez Mendez, Jose Horacio Reyna Verdugo, Martin Gonzalez Ibarra, Luis Alberto Rangel Gonzalez
EPI_ISL_1081457, EPI_ISL_1081458	San Diego County Public Health Laboratory	Andersen lab at Scripps Research	SEARCH Alliance San Diego with Tracy Basler, Jovan Shephard, Brett Austin
EPI_ISL_1081476, EPI_ISL_1081477, EPI_ISL_1081478, EPI_ISL_1081479, EPI_ISL_1081480, EPI_ISL_1081481, EPI_ISL_1081482, EPI_ISL_1081483, EPI_ISL_1081484, EPI_ISL_1081489	Genomica Lab Molecular, M©xico	Andersen lab at Scripps Research	SEARCH Alliance San Diego with Jonathan Gonzalez Garcia, Jose Roman Chavez Mendez, Jose Horacio Reyna Verdugo, Martin Gonzalez Ibarra, Luis Alberto Rangel Gonzalez
EPI_ISL_1081954	"Stefan S. Nicolau"Institute of Virology	"Stefan S. Nicolau"Institute of Virology	Anca Botezatu, Iulia Virginia Iancu, Coralia Bleotu, Mihaela Economescu, Laura Necula, Denisa Dragu, Lilia Matei, Ioana Pitica, Marinela Bostan, Mirela Mihaila, Laura Grecu, Camelia Sultana, Simona Ruta, Ana Iulia Neagu, Adriana Plesa, Saviana Nedeianu, Alina Nastasie, Cristina Mambet, Gabriela Anton, Carmen Cristina Diaconu.
EPI_ISL_1081958, EPI_ISL_1082290, EPI_ISL_1082291, EPI_ISL_1082294	"Stefan S. Nicolau"Institute of Virology	"Stefan S. Nicolau"Institute of Virology	Anca Botezatu, Iulia Virginia Iancu, Coralia Bleotu, Mihaela Economescu, Laura Necula, Denisa Dragu, Lilia Matei, Ioana Pitica, Marinela Bostan, Mirela Mihaila, Laura Grecu, Camelia Sultana, Simona Ruta, Ana Iulia Neagu, Adriana Plesa, Saviana Nedeianu, Alina Nastasie, Cristina Mambet,Gabriela Anton, Carmen Cristina Diaconu
EPI_ISL_1082296	"Stefan S. Nicolau"Institute of Virology	285 Mihai Bravu Ave, Bucharest, Romania	Anca Botezatu, Iulia Virginia Iancu, Coralia Bleotu, Mihaela Economescu, Laura Necula, Denisa Dragu, Lilia Matei, Ioana Pitica, Marinela Bostan, Mirela Mihaila, Laura Grecu, Camelia Sultana, Simona Ruta, Ana Iulia Neagu, Adriana Plesa, Saviana Nedeianu, Alina Nastasie, Cristina Mambet,Gabriela Anton, Carmen Cristina Diaconu
EPI_ISL_1082299, EPI_ISL_1082300, EPI_ISL_1082301, EPI_ISL_1082302, EPI_ISL_1082306, EPI_ISL_1082307, EPI_ISL_1082309, EPI_ISL_1082311, EPI_ISL_1082314, EPI_ISL_1082315	Laboratory of Virology of Federal Budget Health Care Institution "Center of Hygiene and Epidemiology in Arkhangelsk region"	Group of Genomics and Postgenomic Technologies of Central Research Institute of Epidemiology	Samoilov AE, Kaptelova VV, Korneenko EV, Valdokhina AV, Bulanenko VP, Saenko SS, Semyina LV,Ponomareva YA,Shishko LA, Cherkashina AS, Golubeva AG, Speranskaya AS, Tivanova EV, Kondrasheva LV, Akimkin VG
EPI_ISL_1085534, EPI_ISL_1085674	Hôpital Henri Mondor	Department of Virology, Henri Mondor University Hospital, Assistance Publique Hôpitaux de Paris, Université Paris-Est Créteil, INSERM U955	Christophe Rodriguez, Slim Fourati, Vanessa Demontant, Guillaume Gricourt, Melissa N'Debi, Alexandre Soulier, Elisabeth Trawinski, Jean-Michel Pawlotsky
EPI_ISL_1086375, EPI_ISL_1086376	LACEN - Laboratório Central de Saúde Pública do Rio Grande do Norte	Evandro Chagas Institute	Santos, M.C.; Silva, A.M.; Junior, W.D.C.; Barbagelata, L.S.; Ferreira, J.A.; Sousa, E.M.A.; da Silva, P.S.; Pinheiro, K.C.; L.C.; Sousa Junior, E.C.
EPI_ISL_1086377	LACEN - Laboratório Central de Saúde Pública do Paraiba	Evandro Chagas Institute	Santos, M.C.; Silva, A.M.; Junior, W.D.C.; Barbagelata, L.S.; Ferreira, J.A.; Sousa, E.M.A.; da Silva, P.S.; Pinheiro, K.C.; L.C.; Sousa Junior, E.C.
EPI_ISL_1092124, EPI_ISL_1092256, EPI_ISL_1092258, EPI_ISL_1092279, EPI_ISL_1092286, EPI_ISL_1092290, EPI_ISL_1092291, EPI_ISL_1092293, EPI_ISL_1092294, EPI_ISL_1092297, EPI_ISL_1092304, EPI_ISL_1092321			

see above	Protzer Lab	Protzer Lab, Laboratory for Functional Genome Analysis, Dept. Genomics, Gene Center of the LMU Munich	Ulrike Protzer, Dieter Hoffmann, Till Bunse, Eva C.Schulte, Elisabeth Esser, Stefan Krebs, Alexander Graf, Helmut Blum
EPI_ISL_1093435	Nigerian Centre for Disease Control (NCDC)	African Centre of Excellence for Genomics of Infectious Diseases (ACEGID), Redeemer's University	Olawoye, I.B., et al
EPI_ISL_1094252	OK Public Health Laboratory, Oklahoma State DOH	Respiratory Viruses Branch, Division of Viral Diseases, Centers for Disease Control and Prevention	Krista Queen, Yan Li, Ying Tao, Jing Zhang, Anna Uehara, Anna Montmayeur, Clinton R. Paden, Peter W. Cook, Rachel Marine, Mili Sheth, Jasmine Padilla, Sarah Nobles, Mark Burroughs, Lori Rowe, Haibin Wang, Ben L. Rambo-Martin, Dhwani Batra, Justin Lee, Suxiang Tong
EPI_ISL_1094314	OR State PHL-Virology/Immunology Section	Respiratory Viruses Branch, Division of Viral Diseases, Centers for Disease Control and Prevention	Krista Queen, Yan Li, Ying Tao, Jing Zhang, Anna Uehara, Anna Montmayeur, Clinton R. Paden, Peter W. Cook, Rachel Marine, Mili Sheth, Jasmine Padilla, Sarah Nobles, Mark Burroughs, Lori Rowe, Haibin Wang, Ben L. Rambo-Martin, Dhwani Batra, Justin Lee, Suxiang Tong
EPI_ISL_1094340	OK Public Health Laboratory, Oklahoma State DOH	Respiratory Viruses Branch, Division of Viral Diseases, Centers for Disease Control and Prevention	Krista Queen, Yan Li, Ying Tao, Jing Zhang, Anna Uehara, Anna Montmayeur, Clinton R. Paden, Peter W. Cook, Rachel Marine, Mili Sheth, Jasmine Padilla, Sarah Nobles, Mark Burroughs, Lori Rowe, Haibin Wang, Ben L. Rambo-Martin, Dhwani Batra, Justin Lee, Suxiang Tong
EPI_ISL_1094375, EPI_ISL_1094376	DE Public Health Laboratory	Respiratory Viruses Branch, Division of Viral Diseases, Centers for Disease Control and Prevention	Krista Queen, Yan Li, Ying Tao, Jing Zhang, Anna Uehara, Anna Montmayeur, Clinton R. Paden, Peter W. Cook, Rachel Marine, Mili Sheth, Jasmine Padilla, Sarah Nobles, Mark Burroughs, Lori Rowe, Haibin Wang, Ben L. Rambo-Martin, Dhwani Batra, Justin Lee, Suxiang Tong
EPI_ISL_1094473, EPI_ISL_1094475	CT-Dr. Katherine A. Kelley State Public Health Lab	Respiratory Viruses Branch, Division of Viral Diseases, Centers for Disease Control and Prevention	Krista Queen, Yan Li, Ying Tao, Jing Zhang, Anna Uehara, Anna Montmayeur, Clinton R. Paden, Peter W. Cook, Rachel Marine, Mili Sheth, Jasmine Padilla, Sarah Nobles, Mark Burroughs, Lori Rowe, Haibin Wang, Ben L. Rambo-Martin, Dhwani Batra, Justin Lee, Suxiang Tong
EPI_ISL_1094600	MN PHL Division, Minnesota Department of Health	Respiratory Viruses Branch, Division of Viral Diseases, Centers for Disease Control and Prevention	Krista Queen, Yan Li, Ying Tao, Jing Zhang, Anna Uehara, Anna Montmayeur, Clinton R. Paden, Peter W. Cook, Rachel Marine, Mili Sheth, Jasmine Padilla, Sarah Nobles, Mark Burroughs, Lori Rowe, Haibin Wang, Ben L. Rambo-Martin, Dhwani Batra, Justin Lee, Suxiang Tong
EPI_ISL_1094628, EPI_ISL_1094633, EPI_ISL_1094637, EPI_ISL_1094638	DE Public Health Laboratory	Respiratory Viruses Branch, Division of Viral Diseases, Centers for Disease Control and Prevention	Krista Queen, Yan Li, Ying Tao, Jing Zhang, Anna Uehara, Anna Montmayeur, Clinton R. Paden, Peter W. Cook, Rachel Marine, Mili Sheth, Jasmine Padilla, Sarah Nobles, Mark Burroughs, Lori Rowe, Haibin Wang, Ben L. Rambo-Martin, Dhwani Batra, Justin Lee, Suxiang Tong
EPI_ISL_1094645	KS Health and Environmental Laboratories	Respiratory Viruses Branch, Division of Viral Diseases, Centers for Disease Control and Prevention	Krista Queen, Yan Li, Ying Tao, Jing Zhang, Anna Uehara, Anna Montmayeur, Clinton R. Paden, Peter W. Cook, Rachel Marine, Mili Sheth, Jasmine Padilla, Sarah Nobles, Mark Burroughs, Lori Rowe, Haibin Wang, Ben L. Rambo-Martin, Dhwani Batra, Justin Lee, Suxiang Tong
EPI_ISL_1094686	CT-Dr. Katherine A. Kelley State Public Health Lab	Respiratory Viruses Branch, Division of Viral Diseases, Centers for Disease Control and Prevention	Krista Queen, Yan Li, Ying Tao, Jing Zhang, Anna Uehara, Anna Montmayeur, Clinton R. Paden, Peter W. Cook, Rachel Marine, Mili Sheth, Jasmine Padilla, Sarah Nobles, Mark Burroughs, Lori Rowe, Haibin Wang, Ben L. Rambo-Martin, Dhwani Batra, Justin Lee, Suxiang Tong
EPI_ISL_1094727	OR State PHL-Virology/Immunology Section	Respiratory Viruses Branch, Division of Viral Diseases, Centers for Disease Control and Prevention	Krista Queen, Yan Li, Ying Tao, Jing Zhang, Anna Uehara, Anna Montmayeur, Clinton R. Paden, Peter W. Cook, Rachel Marine, Mili Sheth, Jasmine Padilla, Sarah Nobles, Mark Burroughs, Lori Rowe, Haibin Wang, Ben L. Rambo-Martin, Dhwani Batra, Justin Lee, Suxiang Tong
EPI_ISL_1094772	HI Dept. of Health, State Laboratories Division	Respiratory Viruses Branch, Division of Viral Diseases, Centers for Disease Control and Prevention	Krista Queen, Yan Li, Ying Tao, Jing Zhang, Anna Uehara, Anna Montmayeur, Clinton R. Paden, Peter W. Cook, Rachel Marine, Mili Sheth, Jasmine Padilla, Sarah Nobles, Mark Burroughs, Lori Rowe, Haibin Wang, Ben L. Rambo-Martin, Dhwani Batra, Justin Lee, Suxiang Tong
EPI_ISL_1094800, EPI_ISL_1094808, EPI_ISL_1094812	OK Public Health Laboratory, Oklahoma State DOH	Respiratory Viruses Branch, Division of Viral Diseases, Centers for Disease Control and Prevention	Krista Queen, Yan Li, Ying Tao, Jing Zhang, Anna Uehara, Anna Montmayeur, Clinton R. Paden, Peter W. Cook, Rachel Marine, Mili Sheth, Jasmine Padilla, Sarah Nobles, Mark Burroughs, Lori Rowe, Haibin Wang, Ben L. Rambo-Martin, Dhwani Batra, Justin Lee, Suxiang Tong
EPI_ISL_1094842, EPI_ISL_1094845	UT-Unified State Labs: Public Health Utah DOH	Respiratory Viruses Branch, Division of Viral Diseases, Centers for Disease Control and Prevention	Krista Queen, Yan Li, Ying Tao, Jing Zhang, Anna Uehara, Anna Montmayeur, Clinton R. Paden, Peter W. Cook, Rachel Marine, Mili Sheth, Jasmine Padilla, Sarah Nobles, Mark Burroughs, Lori Rowe, Haibin Wang, Ben L. Rambo-Martin, Dhwani Batra, Justin Lee, Suxiang Tong
EPI_ISL_1094888, EPI_ISL_1094891, EPI_ISL_1094892, EPI_ISL_1094893, EPI_ISL_1094894, EPI_ISL_1094896	AL Dept. of Public Health Bureau of Clinical Laboratories	Respiratory Viruses Branch, Division of Viral Diseases, Centers for Disease Control and Prevention	Krista Queen, Yan Li, Ying Tao, Jing Zhang, Anna Uehara, Anna Montmayeur, Clinton R. Paden, Peter W. Cook, Rachel Marine, Mili Sheth, Jasmine Padilla, Sarah Nobles, Mark Burroughs, Lori Rowe, Haibin Wang, Ben L. Rambo-Martin, Dhwani Batra, Justin Lee, Suxiang Tong
EPI_ISL_1094907, EPI_ISL_1094917	ND Dept. of Health Laboratory Services-Microbiology	Respiratory Viruses Branch, Division of Viral Diseases, Centers for Disease Control and Prevention	Krista Queen, Yan Li, Ying Tao, Jing Zhang, Anna Uehara, Anna Montmayeur, Clinton R. Paden, Peter W. Cook, Rachel Marine, Mili Sheth, Jasmine Padilla, Sarah Nobles, Mark Burroughs, Lori Rowe, Haibin Wang, Ben L. Rambo-Martin, Dhwani Batra, Justin Lee, Suxiang Tong
EPI_ISL_1094936, EPI_ISL_1094941	MT Public Health Laboratory	Respiratory Viruses Branch, Division of Viral Diseases, Centers for Disease Control and Prevention	Krista Queen, Yan Li, Ying Tao, Jing Zhang, Anna Uehara, Anna Montmayeur, Clinton R. Paden, Peter W. Cook, Rachel Marine, Mili Sheth, Jasmine Padilla, Sarah Nobles, Mark Burroughs, Lori Rowe, Haibin Wang, Ben L. Rambo-Martin, Dhwani Batra, Justin Lee, Suxiang Tong
EPI_ISL_1094965, EPI_ISL_1094967, EPI_ISL_1094972	OH Department of Health Laboratory	Respiratory Viruses Branch, Division of Viral Diseases, Centers for Disease Control and Prevention	Krista Queen, Yan Li, Ying Tao, Jing Zhang, Anna Uehara, Anna Montmayeur, Clinton R. Paden, Peter W. Cook, Rachel Marine, Mili Sheth, Jasmine Padilla, Sarah Nobles, Mark Burroughs, Lori Rowe, Haibin Wang, Ben L. Rambo-Martin, Dhwani Batra, Justin Lee, Suxiang Tong
EPI_ISL_1094993, EPI_ISL_1094994, EPI_ISL_1094995, EPI_ISL_1094996, EPI_ISL_1094997, EPI_ISL_1094998, EPI_ISL_1094999, EPI_ISL_1095000, EPI_ISL_1095001, EPI_ISL_1095003, EPI_ISL_1095004, EPI_ISL_1095005, EPI_ISL_1095006, EPI_ISL_1095007, EPI_ISL_1095008, EPI_ISL_1095011, EPI_ISL_1095012, EPI_ISL_1095014, EPI_ISL_1095016, EPI_ISL_1095017, EPI_ISL_1095019, EPI_ISL_1095020, EPI_ISL_1095021, EPI_ISL_1095022, EPI_ISL_1095023, EPI_ISL_1095024, EPI_ISL_1095025, EPI_ISL_1095026, EPI_ISL_1095027, EPI_ISL_1095028, EPI_ISL_1095029, EPI_ISL_1095031, EPI_ISL_1095032, EPI_ISL_1095034, EPI_ISL_1095035, EPI_ISL_1095037, EPI_ISL_1095039			
see above	MD DOH Laboratories Administration	Respiratory Viruses Branch, Division of Viral Diseases, Centers for Disease Control and Prevention	Krista Queen, Yan Li, Ying Tao, Jing Zhang, Anna Uehara, Anna Montmayeur, Clinton R. Paden, Peter W. Cook, Rachel Marine, Mili Sheth, Jasmine Padilla, Sarah Nobles, Mark Burroughs, Lori Rowe, Haibin Wang, Ben L. Rambo-Martin, Dhwani Batra, Justin Lee, Suxiang Tong
EPI_ISL_1095239, EPI_ISL_1095241	OR State PHL-Virology/Immunology Section	Respiratory Viruses Branch, Division of Viral Diseases, Centers for Disease Control and Prevention	Krista Queen, Yan Li, Ying Tao, Jing Zhang, Anna Uehara, Anna Montmayeur, Clinton R. Paden, Peter W. Cook, Rachel Marine, Mili Sheth, Jasmine Padilla, Sarah Nobles, Mark Burroughs, Lori Rowe, Haibin Wang, Ben L. Rambo-Martin, Dhwani Batra, Justin Lee, Suxiang Tong
EPI_ISL_1095325, EPI_ISL_1095326, EPI_ISL_1095327, EPI_ISL_1095329, EPI_ISL_1095330, EPI_ISL_1095331, EPI_ISL_1095332	MD DOH Laboratories Administration	Respiratory Viruses Branch, Division of Viral Diseases, Centers for Disease Control and Prevention	Krista Queen, Yan Li, Ying Tao, Jing Zhang, Anna Uehara, Anna Montmayeur, Clinton R. Paden, Peter W. Cook, Rachel Marine, Mili Sheth, Jasmine Padilla, Sarah Nobles, Mark Burroughs, Lori Rowe, Haibin Wang, Ben L. Rambo-Martin, Dhwani Batra, Justin Lee, Suxiang Tong
EPI_ISL_1097386, EPI_ISL_1097387, EPI_ISL_1097388, EPI_ISL_1097390, EPI_ISL_1097391, EPI_ISL_1097392, EPI_ISL_1097393, EPI_ISL_1097395, EPI_ISL_1097396, EPI_ISL_1097400, EPI_ISL_1097401, EPI_ISL_1097402, EPI_ISL_1097403, EPI_ISL_1097405, EPI_ISL_1097406, EPI_ISL_1097407, EPI_ISL_1097409, EPI_ISL_1097412, EPI_ISL_1097413, EPI_ISL_1097415, EPI_ISL_1097417, EPI_ISL_1097418, EPI_ISL_1097419, EPI_ISL_1097420, EPI_ISL_1097421, EPI_ISL_1097422, EPI_ISL_1097423, EPI_ISL_1097424, EPI_ISL_1097425, EPI_ISL_1097426, EPI_ISL_1097427, EPI_ISL_1097428, EPI_ISL_1097429, EPI_ISL_1097430, EPI_ISL_1097431, EPI_ISL_1097432, EPI_ISL_1097434, EPI_ISL_1097435, EPI_ISL_1097436, EPI_ISL_1097437, EPI_ISL_1097438, EPI_ISL_1097439, EPI_ISL_1097440			
see above	Florida Bureau of Public Health Laboratories	Florida Bureau of Public Health Laboratories	Sarah Schmedes, Jason Blanton
EPI_ISL_1098754	Fukuoka Institute of Health and Environmental Sciences	Fukuoka Institute of Health and Environmental Sciences	Asako Nakamura, Yoshiki Etoh, Hiroaki Shigemura, Akira Ohishi, Takayuki Kobayashi, Saori Ueda, Chiharu Katamune, Mitsuhiro Hamasaki, Yuki Ashizuka, Tsuyoshi Sekizuka, Kentaro Itokawa, Rina Tanaka, Masanori Hashino, Makoto Kuroda
EPI_ISL_1104254, EPI_ISL_1104258	Virology Department, Royal Infirmary of Edinburgh, NHS Lothian / School of Biological Sciences, University of Edinburgh	COVID-19 Genomics UK (COG-UK) Consortium	McHugh M, Dewar R, Cotton S, Rooke S, O'Toole Á, Scher E, Hill V, McCrone JT, Colquhoun R, Yu X, Jackson B, Rambaut A, Templeton K
EPI_ISL_1104834, EPI_ISL_1104888, EPI_ISL_1104893, EPI_ISL_1104904, EPI_ISL_1104912, EPI_ISL_1104913, EPI_ISL_1104927, EPI_ISL_1104937, EPI_ISL_1104938, EPI_ISL_1104951, EPI_ISL_1104958, EPI_ISL_1104969, EPI_ISL_1104973, EPI_ISL_1104975, EPI_ISL_1104977, EPI_ISL_1104979, EPI_ISL_1104982, EPI_ISL_1104984, EPI_ISL_1104986, EPI_ISL_1104987, EPI_ISL_1104989, EPI_ISL_1104990, EPI_ISL_1104991, EPI_ISL_1104992, EPI_ISL_1104993, EPI_ISL_1104996, EPI_ISL_1104999, EPI_ISL_1105000, EPI_ISL_1105002, EPI_ISL_1105005, EPI_ISL_1105006, EPI_ISL_1105009, EPI_ISL_1105010, EPI_ISL_1105011, EPI_ISL_1105016, EPI_ISL_1105018, EPI_ISL_1105019, EPI_ISL_1105027, EPI_ISL_1105037, EPI_ISL_1105056, EPI_ISL_1105103, EPI_ISL_1105104, EPI_ISL_1105116, EPI_ISL_1105121, EPI_ISL_1105122, EPI_ISL_1105127, EPI_ISL_1105128, EPI_ISL_1105140, EPI_ISL_1105261, EPI_ISL_1105302			
see above	University College London Hospital	COVID-19 Genomics UK (COG-UK) Consortium	Judith Heaney, Matthew Byott, Catherine Houlihan, Dan Frampton, Stuart Kirk, Moira Spyer and Eleni Nastouli
EPI_ISL_1109653, EPI_ISL_1109656, EPI_ISL_1109658, EPI_ISL_1109659, EPI_ISL_1109667, EPI_ISL_1109668, EPI_ISL_1109672, EPI_ISL_1109674, EPI_ISL_1109676, EPI_ISL_1109681, EPI_ISL_1109684, EPI_ISL_1109689, EPI_ISL_1109693, EPI_ISL_1109695, EPI_ISL_1109707, EPI_ISL_1109712			
see above	Servicio de Microbiología. Hospital Clínico Universitario de Valencia	SeqCOVID-SPAIN consortium/IBV(CSIC)	David Navarro Ortega, Eliseo Albert Vicent, Ignacio Torres and SeqCOVID-SPAIN consortium
EPI_ISL_1109981, EPI_ISL_1109983, EPI_ISL_1110006	MEPHI, Aix Marseille University	MEPHI, Aix Marseille University	Anthony LEVASSEUR
EPI_ISL_1110291	Baylor Scott & White-Temple	Baylor Scott & White-Temple	Ari Rao, Linden Morales, Kimberly Walker, Marcus Volz, Shelby Hendrickson
EPI_ISL_1110410, EPI_ISL_1110411, EPI_ISL_1110412, EPI_ISL_1110435,	Hopital	National Reference Center for Viruses of Respiratory Infections, Institut Pasteur, Paris	Marion Barbet, Sylvie Behillil, Méline Bizard, Angela Brisebarre, Camille Capel, Etienne Simon-Lorière, Vincent Enouf, Maud Vanpeene, Sylvie van der Werf,Pilorge LéA

EPI_ISL_1110492, EPI_ISL_1110493, EPI_ISL_1110494			
EPI_ISL_1110844	Virginia Division of Consolidated Laboratory Services	Virginia Division of Consolidated Laboratory Services	Virginia DCLS
EPI_ISL_1111133, EPI_ISL_1111134, EPI_ISL_1111135, EPI_ISL_1111137, EPI_ISL_1111143, EPI_ISL_1111144, EPI_ISL_1111146, EPI_ISL_1111148, EPI_ISL_1111149, EPI_ISL_1111150, EPI_ISL_1111151, EPI_ISL_1111152, EPI_ISL_1111154, EPI_ISL_1111155, EPI_ISL_1111158, EPI_ISL_1111314, EPI_ISL_1111315, EPI_ISL_1111463, EPI_ISL_1111464, EPI_ISL_1111467, EPI_ISL_1111476, EPI_ISL_1111480, EPI_ISL_1111482, EPI_ISL_1111483, EPI_ISL_1111495, EPI_ISL_1111502			
see above	Laboratorio de Referencia Nacional de Virus Respiratorio. Instituto Nacional de Salud Perú	Laboratorio de Referencia Nacional de Enteropatógenos. Instituto Nacional de Salud del Perú	Ronnie Gavilan Chavez, Junior Caro Castro, Willi Quino Sifuentes, Veronica Hurtado Vela, Iris Silva Molina, Fiorella Orellana Peralta
EPI_ISL_1111508, EPI_ISL_1111580, EPI_ISL_1111584, EPI_ISL_1111588, EPI_ISL_1111590, EPI_ISL_1111591, EPI_ISL_1111592, EPI_ISL_1111596, EPI_ISL_1111597, EPI_ISL_1111598, EPI_ISL_1111600, EPI_ISL_1111601, EPI_ISL_1111605			
see above	Virginia Division of Consolidated Laboratory Services	Virginia Division of Consolidated Laboratory Services	Virginia DCLS
EPI_ISL_1112753	Public Health Center of Ukraine	Charite Universitatsmedizin Berlin, Institute of Virology	Victor M Corman, Barbara Mühlemann, Jörn Beheim-Schwarzbach, Talitha Veith, Julia Schneider, Terry Jones, Roman Rodyna, Ihor Kuzin, Liudmyla Chernenko, Iryna Demchyshyna, Christian Drosten
EPI_ISL_1114406	Wyoming Public Health Laboratory	Wyoming Public Health Laboratory	Jim Mildenberger, Wanda Manley, Noah Hull, Taylor Fearing, Lynette Gumbleton, Channing Weber, Ashley Norberg, Chayse Rowley, Marley Goetz, Brian Dominguez, Elliot Thomasson, Cari Sloma, and Rob Christensen
EPI_ISL_1115111	Lighthouse Lab in Glasgow	Wellcome Sanger Institute for the COVID-19 Genomics UK (COG-UK) Consortium	Harper VanSteenhouse, Yumi Kasai, David Gray, Carol Clugston, Anna Dominiczak and Alex Alderton, Roberto Amato, Jeffrey Barrett, Sonia Goncalves, Ewan Harrison, David K. Jackson, Ian Johnston, Dominic Kwiatkowski, Cordelia Langford, John Sillitoe on behalf of the Wellcome Sanger Institute COVID-19 Surveillance Team
EPI_ISL_1117376	Beijing Center for Disease Prevention and Control	Beijing Center for Disease Prevention and Control	Yang Pan, Shujuan Cui, Fu Li, Zhaomin Feng, Bing Lyu, Zhichao Liang, Lijuan Chen, Daitao Zhang, Quanyi Wang
EPI_ISL_1117377	Beijing Center for Disease Prevention and Control	Beijing Center for Disease Prevention and Control	Yang Pan, Fu Li, Zhaomin Feng, Shujuan Cui, Bing Lyu, Zhichao Liang, Lijuan Chen, Daitao Zhang, Quanyi Wang
EPI_ISL_1117378	Beijing Center for Disease Prevention and Control	Beijing Center for Disease Prevention and Control	Yang Pan, Zhaomin Feng, Fu Li, Shujuan Cui, Bing Lyu, Zhichao Liang, Lijuan Chen, Daitao Zhang, Quanyi Wang
EPI_ISL_1118117, EPI_ISL_1118118, EPI_ISL_1118119, EPI_ISL_1118120, EPI_ISL_1118121	Dept. of Microbiology and Infection Control, Akershus University Hospital HF	Dept. of Microbiology and Infection Control, Akershus University Hospital HF	Hege Vangstein Aamot, Alexander Hesselberg Løvestad
EPI_ISL_1118206	National Institute of Health Research and Development	National Institute of Health Research and Development	Hana Apsari Pawestri, Subangkit, Kartika Dewi Puspa, Arie Ardiansyah Nugraha, Hartanti Dian Ikawati, Krisna Nur Andriana Pangesti, Yuni Rukminiati, Ririn Ramadhany, Agustiningsih, Kindi Adam, Holy Arif Wibowo, Triyani Soekarso, Ni Ketut Susilarini, Nurika Hariastuti, Ully Alfi Nikmah, Reni Herman, Nike Susanti, Herna, Tati Febriyanti, Natalie Laurencia Kipuw, Fauzul Muna, Irene Lorinda Indalao, Nelly Puspandari, Vivi Setiawaty.
EPI_ISL_1118408	Department of Medical Microbiology, St. Olavs hospital	Norwegian Institute of Public Health, Department of Virology	Kathrine Stene-Johansen, Kamilla Heddeland Instefjord, Hilde Elshaug, Garcia Llorente Ignacio, Engebretsen Serina Beate,Pedersen Benedikte Nevjen, Debech Nadia, Atiya R Ali,Marie Paulsen Madsen, Rasmus Riis Kopperud, Hilde Vollan, Karoline Bragstad, Olav Hungnes
EPI_ISL_1118447	Dept. of Medical Microbiology, Stavanger University Hospital, Helse Stavanger HF	Norwegian Institute of Public Health, Department of Virology	Kathrine Stene-Johansen, Kamilla Heddeland Instefjord, Hilde Elshaug, Garcia Llorente Ignacio, Engebretsen Serina Beate,Pedersen Benedikte Nevjen, Debech Nadia, Atiya R Ali,Marie Paulsen Madsen, Rasmus Riis Kopperud, Hilde Vollan, Karoline Bragstad, Olav Hungnes
EPI_ISL_1118463, EPI_ISL_1118464	Department of Medical Microbiology, St. Olavs hospital	Norwegian Institute of Public Health, Department of Virology	Kathrine Stene-Johansen, Kamilla Heddeland Instefjord, Hilde Elshaug, Garcia Llorente Ignacio, Engebretsen Serina Beate,Pedersen Benedikte Nevjen, Debech Nadia, Atiya R Ali,Marie Paulsen Madsen, Rasmus Riis Kopperud, Hilde Vollan, Karoline Bragstad, Olav Hungnes
EPI_ISL_1118467	Akershus University Hospital, Department for Microbiology and Infectious Disease Control	Norwegian Institute of Public Health, Department of Virology	Kathrine Stene-Johansen, Kamilla Heddeland Instefjord, Hilde Elshaug, Garcia Llorente Ignacio, Engebretsen Serina Beate,Pedersen Benedikte Nevjen, Debech Nadia, Atiya R Ali,Marie Paulsen Madsen, Rasmus Riis Kopperud, Hilde Vollan, Karoline Bragstad, Olav Hungnes
EPI_ISL_1120580, EPI_ISL_1120584	Dutch COVID-19 response team	Medical Microbiology, Maastricht University Medical Centre	Jozef Dingemans*, Brian van der Veer*, Erik Beuken, Carmen Reumkens, Lieke van Alphen, Christian Hoebe, Paul Savelkoul
EPI_ISL_1120823, EPI_ISL_1120828, EPI_ISL_1120829, EPI_ISL_1120830	ICH-SION	Laboratory of genomics and metagenomics, Institute of Microbiology, University Hospital Centre and University of Lausanne, Switzerland	Trestan Pillonel, Damien Jacot, Sébastien Aeby, Gilbert Greub, Claire Bertelli
EPI_ISL_1120945, EPI_ISL_1120946, EPI_ISL_1120948, EPI_ISL_1120949, EPI_ISL_1120950, EPI_ISL_1120955, EPI_ISL_1120956, EPI_ISL_1120957	Hospital Universitario 12 de Octubre	SeqCOVID-SPAIN consortium/IBV(CSIC)	Esther Viedma, Raúl Recio, Irene Múñoz-Gallego, Jennifer Villa, Rafael Delgado, Mª Dolores Folgueira and SeqCOVID-SPAIN consortiumconsortium
EPI_ISL_1122059	OHSU Lab Services Molecular Microbiology Lab	Oregon SARS-CoV-2 Genome Sequencing Center	Brendan L. O'Connell, Sally Grindstaff, Kayla Carter, Ruth V. Nichols, Alec J. Hirsch, Donna Hansel, Guang Fan, Xuan, Qin, Daniel N. Streblow, William B. Messer, Andrew C. Adey, Benjamin N. Bimber, Brian J. O'Roak
EPI_ISL_1123290	University of Chittagong	Central Biological Research Laboratory and Department of Biochemistry and Molecular Biology	H. M. Abdullah Al Masud, Mohammad Omar Faruque, Md. Arif Hossain, Shanta Paul, Md. Khondakar Raziur Rahman, Imam Hossen, Sajib Rudra, Md. Omer Faruq, Md. Imranul Hoq, Robiul Hasan Bhuiyan
EPI_ISL_1123291, EPI_ISL_1123292	University of Chittagong	Central Biological Research Laboratory and Department of Biochemistry and Molecular Biology	Robiul Hasan Bhuiyan, Md. Imranul Hoq, Md. Khondakar Raziur Rahman, Imam Hossen, Sajib Rudra, Md. Arif Hossain, Shanta Paul, Md. Omer Faruq, H. M. Abdullah Al Masud, Mohammad Omar Faruque
EPI_ISL_1123432, EPI_ISL_1123433	Pathogen Genomics Center, National Institute of Infectious Diseases	Pathogen Genomics Center, National Institute of Infectious Diseases	Tsuyoshi Sekizuka, Kentaro Itokawa, Rina Tanaka, Masanori Hashino, Makoto Kuroda
EPI_ISL_1123437	Gunma Prefectural Institute of Public Health and Environmental Sciences	Pathogen Genomics Center, National Institute of Infectious Diseases	Tsuyoshi Sekizuka, Kentaro Itokawa, Rina Tanaka, Masanori Hashino, Makoto Kuroda
EPI_ISL_1123459, EPI_ISL_1123460, EPI_ISL_1123465, EPI_ISL_1123466, EPI_ISL_1123488, EPI_ISL_1123489, EPI_ISL_1123490, EPI_ISL_1123491, EPI_ISL_1123492, EPI_ISL_1123493, EPI_ISL_1123494, EPI_ISL_1123496, EPI_ISL_1123498, EPI_ISL_1123501, EPI_ISL_1123502, EPI_ISL_1123504, EPI_ISL_1123506			
see above	Pathogen Genomics Center, National Institute of Infectious Diseases	Pathogen Genomics Center, National Institute of Infectious Diseases	Tsuyoshi Sekizuka, Kentaro Itokawa, Rina Tanaka, Masanori Hashino, Makoto Kuroda
EPI_ISL_1123525, EPI_ISL_1123527, EPI_ISL_1123529, EPI_ISL_1123531, EPI_ISL_1123532, EPI_ISL_1123534, EPI_ISL_1123536, EPI_ISL_1123538, EPI_ISL_1123540, EPI_ISL_1123541, EPI_ISL_1123543, EPI_ISL_1123545, EPI_ISL_1123547, EPI_ISL_1123548, EPI_ISL_1123550, EPI_ISL_1123552, EPI_ISL_1123553, EPI_ISL_1123555, EPI_ISL_1123557, EPI_ISL_1123559, EPI_ISL_1123561, EPI_ISL_1123563, EPI_ISL_1123564, EPI_ISL_1123566, EPI_ISL_1123568, EPI_ISL_1123570, EPI_ISL_1123572, EPI_ISL_1123574, EPI_ISL_1123576, EPI_ISL_1123577, EPI_ISL_1123579, EPI_ISL_1123581			
see above	Gunma Prefectural Institute of Public Health and Environmental Sciences	Pathogen Genomics Center, National Institute of Infectious Diseases	Tsuyoshi Sekizuka, Kentaro Itokawa, Rina Tanaka, Masanori Hashino, Makoto Kuroda
EPI_ISL_1123625	Department of Virus and Microbiological Special Diagnostics, Statens Serum Institut, Copenhagen, Denmark	Aalborg University	Danish Covid-19 Genome Consortium
EPI_ISL_1123667, EPI_ISL_1123669, EPI_ISL_1123671, EPI_ISL_1123673, EPI_ISL_1123674, EPI_ISL_1123676, EPI_ISL_1123678, EPI_ISL_1123683, EPI_ISL_1123685, EPI_ISL_1123792	Pathogen Genomics Center, National Institute of Infectious Diseases	Pathogen Genomics Center, National Institute of Infectious Diseases	Tsuyoshi Sekizuka, Kentaro Itokawa, Rina Tanaka, Masanori Hashino, Makoto Kuroda
EPI_ISL_1123867	Department of Virus and Microbiological Special Diagnostics, Statens Serum Institut, Copenhagen, Denmark	Aalborg University	Danish Covid-19 Genome Consortium
EPI_ISL_1123909, EPI_ISL_1123911, EPI_ISL_1123913, EPI_ISL_1123915, EPI_ISL_1123916, EPI_ISL_1123918, EPI_ISL_1123920, EPI_ISL_1123922, EPI_ISL_1123924, EPI_ISL_1123926, EPI_ISL_1123927, EPI_ISL_1123929, EPI_ISL_1123931, EPI_ISL_1123933, EPI_ISL_1123935, EPI_ISL_1123937, EPI_ISL_1123938, EPI_ISL_1123940, EPI_ISL_1123942, EPI_ISL_1123944, EPI_ISL_1123946, EPI_ISL_1123948			
see above	Pathogen Genomics Center, National Institute of Infectious Diseases	Pathogen Genomics Center, National Institute of Infectious Diseases	Tsuyoshi Sekizuka, Kentaro Itokawa, Rina Tanaka, Masanori Hashino, Makoto Kuroda

EPI_ISL_1124040	Department of Virus and Microbiological Special Diagnostics, Statens Serum Institut, Copenhagen, Denmark	Aalborg University	Danish Covid-19 Genome Consortium
EPI_ISL_1124095, EPI_ISL_1124097, EPI_ISL_1124099, EPI_ISL_1124101, EPI_ISL_1124102, EPI_ISL_1124104	Toyama Institute of Health	Pathogen Genomics Center, National Institute of Infectious Diseases	Tsuyoshi Sekizuka, Kentaro Itokawa, Rina Tanaka, Masanori Hashino, Makoto Kuroda
EPI_ISL_1124160	Department of Virus and Microbiological Special Diagnostics, Statens Serum Institut, Copenhagen, Denmark	Aalborg University	Danish Covid-19 Genome Consortium
EPI_ISL_1124201, EPI_ISL_1124203, EPI_ISL_1124205, EPI_ISL_1124207	Toyama Institute of Health	Pathogen Genomics Center, National Institute of Infectious Diseases	Tsuyoshi Sekizuka, Kentaro Itokawa, Rina Tanaka, Masanori Hashino, Makoto Kuroda
EPI_ISL_1124208, EPI_ISL_1124210, EPI_ISL_1124212, EPI_ISL_1124213, EPI_ISL_1124215, EPI_ISL_1124217, EPI_ISL_1124219, EPI_ISL_1124221, EPI_ISL_1124222, EPI_ISL_1124224, EPI_ISL_1124226, EPI_ISL_1124227, EPI_ISL_1124229, EPI_ISL_1124231			
see above	Pathogen Genomics Center, National Institute of Infectious Diseases	Pathogen Genomics Center, National Institute of Infectious Diseases	Tsuyoshi Sekizuka, Kentaro Itokawa, Rina Tanaka, Masanori Hashino, Makoto Kuroda
EPI_ISL_1124234, EPI_ISL_1124312	Department of Virus and Microbiological Special Diagnostics, Statens Serum Institut, Copenhagen, Denmark	Aalborg University	Danish Covid-19 Genome Consortium
EPI_ISL_1124358, EPI_ISL_1124360, EPI_ISL_1124362	Pathogen Genomics Center, National Institute of Infectious Diseases	Pathogen Genomics Center, National Institute of Infectious Diseases	Tsuyoshi Sekizuka, Kentaro Itokawa, Rina Tanaka, Masanori Hashino, Makoto Kuroda
EPI_ISL_1124363	Department of Virus and Microbiological Special Diagnostics, Statens Serum Institut, Copenhagen, Denmark	Aalborg University	Danish Covid-19 Genome Consortium
EPI_ISL_1124364	Pathogen Genomics Center, National Institute of Infectious Diseases	Pathogen Genomics Center, National Institute of Infectious Diseases	Tsuyoshi Sekizuka, Kentaro Itokawa, Rina Tanaka, Masanori Hashino, Makoto Kuroda
EPI_ISL_1124477, EPI_ISL_1124522, EPI_ISL_1124596, EPI_ISL_1124631	Department of Virus and Microbiological Special Diagnostics, Statens Serum Institut, Copenhagen, Denmark	Aalborg University	Danish Covid-19 Genome Consortium
EPI_ISL_1124653, EPI_ISL_1124655, EPI_ISL_1124657, EPI_ISL_1124659, EPI_ISL_1124661, EPI_ISL_1124662, EPI_ISL_1124664, EPI_ISL_1124666, EPI_ISL_1124668, EPI_ISL_1124669, EPI_ISL_1124694, EPI_ISL_1124695, EPI_ISL_1124697, EPI_ISL_1124699, EPI_ISL_1124701, EPI_ISL_1124702, EPI_ISL_1124704, EPI_ISL_1124706, EPI_ISL_1124708, EPI_ISL_1124710, EPI_ISL_1124711, EPI_ISL_1124713, EPI_ISL_1124715, EPI_ISL_1124717, EPI_ISL_1124719, EPI_ISL_1124721, EPI_ISL_1124722, EPI_ISL_1124724, EPI_ISL_1124726, EPI_ISL_1124728, EPI_ISL_1124730			
see above	Pathogen Genomics Center, National Institute of Infectious Diseases	Pathogen Genomics Center, National Institute of Infectious Diseases	Tsuyoshi Sekizuka, Kentaro Itokawa, Rina Tanaka, Masanori Hashino, Makoto Kuroda
EPI_ISL_1124794, EPI_ISL_1124796	Department of Virus and Microbiological Special Diagnostics, Statens Serum Institut, Copenhagen, Denmark	Aalborg University	Danish Covid-19 Genome Consortium
EPI_ISL_1124807, EPI_ISL_1124809, EPI_ISL_1124811, EPI_ISL_1124813, EPI_ISL_1124814, EPI_ISL_1124816, EPI_ISL_1124818, EPI_ISL_1124820, EPI_ISL_1124822, EPI_ISL_1124823, EPI_ISL_1124825, EPI_ISL_1124827, EPI_ISL_1124828, EPI_ISL_1124830, EPI_ISL_1124832, EPI_ISL_1124834, EPI_ISL_1124835, EPI_ISL_1124837, EPI_ISL_1124839, EPI_ISL_1124841, EPI_ISL_1124842, EPI_ISL_1124844, EPI_ISL_1124846, EPI_ISL_1124848, EPI_ISL_1124849, EPI_ISL_1124851, EPI_ISL_1124908, EPI_ISL_1124910, EPI_ISL_1124911, EPI_ISL_1124913, EPI_ISL_1124915, EPI_ISL_1124917, EPI_ISL_1124919, EPI_ISL_1124921, EPI_ISL_1124922, EPI_ISL_1124930, EPI_ISL_1124931			
see above	Pathogen Genomics Center, National Institute of Infectious Diseases	Pathogen Genomics Center, National Institute of Infectious Diseases	Tsuyoshi Sekizuka, Kentaro Itokawa, Rina Tanaka, Masanori Hashino, Makoto Kuroda
EPI_ISL_1124989, EPI_ISL_1125053, EPI_ISL_1125132, EPI_ISL_1125134, EPI_ISL_1125211, EPI_ISL_1125326, EPI_ISL_1125344, EPI_ISL_1125345, EPI_ISL_1125372, EPI_ISL_1125432, EPI_ISL_1125442, EPI_ISL_1125508, EPI_ISL_1125592, EPI_ISL_1125655, EPI_ISL_1125668, EPI_ISL_1125722, EPI_ISL_1125772, EPI_ISL_1125775, EPI_ISL_1125827, EPI_ISL_1125847, EPI_ISL_1125870, EPI_ISL_1125877, EPI_ISL_1125920, EPI_ISL_1125956, EPI_ISL_1125997, EPI_ISL_1126022, EPI_ISL_1126023, EPI_ISL_1126063, EPI_ISL_1126077, EPI_ISL_1126104, EPI_ISL_1126200, EPI_ISL_1126223, EPI_ISL_1126350, EPI_ISL_1126352, EPI_ISL_1126376, EPI_ISL_1126382, EPI_ISL_1126406, EPI_ISL_1126427, EPI_ISL_1126435, EPI_ISL_1126480, EPI_ISL_1126495, EPI_ISL_1126572, EPI_ISL_1126626, EPI_ISL_1126644, EPI_ISL_1126645, EPI_ISL_1126666, EPI_ISL_1126691, EPI_ISL_1126732, EPI_ISL_1126778, EPI_ISL_1126820, EPI_ISL_1126892, EPI_ISL_1126983, EPI_ISL_1127000, EPI_ISL_1127016, EPI_ISL_1127019, EPI_ISL_1127072			
see above	Department of Virus and Microbiological Special Diagnostics, Statens Serum Institut, Copenhagen, Denmark	Aalborg University	Danish Covid-19 Genome Consortium
EPI_ISL_1127139, EPI_ISL_1127146, EPI_ISL_1127147, EPI_ISL_1127153, EPI_ISL_1127154, EPI_ISL_1127163, EPI_ISL_1127186, EPI_ISL_1127187, EPI_ISL_1127188, EPI_ISL_1127189, EPI_ISL_1127190, EPI_ISL_1127191, EPI_ISL_1127192, EPI_ISL_1127193, EPI_ISL_1127194, EPI_ISL_1127195, EPI_ISL_1127196, EPI_ISL_1127197, EPI_ISL_1127198, EPI_ISL_1127199, EPI_ISL_1127200, EPI_ISL_1127201, EPI_ISL_1127202, EPI_ISL_1127203, EPI_ISL_1127204, EPI_ISL_1127205, EPI_ISL_1127206, EPI_ISL_1127207, EPI_ISL_1127208, EPI_ISL_1127209, EPI_ISL_1127210, EPI_ISL_1127211, EPI_ISL_1127274, EPI_ISL_1127313, EPI_ISL_1127314, EPI_ISL_1127315, EPI_ISL_1127316, EPI_ISL_1127317, EPI_ISL_1127318, EPI_ISL_1127319, EPI_ISL_1127320, EPI_ISL_1127321, EPI_ISL_1127322, EPI_ISL_1127323, EPI_ISL_1127324, EPI_ISL_1127325, EPI_ISL_1127452, EPI_ISL_1127453, EPI_ISL_1127454, EPI_ISL_1127455, EPI_ISL_1127456, EPI_ISL_1127457, EPI_ISL_1127458, EPI_ISL_1127459, EPI_ISL_1127460, EPI_ISL_1127461, EPI_ISL_1127462, EPI_ISL_1127463, EPI_ISL_1127464, EPI_ISL_1127465, EPI_ISL_1127466, EPI_ISL_1127467, EPI_ISL_1127468, EPI_ISL_1127469, EPI_ISL_1127470, EPI_ISL_1127471, EPI_ISL_1127472, EPI_ISL_1127473, EPI_ISL_1127474, EPI_ISL_1127475, EPI_ISL_1127476, EPI_ISL_1127477, EPI_ISL_1127478, EPI_ISL_1127479, EPI_ISL_1127480, EPI_ISL_1127481, EPI_ISL_1127482, EPI_ISL_1127483, EPI_ISL_1127484, EPI_ISL_1127485, EPI_ISL_1127486, EPI_ISL_1127487, EPI_ISL_1127488, EPI_ISL_1127489, EPI_ISL_1127490, EPI_ISL_1127491, EPI_ISL_1127492, EPI_ISL_1127493, EPI_ISL_1127494, EPI_ISL_1127495, EPI_ISL_1127496, EPI_ISL_1127497, EPI_ISL_1127498, EPI_ISL_1127499, EPI_ISL_1127500, EPI_ISL_1127501, EPI_ISL_1127502, EPI_ISL_1127503, EPI_ISL_1127504, EPI_ISL_1127505, EPI_ISL_1127506, EPI_ISL_1127507, EPI_ISL_1127508, EPI_ISL_1127509, EPI_ISL_1127510, EPI_ISL_1127511, EPI_ISL_1127512, EPI_ISL_1127513, EPI_ISL_1127514, EPI_ISL_1127515, EPI_ISL_1127516, EPI_ISL_1127517, EPI_ISL_1127518, EPI_ISL_1127519, EPI_ISL_1127520, EPI_ISL_1127521, EPI_ISL_1127522, EPI_ISL_1127523, EPI_ISL_1127524, EPI_ISL_1127525, EPI_ISL_1127526, EPI_ISL_1127527, EPI_ISL_1127528, EPI_ISL_1127529, EPI_ISL_1127530, EPI_ISL_1127531, EPI_ISL_1127532, EPI_ISL_1127533, EPI_ISL_1127534, EPI_ISL_1127535, EPI_ISL_1127536, EPI_ISL_1127537, EPI_ISL_1127739, EPI_ISL_1127740, EPI_ISL_1127741, EPI_ISL_1127742, EPI_ISL_1127743, EPI_ISL_1127744, EPI_ISL_1127745, EPI_ISL_1127746, EPI_ISL_1127747, EPI_ISL_1127748, EPI_ISL_1127749, EPI_ISL_1127750, EPI_ISL_1127751, EPI_ISL_1127752, EPI_ISL_1127753, EPI_ISL_1127819, EPI_ISL_1127820, EPI_ISL_1127821, EPI_ISL_1127822, EPI_ISL_1127823, EPI_ISL_1127824, EPI_ISL_1127825, EPI_ISL_1127826, EPI_ISL_1127827, EPI_ISL_1127828, EPI_ISL_1127829, EPI_ISL_1127830, EPI_ISL_1127831, EPI_ISL_1127832, EPI_ISL_1127833, EPI_ISL_1127834, EPI_ISL_1127835, EPI_ISL_1127836, EPI_ISL_1127837, EPI_ISL_1127838, EPI_ISL_1127839, EPI_ISL_1127840, EPI_ISL_1127841, EPI_ISL_1127842, EPI_ISL_1127843, EPI_ISL_1127844, EPI_ISL_1127845, EPI_ISL_1127846, EPI_ISL_1127847, EPI_ISL_1127848, EPI_ISL_1127849, EPI_ISL_1127850, EPI_ISL_1127851, EPI_ISL_1127852, EPI_ISL_1127853, EPI_ISL_1127854, EPI_ISL_1127855			
see above	Pathogen Genomics Center, National Institute of Infectious Diseases	Pathogen Genomics Center, National Institute of Infectious Diseases	Tsuyoshi Sekizuka, Kentaro Itokawa, Rina Tanaka, Masanori Hashino, Makoto Kuroda
EPI_ISL_1127954, EPI_ISL_1127955, EPI_ISL_1127956, EPI_ISL_1127957, EPI_ISL_1127958, EPI_ISL_1127959, EPI_ISL_1127960, EPI_ISL_1127961, EPI_ISL_1127962, EPI_ISL_1127963, EPI_ISL_1127964, EPI_ISL_1127965, EPI_ISL_1127966, EPI_ISL_1127967, EPI_ISL_1127968, EPI_ISL_1127969, EPI_ISL_1127970, EPI_ISL_1127971, EPI_ISL_1127972, EPI_ISL_1127973, EPI_ISL_1127974, EPI_ISL_1127975, EPI_ISL_1127976, EPI_ISL_1127977			
see above	Osaka Institute of Public Health, Morinomiya Center	Pathogen Genomics Center, National Institute of Infectious Diseases	Tsuyoshi Sekizuka, Kentaro Itokawa, Rina Tanaka, Masanori Hashino, Makoto Kuroda
EPI_ISL_1128000, EPI_ISL_1128001, EPI_ISL_1128002, EPI_ISL_1128003, EPI_ISL_1128004, EPI_ISL_1128005, EPI_ISL_1128006, EPI_ISL_1128007, EPI_ISL_1128011, EPI_ISL_1128012, EPI_ISL_1128013, EPI_ISL_1128014			
see above	Kanagawa Prefectural Institute of Public Health	Pathogen Genomics Center, National Institute of Infectious Diseases	Tsuyoshi Sekizuka, Kentaro Itokawa, Rina Tanaka, Masanori Hashino, Makoto Kuroda
EPI_ISL_1128057, EPI_ISL_1128058, EPI_ISL_1128059, EPI_ISL_1128060, EPI_ISL_1128061	Pathogen Genomics Center, National Institute of Infectious Diseases	Pathogen Genomics Center, National Institute of Infectious Diseases	Tsuyoshi Sekizuka, Kentaro Itokawa, Rina Tanaka, Masanori Hashino, Makoto Kuroda
EPI_ISL_1128148	Department of Infectious Diseases, Kobe Institute of Health	Pathogen Genomics Center, National Institute of Infectious Diseases	Tsuyoshi Sekizuka, Kentaro Itokawa, Rina Tanaka, Masanori Hashino, Makoto Kuroda
EPI_ISL_1128157	Pathogen Genomics Center, National Institute of Infectious Diseases	Pathogen Genomics Center, National Institute of Infectious Diseases	Tsuyoshi Sekizuka, Kentaro Itokawa, Rina Tanaka, Masanori Hashino, Makoto Kuroda
EPI_ISL_1128158, EPI_ISL_1128161	Department of Infectious Diseases, Kobe Institute of Health	Pathogen Genomics Center, National Institute of Infectious Diseases	Tsuyoshi Sekizuka, Kentaro Itokawa, Rina Tanaka, Masanori Hashino, Makoto Kuroda
EPI_ISL_1128163, EPI_ISL_1128164, EPI_ISL_1128168, EPI_ISL_1128170, EPI_ISL_1128171, EPI_ISL_1128172, EPI_ISL_1128174, EPI_ISL_1128180, EPI_ISL_1128181, EPI_ISL_1128182, EPI_ISL_1128183, EPI_ISL_1128184, EPI_ISL_1128185, EPI_ISL_1128186, EPI_ISL_1128187, EPI_ISL_1128188, EPI_ISL_1128189, EPI_ISL_1128190, EPI_ISL_1128191, EPI_ISL_1128192, EPI_ISL_1128193, EPI_ISL_1128194, EPI_ISL_1128195			
see above	Pathogen Genomics Center, National Institute of Infectious Diseases	Pathogen Genomics Center, National Institute of Infectious Diseases	Tsuyoshi Sekizuka, Kentaro Itokawa, Rina Tanaka, Masanori Hashino, Makoto Kuroda

EPI_ISL_1128198, EPI_ISL_1128199, EPI_ISL_1128200, EPI_ISL_1128201, EPI_ISL_1128202, EPI_ISL_1128203, EPI_ISL_1128204, EPI_ISL_1128205, EPI_ISL_1128206, EPI_ISL_1128207, EPI_ISL_1128208, EPI_ISL_1128209, EPI_ISL_1128210, EPI_ISL_1128211, EPI_ISL_1128212, EPI_ISL_1128213, EPI_ISL_1128214, EPI_ISL_1128215, EPI_ISL_1128216, EPI_ISL_1128217, EPI_ISL_1128218, EPI_ISL_1128219, EPI_ISL_1128220, EPI_ISL_1128221, EPI_ISL_1128222, EPI_ISL_1128223, EPI_ISL_1128224, EPI_ISL_1128225, EPI_ISL_1128226, EPI_ISL_1128227, EPI_ISL_1128228, EPI_ISL_1128237, EPI_ISL_1128238, EPI_ISL_1128239, EPI_ISL_1128240, EPI_ISL_1128241, EPI_ISL_1128242, EPI_ISL_1128243, EPI_ISL_1128244, EPI_ISL_1128245, EPI_ISL_1128246, EPI_ISL_1128247, EPI_ISL_1128248, EPI_ISL_1128249, EPI_ISL_1128250, EPI_ISL_1128251, EPI_ISL_1128252, EPI_ISL_1128253, EPI_ISL_1128254, EPI_ISL_1128255, EPI_ISL_1128256, EPI_ISL_1128257, EPI_ISL_1128258, EPI_ISL_1128259, EPI_ISL_1128260, EPI_ISL_1128261, EPI_ISL_1128262, EPI_ISL_1128263, EPI_ISL_1128264, EPI_ISL_1128265, EPI_ISL_1128266, EPI_ISL_1128267, EPI_ISL_1128268, EPI_ISL_1128269, EPI_ISL_1128270, EPI_ISL_1128271			
see above	Department of Infectious Diseases, Kobe Institute of Health	Pathogen Genomics Center, National Institute of Infectious Diseases	Tsuyoshi Sekizuka, Kentaro Itokawa, Rina Tanaka, Masanori Hashino, Makoto Kuroda
EPI_ISL_1128282, EPI_ISL_1128283, EPI_ISL_1128284, EPI_ISL_1128285, EPI_ISL_1128286, EPI_ISL_1128287, EPI_ISL_1128288, EPI_ISL_1128289, EPI_ISL_1128290, EPI_ISL_1128291, EPI_ISL_1128292, EPI_ISL_1128293, EPI_ISL_1128294, EPI_ISL_1128295, EPI_ISL_1128296, EPI_ISL_1128297, EPI_ISL_1128298, EPI_ISL_1128299, EPI_ISL_1128300, EPI_ISL_1128301, EPI_ISL_1128302, EPI_ISL_1128303, EPI_ISL_1128304, EPI_ISL_1128305, EPI_ISL_1128306, EPI_ISL_1128307, EPI_ISL_1128308, EPI_ISL_1128309, EPI_ISL_1128310, EPI_ISL_1128311, EPI_ISL_1128312, EPI_ISL_1128313, EPI_ISL_1128314, EPI_ISL_1128315, EPI_ISL_1128316, EPI_ISL_1128317, EPI_ISL_1128318, EPI_ISL_1128319, EPI_ISL_1128320, EPI_ISL_1128321, EPI_ISL_1128322, EPI_ISL_1128323, EPI_ISL_1128324, EPI_ISL_1128325, EPI_ISL_1128326, EPI_ISL_1128327, EPI_ISL_1128328, EPI_ISL_1128329, EPI_ISL_1128330, EPI_ISL_1128331, EPI_ISL_1128332, EPI_ISL_1128333, EPI_ISL_1128334, EPI_ISL_1128335, EPI_ISL_1128336, EPI_ISL_1128337, EPI_ISL_1128338, EPI_ISL_1128339, EPI_ISL_1128340, EPI_ISL_1128341, EPI_ISL_1128342, EPI_ISL_1128343, EPI_ISL_1128344, EPI_ISL_1128345, EPI_ISL_1128346, EPI_ISL_1128347, EPI_ISL_1128348, EPI_ISL_1128349, EPI_ISL_1128350, EPI_ISL_1128351, EPI_ISL_1128352, EPI_ISL_1128353, EPI_ISL_1128354, EPI_ISL_1128355, EPI_ISL_1128356, EPI_ISL_1128357, EPI_ISL_1128358, EPI_ISL_1128359, EPI_ISL_1128360, EPI_ISL_1128361, EPI_ISL_1128362, EPI_ISL_1128363, EPI_ISL_1128364, EPI_ISL_1128365, EPI_ISL_1128366, EPI_ISL_1128367, EPI_ISL_1128368, EPI_ISL_1128369, EPI_ISL_1128370, EPI_ISL_1128371, EPI_ISL_1128372, EPI_ISL_1128373, EPI_ISL_1128374, EPI_ISL_1128375, EPI_ISL_1128376, EPI_ISL_1128377, EPI_ISL_1128378, EPI_ISL_1128379, EPI_ISL_1128380, EPI_ISL_1128381, EPI_ISL_1128382, EPI_ISL_1128383, EPI_ISL_1128384, EPI_ISL_1128385, EPI_ISL_1128386, EPI_ISL_1128387, EPI_ISL_1128388, EPI_ISL_1128389, EPI_ISL_1128390, EPI_ISL_1128391, EPI_ISL_1128392, EPI_ISL_1128393, EPI_ISL_1128394, EPI_ISL_1128395, EPI_ISL_1128396, EPI_ISL_1128397, EPI_ISL_1128398, EPI_ISL_1128399, EPI_ISL_1128400, EPI_ISL_1128401, EPI_ISL_1128402, EPI_ISL_1128403, EPI_ISL_1128404, EPI_ISL_1128405, EPI_ISL_1128406, EPI_ISL_1128407, EPI_ISL_1128408, EPI_ISL_1128409, EPI_ISL_1128410, EPI_ISL_1128411, EPI_ISL_1128412, EPI_ISL_1128413, EPI_ISL_1128414, EPI_ISL_1128415, EPI_ISL_1128416, EPI_ISL_1128417, EPI_ISL_1128418, EPI_ISL_1128419, EPI_ISL_1128420, EPI_ISL_1128421, EPI_ISL_1128422, EPI_ISL_1128423, EPI_ISL_1128424, EPI_ISL_1128425, EPI_ISL_1128426, EPI_ISL_1128427, EPI_ISL_1128428, EPI_ISL_1128429, EPI_ISL_1128430, EPI_ISL_1128431, EPI_ISL_1128432, EPI_ISL_1128433, EPI_ISL_1128434, EPI_ISL_1128435, EPI_ISL_1128436, EPI_ISL_1128437, EPI_ISL_1128438, EPI_ISL_1128439, EPI_ISL_1128440, EPI_ISL_1128441, EPI_ISL_1128442, EPI_ISL_1128443, EPI_ISL_1128444, EPI_ISL_1128445, EPI_ISL_1128455, EPI_ISL_1128456, EPI_ISL_1128457, EPI_ISL_1128458, EPI_ISL_1128459, EPI_ISL_1128460, EPI_ISL_1128461, EPI_ISL_1128462, EPI_ISL_1128463, EPI_ISL_1128464, EPI_ISL_1128465, EPI_ISL_1128466, EPI_ISL_1128467, EPI_ISL_1128468, EPI_ISL_1128469, EPI_ISL_1128470, EPI_ISL_1128471, EPI_ISL_1128472, EPI_ISL_1128473, EPI_ISL_1128474, EPI_ISL_1128475, EPI_ISL_1128476, EPI_ISL_1128477, EPI_ISL_1128478, EPI_ISL_1128479, EPI_ISL_1128480, EPI_ISL_1128481, EPI_ISL_1128482, EPI_ISL_1128483, EPI_ISL_1128484, EPI_ISL_1128485, EPI_ISL_1128486, EPI_ISL_1128487, EPI_ISL_1128488, EPI_ISL_1128489, EPI_ISL_1128492, EPI_ISL_1128528, EPI_ISL_1128529, EPI_ISL_1128530, EPI_ISL_1128531, EPI_ISL_1128532, EPI_ISL_1128533, EPI_ISL_1128534, EPI_ISL_1128535, EPI_ISL_1128536, EPI_ISL_1128537, EPI_ISL_1128538, EPI_ISL_1128539, EPI_ISL_1128540, EPI_ISL_1128541, EPI_ISL_1128542, EPI_ISL_1128543, EPI_ISL_1128544, EPI_ISL_1128545, EPI_ISL_1128546, EPI_ISL_1128547, EPI_ISL_1128548, EPI_ISL_1128549, EPI_ISL_1128550, EPI_ISL_1128551, EPI_ISL_1128552, EPI_ISL_1128553, EPI_ISL_1128554, EPI_ISL_1128555, EPI_ISL_1128556, EPI_ISL_1128557, EPI_ISL_1128558, EPI_ISL_1128559, EPI_ISL_1128560, EPI_ISL_1128561, EPI_ISL_1128562, EPI_ISL_1128563, EPI_ISL_1128564, EPI_ISL_1128565, EPI_ISL_1128566, EPI_ISL_1128567, EPI_ISL_1128568, EPI_ISL_1128569, EPI_ISL_1128570, EPI_ISL_1128571, EPI_ISL_1128572, EPI_ISL_1128573, EPI_ISL_1128574, EPI_ISL_1128575, EPI_ISL_1128576, EPI_ISL_1128577, EPI_ISL_1128603, EPI_ISL_1128604, EPI_ISL_1128605, EPI_ISL_1128606, EPI_ISL_1128607, EPI_ISL_1128608, EPI_ISL_1128609, EPI_ISL_1128610, EPI_ISL_1128611, EPI_ISL_1128612, EPI_ISL_1128613, EPI_ISL_1128614, EPI_ISL_1128615, EPI_ISL_1128616, EPI_ISL_1128617, EPI_ISL_1128618, EPI_ISL_1128619, EPI_ISL_1128620, EPI_ISL_1128621, EPI_ISL_1128622, EPI_ISL_1128623, EPI_ISL_1128624, EPI_ISL_1128625, EPI_ISL_1128626, EPI_ISL_1128627, EPI_ISL_1128628, EPI_ISL_1128629, EPI_ISL_1128630, EPI_ISL_1128631, EPI_ISL_1128632, EPI_ISL_1128633, EPI_ISL_1128634, EPI_ISL_1128635, EPI_ISL_1128636, EPI_ISL_1128637, EPI_ISL_1128640, EPI_ISL_1128641, EPI_ISL_1128642, EPI_ISL_1128643, EPI_ISL_1128660, EPI_ISL_1128661, EPI_ISL_1128662, EPI_ISL_1128663, EPI_ISL_1128664, EPI_ISL_1128665, EPI_ISL_1128666, EPI_ISL_1128719, EPI_ISL_1128720, EPI_ISL_1128721, EPI_ISL_1128722, EPI_ISL_1128723, EPI_ISL_1128724, EPI_ISL_1128725, EPI_ISL_1128726, EPI_ISL_1128727, EPI_ISL_1128728, EPI_ISL_1128729, EPI_ISL_1128730, EPI_ISL_1128731, EPI_ISL_1128732, EPI_ISL_1128733, EPI_ISL_1128734, EPI_ISL_1128735, EPI_ISL_1128736, EPI_ISL_1128737, EPI_ISL_1128738, EPI_ISL_1128739, EPI_ISL_1128740, EPI_ISL_1128741, EPI_ISL_1128742, EPI_ISL_1128769, EPI_ISL_1128770, EPI_ISL_1128771, EPI_ISL_1128772, EPI_ISL_1128773, EPI_ISL_1128774, EPI_ISL_1128787, EPI_ISL_1128788, EPI_ISL_1128790, EPI_ISL_1128792, EPI_ISL_1128793, EPI_ISL_1128795, EPI_ISL_1128797, EPI_ISL_1128799, EPI_ISL_1128800, EPI_ISL_1128802, EPI_ISL_1128804, EPI_ISL_1128806, EPI_ISL_1128807, EPI_ISL_1128809, EPI_ISL_1128811, EPI_ISL_1128813, EPI_ISL_1128814, EPI_ISL_1128816, EPI_ISL_1128818, EPI_ISL_1128820, EPI_ISL_1128821, EPI_ISL_1128823, EPI_ISL_1128825, EPI_ISL_1128845, EPI_ISL_1128847, EPI_ISL_1128848, EPI_ISL_1128850, EPI_ISL_1128852, EPI_ISL_1128854, EPI_ISL_1128856, EPI_ISL_1128857, EPI_ISL_1128859, EPI_ISL_1128861, EPI_ISL_1128863, EPI_ISL_1128864, EPI_ISL_1128865, EPI_ISL_1128867, EPI_ISL_1128869, EPI_ISL_1128871, EPI_ISL_1128872, EPI_ISL_1128874, EPI_ISL_1128876, EPI_ISL_1128877, EPI_ISL_1128879, EPI_ISL_1128881, EPI_ISL_1128883, EPI_ISL_1128885, EPI_ISL_1128886, EPI_ISL_1128888, EPI_ISL_1128890, EPI_ISL_1128891, EPI_ISL_1128893, EPI_ISL_1128895, EPI_ISL_1128897, EPI_ISL_1128898, EPI_ISL_1128900, EPI_ISL_1128902, EPI_ISL_1128904, EPI_ISL_1128905, EPI_ISL_1128907, EPI_ISL_1128910, EPI_ISL_1128912, EPI_ISL_1128915, EPI_ISL_1128917, EPI_ISL_1128920, EPI_ISL_1128922, EPI_ISL_1128924, EPI_ISL_1128926, EPI_ISL_1128927, EPI_ISL_1128929, EPI_ISL_1128931, EPI_ISL_1128933, EPI_ISL_1128934, EPI_ISL_1128936, EPI_ISL_1128938, EPI_ISL_1128940, EPI_ISL_1128942, EPI_ISL_1128943, EPI_ISL_1128951, EPI_ISL_1128952, EPI_ISL_1128953, EPI_ISL_1128954, EPI_ISL_1128955, EPI_ISL_1128956, EPI_ISL_1128957, EPI_ISL_1128958, EPI_ISL_1128959, EPI_ISL_1128960, EPI_ISL_1128964, EPI_ISL_1128965, EPI_ISL_1128966, EPI_ISL_1128967, EPI_ISL_1128968, EPI_ISL_1128969, EPI_ISL_1128970, EPI_ISL_1128971, EPI_ISL_1128972, EPI_ISL_1128973, EPI_ISL_1128974, EPI_ISL_1128975, EPI_ISL_1128976, EPI_ISL_1128977, EPI_ISL_1128978, EPI_ISL_1128979, EPI_ISL_1128980, EPI_ISL_1128981, EPI_ISL_1128982, EPI_ISL_1128983, EPI_ISL_1128984, EPI_ISL_1128985, EPI_ISL_1128986, EPI_ISL_1128987, EPI_ISL_1128988, EPI_ISL_1128989, EPI_ISL_1128990, EPI_ISL_1128991, EPI_ISL_1128992, EPI_ISL_1128993, EPI_ISL_1128994, EPI_ISL_1128995, EPI_ISL_1128996, EPI_ISL_1128997, EPI_ISL_1128998, EPI_ISL_1128999, EPI_ISL_1129000, EPI_ISL_1129001, EPI_ISL_1129002, EPI_ISL_1129003, EPI_ISL_1129004, EPI_ISL_1129005, EPI_ISL_1129006, EPI_ISL_1129007, EPI_ISL_1129008, EPI_ISL_1129009, EPI_ISL_1129010, EPI_ISL_1129011, EPI_ISL_1129012, EPI_ISL_1129013, EPI_ISL_1129014, EPI_ISL_1129015, EPI_ISL_1129016, EPI_ISL_1129017, EPI_ISL_1129018, EPI_ISL_1129019, EPI_ISL_1129020, EPI_ISL_1129021, EPI_ISL_1129022, EPI_ISL_1129023, EPI_ISL_1129024, EPI_ISL_1129025, EPI_ISL_1129026, EPI_ISL_1129027, EPI_ISL_1129028, EPI_ISL_1129029, EPI_ISL_1129030, EPI_ISL_1129031, EPI_ISL_1129032, EPI_ISL_1129058, EPI_ISL_1129059, EPI_ISL_1129060, EPI_ISL_1129061, EPI_ISL_1129062, EPI_ISL_1129063, EPI_ISL_1129064, EPI_ISL_1129065, EPI_ISL_1129066, EPI_ISL_1129067, EPI_ISL_1129068, EPI_ISL_1129069, EPI_ISL_1129089, EPI_ISL_1129090, EPI_ISL_1129091, EPI_ISL_1129092, EPI_ISL_1129093, EPI_ISL_1129100, EPI_ISL_1129101, EPI_ISL_1129102, EPI_ISL_1129103, EPI_ISL_1129110, EPI_ISL_1129111, EPI_ISL_1129165, EPI_ISL_1129166, EPI_ISL_1129167, EPI_ISL_1129168, EPI_ISL_1129186, EPI_ISL_1129187, EPI_ISL_1129188, EPI_ISL_1129189, EPI_ISL_1129190, EPI_ISL_1129191, EPI_ISL_1129192, EPI_ISL_1129193, EPI_ISL_1129194, EPI_ISL_1129195, EPI_ISL_1129196, EPI_ISL_1129204, EPI_ISL_1129205, EPI_ISL_1129206, EPI_ISL_1129207, EPI_ISL_1129208, EPI_ISL_1129209			
see above	Pathogen Genomics Center, National Institute of Infectious Diseases	Pathogen Genomics Center, National Institute of Infectious Diseases	Tsuyoshi Sekizuka, Kentaro Itokawa, Rina Tanaka, Masanori Hashino, Makoto Kuroda
EPI_ISL_1129280, EPI_ISL_1129345	Viollier AG	Department of Biosystems Science and Engineering, ETH Zürich	Christian Beisel, Sarah Nadeau, Chaoran Chen, Ivan Topolsky, Philipp Jablonski, Lara Fuhrmann, David Dreifuss, Katharina Jahn, Rebecca Denes, Mirjam Feldkamp, Ina Nissen, Natascha Santacroce, Elodie Burcklen, Christiane Beckmann, Maurice Redondo, Olivier Kobel, Christoph Noppen, Sophie Seidel, Noemie Santamaria de Souza, Niko Beerenwinkel, Tanja Stadler
EPI_ISL_1129389	Viollier AG	Department of Biosystems Science and Engineering, ETH Zürich	Chaoran Chen, Sarah Nadeau, Catharine Aquino, Ivan Topolsky, Philipp Jablonski, Lara Fuhrmann, David Dreifuss, Katharina Jahn, Andreia Cabral de Gouvea, Maria Domenica Moccia, Simon Grüter, Timothy Sykes, Lennart Opitz, Griffin White, Laura Neff, Doris Popovic, Andrea Patrignani, Jay Tracy, Ralph Schlapbach, Christiane Beckmann, Maurice Redondo, Olivier Kobel, Christoph Noppen, Sophie Seidel, Noemie Santamaria de Souza, Niko Beerenwinkel, Tanja Stadler
EPI_ISL_1129464	Viollier AG	Department of Biosystems Science and Engineering, ETH Zürich	Christian Beisel, Sarah Nadeau, Chaoran Chen, Ivan Topolsky, Philipp Jablonski, Lara Fuhrmann, David Dreifuss, Katharina Jahn, Rebecca Denes, Mirjam Feldkamp, Ina Nissen, Natascha Santacroce, Elodie Burcklen, Christiane Beckmann, Maurice Redondo, Olivier Kobel, Christoph Noppen, Sophie Seidel, Noemie Santamaria de Souza, Niko Beerenwinkel, Tanja Stadler
EPI_ISL_1129475, EPI_ISL_1129478, EPI_ISL_1129482, EPI_ISL_1129511, EPI_ISL_1129538, EPI_ISL_1129539, EPI_ISL_1129543, EPI_ISL_1129544, EPI_ISL_1129551, EPI_ISL_1129576, EPI_ISL_1129584, EPI_ISL_1129642, EPI_ISL_1129663, EPI_ISL_1129705, EPI_ISL_1129719			
see above	Viollier AG	Department of Biosystems Science and Engineering, ETH Zürich	Chaoran Chen, Sarah Nadeau, Catharine Aquino, Ivan Topolsky, Philipp Jablonski, Lara Fuhrmann, David Dreifuss, Katharina Jahn, Andreia Cabral de Gouvea, Maria Domenica Moccia, Simon Grüter, Timothy Sykes, Lennart Opitz, Griffin White, Laura Neff, Doris Popovic, Andrea Patrignani, Jay Tracy, Ralph Schlapbach, Christiane Beckmann, Maurice Redondo, Olivier Kobel, Christoph Noppen, Sophie Seidel, Noemie Santamaria de Souza, Niko Beerenwinkel, Tanja Stadler
EPI_ISL_1129772	Viollier AG	Department of Biosystems Science and Engineering, ETH Zürich	Christian Beisel, Sarah Nadeau, Chaoran Chen, Ivan Topolsky, Philipp Jablonski, Lara Fuhrmann, David Dreifuss, Katharina Jahn, Rebecca Denes, Mirjam Feldkamp, Ina Nissen, Natascha Santacroce, Elodie Burcklen, Christiane Beckmann, Maurice Redondo, Olivier Kobel, Christoph Noppen, Sophie Seidel, Noemie Santamaria de Souza, Niko Beerenwinkel, Tanja Stadler
EPI_ISL_1129822, EPI_ISL_1129827, EPI_ISL_1129851, EPI_ISL_1129895	Viollier AG	Department of Biosystems Science and Engineering, ETH Zürich	Chaoran Chen, Sarah Nadeau, Catharine Aquino, Ivan Topolsky, Philipp Jablonski, Lara Fuhrmann, David Dreifuss, Katharina Jahn, Andreia Cabral de Gouvea, Maria Domenica Moccia, Simon Grüter, Timothy Sykes, Lennart Opitz, Griffin White, Laura Neff, Doris Popovic, Andrea Patrignani, Jay Tracy, Ralph Schlapbach, Christiane Beckmann, Maurice Redondo, Olivier Kobel, Christoph Noppen, Sophie Seidel, Noemie Santamaria de Souza, Niko Beerenwinkel, Tanja Stadler
EPI_ISL_1129921	Viollier AG	Department of Biosystems Science and Engineering, ETH Zürich	Christian Beisel, Sarah Nadeau, Chaoran Chen, Ivan Topolsky, Philipp Jablonski, Lara Fuhrmann, David Dreifuss, Katharina Jahn, Rebecca Denes, Mirjam Feldkamp, Ina Nissen, Natascha Santacroce, Elodie Burcklen, Christiane Beckmann, Maurice Redondo, Olivier Kobel, Christoph Noppen, Sophie Seidel, Noemie Santamaria de Souza, Niko Beerenwinkel, Tanja Stadler
EPI_ISL_1129996, EPI_ISL_1130005, EPI_ISL_1130191	Viollier AG	Department of Biosystems Science and Engineering, ETH Zürich	Chaoran Chen, Sarah Nadeau, Catharine Aquino, Ivan Topolsky, Philipp Jablonski, Lara Fuhrmann, David Dreifuss, Katharina Jahn, Andreia Cabral de Gouvea, Maria Domenica Moccia, Simon Grüter, Timothy Sykes, Lennart Opitz, Griffin White, Laura Neff, Doris Popovic, Andrea Patrignani, Jay Tracy, Ralph Schlapbach, Christiane Beckmann, Maurice Redondo, Olivier Kobel, Christoph Noppen, Sophie Seidel, Noemie Santamaria de Souza, Niko Beerenwinkel, Tanja Stadler
EPI_ISL_1130228	Viollier AG	Department of Biosystems Science and Engineering, ETH Zürich	Christian Beisel, Sarah Nadeau, Chaoran Chen, Ivan Topolsky, Philipp Jablonski, Lara Fuhrmann, David Dreifuss, Katharina Jahn, Rebecca Denes, Mirjam Feldkamp, Ina Nissen, Natascha Santacroce, Elodie Burcklen, Christiane Beckmann, Maurice Redondo, Olivier Kobel, Christoph Noppen, Sophie Seidel, Noemie Santamaria de Souza, Niko Beerenwinkel, Tanja Stadler
EPI_ISL_1130293	Viollier AG	Department of Biosystems Science and Engineering, ETH Zürich	Chaoran Chen, Sarah Nadeau, Catharine Aquino, Ivan Topolsky, Philipp Jablonski, Lara Fuhrmann, David Dreifuss, Katharina Jahn, Andreia Cabral de Gouvea, Maria Domenica Moccia, Simon Grüter, Timothy Sykes, Lennart Opitz, Griffin White, Laura Neff, Doris Popovic, Andrea Patrignani, Jay Tracy, Ralph Schlapbach, Christiane Beckmann, Maurice Redondo, Olivier Kobel, Christoph Noppen, Sophie Seidel, Noemie Santamaria de Souza, Niko Beerenwinkel, Tanja Stadler
EPI_ISL_1130360	Viollier AG	Department of Biosystems Science and Engineering, ETH Zürich	Christian Beisel, Sarah Nadeau, Chaoran Chen, Ivan Topolsky, Philipp Jablonski, Lara Fuhrmann, David Dreifuss, Katharina Jahn, Rebecca Denes, Mirjam Feldkamp, Ina Nissen, Natascha Santacroce, Elodie Burcklen, Christiane Beckmann, Maurice Redondo, Olivier Kobel, Christoph Noppen, Sophie Seidel, Noemie Santamaria de Souza, Niko Beerenwinkel, Tanja Stadler
EPI_ISL_1130443, EPI_ISL_1130445	Viollier AG	Department of Biosystems Science and Engineering, ETH	Chaoran Chen, Sarah Nadeau, Catharine Aquino, Ivan Topolsky, Philipp Jablonski, Lara Fuhrmann, David Dreifuss, Katharina Jahn, Andreia Cabral de

		Zürich	Gouvea, Maria Domenica Moccia, Simon Grüter, Timothy Sykes, Lennart Opitz, Griffin White, Laura Neff, Doris Popovic, Andrea Patrignani, Jay Tracy, Ralph Schlapbach, Christiane Beckmann, Maurice Redondo, Olivier Kobel, Christoph Noppen, Sophie Seidel, Noemie Santamaria de Souza, Niko Beerenwinkel, Tanja Stadler
EPI_ISL_1130451, EPI_ISL_1130579	Viollier AG	Department of Biosystems Science and Engineering, ETH Zürich	Christian Beisel, Sarah Nadeau, Chaoran Chen, Ivan Topolsky, Philipp Jablonski, Lara Fuhrmann, David Dreifuss, Katharina Jahn, Rebecca Denes, Mirjam Feldkamp, Ina Nissen, Natascha Santacroce, Elodie Burcklen, Christiane Beckmann, Maurice Redondo, Olivier Kobel, Christoph Noppen, Sophie Seidel, Noemie Santamaria de Souza, Niko Beerenwinkel, Tanja Stadler
EPI_ISL_1130662, EPI_ISL_1130754, EPI_ISL_1130787, EPI_ISL_1130795	Viollier AG	Department of Biosystems Science and Engineering, ETH Zürich	Chaoran Chen, Sarah Nadeau, Catharine Aquino, Ivan Topolsky, Philipp Jablonski, Lara Fuhrmann, David Dreifuss, Katharina Jahn, Andreia Cabral de Gouvea, Maria Domenica Moccia, Simon Grüter, Timothy Sykes, Lennart Opitz, Griffin White, Laura Neff, Doris Popovic, Andrea Patrignani, Jay Tracy, Ralph Schlapbach, Christiane Beckmann, Maurice Redondo, Olivier Kobel, Christoph Noppen, Sophie Seidel, Noemie Santamaria de Souza, Niko Beerenwinkel, Tanja Stadler
EPI_ISL_1130816	Viollier AG	Department of Biosystems Science and Engineering, ETH Zürich	Chaoran Chen, Sarah Nadeau, Ivan Topolsky, Emmanouil Dermitzakis, Keith Harshman, Ioannis Xenarios, Henri Pegeot, Lorenzo Cerutti, Deborah Penet, Philipp Jablonski, Lara Fuhrmann, David Dreifuss, Katharina Jahn, Christiane Beckmann, Maurice Redondo, Olivier Kobel, Christoph Noppen, Sophie Seidel, Noemie Santamaria de Souza, Niko Beerenwinkel, Tanja Stadler
EPI_ISL_1130830, EPI_ISL_1130841, EPI_ISL_1130921	Viollier AG	Department of Biosystems Science and Engineering, ETH Zürich	Chaoran Chen, Sarah Nadeau, Catharine Aquino, Ivan Topolsky, Philipp Jablonski, Lara Fuhrmann, David Dreifuss, Katharina Jahn, Andreia Cabral de Gouvea, Maria Domenica Moccia, Simon Grüter, Timothy Sykes, Lennart Opitz, Griffin White, Laura Neff, Doris Popovic, Andrea Patrignani, Jay Tracy, Ralph Schlapbach, Christiane Beckmann, Maurice Redondo, Olivier Kobel, Christoph Noppen, Sophie Seidel, Noemie Santamaria de Souza, Niko Beerenwinkel, Tanja Stadler
EPI_ISL_1130978	Viollier AG	Department of Biosystems Science and Engineering, ETH Zürich	Chaoran Chen, Sarah Nadeau, Ivan Topolsky, Emmanouil Dermitzakis, Keith Harshman, Ioannis Xenarios, Henri Pegeot, Lorenzo Cerutti, Deborah Penet, Philipp Jablonski, Lara Fuhrmann, David Dreifuss, Katharina Jahn, Christiane Beckmann, Maurice Redondo, Olivier Kobel, Christoph Noppen, Sophie Seidel, Noemie Santamaria de Souza, Niko Beerenwinkel, Tanja Stadler
EPI_ISL_1131013, EPI_ISL_1131014	Viollier AG	Department of Biosystems Science and Engineering, ETH Zürich	Christian Beisel, Sarah Nadeau, Chaoran Chen, Ivan Topolsky, Philipp Jablonski, Lara Fuhrmann, David Dreifuss, Katharina Jahn, Rebecca Denes, Mirjam Feldkamp, Ina Nissen, Natascha Santacroce, Elodie Burcklen, Christiane Beckmann, Maurice Redondo, Olivier Kobel, Christoph Noppen, Sophie Seidel, Noemie Santamaria de Souza, Niko Beerenwinkel, Tanja Stadler
EPI_ISL_1131132	Viollier AG	Department of Biosystems Science and Engineering, ETH Zürich	Chaoran Chen, Sarah Nadeau, Catharine Aquino, Ivan Topolsky, Philipp Jablonski, Lara Fuhrmann, David Dreifuss, Katharina Jahn, Andreia Cabral de Gouvea, Maria Domenica Moccia, Simon Grüter, Timothy Sykes, Lennart Opitz, Griffin White, Laura Neff, Doris Popovic, Andrea Patrignani, Jay Tracy, Ralph Schlapbach, Christiane Beckmann, Maurice Redondo, Olivier Kobel, Christoph Noppen, Sophie Seidel, Noemie Santamaria de Souza, Niko Beerenwinkel, Tanja Stadler
EPI_ISL_1131140, EPI_ISL_1131141, EPI_ISL_1131146, EPI_ISL_1131157, EPI_ISL_1131158	Pathogen Genomics Center, National Institute of Infectious Diseases	Pathogen Genomics Center, National Institute of Infectious Diseases	Tsuyoshi Sekizuka, Kentaro Itokawa, Rina Tanaka, Masanori Hashino, Makoto Kuroda
EPI_ISL_1131164, EPI_ISL_1131165, EPI_ISL_1131166, EPI_ISL_1131167, EPI_ISL_1131168, EPI_ISL_1131169, EPI_ISL_1131170, EPI_ISL_1131171, EPI_ISL_1131172, EPI_ISL_1131173	Niigata Prefectural Institute of Public Health and Environmental Sciences	Pathogen Genomics Center, National Institute of Infectious Diseases	Tsuyoshi Sekizuka, Kentaro Itokawa, Rina Tanaka, Masanori Hashino, Makoto Kuroda
EPI_ISL_1131186, EPI_ISL_1131216, EPI_ISL_1131217, EPI_ISL_1131218, EPI_ISL_1131219, EPI_ISL_1131220, EPI_ISL_1131221, EPI_ISL_1131222, EPI_ISL_1131223, EPI_ISL_1131224, EPI_ISL_1131250, EPI_ISL_1131251, EPI_ISL_1131252, EPI_ISL_1131253, EPI_ISL_1131254, EPI_ISL_1131255, EPI_ISL_1131256, EPI_ISL_1131257, EPI_ISL_1131258, EPI_ISL_1131259, EPI_ISL_1131260, EPI_ISL_1131261, EPI_ISL_1131262, EPI_ISL_1131263, EPI_ISL_1131264, EPI_ISL_1131265, EPI_ISL_1131266, EPI_ISL_1131267, EPI_ISL_1131268, EPI_ISL_1131269, EPI_ISL_1131270, EPI_ISL_1131271, EPI_ISL_1131272, EPI_ISL_1131273, EPI_ISL_1131274, EPI_ISL_1131275, EPI_ISL_1131276, EPI_ISL_1131277, EPI_ISL_1131278, EPI_ISL_1131279, EPI_ISL_1131280, EPI_ISL_1131281, EPI_ISL_1131282, EPI_ISL_1131283, EPI_ISL_1131284, EPI_ISL_1131285, EPI_ISL_1131286, EPI_ISL_1131287, EPI_ISL_1131288, EPI_ISL_1131289, EPI_ISL_1131290, EPI_ISL_1131291, EPI_ISL_1131292, EPI_ISL_1131293, EPI_ISL_1131294, EPI_ISL_1131295, EPI_ISL_1131296, EPI_ISL_1131297, EPI_ISL_1131298, EPI_ISL_1131299, EPI_ISL_1131300, EPI_ISL_1131301, EPI_ISL_1131302, EPI_ISL_1131303, EPI_ISL_1131304, EPI_ISL_1131305, EPI_ISL_1131306, EPI_ISL_1131307, EPI_ISL_1131308, EPI_ISL_1131309, EPI_ISL_1131310, EPI_ISL_1131311, EPI_ISL_1131312, EPI_ISL_1131313, EPI_ISL_1131314, EPI_ISL_1131315, EPI_ISL_1131316, EPI_ISL_1131317, EPI_ISL_1131318, EPI_ISL_1131319, EPI_ISL_1131320, EPI_ISL_1131321, EPI_ISL_1131322, EPI_ISL_1131323, EPI_ISL_1131324, EPI_ISL_1131325, EPI_ISL_1131326, EPI_ISL_1131327, EPI_ISL_1131328, EPI_ISL_1131329, EPI_ISL_1131330, EPI_ISL_1131331, EPI_ISL_1131332, EPI_ISL_1131333, EPI_ISL_1131334, EPI_ISL_1131335, EPI_ISL_1131336, EPI_ISL_1131337, EPI_ISL_1131338, EPI_ISL_1131339, EPI_ISL_1131340, EPI_ISL_1131341, EPI_ISL_1131342, EPI_ISL_1131343, EPI_ISL_1131344, EPI_ISL_1131345, EPI_ISL_1131346, EPI_ISL_1131347, EPI_ISL_1131348, EPI_ISL_1131349, EPI_ISL_1131350, EPI_ISL_1131351, EPI_ISL_1131352, EPI_ISL_1131353, EPI_ISL_1131354, EPI_ISL_1131355, EPI_ISL_1131356, EPI_ISL_1131357, EPI_ISL_1131358, EPI_ISL_1131359, EPI_ISL_1131360, EPI_ISL_1131361, EPI_ISL_1131362, EPI_ISL_1131363, EPI_ISL_1131364, EPI_ISL_1131365, EPI_ISL_1131366, EPI_ISL_1131367, EPI_ISL_1131368, EPI_ISL_1131369, EPI_ISL_1131370, EPI_ISL_1131371, EPI_ISL_1131372, EPI_ISL_1131373, EPI_ISL_1131374, EPI_ISL_1131375, EPI_ISL_1131376, EPI_ISL_1131377, EPI_ISL_1131378, EPI_ISL_1131379, EPI_ISL_1131381, EPI_ISL_1131382, EPI_ISL_1131383, EPI_ISL_1131384, EPI_ISL_1131385, EPI_ISL_1131386, EPI_ISL_1131387, EPI_ISL_1131388, EPI_ISL_1131389, EPI_ISL_1131390, EPI_ISL_1131391, EPI_ISL_1131392, EPI_ISL_1131393, EPI_ISL_1131394, EPI_ISL_1131395, EPI_ISL_1131396, EPI_ISL_1131397, EPI_ISL_1131398, EPI_ISL_1131399, EPI_ISL_1131400, EPI_ISL_1131401			
see above	Pathogen Genomics Center, National Institute of Infectious Diseases	Pathogen Genomics Center, National Institute of Infectious Diseases	Tsuyoshi Sekizuka, Kentaro Itokawa, Rina Tanaka, Masanori Hashino, Makoto Kuroda
EPI_ISL_1131412, EPI_ISL_1131413, EPI_ISL_1131414, EPI_ISL_1131415, EPI_ISL_1131416, EPI_ISL_1131417	Osaka Institute of Public Health, Morinomiya Center	Pathogen Genomics Center, National Institute of Infectious Diseases	Tsuyoshi Sekizuka, Kentaro Itokawa, Rina Tanaka, Masanori Hashino, Makoto Kuroda
EPI_ISL_1131446, EPI_ISL_1131447, EPI_ISL_1131448, EPI_ISL_1131449, EPI_ISL_1131450, EPI_ISL_1131451, EPI_ISL_1131452, EPI_ISL_1131456, EPI_ISL_1131457, EPI_ISL_1131458, EPI_ISL_1131459, EPI_ISL_1131460, EPI_ISL_1131461, EPI_ISL_1131462, EPI_ISL_1131463, EPI_ISL_1131464, EPI_ISL_1131465, EPI_ISL_1131466, EPI_ISL_1131467, EPI_ISL_1131468, EPI_ISL_1131469, EPI_ISL_1131470, EPI_ISL_1131471, EPI_ISL_1131472, EPI_ISL_1131473, EPI_ISL_1131474, EPI_ISL_1131475, EPI_ISL_1131476, EPI_ISL_1131477, EPI_ISL_1131478, EPI_ISL_1131479, EPI_ISL_1131480, EPI_ISL_1131481, EPI_ISL_1131482, EPI_ISL_1131483, EPI_ISL_1131484, EPI_ISL_1131485, EPI_ISL_1131486, EPI_ISL_1131487, EPI_ISL_1131488, EPI_ISL_1131489, EPI_ISL_1131490, EPI_ISL_1131491, EPI_ISL_1131492, EPI_ISL_1131493, EPI_ISL_1131494, EPI_ISL_1131495, EPI_ISL_1131496, EPI_ISL_1131497, EPI_ISL_1131498, EPI_ISL_1131499, EPI_ISL_1131500, EPI_ISL_1131501, EPI_ISL_1131502, EPI_ISL_1131503, EPI_ISL_1131504, EPI_ISL_1131505, EPI_ISL_1131506, EPI_ISL_1131507, EPI_ISL_1131508, EPI_ISL_1131509, EPI_ISL_1131510, EPI_ISL_1131511, EPI_ISL_1131536, EPI_ISL_1131537, EPI_ISL_1131538, EPI_ISL_1131561, EPI_ISL_1131562, EPI_ISL_1131563, EPI_ISL_1131564, EPI_ISL_1131565, EPI_ISL_1131566, EPI_ISL_1131567, EPI_ISL_1131568, EPI_ISL_1131569, EPI_ISL_1131570, EPI_ISL_1131571, EPI_ISL_1131572, EPI_ISL_1131573, EPI_ISL_1131574, EPI_ISL_1131575, EPI_ISL_1131576, EPI_ISL_1131577, EPI_ISL_1131578, EPI_ISL_1131579, EPI_ISL_1131580, EPI_ISL_1131581, EPI_ISL_1131582, EPI_ISL_1131583, EPI_ISL_1131584, EPI_ISL_1131585, EPI_ISL_1131586, EPI_ISL_1131587, EPI_ISL_1131588, EPI_ISL_1131589, EPI_ISL_1131590, EPI_ISL_1131591, EPI_ISL_1131592, EPI_ISL_1131715, EPI_ISL_1131716, EPI_ISL_1131717, EPI_ISL_1131718, EPI_ISL_1131719, EPI_ISL_1131720, EPI_ISL_1131721, EPI_ISL_1131722, EPI_ISL_1131723, EPI_ISL_1131724, EPI_ISL_1131725, EPI_ISL_1131726, EPI_ISL_1131727, EPI_ISL_1131728, EPI_ISL_1131729, EPI_ISL_1131730, EPI_ISL_1131731, EPI_ISL_1131732, EPI_ISL_1131733, EPI_ISL_1131734, EPI_ISL_1131735, EPI_ISL_1131736, EPI_ISL_1131737, EPI_ISL_1131738, EPI_ISL_1131739, EPI_ISL_1131740, EPI_ISL_1131741, EPI_ISL_1131742, EPI_ISL_1131743, EPI_ISL_1131744, EPI_ISL_1131745, EPI_ISL_1131746, EPI_ISL_1131747, EPI_ISL_1131748, EPI_ISL_1131749, EPI_ISL_1131750, EPI_ISL_1131751, EPI_ISL_1131752, EPI_ISL_1131753, EPI_ISL_1131754, EPI_ISL_1131755, EPI_ISL_1131756, EPI_ISL_1131757, EPI_ISL_1131758, EPI_ISL_1131759, EPI_ISL_1131760, EPI_ISL_1131761, EPI_ISL_1131762, EPI_ISL_1131763, EPI_ISL_1131764, EPI_ISL_1131765, EPI_ISL_1131766, EPI_ISL_1131767, EPI_ISL_1131768, EPI_ISL_1131769, EPI_ISL_1131770, EPI_ISL_1131771, EPI_ISL_1131772, EPI_ISL_1131773, EPI_ISL_1131774, EPI_ISL_1131775, EPI_ISL_1131776, EPI_ISL_1131777, EPI_ISL_1131778, EPI_ISL_1131779, EPI_ISL_1131780, EPI_ISL_1131781			
see above	Pathogen Genomics Center, National Institute of Infectious Diseases	Pathogen Genomics Center, National Institute of Infectious Diseases	Tsuyoshi Sekizuka, Kentaro Itokawa, Rina Tanaka, Masanori Hashino, Makoto Kuroda
EPI_ISL_1131850, EPI_ISL_1131851, EPI_ISL_1131852, EPI_ISL_1131853, EPI_ISL_1131854, EPI_ISL_1131855, EPI_ISL_1131856, EPI_ISL_1131857, EPI_ISL_1131858, EPI_ISL_1131859, EPI_ISL_1131860			
see above	Niigata Prefectural Institute of Public Health and Environmental Sciences	Pathogen Genomics Center, National Institute of Infectious Diseases	Tsuyoshi Sekizuka, Kentaro Itokawa, Rina Tanaka, Masanori Hashino, Makoto Kuroda
EPI_ISL_1131861, EPI_ISL_1131862, EPI_ISL_1131863, EPI_ISL_1131864, EPI_ISL_1131865, EPI_ISL_1131866, EPI_ISL_1131867, EPI_ISL_1131868, EPI_ISL_1131869, EPI_ISL_1131870, EPI_ISL_1131871, EPI_ISL_1131872, EPI_ISL_1131873, EPI_ISL_1131874, EPI_ISL_1131875, EPI_ISL_1131876, EPI_ISL_1131877, EPI_ISL_1131878, EPI_ISL_1131879, EPI_ISL_1131880, EPI_ISL_1131881, EPI_ISL_1131882, EPI_ISL_1131883, EPI_ISL_1131884, EPI_ISL_1131885, EPI_ISL_1131886, EPI_ISL_1131887, EPI_ISL_1131888, EPI_ISL_1131889, EPI_ISL_1132005, EPI_ISL_1132006, EPI_ISL_1132007, EPI_ISL_1132008, EPI_ISL_1132009, EPI_ISL_1132010, EPI_ISL_1132011, EPI_ISL_1132012, EPI_ISL_1132013, EPI_ISL_1132014, EPI_ISL_1132015, EPI_ISL_1132016, EPI_ISL_1132017, EPI_ISL_1132018, EPI_ISL_1132019, EPI_ISL_1132020, EPI_ISL_1132021, EPI_ISL_1132022, EPI_ISL_1132023, EPI_ISL_1132024, EPI_ISL_1132025, EPI_ISL_1132026, EPI_ISL_1132027, EPI_ISL_1132028, EPI_ISL_1132029, EPI_ISL_1132130, EPI_ISL_1132131, EPI_ISL_1132132			
see above	Pathogen Genomics Center, National Institute of Infectious Diseases	Pathogen Genomics Center, National Institute of Infectious Diseases	Tsuyoshi Sekizuka, Kentaro Itokawa, Rina Tanaka, Masanori Hashino, Makoto Kuroda
EPI_ISL_1132671, EPI_ISL_1132672, EPI_ISL_1132720, EPI_ISL_1132721, EPI_ISL_1132728	Vaccines and Infectious Diseases Analytics Research Unit (VIDA)	KRISP, KZn Research Innovation and Sequencing Platform	Baillie Vicky, du Plessis Jeanine, Giandhari Jennifer, Pillay Sureshnee, Naidoo Yeshnee, Tegally Houriiyah, de Oliveira Tulio, Madhi Shabir
EPI_ISL_1133253	University of Chittagong	Central Biological Research Laboratory and Department of Biochemistry and Molecular Biology	Mohammad Omar Faruque, H. M. Abdullah Al Masud, Imam Hossen, Md. Khondakar Raziur Rahman, Sajib Rudra, Md. Arif Hossain, Shanta Paul, Md. Omer Faruq, Robiul Hasan Bhuiyan, Md. Imranul Hoq
EPI_ISL_1133254	University of Chittagong	Central Biological Research Laboratory and Department of	H. M. Abdullah Al Masud, Mohammad Omar Faruque, Sajib Rudra, Md. Khondakar Raziur Rahman, Imam Hossen, Md. Arif Hossain, Shanta Paul, Md.

		Biochemistry and Molecular Biology	Omer Faruq, Md. Imranul Hoq, Robiul Hasan Bhuiyan
EPI_ISL_1135111	Arizona State Public Health Laboratory	Arizona State Public Health Laboratory	Trung Huynh, Jessica Escobar, Katherine Fullerton, Nobuko Fukushima, Stacy White, Linda Getsinger, Victor Waddell
EPI_ISL_1137005, EPI_ISL_1137010, EPI_ISL_1137027, EPI_ISL_1137028, EPI_ISL_1137029, EPI_ISL_1137035, EPI_ISL_1137036, EPI_ISL_1137037	Delaware Public Health Lab	Delaware Public Health Lab	Gregory Hovan
EPI_ISL_1137073	Kansas Health and Environmental Lab	Kansas Health and Environmental Lab	Mike Grose, Jonathan Barnell, Ben Olsen, and Phil Adam
EPI_ISL_1137195, EPI_ISL_1137196, EPI_ISL_1137197, EPI_ISL_1137198, EPI_ISL_1137199, EPI_ISL_1137200, EPI_ISL_1137201, EPI_ISL_1137202, EPI_ISL_1137203, EPI_ISL_1137205, EPI_ISL_1137206, EPI_ISL_1137208			
see above	UPMC Clinical Microbiology Laboratory	Microbial Genome Sequencing Center; Microbial Genomic Epidemiology Laboratory, University of Pittsburgh	Lee H. Harrison, Jane W. Marsh, Marissa P. Griffith, Stephanie L. Mitchell, Vatsala R. Srinivasa, Kady D. Waggle, Daniel J. Snyder, Vaughn S. Cooper
EPI_ISL_1137605, EPI_ISL_1137606, EPI_ISL_1137607	Wyoming Public Health Laboratory	Wyoming Public Health Laboratory	Jim Mildenberger, Wanda Manley, Noah Hull, Taylor Fearing, Lynette Gumbleton, Channing Weber, Ashley Norberg, Chayse Rowley, Marley Goetz, Brian Dominguez, Elliot Thomasson, Cari Sloma, and Rob Christensen
EPI_ISL_1138411	Hospital HIA Percy, Medical Biology	National Reference Center for Viruses of Respiratory Infections, Institut Pasteur, Paris	Marion Barbet, Sylvie Behillil, Méline Bizard, Angela Brisebarre, Camille Capel, Etienne Simon-Lorière, Vincent Enouf, Maud Vanpeene, Sylvie van der Werf,Vincent Foissaud
EPI_ISL_1138422	Laboratorio de Referencia Nacional de Virus Respiratorio. Instituto Nacional de Salud Perú	Laboratorio de Referencia Nacional de Biotecnología y Biología Molecular. Instituto Nacional de Salud Perú	Carlos Padilla Rojas, Karolyn Vega Chozo, Luis Barcena, Priscila Lope Pari, Omar Caceres Rey, Marco Galarza Perez, Maribel Huaringa Nuñez, Johanna Balbuena Torrez, Henri Bailon Calderon, Nancy Rojas Serrano
EPI_ISL_1138513, EPI_ISL_1138514, EPI_ISL_1138515	SA Pärnu Hospital Laboratory	1. Laboratory of Communicable Diseases (Estonia); 2. Eurofins Genomics Europe Sequencing GmbH	Liidia Dotsenko
EPI_ISL_1138612, EPI_ISL_1138619, EPI_ISL_1138626, EPI_ISL_1138633, EPI_ISL_1138668, EPI_ISL_1138683, EPI_ISL_1138686, EPI_ISL_1138702, EPI_ISL_1138705, EPI_ISL_1138710, EPI_ISL_1138712, EPI_ISL_1138717, EPI_ISL_1138718, EPI_ISL_1138720			
see above	Institut für Medizinische Virologie, Universitätsklinikum Frankfurt	Institut für Medizinische Virologie, Universitätsklinikum Frankfurt	Barbara Muehlemann et al
EPI_ISL_1140394, EPI_ISL_1140395, EPI_ISL_1140396, EPI_ISL_1140400, EPI_ISL_1140403, EPI_ISL_1140406, EPI_ISL_1140410, EPI_ISL_1140412, EPI_ISL_1140413, EPI_ISL_1140459, EPI_ISL_1140460, EPI_ISL_1140464, EPI_ISL_1140467, EPI_ISL_1140468, EPI_ISL_1140469, EPI_ISL_1140472, EPI_ISL_1140473			
see above	Labor ZOTZ|KLIMAS; MVZ Düsseldorf-Centrum	Robert Koch Institute	unknown
EPI_ISL_1143962, EPI_ISL_1143977	Bayerisches Landesamt für Gesundheit und Lebensmittelsicherheit (LGL)	Robert Koch Institute	unknown
EPI_ISL_1144923, EPI_ISL_1144924, EPI_ISL_1144925, EPI_ISL_1144927, EPI_ISL_1144930, EPI_ISL_1144931, EPI_ISL_1144934, EPI_ISL_1144936, EPI_ISL_1144937	Medizinische Hochschule Hannover Institut für Virologie	Robert Koch Institute	unknown
EPI_ISL_1147655	Uniklinikum Jena	Robert Koch Institute	unknown
EPI_ISL_1157006, EPI_ISL_1157008, EPI_ISL_1157009, EPI_ISL_1157011, EPI_ISL_1157013, EPI_ISL_1157017, EPI_ISL_1157020, EPI_ISL_1157022, EPI_ISL_1157024, EPI_ISL_1157025, EPI_ISL_1157027, EPI_ISL_1157031, EPI_ISL_1157033			
see above	Virginia Division of Consolidated Laboratory Services	Virginia Division of Consolidated Laboratory Services	Virginia DCLS
EPI_ISL_1157071, EPI_ISL_1157072, EPI_ISL_1157073, EPI_ISL_1157074, EPI_ISL_1157075, EPI_ISL_1157076, EPI_ISL_1157077, EPI_ISL_1157079, EPI_ISL_1157080, EPI_ISL_1157081, EPI_ISL_1157083, EPI_ISL_1157085, EPI_ISL_1157086, EPI_ISL_1157087, EPI_ISL_1157088, EPI_ISL_1157089, EPI_ISL_1157092, EPI_ISL_1157093, EPI_ISL_1157094, EPI_ISL_1157097, EPI_ISL_1157098, EPI_ISL_1157099			
see above	Eurofins LifeCodexx GmbH	Robert Koch Institute	unknown
EPI_ISL_1159104, EPI_ISL_1159105	Department of Health Technology and Informatics, The Hong Kong Polytechnic University	Department of Health Technology and Informatics, The Hong Kong Polytechnic University	Gilman Kit-Hang Siu, Lam-Kwong Lee, Kenneth Siu-Sing Leung, Jake Siu-Lun Leung, Timothy Ting-Leung Ng, Chloe Toi-Mei Chan, Kingsley King-Gee Tam, Hiu-Yin Lao, Denise Sze-Hang Wong, Alan Ka-Lun Wu, Miranda Chong-Yee Yau, Yvette Wai-Man Lai, Kitty Sau-Chun Fung, Sandy Ka-Yee Chau, Barry Kin-Chung Wong, Wing-Kin To, Kristine Luk, Alex Yat-Man Ho, Tak-Lun Que, Kam-Tong Yip, Wing Cheong Yam, David Ho-Keung Shum, Shea Ping Yip
EPI_ISL_1159778, EPI_ISL_1159792, EPI_ISL_1159804	Wisconsin State Laboratory of Hygiene Communicable Disease Division	Wisconsin State Laboratory of Hygiene Communicable Disease Division	Kelsey R. Florek, Abigail C. Shockey
EPI_ISL_1163533, EPI_ISL_1163534, EPI_ISL_1163535, EPI_ISL_1163536, EPI_ISL_1163537, EPI_ISL_1163539, EPI_ISL_1163540, EPI_ISL_1163541, EPI_ISL_1163542, EPI_ISL_1163543, EPI_ISL_1163544, EPI_ISL_1163546, EPI_ISL_1163547, EPI_ISL_1163548, EPI_ISL_1163549			
see above	Maine HETL	Tewhey Lab, The Jackson Laboratory	Matluk,N., Dewey,H., Iosue,F., Barter,M., Lynch,R., Munger,H. and Tewhey,R.
EPI_ISL_1164699, EPI_ISL_1164700, EPI_ISL_1164701, EPI_ISL_1164702, EPI_ISL_1164703, EPI_ISL_1164704, EPI_ISL_1164705, EPI_ISL_1164706, EPI_ISL_1164707, EPI_ISL_1164708, EPI_ISL_1164709, EPI_ISL_1164710, EPI_ISL_1164711, EPI_ISL_1164712, EPI_ISL_1164713, EPI_ISL_1164716, EPI_ISL_1164717, EPI_ISL_1164718, EPI_ISL_1164719, EPI_ISL_1164720, EPI_ISL_1164722, EPI_ISL_1164724, EPI_ISL_1164725, EPI_ISL_1164726, EPI_ISL_1164727			
see above	Department of Molecular Virology, Cyprus Institute of Neurology and Genetics	Department of Molecular Virology, Cyprus Institute of Neurology and Genetics	Jan Richter, Pavlos Fanis, Christina Tryfonos, Dana Koptides, George Krashias, Stavros Bashiardes, Andreas Hadjisavvas, Maria Loizidou, Anastasis Oulas, Denise Alexandrou, Olga Kalakouta, Mihalis Panayiotidis, George Spyrou, Christina Christodoulou
EPI_ISL_1164994	LACEN - Laboratório Central de Saúde Pública do Paraiba	Evandro Chagas Institute	Santos, M.C.; Silva, A.M.; Junior, W.D.C.; Barbagelata, L.S.; Ferreira, J.A.; Sousa, E.M.A.; da Silva, P.S.; Pinheiro, K.C.; L.C.; Sousa Junior, E.C.
EPI_ISL_1164995	LACEN - Laboratório Central de Saúde Pública do Ceará	Evandro Chagas Institute	Santos, M.C.; Silva, A.M.; Junior, W.D.C.; Barbagelata, L.S.; Ferreira, J.A.; Sousa, E.M.A.; da Silva, P.S.; Pinheiro, K.C.; L.C.; Sousa Junior, E.C.
EPI_ISL_1164996	LACEN - Laboratório Central de Saúde Pública do Maranhao	Evandro Chagas Institute	Santos, M.C.; Silva, A.M.; Junior, W.D.C.; Barbagelata, L.S.; Ferreira, J.A.; Sousa, E.M.A.; da Silva, P.S.; Pinheiro, K.C.; L.C.; Sousa Junior, E.C.
EPI_ISL_1164999	LACEN - Laboratório Central de Saúde Pública do Paraiba	Evandro Chagas Institute	Santos, M.C.; Silva, A.M.; Junior, W.D.C.; Barbagelata, L.S.; Ferreira, J.A.; Sousa, E.M.A.; da Silva, P.S.; Pinheiro, K.C.; L.C.; Sousa Junior, E.C.
EPI_ISL_1167135, EPI_ISL_1167141, EPI_ISL_1167145, EPI_ISL_1167146	Iressef Genomics lab	L'institut de Recherche en Santé, de Surveillance Épidémiologique et de Formation (IRESSEF)	Souleymane MBOUP, Abdou PADANE, Abdoulie KANTEH ,Abdul Karim SESAY , Khadim GUEYE, Papa Alassane DIAW, Birahim Piere NDIAYE, Barada CISSE, Aminata MBOUP, Moustapha MBOW, Ndeye Coumba Toure KANE, Nafisatou LEYE, Gora LO, Ambroise AHOUIDI , Astou Gaye GAYE, Aminata DIA, Yacine DIA
EPI_ISL_1167703, EPI_ISL_1167753, EPI_ISL_1167756, EPI_ISL_1167790, EPI_ISL_1167796, EPI_ISL_1167810, EPI_ISL_1167829, EPI_ISL_1167847, EPI_ISL_1167863, EPI_ISL_1167866, EPI_ISL_1167877, EPI_ISL_1167878, EPI_ISL_1167879, EPI_ISL_1167885, EPI_ISL_1167887, EPI_ISL_1167924, EPI_ISL_1167928, EPI_ISL_1167930			
see above	Genetica Molecular and Subdepartamento de Virologia ISP Chile	Instituto de Salud Publica de Chile	Javier Tognarelli, Karen Orostica, Barbara Parra, Loredana Arata, Jaime Lagos, Gisselle Barra, Patricia Bustos, Rodrigo Fasce, Andres Castillo, Jorge Fernandez
EPI_ISL_1167940, EPI_ISL_1167941, EPI_ISL_1167944, EPI_ISL_1167947, EPI_ISL_1167948, EPI_ISL_1167950, EPI_ISL_1167952, EPI_ISL_1167953, EPI_ISL_1167954, EPI_ISL_1167957, EPI_ISL_1167959, EPI_ISL_1167962, EPI_ISL_1167966, EPI_ISL_1167971, EPI_ISL_1167972, EPI_ISL_1167974, EPI_ISL_1167977, EPI_ISL_1167980, EPI_ISL_1167982, EPI_ISL_1167985, EPI_ISL_1167987			
see above	GA Department of Public Health Laboratory	Genomics and Discovery, Respiratory Viruses Branch, Division of Viral Diseases, Centers for Disease Control and Prevention	Brian Lynch, Krista Queen, Yan Li, Ying Tao, Jing Zhang, Anna Uehara, Anna Montmayeur, Clinton R. Paden, Peter W. Cook, Rachel Marine, Haibin Wang,, Suxiang Tong
EPI_ISL_1167990, EPI_ISL_1167991, EPI_ISL_1167993, EPI_ISL_1167996	MO State Public Health Laboratory	Genomics and Discovery, Respiratory Viruses Branch, Division of Viral Diseases, Centers for Disease Control and Prevention	Brian Lynch, Krista Queen, Yan Li, Ying Tao, Jing Zhang, Anna Uehara, Anna Montmayeur, Clinton R. Paden, Peter W. Cook, Rachel Marine, Haibin Wang,, Suxiang Tong
EPI_ISL_1168001, EPI_ISL_1168003, EPI_ISL_1168006	GA Department of Public Health Laboratory	Genomics and Discovery, Respiratory Viruses Branch, Division of Viral Diseases, Centers for Disease Control and	Brian Lynch, Krista Queen, Yan Li, Ying Tao, Jing Zhang, Anna Uehara, Anna Montmayeur, Clinton R. Paden, Peter W. Cook, Rachel Marine, Haibin Wang,, Suxiang Tong

		Prevention	
EPI_ISL_1168007, EPI_ISL_1168014	MO State Public Health Laboratory	Genomics and Discovery, Respiratory Viruses Branch, Division of Viral Diseases, Centers for Disease Control and Prevention	Brian Lynch, Krista Queen, Yan Li, Ying Tao, Jing Zhang, Anna Uehara, Anna Montmayeur, Clinton R. Paden, Peter W. Cook, Rachel Marine, Haibin Wang,, Suxiang Tong
EPI_ISL_1168449	DC Public Health Lab/ Dept. of Forensic Sciences	DC Public Health Lab/ Dept. of Forensic Sciences	Scott Nguyen, Janis Doss, Elizabeth Zelaya, Sarah Scott, Connie Maza, Monica Mann, Brittany Hamilton, David Payne, Jocelyn Hauser
EPI_ISL_1168658, EPI_ISL_1168659, EPI_ISL_1168666, EPI_ISL_1168672, EPI_ISL_1168675	Centers for Disease Control and Prevention, Dengue Branch	Centers for Disease Control and Prevention, Dengue Branch	Gilberto A. Santiago, Glenda Gonzalez, Betzabel Flores, Keyla Charriez, Gabriela Paz-Bailey, Jorge L. Munoz-Jordan
EPI_ISL_1168770, EPI_ISL_1168771	Beijing Center for Disease Prevention and Control	Beijing Center for Disease Prevention and Control	Yang Pan, Fu Li, Zhaomin Feng, Shujuan Cui, Bing Lyu, Zhichao Liang, Lijuan Chen, Daitao Zhang, Quanyi Wang
EPI_ISL_1168773, EPI_ISL_1168774, EPI_ISL_1168778, EPI_ISL_1168779, EPI_ISL_1168780, EPI_ISL_1168781, EPI_ISL_1168782, EPI_ISL_1168783, EPI_ISL_1168784, EPI_ISL_1168785, EPI_ISL_1168786, EPI_ISL_1168787, EPI_ISL_1168788, EPI_ISL_1168789, EPI_ISL_1168790, EPI_ISL_1168791, EPI_ISL_1168792, EPI_ISL_1168800, EPI_ISL_1168801, EPI_ISL_1168802, EPI_ISL_1168803, EPI_ISL_1168804, EPI_ISL_1168805, EPI_ISL_1168806, EPI_ISL_1168807, EPI_ISL_1168808, EPI_ISL_1168809, EPI_ISL_1168810, EPI_ISL_1168811, EPI_ISL_1168812, EPI_ISL_1168813, EPI_ISL_1168814, EPI_ISL_1168815, EPI_ISL_1168816, EPI_ISL_1168817, EPI_ISL_1168818, EPI_ISL_1168819, EPI_ISL_1168820, EPI_ISL_1168821, EPI_ISL_1168822, EPI_ISL_1168823, EPI_ISL_1168824, EPI_ISL_1168825, EPI_ISL_1168826, EPI_ISL_1168827, EPI_ISL_1168828, EPI_ISL_1168829, EPI_ISL_1168830, EPI_ISL_1168831, EPI_ISL_1168832, EPI_ISL_1168833, EPI_ISL_1168834, EPI_ISL_1168835, EPI_ISL_1168836, EPI_ISL_1168837, EPI_ISL_1168838, EPI_ISL_1168839, EPI_ISL_1168840, EPI_ISL_1168841, EPI_ISL_1168842, EPI_ISL_1168845, EPI_ISL_1168847, EPI_ISL_1168848, EPI_ISL_1168849, EPI_ISL_1168850, EPI_ISL_1168851, EPI_ISL_1168852, EPI_ISL_1168853, EPI_ISL_1168854, EPI_ISL_1168858, EPI_ISL_1168859, EPI_ISL_1168860, EPI_ISL_1168861, EPI_ISL_1168862, EPI_ISL_1168863, EPI_ISL_1168864, EPI_ISL_1168865, EPI_ISL_1168867, EPI_ISL_1168868, EPI_ISL_1168869, EPI_ISL_1168870, EPI_ISL_1168871, EPI_ISL_1168872, EPI_ISL_1168873, EPI_ISL_1168874, EPI_ISL_1168875, EPI_ISL_1168880, EPI_ISL_1168883, EPI_ISL_1168897, EPI_ISL_1168898, EPI_ISL_1168899, EPI_ISL_1168900, EPI_ISL_1168901			
see above	Department of Infectious Diseases, Kobe Institute of Health	Department of Infectious Diseases, Kobe Institute of Health	Ryohei Nomoto, Noriko Nakanishi, Tomotada Iwamoto, Tsuyoshi Sekizuka, Kentaro Itokawa, Rina Tanaka, Masanori Hashino, Makoto Kuroda
EPI_ISL_1169499	Commonwealth Healthcare Center	Genomics and Discovery, Respiratory Viruses Branch, Division of Viral Diseases, Centers for Disease Control and Prevention	Krista Queen, Yan Li, Ying Tao, Jing Zhang, Anna Uehara, Anna Montmayeur, Clinton R. Paden, Peter W. Cook, Rachel Marine, Mili Sheth, Jasmine Padilla, Sarah Nobles, Mark Burroughs, Lori Rowe, Haibin Wang, Ben L. Rambo-Martin, Dhwani Batra, Justin Lee, Suxiang Tong
EPI_ISL_1171128, EPI_ISL_1171310	OHSU Lab Services Molecular Microbiology Lab	Ginkgo Bioworks Clinical Laboratory	Brendan L. O'Connell, Sally Grindstaff, Kayla Carter, Ruth V. Nichols, Malaika Mckenzie-Bennett, James McGann, Jim Griffin, Keith Robison, Alex Plocik, Becky Schilling, Martha Pierson, Rebecca Littlefield, Michelle Spencer, Amanda Riccardi, Birgitte Simen, Alec J. Hirsch, Donna Hansel, Guang Fan, Xuan Qin, Daniel N. Streblow, William B. Messer, Andrew C. Adey, Benjamin N. Bimber, Brian J. O'Roak
EPI_ISL_1171744, EPI_ISL_1171750	Wyoming Public Health Laboratory	Wyoming Public Health Laboratory	Jim Mildenberger, Wanda Manley, Noah Hull, Taylor Fearing, Lynette Gumbleton, Channing Weber, Ashley Norberg, Chayse Rowley, Marley Goetz, Brian Dominguez, Elliot Thomasson, Cari Sloma, and Rob Christensen
EPI_ISL_538498	RSUD Soediono Madiun East Java	National Institute of Health Research and Development	Pawestri, HA; Subangkit; Puspa, KD; Nugraha, AA; Ikawati, HD; Pangesti, KNA; Soekarso, T; Paisal; Setiawaty,V.
EPI_ISL_538503	RSUD Bali Mandara Denpasar Bali	National Institute of Health Research and Development	Pawestri, HA; Subangkit; Puspa, KD; Nugraha, AA; Ikawati, HD; Pangesti, KNA; Soekarso, T; Paisal; Setiawaty,V.
EPI_ISL_538511	RSUD Wahidin Sudirohusodo Mojokerto East Java	National Institute of Health Research and Development	Pawestri, HA; Subangkit; Puspa, KD; Nugraha, AA; Ikawati, HD; Pangesti, KNA; Soekarso, T; Paisal; Setiawaty,V.
EPI_ISL_538513	Provincial Health Laboratory Bekasi West Java	National Institute of Health Research and Development	Pawestri, HA; Subangkit; Puspa, KD; Nugraha, AA; Ikawati, HD; Pangesti, KNA; Soekarso, T; Paisal; Setiawaty,V.
EPI_ISL_700688, EPI_ISL_700689, EPI_ISL_700698	Respiratory Virus Unit, National Infection Service, Public Health England	COVID-19 Genomics UK (COG-UK) Consortium	PHE Covid Sequencing Team
EPI_ISL_704609	Quadram Institute Bioscience	COVID-19 Genomics UK (COG-UK) Consortium	Dave J. Baker, Gemma L. Kay, Alp Aydin, Thanh Le-Viet, Steven Rudder, Ana P. Tedim, Anastasia Kolyva, Maria Diaz, Leonardo de Oliveira Martins, Nabil-Fareed Alikhan, Lizzie Meadows, Rachael Stanley, Ngozi Elumogo, Muhammed Yasir, Nicholas M. Thomson, Alexander J Trotter, Rachel Gilroy, Samuel Bloomfield, Claire Stuart, Andrew Bell, Reenesh Prakash, Samir Dervisevic, Alison E. Mather, John Wain, Mark Webber, Andrew J. Page, Justin O'Grady
EPI_ISL_704674	University College London Hospital	COVID-19 Genomics UK (COG-UK) Consortium	Judith Heaney, Matthew Byott, Catherine Houlihan, Dan Frampton, Stuart Kirk, Moira Spyer and Eleni Nastouli
EPI_ISL_704728, EPI_ISL_704736, EPI_ISL_704744, EPI_ISL_704746	Quadram Institute Bioscience	COVID-19 Genomics UK (COG-UK) Consortium	Dave J. Baker, Gemma L. Kay, Alp Aydin, Thanh Le-Viet, Steven Rudder, Ana P. Tedim, Anastasia Kolyva, Maria Diaz, Leonardo de Oliveira Martins, Nabil-Fareed Alikhan, Lizzie Meadows, Rachael Stanley, Ngozi Elumogo, Muhammed Yasir, Nicholas M. Thomson, Alexander J Trotter, Rachel Gilroy, Samuel Bloomfield, Claire Stuart, Andrew Bell, Reenesh Prakash, Samir Dervisevic, Alison E. Mather, John Wain, Mark Webber, Andrew J. Page, Justin O'Grady
EPI_ISL_705732	West of Scotland Specialist Virology Centre, NHSGGC / MRC-University of Glasgow Centre for Virus Research	COVID-19 Genomics UK (COG-UK) Consortium	Ana da Silva Filipe, Natasha Johnson, Kathy Smollett, Daniel Mair, Stephen Carmichael, Alice Broos, Lily Tong, Jenna Nichols, Kyriaki Nomikou; Sarah McDonald; Richard Orton, Joseph Hughes, Sreenu Vattipally, David L Robertson; Alasdair MacLean, Rory Gunson; Sharif Shaaban, Matthew Holden; Rachel Blacow, Guy Mollett, Kathy Li, James Shepherd, Antonia Ho, Emma Thomson
EPI_ISL_705783, EPI_ISL_705786, EPI_ISL_705787, EPI_ISL_705788, EPI_ISL_705789, EPI_ISL_705790, EPI_ISL_705791, EPI_ISL_705792, EPI_ISL_705793	Virology Department, Royal Infirmary of Edinburgh, NHS Lothian / School of Biological Sciences, University of Edinburgh / Institute of Genetics and Molecular Medicine, University of Edinburgh	COVID-19 Genomics UK (COG-UK) Consortium	McHugh M, Dewar R, Rooke S, Gallagher M, Balcaza C, O'Toole Á, Scher E, Hill V, McCrone JT, Colquhoun R, Yu X, Jackson B, Rambaut A, Williams TC, Templeton K
EPI_ISL_705958	University College London Hospital	COVID-19 Genomics UK (COG-UK) Consortium	Judith Heaney, Matthew Byott, Catherine Houlihan, Dan Frampton, Stuart Kirk, Moira Spyer and Eleni Nastouli
EPI_ISL_706380, EPI_ISL_706382	Quadram Institute Bioscience	COVID-19 Genomics UK (COG-UK) Consortium	Dave J. Baker, Gemma L. Kay, Alp Aydin, Thanh Le-Viet, Steven Rudder, Ana P. Tedim, Anastasia Kolyva, Maria Diaz, Leonardo de Oliveira Martins, Nabil-Fareed Alikhan, Lizzie Meadows, Rachael Stanley, Ngozi Elumogo, Muhammed Yasir, Nicholas M. Thomson, Alexander J Trotter, Rachel Gilroy, Samuel Bloomfield, Claire Stuart, Andrew Bell, Reenesh Prakash, Samir Dervisevic, Alison E. Mather, John Wain, Mark Webber, Andrew J. Page, Justin O'Grady
EPI_ISL_706977, EPI_ISL_706979, EPI_ISL_706980, EPI_ISL_706981, EPI_ISL_706982	Virology Department, Sheffield Teaching Hospitals NHS Foundation Trust/Department of Infection, Immunity and Cardiovascular Disease, The Medical School, University of Sheffield	COVID-19 Genomics UK (COG-UK) Consortium	Thushan de Silva, Matthew Parker, Nikki Smith, Adri Angyal, Rebecca Brown, Luke Green, Rachel Tucker, Paul Parsons, Danielle Groves, Katie Johnson, Laura Carrilero, Alex Keeley, Dave Partridge, Matthew Wyles, Benjamin Lindsey, Mehmet Yavuz, Mohammad Raza, Cariad Evans
EPI_ISL_707795, EPI_ISL_707797, EPI_ISL_707800, EPI_ISL_707804	LabPLUS	Institute of Environmental Science and Research (ESR)	Xiaoyun Ren, Matt Storey, Nikki Freed, Muhammad Faisal, Jing Wang, Hermes Perez, Anja Werno, Antje van der Linden, Arlo Upton, Chris Mansell, David Hammer, Dragana Drinkovic, Gary McAuliffe, Hana Sofia Andersson, James Ussher, Jill Sherwood, Josh Freeman, Julia Howard, Juliet Elvy, Mary DeAlmeida, Matt Blakiston, Matthew Rogers, Max Bloomfield, Michael Addidle, Michelle Balm, Sally Roberts, Sarah Jefferies, Sharmini Muttaiyah, Susan Morpeth, Susan Taylor, Timothy Blackmore, Vani Sathyendran, Veronica Playle, Virginia Hope, Erasmus Smit, Lauren Jelly, Olin Silander, Joep de Ligt
EPI_ISL_707806	Middlemore Hospital	Institute of Environmental Science and Research (ESR)	Xiaoyun Ren, Matt Storey, Nikki Freed, Muhammad Faisal, Jing Wang, Hermes Perez, Anja Werno, Antje van der Linden, Arlo Upton, Chris Mansell, David Hammer, Dragana Drinkovic, Gary McAuliffe, Hana Sofia Andersson, James Ussher, Jill Sherwood, Josh Freeman, Julia Howard, Juliet Elvy, Mary DeAlmeida, Matt Blakiston, Matthew Rogers, Max Bloomfield, Michael Addidle, Michelle Balm, Sally Roberts, Sarah Jefferies, Sharmini Muttaiyah, Susan Morpeth, Susan Taylor, Timothy Blackmore, Vani Sathyendran, Veronica Playle, Virginia Hope, Erasmus Smit, Lauren Jelly, Olin Silander, Joep de Ligt
EPI_ISL_708496, EPI_ISL_708497, EPI_ISL_708498, EPI_ISL_708499, EPI_ISL_708500, EPI_ISL_708501, EPI_ISL_708502, EPI_ISL_708503	Minnesota Department of Health, Public Health Laboratory	Minnesota Department of Health, Public Health Laboratory	Alexandra Lorentz, Jacob Garfin, Matt Plumb, and Xiong Wang
EPI_ISL_708841, EPI_ISL_708845, EPI_ISL_708846, EPI_ISL_708851, EPI_ISL_708852, EPI_ISL_708853, EPI_ISL_708854	Lighthouse Lab in Alderley Park	Wellcome Sanger Institute for the COVID-19 Genomics UK (COG-UK) Consortium	Jacquelyn Wynn, Mairead Hyland, The Lighthouse Lab in Alderley Park and Alex Alderton, Roberto Amato, Sonia Goncalves, Ewan Harrison, David K. Jackson, Ian Johnston, Dominic Kwiatkowski, Cordelia Langford, John Sillitoe on behalf of the Wellcome Sanger Institute COVID-19 Surveillance Team
EPI_ISL_708855	Lighthouse Lab in Glasgow	Wellcome Sanger Institute for the COVID-19 Genomics UK (COG-UK) Consortium	Harper VanSteenhouse, Yumi Kasai, David Gray, Carol Clugston, Anna Dominiczak and Alex Alderton, Roberto Amato, Sonia Goncalves, Ewan Harrison, David K. Jackson, Ian Johnston, Dominic Kwiatkowski, Cordelia Langford, John Sillitoe on behalf of the Wellcome Sanger Institute COVID-19 Surveillance

			Team
EPI_ISL_708864, EPI_ISL_708865, EPI_ISL_708866, EPI_ISL_708867, EPI_ISL_708868, EPI_ISL_708870, EPI_ISL_708872, EPI_ISL_708876, EPI_ISL_708878	Lighthouse Lab in Alderley Park	Wellcome Sanger Institute for the COVID-19 Genomics UK (COG-UK) Consortium	Jacquelyn Wynn, Mairead Hyland, The Lighthouse Lab in Alderley Park and Alex Alderton, Roberto Amato, Sonia Goncalves, Ewan Harrison, David K. Jackson, Ian Johnston, Dominic Kwiatkowski, Cordelia Langford, John Sillitoe on behalf of the Wellcome Sanger Institute COVID-19 Surveillance Team
EPI_ISL_708884	Lighthouse Lab in Glasgow	Wellcome Sanger Institute for the COVID-19 Genomics UK (COG-UK) Consortium	Harper VanSteenhouse, Yumi Kasai, David Gray, Carol Clugston, Anna Dominiczak and Alex Alderton, Roberto Amato, Sonia Goncalves, Ewan Harrison, David K. Jackson, Ian Johnston, Dominic Kwiatkowski, Cordelia Langford, John Sillitoe on behalf of the Wellcome Sanger Institute COVID-19 Surveillance Team
EPI_ISL_708886, EPI_ISL_708887, EPI_ISL_708888, EPI_ISL_708892, EPI_ISL_708894, EPI_ISL_708903, EPI_ISL_708907, EPI_ISL_708911, EPI_ISL_708914, EPI_ISL_708919	Lighthouse Lab in Alderley Park	Wellcome Sanger Institute for the COVID-19 Genomics UK (COG-UK) Consortium	Jacquelyn Wynn, Mairead Hyland, The Lighthouse Lab in Alderley Park and Alex Alderton, Roberto Amato, Sonia Goncalves, Ewan Harrison, David K. Jackson, Ian Johnston, Dominic Kwiatkowski, Cordelia Langford, John Sillitoe on behalf of the Wellcome Sanger Institute COVID-19 Surveillance Team
EPI_ISL_708922	Lighthouse Lab in Glasgow	Wellcome Sanger Institute for the COVID-19 Genomics UK (COG-UK) Consortium	Harper VanSteenhouse, Yumi Kasai, David Gray, Carol Clugston, Anna Dominiczak and Alex Alderton, Roberto Amato, Sonia Goncalves, Ewan Harrison, David K. Jackson, Ian Johnston, Dominic Kwiatkowski, Cordelia Langford, John Sillitoe on behalf of the Wellcome Sanger Institute COVID-19 Surveillance Team
EPI_ISL_708924, EPI_ISL_708929, EPI_ISL_708931	Lighthouse Lab in Alderley Park	Wellcome Sanger Institute for the COVID-19 Genomics UK (COG-UK) Consortium	Jacquelyn Wynn, Mairead Hyland, The Lighthouse Lab in Alderley Park and Alex Alderton, Roberto Amato, Sonia Goncalves, Ewan Harrison, David K. Jackson, Ian Johnston, Dominic Kwiatkowski, Cordelia Langford, John Sillitoe on behalf of the Wellcome Sanger Institute COVID-19 Surveillance Team
EPI_ISL_708933	Lighthouse Lab in Glasgow	Wellcome Sanger Institute for the COVID-19 Genomics UK (COG-UK) Consortium	Harper VanSteenhouse, Yumi Kasai, David Gray, Carol Clugston, Anna Dominiczak and Alex Alderton, Roberto Amato, Sonia Goncalves, Ewan Harrison, David K. Jackson, Ian Johnston, Dominic Kwiatkowski, Cordelia Langford, John Sillitoe on behalf of the Wellcome Sanger Institute COVID-19 Surveillance Team
EPI_ISL_708939, EPI_ISL_708940, EPI_ISL_708942, EPI_ISL_708947, EPI_ISL_708949	Lighthouse Lab in Alderley Park	Wellcome Sanger Institute for the COVID-19 Genomics UK (COG-UK) Consortium	Jacquelyn Wynn, Mairead Hyland, The Lighthouse Lab in Alderley Park and Alex Alderton, Roberto Amato, Sonia Goncalves, Ewan Harrison, David K. Jackson, Ian Johnston, Dominic Kwiatkowski, Cordelia Langford, John Sillitoe on behalf of the Wellcome Sanger Institute COVID-19 Surveillance Team
EPI_ISL_708953	Lighthouse Lab in Glasgow	Wellcome Sanger Institute for the COVID-19 Genomics UK (COG-UK) Consortium	Harper VanSteenhouse, Yumi Kasai, David Gray, Carol Clugston, Anna Dominiczak and Alex Alderton, Roberto Amato, Sonia Goncalves, Ewan Harrison, David K. Jackson, Ian Johnston, Dominic Kwiatkowski, Cordelia Langford, John Sillitoe on behalf of the Wellcome Sanger Institute COVID-19 Surveillance Team
EPI_ISL_708955	Lighthouse Lab in Alderley Park	Wellcome Sanger Institute for the COVID-19 Genomics UK (COG-UK) Consortium	Jacquelyn Wynn, Mairead Hyland, The Lighthouse Lab in Alderley Park and Alex Alderton, Roberto Amato, Sonia Goncalves, Ewan Harrison, David K. Jackson, Ian Johnston, Dominic Kwiatkowski, Cordelia Langford, John Sillitoe on behalf of the Wellcome Sanger Institute COVID-19 Surveillance Team
EPI_ISL_708957	Lighthouse Lab in Glasgow	Wellcome Sanger Institute for the COVID-19 Genomics UK (COG-UK) Consortium	Harper VanSteenhouse, Yumi Kasai, David Gray, Carol Clugston, Anna Dominiczak and Alex Alderton, Roberto Amato, Sonia Goncalves, Ewan Harrison, David K. Jackson, Ian Johnston, Dominic Kwiatkowski, Cordelia Langford, John Sillitoe on behalf of the Wellcome Sanger Institute COVID-19 Surveillance Team
EPI_ISL_708959, EPI_ISL_708960, EPI_ISL_708967, EPI_ISL_708968, EPI_ISL_708970, EPI_ISL_708972, EPI_ISL_708973, EPI_ISL_708974, EPI_ISL_708977, EPI_ISL_708980, EPI_ISL_708981, EPI_ISL_708982			
see above	Lighthouse Lab in Alderley Park	Wellcome Sanger Institute for the COVID-19 Genomics UK (COG-UK) Consortium	Jacquelyn Wynn, Mairead Hyland, The Lighthouse Lab in Alderley Park and Alex Alderton, Roberto Amato, Sonia Goncalves, Ewan Harrison, David K. Jackson, Ian Johnston, Dominic Kwiatkowski, Cordelia Langford, John Sillitoe on behalf of the Wellcome Sanger Institute COVID-19 Surveillance Team
EPI_ISL_708985	Lighthouse Lab in Glasgow	Wellcome Sanger Institute for the COVID-19 Genomics UK (COG-UK) Consortium	Harper VanSteenhouse, Yumi Kasai, David Gray, Carol Clugston, Anna Dominiczak and Alex Alderton, Roberto Amato, Sonia Goncalves, Ewan Harrison, David K. Jackson, Ian Johnston, Dominic Kwiatkowski, Cordelia Langford, John Sillitoe on behalf of the Wellcome Sanger Institute COVID-19 Surveillance Team
EPI_ISL_708987, EPI_ISL_708988	Lighthouse Lab in Alderley Park	Wellcome Sanger Institute for the COVID-19 Genomics UK (COG-UK) Consortium	Jacquelyn Wynn, Mairead Hyland, The Lighthouse Lab in Alderley Park and Alex Alderton, Roberto Amato, Sonia Goncalves, Ewan Harrison, David K. Jackson, Ian Johnston, Dominic Kwiatkowski, Cordelia Langford, John Sillitoe on behalf of the Wellcome Sanger Institute COVID-19 Surveillance Team
EPI_ISL_708992, EPI_ISL_708993, EPI_ISL_708994, EPI_ISL_708995, EPI_ISL_708996, EPI_ISL_708999, EPI_ISL_709000, EPI_ISL_709001, EPI_ISL_709002, EPI_ISL_709003	Lighthouse Lab in Cambridge	Wellcome Sanger Institute for the COVID-19 Genomics UK (COG-UK) Consortium	Rob Howes, The Lighthouse Lab in Cambridge and Alex Alderton, Roberto Amato, Sonia Goncalves, Ewan Harrison, David K. Jackson, Ian Johnston, Dominic Kwiatkowski, Cordelia Langford, John Sillitoe on behalf of the Wellcome Sanger Institute COVID-19 Surveillance Team
EPI_ISL_709004, EPI_ISL_709005	Lighthouse Lab in Glasgow	Wellcome Sanger Institute for the COVID-19 Genomics UK (COG-UK) Consortium	Harper VanSteenhouse, Yumi Kasai, David Gray, Carol Clugston, Anna Dominiczak and Alex Alderton, Roberto Amato, Sonia Goncalves, Ewan Harrison, David K. Jackson, Ian Johnston, Dominic Kwiatkowski, Cordelia Langford, John Sillitoe on behalf of the Wellcome Sanger Institute COVID-19 Surveillance Team
EPI_ISL_709006, EPI_ISL_709007, EPI_ISL_709008	Lighthouse Lab in Cambridge	Wellcome Sanger Institute for the COVID-19 Genomics UK (COG-UK) Consortium	Rob Howes, The Lighthouse Lab in Cambridge and Alex Alderton, Roberto Amato, Sonia Goncalves, Ewan Harrison, David K. Jackson, Ian Johnston, Dominic Kwiatkowski, Cordelia Langford, John Sillitoe on behalf of the Wellcome Sanger Institute COVID-19 Surveillance Team
EPI_ISL_709009	Lighthouse Lab in Glasgow	Wellcome Sanger Institute for the COVID-19 Genomics UK (COG-UK) Consortium	Harper VanSteenhouse, Yumi Kasai, David Gray, Carol Clugston, Anna Dominiczak and Alex Alderton, Roberto Amato, Sonia Goncalves, Ewan Harrison, David K. Jackson, Ian Johnston, Dominic Kwiatkowski, Cordelia Langford, John Sillitoe on behalf of the Wellcome Sanger Institute COVID-19 Surveillance Team
EPI_ISL_709011	Lighthouse Lab in Cambridge	Wellcome Sanger Institute for the COVID-19 Genomics UK (COG-UK) Consortium	Rob Howes, The Lighthouse Lab in Cambridge and Alex Alderton, Roberto Amato, Sonia Goncalves, Ewan Harrison, David K. Jackson, Ian Johnston, Dominic Kwiatkowski, Cordelia Langford, John Sillitoe on behalf of the Wellcome Sanger Institute COVID-19 Surveillance Team
EPI_ISL_709013	Lighthouse Lab in Glasgow	Wellcome Sanger Institute for the COVID-19 Genomics UK (COG-UK) Consortium	Harper VanSteenhouse, Yumi Kasai, David Gray, Carol Clugston, Anna Dominiczak and Alex Alderton, Roberto Amato, Sonia Goncalves, Ewan Harrison, David K. Jackson, Ian Johnston, Dominic Kwiatkowski, Cordelia Langford, John Sillitoe on behalf of the Wellcome Sanger Institute COVID-19 Surveillance Team
EPI_ISL_709019, EPI_ISL_709020, EPI_ISL_709021	Lighthouse Lab in Cambridge	Wellcome Sanger Institute for the COVID-19 Genomics UK (COG-UK) Consortium	Rob Howes, The Lighthouse Lab in Cambridge and Alex Alderton, Roberto Amato, Sonia Goncalves, Ewan Harrison, David K. Jackson, Ian Johnston, Dominic Kwiatkowski, Cordelia Langford, John Sillitoe on behalf of the Wellcome Sanger Institute COVID-19 Surveillance Team
EPI_ISL_709022	Lighthouse Lab in Glasgow	Wellcome Sanger Institute for the COVID-19 Genomics UK (COG-UK) Consortium	Harper VanSteenhouse, Yumi Kasai, David Gray, Carol Clugston, Anna Dominiczak and Alex Alderton, Roberto Amato, Sonia Goncalves, Ewan Harrison, David K. Jackson, Ian Johnston, Dominic Kwiatkowski, Cordelia Langford, John Sillitoe on behalf of the Wellcome Sanger Institute COVID-19 Surveillance Team
EPI_ISL_709023, EPI_ISL_709024, EPI_ISL_709025, EPI_ISL_709026, EPI_ISL_709027, EPI_ISL_709028, EPI_ISL_709029, EPI_ISL_709030, EPI_ISL_709032, EPI_ISL_709035	Lighthouse Lab in Cambridge	Wellcome Sanger Institute for the COVID-19 Genomics UK (COG-UK) Consortium	Rob Howes, The Lighthouse Lab in Cambridge and Alex Alderton, Roberto Amato, Sonia Goncalves, Ewan Harrison, David K. Jackson, Ian Johnston, Dominic Kwiatkowski, Cordelia Langford, John Sillitoe on behalf of the Wellcome Sanger Institute COVID-19 Surveillance Team
EPI_ISL_709036	Lighthouse Lab in Glasgow	Wellcome Sanger Institute for the COVID-19 Genomics UK (COG-UK) Consortium	Harper VanSteenhouse, Yumi Kasai, David Gray, Carol Clugston, Anna Dominiczak and Alex Alderton, Roberto Amato, Sonia Goncalves, Ewan Harrison, David K. Jackson, Ian Johnston, Dominic Kwiatkowski, Cordelia Langford, John Sillitoe on behalf of the Wellcome Sanger Institute COVID-19 Surveillance Team
EPI_ISL_709037, EPI_ISL_709038	Lighthouse Lab in Cambridge	Wellcome Sanger Institute for the COVID-19 Genomics UK (COG-UK) Consortium	Rob Howes, The Lighthouse Lab in Cambridge and Alex Alderton, Roberto Amato, Sonia Goncalves, Ewan Harrison, David K. Jackson, Ian Johnston, Dominic Kwiatkowski, Cordelia Langford, John Sillitoe on behalf of the Wellcome Sanger Institute COVID-19 Surveillance Team

EPI_ISL_709040	Lighthouse Lab in Glasgow	Wellcome Sanger Institute for the COVID-19 Genomics UK (COG-UK) Consortium	Harper VanSteenhouse, Yumi Kasai, David Gray, Carol Clugston, Anna Dominiczak and Alex Alderton, Roberto Amato, Sonia Goncalves, Ewan Harrison, David K. Jackson, Ian Johnston, Dominic Kwiatkowski, Cordelia Langford, John Sillitoe on behalf of the Wellcome Sanger Institute COVID-19 Surveillance Team
EPI_ISL_709042, EPI_ISL_709043, EPI_ISL_709049, EPI_ISL_709052, EPI_ISL_709053, EPI_ISL_709055	Lighthouse Lab in Cambridge	Wellcome Sanger Institute for the COVID-19 Genomics UK (COG-UK) Consortium	Rob Howes, The Lighthouse Lab in Cambridge and Alex Alderton, Roberto Amato, Sonia Goncalves, Ewan Harrison, David K. Jackson, Ian Johnston, Dominic Kwiatkowski, Cordelia Langford, John Sillitoe on behalf of the Wellcome Sanger Institute COVID-19 Surveillance Team
EPI_ISL_709056	Lighthouse Lab in Glasgow	Wellcome Sanger Institute for the COVID-19 Genomics UK (COG-UK) Consortium	Harper VanSteenhouse, Yumi Kasai, David Gray, Carol Clugston, Anna Dominiczak and Alex Alderton, Roberto Amato, Sonia Goncalves, Ewan Harrison, David K. Jackson, Ian Johnston, Dominic Kwiatkowski, Cordelia Langford, John Sillitoe on behalf of the Wellcome Sanger Institute COVID-19 Surveillance Team
EPI_ISL_709057	Lighthouse Lab in Cambridge	Wellcome Sanger Institute for the COVID-19 Genomics UK (COG-UK) Consortium	Rob Howes, The Lighthouse Lab in Cambridge and Alex Alderton, Roberto Amato, Sonia Goncalves, Ewan Harrison, David K. Jackson, Ian Johnston, Dominic Kwiatkowski, Cordelia Langford, John Sillitoe on behalf of the Wellcome Sanger Institute COVID-19 Surveillance Team
EPI_ISL_709058, EPI_ISL_709060	Lighthouse Lab in Glasgow	Wellcome Sanger Institute for the COVID-19 Genomics UK (COG-UK) Consortium	Harper VanSteenhouse, Yumi Kasai, David Gray, Carol Clugston, Anna Dominiczak and Alex Alderton, Roberto Amato, Sonia Goncalves, Ewan Harrison, David K. Jackson, Ian Johnston, Dominic Kwiatkowski, Cordelia Langford, John Sillitoe on behalf of the Wellcome Sanger Institute COVID-19 Surveillance Team
EPI_ISL_709061, EPI_ISL_709062, EPI_ISL_709064	Lighthouse Lab in Cambridge	Wellcome Sanger Institute for the COVID-19 Genomics UK (COG-UK) Consortium	Rob Howes, The Lighthouse Lab in Cambridge and Alex Alderton, Roberto Amato, Sonia Goncalves, Ewan Harrison, David K. Jackson, Ian Johnston, Dominic Kwiatkowski, Cordelia Langford, John Sillitoe on behalf of the Wellcome Sanger Institute COVID-19 Surveillance Team
EPI_ISL_709065	Lighthouse Lab in Glasgow	Wellcome Sanger Institute for the COVID-19 Genomics UK (COG-UK) Consortium	Harper VanSteenhouse, Yumi Kasai, David Gray, Carol Clugston, Anna Dominiczak and Alex Alderton, Roberto Amato, Sonia Goncalves, Ewan Harrison, David K. Jackson, Ian Johnston, Dominic Kwiatkowski, Cordelia Langford, John Sillitoe on behalf of the Wellcome Sanger Institute COVID-19 Surveillance Team
EPI_ISL_709067, EPI_ISL_709068, EPI_ISL_709070, EPI_ISL_709071, EPI_ISL_709074, EPI_ISL_709075, EPI_ISL_709076	Lighthouse Lab in Cambridge	Wellcome Sanger Institute for the COVID-19 Genomics UK (COG-UK) Consortium	Rob Howes, The Lighthouse Lab in Cambridge and Alex Alderton, Roberto Amato, Sonia Goncalves, Ewan Harrison, David K. Jackson, Ian Johnston, Dominic Kwiatkowski, Cordelia Langford, John Sillitoe on behalf of the Wellcome Sanger Institute COVID-19 Surveillance Team
EPI_ISL_709077, EPI_ISL_709078, EPI_ISL_709079	Lighthouse Lab in Glasgow	Wellcome Sanger Institute for the COVID-19 Genomics UK (COG-UK) Consortium	Harper VanSteenhouse, Yumi Kasai, David Gray, Carol Clugston, Anna Dominiczak and Alex Alderton, Roberto Amato, Sonia Goncalves, Ewan Harrison, David K. Jackson, Ian Johnston, Dominic Kwiatkowski, Cordelia Langford, John Sillitoe on behalf of the Wellcome Sanger Institute COVID-19 Surveillance Team
EPI_ISL_709081	Lighthouse Lab in Cambridge	Wellcome Sanger Institute for the COVID-19 Genomics UK (COG-UK) Consortium	Rob Howes, The Lighthouse Lab in Cambridge and Alex Alderton, Roberto Amato, Sonia Goncalves, Ewan Harrison, David K. Jackson, Ian Johnston, Dominic Kwiatkowski, Cordelia Langford, John Sillitoe on behalf of the Wellcome Sanger Institute COVID-19 Surveillance Team
EPI_ISL_709082	Lighthouse Lab in Glasgow	Wellcome Sanger Institute for the COVID-19 Genomics UK (COG-UK) Consortium	Harper VanSteenhouse, Yumi Kasai, David Gray, Carol Clugston, Anna Dominiczak and Alex Alderton, Roberto Amato, Sonia Goncalves, Ewan Harrison, David K. Jackson, Ian Johnston, Dominic Kwiatkowski, Cordelia Langford, John Sillitoe on behalf of the Wellcome Sanger Institute COVID-19 Surveillance Team
EPI_ISL_709086, EPI_ISL_709087, EPI_ISL_709088, EPI_ISL_709090	Lighthouse Lab in Cambridge	Wellcome Sanger Institute for the COVID-19 Genomics UK (COG-UK) Consortium	Rob Howes, The Lighthouse Lab in Cambridge and Alex Alderton, Roberto Amato, Sonia Goncalves, Ewan Harrison, David K. Jackson, Ian Johnston, Dominic Kwiatkowski, Cordelia Langford, John Sillitoe on behalf of the Wellcome Sanger Institute COVID-19 Surveillance Team
EPI_ISL_709091	Lighthouse Lab in Glasgow	Wellcome Sanger Institute for the COVID-19 Genomics UK (COG-UK) Consortium	Harper VanSteenhouse, Yumi Kasai, David Gray, Carol Clugston, Anna Dominiczak and Alex Alderton, Roberto Amato, Sonia Goncalves, Ewan Harrison, David K. Jackson, Ian Johnston, Dominic Kwiatkowski, Cordelia Langford, John Sillitoe on behalf of the Wellcome Sanger Institute COVID-19 Surveillance Team
EPI_ISL_709095, EPI_ISL_709096, EPI_ISL_709099, EPI_ISL_709100, EPI_ISL_709105, EPI_ISL_709106, EPI_ISL_709113, EPI_ISL_709114, EPI_ISL_709116, EPI_ISL_709117, EPI_ISL_709118			
see above	Lighthouse Lab in Cambridge	Wellcome Sanger Institute for the COVID-19 Genomics UK (COG-UK) Consortium	Rob Howes, The Lighthouse Lab in Cambridge and Alex Alderton, Roberto Amato, Sonia Goncalves, Ewan Harrison, David K. Jackson, Ian Johnston, Dominic Kwiatkowski, Cordelia Langford, John Sillitoe on behalf of the Wellcome Sanger Institute COVID-19 Surveillance Team
EPI_ISL_709119	Lighthouse Lab in Glasgow	Wellcome Sanger Institute for the COVID-19 Genomics UK (COG-UK) Consortium	Harper VanSteenhouse, Yumi Kasai, David Gray, Carol Clugston, Anna Dominiczak and Alex Alderton, Roberto Amato, Sonia Goncalves, Ewan Harrison, David K. Jackson, Ian Johnston, Dominic Kwiatkowski, Cordelia Langford, John Sillitoe on behalf of the Wellcome Sanger Institute COVID-19 Surveillance Team
EPI_ISL_709121, EPI_ISL_709122, EPI_ISL_709123, EPI_ISL_709124, EPI_ISL_709129, EPI_ISL_709130	Lighthouse Lab in Cambridge	Wellcome Sanger Institute for the COVID-19 Genomics UK (COG-UK) Consortium	Rob Howes, The Lighthouse Lab in Cambridge and Alex Alderton, Roberto Amato, Sonia Goncalves, Ewan Harrison, David K. Jackson, Ian Johnston, Dominic Kwiatkowski, Cordelia Langford, John Sillitoe on behalf of the Wellcome Sanger Institute COVID-19 Surveillance Team
EPI_ISL_709133	Lighthouse Lab in Glasgow	Wellcome Sanger Institute for the COVID-19 Genomics UK (COG-UK) Consortium	Harper VanSteenhouse, Yumi Kasai, David Gray, Carol Clugston, Anna Dominiczak and Alex Alderton, Roberto Amato, Sonia Goncalves, Ewan Harrison, David K. Jackson, Ian Johnston, Dominic Kwiatkowski, Cordelia Langford, John Sillitoe on behalf of the Wellcome Sanger Institute COVID-19 Surveillance Team
EPI_ISL_709134	Lighthouse Lab in Cambridge	Wellcome Sanger Institute for the COVID-19 Genomics UK (COG-UK) Consortium	Rob Howes, The Lighthouse Lab in Cambridge and Alex Alderton, Roberto Amato, Sonia Goncalves, Ewan Harrison, David K. Jackson, Ian Johnston, Dominic Kwiatkowski, Cordelia Langford, John Sillitoe on behalf of the Wellcome Sanger Institute COVID-19 Surveillance Team
EPI_ISL_709135	Lighthouse Lab in Glasgow	Wellcome Sanger Institute for the COVID-19 Genomics UK (COG-UK) Consortium	Harper VanSteenhouse, Yumi Kasai, David Gray, Carol Clugston, Anna Dominiczak and Alex Alderton, Roberto Amato, Sonia Goncalves, Ewan Harrison, David K. Jackson, Ian Johnston, Dominic Kwiatkowski, Cordelia Langford, John Sillitoe on behalf of the Wellcome Sanger Institute COVID-19 Surveillance Team
EPI_ISL_709136, EPI_ISL_709138	Lighthouse Lab in Cambridge	Wellcome Sanger Institute for the COVID-19 Genomics UK (COG-UK) Consortium	Rob Howes, The Lighthouse Lab in Cambridge and Alex Alderton, Roberto Amato, Sonia Goncalves, Ewan Harrison, David K. Jackson, Ian Johnston, Dominic Kwiatkowski, Cordelia Langford, John Sillitoe on behalf of the Wellcome Sanger Institute COVID-19 Surveillance Team
EPI_ISL_709139, EPI_ISL_709141, EPI_ISL_709142	Lighthouse Lab in Glasgow	Wellcome Sanger Institute for the COVID-19 Genomics UK (COG-UK) Consortium	Harper VanSteenhouse, Yumi Kasai, David Gray, Carol Clugston, Anna Dominiczak and Alex Alderton, Roberto Amato, Sonia Goncalves, Ewan Harrison, David K. Jackson, Ian Johnston, Dominic Kwiatkowski, Cordelia Langford, John Sillitoe on behalf of the Wellcome Sanger Institute COVID-19 Surveillance Team
EPI_ISL_709143	Lighthouse Lab in Cambridge	Wellcome Sanger Institute for the COVID-19 Genomics UK (COG-UK) Consortium	Rob Howes, The Lighthouse Lab in Cambridge and Alex Alderton, Roberto Amato, Sonia Goncalves, Ewan Harrison, David K. Jackson, Ian Johnston, Dominic Kwiatkowski, Cordelia Langford, John Sillitoe on behalf of the Wellcome Sanger Institute COVID-19 Surveillance Team
EPI_ISL_709144	Lighthouse Lab in Glasgow	Wellcome Sanger Institute for the COVID-19 Genomics UK (COG-UK) Consortium	Harper VanSteenhouse, Yumi Kasai, David Gray, Carol Clugston, Anna Dominiczak and Alex Alderton, Roberto Amato, Sonia Goncalves, Ewan Harrison, David K. Jackson, Ian Johnston, Dominic Kwiatkowski, Cordelia Langford, John Sillitoe on behalf of the Wellcome Sanger Institute COVID-19 Surveillance Team
EPI_ISL_709146, EPI_ISL_709147	Lighthouse Lab in Cambridge	Wellcome Sanger Institute for the COVID-19 Genomics UK (COG-UK) Consortium	Rob Howes, The Lighthouse Lab in Cambridge and Alex Alderton, Roberto Amato, Sonia Goncalves, Ewan Harrison, David K. Jackson, Ian Johnston, Dominic Kwiatkowski, Cordelia Langford, John Sillitoe on behalf of the Wellcome Sanger Institute COVID-19 Surveillance Team
EPI_ISL_709150	Lighthouse Lab in Glasgow	Wellcome Sanger Institute for the COVID-19 Genomics UK (COG-UK) Consortium	Harper VanSteenhouse, Yumi Kasai, David Gray, Carol Clugston, Anna Dominiczak and Alex Alderton, Roberto Amato, Sonia Goncalves, Ewan Harrison, David K. Jackson, Ian Johnston, Dominic Kwiatkowski, Cordelia Langford, John Sillitoe on behalf of the Wellcome Sanger Institute COVID-19 Surveillance Team
EPI_ISL_709153, EPI_ISL_709154, EPI_ISL_709155, EPI_ISL_709158	Lighthouse Lab in Cambridge	Wellcome Sanger Institute for the COVID-19 Genomics UK (COG-UK) Consortium	Rob Howes, The Lighthouse Lab in Cambridge and Alex Alderton, Roberto Amato, Sonia Goncalves, Ewan Harrison, David K. Jackson, Ian Johnston, Dominic Kwiatkowski, Cordelia Langford, John Sillitoe on behalf of the Wellcome Sanger Institute COVID-19 Surveillance Team
EPI_ISL_709160	Lighthouse Lab in Glasgow	Wellcome Sanger Institute for the COVID-19 Genomics UK (COG-UK) Consortium	Harper VanSteenhouse, Yumi Kasai, David Gray, Carol Clugston, Anna Dominiczak and Alex Alderton, Roberto Amato, Sonia Goncalves, Ewan Harrison, David K. Jackson, Ian Johnston, Dominic Kwiatkowski, Cordelia Langford, John Sillitoe on behalf of the Wellcome Sanger Institute COVID-19 Surveillance Team

EPI_ISL_709162, EPI_ISL_709163, EPI_ISL_709164, EPI_ISL_709169	Lighthouse Lab in Cambridge	Wellcome Sanger Institute for the COVID-19 Genomics UK (COG-UK) Consortium	Rob Howes, The Lighthouse Lab in Cambridge and Alex Alderton, Roberto Amato, Sonia Goncalves, Ewan Harrison, David K. Jackson, Ian Johnston, Dominic Kwiatkowski, Cordelia Langford, John Sillitoe on behalf of the Wellcome Sanger Institute COVID-19 Surveillance Team
EPI_ISL_709170, EPI_ISL_709174	Lighthouse Lab in Glasgow	Wellcome Sanger Institute for the COVID-19 Genomics UK (COG-UK) Consortium	Harper VanSteenhouse, Yumi Kasai, David Gray, Carol Clugston, Anna Dominiczak and Alex Alderton, Roberto Amato, Sonia Goncalves, Ewan Harrison, David K. Jackson, Ian Johnston, Dominic Kwiatkowski, Cordelia Langford, John Sillitoe on behalf of the Wellcome Sanger Institute COVID-19 Surveillance Team
EPI_ISL_709175, EPI_ISL_709176, EPI_ISL_709178, EPI_ISL_709179, EPI_ISL_709185, EPI_ISL_709186, EPI_ISL_709188	Lighthouse Lab in Cambridge	Wellcome Sanger Institute for the COVID-19 Genomics UK (COG-UK) Consortium	Rob Howes, The Lighthouse Lab in Cambridge and Alex Alderton, Roberto Amato, Sonia Goncalves, Ewan Harrison, David K. Jackson, Ian Johnston, Dominic Kwiatkowski, Cordelia Langford, John Sillitoe on behalf of the Wellcome Sanger Institute COVID-19 Surveillance Team
EPI_ISL_709189, EPI_ISL_709190, EPI_ISL_709191	Lighthouse Lab in Glasgow	Wellcome Sanger Institute for the COVID-19 Genomics UK (COG-UK) Consortium	Harper VanSteenhouse, Yumi Kasai, David Gray, Carol Clugston, Anna Dominiczak and Alex Alderton, Roberto Amato, Sonia Goncalves, Ewan Harrison, David K. Jackson, Ian Johnston, Dominic Kwiatkowski, Cordelia Langford, John Sillitoe on behalf of the Wellcome Sanger Institute COVID-19 Surveillance Team
EPI_ISL_709194, EPI_ISL_709196, EPI_ISL_709197	Lighthouse Lab in Cambridge	Wellcome Sanger Institute for the COVID-19 Genomics UK (COG-UK) Consortium	Rob Howes, The Lighthouse Lab in Cambridge and Alex Alderton, Roberto Amato, Sonia Goncalves, Ewan Harrison, David K. Jackson, Ian Johnston, Dominic Kwiatkowski, Cordelia Langford, John Sillitoe on behalf of the Wellcome Sanger Institute COVID-19 Surveillance Team
EPI_ISL_709199	Lighthouse Lab in Glasgow	Wellcome Sanger Institute for the COVID-19 Genomics UK (COG-UK) Consortium	Harper VanSteenhouse, Yumi Kasai, David Gray, Carol Clugston, Anna Dominiczak and Alex Alderton, Roberto Amato, Sonia Goncalves, Ewan Harrison, David K. Jackson, Ian Johnston, Dominic Kwiatkowski, Cordelia Langford, John Sillitoe on behalf of the Wellcome Sanger Institute COVID-19 Surveillance Team
EPI_ISL_709200	Lighthouse Lab in Cambridge	Wellcome Sanger Institute for the COVID-19 Genomics UK (COG-UK) Consortium	Rob Howes, The Lighthouse Lab in Cambridge and Alex Alderton, Roberto Amato, Sonia Goncalves, Ewan Harrison, David K. Jackson, Ian Johnston, Dominic Kwiatkowski, Cordelia Langford, John Sillitoe on behalf of the Wellcome Sanger Institute COVID-19 Surveillance Team
EPI_ISL_709202	Lighthouse Lab in Glasgow	Wellcome Sanger Institute for the COVID-19 Genomics UK (COG-UK) Consortium	Harper VanSteenhouse, Yumi Kasai, David Gray, Carol Clugston, Anna Dominiczak and Alex Alderton, Roberto Amato, Sonia Goncalves, Ewan Harrison, David K. Jackson, Ian Johnston, Dominic Kwiatkowski, Cordelia Langford, John Sillitoe on behalf of the Wellcome Sanger Institute COVID-19 Surveillance Team
EPI_ISL_709204, EPI_ISL_709206, EPI_ISL_709207, EPI_ISL_709208, EPI_ISL_709210, EPI_ISL_709212, EPI_ISL_709217, EPI_ISL_709218, EPI_ISL_709223, EPI_ISL_709224, EPI_ISL_709226, EPI_ISL_709227, EPI_ISL_709229			
see above	Lighthouse Lab in Cambridge	Wellcome Sanger Institute for the COVID-19 Genomics UK (COG-UK) Consortium	Rob Howes, The Lighthouse Lab in Cambridge and Alex Alderton, Roberto Amato, Sonia Goncalves, Ewan Harrison, David K. Jackson, Ian Johnston, Dominic Kwiatkowski, Cordelia Langford, John Sillitoe on behalf of the Wellcome Sanger Institute COVID-19 Surveillance Team
EPI_ISL_709231	Lighthouse Lab in Glasgow	Wellcome Sanger Institute for the COVID-19 Genomics UK (COG-UK) Consortium	Harper VanSteenhouse, Yumi Kasai, David Gray, Carol Clugston, Anna Dominiczak and Alex Alderton, Roberto Amato, Sonia Goncalves, Ewan Harrison, David K. Jackson, Ian Johnston, Dominic Kwiatkowski, Cordelia Langford, John Sillitoe on behalf of the Wellcome Sanger Institute COVID-19 Surveillance Team
EPI_ISL_709232, EPI_ISL_709233	Lighthouse Lab in Cambridge	Wellcome Sanger Institute for the COVID-19 Genomics UK (COG-UK) Consortium	Rob Howes, The Lighthouse Lab in Cambridge and Alex Alderton, Roberto Amato, Sonia Goncalves, Ewan Harrison, David K. Jackson, Ian Johnston, Dominic Kwiatkowski, Cordelia Langford, John Sillitoe on behalf of the Wellcome Sanger Institute COVID-19 Surveillance Team
EPI_ISL_709234	Lighthouse Lab in Glasgow	Wellcome Sanger Institute for the COVID-19 Genomics UK (COG-UK) Consortium	Harper VanSteenhouse, Yumi Kasai, David Gray, Carol Clugston, Anna Dominiczak and Alex Alderton, Roberto Amato, Sonia Goncalves, Ewan Harrison, David K. Jackson, Ian Johnston, Dominic Kwiatkowski, Cordelia Langford, John Sillitoe on behalf of the Wellcome Sanger Institute COVID-19 Surveillance Team
EPI_ISL_709238, EPI_ISL_709241	Lighthouse Lab in Cambridge	Wellcome Sanger Institute for the COVID-19 Genomics UK (COG-UK) Consortium	Rob Howes, The Lighthouse Lab in Cambridge and Alex Alderton, Roberto Amato, Sonia Goncalves, Ewan Harrison, David K. Jackson, Ian Johnston, Dominic Kwiatkowski, Cordelia Langford, John Sillitoe on behalf of the Wellcome Sanger Institute COVID-19 Surveillance Team
EPI_ISL_709242, EPI_ISL_709243	Lighthouse Lab in Glasgow	Wellcome Sanger Institute for the COVID-19 Genomics UK (COG-UK) Consortium	Harper VanSteenhouse, Yumi Kasai, David Gray, Carol Clugston, Anna Dominiczak and Alex Alderton, Roberto Amato, Sonia Goncalves, Ewan Harrison, David K. Jackson, Ian Johnston, Dominic Kwiatkowski, Cordelia Langford, John Sillitoe on behalf of the Wellcome Sanger Institute COVID-19 Surveillance Team
EPI_ISL_709244	Lighthouse Lab in Cambridge	Wellcome Sanger Institute for the COVID-19 Genomics UK (COG-UK) Consortium	Rob Howes, The Lighthouse Lab in Cambridge and Alex Alderton, Roberto Amato, Sonia Goncalves, Ewan Harrison, David K. Jackson, Ian Johnston, Dominic Kwiatkowski, Cordelia Langford, John Sillitoe on behalf of the Wellcome Sanger Institute COVID-19 Surveillance Team
EPI_ISL_709245	Lighthouse Lab in Glasgow	Wellcome Sanger Institute for the COVID-19 Genomics UK (COG-UK) Consortium	Harper VanSteenhouse, Yumi Kasai, David Gray, Carol Clugston, Anna Dominiczak and Alex Alderton, Roberto Amato, Sonia Goncalves, Ewan Harrison, David K. Jackson, Ian Johnston, Dominic Kwiatkowski, Cordelia Langford, John Sillitoe on behalf of the Wellcome Sanger Institute COVID-19 Surveillance Team
EPI_ISL_709246, EPI_ISL_709249, EPI_ISL_709250, EPI_ISL_709251, EPI_ISL_709253, EPI_ISL_709254, EPI_ISL_709256, EPI_ISL_709257, EPI_ISL_709259, EPI_ISL_709260, EPI_ISL_709261			
see above	Lighthouse Lab in Cambridge	Wellcome Sanger Institute for the COVID-19 Genomics UK (COG-UK) Consortium	Rob Howes, The Lighthouse Lab in Cambridge and Alex Alderton, Roberto Amato, Sonia Goncalves, Ewan Harrison, David K. Jackson, Ian Johnston, Dominic Kwiatkowski, Cordelia Langford, John Sillitoe on behalf of the Wellcome Sanger Institute COVID-19 Surveillance Team
EPI_ISL_709264	Lighthouse Lab in Glasgow	Wellcome Sanger Institute for the COVID-19 Genomics UK (COG-UK) Consortium	Harper VanSteenhouse, Yumi Kasai, David Gray, Carol Clugston, Anna Dominiczak and Alex Alderton, Roberto Amato, Sonia Goncalves, Ewan Harrison, David K. Jackson, Ian Johnston, Dominic Kwiatkowski, Cordelia Langford, John Sillitoe on behalf of the Wellcome Sanger Institute COVID-19 Surveillance Team
EPI_ISL_709267, EPI_ISL_709268, EPI_ISL_709272, EPI_ISL_709273	Lighthouse Lab in Cambridge	Wellcome Sanger Institute for the COVID-19 Genomics UK (COG-UK) Consortium	Rob Howes, The Lighthouse Lab in Cambridge and Alex Alderton, Roberto Amato, Sonia Goncalves, Ewan Harrison, David K. Jackson, Ian Johnston, Dominic Kwiatkowski, Cordelia Langford, John Sillitoe on behalf of the Wellcome Sanger Institute COVID-19 Surveillance Team
EPI_ISL_709274	Lighthouse Lab in Glasgow	Wellcome Sanger Institute for the COVID-19 Genomics UK (COG-UK) Consortium	Harper VanSteenhouse, Yumi Kasai, David Gray, Carol Clugston, Anna Dominiczak and Alex Alderton, Roberto Amato, Sonia Goncalves, Ewan Harrison, David K. Jackson, Ian Johnston, Dominic Kwiatkowski, Cordelia Langford, John Sillitoe on behalf of the Wellcome Sanger Institute COVID-19 Surveillance Team
EPI_ISL_709275	Lighthouse Lab in Cambridge	Wellcome Sanger Institute for the COVID-19 Genomics UK (COG-UK) Consortium	Rob Howes, The Lighthouse Lab in Cambridge and Alex Alderton, Roberto Amato, Sonia Goncalves, Ewan Harrison, David K. Jackson, Ian Johnston, Dominic Kwiatkowski, Cordelia Langford, John Sillitoe on behalf of the Wellcome Sanger Institute COVID-19 Surveillance Team
EPI_ISL_709277	Lighthouse Lab in Glasgow	Wellcome Sanger Institute for the COVID-19 Genomics UK (COG-UK) Consortium	Harper VanSteenhouse, Yumi Kasai, David Gray, Carol Clugston, Anna Dominiczak and Alex Alderton, Roberto Amato, Sonia Goncalves, Ewan Harrison, David K. Jackson, Ian Johnston, Dominic Kwiatkowski, Cordelia Langford, John Sillitoe on behalf of the Wellcome Sanger Institute COVID-19 Surveillance Team
EPI_ISL_709278, EPI_ISL_709279, EPI_ISL_709281	Lighthouse Lab in Cambridge	Wellcome Sanger Institute for the COVID-19 Genomics UK (COG-UK) Consortium	Rob Howes, The Lighthouse Lab in Cambridge and Alex Alderton, Roberto Amato, Sonia Goncalves, Ewan Harrison, David K. Jackson, Ian Johnston, Dominic Kwiatkowski, Cordelia Langford, John Sillitoe on behalf of the Wellcome Sanger Institute COVID-19 Surveillance Team
EPI_ISL_709282, EPI_ISL_709283	Lighthouse Lab in Glasgow	Wellcome Sanger Institute for the COVID-19 Genomics UK (COG-UK) Consortium	Harper VanSteenhouse, Yumi Kasai, David Gray, Carol Clugston, Anna Dominiczak and Alex Alderton, Roberto Amato, Sonia Goncalves, Ewan Harrison, David K. Jackson, Ian Johnston, Dominic Kwiatkowski, Cordelia Langford, John Sillitoe on behalf of the Wellcome Sanger Institute COVID-19 Surveillance Team
EPI_ISL_709284	Lighthouse Lab in Cambridge	Wellcome Sanger Institute for the COVID-19 Genomics UK (COG-UK) Consortium	Rob Howes, The Lighthouse Lab in Cambridge and Alex Alderton, Roberto Amato, Sonia Goncalves, Ewan Harrison, David K. Jackson, Ian Johnston, Dominic Kwiatkowski, Cordelia Langford, John Sillitoe on behalf of the Wellcome Sanger Institute COVID-19 Surveillance Team
EPI_ISL_709285	Lighthouse Lab in Glasgow	Wellcome Sanger Institute for the COVID-19 Genomics UK (COG-UK) Consortium	Harper VanSteenhouse, Yumi Kasai, David Gray, Carol Clugston, Anna Dominiczak and Alex Alderton, Roberto Amato, Sonia Goncalves, Ewan Harrison, David K. Jackson, Ian Johnston, Dominic Kwiatkowski, Cordelia Langford, John Sillitoe on behalf of the Wellcome Sanger Institute COVID-19 Surveillance Team
EPI_ISL_709286	Lighthouse Lab in Cambridge	Wellcome Sanger Institute for the COVID-19 Genomics UK (COG-UK) Consortium	Rob Howes, The Lighthouse Lab in Cambridge and Alex Alderton, Roberto Amato, Sonia Goncalves, Ewan Harrison, David K. Jackson, Ian Johnston, Dominic Kwiatkowski, Cordelia Langford, John Sillitoe on behalf of the Wellcome Sanger Institute COVID-19 Surveillance Team
EPI_ISL_709290, EPI_ISL_709291, EPI_ISL_709292	Lighthouse Lab in Glasgow	Wellcome Sanger Institute for the COVID-19 Genomics UK (COG-UK) Consortium	Harper VanSteenhouse, Yumi Kasai, David Gray, Carol Clugston, Anna Dominiczak and Alex Alderton, Roberto Amato, Sonia Goncalves, Ewan Harrison, David K. Jackson, Ian Johnston, Dominic Kwiatkowski, Cordelia Langford, John Sillitoe on behalf of the Wellcome Sanger Institute COVID-19 Surveillance

			Team
EPI_ISL_709293, EPI_ISL_709294	Lighthouse Lab in Cambridge	Wellcome Sanger Institute for the COVID-19 Genomics UK (COG-UK) Consortium	Rob Howes, The Lighthouse Lab in Cambridge and Alex Alderton, Roberto Amato, Sonia Goncalves, Ewan Harrison, David K. Jackson, Ian Johnston, Dominic Kwiatkowski, Cordelia Langford, John Sillitoe on behalf of the Wellcome Sanger Institute COVID-19 Surveillance Team
EPI_ISL_709295, EPI_ISL_709296, EPI_ISL_709298, EPI_ISL_709299, EPI_ISL_709300, EPI_ISL_709303, EPI_ISL_709304, EPI_ISL_709305	Lighthouse Lab in Alderley Park	Wellcome Sanger Institute for the COVID-19 Genomics UK (COG-UK) Consortium	Jacquelyn Wynn, Mairead Hyland, The Lighthouse Lab in Alderley Park and Alex Alderton, Roberto Amato, Sonia Goncalves, Ewan Harrison, David K. Jackson, Ian Johnston, Dominic Kwiatkowski, Cordelia Langford, John Sillitoe on behalf of the Wellcome Sanger Institute COVID-19 Surveillance Team
EPI_ISL_709307	Lighthouse Lab in Glasgow	Wellcome Sanger Institute for the COVID-19 Genomics UK (COG-UK) Consortium	Harper VanSteenhouse, Yumi Kasai, David Gray, Carol Clugston, Anna Dominiczak and Alex Alderton, Roberto Amato, Sonia Goncalves, Ewan Harrison, David K. Jackson, Ian Johnston, Dominic Kwiatkowski, Cordelia Langford, John Sillitoe on behalf of the Wellcome Sanger Institute COVID-19 Surveillance Team
EPI_ISL_709308, EPI_ISL_709309, EPI_ISL_709310	Lighthouse Lab in Alderley Park	Wellcome Sanger Institute for the COVID-19 Genomics UK (COG-UK) Consortium	Jacquelyn Wynn, Mairead Hyland, The Lighthouse Lab in Alderley Park and Alex Alderton, Roberto Amato, Sonia Goncalves, Ewan Harrison, David K. Jackson, Ian Johnston, Dominic Kwiatkowski, Cordelia Langford, John Sillitoe on behalf of the Wellcome Sanger Institute COVID-19 Surveillance Team
EPI_ISL_709311, EPI_ISL_709312	Lighthouse Lab in Glasgow	Wellcome Sanger Institute for the COVID-19 Genomics UK (COG-UK) Consortium	Harper VanSteenhouse, Yumi Kasai, David Gray, Carol Clugston, Anna Dominiczak and Alex Alderton, Roberto Amato, Sonia Goncalves, Ewan Harrison, David K. Jackson, Ian Johnston, Dominic Kwiatkowski, Cordelia Langford, John Sillitoe on behalf of the Wellcome Sanger Institute COVID-19 Surveillance Team
EPI_ISL_709313, EPI_ISL_709316, EPI_ISL_709317, EPI_ISL_709318, EPI_ISL_709319	Lighthouse Lab in Alderley Park	Wellcome Sanger Institute for the COVID-19 Genomics UK (COG-UK) Consortium	Jacquelyn Wynn, Mairead Hyland, The Lighthouse Lab in Alderley Park and Alex Alderton, Roberto Amato, Sonia Goncalves, Ewan Harrison, David K. Jackson, Ian Johnston, Dominic Kwiatkowski, Cordelia Langford, John Sillitoe on behalf of the Wellcome Sanger Institute COVID-19 Surveillance Team
EPI_ISL_709320	Lighthouse Lab in Glasgow	Wellcome Sanger Institute for the COVID-19 Genomics UK (COG-UK) Consortium	Harper VanSteenhouse, Yumi Kasai, David Gray, Carol Clugston, Anna Dominiczak and Alex Alderton, Roberto Amato, Sonia Goncalves, Ewan Harrison, David K. Jackson, Ian Johnston, Dominic Kwiatkowski, Cordelia Langford, John Sillitoe on behalf of the Wellcome Sanger Institute COVID-19 Surveillance Team
EPI_ISL_709321, EPI_ISL_709322	Lighthouse Lab in Alderley Park	Wellcome Sanger Institute for the COVID-19 Genomics UK (COG-UK) Consortium	Jacquelyn Wynn, Mairead Hyland, The Lighthouse Lab in Alderley Park and Alex Alderton, Roberto Amato, Sonia Goncalves, Ewan Harrison, David K. Jackson, Ian Johnston, Dominic Kwiatkowski, Cordelia Langford, John Sillitoe on behalf of the Wellcome Sanger Institute COVID-19 Surveillance Team
EPI_ISL_709324, EPI_ISL_709327	Lighthouse Lab in Glasgow	Wellcome Sanger Institute for the COVID-19 Genomics UK (COG-UK) Consortium	Harper VanSteenhouse, Yumi Kasai, David Gray, Carol Clugston, Anna Dominiczak and Alex Alderton, Roberto Amato, Sonia Goncalves, Ewan Harrison, David K. Jackson, Ian Johnston, Dominic Kwiatkowski, Cordelia Langford, John Sillitoe on behalf of the Wellcome Sanger Institute COVID-19 Surveillance Team
EPI_ISL_709328, EPI_ISL_709329, EPI_ISL_709330, EPI_ISL_709333	Lighthouse Lab in Alderley Park	Wellcome Sanger Institute for the COVID-19 Genomics UK (COG-UK) Consortium	Jacquelyn Wynn, Mairead Hyland, The Lighthouse Lab in Alderley Park and Alex Alderton, Roberto Amato, Sonia Goncalves, Ewan Harrison, David K. Jackson, Ian Johnston, Dominic Kwiatkowski, Cordelia Langford, John Sillitoe on behalf of the Wellcome Sanger Institute COVID-19 Surveillance Team
EPI_ISL_709334	Lighthouse Lab in Glasgow	Wellcome Sanger Institute for the COVID-19 Genomics UK (COG-UK) Consortium	Harper VanSteenhouse, Yumi Kasai, David Gray, Carol Clugston, Anna Dominiczak and Alex Alderton, Roberto Amato, Sonia Goncalves, Ewan Harrison, David K. Jackson, Ian Johnston, Dominic Kwiatkowski, Cordelia Langford, John Sillitoe on behalf of the Wellcome Sanger Institute COVID-19 Surveillance Team
EPI_ISL_709335	Lighthouse Lab in Alderley Park	Wellcome Sanger Institute for the COVID-19 Genomics UK (COG-UK) Consortium	Jacquelyn Wynn, Mairead Hyland, The Lighthouse Lab in Alderley Park and Alex Alderton, Roberto Amato, Sonia Goncalves, Ewan Harrison, David K. Jackson, Ian Johnston, Dominic Kwiatkowski, Cordelia Langford, John Sillitoe on behalf of the Wellcome Sanger Institute COVID-19 Surveillance Team
EPI_ISL_709336	Lighthouse Lab in Glasgow	Wellcome Sanger Institute for the COVID-19 Genomics UK (COG-UK) Consortium	Harper VanSteenhouse, Yumi Kasai, David Gray, Carol Clugston, Anna Dominiczak and Alex Alderton, Roberto Amato, Sonia Goncalves, Ewan Harrison, David K. Jackson, Ian Johnston, Dominic Kwiatkowski, Cordelia Langford, John Sillitoe on behalf of the Wellcome Sanger Institute COVID-19 Surveillance Team
EPI_ISL_709338, EPI_ISL_709339, EPI_ISL_709340, EPI_ISL_709344	Lighthouse Lab in Alderley Park	Wellcome Sanger Institute for the COVID-19 Genomics UK (COG-UK) Consortium	Jacquelyn Wynn, Mairead Hyland, The Lighthouse Lab in Alderley Park and Alex Alderton, Roberto Amato, Sonia Goncalves, Ewan Harrison, David K. Jackson, Ian Johnston, Dominic Kwiatkowski, Cordelia Langford, John Sillitoe on behalf of the Wellcome Sanger Institute COVID-19 Surveillance Team
EPI_ISL_709345	Lighthouse Lab in Glasgow	Wellcome Sanger Institute for the COVID-19 Genomics UK (COG-UK) Consortium	Harper VanSteenhouse, Yumi Kasai, David Gray, Carol Clugston, Anna Dominiczak and Alex Alderton, Roberto Amato, Sonia Goncalves, Ewan Harrison, David K. Jackson, Ian Johnston, Dominic Kwiatkowski, Cordelia Langford, John Sillitoe on behalf of the Wellcome Sanger Institute COVID-19 Surveillance Team
EPI_ISL_709348, EPI_ISL_709350, EPI_ISL_709351	Lighthouse Lab in Alderley Park	Wellcome Sanger Institute for the COVID-19 Genomics UK (COG-UK) Consortium	Jacquelyn Wynn, Mairead Hyland, The Lighthouse Lab in Alderley Park and Alex Alderton, Roberto Amato, Sonia Goncalves, Ewan Harrison, David K. Jackson, Ian Johnston, Dominic Kwiatkowski, Cordelia Langford, John Sillitoe on behalf of the Wellcome Sanger Institute COVID-19 Surveillance Team
EPI_ISL_709353	Lighthouse Lab in Glasgow	Wellcome Sanger Institute for the COVID-19 Genomics UK (COG-UK) Consortium	Harper VanSteenhouse, Yumi Kasai, David Gray, Carol Clugston, Anna Dominiczak and Alex Alderton, Roberto Amato, Sonia Goncalves, Ewan Harrison, David K. Jackson, Ian Johnston, Dominic Kwiatkowski, Cordelia Langford, John Sillitoe on behalf of the Wellcome Sanger Institute COVID-19 Surveillance Team
EPI_ISL_709355, EPI_ISL_709356, EPI_ISL_709357	Lighthouse Lab in Alderley Park	Wellcome Sanger Institute for the COVID-19 Genomics UK (COG-UK) Consortium	Jacquelyn Wynn, Mairead Hyland, The Lighthouse Lab in Alderley Park and Alex Alderton, Roberto Amato, Sonia Goncalves, Ewan Harrison, David K. Jackson, Ian Johnston, Dominic Kwiatkowski, Cordelia Langford, John Sillitoe on behalf of the Wellcome Sanger Institute COVID-19 Surveillance Team
EPI_ISL_709359, EPI_ISL_709360	Lighthouse Lab in Glasgow	Wellcome Sanger Institute for the COVID-19 Genomics UK (COG-UK) Consortium	Harper VanSteenhouse, Yumi Kasai, David Gray, Carol Clugston, Anna Dominiczak and Alex Alderton, Roberto Amato, Sonia Goncalves, Ewan Harrison, David K. Jackson, Ian Johnston, Dominic Kwiatkowski, Cordelia Langford, John Sillitoe on behalf of the Wellcome Sanger Institute COVID-19 Surveillance Team
EPI_ISL_709362, EPI_ISL_709363	Lighthouse Lab in Alderley Park	Wellcome Sanger Institute for the COVID-19 Genomics UK (COG-UK) Consortium	Jacquelyn Wynn, Mairead Hyland, The Lighthouse Lab in Alderley Park and Alex Alderton, Roberto Amato, Sonia Goncalves, Ewan Harrison, David K. Jackson, Ian Johnston, Dominic Kwiatkowski, Cordelia Langford, John Sillitoe on behalf of the Wellcome Sanger Institute COVID-19 Surveillance Team
EPI_ISL_709364, EPI_ISL_709365, EPI_ISL_709367	Lighthouse Lab in Glasgow	Wellcome Sanger Institute for the COVID-19 Genomics UK (COG-UK) Consortium	Harper VanSteenhouse, Yumi Kasai, David Gray, Carol Clugston, Anna Dominiczak and Alex Alderton, Roberto Amato, Sonia Goncalves, Ewan Harrison, David K. Jackson, Ian Johnston, Dominic Kwiatkowski, Cordelia Langford, John Sillitoe on behalf of the Wellcome Sanger Institute COVID-19 Surveillance Team
EPI_ISL_709368, EPI_ISL_709369, EPI_ISL_709370, EPI_ISL_709371, EPI_ISL_709372	Lighthouse Lab in Alderley Park	Wellcome Sanger Institute for the COVID-19 Genomics UK (COG-UK) Consortium	Jacquelyn Wynn, Mairead Hyland, The Lighthouse Lab in Alderley Park and Alex Alderton, Roberto Amato, Sonia Goncalves, Ewan Harrison, David K. Jackson, Ian Johnston, Dominic Kwiatkowski, Cordelia Langford, John Sillitoe on behalf of the Wellcome Sanger Institute COVID-19 Surveillance Team
EPI_ISL_709373	Lighthouse Lab in Glasgow	Wellcome Sanger Institute for the COVID-19 Genomics UK (COG-UK) Consortium	Harper VanSteenhouse, Yumi Kasai, David Gray, Carol Clugston, Anna Dominiczak and Alex Alderton, Roberto Amato, Sonia Goncalves, Ewan Harrison, David K. Jackson, Ian Johnston, Dominic Kwiatkowski, Cordelia Langford, John Sillitoe on behalf of the Wellcome Sanger Institute COVID-19 Surveillance Team
EPI_ISL_709374	Lighthouse Lab in Alderley Park	Wellcome Sanger Institute for the COVID-19 Genomics UK (COG-UK) Consortium	Jacquelyn Wynn, Mairead Hyland, The Lighthouse Lab in Alderley Park and Alex Alderton, Roberto Amato, Sonia Goncalves, Ewan Harrison, David K. Jackson, Ian Johnston, Dominic Kwiatkowski, Cordelia Langford, John Sillitoe on behalf of the Wellcome Sanger Institute COVID-19 Surveillance Team
EPI_ISL_709376	Lighthouse Lab in Glasgow	Wellcome Sanger Institute for the COVID-19 Genomics UK (COG-UK) Consortium	Harper VanSteenhouse, Yumi Kasai, David Gray, Carol Clugston, Anna Dominiczak and Alex Alderton, Roberto Amato, Sonia Goncalves, Ewan Harrison, David K. Jackson, Ian Johnston, Dominic Kwiatkowski, Cordelia Langford, John Sillitoe on behalf of the Wellcome Sanger Institute COVID-19 Surveillance Team
EPI_ISL_709379, EPI_ISL_709381	Lighthouse Lab in Alderley Park	Wellcome Sanger Institute for the COVID-19 Genomics UK (COG-UK) Consortium	Jacquelyn Wynn, Mairead Hyland, The Lighthouse Lab in Alderley Park and Alex Alderton, Roberto Amato, Sonia Goncalves, Ewan Harrison, David K. Jackson, Ian Johnston, Dominic Kwiatkowski, Cordelia Langford, John Sillitoe on behalf of the Wellcome Sanger Institute COVID-19 Surveillance Team
EPI_ISL_709383, EPI_ISL_709384	Lighthouse Lab in Glasgow	Wellcome Sanger Institute for the COVID-19 Genomics UK (COG-UK) Consortium	Harper VanSteenhouse, Yumi Kasai, David Gray, Carol Clugston, Anna Dominiczak and Alex Alderton, Roberto Amato, Sonia Goncalves, Ewan Harrison, David K. Jackson, Ian Johnston, Dominic Kwiatkowski, Cordelia Langford, John Sillitoe on behalf of the Wellcome Sanger Institute COVID-19 Surveillance Team
EPI_ISL_709386, EPI_ISL_709387,	Lighthouse Lab in Alderley Park	Wellcome Sanger Institute for the COVID-19 Genomics UK	Jacquelyn Wynn, Mairead Hyland, The Lighthouse Lab in Alderley Park and Alex Alderton, Roberto Amato, Sonia Goncalves, Ewan Harrison, David K.

EPI_ISL_709389, EPI_ISL_709391		(COG-UK) Consortium	Jackson, Ian Johnston, Dominic Kwiatkowski, Cordelia Langford, John Sillitoe on behalf of the Wellcome Sanger Institute COVID-19 Surveillance Team
EPI_ISL_709392	Lighthouse Lab in Glasgow	Wellcome Sanger Institute for the COVID-19 Genomics UK (COG-UK) Consortium	Harper VanSteenhouse, Yumi Kasai, David Gray, Carol Clugston, Anna Dominiczak and Alex Alderton, Roberto Amato, Sonia Goncalves, Ewan Harrison, David K. Jackson, Ian Johnston, Dominic Kwiatkowski, Cordelia Langford, John Sillitoe on behalf of the Wellcome Sanger Institute COVID-19 Surveillance Team
EPI_ISL_709393	Lighthouse Lab in Alderley Park	Wellcome Sanger Institute for the COVID-19 Genomics UK (COG-UK) Consortium	Jacquelyn Wynn, Mairead Hyland, The Lighthouse Lab in Alderley Park and Alex Alderton, Roberto Amato, Sonia Goncalves, Ewan Harrison, David K. Jackson, Ian Johnston, Dominic Kwiatkowski, Cordelia Langford, John Sillitoe on behalf of the Wellcome Sanger Institute COVID-19 Surveillance Team
EPI_ISL_709395, EPI_ISL_709396	Lighthouse Lab in Glasgow	Wellcome Sanger Institute for the COVID-19 Genomics UK (COG-UK) Consortium	Harper VanSteenhouse, Yumi Kasai, David Gray, Carol Clugston, Anna Dominiczak and Alex Alderton, Roberto Amato, Sonia Goncalves, Ewan Harrison, David K. Jackson, Ian Johnston, Dominic Kwiatkowski, Cordelia Langford, John Sillitoe on behalf of the Wellcome Sanger Institute COVID-19 Surveillance Team
EPI_ISL_709397, EPI_ISL_709401, EPI_ISL_709402	Lighthouse Lab in Alderley Park	Wellcome Sanger Institute for the COVID-19 Genomics UK (COG-UK) Consortium	Jacquelyn Wynn, Mairead Hyland, The Lighthouse Lab in Alderley Park and Alex Alderton, Roberto Amato, Sonia Goncalves, Ewan Harrison, David K. Jackson, Ian Johnston, Dominic Kwiatkowski, Cordelia Langford, John Sillitoe on behalf of the Wellcome Sanger Institute COVID-19 Surveillance Team
EPI_ISL_709403, EPI_ISL_709404	Lighthouse Lab in Glasgow	Wellcome Sanger Institute for the COVID-19 Genomics UK (COG-UK) Consortium	Harper VanSteenhouse, Yumi Kasai, David Gray, Carol Clugston, Anna Dominiczak and Alex Alderton, Roberto Amato, Sonia Goncalves, Ewan Harrison, David K. Jackson, Ian Johnston, Dominic Kwiatkowski, Cordelia Langford, John Sillitoe on behalf of the Wellcome Sanger Institute COVID-19 Surveillance Team
EPI_ISL_709405	Lighthouse Lab in Alderley Park	Wellcome Sanger Institute for the COVID-19 Genomics UK (COG-UK) Consortium	Jacquelyn Wynn, Mairead Hyland, The Lighthouse Lab in Alderley Park and Alex Alderton, Roberto Amato, Sonia Goncalves, Ewan Harrison, David K. Jackson, Ian Johnston, Dominic Kwiatkowski, Cordelia Langford, John Sillitoe on behalf of the Wellcome Sanger Institute COVID-19 Surveillance Team
EPI_ISL_709407	Lighthouse Lab in Glasgow	Wellcome Sanger Institute for the COVID-19 Genomics UK (COG-UK) Consortium	Harper VanSteenhouse, Yumi Kasai, David Gray, Carol Clugston, Anna Dominiczak and Alex Alderton, Roberto Amato, Sonia Goncalves, Ewan Harrison, David K. Jackson, Ian Johnston, Dominic Kwiatkowski, Cordelia Langford, John Sillitoe on behalf of the Wellcome Sanger Institute COVID-19 Surveillance Team
EPI_ISL_709408, EPI_ISL_709410, EPI_ISL_709415, EPI_ISL_709416	Lighthouse Lab in Alderley Park	Wellcome Sanger Institute for the COVID-19 Genomics UK (COG-UK) Consortium	Jacquelyn Wynn, Mairead Hyland, The Lighthouse Lab in Alderley Park and Alex Alderton, Roberto Amato, Sonia Goncalves, Ewan Harrison, David K. Jackson, Ian Johnston, Dominic Kwiatkowski, Cordelia Langford, John Sillitoe on behalf of the Wellcome Sanger Institute COVID-19 Surveillance Team
EPI_ISL_709417	Lighthouse Lab in Glasgow	Wellcome Sanger Institute for the COVID-19 Genomics UK (COG-UK) Consortium	Harper VanSteenhouse, Yumi Kasai, David Gray, Carol Clugston, Anna Dominiczak and Alex Alderton, Roberto Amato, Sonia Goncalves, Ewan Harrison, David K. Jackson, Ian Johnston, Dominic Kwiatkowski, Cordelia Langford, John Sillitoe on behalf of the Wellcome Sanger Institute COVID-19 Surveillance Team
EPI_ISL_709418	Lighthouse Lab in Alderley Park	Wellcome Sanger Institute for the COVID-19 Genomics UK (COG-UK) Consortium	Jacquelyn Wynn, Mairead Hyland, The Lighthouse Lab in Alderley Park and Alex Alderton, Roberto Amato, Sonia Goncalves, Ewan Harrison, David K. Jackson, Ian Johnston, Dominic Kwiatkowski, Cordelia Langford, John Sillitoe on behalf of the Wellcome Sanger Institute COVID-19 Surveillance Team
EPI_ISL_709419	Lighthouse Lab in Glasgow	Wellcome Sanger Institute for the COVID-19 Genomics UK (COG-UK) Consortium	Harper VanSteenhouse, Yumi Kasai, David Gray, Carol Clugston, Anna Dominiczak and Alex Alderton, Roberto Amato, Sonia Goncalves, Ewan Harrison, David K. Jackson, Ian Johnston, Dominic Kwiatkowski, Cordelia Langford, John Sillitoe on behalf of the Wellcome Sanger Institute COVID-19 Surveillance Team
EPI_ISL_709420	Lighthouse Lab in Alderley Park	Wellcome Sanger Institute for the COVID-19 Genomics UK (COG-UK) Consortium	Jacquelyn Wynn, Mairead Hyland, The Lighthouse Lab in Alderley Park and Alex Alderton, Roberto Amato, Sonia Goncalves, Ewan Harrison, David K. Jackson, Ian Johnston, Dominic Kwiatkowski, Cordelia Langford, John Sillitoe on behalf of the Wellcome Sanger Institute COVID-19 Surveillance Team
EPI_ISL_709424	Lighthouse Lab in Glasgow	Wellcome Sanger Institute for the COVID-19 Genomics UK (COG-UK) Consortium	Harper VanSteenhouse, Yumi Kasai, David Gray, Carol Clugston, Anna Dominiczak and Alex Alderton, Roberto Amato, Sonia Goncalves, Ewan Harrison, David K. Jackson, Ian Johnston, Dominic Kwiatkowski, Cordelia Langford, John Sillitoe on behalf of the Wellcome Sanger Institute COVID-19 Surveillance Team
EPI_ISL_709428	Lighthouse Lab in Alderley Park	Wellcome Sanger Institute for the COVID-19 Genomics UK (COG-UK) Consortium	Jacquelyn Wynn, Mairead Hyland, The Lighthouse Lab in Alderley Park and Alex Alderton, Roberto Amato, Sonia Goncalves, Ewan Harrison, David K. Jackson, Ian Johnston, Dominic Kwiatkowski, Cordelia Langford, John Sillitoe on behalf of the Wellcome Sanger Institute COVID-19 Surveillance Team
EPI_ISL_709429	Lighthouse Lab in Glasgow	Wellcome Sanger Institute for the COVID-19 Genomics UK (COG-UK) Consortium	Harper VanSteenhouse, Yumi Kasai, David Gray, Carol Clugston, Anna Dominiczak and Alex Alderton, Roberto Amato, Sonia Goncalves, Ewan Harrison, David K. Jackson, Ian Johnston, Dominic Kwiatkowski, Cordelia Langford, John Sillitoe on behalf of the Wellcome Sanger Institute COVID-19 Surveillance Team
EPI_ISL_709430, EPI_ISL_709431, EPI_ISL_709432, EPI_ISL_709433, EPI_ISL_709434, EPI_ISL_709435	Lighthouse Lab in Alderley Park	Wellcome Sanger Institute for the COVID-19 Genomics UK (COG-UK) Consortium	Jacquelyn Wynn, Mairead Hyland, The Lighthouse Lab in Alderley Park and Alex Alderton, Roberto Amato, Sonia Goncalves, Ewan Harrison, David K. Jackson, Ian Johnston, Dominic Kwiatkowski, Cordelia Langford, John Sillitoe on behalf of the Wellcome Sanger Institute COVID-19 Surveillance Team
EPI_ISL_709436	Lighthouse Lab in Glasgow	Wellcome Sanger Institute for the COVID-19 Genomics UK (COG-UK) Consortium	Harper VanSteenhouse, Yumi Kasai, David Gray, Carol Clugston, Anna Dominiczak and Alex Alderton, Roberto Amato, Sonia Goncalves, Ewan Harrison, David K. Jackson, Ian Johnston, Dominic Kwiatkowski, Cordelia Langford, John Sillitoe on behalf of the Wellcome Sanger Institute COVID-19 Surveillance Team
EPI_ISL_709437	Lighthouse Lab in Alderley Park	Wellcome Sanger Institute for the COVID-19 Genomics UK (COG-UK) Consortium	Jacquelyn Wynn, Mairead Hyland, The Lighthouse Lab in Alderley Park and Alex Alderton, Roberto Amato, Sonia Goncalves, Ewan Harrison, David K. Jackson, Ian Johnston, Dominic Kwiatkowski, Cordelia Langford, John Sillitoe on behalf of the Wellcome Sanger Institute COVID-19 Surveillance Team
EPI_ISL_709438, EPI_ISL_709439, EPI_ISL_709440, EPI_ISL_709441	Lighthouse Lab in Glasgow	Wellcome Sanger Institute for the COVID-19 Genomics UK (COG-UK) Consortium	Harper VanSteenhouse, Yumi Kasai, David Gray, Carol Clugston, Anna Dominiczak and Alex Alderton, Roberto Amato, Sonia Goncalves, Ewan Harrison, David K. Jackson, Ian Johnston, Dominic Kwiatkowski, Cordelia Langford, John Sillitoe on behalf of the Wellcome Sanger Institute COVID-19 Surveillance Team
EPI_ISL_709442	Lighthouse Lab in Alderley Park	Wellcome Sanger Institute for the COVID-19 Genomics UK (COG-UK) Consortium	Jacquelyn Wynn, Mairead Hyland, The Lighthouse Lab in Alderley Park and Alex Alderton, Roberto Amato, Sonia Goncalves, Ewan Harrison, David K. Jackson, Ian Johnston, Dominic Kwiatkowski, Cordelia Langford, John Sillitoe on behalf of the Wellcome Sanger Institute COVID-19 Surveillance Team
EPI_ISL_709443	Lighthouse Lab in Glasgow	Wellcome Sanger Institute for the COVID-19 Genomics UK (COG-UK) Consortium	Harper VanSteenhouse, Yumi Kasai, David Gray, Carol Clugston, Anna Dominiczak and Alex Alderton, Roberto Amato, Sonia Goncalves, Ewan Harrison, David K. Jackson, Ian Johnston, Dominic Kwiatkowski, Cordelia Langford, John Sillitoe on behalf of the Wellcome Sanger Institute COVID-19 Surveillance Team
EPI_ISL_709444	Lighthouse Lab in Alderley Park	Wellcome Sanger Institute for the COVID-19 Genomics UK (COG-UK) Consortium	Jacquelyn Wynn, Mairead Hyland, The Lighthouse Lab in Alderley Park and Alex Alderton, Roberto Amato, Sonia Goncalves, Ewan Harrison, David K. Jackson, Ian Johnston, Dominic Kwiatkowski, Cordelia Langford, John Sillitoe on behalf of the Wellcome Sanger Institute COVID-19 Surveillance Team
EPI_ISL_709446	Lighthouse Lab in Glasgow	Wellcome Sanger Institute for the COVID-19 Genomics UK (COG-UK) Consortium	Harper VanSteenhouse, Yumi Kasai, David Gray, Carol Clugston, Anna Dominiczak and Alex Alderton, Roberto Amato, Sonia Goncalves, Ewan Harrison, David K. Jackson, Ian Johnston, Dominic Kwiatkowski, Cordelia Langford, John Sillitoe on behalf of the Wellcome Sanger Institute COVID-19 Surveillance Team
EPI_ISL_709447, EPI_ISL_709448	Lighthouse Lab in Alderley Park	Wellcome Sanger Institute for the COVID-19 Genomics UK (COG-UK) Consortium	Jacquelyn Wynn, Mairead Hyland, The Lighthouse Lab in Alderley Park and Alex Alderton, Roberto Amato, Sonia Goncalves, Ewan Harrison, David K. Jackson, Ian Johnston, Dominic Kwiatkowski, Cordelia Langford, John Sillitoe on behalf of the Wellcome Sanger Institute COVID-19 Surveillance Team
EPI_ISL_709450	Lighthouse Lab in Glasgow	Wellcome Sanger Institute for the COVID-19 Genomics UK (COG-UK) Consortium	Harper VanSteenhouse, Yumi Kasai, David Gray, Carol Clugston, Anna Dominiczak and Alex Alderton, Roberto Amato, Sonia Goncalves, Ewan Harrison, David K. Jackson, Ian Johnston, Dominic Kwiatkowski, Cordelia Langford, John Sillitoe on behalf of the Wellcome Sanger Institute COVID-19 Surveillance Team
EPI_ISL_709451, EPI_ISL_709452	Lighthouse Lab in Alderley Park	Wellcome Sanger Institute for the COVID-19 Genomics UK (COG-UK) Consortium	Jacquelyn Wynn, Mairead Hyland, The Lighthouse Lab in Alderley Park and Alex Alderton, Roberto Amato, Sonia Goncalves, Ewan Harrison, David K. Jackson, Ian Johnston, Dominic Kwiatkowski, Cordelia Langford, John Sillitoe on behalf of the Wellcome Sanger Institute COVID-19 Surveillance Team
EPI_ISL_709453, EPI_ISL_709454	Lighthouse Lab in Glasgow	Wellcome Sanger Institute for the COVID-19 Genomics UK (COG-UK) Consortium	Harper VanSteenhouse, Yumi Kasai, David Gray, Carol Clugston, Anna Dominiczak and Alex Alderton, Roberto Amato, Sonia Goncalves, Ewan Harrison, David K. Jackson, Ian Johnston, Dominic Kwiatkowski, Cordelia Langford, John Sillitoe on behalf of the Wellcome Sanger Institute COVID-19 Surveillance Team
EPI_ISL_709455	Lighthouse Lab in Alderley Park	Wellcome Sanger Institute for the COVID-19 Genomics UK (COG-UK) Consortium	Jacquelyn Wynn, Mairead Hyland, The Lighthouse Lab in Alderley Park and Alex Alderton, Roberto Amato, Sonia Goncalves, Ewan Harrison, David K. Jackson, Ian Johnston, Dominic Kwiatkowski, Cordelia Langford, John Sillitoe on behalf of the Wellcome Sanger Institute COVID-19 Surveillance Team
EPI_ISL_709456, EPI_ISL_709457,	Lighthouse Lab in Glasgow	Wellcome Sanger Institute for the COVID-19 Genomics UK	Harper VanSteenhouse, Yumi Kasai, David Gray, Carol Clugston, Anna Dominiczak and Alex Alderton, Roberto Amato, Sonia Goncalves, Ewan Harrison,

EPI_ISL_709458		(COG-UK) Consortium	David K. Jackson, Ian Johnston, Dominic Kwiatkowski, Cordelia Langford, John Sillitoe on behalf of the Wellcome Sanger Institute COVID-19 Surveillance Team
EPI_ISL_709460, EPI_ISL_709461, EPI_ISL_709463	Lighthouse Lab in Alderley Park	Wellcome Sanger Institute for the COVID-19 Genomics UK (COG-UK) Consortium	Jacquelyn Wynn, Mairead Hyland, The Lighthouse Lab in Alderley Park and Alex Alderton, Roberto Amato, Sonia Goncalves, Ewan Harrison, David K. Jackson, Ian Johnston, Dominic Kwiatkowski, Cordelia Langford, John Sillitoe on behalf of the Wellcome Sanger Institute COVID-19 Surveillance Team
EPI_ISL_709464, EPI_ISL_709465, EPI_ISL_709466	Lighthouse Lab in Glasgow	Wellcome Sanger Institute for the COVID-19 Genomics UK (COG-UK) Consortium	Harper VanSteenhouse, Yumi Kasai, David Gray, Carol Clugston, Anna Dominiczak and Alex Alderton, Roberto Amato, Sonia Goncalves, Ewan Harrison, David K. Jackson, Ian Johnston, Dominic Kwiatkowski, Cordelia Langford, John Sillitoe on behalf of the Wellcome Sanger Institute COVID-19 Surveillance Team
EPI_ISL_709467, EPI_ISL_709468, EPI_ISL_709470, EPI_ISL_709471, EPI_ISL_709473	Lighthouse Lab in Alderley Park	Wellcome Sanger Institute for the COVID-19 Genomics UK (COG-UK) Consortium	Jacquelyn Wynn, Mairead Hyland, The Lighthouse Lab in Alderley Park and Alex Alderton, Roberto Amato, Sonia Goncalves, Ewan Harrison, David K. Jackson, Ian Johnston, Dominic Kwiatkowski, Cordelia Langford, John Sillitoe on behalf of the Wellcome Sanger Institute COVID-19 Surveillance Team
EPI_ISL_709474	Lighthouse Lab in Glasgow	Wellcome Sanger Institute for the COVID-19 Genomics UK (COG-UK) Consortium	Harper VanSteenhouse, Yumi Kasai, David Gray, Carol Clugston, Anna Dominiczak and Alex Alderton, Roberto Amato, Sonia Goncalves, Ewan Harrison, David K. Jackson, Ian Johnston, Dominic Kwiatkowski, Cordelia Langford, John Sillitoe on behalf of the Wellcome Sanger Institute COVID-19 Surveillance Team
EPI_ISL_709475, EPI_ISL_709476, EPI_ISL_709477, EPI_ISL_709478	Lighthouse Lab in Alderley Park	Wellcome Sanger Institute for the COVID-19 Genomics UK (COG-UK) Consortium	Jacquelyn Wynn, Mairead Hyland, The Lighthouse Lab in Alderley Park and Alex Alderton, Roberto Amato, Sonia Goncalves, Ewan Harrison, David K. Jackson, Ian Johnston, Dominic Kwiatkowski, Cordelia Langford, John Sillitoe on behalf of the Wellcome Sanger Institute COVID-19 Surveillance Team
EPI_ISL_709481, EPI_ISL_709482	Lighthouse Lab in Glasgow	Wellcome Sanger Institute for the COVID-19 Genomics UK (COG-UK) Consortium	Harper VanSteenhouse, Yumi Kasai, David Gray, Carol Clugston, Anna Dominiczak and Alex Alderton, Roberto Amato, Sonia Goncalves, Ewan Harrison, David K. Jackson, Ian Johnston, Dominic Kwiatkowski, Cordelia Langford, John Sillitoe on behalf of the Wellcome Sanger Institute COVID-19 Surveillance Team
EPI_ISL_709484	Lighthouse Lab in Alderley Park	Wellcome Sanger Institute for the COVID-19 Genomics UK (COG-UK) Consortium	Jacquelyn Wynn, Mairead Hyland, The Lighthouse Lab in Alderley Park and Alex Alderton, Roberto Amato, Sonia Goncalves, Ewan Harrison, David K. Jackson, Ian Johnston, Dominic Kwiatkowski, Cordelia Langford, John Sillitoe on behalf of the Wellcome Sanger Institute COVID-19 Surveillance Team
EPI_ISL_709487, EPI_ISL_709490	Lighthouse Lab in Glasgow	Wellcome Sanger Institute for the COVID-19 Genomics UK (COG-UK) Consortium	Harper VanSteenhouse, Yumi Kasai, David Gray, Carol Clugston, Anna Dominiczak and Alex Alderton, Roberto Amato, Sonia Goncalves, Ewan Harrison, David K. Jackson, Ian Johnston, Dominic Kwiatkowski, Cordelia Langford, John Sillitoe on behalf of the Wellcome Sanger Institute COVID-19 Surveillance Team
EPI_ISL_709492	Lighthouse Lab in Alderley Park	Wellcome Sanger Institute for the COVID-19 Genomics UK (COG-UK) Consortium	Jacquelyn Wynn, Mairead Hyland, The Lighthouse Lab in Alderley Park and Alex Alderton, Roberto Amato, Sonia Goncalves, Ewan Harrison, David K. Jackson, Ian Johnston, Dominic Kwiatkowski, Cordelia Langford, John Sillitoe on behalf of the Wellcome Sanger Institute COVID-19 Surveillance Team
EPI_ISL_709496, EPI_ISL_709497, EPI_ISL_709498, EPI_ISL_709500, EPI_ISL_709502, EPI_ISL_709503	Lighthouse Lab in Glasgow	Wellcome Sanger Institute for the COVID-19 Genomics UK (COG-UK) Consortium	Harper VanSteenhouse, Yumi Kasai, David Gray, Carol Clugston, Anna Dominiczak and Alex Alderton, Roberto Amato, Sonia Goncalves, Ewan Harrison, David K. Jackson, Ian Johnston, Dominic Kwiatkowski, Cordelia Langford, John Sillitoe on behalf of the Wellcome Sanger Institute COVID-19 Surveillance Team
EPI_ISL_709504, EPI_ISL_709505, EPI_ISL_709506, EPI_ISL_709507, EPI_ISL_709508	Lighthouse Lab in Alderley Park	Wellcome Sanger Institute for the COVID-19 Genomics UK (COG-UK) Consortium	Jacquelyn Wynn, Mairead Hyland, The Lighthouse Lab in Alderley Park and Alex Alderton, Roberto Amato, Sonia Goncalves, Ewan Harrison, David K. Jackson, Ian Johnston, Dominic Kwiatkowski, Cordelia Langford, John Sillitoe on behalf of the Wellcome Sanger Institute COVID-19 Surveillance Team
EPI_ISL_709511	Lighthouse Lab in Glasgow	Wellcome Sanger Institute for the COVID-19 Genomics UK (COG-UK) Consortium	Harper VanSteenhouse, Yumi Kasai, David Gray, Carol Clugston, Anna Dominiczak and Alex Alderton, Roberto Amato, Sonia Goncalves, Ewan Harrison, David K. Jackson, Ian Johnston, Dominic Kwiatkowski, Cordelia Langford, John Sillitoe on behalf of the Wellcome Sanger Institute COVID-19 Surveillance Team
EPI_ISL_709512, EPI_ISL_709514, EPI_ISL_709516	Lighthouse Lab in Alderley Park	Wellcome Sanger Institute for the COVID-19 Genomics UK (COG-UK) Consortium	Jacquelyn Wynn, Mairead Hyland, The Lighthouse Lab in Alderley Park and Alex Alderton, Roberto Amato, Sonia Goncalves, Ewan Harrison, David K. Jackson, Ian Johnston, Dominic Kwiatkowski, Cordelia Langford, John Sillitoe on behalf of the Wellcome Sanger Institute COVID-19 Surveillance Team
EPI_ISL_709518	Lighthouse Lab in Glasgow	Wellcome Sanger Institute for the COVID-19 Genomics UK (COG-UK) Consortium	Harper VanSteenhouse, Yumi Kasai, David Gray, Carol Clugston, Anna Dominiczak and Alex Alderton, Roberto Amato, Sonia Goncalves, Ewan Harrison, David K. Jackson, Ian Johnston, Dominic Kwiatkowski, Cordelia Langford, John Sillitoe on behalf of the Wellcome Sanger Institute COVID-19 Surveillance Team
EPI_ISL_709519, EPI_ISL_709520, EPI_ISL_709521	Lighthouse Lab in Alderley Park	Wellcome Sanger Institute for the COVID-19 Genomics UK (COG-UK) Consortium	Jacquelyn Wynn, Mairead Hyland, The Lighthouse Lab in Alderley Park and Alex Alderton, Roberto Amato, Sonia Goncalves, Ewan Harrison, David K. Jackson, Ian Johnston, Dominic Kwiatkowski, Cordelia Langford, John Sillitoe on behalf of the Wellcome Sanger Institute COVID-19 Surveillance Team
EPI_ISL_709522	Lighthouse Lab in Glasgow	Wellcome Sanger Institute for the COVID-19 Genomics UK (COG-UK) Consortium	Harper VanSteenhouse, Yumi Kasai, David Gray, Carol Clugston, Anna Dominiczak and Alex Alderton, Roberto Amato, Sonia Goncalves, Ewan Harrison, David K. Jackson, Ian Johnston, Dominic Kwiatkowski, Cordelia Langford, John Sillitoe on behalf of the Wellcome Sanger Institute COVID-19 Surveillance Team
EPI_ISL_709523	Lighthouse Lab in Alderley Park	Wellcome Sanger Institute for the COVID-19 Genomics UK (COG-UK) Consortium	Jacquelyn Wynn, Mairead Hyland, The Lighthouse Lab in Alderley Park and Alex Alderton, Roberto Amato, Sonia Goncalves, Ewan Harrison, David K. Jackson, Ian Johnston, Dominic Kwiatkowski, Cordelia Langford, John Sillitoe on behalf of the Wellcome Sanger Institute COVID-19 Surveillance Team
EPI_ISL_709524, EPI_ISL_709525, EPI_ISL_709527	Lighthouse Lab in Glasgow	Wellcome Sanger Institute for the COVID-19 Genomics UK (COG-UK) Consortium	Harper VanSteenhouse, Yumi Kasai, David Gray, Carol Clugston, Anna Dominiczak and Alex Alderton, Roberto Amato, Sonia Goncalves, Ewan Harrison, David K. Jackson, Ian Johnston, Dominic Kwiatkowski, Cordelia Langford, John Sillitoe on behalf of the Wellcome Sanger Institute COVID-19 Surveillance Team
EPI_ISL_709529	Lighthouse Lab in Alderley Park	Wellcome Sanger Institute for the COVID-19 Genomics UK (COG-UK) Consortium	Jacquelyn Wynn, Mairead Hyland, The Lighthouse Lab in Alderley Park and Alex Alderton, Roberto Amato, Sonia Goncalves, Ewan Harrison, David K. Jackson, Ian Johnston, Dominic Kwiatkowski, Cordelia Langford, John Sillitoe on behalf of the Wellcome Sanger Institute COVID-19 Surveillance Team
EPI_ISL_709530	Lighthouse Lab in Glasgow	Wellcome Sanger Institute for the COVID-19 Genomics UK (COG-UK) Consortium	Harper VanSteenhouse, Yumi Kasai, David Gray, Carol Clugston, Anna Dominiczak and Alex Alderton, Roberto Amato, Sonia Goncalves, Ewan Harrison, David K. Jackson, Ian Johnston, Dominic Kwiatkowski, Cordelia Langford, John Sillitoe on behalf of the Wellcome Sanger Institute COVID-19 Surveillance Team
EPI_ISL_709532, EPI_ISL_709534, EPI_ISL_709535, EPI_ISL_709536	Lighthouse Lab in Alderley Park	Wellcome Sanger Institute for the COVID-19 Genomics UK (COG-UK) Consortium	Jacquelyn Wynn, Mairead Hyland, The Lighthouse Lab in Alderley Park and Alex Alderton, Roberto Amato, Sonia Goncalves, Ewan Harrison, David K. Jackson, Ian Johnston, Dominic Kwiatkowski, Cordelia Langford, John Sillitoe on behalf of the Wellcome Sanger Institute COVID-19 Surveillance Team
EPI_ISL_709539, EPI_ISL_709540, EPI_ISL_709541	Lighthouse Lab in Glasgow	Wellcome Sanger Institute for the COVID-19 Genomics UK (COG-UK) Consortium	Harper VanSteenhouse, Yumi Kasai, David Gray, Carol Clugston, Anna Dominiczak and Alex Alderton, Roberto Amato, Sonia Goncalves, Ewan Harrison, David K. Jackson, Ian Johnston, Dominic Kwiatkowski, Cordelia Langford, John Sillitoe on behalf of the Wellcome Sanger Institute COVID-19 Surveillance Team
EPI_ISL_709543, EPI_ISL_709544, EPI_ISL_709545, EPI_ISL_709546	Lighthouse Lab in Alderley Park	Wellcome Sanger Institute for the COVID-19 Genomics UK (COG-UK) Consortium	Jacquelyn Wynn, Mairead Hyland, The Lighthouse Lab in Alderley Park and Alex Alderton, Roberto Amato, Sonia Goncalves, Ewan Harrison, David K. Jackson, Ian Johnston, Dominic Kwiatkowski, Cordelia Langford, John Sillitoe on behalf of the Wellcome Sanger Institute COVID-19 Surveillance Team
EPI_ISL_709549	Lighthouse Lab in Glasgow	Wellcome Sanger Institute for the COVID-19 Genomics UK (COG-UK) Consortium	Harper VanSteenhouse, Yumi Kasai, David Gray, Carol Clugston, Anna Dominiczak and Alex Alderton, Roberto Amato, Sonia Goncalves, Ewan Harrison, David K. Jackson, Ian Johnston, Dominic Kwiatkowski, Cordelia Langford, John Sillitoe on behalf of the Wellcome Sanger Institute COVID-19 Surveillance Team
EPI_ISL_709550, EPI_ISL_709552	Lighthouse Lab in Alderley Park	Wellcome Sanger Institute for the COVID-19 Genomics UK (COG-UK) Consortium	Jacquelyn Wynn, Mairead Hyland, The Lighthouse Lab in Alderley Park and Alex Alderton, Roberto Amato, Sonia Goncalves, Ewan Harrison, David K. Jackson, Ian Johnston, Dominic Kwiatkowski, Cordelia Langford, John Sillitoe on behalf of the Wellcome Sanger Institute COVID-19 Surveillance Team
EPI_ISL_709555, EPI_ISL_709558	Lighthouse Lab in Glasgow	Wellcome Sanger Institute for the COVID-19 Genomics UK (COG-UK) Consortium	Harper VanSteenhouse, Yumi Kasai, David Gray, Carol Clugston, Anna Dominiczak and Alex Alderton, Roberto Amato, Sonia Goncalves, Ewan Harrison, David K. Jackson, Ian Johnston, Dominic Kwiatkowski, Cordelia Langford, John Sillitoe on behalf of the Wellcome Sanger Institute COVID-19 Surveillance Team
EPI_ISL_709559, EPI_ISL_709560	Lighthouse Lab in Alderley Park	Wellcome Sanger Institute for the COVID-19 Genomics UK (COG-UK) Consortium	Jacquelyn Wynn, Mairead Hyland, The Lighthouse Lab in Alderley Park and Alex Alderton, Roberto Amato, Sonia Goncalves, Ewan Harrison, David K. Jackson, Ian Johnston, Dominic Kwiatkowski, Cordelia Langford, John Sillitoe on behalf of the Wellcome Sanger Institute COVID-19 Surveillance Team
EPI_ISL_709561	Lighthouse Lab in Glasgow	Wellcome Sanger Institute for the COVID-19 Genomics UK (COG-UK) Consortium	Harper VanSteenhouse, Yumi Kasai, David Gray, Carol Clugston, Anna Dominiczak and Alex Alderton, Roberto Amato, Sonia Goncalves, Ewan Harrison, David K. Jackson, Ian Johnston, Dominic Kwiatkowski, Cordelia Langford, John Sillitoe on behalf of the Wellcome Sanger Institute COVID-19 Surveillance

			Team
EPI_ISL_709563, EPI_ISL_709564	Lighthouse Lab in Alderley Park	Wellcome Sanger Institute for the COVID-19 Genomics UK (COG-UK) Consortium	Jacquelyn Wynn, Mairead Hyland, The Lighthouse Lab in Alderley Park and Alex Alderton, Roberto Amato, Sonia Goncalves, Ewan Harrison, David K. Jackson, Ian Johnston, Dominic Kwiatkowski, Cordelia Langford, John Sillitoe on behalf of the Wellcome Sanger Institute COVID-19 Surveillance Team
EPI_ISL_709565	Lighthouse Lab in Glasgow	Wellcome Sanger Institute for the COVID-19 Genomics UK (COG-UK) Consortium	Harper VanSteenhouse, Yumi Kasai, David Gray, Carol Clugston, Anna Dominiczak and Alex Alderton, Roberto Amato, Sonia Goncalves, Ewan Harrison, David K. Jackson, Ian Johnston, Dominic Kwiatkowski, Cordelia Langford, John Sillitoe on behalf of the Wellcome Sanger Institute COVID-19 Surveillance Team
EPI_ISL_710513	Area of Virology, Serology and Virology Division (SAViD), New South Wales Health Pathology Randwick	Area of Virology, Serology and Virology Division (SAViD), New South Wales Health Pathology Randwick	Rawlinson, W., Deveson, I., Bull, R.
EPI_ISL_710591	Narhalsan Fjallbacka VC	The Public Health Agency of Sweden	Department of Microbiology, The Public Health Agency of Sweden
EPI_ISL_710603	Klinisk mikrobiologi	The Public Health Agency of Sweden	Department of Microbiology, The Public Health Agency of Sweden
EPI_ISL_710614, EPI_ISL_710617	Lighthouse Lab in Alderley Park	Wellcome Sanger Institute for the COVID-19 Genomics UK (COG-UK) Consortium	Jacquelyn Wynn, Mairead Hyland, The Lighthouse Lab in Alderley Park and Alex Alderton, Roberto Amato, Sonia Goncalves, Ewan Harrison, David K. Jackson, Ian Johnston, Dominic Kwiatkowski, Cordelia Langford, John Sillitoe on behalf of the Wellcome Sanger Institute COVID-19 Surveillance Team
EPI_ISL_710619	Lighthouse Lab in Glasgow	Wellcome Sanger Institute for the COVID-19 Genomics UK (COG-UK) Consortium	Harper VanSteenhouse, Yumi Kasai, David Gray, Carol Clugston, Anna Dominiczak and Alex Alderton, Roberto Amato, Sonia Goncalves, Ewan Harrison, David K. Jackson, Ian Johnston, Dominic Kwiatkowski, Cordelia Langford, John Sillitoe on behalf of the Wellcome Sanger Institute COVID-19 Surveillance Team
EPI_ISL_710620	Lighthouse Lab in Alderley Park	Wellcome Sanger Institute for the COVID-19 Genomics UK (COG-UK) Consortium	Jacquelyn Wynn, Mairead Hyland, The Lighthouse Lab in Alderley Park and Alex Alderton, Roberto Amato, Sonia Goncalves, Ewan Harrison, David K. Jackson, Ian Johnston, Dominic Kwiatkowski, Cordelia Langford, John Sillitoe on behalf of the Wellcome Sanger Institute COVID-19 Surveillance Team
EPI_ISL_710621, EPI_ISL_710622, EPI_ISL_710627	Lighthouse Lab in Glasgow	Wellcome Sanger Institute for the COVID-19 Genomics UK (COG-UK) Consortium	Harper VanSteenhouse, Yumi Kasai, David Gray, Carol Clugston, Anna Dominiczak and Alex Alderton, Roberto Amato, Sonia Goncalves, Ewan Harrison, David K. Jackson, Ian Johnston, Dominic Kwiatkowski, Cordelia Langford, John Sillitoe on behalf of the Wellcome Sanger Institute COVID-19 Surveillance Team
EPI_ISL_710628	Lighthouse Lab in Alderley Park	Wellcome Sanger Institute for the COVID-19 Genomics UK (COG-UK) Consortium	Jacquelyn Wynn, Mairead Hyland, The Lighthouse Lab in Alderley Park and Alex Alderton, Roberto Amato, Sonia Goncalves, Ewan Harrison, David K. Jackson, Ian Johnston, Dominic Kwiatkowski, Cordelia Langford, John Sillitoe on behalf of the Wellcome Sanger Institute COVID-19 Surveillance Team
EPI_ISL_710629, EPI_ISL_710633, EPI_ISL_710635, EPI_ISL_710637	Lighthouse Lab in Glasgow	Wellcome Sanger Institute for the COVID-19 Genomics UK (COG-UK) Consortium	Harper VanSteenhouse, Yumi Kasai, David Gray, Carol Clugston, Anna Dominiczak and Alex Alderton, Roberto Amato, Sonia Goncalves, Ewan Harrison, David K. Jackson, Ian Johnston, Dominic Kwiatkowski, Cordelia Langford, John Sillitoe on behalf of the Wellcome Sanger Institute COVID-19 Surveillance Team
EPI_ISL_710638	Lighthouse Lab in Alderley Park	Wellcome Sanger Institute for the COVID-19 Genomics UK (COG-UK) Consortium	Jacquelyn Wynn, Mairead Hyland, The Lighthouse Lab in Alderley Park and Alex Alderton, Roberto Amato, Sonia Goncalves, Ewan Harrison, David K. Jackson, Ian Johnston, Dominic Kwiatkowski, Cordelia Langford, John Sillitoe on behalf of the Wellcome Sanger Institute COVID-19 Surveillance Team
EPI_ISL_710640, EPI_ISL_710641, EPI_ISL_710642, EPI_ISL_710643	Lighthouse Lab in Glasgow	Wellcome Sanger Institute for the COVID-19 Genomics UK (COG-UK) Consortium	Harper VanSteenhouse, Yumi Kasai, David Gray, Carol Clugston, Anna Dominiczak and Alex Alderton, Roberto Amato, Sonia Goncalves, Ewan Harrison, David K. Jackson, Ian Johnston, Dominic Kwiatkowski, Cordelia Langford, John Sillitoe on behalf of the Wellcome Sanger Institute COVID-19 Surveillance Team
EPI_ISL_710645	Lighthouse Lab in Alderley Park	Wellcome Sanger Institute for the COVID-19 Genomics UK (COG-UK) Consortium	Jacquelyn Wynn, Mairead Hyland, The Lighthouse Lab in Alderley Park and Alex Alderton, Roberto Amato, Sonia Goncalves, Ewan Harrison, David K. Jackson, Ian Johnston, Dominic Kwiatkowski, Cordelia Langford, John Sillitoe on behalf of the Wellcome Sanger Institute COVID-19 Surveillance Team
EPI_ISL_710646, EPI_ISL_710651, EPI_ISL_710654, EPI_ISL_710655	Lighthouse Lab in Glasgow	Wellcome Sanger Institute for the COVID-19 Genomics UK (COG-UK) Consortium	Harper VanSteenhouse, Yumi Kasai, David Gray, Carol Clugston, Anna Dominiczak and Alex Alderton, Roberto Amato, Sonia Goncalves, Ewan Harrison, David K. Jackson, Ian Johnston, Dominic Kwiatkowski, Cordelia Langford, John Sillitoe on behalf of the Wellcome Sanger Institute COVID-19 Surveillance Team
EPI_ISL_710656	Lighthouse Lab in Alderley Park	Wellcome Sanger Institute for the COVID-19 Genomics UK (COG-UK) Consortium	Jacquelyn Wynn, Mairead Hyland, The Lighthouse Lab in Alderley Park and Alex Alderton, Roberto Amato, Sonia Goncalves, Ewan Harrison, David K. Jackson, Ian Johnston, Dominic Kwiatkowski, Cordelia Langford, John Sillitoe on behalf of the Wellcome Sanger Institute COVID-19 Surveillance Team
EPI_ISL_710657, EPI_ISL_710658, EPI_ISL_710659, EPI_ISL_710660	Lighthouse Lab in Glasgow	Wellcome Sanger Institute for the COVID-19 Genomics UK (COG-UK) Consortium	Harper VanSteenhouse, Yumi Kasai, David Gray, Carol Clugston, Anna Dominiczak and Alex Alderton, Roberto Amato, Sonia Goncalves, Ewan Harrison, David K. Jackson, Ian Johnston, Dominic Kwiatkowski, Cordelia Langford, John Sillitoe on behalf of the Wellcome Sanger Institute COVID-19 Surveillance Team
EPI_ISL_710661	Lighthouse Lab in Alderley Park	Wellcome Sanger Institute for the COVID-19 Genomics UK (COG-UK) Consortium	Jacquelyn Wynn, Mairead Hyland, The Lighthouse Lab in Alderley Park and Alex Alderton, Roberto Amato, Sonia Goncalves, Ewan Harrison, David K. Jackson, Ian Johnston, Dominic Kwiatkowski, Cordelia Langford, John Sillitoe on behalf of the Wellcome Sanger Institute COVID-19 Surveillance Team
EPI_ISL_710664	Lighthouse Lab in Glasgow	Wellcome Sanger Institute for the COVID-19 Genomics UK (COG-UK) Consortium	Harper VanSteenhouse, Yumi Kasai, David Gray, Carol Clugston, Anna Dominiczak and Alex Alderton, Roberto Amato, Sonia Goncalves, Ewan Harrison, David K. Jackson, Ian Johnston, Dominic Kwiatkowski, Cordelia Langford, John Sillitoe on behalf of the Wellcome Sanger Institute COVID-19 Surveillance Team
EPI_ISL_710665	Lighthouse Lab in Alderley Park	Wellcome Sanger Institute for the COVID-19 Genomics UK (COG-UK) Consortium	Jacquelyn Wynn, Mairead Hyland, The Lighthouse Lab in Alderley Park and Alex Alderton, Roberto Amato, Sonia Goncalves, Ewan Harrison, David K. Jackson, Ian Johnston, Dominic Kwiatkowski, Cordelia Langford, John Sillitoe on behalf of the Wellcome Sanger Institute COVID-19 Surveillance Team
EPI_ISL_710666, EPI_ISL_710667, EPI_ISL_710668, EPI_ISL_710670	Lighthouse Lab in Glasgow	Wellcome Sanger Institute for the COVID-19 Genomics UK (COG-UK) Consortium	Harper VanSteenhouse, Yumi Kasai, David Gray, Carol Clugston, Anna Dominiczak and Alex Alderton, Roberto Amato, Sonia Goncalves, Ewan Harrison, David K. Jackson, Ian Johnston, Dominic Kwiatkowski, Cordelia Langford, John Sillitoe on behalf of the Wellcome Sanger Institute COVID-19 Surveillance Team
EPI_ISL_710671	Lighthouse Lab in Alderley Park	Wellcome Sanger Institute for the COVID-19 Genomics UK (COG-UK) Consortium	Jacquelyn Wynn, Mairead Hyland, The Lighthouse Lab in Alderley Park and Alex Alderton, Roberto Amato, Sonia Goncalves, Ewan Harrison, David K. Jackson, Ian Johnston, Dominic Kwiatkowski, Cordelia Langford, John Sillitoe on behalf of the Wellcome Sanger Institute COVID-19 Surveillance Team
EPI_ISL_710672, EPI_ISL_710673, EPI_ISL_710675	Lighthouse Lab in Glasgow	Wellcome Sanger Institute for the COVID-19 Genomics UK (COG-UK) Consortium	Harper VanSteenhouse, Yumi Kasai, David Gray, Carol Clugston, Anna Dominiczak and Alex Alderton, Roberto Amato, Sonia Goncalves, Ewan Harrison, David K. Jackson, Ian Johnston, Dominic Kwiatkowski, Cordelia Langford, John Sillitoe on behalf of the Wellcome Sanger Institute COVID-19 Surveillance Team
EPI_ISL_710676	Lighthouse Lab in Alderley Park	Wellcome Sanger Institute for the COVID-19 Genomics UK (COG-UK) Consortium	Jacquelyn Wynn, Mairead Hyland, The Lighthouse Lab in Alderley Park and Alex Alderton, Roberto Amato, Sonia Goncalves, Ewan Harrison, David K. Jackson, Ian Johnston, Dominic Kwiatkowski, Cordelia Langford, John Sillitoe on behalf of the Wellcome Sanger Institute COVID-19 Surveillance Team
EPI_ISL_710677, EPI_ISL_710679, EPI_ISL_710684, EPI_ISL_710685, EPI_ISL_710686	Lighthouse Lab in Glasgow	Wellcome Sanger Institute for the COVID-19 Genomics UK (COG-UK) Consortium	Harper VanSteenhouse, Yumi Kasai, David Gray, Carol Clugston, Anna Dominiczak and Alex Alderton, Roberto Amato, Sonia Goncalves, Ewan Harrison, David K. Jackson, Ian Johnston, Dominic Kwiatkowski, Cordelia Langford, John Sillitoe on behalf of the Wellcome Sanger Institute COVID-19 Surveillance Team
EPI_ISL_710689	Lighthouse Lab in Alderley Park	Wellcome Sanger Institute for the COVID-19 Genomics UK (COG-UK) Consortium	Jacquelyn Wynn, Mairead Hyland, The Lighthouse Lab in Alderley Park and Alex Alderton, Roberto Amato, Sonia Goncalves, Ewan Harrison, David K. Jackson, Ian Johnston, Dominic Kwiatkowski, Cordelia Langford, John Sillitoe on behalf of the Wellcome Sanger Institute COVID-19 Surveillance Team
EPI_ISL_710690, EPI_ISL_710691	Lighthouse Lab in Glasgow	Wellcome Sanger Institute for the COVID-19 Genomics UK (COG-UK) Consortium	Harper VanSteenhouse, Yumi Kasai, David Gray, Carol Clugston, Anna Dominiczak and Alex Alderton, Roberto Amato, Sonia Goncalves, Ewan Harrison, David K. Jackson, Ian Johnston, Dominic Kwiatkowski, Cordelia Langford, John Sillitoe on behalf of the Wellcome Sanger Institute COVID-19 Surveillance Team
EPI_ISL_710693	Lighthouse Lab in Alderley Park	Wellcome Sanger Institute for the COVID-19 Genomics UK (COG-UK) Consortium	Jacquelyn Wynn, Mairead Hyland, The Lighthouse Lab in Alderley Park and Alex Alderton, Roberto Amato, Sonia Goncalves, Ewan Harrison, David K. Jackson, Ian Johnston, Dominic Kwiatkowski, Cordelia Langford, John Sillitoe on behalf of the Wellcome Sanger Institute COVID-19 Surveillance Team
EPI_ISL_710697	Lighthouse Lab in Glasgow	Wellcome Sanger Institute for the COVID-19 Genomics UK (COG-UK) Consortium	Harper VanSteenhouse, Yumi Kasai, David Gray, Carol Clugston, Anna Dominiczak and Alex Alderton, Roberto Amato, Sonia Goncalves, Ewan Harrison, David K. Jackson, Ian Johnston, Dominic Kwiatkowski, Cordelia Langford, John Sillitoe on behalf of the Wellcome Sanger Institute COVID-19 Surveillance Team
EPI_ISL_710698	Lighthouse Lab in Alderley Park	Wellcome Sanger Institute for the COVID-19 Genomics UK (COG-UK) Consortium	Jacquelyn Wynn, Mairead Hyland, The Lighthouse Lab in Alderley Park and Alex Alderton, Roberto Amato, Sonia Goncalves, Ewan Harrison, David K. Jackson, Ian Johnston, Dominic Kwiatkowski, Cordelia Langford, John Sillitoe on behalf of the Wellcome Sanger Institute COVID-19 Surveillance Team

EPI_ISL_712250, EPI_ISL_712251, EPI_ISL_712252, EPI_ISL_712253, EPI_ISL_712295, EPI_ISL_712321, EPI_ISL_712340, EPI_ISL_712341, EPI_ISL_712342, EPI_ISL_712344, EPI_ISL_712345, EPI_ISL_712346, EPI_ISL_712455, EPI_ISL_712456, EPI_ISL_712457, EPI_ISL_712458, EPI_ISL_712459, EPI_ISL_712460, EPI_ISL_712461, EPI_ISL_712462, EPI_ISL_712463, EPI_ISL_712464, EPI_ISL_712465, EPI_ISL_712466, EPI_ISL_712467, EPI_ISL_712468, EPI_ISL_712469, EPI_ISL_712470, EPI_ISL_712471, EPI_ISL_712472, EPI_ISL_712473, EPI_ISL_712474, EPI_ISL_712475, EPI_ISL_712476, EPI_ISL_712477, EPI_ISL_712478, EPI_ISL_712479, EPI_ISL_712481, EPI_ISL_712482, EPI_ISL_712483, EPI_ISL_712484, EPI_ISL_712487, EPI_ISL_712488, EPI_ISL_712489, EPI_ISL_712490, EPI_ISL_712491, EPI_ISL_712492, EPI_ISL_712493, EPI_ISL_712494, EPI_ISL_712495, EPI_ISL_712496, EPI_ISL_712497, EPI_ISL_712498, EPI_ISL_712499, EPI_ISL_712500, EPI_ISL_712501, EPI_ISL_712502, EPI_ISL_712503, EPI_ISL_712504, EPI_ISL_712505, EPI_ISL_712506, EPI_ISL_712507, EPI_ISL_712508, EPI_ISL_712509, EPI_ISL_712511, EPI_ISL_712512, EPI_ISL_712513, EPI_ISL_712514, EPI_ISL_712515, EPI_ISL_712516, EPI_ISL_712517, EPI_ISL_712518, EPI_ISL_712519, EPI_ISL_712520, EPI_ISL_712521, EPI_ISL_712522, EPI_ISL_712523, EPI_ISL_712524, EPI_ISL_712525, EPI_ISL_712526, EPI_ISL_712527, EPI_ISL_712528, EPI_ISL_712529, EPI_ISL_712530			
see above	Department of Virus and Microbiological Special Diagnostics, Statens Serum Institut, Copenhagen, Denmark	Albertsen Lab, Department of Chemistry and Bioscience, Aalborg University, Denmark	Danish Covid-19 Genome Consortium
EPI_ISL_717701, EPI_ISL_717704, EPI_ISL_717705, EPI_ISL_717706, EPI_ISL_717708, EPI_ISL_717710, EPI_ISL_717711, EPI_ISL_717713	Area of Virology, Serology and Virology Division (SAViD), New South Wales Health Pathology Randwick	Virology Research Laboratory; Area of Virology, Serology and Virology Division (SAViD), New South Wales Health Pathology Randwick	Foster, C.; Au, J.; Ruiz Silva, M.; Deveson, I.; Bull, R.; Van Hal, S.; Rawlinson, W.
EPI_ISL_717978	Army Medical Center, Scientific Department, Virology Laboratory	Army Medical Center, Scientific Department, Virology Laboratory	Silvia Fillo, Giovanni Faggioni, Riccardo De Santis, Antonella Fortunato, Anna Anselmo, Vanessa Vera Fain, Francesco Giordani, Nino D'Amore, Anella Monte, Marzia Cavalli, Alessandra Amoroso, Stella Lia, Roberta Sorrentino, Rossella Tirelli, Federica Galeano, Annalisa Pelo, Margherita De Santis, Giulia Campoli, Andrea Ciammaruconi, Florigio Lista.
EPI_ISL_718024	Lab voor klinische biologie	Onderzoeksgroep Virologie	Laurens Lambrechts, Nick Vereecke, Marthe Pauwels, Bruno Verhasselt, Linos Vandekerckhove, Hans Nauwynck, Sebastiaan Theuns
EPI_ISL_718026	Lab voor klinische biologie	Onderzoeksgroep Virologie	Nick Vereecke, Laurens Lambrechts, Marthe Pauwels, Bruno Verhasselt, Linos Vandekerckhove, Hans Nauwynck, Sebastiaan Theuns
EPI_ISL_718280	Institute for Medical Research, Infectious Disease Research Centre, National Institutes of Health, Ministry of Health Malaysia	Institute for Medical Research, Infectious Disease Research Centre, National Institutes of Health, Ministry of Health Malaysia	Suppiah J, Kamel K, Mohd-Zawawi Z, Thayan R
EPI_ISL_718318, EPI_ISL_718320, EPI_ISL_718321, EPI_ISL_718322, EPI_ISL_718323, EPI_ISL_718324, EPI_ISL_718325, EPI_ISL_718327, EPI_ISL_718328, EPI_ISL_718329, EPI_ISL_718330, EPI_ISL_718331, EPI_ISL_718333, EPI_ISL_718334, EPI_ISL_718335, EPI_ISL_718336, EPI_ISL_718339, EPI_ISL_718340, EPI_ISL_718341, EPI_ISL_718342, EPI_ISL_718343, EPI_ISL_718344, EPI_ISL_718346, EPI_ISL_718347, EPI_ISL_718348, EPI_ISL_718349, EPI_ISL_718350, EPI_ISL_718351, EPI_ISL_718352, EPI_ISL_718355, EPI_ISL_718356, EPI_ISL_718357, EPI_ISL_718359, EPI_ISL_718360, EPI_ISL_718361, EPI_ISL_718364, EPI_ISL_718366, EPI_ISL_718367, EPI_ISL_718368, EPI_ISL_718369, EPI_ISL_718370, EPI_ISL_718371, EPI_ISL_718372, EPI_ISL_718374, EPI_ISL_718376, EPI_ISL_718377, EPI_ISL_718378, EPI_ISL_718379, EPI_ISL_718380, EPI_ISL_718381, EPI_ISL_718382, EPI_ISL_718383, EPI_ISL_718384, EPI_ISL_718386, EPI_ISL_718387, EPI_ISL_718388, EPI_ISL_718390, EPI_ISL_718391, EPI_ISL_718392, EPI_ISL_718394, EPI_ISL_718395, EPI_ISL_718397, EPI_ISL_718399, EPI_ISL_718401, EPI_ISL_718405, EPI_ISL_718406, EPI_ISL_718407, EPI_ISL_718408, EPI_ISL_718409, EPI_ISL_718410, EPI_ISL_718413, EPI_ISL_718415, EPI_ISL_718416, EPI_ISL_718417, EPI_ISL_718419, EPI_ISL_718420, EPI_ISL_718422, EPI_ISL_718423, EPI_ISL_718424, EPI_ISL_718425, EPI_ISL_718426, EPI_ISL_718427, EPI_ISL_718429, EPI_ISL_718431, EPI_ISL_718433, EPI_ISL_718434, EPI_ISL_718435, EPI_ISL_718436, EPI_ISL_718437, EPI_ISL_718438, EPI_ISL_718439, EPI_ISL_718440, EPI_ISL_718441, EPI_ISL_718444, EPI_ISL_718445, EPI_ISL_718446, EPI_ISL_718448, EPI_ISL_718449, EPI_ISL_718450, EPI_ISL_718451, EPI_ISL_718452, EPI_ISL_718453, EPI_ISL_718454, EPI_ISL_718455, EPI_ISL_718456, EPI_ISL_718459, EPI_ISL_718461, EPI_ISL_718462, EPI_ISL_718463, EPI_ISL_718465, EPI_ISL_718466, EPI_ISL_718467, EPI_ISL_718469, EPI_ISL_718470, EPI_ISL_718471, EPI_ISL_718472, EPI_ISL_718474, EPI_ISL_718475, EPI_ISL_718477, EPI_ISL_718478, EPI_ISL_718480, EPI_ISL_718481, EPI_ISL_718482, EPI_ISL_718483, EPI_ISL_718485, EPI_ISL_718487, EPI_ISL_718488, EPI_ISL_718489, EPI_ISL_718490, EPI_ISL_718491, EPI_ISL_718493, EPI_ISL_718494, EPI_ISL_718495, EPI_ISL_718496, EPI_ISL_718497, EPI_ISL_718499, EPI_ISL_718505, EPI_ISL_718507, EPI_ISL_718508, EPI_ISL_718512, EPI_ISL_718513, EPI_ISL_718515, EPI_ISL_718517, EPI_ISL_718518, EPI_ISL_718520, EPI_ISL_718521, EPI_ISL_718522, EPI_ISL_718524, EPI_ISL_718525, EPI_ISL_718526, EPI_ISL_718527, EPI_ISL_718529, EPI_ISL_718530, EPI_ISL_718531, EPI_ISL_718533, EPI_ISL_718534, EPI_ISL_718536, EPI_ISL_718537, EPI_ISL_718538, EPI_ISL_718539, EPI_ISL_718540, EPI_ISL_718542, EPI_ISL_718544, EPI_ISL_718545, EPI_ISL_718546, EPI_ISL_718547, EPI_ISL_718548, EPI_ISL_718549, EPI_ISL_718551, EPI_ISL_718553, EPI_ISL_718554, EPI_ISL_718555, EPI_ISL_718556, EPI_ISL_718558, EPI_ISL_718560, EPI_ISL_718561, EPI_ISL_718562, EPI_ISL_718565, EPI_ISL_718568, EPI_ISL_718570, EPI_ISL_718573, EPI_ISL_718574, EPI_ISL_718576, EPI_ISL_718577, EPI_ISL_718580, EPI_ISL_718581, EPI_ISL_718583, EPI_ISL_718585, EPI_ISL_718586, EPI_ISL_718587, EPI_ISL_718588, EPI_ISL_718589, EPI_ISL_718590, EPI_ISL_718591, EPI_ISL_718592, EPI_ISL_718594, EPI_ISL_718596, EPI_ISL_718603, EPI_ISL_718604, EPI_ISL_718608, EPI_ISL_718613, EPI_ISL_718614, EPI_ISL_718616, EPI_ISL_718619, EPI_ISL_718620, EPI_ISL_718623, EPI_ISL_718626, EPI_ISL_718632			
see above	Lighthouse Lab in Cambridge	Wellcome Sanger Institute for the COVID-19 Genomics UK (COG-UK) Consortium	Rob Howes, The Lighthouse Lab in Cambridge and Alex Alderton, Roberto Amato, Sonia Goncalves, Ewan Harrison, David K. Jackson, Ian Johnston, Dominic Kwiatkowski, Cordelia Langford, John Sillitoe on behalf of the Wellcome Sanger Institute COVID-19 Surveillance Team
EPI_ISL_718637, EPI_ISL_718639, EPI_ISL_718640, EPI_ISL_718643, EPI_ISL_718644	Lighthouse Lab in Alderley Park	Wellcome Sanger Institute for the COVID-19 Genomics UK (COG-UK) Consortium	Jacquelyn Wynn, Mairead Hyland, The Lighthouse Lab in Alderley Park and Alex Alderton, Roberto Amato, Sonia Goncalves, Ewan Harrison, David K. Jackson, Ian Johnston, Dominic Kwiatkowski, Cordelia Langford, John Sillitoe on behalf of the Wellcome Sanger Institute COVID-19 Surveillance Team
EPI_ISL_718645, EPI_ISL_718646, EPI_ISL_718648, EPI_ISL_718649, EPI_ISL_718651	Lighthouse Lab in Cambridge	Wellcome Sanger Institute for the COVID-19 Genomics UK (COG-UK) Consortium	Rob Howes, The Lighthouse Lab in Cambridge and Alex Alderton, Roberto Amato, Sonia Goncalves, Ewan Harrison, David K. Jackson, Ian Johnston, Dominic Kwiatkowski, Cordelia Langford, John Sillitoe on behalf of the Wellcome Sanger Institute COVID-19 Surveillance Team
EPI_ISL_718653	Lighthouse Lab in Alderley Park	Wellcome Sanger Institute for the COVID-19 Genomics UK (COG-UK) Consortium	Jacquelyn Wynn, Mairead Hyland, The Lighthouse Lab in Alderley Park and Alex Alderton, Roberto Amato, Sonia Goncalves, Ewan Harrison, David K. Jackson, Ian Johnston, Dominic Kwiatkowski, Cordelia Langford, John Sillitoe on behalf of the Wellcome Sanger Institute COVID-19 Surveillance Team
EPI_ISL_718655, EPI_ISL_718656	Lighthouse Lab in Cambridge	Wellcome Sanger Institute for the COVID-19 Genomics UK (COG-UK) Consortium	Rob Howes, The Lighthouse Lab in Cambridge and Alex Alderton, Roberto Amato, Sonia Goncalves, Ewan Harrison, David K. Jackson, Ian Johnston, Dominic Kwiatkowski, Cordelia Langford, John Sillitoe on behalf of the Wellcome Sanger Institute COVID-19 Surveillance Team
EPI_ISL_718657, EPI_ISL_718658	Lighthouse Lab in Alderley Park	Wellcome Sanger Institute for the COVID-19 Genomics UK (COG-UK) Consortium	Jacquelyn Wynn, Mairead Hyland, The Lighthouse Lab in Alderley Park and Alex Alderton, Roberto Amato, Sonia Goncalves, Ewan Harrison, David K. Jackson, Ian Johnston, Dominic Kwiatkowski, Cordelia Langford, John Sillitoe on behalf of the Wellcome Sanger Institute COVID-19 Surveillance Team
EPI_ISL_718659, EPI_ISL_718660, EPI_ISL_718661	Lighthouse Lab in Cambridge	Wellcome Sanger Institute for the COVID-19 Genomics UK (COG-UK) Consortium	Rob Howes, The Lighthouse Lab in Cambridge and Alex Alderton, Roberto Amato, Sonia Goncalves, Ewan Harrison, David K. Jackson, Ian Johnston, Dominic Kwiatkowski, Cordelia Langford, John Sillitoe on behalf of the Wellcome Sanger Institute COVID-19 Surveillance Team
EPI_ISL_718662, EPI_ISL_718663, EPI_ISL_718664, EPI_ISL_718665, EPI_ISL_718666	Lighthouse Lab in Alderley Park	Wellcome Sanger Institute for the COVID-19 Genomics UK (COG-UK) Consortium	Jacquelyn Wynn, Mairead Hyland, The Lighthouse Lab in Alderley Park and Alex Alderton, Roberto Amato, Sonia Goncalves, Ewan Harrison, David K. Jackson, Ian Johnston, Dominic Kwiatkowski, Cordelia Langford, John Sillitoe on behalf of the Wellcome Sanger Institute COVID-19 Surveillance Team
EPI_ISL_718667, EPI_ISL_718668, EPI_ISL_718669	Lighthouse Lab in Cambridge	Wellcome Sanger Institute for the COVID-19 Genomics UK (COG-UK) Consortium	Rob Howes, The Lighthouse Lab in Cambridge and Alex Alderton, Roberto Amato, Sonia Goncalves, Ewan Harrison, David K. Jackson, Ian Johnston, Dominic Kwiatkowski, Cordelia Langford, John Sillitoe on behalf of the Wellcome Sanger Institute COVID-19 Surveillance Team
EPI_ISL_718670	Lighthouse Lab in Alderley Park	Wellcome Sanger Institute for the COVID-19 Genomics UK (COG-UK) Consortium	Jacquelyn Wynn, Mairead Hyland, The Lighthouse Lab in Alderley Park and Alex Alderton, Roberto Amato, Sonia Goncalves, Ewan Harrison, David K. Jackson, Ian Johnston, Dominic Kwiatkowski, Cordelia Langford, John Sillitoe on behalf of the Wellcome Sanger Institute COVID-19 Surveillance Team
EPI_ISL_718671	Lighthouse Lab in Cambridge	Wellcome Sanger Institute for the COVID-19 Genomics UK (COG-UK) Consortium	Rob Howes, The Lighthouse Lab in Cambridge and Alex Alderton, Roberto Amato, Sonia Goncalves, Ewan Harrison, David K. Jackson, Ian Johnston, Dominic Kwiatkowski, Cordelia Langford, John Sillitoe on behalf of the Wellcome Sanger Institute COVID-19 Surveillance Team
EPI_ISL_718674, EPI_ISL_718676, EPI_ISL_718678	Lighthouse Lab in Alderley Park	Wellcome Sanger Institute for the COVID-19 Genomics UK (COG-UK) Consortium	Jacquelyn Wynn, Mairead Hyland, The Lighthouse Lab in Alderley Park and Alex Alderton, Roberto Amato, Sonia Goncalves, Ewan Harrison, David K. Jackson, Ian Johnston, Dominic Kwiatkowski, Cordelia Langford, John Sillitoe on behalf of the Wellcome Sanger Institute COVID-19 Surveillance Team
EPI_ISL_718679, EPI_ISL_718680, EPI_ISL_718681, EPI_ISL_718683, EPI_ISL_718684	Lighthouse Lab in Cambridge	Wellcome Sanger Institute for the COVID-19 Genomics UK (COG-UK) Consortium	Rob Howes, The Lighthouse Lab in Cambridge and Alex Alderton, Roberto Amato, Sonia Goncalves, Ewan Harrison, David K. Jackson, Ian Johnston, Dominic Kwiatkowski, Cordelia Langford, John Sillitoe on behalf of the Wellcome Sanger Institute COVID-19 Surveillance Team
EPI_ISL_718686, EPI_ISL_718687	Lighthouse Lab in Alderley Park	Wellcome Sanger Institute for the COVID-19 Genomics UK (COG-UK) Consortium	Jacquelyn Wynn, Mairead Hyland, The Lighthouse Lab in Alderley Park and Alex Alderton, Roberto Amato, Sonia Goncalves, Ewan Harrison, David K. Jackson, Ian Johnston, Dominic Kwiatkowski, Cordelia Langford, John Sillitoe on behalf of the Wellcome Sanger Institute COVID-19 Surveillance Team
EPI_ISL_718688, EPI_ISL_718689, EPI_ISL_718690	Lighthouse Lab in Cambridge	Wellcome Sanger Institute for the COVID-19 Genomics UK (COG-UK) Consortium	Rob Howes, The Lighthouse Lab in Cambridge and Alex Alderton, Roberto Amato, Sonia Goncalves, Ewan Harrison, David K. Jackson, Ian Johnston, Dominic Kwiatkowski, Cordelia Langford, John Sillitoe on behalf of the Wellcome Sanger Institute COVID-19 Surveillance Team
EPI_ISL_718691	Lighthouse Lab in Alderley Park	Wellcome Sanger Institute for the COVID-19 Genomics UK (COG-UK) Consortium	Jacquelyn Wynn, Mairead Hyland, The Lighthouse Lab in Alderley Park and Alex Alderton, Roberto Amato, Sonia Goncalves, Ewan Harrison, David K. Jackson, Ian Johnston, Dominic Kwiatkowski, Cordelia Langford, John Sillitoe on behalf of the Wellcome Sanger Institute COVID-19 Surveillance Team
EPI_ISL_718692, EPI_ISL_718693	Lighthouse Lab in Cambridge	Wellcome Sanger Institute for the COVID-19 Genomics UK (COG-UK) Consortium	Rob Howes, The Lighthouse Lab in Cambridge and Alex Alderton, Roberto Amato, Sonia Goncalves, Ewan Harrison, David K. Jackson, Ian Johnston, Dominic Kwiatkowski, Cordelia Langford, John Sillitoe on behalf of the Wellcome Sanger Institute COVID-19 Surveillance Team
EPI_ISL_718694, EPI_ISL_718695	Lighthouse Lab in Alderley Park	Wellcome Sanger Institute for the COVID-19 Genomics UK (COG-UK) Consortium	Jacquelyn Wynn, Mairead Hyland, The Lighthouse Lab in Alderley Park and Alex Alderton, Roberto Amato, Sonia Goncalves, Ewan Harrison, David K. Jackson, Ian Johnston, Dominic Kwiatkowski, Cordelia Langford, John Sillitoe on behalf of the Wellcome Sanger Institute COVID-19 Surveillance Team
EPI_ISL_718696, EPI_ISL_718697, EPI_ISL_718698, EPI_ISL_718699	Lighthouse Lab in Cambridge	Wellcome Sanger Institute for the COVID-19 Genomics UK (COG-UK) Consortium	Rob Howes, The Lighthouse Lab in Cambridge and Alex Alderton, Roberto Amato, Sonia Goncalves, Ewan Harrison, David K. Jackson, Ian Johnston, Dominic Kwiatkowski, Cordelia Langford, John Sillitoe on behalf of the Wellcome Sanger Institute COVID-19 Surveillance Team
EPI_ISL_718700	Lighthouse Lab in Alderley Park	Wellcome Sanger Institute for the COVID-19 Genomics UK	Jacquelyn Wynn, Mairead Hyland, The Lighthouse Lab in Alderley Park and Alex Alderton, Roberto Amato, Sonia Goncalves, Ewan Harrison, David K.

		(COG-UK) Consortium	Jackson, Ian Johnston, Dominic Kwiatkowski, Cordelia Langford, John Sillitoe on behalf of the Wellcome Sanger Institute COVID-19 Surveillance Team
EPI_ISL_718701, EPI_ISL_718702, EPI_ISL_718703, EPI_ISL_718704	Lighthouse Lab in Cambridge	Wellcome Sanger Institute for the COVID-19 Genomics UK (COG-UK) Consortium	Rob Howes, The Lighthouse Lab in Cambridge and Alex Alderton, Roberto Amato, Sonia Goncalves, Ewan Harrison, David K. Jackson, Ian Johnston, Dominic Kwiatkowski, Cordelia Langford, John Sillitoe on behalf of the Wellcome Sanger Institute COVID-19 Surveillance Team
EPI_ISL_718707	Lighthouse Lab in Alderley Park	Wellcome Sanger Institute for the COVID-19 Genomics UK (COG-UK) Consortium	Jacquelyn Wynn, Mairead Hyland, The Lighthouse Lab in Alderley Park and Alex Alderton, Roberto Amato, Sonia Goncalves, Ewan Harrison, David K. Jackson, Ian Johnston, Dominic Kwiatkowski, Cordelia Langford, John Sillitoe on behalf of the Wellcome Sanger Institute COVID-19 Surveillance Team
EPI_ISL_718708	Lighthouse Lab in Cambridge	Wellcome Sanger Institute for the COVID-19 Genomics UK (COG-UK) Consortium	Rob Howes, The Lighthouse Lab in Cambridge and Alex Alderton, Roberto Amato, Sonia Goncalves, Ewan Harrison, David K. Jackson, Ian Johnston, Dominic Kwiatkowski, Cordelia Langford, John Sillitoe on behalf of the Wellcome Sanger Institute COVID-19 Surveillance Team
EPI_ISL_718710, EPI_ISL_718711, EPI_ISL_718712, EPI_ISL_718713, EPI_ISL_718714, EPI_ISL_718715, EPI_ISL_718716	Lighthouse Lab in Alderley Park	Wellcome Sanger Institute for the COVID-19 Genomics UK (COG-UK) Consortium	Jacquelyn Wynn, Mairead Hyland, The Lighthouse Lab in Alderley Park and Alex Alderton, Roberto Amato, Sonia Goncalves, Ewan Harrison, David K. Jackson, Ian Johnston, Dominic Kwiatkowski, Cordelia Langford, John Sillitoe on behalf of the Wellcome Sanger Institute COVID-19 Surveillance Team
EPI_ISL_718717	Lighthouse Lab in Cambridge	Wellcome Sanger Institute for the COVID-19 Genomics UK (COG-UK) Consortium	Rob Howes, The Lighthouse Lab in Cambridge and Alex Alderton, Roberto Amato, Sonia Goncalves, Ewan Harrison, David K. Jackson, Ian Johnston, Dominic Kwiatkowski, Cordelia Langford, John Sillitoe on behalf of the Wellcome Sanger Institute COVID-19 Surveillance Team
EPI_ISL_718718, EPI_ISL_718719, EPI_ISL_718721	Lighthouse Lab in Alderley Park	Wellcome Sanger Institute for the COVID-19 Genomics UK (COG-UK) Consortium	Jacquelyn Wynn, Mairead Hyland, The Lighthouse Lab in Alderley Park and Alex Alderton, Roberto Amato, Sonia Goncalves, Ewan Harrison, David K. Jackson, Ian Johnston, Dominic Kwiatkowski, Cordelia Langford, John Sillitoe on behalf of the Wellcome Sanger Institute COVID-19 Surveillance Team
EPI_ISL_718722	Lighthouse Lab in Cambridge	Wellcome Sanger Institute for the COVID-19 Genomics UK (COG-UK) Consortium	Rob Howes, The Lighthouse Lab in Cambridge and Alex Alderton, Roberto Amato, Sonia Goncalves, Ewan Harrison, David K. Jackson, Ian Johnston, Dominic Kwiatkowski, Cordelia Langford, John Sillitoe on behalf of the Wellcome Sanger Institute COVID-19 Surveillance Team
EPI_ISL_718724, EPI_ISL_718725, EPI_ISL_718728, EPI_ISL_718729	Lighthouse Lab in Alderley Park	Wellcome Sanger Institute for the COVID-19 Genomics UK (COG-UK) Consortium	Jacquelyn Wynn, Mairead Hyland, The Lighthouse Lab in Alderley Park and Alex Alderton, Roberto Amato, Sonia Goncalves, Ewan Harrison, David K. Jackson, Ian Johnston, Dominic Kwiatkowski, Cordelia Langford, John Sillitoe on behalf of the Wellcome Sanger Institute COVID-19 Surveillance Team
EPI_ISL_718730, EPI_ISL_718732, EPI_ISL_718733, EPI_ISL_718734	Lighthouse Lab in Cambridge	Wellcome Sanger Institute for the COVID-19 Genomics UK (COG-UK) Consortium	Rob Howes, The Lighthouse Lab in Cambridge and Alex Alderton, Roberto Amato, Sonia Goncalves, Ewan Harrison, David K. Jackson, Ian Johnston, Dominic Kwiatkowski, Cordelia Langford, John Sillitoe on behalf of the Wellcome Sanger Institute COVID-19 Surveillance Team
EPI_ISL_718735, EPI_ISL_718738, EPI_ISL_718739, EPI_ISL_718740	Lighthouse Lab in Alderley Park	Wellcome Sanger Institute for the COVID-19 Genomics UK (COG-UK) Consortium	Jacquelyn Wynn, Mairead Hyland, The Lighthouse Lab in Alderley Park and Alex Alderton, Roberto Amato, Sonia Goncalves, Ewan Harrison, David K. Jackson, Ian Johnston, Dominic Kwiatkowski, Cordelia Langford, John Sillitoe on behalf of the Wellcome Sanger Institute COVID-19 Surveillance Team
EPI_ISL_718741	Lighthouse Lab in Cambridge	Wellcome Sanger Institute for the COVID-19 Genomics UK (COG-UK) Consortium	Rob Howes, The Lighthouse Lab in Cambridge and Alex Alderton, Roberto Amato, Sonia Goncalves, Ewan Harrison, David K. Jackson, Ian Johnston, Dominic Kwiatkowski, Cordelia Langford, John Sillitoe on behalf of the Wellcome Sanger Institute COVID-19 Surveillance Team
EPI_ISL_718742	Lighthouse Lab in Alderley Park	Wellcome Sanger Institute for the COVID-19 Genomics UK (COG-UK) Consortium	Jacquelyn Wynn, Mairead Hyland, The Lighthouse Lab in Alderley Park and Alex Alderton, Roberto Amato, Sonia Goncalves, Ewan Harrison, David K. Jackson, Ian Johnston, Dominic Kwiatkowski, Cordelia Langford, John Sillitoe on behalf of the Wellcome Sanger Institute COVID-19 Surveillance Team
EPI_ISL_718743	Lighthouse Lab in Cambridge	Wellcome Sanger Institute for the COVID-19 Genomics UK (COG-UK) Consortium	Rob Howes, The Lighthouse Lab in Cambridge and Alex Alderton, Roberto Amato, Sonia Goncalves, Ewan Harrison, David K. Jackson, Ian Johnston, Dominic Kwiatkowski, Cordelia Langford, John Sillitoe on behalf of the Wellcome Sanger Institute COVID-19 Surveillance Team
EPI_ISL_718745, EPI_ISL_718746, EPI_ISL_718747	Lighthouse Lab in Alderley Park	Wellcome Sanger Institute for the COVID-19 Genomics UK (COG-UK) Consortium	Jacquelyn Wynn, Mairead Hyland, The Lighthouse Lab in Alderley Park and Alex Alderton, Roberto Amato, Sonia Goncalves, Ewan Harrison, David K. Jackson, Ian Johnston, Dominic Kwiatkowski, Cordelia Langford, John Sillitoe on behalf of the Wellcome Sanger Institute COVID-19 Surveillance Team
EPI_ISL_718748, EPI_ISL_718749	Lighthouse Lab in Cambridge	Wellcome Sanger Institute for the COVID-19 Genomics UK (COG-UK) Consortium	Rob Howes, The Lighthouse Lab in Cambridge and Alex Alderton, Roberto Amato, Sonia Goncalves, Ewan Harrison, David K. Jackson, Ian Johnston, Dominic Kwiatkowski, Cordelia Langford, John Sillitoe on behalf of the Wellcome Sanger Institute COVID-19 Surveillance Team
EPI_ISL_718750	Lighthouse Lab in Alderley Park	Wellcome Sanger Institute for the COVID-19 Genomics UK (COG-UK) Consortium	Jacquelyn Wynn, Mairead Hyland, The Lighthouse Lab in Alderley Park and Alex Alderton, Roberto Amato, Sonia Goncalves, Ewan Harrison, David K. Jackson, Ian Johnston, Dominic Kwiatkowski, Cordelia Langford, John Sillitoe on behalf of the Wellcome Sanger Institute COVID-19 Surveillance Team
EPI_ISL_718751, EPI_ISL_718752, EPI_ISL_718756, EPI_ISL_718758	Lighthouse Lab in Cambridge	Wellcome Sanger Institute for the COVID-19 Genomics UK (COG-UK) Consortium	Rob Howes, The Lighthouse Lab in Cambridge and Alex Alderton, Roberto Amato, Sonia Goncalves, Ewan Harrison, David K. Jackson, Ian Johnston, Dominic Kwiatkowski, Cordelia Langford, John Sillitoe on behalf of the Wellcome Sanger Institute COVID-19 Surveillance Team
EPI_ISL_718759	Lighthouse Lab in Alderley Park	Wellcome Sanger Institute for the COVID-19 Genomics UK (COG-UK) Consortium	Jacquelyn Wynn, Mairead Hyland, The Lighthouse Lab in Alderley Park and Alex Alderton, Roberto Amato, Sonia Goncalves, Ewan Harrison, David K. Jackson, Ian Johnston, Dominic Kwiatkowski, Cordelia Langford, John Sillitoe on behalf of the Wellcome Sanger Institute COVID-19 Surveillance Team
EPI_ISL_718760	Lighthouse Lab in Cambridge	Wellcome Sanger Institute for the COVID-19 Genomics UK (COG-UK) Consortium	Rob Howes, The Lighthouse Lab in Cambridge and Alex Alderton, Roberto Amato, Sonia Goncalves, Ewan Harrison, David K. Jackson, Ian Johnston, Dominic Kwiatkowski, Cordelia Langford, John Sillitoe on behalf of the Wellcome Sanger Institute COVID-19 Surveillance Team
EPI_ISL_718761, EPI_ISL_718762, EPI_ISL_718765, EPI_ISL_718766, EPI_ISL_718767	Lighthouse Lab in Alderley Park	Wellcome Sanger Institute for the COVID-19 Genomics UK (COG-UK) Consortium	Jacquelyn Wynn, Mairead Hyland, The Lighthouse Lab in Alderley Park and Alex Alderton, Roberto Amato, Sonia Goncalves, Ewan Harrison, David K. Jackson, Ian Johnston, Dominic Kwiatkowski, Cordelia Langford, John Sillitoe on behalf of the Wellcome Sanger Institute COVID-19 Surveillance Team
EPI_ISL_718768	Lighthouse Lab in Cambridge	Wellcome Sanger Institute for the COVID-19 Genomics UK (COG-UK) Consortium	Rob Howes, The Lighthouse Lab in Cambridge and Alex Alderton, Roberto Amato, Sonia Goncalves, Ewan Harrison, David K. Jackson, Ian Johnston, Dominic Kwiatkowski, Cordelia Langford, John Sillitoe on behalf of the Wellcome Sanger Institute COVID-19 Surveillance Team
EPI_ISL_718769	Lighthouse Lab in Alderley Park	Wellcome Sanger Institute for the COVID-19 Genomics UK (COG-UK) Consortium	Jacquelyn Wynn, Mairead Hyland, The Lighthouse Lab in Alderley Park and Alex Alderton, Roberto Amato, Sonia Goncalves, Ewan Harrison, David K. Jackson, Ian Johnston, Dominic Kwiatkowski, Cordelia Langford, John Sillitoe on behalf of the Wellcome Sanger Institute COVID-19 Surveillance Team
EPI_ISL_718771	Lighthouse Lab in Cambridge	Wellcome Sanger Institute for the COVID-19 Genomics UK (COG-UK) Consortium	Rob Howes, The Lighthouse Lab in Cambridge and Alex Alderton, Roberto Amato, Sonia Goncalves, Ewan Harrison, David K. Jackson, Ian Johnston, Dominic Kwiatkowski, Cordelia Langford, John Sillitoe on behalf of the Wellcome Sanger Institute COVID-19 Surveillance Team
EPI_ISL_718772	Lighthouse Lab in Alderley Park	Wellcome Sanger Institute for the COVID-19 Genomics UK (COG-UK) Consortium	Jacquelyn Wynn, Mairead Hyland, The Lighthouse Lab in Alderley Park and Alex Alderton, Roberto Amato, Sonia Goncalves, Ewan Harrison, David K. Jackson, Ian Johnston, Dominic Kwiatkowski, Cordelia Langford, John Sillitoe on behalf of the Wellcome Sanger Institute COVID-19 Surveillance Team
EPI_ISL_718775, EPI_ISL_718776	Lighthouse Lab in Cambridge	Wellcome Sanger Institute for the COVID-19 Genomics UK (COG-UK) Consortium	Rob Howes, The Lighthouse Lab in Cambridge and Alex Alderton, Roberto Amato, Sonia Goncalves, Ewan Harrison, David K. Jackson, Ian Johnston, Dominic Kwiatkowski, Cordelia Langford, John Sillitoe on behalf of the Wellcome Sanger Institute COVID-19 Surveillance Team
EPI_ISL_718777	Lighthouse Lab in Alderley Park	Wellcome Sanger Institute for the COVID-19 Genomics UK (COG-UK) Consortium	Jacquelyn Wynn, Mairead Hyland, The Lighthouse Lab in Alderley Park and Alex Alderton, Roberto Amato, Sonia Goncalves, Ewan Harrison, David K. Jackson, Ian Johnston, Dominic Kwiatkowski, Cordelia Langford, John Sillitoe on behalf of the Wellcome Sanger Institute COVID-19 Surveillance Team
EPI_ISL_718778	Lighthouse Lab in Cambridge	Wellcome Sanger Institute for the COVID-19 Genomics UK (COG-UK) Consortium	Rob Howes, The Lighthouse Lab in Cambridge and Alex Alderton, Roberto Amato, Sonia Goncalves, Ewan Harrison, David K. Jackson, Ian Johnston, Dominic Kwiatkowski, Cordelia Langford, John Sillitoe on behalf of the Wellcome Sanger Institute COVID-19 Surveillance Team
EPI_ISL_718779, EPI_ISL_718780, EPI_ISL_718781, EPI_ISL_718782, EPI_ISL_718783	Lighthouse Lab in Alderley Park	Wellcome Sanger Institute for the COVID-19 Genomics UK (COG-UK) Consortium	Jacquelyn Wynn, Mairead Hyland, The Lighthouse Lab in Alderley Park and Alex Alderton, Roberto Amato, Sonia Goncalves, Ewan Harrison, David K. Jackson, Ian Johnston, Dominic Kwiatkowski, Cordelia Langford, John Sillitoe on behalf of the Wellcome Sanger Institute COVID-19 Surveillance Team
EPI_ISL_718785, EPI_ISL_718788	Lighthouse Lab in Cambridge	Wellcome Sanger Institute for the COVID-19 Genomics UK (COG-UK) Consortium	Rob Howes, The Lighthouse Lab in Cambridge and Alex Alderton, Roberto Amato, Sonia Goncalves, Ewan Harrison, David K. Jackson, Ian Johnston, Dominic Kwiatkowski, Cordelia Langford, John Sillitoe on behalf of the Wellcome Sanger Institute COVID-19 Surveillance Team
EPI_ISL_718789	Lighthouse Lab in Alderley Park	Wellcome Sanger Institute for the COVID-19 Genomics UK (COG-UK) Consortium	Jacquelyn Wynn, Mairead Hyland, The Lighthouse Lab in Alderley Park and Alex Alderton, Roberto Amato, Sonia Goncalves, Ewan Harrison, David K. Jackson, Ian Johnston, Dominic Kwiatkowski, Cordelia Langford, John Sillitoe on behalf of the Wellcome Sanger Institute COVID-19 Surveillance Team
EPI_ISL_718790	Lighthouse Lab in Cambridge	Wellcome Sanger Institute for the COVID-19 Genomics UK (COG-UK) Consortium	Rob Howes, The Lighthouse Lab in Cambridge and Alex Alderton, Roberto Amato, Sonia Goncalves, Ewan Harrison, David K. Jackson, Ian Johnston, Dominic Kwiatkowski, Cordelia Langford, John Sillitoe on behalf of the Wellcome Sanger Institute COVID-19 Surveillance Team
EPI_ISL_718791	Lighthouse Lab in Alderley Park	Wellcome Sanger Institute for the COVID-19 Genomics UK (COG-UK) Consortium	Jacquelyn Wynn, Mairead Hyland, The Lighthouse Lab in Alderley Park and Alex Alderton, Roberto Amato, Sonia Goncalves, Ewan Harrison, David K. Jackson, Ian Johnston, Dominic Kwiatkowski, Cordelia Langford, John Sillitoe on behalf of the Wellcome Sanger Institute COVID-19 Surveillance Team
EPI_ISL_718792, EPI_ISL_718793, EPI_ISL_718794	Lighthouse Lab in Cambridge	Wellcome Sanger Institute for the COVID-19 Genomics UK (COG-UK) Consortium	Rob Howes, The Lighthouse Lab in Cambridge and Alex Alderton, Roberto Amato, Sonia Goncalves, Ewan Harrison, David K. Jackson, Ian Johnston, Dominic Kwiatkowski, Cordelia Langford, John Sillitoe on behalf of the Wellcome Sanger Institute COVID-19 Surveillance Team

EPI_ISL_718795	Lighthouse Lab in Alderley Park	Wellcome Sanger Institute for the COVID-19 Genomics UK (COG-UK) Consortium	Jacquelyn Wynn, Mairead Hyland, The Lighthouse Lab in Alderley Park and Alex Alderton, Roberto Amato, Sonia Goncalves, Ewan Harrison, David K. Jackson, Ian Johnston, Dominic Kwiatkowski, Cordelia Langford, John Sillitoe on behalf of the Wellcome Sanger Institute COVID-19 Surveillance Team
EPI_ISL_718797	Lighthouse Lab in Cambridge	Wellcome Sanger Institute for the COVID-19 Genomics UK (COG-UK) Consortium	Rob Howes, The Lighthouse Lab in Cambridge and Alex Alderton, Roberto Amato, Sonia Goncalves, Ewan Harrison, David K. Jackson, Ian Johnston, Dominic Kwiatkowski, Cordelia Langford, John Sillitoe on behalf of the Wellcome Sanger Institute COVID-19 Surveillance Team
EPI_ISL_718799	Lighthouse Lab in Alderley Park	Wellcome Sanger Institute for the COVID-19 Genomics UK (COG-UK) Consortium	Jacquelyn Wynn, Mairead Hyland, The Lighthouse Lab in Alderley Park and Alex Alderton, Roberto Amato, Sonia Goncalves, Ewan Harrison, David K. Jackson, Ian Johnston, Dominic Kwiatkowski, Cordelia Langford, John Sillitoe on behalf of the Wellcome Sanger Institute COVID-19 Surveillance Team
EPI_ISL_718800	Lighthouse Lab in Cambridge	Wellcome Sanger Institute for the COVID-19 Genomics UK (COG-UK) Consortium	Rob Howes, The Lighthouse Lab in Cambridge and Alex Alderton, Roberto Amato, Sonia Goncalves, Ewan Harrison, David K. Jackson, Ian Johnston, Dominic Kwiatkowski, Cordelia Langford, John Sillitoe on behalf of the Wellcome Sanger Institute COVID-19 Surveillance Team
EPI_ISL_718801, EPI_ISL_718802	Lighthouse Lab in Alderley Park	Wellcome Sanger Institute for the COVID-19 Genomics UK (COG-UK) Consortium	Jacquelyn Wynn, Mairead Hyland, The Lighthouse Lab in Alderley Park and Alex Alderton, Roberto Amato, Sonia Goncalves, Ewan Harrison, David K. Jackson, Ian Johnston, Dominic Kwiatkowski, Cordelia Langford, John Sillitoe on behalf of the Wellcome Sanger Institute COVID-19 Surveillance Team
EPI_ISL_718804, EPI_ISL_718805	Lighthouse Lab in Cambridge	Wellcome Sanger Institute for the COVID-19 Genomics UK (COG-UK) Consortium	Rob Howes, The Lighthouse Lab in Cambridge and Alex Alderton, Roberto Amato, Sonia Goncalves, Ewan Harrison, David K. Jackson, Ian Johnston, Dominic Kwiatkowski, Cordelia Langford, John Sillitoe on behalf of the Wellcome Sanger Institute COVID-19 Surveillance Team
EPI_ISL_718807, EPI_ISL_718808	Lighthouse Lab in Alderley Park	Wellcome Sanger Institute for the COVID-19 Genomics UK (COG-UK) Consortium	Jacquelyn Wynn, Mairead Hyland, The Lighthouse Lab in Alderley Park and Alex Alderton, Roberto Amato, Sonia Goncalves, Ewan Harrison, David K. Jackson, Ian Johnston, Dominic Kwiatkowski, Cordelia Langford, John Sillitoe on behalf of the Wellcome Sanger Institute COVID-19 Surveillance Team
EPI_ISL_718809	Lighthouse Lab in Cambridge	Wellcome Sanger Institute for the COVID-19 Genomics UK (COG-UK) Consortium	Rob Howes, The Lighthouse Lab in Cambridge and Alex Alderton, Roberto Amato, Sonia Goncalves, Ewan Harrison, David K. Jackson, Ian Johnston, Dominic Kwiatkowski, Cordelia Langford, John Sillitoe on behalf of the Wellcome Sanger Institute COVID-19 Surveillance Team
EPI_ISL_718811, EPI_ISL_718812, EPI_ISL_718813	Lighthouse Lab in Alderley Park	Wellcome Sanger Institute for the COVID-19 Genomics UK (COG-UK) Consortium	Jacquelyn Wynn, Mairead Hyland, The Lighthouse Lab in Alderley Park and Alex Alderton, Roberto Amato, Sonia Goncalves, Ewan Harrison, David K. Jackson, Ian Johnston, Dominic Kwiatkowski, Cordelia Langford, John Sillitoe on behalf of the Wellcome Sanger Institute COVID-19 Surveillance Team
EPI_ISL_718814, EPI_ISL_718816, EPI_ISL_718817	Lighthouse Lab in Cambridge	Wellcome Sanger Institute for the COVID-19 Genomics UK (COG-UK) Consortium	Rob Howes, The Lighthouse Lab in Cambridge and Alex Alderton, Roberto Amato, Sonia Goncalves, Ewan Harrison, David K. Jackson, Ian Johnston, Dominic Kwiatkowski, Cordelia Langford, John Sillitoe on behalf of the Wellcome Sanger Institute COVID-19 Surveillance Team
EPI_ISL_718819	Lighthouse Lab in Alderley Park	Wellcome Sanger Institute for the COVID-19 Genomics UK (COG-UK) Consortium	Jacquelyn Wynn, Mairead Hyland, The Lighthouse Lab in Alderley Park and Alex Alderton, Roberto Amato, Sonia Goncalves, Ewan Harrison, David K. Jackson, Ian Johnston, Dominic Kwiatkowski, Cordelia Langford, John Sillitoe on behalf of the Wellcome Sanger Institute COVID-19 Surveillance Team
EPI_ISL_718822	Lighthouse Lab in Cambridge	Wellcome Sanger Institute for the COVID-19 Genomics UK (COG-UK) Consortium	Rob Howes, The Lighthouse Lab in Cambridge and Alex Alderton, Roberto Amato, Sonia Goncalves, Ewan Harrison, David K. Jackson, Ian Johnston, Dominic Kwiatkowski, Cordelia Langford, John Sillitoe on behalf of the Wellcome Sanger Institute COVID-19 Surveillance Team
EPI_ISL_718823	Lighthouse Lab in Alderley Park	Wellcome Sanger Institute for the COVID-19 Genomics UK (COG-UK) Consortium	Jacquelyn Wynn, Mairead Hyland, The Lighthouse Lab in Alderley Park and Alex Alderton, Roberto Amato, Sonia Goncalves, Ewan Harrison, David K. Jackson, Ian Johnston, Dominic Kwiatkowski, Cordelia Langford, John Sillitoe on behalf of the Wellcome Sanger Institute COVID-19 Surveillance Team
EPI_ISL_718824, EPI_ISL_718825, EPI_ISL_718826	Lighthouse Lab in Cambridge	Wellcome Sanger Institute for the COVID-19 Genomics UK (COG-UK) Consortium	Rob Howes, The Lighthouse Lab in Cambridge and Alex Alderton, Roberto Amato, Sonia Goncalves, Ewan Harrison, David K. Jackson, Ian Johnston, Dominic Kwiatkowski, Cordelia Langford, John Sillitoe on behalf of the Wellcome Sanger Institute COVID-19 Surveillance Team
EPI_ISL_718827, EPI_ISL_718828, EPI_ISL_718829, EPI_ISL_718830, EPI_ISL_718831, EPI_ISL_718832, EPI_ISL_718833	Lighthouse Lab in Alderley Park	Wellcome Sanger Institute for the COVID-19 Genomics UK (COG-UK) Consortium	Jacquelyn Wynn, Mairead Hyland, The Lighthouse Lab in Alderley Park and Alex Alderton, Roberto Amato, Sonia Goncalves, Ewan Harrison, David K. Jackson, Ian Johnston, Dominic Kwiatkowski, Cordelia Langford, John Sillitoe on behalf of the Wellcome Sanger Institute COVID-19 Surveillance Team
EPI_ISL_718834, EPI_ISL_718835, EPI_ISL_718836	Lighthouse Lab in Cambridge	Wellcome Sanger Institute for the COVID-19 Genomics UK (COG-UK) Consortium	Rob Howes, The Lighthouse Lab in Cambridge and Alex Alderton, Roberto Amato, Sonia Goncalves, Ewan Harrison, David K. Jackson, Ian Johnston, Dominic Kwiatkowski, Cordelia Langford, John Sillitoe on behalf of the Wellcome Sanger Institute COVID-19 Surveillance Team
EPI_ISL_718837, EPI_ISL_718839, EPI_ISL_718841	Lighthouse Lab in Alderley Park	Wellcome Sanger Institute for the COVID-19 Genomics UK (COG-UK) Consortium	Jacquelyn Wynn, Mairead Hyland, The Lighthouse Lab in Alderley Park and Alex Alderton, Roberto Amato, Sonia Goncalves, Ewan Harrison, David K. Jackson, Ian Johnston, Dominic Kwiatkowski, Cordelia Langford, John Sillitoe on behalf of the Wellcome Sanger Institute COVID-19 Surveillance Team
EPI_ISL_718842, EPI_ISL_718843	Lighthouse Lab in Cambridge	Wellcome Sanger Institute for the COVID-19 Genomics UK (COG-UK) Consortium	Rob Howes, The Lighthouse Lab in Cambridge and Alex Alderton, Roberto Amato, Sonia Goncalves, Ewan Harrison, David K. Jackson, Ian Johnston, Dominic Kwiatkowski, Cordelia Langford, John Sillitoe on behalf of the Wellcome Sanger Institute COVID-19 Surveillance Team
EPI_ISL_718844	Lighthouse Lab in Alderley Park	Wellcome Sanger Institute for the COVID-19 Genomics UK (COG-UK) Consortium	Jacquelyn Wynn, Mairead Hyland, The Lighthouse Lab in Alderley Park and Alex Alderton, Roberto Amato, Sonia Goncalves, Ewan Harrison, David K. Jackson, Ian Johnston, Dominic Kwiatkowski, Cordelia Langford, John Sillitoe on behalf of the Wellcome Sanger Institute COVID-19 Surveillance Team
EPI_ISL_718845	Lighthouse Lab in Cambridge	Wellcome Sanger Institute for the COVID-19 Genomics UK (COG-UK) Consortium	Rob Howes, The Lighthouse Lab in Cambridge and Alex Alderton, Roberto Amato, Sonia Goncalves, Ewan Harrison, David K. Jackson, Ian Johnston, Dominic Kwiatkowski, Cordelia Langford, John Sillitoe on behalf of the Wellcome Sanger Institute COVID-19 Surveillance Team
EPI_ISL_718847	Lighthouse Lab in Alderley Park	Wellcome Sanger Institute for the COVID-19 Genomics UK (COG-UK) Consortium	Jacquelyn Wynn, Mairead Hyland, The Lighthouse Lab in Alderley Park and Alex Alderton, Roberto Amato, Sonia Goncalves, Ewan Harrison, David K. Jackson, Ian Johnston, Dominic Kwiatkowski, Cordelia Langford, John Sillitoe on behalf of the Wellcome Sanger Institute COVID-19 Surveillance Team
EPI_ISL_718848, EPI_ISL_718851, EPI_ISL_718852	Lighthouse Lab in Cambridge	Wellcome Sanger Institute for the COVID-19 Genomics UK (COG-UK) Consortium	Rob Howes, The Lighthouse Lab in Cambridge and Alex Alderton, Roberto Amato, Sonia Goncalves, Ewan Harrison, David K. Jackson, Ian Johnston, Dominic Kwiatkowski, Cordelia Langford, John Sillitoe on behalf of the Wellcome Sanger Institute COVID-19 Surveillance Team
EPI_ISL_718853, EPI_ISL_718854	Lighthouse Lab in Alderley Park	Wellcome Sanger Institute for the COVID-19 Genomics UK (COG-UK) Consortium	Jacquelyn Wynn, Mairead Hyland, The Lighthouse Lab in Alderley Park and Alex Alderton, Roberto Amato, Sonia Goncalves, Ewan Harrison, David K. Jackson, Ian Johnston, Dominic Kwiatkowski, Cordelia Langford, John Sillitoe on behalf of the Wellcome Sanger Institute COVID-19 Surveillance Team
EPI_ISL_718855, EPI_ISL_718856, EPI_ISL_718857	Lighthouse Lab in Cambridge	Wellcome Sanger Institute for the COVID-19 Genomics UK (COG-UK) Consortium	Rob Howes, The Lighthouse Lab in Cambridge and Alex Alderton, Roberto Amato, Sonia Goncalves, Ewan Harrison, David K. Jackson, Ian Johnston, Dominic Kwiatkowski, Cordelia Langford, John Sillitoe on behalf of the Wellcome Sanger Institute COVID-19 Surveillance Team
EPI_ISL_718858, EPI_ISL_718859	Lighthouse Lab in Alderley Park	Wellcome Sanger Institute for the COVID-19 Genomics UK (COG-UK) Consortium	Jacquelyn Wynn, Mairead Hyland, The Lighthouse Lab in Alderley Park and Alex Alderton, Roberto Amato, Sonia Goncalves, Ewan Harrison, David K. Jackson, Ian Johnston, Dominic Kwiatkowski, Cordelia Langford, John Sillitoe on behalf of the Wellcome Sanger Institute COVID-19 Surveillance Team
EPI_ISL_718860, EPI_ISL_718861, EPI_ISL_718863	Lighthouse Lab in Cambridge	Wellcome Sanger Institute for the COVID-19 Genomics UK (COG-UK) Consortium	Rob Howes, The Lighthouse Lab in Cambridge and Alex Alderton, Roberto Amato, Sonia Goncalves, Ewan Harrison, David K. Jackson, Ian Johnston, Dominic Kwiatkowski, Cordelia Langford, John Sillitoe on behalf of the Wellcome Sanger Institute COVID-19 Surveillance Team
EPI_ISL_718864, EPI_ISL_718865	Lighthouse Lab in Alderley Park	Wellcome Sanger Institute for the COVID-19 Genomics UK (COG-UK) Consortium	Jacquelyn Wynn, Mairead Hyland, The Lighthouse Lab in Alderley Park and Alex Alderton, Roberto Amato, Sonia Goncalves, Ewan Harrison, David K. Jackson, Ian Johnston, Dominic Kwiatkowski, Cordelia Langford, John Sillitoe on behalf of the Wellcome Sanger Institute COVID-19 Surveillance Team
EPI_ISL_718867, EPI_ISL_718868, EPI_ISL_718869	Lighthouse Lab in Cambridge	Wellcome Sanger Institute for the COVID-19 Genomics UK (COG-UK) Consortium	Rob Howes, The Lighthouse Lab in Cambridge and Alex Alderton, Roberto Amato, Sonia Goncalves, Ewan Harrison, David K. Jackson, Ian Johnston, Dominic Kwiatkowski, Cordelia Langford, John Sillitoe on behalf of the Wellcome Sanger Institute COVID-19 Surveillance Team
EPI_ISL_718870, EPI_ISL_718871, EPI_ISL_718872, EPI_ISL_718873	Lighthouse Lab in Alderley Park	Wellcome Sanger Institute for the COVID-19 Genomics UK (COG-UK) Consortium	Jacquelyn Wynn, Mairead Hyland, The Lighthouse Lab in Alderley Park and Alex Alderton, Roberto Amato, Sonia Goncalves, Ewan Harrison, David K. Jackson, Ian Johnston, Dominic Kwiatkowski, Cordelia Langford, John Sillitoe on behalf of the Wellcome Sanger Institute COVID-19 Surveillance Team
EPI_ISL_718879	Lighthouse Lab in Cambridge	Wellcome Sanger Institute for the COVID-19 Genomics UK (COG-UK) Consortium	Rob Howes, The Lighthouse Lab in Cambridge and Alex Alderton, Roberto Amato, Sonia Goncalves, Ewan Harrison, David K. Jackson, Ian Johnston, Dominic Kwiatkowski, Cordelia Langford, John Sillitoe on behalf of the Wellcome Sanger Institute COVID-19 Surveillance Team
EPI_ISL_718880, EPI_ISL_718881	Lighthouse Lab in Alderley Park	Wellcome Sanger Institute for the COVID-19 Genomics UK (COG-UK) Consortium	Jacquelyn Wynn, Mairead Hyland, The Lighthouse Lab in Alderley Park and Alex Alderton, Roberto Amato, Sonia Goncalves, Ewan Harrison, David K. Jackson, Ian Johnston, Dominic Kwiatkowski, Cordelia Langford, John Sillitoe on behalf of the Wellcome Sanger Institute COVID-19 Surveillance Team
EPI_ISL_718882	Lighthouse Lab in Cambridge	Wellcome Sanger Institute for the COVID-19 Genomics UK (COG-UK) Consortium	Rob Howes, The Lighthouse Lab in Cambridge and Alex Alderton, Roberto Amato, Sonia Goncalves, Ewan Harrison, David K. Jackson, Ian Johnston, Dominic Kwiatkowski, Cordelia Langford, John Sillitoe on behalf of the Wellcome Sanger Institute COVID-19 Surveillance Team
EPI_ISL_718885, EPI_ISL_718886, EPI_ISL_718888, EPI_ISL_718890, EPI_ISL_718891	Lighthouse Lab in Alderley Park	Wellcome Sanger Institute for the COVID-19 Genomics UK (COG-UK) Consortium	Jacquelyn Wynn, Mairead Hyland, The Lighthouse Lab in Alderley Park and Alex Alderton, Roberto Amato, Sonia Goncalves, Ewan Harrison, David K. Jackson, Ian Johnston, Dominic Kwiatkowski, Cordelia Langford, John Sillitoe on behalf of the Wellcome Sanger Institute COVID-19 Surveillance Team
EPI_ISL_718892, EPI_ISL_718893, EPI_ISL_718898, EPI_ISL_718900	Lighthouse Lab in Cambridge	Wellcome Sanger Institute for the COVID-19 Genomics UK (COG-UK) Consortium	Rob Howes, The Lighthouse Lab in Cambridge and Alex Alderton, Roberto Amato, Sonia Goncalves, Ewan Harrison, David K. Jackson, Ian Johnston, Dominic Kwiatkowski, Cordelia Langford, John Sillitoe on behalf of the Wellcome Sanger Institute COVID-19 Surveillance Team

EPI_ISL_718902, EPI_ISL_718903	Lighthouse Lab in Alderley Park	Wellcome Sanger Institute for the COVID-19 Genomics UK (COG-UK) Consortium	Jacquelyn Wynn, Mairead Hyland, The Lighthouse Lab in Alderley Park and Alex Alderton, Roberto Amato, Sonia Goncalves, Ewan Harrison, David K. Jackson, Ian Johnston, Dominic Kwiatkowski, Cordelia Langford, John Sillitoe on behalf of the Wellcome Sanger Institute COVID-19 Surveillance Team
EPI_ISL_718904, EPI_ISL_718905, EPI_ISL_718906	Lighthouse Lab in Cambridge	Wellcome Sanger Institute for the COVID-19 Genomics UK (COG-UK) Consortium	Rob Howes, The Lighthouse Lab in Cambridge and Alex Alderton, Roberto Amato, Sonia Goncalves, Ewan Harrison, David K. Jackson, Ian Johnston, Dominic Kwiatkowski, Cordelia Langford, John Sillitoe on behalf of the Wellcome Sanger Institute COVID-19 Surveillance Team
EPI_ISL_718907, EPI_ISL_718908	Lighthouse Lab in Alderley Park	Wellcome Sanger Institute for the COVID-19 Genomics UK (COG-UK) Consortium	Jacquelyn Wynn, Mairead Hyland, The Lighthouse Lab in Alderley Park and Alex Alderton, Roberto Amato, Sonia Goncalves, Ewan Harrison, David K. Jackson, Ian Johnston, Dominic Kwiatkowski, Cordelia Langford, John Sillitoe on behalf of the Wellcome Sanger Institute COVID-19 Surveillance Team
EPI_ISL_718909	Lighthouse Lab in Cambridge	Wellcome Sanger Institute for the COVID-19 Genomics UK (COG-UK) Consortium	Rob Howes, The Lighthouse Lab in Cambridge and Alex Alderton, Roberto Amato, Sonia Goncalves, Ewan Harrison, David K. Jackson, Ian Johnston, Dominic Kwiatkowski, Cordelia Langford, John Sillitoe on behalf of the Wellcome Sanger Institute COVID-19 Surveillance Team
EPI_ISL_718911	Lighthouse Lab in Alderley Park	Wellcome Sanger Institute for the COVID-19 Genomics UK (COG-UK) Consortium	Jacquelyn Wynn, Mairead Hyland, The Lighthouse Lab in Alderley Park and Alex Alderton, Roberto Amato, Sonia Goncalves, Ewan Harrison, David K. Jackson, Ian Johnston, Dominic Kwiatkowski, Cordelia Langford, John Sillitoe on behalf of the Wellcome Sanger Institute COVID-19 Surveillance Team
EPI_ISL_718912, EPI_ISL_718913	Lighthouse Lab in Cambridge	Wellcome Sanger Institute for the COVID-19 Genomics UK (COG-UK) Consortium	Rob Howes, The Lighthouse Lab in Cambridge and Alex Alderton, Roberto Amato, Sonia Goncalves, Ewan Harrison, David K. Jackson, Ian Johnston, Dominic Kwiatkowski, Cordelia Langford, John Sillitoe on behalf of the Wellcome Sanger Institute COVID-19 Surveillance Team
EPI_ISL_718914, EPI_ISL_718915	Lighthouse Lab in Alderley Park	Wellcome Sanger Institute for the COVID-19 Genomics UK (COG-UK) Consortium	Jacquelyn Wynn, Mairead Hyland, The Lighthouse Lab in Alderley Park and Alex Alderton, Roberto Amato, Sonia Goncalves, Ewan Harrison, David K. Jackson, Ian Johnston, Dominic Kwiatkowski, Cordelia Langford, John Sillitoe on behalf of the Wellcome Sanger Institute COVID-19 Surveillance Team
EPI_ISL_718916	Lighthouse Lab in Cambridge	Wellcome Sanger Institute for the COVID-19 Genomics UK (COG-UK) Consortium	Rob Howes, The Lighthouse Lab in Cambridge and Alex Alderton, Roberto Amato, Sonia Goncalves, Ewan Harrison, David K. Jackson, Ian Johnston, Dominic Kwiatkowski, Cordelia Langford, John Sillitoe on behalf of the Wellcome Sanger Institute COVID-19 Surveillance Team
EPI_ISL_718917	Lighthouse Lab in Alderley Park	Wellcome Sanger Institute for the COVID-19 Genomics UK (COG-UK) Consortium	Jacquelyn Wynn, Mairead Hyland, The Lighthouse Lab in Alderley Park and Alex Alderton, Roberto Amato, Sonia Goncalves, Ewan Harrison, David K. Jackson, Ian Johnston, Dominic Kwiatkowski, Cordelia Langford, John Sillitoe on behalf of the Wellcome Sanger Institute COVID-19 Surveillance Team
EPI_ISL_718921	Lighthouse Lab in Cambridge	Wellcome Sanger Institute for the COVID-19 Genomics UK (COG-UK) Consortium	Rob Howes, The Lighthouse Lab in Cambridge and Alex Alderton, Roberto Amato, Sonia Goncalves, Ewan Harrison, David K. Jackson, Ian Johnston, Dominic Kwiatkowski, Cordelia Langford, John Sillitoe on behalf of the Wellcome Sanger Institute COVID-19 Surveillance Team
EPI_ISL_718922, EPI_ISL_718923	Lighthouse Lab in Alderley Park	Wellcome Sanger Institute for the COVID-19 Genomics UK (COG-UK) Consortium	Jacquelyn Wynn, Mairead Hyland, The Lighthouse Lab in Alderley Park and Alex Alderton, Roberto Amato, Sonia Goncalves, Ewan Harrison, David K. Jackson, Ian Johnston, Dominic Kwiatkowski, Cordelia Langford, John Sillitoe on behalf of the Wellcome Sanger Institute COVID-19 Surveillance Team
EPI_ISL_718924, EPI_ISL_718925	Lighthouse Lab in Cambridge	Wellcome Sanger Institute for the COVID-19 Genomics UK (COG-UK) Consortium	Rob Howes, The Lighthouse Lab in Cambridge and Alex Alderton, Roberto Amato, Sonia Goncalves, Ewan Harrison, David K. Jackson, Ian Johnston, Dominic Kwiatkowski, Cordelia Langford, John Sillitoe on behalf of the Wellcome Sanger Institute COVID-19 Surveillance Team
EPI_ISL_718926	Lighthouse Lab in Alderley Park	Wellcome Sanger Institute for the COVID-19 Genomics UK (COG-UK) Consortium	Jacquelyn Wynn, Mairead Hyland, The Lighthouse Lab in Alderley Park and Alex Alderton, Roberto Amato, Sonia Goncalves, Ewan Harrison, David K. Jackson, Ian Johnston, Dominic Kwiatkowski, Cordelia Langford, John Sillitoe on behalf of the Wellcome Sanger Institute COVID-19 Surveillance Team
EPI_ISL_718927, EPI_ISL_718928	Lighthouse Lab in Cambridge	Wellcome Sanger Institute for the COVID-19 Genomics UK (COG-UK) Consortium	Rob Howes, The Lighthouse Lab in Cambridge and Alex Alderton, Roberto Amato, Sonia Goncalves, Ewan Harrison, David K. Jackson, Ian Johnston, Dominic Kwiatkowski, Cordelia Langford, John Sillitoe on behalf of the Wellcome Sanger Institute COVID-19 Surveillance Team
EPI_ISL_718929, EPI_ISL_718930	Lighthouse Lab in Alderley Park	Wellcome Sanger Institute for the COVID-19 Genomics UK (COG-UK) Consortium	Jacquelyn Wynn, Mairead Hyland, The Lighthouse Lab in Alderley Park and Alex Alderton, Roberto Amato, Sonia Goncalves, Ewan Harrison, David K. Jackson, Ian Johnston, Dominic Kwiatkowski, Cordelia Langford, John Sillitoe on behalf of the Wellcome Sanger Institute COVID-19 Surveillance Team
EPI_ISL_718931	Lighthouse Lab in Cambridge	Wellcome Sanger Institute for the COVID-19 Genomics UK (COG-UK) Consortium	Rob Howes, The Lighthouse Lab in Cambridge and Alex Alderton, Roberto Amato, Sonia Goncalves, Ewan Harrison, David K. Jackson, Ian Johnston, Dominic Kwiatkowski, Cordelia Langford, John Sillitoe on behalf of the Wellcome Sanger Institute COVID-19 Surveillance Team
EPI_ISL_718932	Lighthouse Lab in Alderley Park	Wellcome Sanger Institute for the COVID-19 Genomics UK (COG-UK) Consortium	Jacquelyn Wynn, Mairead Hyland, The Lighthouse Lab in Alderley Park and Alex Alderton, Roberto Amato, Sonia Goncalves, Ewan Harrison, David K. Jackson, Ian Johnston, Dominic Kwiatkowski, Cordelia Langford, John Sillitoe on behalf of the Wellcome Sanger Institute COVID-19 Surveillance Team
EPI_ISL_718933, EPI_ISL_718934	Lighthouse Lab in Cambridge	Wellcome Sanger Institute for the COVID-19 Genomics UK (COG-UK) Consortium	Rob Howes, The Lighthouse Lab in Cambridge and Alex Alderton, Roberto Amato, Sonia Goncalves, Ewan Harrison, David K. Jackson, Ian Johnston, Dominic Kwiatkowski, Cordelia Langford, John Sillitoe on behalf of the Wellcome Sanger Institute COVID-19 Surveillance Team
EPI_ISL_718935	Lighthouse Lab in Alderley Park	Wellcome Sanger Institute for the COVID-19 Genomics UK (COG-UK) Consortium	Jacquelyn Wynn, Mairead Hyland, The Lighthouse Lab in Alderley Park and Alex Alderton, Roberto Amato, Sonia Goncalves, Ewan Harrison, David K. Jackson, Ian Johnston, Dominic Kwiatkowski, Cordelia Langford, John Sillitoe on behalf of the Wellcome Sanger Institute COVID-19 Surveillance Team
EPI_ISL_718937, EPI_ISL_718938, EPI_ISL_718941, EPI_ISL_718942	Lighthouse Lab in Cambridge	Wellcome Sanger Institute for the COVID-19 Genomics UK (COG-UK) Consortium	Rob Howes, The Lighthouse Lab in Cambridge and Alex Alderton, Roberto Amato, Sonia Goncalves, Ewan Harrison, David K. Jackson, Ian Johnston, Dominic Kwiatkowski, Cordelia Langford, John Sillitoe on behalf of the Wellcome Sanger Institute COVID-19 Surveillance Team
EPI_ISL_718943	Lighthouse Lab in Alderley Park	Wellcome Sanger Institute for the COVID-19 Genomics UK (COG-UK) Consortium	Jacquelyn Wynn, Mairead Hyland, The Lighthouse Lab in Alderley Park and Alex Alderton, Roberto Amato, Sonia Goncalves, Ewan Harrison, David K. Jackson, Ian Johnston, Dominic Kwiatkowski, Cordelia Langford, John Sillitoe on behalf of the Wellcome Sanger Institute COVID-19 Surveillance Team
EPI_ISL_718944	Lighthouse Lab in Cambridge	Wellcome Sanger Institute for the COVID-19 Genomics UK (COG-UK) Consortium	Rob Howes, The Lighthouse Lab in Cambridge and Alex Alderton, Roberto Amato, Sonia Goncalves, Ewan Harrison, David K. Jackson, Ian Johnston, Dominic Kwiatkowski, Cordelia Langford, John Sillitoe on behalf of the Wellcome Sanger Institute COVID-19 Surveillance Team
EPI_ISL_718945	Lighthouse Lab in Alderley Park	Wellcome Sanger Institute for the COVID-19 Genomics UK (COG-UK) Consortium	Jacquelyn Wynn, Mairead Hyland, The Lighthouse Lab in Alderley Park and Alex Alderton, Roberto Amato, Sonia Goncalves, Ewan Harrison, David K. Jackson, Ian Johnston, Dominic Kwiatkowski, Cordelia Langford, John Sillitoe on behalf of the Wellcome Sanger Institute COVID-19 Surveillance Team
EPI_ISL_718946, EPI_ISL_718949, EPI_ISL_718951	Lighthouse Lab in Cambridge	Wellcome Sanger Institute for the COVID-19 Genomics UK (COG-UK) Consortium	Rob Howes, The Lighthouse Lab in Cambridge and Alex Alderton, Roberto Amato, Sonia Goncalves, Ewan Harrison, David K. Jackson, Ian Johnston, Dominic Kwiatkowski, Cordelia Langford, John Sillitoe on behalf of the Wellcome Sanger Institute COVID-19 Surveillance Team
EPI_ISL_718952, EPI_ISL_718953	Lighthouse Lab in Alderley Park	Wellcome Sanger Institute for the COVID-19 Genomics UK (COG-UK) Consortium	Jacquelyn Wynn, Mairead Hyland, The Lighthouse Lab in Alderley Park and Alex Alderton, Roberto Amato, Sonia Goncalves, Ewan Harrison, David K. Jackson, Ian Johnston, Dominic Kwiatkowski, Cordelia Langford, John Sillitoe on behalf of the Wellcome Sanger Institute COVID-19 Surveillance Team
EPI_ISL_718954	Lighthouse Lab in Cambridge	Wellcome Sanger Institute for the COVID-19 Genomics UK (COG-UK) Consortium	Rob Howes, The Lighthouse Lab in Cambridge and Alex Alderton, Roberto Amato, Sonia Goncalves, Ewan Harrison, David K. Jackson, Ian Johnston, Dominic Kwiatkowski, Cordelia Langford, John Sillitoe on behalf of the Wellcome Sanger Institute COVID-19 Surveillance Team
EPI_ISL_718955	Lighthouse Lab in Alderley Park	Wellcome Sanger Institute for the COVID-19 Genomics UK (COG-UK) Consortium	Jacquelyn Wynn, Mairead Hyland, The Lighthouse Lab in Alderley Park and Alex Alderton, Roberto Amato, Sonia Goncalves, Ewan Harrison, David K. Jackson, Ian Johnston, Dominic Kwiatkowski, Cordelia Langford, John Sillitoe on behalf of the Wellcome Sanger Institute COVID-19 Surveillance Team
EPI_ISL_718956, EPI_ISL_718958	Lighthouse Lab in Cambridge	Wellcome Sanger Institute for the COVID-19 Genomics UK (COG-UK) Consortium	Rob Howes, The Lighthouse Lab in Cambridge and Alex Alderton, Roberto Amato, Sonia Goncalves, Ewan Harrison, David K. Jackson, Ian Johnston, Dominic Kwiatkowski, Cordelia Langford, John Sillitoe on behalf of the Wellcome Sanger Institute COVID-19 Surveillance Team
EPI_ISL_718959	Lighthouse Lab in Alderley Park	Wellcome Sanger Institute for the COVID-19 Genomics UK (COG-UK) Consortium	Jacquelyn Wynn, Mairead Hyland, The Lighthouse Lab in Alderley Park and Alex Alderton, Roberto Amato, Sonia Goncalves, Ewan Harrison, David K. Jackson, Ian Johnston, Dominic Kwiatkowski, Cordelia Langford, John Sillitoe on behalf of the Wellcome Sanger Institute COVID-19 Surveillance Team
EPI_ISL_718960	Lighthouse Lab in Cambridge	Wellcome Sanger Institute for the COVID-19 Genomics UK (COG-UK) Consortium	Rob Howes, The Lighthouse Lab in Cambridge and Alex Alderton, Roberto Amato, Sonia Goncalves, Ewan Harrison, David K. Jackson, Ian Johnston, Dominic Kwiatkowski, Cordelia Langford, John Sillitoe on behalf of the Wellcome Sanger Institute COVID-19 Surveillance Team
EPI_ISL_718962, EPI_ISL_718963	Lighthouse Lab in Alderley Park	Wellcome Sanger Institute for the COVID-19 Genomics UK (COG-UK) Consortium	Jacquelyn Wynn, Mairead Hyland, The Lighthouse Lab in Alderley Park and Alex Alderton, Roberto Amato, Sonia Goncalves, Ewan Harrison, David K. Jackson, Ian Johnston, Dominic Kwiatkowski, Cordelia Langford, John Sillitoe on behalf of the Wellcome Sanger Institute COVID-19 Surveillance Team
EPI_ISL_718965	Lighthouse Lab in Cambridge	Wellcome Sanger Institute for the COVID-19 Genomics UK (COG-UK) Consortium	Rob Howes, The Lighthouse Lab in Cambridge and Alex Alderton, Roberto Amato, Sonia Goncalves, Ewan Harrison, David K. Jackson, Ian Johnston, Dominic Kwiatkowski, Cordelia Langford, John Sillitoe on behalf of the Wellcome Sanger Institute COVID-19 Surveillance Team
EPI_ISL_718966, EPI_ISL_718967	Lighthouse Lab in Alderley Park	Wellcome Sanger Institute for the COVID-19 Genomics UK (COG-UK) Consortium	Jacquelyn Wynn, Mairead Hyland, The Lighthouse Lab in Alderley Park and Alex Alderton, Roberto Amato, Sonia Goncalves, Ewan Harrison, David K. Jackson, Ian Johnston, Dominic Kwiatkowski, Cordelia Langford, John Sillitoe on behalf of the Wellcome Sanger Institute COVID-19 Surveillance Team
EPI_ISL_718968, EPI_ISL_718969	Lighthouse Lab in Cambridge	Wellcome Sanger Institute for the COVID-19 Genomics UK (COG-UK) Consortium	Rob Howes, The Lighthouse Lab in Cambridge and Alex Alderton, Roberto Amato, Sonia Goncalves, Ewan Harrison, David K. Jackson, Ian Johnston, Dominic Kwiatkowski, Cordelia Langford, John Sillitoe on behalf of the Wellcome Sanger Institute COVID-19 Surveillance Team
EPI_ISL_718970	Lighthouse Lab in Alderley Park	Wellcome Sanger Institute for the COVID-19 Genomics UK (COG-UK) Consortium	Jacquelyn Wynn, Mairead Hyland, The Lighthouse Lab in Alderley Park and Alex Alderton, Roberto Amato, Sonia Goncalves, Ewan Harrison, David K. Jackson, Ian Johnston, Dominic Kwiatkowski, Cordelia Langford, John Sillitoe on behalf of the Wellcome Sanger Institute COVID-19 Surveillance Team

EPI_ISL_718971, EPI_ISL_718972	Lighthouse Lab in Cambridge	Wellcome Sanger Institute for the COVID-19 Genomics UK (COG-UK) Consortium	Rob Howes, The Lighthouse Lab in Cambridge and Alex Alderton, Roberto Amato, Sonia Goncalves, Ewan Harrison, David K. Jackson, Ian Johnston, Dominic Kwiatkowski, Cordelia Langford, John Sillitoe on behalf of the Wellcome Sanger Institute COVID-19 Surveillance Team
EPI_ISL_718974, EPI_ISL_718976, EPI_ISL_718977, EPI_ISL_718978, EPI_ISL_718979	Lighthouse Lab in Alderley Park	Wellcome Sanger Institute for the COVID-19 Genomics UK (COG-UK) Consortium	Jacquelyn Wynn, Mairead Hyland, The Lighthouse Lab in Alderley Park and Alex Alderton, Roberto Amato, Sonia Goncalves, Ewan Harrison, David K. Jackson, Ian Johnston, Dominic Kwiatkowski, Cordelia Langford, John Sillitoe on behalf of the Wellcome Sanger Institute COVID-19 Surveillance Team

EPI_ISL_718981, EPI_ISL_718982, EPI_ISL_718984, EPI_ISL_718985, EPI_ISL_718987, EPI_ISL_718988, EPI_ISL_718989, EPI_ISL_718991, EPI_ISL_718992, EPI_ISL_718995, EPI_ISL_718997, EPI_ISL_718998, EPI_ISL_718999, EPI_ISL_719000, EPI_ISL_719001, EPI_ISL_719002, EPI_ISL_719003, EPI_ISL_719004, EPI_ISL_719005, EPI_ISL_719006, EPI_ISL_719007, EPI_ISL_719009, EPI_ISL_719010, EPI_ISL_719013, EPI_ISL_719014, EPI_ISL_719015, EPI_ISL_719017, EPI_ISL_719019, EPI_ISL_719020, EPI_ISL_719021, EPI_ISL_719022, EPI_ISL_719023, EPI_ISL_719024, EPI_ISL_719026, EPI_ISL_719027, EPI_ISL_719028, EPI_ISL_719029, EPI_ISL_719030, EPI_ISL_719033, EPI_ISL_719035, EPI_ISL_719038, EPI_ISL_719039, EPI_ISL_719040, EPI_ISL_719043, EPI_ISL_719044, EPI_ISL_719045, EPI_ISL_719046, EPI_ISL_719047, EPI_ISL_719048, EPI_ISL_719049, EPI_ISL_719050, EPI_ISL_719052, EPI_ISL_719053, EPI_ISL_719055, EPI_ISL_719056, EPI_ISL_719057, EPI_ISL_719058, EPI_ISL_719059, EPI_ISL_719060, EPI_ISL_719061, EPI_ISL_719063, EPI_ISL_719064, EPI_ISL_719065, EPI_ISL_719066, EPI_ISL_719067, EPI_ISL_719069, EPI_ISL_719072, EPI_ISL_719074, EPI_ISL_719075, EPI_ISL_719076, EPI_ISL_719077, EPI_ISL_719078, EPI_ISL_719079, EPI_ISL_719080, EPI_ISL_719081, EPI_ISL_719082, EPI_ISL_719083, EPI_ISL_719084, EPI_ISL_719087, EPI_ISL_719088, EPI_ISL_719089, EPI_ISL_719090, EPI_ISL_719093, EPI_ISL_719096, EPI_ISL_719097, EPI_ISL_719100, EPI_ISL_719104, EPI_ISL_719105, EPI_ISL_719106, EPI_ISL_719107, EPI_ISL_719108, EPI_ISL_719109, EPI_ISL_719111, EPI_ISL_719112, EPI_ISL_719113, EPI_ISL_719114, EPI_ISL_719116, EPI_ISL_719118, EPI_ISL_719119, EPI_ISL_719120, EPI_ISL_719121, EPI_ISL_719122, EPI_ISL_719123, EPI_ISL_719124, EPI_ISL_719126, EPI_ISL_719127, EPI_ISL_719128, EPI_ISL_719130, EPI_ISL_719131, EPI_ISL_719132, EPI_ISL_719133, EPI_ISL_719134, EPI_ISL_719137, EPI_ISL_719138, EPI_ISL_719139, EPI_ISL_719142, EPI_ISL_719143, EPI_ISL_719145, EPI_ISL_719148, EPI_ISL_719149, EPI_ISL_719150, EPI_ISL_719151, EPI_ISL_719152, EPI_ISL_719153, EPI_ISL_719154, EPI_ISL_719155, EPI_ISL_719157, EPI_ISL_719159, EPI_ISL_719160, EPI_ISL_719161, EPI_ISL_719162, EPI_ISL_719163, EPI_ISL_719164, EPI_ISL_719165, EPI_ISL_719167, EPI_ISL_719168, EPI_ISL_719170, EPI_ISL_719171, EPI_ISL_719172, EPI_ISL_719173, EPI_ISL_719175, EPI_ISL_719177, EPI_ISL_719178, EPI_ISL_719181, EPI_ISL_719182, EPI_ISL_719183, EPI_ISL_719185, EPI_ISL_719188, EPI_ISL_719189, EPI_ISL_719191, EPI_ISL_719193, EPI_ISL_719194, EPI_ISL_719195, EPI_ISL_719197, EPI_ISL_719200, EPI_ISL_719201, EPI_ISL_719202, EPI_ISL_719203, EPI_ISL_719204, EPI_ISL_719205, EPI_ISL_719206, EPI_ISL_719207, EPI_ISL_719208, EPI_ISL_719209, EPI_ISL_719210, EPI_ISL_719211, EPI_ISL_719213, EPI_ISL_719215, EPI_ISL_719216, EPI_ISL_719218, EPI_ISL_719219, EPI_ISL_719220, EPI_ISL_719221, EPI_ISL_719222, EPI_ISL_719223, EPI_ISL_719224, EPI_ISL_719226, EPI_ISL_719227, EPI_ISL_719228, EPI_ISL_719229, EPI_ISL_719230, EPI_ISL_719231, EPI_ISL_719232, EPI_ISL_719233, EPI_ISL_719234, EPI_ISL_719235, EPI_ISL_719238, EPI_ISL_719239, EPI_ISL_719240, EPI_ISL_719241, EPI_ISL_719242, EPI_ISL_719244, EPI_ISL_719245, EPI_ISL_719246, EPI_ISL_719248, EPI_ISL_719249, EPI_ISL_719250, EPI_ISL_719254, EPI_ISL_719255, EPI_ISL_719256, EPI_ISL_719258, EPI_ISL_719260, EPI_ISL_719262, EPI_ISL_719264, EPI_ISL_719266, EPI_ISL_719267, EPI_ISL_719268, EPI_ISL_719269, EPI_ISL_719270, EPI_ISL_719271, EPI_ISL_719272, EPI_ISL_719274, EPI_ISL_719275, EPI_ISL_719276, EPI_ISL_719280, EPI_ISL_719282, EPI_ISL_719283, EPI_ISL_719284, EPI_ISL_719285, EPI_ISL_719286, EPI_ISL_719287, EPI_ISL_719288, EPI_ISL_719290, EPI_ISL_719291, EPI_ISL_719292, EPI_ISL_719293, EPI_ISL_719294, EPI_ISL_719295, EPI_ISL_719296, EPI_ISL_719297, EPI_ISL_719298, EPI_ISL_719299, EPI_ISL_719301, EPI_ISL_719302, EPI_ISL_719303, EPI_ISL_719304, EPI_ISL_719305, EPI_ISL_719306, EPI_ISL_719309, EPI_ISL_719310, EPI_ISL_719313, EPI_ISL_719314, EPI_ISL_719315, EPI_ISL_719316, EPI_ISL_719317, EPI_ISL_719318, EPI_ISL_719319, EPI_ISL_719320, EPI_ISL_719322, EPI_ISL_719326, EPI_ISL_719328, EPI_ISL_719329, EPI_ISL_719330, EPI_ISL_719331, EPI_ISL_719333, EPI_ISL_719335, EPI_ISL_719336, EPI_ISL_719337, EPI_ISL_719338, EPI_ISL_719340, EPI_ISL_719344, EPI_ISL_719346, EPI_ISL_719348, EPI_ISL_719349, EPI_ISL_719350, EPI_ISL_719351, EPI_ISL_719352, EPI_ISL_719353, EPI_ISL_719355, EPI_ISL_719356, EPI_ISL_719357, EPI_ISL_719358, EPI_ISL_719359, EPI_ISL_719360, EPI_ISL_719361, EPI_ISL_719362, EPI_ISL_719365, EPI_ISL_719366, EPI_ISL_719367, EPI_ISL_719368, EPI_ISL_719370, EPI_ISL_719371, EPI_ISL_719372, EPI_ISL_719373, EPI_ISL_719374, EPI_ISL_719375, EPI_ISL_719380, EPI_ISL_719381, EPI_ISL_719383, EPI_ISL_719384, EPI_ISL_719386, EPI_ISL_719388, EPI_ISL_719390, EPI_ISL_719391, EPI_ISL_719392, EPI_ISL_719393, EPI_ISL_719394, EPI_ISL_719395, EPI_ISL_719396, EPI_ISL_719397, EPI_ISL_719398, EPI_ISL_719399, EPI_ISL_719402, EPI_ISL_719403, EPI_ISL_719404, EPI_ISL_719406, EPI_ISL_719407, EPI_ISL_719408, EPI_ISL_719410, EPI_ISL_719411, EPI_ISL_719414, EPI_ISL_719415, EPI_ISL_719416, EPI_ISL_719417, EPI_ISL_719418, EPI_ISL_719419, EPI_ISL_719420, EPI_ISL_719424, EPI_ISL_719427, EPI_ISL_719428, EPI_ISL_719429, EPI_ISL_719431, EPI_ISL_719434, EPI_ISL_719435, EPI_ISL_719436, EPI_ISL_719437, EPI_ISL_719438, EPI_ISL_719439, EPI_ISL_719440, EPI_ISL_719441, EPI_ISL_719442, EPI_ISL_719444, EPI_ISL_719445, EPI_ISL_719446, EPI_ISL_719447, EPI_ISL_719448, EPI_ISL_719449, EPI_ISL_719450, EPI_ISL_719452, EPI_ISL_719453, EPI_ISL_719455, EPI_ISL_719457, EPI_ISL_719458, EPI_ISL_719459, EPI_ISL_719460, EPI_ISL_719463, EPI_ISL_719464, EPI_ISL_719466, EPI_ISL_719467, EPI_ISL_719469, EPI_ISL_719470, EPI_ISL_719472, EPI_ISL_719474, EPI_ISL_719475, EPI_ISL_719476, EPI_ISL_719477, EPI_ISL_719478, EPI_ISL_719480, EPI_ISL_719481, EPI_ISL_719482, EPI_ISL_719483, EPI_ISL_719485, EPI_ISL_719486, EPI_ISL_719487, EPI_ISL_719488, EPI_ISL_719489, EPI_ISL_719490, EPI_ISL_719491, EPI_ISL_719493, EPI_ISL_719495, EPI_ISL_719496, EPI_ISL_719497, EPI_ISL_719498, EPI_ISL_719500, EPI_ISL_719501, EPI_ISL_719503, EPI_ISL_719506, EPI_ISL_719507, EPI_ISL_719508, EPI_ISL_719509, EPI_ISL_719511, EPI_ISL_719512, EPI_ISL_719513, EPI_ISL_719514, EPI_ISL_719516, EPI_ISL_719517, EPI_ISL_719518, EPI_ISL_719519, EPI_ISL_719522, EPI_ISL_719523, EPI_ISL_719524, EPI_ISL_719525, EPI_ISL_719526, EPI_ISL_719530, EPI_ISL_719532, EPI_ISL_719533, EPI_ISL_719535, EPI_ISL_719537, EPI_ISL_719538, EPI_ISL_719539, EPI_ISL_719540, EPI_ISL_719541, EPI_ISL_719542, EPI_ISL_719543, EPI_ISL_719544, EPI_ISL_719545, EPI_ISL_719547, EPI_ISL_719548, EPI_ISL_719549, EPI_ISL_719550, EPI_ISL_719552, EPI_ISL_719553, EPI_ISL_719555, EPI_ISL_719556, EPI_ISL_719558, EPI_ISL_719559, EPI_ISL_719563, EPI_ISL_719565, EPI_ISL_719566, EPI_ISL_719568, EPI_ISL_719570, EPI_ISL_719571, EPI_ISL_719572, EPI_ISL_719574, EPI_ISL_719575, EPI_ISL_719576, EPI_ISL_719577, EPI_ISL_719578, EPI_ISL_719579, EPI_ISL_719580, EPI_ISL_719581, EPI_ISL_719582, EPI_ISL_719583, EPI_ISL_719584, EPI_ISL_719587, EPI_ISL_719588, EPI_ISL_719589, EPI_ISL_719592, EPI_ISL_719596, EPI_ISL_719597, EPI_ISL_719598, EPI_ISL_719599, EPI_ISL_719600, EPI_ISL_719602, EPI_ISL_719603, EPI_ISL_719604

see above	Lighthouse Lab in Cambridge	Wellcome Sanger Institute for the COVID-19 Genomics UK (COG-UK) Consortium	Rob Howes, The Lighthouse Lab in Cambridge and Alex Alderton, Roberto Amato, Sonia Goncalves, Ewan Harrison, David K. Jackson, Ian Johnston, Dominic Kwiatkowski, Cordelia Langford, John Sillitoe on behalf of the Wellcome Sanger Institute COVID-19 Surveillance Team
EPI_ISL_720668	Lighthouse Lab in Glasgow	Wellcome Sanger Institute for the COVID-19 Genomics UK (COG-UK) Consortium	Harper VanSteenhouse, Yumi Kasai, David Gray, Carol Clugston, Anna Dominiczak and Alex Alderton, Roberto Amato, Sonia Goncalves, Ewan Harrison, David K. Jackson, Ian Johnston, Dominic Kwiatkowski, Cordelia Langford, John Sillitoe on behalf of the Wellcome Sanger Institute COVID-19 Surveillance Team
EPI_ISL_720671	Lighthouse Lab in Milton Keynes	Wellcome Sanger Institute for the COVID-19 Genomics UK (COG-UK) Consortium	The Lighthouse Lab in Milton Keynes and Alex Alderton, Roberto Amato, Sonia Goncalves, Ewan Harrison, David K. Jackson, Ian Johnston, Dominic Kwiatkowski, Cordelia Langford, John Sillitoe on behalf of the Wellcome Sanger Institute COVID-19 Surveillance Team
EPI_ISL_720672	Lighthouse Lab in Glasgow	Wellcome Sanger Institute for the COVID-19 Genomics UK (COG-UK) Consortium	Harper VanSteenhouse, Yumi Kasai, David Gray, Carol Clugston, Anna Dominiczak and Alex Alderton, Roberto Amato, Sonia Goncalves, Ewan Harrison, David K. Jackson, Ian Johnston, Dominic Kwiatkowski, Cordelia Langford, John Sillitoe on behalf of the Wellcome Sanger Institute COVID-19 Surveillance Team
EPI_ISL_720674, EPI_ISL_720676, EPI_ISL_720679, EPI_ISL_720681	Lighthouse Lab in Milton Keynes	Wellcome Sanger Institute for the COVID-19 Genomics UK (COG-UK) Consortium	The Lighthouse Lab in Milton Keynes and Alex Alderton, Roberto Amato, Sonia Goncalves, Ewan Harrison, David K. Jackson, Ian Johnston, Dominic Kwiatkowski, Cordelia Langford, John Sillitoe on behalf of the Wellcome Sanger Institute COVID-19 Surveillance Team
EPI_ISL_720685	Lighthouse Lab in Alderley Park	Wellcome Sanger Institute for the COVID-19 Genomics UK (COG-UK) Consortium	Jacquelyn Wynn, Mairead Hyland, The Lighthouse Lab in Alderley Park and Alex Alderton, Roberto Amato, Sonia Goncalves, Ewan Harrison, David K. Jackson, Ian Johnston, Dominic Kwiatkowski, Cordelia Langford, John Sillitoe on behalf of the Wellcome Sanger Institute COVID-19 Surveillance Team
EPI_ISL_720687	Lighthouse Lab in Milton Keynes	Wellcome Sanger Institute for the COVID-19 Genomics UK (COG-UK) Consortium	The Lighthouse Lab in Milton Keynes and Alex Alderton, Roberto Amato, Sonia Goncalves, Ewan Harrison, David K. Jackson, Ian Johnston, Dominic Kwiatkowski, Cordelia Langford, John Sillitoe on behalf of the Wellcome Sanger Institute COVID-19 Surveillance Team
EPI_ISL_720688	Lighthouse Lab in Alderley Park	Wellcome Sanger Institute for the COVID-19 Genomics UK (COG-UK) Consortium	Jacquelyn Wynn, Mairead Hyland, The Lighthouse Lab in Alderley Park and Alex Alderton, Roberto Amato, Sonia Goncalves, Ewan Harrison, David K. Jackson, Ian Johnston, Dominic Kwiatkowski, Cordelia Langford, John Sillitoe on behalf of the Wellcome Sanger Institute COVID-19 Surveillance Team
EPI_ISL_720689, EPI_ISL_720691, EPI_ISL_720692, EPI_ISL_720693	Lighthouse Lab in Milton Keynes	Wellcome Sanger Institute for the COVID-19 Genomics UK (COG-UK) Consortium	The Lighthouse Lab in Milton Keynes and Alex Alderton, Roberto Amato, Sonia Goncalves, Ewan Harrison, David K. Jackson, Ian Johnston, Dominic Kwiatkowski, Cordelia Langford, John Sillitoe on behalf of the Wellcome Sanger Institute COVID-19 Surveillance Team
EPI_ISL_720694	Lighthouse Lab in Alderley Park	Wellcome Sanger Institute for the COVID-19 Genomics UK (COG-UK) Consortium	Jacquelyn Wynn, Mairead Hyland, The Lighthouse Lab in Alderley Park and Alex Alderton, Roberto Amato, Sonia Goncalves, Ewan Harrison, David K. Jackson, Ian Johnston, Dominic Kwiatkowski, Cordelia Langford, John Sillitoe on behalf of the Wellcome Sanger Institute COVID-19 Surveillance Team
EPI_ISL_720696, EPI_ISL_720698, EPI_ISL_720701, EPI_ISL_720706, EPI_ISL_720707, EPI_ISL_720708	Lighthouse Lab in Milton Keynes	Wellcome Sanger Institute for the COVID-19 Genomics UK (COG-UK) Consortium	The Lighthouse Lab in Milton Keynes and Alex Alderton, Roberto Amato, Sonia Goncalves, Ewan Harrison, David K. Jackson, Ian Johnston, Dominic Kwiatkowski, Cordelia Langford, John Sillitoe on behalf of the Wellcome Sanger Institute COVID-19 Surveillance Team
EPI_ISL_720709	Lighthouse Lab in Alderley Park	Wellcome Sanger Institute for the COVID-19 Genomics UK (COG-UK) Consortium	Jacquelyn Wynn, Mairead Hyland, The Lighthouse Lab in Alderley Park and Alex Alderton, Roberto Amato, Sonia Goncalves, Ewan Harrison, David K. Jackson, Ian Johnston, Dominic Kwiatkowski, Cordelia Langford, John Sillitoe on behalf of the Wellcome Sanger Institute COVID-19 Surveillance Team
EPI_ISL_720710	Lighthouse Lab in Milton Keynes	Wellcome Sanger Institute for the COVID-19 Genomics UK (COG-UK) Consortium	The Lighthouse Lab in Milton Keynes and Alex Alderton, Roberto Amato, Sonia Goncalves, Ewan Harrison, David K. Jackson, Ian Johnston, Dominic Kwiatkowski, Cordelia Langford, John Sillitoe on behalf of the Wellcome Sanger Institute COVID-19 Surveillance Team
EPI_ISL_720711	Lighthouse Lab in Alderley Park	Wellcome Sanger Institute for the COVID-19 Genomics UK (COG-UK) Consortium	Jacquelyn Wynn, Mairead Hyland, The Lighthouse Lab in Alderley Park and Alex Alderton, Roberto Amato, Sonia Goncalves, Ewan Harrison, David K. Jackson, Ian Johnston, Dominic Kwiatkowski, Cordelia Langford, John Sillitoe on behalf of the Wellcome Sanger Institute COVID-19 Surveillance Team
EPI_ISL_720717, EPI_ISL_720719, EPI_ISL_720721	Lighthouse Lab in Milton Keynes	Wellcome Sanger Institute for the COVID-19 Genomics UK (COG-UK) Consortium	The Lighthouse Lab in Milton Keynes and Alex Alderton, Roberto Amato, Sonia Goncalves, Ewan Harrison, David K. Jackson, Ian Johnston, Dominic Kwiatkowski, Cordelia Langford, John Sillitoe on behalf of the Wellcome Sanger Institute COVID-19 Surveillance Team
EPI_ISL_720722	Lighthouse Lab in Alderley Park	Wellcome Sanger Institute for the COVID-19 Genomics UK (COG-UK) Consortium	Jacquelyn Wynn, Mairead Hyland, The Lighthouse Lab in Alderley Park and Alex Alderton, Roberto Amato, Sonia Goncalves, Ewan Harrison, David K. Jackson, Ian Johnston, Dominic Kwiatkowski, Cordelia Langford, John Sillitoe on behalf of the Wellcome Sanger Institute COVID-19 Surveillance Team
EPI_ISL_720723, EPI_ISL_720724	Lighthouse Lab in Milton Keynes	Wellcome Sanger Institute for the COVID-19 Genomics UK (COG-UK) Consortium	The Lighthouse Lab in Milton Keynes and Alex Alderton, Roberto Amato, Sonia Goncalves, Ewan Harrison, David K. Jackson, Ian Johnston, Dominic Kwiatkowski, Cordelia Langford, John Sillitoe on behalf of the Wellcome Sanger Institute COVID-19 Surveillance Team
EPI_ISL_720728	Lighthouse Lab in Glasgow	Wellcome Sanger Institute for the COVID-19 Genomics UK (COG-UK) Consortium	Harper VanSteenhouse, Yumi Kasai, David Gray, Carol Clugston, Anna Dominiczak and Alex Alderton, Roberto Amato, Sonia Goncalves, Ewan Harrison, David K. Jackson, Ian Johnston, Dominic Kwiatkowski, Cordelia Langford, John Sillitoe on behalf of the Wellcome Sanger Institute COVID-19 Surveillance Team
EPI_ISL_720730, EPI_ISL_720731	Lighthouse Lab in Milton Keynes	Wellcome Sanger Institute for the COVID-19 Genomics UK (COG-UK) Consortium	The Lighthouse Lab in Milton Keynes and Alex Alderton, Roberto Amato, Sonia Goncalves, Ewan Harrison, David K. Jackson, Ian Johnston, Dominic Kwiatkowski, Cordelia Langford, John Sillitoe on behalf of the Wellcome Sanger Institute COVID-19 Surveillance Team
EPI_ISL_720733	Lighthouse Lab in Glasgow	Wellcome Sanger Institute for the COVID-19 Genomics UK (COG-UK) Consortium	Harper VanSteenhouse, Yumi Kasai, David Gray, Carol Clugston, Anna Dominiczak and Alex Alderton, Roberto Amato, Sonia Goncalves, Ewan Harrison, David K. Jackson, Ian Johnston, Dominic Kwiatkowski, Cordelia Langford, John Sillitoe on behalf of the Wellcome Sanger Institute COVID-19 Surveillance Team
EPI_ISL_720736	Lighthouse Lab in Milton Keynes	Wellcome Sanger Institute for the COVID-19 Genomics UK	The Lighthouse Lab in Milton Keynes and Alex Alderton, Roberto Amato, Sonia Goncalves, Ewan Harrison, David K. Jackson, Ian Johnston, Dominic

		(COG-UK) Consortium	Kwiatkowski, Cordelia Langford, John Sillitoe on behalf of the Wellcome Sanger Institute COVID-19 Surveillance Team
EPI_ISL_720738	Lighthouse Lab in Glasgow	Wellcome Sanger Institute for the COVID-19 Genomics UK (COG-UK) Consortium	Harper VanSteenhouse, Yumi Kasai, David Gray, Carol Clugston, Anna Dominiczak and Alex Alderton, Roberto Amato, Sonia Goncalves, Ewan Harrison, David K. Jackson, Ian Johnston, Dominic Kwiatkowski, Cordelia Langford, John Sillitoe on behalf of the Wellcome Sanger Institute COVID-19 Surveillance Team
EPI_ISL_720739	Lighthouse Lab in Milton Keynes	Wellcome Sanger Institute for the COVID-19 Genomics UK (COG-UK) Consortium	The Lighthouse Lab in Milton Keynes and Alex Alderton, Roberto Amato, Sonia Goncalves, Ewan Harrison, David K. Jackson, Ian Johnston, Dominic Kwiatkowski, Cordelia Langford, John Sillitoe on behalf of the Wellcome Sanger Institute COVID-19 Surveillance Team
EPI_ISL_720741	Lighthouse Lab in Alderley Park	Wellcome Sanger Institute for the COVID-19 Genomics UK (COG-UK) Consortium	Jacquelyn Wynn, Mairead Hyland, The Lighthouse Lab in Alderley Park and Alex Alderton, Roberto Amato, Sonia Goncalves, Ewan Harrison, David K. Jackson, Ian Johnston, Dominic Kwiatkowski, Cordelia Langford, John Sillitoe on behalf of the Wellcome Sanger Institute COVID-19 Surveillance Team
EPI_ISL_720742, EPI_ISL_720743, EPI_ISL_720746, EPI_ISL_720747, EPI_ISL_720748	Lighthouse Lab in Milton Keynes	Wellcome Sanger Institute for the COVID-19 Genomics UK (COG-UK) Consortium	The Lighthouse Lab in Milton Keynes and Alex Alderton, Roberto Amato, Sonia Goncalves, Ewan Harrison, David K. Jackson, Ian Johnston, Dominic Kwiatkowski, Cordelia Langford, John Sillitoe on behalf of the Wellcome Sanger Institute COVID-19 Surveillance Team
EPI_ISL_720752	Lighthouse Lab in Glasgow	Wellcome Sanger Institute for the COVID-19 Genomics UK (COG-UK) Consortium	Harper VanSteenhouse, Yumi Kasai, David Gray, Carol Clugston, Anna Dominiczak and Alex Alderton, Roberto Amato, Sonia Goncalves, Ewan Harrison, David K. Jackson, Ian Johnston, Dominic Kwiatkowski, Cordelia Langford, John Sillitoe on behalf of the Wellcome Sanger Institute COVID-19 Surveillance Team
EPI_ISL_720753	Lighthouse Lab in Milton Keynes	Wellcome Sanger Institute for the COVID-19 Genomics UK (COG-UK) Consortium	The Lighthouse Lab in Milton Keynes and Alex Alderton, Roberto Amato, Sonia Goncalves, Ewan Harrison, David K. Jackson, Ian Johnston, Dominic Kwiatkowski, Cordelia Langford, John Sillitoe on behalf of the Wellcome Sanger Institute COVID-19 Surveillance Team
EPI_ISL_720755	Lighthouse Lab in Glasgow	Wellcome Sanger Institute for the COVID-19 Genomics UK (COG-UK) Consortium	Harper VanSteenhouse, Yumi Kasai, David Gray, Carol Clugston, Anna Dominiczak and Alex Alderton, Roberto Amato, Sonia Goncalves, Ewan Harrison, David K. Jackson, Ian Johnston, Dominic Kwiatkowski, Cordelia Langford, John Sillitoe on behalf of the Wellcome Sanger Institute COVID-19 Surveillance Team
EPI_ISL_720756	Lighthouse Lab in Alderley Park	Wellcome Sanger Institute for the COVID-19 Genomics UK (COG-UK) Consortium	Jacquelyn Wynn, Mairead Hyland, The Lighthouse Lab in Alderley Park and Alex Alderton, Roberto Amato, Sonia Goncalves, Ewan Harrison, David K. Jackson, Ian Johnston, Dominic Kwiatkowski, Cordelia Langford, John Sillitoe on behalf of the Wellcome Sanger Institute COVID-19 Surveillance Team
EPI_ISL_720757	Lighthouse Lab in Milton Keynes	Wellcome Sanger Institute for the COVID-19 Genomics UK (COG-UK) Consortium	The Lighthouse Lab in Milton Keynes and Alex Alderton, Roberto Amato, Sonia Goncalves, Ewan Harrison, David K. Jackson, Ian Johnston, Dominic Kwiatkowski, Cordelia Langford, John Sillitoe on behalf of the Wellcome Sanger Institute COVID-19 Surveillance Team
EPI_ISL_720764, EPI_ISL_720765	Lighthouse Lab in Alderley Park	Wellcome Sanger Institute for the COVID-19 Genomics UK (COG-UK) Consortium	Jacquelyn Wynn, Mairead Hyland, The Lighthouse Lab in Alderley Park and Alex Alderton, Roberto Amato, Sonia Goncalves, Ewan Harrison, David K. Jackson, Ian Johnston, Dominic Kwiatkowski, Cordelia Langford, John Sillitoe on behalf of the Wellcome Sanger Institute COVID-19 Surveillance Team
EPI_ISL_720767	Lighthouse Lab in Glasgow	Wellcome Sanger Institute for the COVID-19 Genomics UK (COG-UK) Consortium	Harper VanSteenhouse, Yumi Kasai, David Gray, Carol Clugston, Anna Dominiczak and Alex Alderton, Roberto Amato, Sonia Goncalves, Ewan Harrison, David K. Jackson, Ian Johnston, Dominic Kwiatkowski, Cordelia Langford, John Sillitoe on behalf of the Wellcome Sanger Institute COVID-19 Surveillance Team
EPI_ISL_720768, EPI_ISL_720769	Lighthouse Lab in Milton Keynes	Wellcome Sanger Institute for the COVID-19 Genomics UK (COG-UK) Consortium	The Lighthouse Lab in Milton Keynes and Alex Alderton, Roberto Amato, Sonia Goncalves, Ewan Harrison, David K. Jackson, Ian Johnston, Dominic Kwiatkowski, Cordelia Langford, John Sillitoe on behalf of the Wellcome Sanger Institute COVID-19 Surveillance Team
EPI_ISL_720770	Lighthouse Lab in Alderley Park	Wellcome Sanger Institute for the COVID-19 Genomics UK (COG-UK) Consortium	Jacquelyn Wynn, Mairead Hyland, The Lighthouse Lab in Alderley Park and Alex Alderton, Roberto Amato, Sonia Goncalves, Ewan Harrison, David K. Jackson, Ian Johnston, Dominic Kwiatkowski, Cordelia Langford, John Sillitoe on behalf of the Wellcome Sanger Institute COVID-19 Surveillance Team
EPI_ISL_720771, EPI_ISL_720772	Lighthouse Lab in Milton Keynes	Wellcome Sanger Institute for the COVID-19 Genomics UK (COG-UK) Consortium	The Lighthouse Lab in Milton Keynes and Alex Alderton, Roberto Amato, Sonia Goncalves, Ewan Harrison, David K. Jackson, Ian Johnston, Dominic Kwiatkowski, Cordelia Langford, John Sillitoe on behalf of the Wellcome Sanger Institute COVID-19 Surveillance Team
EPI_ISL_720773	Lighthouse Lab in Alderley Park	Wellcome Sanger Institute for the COVID-19 Genomics UK (COG-UK) Consortium	Jacquelyn Wynn, Mairead Hyland, The Lighthouse Lab in Alderley Park and Alex Alderton, Roberto Amato, Sonia Goncalves, Ewan Harrison, David K. Jackson, Ian Johnston, Dominic Kwiatkowski, Cordelia Langford, John Sillitoe on behalf of the Wellcome Sanger Institute COVID-19 Surveillance Team
EPI_ISL_720774, EPI_ISL_720775	Lighthouse Lab in Milton Keynes	Wellcome Sanger Institute for the COVID-19 Genomics UK (COG-UK) Consortium	The Lighthouse Lab in Milton Keynes and Alex Alderton, Roberto Amato, Sonia Goncalves, Ewan Harrison, David K. Jackson, Ian Johnston, Dominic Kwiatkowski, Cordelia Langford, John Sillitoe on behalf of the Wellcome Sanger Institute COVID-19 Surveillance Team
EPI_ISL_720776	Lighthouse Lab in Glasgow	Wellcome Sanger Institute for the COVID-19 Genomics UK (COG-UK) Consortium	Harper VanSteenhouse, Yumi Kasai, David Gray, Carol Clugston, Anna Dominiczak and Alex Alderton, Roberto Amato, Sonia Goncalves, Ewan Harrison, David K. Jackson, Ian Johnston, Dominic Kwiatkowski, Cordelia Langford, John Sillitoe on behalf of the Wellcome Sanger Institute COVID-19 Surveillance Team
EPI_ISL_720778	Lighthouse Lab in Milton Keynes	Wellcome Sanger Institute for the COVID-19 Genomics UK (COG-UK) Consortium	The Lighthouse Lab in Milton Keynes and Alex Alderton, Roberto Amato, Sonia Goncalves, Ewan Harrison, David K. Jackson, Ian Johnston, Dominic Kwiatkowski, Cordelia Langford, John Sillitoe on behalf of the Wellcome Sanger Institute COVID-19 Surveillance Team
EPI_ISL_720779, EPI_ISL_720780	Lighthouse Lab in Alderley Park	Wellcome Sanger Institute for the COVID-19 Genomics UK (COG-UK) Consortium	Jacquelyn Wynn, Mairead Hyland, The Lighthouse Lab in Alderley Park and Alex Alderton, Roberto Amato, Sonia Goncalves, Ewan Harrison, David K. Jackson, Ian Johnston, Dominic Kwiatkowski, Cordelia Langford, John Sillitoe on behalf of the Wellcome Sanger Institute COVID-19 Surveillance Team
EPI_ISL_720781, EPI_ISL_720783, EPI_ISL_720784, EPI_ISL_720785	Lighthouse Lab in Milton Keynes	Wellcome Sanger Institute for the COVID-19 Genomics UK (COG-UK) Consortium	The Lighthouse Lab in Milton Keynes and Alex Alderton, Roberto Amato, Sonia Goncalves, Ewan Harrison, David K. Jackson, Ian Johnston, Dominic Kwiatkowski, Cordelia Langford, John Sillitoe on behalf of the Wellcome Sanger Institute COVID-19 Surveillance Team
EPI_ISL_720787	Lighthouse Lab in Glasgow	Wellcome Sanger Institute for the COVID-19 Genomics UK (COG-UK) Consortium	Harper VanSteenhouse, Yumi Kasai, David Gray, Carol Clugston, Anna Dominiczak and Alex Alderton, Roberto Amato, Sonia Goncalves, Ewan Harrison, David K. Jackson, Ian Johnston, Dominic Kwiatkowski, Cordelia Langford, John Sillitoe on behalf of the Wellcome Sanger Institute COVID-19 Surveillance Team
EPI_ISL_720789, EPI_ISL_720791	Lighthouse Lab in Milton Keynes	Wellcome Sanger Institute for the COVID-19 Genomics UK (COG-UK) Consortium	The Lighthouse Lab in Milton Keynes and Alex Alderton, Roberto Amato, Sonia Goncalves, Ewan Harrison, David K. Jackson, Ian Johnston, Dominic Kwiatkowski, Cordelia Langford, John Sillitoe on behalf of the Wellcome Sanger Institute COVID-19 Surveillance Team
EPI_ISL_720792	Lighthouse Lab in Glasgow	Wellcome Sanger Institute for the COVID-19 Genomics UK (COG-UK) Consortium	Harper VanSteenhouse, Yumi Kasai, David Gray, Carol Clugston, Anna Dominiczak and Alex Alderton, Roberto Amato, Sonia Goncalves, Ewan Harrison, David K. Jackson, Ian Johnston, Dominic Kwiatkowski, Cordelia Langford, John Sillitoe on behalf of the Wellcome Sanger Institute COVID-19 Surveillance Team
EPI_ISL_720794, EPI_ISL_720797, EPI_ISL_720799, EPI_ISL_720801, EPI_ISL_720804, EPI_ISL_720808, EPI_ISL_720809	Lighthouse Lab in Milton Keynes	Wellcome Sanger Institute for the COVID-19 Genomics UK (COG-UK) Consortium	The Lighthouse Lab in Milton Keynes and Alex Alderton, Roberto Amato, Sonia Goncalves, Ewan Harrison, David K. Jackson, Ian Johnston, Dominic Kwiatkowski, Cordelia Langford, John Sillitoe on behalf of the Wellcome Sanger Institute COVID-19 Surveillance Team
EPI_ISL_720810	Lighthouse Lab in Alderley Park	Wellcome Sanger Institute for the COVID-19 Genomics UK (COG-UK) Consortium	Jacquelyn Wynn, Mairead Hyland, The Lighthouse Lab in Alderley Park and Alex Alderton, Roberto Amato, Sonia Goncalves, Ewan Harrison, David K. Jackson, Ian Johnston, Dominic Kwiatkowski, Cordelia Langford, John Sillitoe on behalf of the Wellcome Sanger Institute COVID-19 Surveillance Team
EPI_ISL_720811	Lighthouse Lab in Glasgow	Wellcome Sanger Institute for the COVID-19 Genomics UK (COG-UK) Consortium	Harper VanSteenhouse, Yumi Kasai, David Gray, Carol Clugston, Anna Dominiczak and Alex Alderton, Roberto Amato, Sonia Goncalves, Ewan Harrison, David K. Jackson, Ian Johnston, Dominic Kwiatkowski, Cordelia Langford, John Sillitoe on behalf of the Wellcome Sanger Institute COVID-19 Surveillance Team
EPI_ISL_720812	Lighthouse Lab in Milton Keynes	Wellcome Sanger Institute for the COVID-19 Genomics UK (COG-UK) Consortium	The Lighthouse Lab in Milton Keynes and Alex Alderton, Roberto Amato, Sonia Goncalves, Ewan Harrison, David K. Jackson, Ian Johnston, Dominic Kwiatkowski, Cordelia Langford, John Sillitoe on behalf of the Wellcome Sanger Institute COVID-19 Surveillance Team
EPI_ISL_720814	Lighthouse Lab in Alderley Park	Wellcome Sanger Institute for the COVID-19 Genomics UK (COG-UK) Consortium	Jacquelyn Wynn, Mairead Hyland, The Lighthouse Lab in Alderley Park and Alex Alderton, Roberto Amato, Sonia Goncalves, Ewan Harrison, David K. Jackson, Ian Johnston, Dominic Kwiatkowski, Cordelia Langford, John Sillitoe on behalf of the Wellcome Sanger Institute COVID-19 Surveillance Team
EPI_ISL_720817, EPI_ISL_720820, EPI_ISL_720822	Lighthouse Lab in Milton Keynes	Wellcome Sanger Institute for the COVID-19 Genomics UK (COG-UK) Consortium	The Lighthouse Lab in Milton Keynes and Alex Alderton, Roberto Amato, Sonia Goncalves, Ewan Harrison, David K. Jackson, Ian Johnston, Dominic Kwiatkowski, Cordelia Langford, John Sillitoe on behalf of the Wellcome Sanger Institute COVID-19 Surveillance Team
EPI_ISL_720827	Lighthouse Lab in Alderley Park	Wellcome Sanger Institute for the COVID-19 Genomics UK (COG-UK) Consortium	Jacquelyn Wynn, Mairead Hyland, The Lighthouse Lab in Alderley Park and Alex Alderton, Roberto Amato, Sonia Goncalves, Ewan Harrison, David K. Jackson, Ian Johnston, Dominic Kwiatkowski, Cordelia Langford, John Sillitoe on behalf of the Wellcome Sanger Institute COVID-19 Surveillance Team

EPI_ISL_720829, EPI_ISL_720830	Lighthouse Lab in Milton Keynes	Wellcome Sanger Institute for the COVID-19 Genomics UK (COG-UK) Consortium	The Lighthouse Lab in Milton Keynes and Alex Alderton, Roberto Amato, Sonia Goncalves, Ewan Harrison, David K. Jackson, Ian Johnston, Dominic Kwiatkowski, Cordelia Langford, John Sillitoe on behalf of the Wellcome Sanger Institute COVID-19 Surveillance Team
EPI_ISL_720831	Lighthouse Lab in Glasgow	Wellcome Sanger Institute for the COVID-19 Genomics UK (COG-UK) Consortium	Harper VanSteenhouse, Yumi Kasai, David Gray, Carol Clugston, Anna Dominiczak and Alex Alderton, Roberto Amato, Sonia Goncalves, Ewan Harrison, David K. Jackson, Ian Johnston, Dominic Kwiatkowski, Cordelia Langford, John Sillitoe on behalf of the Wellcome Sanger Institute COVID-19 Surveillance Team
EPI_ISL_720832, EPI_ISL_720833	Lighthouse Lab in Milton Keynes	Wellcome Sanger Institute for the COVID-19 Genomics UK (COG-UK) Consortium	The Lighthouse Lab in Milton Keynes and Alex Alderton, Roberto Amato, Sonia Goncalves, Ewan Harrison, David K. Jackson, Ian Johnston, Dominic Kwiatkowski, Cordelia Langford, John Sillitoe on behalf of the Wellcome Sanger Institute COVID-19 Surveillance Team
EPI_ISL_720834	Lighthouse Lab in Glasgow	Wellcome Sanger Institute for the COVID-19 Genomics UK (COG-UK) Consortium	Harper VanSteenhouse, Yumi Kasai, David Gray, Carol Clugston, Anna Dominiczak and Alex Alderton, Roberto Amato, Sonia Goncalves, Ewan Harrison, David K. Jackson, Ian Johnston, Dominic Kwiatkowski, Cordelia Langford, John Sillitoe on behalf of the Wellcome Sanger Institute COVID-19 Surveillance Team
EPI_ISL_720835, EPI_ISL_720838, EPI_ISL_720839	Lighthouse Lab in Milton Keynes	Wellcome Sanger Institute for the COVID-19 Genomics UK (COG-UK) Consortium	The Lighthouse Lab in Milton Keynes and Alex Alderton, Roberto Amato, Sonia Goncalves, Ewan Harrison, David K. Jackson, Ian Johnston, Dominic Kwiatkowski, Cordelia Langford, John Sillitoe on behalf of the Wellcome Sanger Institute COVID-19 Surveillance Team
EPI_ISL_720841	Lighthouse Lab in Glasgow	Wellcome Sanger Institute for the COVID-19 Genomics UK (COG-UK) Consortium	Harper VanSteenhouse, Yumi Kasai, David Gray, Carol Clugston, Anna Dominiczak and Alex Alderton, Roberto Amato, Sonia Goncalves, Ewan Harrison, David K. Jackson, Ian Johnston, Dominic Kwiatkowski, Cordelia Langford, John Sillitoe on behalf of the Wellcome Sanger Institute COVID-19 Surveillance Team
EPI_ISL_720842	Lighthouse Lab in Alderley Park	Wellcome Sanger Institute for the COVID-19 Genomics UK (COG-UK) Consortium	Jacquelyn Wynn, Mairead Hyland, The Lighthouse Lab in Alderley Park and Alex Alderton, Roberto Amato, Sonia Goncalves, Ewan Harrison, David K. Jackson, Ian Johnston, Dominic Kwiatkowski, Cordelia Langford, John Sillitoe on behalf of the Wellcome Sanger Institute COVID-19 Surveillance Team
EPI_ISL_720843, EPI_ISL_720844	Lighthouse Lab in Milton Keynes	Wellcome Sanger Institute for the COVID-19 Genomics UK (COG-UK) Consortium	The Lighthouse Lab in Milton Keynes and Alex Alderton, Roberto Amato, Sonia Goncalves, Ewan Harrison, David K. Jackson, Ian Johnston, Dominic Kwiatkowski, Cordelia Langford, John Sillitoe on behalf of the Wellcome Sanger Institute COVID-19 Surveillance Team
EPI_ISL_720845, EPI_ISL_720846	Lighthouse Lab in Alderley Park	Wellcome Sanger Institute for the COVID-19 Genomics UK (COG-UK) Consortium	Jacquelyn Wynn, Mairead Hyland, The Lighthouse Lab in Alderley Park and Alex Alderton, Roberto Amato, Sonia Goncalves, Ewan Harrison, David K. Jackson, Ian Johnston, Dominic Kwiatkowski, Cordelia Langford, John Sillitoe on behalf of the Wellcome Sanger Institute COVID-19 Surveillance Team
EPI_ISL_720847	Lighthouse Lab in Milton Keynes	Wellcome Sanger Institute for the COVID-19 Genomics UK (COG-UK) Consortium	The Lighthouse Lab in Milton Keynes and Alex Alderton, Roberto Amato, Sonia Goncalves, Ewan Harrison, David K. Jackson, Ian Johnston, Dominic Kwiatkowski, Cordelia Langford, John Sillitoe on behalf of the Wellcome Sanger Institute COVID-19 Surveillance Team
EPI_ISL_720848	Lighthouse Lab in Alderley Park	Wellcome Sanger Institute for the COVID-19 Genomics UK (COG-UK) Consortium	Jacquelyn Wynn, Mairead Hyland, The Lighthouse Lab in Alderley Park and Alex Alderton, Roberto Amato, Sonia Goncalves, Ewan Harrison, David K. Jackson, Ian Johnston, Dominic Kwiatkowski, Cordelia Langford, John Sillitoe on behalf of the Wellcome Sanger Institute COVID-19 Surveillance Team
EPI_ISL_720850	Lighthouse Lab in Glasgow	Wellcome Sanger Institute for the COVID-19 Genomics UK (COG-UK) Consortium	Harper VanSteenhouse, Yumi Kasai, David Gray, Carol Clugston, Anna Dominiczak and Alex Alderton, Roberto Amato, Sonia Goncalves, Ewan Harrison, David K. Jackson, Ian Johnston, Dominic Kwiatkowski, Cordelia Langford, John Sillitoe on behalf of the Wellcome Sanger Institute COVID-19 Surveillance Team
EPI_ISL_720851	Lighthouse Lab in Milton Keynes	Wellcome Sanger Institute for the COVID-19 Genomics UK (COG-UK) Consortium	The Lighthouse Lab in Milton Keynes and Alex Alderton, Roberto Amato, Sonia Goncalves, Ewan Harrison, David K. Jackson, Ian Johnston, Dominic Kwiatkowski, Cordelia Langford, John Sillitoe on behalf of the Wellcome Sanger Institute COVID-19 Surveillance Team
EPI_ISL_720853	Lighthouse Lab in Alderley Park	Wellcome Sanger Institute for the COVID-19 Genomics UK (COG-UK) Consortium	Jacquelyn Wynn, Mairead Hyland, The Lighthouse Lab in Alderley Park and Alex Alderton, Roberto Amato, Sonia Goncalves, Ewan Harrison, David K. Jackson, Ian Johnston, Dominic Kwiatkowski, Cordelia Langford, John Sillitoe on behalf of the Wellcome Sanger Institute COVID-19 Surveillance Team
EPI_ISL_720854, EPI_ISL_720855	Lighthouse Lab in Milton Keynes	Wellcome Sanger Institute for the COVID-19 Genomics UK (COG-UK) Consortium	The Lighthouse Lab in Milton Keynes and Alex Alderton, Roberto Amato, Sonia Goncalves, Ewan Harrison, David K. Jackson, Ian Johnston, Dominic Kwiatkowski, Cordelia Langford, John Sillitoe on behalf of the Wellcome Sanger Institute COVID-19 Surveillance Team
EPI_ISL_720856	Lighthouse Lab in Glasgow	Wellcome Sanger Institute for the COVID-19 Genomics UK (COG-UK) Consortium	Harper VanSteenhouse, Yumi Kasai, David Gray, Carol Clugston, Anna Dominiczak and Alex Alderton, Roberto Amato, Sonia Goncalves, Ewan Harrison, David K. Jackson, Ian Johnston, Dominic Kwiatkowski, Cordelia Langford, John Sillitoe on behalf of the Wellcome Sanger Institute COVID-19 Surveillance Team
EPI_ISL_720858	Lighthouse Lab in Alderley Park	Wellcome Sanger Institute for the COVID-19 Genomics UK (COG-UK) Consortium	Jacquelyn Wynn, Mairead Hyland, The Lighthouse Lab in Alderley Park and Alex Alderton, Roberto Amato, Sonia Goncalves, Ewan Harrison, David K. Jackson, Ian Johnston, Dominic Kwiatkowski, Cordelia Langford, John Sillitoe on behalf of the Wellcome Sanger Institute COVID-19 Surveillance Team
EPI_ISL_720860, EPI_ISL_720861, EPI_ISL_720871, EPI_ISL_720873, EPI_ISL_720874, EPI_ISL_720876	Lighthouse Lab in Milton Keynes	Wellcome Sanger Institute for the COVID-19 Genomics UK (COG-UK) Consortium	The Lighthouse Lab in Milton Keynes and Alex Alderton, Roberto Amato, Sonia Goncalves, Ewan Harrison, David K. Jackson, Ian Johnston, Dominic Kwiatkowski, Cordelia Langford, John Sillitoe on behalf of the Wellcome Sanger Institute COVID-19 Surveillance Team
EPI_ISL_720877	Lighthouse Lab in Glasgow	Wellcome Sanger Institute for the COVID-19 Genomics UK (COG-UK) Consortium	Harper VanSteenhouse, Yumi Kasai, David Gray, Carol Clugston, Anna Dominiczak and Alex Alderton, Roberto Amato, Sonia Goncalves, Ewan Harrison, David K. Jackson, Ian Johnston, Dominic Kwiatkowski, Cordelia Langford, John Sillitoe on behalf of the Wellcome Sanger Institute COVID-19 Surveillance Team
EPI_ISL_720878, EPI_ISL_720879	Lighthouse Lab in Milton Keynes	Wellcome Sanger Institute for the COVID-19 Genomics UK (COG-UK) Consortium	The Lighthouse Lab in Milton Keynes and Alex Alderton, Roberto Amato, Sonia Goncalves, Ewan Harrison, David K. Jackson, Ian Johnston, Dominic Kwiatkowski, Cordelia Langford, John Sillitoe on behalf of the Wellcome Sanger Institute COVID-19 Surveillance Team
EPI_ISL_720880	Lighthouse Lab in Alderley Park	Wellcome Sanger Institute for the COVID-19 Genomics UK (COG-UK) Consortium	Jacquelyn Wynn, Mairead Hyland, The Lighthouse Lab in Alderley Park and Alex Alderton, Roberto Amato, Sonia Goncalves, Ewan Harrison, David K. Jackson, Ian Johnston, Dominic Kwiatkowski, Cordelia Langford, John Sillitoe on behalf of the Wellcome Sanger Institute COVID-19 Surveillance Team
EPI_ISL_720882, EPI_ISL_720883, EPI_ISL_720884	Lighthouse Lab in Milton Keynes	Wellcome Sanger Institute for the COVID-19 Genomics UK (COG-UK) Consortium	The Lighthouse Lab in Milton Keynes and Alex Alderton, Roberto Amato, Sonia Goncalves, Ewan Harrison, David K. Jackson, Ian Johnston, Dominic Kwiatkowski, Cordelia Langford, John Sillitoe on behalf of the Wellcome Sanger Institute COVID-19 Surveillance Team
EPI_ISL_720886	Lighthouse Lab in Alderley Park	Wellcome Sanger Institute for the COVID-19 Genomics UK (COG-UK) Consortium	Jacquelyn Wynn, Mairead Hyland, The Lighthouse Lab in Alderley Park and Alex Alderton, Roberto Amato, Sonia Goncalves, Ewan Harrison, David K. Jackson, Ian Johnston, Dominic Kwiatkowski, Cordelia Langford, John Sillitoe on behalf of the Wellcome Sanger Institute COVID-19 Surveillance Team
EPI_ISL_720887, EPI_ISL_720888, EPI_ISL_720892	Lighthouse Lab in Milton Keynes	Wellcome Sanger Institute for the COVID-19 Genomics UK (COG-UK) Consortium	The Lighthouse Lab in Milton Keynes and Alex Alderton, Roberto Amato, Sonia Goncalves, Ewan Harrison, David K. Jackson, Ian Johnston, Dominic Kwiatkowski, Cordelia Langford, John Sillitoe on behalf of the Wellcome Sanger Institute COVID-19 Surveillance Team
EPI_ISL_720894	Lighthouse Lab in Alderley Park	Wellcome Sanger Institute for the COVID-19 Genomics UK (COG-UK) Consortium	Jacquelyn Wynn, Mairead Hyland, The Lighthouse Lab in Alderley Park and Alex Alderton, Roberto Amato, Sonia Goncalves, Ewan Harrison, David K. Jackson, Ian Johnston, Dominic Kwiatkowski, Cordelia Langford, John Sillitoe on behalf of the Wellcome Sanger Institute COVID-19 Surveillance Team
EPI_ISL_720895, EPI_ISL_720896	Lighthouse Lab in Milton Keynes	Wellcome Sanger Institute for the COVID-19 Genomics UK (COG-UK) Consortium	The Lighthouse Lab in Milton Keynes and Alex Alderton, Roberto Amato, Sonia Goncalves, Ewan Harrison, David K. Jackson, Ian Johnston, Dominic Kwiatkowski, Cordelia Langford, John Sillitoe on behalf of the Wellcome Sanger Institute COVID-19 Surveillance Team
EPI_ISL_720897	Lighthouse Lab in Glasgow	Wellcome Sanger Institute for the COVID-19 Genomics UK (COG-UK) Consortium	Harper VanSteenhouse, Yumi Kasai, David Gray, Carol Clugston, Anna Dominiczak and Alex Alderton, Roberto Amato, Sonia Goncalves, Ewan Harrison, David K. Jackson, Ian Johnston, Dominic Kwiatkowski, Cordelia Langford, John Sillitoe on behalf of the Wellcome Sanger Institute COVID-19 Surveillance Team
EPI_ISL_720898	Lighthouse Lab in Alderley Park	Wellcome Sanger Institute for the COVID-19 Genomics UK (COG-UK) Consortium	Jacquelyn Wynn, Mairead Hyland, The Lighthouse Lab in Alderley Park and Alex Alderton, Roberto Amato, Sonia Goncalves, Ewan Harrison, David K. Jackson, Ian Johnston, Dominic Kwiatkowski, Cordelia Langford, John Sillitoe on behalf of the Wellcome Sanger Institute COVID-19 Surveillance Team
EPI_ISL_720899, EPI_ISL_720901, EPI_ISL_720902, EPI_ISL_720903, EPI_ISL_720906, EPI_ISL_720908	Lighthouse Lab in Milton Keynes	Wellcome Sanger Institute for the COVID-19 Genomics UK (COG-UK) Consortium	The Lighthouse Lab in Milton Keynes and Alex Alderton, Roberto Amato, Sonia Goncalves, Ewan Harrison, David K. Jackson, Ian Johnston, Dominic Kwiatkowski, Cordelia Langford, John Sillitoe on behalf of the Wellcome Sanger Institute COVID-19 Surveillance Team
EPI_ISL_721517	Lighthouse Lab in Alderley Park	Wellcome Sanger Institute for the COVID-19 Genomics UK (COG-UK) Consortium	Jacquelyn Wynn, Mairead Hyland, The Lighthouse Lab in Alderley Park and Alex Alderton, Roberto Amato, Sonia Goncalves, Ewan Harrison, David K. Jackson, Ian Johnston, Dominic Kwiatkowski, Cordelia Langford, John Sillitoe on behalf of the Wellcome Sanger Institute COVID-19 Surveillance Team
EPI_ISL_721521	Lighthouse Lab in Glasgow	Wellcome Sanger Institute for the COVID-19 Genomics UK (COG-UK) Consortium	Harper VanSteenhouse, Yumi Kasai, David Gray, Carol Clugston, Anna Dominiczak and Alex Alderton, Roberto Amato, Sonia Goncalves, Ewan Harrison, David K. Jackson, Ian Johnston, Dominic Kwiatkowski, Cordelia Langford, John Sillitoe on behalf of the Wellcome Sanger Institute COVID-19 Surveillance Team

EPI_ISL_721525	Lighthouse Lab in Alderley Park	Wellcome Sanger Institute for the COVID-19 Genomics UK (COG-UK) Consortium	Jacquelyn Wynn, Mairead Hyland, The Lighthouse Lab in Alderley Park and Alex Alderton, Roberto Amato, Sonia Goncalves, Ewan Harrison, David K. Jackson, Ian Johnston, Dominic Kwiatkowski, Cordelia Langford, John Sillitoe on behalf of the Wellcome Sanger Institute COVID-19 Surveillance Team
EPI_ISL_721820, EPI_ISL_721822, EPI_ISL_721903, EPI_ISL_721973, EPI_ISL_721974, EPI_ISL_721979	Viollier AG	Department of Biosystems Science and Engineering, ETH Zürich	Christian Beisel
EPI_ISL_722370, EPI_ISL_722433, EPI_ISL_722434, EPI_ISL_722456, EPI_ISL_722459, EPI_ISL_722460, EPI_ISL_722461, EPI_ISL_722462, EPI_ISL_722758, EPI_ISL_722759, EPI_ISL_722760, EPI_ISL_722761, EPI_ISL_722762, EPI_ISL_722764, EPI_ISL_722765, EPI_ISL_722766, EPI_ISL_722767, EPI_ISL_722768, EPI_ISL_722769, EPI_ISL_722770, EPI_ISL_722771, EPI_ISL_722772, EPI_ISL_722773, EPI_ISL_722774, EPI_ISL_722775, EPI_ISL_722776, EPI_ISL_722777, EPI_ISL_722778, EPI_ISL_722779, EPI_ISL_722780, EPI_ISL_722781, EPI_ISL_722782, EPI_ISL_722783, EPI_ISL_722784, EPI_ISL_722785, EPI_ISL_722786, EPI_ISL_722787, EPI_ISL_722788, EPI_ISL_722789, EPI_ISL_722790, EPI_ISL_722792, EPI_ISL_722793, EPI_ISL_722794, EPI_ISL_722795, EPI_ISL_722796, EPI_ISL_722797, EPI_ISL_722798, EPI_ISL_722799			
see above	Dutch COVID-19 response team	Erasmus Medical Center	Bas Oude Munnink, Reina Sikkema, David Nieuwenhuijse, Irina Chestakova, Anne van der Linden, Marjan Boter, Emmanuelle Munger, Corine GeurtsvanKessel, Annemiek van der Eijk, Richard Molenkamp, Marion Koopmans, on behalf of the Dutch national COVID-19 response team.
EPI_ISL_722958, EPI_ISL_722960, EPI_ISL_722961, EPI_ISL_722964, EPI_ISL_722965, EPI_ISL_722966	Department of Clinical Microbiology	GIGA Medical Genomics	Keith Durkin, Maria Artesi, Sébastien Bontems, Raphaël Boreux, Bouchra Boujemla, Cécile Meex, Pierrette Melin, Marie-Pierre Hayette, Vincent Bours
EPI_ISL_722979, EPI_ISL_722980, EPI_ISL_722981, EPI_ISL_722984, EPI_ISL_722985, EPI_ISL_722987, EPI_ISL_722991, EPI_ISL_722992, EPI_ISL_722993, EPI_ISL_722994, EPI_ISL_722998, EPI_ISL_722999, EPI_ISL_723000, EPI_ISL_723004, EPI_ISL_723006, EPI_ISL_723024, EPI_ISL_723027, EPI_ISL_723028, EPI_ISL_723029, EPI_ISL_723032, EPI_ISL_723034, EPI_ISL_723035, EPI_ISL_723036, EPI_ISL_723043, EPI_ISL_723044			
see above	Respiratory Virus Unit, National Infection Service, Public Health England	COVID-19 Genomics UK (COG-UK) Consortium	PHE Covid Sequencing Team
EPI_ISL_723137, EPI_ISL_723138, EPI_ISL_723146	Minnesota Department of Health, Public Health Laboratory	Minnesota Department of Health, Public Health Laboratory	Alexandra Lorentz, Jacob Garfin, Matt Plumb, and Xiong Wang
EPI_ISL_723158, EPI_ISL_723159, EPI_ISL_723161, EPI_ISL_723162, EPI_ISL_723163, EPI_ISL_723165, EPI_ISL_723167, EPI_ISL_723168, EPI_ISL_723170, EPI_ISL_723171, EPI_ISL_723172, EPI_ISL_723173, EPI_ISL_723174, EPI_ISL_723175, EPI_ISL_723176, EPI_ISL_723177, EPI_ISL_723182, EPI_ISL_723183, EPI_ISL_723184, EPI_ISL_723186, EPI_ISL_723187, EPI_ISL_723188, EPI_ISL_723189, EPI_ISL_723190, EPI_ISL_723191, EPI_ISL_723194, EPI_ISL_723195, EPI_ISL_723252, EPI_ISL_723277, EPI_ISL_723278, EPI_ISL_723279, EPI_ISL_723367, EPI_ISL_723369, EPI_ISL_723370, EPI_ISL_723371, EPI_ISL_723372, EPI_ISL_723373, EPI_ISL_723374, EPI_ISL_723375, EPI_ISL_723376, EPI_ISL_723377, EPI_ISL_723378, EPI_ISL_723379, EPI_ISL_723380, EPI_ISL_723382, EPI_ISL_723384, EPI_ISL_723385, EPI_ISL_723387, EPI_ISL_723388, EPI_ISL_723389, EPI_ISL_723390, EPI_ISL_723391, EPI_ISL_723392, EPI_ISL_723393, EPI_ISL_723446, EPI_ISL_723454, EPI_ISL_723455, EPI_ISL_723458, EPI_ISL_723460, EPI_ISL_723461, EPI_ISL_723462, EPI_ISL_723463, EPI_ISL_723464, EPI_ISL_723465, EPI_ISL_723467			
see above	Dutch COVID-19 response team	National Institute for Public Health and the Environment (RIVM)	Adam Meijer, Harry Vennema, Jeroen Cremer, Sharon van den Brink, Bas van der Veer, AnneMarie van den Brandt, Florian Zwagemaker, Dennis Schmitz, Chantal Reusken, on behalf of the national COVID-19 response team
EPI_ISL_723922, EPI_ISL_723924, EPI_ISL_723943, EPI_ISL_723944, EPI_ISL_723946	Department of Pathology, University of Cambridge	COVID-19 Genomics UK (COG-UK) Consortium	Aminu S. Jahun, Yasmin Chaudhry, Grant Hall, Iliana Georgana, Myra Hosmillo, Martin D. Curran, Malte Pinckert, Surendra Parmar, Ian Goodfellow
EPI_ISL_724216	West of Scotland Specialist Virology Centre, NHSGGC / MRC-University of Glasgow Centre for Virus Research	COVID-19 Genomics UK (COG-UK) Consortium	Ana da Silva Filipe, Natasha Johnson, Kathy Smollett, Daniel Mair, Stephen Carmichael, Alice Broos, Lily Tong, Jenna Nichols, Kyriaki Nomikou; Sarah McDonald; Richard Orton, Joseph Hughes, Sreenu Vattipally, David L Robertson; Alasdair MacLean, Rory Gunson; Sharif Shaaban, Matthew Holden; Rachel Blacow, Guy Mollett, Kathy Li, James Shepherd, Antonia Ho, Emma Thomson
EPI_ISL_724227, EPI_ISL_724230, EPI_ISL_724231, EPI_ISL_724232, EPI_ISL_724233, EPI_ISL_724235, EPI_ISL_724237, EPI_ISL_724238, EPI_ISL_724239, EPI_ISL_724241, EPI_ISL_724242, EPI_ISL_724244, EPI_ISL_724245, EPI_ISL_724247, EPI_ISL_724248, EPI_ISL_724249, EPI_ISL_724250, EPI_ISL_724251, EPI_ISL_724252			
see above	Virology Department, Royal Infirmary of Edinburgh, NHS Lothian / School of Biological Sciences, University of Edinburgh / Institute of Genetics and Molecular Medicine, University of Edinburgh	COVID-19 Genomics UK (COG-UK) Consortium	McHugh M, Dewar R, Rooke S, Gallagher M, Balcaza C, O'Toole Á, Scher E, Hill V, McCrone JT, Colquhoun R, Yu X, Jackson B, Rambaut A, Williams TC, Templeton K
EPI_ISL_724276	University of Exeter	COVID-19 Genomics UK (COG-UK) Consortium	Ben Temperton,Aaron Jeffries,Michelle Michelsen,Joanna Warwick-Dugdale,Audrey Farbos,Robyn Manley,Stephen Michell,Jane Masoli
EPI_ISL_724368, EPI_ISL_724369, EPI_ISL_724372, EPI_ISL_724382, EPI_ISL_724384	Liverpool Clinical Laboratories	COVID-19 Genomics UK (COG-UK) Consortium	Sam Haldenby, Anita Lucaci, Steve Paterson, Julian Hiscox, Alistair Darby, M Almsaud, A Alrezaihi, Muhannad Alruwaili, Stuart D Armstrong, Jones Benjamin, Eleanor G Bentley, Anu Chawla, Jordan J Clark, Angela Cowell, Richard Eccles, Isabel García-Dorival, Matthew Gemmell, Alessandro Gerada, PKF Gilmore, Richard Gregory, Ximeng Han, Catherine Hartley, Margaret Hughes, Miren Iturriza-Gomara, James Johnson, L Luu, Jenifer Manson, Charlotte Nelson, Elaine O'Toole, Cassie Olateju, Rebekah Penrice-Randal , Lucille Rainbow, N.P Randle, Trevor Ian Robinson, Parul Sharma, Ghada T Shawli, James P Stewart, Neil Swainston, Ecaterina Vamos, Joanne Watts, Mark Whitehead
EPI_ISL_724890, EPI_ISL_724892, EPI_ISL_724896, EPI_ISL_724915, EPI_ISL_725021, EPI_ISL_725023, EPI_ISL_725026, EPI_ISL_725027, EPI_ISL_725028	Northumbria University / South Tees Hospitals NHS Foundation Trust / North Cumbria Integrated Care NHS Foundation Trust / North Tees and Hartlepool NHS Foundation Trust / Newcastle Hospitals NHS Foundation Trust	COVID-19 Genomics UK (COG-UK) Consortium	Darren L Smith,Andrew Nelson,Matthew Bashton,Greg R Young,Joshua Loh,John Allan,Mohammad A Tariq,Giles S Holt,Gary Black,Wen C Yew,Lynn Dover,Paul Baker,Steve Liggett,Sarah Essex,Jane Greenaway,Debra Padgett,Clive Graham,Garren Scott,Edward Barton,Emma Swindells,Brendan Payne,Jennifer Collins,Yusri Taha,Gary Eltringham
EPI_ISL_725049, EPI_ISL_725054, EPI_ISL_725073, EPI_ISL_725074, EPI_ISL_725083, EPI_ISL_725107, EPI_ISL_725108, EPI_ISL_725146, EPI_ISL_725300, EPI_ISL_725343, EPI_ISL_725349, EPI_ISL_725351, EPI_ISL_725363, EPI_ISL_725365, EPI_ISL_725439, EPI_ISL_725441, EPI_ISL_725443, EPI_ISL_725448, EPI_ISL_725455, EPI_ISL_725472, EPI_ISL_725482, EPI_ISL_725484, EPI_ISL_725495			
see above	Quadram Institute Bioscience	COVID-19 Genomics UK (COG-UK) Consortium	Dave J. Baker, Gemma L. Kay, Alp Aydin, Thanh Le-Viet, Steven Rudder, Ana P. Tedim, Anastasia Kolyva, Maria Diaz, Leonardo de Oliveira Martins, Nabil-Fareed Alikhan, Lizzie Meadows, Rachael Stanley, Ngozi Elumogo, Muhammed Yasir, Nicholas M. Thomson, Alexander J Trotter, Rachel Gilroy, Samuel Bloomfield, Claire Stuart, Andrew Bell, Reenesh Prakash, Samir Dervisevic, Alison E. Mather, John Wain, Mark Webber, Andrew J. Page, Justin O'Grady
EPI_ISL_726987, EPI_ISL_727037, EPI_ISL_727042, EPI_ISL_727045, EPI_ISL_727047, EPI_ISL_727049, EPI_ISL_727051, EPI_ISL_727053, EPI_ISL_727054, EPI_ISL_727055, EPI_ISL_727057, EPI_ISL_727058, EPI_ISL_727060, EPI_ISL_727061, EPI_ISL_727062, EPI_ISL_727063, EPI_ISL_727065, EPI_ISL_727066, EPI_ISL_727067, EPI_ISL_727069, EPI_ISL_727070, EPI_ISL_727072, EPI_ISL_727073, EPI_ISL_727075, EPI_ISL_727079, EPI_ISL_727080, EPI_ISL_727081, EPI_ISL_727084, EPI_ISL_727085, EPI_ISL_727086, EPI_ISL_727089, EPI_ISL_727090, EPI_ISL_727092, EPI_ISL_727094, EPI_ISL_727121, EPI_ISL_727159, EPI_ISL_727270, EPI_ISL_727284, EPI_ISL_727301, EPI_ISL_727303, EPI_ISL_727309, EPI_ISL_727311, EPI_ISL_727321, EPI_ISL_727324, EPI_ISL_727326, EPI_ISL_727331, EPI_ISL_727333, EPI_ISL_727334, EPI_ISL_727336, EPI_ISL_727338, EPI_ISL_727339, EPI_ISL_727340, EPI_ISL_727343, EPI_ISL_727349, EPI_ISL_727351, EPI_ISL_727353, EPI_ISL_727356, EPI_ISL_727365, EPI_ISL_727367, EPI_ISL_727380, EPI_ISL_727383, EPI_ISL_727384, EPI_ISL_727387, EPI_ISL_727390, EPI_ISL_727395, EPI_ISL_727402, EPI_ISL_727419, EPI_ISL_727422, EPI_ISL_727428, EPI_ISL_727508, EPI_ISL_727510, EPI_ISL_727525, EPI_ISL_727526, EPI_ISL_727527, EPI_ISL_727529, EPI_ISL_727530, EPI_ISL_727534, EPI_ISL_727535, EPI_ISL_727539, EPI_ISL_727542, EPI_ISL_727543, EPI_ISL_727544, EPI_ISL_727546, EPI_ISL_727553, EPI_ISL_727554, EPI_ISL_727555, EPI_ISL_727556, EPI_ISL_727563, EPI_ISL_727567, EPI_ISL_727568, EPI_ISL_727570, EPI_ISL_727572, EPI_ISL_727573, EPI_ISL_727574, EPI_ISL_727575, EPI_ISL_727577, EPI_ISL_727578, EPI_ISL_727579, EPI_ISL_727583, EPI_ISL_727587, EPI_ISL_727588, EPI_ISL_727593, EPI_ISL_727594, EPI_ISL_727595, EPI_ISL_727600, EPI_ISL_727601, EPI_ISL_727602, EPI_ISL_727606, EPI_ISL_727616, EPI_ISL_727620, EPI_ISL_727621, EPI_ISL_727622, EPI_ISL_727629, EPI_ISL_727635, EPI_ISL_727636, EPI_ISL_727640, EPI_ISL_727643, EPI_ISL_727644, EPI_ISL_727649, EPI_ISL_727650, EPI_ISL_727651, EPI_ISL_727653, EPI_ISL_727656, EPI_ISL_727657, EPI_ISL_727661, EPI_ISL_727673, EPI_ISL_727674, EPI_ISL_727683, EPI_ISL_727688			
see above	Wales Specialist Virology Centre Sequencing lab: Pathogen Genomics Unit	COVID-19 Genomics UK (COG-UK) Consortium	Catherine Moore, Johnathan Evans, Laura Gifford, Malorie Perry, Simon Cottrell, Angela Marchbank, Alec Birchley, Alexander Adams, Amy Gaskin, Bree Gatica-Wilcox, Jason Coombes, Joel Southgate, Lauren Gilbert, Lee Graham, Nicole Pacchiarini, Sara Kumziene-Summerhayes, Sarah Taylor, Sophie Jones, Sara Rey, Matthew Bull, Joanne Watkins, Sally Corden, Tom Connor
EPI_ISL_727866, EPI_ISL_727911	Virology Department, Sheffield Teaching Hospitals NHS Foundation Trust/Department of Infection, Immunity and Cardiovascular Disease, The Medical School, University of Sheffield	COVID-19 Genomics UK (COG-UK) Consortium	Thushan de Silva, Matthew Parker, Nikki Smith, Adri Angyal, Rebecca Brown, Luke Green, Rachel Tucker, Paul Parsons, Danielle Groves, Katie Johnson, Laura Carrilero, Alex Keeley, Dave Partridge, Matthew Wyles, Benjamin Lindsey, Mehmet Yavuz, Mohammad Raza, Cariad Evans
EPI_ISL_728089	University of Wisconsin-Madison AIDS Vaccine Research Laboratories	University of Wisconsin-Madison AIDS Vaccine Research Laboratories	Gage Moreno, Katarina Braun, et al. AIDS Vaccine Research Laboratories
EPI_ISL_728191	National Public Health Laboratory, National Centre for Infectious Diseases	National Public Health Laboratory, National Centre for Infectious Diseases	Tze Minn Mak, Sophie Octavia, Zhenyang Zhou, Lin Cui, Raymond Tzer Pin Lin
EPI_ISL_728282	National Institute for Infectious Diseases, INMI, "L. Spallanzani" IRCCS	National Institute for Infectious Diseases, INMI, "L. Spallanzani" IRCCS	M. Rueca, C.E.M Gruber, B Bartolini, F Messina, E Giombini, A Di Caro, MR Capobianchi

EPI_ISL_728285	National Institute for Infectious Diseases, INMI, "L. Spallanzani" IRCCS	National Institute for Infectious Diseases, INMI, "L. Spallanzani" IRCCS	F Messina, E Giombini, M Rueca, B Bartolini, C.E.M Gruber, MR Capobianchi, A Di Caro
EPI_ISL_728299	Lighthouse Lab in Cambridge	Wellcome Sanger Institute for the COVID-19 Genomics UK (COG-UK) Consortium	Rob Howes, The Lighthouse Lab in Cambridge and Alex Alderton, Roberto Amato, Sonia Goncalves, Ewan Harrison, David K. Jackson, Ian Johnston, Dominic Kwiatkowski, Cordelia Langford, John Sillitoe on behalf of the Wellcome Sanger Institute COVID-19 Surveillance Team
EPI_ISL_728331, EPI_ISL_728333, EPI_ISL_728335	Jena University Hospital, Institute for Infectious Diseases and Infection Control	Institute of infectious medicine & hospital hygiene, CaSe-Group	Spott, Riccardo; Marquet, Mike; Pletz, Matthias W.; Brandt, Christian
EPI_ISL_728566, EPI_ISL_728567, EPI_ISL_728568, EPI_ISL_728569, EPI_ISL_728570, EPI_ISL_728571, EPI_ISL_728572, EPI_ISL_728573, EPI_ISL_728574, EPI_ISL_728575, EPI_ISL_728576, EPI_ISL_728577, EPI_ISL_728578, EPI_ISL_728579, EPI_ISL_728580, EPI_ISL_728581, EPI_ISL_728582, EPI_ISL_728583, EPI_ISL_728584, EPI_ISL_728585, EPI_ISL_728586, EPI_ISL_728587, EPI_ISL_728588, EPI_ISL_728589, EPI_ISL_728590, EPI_ISL_728591, EPI_ISL_728592, EPI_ISL_728593, EPI_ISL_728594, EPI_ISL_728595, EPI_ISL_728596, EPI_ISL_728597, EPI_ISL_728598, EPI_ISL_728599, EPI_ISL_728600, EPI_ISL_728601, EPI_ISL_728602, EPI_ISL_728603, EPI_ISL_728604, EPI_ISL_728605, EPI_ISL_728606, EPI_ISL_728607, EPI_ISL_728608, EPI_ISL_728609, EPI_ISL_728610, EPI_ISL_728611, EPI_ISL_728612, EPI_ISL_728613, EPI_ISL_728614, EPI_ISL_728615, EPI_ISL_728616, EPI_ISL_728617, EPI_ISL_728618, EPI_ISL_728619, EPI_ISL_728620, EPI_ISL_728621, EPI_ISL_728622, EPI_ISL_728623, EPI_ISL_728624, EPI_ISL_728625, EPI_ISL_728626, EPI_ISL_728627, EPI_ISL_728628, EPI_ISL_728629, EPI_ISL_728630, EPI_ISL_728631, EPI_ISL_728632, EPI_ISL_728633, EPI_ISL_728634, EPI_ISL_728635, EPI_ISL_728636, EPI_ISL_728637, EPI_ISL_728642, EPI_ISL_728644, EPI_ISL_728645, EPI_ISL_728646, EPI_ISL_728647, EPI_ISL_728648, EPI_ISL_728649, EPI_ISL_728650, EPI_ISL_728651, EPI_ISL_728652, EPI_ISL_728653, EPI_ISL_728654, EPI_ISL_728655, EPI_ISL_728656, EPI_ISL_728657, EPI_ISL_728658, EPI_ISL_728659, EPI_ISL_728660, EPI_ISL_728661, EPI_ISL_728662, EPI_ISL_728663, EPI_ISL_728664, EPI_ISL_728665, EPI_ISL_728666, EPI_ISL_728667, EPI_ISL_728689, EPI_ISL_728690, EPI_ISL_728691, EPI_ISL_728692, EPI_ISL_728693, EPI_ISL_728694, EPI_ISL_728695, EPI_ISL_728697, EPI_ISL_728698, EPI_ISL_728699, EPI_ISL_728700, EPI_ISL_728701, EPI_ISL_728702, EPI_ISL_728703, EPI_ISL_728721, EPI_ISL_728722, EPI_ISL_728738, EPI_ISL_728739, EPI_ISL_728740, EPI_ISL_728741, EPI_ISL_728742, EPI_ISL_728743, EPI_ISL_728744, EPI_ISL_728745, EPI_ISL_728746, EPI_ISL_728747, EPI_ISL_728763			
see above	Dutch COVID-19 response team	National Institute for Public Health and the Environment (RIVM)	Adam Meijer, Harry Vennema, Jeroen Cremer, Sharon van den Brink, Bas van der Veer, AnneMarie van den Brandt, Florian Zwagemaker, Dennis Schmitz, Chantal Reusken, on behalf of the national COVID-19 response team
EPI_ISL_729053	Viollier AG	Department of Biosystems Science and Engineering, ETH Zürich	Christian Beisel, Sarah Nadeau, Chaoran Chen, Ivan Topolsky, Pedro Ferreira, Philipp Jablonski, Susana Posada-Céspedes, Tobias Schär, Ina Nissen, Natascha Santacroce, Elodie Burcklen, Christiane Beckmann, Maurice Redondo, Olivier Kobel, Christoph Noppen, Sophie Seidel, Noemie Santamaria de Souza, Niko Beerenwinkel, Tanja Stadler
EPI_ISL_729107	Viollier AG	Department of Biosystems Science and Engineering, ETH Zürich	Chaoran Chen, Sarah Nadeau, Catharine Aquino, Ivan Topolsky, Pedro Ferreira, Philipp Jablonski, Susana Posada-Céspedes, Andreia Cabral de Gouvea, Maria Domenica Moccia, Simon Grüter, Timothy Sykes, Lennart Opitz, Ralph Schlapbach, Christiane Beckmann, Maurice Redondo, Olivier Kobel, Christoph Noppen, Sophie Seidel, Noemie Santamaria de Souza, Niko Beerenwinkel, Tanja Stadler
EPI_ISL_729152, EPI_ISL_729154, EPI_ISL_729156, EPI_ISL_729162	Viollier AG	Department of Biosystems Science and Engineering, ETH Zürich	Christian Beisel, Sarah Nadeau, Chaoran Chen, Ivan Topolsky, Pedro Ferreira, Philipp Jablonski, Susana Posada-Céspedes, Tobias Schär, Ina Nissen, Natascha Santacroce, Elodie Burcklen, Christiane Beckmann, Maurice Redondo, Olivier Kobel, Christoph Noppen, Sophie Seidel, Noemie Santamaria de Souza, Niko Beerenwinkel, Tanja Stadler
EPI_ISL_729416, EPI_ISL_729449, EPI_ISL_729450, EPI_ISL_729451, EPI_ISL_729453, EPI_ISL_729454, EPI_ISL_729455, EPI_ISL_729457, EPI_ISL_729458, EPI_ISL_729463, EPI_ISL_729697, EPI_ISL_729698, EPI_ISL_729699, EPI_ISL_729700, EPI_ISL_729701, EPI_ISL_729702, EPI_ISL_729703, EPI_ISL_729705, EPI_ISL_729706, EPI_ISL_729709, EPI_ISL_729710, EPI_ISL_729711, EPI_ISL_729712, EPI_ISL_729713, EPI_ISL_729714, EPI_ISL_729716, EPI_ISL_729719, EPI_ISL_729721, EPI_ISL_729724, EPI_ISL_729725, EPI_ISL_729726, EPI_ISL_729727			
see above	A. Krumbholz, Labor Dr. Krause und Kollegen MVZ GmbH, Kiel	Charité Universitätsmedizin Berlin, Institut für Virologie	Victor M Corman, Barbara Mühlemann, Jörn Beheim-Schwarzbach, Talitha Veith, Julia Schneider, Terry Jones, Christian Drosten
EPI_ISL_730291, EPI_ISL_730299, EPI_ISL_730307, EPI_ISL_730312, EPI_ISL_730313, EPI_ISL_730315, EPI_ISL_730340, EPI_ISL_730353, EPI_ISL_730354, EPI_ISL_730355, EPI_ISL_730358, EPI_ISL_730359, EPI_ISL_730361, EPI_ISL_730362, EPI_ISL_730363, EPI_ISL_730365, EPI_ISL_730366, EPI_ISL_730369			
see above	San Diego County Public Health Laboratory	Andersen lab at Scripps Research	SEARCH Alliance San Diego with Tracy Basler, Jovan Shephard, Brett Austin
EPI_ISL_730621	Limmattal Hospital	Institute of Medical Virology, University of Zurich	Stefan Schmutz, Verena Kufner, Maryam Zaheri, Gabriela Ziltener, Jürg Böni, Michael Huber, Alexandra Trkola
EPI_ISL_730622, EPI_ISL_730626, EPI_ISL_730627	Männedorf Hospital	Institute of Medical Virology, University of Zurich	Stefan Schmutz, Verena Kufner, Maryam Zaheri, Gabriela Ziltener, Jürg Böni, Michael Huber, Alexandra Trkola
EPI_ISL_730630	Limmattal Hospital	Institute of Medical Virology, University of Zurich	Stefan Schmutz, Verena Kufner, Maryam Zaheri, Gabriela Ziltener, Jürg Böni, Michael Huber, Alexandra Trkola
EPI_ISL_730631	Männedorf Hospital	Institute of Medical Virology, University of Zurich	Stefan Schmutz, Verena Kufner, Maryam Zaheri, Gabriela Ziltener, Jürg Böni, Michael Huber, Alexandra Trkola
EPI_ISL_730634	University Hospital Zürich	Institute of Medical Virology, University of Zurich	Stefan Schmutz, Verena Kufner, Maryam Zaheri, Gabriela Ziltener, Jürg Böni, Michael Huber, Alexandra Trkola
EPI_ISL_730635	Limmattal Hospital	Institute of Medical Virology, University of Zurich	Stefan Schmutz, Verena Kufner, Maryam Zaheri, Gabriela Ziltener, Jürg Böni, Michael Huber, Alexandra Trkola
EPI_ISL_730637, EPI_ISL_730638, EPI_ISL_730639	Männedorf Hospital	Institute of Medical Virology, University of Zurich	Stefan Schmutz, Verena Kufner, Maryam Zaheri, Gabriela Ziltener, Jürg Böni, Michael Huber, Alexandra Trkola
EPI_ISL_730654	Lighthouse Lab in Alderley Park	Wellcome Sanger Institute for the COVID-19 Genomics UK (COG-UK) Consortium	Jacquelyn Wynn, Mairead Hyland, The Lighthouse Lab in Alderley Park and Alex Alderton, Roberto Amato, Sonia Goncalves, Ewan Harrison, David K. Jackson, Ian Johnston, Dominic Kwiatkowski, Cordelia Langford, John Sillitoe on behalf of the Wellcome Sanger Institute COVID-19 Surveillance Team
EPI_ISL_730656, EPI_ISL_730657, EPI_ISL_730659	Lighthouse Lab in Glasgow	Wellcome Sanger Institute for the COVID-19 Genomics UK (COG-UK) Consortium	Harper VanSteenhouse, Yumi Kasai, David Gray, Carol Clugston, Anna Dominiczak and Alex Alderton, Roberto Amato, Sonia Goncalves, Ewan Harrison, David K. Jackson, Ian Johnston, Dominic Kwiatkowski, Cordelia Langford, John Sillitoe on behalf of the Wellcome Sanger Institute COVID-19 Surveillance Team
EPI_ISL_730660, EPI_ISL_730662	Lighthouse Lab in Alderley Park	Wellcome Sanger Institute for the COVID-19 Genomics UK (COG-UK) Consortium	Jacquelyn Wynn, Mairead Hyland, The Lighthouse Lab in Alderley Park and Alex Alderton, Roberto Amato, Sonia Goncalves, Ewan Harrison, David K. Jackson, Ian Johnston, Dominic Kwiatkowski, Cordelia Langford, John Sillitoe on behalf of the Wellcome Sanger Institute COVID-19 Surveillance Team
EPI_ISL_730664	Lighthouse Lab in Glasgow	Wellcome Sanger Institute for the COVID-19 Genomics UK (COG-UK) Consortium	Harper VanSteenhouse, Yumi Kasai, David Gray, Carol Clugston, Anna Dominiczak and Alex Alderton, Roberto Amato, Sonia Goncalves, Ewan Harrison, David K. Jackson, Ian Johnston, Dominic Kwiatkowski, Cordelia Langford, John Sillitoe on behalf of the Wellcome Sanger Institute COVID-19 Surveillance Team
EPI_ISL_730665, EPI_ISL_730669, EPI_ISL_730670, EPI_ISL_730671, EPI_ISL_730672	Lighthouse Lab in Alderley Park	Wellcome Sanger Institute for the COVID-19 Genomics UK (COG-UK) Consortium	Jacquelyn Wynn, Mairead Hyland, The Lighthouse Lab in Alderley Park and Alex Alderton, Roberto Amato, Sonia Goncalves, Ewan Harrison, David K. Jackson, Ian Johnston, Dominic Kwiatkowski, Cordelia Langford, John Sillitoe on behalf of the Wellcome Sanger Institute COVID-19 Surveillance Team
EPI_ISL_730673	Lighthouse Lab in Glasgow	Wellcome Sanger Institute for the COVID-19 Genomics UK (COG-UK) Consortium	Harper VanSteenhouse, Yumi Kasai, David Gray, Carol Clugston, Anna Dominiczak and Alex Alderton, Roberto Amato, Sonia Goncalves, Ewan Harrison, David K. Jackson, Ian Johnston, Dominic Kwiatkowski, Cordelia Langford, John Sillitoe on behalf of the Wellcome Sanger Institute COVID-19 Surveillance Team
EPI_ISL_730674	Lighthouse Lab in Alderley Park	Wellcome Sanger Institute for the COVID-19 Genomics UK (COG-UK) Consortium	Jacquelyn Wynn, Mairead Hyland, The Lighthouse Lab in Alderley Park and Alex Alderton, Roberto Amato, Sonia Goncalves, Ewan Harrison, David K. Jackson, Ian Johnston, Dominic Kwiatkowski, Cordelia Langford, John Sillitoe on behalf of the Wellcome Sanger Institute COVID-19 Surveillance Team
EPI_ISL_730676	Lighthouse Lab in Glasgow	Wellcome Sanger Institute for the COVID-19 Genomics UK (COG-UK) Consortium	Harper VanSteenhouse, Yumi Kasai, David Gray, Carol Clugston, Anna Dominiczak and Alex Alderton, Roberto Amato, Sonia Goncalves, Ewan Harrison, David K. Jackson, Ian Johnston, Dominic Kwiatkowski, Cordelia Langford, John Sillitoe on behalf of the Wellcome Sanger Institute COVID-19 Surveillance Team
EPI_ISL_730677	Lighthouse Lab in Alderley Park	Wellcome Sanger Institute for the COVID-19 Genomics UK (COG-UK) Consortium	Jacquelyn Wynn, Mairead Hyland, The Lighthouse Lab in Alderley Park and Alex Alderton, Roberto Amato, Sonia Goncalves, Ewan Harrison, David K. Jackson, Ian Johnston, Dominic Kwiatkowski, Cordelia Langford, John Sillitoe on behalf of the Wellcome Sanger Institute COVID-19 Surveillance Team
EPI_ISL_730678	Lighthouse Lab in Glasgow	Wellcome Sanger Institute for the COVID-19 Genomics UK (COG-UK) Consortium	Harper VanSteenhouse, Yumi Kasai, David Gray, Carol Clugston, Anna Dominiczak and Alex Alderton, Roberto Amato, Sonia Goncalves, Ewan Harrison, David K. Jackson, Ian Johnston, Dominic Kwiatkowski, Cordelia Langford, John Sillitoe on behalf of the Wellcome Sanger Institute COVID-19 Surveillance Team
EPI_ISL_730680, EPI_ISL_730681, EPI_ISL_730682, EPI_ISL_730683, EPI_ISL_730684	Lighthouse Lab in Alderley Park	Wellcome Sanger Institute for the COVID-19 Genomics UK (COG-UK) Consortium	Jacquelyn Wynn, Mairead Hyland, The Lighthouse Lab in Alderley Park and Alex Alderton, Roberto Amato, Sonia Goncalves, Ewan Harrison, David K. Jackson, Ian Johnston, Dominic Kwiatkowski, Cordelia Langford, John Sillitoe on behalf of the Wellcome Sanger Institute COVID-19 Surveillance Team
EPI_ISL_730685	Lighthouse Lab in Glasgow	Wellcome Sanger Institute for the COVID-19 Genomics UK (COG-UK) Consortium	Harper VanSteenhouse, Yumi Kasai, David Gray, Carol Clugston, Anna Dominiczak and Alex Alderton, Roberto Amato, Sonia Goncalves, Ewan Harrison, David K. Jackson, Ian Johnston, Dominic Kwiatkowski, Cordelia Langford, John Sillitoe on behalf of the Wellcome Sanger Institute COVID-19 Surveillance Team
EPI_ISL_730686, EPI_ISL_730687,	Lighthouse Lab in Alderley Park	Wellcome Sanger Institute for the COVID-19 Genomics UK	Jacquelyn Wynn, Mairead Hyland, The Lighthouse Lab in Alderley Park and Alex Alderton, Roberto Amato, Sonia Goncalves, Ewan Harrison, David K.

EPI_ISL_730689, EPI_ISL_730690, EPI_ISL_730692, EPI_ISL_730693, EPI_ISL_730696		(COG-UK) Consortium	Jackson, Ian Johnston, Dominic Kwiatkowski, Cordelia Langford, John Sillitoe on behalf of the Wellcome Sanger Institute COVID-19 Surveillance Team
EPI_ISL_730697	Lighthouse Lab in Glasgow	Wellcome Sanger Institute for the COVID-19 Genomics UK (COG-UK) Consortium	Harper VanSteenhouse, Yumi Kasai, David Gray, Carol Clugston, Anna Dominiczak and Alex Alderton, Roberto Amato, Sonia Goncalves, Ewan Harrison, David K. Jackson, Ian Johnston, Dominic Kwiatkowski, Cordelia Langford, John Sillitoe on behalf of the Wellcome Sanger Institute COVID-19 Surveillance Team
EPI_ISL_730698, EPI_ISL_730700, EPI_ISL_730701, EPI_ISL_730702, EPI_ISL_730704	Lighthouse Lab in Alderley Park	Wellcome Sanger Institute for the COVID-19 Genomics UK (COG-UK) Consortium	Jacquelyn Wynn, Mairead Hyland, The Lighthouse Lab in Alderley Park and Alex Alderton, Roberto Amato, Sonia Goncalves, Ewan Harrison, David K. Jackson, Ian Johnston, Dominic Kwiatkowski, Cordelia Langford, John Sillitoe on behalf of the Wellcome Sanger Institute COVID-19 Surveillance Team
EPI_ISL_730705	Lighthouse Lab in Glasgow	Wellcome Sanger Institute for the COVID-19 Genomics UK (COG-UK) Consortium	Harper VanSteenhouse, Yumi Kasai, David Gray, Carol Clugston, Anna Dominiczak and Alex Alderton, Roberto Amato, Sonia Goncalves, Ewan Harrison, David K. Jackson, Ian Johnston, Dominic Kwiatkowski, Cordelia Langford, John Sillitoe on behalf of the Wellcome Sanger Institute COVID-19 Surveillance Team
EPI_ISL_730706, EPI_ISL_730707, EPI_ISL_730708	Lighthouse Lab in Alderley Park	Wellcome Sanger Institute for the COVID-19 Genomics UK (COG-UK) Consortium	Jacquelyn Wynn, Mairead Hyland, The Lighthouse Lab in Alderley Park and Alex Alderton, Roberto Amato, Sonia Goncalves, Ewan Harrison, David K. Jackson, Ian Johnston, Dominic Kwiatkowski, Cordelia Langford, John Sillitoe on behalf of the Wellcome Sanger Institute COVID-19 Surveillance Team
EPI_ISL_730710	Lighthouse Lab in Glasgow	Wellcome Sanger Institute for the COVID-19 Genomics UK (COG-UK) Consortium	Harper VanSteenhouse, Yumi Kasai, David Gray, Carol Clugston, Anna Dominiczak and Alex Alderton, Roberto Amato, Sonia Goncalves, Ewan Harrison, David K. Jackson, Ian Johnston, Dominic Kwiatkowski, Cordelia Langford, John Sillitoe on behalf of the Wellcome Sanger Institute COVID-19 Surveillance Team
EPI_ISL_730711, EPI_ISL_730714, EPI_ISL_730715, EPI_ISL_730716, EPI_ISL_730717	Lighthouse Lab in Alderley Park	Wellcome Sanger Institute for the COVID-19 Genomics UK (COG-UK) Consortium	Jacquelyn Wynn, Mairead Hyland, The Lighthouse Lab in Alderley Park and Alex Alderton, Roberto Amato, Sonia Goncalves, Ewan Harrison, David K. Jackson, Ian Johnston, Dominic Kwiatkowski, Cordelia Langford, John Sillitoe on behalf of the Wellcome Sanger Institute COVID-19 Surveillance Team
EPI_ISL_730718	Lighthouse Lab in Glasgow	Wellcome Sanger Institute for the COVID-19 Genomics UK (COG-UK) Consortium	Harper VanSteenhouse, Yumi Kasai, David Gray, Carol Clugston, Anna Dominiczak and Alex Alderton, Roberto Amato, Sonia Goncalves, Ewan Harrison, David K. Jackson, Ian Johnston, Dominic Kwiatkowski, Cordelia Langford, John Sillitoe on behalf of the Wellcome Sanger Institute COVID-19 Surveillance Team
EPI_ISL_730720, EPI_ISL_730721, EPI_ISL_730722, EPI_ISL_730724, EPI_ISL_730725, EPI_ISL_730726, EPI_ISL_730727, EPI_ISL_730728, EPI_ISL_730729, EPI_ISL_730730, EPI_ISL_730734, EPI_ISL_730736			
see above	Lighthouse Lab in Alderley Park	Wellcome Sanger Institute for the COVID-19 Genomics UK (COG-UK) Consortium	Jacquelyn Wynn, Mairead Hyland, The Lighthouse Lab in Alderley Park and Alex Alderton, Roberto Amato, Sonia Goncalves, Ewan Harrison, David K. Jackson, Ian Johnston, Dominic Kwiatkowski, Cordelia Langford, John Sillitoe on behalf of the Wellcome Sanger Institute COVID-19 Surveillance Team
EPI_ISL_730737	Lighthouse Lab in Glasgow	Wellcome Sanger Institute for the COVID-19 Genomics UK (COG-UK) Consortium	Harper VanSteenhouse, Yumi Kasai, David Gray, Carol Clugston, Anna Dominiczak and Alex Alderton, Roberto Amato, Sonia Goncalves, Ewan Harrison, David K. Jackson, Ian Johnston, Dominic Kwiatkowski, Cordelia Langford, John Sillitoe on behalf of the Wellcome Sanger Institute COVID-19 Surveillance Team
EPI_ISL_730738, EPI_ISL_730739, EPI_ISL_730740, EPI_ISL_730741, EPI_ISL_730743, EPI_ISL_730744, EPI_ISL_730745, EPI_ISL_730746, EPI_ISL_730747, EPI_ISL_730748, EPI_ISL_730749, EPI_ISL_730751, EPI_ISL_730753, EPI_ISL_730754, EPI_ISL_730755, EPI_ISL_730756, EPI_ISL_730757, EPI_ISL_730758, EPI_ISL_730760, EPI_ISL_730761, EPI_ISL_730764			
see above	Lighthouse Lab in Alderley Park	Wellcome Sanger Institute for the COVID-19 Genomics UK (COG-UK) Consortium	Jacquelyn Wynn, Mairead Hyland, The Lighthouse Lab in Alderley Park and Alex Alderton, Roberto Amato, Sonia Goncalves, Ewan Harrison, David K. Jackson, Ian Johnston, Dominic Kwiatkowski, Cordelia Langford, John Sillitoe on behalf of the Wellcome Sanger Institute COVID-19 Surveillance Team
EPI_ISL_730765	Lighthouse Lab in Glasgow	Wellcome Sanger Institute for the COVID-19 Genomics UK (COG-UK) Consortium	Harper VanSteenhouse, Yumi Kasai, David Gray, Carol Clugston, Anna Dominiczak and Alex Alderton, Roberto Amato, Sonia Goncalves, Ewan Harrison, David K. Jackson, Ian Johnston, Dominic Kwiatkowski, Cordelia Langford, John Sillitoe on behalf of the Wellcome Sanger Institute COVID-19 Surveillance Team
EPI_ISL_730767, EPI_ISL_730768, EPI_ISL_730769, EPI_ISL_730770, EPI_ISL_730772, EPI_ISL_730773, EPI_ISL_730774, EPI_ISL_730776	Lighthouse Lab in Alderley Park	Wellcome Sanger Institute for the COVID-19 Genomics UK (COG-UK) Consortium	Jacquelyn Wynn, Mairead Hyland, The Lighthouse Lab in Alderley Park and Alex Alderton, Roberto Amato, Sonia Goncalves, Ewan Harrison, David K. Jackson, Ian Johnston, Dominic Kwiatkowski, Cordelia Langford, John Sillitoe on behalf of the Wellcome Sanger Institute COVID-19 Surveillance Team
EPI_ISL_730777	Lighthouse Lab in Glasgow	Wellcome Sanger Institute for the COVID-19 Genomics UK (COG-UK) Consortium	Harper VanSteenhouse, Yumi Kasai, David Gray, Carol Clugston, Anna Dominiczak and Alex Alderton, Roberto Amato, Sonia Goncalves, Ewan Harrison, David K. Jackson, Ian Johnston, Dominic Kwiatkowski, Cordelia Langford, John Sillitoe on behalf of the Wellcome Sanger Institute COVID-19 Surveillance Team
EPI_ISL_730779	Lighthouse Lab in Alderley Park	Wellcome Sanger Institute for the COVID-19 Genomics UK (COG-UK) Consortium	Jacquelyn Wynn, Mairead Hyland, The Lighthouse Lab in Alderley Park and Alex Alderton, Roberto Amato, Sonia Goncalves, Ewan Harrison, David K. Jackson, Ian Johnston, Dominic Kwiatkowski, Cordelia Langford, John Sillitoe on behalf of the Wellcome Sanger Institute COVID-19 Surveillance Team
EPI_ISL_730780	Lighthouse Lab in Glasgow	Wellcome Sanger Institute for the COVID-19 Genomics UK (COG-UK) Consortium	Harper VanSteenhouse, Yumi Kasai, David Gray, Carol Clugston, Anna Dominiczak and Alex Alderton, Roberto Amato, Sonia Goncalves, Ewan Harrison, David K. Jackson, Ian Johnston, Dominic Kwiatkowski, Cordelia Langford, John Sillitoe on behalf of the Wellcome Sanger Institute COVID-19 Surveillance Team
EPI_ISL_730782, EPI_ISL_730783, EPI_ISL_730787, EPI_ISL_730788	Lighthouse Lab in Alderley Park	Wellcome Sanger Institute for the COVID-19 Genomics UK (COG-UK) Consortium	Jacquelyn Wynn, Mairead Hyland, The Lighthouse Lab in Alderley Park and Alex Alderton, Roberto Amato, Sonia Goncalves, Ewan Harrison, David K. Jackson, Ian Johnston, Dominic Kwiatkowski, Cordelia Langford, John Sillitoe on behalf of the Wellcome Sanger Institute COVID-19 Surveillance Team
EPI_ISL_730789	Lighthouse Lab in Glasgow	Wellcome Sanger Institute for the COVID-19 Genomics UK (COG-UK) Consortium	Harper VanSteenhouse, Yumi Kasai, David Gray, Carol Clugston, Anna Dominiczak and Alex Alderton, Roberto Amato, Sonia Goncalves, Ewan Harrison, David K. Jackson, Ian Johnston, Dominic Kwiatkowski, Cordelia Langford, John Sillitoe on behalf of the Wellcome Sanger Institute COVID-19 Surveillance Team
EPI_ISL_730790, EPI_ISL_730791, EPI_ISL_730792	Lighthouse Lab in Alderley Park	Wellcome Sanger Institute for the COVID-19 Genomics UK (COG-UK) Consortium	Jacquelyn Wynn, Mairead Hyland, The Lighthouse Lab in Alderley Park and Alex Alderton, Roberto Amato, Sonia Goncalves, Ewan Harrison, David K. Jackson, Ian Johnston, Dominic Kwiatkowski, Cordelia Langford, John Sillitoe on behalf of the Wellcome Sanger Institute COVID-19 Surveillance Team
EPI_ISL_730793, EPI_ISL_730795, EPI_ISL_730796	Lighthouse Lab in Glasgow	Wellcome Sanger Institute for the COVID-19 Genomics UK (COG-UK) Consortium	Harper VanSteenhouse, Yumi Kasai, David Gray, Carol Clugston, Anna Dominiczak and Alex Alderton, Roberto Amato, Sonia Goncalves, Ewan Harrison, David K. Jackson, Ian Johnston, Dominic Kwiatkowski, Cordelia Langford, John Sillitoe on behalf of the Wellcome Sanger Institute COVID-19 Surveillance Team
EPI_ISL_730797, EPI_ISL_730798, EPI_ISL_730800	Lighthouse Lab in Alderley Park	Wellcome Sanger Institute for the COVID-19 Genomics UK (COG-UK) Consortium	Jacquelyn Wynn, Mairead Hyland, The Lighthouse Lab in Alderley Park and Alex Alderton, Roberto Amato, Sonia Goncalves, Ewan Harrison, David K. Jackson, Ian Johnston, Dominic Kwiatkowski, Cordelia Langford, John Sillitoe on behalf of the Wellcome Sanger Institute COVID-19 Surveillance Team
EPI_ISL_730801, EPI_ISL_730802, EPI_ISL_730805, EPI_ISL_730806	Lighthouse Lab in Glasgow	Wellcome Sanger Institute for the COVID-19 Genomics UK (COG-UK) Consortium	Harper VanSteenhouse, Yumi Kasai, David Gray, Carol Clugston, Anna Dominiczak and Alex Alderton, Roberto Amato, Sonia Goncalves, Ewan Harrison, David K. Jackson, Ian Johnston, Dominic Kwiatkowski, Cordelia Langford, John Sillitoe on behalf of the Wellcome Sanger Institute COVID-19 Surveillance Team
EPI_ISL_730807	Lighthouse Lab in Alderley Park	Wellcome Sanger Institute for the COVID-19 Genomics UK (COG-UK) Consortium	Jacquelyn Wynn, Mairead Hyland, The Lighthouse Lab in Alderley Park and Alex Alderton, Roberto Amato, Sonia Goncalves, Ewan Harrison, David K. Jackson, Ian Johnston, Dominic Kwiatkowski, Cordelia Langford, John Sillitoe on behalf of the Wellcome Sanger Institute COVID-19 Surveillance Team
EPI_ISL_730808, EPI_ISL_730809	Lighthouse Lab in Glasgow	Wellcome Sanger Institute for the COVID-19 Genomics UK (COG-UK) Consortium	Harper VanSteenhouse, Yumi Kasai, David Gray, Carol Clugston, Anna Dominiczak and Alex Alderton, Roberto Amato, Sonia Goncalves, Ewan Harrison, David K. Jackson, Ian Johnston, Dominic Kwiatkowski, Cordelia Langford, John Sillitoe on behalf of the Wellcome Sanger Institute COVID-19 Surveillance Team
EPI_ISL_730810, EPI_ISL_730811, EPI_ISL_730812, EPI_ISL_730814	Lighthouse Lab in Alderley Park	Wellcome Sanger Institute for the COVID-19 Genomics UK (COG-UK) Consortium	Jacquelyn Wynn, Mairead Hyland, The Lighthouse Lab in Alderley Park and Alex Alderton, Roberto Amato, Sonia Goncalves, Ewan Harrison, David K. Jackson, Ian Johnston, Dominic Kwiatkowski, Cordelia Langford, John Sillitoe on behalf of the Wellcome Sanger Institute COVID-19 Surveillance Team
EPI_ISL_730815	Lighthouse Lab in Glasgow	Wellcome Sanger Institute for the COVID-19 Genomics UK (COG-UK) Consortium	Harper VanSteenhouse, Yumi Kasai, David Gray, Carol Clugston, Anna Dominiczak and Alex Alderton, Roberto Amato, Sonia Goncalves, Ewan Harrison, David K. Jackson, Ian Johnston, Dominic Kwiatkowski, Cordelia Langford, John Sillitoe on behalf of the Wellcome Sanger Institute COVID-19 Surveillance Team

EPI_ISL_730817, EPI_ISL_730819, EPI_ISL_730821, EPI_ISL_730822, EPI_ISL_730823	Lighthouse Lab in Alderley Park	Wellcome Sanger Institute for the COVID-19 Genomics UK (COG-UK) Consortium	Jacquelyn Wynn, Mairead Hyland, The Lighthouse Lab in Alderley Park and Alex Alderton, Roberto Amato, Sonia Goncalves, Ewan Harrison, David K. Jackson, Ian Johnston, Dominic Kwiatkowski, Cordelia Langford, John Sillitoe on behalf of the Wellcome Sanger Institute COVID-19 Surveillance Team
EPI_ISL_730824	Lighthouse Lab in Glasgow	Wellcome Sanger Institute for the COVID-19 Genomics UK (COG-UK) Consortium	Harper VanSteenhouse, Yumi Kasai, David Gray, Carol Clugston, Anna Dominiczak and Alex Alderton, Roberto Amato, Sonia Goncalves, Ewan Harrison, David K. Jackson, Ian Johnston, Dominic Kwiatkowski, Cordelia Langford, John Sillitoe on behalf of the Wellcome Sanger Institute COVID-19 Surveillance Team
EPI_ISL_730825, EPI_ISL_730829, EPI_ISL_730830	Lighthouse Lab in Alderley Park	Wellcome Sanger Institute for the COVID-19 Genomics UK (COG-UK) Consortium	Jacquelyn Wynn, Mairead Hyland, The Lighthouse Lab in Alderley Park and Alex Alderton, Roberto Amato, Sonia Goncalves, Ewan Harrison, David K. Jackson, Ian Johnston, Dominic Kwiatkowski, Cordelia Langford, John Sillitoe on behalf of the Wellcome Sanger Institute COVID-19 Surveillance Team
EPI_ISL_730831, EPI_ISL_730832	Lighthouse Lab in Glasgow	Wellcome Sanger Institute for the COVID-19 Genomics UK (COG-UK) Consortium	Harper VanSteenhouse, Yumi Kasai, David Gray, Carol Clugston, Anna Dominiczak and Alex Alderton, Roberto Amato, Sonia Goncalves, Ewan Harrison, David K. Jackson, Ian Johnston, Dominic Kwiatkowski, Cordelia Langford, John Sillitoe on behalf of the Wellcome Sanger Institute COVID-19 Surveillance Team
EPI_ISL_730834, EPI_ISL_730835, EPI_ISL_730836, EPI_ISL_730837, EPI_ISL_730838, EPI_ISL_730839	Lighthouse Lab in Alderley Park	Wellcome Sanger Institute for the COVID-19 Genomics UK (COG-UK) Consortium	Jacquelyn Wynn, Mairead Hyland, The Lighthouse Lab in Alderley Park and Alex Alderton, Roberto Amato, Sonia Goncalves, Ewan Harrison, David K. Jackson, Ian Johnston, Dominic Kwiatkowski, Cordelia Langford, John Sillitoe on behalf of the Wellcome Sanger Institute COVID-19 Surveillance Team
EPI_ISL_730840	Lighthouse Lab in Glasgow	Wellcome Sanger Institute for the COVID-19 Genomics UK (COG-UK) Consortium	Harper VanSteenhouse, Yumi Kasai, David Gray, Carol Clugston, Anna Dominiczak and Alex Alderton, Roberto Amato, Sonia Goncalves, Ewan Harrison, David K. Jackson, Ian Johnston, Dominic Kwiatkowski, Cordelia Langford, John Sillitoe on behalf of the Wellcome Sanger Institute COVID-19 Surveillance Team
EPI_ISL_730842, EPI_ISL_730843, EPI_ISL_730844, EPI_ISL_730845	Lighthouse Lab in Alderley Park	Wellcome Sanger Institute for the COVID-19 Genomics UK (COG-UK) Consortium	Jacquelyn Wynn, Mairead Hyland, The Lighthouse Lab in Alderley Park and Alex Alderton, Roberto Amato, Sonia Goncalves, Ewan Harrison, David K. Jackson, Ian Johnston, Dominic Kwiatkowski, Cordelia Langford, John Sillitoe on behalf of the Wellcome Sanger Institute COVID-19 Surveillance Team
EPI_ISL_730846, EPI_ISL_730847	Lighthouse Lab in Glasgow	Wellcome Sanger Institute for the COVID-19 Genomics UK (COG-UK) Consortium	Harper VanSteenhouse, Yumi Kasai, David Gray, Carol Clugston, Anna Dominiczak and Alex Alderton, Roberto Amato, Sonia Goncalves, Ewan Harrison, David K. Jackson, Ian Johnston, Dominic Kwiatkowski, Cordelia Langford, John Sillitoe on behalf of the Wellcome Sanger Institute COVID-19 Surveillance Team
EPI_ISL_730848	Lighthouse Lab in Alderley Park	Wellcome Sanger Institute for the COVID-19 Genomics UK (COG-UK) Consortium	Jacquelyn Wynn, Mairead Hyland, The Lighthouse Lab in Alderley Park and Alex Alderton, Roberto Amato, Sonia Goncalves, Ewan Harrison, David K. Jackson, Ian Johnston, Dominic Kwiatkowski, Cordelia Langford, John Sillitoe on behalf of the Wellcome Sanger Institute COVID-19 Surveillance Team
EPI_ISL_730849, EPI_ISL_730850	Lighthouse Lab in Glasgow	Wellcome Sanger Institute for the COVID-19 Genomics UK (COG-UK) Consortium	Harper VanSteenhouse, Yumi Kasai, David Gray, Carol Clugston, Anna Dominiczak and Alex Alderton, Roberto Amato, Sonia Goncalves, Ewan Harrison, David K. Jackson, Ian Johnston, Dominic Kwiatkowski, Cordelia Langford, John Sillitoe on behalf of the Wellcome Sanger Institute COVID-19 Surveillance Team
EPI_ISL_730851, EPI_ISL_730853, EPI_ISL_730855, EPI_ISL_730856, EPI_ISL_730857	Lighthouse Lab in Alderley Park	Wellcome Sanger Institute for the COVID-19 Genomics UK (COG-UK) Consortium	Jacquelyn Wynn, Mairead Hyland, The Lighthouse Lab in Alderley Park and Alex Alderton, Roberto Amato, Sonia Goncalves, Ewan Harrison, David K. Jackson, Ian Johnston, Dominic Kwiatkowski, Cordelia Langford, John Sillitoe on behalf of the Wellcome Sanger Institute COVID-19 Surveillance Team
EPI_ISL_730858	Lighthouse Lab in Glasgow	Wellcome Sanger Institute for the COVID-19 Genomics UK (COG-UK) Consortium	Harper VanSteenhouse, Yumi Kasai, David Gray, Carol Clugston, Anna Dominiczak and Alex Alderton, Roberto Amato, Sonia Goncalves, Ewan Harrison, David K. Jackson, Ian Johnston, Dominic Kwiatkowski, Cordelia Langford, John Sillitoe on behalf of the Wellcome Sanger Institute COVID-19 Surveillance Team
EPI_ISL_730859	Lighthouse Lab in Alderley Park	Wellcome Sanger Institute for the COVID-19 Genomics UK (COG-UK) Consortium	Jacquelyn Wynn, Mairead Hyland, The Lighthouse Lab in Alderley Park and Alex Alderton, Roberto Amato, Sonia Goncalves, Ewan Harrison, David K. Jackson, Ian Johnston, Dominic Kwiatkowski, Cordelia Langford, John Sillitoe on behalf of the Wellcome Sanger Institute COVID-19 Surveillance Team
EPI_ISL_730860	Lighthouse Lab in Glasgow	Wellcome Sanger Institute for the COVID-19 Genomics UK (COG-UK) Consortium	Harper VanSteenhouse, Yumi Kasai, David Gray, Carol Clugston, Anna Dominiczak and Alex Alderton, Roberto Amato, Sonia Goncalves, Ewan Harrison, David K. Jackson, Ian Johnston, Dominic Kwiatkowski, Cordelia Langford, John Sillitoe on behalf of the Wellcome Sanger Institute COVID-19 Surveillance Team
EPI_ISL_730861	Lighthouse Lab in Alderley Park	Wellcome Sanger Institute for the COVID-19 Genomics UK (COG-UK) Consortium	Jacquelyn Wynn, Mairead Hyland, The Lighthouse Lab in Alderley Park and Alex Alderton, Roberto Amato, Sonia Goncalves, Ewan Harrison, David K. Jackson, Ian Johnston, Dominic Kwiatkowski, Cordelia Langford, John Sillitoe on behalf of the Wellcome Sanger Institute COVID-19 Surveillance Team
EPI_ISL_730862	Lighthouse Lab in Glasgow	Wellcome Sanger Institute for the COVID-19 Genomics UK (COG-UK) Consortium	Harper VanSteenhouse, Yumi Kasai, David Gray, Carol Clugston, Anna Dominiczak and Alex Alderton, Roberto Amato, Sonia Goncalves, Ewan Harrison, David K. Jackson, Ian Johnston, Dominic Kwiatkowski, Cordelia Langford, John Sillitoe on behalf of the Wellcome Sanger Institute COVID-19 Surveillance Team
EPI_ISL_730863	Lighthouse Lab in Alderley Park	Wellcome Sanger Institute for the COVID-19 Genomics UK (COG-UK) Consortium	Jacquelyn Wynn, Mairead Hyland, The Lighthouse Lab in Alderley Park and Alex Alderton, Roberto Amato, Sonia Goncalves, Ewan Harrison, David K. Jackson, Ian Johnston, Dominic Kwiatkowski, Cordelia Langford, John Sillitoe on behalf of the Wellcome Sanger Institute COVID-19 Surveillance Team
EPI_ISL_730864	Lighthouse Lab in Glasgow	Wellcome Sanger Institute for the COVID-19 Genomics UK (COG-UK) Consortium	Harper VanSteenhouse, Yumi Kasai, David Gray, Carol Clugston, Anna Dominiczak and Alex Alderton, Roberto Amato, Sonia Goncalves, Ewan Harrison, David K. Jackson, Ian Johnston, Dominic Kwiatkowski, Cordelia Langford, John Sillitoe on behalf of the Wellcome Sanger Institute COVID-19 Surveillance Team
EPI_ISL_730865, EPI_ISL_730866	Lighthouse Lab in Alderley Park	Wellcome Sanger Institute for the COVID-19 Genomics UK (COG-UK) Consortium	Jacquelyn Wynn, Mairead Hyland, The Lighthouse Lab in Alderley Park and Alex Alderton, Roberto Amato, Sonia Goncalves, Ewan Harrison, David K. Jackson, Ian Johnston, Dominic Kwiatkowski, Cordelia Langford, John Sillitoe on behalf of the Wellcome Sanger Institute COVID-19 Surveillance Team
EPI_ISL_730867	Lighthouse Lab in Glasgow	Wellcome Sanger Institute for the COVID-19 Genomics UK (COG-UK) Consortium	Harper VanSteenhouse, Yumi Kasai, David Gray, Carol Clugston, Anna Dominiczak and Alex Alderton, Roberto Amato, Sonia Goncalves, Ewan Harrison, David K. Jackson, Ian Johnston, Dominic Kwiatkowski, Cordelia Langford, John Sillitoe on behalf of the Wellcome Sanger Institute COVID-19 Surveillance Team
EPI_ISL_730868	Lighthouse Lab in Alderley Park	Wellcome Sanger Institute for the COVID-19 Genomics UK (COG-UK) Consortium	Jacquelyn Wynn, Mairead Hyland, The Lighthouse Lab in Alderley Park and Alex Alderton, Roberto Amato, Sonia Goncalves, Ewan Harrison, David K. Jackson, Ian Johnston, Dominic Kwiatkowski, Cordelia Langford, John Sillitoe on behalf of the Wellcome Sanger Institute COVID-19 Surveillance Team
EPI_ISL_730869	Lighthouse Lab in Glasgow	Wellcome Sanger Institute for the COVID-19 Genomics UK (COG-UK) Consortium	Harper VanSteenhouse, Yumi Kasai, David Gray, Carol Clugston, Anna Dominiczak and Alex Alderton, Roberto Amato, Sonia Goncalves, Ewan Harrison, David K. Jackson, Ian Johnston, Dominic Kwiatkowski, Cordelia Langford, John Sillitoe on behalf of the Wellcome Sanger Institute COVID-19 Surveillance Team
EPI_ISL_730870, EPI_ISL_730871, EPI_ISL_730873	Lighthouse Lab in Alderley Park	Wellcome Sanger Institute for the COVID-19 Genomics UK (COG-UK) Consortium	Jacquelyn Wynn, Mairead Hyland, The Lighthouse Lab in Alderley Park and Alex Alderton, Roberto Amato, Sonia Goncalves, Ewan Harrison, David K. Jackson, Ian Johnston, Dominic Kwiatkowski, Cordelia Langford, John Sillitoe on behalf of the Wellcome Sanger Institute COVID-19 Surveillance Team
EPI_ISL_730875	Lighthouse Lab in Glasgow	Wellcome Sanger Institute for the COVID-19 Genomics UK (COG-UK) Consortium	Harper VanSteenhouse, Yumi Kasai, David Gray, Carol Clugston, Anna Dominiczak and Alex Alderton, Roberto Amato, Sonia Goncalves, Ewan Harrison, David K. Jackson, Ian Johnston, Dominic Kwiatkowski, Cordelia Langford, John Sillitoe on behalf of the Wellcome Sanger Institute COVID-19 Surveillance Team
EPI_ISL_730878, EPI_ISL_730881, EPI_ISL_730883, EPI_ISL_730884, EPI_ISL_730886, EPI_ISL_730888, EPI_ISL_730890, EPI_ISL_730891, EPI_ISL_730892, EPI_ISL_730893, EPI_ISL_730895, EPI_ISL_730896, EPI_ISL_730897, EPI_ISL_730899, EPI_ISL_730903, EPI_ISL_730904, EPI_ISL_730905, EPI_ISL_730907, EPI_ISL_730909			
see above	Lighthouse Lab in Alderley Park	Wellcome Sanger Institute for the COVID-19 Genomics UK (COG-UK) Consortium	Jacquelyn Wynn, Mairead Hyland, The Lighthouse Lab in Alderley Park and Alex Alderton, Roberto Amato, Sonia Goncalves, Ewan Harrison, David K. Jackson, Ian Johnston, Dominic Kwiatkowski, Cordelia Langford, John Sillitoe on behalf of the Wellcome Sanger Institute COVID-19 Surveillance Team
EPI_ISL_730910, EPI_ISL_730911	Lighthouse Lab in Glasgow	Wellcome Sanger Institute for the COVID-19 Genomics UK (COG-UK) Consortium	Harper VanSteenhouse, Yumi Kasai, David Gray, Carol Clugston, Anna Dominiczak and Alex Alderton, Roberto Amato, Sonia Goncalves, Ewan Harrison, David K. Jackson, Ian Johnston, Dominic Kwiatkowski, Cordelia Langford, John Sillitoe on behalf of the Wellcome Sanger Institute COVID-19 Surveillance Team
EPI_ISL_730912	Lighthouse Lab in Alderley Park	Wellcome Sanger Institute for the COVID-19 Genomics UK (COG-UK) Consortium	Jacquelyn Wynn, Mairead Hyland, The Lighthouse Lab in Alderley Park and Alex Alderton, Roberto Amato, Sonia Goncalves, Ewan Harrison, David K. Jackson, Ian Johnston, Dominic Kwiatkowski, Cordelia Langford, John Sillitoe on behalf of the Wellcome Sanger Institute COVID-19 Surveillance Team
EPI_ISL_730913	Lighthouse Lab in Glasgow	Wellcome Sanger Institute for the COVID-19 Genomics UK	Harper VanSteenhouse, Yumi Kasai, David Gray, Carol Clugston, Anna Dominiczak and Alex Alderton, Roberto Amato, Sonia Goncalves, Ewan Harrison,

		(COG-UK) Consortium	David K. Jackson, Ian Johnston, Dominic Kwiatkowski, Cordelia Langford, John Sillitoe on behalf of the Wellcome Sanger Institute COVID-19 Surveillance Team
EPI_ISL_730916, EPI_ISL_730917, EPI_ISL_730919, EPI_ISL_730920, EPI_ISL_730922, EPI_ISL_730923, EPI_ISL_730924, EPI_ISL_730925, EPI_ISL_730933, EPI_ISL_730934, EPI_ISL_730935, EPI_ISL_730936, EPI_ISL_730937, EPI_ISL_730938, EPI_ISL_730940, EPI_ISL_730941, EPI_ISL_730944, EPI_ISL_730945, EPI_ISL_730947, EPI_ISL_730949, EPI_ISL_730950, EPI_ISL_730951, EPI_ISL_730953, EPI_ISL_730954, EPI_ISL_730957, EPI_ISL_730958, EPI_ISL_730960, EPI_ISL_730962, EPI_ISL_730964, EPI_ISL_730968, EPI_ISL_730971, EPI_ISL_730972, EPI_ISL_730973, EPI_ISL_730974, EPI_ISL_730975, EPI_ISL_730976, EPI_ISL_730977, EPI_ISL_730980, EPI_ISL_730981, EPI_ISL_730982, EPI_ISL_730983, EPI_ISL_730984, EPI_ISL_730985, EPI_ISL_730989, EPI_ISL_730990, EPI_ISL_730991, EPI_ISL_730992, EPI_ISL_730994, EPI_ISL_730995, EPI_ISL_730996, EPI_ISL_730997, EPI_ISL_730998, EPI_ISL_730999, EPI_ISL_731000, EPI_ISL_731002, EPI_ISL_731003, EPI_ISL_731004, EPI_ISL_731006, EPI_ISL_731008, EPI_ISL_731009, EPI_ISL_731010, EPI_ISL_731011, EPI_ISL_731014, EPI_ISL_731015, EPI_ISL_731016, EPI_ISL_731017, EPI_ISL_731018, EPI_ISL_731019, EPI_ISL_731020, EPI_ISL_731023, EPI_ISL_731026, EPI_ISL_731027, EPI_ISL_731028, EPI_ISL_731029, EPI_ISL_731030, EPI_ISL_731031, EPI_ISL_731033, EPI_ISL_731034, EPI_ISL_731036, EPI_ISL_731038, EPI_ISL_731039, EPI_ISL_731040, EPI_ISL_731041, EPI_ISL_731042, EPI_ISL_731044, EPI_ISL_731045, EPI_ISL_731046, EPI_ISL_731048, EPI_ISL_731049, EPI_ISL_731051, EPI_ISL_731053, EPI_ISL_731055, EPI_ISL_731056, EPI_ISL_731057, EPI_ISL_731058, EPI_ISL_731060, EPI_ISL_731061, EPI_ISL_731062, EPI_ISL_731063, EPI_ISL_731064, EPI_ISL_731065, EPI_ISL_731066, EPI_ISL_731067, EPI_ISL_731068, EPI_ISL_731070, EPI_ISL_731071, EPI_ISL_731072, EPI_ISL_731074, EPI_ISL_731075, EPI_ISL_731077, EPI_ISL_731078, EPI_ISL_731080, EPI_ISL_731081, EPI_ISL_731082, EPI_ISL_731083, EPI_ISL_731085, EPI_ISL_731086, EPI_ISL_731087, EPI_ISL_731088, EPI_ISL_731089, EPI_ISL_731090, EPI_ISL_731092, EPI_ISL_731093, EPI_ISL_731094, EPI_ISL_731095, EPI_ISL_731096, EPI_ISL_731097, EPI_ISL_731098, EPI_ISL_731100, EPI_ISL_731101, EPI_ISL_731102, EPI_ISL_731103, EPI_ISL_731104, EPI_ISL_731105, EPI_ISL_731106, EPI_ISL_731108, EPI_ISL_731109, EPI_ISL_731110, EPI_ISL_731112, EPI_ISL_731113, EPI_ISL_731114, EPI_ISL_731116, EPI_ISL_731117, EPI_ISL_731118, EPI_ISL_731119, EPI_ISL_731120, EPI_ISL_731121, EPI_ISL_731123, EPI_ISL_731124, EPI_ISL_731125, EPI_ISL_731126, EPI_ISL_731127, EPI_ISL_731128, EPI_ISL_731129, EPI_ISL_731131, EPI_ISL_731132, EPI_ISL_731133, EPI_ISL_731135, EPI_ISL_731137, EPI_ISL_731138, EPI_ISL_731139, EPI_ISL_731140, EPI_ISL_731142, EPI_ISL_731143, EPI_ISL_731144, EPI_ISL_731145, EPI_ISL_731148, EPI_ISL_731149, EPI_ISL_731150, EPI_ISL_731152, EPI_ISL_731154, EPI_ISL_731156, EPI_ISL_731157, EPI_ISL_731160, EPI_ISL_731161, EPI_ISL_731163, EPI_ISL_731164, EPI_ISL_731165, EPI_ISL_731166, EPI_ISL_731168, EPI_ISL_731170, EPI_ISL_731172, EPI_ISL_731173, EPI_ISL_731174, EPI_ISL_731175, EPI_ISL_731176, EPI_ISL_731177, EPI_ISL_731178, EPI_ISL_731181, EPI_ISL_731182, EPI_ISL_731183, EPI_ISL_731185, EPI_ISL_731186, EPI_ISL_731188, EPI_ISL_731189, EPI_ISL_731190, EPI_ISL_731191, EPI_ISL_731192, EPI_ISL_731196, EPI_ISL_731197, EPI_ISL_731200, EPI_ISL_731201, EPI_ISL_731202, EPI_ISL_731203, EPI_ISL_731204, EPI_ISL_731205, EPI_ISL_731207, EPI_ISL_731208, EPI_ISL_731209, EPI_ISL_731211, EPI_ISL_731212, EPI_ISL_731213, EPI_ISL_731214, EPI_ISL_731215, EPI_ISL_731219, EPI_ISL_731220, EPI_ISL_731224, EPI_ISL_731228, EPI_ISL_731229, EPI_ISL_731230, EPI_ISL_731231, EPI_ISL_731232, EPI_ISL_731233, EPI_ISL_731234, EPI_ISL_731235, EPI_ISL_731236, EPI_ISL_731237, EPI_ISL_731239, EPI_ISL_731240, EPI_ISL_731241, EPI_ISL_731243, EPI_ISL_731247, EPI_ISL_731248, EPI_ISL_731249, EPI_ISL_731250, EPI_ISL_731254			
see above	Lighthouse Lab in Alderley Park	Wellcome Sanger Institute for the COVID-19 Genomics UK (COG-UK) Consortium	Jacquelyn Wynn, Mairead Hyland, The Lighthouse Lab in Alderley Park and Alex Alderton, Roberto Amato, Sonia Goncalves, Ewan Harrison, David K. Jackson, Ian Johnston, Dominic Kwiatkowski, Cordelia Langford, John Sillitoe on behalf of the Wellcome Sanger Institute COVID-19 Surveillance Team
EPI_ISL_731255, EPI_ISL_731256, EPI_ISL_731257, EPI_ISL_731259	Lighthouse Lab in Glasgow	Wellcome Sanger Institute for the COVID-19 Genomics UK (COG-UK) Consortium	Harper VanSteenhouse, Yumi Kasai, David Gray, Carol Clugston, Anna Dominiczak and Alex Alderton, Roberto Amato, Sonia Goncalves, Ewan Harrison, David K. Jackson, Ian Johnston, Dominic Kwiatkowski, Cordelia Langford, John Sillitoe on behalf of the Wellcome Sanger Institute COVID-19 Surveillance Team
EPI_ISL_731260	Lighthouse Lab in Alderley Park	Wellcome Sanger Institute for the COVID-19 Genomics UK (COG-UK) Consortium	Jacquelyn Wynn, Mairead Hyland, The Lighthouse Lab in Alderley Park and Alex Alderton, Roberto Amato, Sonia Goncalves, Ewan Harrison, David K. Jackson, Ian Johnston, Dominic Kwiatkowski, Cordelia Langford, John Sillitoe on behalf of the Wellcome Sanger Institute COVID-19 Surveillance Team
EPI_ISL_731261, EPI_ISL_731262	Lighthouse Lab in Glasgow	Wellcome Sanger Institute for the COVID-19 Genomics UK (COG-UK) Consortium	Harper VanSteenhouse, Yumi Kasai, David Gray, Carol Clugston, Anna Dominiczak and Alex Alderton, Roberto Amato, Sonia Goncalves, Ewan Harrison, David K. Jackson, Ian Johnston, Dominic Kwiatkowski, Cordelia Langford, John Sillitoe on behalf of the Wellcome Sanger Institute COVID-19 Surveillance Team
EPI_ISL_731265, EPI_ISL_731268, EPI_ISL_731269	Lighthouse Lab in Alderley Park	Wellcome Sanger Institute for the COVID-19 Genomics UK (COG-UK) Consortium	Jacquelyn Wynn, Mairead Hyland, The Lighthouse Lab in Alderley Park and Alex Alderton, Roberto Amato, Sonia Goncalves, Ewan Harrison, David K. Jackson, Ian Johnston, Dominic Kwiatkowski, Cordelia Langford, John Sillitoe on behalf of the Wellcome Sanger Institute COVID-19 Surveillance Team
EPI_ISL_731271	Lighthouse Lab in Glasgow	Wellcome Sanger Institute for the COVID-19 Genomics UK (COG-UK) Consortium	Harper VanSteenhouse, Yumi Kasai, David Gray, Carol Clugston, Anna Dominiczak and Alex Alderton, Roberto Amato, Sonia Goncalves, Ewan Harrison, David K. Jackson, Ian Johnston, Dominic Kwiatkowski, Cordelia Langford, John Sillitoe on behalf of the Wellcome Sanger Institute COVID-19 Surveillance Team
EPI_ISL_731272	Lighthouse Lab in Alderley Park	Wellcome Sanger Institute for the COVID-19 Genomics UK (COG-UK) Consortium	Jacquelyn Wynn, Mairead Hyland, The Lighthouse Lab in Alderley Park and Alex Alderton, Roberto Amato, Sonia Goncalves, Ewan Harrison, David K. Jackson, Ian Johnston, Dominic Kwiatkowski, Cordelia Langford, John Sillitoe on behalf of the Wellcome Sanger Institute COVID-19 Surveillance Team
EPI_ISL_731273	Lighthouse Lab in Glasgow	Wellcome Sanger Institute for the COVID-19 Genomics UK (COG-UK) Consortium	Harper VanSteenhouse, Yumi Kasai, David Gray, Carol Clugston, Anna Dominiczak and Alex Alderton, Roberto Amato, Sonia Goncalves, Ewan Harrison, David K. Jackson, Ian Johnston, Dominic Kwiatkowski, Cordelia Langford, John Sillitoe on behalf of the Wellcome Sanger Institute COVID-19 Surveillance Team
EPI_ISL_731274, EPI_ISL_731275, EPI_ISL_731277, EPI_ISL_731278	Lighthouse Lab in Alderley Park	Wellcome Sanger Institute for the COVID-19 Genomics UK (COG-UK) Consortium	Jacquelyn Wynn, Mairead Hyland, The Lighthouse Lab in Alderley Park and Alex Alderton, Roberto Amato, Sonia Goncalves, Ewan Harrison, David K. Jackson, Ian Johnston, Dominic Kwiatkowski, Cordelia Langford, John Sillitoe on behalf of the Wellcome Sanger Institute COVID-19 Surveillance Team
EPI_ISL_731280, EPI_ISL_731281, EPI_ISL_731282, EPI_ISL_731283, EPI_ISL_731284, EPI_ISL_731286, EPI_ISL_731287	Lighthouse Lab in Glasgow	Wellcome Sanger Institute for the COVID-19 Genomics UK (COG-UK) Consortium	Harper VanSteenhouse, Yumi Kasai, David Gray, Carol Clugston, Anna Dominiczak and Alex Alderton, Roberto Amato, Sonia Goncalves, Ewan Harrison, David K. Jackson, Ian Johnston, Dominic Kwiatkowski, Cordelia Langford, John Sillitoe on behalf of the Wellcome Sanger Institute COVID-19 Surveillance Team
EPI_ISL_731292	Lighthouse Lab in Alderley Park	Wellcome Sanger Institute for the COVID-19 Genomics UK (COG-UK) Consortium	Jacquelyn Wynn, Mairead Hyland, The Lighthouse Lab in Alderley Park and Alex Alderton, Roberto Amato, Sonia Goncalves, Ewan Harrison, David K. Jackson, Ian Johnston, Dominic Kwiatkowski, Cordelia Langford, John Sillitoe on behalf of the Wellcome Sanger Institute COVID-19 Surveillance Team
EPI_ISL_731293	Lighthouse Lab in Glasgow	Wellcome Sanger Institute for the COVID-19 Genomics UK (COG-UK) Consortium	Harper VanSteenhouse, Yumi Kasai, David Gray, Carol Clugston, Anna Dominiczak and Alex Alderton, Roberto Amato, Sonia Goncalves, Ewan Harrison, David K. Jackson, Ian Johnston, Dominic Kwiatkowski, Cordelia Langford, John Sillitoe on behalf of the Wellcome Sanger Institute COVID-19 Surveillance Team
EPI_ISL_731294	Lighthouse Lab in Alderley Park	Wellcome Sanger Institute for the COVID-19 Genomics UK (COG-UK) Consortium	Jacquelyn Wynn, Mairead Hyland, The Lighthouse Lab in Alderley Park and Alex Alderton, Roberto Amato, Sonia Goncalves, Ewan Harrison, David K. Jackson, Ian Johnston, Dominic Kwiatkowski, Cordelia Langford, John Sillitoe on behalf of the Wellcome Sanger Institute COVID-19 Surveillance Team
EPI_ISL_731295	Lighthouse Lab in Glasgow	Wellcome Sanger Institute for the COVID-19 Genomics UK (COG-UK) Consortium	Harper VanSteenhouse, Yumi Kasai, David Gray, Carol Clugston, Anna Dominiczak and Alex Alderton, Roberto Amato, Sonia Goncalves, Ewan Harrison, David K. Jackson, Ian Johnston, Dominic Kwiatkowski, Cordelia Langford, John Sillitoe on behalf of the Wellcome Sanger Institute COVID-19 Surveillance Team
EPI_ISL_731298	Lighthouse Lab in Alderley Park	Wellcome Sanger Institute for the COVID-19 Genomics UK (COG-UK) Consortium	Jacquelyn Wynn, Mairead Hyland, The Lighthouse Lab in Alderley Park and Alex Alderton, Roberto Amato, Sonia Goncalves, Ewan Harrison, David K. Jackson, Ian Johnston, Dominic Kwiatkowski, Cordelia Langford, John Sillitoe on behalf of the Wellcome Sanger Institute COVID-19 Surveillance Team
EPI_ISL_731299	Lighthouse Lab in Glasgow	Wellcome Sanger Institute for the COVID-19 Genomics UK (COG-UK) Consortium	Harper VanSteenhouse, Yumi Kasai, David Gray, Carol Clugston, Anna Dominiczak and Alex Alderton, Roberto Amato, Sonia Goncalves, Ewan Harrison, David K. Jackson, Ian Johnston, Dominic Kwiatkowski, Cordelia Langford, John Sillitoe on behalf of the Wellcome Sanger Institute COVID-19 Surveillance Team
EPI_ISL_731301, EPI_ISL_731302, EPI_ISL_731304	Lighthouse Lab in Alderley Park	Wellcome Sanger Institute for the COVID-19 Genomics UK (COG-UK) Consortium	Jacquelyn Wynn, Mairead Hyland, The Lighthouse Lab in Alderley Park and Alex Alderton, Roberto Amato, Sonia Goncalves, Ewan Harrison, David K. Jackson, Ian Johnston, Dominic Kwiatkowski, Cordelia Langford, John Sillitoe on behalf of the Wellcome Sanger Institute COVID-19 Surveillance Team
EPI_ISL_731305, EPI_ISL_731309, EPI_ISL_731310	Lighthouse Lab in Glasgow	Wellcome Sanger Institute for the COVID-19 Genomics UK (COG-UK) Consortium	Harper VanSteenhouse, Yumi Kasai, David Gray, Carol Clugston, Anna Dominiczak and Alex Alderton, Roberto Amato, Sonia Goncalves, Ewan Harrison, David K. Jackson, Ian Johnston, Dominic Kwiatkowski, Cordelia Langford, John Sillitoe on behalf of the Wellcome Sanger Institute COVID-19 Surveillance Team
EPI_ISL_731311, EPI_ISL_731312	Lighthouse Lab in Alderley Park	Wellcome Sanger Institute for the COVID-19 Genomics UK (COG-UK) Consortium	Jacquelyn Wynn, Mairead Hyland, The Lighthouse Lab in Alderley Park and Alex Alderton, Roberto Amato, Sonia Goncalves, Ewan Harrison, David K. Jackson, Ian Johnston, Dominic Kwiatkowski, Cordelia Langford, John Sillitoe on behalf of the Wellcome Sanger Institute COVID-19 Surveillance Team
EPI_ISL_731314	Lighthouse Lab in Glasgow	Wellcome Sanger Institute for the COVID-19 Genomics UK (COG-UK) Consortium	Harper VanSteenhouse, Yumi Kasai, David Gray, Carol Clugston, Anna Dominiczak and Alex Alderton, Roberto Amato, Sonia Goncalves, Ewan Harrison, David K. Jackson, Ian Johnston, Dominic Kwiatkowski, Cordelia Langford, John Sillitoe on behalf of the Wellcome Sanger Institute COVID-19 Surveillance Team
EPI_ISL_731315, EPI_ISL_731316, EPI_ISL_731317, EPI_ISL_731318	Lighthouse Lab in Alderley Park	Wellcome Sanger Institute for the COVID-19 Genomics UK (COG-UK) Consortium	Jacquelyn Wynn, Mairead Hyland, The Lighthouse Lab in Alderley Park and Alex Alderton, Roberto Amato, Sonia Goncalves, Ewan Harrison, David K. Jackson, Ian Johnston, Dominic Kwiatkowski, Cordelia Langford, John Sillitoe on behalf of the Wellcome Sanger Institute COVID-19 Surveillance Team
EPI_ISL_731319, EPI_ISL_731320	Lighthouse Lab in Glasgow	Wellcome Sanger Institute for the COVID-19 Genomics UK (COG-UK) Consortium	Harper VanSteenhouse, Yumi Kasai, David Gray, Carol Clugston, Anna Dominiczak and Alex Alderton, Roberto Amato, Sonia Goncalves, Ewan Harrison, David K. Jackson, Ian Johnston, Dominic Kwiatkowski, Cordelia Langford, John Sillitoe on behalf of the Wellcome Sanger Institute COVID-19 Surveillance Team
EPI_ISL_731324	Lighthouse Lab in Alderley Park	Wellcome Sanger Institute for the COVID-19 Genomics UK (COG-UK) Consortium	Jacquelyn Wynn, Mairead Hyland, The Lighthouse Lab in Alderley Park and Alex Alderton, Roberto Amato, Sonia Goncalves, Ewan Harrison, David K. Jackson, Ian Johnston, Dominic Kwiatkowski, Cordelia Langford, John Sillitoe on behalf of the Wellcome Sanger Institute COVID-19 Surveillance Team
EPI_ISL_731327	Lighthouse Lab in Glasgow	Wellcome Sanger Institute for the COVID-19 Genomics UK	Harper VanSteenhouse, Yumi Kasai, David Gray, Carol Clugston, Anna Dominiczak and Alex Alderton, Roberto Amato, Sonia Goncalves, Ewan Harrison,

		(COG-UK) Consortium	David K. Jackson, Ian Johnston, Dominic Kwiatkowski, Cordelia Langford, John Sillitoe on behalf of the Wellcome Sanger Institute COVID-19 Surveillance Team
EPI_ISL_731329, EPI_ISL_731331	Lighthouse Lab in Alderley Park	Wellcome Sanger Institute for the COVID-19 Genomics UK (COG-UK) Consortium	Jacquelyn Wynn, Mairead Hyland, The Lighthouse Lab in Alderley Park and Alex Alderton, Roberto Amato, Sonia Goncalves, Ewan Harrison, David K. Jackson, Ian Johnston, Dominic Kwiatkowski, Cordelia Langford, John Sillitoe on behalf of the Wellcome Sanger Institute COVID-19 Surveillance Team
EPI_ISL_731333, EPI_ISL_731334, EPI_ISL_731338	Lighthouse Lab in Glasgow	Wellcome Sanger Institute for the COVID-19 Genomics UK (COG-UK) Consortium	Harper VanSteenhouse, Yumi Kasai, David Gray, Carol Clugston, Anna Dominiczak and Alex Alderton, Roberto Amato, Sonia Goncalves, Ewan Harrison, David K. Jackson, Ian Johnston, Dominic Kwiatkowski, Cordelia Langford, John Sillitoe on behalf of the Wellcome Sanger Institute COVID-19 Surveillance Team
EPI_ISL_731339	Lighthouse Lab in Alderley Park	Wellcome Sanger Institute for the COVID-19 Genomics UK (COG-UK) Consortium	Jacquelyn Wynn, Mairead Hyland, The Lighthouse Lab in Alderley Park and Alex Alderton, Roberto Amato, Sonia Goncalves, Ewan Harrison, David K. Jackson, Ian Johnston, Dominic Kwiatkowski, Cordelia Langford, John Sillitoe on behalf of the Wellcome Sanger Institute COVID-19 Surveillance Team
EPI_ISL_731341	Lighthouse Lab in Glasgow	Wellcome Sanger Institute for the COVID-19 Genomics UK (COG-UK) Consortium	Harper VanSteenhouse, Yumi Kasai, David Gray, Carol Clugston, Anna Dominiczak and Alex Alderton, Roberto Amato, Sonia Goncalves, Ewan Harrison, David K. Jackson, Ian Johnston, Dominic Kwiatkowski, Cordelia Langford, John Sillitoe on behalf of the Wellcome Sanger Institute COVID-19 Surveillance Team
EPI_ISL_731342, EPI_ISL_731343, EPI_ISL_731344	Lighthouse Lab in Alderley Park	Wellcome Sanger Institute for the COVID-19 Genomics UK (COG-UK) Consortium	Jacquelyn Wynn, Mairead Hyland, The Lighthouse Lab in Alderley Park and Alex Alderton, Roberto Amato, Sonia Goncalves, Ewan Harrison, David K. Jackson, Ian Johnston, Dominic Kwiatkowski, Cordelia Langford, John Sillitoe on behalf of the Wellcome Sanger Institute COVID-19 Surveillance Team
EPI_ISL_731345, EPI_ISL_731346, EPI_ISL_731347, EPI_ISL_731349	Lighthouse Lab in Glasgow	Wellcome Sanger Institute for the COVID-19 Genomics UK (COG-UK) Consortium	Harper VanSteenhouse, Yumi Kasai, David Gray, Carol Clugston, Anna Dominiczak and Alex Alderton, Roberto Amato, Sonia Goncalves, Ewan Harrison, David K. Jackson, Ian Johnston, Dominic Kwiatkowski, Cordelia Langford, John Sillitoe on behalf of the Wellcome Sanger Institute COVID-19 Surveillance Team
EPI_ISL_731350, EPI_ISL_731351	Lighthouse Lab in Alderley Park	Wellcome Sanger Institute for the COVID-19 Genomics UK (COG-UK) Consortium	Jacquelyn Wynn, Mairead Hyland, The Lighthouse Lab in Alderley Park and Alex Alderton, Roberto Amato, Sonia Goncalves, Ewan Harrison, David K. Jackson, Ian Johnston, Dominic Kwiatkowski, Cordelia Langford, John Sillitoe on behalf of the Wellcome Sanger Institute COVID-19 Surveillance Team
EPI_ISL_731353, EPI_ISL_731354	Lighthouse Lab in Glasgow	Wellcome Sanger Institute for the COVID-19 Genomics UK (COG-UK) Consortium	Harper VanSteenhouse, Yumi Kasai, David Gray, Carol Clugston, Anna Dominiczak and Alex Alderton, Roberto Amato, Sonia Goncalves, Ewan Harrison, David K. Jackson, Ian Johnston, Dominic Kwiatkowski, Cordelia Langford, John Sillitoe on behalf of the Wellcome Sanger Institute COVID-19 Surveillance Team
EPI_ISL_731358	Lighthouse Lab in Alderley Park	Wellcome Sanger Institute for the COVID-19 Genomics UK (COG-UK) Consortium	Jacquelyn Wynn, Mairead Hyland, The Lighthouse Lab in Alderley Park and Alex Alderton, Roberto Amato, Sonia Goncalves, Ewan Harrison, David K. Jackson, Ian Johnston, Dominic Kwiatkowski, Cordelia Langford, John Sillitoe on behalf of the Wellcome Sanger Institute COVID-19 Surveillance Team
EPI_ISL_731361	Lighthouse Lab in Glasgow	Wellcome Sanger Institute for the COVID-19 Genomics UK (COG-UK) Consortium	Harper VanSteenhouse, Yumi Kasai, David Gray, Carol Clugston, Anna Dominiczak and Alex Alderton, Roberto Amato, Sonia Goncalves, Ewan Harrison, David K. Jackson, Ian Johnston, Dominic Kwiatkowski, Cordelia Langford, John Sillitoe on behalf of the Wellcome Sanger Institute COVID-19 Surveillance Team
EPI_ISL_731362, EPI_ISL_731363, EPI_ISL_731364, EPI_ISL_731365, EPI_ISL_731366	Lighthouse Lab in Alderley Park	Wellcome Sanger Institute for the COVID-19 Genomics UK (COG-UK) Consortium	Jacquelyn Wynn, Mairead Hyland, The Lighthouse Lab in Alderley Park and Alex Alderton, Roberto Amato, Sonia Goncalves, Ewan Harrison, David K. Jackson, Ian Johnston, Dominic Kwiatkowski, Cordelia Langford, John Sillitoe on behalf of the Wellcome Sanger Institute COVID-19 Surveillance Team
EPI_ISL_731367	Lighthouse Lab in Glasgow	Wellcome Sanger Institute for the COVID-19 Genomics UK (COG-UK) Consortium	Harper VanSteenhouse, Yumi Kasai, David Gray, Carol Clugston, Anna Dominiczak and Alex Alderton, Roberto Amato, Sonia Goncalves, Ewan Harrison, David K. Jackson, Ian Johnston, Dominic Kwiatkowski, Cordelia Langford, John Sillitoe on behalf of the Wellcome Sanger Institute COVID-19 Surveillance Team
EPI_ISL_731369, EPI_ISL_731371	Lighthouse Lab in Alderley Park	Wellcome Sanger Institute for the COVID-19 Genomics UK (COG-UK) Consortium	Jacquelyn Wynn, Mairead Hyland, The Lighthouse Lab in Alderley Park and Alex Alderton, Roberto Amato, Sonia Goncalves, Ewan Harrison, David K. Jackson, Ian Johnston, Dominic Kwiatkowski, Cordelia Langford, John Sillitoe on behalf of the Wellcome Sanger Institute COVID-19 Surveillance Team
EPI_ISL_731373	Lighthouse Lab in Glasgow	Wellcome Sanger Institute for the COVID-19 Genomics UK (COG-UK) Consortium	Harper VanSteenhouse, Yumi Kasai, David Gray, Carol Clugston, Anna Dominiczak and Alex Alderton, Roberto Amato, Sonia Goncalves, Ewan Harrison, David K. Jackson, Ian Johnston, Dominic Kwiatkowski, Cordelia Langford, John Sillitoe on behalf of the Wellcome Sanger Institute COVID-19 Surveillance Team
EPI_ISL_731374, EPI_ISL_731375	Lighthouse Lab in Alderley Park	Wellcome Sanger Institute for the COVID-19 Genomics UK (COG-UK) Consortium	Jacquelyn Wynn, Mairead Hyland, The Lighthouse Lab in Alderley Park and Alex Alderton, Roberto Amato, Sonia Goncalves, Ewan Harrison, David K. Jackson, Ian Johnston, Dominic Kwiatkowski, Cordelia Langford, John Sillitoe on behalf of the Wellcome Sanger Institute COVID-19 Surveillance Team
EPI_ISL_731376	Lighthouse Lab in Glasgow	Wellcome Sanger Institute for the COVID-19 Genomics UK (COG-UK) Consortium	Harper VanSteenhouse, Yumi Kasai, David Gray, Carol Clugston, Anna Dominiczak and Alex Alderton, Roberto Amato, Sonia Goncalves, Ewan Harrison, David K. Jackson, Ian Johnston, Dominic Kwiatkowski, Cordelia Langford, John Sillitoe on behalf of the Wellcome Sanger Institute COVID-19 Surveillance Team
EPI_ISL_731377	Lighthouse Lab in Alderley Park	Wellcome Sanger Institute for the COVID-19 Genomics UK (COG-UK) Consortium	Jacquelyn Wynn, Mairead Hyland, The Lighthouse Lab in Alderley Park and Alex Alderton, Roberto Amato, Sonia Goncalves, Ewan Harrison, David K. Jackson, Ian Johnston, Dominic Kwiatkowski, Cordelia Langford, John Sillitoe on behalf of the Wellcome Sanger Institute COVID-19 Surveillance Team
EPI_ISL_731380, EPI_ISL_731382	Lighthouse Lab in Glasgow	Wellcome Sanger Institute for the COVID-19 Genomics UK (COG-UK) Consortium	Harper VanSteenhouse, Yumi Kasai, David Gray, Carol Clugston, Anna Dominiczak and Alex Alderton, Roberto Amato, Sonia Goncalves, Ewan Harrison, David K. Jackson, Ian Johnston, Dominic Kwiatkowski, Cordelia Langford, John Sillitoe on behalf of the Wellcome Sanger Institute COVID-19 Surveillance Team
EPI_ISL_731385	Lighthouse Lab in Alderley Park	Wellcome Sanger Institute for the COVID-19 Genomics UK (COG-UK) Consortium	Jacquelyn Wynn, Mairead Hyland, The Lighthouse Lab in Alderley Park and Alex Alderton, Roberto Amato, Sonia Goncalves, Ewan Harrison, David K. Jackson, Ian Johnston, Dominic Kwiatkowski, Cordelia Langford, John Sillitoe on behalf of the Wellcome Sanger Institute COVID-19 Surveillance Team
EPI_ISL_731386	Lighthouse Lab in Glasgow	Wellcome Sanger Institute for the COVID-19 Genomics UK (COG-UK) Consortium	Harper VanSteenhouse, Yumi Kasai, David Gray, Carol Clugston, Anna Dominiczak and Alex Alderton, Roberto Amato, Sonia Goncalves, Ewan Harrison, David K. Jackson, Ian Johnston, Dominic Kwiatkowski, Cordelia Langford, John Sillitoe on behalf of the Wellcome Sanger Institute COVID-19 Surveillance Team
EPI_ISL_731387, EPI_ISL_731388, EPI_ISL_731389	Lighthouse Lab in Alderley Park	Wellcome Sanger Institute for the COVID-19 Genomics UK (COG-UK) Consortium	Jacquelyn Wynn, Mairead Hyland, The Lighthouse Lab in Alderley Park and Alex Alderton, Roberto Amato, Sonia Goncalves, Ewan Harrison, David K. Jackson, Ian Johnston, Dominic Kwiatkowski, Cordelia Langford, John Sillitoe on behalf of the Wellcome Sanger Institute COVID-19 Surveillance Team
EPI_ISL_731390	Lighthouse Lab in Glasgow	Wellcome Sanger Institute for the COVID-19 Genomics UK (COG-UK) Consortium	Harper VanSteenhouse, Yumi Kasai, David Gray, Carol Clugston, Anna Dominiczak and Alex Alderton, Roberto Amato, Sonia Goncalves, Ewan Harrison, David K. Jackson, Ian Johnston, Dominic Kwiatkowski, Cordelia Langford, John Sillitoe on behalf of the Wellcome Sanger Institute COVID-19 Surveillance Team
EPI_ISL_731391	Lighthouse Lab in Alderley Park	Wellcome Sanger Institute for the COVID-19 Genomics UK (COG-UK) Consortium	Jacquelyn Wynn, Mairead Hyland, The Lighthouse Lab in Alderley Park and Alex Alderton, Roberto Amato, Sonia Goncalves, Ewan Harrison, David K. Jackson, Ian Johnston, Dominic Kwiatkowski, Cordelia Langford, John Sillitoe on behalf of the Wellcome Sanger Institute COVID-19 Surveillance Team
EPI_ISL_731393	Lighthouse Lab in Glasgow	Wellcome Sanger Institute for the COVID-19 Genomics UK (COG-UK) Consortium	Harper VanSteenhouse, Yumi Kasai, David Gray, Carol Clugston, Anna Dominiczak and Alex Alderton, Roberto Amato, Sonia Goncalves, Ewan Harrison, David K. Jackson, Ian Johnston, Dominic Kwiatkowski, Cordelia Langford, John Sillitoe on behalf of the Wellcome Sanger Institute COVID-19 Surveillance Team
EPI_ISL_731395	Lighthouse Lab in Alderley Park	Wellcome Sanger Institute for the COVID-19 Genomics UK (COG-UK) Consortium	Jacquelyn Wynn, Mairead Hyland, The Lighthouse Lab in Alderley Park and Alex Alderton, Roberto Amato, Sonia Goncalves, Ewan Harrison, David K. Jackson, Ian Johnston, Dominic Kwiatkowski, Cordelia Langford, John Sillitoe on behalf of the Wellcome Sanger Institute COVID-19 Surveillance Team
EPI_ISL_731398	Lighthouse Lab in Glasgow	Wellcome Sanger Institute for the COVID-19 Genomics UK (COG-UK) Consortium	Harper VanSteenhouse, Yumi Kasai, David Gray, Carol Clugston, Anna Dominiczak and Alex Alderton, Roberto Amato, Sonia Goncalves, Ewan Harrison, David K. Jackson, Ian Johnston, Dominic Kwiatkowski, Cordelia Langford, John Sillitoe on behalf of the Wellcome Sanger Institute COVID-19 Surveillance Team
EPI_ISL_731399	Lighthouse Lab in Alderley Park	Wellcome Sanger Institute for the COVID-19 Genomics UK (COG-UK) Consortium	Jacquelyn Wynn, Mairead Hyland, The Lighthouse Lab in Alderley Park and Alex Alderton, Roberto Amato, Sonia Goncalves, Ewan Harrison, David K. Jackson, Ian Johnston, Dominic Kwiatkowski, Cordelia Langford, John Sillitoe on behalf of the Wellcome Sanger Institute COVID-19 Surveillance Team
EPI_ISL_731400, EPI_ISL_731401, EPI_ISL_731402, EPI_ISL_731403, EPI_ISL_731404, EPI_ISL_731405,	Lighthouse Lab in Glasgow	Wellcome Sanger Institute for the COVID-19 Genomics UK (COG-UK) Consortium	Harper VanSteenhouse, Yumi Kasai, David Gray, Carol Clugston, Anna Dominiczak and Alex Alderton, Roberto Amato, Sonia Goncalves, Ewan Harrison, David K. Jackson, Ian Johnston, Dominic Kwiatkowski, Cordelia Langford, John Sillitoe on behalf of the Wellcome Sanger Institute COVID-19 Surveillance Team

EPI_ISL_731406, EPI_ISL_731407			
EPI_ISL_731410	Lighthouse Lab in Alderley Park	Wellcome Sanger Institute for the COVID-19 Genomics UK (COG-UK) Consortium	Jacquelyn Wynn, Mairead Hyland, The Lighthouse Lab in Alderley Park and Alex Alderton, Roberto Amato, Sonia Goncalves, Ewan Harrison, David K. Jackson, Ian Johnston, Dominic Kwiatkowski, Cordelia Langford, John Sillitoe on behalf of the Wellcome Sanger Institute COVID-19 Surveillance Team
EPI_ISL_731411	Lighthouse Lab in Glasgow	Wellcome Sanger Institute for the COVID-19 Genomics UK (COG-UK) Consortium	Harper VanSteenhouse, Yumi Kasai, David Gray, Carol Clugston, Anna Dominiczak and Alex Alderton, Roberto Amato, Sonia Goncalves, Ewan Harrison, David K. Jackson, Ian Johnston, Dominic Kwiatkowski, Cordelia Langford, John Sillitoe on behalf of the Wellcome Sanger Institute COVID-19 Surveillance Team
EPI_ISL_731412, EPI_ISL_731413, EPI_ISL_731415	Lighthouse Lab in Alderley Park	Wellcome Sanger Institute for the COVID-19 Genomics UK (COG-UK) Consortium	Jacquelyn Wynn, Mairead Hyland, The Lighthouse Lab in Alderley Park and Alex Alderton, Roberto Amato, Sonia Goncalves, Ewan Harrison, David K. Jackson, Ian Johnston, Dominic Kwiatkowski, Cordelia Langford, John Sillitoe on behalf of the Wellcome Sanger Institute COVID-19 Surveillance Team
EPI_ISL_731417	Lighthouse Lab in Glasgow	Wellcome Sanger Institute for the COVID-19 Genomics UK (COG-UK) Consortium	Harper VanSteenhouse, Yumi Kasai, David Gray, Carol Clugston, Anna Dominiczak and Alex Alderton, Roberto Amato, Sonia Goncalves, Ewan Harrison, David K. Jackson, Ian Johnston, Dominic Kwiatkowski, Cordelia Langford, John Sillitoe on behalf of the Wellcome Sanger Institute COVID-19 Surveillance Team
EPI_ISL_731418	Lighthouse Lab in Alderley Park	Wellcome Sanger Institute for the COVID-19 Genomics UK (COG-UK) Consortium	Jacquelyn Wynn, Mairead Hyland, The Lighthouse Lab in Alderley Park and Alex Alderton, Roberto Amato, Sonia Goncalves, Ewan Harrison, David K. Jackson, Ian Johnston, Dominic Kwiatkowski, Cordelia Langford, John Sillitoe on behalf of the Wellcome Sanger Institute COVID-19 Surveillance Team
EPI_ISL_731421	Lighthouse Lab in Glasgow	Wellcome Sanger Institute for the COVID-19 Genomics UK (COG-UK) Consortium	Harper VanSteenhouse, Yumi Kasai, David Gray, Carol Clugston, Anna Dominiczak and Alex Alderton, Roberto Amato, Sonia Goncalves, Ewan Harrison, David K. Jackson, Ian Johnston, Dominic Kwiatkowski, Cordelia Langford, John Sillitoe on behalf of the Wellcome Sanger Institute COVID-19 Surveillance Team
EPI_ISL_731422, EPI_ISL_731424	Lighthouse Lab in Alderley Park	Wellcome Sanger Institute for the COVID-19 Genomics UK (COG-UK) Consortium	Jacquelyn Wynn, Mairead Hyland, The Lighthouse Lab in Alderley Park and Alex Alderton, Roberto Amato, Sonia Goncalves, Ewan Harrison, David K. Jackson, Ian Johnston, Dominic Kwiatkowski, Cordelia Langford, John Sillitoe on behalf of the Wellcome Sanger Institute COVID-19 Surveillance Team
EPI_ISL_731425, EPI_ISL_731426	Lighthouse Lab in Glasgow	Wellcome Sanger Institute for the COVID-19 Genomics UK (COG-UK) Consortium	Harper VanSteenhouse, Yumi Kasai, David Gray, Carol Clugston, Anna Dominiczak and Alex Alderton, Roberto Amato, Sonia Goncalves, Ewan Harrison, David K. Jackson, Ian Johnston, Dominic Kwiatkowski, Cordelia Langford, John Sillitoe on behalf of the Wellcome Sanger Institute COVID-19 Surveillance Team
EPI_ISL_731427, EPI_ISL_731428	Lighthouse Lab in Alderley Park	Wellcome Sanger Institute for the COVID-19 Genomics UK (COG-UK) Consortium	Jacquelyn Wynn, Mairead Hyland, The Lighthouse Lab in Alderley Park and Alex Alderton, Roberto Amato, Sonia Goncalves, Ewan Harrison, David K. Jackson, Ian Johnston, Dominic Kwiatkowski, Cordelia Langford, John Sillitoe on behalf of the Wellcome Sanger Institute COVID-19 Surveillance Team
EPI_ISL_731431, EPI_ISL_731432, EPI_ISL_731435	Lighthouse Lab in Glasgow	Wellcome Sanger Institute for the COVID-19 Genomics UK (COG-UK) Consortium	Harper VanSteenhouse, Yumi Kasai, David Gray, Carol Clugston, Anna Dominiczak and Alex Alderton, Roberto Amato, Sonia Goncalves, Ewan Harrison, David K. Jackson, Ian Johnston, Dominic Kwiatkowski, Cordelia Langford, John Sillitoe on behalf of the Wellcome Sanger Institute COVID-19 Surveillance Team
EPI_ISL_731436	Lighthouse Lab in Alderley Park	Wellcome Sanger Institute for the COVID-19 Genomics UK (COG-UK) Consortium	Jacquelyn Wynn, Mairead Hyland, The Lighthouse Lab in Alderley Park and Alex Alderton, Roberto Amato, Sonia Goncalves, Ewan Harrison, David K. Jackson, Ian Johnston, Dominic Kwiatkowski, Cordelia Langford, John Sillitoe on behalf of the Wellcome Sanger Institute COVID-19 Surveillance Team
EPI_ISL_731438	Lighthouse Lab in Glasgow	Wellcome Sanger Institute for the COVID-19 Genomics UK (COG-UK) Consortium	Harper VanSteenhouse, Yumi Kasai, David Gray, Carol Clugston, Anna Dominiczak and Alex Alderton, Roberto Amato, Sonia Goncalves, Ewan Harrison, David K. Jackson, Ian Johnston, Dominic Kwiatkowski, Cordelia Langford, John Sillitoe on behalf of the Wellcome Sanger Institute COVID-19 Surveillance Team
EPI_ISL_731439, EPI_ISL_731440, EPI_ISL_731442	Lighthouse Lab in Alderley Park	Wellcome Sanger Institute for the COVID-19 Genomics UK (COG-UK) Consortium	Jacquelyn Wynn, Mairead Hyland, The Lighthouse Lab in Alderley Park and Alex Alderton, Roberto Amato, Sonia Goncalves, Ewan Harrison, David K. Jackson, Ian Johnston, Dominic Kwiatkowski, Cordelia Langford, John Sillitoe on behalf of the Wellcome Sanger Institute COVID-19 Surveillance Team
EPI_ISL_731444, EPI_ISL_731447	Lighthouse Lab in Glasgow	Wellcome Sanger Institute for the COVID-19 Genomics UK (COG-UK) Consortium	Harper VanSteenhouse, Yumi Kasai, David Gray, Carol Clugston, Anna Dominiczak and Alex Alderton, Roberto Amato, Sonia Goncalves, Ewan Harrison, David K. Jackson, Ian Johnston, Dominic Kwiatkowski, Cordelia Langford, John Sillitoe on behalf of the Wellcome Sanger Institute COVID-19 Surveillance Team
EPI_ISL_731448	Lighthouse Lab in Alderley Park	Wellcome Sanger Institute for the COVID-19 Genomics UK (COG-UK) Consortium	Jacquelyn Wynn, Mairead Hyland, The Lighthouse Lab in Alderley Park and Alex Alderton, Roberto Amato, Sonia Goncalves, Ewan Harrison, David K. Jackson, Ian Johnston, Dominic Kwiatkowski, Cordelia Langford, John Sillitoe on behalf of the Wellcome Sanger Institute COVID-19 Surveillance Team
EPI_ISL_731449, EPI_ISL_731450	Lighthouse Lab in Glasgow	Wellcome Sanger Institute for the COVID-19 Genomics UK (COG-UK) Consortium	Harper VanSteenhouse, Yumi Kasai, David Gray, Carol Clugston, Anna Dominiczak and Alex Alderton, Roberto Amato, Sonia Goncalves, Ewan Harrison, David K. Jackson, Ian Johnston, Dominic Kwiatkowski, Cordelia Langford, John Sillitoe on behalf of the Wellcome Sanger Institute COVID-19 Surveillance Team
EPI_ISL_731451	Lighthouse Lab in Alderley Park	Wellcome Sanger Institute for the COVID-19 Genomics UK (COG-UK) Consortium	Jacquelyn Wynn, Mairead Hyland, The Lighthouse Lab in Alderley Park and Alex Alderton, Roberto Amato, Sonia Goncalves, Ewan Harrison, David K. Jackson, Ian Johnston, Dominic Kwiatkowski, Cordelia Langford, John Sillitoe on behalf of the Wellcome Sanger Institute COVID-19 Surveillance Team
EPI_ISL_731452, EPI_ISL_731453	Lighthouse Lab in Glasgow	Wellcome Sanger Institute for the COVID-19 Genomics UK (COG-UK) Consortium	Harper VanSteenhouse, Yumi Kasai, David Gray, Carol Clugston, Anna Dominiczak and Alex Alderton, Roberto Amato, Sonia Goncalves, Ewan Harrison, David K. Jackson, Ian Johnston, Dominic Kwiatkowski, Cordelia Langford, John Sillitoe on behalf of the Wellcome Sanger Institute COVID-19 Surveillance Team
EPI_ISL_731455, EPI_ISL_731457	Lighthouse Lab in Alderley Park	Wellcome Sanger Institute for the COVID-19 Genomics UK (COG-UK) Consortium	Jacquelyn Wynn, Mairead Hyland, The Lighthouse Lab in Alderley Park and Alex Alderton, Roberto Amato, Sonia Goncalves, Ewan Harrison, David K. Jackson, Ian Johnston, Dominic Kwiatkowski, Cordelia Langford, John Sillitoe on behalf of the Wellcome Sanger Institute COVID-19 Surveillance Team
EPI_ISL_731458, EPI_ISL_731459	Lighthouse Lab in Glasgow	Wellcome Sanger Institute for the COVID-19 Genomics UK (COG-UK) Consortium	Harper VanSteenhouse, Yumi Kasai, David Gray, Carol Clugston, Anna Dominiczak and Alex Alderton, Roberto Amato, Sonia Goncalves, Ewan Harrison, David K. Jackson, Ian Johnston, Dominic Kwiatkowski, Cordelia Langford, John Sillitoe on behalf of the Wellcome Sanger Institute COVID-19 Surveillance Team
EPI_ISL_731460, EPI_ISL_731461, EPI_ISL_731462, EPI_ISL_731463, EPI_ISL_731465	Lighthouse Lab in Alderley Park	Wellcome Sanger Institute for the COVID-19 Genomics UK (COG-UK) Consortium	Jacquelyn Wynn, Mairead Hyland, The Lighthouse Lab in Alderley Park and Alex Alderton, Roberto Amato, Sonia Goncalves, Ewan Harrison, David K. Jackson, Ian Johnston, Dominic Kwiatkowski, Cordelia Langford, John Sillitoe on behalf of the Wellcome Sanger Institute COVID-19 Surveillance Team
EPI_ISL_731466	Lighthouse Lab in Glasgow	Wellcome Sanger Institute for the COVID-19 Genomics UK (COG-UK) Consortium	Harper VanSteenhouse, Yumi Kasai, David Gray, Carol Clugston, Anna Dominiczak and Alex Alderton, Roberto Amato, Sonia Goncalves, Ewan Harrison, David K. Jackson, Ian Johnston, Dominic Kwiatkowski, Cordelia Langford, John Sillitoe on behalf of the Wellcome Sanger Institute COVID-19 Surveillance Team
EPI_ISL_731468, EPI_ISL_731470, EPI_ISL_731473, EPI_ISL_731474, EPI_ISL_731475	Lighthouse Lab in Alderley Park	Wellcome Sanger Institute for the COVID-19 Genomics UK (COG-UK) Consortium	Jacquelyn Wynn, Mairead Hyland, The Lighthouse Lab in Alderley Park and Alex Alderton, Roberto Amato, Sonia Goncalves, Ewan Harrison, David K. Jackson, Ian Johnston, Dominic Kwiatkowski, Cordelia Langford, John Sillitoe on behalf of the Wellcome Sanger Institute COVID-19 Surveillance Team
EPI_ISL_731476, EPI_ISL_731477	Lighthouse Lab in Glasgow	Wellcome Sanger Institute for the COVID-19 Genomics UK (COG-UK) Consortium	Harper VanSteenhouse, Yumi Kasai, David Gray, Carol Clugston, Anna Dominiczak and Alex Alderton, Roberto Amato, Sonia Goncalves, Ewan Harrison, David K. Jackson, Ian Johnston, Dominic Kwiatkowski, Cordelia Langford, John Sillitoe on behalf of the Wellcome Sanger Institute COVID-19 Surveillance Team
EPI_ISL_731481, EPI_ISL_731483	Lighthouse Lab in Alderley Park	Wellcome Sanger Institute for the COVID-19 Genomics UK (COG-UK) Consortium	Jacquelyn Wynn, Mairead Hyland, The Lighthouse Lab in Alderley Park and Alex Alderton, Roberto Amato, Sonia Goncalves, Ewan Harrison, David K. Jackson, Ian Johnston, Dominic Kwiatkowski, Cordelia Langford, John Sillitoe on behalf of the Wellcome Sanger Institute COVID-19 Surveillance Team
EPI_ISL_731484, EPI_ISL_731485, EPI_ISL_731486, EPI_ISL_731488	Lighthouse Lab in Glasgow	Wellcome Sanger Institute for the COVID-19 Genomics UK (COG-UK) Consortium	Harper VanSteenhouse, Yumi Kasai, David Gray, Carol Clugston, Anna Dominiczak and Alex Alderton, Roberto Amato, Sonia Goncalves, Ewan Harrison, David K. Jackson, Ian Johnston, Dominic Kwiatkowski, Cordelia Langford, John Sillitoe on behalf of the Wellcome Sanger Institute COVID-19 Surveillance Team
EPI_ISL_731489	Lighthouse Lab in Alderley Park	Wellcome Sanger Institute for the COVID-19 Genomics UK (COG-UK) Consortium	Jacquelyn Wynn, Mairead Hyland, The Lighthouse Lab in Alderley Park and Alex Alderton, Roberto Amato, Sonia Goncalves, Ewan Harrison, David K. Jackson, Ian Johnston, Dominic Kwiatkowski, Cordelia Langford, John Sillitoe on behalf of the Wellcome Sanger Institute COVID-19 Surveillance Team
EPI_ISL_731491	Lighthouse Lab in Glasgow	Wellcome Sanger Institute for the COVID-19 Genomics UK (COG-UK) Consortium	Harper VanSteenhouse, Yumi Kasai, David Gray, Carol Clugston, Anna Dominiczak and Alex Alderton, Roberto Amato, Sonia Goncalves, Ewan Harrison, David K. Jackson, Ian Johnston, Dominic Kwiatkowski, Cordelia Langford, John Sillitoe on behalf of the Wellcome Sanger Institute COVID-19 Surveillance Team

EPI_ISL_731492	Lighthouse Lab in Alderley Park	Wellcome Sanger Institute for the COVID-19 Genomics UK (COG-UK) Consortium	Jacquelyn Wynn, Mairead Hyland, The Lighthouse Lab in Alderley Park and Alex Alderton, Roberto Amato, Sonia Goncalves, Ewan Harrison, David K. Jackson, Ian Johnston, Dominic Kwiatkowski, Cordelia Langford, John Sillitoe on behalf of the Wellcome Sanger Institute COVID-19 Surveillance Team
EPI_ISL_731493, EPI_ISL_731494	Lighthouse Lab in Glasgow	Wellcome Sanger Institute for the COVID-19 Genomics UK (COG-UK) Consortium	Harper VanSteenhouse, Yumi Kasai, David Gray, Carol Clugston, Anna Dominiczak and Alex Alderton, Roberto Amato, Sonia Goncalves, Ewan Harrison, David K. Jackson, Ian Johnston, Dominic Kwiatkowski, Cordelia Langford, John Sillitoe on behalf of the Wellcome Sanger Institute COVID-19 Surveillance Team
EPI_ISL_731495	Lighthouse Lab in Alderley Park	Wellcome Sanger Institute for the COVID-19 Genomics UK (COG-UK) Consortium	Jacquelyn Wynn, Mairead Hyland, The Lighthouse Lab in Alderley Park and Alex Alderton, Roberto Amato, Sonia Goncalves, Ewan Harrison, David K. Jackson, Ian Johnston, Dominic Kwiatkowski, Cordelia Langford, John Sillitoe on behalf of the Wellcome Sanger Institute COVID-19 Surveillance Team
EPI_ISL_731496, EPI_ISL_731497	Lighthouse Lab in Glasgow	Wellcome Sanger Institute for the COVID-19 Genomics UK (COG-UK) Consortium	Harper VanSteenhouse, Yumi Kasai, David Gray, Carol Clugston, Anna Dominiczak and Alex Alderton, Roberto Amato, Sonia Goncalves, Ewan Harrison, David K. Jackson, Ian Johnston, Dominic Kwiatkowski, Cordelia Langford, John Sillitoe on behalf of the Wellcome Sanger Institute COVID-19 Surveillance Team
EPI_ISL_731498	Lighthouse Lab in Alderley Park	Wellcome Sanger Institute for the COVID-19 Genomics UK (COG-UK) Consortium	Jacquelyn Wynn, Mairead Hyland, The Lighthouse Lab in Alderley Park and Alex Alderton, Roberto Amato, Sonia Goncalves, Ewan Harrison, David K. Jackson, Ian Johnston, Dominic Kwiatkowski, Cordelia Langford, John Sillitoe on behalf of the Wellcome Sanger Institute COVID-19 Surveillance Team
EPI_ISL_731499	Lighthouse Lab in Glasgow	Wellcome Sanger Institute for the COVID-19 Genomics UK (COG-UK) Consortium	Harper VanSteenhouse, Yumi Kasai, David Gray, Carol Clugston, Anna Dominiczak and Alex Alderton, Roberto Amato, Sonia Goncalves, Ewan Harrison, David K. Jackson, Ian Johnston, Dominic Kwiatkowski, Cordelia Langford, John Sillitoe on behalf of the Wellcome Sanger Institute COVID-19 Surveillance Team
EPI_ISL_731500	Lighthouse Lab in Alderley Park	Wellcome Sanger Institute for the COVID-19 Genomics UK (COG-UK) Consortium	Jacquelyn Wynn, Mairead Hyland, The Lighthouse Lab in Alderley Park and Alex Alderton, Roberto Amato, Sonia Goncalves, Ewan Harrison, David K. Jackson, Ian Johnston, Dominic Kwiatkowski, Cordelia Langford, John Sillitoe on behalf of the Wellcome Sanger Institute COVID-19 Surveillance Team
EPI_ISL_731501	Lighthouse Lab in Glasgow	Wellcome Sanger Institute for the COVID-19 Genomics UK (COG-UK) Consortium	Harper VanSteenhouse, Yumi Kasai, David Gray, Carol Clugston, Anna Dominiczak and Alex Alderton, Roberto Amato, Sonia Goncalves, Ewan Harrison, David K. Jackson, Ian Johnston, Dominic Kwiatkowski, Cordelia Langford, John Sillitoe on behalf of the Wellcome Sanger Institute COVID-19 Surveillance Team
EPI_ISL_731502, EPI_ISL_731503, EPI_ISL_731506	Lighthouse Lab in Alderley Park	Wellcome Sanger Institute for the COVID-19 Genomics UK (COG-UK) Consortium	Jacquelyn Wynn, Mairead Hyland, The Lighthouse Lab in Alderley Park and Alex Alderton, Roberto Amato, Sonia Goncalves, Ewan Harrison, David K. Jackson, Ian Johnston, Dominic Kwiatkowski, Cordelia Langford, John Sillitoe on behalf of the Wellcome Sanger Institute COVID-19 Surveillance Team
EPI_ISL_731507	Lighthouse Lab in Glasgow	Wellcome Sanger Institute for the COVID-19 Genomics UK (COG-UK) Consortium	Harper VanSteenhouse, Yumi Kasai, David Gray, Carol Clugston, Anna Dominiczak and Alex Alderton, Roberto Amato, Sonia Goncalves, Ewan Harrison, David K. Jackson, Ian Johnston, Dominic Kwiatkowski, Cordelia Langford, John Sillitoe on behalf of the Wellcome Sanger Institute COVID-19 Surveillance Team
EPI_ISL_731510, EPI_ISL_731511, EPI_ISL_731512, EPI_ISL_731514, EPI_ISL_731516	Lighthouse Lab in Alderley Park	Wellcome Sanger Institute for the COVID-19 Genomics UK (COG-UK) Consortium	Jacquelyn Wynn, Mairead Hyland, The Lighthouse Lab in Alderley Park and Alex Alderton, Roberto Amato, Sonia Goncalves, Ewan Harrison, David K. Jackson, Ian Johnston, Dominic Kwiatkowski, Cordelia Langford, John Sillitoe on behalf of the Wellcome Sanger Institute COVID-19 Surveillance Team
EPI_ISL_731519	Lighthouse Lab in Glasgow	Wellcome Sanger Institute for the COVID-19 Genomics UK (COG-UK) Consortium	Harper VanSteenhouse, Yumi Kasai, David Gray, Carol Clugston, Anna Dominiczak and Alex Alderton, Roberto Amato, Sonia Goncalves, Ewan Harrison, David K. Jackson, Ian Johnston, Dominic Kwiatkowski, Cordelia Langford, John Sillitoe on behalf of the Wellcome Sanger Institute COVID-19 Surveillance Team
EPI_ISL_731520, EPI_ISL_731521, EPI_ISL_731525, EPI_ISL_731526, EPI_ISL_731527, EPI_ISL_731528, EPI_ISL_731530, EPI_ISL_731532, EPI_ISL_731534	Lighthouse Lab in Alderley Park	Wellcome Sanger Institute for the COVID-19 Genomics UK (COG-UK) Consortium	Jacquelyn Wynn, Mairead Hyland, The Lighthouse Lab in Alderley Park and Alex Alderton, Roberto Amato, Sonia Goncalves, Ewan Harrison, David K. Jackson, Ian Johnston, Dominic Kwiatkowski, Cordelia Langford, John Sillitoe on behalf of the Wellcome Sanger Institute COVID-19 Surveillance Team
EPI_ISL_731535, EPI_ISL_731536	Lighthouse Lab in Glasgow	Wellcome Sanger Institute for the COVID-19 Genomics UK (COG-UK) Consortium	Harper VanSteenhouse, Yumi Kasai, David Gray, Carol Clugston, Anna Dominiczak and Alex Alderton, Roberto Amato, Sonia Goncalves, Ewan Harrison, David K. Jackson, Ian Johnston, Dominic Kwiatkowski, Cordelia Langford, John Sillitoe on behalf of the Wellcome Sanger Institute COVID-19 Surveillance Team
EPI_ISL_731539, EPI_ISL_731540	Lighthouse Lab in Alderley Park	Wellcome Sanger Institute for the COVID-19 Genomics UK (COG-UK) Consortium	Jacquelyn Wynn, Mairead Hyland, The Lighthouse Lab in Alderley Park and Alex Alderton, Roberto Amato, Sonia Goncalves, Ewan Harrison, David K. Jackson, Ian Johnston, Dominic Kwiatkowski, Cordelia Langford, John Sillitoe on behalf of the Wellcome Sanger Institute COVID-19 Surveillance Team
EPI_ISL_731541	Lighthouse Lab in Glasgow	Wellcome Sanger Institute for the COVID-19 Genomics UK (COG-UK) Consortium	Harper VanSteenhouse, Yumi Kasai, David Gray, Carol Clugston, Anna Dominiczak and Alex Alderton, Roberto Amato, Sonia Goncalves, Ewan Harrison, David K. Jackson, Ian Johnston, Dominic Kwiatkowski, Cordelia Langford, John Sillitoe on behalf of the Wellcome Sanger Institute COVID-19 Surveillance Team
EPI_ISL_731542, EPI_ISL_731543, EPI_ISL_731545, EPI_ISL_731547	Lighthouse Lab in Alderley Park	Wellcome Sanger Institute for the COVID-19 Genomics UK (COG-UK) Consortium	Jacquelyn Wynn, Mairead Hyland, The Lighthouse Lab in Alderley Park and Alex Alderton, Roberto Amato, Sonia Goncalves, Ewan Harrison, David K. Jackson, Ian Johnston, Dominic Kwiatkowski, Cordelia Langford, John Sillitoe on behalf of the Wellcome Sanger Institute COVID-19 Surveillance Team
EPI_ISL_731548	Lighthouse Lab in Glasgow	Wellcome Sanger Institute for the COVID-19 Genomics UK (COG-UK) Consortium	Harper VanSteenhouse, Yumi Kasai, David Gray, Carol Clugston, Anna Dominiczak and Alex Alderton, Roberto Amato, Sonia Goncalves, Ewan Harrison, David K. Jackson, Ian Johnston, Dominic Kwiatkowski, Cordelia Langford, John Sillitoe on behalf of the Wellcome Sanger Institute COVID-19 Surveillance Team
EPI_ISL_731549	Lighthouse Lab in Alderley Park	Wellcome Sanger Institute for the COVID-19 Genomics UK (COG-UK) Consortium	Jacquelyn Wynn, Mairead Hyland, The Lighthouse Lab in Alderley Park and Alex Alderton, Roberto Amato, Sonia Goncalves, Ewan Harrison, David K. Jackson, Ian Johnston, Dominic Kwiatkowski, Cordelia Langford, John Sillitoe on behalf of the Wellcome Sanger Institute COVID-19 Surveillance Team
EPI_ISL_731550, EPI_ISL_731551	Lighthouse Lab in Glasgow	Wellcome Sanger Institute for the COVID-19 Genomics UK (COG-UK) Consortium	Harper VanSteenhouse, Yumi Kasai, David Gray, Carol Clugston, Anna Dominiczak and Alex Alderton, Roberto Amato, Sonia Goncalves, Ewan Harrison, David K. Jackson, Ian Johnston, Dominic Kwiatkowski, Cordelia Langford, John Sillitoe on behalf of the Wellcome Sanger Institute COVID-19 Surveillance Team
EPI_ISL_731553, EPI_ISL_731554, EPI_ISL_731558, EPI_ISL_731561, EPI_ISL_731562, EPI_ISL_731564, EPI_ISL_731565, EPI_ISL_731567, EPI_ISL_731568, EPI_ISL_731572, EPI_ISL_731573, EPI_ISL_731574, EPI_ISL_731575, EPI_ISL_731577, EPI_ISL_731578, EPI_ISL_731580, EPI_ISL_731581, EPI_ISL_731582, EPI_ISL_731583, EPI_ISL_731584, EPI_ISL_731585, EPI_ISL_731587, EPI_ISL_731588, EPI_ISL_731592, EPI_ISL_731598, EPI_ISL_731599, EPI_ISL_731601, EPI_ISL_731604, EPI_ISL_731606, EPI_ISL_731607, EPI_ISL_731609, EPI_ISL_731611, EPI_ISL_731613, EPI_ISL_731614, EPI_ISL_731615, EPI_ISL_731616, EPI_ISL_731617, EPI_ISL_731618, EPI_ISL_731619, EPI_ISL_731622, EPI_ISL_731623, EPI_ISL_731625, EPI_ISL_731627, EPI_ISL_731629, EPI_ISL_731630, EPI_ISL_731633, EPI_ISL_731634, EPI_ISL_731635, EPI_ISL_731636, EPI_ISL_731637, EPI_ISL_731638, EPI_ISL_731639, EPI_ISL_731640, EPI_ISL_731641, EPI_ISL_731642, EPI_ISL_731643, EPI_ISL_731644, EPI_ISL_731646, EPI_ISL_731647, EPI_ISL_731648, EPI_ISL_731649, EPI_ISL_731652, EPI_ISL_731653, EPI_ISL_731654, EPI_ISL_731655, EPI_ISL_731656, EPI_ISL_731659, EPI_ISL_731660, EPI_ISL_731661, EPI_ISL_731663, EPI_ISL_731664, EPI_ISL_731665, EPI_ISL_731667, EPI_ISL_731669, EPI_ISL_731671, EPI_ISL_731672, EPI_ISL_731673, EPI_ISL_731675, EPI_ISL_731677, EPI_ISL_731678, EPI_ISL_731679, EPI_ISL_731680, EPI_ISL_731682, EPI_ISL_731683, EPI_ISL_731684, EPI_ISL_731686, EPI_ISL_731688, EPI_ISL_731689, EPI_ISL_731691, EPI_ISL_731693, EPI_ISL_731694, EPI_ISL_731696, EPI_ISL_731697, EPI_ISL_731698, EPI_ISL_731699, EPI_ISL_731700, EPI_ISL_731701, EPI_ISL_731702, EPI_ISL_731703, EPI_ISL_731704, EPI_ISL_731707, EPI_ISL_731708, EPI_ISL_731709, EPI_ISL_731711, EPI_ISL_731712, EPI_ISL_731717, EPI_ISL_731718, EPI_ISL_731720, EPI_ISL_731721, EPI_ISL_731724, EPI_ISL_731727, EPI_ISL_731728, EPI_ISL_731730, EPI_ISL_731731, EPI_ISL_731732, EPI_ISL_731733, EPI_ISL_731736, EPI_ISL_731737, EPI_ISL_731738, EPI_ISL_731739, EPI_ISL_731740, EPI_ISL_731741, EPI_ISL_731742, EPI_ISL_731743, EPI_ISL_731744, EPI_ISL_731745, EPI_ISL_731747, EPI_ISL_731749, EPI_ISL_731750, EPI_ISL_731751, EPI_ISL_731752, EPI_ISL_731753, EPI_ISL_731754, EPI_ISL_731755, EPI_ISL_731756, EPI_ISL_731757, EPI_ISL_731758, EPI_ISL_731760, EPI_ISL_731761, EPI_ISL_731762, EPI_ISL_731765, EPI_ISL_731767, EPI_ISL_731769, EPI_ISL_731770, EPI_ISL_731771, EPI_ISL_731774, EPI_ISL_731775, EPI_ISL_731776, EPI_ISL_731777, EPI_ISL_731778, EPI_ISL_731779, EPI_ISL_731780, EPI_ISL_731782, EPI_ISL_731784, EPI_ISL_731786, EPI_ISL_731788, EPI_ISL_731789, EPI_ISL_731790, EPI_ISL_731792, EPI_ISL_731794, EPI_ISL_731795, EPI_ISL_731796, EPI_ISL_731798, EPI_ISL_731799, EPI_ISL_731801, EPI_ISL_731804, EPI_ISL_731805, EPI_ISL_731806, EPI_ISL_731808, EPI_ISL_731809, EPI_ISL_731810, EPI_ISL_731812, EPI_ISL_731813, EPI_ISL_731814, EPI_ISL_731816, EPI_ISL_731817, EPI_ISL_731818, EPI_ISL_731819, EPI_ISL_731820, EPI_ISL_731823, EPI_ISL_731825, EPI_ISL_731827, EPI_ISL_731828, EPI_ISL_731829, EPI_ISL_731830, EPI_ISL_731832, EPI_ISL_731833, EPI_ISL_731834, EPI_ISL_731835, EPI_ISL_731836, EPI_ISL_731837, EPI_ISL_731839, EPI_ISL_731840, EPI_ISL_731841, EPI_ISL_731842, EPI_ISL_731843, EPI_ISL_731844, EPI_ISL_731845, EPI_ISL_731846, EPI_ISL_731849, EPI_ISL_731850, EPI_ISL_731852, EPI_ISL_731853, EPI_ISL_731854, EPI_ISL_731857, EPI_ISL_731859, EPI_ISL_731860, EPI_ISL_731862, EPI_ISL_731863, EPI_ISL_731865, EPI_ISL_731867, EPI_ISL_731868, EPI_ISL_731869, EPI_ISL_731870, EPI_ISL_731872, EPI_ISL_731873, EPI_ISL_731874, EPI_ISL_731875, EPI_ISL_731876, EPI_ISL_731878, EPI_ISL_731879, EPI_ISL_731880, EPI_ISL_731882, EPI_ISL_731883, EPI_ISL_731884, EPI_ISL_731886, EPI_ISL_731887, EPI_ISL_731888, EPI_ISL_731890			
see above	Lighthouse Lab in Alderley Park	Wellcome Sanger Institute for the COVID-19 Genomics UK (COG-UK) Consortium	Jacquelyn Wynn, Mairead Hyland, The Lighthouse Lab in Alderley Park and Alex Alderton, Roberto Amato, Sonia Goncalves, Ewan Harrison, David K. Jackson, Ian Johnston, Dominic Kwiatkowski, Cordelia Langford, John Sillitoe on behalf of the Wellcome Sanger Institute COVID-19 Surveillance Team
EPI_ISL_732114, EPI_ISL_732115, EPI_ISL_732116	Instituto Nacional de Saude (INSA)	Instituto Nacional de Saude (INSA)	Borges et al
EPI_ISL_732516	National Virus Reference Laboratory	National Virus Reference Laboratory	Michael Carr, Gabriel Gonzalez, Jonathan Dean, Daniel Hare, Cillian F De Gascun
EPI_ISL_732570, EPI_ISL_732572, EPI_ISL_732574, EPI_ISL_732575, EPI_ISL_732576, EPI_ISL_732577, EPI_ISL_732578, EPI_ISL_732579, EPI_ISL_732580, EPI_ISL_732583, EPI_ISL_732584, EPI_ISL_732585, EPI_ISL_732586, EPI_ISL_732587, EPI_ISL_732588, EPI_ISL_732589, EPI_ISL_732590, EPI_ISL_732591, EPI_ISL_732592, EPI_ISL_732593, EPI_ISL_732594, EPI_ISL_732595, EPI_ISL_732596, EPI_ISL_732598, EPI_ISL_732600, EPI_ISL_732604, EPI_ISL_732605, EPI_ISL_732606, EPI_ISL_732608, EPI_ISL_732609, EPI_ISL_732610, EPI_ISL_732612, EPI_ISL_732615, EPI_ISL_732616, EPI_ISL_732617, EPI_ISL_732619, EPI_ISL_732620, EPI_ISL_732621, EPI_ISL_732622, EPI_ISL_732623, EPI_ISL_732624			

see above	Department of Virology and Immunology, University of Helsinki and Helsinki University Hospital, Huslab Finland	Department of Virology, Faculty of Medicine, University of Helsinki, Helsinki, Finland	Teemu Smura, Ravi Kant, Phuoc Truong, Hussein Alburkat, Hannimari Kallio-Kokko, Jenni Virtanen, Maija Suvanto, Sari Hannula, Harri Kangas, Pekka Ellonen, Olli Vapalahti
EPI_ISL_732656	Narhalsan Fjallbacka VC	The Public Health Agency of Sweden	Department of Microbiology, The Public Health Agency of Sweden
EPI_ISL_732657	Barnakuten	The Public Health Agency of Sweden	Department of Microbiology, The Public Health Agency of Sweden
EPI_ISL_732964, EPI_ISL_732967	LabPLUS	Institute of Environmental Science and Research (ESR)	Xiaoyun Ren, Matt Storey, Nikki Freed, Muhammad Faisal, Jing Wang, Hermes Perez, Anja Werno, Antje van der Linden, Arlo Upton, Chris Mansell, David Hammer, Dragana Drinkovic, Gary McAuliffe, Hana Sofia Andersson, James Ussher, Jill Sherwood, Josh Freeman, Julia Howard, Juliet Elvy, Mary DeAlmeida, Matt Blakiston, Matthew Rogers, Max Bloomfield, Michael Addidle, Michelle Balm, Sally Roberts, Sarah Jefferies, Sharmini Muttaiyah, Susan Morpeth, Susan Taylor, Timothy Blackmore, Vani Sathyendran, Veronica Playle, Virginia Hope, Erasmus Smit, Lauren Jelly, Olin Silander, Joep de Ligt
EPI_ISL_733499	1-Laboratory of Microbiology, National Reference Lab, Charles Nicolle Hospital; 2-University of Tunis ElManar, Faculty of Medicine of Tunis, LR99ES09, Tunis, Tunisia	1-Clinical and Experimental Pharmacology Lab, LR16SP02, National Center of Pharmacovigilance, University of Tunis El Manar, Tunis, Tunisia. 2-Neurodegenerative diseases and psychiatric troubles, LR18SP03, Razi Hospital, University of Tunis El Manar, Tunis, Tunisia. 3- Ministry of Health, National Observatory of New and Emerging Diseases, 1006, Tunis, Tunisia	Ilhem Boutiba-Ben Boubaker, Sameh Trabelsi, Nissaf Ben Alaya, Maher Kharrat, Alia Ben Kahla, Jalila Ben Khelil, Salma Abid, Sana Ferjani, Mouna Ben Sassi, Mouna Safer, Guedi Ali Barreh, Habiba Ben Romdhane, Souissi Amira, Sarra Chamman, Hanen El Jebari, Asma Ferjani, Gaies Emna, Riadh Daghfous, Riadh Gouider.
EPI_ISL_733572, EPI_ISL_733573, EPI_ISL_733575	Temporary Specimen Collection Centre	Hong Kong Department of Health	Alan K.L. Tsang, Peter C.W. Yip, Edman T.K. Lam, Rickjason C.W. Chan, Dominic N.C. Tsang
EPI_ISL_733593	Respiratory Virus Unit, National Infection Service, Public Health England	COVID-19 Genomics UK (COG-UK) Consortium	PHE Covid Sequencing Team
EPI_ISL_733598	Lighthouse Lab in Alderley Park	Wellcome Sanger Institute for the COVID-19 Genomics UK (COG-UK) Consortium	Jacquelyn Wynn, Mairead Hyland, The Lighthouse Lab in Alderley Park and Alex Alderton, Roberto Amato, Sonia Goncalves, Ewan Harrison, David K. Jackson, Ian Johnston, Dominic Kwiatkowski, Cordelia Langford, John Sillitoe on behalf of the Wellcome Sanger Institute COVID-19 Surveillance Team
EPI_ISL_733599, EPI_ISL_733601	Lighthouse Lab in Cambridge	Wellcome Sanger Institute for the COVID-19 Genomics UK (COG-UK) Consortium	Rob Howes, The Lighthouse Lab in Cambridge and Alex Alderton, Roberto Amato, Sonia Goncalves, Ewan Harrison, David K. Jackson, Ian Johnston, Dominic Kwiatkowski, Cordelia Langford, John Sillitoe on behalf of the Wellcome Sanger Institute COVID-19 Surveillance Team
EPI_ISL_733603	Lighthouse Lab in Alderley Park	Wellcome Sanger Institute for the COVID-19 Genomics UK (COG-UK) Consortium	Jacquelyn Wynn, Mairead Hyland, The Lighthouse Lab in Alderley Park and Alex Alderton, Roberto Amato, Sonia Goncalves, Ewan Harrison, David K. Jackson, Ian Johnston, Dominic Kwiatkowski, Cordelia Langford, John Sillitoe on behalf of the Wellcome Sanger Institute COVID-19 Surveillance Team
EPI_ISL_733604	Lighthouse Lab in Cambridge	Wellcome Sanger Institute for the COVID-19 Genomics UK (COG-UK) Consortium	Rob Howes, The Lighthouse Lab in Cambridge and Alex Alderton, Roberto Amato, Sonia Goncalves, Ewan Harrison, David K. Jackson, Ian Johnston, Dominic Kwiatkowski, Cordelia Langford, John Sillitoe on behalf of the Wellcome Sanger Institute COVID-19 Surveillance Team
EPI_ISL_733607	Lighthouse Lab in Milton Keynes	Wellcome Sanger Institute for the COVID-19 Genomics UK (COG-UK) Consortium	The Lighthouse Lab in Milton Keynes and Alex Alderton, Roberto Amato, Sonia Goncalves, Ewan Harrison, David K. Jackson, Ian Johnston, Dominic Kwiatkowski, Cordelia Langford, John Sillitoe on behalf of the Wellcome Sanger Institute COVID-19 Surveillance Team
EPI_ISL_733610	Lighthouse Lab in Cambridge	Wellcome Sanger Institute for the COVID-19 Genomics UK (COG-UK) Consortium	Rob Howes, The Lighthouse Lab in Cambridge and Alex Alderton, Roberto Amato, Sonia Goncalves, Ewan Harrison, David K. Jackson, Ian Johnston, Dominic Kwiatkowski, Cordelia Langford, John Sillitoe on behalf of the Wellcome Sanger Institute COVID-19 Surveillance Team
EPI_ISL_733611, EPI_ISL_733613	Lighthouse Lab in Alderley Park	Wellcome Sanger Institute for the COVID-19 Genomics UK (COG-UK) Consortium	Jacquelyn Wynn, Mairead Hyland, The Lighthouse Lab in Alderley Park and Alex Alderton, Roberto Amato, Sonia Goncalves, Ewan Harrison, David K. Jackson, Ian Johnston, Dominic Kwiatkowski, Cordelia Langford, John Sillitoe on behalf of the Wellcome Sanger Institute COVID-19 Surveillance Team
EPI_ISL_733614	Lighthouse Lab in Cambridge	Wellcome Sanger Institute for the COVID-19 Genomics UK (COG-UK) Consortium	Rob Howes, The Lighthouse Lab in Cambridge and Alex Alderton, Roberto Amato, Sonia Goncalves, Ewan Harrison, David K. Jackson, Ian Johnston, Dominic Kwiatkowski, Cordelia Langford, John Sillitoe on behalf of the Wellcome Sanger Institute COVID-19 Surveillance Team
EPI_ISL_733617	Lighthouse Lab in Milton Keynes	Wellcome Sanger Institute for the COVID-19 Genomics UK (COG-UK) Consortium	The Lighthouse Lab in Milton Keynes and Alex Alderton, Roberto Amato, Sonia Goncalves, Ewan Harrison, David K. Jackson, Ian Johnston, Dominic Kwiatkowski, Cordelia Langford, John Sillitoe on behalf of the Wellcome Sanger Institute COVID-19 Surveillance Team
EPI_ISL_733618	Lighthouse Lab in Alderley Park	Wellcome Sanger Institute for the COVID-19 Genomics UK (COG-UK) Consortium	Jacquelyn Wynn, Mairead Hyland, The Lighthouse Lab in Alderley Park and Alex Alderton, Roberto Amato, Sonia Goncalves, Ewan Harrison, David K. Jackson, Ian Johnston, Dominic Kwiatkowski, Cordelia Langford, John Sillitoe on behalf of the Wellcome Sanger Institute COVID-19 Surveillance Team
EPI_ISL_733619	Lighthouse Lab in Cambridge	Wellcome Sanger Institute for the COVID-19 Genomics UK (COG-UK) Consortium	Rob Howes, The Lighthouse Lab in Cambridge and Alex Alderton, Roberto Amato, Sonia Goncalves, Ewan Harrison, David K. Jackson, Ian Johnston, Dominic Kwiatkowski, Cordelia Langford, John Sillitoe on behalf of the Wellcome Sanger Institute COVID-19 Surveillance Team
EPI_ISL_733621	Lighthouse Lab in Alderley Park	Wellcome Sanger Institute for the COVID-19 Genomics UK (COG-UK) Consortium	Jacquelyn Wynn, Mairead Hyland, The Lighthouse Lab in Alderley Park and Alex Alderton, Roberto Amato, Sonia Goncalves, Ewan Harrison, David K. Jackson, Ian Johnston, Dominic Kwiatkowski, Cordelia Langford, John Sillitoe on behalf of the Wellcome Sanger Institute COVID-19 Surveillance Team
EPI_ISL_733622, EPI_ISL_733624, EPI_ISL_733626, EPI_ISL_733627	Lighthouse Lab in Cambridge	Wellcome Sanger Institute for the COVID-19 Genomics UK (COG-UK) Consortium	Rob Howes, The Lighthouse Lab in Cambridge and Alex Alderton, Roberto Amato, Sonia Goncalves, Ewan Harrison, David K. Jackson, Ian Johnston, Dominic Kwiatkowski, Cordelia Langford, John Sillitoe on behalf of the Wellcome Sanger Institute COVID-19 Surveillance Team
EPI_ISL_733628	Lighthouse Lab in Milton Keynes	Wellcome Sanger Institute for the COVID-19 Genomics UK (COG-UK) Consortium	The Lighthouse Lab in Milton Keynes and Alex Alderton, Roberto Amato, Sonia Goncalves, Ewan Harrison, David K. Jackson, Ian Johnston, Dominic Kwiatkowski, Cordelia Langford, John Sillitoe on behalf of the Wellcome Sanger Institute COVID-19 Surveillance Team
EPI_ISL_733629, EPI_ISL_733631, EPI_ISL_733632, EPI_ISL_733633	Lighthouse Lab in Cambridge	Wellcome Sanger Institute for the COVID-19 Genomics UK (COG-UK) Consortium	Rob Howes, The Lighthouse Lab in Cambridge and Alex Alderton, Roberto Amato, Sonia Goncalves, Ewan Harrison, David K. Jackson, Ian Johnston, Dominic Kwiatkowski, Cordelia Langford, John Sillitoe on behalf of the Wellcome Sanger Institute COVID-19 Surveillance Team
EPI_ISL_733636	Lighthouse Lab in Milton Keynes	Wellcome Sanger Institute for the COVID-19 Genomics UK (COG-UK) Consortium	The Lighthouse Lab in Milton Keynes and Alex Alderton, Roberto Amato, Sonia Goncalves, Ewan Harrison, David K. Jackson, Ian Johnston, Dominic Kwiatkowski, Cordelia Langford, John Sillitoe on behalf of the Wellcome Sanger Institute COVID-19 Surveillance Team
EPI_ISL_733637, EPI_ISL_733640	Lighthouse Lab in Cambridge	Wellcome Sanger Institute for the COVID-19 Genomics UK (COG-UK) Consortium	Rob Howes, The Lighthouse Lab in Cambridge and Alex Alderton, Roberto Amato, Sonia Goncalves, Ewan Harrison, David K. Jackson, Ian Johnston, Dominic Kwiatkowski, Cordelia Langford, John Sillitoe on behalf of the Wellcome Sanger Institute COVID-19 Surveillance Team
EPI_ISL_733641, EPI_ISL_733643	Lighthouse Lab in Alderley Park	Wellcome Sanger Institute for the COVID-19 Genomics UK (COG-UK) Consortium	Jacquelyn Wynn, Mairead Hyland, The Lighthouse Lab in Alderley Park and Alex Alderton, Roberto Amato, Sonia Goncalves, Ewan Harrison, David K. Jackson, Ian Johnston, Dominic Kwiatkowski, Cordelia Langford, John Sillitoe on behalf of the Wellcome Sanger Institute COVID-19 Surveillance Team
EPI_ISL_733644, EPI_ISL_733648	Lighthouse Lab in Milton Keynes	Wellcome Sanger Institute for the COVID-19 Genomics UK (COG-UK) Consortium	The Lighthouse Lab in Milton Keynes and Alex Alderton, Roberto Amato, Sonia Goncalves, Ewan Harrison, David K. Jackson, Ian Johnston, Dominic Kwiatkowski, Cordelia Langford, John Sillitoe on behalf of the Wellcome Sanger Institute COVID-19 Surveillance Team
EPI_ISL_733649, EPI_ISL_733650, EPI_ISL_733655	Lighthouse Lab in Cambridge	Wellcome Sanger Institute for the COVID-19 Genomics UK (COG-UK) Consortium	Rob Howes, The Lighthouse Lab in Cambridge and Alex Alderton, Roberto Amato, Sonia Goncalves, Ewan Harrison, David K. Jackson, Ian Johnston, Dominic Kwiatkowski, Cordelia Langford, John Sillitoe on behalf of the Wellcome Sanger Institute COVID-19 Surveillance Team
EPI_ISL_733657	Lighthouse Lab in Milton Keynes	Wellcome Sanger Institute for the COVID-19 Genomics UK (COG-UK) Consortium	The Lighthouse Lab in Milton Keynes and Alex Alderton, Roberto Amato, Sonia Goncalves, Ewan Harrison, David K. Jackson, Ian Johnston, Dominic Kwiatkowski, Cordelia Langford, John Sillitoe on behalf of the Wellcome Sanger Institute COVID-19 Surveillance Team
EPI_ISL_733660, EPI_ISL_733661, EPI_ISL_733662, EPI_ISL_733663, EPI_ISL_733665	Lighthouse Lab in Cambridge	Wellcome Sanger Institute for the COVID-19 Genomics UK (COG-UK) Consortium	Rob Howes, The Lighthouse Lab in Cambridge and Alex Alderton, Roberto Amato, Sonia Goncalves, Ewan Harrison, David K. Jackson, Ian Johnston, Dominic Kwiatkowski, Cordelia Langford, John Sillitoe on behalf of the Wellcome Sanger Institute COVID-19 Surveillance Team
EPI_ISL_733666	Lighthouse Lab in Milton Keynes	Wellcome Sanger Institute for the COVID-19 Genomics UK (COG-UK) Consortium	The Lighthouse Lab in Milton Keynes and Alex Alderton, Roberto Amato, Sonia Goncalves, Ewan Harrison, David K. Jackson, Ian Johnston, Dominic Kwiatkowski, Cordelia Langford, John Sillitoe on behalf of the Wellcome Sanger Institute COVID-19 Surveillance Team
EPI_ISL_733668	Lighthouse Lab in Cambridge	Wellcome Sanger Institute for the COVID-19 Genomics UK (COG-UK) Consortium	Rob Howes, The Lighthouse Lab in Cambridge and Alex Alderton, Roberto Amato, Sonia Goncalves, Ewan Harrison, David K. Jackson, Ian Johnston, Dominic Kwiatkowski, Cordelia Langford, John Sillitoe on behalf of the Wellcome Sanger Institute COVID-19 Surveillance Team
EPI_ISL_733669	Lighthouse Lab in Milton Keynes	Wellcome Sanger Institute for the COVID-19 Genomics UK (COG-UK) Consortium	The Lighthouse Lab in Milton Keynes and Alex Alderton, Roberto Amato, Sonia Goncalves, Ewan Harrison, David K. Jackson, Ian Johnston, Dominic Kwiatkowski, Cordelia Langford, John Sillitoe on behalf of the Wellcome Sanger Institute COVID-19 Surveillance Team
EPI_ISL_733670, EPI_ISL_733671, EPI_ISL_733672	Lighthouse Lab in Cambridge	Wellcome Sanger Institute for the COVID-19 Genomics UK (COG-UK) Consortium	Rob Howes, The Lighthouse Lab in Cambridge and Alex Alderton, Roberto Amato, Sonia Goncalves, Ewan Harrison, David K. Jackson, Ian Johnston, Dominic Kwiatkowski, Cordelia Langford, John Sillitoe on behalf of the Wellcome Sanger Institute COVID-19 Surveillance Team

EPI_ISL_733673	Lighthouse Lab in Milton Keynes	Wellcome Sanger Institute for the COVID-19 Genomics UK (COG-UK) Consortium	The Lighthouse Lab in Milton Keynes and Alex Alderton, Roberto Amato, Sonia Goncalves, Ewan Harrison, David K. Jackson, Ian Johnston, Dominic Kwiatkowski, Cordelia Langford, John Sillitoe on behalf of the Wellcome Sanger Institute COVID-19 Surveillance Team
EPI_ISL_733675, EPI_ISL_733676, EPI_ISL_733677	Lighthouse Lab in Cambridge	Wellcome Sanger Institute for the COVID-19 Genomics UK (COG-UK) Consortium	Rob Howes, The Lighthouse Lab in Cambridge and Alex Alderton, Roberto Amato, Sonia Goncalves, Ewan Harrison, David K. Jackson, Ian Johnston, Dominic Kwiatkowski, Cordelia Langford, John Sillitoe on behalf of the Wellcome Sanger Institute COVID-19 Surveillance Team
EPI_ISL_733678	Lighthouse Lab in Milton Keynes	Wellcome Sanger Institute for the COVID-19 Genomics UK (COG-UK) Consortium	The Lighthouse Lab in Milton Keynes and Alex Alderton, Roberto Amato, Sonia Goncalves, Ewan Harrison, David K. Jackson, Ian Johnston, Dominic Kwiatkowski, Cordelia Langford, John Sillitoe on behalf of the Wellcome Sanger Institute COVID-19 Surveillance Team
EPI_ISL_733680	Lighthouse Lab in Cambridge	Wellcome Sanger Institute for the COVID-19 Genomics UK (COG-UK) Consortium	Rob Howes, The Lighthouse Lab in Cambridge and Alex Alderton, Roberto Amato, Sonia Goncalves, Ewan Harrison, David K. Jackson, Ian Johnston, Dominic Kwiatkowski, Cordelia Langford, John Sillitoe on behalf of the Wellcome Sanger Institute COVID-19 Surveillance Team
EPI_ISL_733682, EPI_ISL_733683	Lighthouse Lab in Alderley Park	Wellcome Sanger Institute for the COVID-19 Genomics UK (COG-UK) Consortium	Jacquelyn Wynn, Mairead Hyland, The Lighthouse Lab in Alderley Park and Alex Alderton, Roberto Amato, Sonia Goncalves, Ewan Harrison, David K. Jackson, Ian Johnston, Dominic Kwiatkowski, Cordelia Langford, John Sillitoe on behalf of the Wellcome Sanger Institute COVID-19 Surveillance Team
EPI_ISL_733684	Lighthouse Lab in Cambridge	Wellcome Sanger Institute for the COVID-19 Genomics UK (COG-UK) Consortium	Rob Howes, The Lighthouse Lab in Cambridge and Alex Alderton, Roberto Amato, Sonia Goncalves, Ewan Harrison, David K. Jackson, Ian Johnston, Dominic Kwiatkowski, Cordelia Langford, John Sillitoe on behalf of the Wellcome Sanger Institute COVID-19 Surveillance Team
EPI_ISL_733687	Lighthouse Lab in Milton Keynes	Wellcome Sanger Institute for the COVID-19 Genomics UK (COG-UK) Consortium	The Lighthouse Lab in Milton Keynes and Alex Alderton, Roberto Amato, Sonia Goncalves, Ewan Harrison, David K. Jackson, Ian Johnston, Dominic Kwiatkowski, Cordelia Langford, John Sillitoe on behalf of the Wellcome Sanger Institute COVID-19 Surveillance Team
EPI_ISL_733689	Lighthouse Lab in Cambridge	Wellcome Sanger Institute for the COVID-19 Genomics UK (COG-UK) Consortium	Rob Howes, The Lighthouse Lab in Cambridge and Alex Alderton, Roberto Amato, Sonia Goncalves, Ewan Harrison, David K. Jackson, Ian Johnston, Dominic Kwiatkowski, Cordelia Langford, John Sillitoe on behalf of the Wellcome Sanger Institute COVID-19 Surveillance Team
EPI_ISL_733693	Lighthouse Lab in Alderley Park	Wellcome Sanger Institute for the COVID-19 Genomics UK (COG-UK) Consortium	Jacquelyn Wynn, Mairead Hyland, The Lighthouse Lab in Alderley Park and Alex Alderton, Roberto Amato, Sonia Goncalves, Ewan Harrison, David K. Jackson, Ian Johnston, Dominic Kwiatkowski, Cordelia Langford, John Sillitoe on behalf of the Wellcome Sanger Institute COVID-19 Surveillance Team
EPI_ISL_733696	Lighthouse Lab in Cambridge	Wellcome Sanger Institute for the COVID-19 Genomics UK (COG-UK) Consortium	Rob Howes, The Lighthouse Lab in Cambridge and Alex Alderton, Roberto Amato, Sonia Goncalves, Ewan Harrison, David K. Jackson, Ian Johnston, Dominic Kwiatkowski, Cordelia Langford, John Sillitoe on behalf of the Wellcome Sanger Institute COVID-19 Surveillance Team
EPI_ISL_733697	Lighthouse Lab in Alderley Park	Wellcome Sanger Institute for the COVID-19 Genomics UK (COG-UK) Consortium	Jacquelyn Wynn, Mairead Hyland, The Lighthouse Lab in Alderley Park and Alex Alderton, Roberto Amato, Sonia Goncalves, Ewan Harrison, David K. Jackson, Ian Johnston, Dominic Kwiatkowski, Cordelia Langford, John Sillitoe on behalf of the Wellcome Sanger Institute COVID-19 Surveillance Team
EPI_ISL_733698	Lighthouse Lab in Cambridge	Wellcome Sanger Institute for the COVID-19 Genomics UK (COG-UK) Consortium	Rob Howes, The Lighthouse Lab in Cambridge and Alex Alderton, Roberto Amato, Sonia Goncalves, Ewan Harrison, David K. Jackson, Ian Johnston, Dominic Kwiatkowski, Cordelia Langford, John Sillitoe on behalf of the Wellcome Sanger Institute COVID-19 Surveillance Team
EPI_ISL_733699, EPI_ISL_733701, EPI_ISL_733703	Lighthouse Lab in Alderley Park	Wellcome Sanger Institute for the COVID-19 Genomics UK (COG-UK) Consortium	Jacquelyn Wynn, Mairead Hyland, The Lighthouse Lab in Alderley Park and Alex Alderton, Roberto Amato, Sonia Goncalves, Ewan Harrison, David K. Jackson, Ian Johnston, Dominic Kwiatkowski, Cordelia Langford, John Sillitoe on behalf of the Wellcome Sanger Institute COVID-19 Surveillance Team
EPI_ISL_733705	Lighthouse Lab in Cambridge	Wellcome Sanger Institute for the COVID-19 Genomics UK (COG-UK) Consortium	Rob Howes, The Lighthouse Lab in Cambridge and Alex Alderton, Roberto Amato, Sonia Goncalves, Ewan Harrison, David K. Jackson, Ian Johnston, Dominic Kwiatkowski, Cordelia Langford, John Sillitoe on behalf of the Wellcome Sanger Institute COVID-19 Surveillance Team
EPI_ISL_733707, EPI_ISL_733711	Lighthouse Lab in Milton Keynes	Wellcome Sanger Institute for the COVID-19 Genomics UK (COG-UK) Consortium	The Lighthouse Lab in Milton Keynes and Alex Alderton, Roberto Amato, Sonia Goncalves, Ewan Harrison, David K. Jackson, Ian Johnston, Dominic Kwiatkowski, Cordelia Langford, John Sillitoe on behalf of the Wellcome Sanger Institute COVID-19 Surveillance Team
EPI_ISL_733712	Lighthouse Lab in Alderley Park	Wellcome Sanger Institute for the COVID-19 Genomics UK (COG-UK) Consortium	Jacquelyn Wynn, Mairead Hyland, The Lighthouse Lab in Alderley Park and Alex Alderton, Roberto Amato, Sonia Goncalves, Ewan Harrison, David K. Jackson, Ian Johnston, Dominic Kwiatkowski, Cordelia Langford, John Sillitoe on behalf of the Wellcome Sanger Institute COVID-19 Surveillance Team
EPI_ISL_733714, EPI_ISL_733715, EPI_ISL_733716, EPI_ISL_733720, EPI_ISL_733721, EPI_ISL_733722, EPI_ISL_733723	Lighthouse Lab in Cambridge	Wellcome Sanger Institute for the COVID-19 Genomics UK (COG-UK) Consortium	Rob Howes, The Lighthouse Lab in Cambridge and Alex Alderton, Roberto Amato, Sonia Goncalves, Ewan Harrison, David K. Jackson, Ian Johnston, Dominic Kwiatkowski, Cordelia Langford, John Sillitoe on behalf of the Wellcome Sanger Institute COVID-19 Surveillance Team
EPI_ISL_733725	Lighthouse Lab in Alderley Park	Wellcome Sanger Institute for the COVID-19 Genomics UK (COG-UK) Consortium	Jacquelyn Wynn, Mairead Hyland, The Lighthouse Lab in Alderley Park and Alex Alderton, Roberto Amato, Sonia Goncalves, Ewan Harrison, David K. Jackson, Ian Johnston, Dominic Kwiatkowski, Cordelia Langford, John Sillitoe on behalf of the Wellcome Sanger Institute COVID-19 Surveillance Team
EPI_ISL_733726, EPI_ISL_733727, EPI_ISL_733729	Lighthouse Lab in Cambridge	Wellcome Sanger Institute for the COVID-19 Genomics UK (COG-UK) Consortium	Rob Howes, The Lighthouse Lab in Cambridge and Alex Alderton, Roberto Amato, Sonia Goncalves, Ewan Harrison, David K. Jackson, Ian Johnston, Dominic Kwiatkowski, Cordelia Langford, John Sillitoe on behalf of the Wellcome Sanger Institute COVID-19 Surveillance Team
EPI_ISL_733731	Lighthouse Lab in Alderley Park	Wellcome Sanger Institute for the COVID-19 Genomics UK (COG-UK) Consortium	Jacquelyn Wynn, Mairead Hyland, The Lighthouse Lab in Alderley Park and Alex Alderton, Roberto Amato, Sonia Goncalves, Ewan Harrison, David K. Jackson, Ian Johnston, Dominic Kwiatkowski, Cordelia Langford, John Sillitoe on behalf of the Wellcome Sanger Institute COVID-19 Surveillance Team
EPI_ISL_733732, EPI_ISL_733733	Lighthouse Lab in Cambridge	Wellcome Sanger Institute for the COVID-19 Genomics UK (COG-UK) Consortium	Rob Howes, The Lighthouse Lab in Cambridge and Alex Alderton, Roberto Amato, Sonia Goncalves, Ewan Harrison, David K. Jackson, Ian Johnston, Dominic Kwiatkowski, Cordelia Langford, John Sillitoe on behalf of the Wellcome Sanger Institute COVID-19 Surveillance Team
EPI_ISL_733735	Lighthouse Lab in Milton Keynes	Wellcome Sanger Institute for the COVID-19 Genomics UK (COG-UK) Consortium	The Lighthouse Lab in Milton Keynes and Alex Alderton, Roberto Amato, Sonia Goncalves, Ewan Harrison, David K. Jackson, Ian Johnston, Dominic Kwiatkowski, Cordelia Langford, John Sillitoe on behalf of the Wellcome Sanger Institute COVID-19 Surveillance Team
EPI_ISL_733737	Lighthouse Lab in Alderley Park	Wellcome Sanger Institute for the COVID-19 Genomics UK (COG-UK) Consortium	Jacquelyn Wynn, Mairead Hyland, The Lighthouse Lab in Alderley Park and Alex Alderton, Roberto Amato, Sonia Goncalves, Ewan Harrison, David K. Jackson, Ian Johnston, Dominic Kwiatkowski, Cordelia Langford, John Sillitoe on behalf of the Wellcome Sanger Institute COVID-19 Surveillance Team
EPI_ISL_733738	Lighthouse Lab in Cambridge	Wellcome Sanger Institute for the COVID-19 Genomics UK (COG-UK) Consortium	Rob Howes, The Lighthouse Lab in Cambridge and Alex Alderton, Roberto Amato, Sonia Goncalves, Ewan Harrison, David K. Jackson, Ian Johnston, Dominic Kwiatkowski, Cordelia Langford, John Sillitoe on behalf of the Wellcome Sanger Institute COVID-19 Surveillance Team
EPI_ISL_733739	Lighthouse Lab in Milton Keynes	Wellcome Sanger Institute for the COVID-19 Genomics UK (COG-UK) Consortium	The Lighthouse Lab in Milton Keynes and Alex Alderton, Roberto Amato, Sonia Goncalves, Ewan Harrison, David K. Jackson, Ian Johnston, Dominic Kwiatkowski, Cordelia Langford, John Sillitoe on behalf of the Wellcome Sanger Institute COVID-19 Surveillance Team
EPI_ISL_733742	Lighthouse Lab in Alderley Park	Wellcome Sanger Institute for the COVID-19 Genomics UK (COG-UK) Consortium	Jacquelyn Wynn, Mairead Hyland, The Lighthouse Lab in Alderley Park and Alex Alderton, Roberto Amato, Sonia Goncalves, Ewan Harrison, David K. Jackson, Ian Johnston, Dominic Kwiatkowski, Cordelia Langford, John Sillitoe on behalf of the Wellcome Sanger Institute COVID-19 Surveillance Team
EPI_ISL_733744, EPI_ISL_733745	Lighthouse Lab in Cambridge	Wellcome Sanger Institute for the COVID-19 Genomics UK (COG-UK) Consortium	Rob Howes, The Lighthouse Lab in Cambridge and Alex Alderton, Roberto Amato, Sonia Goncalves, Ewan Harrison, David K. Jackson, Ian Johnston, Dominic Kwiatkowski, Cordelia Langford, John Sillitoe on behalf of the Wellcome Sanger Institute COVID-19 Surveillance Team
EPI_ISL_733746, EPI_ISL_733747, EPI_ISL_733749	Lighthouse Lab in Milton Keynes	Wellcome Sanger Institute for the COVID-19 Genomics UK (COG-UK) Consortium	The Lighthouse Lab in Milton Keynes and Alex Alderton, Roberto Amato, Sonia Goncalves, Ewan Harrison, David K. Jackson, Ian Johnston, Dominic Kwiatkowski, Cordelia Langford, John Sillitoe on behalf of the Wellcome Sanger Institute COVID-19 Surveillance Team
EPI_ISL_733751, EPI_ISL_733752, EPI_ISL_733753	Lighthouse Lab in Cambridge	Wellcome Sanger Institute for the COVID-19 Genomics UK (COG-UK) Consortium	Rob Howes, The Lighthouse Lab in Cambridge and Alex Alderton, Roberto Amato, Sonia Goncalves, Ewan Harrison, David K. Jackson, Ian Johnston, Dominic Kwiatkowski, Cordelia Langford, John Sillitoe on behalf of the Wellcome Sanger Institute COVID-19 Surveillance Team
EPI_ISL_733754, EPI_ISL_733755	Lighthouse Lab in Alderley Park	Wellcome Sanger Institute for the COVID-19 Genomics UK (COG-UK) Consortium	Jacquelyn Wynn, Mairead Hyland, The Lighthouse Lab in Alderley Park and Alex Alderton, Roberto Amato, Sonia Goncalves, Ewan Harrison, David K. Jackson, Ian Johnston, Dominic Kwiatkowski, Cordelia Langford, John Sillitoe on behalf of the Wellcome Sanger Institute COVID-19 Surveillance Team
EPI_ISL_733757	Lighthouse Lab in Milton Keynes	Wellcome Sanger Institute for the COVID-19 Genomics UK (COG-UK) Consortium	The Lighthouse Lab in Milton Keynes and Alex Alderton, Roberto Amato, Sonia Goncalves, Ewan Harrison, David K. Jackson, Ian Johnston, Dominic Kwiatkowski, Cordelia Langford, John Sillitoe on behalf of the Wellcome Sanger Institute COVID-19 Surveillance Team
EPI_ISL_733758	Lighthouse Lab in Cambridge	Wellcome Sanger Institute for the COVID-19 Genomics UK (COG-UK) Consortium	Rob Howes, The Lighthouse Lab in Cambridge and Alex Alderton, Roberto Amato, Sonia Goncalves, Ewan Harrison, David K. Jackson, Ian Johnston, Dominic Kwiatkowski, Cordelia Langford, John Sillitoe on behalf of the Wellcome Sanger Institute COVID-19 Surveillance Team
EPI_ISL_733759	Lighthouse Lab in Alderley Park	Wellcome Sanger Institute for the COVID-19 Genomics UK (COG-UK) Consortium	Jacquelyn Wynn, Mairead Hyland, The Lighthouse Lab in Alderley Park and Alex Alderton, Roberto Amato, Sonia Goncalves, Ewan Harrison, David K. Jackson, Ian Johnston, Dominic Kwiatkowski, Cordelia Langford, John Sillitoe on behalf of the Wellcome Sanger Institute COVID-19 Surveillance Team
EPI_ISL_733760, EPI_ISL_733761	Lighthouse Lab in Milton Keynes	Wellcome Sanger Institute for the COVID-19 Genomics UK (COG-UK) Consortium	The Lighthouse Lab in Milton Keynes and Alex Alderton, Roberto Amato, Sonia Goncalves, Ewan Harrison, David K. Jackson, Ian Johnston, Dominic Kwiatkowski, Cordelia Langford, John Sillitoe on behalf of the Wellcome Sanger Institute COVID-19 Surveillance Team

EPI_ISL_733765	Lighthouse Lab in Cambridge	Wellcome Sanger Institute for the COVID-19 Genomics UK (COG-UK) Consortium	Rob Howes, The Lighthouse Lab in Cambridge and Alex Alderton, Roberto Amato, Sonia Goncalves, Ewan Harrison, David K. Jackson, Ian Johnston, Dominic Kwiatkowski, Cordelia Langford, John Sillitoe on behalf of the Wellcome Sanger Institute COVID-19 Surveillance Team
EPI_ISL_733766	Lighthouse Lab in Alderley Park	Wellcome Sanger Institute for the COVID-19 Genomics UK (COG-UK) Consortium	Jacquelyn Wynn, Mairead Hyland, The Lighthouse Lab in Alderley Park and Alex Alderton, Roberto Amato, Sonia Goncalves, Ewan Harrison, David K. Jackson, Ian Johnston, Dominic Kwiatkowski, Cordelia Langford, John Sillitoe on behalf of the Wellcome Sanger Institute COVID-19 Surveillance Team
EPI_ISL_733770	Lighthouse Lab in Cambridge	Wellcome Sanger Institute for the COVID-19 Genomics UK (COG-UK) Consortium	Rob Howes, The Lighthouse Lab in Cambridge and Alex Alderton, Roberto Amato, Sonia Goncalves, Ewan Harrison, David K. Jackson, Ian Johnston, Dominic Kwiatkowski, Cordelia Langford, John Sillitoe on behalf of the Wellcome Sanger Institute COVID-19 Surveillance Team
EPI_ISL_733771	Lighthouse Lab in Milton Keynes	Wellcome Sanger Institute for the COVID-19 Genomics UK (COG-UK) Consortium	The Lighthouse Lab in Milton Keynes and Alex Alderton, Roberto Amato, Sonia Goncalves, Ewan Harrison, David K. Jackson, Ian Johnston, Dominic Kwiatkowski, Cordelia Langford, John Sillitoe on behalf of the Wellcome Sanger Institute COVID-19 Surveillance Team
EPI_ISL_733772, EPI_ISL_733773, EPI_ISL_733774	Lighthouse Lab in Cambridge	Wellcome Sanger Institute for the COVID-19 Genomics UK (COG-UK) Consortium	Rob Howes, The Lighthouse Lab in Cambridge and Alex Alderton, Roberto Amato, Sonia Goncalves, Ewan Harrison, David K. Jackson, Ian Johnston, Dominic Kwiatkowski, Cordelia Langford, John Sillitoe on behalf of the Wellcome Sanger Institute COVID-19 Surveillance Team
EPI_ISL_733777	Lighthouse Lab in Alderley Park	Wellcome Sanger Institute for the COVID-19 Genomics UK (COG-UK) Consortium	Jacquelyn Wynn, Mairead Hyland, The Lighthouse Lab in Alderley Park and Alex Alderton, Roberto Amato, Sonia Goncalves, Ewan Harrison, David K. Jackson, Ian Johnston, Dominic Kwiatkowski, Cordelia Langford, John Sillitoe on behalf of the Wellcome Sanger Institute COVID-19 Surveillance Team
EPI_ISL_733778, EPI_ISL_733779, EPI_ISL_733782	Lighthouse Lab in Cambridge	Wellcome Sanger Institute for the COVID-19 Genomics UK (COG-UK) Consortium	Rob Howes, The Lighthouse Lab in Cambridge and Alex Alderton, Roberto Amato, Sonia Goncalves, Ewan Harrison, David K. Jackson, Ian Johnston, Dominic Kwiatkowski, Cordelia Langford, John Sillitoe on behalf of the Wellcome Sanger Institute COVID-19 Surveillance Team
EPI_ISL_733783	Lighthouse Lab in Alderley Park	Wellcome Sanger Institute for the COVID-19 Genomics UK (COG-UK) Consortium	Jacquelyn Wynn, Mairead Hyland, The Lighthouse Lab in Alderley Park and Alex Alderton, Roberto Amato, Sonia Goncalves, Ewan Harrison, David K. Jackson, Ian Johnston, Dominic Kwiatkowski, Cordelia Langford, John Sillitoe on behalf of the Wellcome Sanger Institute COVID-19 Surveillance Team
EPI_ISL_733784	Lighthouse Lab in Cambridge	Wellcome Sanger Institute for the COVID-19 Genomics UK (COG-UK) Consortium	Rob Howes, The Lighthouse Lab in Cambridge and Alex Alderton, Roberto Amato, Sonia Goncalves, Ewan Harrison, David K. Jackson, Ian Johnston, Dominic Kwiatkowski, Cordelia Langford, John Sillitoe on behalf of the Wellcome Sanger Institute COVID-19 Surveillance Team
EPI_ISL_733785	Lighthouse Lab in Alderley Park	Wellcome Sanger Institute for the COVID-19 Genomics UK (COG-UK) Consortium	Jacquelyn Wynn, Mairead Hyland, The Lighthouse Lab in Alderley Park and Alex Alderton, Roberto Amato, Sonia Goncalves, Ewan Harrison, David K. Jackson, Ian Johnston, Dominic Kwiatkowski, Cordelia Langford, John Sillitoe on behalf of the Wellcome Sanger Institute COVID-19 Surveillance Team
EPI_ISL_733787, EPI_ISL_733788, EPI_ISL_733790, EPI_ISL_733792, EPI_ISL_733793	Lighthouse Lab in Cambridge	Wellcome Sanger Institute for the COVID-19 Genomics UK (COG-UK) Consortium	Rob Howes, The Lighthouse Lab in Cambridge and Alex Alderton, Roberto Amato, Sonia Goncalves, Ewan Harrison, David K. Jackson, Ian Johnston, Dominic Kwiatkowski, Cordelia Langford, John Sillitoe on behalf of the Wellcome Sanger Institute COVID-19 Surveillance Team
EPI_ISL_733794	Lighthouse Lab in Alderley Park	Wellcome Sanger Institute for the COVID-19 Genomics UK (COG-UK) Consortium	Jacquelyn Wynn, Mairead Hyland, The Lighthouse Lab in Alderley Park and Alex Alderton, Roberto Amato, Sonia Goncalves, Ewan Harrison, David K. Jackson, Ian Johnston, Dominic Kwiatkowski, Cordelia Langford, John Sillitoe on behalf of the Wellcome Sanger Institute COVID-19 Surveillance Team
EPI_ISL_733796	Lighthouse Lab in Milton Keynes	Wellcome Sanger Institute for the COVID-19 Genomics UK (COG-UK) Consortium	The Lighthouse Lab in Milton Keynes and Alex Alderton, Roberto Amato, Sonia Goncalves, Ewan Harrison, David K. Jackson, Ian Johnston, Dominic Kwiatkowski, Cordelia Langford, John Sillitoe on behalf of the Wellcome Sanger Institute COVID-19 Surveillance Team
EPI_ISL_733797	Lighthouse Lab in Alderley Park	Wellcome Sanger Institute for the COVID-19 Genomics UK (COG-UK) Consortium	Jacquelyn Wynn, Mairead Hyland, The Lighthouse Lab in Alderley Park and Alex Alderton, Roberto Amato, Sonia Goncalves, Ewan Harrison, David K. Jackson, Ian Johnston, Dominic Kwiatkowski, Cordelia Langford, John Sillitoe on behalf of the Wellcome Sanger Institute COVID-19 Surveillance Team
EPI_ISL_733802, EPI_ISL_733804	Lighthouse Lab in Cambridge	Wellcome Sanger Institute for the COVID-19 Genomics UK (COG-UK) Consortium	Rob Howes, The Lighthouse Lab in Cambridge and Alex Alderton, Roberto Amato, Sonia Goncalves, Ewan Harrison, David K. Jackson, Ian Johnston, Dominic Kwiatkowski, Cordelia Langford, John Sillitoe on behalf of the Wellcome Sanger Institute COVID-19 Surveillance Team
EPI_ISL_733806	Lighthouse Lab in Alderley Park	Wellcome Sanger Institute for the COVID-19 Genomics UK (COG-UK) Consortium	Jacquelyn Wynn, Mairead Hyland, The Lighthouse Lab in Alderley Park and Alex Alderton, Roberto Amato, Sonia Goncalves, Ewan Harrison, David K. Jackson, Ian Johnston, Dominic Kwiatkowski, Cordelia Langford, John Sillitoe on behalf of the Wellcome Sanger Institute COVID-19 Surveillance Team
EPI_ISL_733809	Lighthouse Lab in Cambridge	Wellcome Sanger Institute for the COVID-19 Genomics UK (COG-UK) Consortium	Rob Howes, The Lighthouse Lab in Cambridge and Alex Alderton, Roberto Amato, Sonia Goncalves, Ewan Harrison, David K. Jackson, Ian Johnston, Dominic Kwiatkowski, Cordelia Langford, John Sillitoe on behalf of the Wellcome Sanger Institute COVID-19 Surveillance Team
EPI_ISL_733811, EPI_ISL_733813	Lighthouse Lab in Milton Keynes	Wellcome Sanger Institute for the COVID-19 Genomics UK (COG-UK) Consortium	The Lighthouse Lab in Milton Keynes and Alex Alderton, Roberto Amato, Sonia Goncalves, Ewan Harrison, David K. Jackson, Ian Johnston, Dominic Kwiatkowski, Cordelia Langford, John Sillitoe on behalf of the Wellcome Sanger Institute COVID-19 Surveillance Team
EPI_ISL_733816, EPI_ISL_733817	Lighthouse Lab in Cambridge	Wellcome Sanger Institute for the COVID-19 Genomics UK (COG-UK) Consortium	Rob Howes, The Lighthouse Lab in Cambridge and Alex Alderton, Roberto Amato, Sonia Goncalves, Ewan Harrison, David K. Jackson, Ian Johnston, Dominic Kwiatkowski, Cordelia Langford, John Sillitoe on behalf of the Wellcome Sanger Institute COVID-19 Surveillance Team
EPI_ISL_733820	Lighthouse Lab in Milton Keynes	Wellcome Sanger Institute for the COVID-19 Genomics UK (COG-UK) Consortium	The Lighthouse Lab in Milton Keynes and Alex Alderton, Roberto Amato, Sonia Goncalves, Ewan Harrison, David K. Jackson, Ian Johnston, Dominic Kwiatkowski, Cordelia Langford, John Sillitoe on behalf of the Wellcome Sanger Institute COVID-19 Surveillance Team
EPI_ISL_733821, EPI_ISL_733822, EPI_ISL_733823, EPI_ISL_733824	Lighthouse Lab in Cambridge	Wellcome Sanger Institute for the COVID-19 Genomics UK (COG-UK) Consortium	Rob Howes, The Lighthouse Lab in Cambridge and Alex Alderton, Roberto Amato, Sonia Goncalves, Ewan Harrison, David K. Jackson, Ian Johnston, Dominic Kwiatkowski, Cordelia Langford, John Sillitoe on behalf of the Wellcome Sanger Institute COVID-19 Surveillance Team
EPI_ISL_733825, EPI_ISL_733826, EPI_ISL_733828, EPI_ISL_733829, EPI_ISL_733832	Lighthouse Lab in Milton Keynes	Wellcome Sanger Institute for the COVID-19 Genomics UK (COG-UK) Consortium	The Lighthouse Lab in Milton Keynes and Alex Alderton, Roberto Amato, Sonia Goncalves, Ewan Harrison, David K. Jackson, Ian Johnston, Dominic Kwiatkowski, Cordelia Langford, John Sillitoe on behalf of the Wellcome Sanger Institute COVID-19 Surveillance Team
EPI_ISL_733833	Lighthouse Lab in Alderley Park	Wellcome Sanger Institute for the COVID-19 Genomics UK (COG-UK) Consortium	Jacquelyn Wynn, Mairead Hyland, The Lighthouse Lab in Alderley Park and Alex Alderton, Roberto Amato, Sonia Goncalves, Ewan Harrison, David K. Jackson, Ian Johnston, Dominic Kwiatkowski, Cordelia Langford, John Sillitoe on behalf of the Wellcome Sanger Institute COVID-19 Surveillance Team
EPI_ISL_733834, EPI_ISL_733835	Lighthouse Lab in Cambridge	Wellcome Sanger Institute for the COVID-19 Genomics UK (COG-UK) Consortium	Rob Howes, The Lighthouse Lab in Cambridge and Alex Alderton, Roberto Amato, Sonia Goncalves, Ewan Harrison, David K. Jackson, Ian Johnston, Dominic Kwiatkowski, Cordelia Langford, John Sillitoe on behalf of the Wellcome Sanger Institute COVID-19 Surveillance Team
EPI_ISL_733837	Lighthouse Lab in Alderley Park	Wellcome Sanger Institute for the COVID-19 Genomics UK (COG-UK) Consortium	Jacquelyn Wynn, Mairead Hyland, The Lighthouse Lab in Alderley Park and Alex Alderton, Roberto Amato, Sonia Goncalves, Ewan Harrison, David K. Jackson, Ian Johnston, Dominic Kwiatkowski, Cordelia Langford, John Sillitoe on behalf of the Wellcome Sanger Institute COVID-19 Surveillance Team
EPI_ISL_733838	Lighthouse Lab in Milton Keynes	Wellcome Sanger Institute for the COVID-19 Genomics UK (COG-UK) Consortium	The Lighthouse Lab in Milton Keynes and Alex Alderton, Roberto Amato, Sonia Goncalves, Ewan Harrison, David K. Jackson, Ian Johnston, Dominic Kwiatkowski, Cordelia Langford, John Sillitoe on behalf of the Wellcome Sanger Institute COVID-19 Surveillance Team
EPI_ISL_733839	Lighthouse Lab in Cambridge	Wellcome Sanger Institute for the COVID-19 Genomics UK (COG-UK) Consortium	Rob Howes, The Lighthouse Lab in Cambridge and Alex Alderton, Roberto Amato, Sonia Goncalves, Ewan Harrison, David K. Jackson, Ian Johnston, Dominic Kwiatkowski, Cordelia Langford, John Sillitoe on behalf of the Wellcome Sanger Institute COVID-19 Surveillance Team
EPI_ISL_733842	Lighthouse Lab in Milton Keynes	Wellcome Sanger Institute for the COVID-19 Genomics UK (COG-UK) Consortium	The Lighthouse Lab in Milton Keynes and Alex Alderton, Roberto Amato, Sonia Goncalves, Ewan Harrison, David K. Jackson, Ian Johnston, Dominic Kwiatkowski, Cordelia Langford, John Sillitoe on behalf of the Wellcome Sanger Institute COVID-19 Surveillance Team
EPI_ISL_733844	Lighthouse Lab in Alderley Park	Wellcome Sanger Institute for the COVID-19 Genomics UK (COG-UK) Consortium	Jacquelyn Wynn, Mairead Hyland, The Lighthouse Lab in Alderley Park and Alex Alderton, Roberto Amato, Sonia Goncalves, Ewan Harrison, David K. Jackson, Ian Johnston, Dominic Kwiatkowski, Cordelia Langford, John Sillitoe on behalf of the Wellcome Sanger Institute COVID-19 Surveillance Team
EPI_ISL_733845	Lighthouse Lab in Milton Keynes	Wellcome Sanger Institute for the COVID-19 Genomics UK (COG-UK) Consortium	The Lighthouse Lab in Milton Keynes and Alex Alderton, Roberto Amato, Sonia Goncalves, Ewan Harrison, David K. Jackson, Ian Johnston, Dominic Kwiatkowski, Cordelia Langford, John Sillitoe on behalf of the Wellcome Sanger Institute COVID-19 Surveillance Team
EPI_ISL_733846	Lighthouse Lab in Cambridge	Wellcome Sanger Institute for the COVID-19 Genomics UK (COG-UK) Consortium	Rob Howes, The Lighthouse Lab in Cambridge and Alex Alderton, Roberto Amato, Sonia Goncalves, Ewan Harrison, David K. Jackson, Ian Johnston, Dominic Kwiatkowski, Cordelia Langford, John Sillitoe on behalf of the Wellcome Sanger Institute COVID-19 Surveillance Team
EPI_ISL_733848	Lighthouse Lab in Alderley Park	Wellcome Sanger Institute for the COVID-19 Genomics UK (COG-UK) Consortium	Jacquelyn Wynn, Mairead Hyland, The Lighthouse Lab in Alderley Park and Alex Alderton, Roberto Amato, Sonia Goncalves, Ewan Harrison, David K. Jackson, Ian Johnston, Dominic Kwiatkowski, Cordelia Langford, John Sillitoe on behalf of the Wellcome Sanger Institute COVID-19 Surveillance Team
EPI_ISL_733849	Lighthouse Lab in Cambridge	Wellcome Sanger Institute for the COVID-19 Genomics UK (COG-UK) Consortium	Rob Howes, The Lighthouse Lab in Cambridge and Alex Alderton, Roberto Amato, Sonia Goncalves, Ewan Harrison, David K. Jackson, Ian Johnston, Dominic Kwiatkowski, Cordelia Langford, John Sillitoe on behalf of the Wellcome Sanger Institute COVID-19 Surveillance Team
EPI_ISL_733851	Lighthouse Lab in Milton Keynes	Wellcome Sanger Institute for the COVID-19 Genomics UK (COG-UK) Consortium	The Lighthouse Lab in Milton Keynes and Alex Alderton, Roberto Amato, Sonia Goncalves, Ewan Harrison, David K. Jackson, Ian Johnston, Dominic Kwiatkowski, Cordelia Langford, John Sillitoe on behalf of the Wellcome Sanger Institute COVID-19 Surveillance Team
EPI_ISL_733853, EPI_ISL_733854	Lighthouse Lab in Cambridge	Wellcome Sanger Institute for the COVID-19 Genomics UK	Rob Howes, The Lighthouse Lab in Cambridge and Alex Alderton, Roberto Amato, Sonia Goncalves, Ewan Harrison, David K. Jackson, Ian Johnston,

		(COG-UK) Consortium	Dominic Kwiatkowski, Cordelia Langford, John Sillitoe on behalf of the Wellcome Sanger Institute COVID-19 Surveillance Team
EPI_ISL_733855	Lighthouse Lab in Milton Keynes	Wellcome Sanger Institute for the COVID-19 Genomics UK (COG-UK) Consortium	The Lighthouse Lab in Milton Keynes and Alex Alderton, Roberto Amato, Sonia Goncalves, Ewan Harrison, David K. Jackson, Ian Johnston, Dominic Kwiatkowski, Cordelia Langford, John Sillitoe on behalf of the Wellcome Sanger Institute COVID-19 Surveillance Team
EPI_ISL_733857	Lighthouse Lab in Cambridge	Wellcome Sanger Institute for the COVID-19 Genomics UK (COG-UK) Consortium	Rob Howes, The Lighthouse Lab in Cambridge and Alex Alderton, Roberto Amato, Sonia Goncalves, Ewan Harrison, David K. Jackson, Ian Johnston, Dominic Kwiatkowski, Cordelia Langford, John Sillitoe on behalf of the Wellcome Sanger Institute COVID-19 Surveillance Team
EPI_ISL_733858	Lighthouse Lab in Milton Keynes	Wellcome Sanger Institute for the COVID-19 Genomics UK (COG-UK) Consortium	The Lighthouse Lab in Milton Keynes and Alex Alderton, Roberto Amato, Sonia Goncalves, Ewan Harrison, David K. Jackson, Ian Johnston, Dominic Kwiatkowski, Cordelia Langford, John Sillitoe on behalf of the Wellcome Sanger Institute COVID-19 Surveillance Team
EPI_ISL_733860	Lighthouse Lab in Cambridge	Wellcome Sanger Institute for the COVID-19 Genomics UK (COG-UK) Consortium	Rob Howes, The Lighthouse Lab in Cambridge and Alex Alderton, Roberto Amato, Sonia Goncalves, Ewan Harrison, David K. Jackson, Ian Johnston, Dominic Kwiatkowski, Cordelia Langford, John Sillitoe on behalf of the Wellcome Sanger Institute COVID-19 Surveillance Team
EPI_ISL_733861	Lighthouse Lab in Milton Keynes	Wellcome Sanger Institute for the COVID-19 Genomics UK (COG-UK) Consortium	The Lighthouse Lab in Milton Keynes and Alex Alderton, Roberto Amato, Sonia Goncalves, Ewan Harrison, David K. Jackson, Ian Johnston, Dominic Kwiatkowski, Cordelia Langford, John Sillitoe on behalf of the Wellcome Sanger Institute COVID-19 Surveillance Team
EPI_ISL_733862	Lighthouse Lab in Cambridge	Wellcome Sanger Institute for the COVID-19 Genomics UK (COG-UK) Consortium	Rob Howes, The Lighthouse Lab in Cambridge and Alex Alderton, Roberto Amato, Sonia Goncalves, Ewan Harrison, David K. Jackson, Ian Johnston, Dominic Kwiatkowski, Cordelia Langford, John Sillitoe on behalf of the Wellcome Sanger Institute COVID-19 Surveillance Team
EPI_ISL_733863, EPI_ISL_733865	Lighthouse Lab in Milton Keynes	Wellcome Sanger Institute for the COVID-19 Genomics UK (COG-UK) Consortium	The Lighthouse Lab in Milton Keynes and Alex Alderton, Roberto Amato, Sonia Goncalves, Ewan Harrison, David K. Jackson, Ian Johnston, Dominic Kwiatkowski, Cordelia Langford, John Sillitoe on behalf of the Wellcome Sanger Institute COVID-19 Surveillance Team
EPI_ISL_733868, EPI_ISL_733870	Lighthouse Lab in Cambridge	Wellcome Sanger Institute for the COVID-19 Genomics UK (COG-UK) Consortium	Rob Howes, The Lighthouse Lab in Cambridge and Alex Alderton, Roberto Amato, Sonia Goncalves, Ewan Harrison, David K. Jackson, Ian Johnston, Dominic Kwiatkowski, Cordelia Langford, John Sillitoe on behalf of the Wellcome Sanger Institute COVID-19 Surveillance Team
EPI_ISL_733872	Lighthouse Lab in Milton Keynes	Wellcome Sanger Institute for the COVID-19 Genomics UK (COG-UK) Consortium	The Lighthouse Lab in Milton Keynes and Alex Alderton, Roberto Amato, Sonia Goncalves, Ewan Harrison, David K. Jackson, Ian Johnston, Dominic Kwiatkowski, Cordelia Langford, John Sillitoe on behalf of the Wellcome Sanger Institute COVID-19 Surveillance Team
EPI_ISL_733876	Lighthouse Lab in Cambridge	Wellcome Sanger Institute for the COVID-19 Genomics UK (COG-UK) Consortium	Rob Howes, The Lighthouse Lab in Cambridge and Alex Alderton, Roberto Amato, Sonia Goncalves, Ewan Harrison, David K. Jackson, Ian Johnston, Dominic Kwiatkowski, Cordelia Langford, John Sillitoe on behalf of the Wellcome Sanger Institute COVID-19 Surveillance Team
EPI_ISL_733877	Lighthouse Lab in Alderley Park	Wellcome Sanger Institute for the COVID-19 Genomics UK (COG-UK) Consortium	Jacquelyn Wynn, Mairead Hyland, The Lighthouse Lab in Alderley Park and Alex Alderton, Roberto Amato, Sonia Goncalves, Ewan Harrison, David K. Jackson, Ian Johnston, Dominic Kwiatkowski, Cordelia Langford, John Sillitoe on behalf of the Wellcome Sanger Institute COVID-19 Surveillance Team
EPI_ISL_733878	Lighthouse Lab in Cambridge	Wellcome Sanger Institute for the COVID-19 Genomics UK (COG-UK) Consortium	Rob Howes, The Lighthouse Lab in Cambridge and Alex Alderton, Roberto Amato, Sonia Goncalves, Ewan Harrison, David K. Jackson, Ian Johnston, Dominic Kwiatkowski, Cordelia Langford, John Sillitoe on behalf of the Wellcome Sanger Institute COVID-19 Surveillance Team
EPI_ISL_733880, EPI_ISL_733882	Lighthouse Lab in Alderley Park	Wellcome Sanger Institute for the COVID-19 Genomics UK (COG-UK) Consortium	Jacquelyn Wynn, Mairead Hyland, The Lighthouse Lab in Alderley Park and Alex Alderton, Roberto Amato, Sonia Goncalves, Ewan Harrison, David K. Jackson, Ian Johnston, Dominic Kwiatkowski, Cordelia Langford, John Sillitoe on behalf of the Wellcome Sanger Institute COVID-19 Surveillance Team
EPI_ISL_733884	Lighthouse Lab in Milton Keynes	Wellcome Sanger Institute for the COVID-19 Genomics UK (COG-UK) Consortium	The Lighthouse Lab in Milton Keynes and Alex Alderton, Roberto Amato, Sonia Goncalves, Ewan Harrison, David K. Jackson, Ian Johnston, Dominic Kwiatkowski, Cordelia Langford, John Sillitoe on behalf of the Wellcome Sanger Institute COVID-19 Surveillance Team
EPI_ISL_733886	Lighthouse Lab in Cambridge	Wellcome Sanger Institute for the COVID-19 Genomics UK (COG-UK) Consortium	Rob Howes, The Lighthouse Lab in Cambridge and Alex Alderton, Roberto Amato, Sonia Goncalves, Ewan Harrison, David K. Jackson, Ian Johnston, Dominic Kwiatkowski, Cordelia Langford, John Sillitoe on behalf of the Wellcome Sanger Institute COVID-19 Surveillance Team
EPI_ISL_733889, EPI_ISL_733891	Lighthouse Lab in Milton Keynes	Wellcome Sanger Institute for the COVID-19 Genomics UK (COG-UK) Consortium	The Lighthouse Lab in Milton Keynes and Alex Alderton, Roberto Amato, Sonia Goncalves, Ewan Harrison, David K. Jackson, Ian Johnston, Dominic Kwiatkowski, Cordelia Langford, John Sillitoe on behalf of the Wellcome Sanger Institute COVID-19 Surveillance Team
EPI_ISL_733892, EPI_ISL_733895	Lighthouse Lab in Cambridge	Wellcome Sanger Institute for the COVID-19 Genomics UK (COG-UK) Consortium	Rob Howes, The Lighthouse Lab in Cambridge and Alex Alderton, Roberto Amato, Sonia Goncalves, Ewan Harrison, David K. Jackson, Ian Johnston, Dominic Kwiatkowski, Cordelia Langford, John Sillitoe on behalf of the Wellcome Sanger Institute COVID-19 Surveillance Team
EPI_ISL_733897	Lighthouse Lab in Alderley Park	Wellcome Sanger Institute for the COVID-19 Genomics UK (COG-UK) Consortium	Jacquelyn Wynn, Mairead Hyland, The Lighthouse Lab in Alderley Park and Alex Alderton, Roberto Amato, Sonia Goncalves, Ewan Harrison, David K. Jackson, Ian Johnston, Dominic Kwiatkowski, Cordelia Langford, John Sillitoe on behalf of the Wellcome Sanger Institute COVID-19 Surveillance Team
EPI_ISL_733898, EPI_ISL_733899	Lighthouse Lab in Cambridge	Wellcome Sanger Institute for the COVID-19 Genomics UK (COG-UK) Consortium	Rob Howes, The Lighthouse Lab in Cambridge and Alex Alderton, Roberto Amato, Sonia Goncalves, Ewan Harrison, David K. Jackson, Ian Johnston, Dominic Kwiatkowski, Cordelia Langford, John Sillitoe on behalf of the Wellcome Sanger Institute COVID-19 Surveillance Team
EPI_ISL_733901	Lighthouse Lab in Alderley Park	Wellcome Sanger Institute for the COVID-19 Genomics UK (COG-UK) Consortium	Jacquelyn Wynn, Mairead Hyland, The Lighthouse Lab in Alderley Park and Alex Alderton, Roberto Amato, Sonia Goncalves, Ewan Harrison, David K. Jackson, Ian Johnston, Dominic Kwiatkowski, Cordelia Langford, John Sillitoe on behalf of the Wellcome Sanger Institute COVID-19 Surveillance Team
EPI_ISL_733902, EPI_ISL_733903, EPI_ISL_733904, EPI_ISL_733905	Lighthouse Lab in Milton Keynes	Wellcome Sanger Institute for the COVID-19 Genomics UK (COG-UK) Consortium	The Lighthouse Lab in Milton Keynes and Alex Alderton, Roberto Amato, Sonia Goncalves, Ewan Harrison, David K. Jackson, Ian Johnston, Dominic Kwiatkowski, Cordelia Langford, John Sillitoe on behalf of the Wellcome Sanger Institute COVID-19 Surveillance Team
EPI_ISL_733906, EPI_ISL_733907, EPI_ISL_733908, EPI_ISL_733909	Lighthouse Lab in Cambridge	Wellcome Sanger Institute for the COVID-19 Genomics UK (COG-UK) Consortium	Rob Howes, The Lighthouse Lab in Cambridge and Alex Alderton, Roberto Amato, Sonia Goncalves, Ewan Harrison, David K. Jackson, Ian Johnston, Dominic Kwiatkowski, Cordelia Langford, John Sillitoe on behalf of the Wellcome Sanger Institute COVID-19 Surveillance Team
EPI_ISL_733912	Lighthouse Lab in Alderley Park	Wellcome Sanger Institute for the COVID-19 Genomics UK (COG-UK) Consortium	Jacquelyn Wynn, Mairead Hyland, The Lighthouse Lab in Alderley Park and Alex Alderton, Roberto Amato, Sonia Goncalves, Ewan Harrison, David K. Jackson, Ian Johnston, Dominic Kwiatkowski, Cordelia Langford, John Sillitoe on behalf of the Wellcome Sanger Institute COVID-19 Surveillance Team
EPI_ISL_733915	Lighthouse Lab in Cambridge	Wellcome Sanger Institute for the COVID-19 Genomics UK (COG-UK) Consortium	Rob Howes, The Lighthouse Lab in Cambridge and Alex Alderton, Roberto Amato, Sonia Goncalves, Ewan Harrison, David K. Jackson, Ian Johnston, Dominic Kwiatkowski, Cordelia Langford, John Sillitoe on behalf of the Wellcome Sanger Institute COVID-19 Surveillance Team
EPI_ISL_733917, EPI_ISL_733918, EPI_ISL_733919, EPI_ISL_733921, EPI_ISL_733923, EPI_ISL_733924, EPI_ISL_733925, EPI_ISL_733927, EPI_ISL_733929, EPI_ISL_733930, EPI_ISL_733931, EPI_ISL_733934, EPI_ISL_733935, EPI_ISL_733936, EPI_ISL_733937, EPI_ISL_733943, EPI_ISL_733944, EPI_ISL_733946, EPI_ISL_733949, EPI_ISL_733950, EPI_ISL_733951, EPI_ISL_733952, EPI_ISL_733955, EPI_ISL_733956, EPI_ISL_733957, EPI_ISL_733960, EPI_ISL_733961, EPI_ISL_733962, EPI_ISL_733964, EPI_ISL_733967, EPI_ISL_733970, EPI_ISL_733972, EPI_ISL_733973, EPI_ISL_733974, EPI_ISL_733977, EPI_ISL_733978, EPI_ISL_733980, EPI_ISL_733983, EPI_ISL_733986, EPI_ISL_733987, EPI_ISL_733988, EPI_ISL_733989, EPI_ISL_733990, EPI_ISL_733991, EPI_ISL_733994, EPI_ISL_733996, EPI_ISL_733997, EPI_ISL_733998, EPI_ISL_733999, EPI_ISL_734001, EPI_ISL_734002, EPI_ISL_734004, EPI_ISL_734005, EPI_ISL_734008, EPI_ISL_734009, EPI_ISL_734010, EPI_ISL_734011, EPI_ISL_734012, EPI_ISL_734015, EPI_ISL_734020, EPI_ISL_734021, EPI_ISL_734023, EPI_ISL_734024, EPI_ISL_734025, EPI_ISL_734026, EPI_ISL_734027, EPI_ISL_734029, EPI_ISL_734030, EPI_ISL_734031, EPI_ISL_734032, EPI_ISL_734033, EPI_ISL_734034, EPI_ISL_734035, EPI_ISL_734036, EPI_ISL_734037, EPI_ISL_734038, EPI_ISL_734039, EPI_ISL_734040, EPI_ISL_734041, EPI_ISL_734045, EPI_ISL_734046, EPI_ISL_734047, EPI_ISL_734050, EPI_ISL_734053, EPI_ISL_734055, EPI_ISL_734056, EPI_ISL_734057, EPI_ISL_734058, EPI_ISL_734059, EPI_ISL_734062, EPI_ISL_734065, EPI_ISL_734068, EPI_ISL_734069, EPI_ISL_734070, EPI_ISL_734071, EPI_ISL_734072, EPI_ISL_734075, EPI_ISL_734076, EPI_ISL_734079, EPI_ISL_734080, EPI_ISL_734081, EPI_ISL_734082, EPI_ISL_734083, EPI_ISL_734086, EPI_ISL_734087, EPI_ISL_734088, EPI_ISL_734089, EPI_ISL_734090, EPI_ISL_734093, EPI_ISL_734095, EPI_ISL_734096, EPI_ISL_734097, EPI_ISL_734098, EPI_ISL_734099, EPI_ISL_734101, EPI_ISL_734102, EPI_ISL_734103, EPI_ISL_734104, EPI_ISL_734105, EPI_ISL_734106, EPI_ISL_734110, EPI_ISL_734111, EPI_ISL_734113, EPI_ISL_734114, EPI_ISL_734115, EPI_ISL_734116, EPI_ISL_734117, EPI_ISL_734118, EPI_ISL_734120, EPI_ISL_734121, EPI_ISL_734122, EPI_ISL_734124, EPI_ISL_734125, EPI_ISL_734127, EPI_ISL_734129, EPI_ISL_734130, EPI_ISL_734133, EPI_ISL_734134, EPI_ISL_734135, EPI_ISL_734136, EPI_ISL_734137, EPI_ISL_734138, EPI_ISL_734141, EPI_ISL_734143, EPI_ISL_734145, EPI_ISL_734146, EPI_ISL_734147, EPI_ISL_734148, EPI_ISL_734149, EPI_ISL_734150, EPI_ISL_734152, EPI_ISL_734155, EPI_ISL_734156, EPI_ISL_734157, EPI_ISL_734158, EPI_ISL_734159, EPI_ISL_734161, EPI_ISL_734162, EPI_ISL_734164			
see above	Lighthouse Lab in Glasgow	Wellcome Sanger Institute for the COVID-19 Genomics UK (COG-UK) Consortium	Harper VanSteenhouse, Yumi Kasai, David Gray, Carol Clugston, Anna Dominiczak and Alex Alderton, Roberto Amato, Sonia Goncalves, Ewan Harrison, David K. Jackson, Ian Johnston, Dominic Kwiatkowski, Cordelia Langford, John Sillitoe on behalf of the Wellcome Sanger Institute COVID-19 Surveillance Team
EPI_ISL_735240	UZ Leuven, National Reference Laboratory for Coronaviruses, Laboratory Medicine, Leuven, Belgium	KU Leuven, Rega Institute, Clinical and Epidemiological Virology	Tony Wawina-Bokalanga, Joan Marti-Carerras, Bert Vanmechelen, Piet Maes
EPI_ISL_735384, EPI_ISL_735386, EPI_ISL_735387, EPI_ISL_735389	National Virus Reference Laboratory	National Virus Reference Laboratory	Michael Carr, Gabriel Gonzalez, Jonathan Dean, Daniel Hare, Cillian F De Gascun
EPI_ISL_735498, EPI_ISL_735499	University of Chittagong	Central Biological Research Laboratory and Department of Biochemistry and Molecular Biology	Mohammad Omar Faruque, H. M. Abdullah Al Masud, Imam Hossen, Md. Khondakar Raziur Rahman, Sajib Rudra, Md. Arif Hossain, Shanta Paul, Md. Omer Faruq, Robiul Hasan Bhuiyan, Md. Imranul Hoq
EPI_ISL_735500	University of Chittagong	Central Biological Research Laboratory and Department of Biochemistry and Molecular Biology	H. M. Abdullah Al Masud, Mohammad Omar Faruque, Sajib Rudra, Md. Khondakar Raziur Rahman, Imam Hossen, Md. Arif Hossain, Shanta Paul, Md. Omer Faruq, Md. Imranul Hoq, Robiul Hasan Bhuiyan
EPI_ISL_735510	National Institute for Infectious Diseases, INMI, "L. Spallanzani" IRCCS	National Institute for Infectious Diseases, INMI, "L. Spallanzani" IRCCS	M. Rueca, C.E.M Gruber, E. Giombini, F. Messina, B. Bartolini, F. Carletti, A. Di Caro, M.R Capobianchi
EPI_ISL_735512, EPI_ISL_735513, EPI_ISL_735514, EPI_ISL_735516, EPI_ISL_735517, EPI_ISL_735521, EPI_ISL_735524, EPI_ISL_735525, EPI_ISL_735526, EPI_ISL_735529, EPI_ISL_735531, EPI_ISL_735533, EPI_ISL_735534, EPI_ISL_735535, EPI_ISL_735536, EPI_ISL_735537, EPI_ISL_735538, EPI_ISL_735539,			

EPI_ISL_735540, EPI_ISL_735542, EPI_ISL_735543, EPI_ISL_735547, EPI_ISL_735548, EPI_ISL_735549, EPI_ISL_735550, EPI_ISL_735553, EPI_ISL_735554, EPI_ISL_735555, EPI_ISL_735558, EPI_ISL_735560, EPI_ISL_735561, EPI_ISL_735565, EPI_ISL_735566, EPI_ISL_735570, EPI_ISL_735571, EPI_ISL_735572, EPI_ISL_735574, EPI_ISL_735575, EPI_ISL_735576, EPI_ISL_735577, EPI_ISL_735579, EPI_ISL_735580, EPI_ISL_735581, EPI_ISL_735582, EPI_ISL_735583, EPI_ISL_735584, EPI_ISL_735585, EPI_ISL_735586, EPI_ISL_735587, EPI_ISL_735589, EPI_ISL_735591, EPI_ISL_735593, EPI_ISL_735595, EPI_ISL_735597, EPI_ISL_735598, EPI_ISL_735602, EPI_ISL_735603, EPI_ISL_735604, EPI_ISL_735607, EPI_ISL_735608, EPI_ISL_735609, EPI_ISL_735610, EPI_ISL_735611, EPI_ISL_735612, EPI_ISL_735614, EPI_ISL_735615, EPI_ISL_735616, EPI_ISL_735619, EPI_ISL_735620, EPI_ISL_735621, EPI_ISL_735622, EPI_ISL_735623, EPI_ISL_735624, EPI_ISL_735625, EPI_ISL_735626, EPI_ISL_735627, EPI_ISL_735628, EPI_ISL_735629, EPI_ISL_735630, EPI_ISL_735631, EPI_ISL_735633, EPI_ISL_735634, EPI_ISL_735635, EPI_ISL_735636, EPI_ISL_735640, EPI_ISL_735642, EPI_ISL_735643, EPI_ISL_735644, EPI_ISL_735645, EPI_ISL_735646, EPI_ISL_735648, EPI_ISL_735649, EPI_ISL_735650, EPI_ISL_735651, EPI_ISL_735652, EPI_ISL_735654, EPI_ISL_735655, EPI_ISL_735657, EPI_ISL_735658, EPI_ISL_735659, EPI_ISL_735660, EPI_ISL_735661, EPI_ISL_735662, EPI_ISL_735663, EPI_ISL_735664, EPI_ISL_735665, EPI_ISL_735667, EPI_ISL_735670, EPI_ISL_735672, EPI_ISL_735673, EPI_ISL_735677, EPI_ISL_735678, EPI_ISL_735682, EPI_ISL_735683, EPI_ISL_735684, EPI_ISL_735685, EPI_ISL_735686, EPI_ISL_735687, EPI_ISL_735688, EPI_ISL_735689, EPI_ISL_735690, EPI_ISL_735691, EPI_ISL_735692, EPI_ISL_735693, EPI_ISL_735694, EPI_ISL_735695, EPI_ISL_735696, EPI_ISL_735697, EPI_ISL_735698, EPI_ISL_735699, EPI_ISL_735700, EPI_ISL_735701, EPI_ISL_735702, EPI_ISL_735703, EPI_ISL_735704, EPI_ISL_735706, EPI_ISL_735707, EPI_ISL_735708, EPI_ISL_735709, EPI_ISL_735711, EPI_ISL_735712, EPI_ISL_735713, EPI_ISL_735715, EPI_ISL_735717, EPI_ISL_735718, EPI_ISL_735719, EPI_ISL_735720, EPI_ISL_735721, EPI_ISL_735722, EPI_ISL_735724, EPI_ISL_735725, EPI_ISL_735726, EPI_ISL_735727, EPI_ISL_735728, EPI_ISL_735729, EPI_ISL_735730, EPI_ISL_735731, EPI_ISL_735732, EPI_ISL_735734, EPI_ISL_735735, EPI_ISL_735736, EPI_ISL_735737, EPI_ISL_735738, EPI_ISL_735739, EPI_ISL_735740, EPI_ISL_735741, EPI_ISL_735742, EPI_ISL_735744, EPI_ISL_735745, EPI_ISL_735747, EPI_ISL_735748, EPI_ISL_735750, EPI_ISL_735752, EPI_ISL_735754, EPI_ISL_735755, EPI_ISL_735757, EPI_ISL_735761, EPI_ISL_735763, EPI_ISL_735764, EPI_ISL_735766, EPI_ISL_735767, EPI_ISL_735768, EPI_ISL_735770, EPI_ISL_735771, EPI_ISL_735772, EPI_ISL_735774, EPI_ISL_735775, EPI_ISL_735776, EPI_ISL_735777, EPI_ISL_735780, EPI_ISL_735781, EPI_ISL_735782, EPI_ISL_735783, EPI_ISL_735784, EPI_ISL_735785, EPI_ISL_735786, EPI_ISL_735787, EPI_ISL_735788, EPI_ISL_735789, EPI_ISL_735792, EPI_ISL_735793, EPI_ISL_735795, EPI_ISL_735796, EPI_ISL_735797, EPI_ISL_735798, EPI_ISL_735799, EPI_ISL_735800, EPI_ISL_735801, EPI_ISL_735802, EPI_ISL_735804, EPI_ISL_735805, EPI_ISL_735806, EPI_ISL_735807, EPI_ISL_735808, EPI_ISL_735810, EPI_ISL_735811, EPI_ISL_735812, EPI_ISL_735813, EPI_ISL_735814, EPI_ISL_735815, EPI_ISL_735816, EPI_ISL_735817, EPI_ISL_735818, EPI_ISL_735819, EPI_ISL_735820, EPI_ISL_735821, EPI_ISL_735822, EPI_ISL_735824, EPI_ISL_735825, EPI_ISL_735826, EPI_ISL_735828, EPI_ISL_735829, EPI_ISL_735832, EPI_ISL_735833, EPI_ISL_735834, EPI_ISL_735835, EPI_ISL_735837, EPI_ISL_735838, EPI_ISL_735839, EPI_ISL_735840, EPI_ISL_735841, EPI_ISL_735842, EPI_ISL_735843, EPI_ISL_735844, EPI_ISL_735846, EPI_ISL_735849, EPI_ISL_735850, EPI_ISL_735851, EPI_ISL_735852, EPI_ISL_735854, EPI_ISL_735855, EPI_ISL_735856, EPI_ISL_735857, EPI_ISL_735858, EPI_ISL_735859

see above	Lighthouse Lab in Milton Keynes	Wellcome Sanger Institute for the COVID-19 Genomics UK (COG-UK) Consortium	The Lighthouse Lab in Milton Keynes and Alex Alderton, Roberto Amato, Sonia Goncalves, Ewan Harrison, David K. Jackson, Ian Johnston, Dominic Kwiatkowski, Cordelia Langford, John Sillitoe on behalf of the Wellcome Sanger Institute COVID-19 Surveillance Team
EPI_ISL_735861	Lighthouse Lab in Glasgow	Wellcome Sanger Institute for the COVID-19 Genomics UK (COG-UK) Consortium	Harper VanSteenhouse, Yumi Kasai, David Gray, Carol Clugston, Anna Dominiczak and Alex Alderton, Roberto Amato, Sonia Goncalves, Ewan Harrison, David K. Jackson, Ian Johnston, Dominic Kwiatkowski, Cordelia Langford, John Sillitoe on behalf of the Wellcome Sanger Institute COVID-19 Surveillance Team
EPI_ISL_735862, EPI_ISL_735863	Lighthouse Lab in Alderley Park	Wellcome Sanger Institute for the COVID-19 Genomics UK (COG-UK) Consortium	Jacquelyn Wynn, Mairead Hyland, The Lighthouse Lab in Alderley Park and Alex Alderton, Roberto Amato, Sonia Goncalves, Ewan Harrison, David K. Jackson, Ian Johnston, Dominic Kwiatkowski, Cordelia Langford, John Sillitoe on behalf of the Wellcome Sanger Institute COVID-19 Surveillance Team
EPI_ISL_735864	Lighthouse Lab in Glasgow	Wellcome Sanger Institute for the COVID-19 Genomics UK (COG-UK) Consortium	Harper VanSteenhouse, Yumi Kasai, David Gray, Carol Clugston, Anna Dominiczak and Alex Alderton, Roberto Amato, Sonia Goncalves, Ewan Harrison, David K. Jackson, Ian Johnston, Dominic Kwiatkowski, Cordelia Langford, John Sillitoe on behalf of the Wellcome Sanger Institute COVID-19 Surveillance Team
EPI_ISL_735865, EPI_ISL_735866, EPI_ISL_735868, EPI_ISL_735871, EPI_ISL_735872, EPI_ISL_735873, EPI_ISL_735874, EPI_ISL_735875, EPI_ISL_735876, EPI_ISL_735877	Lighthouse Lab in Alderley Park	Wellcome Sanger Institute for the COVID-19 Genomics UK (COG-UK) Consortium	Jacquelyn Wynn, Mairead Hyland, The Lighthouse Lab in Alderley Park and Alex Alderton, Roberto Amato, Sonia Goncalves, Ewan Harrison, David K. Jackson, Ian Johnston, Dominic Kwiatkowski, Cordelia Langford, John Sillitoe on behalf of the Wellcome Sanger Institute COVID-19 Surveillance Team
EPI_ISL_735878	Lighthouse Lab in Glasgow	Wellcome Sanger Institute for the COVID-19 Genomics UK (COG-UK) Consortium	Harper VanSteenhouse, Yumi Kasai, David Gray, Carol Clugston, Anna Dominiczak and Alex Alderton, Roberto Amato, Sonia Goncalves, Ewan Harrison, David K. Jackson, Ian Johnston, Dominic Kwiatkowski, Cordelia Langford, John Sillitoe on behalf of the Wellcome Sanger Institute COVID-19 Surveillance Team
EPI_ISL_735881, EPI_ISL_735882, EPI_ISL_735883, EPI_ISL_735887, EPI_ISL_735888, EPI_ISL_735889, EPI_ISL_735890, EPI_ISL_735891, EPI_ISL_735893	Lighthouse Lab in Alderley Park	Wellcome Sanger Institute for the COVID-19 Genomics UK (COG-UK) Consortium	Jacquelyn Wynn, Mairead Hyland, The Lighthouse Lab in Alderley Park and Alex Alderton, Roberto Amato, Sonia Goncalves, Ewan Harrison, David K. Jackson, Ian Johnston, Dominic Kwiatkowski, Cordelia Langford, John Sillitoe on behalf of the Wellcome Sanger Institute COVID-19 Surveillance Team
EPI_ISL_735894	Lighthouse Lab in Glasgow	Wellcome Sanger Institute for the COVID-19 Genomics UK (COG-UK) Consortium	Harper VanSteenhouse, Yumi Kasai, David Gray, Carol Clugston, Anna Dominiczak and Alex Alderton, Roberto Amato, Sonia Goncalves, Ewan Harrison, David K. Jackson, Ian Johnston, Dominic Kwiatkowski, Cordelia Langford, John Sillitoe on behalf of the Wellcome Sanger Institute COVID-19 Surveillance Team
EPI_ISL_735895, EPI_ISL_735898, EPI_ISL_735899, EPI_ISL_735900, EPI_ISL_735901, EPI_ISL_735902, EPI_ISL_735903, EPI_ISL_735907	Lighthouse Lab in Alderley Park	Wellcome Sanger Institute for the COVID-19 Genomics UK (COG-UK) Consortium	Jacquelyn Wynn, Mairead Hyland, The Lighthouse Lab in Alderley Park and Alex Alderton, Roberto Amato, Sonia Goncalves, Ewan Harrison, David K. Jackson, Ian Johnston, Dominic Kwiatkowski, Cordelia Langford, John Sillitoe on behalf of the Wellcome Sanger Institute COVID-19 Surveillance Team
EPI_ISL_735909	Lighthouse Lab in Glasgow	Wellcome Sanger Institute for the COVID-19 Genomics UK (COG-UK) Consortium	Harper VanSteenhouse, Yumi Kasai, David Gray, Carol Clugston, Anna Dominiczak and Alex Alderton, Roberto Amato, Sonia Goncalves, Ewan Harrison, David K. Jackson, Ian Johnston, Dominic Kwiatkowski, Cordelia Langford, John Sillitoe on behalf of the Wellcome Sanger Institute COVID-19 Surveillance Team
EPI_ISL_735913, EPI_ISL_735915, EPI_ISL_735916, EPI_ISL_735919	Lighthouse Lab in Alderley Park	Wellcome Sanger Institute for the COVID-19 Genomics UK (COG-UK) Consortium	Jacquelyn Wynn, Mairead Hyland, The Lighthouse Lab in Alderley Park and Alex Alderton, Roberto Amato, Sonia Goncalves, Ewan Harrison, David K. Jackson, Ian Johnston, Dominic Kwiatkowski, Cordelia Langford, John Sillitoe on behalf of the Wellcome Sanger Institute COVID-19 Surveillance Team
EPI_ISL_735920	Lighthouse Lab in Glasgow	Wellcome Sanger Institute for the COVID-19 Genomics UK (COG-UK) Consortium	Harper VanSteenhouse, Yumi Kasai, David Gray, Carol Clugston, Anna Dominiczak and Alex Alderton, Roberto Amato, Sonia Goncalves, Ewan Harrison, David K. Jackson, Ian Johnston, Dominic Kwiatkowski, Cordelia Langford, John Sillitoe on behalf of the Wellcome Sanger Institute COVID-19 Surveillance Team
EPI_ISL_735922, EPI_ISL_735924, EPI_ISL_735926	Lighthouse Lab in Alderley Park	Wellcome Sanger Institute for the COVID-19 Genomics UK (COG-UK) Consortium	Jacquelyn Wynn, Mairead Hyland, The Lighthouse Lab in Alderley Park and Alex Alderton, Roberto Amato, Sonia Goncalves, Ewan Harrison, David K. Jackson, Ian Johnston, Dominic Kwiatkowski, Cordelia Langford, John Sillitoe on behalf of the Wellcome Sanger Institute COVID-19 Surveillance Team
EPI_ISL_735927	Lighthouse Lab in Glasgow	Wellcome Sanger Institute for the COVID-19 Genomics UK (COG-UK) Consortium	Harper VanSteenhouse, Yumi Kasai, David Gray, Carol Clugston, Anna Dominiczak and Alex Alderton, Roberto Amato, Sonia Goncalves, Ewan Harrison, David K. Jackson, Ian Johnston, Dominic Kwiatkowski, Cordelia Langford, John Sillitoe on behalf of the Wellcome Sanger Institute COVID-19 Surveillance Team
EPI_ISL_735928, EPI_ISL_735929, EPI_ISL_735930, EPI_ISL_735934, EPI_ISL_735935	Lighthouse Lab in Alderley Park	Wellcome Sanger Institute for the COVID-19 Genomics UK (COG-UK) Consortium	Jacquelyn Wynn, Mairead Hyland, The Lighthouse Lab in Alderley Park and Alex Alderton, Roberto Amato, Sonia Goncalves, Ewan Harrison, David K. Jackson, Ian Johnston, Dominic Kwiatkowski, Cordelia Langford, John Sillitoe on behalf of the Wellcome Sanger Institute COVID-19 Surveillance Team
EPI_ISL_735937	Lighthouse Lab in Glasgow	Wellcome Sanger Institute for the COVID-19 Genomics UK (COG-UK) Consortium	Harper VanSteenhouse, Yumi Kasai, David Gray, Carol Clugston, Anna Dominiczak and Alex Alderton, Roberto Amato, Sonia Goncalves, Ewan Harrison, David K. Jackson, Ian Johnston, Dominic Kwiatkowski, Cordelia Langford, John Sillitoe on behalf of the Wellcome Sanger Institute COVID-19 Surveillance Team
EPI_ISL_735938	Lighthouse Lab in Alderley Park	Wellcome Sanger Institute for the COVID-19 Genomics UK (COG-UK) Consortium	Jacquelyn Wynn, Mairead Hyland, The Lighthouse Lab in Alderley Park and Alex Alderton, Roberto Amato, Sonia Goncalves, Ewan Harrison, David K. Jackson, Ian Johnston, Dominic Kwiatkowski, Cordelia Langford, John Sillitoe on behalf of the Wellcome Sanger Institute COVID-19 Surveillance Team
EPI_ISL_735939	Lighthouse Lab in Glasgow	Wellcome Sanger Institute for the COVID-19 Genomics UK (COG-UK) Consortium	Harper VanSteenhouse, Yumi Kasai, David Gray, Carol Clugston, Anna Dominiczak and Alex Alderton, Roberto Amato, Sonia Goncalves, Ewan Harrison, David K. Jackson, Ian Johnston, Dominic Kwiatkowski, Cordelia Langford, John Sillitoe on behalf of the Wellcome Sanger Institute COVID-19 Surveillance Team
EPI_ISL_735940, EPI_ISL_735941, EPI_ISL_735944, EPI_ISL_735946, EPI_ISL_735947, EPI_ISL_735948	Lighthouse Lab in Alderley Park	Wellcome Sanger Institute for the COVID-19 Genomics UK (COG-UK) Consortium	Jacquelyn Wynn, Mairead Hyland, The Lighthouse Lab in Alderley Park and Alex Alderton, Roberto Amato, Sonia Goncalves, Ewan Harrison, David K. Jackson, Ian Johnston, Dominic Kwiatkowski, Cordelia Langford, John Sillitoe on behalf of the Wellcome Sanger Institute COVID-19 Surveillance Team
EPI_ISL_735949	Lighthouse Lab in Glasgow	Wellcome Sanger Institute for the COVID-19 Genomics UK (COG-UK) Consortium	Harper VanSteenhouse, Yumi Kasai, David Gray, Carol Clugston, Anna Dominiczak and Alex Alderton, Roberto Amato, Sonia Goncalves, Ewan Harrison, David K. Jackson, Ian Johnston, Dominic Kwiatkowski, Cordelia Langford, John Sillitoe on behalf of the Wellcome Sanger Institute COVID-19 Surveillance Team
EPI_ISL_735950, EPI_ISL_735951	Lighthouse Lab in Alderley Park	Wellcome Sanger Institute for the COVID-19 Genomics UK (COG-UK) Consortium	Jacquelyn Wynn, Mairead Hyland, The Lighthouse Lab in Alderley Park and Alex Alderton, Roberto Amato, Sonia Goncalves, Ewan Harrison, David K. Jackson, Ian Johnston, Dominic Kwiatkowski, Cordelia Langford, John Sillitoe on behalf of the Wellcome Sanger Institute COVID-19 Surveillance Team

EPI_ISL_735952	Lighthouse Lab in Glasgow	Wellcome Sanger Institute for the COVID-19 Genomics UK (COG-UK) Consortium	Harper VanSteenhouse, Yumi Kasai, David Gray, Carol Clugston, Anna Dominiczak and Alex Alderton, Roberto Amato, Sonia Goncalves, Ewan Harrison, David K. Jackson, Ian Johnston, Dominic Kwiatkowski, Cordelia Langford, John Sillitoe on behalf of the Wellcome Sanger Institute COVID-19 Surveillance Team
EPI_ISL_735953, EPI_ISL_735954, EPI_ISL_735955	Lighthouse Lab in Alderley Park	Wellcome Sanger Institute for the COVID-19 Genomics UK (COG-UK) Consortium	Jacquelyn Wynn, Mairead Hyland, The Lighthouse Lab in Alderley Park and Alex Alderton, Roberto Amato, Sonia Goncalves, Ewan Harrison, David K. Jackson, Ian Johnston, Dominic Kwiatkowski, Cordelia Langford, John Sillitoe on behalf of the Wellcome Sanger Institute COVID-19 Surveillance Team
EPI_ISL_735956	Lighthouse Lab in Glasgow	Wellcome Sanger Institute for the COVID-19 Genomics UK (COG-UK) Consortium	Harper VanSteenhouse, Yumi Kasai, David Gray, Carol Clugston, Anna Dominiczak and Alex Alderton, Roberto Amato, Sonia Goncalves, Ewan Harrison, David K. Jackson, Ian Johnston, Dominic Kwiatkowski, Cordelia Langford, John Sillitoe on behalf of the Wellcome Sanger Institute COVID-19 Surveillance Team
EPI_ISL_735957, EPI_ISL_735958, EPI_ISL_735959, EPI_ISL_735960	Lighthouse Lab in Alderley Park	Wellcome Sanger Institute for the COVID-19 Genomics UK (COG-UK) Consortium	Jacquelyn Wynn, Mairead Hyland, The Lighthouse Lab in Alderley Park and Alex Alderton, Roberto Amato, Sonia Goncalves, Ewan Harrison, David K. Jackson, Ian Johnston, Dominic Kwiatkowski, Cordelia Langford, John Sillitoe on behalf of the Wellcome Sanger Institute COVID-19 Surveillance Team
EPI_ISL_735961	Lighthouse Lab in Glasgow	Wellcome Sanger Institute for the COVID-19 Genomics UK (COG-UK) Consortium	Harper VanSteenhouse, Yumi Kasai, David Gray, Carol Clugston, Anna Dominiczak and Alex Alderton, Roberto Amato, Sonia Goncalves, Ewan Harrison, David K. Jackson, Ian Johnston, Dominic Kwiatkowski, Cordelia Langford, John Sillitoe on behalf of the Wellcome Sanger Institute COVID-19 Surveillance Team
EPI_ISL_735962, EPI_ISL_735963, EPI_ISL_735964	Lighthouse Lab in Alderley Park	Wellcome Sanger Institute for the COVID-19 Genomics UK (COG-UK) Consortium	Jacquelyn Wynn, Mairead Hyland, The Lighthouse Lab in Alderley Park and Alex Alderton, Roberto Amato, Sonia Goncalves, Ewan Harrison, David K. Jackson, Ian Johnston, Dominic Kwiatkowski, Cordelia Langford, John Sillitoe on behalf of the Wellcome Sanger Institute COVID-19 Surveillance Team
EPI_ISL_735965	Lighthouse Lab in Glasgow	Wellcome Sanger Institute for the COVID-19 Genomics UK (COG-UK) Consortium	Harper VanSteenhouse, Yumi Kasai, David Gray, Carol Clugston, Anna Dominiczak and Alex Alderton, Roberto Amato, Sonia Goncalves, Ewan Harrison, David K. Jackson, Ian Johnston, Dominic Kwiatkowski, Cordelia Langford, John Sillitoe on behalf of the Wellcome Sanger Institute COVID-19 Surveillance Team
EPI_ISL_735966, EPI_ISL_735967, EPI_ISL_735970	Lighthouse Lab in Alderley Park	Wellcome Sanger Institute for the COVID-19 Genomics UK (COG-UK) Consortium	Jacquelyn Wynn, Mairead Hyland, The Lighthouse Lab in Alderley Park and Alex Alderton, Roberto Amato, Sonia Goncalves, Ewan Harrison, David K. Jackson, Ian Johnston, Dominic Kwiatkowski, Cordelia Langford, John Sillitoe on behalf of the Wellcome Sanger Institute COVID-19 Surveillance Team
EPI_ISL_735972	Lighthouse Lab in Glasgow	Wellcome Sanger Institute for the COVID-19 Genomics UK (COG-UK) Consortium	Harper VanSteenhouse, Yumi Kasai, David Gray, Carol Clugston, Anna Dominiczak and Alex Alderton, Roberto Amato, Sonia Goncalves, Ewan Harrison, David K. Jackson, Ian Johnston, Dominic Kwiatkowski, Cordelia Langford, John Sillitoe on behalf of the Wellcome Sanger Institute COVID-19 Surveillance Team
EPI_ISL_735974, EPI_ISL_735975, EPI_ISL_735976	Lighthouse Lab in Alderley Park	Wellcome Sanger Institute for the COVID-19 Genomics UK (COG-UK) Consortium	Jacquelyn Wynn, Mairead Hyland, The Lighthouse Lab in Alderley Park and Alex Alderton, Roberto Amato, Sonia Goncalves, Ewan Harrison, David K. Jackson, Ian Johnston, Dominic Kwiatkowski, Cordelia Langford, John Sillitoe on behalf of the Wellcome Sanger Institute COVID-19 Surveillance Team
EPI_ISL_735978	Lighthouse Lab in Glasgow	Wellcome Sanger Institute for the COVID-19 Genomics UK (COG-UK) Consortium	Harper VanSteenhouse, Yumi Kasai, David Gray, Carol Clugston, Anna Dominiczak and Alex Alderton, Roberto Amato, Sonia Goncalves, Ewan Harrison, David K. Jackson, Ian Johnston, Dominic Kwiatkowski, Cordelia Langford, John Sillitoe on behalf of the Wellcome Sanger Institute COVID-19 Surveillance Team
EPI_ISL_735980	Lighthouse Lab in Alderley Park	Wellcome Sanger Institute for the COVID-19 Genomics UK (COG-UK) Consortium	Jacquelyn Wynn, Mairead Hyland, The Lighthouse Lab in Alderley Park and Alex Alderton, Roberto Amato, Sonia Goncalves, Ewan Harrison, David K. Jackson, Ian Johnston, Dominic Kwiatkowski, Cordelia Langford, John Sillitoe on behalf of the Wellcome Sanger Institute COVID-19 Surveillance Team
EPI_ISL_735982	Lighthouse Lab in Glasgow	Wellcome Sanger Institute for the COVID-19 Genomics UK (COG-UK) Consortium	Harper VanSteenhouse, Yumi Kasai, David Gray, Carol Clugston, Anna Dominiczak and Alex Alderton, Roberto Amato, Sonia Goncalves, Ewan Harrison, David K. Jackson, Ian Johnston, Dominic Kwiatkowski, Cordelia Langford, John Sillitoe on behalf of the Wellcome Sanger Institute COVID-19 Surveillance Team
EPI_ISL_735983, EPI_ISL_735984, EPI_ISL_735986	Lighthouse Lab in Alderley Park	Wellcome Sanger Institute for the COVID-19 Genomics UK (COG-UK) Consortium	Jacquelyn Wynn, Mairead Hyland, The Lighthouse Lab in Alderley Park and Alex Alderton, Roberto Amato, Sonia Goncalves, Ewan Harrison, David K. Jackson, Ian Johnston, Dominic Kwiatkowski, Cordelia Langford, John Sillitoe on behalf of the Wellcome Sanger Institute COVID-19 Surveillance Team
EPI_ISL_735987	Lighthouse Lab in Glasgow	Wellcome Sanger Institute for the COVID-19 Genomics UK (COG-UK) Consortium	Harper VanSteenhouse, Yumi Kasai, David Gray, Carol Clugston, Anna Dominiczak and Alex Alderton, Roberto Amato, Sonia Goncalves, Ewan Harrison, David K. Jackson, Ian Johnston, Dominic Kwiatkowski, Cordelia Langford, John Sillitoe on behalf of the Wellcome Sanger Institute COVID-19 Surveillance Team
EPI_ISL_735988, EPI_ISL_735989, EPI_ISL_735990, EPI_ISL_735991, EPI_ISL_735992	Lighthouse Lab in Alderley Park	Wellcome Sanger Institute for the COVID-19 Genomics UK (COG-UK) Consortium	Jacquelyn Wynn, Mairead Hyland, The Lighthouse Lab in Alderley Park and Alex Alderton, Roberto Amato, Sonia Goncalves, Ewan Harrison, David K. Jackson, Ian Johnston, Dominic Kwiatkowski, Cordelia Langford, John Sillitoe on behalf of the Wellcome Sanger Institute COVID-19 Surveillance Team
EPI_ISL_735993	Lighthouse Lab in Glasgow	Wellcome Sanger Institute for the COVID-19 Genomics UK (COG-UK) Consortium	Harper VanSteenhouse, Yumi Kasai, David Gray, Carol Clugston, Anna Dominiczak and Alex Alderton, Roberto Amato, Sonia Goncalves, Ewan Harrison, David K. Jackson, Ian Johnston, Dominic Kwiatkowski, Cordelia Langford, John Sillitoe on behalf of the Wellcome Sanger Institute COVID-19 Surveillance Team
EPI_ISL_735994	Lighthouse Lab in Alderley Park	Wellcome Sanger Institute for the COVID-19 Genomics UK (COG-UK) Consortium	Jacquelyn Wynn, Mairead Hyland, The Lighthouse Lab in Alderley Park and Alex Alderton, Roberto Amato, Sonia Goncalves, Ewan Harrison, David K. Jackson, Ian Johnston, Dominic Kwiatkowski, Cordelia Langford, John Sillitoe on behalf of the Wellcome Sanger Institute COVID-19 Surveillance Team
EPI_ISL_735995, EPI_ISL_735996	Lighthouse Lab in Glasgow	Wellcome Sanger Institute for the COVID-19 Genomics UK (COG-UK) Consortium	Harper VanSteenhouse, Yumi Kasai, David Gray, Carol Clugston, Anna Dominiczak and Alex Alderton, Roberto Amato, Sonia Goncalves, Ewan Harrison, David K. Jackson, Ian Johnston, Dominic Kwiatkowski, Cordelia Langford, John Sillitoe on behalf of the Wellcome Sanger Institute COVID-19 Surveillance Team
EPI_ISL_735999, EPI_ISL_736000	Lighthouse Lab in Alderley Park	Wellcome Sanger Institute for the COVID-19 Genomics UK (COG-UK) Consortium	Jacquelyn Wynn, Mairead Hyland, The Lighthouse Lab in Alderley Park and Alex Alderton, Roberto Amato, Sonia Goncalves, Ewan Harrison, David K. Jackson, Ian Johnston, Dominic Kwiatkowski, Cordelia Langford, John Sillitoe on behalf of the Wellcome Sanger Institute COVID-19 Surveillance Team
EPI_ISL_736001	Lighthouse Lab in Glasgow	Wellcome Sanger Institute for the COVID-19 Genomics UK (COG-UK) Consortium	Harper VanSteenhouse, Yumi Kasai, David Gray, Carol Clugston, Anna Dominiczak and Alex Alderton, Roberto Amato, Sonia Goncalves, Ewan Harrison, David K. Jackson, Ian Johnston, Dominic Kwiatkowski, Cordelia Langford, John Sillitoe on behalf of the Wellcome Sanger Institute COVID-19 Surveillance Team
EPI_ISL_736002	Lighthouse Lab in Alderley Park	Wellcome Sanger Institute for the COVID-19 Genomics UK (COG-UK) Consortium	Jacquelyn Wynn, Mairead Hyland, The Lighthouse Lab in Alderley Park and Alex Alderton, Roberto Amato, Sonia Goncalves, Ewan Harrison, David K. Jackson, Ian Johnston, Dominic Kwiatkowski, Cordelia Langford, John Sillitoe on behalf of the Wellcome Sanger Institute COVID-19 Surveillance Team
EPI_ISL_736003	Lighthouse Lab in Glasgow	Wellcome Sanger Institute for the COVID-19 Genomics UK (COG-UK) Consortium	Harper VanSteenhouse, Yumi Kasai, David Gray, Carol Clugston, Anna Dominiczak and Alex Alderton, Roberto Amato, Sonia Goncalves, Ewan Harrison, David K. Jackson, Ian Johnston, Dominic Kwiatkowski, Cordelia Langford, John Sillitoe on behalf of the Wellcome Sanger Institute COVID-19 Surveillance Team
EPI_ISL_736004	Lighthouse Lab in Alderley Park	Wellcome Sanger Institute for the COVID-19 Genomics UK (COG-UK) Consortium	Jacquelyn Wynn, Mairead Hyland, The Lighthouse Lab in Alderley Park and Alex Alderton, Roberto Amato, Sonia Goncalves, Ewan Harrison, David K. Jackson, Ian Johnston, Dominic Kwiatkowski, Cordelia Langford, John Sillitoe on behalf of the Wellcome Sanger Institute COVID-19 Surveillance Team
EPI_ISL_736007	Lighthouse Lab in Glasgow	Wellcome Sanger Institute for the COVID-19 Genomics UK (COG-UK) Consortium	Harper VanSteenhouse, Yumi Kasai, David Gray, Carol Clugston, Anna Dominiczak and Alex Alderton, Roberto Amato, Sonia Goncalves, Ewan Harrison, David K. Jackson, Ian Johnston, Dominic Kwiatkowski, Cordelia Langford, John Sillitoe on behalf of the Wellcome Sanger Institute COVID-19 Surveillance Team
EPI_ISL_736008, EPI_ISL_736010, EPI_ISL_736011, EPI_ISL_736012, EPI_ISL_736013, EPI_ISL_736014	Lighthouse Lab in Alderley Park	Wellcome Sanger Institute for the COVID-19 Genomics UK (COG-UK) Consortium	Jacquelyn Wynn, Mairead Hyland, The Lighthouse Lab in Alderley Park and Alex Alderton, Roberto Amato, Sonia Goncalves, Ewan Harrison, David K. Jackson, Ian Johnston, Dominic Kwiatkowski, Cordelia Langford, John Sillitoe on behalf of the Wellcome Sanger Institute COVID-19 Surveillance Team
EPI_ISL_736017, EPI_ISL_736018, EPI_ISL_736019	Lighthouse Lab in Glasgow	Wellcome Sanger Institute for the COVID-19 Genomics UK (COG-UK) Consortium	Harper VanSteenhouse, Yumi Kasai, David Gray, Carol Clugston, Anna Dominiczak and Alex Alderton, Roberto Amato, Sonia Goncalves, Ewan Harrison, David K. Jackson, Ian Johnston, Dominic Kwiatkowski, Cordelia Langford, John Sillitoe on behalf of the Wellcome Sanger Institute COVID-19 Surveillance Team
EPI_ISL_736021, EPI_ISL_736022, EPI_ISL_736023	Lighthouse Lab in Alderley Park	Wellcome Sanger Institute for the COVID-19 Genomics UK (COG-UK) Consortium	Jacquelyn Wynn, Mairead Hyland, The Lighthouse Lab in Alderley Park and Alex Alderton, Roberto Amato, Sonia Goncalves, Ewan Harrison, David K. Jackson, Ian Johnston, Dominic Kwiatkowski, Cordelia Langford, John Sillitoe on behalf of the Wellcome Sanger Institute COVID-19 Surveillance Team
EPI_ISL_736024, EPI_ISL_736027	Lighthouse Lab in Glasgow	Wellcome Sanger Institute for the COVID-19 Genomics UK	Harper VanSteenhouse, Yumi Kasai, David Gray, Carol Clugston, Anna Dominiczak and Alex Alderton, Roberto Amato, Sonia Goncalves, Ewan Harrison,

		(COG-UK) Consortium	David K. Jackson, Ian Johnston, Dominic Kwiatkowski, Cordelia Langford, John Sillitoe on behalf of the Wellcome Sanger Institute COVID-19 Surveillance Team
EPI_ISL_736028, EPI_ISL_736029, EPI_ISL_736030, EPI_ISL_736032	Lighthouse Lab in Alderley Park	Wellcome Sanger Institute for the COVID-19 Genomics UK (COG-UK) Consortium	Jacquelyn Wynn, Mairead Hyland, The Lighthouse Lab in Alderley Park and Alex Alderton, Roberto Amato, Sonia Goncalves, Ewan Harrison, David K. Jackson, Ian Johnston, Dominic Kwiatkowski, Cordelia Langford, John Sillitoe on behalf of the Wellcome Sanger Institute COVID-19 Surveillance Team
EPI_ISL_736033	Lighthouse Lab in Glasgow	Wellcome Sanger Institute for the COVID-19 Genomics UK (COG-UK) Consortium	Harper VanSteenhouse, Yumi Kasai, David Gray, Carol Clugston, Anna Dominiczak and Alex Alderton, Roberto Amato, Sonia Goncalves, Ewan Harrison, David K. Jackson, Ian Johnston, Dominic Kwiatkowski, Cordelia Langford, John Sillitoe on behalf of the Wellcome Sanger Institute COVID-19 Surveillance Team
EPI_ISL_736034, EPI_ISL_736036, EPI_ISL_736037	Lighthouse Lab in Alderley Park	Wellcome Sanger Institute for the COVID-19 Genomics UK (COG-UK) Consortium	Jacquelyn Wynn, Mairead Hyland, The Lighthouse Lab in Alderley Park and Alex Alderton, Roberto Amato, Sonia Goncalves, Ewan Harrison, David K. Jackson, Ian Johnston, Dominic Kwiatkowski, Cordelia Langford, John Sillitoe on behalf of the Wellcome Sanger Institute COVID-19 Surveillance Team
EPI_ISL_736038	Lighthouse Lab in Glasgow	Wellcome Sanger Institute for the COVID-19 Genomics UK (COG-UK) Consortium	Harper VanSteenhouse, Yumi Kasai, David Gray, Carol Clugston, Anna Dominiczak and Alex Alderton, Roberto Amato, Sonia Goncalves, Ewan Harrison, David K. Jackson, Ian Johnston, Dominic Kwiatkowski, Cordelia Langford, John Sillitoe on behalf of the Wellcome Sanger Institute COVID-19 Surveillance Team
EPI_ISL_736039, EPI_ISL_736040	Lighthouse Lab in Alderley Park	Wellcome Sanger Institute for the COVID-19 Genomics UK (COG-UK) Consortium	Jacquelyn Wynn, Mairead Hyland, The Lighthouse Lab in Alderley Park and Alex Alderton, Roberto Amato, Sonia Goncalves, Ewan Harrison, David K. Jackson, Ian Johnston, Dominic Kwiatkowski, Cordelia Langford, John Sillitoe on behalf of the Wellcome Sanger Institute COVID-19 Surveillance Team
EPI_ISL_736041	Lighthouse Lab in Glasgow	Wellcome Sanger Institute for the COVID-19 Genomics UK (COG-UK) Consortium	Harper VanSteenhouse, Yumi Kasai, David Gray, Carol Clugston, Anna Dominiczak and Alex Alderton, Roberto Amato, Sonia Goncalves, Ewan Harrison, David K. Jackson, Ian Johnston, Dominic Kwiatkowski, Cordelia Langford, John Sillitoe on behalf of the Wellcome Sanger Institute COVID-19 Surveillance Team
EPI_ISL_736042, EPI_ISL_736043, EPI_ISL_736044, EPI_ISL_736045, EPI_ISL_736046, EPI_ISL_736047, EPI_ISL_736048, EPI_ISL_736050, EPI_ISL_736051	Lighthouse Lab in Alderley Park	Wellcome Sanger Institute for the COVID-19 Genomics UK (COG-UK) Consortium	Jacquelyn Wynn, Mairead Hyland, The Lighthouse Lab in Alderley Park and Alex Alderton, Roberto Amato, Sonia Goncalves, Ewan Harrison, David K. Jackson, Ian Johnston, Dominic Kwiatkowski, Cordelia Langford, John Sillitoe on behalf of the Wellcome Sanger Institute COVID-19 Surveillance Team
EPI_ISL_736052	Lighthouse Lab in Glasgow	Wellcome Sanger Institute for the COVID-19 Genomics UK (COG-UK) Consortium	Harper VanSteenhouse, Yumi Kasai, David Gray, Carol Clugston, Anna Dominiczak and Alex Alderton, Roberto Amato, Sonia Goncalves, Ewan Harrison, David K. Jackson, Ian Johnston, Dominic Kwiatkowski, Cordelia Langford, John Sillitoe on behalf of the Wellcome Sanger Institute COVID-19 Surveillance Team
EPI_ISL_736053, EPI_ISL_736054, EPI_ISL_736055, EPI_ISL_736056	Lighthouse Lab in Alderley Park	Wellcome Sanger Institute for the COVID-19 Genomics UK (COG-UK) Consortium	Jacquelyn Wynn, Mairead Hyland, The Lighthouse Lab in Alderley Park and Alex Alderton, Roberto Amato, Sonia Goncalves, Ewan Harrison, David K. Jackson, Ian Johnston, Dominic Kwiatkowski, Cordelia Langford, John Sillitoe on behalf of the Wellcome Sanger Institute COVID-19 Surveillance Team
EPI_ISL_736057, EPI_ISL_736059	Lighthouse Lab in Glasgow	Wellcome Sanger Institute for the COVID-19 Genomics UK (COG-UK) Consortium	Harper VanSteenhouse, Yumi Kasai, David Gray, Carol Clugston, Anna Dominiczak and Alex Alderton, Roberto Amato, Sonia Goncalves, Ewan Harrison, David K. Jackson, Ian Johnston, Dominic Kwiatkowski, Cordelia Langford, John Sillitoe on behalf of the Wellcome Sanger Institute COVID-19 Surveillance Team
EPI_ISL_736060, EPI_ISL_736061, EPI_ISL_736062, EPI_ISL_736063	Lighthouse Lab in Alderley Park	Wellcome Sanger Institute for the COVID-19 Genomics UK (COG-UK) Consortium	Jacquelyn Wynn, Mairead Hyland, The Lighthouse Lab in Alderley Park and Alex Alderton, Roberto Amato, Sonia Goncalves, Ewan Harrison, David K. Jackson, Ian Johnston, Dominic Kwiatkowski, Cordelia Langford, John Sillitoe on behalf of the Wellcome Sanger Institute COVID-19 Surveillance Team
EPI_ISL_736066	Lighthouse Lab in Glasgow	Wellcome Sanger Institute for the COVID-19 Genomics UK (COG-UK) Consortium	Harper VanSteenhouse, Yumi Kasai, David Gray, Carol Clugston, Anna Dominiczak and Alex Alderton, Roberto Amato, Sonia Goncalves, Ewan Harrison, David K. Jackson, Ian Johnston, Dominic Kwiatkowski, Cordelia Langford, John Sillitoe on behalf of the Wellcome Sanger Institute COVID-19 Surveillance Team
EPI_ISL_736067, EPI_ISL_736068, EPI_ISL_736069, EPI_ISL_736070, EPI_ISL_736071	Lighthouse Lab in Alderley Park	Wellcome Sanger Institute for the COVID-19 Genomics UK (COG-UK) Consortium	Jacquelyn Wynn, Mairead Hyland, The Lighthouse Lab in Alderley Park and Alex Alderton, Roberto Amato, Sonia Goncalves, Ewan Harrison, David K. Jackson, Ian Johnston, Dominic Kwiatkowski, Cordelia Langford, John Sillitoe on behalf of the Wellcome Sanger Institute COVID-19 Surveillance Team
EPI_ISL_736072	Lighthouse Lab in Glasgow	Wellcome Sanger Institute for the COVID-19 Genomics UK (COG-UK) Consortium	Harper VanSteenhouse, Yumi Kasai, David Gray, Carol Clugston, Anna Dominiczak and Alex Alderton, Roberto Amato, Sonia Goncalves, Ewan Harrison, David K. Jackson, Ian Johnston, Dominic Kwiatkowski, Cordelia Langford, John Sillitoe on behalf of the Wellcome Sanger Institute COVID-19 Surveillance Team
EPI_ISL_736073, EPI_ISL_736074, EPI_ISL_736076, EPI_ISL_736078, EPI_ISL_736079, EPI_ISL_736080, EPI_ISL_736081, EPI_ISL_736083, EPI_ISL_736084, EPI_ISL_736086, EPI_ISL_736087, EPI_ISL_736088, EPI_ISL_736089			
see above	Lighthouse Lab in Alderley Park	Wellcome Sanger Institute for the COVID-19 Genomics UK (COG-UK) Consortium	Jacquelyn Wynn, Mairead Hyland, The Lighthouse Lab in Alderley Park and Alex Alderton, Roberto Amato, Sonia Goncalves, Ewan Harrison, David K. Jackson, Ian Johnston, Dominic Kwiatkowski, Cordelia Langford, John Sillitoe on behalf of the Wellcome Sanger Institute COVID-19 Surveillance Team
EPI_ISL_736090, EPI_ISL_736091, EPI_ISL_736092	Lighthouse Lab in Glasgow	Wellcome Sanger Institute for the COVID-19 Genomics UK (COG-UK) Consortium	Harper VanSteenhouse, Yumi Kasai, David Gray, Carol Clugston, Anna Dominiczak and Alex Alderton, Roberto Amato, Sonia Goncalves, Ewan Harrison, David K. Jackson, Ian Johnston, Dominic Kwiatkowski, Cordelia Langford, John Sillitoe on behalf of the Wellcome Sanger Institute COVID-19 Surveillance Team
EPI_ISL_736093	Lighthouse Lab in Alderley Park	Wellcome Sanger Institute for the COVID-19 Genomics UK (COG-UK) Consortium	Jacquelyn Wynn, Mairead Hyland, The Lighthouse Lab in Alderley Park and Alex Alderton, Roberto Amato, Sonia Goncalves, Ewan Harrison, David K. Jackson, Ian Johnston, Dominic Kwiatkowski, Cordelia Langford, John Sillitoe on behalf of the Wellcome Sanger Institute COVID-19 Surveillance Team
EPI_ISL_736096	Lighthouse Lab in Glasgow	Wellcome Sanger Institute for the COVID-19 Genomics UK (COG-UK) Consortium	Harper VanSteenhouse, Yumi Kasai, David Gray, Carol Clugston, Anna Dominiczak and Alex Alderton, Roberto Amato, Sonia Goncalves, Ewan Harrison, David K. Jackson, Ian Johnston, Dominic Kwiatkowski, Cordelia Langford, John Sillitoe on behalf of the Wellcome Sanger Institute COVID-19 Surveillance Team
EPI_ISL_736097, EPI_ISL_736098	Lighthouse Lab in Alderley Park	Wellcome Sanger Institute for the COVID-19 Genomics UK (COG-UK) Consortium	Jacquelyn Wynn, Mairead Hyland, The Lighthouse Lab in Alderley Park and Alex Alderton, Roberto Amato, Sonia Goncalves, Ewan Harrison, David K. Jackson, Ian Johnston, Dominic Kwiatkowski, Cordelia Langford, John Sillitoe on behalf of the Wellcome Sanger Institute COVID-19 Surveillance Team
EPI_ISL_736099, EPI_ISL_736102, EPI_ISL_736104	Lighthouse Lab in Glasgow	Wellcome Sanger Institute for the COVID-19 Genomics UK (COG-UK) Consortium	Harper VanSteenhouse, Yumi Kasai, David Gray, Carol Clugston, Anna Dominiczak and Alex Alderton, Roberto Amato, Sonia Goncalves, Ewan Harrison, David K. Jackson, Ian Johnston, Dominic Kwiatkowski, Cordelia Langford, John Sillitoe on behalf of the Wellcome Sanger Institute COVID-19 Surveillance Team
EPI_ISL_736106	Lighthouse Lab in Alderley Park	Wellcome Sanger Institute for the COVID-19 Genomics UK (COG-UK) Consortium	Jacquelyn Wynn, Mairead Hyland, The Lighthouse Lab in Alderley Park and Alex Alderton, Roberto Amato, Sonia Goncalves, Ewan Harrison, David K. Jackson, Ian Johnston, Dominic Kwiatkowski, Cordelia Langford, John Sillitoe on behalf of the Wellcome Sanger Institute COVID-19 Surveillance Team
EPI_ISL_736107	Lighthouse Lab in Glasgow	Wellcome Sanger Institute for the COVID-19 Genomics UK (COG-UK) Consortium	Harper VanSteenhouse, Yumi Kasai, David Gray, Carol Clugston, Anna Dominiczak and Alex Alderton, Roberto Amato, Sonia Goncalves, Ewan Harrison, David K. Jackson, Ian Johnston, Dominic Kwiatkowski, Cordelia Langford, John Sillitoe on behalf of the Wellcome Sanger Institute COVID-19 Surveillance Team
EPI_ISL_736108, EPI_ISL_736109, EPI_ISL_736110, EPI_ISL_736111, EPI_ISL_736112, EPI_ISL_736114, EPI_ISL_736116, EPI_ISL_736117, EPI_ISL_736119, EPI_ISL_736121, EPI_ISL_736123, EPI_ISL_736125, EPI_ISL_736127, EPI_ISL_736128, EPI_ISL_736129			
see above	Lighthouse Lab in Alderley Park	Wellcome Sanger Institute for the COVID-19 Genomics UK (COG-UK) Consortium	Jacquelyn Wynn, Mairead Hyland, The Lighthouse Lab in Alderley Park and Alex Alderton, Roberto Amato, Sonia Goncalves, Ewan Harrison, David K. Jackson, Ian Johnston, Dominic Kwiatkowski, Cordelia Langford, John Sillitoe on behalf of the Wellcome Sanger Institute COVID-19 Surveillance Team
EPI_ISL_736130, EPI_ISL_736131, EPI_ISL_736132	Lighthouse Lab in Glasgow	Wellcome Sanger Institute for the COVID-19 Genomics UK (COG-UK) Consortium	Harper VanSteenhouse, Yumi Kasai, David Gray, Carol Clugston, Anna Dominiczak and Alex Alderton, Roberto Amato, Sonia Goncalves, Ewan Harrison, David K. Jackson, Ian Johnston, Dominic Kwiatkowski, Cordelia Langford, John Sillitoe on behalf of the Wellcome Sanger Institute COVID-19 Surveillance Team
EPI_ISL_736133	Lighthouse Lab in Alderley Park	Wellcome Sanger Institute for the COVID-19 Genomics UK (COG-UK) Consortium	Jacquelyn Wynn, Mairead Hyland, The Lighthouse Lab in Alderley Park and Alex Alderton, Roberto Amato, Sonia Goncalves, Ewan Harrison, David K. Jackson, Ian Johnston, Dominic Kwiatkowski, Cordelia Langford, John Sillitoe on behalf of the Wellcome Sanger Institute COVID-19 Surveillance Team
EPI_ISL_736135	Lighthouse Lab in Glasgow	Wellcome Sanger Institute for the COVID-19 Genomics UK (COG-UK) Consortium	Harper VanSteenhouse, Yumi Kasai, David Gray, Carol Clugston, Anna Dominiczak and Alex Alderton, Roberto Amato, Sonia Goncalves, Ewan Harrison, David K. Jackson, Ian Johnston, Dominic Kwiatkowski, Cordelia Langford, John Sillitoe on behalf of the Wellcome Sanger Institute COVID-19 Surveillance Team

EPI_ISL_736136, EPI_ISL_736137, EPI_ISL_736138, EPI_ISL_736139, EPI_ISL_736141, EPI_ISL_736142	Lighthouse Lab in Alderley Park	Wellcome Sanger Institute for the COVID-19 Genomics UK (COG-UK) Consortium	Jacquelyn Wynn, Mairead Hyland, The Lighthouse Lab in Alderley Park and Alex Alderton, Roberto Amato, Sonia Goncalves, Ewan Harrison, David K. Jackson, Ian Johnston, Dominic Kwiatkowski, Cordelia Langford, John Sillitoe on behalf of the Wellcome Sanger Institute COVID-19 Surveillance Team
EPI_ISL_736143	Lighthouse Lab in Glasgow	Wellcome Sanger Institute for the COVID-19 Genomics UK (COG-UK) Consortium	Harper VanSteenhouse, Yumi Kasai, David Gray, Carol Clugston, Anna Dominiczak and Alex Alderton, Roberto Amato, Sonia Goncalves, Ewan Harrison, David K. Jackson, Ian Johnston, Dominic Kwiatkowski, Cordelia Langford, John Sillitoe on behalf of the Wellcome Sanger Institute COVID-19 Surveillance Team
EPI_ISL_736145	Lighthouse Lab in Alderley Park	Wellcome Sanger Institute for the COVID-19 Genomics UK (COG-UK) Consortium	Jacquelyn Wynn, Mairead Hyland, The Lighthouse Lab in Alderley Park and Alex Alderton, Roberto Amato, Sonia Goncalves, Ewan Harrison, David K. Jackson, Ian Johnston, Dominic Kwiatkowski, Cordelia Langford, John Sillitoe on behalf of the Wellcome Sanger Institute COVID-19 Surveillance Team
EPI_ISL_736146	Lighthouse Lab in Glasgow	Wellcome Sanger Institute for the COVID-19 Genomics UK (COG-UK) Consortium	Harper VanSteenhouse, Yumi Kasai, David Gray, Carol Clugston, Anna Dominiczak and Alex Alderton, Roberto Amato, Sonia Goncalves, Ewan Harrison, David K. Jackson, Ian Johnston, Dominic Kwiatkowski, Cordelia Langford, John Sillitoe on behalf of the Wellcome Sanger Institute COVID-19 Surveillance Team
EPI_ISL_736147, EPI_ISL_736148, EPI_ISL_736150	Lighthouse Lab in Alderley Park	Wellcome Sanger Institute for the COVID-19 Genomics UK (COG-UK) Consortium	Jacquelyn Wynn, Mairead Hyland, The Lighthouse Lab in Alderley Park and Alex Alderton, Roberto Amato, Sonia Goncalves, Ewan Harrison, David K. Jackson, Ian Johnston, Dominic Kwiatkowski, Cordelia Langford, John Sillitoe on behalf of the Wellcome Sanger Institute COVID-19 Surveillance Team
EPI_ISL_736151	Lighthouse Lab in Glasgow	Wellcome Sanger Institute for the COVID-19 Genomics UK (COG-UK) Consortium	Harper VanSteenhouse, Yumi Kasai, David Gray, Carol Clugston, Anna Dominiczak and Alex Alderton, Roberto Amato, Sonia Goncalves, Ewan Harrison, David K. Jackson, Ian Johnston, Dominic Kwiatkowski, Cordelia Langford, John Sillitoe on behalf of the Wellcome Sanger Institute COVID-19 Surveillance Team
EPI_ISL_736155, EPI_ISL_736157, EPI_ISL_736158	Lighthouse Lab in Alderley Park	Wellcome Sanger Institute for the COVID-19 Genomics UK (COG-UK) Consortium	Jacquelyn Wynn, Mairead Hyland, The Lighthouse Lab in Alderley Park and Alex Alderton, Roberto Amato, Sonia Goncalves, Ewan Harrison, David K. Jackson, Ian Johnston, Dominic Kwiatkowski, Cordelia Langford, John Sillitoe on behalf of the Wellcome Sanger Institute COVID-19 Surveillance Team
EPI_ISL_736159	Lighthouse Lab in Glasgow	Wellcome Sanger Institute for the COVID-19 Genomics UK (COG-UK) Consortium	Harper VanSteenhouse, Yumi Kasai, David Gray, Carol Clugston, Anna Dominiczak and Alex Alderton, Roberto Amato, Sonia Goncalves, Ewan Harrison, David K. Jackson, Ian Johnston, Dominic Kwiatkowski, Cordelia Langford, John Sillitoe on behalf of the Wellcome Sanger Institute COVID-19 Surveillance Team
EPI_ISL_736160, EPI_ISL_736161	Lighthouse Lab in Alderley Park	Wellcome Sanger Institute for the COVID-19 Genomics UK (COG-UK) Consortium	Jacquelyn Wynn, Mairead Hyland, The Lighthouse Lab in Alderley Park and Alex Alderton, Roberto Amato, Sonia Goncalves, Ewan Harrison, David K. Jackson, Ian Johnston, Dominic Kwiatkowski, Cordelia Langford, John Sillitoe on behalf of the Wellcome Sanger Institute COVID-19 Surveillance Team
EPI_ISL_736162	Lighthouse Lab in Glasgow	Wellcome Sanger Institute for the COVID-19 Genomics UK (COG-UK) Consortium	Harper VanSteenhouse, Yumi Kasai, David Gray, Carol Clugston, Anna Dominiczak and Alex Alderton, Roberto Amato, Sonia Goncalves, Ewan Harrison, David K. Jackson, Ian Johnston, Dominic Kwiatkowski, Cordelia Langford, John Sillitoe on behalf of the Wellcome Sanger Institute COVID-19 Surveillance Team
EPI_ISL_736164	Lighthouse Lab in Alderley Park	Wellcome Sanger Institute for the COVID-19 Genomics UK (COG-UK) Consortium	Jacquelyn Wynn, Mairead Hyland, The Lighthouse Lab in Alderley Park and Alex Alderton, Roberto Amato, Sonia Goncalves, Ewan Harrison, David K. Jackson, Ian Johnston, Dominic Kwiatkowski, Cordelia Langford, John Sillitoe on behalf of the Wellcome Sanger Institute COVID-19 Surveillance Team
EPI_ISL_736168, EPI_ISL_736169	Lighthouse Lab in Glasgow	Wellcome Sanger Institute for the COVID-19 Genomics UK (COG-UK) Consortium	Harper VanSteenhouse, Yumi Kasai, David Gray, Carol Clugston, Anna Dominiczak and Alex Alderton, Roberto Amato, Sonia Goncalves, Ewan Harrison, David K. Jackson, Ian Johnston, Dominic Kwiatkowski, Cordelia Langford, John Sillitoe on behalf of the Wellcome Sanger Institute COVID-19 Surveillance Team
EPI_ISL_736170, EPI_ISL_736172, EPI_ISL_736174, EPI_ISL_736177, EPI_ISL_736179	Lighthouse Lab in Alderley Park	Wellcome Sanger Institute for the COVID-19 Genomics UK (COG-UK) Consortium	Jacquelyn Wynn, Mairead Hyland, The Lighthouse Lab in Alderley Park and Alex Alderton, Roberto Amato, Sonia Goncalves, Ewan Harrison, David K. Jackson, Ian Johnston, Dominic Kwiatkowski, Cordelia Langford, John Sillitoe on behalf of the Wellcome Sanger Institute COVID-19 Surveillance Team
EPI_ISL_736183, EPI_ISL_736184	Lighthouse Lab in Glasgow	Wellcome Sanger Institute for the COVID-19 Genomics UK (COG-UK) Consortium	Harper VanSteenhouse, Yumi Kasai, David Gray, Carol Clugston, Anna Dominiczak and Alex Alderton, Roberto Amato, Sonia Goncalves, Ewan Harrison, David K. Jackson, Ian Johnston, Dominic Kwiatkowski, Cordelia Langford, John Sillitoe on behalf of the Wellcome Sanger Institute COVID-19 Surveillance Team
EPI_ISL_736186, EPI_ISL_736187	Lighthouse Lab in Alderley Park	Wellcome Sanger Institute for the COVID-19 Genomics UK (COG-UK) Consortium	Jacquelyn Wynn, Mairead Hyland, The Lighthouse Lab in Alderley Park and Alex Alderton, Roberto Amato, Sonia Goncalves, Ewan Harrison, David K. Jackson, Ian Johnston, Dominic Kwiatkowski, Cordelia Langford, John Sillitoe on behalf of the Wellcome Sanger Institute COVID-19 Surveillance Team
EPI_ISL_736190, EPI_ISL_736191	Lighthouse Lab in Glasgow	Wellcome Sanger Institute for the COVID-19 Genomics UK (COG-UK) Consortium	Harper VanSteenhouse, Yumi Kasai, David Gray, Carol Clugston, Anna Dominiczak and Alex Alderton, Roberto Amato, Sonia Goncalves, Ewan Harrison, David K. Jackson, Ian Johnston, Dominic Kwiatkowski, Cordelia Langford, John Sillitoe on behalf of the Wellcome Sanger Institute COVID-19 Surveillance Team
EPI_ISL_736193, EPI_ISL_736194, EPI_ISL_736195, EPI_ISL_736197, EPI_ISL_736198, EPI_ISL_736199, EPI_ISL_736200	Lighthouse Lab in Alderley Park	Wellcome Sanger Institute for the COVID-19 Genomics UK (COG-UK) Consortium	Jacquelyn Wynn, Mairead Hyland, The Lighthouse Lab in Alderley Park and Alex Alderton, Roberto Amato, Sonia Goncalves, Ewan Harrison, David K. Jackson, Ian Johnston, Dominic Kwiatkowski, Cordelia Langford, John Sillitoe on behalf of the Wellcome Sanger Institute COVID-19 Surveillance Team
EPI_ISL_736202, EPI_ISL_736203, EPI_ISL_736208	Lighthouse Lab in Milton Keynes	Wellcome Sanger Institute for the COVID-19 Genomics UK (COG-UK) Consortium	The Lighthouse Lab in Milton Keynes and Alex Alderton, Roberto Amato, Sonia Goncalves, Ewan Harrison, David K. Jackson, Ian Johnston, Dominic Kwiatkowski, Cordelia Langford, John Sillitoe on behalf of the Wellcome Sanger Institute COVID-19 Surveillance Team
EPI_ISL_736209	Lighthouse Lab in Glasgow	Wellcome Sanger Institute for the COVID-19 Genomics UK (COG-UK) Consortium	Harper VanSteenhouse, Yumi Kasai, David Gray, Carol Clugston, Anna Dominiczak and Alex Alderton, Roberto Amato, Sonia Goncalves, Ewan Harrison, David K. Jackson, Ian Johnston, Dominic Kwiatkowski, Cordelia Langford, John Sillitoe on behalf of the Wellcome Sanger Institute COVID-19 Surveillance Team
EPI_ISL_736211, EPI_ISL_736215, EPI_ISL_736216, EPI_ISL_736217	Lighthouse Lab in Milton Keynes	Wellcome Sanger Institute for the COVID-19 Genomics UK (COG-UK) Consortium	The Lighthouse Lab in Milton Keynes and Alex Alderton, Roberto Amato, Sonia Goncalves, Ewan Harrison, David K. Jackson, Ian Johnston, Dominic Kwiatkowski, Cordelia Langford, John Sillitoe on behalf of the Wellcome Sanger Institute COVID-19 Surveillance Team
EPI_ISL_736220	Lighthouse Lab in Glasgow	Wellcome Sanger Institute for the COVID-19 Genomics UK (COG-UK) Consortium	Harper VanSteenhouse, Yumi Kasai, David Gray, Carol Clugston, Anna Dominiczak and Alex Alderton, Roberto Amato, Sonia Goncalves, Ewan Harrison, David K. Jackson, Ian Johnston, Dominic Kwiatkowski, Cordelia Langford, John Sillitoe on behalf of the Wellcome Sanger Institute COVID-19 Surveillance Team
EPI_ISL_736224, EPI_ISL_736225, EPI_ISL_736226, EPI_ISL_736227, EPI_ISL_736233	Lighthouse Lab in Milton Keynes	Wellcome Sanger Institute for the COVID-19 Genomics UK (COG-UK) Consortium	The Lighthouse Lab in Milton Keynes and Alex Alderton, Roberto Amato, Sonia Goncalves, Ewan Harrison, David K. Jackson, Ian Johnston, Dominic Kwiatkowski, Cordelia Langford, John Sillitoe on behalf of the Wellcome Sanger Institute COVID-19 Surveillance Team
EPI_ISL_736241	Lighthouse Lab in Glasgow	Wellcome Sanger Institute for the COVID-19 Genomics UK (COG-UK) Consortium	Harper VanSteenhouse, Yumi Kasai, David Gray, Carol Clugston, Anna Dominiczak and Alex Alderton, Roberto Amato, Sonia Goncalves, Ewan Harrison, David K. Jackson, Ian Johnston, Dominic Kwiatkowski, Cordelia Langford, John Sillitoe on behalf of the Wellcome Sanger Institute COVID-19 Surveillance Team
EPI_ISL_736244	Lighthouse Lab in Milton Keynes	Wellcome Sanger Institute for the COVID-19 Genomics UK (COG-UK) Consortium	The Lighthouse Lab in Milton Keynes and Alex Alderton, Roberto Amato, Sonia Goncalves, Ewan Harrison, David K. Jackson, Ian Johnston, Dominic Kwiatkowski, Cordelia Langford, John Sillitoe on behalf of the Wellcome Sanger Institute COVID-19 Surveillance Team
EPI_ISL_736248	Lighthouse Lab in Glasgow	Wellcome Sanger Institute for the COVID-19 Genomics UK (COG-UK) Consortium	Harper VanSteenhouse, Yumi Kasai, David Gray, Carol Clugston, Anna Dominiczak and Alex Alderton, Roberto Amato, Sonia Goncalves, Ewan Harrison, David K. Jackson, Ian Johnston, Dominic Kwiatkowski, Cordelia Langford, John Sillitoe on behalf of the Wellcome Sanger Institute COVID-19 Surveillance Team
EPI_ISL_736249	Lighthouse Lab in Milton Keynes	Wellcome Sanger Institute for the COVID-19 Genomics UK (COG-UK) Consortium	The Lighthouse Lab in Milton Keynes and Alex Alderton, Roberto Amato, Sonia Goncalves, Ewan Harrison, David K. Jackson, Ian Johnston, Dominic Kwiatkowski, Cordelia Langford, John Sillitoe on behalf of the Wellcome Sanger Institute COVID-19 Surveillance Team
EPI_ISL_736250	Lighthouse Lab in Glasgow	Wellcome Sanger Institute for the COVID-19 Genomics UK (COG-UK) Consortium	Harper VanSteenhouse, Yumi Kasai, David Gray, Carol Clugston, Anna Dominiczak and Alex Alderton, Roberto Amato, Sonia Goncalves, Ewan Harrison, David K. Jackson, Ian Johnston, Dominic Kwiatkowski, Cordelia Langford, John Sillitoe on behalf of the Wellcome Sanger Institute COVID-19 Surveillance Team
EPI_ISL_736251	Lighthouse Lab in Milton Keynes	Wellcome Sanger Institute for the COVID-19 Genomics UK (COG-UK) Consortium	The Lighthouse Lab in Milton Keynes and Alex Alderton, Roberto Amato, Sonia Goncalves, Ewan Harrison, David K. Jackson, Ian Johnston, Dominic Kwiatkowski, Cordelia Langford, John Sillitoe on behalf of the Wellcome Sanger Institute COVID-19 Surveillance Team

EPI_ISL_736254	Lighthouse Lab in Glasgow	Wellcome Sanger Institute for the COVID-19 Genomics UK (COG-UK) Consortium	Harper VanSteenhouse, Yumi Kasai, David Gray, Carol Clugston, Anna Dominiczak and Alex Alderton, Roberto Amato, Sonia Goncalves, Ewan Harrison, David K. Jackson, Ian Johnston, Dominic Kwiatkowski, Cordelia Langford, John Sillitoe on behalf of the Wellcome Sanger Institute COVID-19 Surveillance Team
EPI_ISL_736256, EPI_ISL_736260	Lighthouse Lab in Milton Keynes	Wellcome Sanger Institute for the COVID-19 Genomics UK (COG-UK) Consortium	The Lighthouse Lab in Milton Keynes and Alex Alderton, Roberto Amato, Sonia Goncalves, Ewan Harrison, David K. Jackson, Ian Johnston, Dominic Kwiatkowski, Cordelia Langford, John Sillitoe on behalf of the Wellcome Sanger Institute COVID-19 Surveillance Team
EPI_ISL_736261	Lighthouse Lab in Glasgow	Wellcome Sanger Institute for the COVID-19 Genomics UK (COG-UK) Consortium	Harper VanSteenhouse, Yumi Kasai, David Gray, Carol Clugston, Anna Dominiczak and Alex Alderton, Roberto Amato, Sonia Goncalves, Ewan Harrison, David K. Jackson, Ian Johnston, Dominic Kwiatkowski, Cordelia Langford, John Sillitoe on behalf of the Wellcome Sanger Institute COVID-19 Surveillance Team
EPI_ISL_736262, EPI_ISL_736264, EPI_ISL_736267, EPI_ISL_736268, EPI_ISL_736270, EPI_ISL_736274	Lighthouse Lab in Milton Keynes	Wellcome Sanger Institute for the COVID-19 Genomics UK (COG-UK) Consortium	The Lighthouse Lab in Milton Keynes and Alex Alderton, Roberto Amato, Sonia Goncalves, Ewan Harrison, David K. Jackson, Ian Johnston, Dominic Kwiatkowski, Cordelia Langford, John Sillitoe on behalf of the Wellcome Sanger Institute COVID-19 Surveillance Team
EPI_ISL_736275, EPI_ISL_736277	Lighthouse Lab in Glasgow	Wellcome Sanger Institute for the COVID-19 Genomics UK (COG-UK) Consortium	Harper VanSteenhouse, Yumi Kasai, David Gray, Carol Clugston, Anna Dominiczak and Alex Alderton, Roberto Amato, Sonia Goncalves, Ewan Harrison, David K. Jackson, Ian Johnston, Dominic Kwiatkowski, Cordelia Langford, John Sillitoe on behalf of the Wellcome Sanger Institute COVID-19 Surveillance Team
EPI_ISL_736282, EPI_ISL_736283	Lighthouse Lab in Milton Keynes	Wellcome Sanger Institute for the COVID-19 Genomics UK (COG-UK) Consortium	The Lighthouse Lab in Milton Keynes and Alex Alderton, Roberto Amato, Sonia Goncalves, Ewan Harrison, David K. Jackson, Ian Johnston, Dominic Kwiatkowski, Cordelia Langford, John Sillitoe on behalf of the Wellcome Sanger Institute COVID-19 Surveillance Team
EPI_ISL_736284	Lighthouse Lab in Glasgow	Wellcome Sanger Institute for the COVID-19 Genomics UK (COG-UK) Consortium	Harper VanSteenhouse, Yumi Kasai, David Gray, Carol Clugston, Anna Dominiczak and Alex Alderton, Roberto Amato, Sonia Goncalves, Ewan Harrison, David K. Jackson, Ian Johnston, Dominic Kwiatkowski, Cordelia Langford, John Sillitoe on behalf of the Wellcome Sanger Institute COVID-19 Surveillance Team
EPI_ISL_736286	Lighthouse Lab in Milton Keynes	Wellcome Sanger Institute for the COVID-19 Genomics UK (COG-UK) Consortium	The Lighthouse Lab in Milton Keynes and Alex Alderton, Roberto Amato, Sonia Goncalves, Ewan Harrison, David K. Jackson, Ian Johnston, Dominic Kwiatkowski, Cordelia Langford, John Sillitoe on behalf of the Wellcome Sanger Institute COVID-19 Surveillance Team
EPI_ISL_736287	Lighthouse Lab in Glasgow	Wellcome Sanger Institute for the COVID-19 Genomics UK (COG-UK) Consortium	Harper VanSteenhouse, Yumi Kasai, David Gray, Carol Clugston, Anna Dominiczak and Alex Alderton, Roberto Amato, Sonia Goncalves, Ewan Harrison, David K. Jackson, Ian Johnston, Dominic Kwiatkowski, Cordelia Langford, John Sillitoe on behalf of the Wellcome Sanger Institute COVID-19 Surveillance Team
EPI_ISL_736288	Lighthouse Lab in Milton Keynes	Wellcome Sanger Institute for the COVID-19 Genomics UK (COG-UK) Consortium	The Lighthouse Lab in Milton Keynes and Alex Alderton, Roberto Amato, Sonia Goncalves, Ewan Harrison, David K. Jackson, Ian Johnston, Dominic Kwiatkowski, Cordelia Langford, John Sillitoe on behalf of the Wellcome Sanger Institute COVID-19 Surveillance Team
EPI_ISL_736290	Lighthouse Lab in Glasgow	Wellcome Sanger Institute for the COVID-19 Genomics UK (COG-UK) Consortium	Harper VanSteenhouse, Yumi Kasai, David Gray, Carol Clugston, Anna Dominiczak and Alex Alderton, Roberto Amato, Sonia Goncalves, Ewan Harrison, David K. Jackson, Ian Johnston, Dominic Kwiatkowski, Cordelia Langford, John Sillitoe on behalf of the Wellcome Sanger Institute COVID-19 Surveillance Team
EPI_ISL_736291, EPI_ISL_736294, EPI_ISL_736298, EPI_ISL_736299, EPI_ISL_736302	Lighthouse Lab in Milton Keynes	Wellcome Sanger Institute for the COVID-19 Genomics UK (COG-UK) Consortium	The Lighthouse Lab in Milton Keynes and Alex Alderton, Roberto Amato, Sonia Goncalves, Ewan Harrison, David K. Jackson, Ian Johnston, Dominic Kwiatkowski, Cordelia Langford, John Sillitoe on behalf of the Wellcome Sanger Institute COVID-19 Surveillance Team
EPI_ISL_736304	Lighthouse Lab in Glasgow	Wellcome Sanger Institute for the COVID-19 Genomics UK (COG-UK) Consortium	Harper VanSteenhouse, Yumi Kasai, David Gray, Carol Clugston, Anna Dominiczak and Alex Alderton, Roberto Amato, Sonia Goncalves, Ewan Harrison, David K. Jackson, Ian Johnston, Dominic Kwiatkowski, Cordelia Langford, John Sillitoe on behalf of the Wellcome Sanger Institute COVID-19 Surveillance Team
EPI_ISL_736305, EPI_ISL_736308, EPI_ISL_736310	Lighthouse Lab in Milton Keynes	Wellcome Sanger Institute for the COVID-19 Genomics UK (COG-UK) Consortium	The Lighthouse Lab in Milton Keynes and Alex Alderton, Roberto Amato, Sonia Goncalves, Ewan Harrison, David K. Jackson, Ian Johnston, Dominic Kwiatkowski, Cordelia Langford, John Sillitoe on behalf of the Wellcome Sanger Institute COVID-19 Surveillance Team
EPI_ISL_736313, EPI_ISL_736316	Lighthouse Lab in Glasgow	Wellcome Sanger Institute for the COVID-19 Genomics UK (COG-UK) Consortium	Harper VanSteenhouse, Yumi Kasai, David Gray, Carol Clugston, Anna Dominiczak and Alex Alderton, Roberto Amato, Sonia Goncalves, Ewan Harrison, David K. Jackson, Ian Johnston, Dominic Kwiatkowski, Cordelia Langford, John Sillitoe on behalf of the Wellcome Sanger Institute COVID-19 Surveillance Team
EPI_ISL_736317, EPI_ISL_736319	Lighthouse Lab in Milton Keynes	Wellcome Sanger Institute for the COVID-19 Genomics UK (COG-UK) Consortium	The Lighthouse Lab in Milton Keynes and Alex Alderton, Roberto Amato, Sonia Goncalves, Ewan Harrison, David K. Jackson, Ian Johnston, Dominic Kwiatkowski, Cordelia Langford, John Sillitoe on behalf of the Wellcome Sanger Institute COVID-19 Surveillance Team
EPI_ISL_736320, EPI_ISL_736321, EPI_ISL_736323	Lighthouse Lab in Glasgow	Wellcome Sanger Institute for the COVID-19 Genomics UK (COG-UK) Consortium	Harper VanSteenhouse, Yumi Kasai, David Gray, Carol Clugston, Anna Dominiczak and Alex Alderton, Roberto Amato, Sonia Goncalves, Ewan Harrison, David K. Jackson, Ian Johnston, Dominic Kwiatkowski, Cordelia Langford, John Sillitoe on behalf of the Wellcome Sanger Institute COVID-19 Surveillance Team
EPI_ISL_736324	Lighthouse Lab in Milton Keynes	Wellcome Sanger Institute for the COVID-19 Genomics UK (COG-UK) Consortium	The Lighthouse Lab in Milton Keynes and Alex Alderton, Roberto Amato, Sonia Goncalves, Ewan Harrison, David K. Jackson, Ian Johnston, Dominic Kwiatkowski, Cordelia Langford, John Sillitoe on behalf of the Wellcome Sanger Institute COVID-19 Surveillance Team
EPI_ISL_736325	Lighthouse Lab in Glasgow	Wellcome Sanger Institute for the COVID-19 Genomics UK (COG-UK) Consortium	Harper VanSteenhouse, Yumi Kasai, David Gray, Carol Clugston, Anna Dominiczak and Alex Alderton, Roberto Amato, Sonia Goncalves, Ewan Harrison, David K. Jackson, Ian Johnston, Dominic Kwiatkowski, Cordelia Langford, John Sillitoe on behalf of the Wellcome Sanger Institute COVID-19 Surveillance Team
EPI_ISL_736326	Lighthouse Lab in Milton Keynes	Wellcome Sanger Institute for the COVID-19 Genomics UK (COG-UK) Consortium	The Lighthouse Lab in Milton Keynes and Alex Alderton, Roberto Amato, Sonia Goncalves, Ewan Harrison, David K. Jackson, Ian Johnston, Dominic Kwiatkowski, Cordelia Langford, John Sillitoe on behalf of the Wellcome Sanger Institute COVID-19 Surveillance Team
EPI_ISL_736327	Lighthouse Lab in Glasgow	Wellcome Sanger Institute for the COVID-19 Genomics UK (COG-UK) Consortium	Harper VanSteenhouse, Yumi Kasai, David Gray, Carol Clugston, Anna Dominiczak and Alex Alderton, Roberto Amato, Sonia Goncalves, Ewan Harrison, David K. Jackson, Ian Johnston, Dominic Kwiatkowski, Cordelia Langford, John Sillitoe on behalf of the Wellcome Sanger Institute COVID-19 Surveillance Team
EPI_ISL_736329	Lighthouse Lab in Milton Keynes	Wellcome Sanger Institute for the COVID-19 Genomics UK (COG-UK) Consortium	The Lighthouse Lab in Milton Keynes and Alex Alderton, Roberto Amato, Sonia Goncalves, Ewan Harrison, David K. Jackson, Ian Johnston, Dominic Kwiatkowski, Cordelia Langford, John Sillitoe on behalf of the Wellcome Sanger Institute COVID-19 Surveillance Team
EPI_ISL_736330, EPI_ISL_736331, EPI_ISL_736332, EPI_ISL_736334	Lighthouse Lab in Glasgow	Wellcome Sanger Institute for the COVID-19 Genomics UK (COG-UK) Consortium	Harper VanSteenhouse, Yumi Kasai, David Gray, Carol Clugston, Anna Dominiczak and Alex Alderton, Roberto Amato, Sonia Goncalves, Ewan Harrison, David K. Jackson, Ian Johnston, Dominic Kwiatkowski, Cordelia Langford, John Sillitoe on behalf of the Wellcome Sanger Institute COVID-19 Surveillance Team
EPI_ISL_736335, EPI_ISL_736336, EPI_ISL_736340, EPI_ISL_736341, EPI_ISL_736344, EPI_ISL_736348	Lighthouse Lab in Milton Keynes	Wellcome Sanger Institute for the COVID-19 Genomics UK (COG-UK) Consortium	The Lighthouse Lab in Milton Keynes and Alex Alderton, Roberto Amato, Sonia Goncalves, Ewan Harrison, David K. Jackson, Ian Johnston, Dominic Kwiatkowski, Cordelia Langford, John Sillitoe on behalf of the Wellcome Sanger Institute COVID-19 Surveillance Team
EPI_ISL_736352	Lighthouse Lab in Glasgow	Wellcome Sanger Institute for the COVID-19 Genomics UK (COG-UK) Consortium	Harper VanSteenhouse, Yumi Kasai, David Gray, Carol Clugston, Anna Dominiczak and Alex Alderton, Roberto Amato, Sonia Goncalves, Ewan Harrison, David K. Jackson, Ian Johnston, Dominic Kwiatkowski, Cordelia Langford, John Sillitoe on behalf of the Wellcome Sanger Institute COVID-19 Surveillance Team
EPI_ISL_736353, EPI_ISL_736354, EPI_ISL_736357, EPI_ISL_736360	Lighthouse Lab in Milton Keynes	Wellcome Sanger Institute for the COVID-19 Genomics UK (COG-UK) Consortium	The Lighthouse Lab in Milton Keynes and Alex Alderton, Roberto Amato, Sonia Goncalves, Ewan Harrison, David K. Jackson, Ian Johnston, Dominic Kwiatkowski, Cordelia Langford, John Sillitoe on behalf of the Wellcome Sanger Institute COVID-19 Surveillance Team
EPI_ISL_736362	Lighthouse Lab in Glasgow	Wellcome Sanger Institute for the COVID-19 Genomics UK (COG-UK) Consortium	Harper VanSteenhouse, Yumi Kasai, David Gray, Carol Clugston, Anna Dominiczak and Alex Alderton, Roberto Amato, Sonia Goncalves, Ewan Harrison, David K. Jackson, Ian Johnston, Dominic Kwiatkowski, Cordelia Langford, John Sillitoe on behalf of the Wellcome Sanger Institute COVID-19 Surveillance Team
EPI_ISL_736364, EPI_ISL_736365, EPI_ISL_736366, EPI_ISL_736368, EPI_ISL_736369	Lighthouse Lab in Milton Keynes	Wellcome Sanger Institute for the COVID-19 Genomics UK (COG-UK) Consortium	The Lighthouse Lab in Milton Keynes and Alex Alderton, Roberto Amato, Sonia Goncalves, Ewan Harrison, David K. Jackson, Ian Johnston, Dominic Kwiatkowski, Cordelia Langford, John Sillitoe on behalf of the Wellcome Sanger Institute COVID-19 Surveillance Team

EPI_ISL_736371	Lighthouse Lab in Glasgow	Wellcome Sanger Institute for the COVID-19 Genomics UK (COG-UK) Consortium	Harper VanSteenhouse, Yumi Kasai, David Gray, Carol Clugston, Anna Dominiczak and Alex Alderton, Roberto Amato, Sonia Goncalves, Ewan Harrison, David K. Jackson, Ian Johnston, Dominic Kwiatkowski, Cordelia Langford, John Sillitoe on behalf of the Wellcome Sanger Institute COVID-19 Surveillance Team
EPI_ISL_736374, EPI_ISL_736375	Lighthouse Lab in Milton Keynes	Wellcome Sanger Institute for the COVID-19 Genomics UK (COG-UK) Consortium	The Lighthouse Lab in Milton Keynes and Alex Alderton, Roberto Amato, Sonia Goncalves, Ewan Harrison, David K. Jackson, Ian Johnston, Dominic Kwiatkowski, Cordelia Langford, John Sillitoe on behalf of the Wellcome Sanger Institute COVID-19 Surveillance Team
EPI_ISL_736378	Lighthouse Lab in Glasgow	Wellcome Sanger Institute for the COVID-19 Genomics UK (COG-UK) Consortium	Harper VanSteenhouse, Yumi Kasai, David Gray, Carol Clugston, Anna Dominiczak and Alex Alderton, Roberto Amato, Sonia Goncalves, Ewan Harrison, David K. Jackson, Ian Johnston, Dominic Kwiatkowski, Cordelia Langford, John Sillitoe on behalf of the Wellcome Sanger Institute COVID-19 Surveillance Team
EPI_ISL_736379, EPI_ISL_736384, EPI_ISL_736385, EPI_ISL_736387	Lighthouse Lab in Milton Keynes	Wellcome Sanger Institute for the COVID-19 Genomics UK (COG-UK) Consortium	The Lighthouse Lab in Milton Keynes and Alex Alderton, Roberto Amato, Sonia Goncalves, Ewan Harrison, David K. Jackson, Ian Johnston, Dominic Kwiatkowski, Cordelia Langford, John Sillitoe on behalf of the Wellcome Sanger Institute COVID-19 Surveillance Team
EPI_ISL_736388, EPI_ISL_736391	Lighthouse Lab in Glasgow	Wellcome Sanger Institute for the COVID-19 Genomics UK (COG-UK) Consortium	Harper VanSteenhouse, Yumi Kasai, David Gray, Carol Clugston, Anna Dominiczak and Alex Alderton, Roberto Amato, Sonia Goncalves, Ewan Harrison, David K. Jackson, Ian Johnston, Dominic Kwiatkowski, Cordelia Langford, John Sillitoe on behalf of the Wellcome Sanger Institute COVID-19 Surveillance Team
EPI_ISL_736392	Lighthouse Lab in Milton Keynes	Wellcome Sanger Institute for the COVID-19 Genomics UK (COG-UK) Consortium	The Lighthouse Lab in Milton Keynes and Alex Alderton, Roberto Amato, Sonia Goncalves, Ewan Harrison, David K. Jackson, Ian Johnston, Dominic Kwiatkowski, Cordelia Langford, John Sillitoe on behalf of the Wellcome Sanger Institute COVID-19 Surveillance Team
EPI_ISL_736394	Lighthouse Lab in Glasgow	Wellcome Sanger Institute for the COVID-19 Genomics UK (COG-UK) Consortium	Harper VanSteenhouse, Yumi Kasai, David Gray, Carol Clugston, Anna Dominiczak and Alex Alderton, Roberto Amato, Sonia Goncalves, Ewan Harrison, David K. Jackson, Ian Johnston, Dominic Kwiatkowski, Cordelia Langford, John Sillitoe on behalf of the Wellcome Sanger Institute COVID-19 Surveillance Team
EPI_ISL_736395	Lighthouse Lab in Milton Keynes	Wellcome Sanger Institute for the COVID-19 Genomics UK (COG-UK) Consortium	The Lighthouse Lab in Milton Keynes and Alex Alderton, Roberto Amato, Sonia Goncalves, Ewan Harrison, David K. Jackson, Ian Johnston, Dominic Kwiatkowski, Cordelia Langford, John Sillitoe on behalf of the Wellcome Sanger Institute COVID-19 Surveillance Team
EPI_ISL_736398, EPI_ISL_736400	Lighthouse Lab in Glasgow	Wellcome Sanger Institute for the COVID-19 Genomics UK (COG-UK) Consortium	Harper VanSteenhouse, Yumi Kasai, David Gray, Carol Clugston, Anna Dominiczak and Alex Alderton, Roberto Amato, Sonia Goncalves, Ewan Harrison, David K. Jackson, Ian Johnston, Dominic Kwiatkowski, Cordelia Langford, John Sillitoe on behalf of the Wellcome Sanger Institute COVID-19 Surveillance Team
EPI_ISL_736401, EPI_ISL_736403	Lighthouse Lab in Milton Keynes	Wellcome Sanger Institute for the COVID-19 Genomics UK (COG-UK) Consortium	The Lighthouse Lab in Milton Keynes and Alex Alderton, Roberto Amato, Sonia Goncalves, Ewan Harrison, David K. Jackson, Ian Johnston, Dominic Kwiatkowski, Cordelia Langford, John Sillitoe on behalf of the Wellcome Sanger Institute COVID-19 Surveillance Team
EPI_ISL_736404	Lighthouse Lab in Glasgow	Wellcome Sanger Institute for the COVID-19 Genomics UK (COG-UK) Consortium	Harper VanSteenhouse, Yumi Kasai, David Gray, Carol Clugston, Anna Dominiczak and Alex Alderton, Roberto Amato, Sonia Goncalves, Ewan Harrison, David K. Jackson, Ian Johnston, Dominic Kwiatkowski, Cordelia Langford, John Sillitoe on behalf of the Wellcome Sanger Institute COVID-19 Surveillance Team
EPI_ISL_736405, EPI_ISL_736409, EPI_ISL_736410	Lighthouse Lab in Milton Keynes	Wellcome Sanger Institute for the COVID-19 Genomics UK (COG-UK) Consortium	The Lighthouse Lab in Milton Keynes and Alex Alderton, Roberto Amato, Sonia Goncalves, Ewan Harrison, David K. Jackson, Ian Johnston, Dominic Kwiatkowski, Cordelia Langford, John Sillitoe on behalf of the Wellcome Sanger Institute COVID-19 Surveillance Team
EPI_ISL_736411, EPI_ISL_736412	Lighthouse Lab in Glasgow	Wellcome Sanger Institute for the COVID-19 Genomics UK (COG-UK) Consortium	Harper VanSteenhouse, Yumi Kasai, David Gray, Carol Clugston, Anna Dominiczak and Alex Alderton, Roberto Amato, Sonia Goncalves, Ewan Harrison, David K. Jackson, Ian Johnston, Dominic Kwiatkowski, Cordelia Langford, John Sillitoe on behalf of the Wellcome Sanger Institute COVID-19 Surveillance Team
EPI_ISL_736413, EPI_ISL_736415, EPI_ISL_736418, EPI_ISL_736420, EPI_ISL_736421	Lighthouse Lab in Milton Keynes	Wellcome Sanger Institute for the COVID-19 Genomics UK (COG-UK) Consortium	The Lighthouse Lab in Milton Keynes and Alex Alderton, Roberto Amato, Sonia Goncalves, Ewan Harrison, David K. Jackson, Ian Johnston, Dominic Kwiatkowski, Cordelia Langford, John Sillitoe on behalf of the Wellcome Sanger Institute COVID-19 Surveillance Team
EPI_ISL_736423	Lighthouse Lab in Glasgow	Wellcome Sanger Institute for the COVID-19 Genomics UK (COG-UK) Consortium	Harper VanSteenhouse, Yumi Kasai, David Gray, Carol Clugston, Anna Dominiczak and Alex Alderton, Roberto Amato, Sonia Goncalves, Ewan Harrison, David K. Jackson, Ian Johnston, Dominic Kwiatkowski, Cordelia Langford, John Sillitoe on behalf of the Wellcome Sanger Institute COVID-19 Surveillance Team
EPI_ISL_736424	Lighthouse Lab in Milton Keynes	Wellcome Sanger Institute for the COVID-19 Genomics UK (COG-UK) Consortium	The Lighthouse Lab in Milton Keynes and Alex Alderton, Roberto Amato, Sonia Goncalves, Ewan Harrison, David K. Jackson, Ian Johnston, Dominic Kwiatkowski, Cordelia Langford, John Sillitoe on behalf of the Wellcome Sanger Institute COVID-19 Surveillance Team
EPI_ISL_736431	Lighthouse Lab in Glasgow	Wellcome Sanger Institute for the COVID-19 Genomics UK (COG-UK) Consortium	Harper VanSteenhouse, Yumi Kasai, David Gray, Carol Clugston, Anna Dominiczak and Alex Alderton, Roberto Amato, Sonia Goncalves, Ewan Harrison, David K. Jackson, Ian Johnston, Dominic Kwiatkowski, Cordelia Langford, John Sillitoe on behalf of the Wellcome Sanger Institute COVID-19 Surveillance Team
EPI_ISL_736432, EPI_ISL_736433, EPI_ISL_736434	Lighthouse Lab in Milton Keynes	Wellcome Sanger Institute for the COVID-19 Genomics UK (COG-UK) Consortium	The Lighthouse Lab in Milton Keynes and Alex Alderton, Roberto Amato, Sonia Goncalves, Ewan Harrison, David K. Jackson, Ian Johnston, Dominic Kwiatkowski, Cordelia Langford, John Sillitoe on behalf of the Wellcome Sanger Institute COVID-19 Surveillance Team
EPI_ISL_736437, EPI_ISL_736438	Lighthouse Lab in Glasgow	Wellcome Sanger Institute for the COVID-19 Genomics UK (COG-UK) Consortium	Harper VanSteenhouse, Yumi Kasai, David Gray, Carol Clugston, Anna Dominiczak and Alex Alderton, Roberto Amato, Sonia Goncalves, Ewan Harrison, David K. Jackson, Ian Johnston, Dominic Kwiatkowski, Cordelia Langford, John Sillitoe on behalf of the Wellcome Sanger Institute COVID-19 Surveillance Team
EPI_ISL_736439, EPI_ISL_736440	Lighthouse Lab in Milton Keynes	Wellcome Sanger Institute for the COVID-19 Genomics UK (COG-UK) Consortium	The Lighthouse Lab in Milton Keynes and Alex Alderton, Roberto Amato, Sonia Goncalves, Ewan Harrison, David K. Jackson, Ian Johnston, Dominic Kwiatkowski, Cordelia Langford, John Sillitoe on behalf of the Wellcome Sanger Institute COVID-19 Surveillance Team
EPI_ISL_736442, EPI_ISL_736444	Lighthouse Lab in Glasgow	Wellcome Sanger Institute for the COVID-19 Genomics UK (COG-UK) Consortium	Harper VanSteenhouse, Yumi Kasai, David Gray, Carol Clugston, Anna Dominiczak and Alex Alderton, Roberto Amato, Sonia Goncalves, Ewan Harrison, David K. Jackson, Ian Johnston, Dominic Kwiatkowski, Cordelia Langford, John Sillitoe on behalf of the Wellcome Sanger Institute COVID-19 Surveillance Team
EPI_ISL_736446, EPI_ISL_736448, EPI_ISL_736449, EPI_ISL_736450	Lighthouse Lab in Milton Keynes	Wellcome Sanger Institute for the COVID-19 Genomics UK (COG-UK) Consortium	The Lighthouse Lab in Milton Keynes and Alex Alderton, Roberto Amato, Sonia Goncalves, Ewan Harrison, David K. Jackson, Ian Johnston, Dominic Kwiatkowski, Cordelia Langford, John Sillitoe on behalf of the Wellcome Sanger Institute COVID-19 Surveillance Team
EPI_ISL_736451	Lighthouse Lab in Glasgow	Wellcome Sanger Institute for the COVID-19 Genomics UK (COG-UK) Consortium	Harper VanSteenhouse, Yumi Kasai, David Gray, Carol Clugston, Anna Dominiczak and Alex Alderton, Roberto Amato, Sonia Goncalves, Ewan Harrison, David K. Jackson, Ian Johnston, Dominic Kwiatkowski, Cordelia Langford, John Sillitoe on behalf of the Wellcome Sanger Institute COVID-19 Surveillance Team
EPI_ISL_736452, EPI_ISL_736453, EPI_ISL_736457	Lighthouse Lab in Milton Keynes	Wellcome Sanger Institute for the COVID-19 Genomics UK (COG-UK) Consortium	The Lighthouse Lab in Milton Keynes and Alex Alderton, Roberto Amato, Sonia Goncalves, Ewan Harrison, David K. Jackson, Ian Johnston, Dominic Kwiatkowski, Cordelia Langford, John Sillitoe on behalf of the Wellcome Sanger Institute COVID-19 Surveillance Team
EPI_ISL_736461	Lighthouse Lab in Glasgow	Wellcome Sanger Institute for the COVID-19 Genomics UK (COG-UK) Consortium	Harper VanSteenhouse, Yumi Kasai, David Gray, Carol Clugston, Anna Dominiczak and Alex Alderton, Roberto Amato, Sonia Goncalves, Ewan Harrison, David K. Jackson, Ian Johnston, Dominic Kwiatkowski, Cordelia Langford, John Sillitoe on behalf of the Wellcome Sanger Institute COVID-19 Surveillance Team
EPI_ISL_736462, EPI_ISL_736463	Lighthouse Lab in Milton Keynes	Wellcome Sanger Institute for the COVID-19 Genomics UK (COG-UK) Consortium	The Lighthouse Lab in Milton Keynes and Alex Alderton, Roberto Amato, Sonia Goncalves, Ewan Harrison, David K. Jackson, Ian Johnston, Dominic Kwiatkowski, Cordelia Langford, John Sillitoe on behalf of the Wellcome Sanger Institute COVID-19 Surveillance Team
EPI_ISL_736464	Lighthouse Lab in Glasgow	Wellcome Sanger Institute for the COVID-19 Genomics UK (COG-UK) Consortium	Harper VanSteenhouse, Yumi Kasai, David Gray, Carol Clugston, Anna Dominiczak and Alex Alderton, Roberto Amato, Sonia Goncalves, Ewan Harrison, David K. Jackson, Ian Johnston, Dominic Kwiatkowski, Cordelia Langford, John Sillitoe on behalf of the Wellcome Sanger Institute COVID-19 Surveillance Team
EPI_ISL_736465, EPI_ISL_736467	Lighthouse Lab in Milton Keynes	Wellcome Sanger Institute for the COVID-19 Genomics UK (COG-UK) Consortium	The Lighthouse Lab in Milton Keynes and Alex Alderton, Roberto Amato, Sonia Goncalves, Ewan Harrison, David K. Jackson, Ian Johnston, Dominic Kwiatkowski, Cordelia Langford, John Sillitoe on behalf of the Wellcome Sanger Institute COVID-19 Surveillance Team
EPI_ISL_736468	Lighthouse Lab in Glasgow	Wellcome Sanger Institute for the COVID-19 Genomics UK (COG-UK) Consortium	Harper VanSteenhouse, Yumi Kasai, David Gray, Carol Clugston, Anna Dominiczak and Alex Alderton, Roberto Amato, Sonia Goncalves, Ewan Harrison, David K. Jackson, Ian Johnston, Dominic Kwiatkowski, Cordelia Langford, John Sillitoe on behalf of the Wellcome Sanger Institute COVID-19 Surveillance

			Team
EPI_ISL_736472, EPI_ISL_736473	Lighthouse Lab in Milton Keynes	Wellcome Sanger Institute for the COVID-19 Genomics UK (COG-UK) Consortium	The Lighthouse Lab in Milton Keynes and Alex Alderton, Roberto Amato, Sonia Goncalves, Ewan Harrison, David K. Jackson, Ian Johnston, Dominic Kwiatkowski, Cordelia Langford, John Sillitoe on behalf of the Wellcome Sanger Institute COVID-19 Surveillance Team
EPI_ISL_736476, EPI_ISL_736478, EPI_ISL_736479	Lighthouse Lab in Glasgow	Wellcome Sanger Institute for the COVID-19 Genomics UK (COG-UK) Consortium	Harper VanSteenhouse, Yumi Kasai, David Gray, Carol Clugston, Anna Dominiczak and Alex Alderton, Roberto Amato, Sonia Goncalves, Ewan Harrison, David K. Jackson, Ian Johnston, Dominic Kwiatkowski, Cordelia Langford, John Sillitoe on behalf of the Wellcome Sanger Institute COVID-19 Surveillance Team
EPI_ISL_736480	Lighthouse Lab in Milton Keynes	Wellcome Sanger Institute for the COVID-19 Genomics UK (COG-UK) Consortium	The Lighthouse Lab in Milton Keynes and Alex Alderton, Roberto Amato, Sonia Goncalves, Ewan Harrison, David K. Jackson, Ian Johnston, Dominic Kwiatkowski, Cordelia Langford, John Sillitoe on behalf of the Wellcome Sanger Institute COVID-19 Surveillance Team
EPI_ISL_736482	Lighthouse Lab in Alderley Park	Wellcome Sanger Institute for the COVID-19 Genomics UK (COG-UK) Consortium	Jacquelyn Wynn, Mairead Hyland, The Lighthouse Lab in Alderley Park and Alex Alderton, Roberto Amato, Sonia Goncalves, Ewan Harrison, David K. Jackson, Ian Johnston, Dominic Kwiatkowski, Cordelia Langford, John Sillitoe on behalf of the Wellcome Sanger Institute COVID-19 Surveillance Team
EPI_ISL_736488	Lighthouse Lab in Glasgow	Wellcome Sanger Institute for the COVID-19 Genomics UK (COG-UK) Consortium	Harper VanSteenhouse, Yumi Kasai, David Gray, Carol Clugston, Anna Dominiczak and Alex Alderton, Roberto Amato, Sonia Goncalves, Ewan Harrison, David K. Jackson, Ian Johnston, Dominic Kwiatkowski, Cordelia Langford, John Sillitoe on behalf of the Wellcome Sanger Institute COVID-19 Surveillance Team
EPI_ISL_736494, EPI_ISL_736495, EPI_ISL_736498, EPI_ISL_736500	Lighthouse Lab in Alderley Park	Wellcome Sanger Institute for the COVID-19 Genomics UK (COG-UK) Consortium	Jacquelyn Wynn, Mairead Hyland, The Lighthouse Lab in Alderley Park and Alex Alderton, Roberto Amato, Sonia Goncalves, Ewan Harrison, David K. Jackson, Ian Johnston, Dominic Kwiatkowski, Cordelia Langford, John Sillitoe on behalf of the Wellcome Sanger Institute COVID-19 Surveillance Team
EPI_ISL_736503	Lighthouse Lab in Glasgow	Wellcome Sanger Institute for the COVID-19 Genomics UK (COG-UK) Consortium	Harper VanSteenhouse, Yumi Kasai, David Gray, Carol Clugston, Anna Dominiczak and Alex Alderton, Roberto Amato, Sonia Goncalves, Ewan Harrison, David K. Jackson, Ian Johnston, Dominic Kwiatkowski, Cordelia Langford, John Sillitoe on behalf of the Wellcome Sanger Institute COVID-19 Surveillance Team
EPI_ISL_736504, EPI_ISL_736508	Lighthouse Lab in Milton Keynes	Wellcome Sanger Institute for the COVID-19 Genomics UK (COG-UK) Consortium	The Lighthouse Lab in Milton Keynes and Alex Alderton, Roberto Amato, Sonia Goncalves, Ewan Harrison, David K. Jackson, Ian Johnston, Dominic Kwiatkowski, Cordelia Langford, John Sillitoe on behalf of the Wellcome Sanger Institute COVID-19 Surveillance Team
EPI_ISL_736511	Lighthouse Lab in Alderley Park	Wellcome Sanger Institute for the COVID-19 Genomics UK (COG-UK) Consortium	Jacquelyn Wynn, Mairead Hyland, The Lighthouse Lab in Alderley Park and Alex Alderton, Roberto Amato, Sonia Goncalves, Ewan Harrison, David K. Jackson, Ian Johnston, Dominic Kwiatkowski, Cordelia Langford, John Sillitoe on behalf of the Wellcome Sanger Institute COVID-19 Surveillance Team
EPI_ISL_736514, EPI_ISL_736515	Lighthouse Lab in Milton Keynes	Wellcome Sanger Institute for the COVID-19 Genomics UK (COG-UK) Consortium	The Lighthouse Lab in Milton Keynes and Alex Alderton, Roberto Amato, Sonia Goncalves, Ewan Harrison, David K. Jackson, Ian Johnston, Dominic Kwiatkowski, Cordelia Langford, John Sillitoe on behalf of the Wellcome Sanger Institute COVID-19 Surveillance Team
EPI_ISL_736516, EPI_ISL_736518, EPI_ISL_736521	Lighthouse Lab in Alderley Park	Wellcome Sanger Institute for the COVID-19 Genomics UK (COG-UK) Consortium	Jacquelyn Wynn, Mairead Hyland, The Lighthouse Lab in Alderley Park and Alex Alderton, Roberto Amato, Sonia Goncalves, Ewan Harrison, David K. Jackson, Ian Johnston, Dominic Kwiatkowski, Cordelia Langford, John Sillitoe on behalf of the Wellcome Sanger Institute COVID-19 Surveillance Team
EPI_ISL_736525, EPI_ISL_736527	Lighthouse Lab in Milton Keynes	Wellcome Sanger Institute for the COVID-19 Genomics UK (COG-UK) Consortium	The Lighthouse Lab in Milton Keynes and Alex Alderton, Roberto Amato, Sonia Goncalves, Ewan Harrison, David K. Jackson, Ian Johnston, Dominic Kwiatkowski, Cordelia Langford, John Sillitoe on behalf of the Wellcome Sanger Institute COVID-19 Surveillance Team
EPI_ISL_736533	Lighthouse Lab in Glasgow	Wellcome Sanger Institute for the COVID-19 Genomics UK (COG-UK) Consortium	Harper VanSteenhouse, Yumi Kasai, David Gray, Carol Clugston, Anna Dominiczak and Alex Alderton, Roberto Amato, Sonia Goncalves, Ewan Harrison, David K. Jackson, Ian Johnston, Dominic Kwiatkowski, Cordelia Langford, John Sillitoe on behalf of the Wellcome Sanger Institute COVID-19 Surveillance Team
EPI_ISL_736535, EPI_ISL_736537, EPI_ISL_736539, EPI_ISL_736540, EPI_ISL_736541	Lighthouse Lab in Alderley Park	Wellcome Sanger Institute for the COVID-19 Genomics UK (COG-UK) Consortium	Jacquelyn Wynn, Mairead Hyland, The Lighthouse Lab in Alderley Park and Alex Alderton, Roberto Amato, Sonia Goncalves, Ewan Harrison, David K. Jackson, Ian Johnston, Dominic Kwiatkowski, Cordelia Langford, John Sillitoe on behalf of the Wellcome Sanger Institute COVID-19 Surveillance Team
EPI_ISL_736542	Lighthouse Lab in Milton Keynes	Wellcome Sanger Institute for the COVID-19 Genomics UK (COG-UK) Consortium	The Lighthouse Lab in Milton Keynes and Alex Alderton, Roberto Amato, Sonia Goncalves, Ewan Harrison, David K. Jackson, Ian Johnston, Dominic Kwiatkowski, Cordelia Langford, John Sillitoe on behalf of the Wellcome Sanger Institute COVID-19 Surveillance Team
EPI_ISL_736545, EPI_ISL_736548	Lighthouse Lab in Alderley Park	Wellcome Sanger Institute for the COVID-19 Genomics UK (COG-UK) Consortium	Jacquelyn Wynn, Mairead Hyland, The Lighthouse Lab in Alderley Park and Alex Alderton, Roberto Amato, Sonia Goncalves, Ewan Harrison, David K. Jackson, Ian Johnston, Dominic Kwiatkowski, Cordelia Langford, John Sillitoe on behalf of the Wellcome Sanger Institute COVID-19 Surveillance Team
EPI_ISL_736550	Lighthouse Lab in Milton Keynes	Wellcome Sanger Institute for the COVID-19 Genomics UK (COG-UK) Consortium	The Lighthouse Lab in Milton Keynes and Alex Alderton, Roberto Amato, Sonia Goncalves, Ewan Harrison, David K. Jackson, Ian Johnston, Dominic Kwiatkowski, Cordelia Langford, John Sillitoe on behalf of the Wellcome Sanger Institute COVID-19 Surveillance Team
EPI_ISL_736553, EPI_ISL_736554, EPI_ISL_736555	Lighthouse Lab in Alderley Park	Wellcome Sanger Institute for the COVID-19 Genomics UK (COG-UK) Consortium	Jacquelyn Wynn, Mairead Hyland, The Lighthouse Lab in Alderley Park and Alex Alderton, Roberto Amato, Sonia Goncalves, Ewan Harrison, David K. Jackson, Ian Johnston, Dominic Kwiatkowski, Cordelia Langford, John Sillitoe on behalf of the Wellcome Sanger Institute COVID-19 Surveillance Team
EPI_ISL_736563, EPI_ISL_736565	Lighthouse Lab in Milton Keynes	Wellcome Sanger Institute for the COVID-19 Genomics UK (COG-UK) Consortium	The Lighthouse Lab in Milton Keynes and Alex Alderton, Roberto Amato, Sonia Goncalves, Ewan Harrison, David K. Jackson, Ian Johnston, Dominic Kwiatkowski, Cordelia Langford, John Sillitoe on behalf of the Wellcome Sanger Institute COVID-19 Surveillance Team
EPI_ISL_736567	Lighthouse Lab in Glasgow	Wellcome Sanger Institute for the COVID-19 Genomics UK (COG-UK) Consortium	Harper VanSteenhouse, Yumi Kasai, David Gray, Carol Clugston, Anna Dominiczak and Alex Alderton, Roberto Amato, Sonia Goncalves, Ewan Harrison, David K. Jackson, Ian Johnston, Dominic Kwiatkowski, Cordelia Langford, John Sillitoe on behalf of the Wellcome Sanger Institute COVID-19 Surveillance Team
EPI_ISL_736569, EPI_ISL_736572, EPI_ISL_736577, EPI_ISL_736581	Lighthouse Lab in Alderley Park	Wellcome Sanger Institute for the COVID-19 Genomics UK (COG-UK) Consortium	Jacquelyn Wynn, Mairead Hyland, The Lighthouse Lab in Alderley Park and Alex Alderton, Roberto Amato, Sonia Goncalves, Ewan Harrison, David K. Jackson, Ian Johnston, Dominic Kwiatkowski, Cordelia Langford, John Sillitoe on behalf of the Wellcome Sanger Institute COVID-19 Surveillance Team
EPI_ISL_736582	Lighthouse Lab in Glasgow	Wellcome Sanger Institute for the COVID-19 Genomics UK (COG-UK) Consortium	Harper VanSteenhouse, Yumi Kasai, David Gray, Carol Clugston, Anna Dominiczak and Alex Alderton, Roberto Amato, Sonia Goncalves, Ewan Harrison, David K. Jackson, Ian Johnston, Dominic Kwiatkowski, Cordelia Langford, John Sillitoe on behalf of the Wellcome Sanger Institute COVID-19 Surveillance Team
EPI_ISL_736587	Lighthouse Lab in Alderley Park	Wellcome Sanger Institute for the COVID-19 Genomics UK (COG-UK) Consortium	Jacquelyn Wynn, Mairead Hyland, The Lighthouse Lab in Alderley Park and Alex Alderton, Roberto Amato, Sonia Goncalves, Ewan Harrison, David K. Jackson, Ian Johnston, Dominic Kwiatkowski, Cordelia Langford, John Sillitoe on behalf of the Wellcome Sanger Institute COVID-19 Surveillance Team
EPI_ISL_736588	Lighthouse Lab in Milton Keynes	Wellcome Sanger Institute for the COVID-19 Genomics UK (COG-UK) Consortium	The Lighthouse Lab in Milton Keynes and Alex Alderton, Roberto Amato, Sonia Goncalves, Ewan Harrison, David K. Jackson, Ian Johnston, Dominic Kwiatkowski, Cordelia Langford, John Sillitoe on behalf of the Wellcome Sanger Institute COVID-19 Surveillance Team
EPI_ISL_736589	Lighthouse Lab in Alderley Park	Wellcome Sanger Institute for the COVID-19 Genomics UK (COG-UK) Consortium	Jacquelyn Wynn, Mairead Hyland, The Lighthouse Lab in Alderley Park and Alex Alderton, Roberto Amato, Sonia Goncalves, Ewan Harrison, David K. Jackson, Ian Johnston, Dominic Kwiatkowski, Cordelia Langford, John Sillitoe on behalf of the Wellcome Sanger Institute COVID-19 Surveillance Team
EPI_ISL_736590, EPI_ISL_736593	Lighthouse Lab in Milton Keynes	Wellcome Sanger Institute for the COVID-19 Genomics UK (COG-UK) Consortium	The Lighthouse Lab in Milton Keynes and Alex Alderton, Roberto Amato, Sonia Goncalves, Ewan Harrison, David K. Jackson, Ian Johnston, Dominic Kwiatkowski, Cordelia Langford, John Sillitoe on behalf of the Wellcome Sanger Institute COVID-19 Surveillance Team
EPI_ISL_736599	Lighthouse Lab in Alderley Park	Wellcome Sanger Institute for the COVID-19 Genomics UK (COG-UK) Consortium	Jacquelyn Wynn, Mairead Hyland, The Lighthouse Lab in Alderley Park and Alex Alderton, Roberto Amato, Sonia Goncalves, Ewan Harrison, David K. Jackson, Ian Johnston, Dominic Kwiatkowski, Cordelia Langford, John Sillitoe on behalf of the Wellcome Sanger Institute COVID-19 Surveillance Team
EPI_ISL_736608, EPI_ISL_736610	Lighthouse Lab in Milton Keynes	Wellcome Sanger Institute for the COVID-19 Genomics UK (COG-UK) Consortium	The Lighthouse Lab in Milton Keynes and Alex Alderton, Roberto Amato, Sonia Goncalves, Ewan Harrison, David K. Jackson, Ian Johnston, Dominic Kwiatkowski, Cordelia Langford, John Sillitoe on behalf of the Wellcome Sanger Institute COVID-19 Surveillance Team
EPI_ISL_736612, EPI_ISL_736614, EPI_ISL_736615	Lighthouse Lab in Alderley Park	Wellcome Sanger Institute for the COVID-19 Genomics UK (COG-UK) Consortium	Jacquelyn Wynn, Mairead Hyland, The Lighthouse Lab in Alderley Park and Alex Alderton, Roberto Amato, Sonia Goncalves, Ewan Harrison, David K. Jackson, Ian Johnston, Dominic Kwiatkowski, Cordelia Langford, John Sillitoe on behalf of the Wellcome Sanger Institute COVID-19 Surveillance Team
EPI_ISL_736618	Lighthouse Lab in Milton Keynes	Wellcome Sanger Institute for the COVID-19 Genomics UK (COG-UK) Consortium	The Lighthouse Lab in Milton Keynes and Alex Alderton, Roberto Amato, Sonia Goncalves, Ewan Harrison, David K. Jackson, Ian Johnston, Dominic Kwiatkowski, Cordelia Langford, John Sillitoe on behalf of the Wellcome Sanger Institute COVID-19 Surveillance Team
EPI_ISL_736623	Lighthouse Lab in Alderley Park	Wellcome Sanger Institute for the COVID-19 Genomics UK (COG-UK) Consortium	Jacquelyn Wynn, Mairead Hyland, The Lighthouse Lab in Alderley Park and Alex Alderton, Roberto Amato, Sonia Goncalves, Ewan Harrison, David K. Jackson, Ian Johnston, Dominic Kwiatkowski, Cordelia Langford, John Sillitoe on behalf of the Wellcome Sanger Institute COVID-19 Surveillance Team
EPI_ISL_736624	Lighthouse Lab in Glasgow	Wellcome Sanger Institute for the COVID-19 Genomics UK (COG-UK) Consortium	Harper VanSteenhouse, Yumi Kasai, David Gray, Carol Clugston, Anna Dominiczak and Alex Alderton, Roberto Amato, Sonia Goncalves, Ewan Harrison, David K. Jackson, Ian Johnston, Dominic Kwiatkowski, Cordelia Langford, John Sillitoe on behalf of the Wellcome Sanger Institute COVID-19 Surveillance

			Team
EPI_ISL_736625, EPI_ISL_736626	Lighthouse Lab in Alderley Park	Wellcome Sanger Institute for the COVID-19 Genomics UK (COG-UK) Consortium	Jacquelyn Wynn, Mairead Hyland, The Lighthouse Lab in Alderley Park and Alex Alderton, Roberto Amato, Sonia Goncalves, Ewan Harrison, David K. Jackson, Ian Johnston, Dominic Kwiatkowski, Cordelia Langford, John Sillitoe on behalf of the Wellcome Sanger Institute COVID-19 Surveillance Team
EPI_ISL_736627	Lighthouse Lab in Glasgow	Wellcome Sanger Institute for the COVID-19 Genomics UK (COG-UK) Consortium	Harper VanSteenhouse, Yumi Kasai, David Gray, Carol Clugston, Anna Dominiczak and Alex Alderton, Roberto Amato, Sonia Goncalves, Ewan Harrison, David K. Jackson, Ian Johnston, Dominic Kwiatkowski, Cordelia Langford, John Sillitoe on behalf of the Wellcome Sanger Institute COVID-19 Surveillance Team
EPI_ISL_736629	Lighthouse Lab in Alderley Park	Wellcome Sanger Institute for the COVID-19 Genomics UK (COG-UK) Consortium	Jacquelyn Wynn, Mairead Hyland, The Lighthouse Lab in Alderley Park and Alex Alderton, Roberto Amato, Sonia Goncalves, Ewan Harrison, David K. Jackson, Ian Johnston, Dominic Kwiatkowski, Cordelia Langford, John Sillitoe on behalf of the Wellcome Sanger Institute COVID-19 Surveillance Team
EPI_ISL_736630	Lighthouse Lab in Milton Keynes	Wellcome Sanger Institute for the COVID-19 Genomics UK (COG-UK) Consortium	The Lighthouse Lab in Milton Keynes and Alex Alderton, Roberto Amato, Sonia Goncalves, Ewan Harrison, David K. Jackson, Ian Johnston, Dominic Kwiatkowski, Cordelia Langford, John Sillitoe on behalf of the Wellcome Sanger Institute COVID-19 Surveillance Team
EPI_ISL_736631	Lighthouse Lab in Glasgow	Wellcome Sanger Institute for the COVID-19 Genomics UK (COG-UK) Consortium	Harper VanSteenhouse, Yumi Kasai, David Gray, Carol Clugston, Anna Dominiczak and Alex Alderton, Roberto Amato, Sonia Goncalves, Ewan Harrison, David K. Jackson, Ian Johnston, Dominic Kwiatkowski, Cordelia Langford, John Sillitoe on behalf of the Wellcome Sanger Institute COVID-19 Surveillance Team
EPI_ISL_736635, EPI_ISL_736636	Lighthouse Lab in Alderley Park	Wellcome Sanger Institute for the COVID-19 Genomics UK (COG-UK) Consortium	Jacquelyn Wynn, Mairead Hyland, The Lighthouse Lab in Alderley Park and Alex Alderton, Roberto Amato, Sonia Goncalves, Ewan Harrison, David K. Jackson, Ian Johnston, Dominic Kwiatkowski, Cordelia Langford, John Sillitoe on behalf of the Wellcome Sanger Institute COVID-19 Surveillance Team
EPI_ISL_736637	Lighthouse Lab in Milton Keynes	Wellcome Sanger Institute for the COVID-19 Genomics UK (COG-UK) Consortium	The Lighthouse Lab in Milton Keynes and Alex Alderton, Roberto Amato, Sonia Goncalves, Ewan Harrison, David K. Jackson, Ian Johnston, Dominic Kwiatkowski, Cordelia Langford, John Sillitoe on behalf of the Wellcome Sanger Institute COVID-19 Surveillance Team
EPI_ISL_736639, EPI_ISL_736642, EPI_ISL_736644, EPI_ISL_736645, EPI_ISL_736647	Lighthouse Lab in Alderley Park	Wellcome Sanger Institute for the COVID-19 Genomics UK (COG-UK) Consortium	Jacquelyn Wynn, Mairead Hyland, The Lighthouse Lab in Alderley Park and Alex Alderton, Roberto Amato, Sonia Goncalves, Ewan Harrison, David K. Jackson, Ian Johnston, Dominic Kwiatkowski, Cordelia Langford, John Sillitoe on behalf of the Wellcome Sanger Institute COVID-19 Surveillance Team
EPI_ISL_736648	Lighthouse Lab in Milton Keynes	Wellcome Sanger Institute for the COVID-19 Genomics UK (COG-UK) Consortium	The Lighthouse Lab in Milton Keynes and Alex Alderton, Roberto Amato, Sonia Goncalves, Ewan Harrison, David K. Jackson, Ian Johnston, Dominic Kwiatkowski, Cordelia Langford, John Sillitoe on behalf of the Wellcome Sanger Institute COVID-19 Surveillance Team
EPI_ISL_736649	Lighthouse Lab in Alderley Park	Wellcome Sanger Institute for the COVID-19 Genomics UK (COG-UK) Consortium	Jacquelyn Wynn, Mairead Hyland, The Lighthouse Lab in Alderley Park and Alex Alderton, Roberto Amato, Sonia Goncalves, Ewan Harrison, David K. Jackson, Ian Johnston, Dominic Kwiatkowski, Cordelia Langford, John Sillitoe on behalf of the Wellcome Sanger Institute COVID-19 Surveillance Team
EPI_ISL_736650	Lighthouse Lab in Glasgow	Wellcome Sanger Institute for the COVID-19 Genomics UK (COG-UK) Consortium	Harper VanSteenhouse, Yumi Kasai, David Gray, Carol Clugston, Anna Dominiczak and Alex Alderton, Roberto Amato, Sonia Goncalves, Ewan Harrison, David K. Jackson, Ian Johnston, Dominic Kwiatkowski, Cordelia Langford, John Sillitoe on behalf of the Wellcome Sanger Institute COVID-19 Surveillance Team
EPI_ISL_736652, EPI_ISL_736656	Lighthouse Lab in Alderley Park	Wellcome Sanger Institute for the COVID-19 Genomics UK (COG-UK) Consortium	Jacquelyn Wynn, Mairead Hyland, The Lighthouse Lab in Alderley Park and Alex Alderton, Roberto Amato, Sonia Goncalves, Ewan Harrison, David K. Jackson, Ian Johnston, Dominic Kwiatkowski, Cordelia Langford, John Sillitoe on behalf of the Wellcome Sanger Institute COVID-19 Surveillance Team
EPI_ISL_736657	Lighthouse Lab in Glasgow	Wellcome Sanger Institute for the COVID-19 Genomics UK (COG-UK) Consortium	Harper VanSteenhouse, Yumi Kasai, David Gray, Carol Clugston, Anna Dominiczak and Alex Alderton, Roberto Amato, Sonia Goncalves, Ewan Harrison, David K. Jackson, Ian Johnston, Dominic Kwiatkowski, Cordelia Langford, John Sillitoe on behalf of the Wellcome Sanger Institute COVID-19 Surveillance Team
EPI_ISL_736660, EPI_ISL_736661, EPI_ISL_736662, EPI_ISL_736663, EPI_ISL_736664, EPI_ISL_736665, EPI_ISL_736666, EPI_ISL_736669, EPI_ISL_736670, EPI_ISL_736676, EPI_ISL_736680, EPI_ISL_736684, EPI_ISL_736688, EPI_ISL_736690			
see above	Lighthouse Lab in Alderley Park	Wellcome Sanger Institute for the COVID-19 Genomics UK (COG-UK) Consortium	Jacquelyn Wynn, Mairead Hyland, The Lighthouse Lab in Alderley Park and Alex Alderton, Roberto Amato, Sonia Goncalves, Ewan Harrison, David K. Jackson, Ian Johnston, Dominic Kwiatkowski, Cordelia Langford, John Sillitoe on behalf of the Wellcome Sanger Institute COVID-19 Surveillance Team
EPI_ISL_736695	Lighthouse Lab in Glasgow	Wellcome Sanger Institute for the COVID-19 Genomics UK (COG-UK) Consortium	Harper VanSteenhouse, Yumi Kasai, David Gray, Carol Clugston, Anna Dominiczak and Alex Alderton, Roberto Amato, Sonia Goncalves, Ewan Harrison, David K. Jackson, Ian Johnston, Dominic Kwiatkowski, Cordelia Langford, John Sillitoe on behalf of the Wellcome Sanger Institute COVID-19 Surveillance Team
EPI_ISL_736696	Lighthouse Lab in Milton Keynes	Wellcome Sanger Institute for the COVID-19 Genomics UK (COG-UK) Consortium	The Lighthouse Lab in Milton Keynes and Alex Alderton, Roberto Amato, Sonia Goncalves, Ewan Harrison, David K. Jackson, Ian Johnston, Dominic Kwiatkowski, Cordelia Langford, John Sillitoe on behalf of the Wellcome Sanger Institute COVID-19 Surveillance Team
EPI_ISL_736698, EPI_ISL_736701, EPI_ISL_736704, EPI_ISL_736707, EPI_ISL_736709, EPI_ISL_736710	Lighthouse Lab in Alderley Park	Wellcome Sanger Institute for the COVID-19 Genomics UK (COG-UK) Consortium	Jacquelyn Wynn, Mairead Hyland, The Lighthouse Lab in Alderley Park and Alex Alderton, Roberto Amato, Sonia Goncalves, Ewan Harrison, David K. Jackson, Ian Johnston, Dominic Kwiatkowski, Cordelia Langford, John Sillitoe on behalf of the Wellcome Sanger Institute COVID-19 Surveillance Team
EPI_ISL_736716	Lighthouse Lab in Milton Keynes	Wellcome Sanger Institute for the COVID-19 Genomics UK (COG-UK) Consortium	The Lighthouse Lab in Milton Keynes and Alex Alderton, Roberto Amato, Sonia Goncalves, Ewan Harrison, David K. Jackson, Ian Johnston, Dominic Kwiatkowski, Cordelia Langford, John Sillitoe on behalf of the Wellcome Sanger Institute COVID-19 Surveillance Team
EPI_ISL_736717	Lighthouse Lab in Alderley Park	Wellcome Sanger Institute for the COVID-19 Genomics UK (COG-UK) Consortium	Jacquelyn Wynn, Mairead Hyland, The Lighthouse Lab in Alderley Park and Alex Alderton, Roberto Amato, Sonia Goncalves, Ewan Harrison, David K. Jackson, Ian Johnston, Dominic Kwiatkowski, Cordelia Langford, John Sillitoe on behalf of the Wellcome Sanger Institute COVID-19 Surveillance Team
EPI_ISL_736723, EPI_ISL_736724	Lighthouse Lab in Milton Keynes	Wellcome Sanger Institute for the COVID-19 Genomics UK (COG-UK) Consortium	The Lighthouse Lab in Milton Keynes and Alex Alderton, Roberto Amato, Sonia Goncalves, Ewan Harrison, David K. Jackson, Ian Johnston, Dominic Kwiatkowski, Cordelia Langford, John Sillitoe on behalf of the Wellcome Sanger Institute COVID-19 Surveillance Team
EPI_ISL_736726	Lighthouse Lab in Alderley Park	Wellcome Sanger Institute for the COVID-19 Genomics UK (COG-UK) Consortium	Jacquelyn Wynn, Mairead Hyland, The Lighthouse Lab in Alderley Park and Alex Alderton, Roberto Amato, Sonia Goncalves, Ewan Harrison, David K. Jackson, Ian Johnston, Dominic Kwiatkowski, Cordelia Langford, John Sillitoe on behalf of the Wellcome Sanger Institute COVID-19 Surveillance Team
EPI_ISL_736728	Lighthouse Lab in Milton Keynes	Wellcome Sanger Institute for the COVID-19 Genomics UK (COG-UK) Consortium	The Lighthouse Lab in Milton Keynes and Alex Alderton, Roberto Amato, Sonia Goncalves, Ewan Harrison, David K. Jackson, Ian Johnston, Dominic Kwiatkowski, Cordelia Langford, John Sillitoe on behalf of the Wellcome Sanger Institute COVID-19 Surveillance Team
EPI_ISL_736729, EPI_ISL_736730	Lighthouse Lab in Alderley Park	Wellcome Sanger Institute for the COVID-19 Genomics UK (COG-UK) Consortium	Jacquelyn Wynn, Mairead Hyland, The Lighthouse Lab in Alderley Park and Alex Alderton, Roberto Amato, Sonia Goncalves, Ewan Harrison, David K. Jackson, Ian Johnston, Dominic Kwiatkowski, Cordelia Langford, John Sillitoe on behalf of the Wellcome Sanger Institute COVID-19 Surveillance Team
EPI_ISL_736731, EPI_ISL_736733	Lighthouse Lab in Milton Keynes	Wellcome Sanger Institute for the COVID-19 Genomics UK (COG-UK) Consortium	The Lighthouse Lab in Milton Keynes and Alex Alderton, Roberto Amato, Sonia Goncalves, Ewan Harrison, David K. Jackson, Ian Johnston, Dominic Kwiatkowski, Cordelia Langford, John Sillitoe on behalf of the Wellcome Sanger Institute COVID-19 Surveillance Team
EPI_ISL_736739	Lighthouse Lab in Alderley Park	Wellcome Sanger Institute for the COVID-19 Genomics UK (COG-UK) Consortium	Jacquelyn Wynn, Mairead Hyland, The Lighthouse Lab in Alderley Park and Alex Alderton, Roberto Amato, Sonia Goncalves, Ewan Harrison, David K. Jackson, Ian Johnston, Dominic Kwiatkowski, Cordelia Langford, John Sillitoe on behalf of the Wellcome Sanger Institute COVID-19 Surveillance Team
EPI_ISL_736740, EPI_ISL_736744	Lighthouse Lab in Milton Keynes	Wellcome Sanger Institute for the COVID-19 Genomics UK (COG-UK) Consortium	The Lighthouse Lab in Milton Keynes and Alex Alderton, Roberto Amato, Sonia Goncalves, Ewan Harrison, David K. Jackson, Ian Johnston, Dominic Kwiatkowski, Cordelia Langford, John Sillitoe on behalf of the Wellcome Sanger Institute COVID-19 Surveillance Team
EPI_ISL_736745	Lighthouse Lab in Glasgow	Wellcome Sanger Institute for the COVID-19 Genomics UK (COG-UK) Consortium	Harper VanSteenhouse, Yumi Kasai, David Gray, Carol Clugston, Anna Dominiczak and Alex Alderton, Roberto Amato, Sonia Goncalves, Ewan Harrison, David K. Jackson, Ian Johnston, Dominic Kwiatkowski, Cordelia Langford, John Sillitoe on behalf of the Wellcome Sanger Institute COVID-19 Surveillance Team
EPI_ISL_736748	Lighthouse Lab in Alderley Park	Wellcome Sanger Institute for the COVID-19 Genomics UK (COG-UK) Consortium	Jacquelyn Wynn, Mairead Hyland, The Lighthouse Lab in Alderley Park and Alex Alderton, Roberto Amato, Sonia Goncalves, Ewan Harrison, David K. Jackson, Ian Johnston, Dominic Kwiatkowski, Cordelia Langford, John Sillitoe on behalf of the Wellcome Sanger Institute COVID-19 Surveillance Team
EPI_ISL_736749	Lighthouse Lab in Milton Keynes	Wellcome Sanger Institute for the COVID-19 Genomics UK (COG-UK) Consortium	The Lighthouse Lab in Milton Keynes and Alex Alderton, Roberto Amato, Sonia Goncalves, Ewan Harrison, David K. Jackson, Ian Johnston, Dominic Kwiatkowski, Cordelia Langford, John Sillitoe on behalf of the Wellcome Sanger Institute COVID-19 Surveillance Team
EPI_ISL_736750, EPI_ISL_736760, EPI_ISL_736762, EPI_ISL_736765,	Lighthouse Lab in Alderley Park	Wellcome Sanger Institute for the COVID-19 Genomics UK (COG-UK) Consortium	Jacquelyn Wynn, Mairead Hyland, The Lighthouse Lab in Alderley Park and Alex Alderton, Roberto Amato, Sonia Goncalves, Ewan Harrison, David K. Jackson, Ian Johnston, Dominic Kwiatkowski, Cordelia Langford, John Sillitoe on behalf of the Wellcome Sanger Institute COVID-19 Surveillance Team

EPI_ISL_736766, EPI_ISL_736768, EPI_ISL_736770, EPI_ISL_736771, EPI_ISL_736772			
EPI_ISL_736774	Lighthouse Lab in Glasgow	Wellcome Sanger Institute for the COVID-19 Genomics UK (COG-UK) Consortium	Harper VanSteenhouse, Yumi Kasai, David Gray, Carol Clugston, Anna Dominiczak and Alex Alderton, Roberto Amato, Sonia Goncalves, Ewan Harrison, David K. Jackson, Ian Johnston, Dominic Kwiatkowski, Cordelia Langford, John Sillitoe on behalf of the Wellcome Sanger Institute COVID-19 Surveillance Team
EPI_ISL_736996	Istituto Zooprofilattico Sperimentale del Mezzogiorno	National Institute for Infectious Diseases, INMI, "L. Spallanzani" IRCCS	E. Giombini, C.E.M Gruber, F. Messina, M. Rueca, B. Bartolini, F. Carletti, P. Cerino, B. Pierri, C. Buonerba, D. Di Concilio, M.C. Cuomo, A. Di Caro, M.R Capobianchi
EPI_ISL_736997	Istituto Zooprofilattico Sperimentale del Mezzogiorno	National Institute for Infectious Diseases, INMI, "L. Spallanzani" IRCCS	F. Messina, C.E.M Gruber, M. Rueca, B. Bartolini, E. Giombini, F. Carletti, P. Cerino, B. Pierri, C. Buonerba, D. Di Concilio, M.C. Cuomo, M.R. Capobianchi, A. Di Caro
EPI_ISL_737204	Central Virology Laboratory, Israel Ministry of Health	Central Virology Laboratory, Israel Ministry of Health	Neta S. Zuckerman, Efrat Dahan Bucris, Oran Erster, Michal Mandelboim, Orna Mor, Ella Mendelson
EPI_ISL_737239, EPI_ISL_737244, EPI_ISL_737247, EPI_ISL_737249, EPI_ISL_737253, EPI_ISL_737254, EPI_ISL_737260	Los Angeles County PHL	Los Angeles County PHL	P. Hemarajata et al.
EPI_ISL_737313, EPI_ISL_737319, EPI_ISL_737320, EPI_ISL_737322, EPI_ISL_737325, EPI_ISL_737335, EPI_ISL_737343, EPI_ISL_737346, EPI_ISL_737350, EPI_ISL_737351, EPI_ISL_737353, EPI_ISL_737359, EPI_ISL_737369, EPI_ISL_737372, EPI_ISL_737379, EPI_ISL_737381, EPI_ISL_737383, EPI_ISL_737384, EPI_ISL_737385, EPI_ISL_737390, EPI_ISL_737392			
see above	Department of Clinical Microbiology	GIGA Medical Genomics	Keith Durkin, Maria Artesi, Sébastien Bontems, Raphaël Boreux, Bouchra Boujemla, Cécile Meex, Pierrette Melin, Marie-Pierre Hayette, Vincent Bours
EPI_ISL_737876	Viollier AG	Department of Biosystems Science and Engineering, ETH Zürich	Chaoran Chen, Sarah Nadeau, Catharine Aquino, Ivan Topolsky, Philipp Jablonski, Lara Fuhrmann, David Dreifuss, Katharina Jahn, Andreia Cabral de Gouvea, Maria Domenica Moccia, Simon Grüter, Timothy Sykes, Lennart Opitz, Griffin White, Laura Neff, Doris Popovic, Andrea Patrignani, Jay Tracy, Ralph Schlapbach, Christiane Beckmann, Maurice Redondo, Olivier Kobel, Christoph Noppen, Sophie Seidel, Noemie Santamaria de Souza, Niko Beerenwinkel, Tanja Stadler
EPI_ISL_738048	SIESP CHIETI - DRIVE IN ORTONA	Istituto Zooprofilattico Sperimentale dell'Abruzzo e Molise "G. Caporale"	Lorusso A, Marcacci M, Di Domenico M, Ancora M, Curini V, Mangone I, Rinaldi A, Di Pasquale A, Cammà C, Puglia I, Savini G
EPI_ISL_738093, EPI_ISL_738094, EPI_ISL_738095, EPI_ISL_738096, EPI_ISL_738097, EPI_ISL_738098, EPI_ISL_738099, EPI_ISL_738100, EPI_ISL_738101, EPI_ISL_738102, EPI_ISL_738103, EPI_ISL_738104, EPI_ISL_738105, EPI_ISL_738108, EPI_ISL_738109, EPI_ISL_738110, EPI_ISL_738112, EPI_ISL_738113, EPI_ISL_738114			
see above	Instituto Nacional de Saude (INSA)	Instituto Nacional de Saude (INSA)	Borges et al
EPI_ISL_738133	The Public Health Agency of Sweden	The Public Health Agency of Sweden	Oskar Karlsson Lindsjo, Maria Lind Karlberg, Anna-Malin Linde, Sofia Stamouli, Olov Svartstrom, Anna Risberg, Shaman Muradrasoli, Karin Tegmark-Wisell
EPI_ISL_738134	Department of Virology and Immunology, University of Helsinki and Helsinki University Hospital, Huslab Finland	Department of Virology, Faculty of Medicine, University of Helsinki, Helsinki, Finland	Teemu Smura, Olli Vapalahti, Maija Lappalainen, Satu Kurkela
EPI_ISL_738142	Division of Emerging Infectious Diseases, Bureau of Infectious Diseases Diagnosis Control, Korea Disease Control and Prevention Agency	Division of Emerging Infectious Diseases, Bureau of Infectious Diseases Diagnosis Control, Korea Disease Control and Prevention Agency	Jeong-Min Kim, Il-Hwan Kim, Ae Kyung Park, Namjoo Lee, Sang Hee Woo, Heui Man Kim, Eun-Jin Kim
EPI_ISL_738144	University of Bari Biomedical Sciences and Human Oncology	University of Bari Biomedical Sciences and Human Oncology	Maria Chironna, Anna Sallustio, Daniela Loconsole, Marisa Accogli
EPI_ISL_738361, EPI_ISL_738362, EPI_ISL_738363, EPI_ISL_738364, EPI_ISL_738365, EPI_ISL_738366, EPI_ISL_738367, EPI_ISL_738368, EPI_ISL_738369, EPI_ISL_738370, EPI_ISL_738371, EPI_ISL_738372, EPI_ISL_738373, EPI_ISL_738374, EPI_ISL_738375, EPI_ISL_738376, EPI_ISL_738377, EPI_ISL_738378, EPI_ISL_738379, EPI_ISL_738380, EPI_ISL_738381, EPI_ISL_738382, EPI_ISL_738383, EPI_ISL_738384, EPI_ISL_738385, EPI_ISL_738386, EPI_ISL_738387, EPI_ISL_738388, EPI_ISL_738389, EPI_ISL_738390, EPI_ISL_738391, EPI_ISL_738392, EPI_ISL_738393, EPI_ISL_738394, EPI_ISL_738395, EPI_ISL_738396, EPI_ISL_738397, EPI_ISL_738398, EPI_ISL_738399, EPI_ISL_738400, EPI_ISL_738401, EPI_ISL_738402, EPI_ISL_738403, EPI_ISL_738404, EPI_ISL_738405, EPI_ISL_738406, EPI_ISL_738407, EPI_ISL_738408, EPI_ISL_738409, EPI_ISL_738410, EPI_ISL_738411, EPI_ISL_738412, EPI_ISL_738414, EPI_ISL_738415, EPI_ISL_738416, EPI_ISL_738418, EPI_ISL_738420, EPI_ISL_738421, EPI_ISL_738422, EPI_ISL_738423, EPI_ISL_738424, EPI_ISL_738426, EPI_ISL_738427, EPI_ISL_738428, EPI_ISL_738429, EPI_ISL_738430, EPI_ISL_738431, EPI_ISL_738432, EPI_ISL_738433, EPI_ISL_738434, EPI_ISL_738435, EPI_ISL_738437, EPI_ISL_738438, EPI_ISL_738439, EPI_ISL_738440, EPI_ISL_738441, EPI_ISL_738443, EPI_ISL_738444, EPI_ISL_738445, EPI_ISL_738446, EPI_ISL_738449, EPI_ISL_738450, EPI_ISL_738451, EPI_ISL_738452, EPI_ISL_738455, EPI_ISL_738461, EPI_ISL_738462, EPI_ISL_738464, EPI_ISL_738465, EPI_ISL_738467, EPI_ISL_738472, EPI_ISL_738474, EPI_ISL_738476, EPI_ISL_738477, EPI_ISL_738482, EPI_ISL_738483, EPI_ISL_738484, EPI_ISL_738485, EPI_ISL_738487, EPI_ISL_738488, EPI_ISL_738489, EPI_ISL_738490, EPI_ISL_738491, EPI_ISL_738494, EPI_ISL_738499			
see above	UZ Leuven, National Reference Laboratory for Coronaviruses, Laboratory Medicine, Leuven, Belgium	KU Leuven, Rega Institute, Clinical and Epidemiological Virology	Tony Wawina-Bokalanga, Joan Marti-Carerras, Bert Vanmechelen, Piet Maes
EPI_ISL_738712	Renegade	Chan-Zuckerberg Biohub	CZB Cliahub Consortium
EPI_ISL_738750	Santa Clara County Public Health Laboratory	Chan-Zuckerberg Biohub	CZB Cliahub Consortium
EPI_ISL_738752, EPI_ISL_738758, EPI_ISL_738808, EPI_ISL_738829	Orange County Public Health Lab	Chan-Zuckerberg Biohub	CZB Cliahub Consortium
EPI_ISL_738851	Renegade	Chan-Zuckerberg Biohub	CZB Cliahub Consortium
EPI_ISL_738880, EPI_ISL_738939, EPI_ISL_738954	Orange County Public Health Lab	Chan-Zuckerberg Biohub	CZB Cliahub Consortium
EPI_ISL_738967	Santa Clara County Public Health Laboratory	Chan-Zuckerberg Biohub	CZB Cliahub Consortium
EPI_ISL_738977	Renegade	Chan-Zuckerberg Biohub	CZB Cliahub Consortium
EPI_ISL_738981, EPI_ISL_738989, EPI_ISL_738993	Orange County Public Health Lab	Chan-Zuckerberg Biohub	CZB Cliahub Consortium
EPI_ISL_739040, EPI_ISL_739050, EPI_ISL_739058, EPI_ISL_739166	Santa Clara County Public Health Laboratory	Chan-Zuckerberg Biohub	CZB Cliahub Consortium
EPI_ISL_739182, EPI_ISL_739259	Orange County Public Health Lab	Chan-Zuckerberg Biohub	CZB Cliahub Consortium
EPI_ISL_739370	Santa Clara County Public Health Laboratory	Chan-Zuckerberg Biohub	CZB Cliahub Consortium
EPI_ISL_739432, EPI_ISL_739462	Orange County Public Health Lab	Chan-Zuckerberg Biohub	CZB Cliahub Consortium
EPI_ISL_739467	Renegade	Chan-Zuckerberg Biohub	CZB Cliahub Consortium
EPI_ISL_739488	Orange County Public Health Lab	Chan-Zuckerberg Biohub	CZB Cliahub Consortium
EPI_ISL_739593	Santa Clara County Public Health Laboratory	Chan-Zuckerberg Biohub	CZB Cliahub Consortium
EPI_ISL_739599	Orange County Public Health Lab	Chan-Zuckerberg Biohub	CZB Cliahub Consortium
EPI_ISL_739910, EPI_ISL_739936, EPI_ISL_739937, EPI_ISL_739943, EPI_ISL_739971, EPI_ISL_740039, EPI_ISL_740056, EPI_ISL_740061, EPI_ISL_740077, EPI_ISL_740111, EPI_ISL_740160, EPI_ISL_740165, EPI_ISL_740214, EPI_ISL_740237, EPI_ISL_740267, EPI_ISL_740271, EPI_ISL_740274, EPI_ISL_740330, EPI_ISL_740345, EPI_ISL_740375, EPI_ISL_740387, EPI_ISL_740424, EPI_ISL_740489, EPI_ISL_740532, EPI_ISL_740546			
see above	Laboratoire national de santé, Microbiology, Virology	Laboratoire national de santé, Microbiology, Microbial Genomics Platform	Anke Wienecke-Baldacchino, Catherine Ragimbeau,Jessica Tapp, Fatu Djabi, Lise Pignon, Raoul Salmon, Tamir Abdelrahman
EPI_ISL_740981, EPI_ISL_741005, EPI_ISL_741016, EPI_ISL_741017	Department of Pathology, University of Cambridge	COVID-19 Genomics UK (COG-UK) Consortium	Aminu S. Jahun, Yasmin Chaudhry, Grant Hall, Iliana Georgana, Myra Hosmillo, Martin D. Curran, Malte Pinckert, Surendra Parmar, Ian Goodfellow

EPI_ISL_741428, EPI_ISL_741429, EPI_ISL_741430	Northumbria University / South Tees Hospitals NHS Foundation Trust / North Cumbria Integrated Care NHS Foundation Trust / North Tees and Hartlepool NHS Foundation Trust / Newcastle Hospitals NHS Foundation Trust	COVID-19 Genomics UK (COG-UK) Consortium	Darren L Smith,Andrew Nelson,Matthew Bashton,Greg R Young,Joshua Loh,John Allan,Mohammad A Tariq,Giles S Holt,Gary Black,Wen C Yew,Lynn Dover,Paul Baker,Steve Liggett,Sarah Essex,Jane Greenaway,Debra Padgett,Clive Graham,Garren Scott,Edward Barton,Emma Swindells,Brendan Payne,Jennifer Collins,Yusri Taha,Gary Eltringham
EPI_ISL_741488, EPI_ISL_741489, EPI_ISL_741490, EPI_ISL_741492, EPI_ISL_741535, EPI_ISL_741558, EPI_ISL_741560, EPI_ISL_741561, EPI_ISL_741564, EPI_ISL_741568, EPI_ISL_741570, EPI_ISL_741572, EPI_ISL_741573, EPI_ISL_741574, EPI_ISL_741580, EPI_ISL_741584, EPI_ISL_741586, EPI_ISL_741587, EPI_ISL_741588, EPI_ISL_741589, EPI_ISL_741590, EPI_ISL_741591, EPI_ISL_741594, EPI_ISL_741597, EPI_ISL_741599, EPI_ISL_741600, EPI_ISL_741605			
see above	Quadram Institute Bioscience	COVID-19 Genomics UK (COG-UK) Consortium	Dave J. Baker, Gemma L. Kay, Alp Aydin, Thanh Le-Viet, Steven Rudder, Ana P. Tedim, Anastasia Kolyva, Maria Diaz, Leonardo de Oliveira Martins, Nabil-Fareed Alikhan, Lizzie Meadows, Rachael Stanley, Ngozi Elumogo, Muhammed Yasir, Nicholas M. Thomson, Alexander J Trotter, Rachel Gilroy, Samuel Bloomfield, Claire Stuart, Andrew Bell, Reenesh Prakash, Samir Dervisevic, Alison E. Mather, John Wain, Mark Webber, Andrew J. Page, Justin O'Grady
EPI_ISL_741612, EPI_ISL_741623, EPI_ISL_741624, EPI_ISL_741627, EPI_ISL_741629, EPI_ISL_741632, EPI_ISL_741639, EPI_ISL_741640, EPI_ISL_741648, EPI_ISL_741656	Queens Medical Centre, Clinical Microbiology Department / DeepSeq Nottingham	COVID-19 Genomics UK (COG-UK) Consortium	Gemma Clark, Wendy Smith, Manjinder Khakh, Vicki M Fleming, Michelle M Lister, Hannah Howson-Wells, Jonathan Ball, Patrick McClure, Joseph Chappell, Theocharis Tsoleridis, Nadine Holmes, Matthew Carlisle, Christopher Moore, Fei Sang, Johnny Debebe, Victoria Wright, Matthew Loose
EPI_ISL_741675, EPI_ISL_741732	Centre for Enzyme Innovation, University of Portsmouth / Translational Research Laboratory, Portsmouth Hospitals NHS Trust	COVID-19 Genomics UK (COG-UK) Consortium	Angela Beckett,Yann Bourgeois,Garry Scarlett,Sharon Glaysher,Scott Elliott,Kelly Bicknell,Robert Impey,Allyson Lloyd,Sarah Wyllie,Ethan Butcher,Anoop Chauhan,Samuel Robson
EPI_ISL_742178, EPI_ISL_742184, EPI_ISL_742186, EPI_ISL_742188, EPI_ISL_742189, EPI_ISL_742191, EPI_ISL_742192, EPI_ISL_742195, EPI_ISL_742200, EPI_ISL_742201, EPI_ISL_742203, EPI_ISL_742204, EPI_ISL_742205, EPI_ISL_742206, EPI_ISL_742207, EPI_ISL_742208, EPI_ISL_742209, EPI_ISL_742210, EPI_ISL_742212, EPI_ISL_742213, EPI_ISL_742214, EPI_ISL_742215, EPI_ISL_742216, EPI_ISL_742219, EPI_ISL_742220, EPI_ISL_742221, EPI_ISL_742223, EPI_ISL_742224, EPI_ISL_742225, EPI_ISL_742226, EPI_ISL_742227, EPI_ISL_742228, EPI_ISL_742230, EPI_ISL_742231, EPI_ISL_742232, EPI_ISL_742233, EPI_ISL_742234, EPI_ISL_742235, EPI_ISL_742237, EPI_ISL_742238, EPI_ISL_742239, EPI_ISL_742240, EPI_ISL_742242			
see above	Virology Department, Royal Infirmary of Edinburgh, NHS Lothian / School of Biological Sciences, University of Edinburgh / Institute of Genetics and Molecular Medicine, University of Edinburgh	COVID-19 Genomics UK (COG-UK) Consortium	McHugh M, Dewar R, Rooke S, Gallagher M, Balcaza C, O'Toole Á, Scher E, Hill V, McCrone JT, Colquhoun R, Yu X, Jackson B, Rambaut A, Williams TC, Templeton K
EPI_ISL_742289, EPI_ISL_742298, EPI_ISL_742367, EPI_ISL_742369, EPI_ISL_742373, EPI_ISL_742380, EPI_ISL_742407, EPI_ISL_742413, EPI_ISL_742419, EPI_ISL_742427, EPI_ISL_742428, EPI_ISL_742432, EPI_ISL_742433, EPI_ISL_742435, EPI_ISL_742436, EPI_ISL_742446, EPI_ISL_742447, EPI_ISL_742448, EPI_ISL_742450, EPI_ISL_742451, EPI_ISL_742455, EPI_ISL_742456, EPI_ISL_742478, EPI_ISL_742481, EPI_ISL_742484, EPI_ISL_742493, EPI_ISL_742495, EPI_ISL_742496, EPI_ISL_742497, EPI_ISL_742501, EPI_ISL_742539, EPI_ISL_742552, EPI_ISL_742555, EPI_ISL_742560, EPI_ISL_742566, EPI_ISL_742567, EPI_ISL_742575, EPI_ISL_742578, EPI_ISL_742581, EPI_ISL_742639, EPI_ISL_742652, EPI_ISL_742655, EPI_ISL_742661, EPI_ISL_742662, EPI_ISL_742667, EPI_ISL_742682, EPI_ISL_742687, EPI_ISL_742689, EPI_ISL_742692, EPI_ISL_742698, EPI_ISL_742702, EPI_ISL_742704, EPI_ISL_742705, EPI_ISL_742710, EPI_ISL_742715, EPI_ISL_742723, EPI_ISL_742726, EPI_ISL_742732, EPI_ISL_742747, EPI_ISL_742749, EPI_ISL_742751, EPI_ISL_742754, EPI_ISL_742755, EPI_ISL_742757, EPI_ISL_742758, EPI_ISL_742761, EPI_ISL_742762, EPI_ISL_742763, EPI_ISL_742766, EPI_ISL_742769, EPI_ISL_742770, EPI_ISL_742771, EPI_ISL_742773, EPI_ISL_742774, EPI_ISL_742775, EPI_ISL_742777, EPI_ISL_742779, EPI_ISL_742781, EPI_ISL_742782, EPI_ISL_742783, EPI_ISL_742785, EPI_ISL_742787, EPI_ISL_742793, EPI_ISL_742795, EPI_ISL_742797, EPI_ISL_742799, EPI_ISL_742800, EPI_ISL_742803, EPI_ISL_742804, EPI_ISL_742805, EPI_ISL_742808, EPI_ISL_742811, EPI_ISL_742820, EPI_ISL_742825, EPI_ISL_742830, EPI_ISL_742833, EPI_ISL_742834, EPI_ISL_742848, EPI_ISL_742849, EPI_ISL_742850, EPI_ISL_742855, EPI_ISL_742857, EPI_ISL_742860, EPI_ISL_742861, EPI_ISL_742866, EPI_ISL_742879, EPI_ISL_742905, EPI_ISL_742917, EPI_ISL_742923, EPI_ISL_742927, EPI_ISL_742930, EPI_ISL_742933, EPI_ISL_742942, EPI_ISL_742943, EPI_ISL_742945, EPI_ISL_742946, EPI_ISL_742948, EPI_ISL_742957, EPI_ISL_742960, EPI_ISL_742961, EPI_ISL_742962, EPI_ISL_742969, EPI_ISL_742970, EPI_ISL_742971, EPI_ISL_742972, EPI_ISL_742973, EPI_ISL_742975, EPI_ISL_742976, EPI_ISL_742979, EPI_ISL_742980, EPI_ISL_742981, EPI_ISL_742984, EPI_ISL_742986, EPI_ISL_742992, EPI_ISL_742994, EPI_ISL_742996, EPI_ISL_743013, EPI_ISL_743018, EPI_ISL_743019, EPI_ISL_743020, EPI_ISL_743023, EPI_ISL_743031, EPI_ISL_743032, EPI_ISL_743036, EPI_ISL_743041, EPI_ISL_743042, EPI_ISL_743048, EPI_ISL_743053, EPI_ISL_743054, EPI_ISL_743061, EPI_ISL_743064, EPI_ISL_743065, EPI_ISL_743067, EPI_ISL_743070, EPI_ISL_743084, EPI_ISL_743085, EPI_ISL_743088, EPI_ISL_743091, EPI_ISL_743093, EPI_ISL_743096, EPI_ISL_743099, EPI_ISL_743101, EPI_ISL_743103, EPI_ISL_743105, EPI_ISL_743109, EPI_ISL_743112, EPI_ISL_743114, EPI_ISL_743127, EPI_ISL_743140, EPI_ISL_743142, EPI_ISL_743143, EPI_ISL_743158, EPI_ISL_743164, EPI_ISL_743170, EPI_ISL_743177, EPI_ISL_743179, EPI_ISL_743193, EPI_ISL_743207, EPI_ISL_743209, EPI_ISL_743212, EPI_ISL_743213, EPI_ISL_743216, EPI_ISL_743217, EPI_ISL_743218, EPI_ISL_743222, EPI_ISL_743225, EPI_ISL_743237, EPI_ISL_743249, EPI_ISL_743260, EPI_ISL_743263, EPI_ISL_743271, EPI_ISL_743273, EPI_ISL_743279, EPI_ISL_743281, EPI_ISL_743285, EPI_ISL_743286, EPI_ISL_743287, EPI_ISL_743288, EPI_ISL_743291, EPI_ISL_743292, EPI_ISL_743296, EPI_ISL_743299, EPI_ISL_743300, EPI_ISL_743302, EPI_ISL_743304, EPI_ISL_743306, EPI_ISL_743308, EPI_ISL_743311, EPI_ISL_743314, EPI_ISL_743319, EPI_ISL_743322, EPI_ISL_743324, EPI_ISL_743325, EPI_ISL_743329, EPI_ISL_743330, EPI_ISL_743331, EPI_ISL_743333, EPI_ISL_743335, EPI_ISL_743336, EPI_ISL_743337, EPI_ISL_743340, EPI_ISL_743375, EPI_ISL_743379, EPI_ISL_743398, EPI_ISL_743412, EPI_ISL_743413, EPI_ISL_743416, EPI_ISL_743426, EPI_ISL_743427			
see above	Wales Specialist Virology Centre Sequencing lab: Pathogen Genomics Unit	COVID-19 Genomics UK (COG-UK) Consortium	Catherine Moore, Johnathan Evans, Laura Gifford, Malorie Perry, Simon Cottrell, Angela Marchbank, Alec Birchley, Alexander Adams, Amy Gaskin, Bree Gatica-Wilcox, Jason Coombes, Joel Southgate, Lauren Gilbert, Lee Graham, Nicole Pacchiarini, Sara Kumziene-Summerhayes, Sarah Taylor, Sophie Jones, Sara Rey, Matthew Bull, Joanne Watkins, Sally Corden, Tom Connor

We gratefully acknowledge the following Authors from the Originating laboratories responsible for obtaining the specimens, as well as the Submitting laboratories where the genome data were generated and shared via GISAID, on which this research is based.

All Submitters of data may be contacted directly via www.gisaid.org

Authors are sorted alphabetically.

Accession ID	Originating Laboratory	Submitting Laboratory	Authors
EPI_ISL_743447, EPI_ISL_743529, EPI_ISL_743530, EPI_ISL_743531, EPI_ISL_743532, EPI_ISL_743534, EPI_ISL_743536, EPI_ISL_743537, EPI_ISL_743539, EPI_ISL_743540, EPI_ISL_743541, EPI_ISL_743542, EPI_ISL_743547, EPI_ISL_743549, EPI_ISL_743550, EPI_ISL_743552, EPI_ISL_743553, EPI_ISL_743554, EPI_ISL_743556, EPI_ISL_743557, EPI_ISL_743560, EPI_ISL_743563, EPI_ISL_743565, EPI_ISL_743573, EPI_ISL_743588, EPI_ISL_743590, EPI_ISL_743591, EPI_ISL_743596, EPI_ISL_743597, EPI_ISL_743600, EPI_ISL_743602, EPI_ISL_743603, EPI_ISL_743661, EPI_ISL_743664, EPI_ISL_743677, EPI_ISL_743690, EPI_ISL_743694, EPI_ISL_743695, EPI_ISL_743698, EPI_ISL_743726, EPI_ISL_743762, EPI_ISL_743780, EPI_ISL_743782, EPI_ISL_743786, EPI_ISL_743789, EPI_ISL_743792, EPI_ISL_743795, EPI_ISL_743800, EPI_ISL_743805, EPI_ISL_743812, EPI_ISL_743822, EPI_ISL_743823, EPI_ISL_743826, EPI_ISL_743827, EPI_ISL_743828, EPI_ISL_743830, EPI_ISL_743834, EPI_ISL_743838, EPI_ISL_743853, EPI_ISL_743854, EPI_ISL_743855, EPI_ISL_743856, EPI_ISL_743857, EPI_ISL_743858, EPI_ISL_743861, EPI_ISL_743866, EPI_ISL_743871, EPI_ISL_743879, EPI_ISL_743883, EPI_ISL_743886, EPI_ISL_743888, EPI_ISL_743889, EPI_ISL_743904, EPI_ISL_743907, EPI_ISL_743938, EPI_ISL_743939, EPI_ISL_743968, EPI_ISL_743990, EPI_ISL_744002, EPI_ISL_744005, EPI_ISL_744006, EPI_ISL_744007, EPI_ISL_744009, EPI_ISL_744014, EPI_ISL_744015, EPI_ISL_744017, EPI_ISL_744019, EPI_ISL_744021, EPI_ISL_744025, EPI_ISL_744027, EPI_ISL_744033, EPI_ISL_744035, EPI_ISL_744045, EPI_ISL_744046, EPI_ISL_744047, EPI_ISL_744048, EPI_ISL_744055, EPI_ISL_744056, EPI_ISL_744058, EPI_ISL_744060, EPI_ISL_744062, EPI_ISL_744065, EPI_ISL_744066, EPI_ISL_744067, EPI_ISL_744068, EPI_ISL_744070, EPI_ISL_744073, EPI_ISL_744083, EPI_ISL_744085, EPI_ISL_744093, EPI_ISL_744096, EPI_ISL_744117, EPI_ISL_744118, EPI_ISL_744119, EPI_ISL_744123, EPI_ISL_744125, EPI_ISL_744127, EPI_ISL_744128, EPI_ISL_744129			
see above	Wales Specialist Virology Centre Sequencing lab: Pathogen Genomics Unit	COVID-19 Genomics UK (COG-UK) Consortium	Catherine Moore, Johnathan Evans, Laura Gifford, Malorie Perry, Simon Cottrell, Angela Marchbank, Alec Birchley, Alexander Adams, Amy Gaskin, Bree Gatica-Wilcox, Jason Coombes, Joel Southgate, Lauren Gilbert, Lee Graham, Nicole Pacchiarini, Sara Kumziene-Summerhayes, Sarah Taylor, Sophie Jones, Sara Rey, Matthew Bull, Joanne Watkins, Sally Corden, Tom Connor
EPI_ISL_744158, EPI_ISL_744203, EPI_ISL_744206, EPI_ISL_744280, EPI_ISL_744294, EPI_ISL_744395, EPI_ISL_744396, EPI_ISL_744400, EPI_ISL_744435, EPI_ISL_744438, EPI_ISL_744456, EPI_ISL_744483, EPI_ISL_744519, EPI_ISL_744557, EPI_ISL_744605, EPI_ISL_744676, EPI_ISL_744734, EPI_ISL_744756, EPI_ISL_744780, EPI_ISL_744792, EPI_ISL_744793, EPI_ISL_744805, EPI_ISL_744809, EPI_ISL_744879, EPI_ISL_744893, EPI_ISL_744946			
see above	Laboratoire national de santé, Microbiology, Virology	Laboratoire national de santé, Microbiology, Microbial Genomics Platform	Anke Wienecke-Baldacchino, Catherine Ragimbeau,Jessica Tapp, Fatu Djabi, Lise Pignon, Raoul Salmon, Tamir Abdelrahman
EPI_ISL_745030, EPI_ISL_745031	Area of Virology, Serology and Virology Division (SAViD), New South Wales Health Pathology Randwick	Area of Virology, Serology and Virology Division (SAViD), New South Wales Health Pathology Randwick	Rawlinson, W.
EPI_ISL_745041, EPI_ISL_745044, EPI_ISL_745045, EPI_ISL_745046, EPI_ISL_745048, EPI_ISL_745052, EPI_ISL_745054, EPI_ISL_745061, EPI_ISL_745063, EPI_ISL_745066, EPI_ISL_745068, EPI_ISL_745071, EPI_ISL_745073, EPI_ISL_745083			
see above	Israel Central Virology laboratory	Israel Central Virology laboratory	Neta Zuckerman, Efrat Dahan Bucris, Oran Erster, Michal Mandelboim, Orna Mor, Ella Mendelson
EPI_ISL_745140	Tygerberg Hospital wc TBH	National Health Laboratory Service (NHLS), Tygerberg	Susan Engelbrecht, Kayla Delaney, Bronwyn Kleinhans, Houriiyah Tegally, Eduan Wilkindon, Gert van Zyl, Wolfgang Preiser, Tulio de Oliveira
EPI_ISL_745187	Port Nolloth Hospital	National Health Laboratory Service (NHLS), Tygerberg	Susan Engelbrecht, Kayla Delaney, Bronwyn Kleinhans, Houriiyah Tegally, Eduan Wilkindon, Gert van Zyl, Wolfgang Preiser, Tulio de Oliveira
EPI_ISL_745188	Nababeep Hospital	National Health Laboratory Service (NHLS), Tygerberg	Susan Engelbrecht, Kayla Delaney, Bronwyn Kleinhans, Houriiyah Tegally, Eduan Wilkindon, Gert van Zyl, Wolfgang Preiser, Tulio de Oliveira
EPI_ISL_745189	Vredenburg Hospital wc VBG	National Health Laboratory Service (NHLS), Tygerberg	Susan Engelbrecht, Kayla Delaney, Bronwyn Kleinhans, Houriiyah Tegally, Eduan Wilkindon, Gert van Zyl, Wolfgang Preiser, Tulio de Oliveira
EPI_ISL_745192	Ospedale Di Venere	Istituto Zooprofilattico Sperimentale della Puglia e della Basilicata	Parisi A., Capozzi L., Del Sambro L., Bianco A., Chiara M., Pesole G., De Sabato L., Iacobellis M.
EPI_ISL_745193	Ospedale Di Venere	Istituto Zooprofilattico Sperimentale della Puglia e della Basilicata	Parisi A., Bianco A., Capozzi L., Del Sambro L., Chiara M., De Sabato L., Pesole G., Iacobellis M
EPI_ISL_745202, EPI_ISL_745203, EPI_ISL_745206, EPI_ISL_745207	Lab voor klinische biologie	Onderzoeksgroep Virologie	Laurens Lambrechts, Nick Vereecke, Marthe Pauwels, Bruno Verhasselt, Linos Vandekerckhove, Hans Nauwynck, Sebastiaan Theuns
EPI_ISL_745214, EPI_ISL_745216, EPI_ISL_745217	Lab voor klinische biologie	Onderzoeksgroep Virologie	Nick Vereecke, Laurens Lambrechts, Marthe Pauwels, Bruno Verhasselt, Linos Vandekerckhove, Hans Nauwynck, Sebastiaan Theuns
EPI_ISL_745222	UZ Leuven, National Reference Laboratory for Coronaviruses, Laboratory Medicine, Leuven, Belgium	KU Leuven, Rega Institute, Clinical and Epidemiological Virology	Tony Wawina-Bokalanga, Joan Marti-Carerras, Bert Vanmechelen, Piet Maes
EPI_ISL_745332, EPI_ISL_745352, EPI_ISL_745361, EPI_ISL_745363, EPI_ISL_745364, EPI_ISL_745365, EPI_ISL_745366, EPI_ISL_745379, EPI_ISL_745387, EPI_ISL_745391	CNR Virus des Infections Respiratoires - France SUD	CNR Virus des Infections Respiratoires - France SUD	Antonin Bal, Gregory Destras, Claudia Gonzalez, Gwendolyne Burfin, Quentin Semanas, Martine Valette, Bruno Lina, Laurence Josset
EPI_ISL_745446, EPI_ISL_745447	DOHMH Corona	New York City Public Health Laboratory	Jade Wang, et al.
EPI_ISL_745450	DOHMH Morrisania	New York City Public Health Laboratory	Jade Wang, et al.
EPI_ISL_745451	DOHMH Central Harlem	New York City Public Health Laboratory	Jade Wang, et al.
EPI_ISL_745454	DOHMH Crown Heights	New York City Public Health Laboratory	Jade Wang, et al.
EPI_ISL_745456	DOHMH PHL	New York City Public Health Laboratory	Jade Wang, et al.
EPI_ISL_745457	DOHMH Chelsea	New York City Public Health Laboratory	Jade Wang, et al.
EPI_ISL_745459, EPI_ISL_745460, EPI_ISL_745461, EPI_ISL_745464	DOHMH Corona	New York City Public Health Laboratory	Jade Wang, et al.
EPI_ISL_745465	DOHMH PHL	New York City Public Health Laboratory	Jade Wang, et al.
EPI_ISL_745466, EPI_ISL_745467	DOHMH Corona	New York City Public Health Laboratory	Jade Wang, et al.
EPI_ISL_745468	DOHMH Riverside	New York City Public Health Laboratory	Jade Wang, et al.
EPI_ISL_745469	DOHMH Central Harlem	New York City Public Health Laboratory	Jade Wang, et al.
EPI_ISL_746827	National Institute for Infectious Diseases, INMI, "L. Spallanzani" IRCCS	National Institute for Infectious Diseases, INMI, "L. Spallanzani" IRCCS	B Bartolini, C.E.M Gruber, M Rueca, F Messina, E Giombini, MR Capobianchi, A Di Caro
EPI_ISL_746828	National Institute for Infectious Diseases, INMI, "L. Spallanzani" IRCCS	National Institute for Infectious Diseases, INMI, "L. Spallanzani" IRCCS	M Rueca, B Bartolini, C.E.M Gruber, F Messina, E Giombini, A Di Caro, MR Capobianchi
EPI_ISL_746976, EPI_ISL_746986, EPI_ISL_747020, EPI_ISL_747021, EPI_ISL_747022, EPI_ISL_747023, EPI_ISL_747024, EPI_ISL_747025, EPI_ISL_747026, EPI_ISL_747027, EPI_ISL_747028, EPI_ISL_747030, EPI_ISL_747031			
see above	Utah Public Health Laboratory	Utah Public Health Laboratory	Erin Young, Kelly Oakeson, Tara Gallagher
EPI_ISL_747477, EPI_ISL_747481	ULSS 5 Polesana	Istituto Zooprofilattico Sperimentale delle Venezie	Adelaide Milani, Alessia Schivo, Annalisa Salviato, Erika Giorgia Quaranta, Ambra Pastori, Bianca Zecchin, Alice Fusaro, Isabella Monne, Calogero Terregino, Antonia Ricci
EPI_ISL_747495, EPI_ISL_747496, EPI_ISL_747498, EPI_ISL_747500, EPI_ISL_747501, EPI_ISL_747502, EPI_ISL_747504, EPI_ISL_747505, EPI_ISL_747507, EPI_ISL_747509, EPI_ISL_747510, EPI_ISL_747512, EPI_ISL_747519			

see above	Respiratory Virus Unit, National Infection Service, Public Health England	COVID-19 Genomics UK (COG-UK) Consortium	PHE Covid Sequencing Team
EPI_ISL_747523, EPI_ISL_747525	Dutch COVID-19 response team	National Institute for Public Health and the Environment (RIVM)	Adam Meijer, Harry Vennema, Jeroen Cremer, Sharon van den Brink, Bas van der Veer, AnneMarie van den Brandt, Florian Zwagemaker, Dennis Schmitz, Chantal Reusken, on behalf of the national COVID-19 response team
EPI_ISL_747610, EPI_ISL_747612, EPI_ISL_747613, EPI_ISL_747614, EPI_ISL_747616, EPI_ISL_747617, EPI_ISL_747618, EPI_ISL_747619, EPI_ISL_747620, EPI_ISL_747621, EPI_ISL_747622, EPI_ISL_747623, EPI_ISL_747632, EPI_ISL_747663, EPI_ISL_747664, EPI_ISL_747665, EPI_ISL_747666, EPI_ISL_747667, EPI_ISL_747668, EPI_ISL_747669, EPI_ISL_747670, EPI_ISL_747671, EPI_ISL_747672, EPI_ISL_747673, EPI_ISL_747674, EPI_ISL_747681, EPI_ISL_747682, EPI_ISL_747683, EPI_ISL_747684, EPI_ISL_747685, EPI_ISL_747686, EPI_ISL_747687, EPI_ISL_747688, EPI_ISL_747690, EPI_ISL_747692, EPI_ISL_747693, EPI_ISL_747694, EPI_ISL_747695, EPI_ISL_747696, EPI_ISL_747697, EPI_ISL_747698, EPI_ISL_747699, EPI_ISL_747700, EPI_ISL_747701, EPI_ISL_747702, EPI_ISL_747704, EPI_ISL_747705, EPI_ISL_747706, EPI_ISL_747707, EPI_ISL_747708, EPI_ISL_747728, EPI_ISL_747729, EPI_ISL_747757, EPI_ISL_747758, EPI_ISL_747759, EPI_ISL_747760, EPI_ISL_747761, EPI_ISL_747762, EPI_ISL_747763, EPI_ISL_747764, EPI_ISL_747765, EPI_ISL_747766, EPI_ISL_747767, EPI_ISL_747769, EPI_ISL_747770, EPI_ISL_747771, EPI_ISL_747773, EPI_ISL_747774, EPI_ISL_747775, EPI_ISL_747776, EPI_ISL_747777, EPI_ISL_747779, EPI_ISL_747780, EPI_ISL_747781, EPI_ISL_747783, EPI_ISL_747784, EPI_ISL_747786, EPI_ISL_747788, EPI_ISL_747789, EPI_ISL_747791, EPI_ISL_747792, EPI_ISL_747793, EPI_ISL_747794, EPI_ISL_747795, EPI_ISL_747796, EPI_ISL_747797, EPI_ISL_747798, EPI_ISL_747799, EPI_ISL_747801, EPI_ISL_747802, EPI_ISL_747803, EPI_ISL_747804, EPI_ISL_747805, EPI_ISL_747806, EPI_ISL_747807, EPI_ISL_747808, EPI_ISL_747809, EPI_ISL_747810, EPI_ISL_747811, EPI_ISL_747812, EPI_ISL_747813, EPI_ISL_747814, EPI_ISL_747815, EPI_ISL_747816, EPI_ISL_747817, EPI_ISL_747818, EPI_ISL_747819, EPI_ISL_747820, EPI_ISL_747821, EPI_ISL_747822, EPI_ISL_747824, EPI_ISL_747825, EPI_ISL_747826, EPI_ISL_747828, EPI_ISL_747829, EPI_ISL_747830, EPI_ISL_747831, EPI_ISL_747833, EPI_ISL_747834, EPI_ISL_747835, EPI_ISL_747836, EPI_ISL_747837, EPI_ISL_747838, EPI_ISL_747839, EPI_ISL_747840, EPI_ISL_747842, EPI_ISL_747843, EPI_ISL_747844, EPI_ISL_747845, EPI_ISL_747846, EPI_ISL_747847, EPI_ISL_747848, EPI_ISL_747849, EPI_ISL_747851, EPI_ISL_747853, EPI_ISL_747854, EPI_ISL_747855, EPI_ISL_747856, EPI_ISL_747857, EPI_ISL_747858, EPI_ISL_747859, EPI_ISL_747860, EPI_ISL_747861, EPI_ISL_747862, EPI_ISL_747863, EPI_ISL_747864, EPI_ISL_747865, EPI_ISL_747866, EPI_ISL_747867, EPI_ISL_747869, EPI_ISL_747870, EPI_ISL_747871, EPI_ISL_747872, EPI_ISL_747873, EPI_ISL_747875, EPI_ISL_747876, EPI_ISL_747877, EPI_ISL_747878, EPI_ISL_747880, EPI_ISL_747881, EPI_ISL_747882, EPI_ISL_747883, EPI_ISL_747884, EPI_ISL_747885, EPI_ISL_747886, EPI_ISL_747887, EPI_ISL_747888, EPI_ISL_747889, EPI_ISL_747890, EPI_ISL_747892, EPI_ISL_747893, EPI_ISL_747895, EPI_ISL_747896, EPI_ISL_747897, EPI_ISL_747898, EPI_ISL_747899, EPI_ISL_747900, EPI_ISL_747901, EPI_ISL_747902, EPI_ISL_747903, EPI_ISL_747905, EPI_ISL_747907, EPI_ISL_747908, EPI_ISL_747909, EPI_ISL_747910, EPI_ISL_747911, EPI_ISL_747915, EPI_ISL_747916, EPI_ISL_747917, EPI_ISL_747918, EPI_ISL_747919, EPI_ISL_747920, EPI_ISL_747922, EPI_ISL_747924, EPI_ISL_747925, EPI_ISL_747927, EPI_ISL_747928, EPI_ISL_747929, EPI_ISL_747930, EPI_ISL_747931, EPI_ISL_747932, EPI_ISL_747934, EPI_ISL_747935, EPI_ISL_747936, EPI_ISL_747937, EPI_ISL_747938, EPI_ISL_747939, EPI_ISL_747940, EPI_ISL_747941, EPI_ISL_747942, EPI_ISL_747943, EPI_ISL_747945, EPI_ISL_747947, EPI_ISL_747949, EPI_ISL_747950, EPI_ISL_747951, EPI_ISL_747952, EPI_ISL_747953, EPI_ISL_747955, EPI_ISL_747956, EPI_ISL_747958, EPI_ISL_747959, EPI_ISL_747960, EPI_ISL_747961, EPI_ISL_747962, EPI_ISL_747963, EPI_ISL_747964, EPI_ISL_747965, EPI_ISL_747966, EPI_ISL_747967, EPI_ISL_747968, EPI_ISL_747969, EPI_ISL_747970, EPI_ISL_747971, EPI_ISL_747972, EPI_ISL_747973, EPI_ISL_747974, EPI_ISL_747975, EPI_ISL_747976, EPI_ISL_747977, EPI_ISL_747978, EPI_ISL_747979, EPI_ISL_747981, EPI_ISL_747982, EPI_ISL_747983, EPI_ISL_747985, EPI_ISL_747986, EPI_ISL_747987, EPI_ISL_747988, EPI_ISL_747989, EPI_ISL_747990, EPI_ISL_747991, EPI_ISL_747992, EPI_ISL_747993, EPI_ISL_747994, EPI_ISL_747995, EPI_ISL_747996, EPI_ISL_747997, EPI_ISL_747998, EPI_ISL_747999, EPI_ISL_748001, EPI_ISL_748002, EPI_ISL_748003, EPI_ISL_748004, EPI_ISL_748006, EPI_ISL_748007, EPI_ISL_748008, EPI_ISL_748009, EPI_ISL_748010, EPI_ISL_748012, EPI_ISL_748013, EPI_ISL_748014, EPI_ISL_748015, EPI_ISL_748016, EPI_ISL_748017, EPI_ISL_748018, EPI_ISL_748019, EPI_ISL_748020, EPI_ISL_748279, EPI_ISL_748280, EPI_ISL_748282, EPI_ISL_748283, EPI_ISL_748285, EPI_ISL_748286, EPI_ISL_748287, EPI_ISL_748288, EPI_ISL_748289, EPI_ISL_748290, EPI_ISL_748291, EPI_ISL_748292, EPI_ISL_748293, EPI_ISL_748294, EPI_ISL_748295, EPI_ISL_748296, EPI_ISL_748297, EPI_ISL_748298, EPI_ISL_748299, EPI_ISL_748300, EPI_ISL_748301, EPI_ISL_748302, EPI_ISL_748303, EPI_ISL_748304, EPI_ISL_748305, EPI_ISL_748306, EPI_ISL_748307, EPI_ISL_748308, EPI_ISL_748310, EPI_ISL_748311, EPI_ISL_748312, EPI_ISL_748313, EPI_ISL_748314, EPI_ISL_748315, EPI_ISL_748316, EPI_ISL_748317, EPI_ISL_748318, EPI_ISL_748319, EPI_ISL_748320, EPI_ISL_748321, EPI_ISL_748322, EPI_ISL_748323, EPI_ISL_748324, EPI_ISL_748359, EPI_ISL_748360, EPI_ISL_748361, EPI_ISL_748363, EPI_ISL_748364, EPI_ISL_748365, EPI_ISL_748366, EPI_ISL_748367, EPI_ISL_748368, EPI_ISL_748369, EPI_ISL_748370, EPI_ISL_748371, EPI_ISL_748372, EPI_ISL_748373, EPI_ISL_748374, EPI_ISL_748375, EPI_ISL_748376, EPI_ISL_748377, EPI_ISL_748378, EPI_ISL_748379, EPI_ISL_748380, EPI_ISL_748381, EPI_ISL_748382, EPI_ISL_748384, EPI_ISL_748385, EPI_ISL_748386, EPI_ISL_748387, EPI_ISL_748388, EPI_ISL_748389, EPI_ISL_748390, EPI_ISL_748391, EPI_ISL_748392, EPI_ISL_748393, EPI_ISL_748394, EPI_ISL_748395, EPI_ISL_748396, EPI_ISL_748397, EPI_ISL_748398, EPI_ISL_748399, EPI_ISL_748400, EPI_ISL_748401, EPI_ISL_748402, EPI_ISL_748404, EPI_ISL_748405, EPI_ISL_748406, EPI_ISL_748408, EPI_ISL_748410, EPI_ISL_748411, EPI_ISL_748412, EPI_ISL_748413, EPI_ISL_748414, EPI_ISL_748415, EPI_ISL_748416, EPI_ISL_748417, EPI_ISL_748419, EPI_ISL_748420, EPI_ISL_748421, EPI_ISL_748422, EPI_ISL_748423, EPI_ISL_748424, EPI_ISL_748426, EPI_ISL_748427, EPI_ISL_748428, EPI_ISL_748429, EPI_ISL_748430, EPI_ISL_748431, EPI_ISL_748432, EPI_ISL_748433, EPI_ISL_748434, EPI_ISL_748435, EPI_ISL_748436, EPI_ISL_748437, EPI_ISL_748438, EPI_ISL_748439, EPI_ISL_748440, EPI_ISL_748441, EPI_ISL_748442, EPI_ISL_748443, EPI_ISL_748444, EPI_ISL_748446, EPI_ISL_748449, EPI_ISL_748450, EPI_ISL_748451, EPI_ISL_748452, EPI_ISL_748453, EPI_ISL_748455, EPI_ISL_748456, EPI_ISL_748457, EPI_ISL_748458, EPI_ISL_748459, EPI_ISL_748460, EPI_ISL_748461, EPI_ISL_748462, EPI_ISL_748463, EPI_ISL_748464, EPI_ISL_748465, EPI_ISL_748466, EPI_ISL_748470, EPI_ISL_748471, EPI_ISL_748484, EPI_ISL_748485, EPI_ISL_748486, EPI_ISL_748487, EPI_ISL_748488, EPI_ISL_748489, EPI_ISL_748490, EPI_ISL_748491, EPI_ISL_748492, EPI_ISL_748507, EPI_ISL_748532, EPI_ISL_748534, EPI_ISL_748535, EPI_ISL_748536, EPI_ISL_748537, EPI_ISL_748538, EPI_ISL_748539, EPI_ISL_748541, EPI_ISL_748542, EPI_ISL_748543, EPI_ISL_748544, EPI_ISL_748545, EPI_ISL_748546, EPI_ISL_748548, EPI_ISL_748549, EPI_ISL_748550, EPI_ISL_748553, EPI_ISL_748554, EPI_ISL_748555, EPI_ISL_748556, EPI_ISL_748557, EPI_ISL_748558, EPI_ISL_748559, EPI_ISL_748561, EPI_ISL_748562, EPI_ISL_748563, EPI_ISL_748564, EPI_ISL_748565, EPI_ISL_748566, EPI_ISL_748567, EPI_ISL_748568, EPI_ISL_748569, EPI_ISL_748570, EPI_ISL_748571, EPI_ISL_748572, EPI_ISL_748573, EPI_ISL_748574, EPI_ISL_748575, EPI_ISL_748576, EPI_ISL_748577, EPI_ISL_748578, EPI_ISL_748579, EPI_ISL_748580, EPI_ISL_748581, EPI_ISL_748583, EPI_ISL_748584, EPI_ISL_748585, EPI_ISL_748586, EPI_ISL_748587, EPI_ISL_748588, EPI_ISL_748589, EPI_ISL_748590, EPI_ISL_748591, EPI_ISL_748594, EPI_ISL_748596, EPI_ISL_748597, EPI_ISL_748599, EPI_ISL_748600, EPI_ISL_748601, EPI_ISL_748603, EPI_ISL_748604, EPI_ISL_748605, EPI_ISL_748606, EPI_ISL_748607, EPI_ISL_748608, EPI_ISL_748609, EPI_ISL_748610, EPI_ISL_748612, EPI_ISL_748613, EPI_ISL_748614, EPI_ISL_748615, EPI_ISL_748616, EPI_ISL_748617, EPI_ISL_748618, EPI_ISL_748620, EPI_ISL_748621, EPI_ISL_748622, EPI_ISL_748623, EPI_ISL_748624, EPI_ISL_748625, EPI_ISL_748626, EPI_ISL_748627, EPI_ISL_748628, EPI_ISL_748629, EPI_ISL_748630, EPI_ISL_748631, EPI_ISL_748632, EPI_ISL_748634, EPI_ISL_748636, EPI_ISL_748638, EPI_ISL_748639, EPI_ISL_748640, EPI_ISL_748641, EPI_ISL_748642, EPI_ISL_748643, EPI_ISL_748644, EPI_ISL_748645, EPI_ISL_748646, EPI_ISL_748647, EPI_ISL_748648, EPI_ISL_748649, EPI_ISL_748650, EPI_ISL_748652, EPI_ISL_748653, EPI_ISL_748654, EPI_ISL_748655, EPI_ISL_748656, EPI_ISL_748657, EPI_ISL_748658, EPI_ISL_748659, EPI_ISL_748660, EPI_ISL_748661, EPI_ISL_748663, EPI_ISL_748664, EPI_ISL_748666, EPI_ISL_748668, EPI_ISL_748669, EPI_ISL_748670, EPI_ISL_748671, EPI_ISL_748672, EPI_ISL_748673, EPI_ISL_748674, EPI_ISL_748675, EPI_ISL_748676, EPI_ISL_748677, EPI_ISL_748678, EPI_ISL_748679, EPI_ISL_748680, EPI_ISL_748681, EPI_ISL_748682, EPI_ISL_748683, EPI_ISL_748684, EPI_ISL_748685, EPI_ISL_748686, EPI_ISL_748687, EPI_ISL_748688, EPI_ISL_748689, EPI_ISL_748690, EPI_ISL_748691, EPI_ISL_748692, EPI_ISL_748693, EPI_ISL_748694, EPI_ISL_748695, EPI_ISL_748696, EPI_ISL_748697, EPI_ISL_748698, EPI_ISL_748699, EPI_ISL_748700, EPI_ISL_748701, EPI_ISL_748703, EPI_ISL_748704, EPI_ISL_748705, EPI_ISL_748707, EPI_ISL_748708, EPI_ISL_748709, EPI_ISL_748710, EPI_ISL_748711, EPI_ISL_748712, EPI_ISL_748715, EPI_ISL_748716, EPI_ISL_748717, EPI_ISL_748718, EPI_ISL_748719, EPI_ISL_748720, EPI_ISL_748721, EPI_ISL_748722, EPI_ISL_748723, EPI_ISL_748724, EPI_ISL_748725, EPI_ISL_748726, EPI_ISL_748727, EPI_ISL_748728, EPI_ISL_748729, EPI_ISL_748730, EPI_ISL_748731, EPI_ISL_748732, EPI_ISL_748733, EPI_ISL_748735, EPI_ISL_748736, EPI_ISL_748737, EPI_ISL_748738, EPI_ISL_748739, EPI_ISL_748740, EPI_ISL_748741, EPI_ISL_748743, EPI_ISL_748744, EPI_ISL_748745, EPI_ISL_748746, EPI_ISL_748747, EPI_ISL_748748, EPI_ISL_748749, EPI_ISL_748750, EPI_ISL_748751, EPI_ISL_748752, EPI_ISL_748753, EPI_ISL_748755, EPI_ISL_748756, EPI_ISL_748757, EPI_ISL_748758, EPI_ISL_748759, EPI_ISL_748760, EPI_ISL_748761, EPI_ISL_748762, EPI_ISL_748763, EPI_ISL_748764, EPI_ISL_748765, EPI_ISL_748766, EPI_ISL_748767, EPI_ISL_748768, EPI_ISL_748769, EPI_ISL_748770, EPI_ISL_748771, EPI_ISL_748772, EPI_ISL_748773, EPI_ISL_748774, EPI_ISL_748776, EPI_ISL_748777, EPI_ISL_748778, EPI_ISL_748779, EPI_ISL_748780, EPI_ISL_748781, EPI_ISL_748782, EPI_ISL_748783, EPI_ISL_748784, EPI_ISL_748785, EPI_ISL_748787, EPI_ISL_748788, EPI_ISL_748789, EPI_ISL_748790, EPI_ISL_748791, EPI_ISL_748792, EPI_ISL_748794, EPI_ISL_748795, EPI_ISL_748796, EPI_ISL_748797, EPI_ISL_748798, EPI_ISL_748799, EPI_ISL_748800, EPI_ISL_748801, EPI_ISL_748802, EPI_ISL_748803, EPI_ISL_748804, EPI_ISL_748805, EPI_ISL_748806, EPI_ISL_748807, EPI_ISL_748808, EPI_ISL_748809, EPI_ISL_748810, EPI_ISL_748812, EPI_ISL_748813, EPI_ISL_748814, EPI_ISL_748815, EPI_ISL_748816, EPI_ISL_748817, EPI_ISL_748819, EPI_ISL_748820, EPI_ISL_748821, EPI_ISL_748822, EPI_ISL_748824, EPI_ISL_748825, EPI_ISL_748827, EPI_ISL_748828, EPI_ISL_748829, EPI_ISL_748830, EPI_ISL_748831, EPI_ISL_748832, EPI_ISL_748833, EPI_ISL_748835, EPI_ISL_748836, EPI_ISL_748837, EPI_ISL_748838, EPI_ISL_748839, EPI_ISL_748840, EPI_ISL_748841, EPI_ISL_748842, EPI_ISL_748843, EPI_ISL_748844, EPI_ISL_748845, EPI_ISL_748846, EPI_ISL_748847, EPI_ISL_748848, EPI_ISL_748849, EPI_ISL_748850, EPI_ISL_748851, EPI_ISL_748852, EPI_ISL_748853, EPI_ISL_748854, EPI_ISL_748855, EPI_ISL_748856, EPI_ISL_748858, EPI_ISL_748859, EPI_ISL_748860, EPI_ISL_748861, EPI_ISL_748862, EPI_ISL_748863, EPI_ISL_748864, EPI_ISL_748865, EPI_ISL_748866, EPI_ISL_748867, EPI_ISL_748868, EPI_ISL_748869, EPI_ISL_748870, EPI_ISL_748871, EPI_ISL_748873, EPI_ISL_748874, EPI_ISL_748875, EPI_ISL_748876, EPI_ISL_748877, EPI_ISL_748878, EPI_ISL_748879, EPI_ISL_748880, EPI_ISL_748881, EPI_ISL_748882, EPI_ISL_748883, EPI_ISL_748884, EPI_ISL_748885, EPI_ISL_748886, EPI_ISL_748887, EPI_ISL_748888, EPI_ISL_748889, EPI_ISL_748890, EPI_ISL_748891, EPI_ISL_748892, EPI_ISL_748893, EPI_ISL_748895, EPI_ISL_748897, EPI_ISL_748898, EPI_ISL_748899, EPI_ISL_748900, EPI_ISL_748901, EPI_ISL_748902, EPI_ISL_748903, EPI_ISL_748904, EPI_ISL_748905, EPI_ISL_748906, EPI_ISL_748908, EPI_ISL_748909, EPI_ISL_748910, EPI_ISL_748911, EPI_ISL_748912, EPI_ISL_748913, EPI_ISL_748914, EPI_ISL_748915, EPI_ISL_748916, EPI_ISL_748917, EPI_ISL_748919, EPI_ISL_748920, EPI_ISL_748921, EPI_ISL_748922, EPI_ISL_748923, EPI_ISL_748924, EPI_ISL_748925, EPI_ISL_748926, EPI_ISL_748927, EPI_ISL_748928, EPI_ISL_748930, EPI_ISL_748932, EPI_ISL_748933, EPI_ISL_748935, EPI_ISL_748936, EPI_ISL_748937, EPI_ISL_748938, EPI_ISL_748939, EPI_ISL_748940, EPI_ISL_748941, EPI_ISL_748942, EPI_ISL_748943, EPI_ISL_748944, EPI_ISL_748945, EPI_ISL_748947, EPI_ISL_748948, EPI_ISL_748950, EPI_ISL_748952, EPI_ISL_748953, EPI_ISL_748954, EPI_ISL_748955, EPI_ISL_748956, EPI_ISL_748957, EPI_ISL_748958, EPI_ISL_748959, EPI_ISL_748960, EPI_ISL_748961, EPI_ISL_748962, EPI_ISL_748963, EPI_ISL_748965, EPI_ISL_748966, EPI_ISL_748968, EPI_ISL_748969, EPI_ISL_748970, EPI_ISL_748971, EPI_ISL_748972, EPI_ISL_748973, EPI_ISL_748974, EPI_ISL_748975, EPI_ISL_748976, EPI_ISL_748978, EPI_ISL_748979, EPI_ISL_748980, EPI_ISL_748981, EPI_ISL_748982, EPI_ISL_748983, EPI_ISL_748985, EPI_ISL_748986, EPI_ISL_748987, EPI_ISL_748988, EPI_ISL_748989, EPI_ISL_748991, EPI_ISL_748992, EPI_ISL_748994, EPI_ISL_748995, EPI_ISL_748996, EPI_ISL_748998, EPI_ISL_749001, EPI_ISL_749002, EPI_ISL_749004, EPI_ISL_749005, EPI_ISL_749006, EPI_ISL_749007, EPI_ISL_749008, EPI_ISL_749009, EPI_ISL_749010, EPI_ISL_749011, EPI_ISL_749012, EPI_ISL_749014, EPI_ISL_749015, EPI_ISL_749016, EPI_ISL_749018, EPI_ISL_749019, EPI_ISL_749020, EPI_ISL_749021, EPI_ISL_749022, EPI_ISL_749023, EPI_ISL_749024, EPI_ISL_749025, EPI_ISL_749027, EPI_ISL_749028, EPI_ISL_749029, EPI_ISL_749030, EPI_ISL_749031, EPI_ISL_749032, EPI_ISL_749033, EPI_ISL_749035, EPI_ISL_749037, EPI_ISL_749038, EPI_ISL_749039, EPI_ISL_749040, EPI_ISL_749041, EPI_ISL_749042, EPI_ISL_749044, EPI_ISL_749045, EPI_ISL_749046, EPI_ISL_749047, EPI_ISL_749048, EPI_ISL_749049, EPI_ISL_749052, EPI_ISL_749053, EPI_ISL_749055, EPI_ISL_749056, EPI_ISL_749057, EPI_ISL_749060, EPI_ISL_749061, EPI_ISL_749062, EPI_ISL_749063, EPI_ISL_749064, EPI_ISL_749065, EPI_ISL_749066, EPI_ISL_749067, EPI_ISL_749068, EPI_ISL_749069, EPI_ISL_749070, EPI_ISL_749071, EPI_ISL_749072, EPI_ISL_749073, EPI_ISL_749074, EPI_ISL_749075, EPI_ISL_749076, EPI_ISL_749077, EPI_ISL_749078, EPI_ISL_749079, EPI_ISL_749081, EPI_ISL_749082, EPI_ISL_749083, EPI_ISL_749084, EPI_ISL_749086, EPI_ISL_749088, EPI_ISL_749089, EPI_ISL_749090, EPI_ISL_749091, EPI_ISL_749092, EPI_ISL_749093, EPI_ISL_749094, EPI_ISL_749095, EPI_ISL_749096, EPI_ISL_749097, EPI_ISL_749098, EPI_ISL_749100, EPI_ISL_749101, EPI_ISL_749102, EPI_ISL_749103, EPI_ISL_749104, EPI_ISL_749106, EPI_ISL_749107, EPI_ISL_749108, EPI_ISL_749109, EPI_ISL_749110, EPI_ISL_749111, EPI_ISL_749112, EPI_ISL_749113, EPI_ISL_749114, EPI_ISL_749115, EPI_ISL_749116, EPI_ISL_749117, EPI_ISL_749118, EPI_ISL_749119, EPI_ISL_749121, EPI_ISL_749122, EPI_ISL_749123, EPI_ISL_749124, EPI_ISL_749126, EPI_ISL_749127, EPI_ISL_749128, EPI_ISL_749130, EPI_ISL_749131, EPI_ISL_749132, EPI_ISL_749135, EPI_ISL_749136, EPI_ISL_749137, EPI_ISL_749138, EPI_ISL_749139, EPI_ISL_749140, EPI_ISL_749141, EPI_ISL_749142, EPI_ISL_749143, EPI_ISL_749144, EPI_ISL_749145, EPI_ISL_749147, EPI_ISL_749287, EPI_ISL_749288, EPI_ISL_749289, EPI_ISL_749290, EPI_ISL_749291, EPI_ISL_749292, EPI_ISL_749293, EPI_ISL_749294, EPI_ISL_749295, EPI_ISL_749296, EPI_ISL_749297, EPI_ISL_749300, EPI_ISL_749301, EPI_ISL_749302, EPI_ISL_749304, EPI_ISL_749305, EPI_ISL_749306, EPI_ISL_749308, EPI_ISL_749309, EPI_ISL_749310, EPI_ISL_749311, EPI_ISL_749312, EPI_ISL_749313, EPI_ISL_749314, EPI_ISL_749315, EPI_ISL_749316, EPI_ISL_749317, EPI_ISL_749318, EPI_ISL_749319, EPI_ISL_749320, EPI_ISL_749321, EPI_ISL_749322, EPI_ISL_749323, EPI_ISL_749324, EPI_ISL_749325, EPI_ISL_749326, EPI_ISL_749327, EPI_ISL_749328, EPI_ISL_749329, EPI_ISL_749330, EPI_ISL_749331, EPI_ISL_749332, EPI_ISL_749333, EPI_ISL_749334, EPI_ISL_749335, EPI_ISL_749336, EPI_ISL_749337, EPI_ISL_749338, EPI_ISL_749339, EPI_ISL_749340, EPI_ISL_749341, EPI_ISL_749342, EPI_ISL_749343, EPI_ISL_749344, EPI_ISL_749345, EPI_ISL_749346, EPI_ISL_749347, EPI_ISL_749348, EPI_ISL_749349, EPI_ISL_749350, EPI_ISL_749351, EPI_ISL_749352, EPI_ISL_749353, EPI_ISL_749354, EPI_ISL_749355, EPI_ISL_749357, EPI_ISL_749379, EPI_ISL_749380, EPI_ISL_749381, EPI_ISL_749382, EPI_ISL_749383, EPI_ISL_749384, EPI_ISL_749386, EPI_ISL_749388, EPI_ISL_749389, EPI_ISL_749390, EPI_ISL_749391, EPI_ISL_749392, EPI_ISL_749394, EPI_ISL_749395, EPI_ISL_749396, EPI_ISL_749397, EPI_ISL_749398, EPI_ISL_749399, EPI_ISL_749400, EPI_ISL_749401, EPI_ISL_749402, EPI_ISL_749403, EPI_ISL_749404, EPI_ISL_749406, EPI_ISL_749408, EPI_ISL_749409, EPI_ISL_749411, EPI_ISL_749412, EPI_ISL_749413, EPI_ISL_749414, EPI_ISL_749415, EPI_ISL_749418, EPI_ISL_749419, EPI_ISL_749420, EPI_ISL_749422, EPI_ISL_749423, EPI_ISL_749424, EPI_ISL_749426, EPI_ISL_749427, EPI_ISL_749428, EPI_ISL_749430, EPI_ISL_749431, EPI_ISL_749432, EPI_ISL_749433, EPI_ISL_749434, EPI_ISL_749435, EPI_ISL_749437, EPI_ISL_749438, EPI_ISL_749439, EPI_ISL_749440, EPI_ISL_749441, EPI_ISL_749442, EPI_ISL_749443, EPI_ISL_749444, EPI_ISL_749445, EPI_ISL_749446, EPI_ISL_749447, EPI_ISL_749448, EPI_ISL_749450, EPI_ISL_749451, EPI_ISL_749452, EPI_ISL_749453, EPI_ISL_749454, EPI_ISL_749455, EPI_ISL_749456, EPI_ISL_749458, EPI_ISL_749459, EPI_ISL_749460, EPI_ISL_749461, EPI_ISL_749462, EPI_ISL_749463, EPI_ISL_749464, EPI_ISL_749465, EPI_ISL_749466, EPI_ISL_749467, EPI_ISL_749468, EPI_ISL_749469, EPI_ISL_749470, EPI_ISL_749471, EPI_ISL_749472, EPI_ISL_749473, EPI_ISL_749475, EPI_ISL_749476, EPI_ISL_749477, EPI_ISL_749478, EPI_ISL_749479, EPI_ISL_749480, EPI_ISL_749481, EPI_ISL_749483, EPI_ISL_749494, EPI_ISL_749495, EPI_ISL_749496, EPI_ISL_749522, EPI_ISL_749542, EPI_ISL_749544, EPI_ISL_749545, EPI_ISL_749546, EPI_ISL_749547, EPI_ISL_749549, EPI_ISL_749550, EPI_ISL_749551, EPI_ISL_749552, EPI_ISL_749553, EPI_ISL_749556, EPI_ISL_749557, EPI_ISL_749558, EPI_ISL_749559, EPI_ISL_749560, EPI_ISL_749561, EPI_ISL_749562, EPI_ISL_749564, EPI_ISL_749565, EPI_ISL_749566, EPI_ISL_749567, EPI_ISL_749568, EPI_ISL_749569, EPI_ISL_749570, EPI_ISL_749571, EPI_ISL_749572, EPI_ISL_749573, EPI_ISL_749574, EPI_ISL_749575, EPI_ISL_749576, EPI_ISL_749577, EPI_ISL_749578, EPI_ISL_749579, EPI_ISL_749581, EPI_ISL_749582, EPI_ISL_749583, EPI_ISL_749584, EPI_ISL_749585, EPI_ISL_749586, EPI_ISL_749587, EPI_ISL_749588, EPI_ISL_749589, EPI_ISL_749590, EPI_ISL_749591, EPI_ISL_749592, EPI_ISL_749593, EPI_ISL_749594, EPI_ISL_749596, EPI_ISL_749597, EPI_ISL_749598, EPI_ISL_749599, EPI_ISL_749600, EPI_ISL_749602, EPI_ISL_749603, EPI_ISL_749604, EPI_ISL_749605, EPI_ISL_749606, EPI_ISL_749607, EPI_ISL_749608, EPI_ISL_749609, EPI_ISL_749610, EPI_ISL_749611, EPI_ISL_749612, EPI_ISL_749613, EPI_ISL_749616, EPI_ISL_749617, EPI_ISL_749618, EPI_ISL_749619, EPI_ISL_749620, EPI_ISL_749621, EPI_ISL_749623, EPI_ISL_749624, EPI_ISL_749625, EPI_ISL_749626, EPI_ISL_749627, EPI_ISL_749628, EPI_ISL_749629, EPI_ISL_749630, EPI_ISL_749631, EPI_ISL_749632, EPI_ISL_749633, EPI_ISL_749634, EPI_ISL_749635, EPI_ISL_749637, EPI_ISL_749639, EPI_ISL_749640, EPI_ISL_749641, EPI_ISL_749642, EPI_ISL_749644, EPI_ISL_749645, EPI_ISL_749648, EPI_ISL_749649, EPI_ISL_749651, EPI_ISL_749652, EPI_ISL_749654, EPI_ISL_749655, EPI_ISL_749656, EPI_ISL_749657, EPI_ISL_749658, EPI_ISL_749659, EPI_ISL_749660, EPI_ISL_749661, EPI_ISL_749662, EPI_ISL_749663, EPI_ISL_749664, EPI_ISL_749665, EPI_ISL_749666, EPI_ISL_749667, EPI_ISL_749668, EPI_ISL_749669, EPI_ISL_749670, EPI_ISL_749671, EPI_ISL_749672, EPI_ISL_749673, EPI_ISL_749674, EPI_ISL_749675, EPI_ISL_749676, EPI_ISL_749677, EPI_ISL_749678, EPI_ISL_749679, EPI_ISL_749680, EPI_ISL_749681, EPI_ISL_749682, EPI_ISL_749683, EPI_ISL_749684, EPI_ISL_749685, EPI_ISL_749686, EPI_ISL_749687, EPI_ISL_749688, EPI_ISL_749690, EPI_ISL_749691, EPI_ISL_749692, EPI_ISL_749693, EPI_ISL_749694, EPI_ISL_749695, EPI_ISL_749696, EPI_ISL_749697, EPI_ISL_749698, EPI_ISL_749699, EPI_ISL_749700, EPI_ISL_749701, EPI_ISL_749702, EPI_ISL_749703, EPI_ISL_749704, EPI_ISL_749705, EPI_ISL_749707, EPI_ISL_749708, EPI_ISL_749709, EPI_ISL_749710, EPI_ISL_749711, EPI_ISL_749712, EPI_ISL_749713, EPI_ISL_749714, EPI_ISL_749715, EPI_ISL_749716, EPI_ISL_749717, EPI_ISL_749718, EPI_ISL_749719, EPI_ISL_749720, EPI_ISL_749721, EPI_ISL_749722, EPI_ISL_749723, EPI_ISL_749725, EPI_ISL_749726, EPI_ISL_749727, EPI_ISL_749728, EPI_ISL_749729, EPI_ISL_749730, EPI_ISL_749731, EPI_ISL_749732, EPI_ISL_749734, EPI_ISL_749735, EPI_ISL_749737, EPI_ISL_749739, EPI_ISL_749740, EPI_ISL_749741, EPI_ISL_749742, EPI_ISL_749743, EPI_ISL_749744, EPI_ISL_749745, EPI_ISL_749746, EPI_ISL_749747, EPI_ISL_749748, EPI_ISL_749750, EPI_ISL_749751, EPI_ISL_749752, EPI_ISL_749754, EPI_ISL_749755, EPI_ISL_749756, EPI_ISL_749757, EPI_ISL_749758, EPI_ISL_749759, EPI_ISL_749760, EPI_ISL_749761, EPI_ISL_749762, EPI_ISL_749763, EPI_ISL_749764, EPI_ISL_749765, EPI_ISL_749766, EPI_ISL_749767, EPI_ISL_749768, EPI_ISL_749769, EPI_ISL_749770, EPI_ISL_749771, EPI_ISL_749772, EPI_ISL_749773, EPI_ISL_749774, EPI_ISL_749776, EPI_ISL_749777, EPI_ISL_749778, EPI_ISL_749779, EPI_ISL_749780, EPI_ISL_749781, EPI_ISL_749782, EPI_ISL_749783, EPI_ISL_749784, EPI_ISL_749785, EPI_ISL_749787, EPI_ISL_749788, EPI_ISL_749789, EPI_ISL_749790, EPI_ISL_749791, EPI_ISL_749792, EPI_ISL_749793, EPI_ISL_749794, EPI_ISL_749795, EPI_ISL_749796, EPI_ISL_749797, EPI_ISL_749798, EPI_ISL_749799, EPI_ISL_749801, EPI_ISL_749802, EPI_ISL_749803, EPI_ISL_749804, EPI_ISL_749805, EPI_ISL_749806, EPI_ISL_749807, EPI_ISL_749808, EPI_ISL_749809, EPI_ISL_749810, EPI_ISL_749811, EPI_ISL_749812, EPI_ISL_749814, EPI_ISL_749816, EPI_ISL_749818, EPI_ISL_749819, EPI_ISL_749820, EPI_ISL_749821, EPI_ISL_749822, EPI_ISL_749823, EPI_ISL_749824, EPI_ISL_749825, EPI_ISL_749826, EPI_ISL_749827, EPI_ISL_749828, EPI_ISL_749829, EPI_ISL_749830, EPI_ISL_749831, EPI_ISL_749832, EPI_ISL_749833, EPI_ISL_749834, EPI_ISL_749835, EPI_ISL_749836, EPI_ISL_749837, EPI_ISL_749838, EPI_ISL_749839, EPI_ISL_749840, EPI_ISL_749841, EPI_ISL_749842, EPI_ISL_749843, EPI_ISL_749845, EPI_ISL_749846, EPI_ISL_749847, EPI_ISL_749848, EPI_ISL_749849, EPI_ISL_749850, EPI_ISL_749852, EPI_ISL_749853, EPI_ISL_749854, EPI_ISL_749855, EPI_ISL_749857, EPI_ISL_749858, EPI_ISL_749859, EPI_ISL_749860, EPI_ISL_749861, EPI_ISL_749862, EPI_ISL_749863, EPI_ISL_749864, EPI_ISL_749865, EPI_ISL_749866, EPI_ISL_749867, EPI_ISL_749868, EPI_ISL_749869, EPI_ISL_749870, EPI_ISL_749871, EPI_ISL_749872, EPI_ISL_749873, EPI_ISL_749874, EPI_ISL_749875, EPI_ISL_749876, EPI_ISL_749877, EPI_ISL_749878, EPI_ISL_749879, EPI_ISL_749880, EPI_ISL_749881, EPI_ISL_749882, EPI_ISL_749883, EPI_ISL_749884, EPI_ISL_749885, EPI_ISL_749886, EPI_ISL_749887, EPI_ISL_749888, EPI_ISL_749889, EPI_ISL_749890, EPI_ISL_749891, EPI_ISL_749892, EPI_ISL_749893, EPI_ISL_749894, EPI_ISL_749895, EPI_ISL_749896, EPI_ISL_749898, EPI_ISL_749899, EPI_ISL_749900, EPI_ISL_749901, EPI_ISL_749902, EPI_ISL_749903, EPI_ISL_749904, EPI_ISL_749905, EPI_ISL_749907, EPI_ISL_749909, EPI_ISL_749910, EPI_ISL_749911, EPI_ISL_749912, EPI_ISL_749913, EPI_ISL_749914, EPI_ISL_749915, EPI_ISL_749916, EPI_ISL_749917, EPI_ISL_749918, EPI_ISL_749919, EPI_ISL_749920, EPI_ISL_749921, EPI_ISL_749922, EPI_ISL_749923, EPI_ISL_749925, EPI_ISL_749926, EPI_ISL_749927, EPI_ISL_749928, EPI_ISL_749929, EPI_ISL_749930, EPI_ISL_749931,			

EPI_ISL_749932, EPI_ISL_749933, EPI_ISL_749934, EPI_ISL_749935, EPI_ISL_749936, EPI_ISL_749937, EPI_ISL_749938, EPI_ISL_749939, EPI_ISL_749940, EPI_ISL_749941, EPI_ISL_749942, EPI_ISL_749943, EPI_ISL_749944, EPI_ISL_749945, EPI_ISL_749946, EPI_ISL_749947, EPI_ISL_749949, EPI_ISL_749950, EPI_ISL_749951, EPI_ISL_749952, EPI_ISL_749953, EPI_ISL_749955, EPI_ISL_749956, EPI_ISL_749957, EPI_ISL_749958, EPI_ISL_749959, EPI_ISL_749960, EPI_ISL_749961, EPI_ISL_749962, EPI_ISL_749963, EPI_ISL_749964, EPI_ISL_749965, EPI_ISL_749966, EPI_ISL_749967, EPI_ISL_749968, EPI_ISL_749969, EPI_ISL_749970, EPI_ISL_749971, EPI_ISL_749972, EPI_ISL_749973, EPI_ISL_749974, EPI_ISL_749975, EPI_ISL_749976, EPI_ISL_749977, EPI_ISL_749978, EPI_ISL_749980, EPI_ISL_749981, EPI_ISL_749982, EPI_ISL_749983, EPI_ISL_749984, EPI_ISL_749985, EPI_ISL_749986, EPI_ISL_749987, EPI_ISL_749988, EPI_ISL_749989, EPI_ISL_749990, EPI_ISL_749991, EPI_ISL_749992, EPI_ISL_749993, EPI_ISL_749994, EPI_ISL_749995, EPI_ISL_749997, EPI_ISL_749998, EPI_ISL_749999, EPI_ISL_750000, EPI_ISL_750001, EPI_ISL_750002, EPI_ISL_750003, EPI_ISL_750004, EPI_ISL_750005, EPI_ISL_750006, EPI_ISL_750007, EPI_ISL_750008, EPI_ISL_750009, EPI_ISL_750010, EPI_ISL_750011, EPI_ISL_750012, EPI_ISL_750013, EPI_ISL_750014, EPI_ISL_750015, EPI_ISL_750016, EPI_ISL_750017, EPI_ISL_750018, EPI_ISL_750019, EPI_ISL_750020, EPI_ISL_750022, EPI_ISL_750023, EPI_ISL_750024, EPI_ISL_750026, EPI_ISL_750027, EPI_ISL_750029, EPI_ISL_750030, EPI_ISL_750031, EPI_ISL_750032, EPI_ISL_750033, EPI_ISL_750034, EPI_ISL_750035, EPI_ISL_750036, EPI_ISL_750037, EPI_ISL_750038, EPI_ISL_750039, EPI_ISL_750040, EPI_ISL_750041, EPI_ISL_750042, EPI_ISL_750043, EPI_ISL_750044, EPI_ISL_750045, EPI_ISL_750047, EPI_ISL_750048, EPI_ISL_750049, EPI_ISL_750050, EPI_ISL_750051, EPI_ISL_750052, EPI_ISL_750053, EPI_ISL_750054, EPI_ISL_750056, EPI_ISL_750057, EPI_ISL_750058, EPI_ISL_750059, EPI_ISL_750060, EPI_ISL_750061, EPI_ISL_750062, EPI_ISL_750063, EPI_ISL_750064, EPI_ISL_750065, EPI_ISL_750066, EPI_ISL_750067, EPI_ISL_750068, EPI_ISL_750069, EPI_ISL_750070, EPI_ISL_750071, EPI_ISL_750072, EPI_ISL_750073, EPI_ISL_750074, EPI_ISL_750075, EPI_ISL_750076, EPI_ISL_750077, EPI_ISL_750078, EPI_ISL_750079, EPI_ISL_750080, EPI_ISL_750081, EPI_ISL_750082, EPI_ISL_750083, EPI_ISL_750084, EPI_ISL_750085, EPI_ISL_750086, EPI_ISL_750087, EPI_ISL_750088, EPI_ISL_750089, EPI_ISL_750090, EPI_ISL_750091, EPI_ISL_750092, EPI_ISL_750093, EPI_ISL_750094, EPI_ISL_750095, EPI_ISL_750096, EPI_ISL_750097, EPI_ISL_750098, EPI_ISL_750100, EPI_ISL_750101, EPI_ISL_750102, EPI_ISL_750103, EPI_ISL_750104, EPI_ISL_750105, EPI_ISL_750106, EPI_ISL_750107, EPI_ISL_750109, EPI_ISL_750110, EPI_ISL_750111, EPI_ISL_750112, EPI_ISL_750113, EPI_ISL_750114, EPI_ISL_750115, EPI_ISL_750116, EPI_ISL_750117, EPI_ISL_750118, EPI_ISL_750119, EPI_ISL_750120, EPI_ISL_750121, EPI_ISL_750122, EPI_ISL_750123, EPI_ISL_750124, EPI_ISL_750126, EPI_ISL_750127, EPI_ISL_750128, EPI_ISL_750129, EPI_ISL_750130, EPI_ISL_750131, EPI_ISL_750132, EPI_ISL_750133, EPI_ISL_750134, EPI_ISL_750135, EPI_ISL_750136, EPI_ISL_750137, EPI_ISL_750138, EPI_ISL_750139, EPI_ISL_750140, EPI_ISL_750141, EPI_ISL_750142, EPI_ISL_750143, EPI_ISL_750144, EPI_ISL_750145, EPI_ISL_750146, EPI_ISL_750147, EPI_ISL_750149, EPI_ISL_750150, EPI_ISL_750152, EPI_ISL_750153, EPI_ISL_750154, EPI_ISL_750155, EPI_ISL_750156, EPI_ISL_750157, EPI_ISL_750158, EPI_ISL_750159, EPI_ISL_750160, EPI_ISL_750287, EPI_ISL_750288, EPI_ISL_750289, EPI_ISL_750290, EPI_ISL_750291, EPI_ISL_750292, EPI_ISL_750293, EPI_ISL_750294, EPI_ISL_750295, EPI_ISL_750296, EPI_ISL_750297, EPI_ISL_750298, EPI_ISL_750299, EPI_ISL_750300, EPI_ISL_750301, EPI_ISL_750302, EPI_ISL_750303, EPI_ISL_750305, EPI_ISL_750306, EPI_ISL_750307, EPI_ISL_750308, EPI_ISL_750309, EPI_ISL_750310, EPI_ISL_750311, EPI_ISL_750313, EPI_ISL_750314, EPI_ISL_750315, EPI_ISL_750316, EPI_ISL_750317, EPI_ISL_750318, EPI_ISL_750319, EPI_ISL_750320, EPI_ISL_750321, EPI_ISL_750322, EPI_ISL_750323, EPI_ISL_750324, EPI_ISL_750325, EPI_ISL_750326, EPI_ISL_750328, EPI_ISL_750329, EPI_ISL_750330, EPI_ISL_750331, EPI_ISL_750332, EPI_ISL_750333, EPI_ISL_750334, EPI_ISL_750335, EPI_ISL_750337, EPI_ISL_750339, EPI_ISL_750340, EPI_ISL_750341, EPI_ISL_750342, EPI_ISL_750343, EPI_ISL_750344, EPI_ISL_750345, EPI_ISL_750346, EPI_ISL_750347, EPI_ISL_750348, EPI_ISL_750349, EPI_ISL_750350, EPI_ISL_750351, EPI_ISL_750352, EPI_ISL_750353, EPI_ISL_750354, EPI_ISL_750355, EPI_ISL_750356, EPI_ISL_750357, EPI_ISL_750358, EPI_ISL_750359, EPI_ISL_750360, EPI_ISL_750362, EPI_ISL_750363, EPI_ISL_750365, EPI_ISL_750366, EPI_ISL_750367, EPI_ISL_750368, EPI_ISL_750371, EPI_ISL_750372, EPI_ISL_750393, EPI_ISL_750394, EPI_ISL_750395, EPI_ISL_750396, EPI_ISL_750397, EPI_ISL_750398, EPI_ISL_750399, EPI_ISL_750400, EPI_ISL_750401, EPI_ISL_750402, EPI_ISL_750403, EPI_ISL_750404, EPI_ISL_750405, EPI_ISL_750406, EPI_ISL_750407, EPI_ISL_750408, EPI_ISL_750409, EPI_ISL_750410, EPI_ISL_750411, EPI_ISL_750413, EPI_ISL_750414, EPI_ISL_750415, EPI_ISL_750416, EPI_ISL_750417, EPI_ISL_750419, EPI_ISL_750420, EPI_ISL_750421, EPI_ISL_750422, EPI_ISL_750423, EPI_ISL_750424, EPI_ISL_750425, EPI_ISL_750426, EPI_ISL_750427, EPI_ISL_750428, EPI_ISL_750429, EPI_ISL_750431, EPI_ISL_750432, EPI_ISL_750433, EPI_ISL_750434, EPI_ISL_750435, EPI_ISL_750437, EPI_ISL_750438, EPI_ISL_750439, EPI_ISL_750440, EPI_ISL_750441, EPI_ISL_750442, EPI_ISL_750443, EPI_ISL_750444, EPI_ISL_750445, EPI_ISL_750446, EPI_ISL_750447, EPI_ISL_750448, EPI_ISL_750449, EPI_ISL_750450, EPI_ISL_750451, EPI_ISL_750452, EPI_ISL_750453, EPI_ISL_750454, EPI_ISL_750455, EPI_ISL_750456, EPI_ISL_750457, EPI_ISL_750458, EPI_ISL_750459, EPI_ISL_750460, EPI_ISL_750461, EPI_ISL_750462, EPI_ISL_750464, EPI_ISL_750466, EPI_ISL_750467, EPI_ISL_750468, EPI_ISL_750469, EPI_ISL_750470, EPI_ISL_750471, EPI_ISL_750472, EPI_ISL_750473, EPI_ISL_750474, EPI_ISL_750475, EPI_ISL_750476, EPI_ISL_750477, EPI_ISL_750479, EPI_ISL_750480, EPI_ISL_750481, EPI_ISL_750483, EPI_ISL_750484, EPI_ISL_750485, EPI_ISL_750494, EPI_ISL_750495, EPI_ISL_750497, EPI_ISL_750503, EPI_ISL_750512, EPI_ISL_750537, EPI_ISL_750538, EPI_ISL_750539, EPI_ISL_750540, EPI_ISL_750541, EPI_ISL_750542, EPI_ISL_750543, EPI_ISL_750544, EPI_ISL_750545, EPI_ISL_750546, EPI_ISL_750548, EPI_ISL_750549, EPI_ISL_750550, EPI_ISL_750551, EPI_ISL_750552, EPI_ISL_750553, EPI_ISL_750554, EPI_ISL_750555, EPI_ISL_750556, EPI_ISL_750557, EPI_ISL_750558, EPI_ISL_750559, EPI_ISL_750560, EPI_ISL_750561, EPI_ISL_750562, EPI_ISL_750563, EPI_ISL_750564, EPI_ISL_750565, EPI_ISL_750566, EPI_ISL_750567, EPI_ISL_750568, EPI_ISL_750569, EPI_ISL_750570, EPI_ISL_750571, EPI_ISL_750572, EPI_ISL_750573, EPI_ISL_750574, EPI_ISL_750576, EPI_ISL_750577, EPI_ISL_750578, EPI_ISL_750579, EPI_ISL_750580, EPI_ISL_750583, EPI_ISL_750584, EPI_ISL_750585, EPI_ISL_750586, EPI_ISL_750587, EPI_ISL_750588, EPI_ISL_750589, EPI_ISL_750590, EPI_ISL_750591, EPI_ISL_750592, EPI_ISL_750593, EPI_ISL_750594, EPI_ISL_750595, EPI_ISL_750596, EPI_ISL_750597, EPI_ISL_750598, EPI_ISL_750599, EPI_ISL_750600, EPI_ISL_750601, EPI_ISL_750602, EPI_ISL_750603, EPI_ISL_750605, EPI_ISL_750606, EPI_ISL_750607, EPI_ISL_750608, EPI_ISL_750609, EPI_ISL_750610, EPI_ISL_750611, EPI_ISL_750612, EPI_ISL_750613, EPI_ISL_750614, EPI_ISL_750615, EPI_ISL_750616, EPI_ISL_750617, EPI_ISL_750619, EPI_ISL_750620, EPI_ISL_750621, EPI_ISL_750622, EPI_ISL_750623, EPI_ISL_750624, EPI_ISL_750625, EPI_ISL_750628, EPI_ISL_750629, EPI_ISL_750630, EPI_ISL_750631, EPI_ISL_750632, EPI_ISL_750633, EPI_ISL_750634, EPI_ISL_750635, EPI_ISL_750636, EPI_ISL_750637, EPI_ISL_750638, EPI_ISL_750639, EPI_ISL_750640, EPI_ISL_750641, EPI_ISL_750642, EPI_ISL_750643, EPI_ISL_750645, EPI_ISL_750646, EPI_ISL_750647, EPI_ISL_750648, EPI_ISL_750649, EPI_ISL_750651, EPI_ISL_750652, EPI_ISL_750653, EPI_ISL_750654, EPI_ISL_750655, EPI_ISL_750656, EPI_ISL_750657, EPI_ISL_750658, EPI_ISL_750661, EPI_ISL_750662, EPI_ISL_750663, EPI_ISL_750664, EPI_ISL_750666, EPI_ISL_750667, EPI_ISL_750668, EPI_ISL_750669, EPI_ISL_750671, EPI_ISL_750673, EPI_ISL_750674, EPI_ISL_750675, EPI_ISL_750676, EPI_ISL_750677, EPI_ISL_750678, EPI_ISL_750679, EPI_ISL_750680, EPI_ISL_750682, EPI_ISL_750684, EPI_ISL_750685, EPI_ISL_750686, EPI_ISL_750687, EPI_ISL_750688, EPI_ISL_750689, EPI_ISL_750690, EPI_ISL_750691, EPI_ISL_750692, EPI_ISL_750693, EPI_ISL_750695, EPI_ISL_750696, EPI_ISL_750697, EPI_ISL_750698, EPI_ISL_750699, EPI_ISL_750700, EPI_ISL_750701, EPI_ISL_750702, EPI_ISL_750704, EPI_ISL_750705, EPI_ISL_750706, EPI_ISL_750707, EPI_ISL_750708, EPI_ISL_750709, EPI_ISL_750710, EPI_ISL_750711, EPI_ISL_750712, EPI_ISL_750713, EPI_ISL_750714, EPI_ISL_750715, EPI_ISL_750716, EPI_ISL_750717, EPI_ISL_750718, EPI_ISL_750720, EPI_ISL_750721, EPI_ISL_750722, EPI_ISL_750723, EPI_ISL_750725, EPI_ISL_750726, EPI_ISL_750727, EPI_ISL_750728, EPI_ISL_750729, EPI_ISL_750730, EPI_ISL_750731, EPI_ISL_750733, EPI_ISL_750734, EPI_ISL_750735, EPI_ISL_750736, EPI_ISL_750737, EPI_ISL_750738, EPI_ISL_750739, EPI_ISL_750740, EPI_ISL_750741, EPI_ISL_750742, EPI_ISL_750743, EPI_ISL_750744, EPI_ISL_750745, EPI_ISL_750746, EPI_ISL_750747, EPI_ISL_750748, EPI_ISL_750749, EPI_ISL_750750, EPI_ISL_750751, EPI_ISL_750752, EPI_ISL_750753, EPI_ISL_750754, EPI_ISL_750755, EPI_ISL_750756, EPI_ISL_750757, EPI_ISL_750758, EPI_ISL_750759, EPI_ISL_750760, EPI_ISL_750761, EPI_ISL_750762, EPI_ISL_750763, EPI_ISL_750765, EPI_ISL_750766, EPI_ISL_750767, EPI_ISL_750768, EPI_ISL_750769, EPI_ISL_750770, EPI_ISL_750771, EPI_ISL_750772, EPI_ISL_750773, EPI_ISL_750774, EPI_ISL_750775, EPI_ISL_750776, EPI_ISL_750777, EPI_ISL_750778, EPI_ISL_750779, EPI_ISL_750780, EPI_ISL_750781, EPI_ISL_750782, EPI_ISL_750783, EPI_ISL_750784, EPI_ISL_750785, EPI_ISL_750786, EPI_ISL_750787, EPI_ISL_750788, EPI_ISL_750789, EPI_ISL_750790, EPI_ISL_750792, EPI_ISL_750793, EPI_ISL_750794, EPI_ISL_750795, EPI_ISL_750796, EPI_ISL_750797, EPI_ISL_750798, EPI_ISL_750799, EPI_ISL_750800, EPI_ISL_750801, EPI_ISL_750802, EPI_ISL_750803, EPI_ISL_750804, EPI_ISL_750806, EPI_ISL_750807, EPI_ISL_750808, EPI_ISL_750809, EPI_ISL_750810, EPI_ISL_750812, EPI_ISL_750813, EPI_ISL_750814, EPI_ISL_750815, EPI_ISL_750816, EPI_ISL_750817, EPI_ISL_750818, EPI_ISL_750819, EPI_ISL_750821, EPI_ISL_750822, EPI_ISL_750823, EPI_ISL_750824, EPI_ISL_750825, EPI_ISL_750826, EPI_ISL_750827, EPI_ISL_750829, EPI_ISL_750830, EPI_ISL_750831, EPI_ISL_750832, EPI_ISL_750833, EPI_ISL_750834, EPI_ISL_750835, EPI_ISL_750837, EPI_ISL_750838, EPI_ISL_750839, EPI_ISL_750840, EPI_ISL_750841, EPI_ISL_750842, EPI_ISL_750843, EPI_ISL_750844, EPI_ISL_750845, EPI_ISL_750846, EPI_ISL_750847, EPI_ISL_750848, EPI_ISL_750849, EPI_ISL_750850, EPI_ISL_750851, EPI_ISL_750852, EPI_ISL_750853, EPI_ISL_750854, EPI_ISL_750855, EPI_ISL_750856, EPI_ISL_750857, EPI_ISL_750858, EPI_ISL_750859, EPI_ISL_750860, EPI_ISL_750861, EPI_ISL_750862, EPI_ISL_750863, EPI_ISL_750864, EPI_ISL_750865, EPI_ISL_750866, EPI_ISL_750867, EPI_ISL_750868, EPI_ISL_750869, EPI_ISL_750870, EPI_ISL_750871, EPI_ISL_750872, EPI_ISL_750873, EPI_ISL_750874, EPI_ISL_750875, EPI_ISL_750876, EPI_ISL_750877, EPI_ISL_750878, EPI_ISL_750880, EPI_ISL_750881, EPI_ISL_750882, EPI_ISL_750883, EPI_ISL_750884, EPI_ISL_750885, EPI_ISL_750886, EPI_ISL_750887, EPI_ISL_750888, EPI_ISL_750889, EPI_ISL_750890, EPI_ISL_750891, EPI_ISL_750892, EPI_ISL_750893, EPI_ISL_750894, EPI_ISL_750895, EPI_ISL_750896, EPI_ISL_750897, EPI_ISL_750898, EPI_ISL_750900, EPI_ISL_750901, EPI_ISL_750903, EPI_ISL_750904, EPI_ISL_750905, EPI_ISL_750906, EPI_ISL_750907, EPI_ISL_750908, EPI_ISL_750909, EPI_ISL_750910, EPI_ISL_750911, EPI_ISL_750912, EPI_ISL_750913, EPI_ISL_750914, EPI_ISL_750915, EPI_ISL_750916, EPI_ISL_750917, EPI_ISL_750919, EPI_ISL_750920, EPI_ISL_750921, EPI_ISL_750922, EPI_ISL_750923, EPI_ISL_750924, EPI_ISL_750925, EPI_ISL_750926, EPI_ISL_750927, EPI_ISL_750928, EPI_ISL_750929, EPI_ISL_750930, EPI_ISL_750931, EPI_ISL_750932, EPI_ISL_750933, EPI_ISL_750934, EPI_ISL_750935, EPI_ISL_750936, EPI_ISL_750937, EPI_ISL_750938, EPI_ISL_750939, EPI_ISL_750940, EPI_ISL_750941, EPI_ISL_750942, EPI_ISL_750943, EPI_ISL_750944, EPI_ISL_750945, EPI_ISL_750946, EPI_ISL_750947, EPI_ISL_750948, EPI_ISL_750950, EPI_ISL_750951, EPI_ISL_750952, EPI_ISL_750953, EPI_ISL_750954, EPI_ISL_750955, EPI_ISL_750956, EPI_ISL_750957, EPI_ISL_750958, EPI_ISL_750959, EPI_ISL_750960, EPI_ISL_750961, EPI_ISL_750962, EPI_ISL_750963, EPI_ISL_750964, EPI_ISL_750965, EPI_ISL_750966, EPI_ISL_750967, EPI_ISL_750968, EPI_ISL_750969, EPI_ISL_750970, EPI_ISL_750971, EPI_ISL_750972, EPI_ISL_750973, EPI_ISL_750974, EPI_ISL_750975, EPI_ISL_750976, EPI_ISL_750977, EPI_ISL_750978, EPI_ISL_750980, EPI_ISL_750981, EPI_ISL_750982, EPI_ISL_750984, EPI_ISL_750985, EPI_ISL_750987, EPI_ISL_750988, EPI_ISL_750989, EPI_ISL_750991, EPI_ISL_750992, EPI_ISL_750993, EPI_ISL_750994, EPI_ISL_750995, EPI_ISL_750996, EPI_ISL_750997, EPI_ISL_750998, EPI_ISL_750999, EPI_ISL_751000, EPI_ISL_751001, EPI_ISL_751002, EPI_ISL_751003, EPI_ISL_751005, EPI_ISL_751006, EPI_ISL_751007, EPI_ISL_751008, EPI_ISL_751009, EPI_ISL_751010, EPI_ISL_751012, EPI_ISL_751013, EPI_ISL_751014, EPI_ISL_751015, EPI_ISL_751016, EPI_ISL_751017, EPI_ISL_751018, EPI_ISL_751019, EPI_ISL_751020, EPI_ISL_751021, EPI_ISL_751022, EPI_ISL_751024, EPI_ISL_751025, EPI_ISL_751026, EPI_ISL_751028, EPI_ISL_751029, EPI_ISL_751030, EPI_ISL_751031, EPI_ISL_751032, EPI_ISL_751033, EPI_ISL_751035, EPI_ISL_751036, EPI_ISL_751037, EPI_ISL_751038, EPI_ISL_751039, EPI_ISL_751041, EPI_ISL_751042, EPI_ISL_751043, EPI_ISL_751044, EPI_ISL_751045, EPI_ISL_751046, EPI_ISL_751047, EPI_ISL_751048, EPI_ISL_751049, EPI_ISL_751050, EPI_ISL_751051, EPI_ISL_751052, EPI_ISL_751053, EPI_ISL_751054, EPI_ISL_751055, EPI_ISL_751056, EPI_ISL_751057, EPI_ISL_751061, EPI_ISL_751062, EPI_ISL_751063, EPI_ISL_751064, EPI_ISL_751065, EPI_ISL_751066, EPI_ISL_751067, EPI_ISL_751068, EPI_ISL_751069, EPI_ISL_751070, EPI_ISL_751071, EPI_ISL_751072, EPI_ISL_751073, EPI_ISL_751075, EPI_ISL_751076, EPI_ISL_751077, EPI_ISL_751078, EPI_ISL_751079, EPI_ISL_751080, EPI_ISL_751081, EPI_ISL_751083, EPI_ISL_751084, EPI_ISL_751085, EPI_ISL_751086, EPI_ISL_751087, EPI_ISL_751088, EPI_ISL_751090, EPI_ISL_751091, EPI_ISL_751092, EPI_ISL_751093, EPI_ISL_751094, EPI_ISL_751095, EPI_ISL_751096, EPI_ISL_751097, EPI_ISL_751098, EPI_ISL_751099, EPI_ISL_751100, EPI_ISL_751101, EPI_ISL_751102, EPI_ISL_751103, EPI_ISL_751104, EPI_ISL_751105, EPI_ISL_751106, EPI_ISL_751107, EPI_ISL_751108, EPI_ISL_751109, EPI_ISL_751110, EPI_ISL_751111, EPI_ISL_751112, EPI_ISL_751113, EPI_ISL_751114, EPI_ISL_751115, EPI_ISL_751116, EPI_ISL_751117, EPI_ISL_751118, EPI_ISL_751119, EPI_ISL_751120, EPI_ISL_751121, EPI_ISL_751122, EPI_ISL_751123, EPI_ISL_751125, EPI_ISL_751126, EPI_ISL_751127, EPI_ISL_751128, EPI_ISL_751129, EPI_ISL_751130, EPI_ISL_751131, EPI_ISL_751132, EPI_ISL_751133, EPI_ISL_751134, EPI_ISL_751136, EPI_ISL_751137, EPI_ISL_751138, EPI_ISL_751139, EPI_ISL_751140, EPI_ISL_751141, EPI_ISL_751142, EPI_ISL_751144, EPI_ISL_751145, EPI_ISL_751146, EPI_ISL_751147, EPI_ISL_751149, EPI_ISL_751150, EPI_ISL_751152, EPI_ISL_751155, EPI_ISL_751157, EPI_ISL_751158, EPI_ISL_751160, EPI_ISL_751161, EPI_ISL_751162, EPI_ISL_751163, EPI_ISL_751166, EPI_ISL_751167, EPI_ISL_751168, EPI_ISL_751169, EPI_ISL_751170, EPI_ISL_751171, EPI_ISL_751172, EPI_ISL_751173, EPI_ISL_751174, EPI_ISL_751176, EPI_ISL_751177, EPI_ISL_751178, EPI_ISL_751179, EPI_ISL_751181, EPI_ISL_751182, EPI_ISL_751183

see above	Department of Virus and Microbiological Special Diagnostics, Statens Serum Institut, Copenhagen, Denmark	Albertsen Lab, Department of Chemistry and Bioscience, Aalborg University, Denmark	Danish Covid-19 Genome Consortium
EPI_ISL_751192	Limmattal Hospital	Institute of Medical Virology, University of Zurich	Stefan Schmutz, Kevin Steiner, Verena Kufner, Maryam Zaheri, Gabriela Ziltener, Jürg Böni, Michael Huber, Alexandra Trkola, Roberto Buonomano
EPI_ISL_751196, EPI_ISL_751197, EPI_ISL_751199, EPI_ISL_751200	Dienststelle Gesundheit und Sport Kanton Luzern	Institute of Medical Virology, University of Zurich	Stefan Schmutz, Kevin Steiner, Verena Kufner, Maryam Zaheri, Gabriela Ziltener, Jürg Böni, Michael Huber, Alexandra Trkola, Claudia Schmutz, Eva Spieler
EPI_ISL_751555	WY Public Health Laboratory	Genomics and Discovery, Respiratory Viruses Branch, Division of Viral Diseases, Centers for Disease Control and Prevention	Krista Queen, Yan Li, Ying Tao, Jing Zhang, Anna Uehara, Anna Montmayeur, Clinton R. Paden, Peter W. Cook,Rachel Marine, Mili Sheth, Haibin Wang, Justin Lee, Suxiang Tong
EPI_ISL_751557	NV State Public Health Laboratory	Genomics and Discovery, Respiratory Viruses Branch, Division of Viral Diseases, Centers for Disease Control and Prevention	Krista Queen, Yan Li, Ying Tao, Jing Zhang, Anna Uehara, Anna Montmayeur, Clinton R. Paden, Peter W. Cook,Rachel Marine, Mili Sheth, Haibin Wang, Justin Lee, Suxiang Tong
EPI_ISL_751570	KY State Public Health Lab	Genomics and Discovery, Respiratory Viruses Branch, Division of Viral Diseases, Centers for Disease Control and Prevention	Krista Queen, Yan Li, Ying Tao, Jing Zhang, Anna Uehara, Anna Montmayeur, Clinton R. Paden, Peter W. Cook,Rachel Marine, Mili Sheth, Haibin Wang, Justin Lee, Suxiang Tong
EPI_ISL_751589	WVDHHR - Office of Laboratory Services	Genomics and Discovery, Respiratory Viruses Branch, Division of Viral Diseases, Centers for Disease Control and Prevention	Krista Queen, Yan Li, Ying Tao, Jing Zhang, Anna Uehara, Anna Montmayeur, Clinton R. Paden, Peter W. Cook,Rachel Marine, Mili Sheth, Haibin Wang, Justin Lee, Suxiang Tong
EPI_ISL_751591	WY Public Health Laboratory	Genomics and Discovery, Respiratory Viruses Branch, Division of Viral Diseases, Centers for Disease Control and Prevention	Krista Queen, Yan Li, Ying Tao, Jing Zhang, Anna Uehara, Anna Montmayeur, Clinton R. Paden, Peter W. Cook,Rachel Marine, Mili Sheth, Haibin Wang, Justin Lee, Suxiang Tong
EPI_ISL_751596	KY State Public Health Lab	Genomics and Discovery, Respiratory Viruses Branch, Division of Viral Diseases, Centers for Disease Control and Prevention	Krista Queen, Yan Li, Ying Tao, Jing Zhang, Anna Uehara, Anna Montmayeur, Clinton R. Paden, Peter W. Cook,Rachel Marine, Mili Sheth, Haibin Wang, Justin Lee, Suxiang Tong
EPI_ISL_751600	IN State Department of Health Laboratory Services	Genomics and Discovery, Respiratory Viruses Branch, Division of Viral Diseases, Centers for Disease Control and Prevention	Krista Queen, Yan Li, Ying Tao, Jing Zhang, Anna Uehara, Anna Montmayeur, Clinton R. Paden, Peter W. Cook,Rachel Marine, Mili Sheth, Haibin Wang, Justin Lee, Suxiang Tong
EPI_ISL_751611	SC Dept of Health and Env. Control-Bureau of Laboratories	Genomics and Discovery, Respiratory Viruses Branch, Division of Viral Diseases, Centers for Disease Control and	Krista Queen, Yan Li, Ying Tao, Jing Zhang, Anna Uehara, Anna Montmayeur, Clinton R. Paden, Peter W. Cook,Rachel Marine, Mili Sheth, Haibin Wang, Justin Lee, Suxiang Tong

		Prevention	
EPI_ISL_751617	TX DSHS, Lab Services Section MC 1947	Genomics and Discovery, Respiratory Viruses Branch, Division of Viral Diseases, Centers for Disease Control and Prevention	Krista Queen, Yan Li, Ying Tao, Jing Zhang, Anna Uehara, Anna Montmayeur, Clinton R. Paden, Peter W. Cook,Rachel Marine, Mili Sheth, Haibin Wang, Justin Lee, Suxiang Tong
EPI_ISL_751619	WY Public Health Laboratory	Genomics and Discovery, Respiratory Viruses Branch, Division of Viral Diseases, Centers for Disease Control and Prevention	Krista Queen, Yan Li, Ying Tao, Jing Zhang, Anna Uehara, Anna Montmayeur, Clinton R. Paden, Peter W. Cook,Rachel Marine, Mili Sheth, Haibin Wang, Justin Lee, Suxiang Tong
EPI_ISL_751626	IN State Department of Health Laboratory Services	Genomics and Discovery, Respiratory Viruses Branch, Division of Viral Diseases, Centers for Disease Control and Prevention	Krista Queen, Yan Li, Ying Tao, Jing Zhang, Anna Uehara, Anna Montmayeur, Clinton R. Paden, Peter W. Cook,Rachel Marine, Mili Sheth, Haibin Wang, Justin Lee, Suxiang Tong
EPI_ISL_751628	MD DOH Laboratories Administration	Genomics and Discovery, Respiratory Viruses Branch, Division of Viral Diseases, Centers for Disease Control and Prevention	Krista Queen, Yan Li, Ying Tao, Jing Zhang, Anna Uehara, Anna Montmayeur, Clinton R. Paden, Peter W. Cook,Rachel Marine, Mili Sheth, Haibin Wang, Justin Lee, Suxiang Tong
EPI_ISL_751630	CO Dept. of Public Health and Environment, Lab Services Division	Genomics and Discovery, Respiratory Viruses Branch, Division of Viral Diseases, Centers for Disease Control and Prevention	Krista Queen, Yan Li, Ying Tao, Jing Zhang, Anna Uehara, Anna Montmayeur, Clinton R. Paden, Peter W. Cook,Rachel Marine, Mili Sheth, Haibin Wang, Justin Lee, Suxiang Tong
EPI_ISL_751632	IN State Department of Health Laboratory Services	Genomics and Discovery, Respiratory Viruses Branch, Division of Viral Diseases, Centers for Disease Control and Prevention	Krista Queen, Yan Li, Ying Tao, Jing Zhang, Anna Uehara, Anna Montmayeur, Clinton R. Paden, Peter W. Cook,Rachel Marine, Mili Sheth, Haibin Wang, Justin Lee, Suxiang Tong
EPI_ISL_751633	MT Public Health Laboratory	Genomics and Discovery, Respiratory Viruses Branch, Division of Viral Diseases, Centers for Disease Control and Prevention	Krista Queen, Yan Li, Ying Tao, Jing Zhang, Anna Uehara, Anna Montmayeur, Clinton R. Paden, Peter W. Cook,Rachel Marine, Mili Sheth, Haibin Wang, Justin Lee, Suxiang Tong
EPI_ISL_751637	NV State Public Health Laboratory	Genomics and Discovery, Respiratory Viruses Branch, Division of Viral Diseases, Centers for Disease Control and Prevention	Krista Queen, Yan Li, Ying Tao, Jing Zhang, Anna Uehara, Anna Montmayeur, Clinton R. Paden, Peter W. Cook,Rachel Marine, Mili Sheth, Haibin Wang, Justin Lee, Suxiang Tong
EPI_ISL_751640	MT Public Health Laboratory	Genomics and Discovery, Respiratory Viruses Branch, Division of Viral Diseases, Centers for Disease Control and Prevention	Krista Queen, Yan Li, Ying Tao, Jing Zhang, Anna Uehara, Anna Montmayeur, Clinton R. Paden, Peter W. Cook,Rachel Marine, Mili Sheth, Haibin Wang, Justin Lee, Suxiang Tong
EPI_ISL_751643	WY Public Health Laboratory	Genomics and Discovery, Respiratory Viruses Branch, Division of Viral Diseases, Centers for Disease Control and Prevention	Krista Queen, Yan Li, Ying Tao, Jing Zhang, Anna Uehara, Anna Montmayeur, Clinton R. Paden, Peter W. Cook,Rachel Marine, Mili Sheth, Haibin Wang, Justin Lee, Suxiang Tong
EPI_ISL_751646	TX DSHS, Lab Services Section MC 1947	Genomics and Discovery, Respiratory Viruses Branch, Division of Viral Diseases, Centers for Disease Control and Prevention	Krista Queen, Yan Li, Ying Tao, Jing Zhang, Anna Uehara, Anna Montmayeur, Clinton R. Paden, Peter W. Cook,Rachel Marine, Mili Sheth, Haibin Wang, Justin Lee, Suxiang Tong
EPI_ISL_751663	WY Public Health Laboratory	Genomics and Discovery, Respiratory Viruses Branch, Division of Viral Diseases, Centers for Disease Control and Prevention	Krista Queen, Yan Li, Ying Tao, Jing Zhang, Anna Uehara, Anna Montmayeur, Clinton R. Paden, Peter W. Cook,Rachel Marine, Mili Sheth, Haibin Wang, Justin Lee, Suxiang Tong
EPI_ISL_751664, EPI_ISL_751665	SC Dept of Health and Env. Control-Bureau of Laboratories	Genomics and Discovery, Respiratory Viruses Branch, Division of Viral Diseases, Centers for Disease Control and Prevention	Krista Queen, Yan Li, Ying Tao, Jing Zhang, Anna Uehara, Anna Montmayeur, Clinton R. Paden, Peter W. Cook,Rachel Marine, Mili Sheth, Haibin Wang, Justin Lee, Suxiang Tong
EPI_ISL_751667	RI State Health Laboratories	Genomics and Discovery, Respiratory Viruses Branch, Division of Viral Diseases, Centers for Disease Control and Prevention	Krista Queen, Yan Li, Ying Tao, Jing Zhang, Anna Uehara, Anna Montmayeur, Clinton R. Paden, Peter W. Cook,Rachel Marine, Mili Sheth, Haibin Wang, Justin Lee, Suxiang Tong
EPI_ISL_751669	LA Office of Public Health Laboratories	Genomics and Discovery, Respiratory Viruses Branch, Division of Viral Diseases, Centers for Disease Control and Prevention	Krista Queen, Yan Li, Ying Tao, Jing Zhang, Anna Uehara, Anna Montmayeur, Clinton R. Paden, Peter W. Cook,Rachel Marine, Mili Sheth, Haibin Wang, Justin Lee, Suxiang Tong
EPI_ISL_751670	KY State Public Health Lab	Genomics and Discovery, Respiratory Viruses Branch, Division of Viral Diseases, Centers for Disease Control and Prevention	Krista Queen, Yan Li, Ying Tao, Jing Zhang, Anna Uehara, Anna Montmayeur, Clinton R. Paden, Peter W. Cook,Rachel Marine, Mili Sheth, Haibin Wang, Justin Lee, Suxiang Tong
EPI_ISL_751671	RI State Health Laboratories	Genomics and Discovery, Respiratory Viruses Branch, Division of Viral Diseases, Centers for Disease Control and Prevention	Krista Queen, Yan Li, Ying Tao, Jing Zhang, Anna Uehara, Anna Montmayeur, Clinton R. Paden, Peter W. Cook,Rachel Marine, Mili Sheth, Haibin Wang, Justin Lee, Suxiang Tong
EPI_ISL_751684	TX DSHS, Lab Services Section MC 1947	Genomics and Discovery, Respiratory Viruses Branch, Division of Viral Diseases, Centers for Disease Control and Prevention	Krista Queen, Yan Li, Ying Tao, Jing Zhang, Anna Uehara, Anna Montmayeur, Clinton R. Paden, Peter W. Cook,Rachel Marine, Mili Sheth, Haibin Wang, Justin Lee, Suxiang Tong
EPI_ISL_751688	WY Public Health Laboratory	Genomics and Discovery, Respiratory Viruses Branch, Division of Viral Diseases, Centers for Disease Control and Prevention	Krista Queen, Yan Li, Ying Tao, Jing Zhang, Anna Uehara, Anna Montmayeur, Clinton R. Paden, Peter W. Cook,Rachel Marine, Mili Sheth, Haibin Wang, Justin Lee, Suxiang Tong
EPI_ISL_751695	RI State Health Laboratories	Genomics and Discovery, Respiratory Viruses Branch, Division of Viral Diseases, Centers for Disease Control and Prevention	Krista Queen, Yan Li, Ying Tao, Jing Zhang, Anna Uehara, Anna Montmayeur, Clinton R. Paden, Peter W. Cook,Rachel Marine, Mili Sheth, Haibin Wang, Justin Lee, Suxiang Tong
EPI_ISL_751696	LA Office of Public Health Laboratories	Genomics and Discovery, Respiratory Viruses Branch, Division of Viral Diseases, Centers for Disease Control and Prevention	Krista Queen, Yan Li, Ying Tao, Jing Zhang, Anna Uehara, Anna Montmayeur, Clinton R. Paden, Peter W. Cook,Rachel Marine, Mili Sheth, Haibin Wang, Justin Lee, Suxiang Tong
EPI_ISL_751700, EPI_ISL_751703	MD DOH Laboratories Administration	Genomics and Discovery, Respiratory Viruses Branch, Division of Viral Diseases, Centers for Disease Control and Prevention	Krista Queen, Yan Li, Ying Tao, Jing Zhang, Anna Uehara, Anna Montmayeur, Clinton R. Paden, Peter W. Cook,Rachel Marine, Mili Sheth, Haibin Wang, Justin Lee, Suxiang Tong
EPI_ISL_751704, EPI_ISL_751705	CO Dept. of Public Health and Environment, Lab Services Division	Genomics and Discovery, Respiratory Viruses Branch, Division of Viral Diseases, Centers for Disease Control and Prevention	Krista Queen, Yan Li, Ying Tao, Jing Zhang, Anna Uehara, Anna Montmayeur, Clinton R. Paden, Peter W. Cook,Rachel Marine, Mili Sheth, Haibin Wang, Justin Lee, Suxiang Tong
EPI_ISL_751708	NV State Public Health Laboratory	Genomics and Discovery, Respiratory Viruses Branch, Division of Viral Diseases, Centers for Disease Control and Prevention	Krista Queen, Yan Li, Ying Tao, Jing Zhang, Anna Uehara, Anna Montmayeur, Clinton R. Paden, Peter W. Cook,Rachel Marine, Mili Sheth, Haibin Wang, Justin Lee, Suxiang Tong
EPI_ISL_751710, EPI_ISL_751711, EPI_ISL_751713	MT Public Health Laboratory	Genomics and Discovery, Respiratory Viruses Branch, Division of Viral Diseases, Centers for Disease Control and	Krista Queen, Yan Li, Ying Tao, Jing Zhang, Anna Uehara, Anna Montmayeur, Clinton R. Paden, Peter W. Cook,Rachel Marine, Mili Sheth, Haibin Wang, Justin Lee, Suxiang Tong

		Prevention	
EPI_ISL_751714	NV State Public Health Laboratory	Genomics and Discovery, Respiratory Viruses Branch, Division of Viral Diseases, Centers for Disease Control and Prevention	Krista Queen, Yan Li, Ying Tao, Jing Zhang, Anna Uehara, Anna Montmayeur, Clinton R. Paden, Peter W. Cook,Rachel Marine, Mili Sheth, Haibin Wang, Justin Lee, Suxiang Tong
EPI_ISL_751718	SC Dept of Health and Env. Control-Bureau of Laboratories	Genomics and Discovery, Respiratory Viruses Branch, Division of Viral Diseases, Centers for Disease Control and Prevention	Krista Queen, Yan Li, Ying Tao, Jing Zhang, Anna Uehara, Anna Montmayeur, Clinton R. Paden, Peter W. Cook,Rachel Marine, Mili Sheth, Haibin Wang, Justin Lee, Suxiang Tong
EPI_ISL_751722	LA Office of Public Health Laboratories	Genomics and Discovery, Respiratory Viruses Branch, Division of Viral Diseases, Centers for Disease Control and Prevention	Krista Queen, Yan Li, Ying Tao, Jing Zhang, Anna Uehara, Anna Montmayeur, Clinton R. Paden, Peter W. Cook,Rachel Marine, Mili Sheth, Haibin Wang, Justin Lee, Suxiang Tong
EPI_ISL_751728	CO Dept. of Public Health and Environment, Lab Services Division	Genomics and Discovery, Respiratory Viruses Branch, Division of Viral Diseases, Centers for Disease Control and Prevention	Krista Queen, Yan Li, Ying Tao, Jing Zhang, Anna Uehara, Anna Montmayeur, Clinton R. Paden, Peter W. Cook,Rachel Marine, Mili Sheth, Haibin Wang, Justin Lee, Suxiang Tong
EPI_ISL_751736	NV State Public Health Laboratory	Genomics and Discovery, Respiratory Viruses Branch, Division of Viral Diseases, Centers for Disease Control and Prevention	Krista Queen, Yan Li, Ying Tao, Jing Zhang, Anna Uehara, Anna Montmayeur, Clinton R. Paden, Peter W. Cook,Rachel Marine, Mili Sheth, Haibin Wang, Justin Lee, Suxiang Tong
EPI_ISL_751738	MT Public Health Laboratory	Genomics and Discovery, Respiratory Viruses Branch, Division of Viral Diseases, Centers for Disease Control and Prevention	Krista Queen, Yan Li, Ying Tao, Jing Zhang, Anna Uehara, Anna Montmayeur, Clinton R. Paden, Peter W. Cook,Rachel Marine, Mili Sheth, Haibin Wang, Justin Lee, Suxiang Tong
EPI_ISL_751743	WY Public Health Laboratory	Genomics and Discovery, Respiratory Viruses Branch, Division of Viral Diseases, Centers for Disease Control and Prevention	Krista Queen, Yan Li, Ying Tao, Jing Zhang, Anna Uehara, Anna Montmayeur, Clinton R. Paden, Peter W. Cook,Rachel Marine, Mili Sheth, Haibin Wang, Justin Lee, Suxiang Tong
EPI_ISL_751746	WVDHHR - Office of Laboratory Services	Genomics and Discovery, Respiratory Viruses Branch, Division of Viral Diseases, Centers for Disease Control and Prevention	Krista Queen, Yan Li, Ying Tao, Jing Zhang, Anna Uehara, Anna Montmayeur, Clinton R. Paden, Peter W. Cook,Rachel Marine, Mili Sheth, Haibin Wang, Justin Lee, Suxiang Tong
EPI_ISL_751749	TX DSHS, Lab Services Section MC 1947	Genomics and Discovery, Respiratory Viruses Branch, Division of Viral Diseases, Centers for Disease Control and Prevention	Krista Queen, Yan Li, Ying Tao, Jing Zhang, Anna Uehara, Anna Montmayeur, Clinton R. Paden, Peter W. Cook,Rachel Marine, Mili Sheth, Haibin Wang, Justin Lee, Suxiang Tong
EPI_ISL_751751	RI State Health Laboratories	Genomics and Discovery, Respiratory Viruses Branch, Division of Viral Diseases, Centers for Disease Control and Prevention	Krista Queen, Yan Li, Ying Tao, Jing Zhang, Anna Uehara, Anna Montmayeur, Clinton R. Paden, Peter W. Cook,Rachel Marine, Mili Sheth, Haibin Wang, Justin Lee, Suxiang Tong
EPI_ISL_751754	KY State Public Health Lab	Genomics and Discovery, Respiratory Viruses Branch, Division of Viral Diseases, Centers for Disease Control and Prevention	Krista Queen, Yan Li, Ying Tao, Jing Zhang, Anna Uehara, Anna Montmayeur, Clinton R. Paden, Peter W. Cook,Rachel Marine, Mili Sheth, Haibin Wang, Justin Lee, Suxiang Tong
EPI_ISL_751757	RI State Health Laboratories	Genomics and Discovery, Respiratory Viruses Branch, Division of Viral Diseases, Centers for Disease Control and Prevention	Krista Queen, Yan Li, Ying Tao, Jing Zhang, Anna Uehara, Anna Montmayeur, Clinton R. Paden, Peter W. Cook,Rachel Marine, Mili Sheth, Haibin Wang, Justin Lee, Suxiang Tong
EPI_ISL_751763, EPI_ISL_751766	CO Dept. of Public Health and Environment, Lab Services Division	Genomics and Discovery, Respiratory Viruses Branch, Division of Viral Diseases, Centers for Disease Control and Prevention	Krista Queen, Yan Li, Ying Tao, Jing Zhang, Anna Uehara, Anna Montmayeur, Clinton R. Paden, Peter W. Cook,Rachel Marine, Mili Sheth, Haibin Wang, Justin Lee, Suxiang Tong
EPI_ISL_751771	MD DOH Laboratories Administration	Genomics and Discovery, Respiratory Viruses Branch, Division of Viral Diseases, Centers for Disease Control and Prevention	Krista Queen, Yan Li, Ying Tao, Jing Zhang, Anna Uehara, Anna Montmayeur, Clinton R. Paden, Peter W. Cook,Rachel Marine, Mili Sheth, Haibin Wang, Justin Lee, Suxiang Tong
EPI_ISL_751773	NJ Public Health and Environmental Laboratories	Genomics and Discovery, Respiratory Viruses Branch, Division of Viral Diseases, Centers for Disease Control and Prevention	Krista Queen, Yan Li, Ying Tao, Jing Zhang, Anna Uehara, Anna Montmayeur, Clinton R. Paden, Peter W. Cook,Rachel Marine, Mili Sheth, Haibin Wang, Justin Lee, Suxiang Tong
EPI_ISL_751778	NV State Public Health Laboratory	Genomics and Discovery, Respiratory Viruses Branch, Division of Viral Diseases, Centers for Disease Control and Prevention	Krista Queen, Yan Li, Ying Tao, Jing Zhang, Anna Uehara, Anna Montmayeur, Clinton R. Paden, Peter W. Cook,Rachel Marine, Mili Sheth, Haibin Wang, Justin Lee, Suxiang Tong
EPI_ISL_751785, EPI_ISL_751786	WVDHHR - Office of Laboratory Services	Genomics and Discovery, Respiratory Viruses Branch, Division of Viral Diseases, Centers for Disease Control and Prevention	Krista Queen, Yan Li, Ying Tao, Jing Zhang, Anna Uehara, Anna Montmayeur, Clinton R. Paden, Peter W. Cook,Rachel Marine, Mili Sheth, Haibin Wang, Justin Lee, Suxiang Tong
EPI_ISL_751790	RI State Health Laboratories	Genomics and Discovery, Respiratory Viruses Branch, Division of Viral Diseases, Centers for Disease Control and Prevention	Krista Queen, Yan Li, Ying Tao, Jing Zhang, Anna Uehara, Anna Montmayeur, Clinton R. Paden, Peter W. Cook,Rachel Marine, Mili Sheth, Haibin Wang, Justin Lee, Suxiang Tong
EPI_ISL_751805, EPI_ISL_751806, EPI_ISL_751807, EPI_ISL_751808, EPI_ISL_751810, EPI_ISL_751811, EPI_ISL_751812, EPI_ISL_751813, EPI_ISL_751814, EPI_ISL_751816, EPI_ISL_751817, EPI_ISL_751818, EPI_ISL_751819, EPI_ISL_751820, EPI_ISL_751822, EPI_ISL_751823, EPI_ISL_751824, EPI_ISL_751828, EPI_ISL_751832, EPI_ISL_751833, EPI_ISL_751834, EPI_ISL_751838, EPI_ISL_751840, EPI_ISL_751841, EPI_ISL_751842, EPI_ISL_751843, EPI_ISL_751844, EPI_ISL_751845, EPI_ISL_751846			
see above	New Mexico Department of Health Scientific Laboratory	New Mexico Department of Health Scientific Laboratory	D'eldra Malone, Ellie Johnson, Anastacia Griego-Fisher
EPI_ISL_752598	SA Pathology	SA Pathology	Lex Leong, Julien Soubrier, Chuan Kok Lim, Song Gao, Mark Turra, Karin Kassahn, Ivan Bastian, Geoff Higgins
EPI_ISL_753178, EPI_ISL_753179, EPI_ISL_753180, EPI_ISL_753181, EPI_ISL_753182, EPI_ISL_753183, EPI_ISL_753184, EPI_ISL_753185, EPI_ISL_753186, EPI_ISL_753187, EPI_ISL_753188, EPI_ISL_753189, EPI_ISL_753190, EPI_ISL_753191, EPI_ISL_753202, EPI_ISL_753203, EPI_ISL_753205, EPI_ISL_753206, EPI_ISL_753207, EPI_ISL_753208, EPI_ISL_753209, EPI_ISL_753211, EPI_ISL_753212, EPI_ISL_753216, EPI_ISL_753217, EPI_ISL_753219, EPI_ISL_753225, EPI_ISL_753226, EPI_ISL_753228, EPI_ISL_753229, EPI_ISL_753230, EPI_ISL_753231, EPI_ISL_753232, EPI_ISL_753233, EPI_ISL_753236, EPI_ISL_753237, EPI_ISL_753238, EPI_ISL_753239, EPI_ISL_753241, EPI_ISL_753242, EPI_ISL_753243, EPI_ISL_753244, EPI_ISL_753246, EPI_ISL_753248, EPI_ISL_753249, EPI_ISL_753251, EPI_ISL_753252, EPI_ISL_753253, EPI_ISL_753254, EPI_ISL_753255, EPI_ISL_753256, EPI_ISL_753257, EPI_ISL_753258, EPI_ISL_753259, EPI_ISL_753260, EPI_ISL_753261			
see above	State Laboratories Division, Hawaii State Department of Health	State Laboratories Division, Hawaii State Department of Health	Pamela O'Brien, Sabrina Diemert, Drew Kuwazaki, Razvan Sultana, Edward Desmond
EPI_ISL_753559, EPI_ISL_753561, EPI_ISL_753562, EPI_ISL_753563, EPI_ISL_753565, EPI_ISL_753566, EPI_ISL_753567, EPI_ISL_753568, EPI_ISL_753569, EPI_ISL_753570, EPI_ISL_753571, EPI_ISL_753572, EPI_ISL_753573, EPI_ISL_753574, EPI_ISL_753575, EPI_ISL_753576, EPI_ISL_753577, EPI_ISL_753578, EPI_ISL_753579, EPI_ISL_753580, EPI_ISL_753581, EPI_ISL_753582, EPI_ISL_753583, EPI_ISL_753584, EPI_ISL_753585, EPI_ISL_753586, EPI_ISL_753587, EPI_ISL_753588, EPI_ISL_753589, EPI_ISL_753590, EPI_ISL_753591, EPI_ISL_753592, EPI_ISL_753593, EPI_ISL_753594, EPI_ISL_753595, EPI_ISL_753596, EPI_ISL_753597, EPI_ISL_753598, EPI_ISL_753599, EPI_ISL_753600, EPI_ISL_753601, EPI_ISL_753602, EPI_ISL_753603, EPI_ISL_753604, EPI_ISL_753605, EPI_ISL_753606, EPI_ISL_753607, EPI_ISL_753608, EPI_ISL_753609, EPI_ISL_753610, EPI_ISL_753611, EPI_ISL_753612, EPI_ISL_753613, EPI_ISL_753614, EPI_ISL_753615, EPI_ISL_753616, EPI_ISL_753617, EPI_ISL_753618, EPI_ISL_753619, EPI_ISL_753620, EPI_ISL_753621, EPI_ISL_753622, EPI_ISL_753623, EPI_ISL_753624, EPI_ISL_753625, EPI_ISL_753626, EPI_ISL_753628, EPI_ISL_753629, EPI_ISL_753630, EPI_ISL_753631, EPI_ISL_753632, EPI_ISL_753633, EPI_ISL_753635, EPI_ISL_753636, EPI_ISL_753637, EPI_ISL_753638, EPI_ISL_753639, EPI_ISL_753640, EPI_ISL_753641, EPI_ISL_753642, EPI_ISL_753653, EPI_ISL_753654, EPI_ISL_753655, EPI_ISL_753656, EPI_ISL_753657, EPI_ISL_753658, EPI_ISL_753659, EPI_ISL_753660, EPI_ISL_753661, EPI_ISL_753662, EPI_ISL_753663, EPI_ISL_753664, EPI_ISL_753665, EPI_ISL_753666, EPI_ISL_753667, EPI_ISL_753668, EPI_ISL_753669, EPI_ISL_753670, EPI_ISL_753671, EPI_ISL_753672, EPI_ISL_753673, EPI_ISL_753674, EPI_ISL_753675, EPI_ISL_753676, EPI_ISL_753677, EPI_ISL_753678, EPI_ISL_753679, EPI_ISL_753681, EPI_ISL_753682, EPI_ISL_753683, EPI_ISL_753684, EPI_ISL_753685, EPI_ISL_753686, EPI_ISL_753687, EPI_ISL_753688, EPI_ISL_753689, EPI_ISL_753690, EPI_ISL_753691, EPI_ISL_753692			
see above	Clinical virology Laboratory, Children's Hospital Los Angeles	Center for Personalized Medicine, Children's Hospital Los Angeles	Gai et al

EPI_ISL_754059	Specialized Lab for COVID-19 Detection, Department of Genetic Engineering and Biotechnology	Specialized Lab for COVID-19 Detection, Department of Genetic Engineering and Biotechnology	Md. Nazmul Hasan, Md. Akkas Ali, Shamsul H. Prodhan, Hammadul Hoque , Nurnabi Azad Jewel, Md Mohsin Bapary, Diptha Chakraborthy
EPI_ISL_754110, EPI_ISL_754111, EPI_ISL_754113, EPI_ISL_754120, EPI_ISL_754123	UCLA Clinical Micro Lab	Los Angeles County PHL	P. Hemarajata et al.
EPI_ISL_754124, EPI_ISL_754127	USC Clinical Lab	Los Angeles County PHL	P. Hemarajata et al.
EPI_ISL_754130, EPI_ISL_754131	Los Angeles County PHL	Los Angeles County PHL	P. Hemarajata et al.
EPI_ISL_754133, EPI_ISL_754135	USC Clinical Lab	Los Angeles County PHL	P. Hemarajata et al.
EPI_ISL_754142, EPI_ISL_754147, EPI_ISL_754148, EPI_ISL_754149, EPI_ISL_754150, EPI_ISL_754151, EPI_ISL_754158, EPI_ISL_754159, EPI_ISL_754160, EPI_ISL_754163, EPI_ISL_754164, EPI_ISL_754165, EPI_ISL_754166, EPI_ISL_754167, EPI_ISL_754168, EPI_ISL_754170, EPI_ISL_754171, EPI_ISL_754172			
see above	Laboratoire de Microbiologie	National Reference Center for Viruses of Respiratory Infections, Institut Pasteur, Paris	Marion Barbet, Sylvie Behillil, Méline Bizard, Angela Brisebarre, Camille Capel, Etienne Simon-Lorière, Vincent Enouf, Maud Vanpeene, Sylvie van der Werf, Marie-Sarah Fangous
EPI_ISL_754175	Charité Universitätsmedizin Berlin, Institut für Virologie	Charité Universitätsmedizin Berlin, Institut für Virologie	Victor M Corman, Julia Schneider, Jörn Beheim-Schwarzbach, Barbara Mühlemann, Talitha Veith, Terry Jones, Christian Drosten
EPI_ISL_754249, EPI_ISL_754251, EPI_ISL_754252, EPI_ISL_754258, EPI_ISL_754260, EPI_ISL_754262, EPI_ISL_754267, EPI_ISL_754268, EPI_ISL_754269, EPI_ISL_754271, EPI_ISL_754273, EPI_ISL_754275, EPI_ISL_754277, EPI_ISL_754281, EPI_ISL_754282, EPI_ISL_754283, EPI_ISL_754284, EPI_ISL_754285, EPI_ISL_754291, EPI_ISL_754293, EPI_ISL_754310, EPI_ISL_754311, EPI_ISL_754324, EPI_ISL_754341, EPI_ISL_754350, EPI_ISL_754351, EPI_ISL_754355, EPI_ISL_754359, EPI_ISL_754363, EPI_ISL_754366, EPI_ISL_754370, EPI_ISL_754371, EPI_ISL_754372, EPI_ISL_754373, EPI_ISL_754374, EPI_ISL_754381, EPI_ISL_754387			
see above	Respiratory Virus Unit, National Infection Service, Public Health England	COVID-19 Genomics UK (COG-UK) Consortium	PHE Covid Sequencing Team
EPI_ISL_754412, EPI_ISL_754414, EPI_ISL_754415, EPI_ISL_754419, EPI_ISL_754420, EPI_ISL_754423, EPI_ISL_754424, EPI_ISL_754426, EPI_ISL_754436, EPI_ISL_754438, EPI_ISL_754441, EPI_ISL_754448, EPI_ISL_754450, EPI_ISL_754458, EPI_ISL_754461, EPI_ISL_754462, EPI_ISL_754464, EPI_ISL_754465, EPI_ISL_754466, EPI_ISL_754469, EPI_ISL_754470, EPI_ISL_754471, EPI_ISL_754474, EPI_ISL_754476, EPI_ISL_754551, EPI_ISL_754554, EPI_ISL_754555, EPI_ISL_754557, EPI_ISL_754558, EPI_ISL_754559, EPI_ISL_754561, EPI_ISL_754563, EPI_ISL_754565, EPI_ISL_754570, EPI_ISL_754571, EPI_ISL_754573			
see above	Wadsworth Center, New York State Department.of Health	Wadsworth Center, New York State Department.of Health	Kirsten St. George, Daryl M. Lamson, Alexis Russel, Matthew Shudt, Melissa A Leisner, Jonathan Plitnick, Navjot Singh, John Kelly, Sara Griesemer, Erasmus Schneider, Erica Lasek-Nesselquist
EPI_ISL_754623, EPI_ISL_754629, EPI_ISL_754630, EPI_ISL_754632, EPI_ISL_754634, EPI_ISL_754638, EPI_ISL_754639, EPI_ISL_754640, EPI_ISL_754642, EPI_ISL_754644, EPI_ISL_754645, EPI_ISL_754650, EPI_ISL_754651, EPI_ISL_754655, EPI_ISL_754656, EPI_ISL_754658, EPI_ISL_754661, EPI_ISL_754662, EPI_ISL_754663, EPI_ISL_754664, EPI_ISL_754666, EPI_ISL_754670, EPI_ISL_754671, EPI_ISL_754674, EPI_ISL_754675			
see above	University of Wisconsin-Madison AIDS Vaccine Research Laboratories	University of Wisconsin-Madison AIDS Vaccine Research Laboratories	Gage Moreno, Katarina Braun, et al. AIDS Vaccine Research Laboratories
EPI_ISL_754775	Emory Molecular Diagnostics Laboratory, Emory Healthcare	Piantadosi Lab, Emory Department of Pathology	Ahmed Babiker, Anne Piantadosi
EPI_ISL_754847	Korian Villa d'Azon	National Reference Center for Viruses of Respiratory Infections, Institut Pasteur, Paris	Marion Barbet, Sylvie Behillil, Méline Bizard, Angela Brisebarre, Camille Capel, Etienne Simon-Lorière, Vincent Enouf, Maud Vanpeene, Sylvie van der Werf
EPI_ISL_754849, EPI_ISL_754851, EPI_ISL_754852	Laboratoire de Virologie Hôpital Robert Debré	National Reference Center for Viruses of Respiratory Infections, Institut Pasteur, Paris	Marion Barbet, Sylvie Behillil, Méline Bizard, Angela Brisebarre, Camille Capel, Etienne Simon-Lorière, Vincent Enouf, Maud Vanpeene, Sylvie van der Werf, Andreoletti Laurent
EPI_ISL_755148, EPI_ISL_755150, EPI_ISL_755153, EPI_ISL_755158, EPI_ISL_755176, EPI_ISL_755177, EPI_ISL_755186, EPI_ISL_755195	UCSD EXCITE lab	Andersen lab at Scripps Research	SEARCH Alliance San Diego
EPI_ISL_755441, EPI_ISL_755443, EPI_ISL_755490, EPI_ISL_755491, EPI_ISL_755492, EPI_ISL_755493, EPI_ISL_755494, EPI_ISL_755495, EPI_ISL_755496, EPI_ISL_755498, EPI_ISL_755500, EPI_ISL_755503, EPI_ISL_755507, EPI_ISL_755508, EPI_ISL_755511, EPI_ISL_755512, EPI_ISL_755513, EPI_ISL_755514, EPI_ISL_755519, EPI_ISL_755520, EPI_ISL_755522, EPI_ISL_755525, EPI_ISL_755526, EPI_ISL_755527, EPI_ISL_755529, EPI_ISL_755530, EPI_ISL_755531, EPI_ISL_755533, EPI_ISL_755535, EPI_ISL_755536, EPI_ISL_755537, EPI_ISL_755538, EPI_ISL_755539, EPI_ISL_755540, EPI_ISL_755541, EPI_ISL_755543, EPI_ISL_755544, EPI_ISL_755546, EPI_ISL_755547, EPI_ISL_755549, EPI_ISL_755551, EPI_ISL_755553, EPI_ISL_755556, EPI_ISL_755557, EPI_ISL_755558, EPI_ISL_755559, EPI_ISL_755560, EPI_ISL_755561, EPI_ISL_755562, EPI_ISL_755565, EPI_ISL_755567			
see above	Maine Health and Environmental Testing Laboratory	Tewhey Lab, The Jackson Laboratory	Matluk,N., Dewey,H., Iosue,F., Barter,M., Lynch,R., Munger,H. and Tewhey,R.
EPI_ISL_755568, EPI_ISL_755570, EPI_ISL_755571	SA Pathology	SA Pathology	Lex Leong, Julien Soubrier, Chuan Kok Lim, Song Gao, Mark Turra, Karin Kassahn, Ivan Bastian, Geoff Higgins
EPI_ISL_755572, EPI_ISL_755573, EPI_ISL_755574	Center of Advanced Studies and Technology, CAST	Center of Advanced Studies and Technology, CAST	Ferrante,R., Mandatori,D., De Fabritiis,S.
EPI_ISL_755589	Laboratoire national de santé, Microbiology, Virology	Laboratoire national de santé, Microbiology, Microbial Genomics Platform	Anke Wienecke-Baldacchino, Catherine Ragimbeau,Jessica Tapp, Fatu Djabi, Lise Pignon, Raoul Salmon, Tamir Abdelrahman
EPI_ISL_755600, EPI_ISL_755605	Helix/Illumina	Genomics and Discovery, Respiratory Viruses Branch, Division of Viral Diseases, Centers for Disease Control and Prevention	Peter W. Cook, Dhwani Batra, Eileen de Feo, Jan Antico, Christine Tran, Matthew Tolentino, Shannon Wickline, Kim Gietzen, Brad Sickler, Jingtao Liu, Eric Allen, Phil Febbo, Summer Galloway, Nicole L. Washington, Simon White, Geraint Levan, Kelly Schiabor Barrett, Elizabeth Cirulli, Alexandre Bolze, Ary Ascencio, Charlotte Rivera-Garcia, Ryan Cho, Jason Nguyen, Sherry Wang, Jimmy Ramirez, Tyler Cassens, Efren Sandoval, Magnus Isaksson, William Lee, David Becker, Marc Laurent, James Lu, Clinton R. Paden, Suxiang Tong, Duncan MacCannell
EPI_ISL_755630, EPI_ISL_755632, EPI_ISL_755633	LabPLUS	Institute of Environmental Science and Research (ESR)	Xiaoyun Ren, Matt Storey, Nikki Freed, Muhammad Faisal, Jing Wang, Hermes Perez, Anja Werno, Antje van der Linden, Arlo Upton, Chris Mansell, David Hammer, Dragana Drinkovic, Gary McAuliffe, Hana Sofia Andersson, James Ussher, Jill Sherwood, Josh Freeman, Julia Howard, Juliet Elvy, Mary DeAlmeida, Matt Blakiston, Matthew Rogers, Max Bloomfield, Michael Addidle, Michelle Balm, Sally Roberts, Sarah Jefferies, Sharmini Muttaiyah, Susan Morpeth, Susan Taylor, Timothy Blackmore, Vani Sathyendran, Veronica Playle, Virginia Hope, Erasmus Smit, Lauren Jelly, Olin Silander, Joep de Ligt
EPI_ISL_755639	Bundeswehr Institute of Microbiology	Bundeswehr Institute of Microbiology	Malena Bestehorn-Willmann, Mathias Walter, Christina Bugert, Joachim Bugert, Roman Wölfel, Markus Antwerpen
EPI_ISL_755641	Instituto Adolfo Lutz - Regional de Santo Andre	Instituto Adolfo Lutz, Interdiciplinary Procedures Center, Strategic Laboratory	Claudio Tavares Sacchi, Claudia Regina Gonçalves, Erica Valessa Ramos Gomes, Karoline Rodrigues Campos
EPI_ISL_755644, EPI_ISL_755645	Lab LOC - Itapecerica da Serra	Instituto Adolfo Lutz, Interdiciplinary Procedures Center, Strategic Laboratory	Claudio Tavares Sacchi, Claudia Regina Gonçalves, Erica Valessa Ramos Gomes, Karoline Rodrigues Campos
EPI_ISL_755648	Instituto Adolfo Lutz - Regional de Taubate	Instituto Adolfo Lutz, Interdiciplinary Procedures Center, Strategic Laboratory	Claudio Tavares Sacchi, Claudia Regina Gonçalves, Erica Valessa Ramos Gomes, Karoline Rodrigues Campos
EPI_ISL_755649	Instituto Adolfo Lutz - Regional de Santo Andre	Instituto Adolfo Lutz, Interdiciplinary Procedures Center, Strategic Laboratory	Claudio Tavares Sacchi, Claudia Regina Gonçalves, Erica Valessa Ramos Gomes, Karoline Rodrigues Campos
EPI_ISL_755650	Instituto Adolfo Lutz - Regional de Taubate	Instituto Adolfo Lutz, Interdiciplinary Procedures Center, Strategic Laboratory	Claudio Tavares Sacchi, Claudia Regina Gonçalves, Erica Valessa Ramos Gomes, Karoline Rodrigues Campos
EPI_ISL_755651, EPI_ISL_755653, EPI_ISL_755654	Instituto Adolfo Lutz - Central	Instituto Adolfo Lutz, Interdiciplinary Procedures Center, Strategic Laboratory	Claudio Tavares Sacchi, Claudia Regina Gonçalves, Erica Valessa Ramos Gomes, Karoline Rodrigues Campos
EPI_ISL_755655	Instituto Adolfo Lutz - Regional de Campinas	Instituto Adolfo Lutz, Interdiciplinary Procedures Center, Strategic Laboratory	Claudio Tavares Sacchi, Claudia Regina Gonçalves, Erica Valessa Ramos Gomes, Karoline Rodrigues Campos
EPI_ISL_755803, EPI_ISL_755804	Toronto Invasive Bacterial Diseases Network	McMaster University	Allison McGeer, Patryk Aftanas, Hooman Derakhshani, Angel Li, Kuganya Nirmalarajah, Emily Panousis, Ahmed Draia, Jalees Nasir, Michael Surette, Samira Mubareka, Andrew G. McArthur
EPI_ISL_756142, EPI_ISL_756147, EPI_ISL_756151	Respiratory Virus Unit, National Infection Service, Public Health England	COVID-19 Genomics UK (COG-UK) Consortium	PHE Covid Sequencing Team

EPI_ISL_756272	Hospital General Universitario La Princesa	Hospital General Universitario Gregorio Marañón	Sergio Buenestado Serrano, Pedro Sola Campoy, Pilar Catalán, Arturo fraile Torres, Andrés Von Wernitz, Carmen del Arco, Patricia Muñoz, Laura Cardeñoso, Darío García de Viedma.
EPI_ISL_756381, EPI_ISL_756383	Lighthouse Lab in Alderley Park	Wellcome Sanger Institute for the COVID-19 Genomics UK (COG-UK) Consortium	Jacquelyn Wynn, Mairead Hyland, The Lighthouse Lab in Alderley Park and Alex Alderton, Roberto Amato, Sonia Goncalves, Ewan Harrison, David K. Jackson, Ian Johnston, Dominic Kwiatkowski, Cordelia Langford, John Sillitoe on behalf of the Wellcome Sanger Institute COVID-19 Surveillance Team
EPI_ISL_756384, EPI_ISL_756389	Lighthouse Lab in Cambridge	Wellcome Sanger Institute for the COVID-19 Genomics UK (COG-UK) Consortium	Rob Howes, The Lighthouse Lab in Cambridge and Alex Alderton, Roberto Amato, Sonia Goncalves, Ewan Harrison, David K. Jackson, Ian Johnston, Dominic Kwiatkowski, Cordelia Langford, John Sillitoe on behalf of the Wellcome Sanger Institute COVID-19 Surveillance Team
EPI_ISL_756390, EPI_ISL_756391, EPI_ISL_756394	Lighthouse Lab in Alderley Park	Wellcome Sanger Institute for the COVID-19 Genomics UK (COG-UK) Consortium	Jacquelyn Wynn, Mairead Hyland, The Lighthouse Lab in Alderley Park and Alex Alderton, Roberto Amato, Sonia Goncalves, Ewan Harrison, David K. Jackson, Ian Johnston, Dominic Kwiatkowski, Cordelia Langford, John Sillitoe on behalf of the Wellcome Sanger Institute COVID-19 Surveillance Team
EPI_ISL_756395	Lighthouse Lab in Cambridge	Wellcome Sanger Institute for the COVID-19 Genomics UK (COG-UK) Consortium	Rob Howes, The Lighthouse Lab in Cambridge and Alex Alderton, Roberto Amato, Sonia Goncalves, Ewan Harrison, David K. Jackson, Ian Johnston, Dominic Kwiatkowski, Cordelia Langford, John Sillitoe on behalf of the Wellcome Sanger Institute COVID-19 Surveillance Team
EPI_ISL_756396, EPI_ISL_756397, EPI_ISL_756398, EPI_ISL_756400, EPI_ISL_756404, EPI_ISL_756405, EPI_ISL_756407	Lighthouse Lab in Alderley Park	Wellcome Sanger Institute for the COVID-19 Genomics UK (COG-UK) Consortium	Jacquelyn Wynn, Mairead Hyland, The Lighthouse Lab in Alderley Park and Alex Alderton, Roberto Amato, Sonia Goncalves, Ewan Harrison, David K. Jackson, Ian Johnston, Dominic Kwiatkowski, Cordelia Langford, John Sillitoe on behalf of the Wellcome Sanger Institute COVID-19 Surveillance Team
EPI_ISL_756409	Lighthouse Lab in Cambridge	Wellcome Sanger Institute for the COVID-19 Genomics UK (COG-UK) Consortium	Rob Howes, The Lighthouse Lab in Cambridge and Alex Alderton, Roberto Amato, Sonia Goncalves, Ewan Harrison, David K. Jackson, Ian Johnston, Dominic Kwiatkowski, Cordelia Langford, John Sillitoe on behalf of the Wellcome Sanger Institute COVID-19 Surveillance Team
EPI_ISL_756411, EPI_ISL_756412	Lighthouse Lab in Alderley Park	Wellcome Sanger Institute for the COVID-19 Genomics UK (COG-UK) Consortium	Jacquelyn Wynn, Mairead Hyland, The Lighthouse Lab in Alderley Park and Alex Alderton, Roberto Amato, Sonia Goncalves, Ewan Harrison, David K. Jackson, Ian Johnston, Dominic Kwiatkowski, Cordelia Langford, John Sillitoe on behalf of the Wellcome Sanger Institute COVID-19 Surveillance Team
EPI_ISL_756413	Lighthouse Lab in Cambridge	Wellcome Sanger Institute for the COVID-19 Genomics UK (COG-UK) Consortium	Rob Howes, The Lighthouse Lab in Cambridge and Alex Alderton, Roberto Amato, Sonia Goncalves, Ewan Harrison, David K. Jackson, Ian Johnston, Dominic Kwiatkowski, Cordelia Langford, John Sillitoe on behalf of the Wellcome Sanger Institute COVID-19 Surveillance Team
EPI_ISL_756414	Lighthouse Lab in Alderley Park	Wellcome Sanger Institute for the COVID-19 Genomics UK (COG-UK) Consortium	Jacquelyn Wynn, Mairead Hyland, The Lighthouse Lab in Alderley Park and Alex Alderton, Roberto Amato, Sonia Goncalves, Ewan Harrison, David K. Jackson, Ian Johnston, Dominic Kwiatkowski, Cordelia Langford, John Sillitoe on behalf of the Wellcome Sanger Institute COVID-19 Surveillance Team
EPI_ISL_756415, EPI_ISL_756416, EPI_ISL_756417, EPI_ISL_756420	Lighthouse Lab in Cambridge	Wellcome Sanger Institute for the COVID-19 Genomics UK (COG-UK) Consortium	Rob Howes, The Lighthouse Lab in Cambridge and Alex Alderton, Roberto Amato, Sonia Goncalves, Ewan Harrison, David K. Jackson, Ian Johnston, Dominic Kwiatkowski, Cordelia Langford, John Sillitoe on behalf of the Wellcome Sanger Institute COVID-19 Surveillance Team
EPI_ISL_756421	Lighthouse Lab in Alderley Park	Wellcome Sanger Institute for the COVID-19 Genomics UK (COG-UK) Consortium	Jacquelyn Wynn, Mairead Hyland, The Lighthouse Lab in Alderley Park and Alex Alderton, Roberto Amato, Sonia Goncalves, Ewan Harrison, David K. Jackson, Ian Johnston, Dominic Kwiatkowski, Cordelia Langford, John Sillitoe on behalf of the Wellcome Sanger Institute COVID-19 Surveillance Team
EPI_ISL_756422, EPI_ISL_756424	Lighthouse Lab in Cambridge	Wellcome Sanger Institute for the COVID-19 Genomics UK (COG-UK) Consortium	Rob Howes, The Lighthouse Lab in Cambridge and Alex Alderton, Roberto Amato, Sonia Goncalves, Ewan Harrison, David K. Jackson, Ian Johnston, Dominic Kwiatkowski, Cordelia Langford, John Sillitoe on behalf of the Wellcome Sanger Institute COVID-19 Surveillance Team
EPI_ISL_756425	Lighthouse Lab in Alderley Park	Wellcome Sanger Institute for the COVID-19 Genomics UK (COG-UK) Consortium	Jacquelyn Wynn, Mairead Hyland, The Lighthouse Lab in Alderley Park and Alex Alderton, Roberto Amato, Sonia Goncalves, Ewan Harrison, David K. Jackson, Ian Johnston, Dominic Kwiatkowski, Cordelia Langford, John Sillitoe on behalf of the Wellcome Sanger Institute COVID-19 Surveillance Team
EPI_ISL_756426, EPI_ISL_756429, EPI_ISL_756431	Lighthouse Lab in Cambridge	Wellcome Sanger Institute for the COVID-19 Genomics UK (COG-UK) Consortium	Rob Howes, The Lighthouse Lab in Cambridge and Alex Alderton, Roberto Amato, Sonia Goncalves, Ewan Harrison, David K. Jackson, Ian Johnston, Dominic Kwiatkowski, Cordelia Langford, John Sillitoe on behalf of the Wellcome Sanger Institute COVID-19 Surveillance Team
EPI_ISL_756433	Lighthouse Lab in Alderley Park	Wellcome Sanger Institute for the COVID-19 Genomics UK (COG-UK) Consortium	Jacquelyn Wynn, Mairead Hyland, The Lighthouse Lab in Alderley Park and Alex Alderton, Roberto Amato, Sonia Goncalves, Ewan Harrison, David K. Jackson, Ian Johnston, Dominic Kwiatkowski, Cordelia Langford, John Sillitoe on behalf of the Wellcome Sanger Institute COVID-19 Surveillance Team
EPI_ISL_756434	Lighthouse Lab in Cambridge	Wellcome Sanger Institute for the COVID-19 Genomics UK (COG-UK) Consortium	Rob Howes, The Lighthouse Lab in Cambridge and Alex Alderton, Roberto Amato, Sonia Goncalves, Ewan Harrison, David K. Jackson, Ian Johnston, Dominic Kwiatkowski, Cordelia Langford, John Sillitoe on behalf of the Wellcome Sanger Institute COVID-19 Surveillance Team
EPI_ISL_756435, EPI_ISL_756436, EPI_ISL_756437	Lighthouse Lab in Alderley Park	Wellcome Sanger Institute for the COVID-19 Genomics UK (COG-UK) Consortium	Jacquelyn Wynn, Mairead Hyland, The Lighthouse Lab in Alderley Park and Alex Alderton, Roberto Amato, Sonia Goncalves, Ewan Harrison, David K. Jackson, Ian Johnston, Dominic Kwiatkowski, Cordelia Langford, John Sillitoe on behalf of the Wellcome Sanger Institute COVID-19 Surveillance Team
EPI_ISL_756438	Lighthouse Lab in Cambridge	Wellcome Sanger Institute for the COVID-19 Genomics UK (COG-UK) Consortium	Rob Howes, The Lighthouse Lab in Cambridge and Alex Alderton, Roberto Amato, Sonia Goncalves, Ewan Harrison, David K. Jackson, Ian Johnston, Dominic Kwiatkowski, Cordelia Langford, John Sillitoe on behalf of the Wellcome Sanger Institute COVID-19 Surveillance Team
EPI_ISL_756439	Lighthouse Lab in Alderley Park	Wellcome Sanger Institute for the COVID-19 Genomics UK (COG-UK) Consortium	Jacquelyn Wynn, Mairead Hyland, The Lighthouse Lab in Alderley Park and Alex Alderton, Roberto Amato, Sonia Goncalves, Ewan Harrison, David K. Jackson, Ian Johnston, Dominic Kwiatkowski, Cordelia Langford, John Sillitoe on behalf of the Wellcome Sanger Institute COVID-19 Surveillance Team
EPI_ISL_756442	Lighthouse Lab in Cambridge	Wellcome Sanger Institute for the COVID-19 Genomics UK (COG-UK) Consortium	Rob Howes, The Lighthouse Lab in Cambridge and Alex Alderton, Roberto Amato, Sonia Goncalves, Ewan Harrison, David K. Jackson, Ian Johnston, Dominic Kwiatkowski, Cordelia Langford, John Sillitoe on behalf of the Wellcome Sanger Institute COVID-19 Surveillance Team
EPI_ISL_756443	Lighthouse Lab in Alderley Park	Wellcome Sanger Institute for the COVID-19 Genomics UK (COG-UK) Consortium	Jacquelyn Wynn, Mairead Hyland, The Lighthouse Lab in Alderley Park and Alex Alderton, Roberto Amato, Sonia Goncalves, Ewan Harrison, David K. Jackson, Ian Johnston, Dominic Kwiatkowski, Cordelia Langford, John Sillitoe on behalf of the Wellcome Sanger Institute COVID-19 Surveillance Team
EPI_ISL_756444	Lighthouse Lab in Cambridge	Wellcome Sanger Institute for the COVID-19 Genomics UK (COG-UK) Consortium	Rob Howes, The Lighthouse Lab in Cambridge and Alex Alderton, Roberto Amato, Sonia Goncalves, Ewan Harrison, David K. Jackson, Ian Johnston, Dominic Kwiatkowski, Cordelia Langford, John Sillitoe on behalf of the Wellcome Sanger Institute COVID-19 Surveillance Team
EPI_ISL_756445, EPI_ISL_756447	Lighthouse Lab in Alderley Park	Wellcome Sanger Institute for the COVID-19 Genomics UK (COG-UK) Consortium	Jacquelyn Wynn, Mairead Hyland, The Lighthouse Lab in Alderley Park and Alex Alderton, Roberto Amato, Sonia Goncalves, Ewan Harrison, David K. Jackson, Ian Johnston, Dominic Kwiatkowski, Cordelia Langford, John Sillitoe on behalf of the Wellcome Sanger Institute COVID-19 Surveillance Team
EPI_ISL_756448	Lighthouse Lab in Cambridge	Wellcome Sanger Institute for the COVID-19 Genomics UK (COG-UK) Consortium	Rob Howes, The Lighthouse Lab in Cambridge and Alex Alderton, Roberto Amato, Sonia Goncalves, Ewan Harrison, David K. Jackson, Ian Johnston, Dominic Kwiatkowski, Cordelia Langford, John Sillitoe on behalf of the Wellcome Sanger Institute COVID-19 Surveillance Team
EPI_ISL_756450, EPI_ISL_756451, EPI_ISL_756452	Lighthouse Lab in Alderley Park	Wellcome Sanger Institute for the COVID-19 Genomics UK (COG-UK) Consortium	Jacquelyn Wynn, Mairead Hyland, The Lighthouse Lab in Alderley Park and Alex Alderton, Roberto Amato, Sonia Goncalves, Ewan Harrison, David K. Jackson, Ian Johnston, Dominic Kwiatkowski, Cordelia Langford, John Sillitoe on behalf of the Wellcome Sanger Institute COVID-19 Surveillance Team
EPI_ISL_756453, EPI_ISL_756454	Lighthouse Lab in Cambridge	Wellcome Sanger Institute for the COVID-19 Genomics UK (COG-UK) Consortium	Rob Howes, The Lighthouse Lab in Cambridge and Alex Alderton, Roberto Amato, Sonia Goncalves, Ewan Harrison, David K. Jackson, Ian Johnston, Dominic Kwiatkowski, Cordelia Langford, John Sillitoe on behalf of the Wellcome Sanger Institute COVID-19 Surveillance Team
EPI_ISL_756455	Lighthouse Lab in Alderley Park	Wellcome Sanger Institute for the COVID-19 Genomics UK (COG-UK) Consortium	Jacquelyn Wynn, Mairead Hyland, The Lighthouse Lab in Alderley Park and Alex Alderton, Roberto Amato, Sonia Goncalves, Ewan Harrison, David K. Jackson, Ian Johnston, Dominic Kwiatkowski, Cordelia Langford, John Sillitoe on behalf of the Wellcome Sanger Institute COVID-19 Surveillance Team
EPI_ISL_756456	Lighthouse Lab in Cambridge	Wellcome Sanger Institute for the COVID-19 Genomics UK (COG-UK) Consortium	Rob Howes, The Lighthouse Lab in Cambridge and Alex Alderton, Roberto Amato, Sonia Goncalves, Ewan Harrison, David K. Jackson, Ian Johnston, Dominic Kwiatkowski, Cordelia Langford, John Sillitoe on behalf of the Wellcome Sanger Institute COVID-19 Surveillance Team
EPI_ISL_756457, EPI_ISL_756458	Lighthouse Lab in Alderley Park	Wellcome Sanger Institute for the COVID-19 Genomics UK (COG-UK) Consortium	Jacquelyn Wynn, Mairead Hyland, The Lighthouse Lab in Alderley Park and Alex Alderton, Roberto Amato, Sonia Goncalves, Ewan Harrison, David K. Jackson, Ian Johnston, Dominic Kwiatkowski, Cordelia Langford, John Sillitoe on behalf of the Wellcome Sanger Institute COVID-19 Surveillance Team
EPI_ISL_756460	Lighthouse Lab in Cambridge	Wellcome Sanger Institute for the COVID-19 Genomics UK (COG-UK) Consortium	Rob Howes, The Lighthouse Lab in Cambridge and Alex Alderton, Roberto Amato, Sonia Goncalves, Ewan Harrison, David K. Jackson, Ian Johnston, Dominic Kwiatkowski, Cordelia Langford, John Sillitoe on behalf of the Wellcome Sanger Institute COVID-19 Surveillance Team
EPI_ISL_756463, EPI_ISL_756464, EPI_ISL_756466, EPI_ISL_756468	Lighthouse Lab in Alderley Park	Wellcome Sanger Institute for the COVID-19 Genomics UK (COG-UK) Consortium	Jacquelyn Wynn, Mairead Hyland, The Lighthouse Lab in Alderley Park and Alex Alderton, Roberto Amato, Sonia Goncalves, Ewan Harrison, David K. Jackson, Ian Johnston, Dominic Kwiatkowski, Cordelia Langford, John Sillitoe on behalf of the Wellcome Sanger Institute COVID-19 Surveillance Team
EPI_ISL_756469	Lighthouse Lab in Cambridge	Wellcome Sanger Institute for the COVID-19 Genomics UK (COG-UK) Consortium	Rob Howes, The Lighthouse Lab in Cambridge and Alex Alderton, Roberto Amato, Sonia Goncalves, Ewan Harrison, David K. Jackson, Ian Johnston, Dominic Kwiatkowski, Cordelia Langford, John Sillitoe on behalf of the Wellcome Sanger Institute COVID-19 Surveillance Team
EPI_ISL_756470, EPI_ISL_756472, EPI_ISL_756473	Lighthouse Lab in Alderley Park	Wellcome Sanger Institute for the COVID-19 Genomics UK (COG-UK) Consortium	Jacquelyn Wynn, Mairead Hyland, The Lighthouse Lab in Alderley Park and Alex Alderton, Roberto Amato, Sonia Goncalves, Ewan Harrison, David K. Jackson, Ian Johnston, Dominic Kwiatkowski, Cordelia Langford, John Sillitoe on behalf of the Wellcome Sanger Institute COVID-19 Surveillance Team

EPI_ISL_756474, EPI_ISL_756475	Lighthouse Lab in Cambridge	Wellcome Sanger Institute for the COVID-19 Genomics UK (COG-UK) Consortium	Rob Howes, The Lighthouse Lab in Cambridge and Alex Alderton, Roberto Amato, Sonia Goncalves, Ewan Harrison, David K. Jackson, Ian Johnston, Dominic Kwiatkowski, Cordelia Langford, John Sillitoe on behalf of the Wellcome Sanger Institute COVID-19 Surveillance Team
EPI_ISL_756476, EPI_ISL_756477	Lighthouse Lab in Alderley Park	Wellcome Sanger Institute for the COVID-19 Genomics UK (COG-UK) Consortium	Jacquelyn Wynn, Mairead Hyland, The Lighthouse Lab in Alderley Park and Alex Alderton, Roberto Amato, Sonia Goncalves, Ewan Harrison, David K. Jackson, Ian Johnston, Dominic Kwiatkowski, Cordelia Langford, John Sillitoe on behalf of the Wellcome Sanger Institute COVID-19 Surveillance Team
EPI_ISL_756478	Lighthouse Lab in Cambridge	Wellcome Sanger Institute for the COVID-19 Genomics UK (COG-UK) Consortium	Rob Howes, The Lighthouse Lab in Cambridge and Alex Alderton, Roberto Amato, Sonia Goncalves, Ewan Harrison, David K. Jackson, Ian Johnston, Dominic Kwiatkowski, Cordelia Langford, John Sillitoe on behalf of the Wellcome Sanger Institute COVID-19 Surveillance Team
EPI_ISL_756479	Lighthouse Lab in Alderley Park	Wellcome Sanger Institute for the COVID-19 Genomics UK (COG-UK) Consortium	Jacquelyn Wynn, Mairead Hyland, The Lighthouse Lab in Alderley Park and Alex Alderton, Roberto Amato, Sonia Goncalves, Ewan Harrison, David K. Jackson, Ian Johnston, Dominic Kwiatkowski, Cordelia Langford, John Sillitoe on behalf of the Wellcome Sanger Institute COVID-19 Surveillance Team
EPI_ISL_756480, EPI_ISL_756481	Lighthouse Lab in Cambridge	Wellcome Sanger Institute for the COVID-19 Genomics UK (COG-UK) Consortium	Rob Howes, The Lighthouse Lab in Cambridge and Alex Alderton, Roberto Amato, Sonia Goncalves, Ewan Harrison, David K. Jackson, Ian Johnston, Dominic Kwiatkowski, Cordelia Langford, John Sillitoe on behalf of the Wellcome Sanger Institute COVID-19 Surveillance Team
EPI_ISL_756483	Lighthouse Lab in Alderley Park	Wellcome Sanger Institute for the COVID-19 Genomics UK (COG-UK) Consortium	Jacquelyn Wynn, Mairead Hyland, The Lighthouse Lab in Alderley Park and Alex Alderton, Roberto Amato, Sonia Goncalves, Ewan Harrison, David K. Jackson, Ian Johnston, Dominic Kwiatkowski, Cordelia Langford, John Sillitoe on behalf of the Wellcome Sanger Institute COVID-19 Surveillance Team
EPI_ISL_756485	Lighthouse Lab in Cambridge	Wellcome Sanger Institute for the COVID-19 Genomics UK (COG-UK) Consortium	Rob Howes, The Lighthouse Lab in Cambridge and Alex Alderton, Roberto Amato, Sonia Goncalves, Ewan Harrison, David K. Jackson, Ian Johnston, Dominic Kwiatkowski, Cordelia Langford, John Sillitoe on behalf of the Wellcome Sanger Institute COVID-19 Surveillance Team
EPI_ISL_756489	Lighthouse Lab in Alderley Park	Wellcome Sanger Institute for the COVID-19 Genomics UK (COG-UK) Consortium	Jacquelyn Wynn, Mairead Hyland, The Lighthouse Lab in Alderley Park and Alex Alderton, Roberto Amato, Sonia Goncalves, Ewan Harrison, David K. Jackson, Ian Johnston, Dominic Kwiatkowski, Cordelia Langford, John Sillitoe on behalf of the Wellcome Sanger Institute COVID-19 Surveillance Team
EPI_ISL_756491	Lighthouse Lab in Cambridge	Wellcome Sanger Institute for the COVID-19 Genomics UK (COG-UK) Consortium	Rob Howes, The Lighthouse Lab in Cambridge and Alex Alderton, Roberto Amato, Sonia Goncalves, Ewan Harrison, David K. Jackson, Ian Johnston, Dominic Kwiatkowski, Cordelia Langford, John Sillitoe on behalf of the Wellcome Sanger Institute COVID-19 Surveillance Team
EPI_ISL_756493, EPI_ISL_756494, EPI_ISL_756495	Lighthouse Lab in Alderley Park	Wellcome Sanger Institute for the COVID-19 Genomics UK (COG-UK) Consortium	Jacquelyn Wynn, Mairead Hyland, The Lighthouse Lab in Alderley Park and Alex Alderton, Roberto Amato, Sonia Goncalves, Ewan Harrison, David K. Jackson, Ian Johnston, Dominic Kwiatkowski, Cordelia Langford, John Sillitoe on behalf of the Wellcome Sanger Institute COVID-19 Surveillance Team
EPI_ISL_756496, EPI_ISL_756497, EPI_ISL_756499, EPI_ISL_756502	Lighthouse Lab in Cambridge	Wellcome Sanger Institute for the COVID-19 Genomics UK (COG-UK) Consortium	Rob Howes, The Lighthouse Lab in Cambridge and Alex Alderton, Roberto Amato, Sonia Goncalves, Ewan Harrison, David K. Jackson, Ian Johnston, Dominic Kwiatkowski, Cordelia Langford, John Sillitoe on behalf of the Wellcome Sanger Institute COVID-19 Surveillance Team
EPI_ISL_756503	Lighthouse Lab in Alderley Park	Wellcome Sanger Institute for the COVID-19 Genomics UK (COG-UK) Consortium	Jacquelyn Wynn, Mairead Hyland, The Lighthouse Lab in Alderley Park and Alex Alderton, Roberto Amato, Sonia Goncalves, Ewan Harrison, David K. Jackson, Ian Johnston, Dominic Kwiatkowski, Cordelia Langford, John Sillitoe on behalf of the Wellcome Sanger Institute COVID-19 Surveillance Team
EPI_ISL_756505, EPI_ISL_756506, EPI_ISL_756507, EPI_ISL_756508	Lighthouse Lab in Cambridge	Wellcome Sanger Institute for the COVID-19 Genomics UK (COG-UK) Consortium	Rob Howes, The Lighthouse Lab in Cambridge and Alex Alderton, Roberto Amato, Sonia Goncalves, Ewan Harrison, David K. Jackson, Ian Johnston, Dominic Kwiatkowski, Cordelia Langford, John Sillitoe on behalf of the Wellcome Sanger Institute COVID-19 Surveillance Team
EPI_ISL_756510	Lighthouse Lab in Alderley Park	Wellcome Sanger Institute for the COVID-19 Genomics UK (COG-UK) Consortium	Jacquelyn Wynn, Mairead Hyland, The Lighthouse Lab in Alderley Park and Alex Alderton, Roberto Amato, Sonia Goncalves, Ewan Harrison, David K. Jackson, Ian Johnston, Dominic Kwiatkowski, Cordelia Langford, John Sillitoe on behalf of the Wellcome Sanger Institute COVID-19 Surveillance Team
EPI_ISL_756511, EPI_ISL_756513	Lighthouse Lab in Cambridge	Wellcome Sanger Institute for the COVID-19 Genomics UK (COG-UK) Consortium	Rob Howes, The Lighthouse Lab in Cambridge and Alex Alderton, Roberto Amato, Sonia Goncalves, Ewan Harrison, David K. Jackson, Ian Johnston, Dominic Kwiatkowski, Cordelia Langford, John Sillitoe on behalf of the Wellcome Sanger Institute COVID-19 Surveillance Team
EPI_ISL_756514, EPI_ISL_756516	Lighthouse Lab in Alderley Park	Wellcome Sanger Institute for the COVID-19 Genomics UK (COG-UK) Consortium	Jacquelyn Wynn, Mairead Hyland, The Lighthouse Lab in Alderley Park and Alex Alderton, Roberto Amato, Sonia Goncalves, Ewan Harrison, David K. Jackson, Ian Johnston, Dominic Kwiatkowski, Cordelia Langford, John Sillitoe on behalf of the Wellcome Sanger Institute COVID-19 Surveillance Team
EPI_ISL_756517, EPI_ISL_756519	Lighthouse Lab in Cambridge	Wellcome Sanger Institute for the COVID-19 Genomics UK (COG-UK) Consortium	Rob Howes, The Lighthouse Lab in Cambridge and Alex Alderton, Roberto Amato, Sonia Goncalves, Ewan Harrison, David K. Jackson, Ian Johnston, Dominic Kwiatkowski, Cordelia Langford, John Sillitoe on behalf of the Wellcome Sanger Institute COVID-19 Surveillance Team
EPI_ISL_756520	Lighthouse Lab in Alderley Park	Wellcome Sanger Institute for the COVID-19 Genomics UK (COG-UK) Consortium	Jacquelyn Wynn, Mairead Hyland, The Lighthouse Lab in Alderley Park and Alex Alderton, Roberto Amato, Sonia Goncalves, Ewan Harrison, David K. Jackson, Ian Johnston, Dominic Kwiatkowski, Cordelia Langford, John Sillitoe on behalf of the Wellcome Sanger Institute COVID-19 Surveillance Team
EPI_ISL_756521, EPI_ISL_756522	Lighthouse Lab in Cambridge	Wellcome Sanger Institute for the COVID-19 Genomics UK (COG-UK) Consortium	Rob Howes, The Lighthouse Lab in Cambridge and Alex Alderton, Roberto Amato, Sonia Goncalves, Ewan Harrison, David K. Jackson, Ian Johnston, Dominic Kwiatkowski, Cordelia Langford, John Sillitoe on behalf of the Wellcome Sanger Institute COVID-19 Surveillance Team
EPI_ISL_756524	Lighthouse Lab in Alderley Park	Wellcome Sanger Institute for the COVID-19 Genomics UK (COG-UK) Consortium	Jacquelyn Wynn, Mairead Hyland, The Lighthouse Lab in Alderley Park and Alex Alderton, Roberto Amato, Sonia Goncalves, Ewan Harrison, David K. Jackson, Ian Johnston, Dominic Kwiatkowski, Cordelia Langford, John Sillitoe on behalf of the Wellcome Sanger Institute COVID-19 Surveillance Team
EPI_ISL_756525	Lighthouse Lab in Cambridge	Wellcome Sanger Institute for the COVID-19 Genomics UK (COG-UK) Consortium	Rob Howes, The Lighthouse Lab in Cambridge and Alex Alderton, Roberto Amato, Sonia Goncalves, Ewan Harrison, David K. Jackson, Ian Johnston, Dominic Kwiatkowski, Cordelia Langford, John Sillitoe on behalf of the Wellcome Sanger Institute COVID-19 Surveillance Team
EPI_ISL_756527	Lighthouse Lab in Alderley Park	Wellcome Sanger Institute for the COVID-19 Genomics UK (COG-UK) Consortium	Jacquelyn Wynn, Mairead Hyland, The Lighthouse Lab in Alderley Park and Alex Alderton, Roberto Amato, Sonia Goncalves, Ewan Harrison, David K. Jackson, Ian Johnston, Dominic Kwiatkowski, Cordelia Langford, John Sillitoe on behalf of the Wellcome Sanger Institute COVID-19 Surveillance Team
EPI_ISL_756528	Lighthouse Lab in Cambridge	Wellcome Sanger Institute for the COVID-19 Genomics UK (COG-UK) Consortium	Rob Howes, The Lighthouse Lab in Cambridge and Alex Alderton, Roberto Amato, Sonia Goncalves, Ewan Harrison, David K. Jackson, Ian Johnston, Dominic Kwiatkowski, Cordelia Langford, John Sillitoe on behalf of the Wellcome Sanger Institute COVID-19 Surveillance Team
EPI_ISL_756530, EPI_ISL_756531, EPI_ISL_756532	Lighthouse Lab in Alderley Park	Wellcome Sanger Institute for the COVID-19 Genomics UK (COG-UK) Consortium	Jacquelyn Wynn, Mairead Hyland, The Lighthouse Lab in Alderley Park and Alex Alderton, Roberto Amato, Sonia Goncalves, Ewan Harrison, David K. Jackson, Ian Johnston, Dominic Kwiatkowski, Cordelia Langford, John Sillitoe on behalf of the Wellcome Sanger Institute COVID-19 Surveillance Team
EPI_ISL_756534, EPI_ISL_756535	Lighthouse Lab in Cambridge	Wellcome Sanger Institute for the COVID-19 Genomics UK (COG-UK) Consortium	Rob Howes, The Lighthouse Lab in Cambridge and Alex Alderton, Roberto Amato, Sonia Goncalves, Ewan Harrison, David K. Jackson, Ian Johnston, Dominic Kwiatkowski, Cordelia Langford, John Sillitoe on behalf of the Wellcome Sanger Institute COVID-19 Surveillance Team
EPI_ISL_756536, EPI_ISL_756538, EPI_ISL_756539, EPI_ISL_756541	Lighthouse Lab in Alderley Park	Wellcome Sanger Institute for the COVID-19 Genomics UK (COG-UK) Consortium	Jacquelyn Wynn, Mairead Hyland, The Lighthouse Lab in Alderley Park and Alex Alderton, Roberto Amato, Sonia Goncalves, Ewan Harrison, David K. Jackson, Ian Johnston, Dominic Kwiatkowski, Cordelia Langford, John Sillitoe on behalf of the Wellcome Sanger Institute COVID-19 Surveillance Team
EPI_ISL_756542	Lighthouse Lab in Cambridge	Wellcome Sanger Institute for the COVID-19 Genomics UK (COG-UK) Consortium	Rob Howes, The Lighthouse Lab in Cambridge and Alex Alderton, Roberto Amato, Sonia Goncalves, Ewan Harrison, David K. Jackson, Ian Johnston, Dominic Kwiatkowski, Cordelia Langford, John Sillitoe on behalf of the Wellcome Sanger Institute COVID-19 Surveillance Team
EPI_ISL_756544	Lighthouse Lab in Alderley Park	Wellcome Sanger Institute for the COVID-19 Genomics UK (COG-UK) Consortium	Jacquelyn Wynn, Mairead Hyland, The Lighthouse Lab in Alderley Park and Alex Alderton, Roberto Amato, Sonia Goncalves, Ewan Harrison, David K. Jackson, Ian Johnston, Dominic Kwiatkowski, Cordelia Langford, John Sillitoe on behalf of the Wellcome Sanger Institute COVID-19 Surveillance Team
EPI_ISL_756545	Lighthouse Lab in Cambridge	Wellcome Sanger Institute for the COVID-19 Genomics UK (COG-UK) Consortium	Rob Howes, The Lighthouse Lab in Cambridge and Alex Alderton, Roberto Amato, Sonia Goncalves, Ewan Harrison, David K. Jackson, Ian Johnston, Dominic Kwiatkowski, Cordelia Langford, John Sillitoe on behalf of the Wellcome Sanger Institute COVID-19 Surveillance Team
EPI_ISL_756546, EPI_ISL_756550, EPI_ISL_756551, EPI_ISL_756552	Lighthouse Lab in Alderley Park	Wellcome Sanger Institute for the COVID-19 Genomics UK (COG-UK) Consortium	Jacquelyn Wynn, Mairead Hyland, The Lighthouse Lab in Alderley Park and Alex Alderton, Roberto Amato, Sonia Goncalves, Ewan Harrison, David K. Jackson, Ian Johnston, Dominic Kwiatkowski, Cordelia Langford, John Sillitoe on behalf of the Wellcome Sanger Institute COVID-19 Surveillance Team
EPI_ISL_756554	Lighthouse Lab in Cambridge	Wellcome Sanger Institute for the COVID-19 Genomics UK (COG-UK) Consortium	Rob Howes, The Lighthouse Lab in Cambridge and Alex Alderton, Roberto Amato, Sonia Goncalves, Ewan Harrison, David K. Jackson, Ian Johnston, Dominic Kwiatkowski, Cordelia Langford, John Sillitoe on behalf of the Wellcome Sanger Institute COVID-19 Surveillance Team
EPI_ISL_756555, EPI_ISL_756556	Lighthouse Lab in Alderley Park	Wellcome Sanger Institute for the COVID-19 Genomics UK (COG-UK) Consortium	Jacquelyn Wynn, Mairead Hyland, The Lighthouse Lab in Alderley Park and Alex Alderton, Roberto Amato, Sonia Goncalves, Ewan Harrison, David K. Jackson, Ian Johnston, Dominic Kwiatkowski, Cordelia Langford, John Sillitoe on behalf of the Wellcome Sanger Institute COVID-19 Surveillance Team
EPI_ISL_756558, EPI_ISL_756559	Lighthouse Lab in Cambridge	Wellcome Sanger Institute for the COVID-19 Genomics UK (COG-UK) Consortium	Rob Howes, The Lighthouse Lab in Cambridge and Alex Alderton, Roberto Amato, Sonia Goncalves, Ewan Harrison, David K. Jackson, Ian Johnston, Dominic Kwiatkowski, Cordelia Langford, John Sillitoe on behalf of the Wellcome Sanger Institute COVID-19 Surveillance Team
EPI_ISL_756560, EPI_ISL_756562, EPI_ISL_756563, EPI_ISL_756565, EPI_ISL_756566	Lighthouse Lab in Alderley Park	Wellcome Sanger Institute for the COVID-19 Genomics UK (COG-UK) Consortium	Jacquelyn Wynn, Mairead Hyland, The Lighthouse Lab in Alderley Park and Alex Alderton, Roberto Amato, Sonia Goncalves, Ewan Harrison, David K. Jackson, Ian Johnston, Dominic Kwiatkowski, Cordelia Langford, John Sillitoe on behalf of the Wellcome Sanger Institute COVID-19 Surveillance Team
EPI_ISL_756567, EPI_ISL_756568	Lighthouse Lab in Cambridge	Wellcome Sanger Institute for the COVID-19 Genomics UK (COG-UK) Consortium	Rob Howes, The Lighthouse Lab in Cambridge and Alex Alderton, Roberto Amato, Sonia Goncalves, Ewan Harrison, David K. Jackson, Ian Johnston, Dominic Kwiatkowski, Cordelia Langford, John Sillitoe on behalf of the Wellcome Sanger Institute COVID-19 Surveillance Team

EPI_ISL_756569, EPI_ISL_756571	Lighthouse Lab in Alderley Park	Wellcome Sanger Institute for the COVID-19 Genomics UK (COG-UK) Consortium	Jacquelyn Wynn, Mairead Hyland, The Lighthouse Lab in Alderley Park and Alex Alderton, Roberto Amato, Sonia Goncalves, Ewan Harrison, David K. Jackson, Ian Johnston, Dominic Kwiatkowski, Cordelia Langford, John Sillitoe on behalf of the Wellcome Sanger Institute COVID-19 Surveillance Team
EPI_ISL_756572, EPI_ISL_756579	Lighthouse Lab in Cambridge	Wellcome Sanger Institute for the COVID-19 Genomics UK (COG-UK) Consortium	Rob Howes, The Lighthouse Lab in Cambridge and Alex Alderton, Roberto Amato, Sonia Goncalves, Ewan Harrison, David K. Jackson, Ian Johnston, Dominic Kwiatkowski, Cordelia Langford, John Sillitoe on behalf of the Wellcome Sanger Institute COVID-19 Surveillance Team
EPI_ISL_756580	Lighthouse Lab in Alderley Park	Wellcome Sanger Institute for the COVID-19 Genomics UK (COG-UK) Consortium	Jacquelyn Wynn, Mairead Hyland, The Lighthouse Lab in Alderley Park and Alex Alderton, Roberto Amato, Sonia Goncalves, Ewan Harrison, David K. Jackson, Ian Johnston, Dominic Kwiatkowski, Cordelia Langford, John Sillitoe on behalf of the Wellcome Sanger Institute COVID-19 Surveillance Team
EPI_ISL_756582	Lighthouse Lab in Cambridge	Wellcome Sanger Institute for the COVID-19 Genomics UK (COG-UK) Consortium	Rob Howes, The Lighthouse Lab in Cambridge and Alex Alderton, Roberto Amato, Sonia Goncalves, Ewan Harrison, David K. Jackson, Ian Johnston, Dominic Kwiatkowski, Cordelia Langford, John Sillitoe on behalf of the Wellcome Sanger Institute COVID-19 Surveillance Team
EPI_ISL_756583	Lighthouse Lab in Alderley Park	Wellcome Sanger Institute for the COVID-19 Genomics UK (COG-UK) Consortium	Jacquelyn Wynn, Mairead Hyland, The Lighthouse Lab in Alderley Park and Alex Alderton, Roberto Amato, Sonia Goncalves, Ewan Harrison, David K. Jackson, Ian Johnston, Dominic Kwiatkowski, Cordelia Langford, John Sillitoe on behalf of the Wellcome Sanger Institute COVID-19 Surveillance Team
EPI_ISL_756585, EPI_ISL_756586	Lighthouse Lab in Cambridge	Wellcome Sanger Institute for the COVID-19 Genomics UK (COG-UK) Consortium	Rob Howes, The Lighthouse Lab in Cambridge and Alex Alderton, Roberto Amato, Sonia Goncalves, Ewan Harrison, David K. Jackson, Ian Johnston, Dominic Kwiatkowski, Cordelia Langford, John Sillitoe on behalf of the Wellcome Sanger Institute COVID-19 Surveillance Team
EPI_ISL_756587	Lighthouse Lab in Alderley Park	Wellcome Sanger Institute for the COVID-19 Genomics UK (COG-UK) Consortium	Jacquelyn Wynn, Mairead Hyland, The Lighthouse Lab in Alderley Park and Alex Alderton, Roberto Amato, Sonia Goncalves, Ewan Harrison, David K. Jackson, Ian Johnston, Dominic Kwiatkowski, Cordelia Langford, John Sillitoe on behalf of the Wellcome Sanger Institute COVID-19 Surveillance Team
EPI_ISL_756588, EPI_ISL_756589, EPI_ISL_756590, EPI_ISL_756591	Lighthouse Lab in Cambridge	Wellcome Sanger Institute for the COVID-19 Genomics UK (COG-UK) Consortium	Rob Howes, The Lighthouse Lab in Cambridge and Alex Alderton, Roberto Amato, Sonia Goncalves, Ewan Harrison, David K. Jackson, Ian Johnston, Dominic Kwiatkowski, Cordelia Langford, John Sillitoe on behalf of the Wellcome Sanger Institute COVID-19 Surveillance Team
EPI_ISL_756592	Lighthouse Lab in Alderley Park	Wellcome Sanger Institute for the COVID-19 Genomics UK (COG-UK) Consortium	Jacquelyn Wynn, Mairead Hyland, The Lighthouse Lab in Alderley Park and Alex Alderton, Roberto Amato, Sonia Goncalves, Ewan Harrison, David K. Jackson, Ian Johnston, Dominic Kwiatkowski, Cordelia Langford, John Sillitoe on behalf of the Wellcome Sanger Institute COVID-19 Surveillance Team
EPI_ISL_756594	Lighthouse Lab in Cambridge	Wellcome Sanger Institute for the COVID-19 Genomics UK (COG-UK) Consortium	Rob Howes, The Lighthouse Lab in Cambridge and Alex Alderton, Roberto Amato, Sonia Goncalves, Ewan Harrison, David K. Jackson, Ian Johnston, Dominic Kwiatkowski, Cordelia Langford, John Sillitoe on behalf of the Wellcome Sanger Institute COVID-19 Surveillance Team
EPI_ISL_756596, EPI_ISL_756598, EPI_ISL_756599, EPI_ISL_756600, EPI_ISL_756602, EPI_ISL_756603	Lighthouse Lab in Alderley Park	Wellcome Sanger Institute for the COVID-19 Genomics UK (COG-UK) Consortium	Jacquelyn Wynn, Mairead Hyland, The Lighthouse Lab in Alderley Park and Alex Alderton, Roberto Amato, Sonia Goncalves, Ewan Harrison, David K. Jackson, Ian Johnston, Dominic Kwiatkowski, Cordelia Langford, John Sillitoe on behalf of the Wellcome Sanger Institute COVID-19 Surveillance Team
EPI_ISL_756604, EPI_ISL_756607, EPI_ISL_756608	Lighthouse Lab in Cambridge	Wellcome Sanger Institute for the COVID-19 Genomics UK (COG-UK) Consortium	Rob Howes, The Lighthouse Lab in Cambridge and Alex Alderton, Roberto Amato, Sonia Goncalves, Ewan Harrison, David K. Jackson, Ian Johnston, Dominic Kwiatkowski, Cordelia Langford, John Sillitoe on behalf of the Wellcome Sanger Institute COVID-19 Surveillance Team
EPI_ISL_756609	Lighthouse Lab in Alderley Park	Wellcome Sanger Institute for the COVID-19 Genomics UK (COG-UK) Consortium	Jacquelyn Wynn, Mairead Hyland, The Lighthouse Lab in Alderley Park and Alex Alderton, Roberto Amato, Sonia Goncalves, Ewan Harrison, David K. Jackson, Ian Johnston, Dominic Kwiatkowski, Cordelia Langford, John Sillitoe on behalf of the Wellcome Sanger Institute COVID-19 Surveillance Team
EPI_ISL_756611	Lighthouse Lab in Cambridge	Wellcome Sanger Institute for the COVID-19 Genomics UK (COG-UK) Consortium	Rob Howes, The Lighthouse Lab in Cambridge and Alex Alderton, Roberto Amato, Sonia Goncalves, Ewan Harrison, David K. Jackson, Ian Johnston, Dominic Kwiatkowski, Cordelia Langford, John Sillitoe on behalf of the Wellcome Sanger Institute COVID-19 Surveillance Team
EPI_ISL_756612	Lighthouse Lab in Alderley Park	Wellcome Sanger Institute for the COVID-19 Genomics UK (COG-UK) Consortium	Jacquelyn Wynn, Mairead Hyland, The Lighthouse Lab in Alderley Park and Alex Alderton, Roberto Amato, Sonia Goncalves, Ewan Harrison, David K. Jackson, Ian Johnston, Dominic Kwiatkowski, Cordelia Langford, John Sillitoe on behalf of the Wellcome Sanger Institute COVID-19 Surveillance Team
EPI_ISL_756613, EPI_ISL_756615	Lighthouse Lab in Cambridge	Wellcome Sanger Institute for the COVID-19 Genomics UK (COG-UK) Consortium	Rob Howes, The Lighthouse Lab in Cambridge and Alex Alderton, Roberto Amato, Sonia Goncalves, Ewan Harrison, David K. Jackson, Ian Johnston, Dominic Kwiatkowski, Cordelia Langford, John Sillitoe on behalf of the Wellcome Sanger Institute COVID-19 Surveillance Team
EPI_ISL_756616	Lighthouse Lab in Alderley Park	Wellcome Sanger Institute for the COVID-19 Genomics UK (COG-UK) Consortium	Jacquelyn Wynn, Mairead Hyland, The Lighthouse Lab in Alderley Park and Alex Alderton, Roberto Amato, Sonia Goncalves, Ewan Harrison, David K. Jackson, Ian Johnston, Dominic Kwiatkowski, Cordelia Langford, John Sillitoe on behalf of the Wellcome Sanger Institute COVID-19 Surveillance Team
EPI_ISL_756617	Lighthouse Lab in Cambridge	Wellcome Sanger Institute for the COVID-19 Genomics UK (COG-UK) Consortium	Rob Howes, The Lighthouse Lab in Cambridge and Alex Alderton, Roberto Amato, Sonia Goncalves, Ewan Harrison, David K. Jackson, Ian Johnston, Dominic Kwiatkowski, Cordelia Langford, John Sillitoe on behalf of the Wellcome Sanger Institute COVID-19 Surveillance Team
EPI_ISL_756618, EPI_ISL_756620	Lighthouse Lab in Alderley Park	Wellcome Sanger Institute for the COVID-19 Genomics UK (COG-UK) Consortium	Jacquelyn Wynn, Mairead Hyland, The Lighthouse Lab in Alderley Park and Alex Alderton, Roberto Amato, Sonia Goncalves, Ewan Harrison, David K. Jackson, Ian Johnston, Dominic Kwiatkowski, Cordelia Langford, John Sillitoe on behalf of the Wellcome Sanger Institute COVID-19 Surveillance Team
EPI_ISL_756621, EPI_ISL_756622, EPI_ISL_756623	Lighthouse Lab in Cambridge	Wellcome Sanger Institute for the COVID-19 Genomics UK (COG-UK) Consortium	Rob Howes, The Lighthouse Lab in Cambridge and Alex Alderton, Roberto Amato, Sonia Goncalves, Ewan Harrison, David K. Jackson, Ian Johnston, Dominic Kwiatkowski, Cordelia Langford, John Sillitoe on behalf of the Wellcome Sanger Institute COVID-19 Surveillance Team
EPI_ISL_756624	Lighthouse Lab in Alderley Park	Wellcome Sanger Institute for the COVID-19 Genomics UK (COG-UK) Consortium	Jacquelyn Wynn, Mairead Hyland, The Lighthouse Lab in Alderley Park and Alex Alderton, Roberto Amato, Sonia Goncalves, Ewan Harrison, David K. Jackson, Ian Johnston, Dominic Kwiatkowski, Cordelia Langford, John Sillitoe on behalf of the Wellcome Sanger Institute COVID-19 Surveillance Team
EPI_ISL_756628, EPI_ISL_756630, EPI_ISL_756631	Lighthouse Lab in Cambridge	Wellcome Sanger Institute for the COVID-19 Genomics UK (COG-UK) Consortium	Rob Howes, The Lighthouse Lab in Cambridge and Alex Alderton, Roberto Amato, Sonia Goncalves, Ewan Harrison, David K. Jackson, Ian Johnston, Dominic Kwiatkowski, Cordelia Langford, John Sillitoe on behalf of the Wellcome Sanger Institute COVID-19 Surveillance Team
EPI_ISL_756634, EPI_ISL_756635, EPI_ISL_756636	Lighthouse Lab in Alderley Park	Wellcome Sanger Institute for the COVID-19 Genomics UK (COG-UK) Consortium	Jacquelyn Wynn, Mairead Hyland, The Lighthouse Lab in Alderley Park and Alex Alderton, Roberto Amato, Sonia Goncalves, Ewan Harrison, David K. Jackson, Ian Johnston, Dominic Kwiatkowski, Cordelia Langford, John Sillitoe on behalf of the Wellcome Sanger Institute COVID-19 Surveillance Team
EPI_ISL_756637	Lighthouse Lab in Cambridge	Wellcome Sanger Institute for the COVID-19 Genomics UK (COG-UK) Consortium	Rob Howes, The Lighthouse Lab in Cambridge and Alex Alderton, Roberto Amato, Sonia Goncalves, Ewan Harrison, David K. Jackson, Ian Johnston, Dominic Kwiatkowski, Cordelia Langford, John Sillitoe on behalf of the Wellcome Sanger Institute COVID-19 Surveillance Team
EPI_ISL_756638, EPI_ISL_756639, EPI_ISL_756640, EPI_ISL_756641	Lighthouse Lab in Alderley Park	Wellcome Sanger Institute for the COVID-19 Genomics UK (COG-UK) Consortium	Jacquelyn Wynn, Mairead Hyland, The Lighthouse Lab in Alderley Park and Alex Alderton, Roberto Amato, Sonia Goncalves, Ewan Harrison, David K. Jackson, Ian Johnston, Dominic Kwiatkowski, Cordelia Langford, John Sillitoe on behalf of the Wellcome Sanger Institute COVID-19 Surveillance Team
EPI_ISL_756642	Lighthouse Lab in Cambridge	Wellcome Sanger Institute for the COVID-19 Genomics UK (COG-UK) Consortium	Rob Howes, The Lighthouse Lab in Cambridge and Alex Alderton, Roberto Amato, Sonia Goncalves, Ewan Harrison, David K. Jackson, Ian Johnston, Dominic Kwiatkowski, Cordelia Langford, John Sillitoe on behalf of the Wellcome Sanger Institute COVID-19 Surveillance Team
EPI_ISL_756644, EPI_ISL_756645, EPI_ISL_756648	Lighthouse Lab in Alderley Park	Wellcome Sanger Institute for the COVID-19 Genomics UK (COG-UK) Consortium	Jacquelyn Wynn, Mairead Hyland, The Lighthouse Lab in Alderley Park and Alex Alderton, Roberto Amato, Sonia Goncalves, Ewan Harrison, David K. Jackson, Ian Johnston, Dominic Kwiatkowski, Cordelia Langford, John Sillitoe on behalf of the Wellcome Sanger Institute COVID-19 Surveillance Team
EPI_ISL_756649	Lighthouse Lab in Cambridge	Wellcome Sanger Institute for the COVID-19 Genomics UK (COG-UK) Consortium	Rob Howes, The Lighthouse Lab in Cambridge and Alex Alderton, Roberto Amato, Sonia Goncalves, Ewan Harrison, David K. Jackson, Ian Johnston, Dominic Kwiatkowski, Cordelia Langford, John Sillitoe on behalf of the Wellcome Sanger Institute COVID-19 Surveillance Team
EPI_ISL_756650, EPI_ISL_756654	Lighthouse Lab in Alderley Park	Wellcome Sanger Institute for the COVID-19 Genomics UK (COG-UK) Consortium	Jacquelyn Wynn, Mairead Hyland, The Lighthouse Lab in Alderley Park and Alex Alderton, Roberto Amato, Sonia Goncalves, Ewan Harrison, David K. Jackson, Ian Johnston, Dominic Kwiatkowski, Cordelia Langford, John Sillitoe on behalf of the Wellcome Sanger Institute COVID-19 Surveillance Team
EPI_ISL_756655	Lighthouse Lab in Cambridge	Wellcome Sanger Institute for the COVID-19 Genomics UK (COG-UK) Consortium	Rob Howes, The Lighthouse Lab in Cambridge and Alex Alderton, Roberto Amato, Sonia Goncalves, Ewan Harrison, David K. Jackson, Ian Johnston, Dominic Kwiatkowski, Cordelia Langford, John Sillitoe on behalf of the Wellcome Sanger Institute COVID-19 Surveillance Team
EPI_ISL_756656, EPI_ISL_756657, EPI_ISL_756658	Lighthouse Lab in Alderley Park	Wellcome Sanger Institute for the COVID-19 Genomics UK (COG-UK) Consortium	Jacquelyn Wynn, Mairead Hyland, The Lighthouse Lab in Alderley Park and Alex Alderton, Roberto Amato, Sonia Goncalves, Ewan Harrison, David K. Jackson, Ian Johnston, Dominic Kwiatkowski, Cordelia Langford, John Sillitoe on behalf of the Wellcome Sanger Institute COVID-19 Surveillance Team
EPI_ISL_756659, EPI_ISL_756660	Lighthouse Lab in Cambridge	Wellcome Sanger Institute for the COVID-19 Genomics UK (COG-UK) Consortium	Rob Howes, The Lighthouse Lab in Cambridge and Alex Alderton, Roberto Amato, Sonia Goncalves, Ewan Harrison, David K. Jackson, Ian Johnston, Dominic Kwiatkowski, Cordelia Langford, John Sillitoe on behalf of the Wellcome Sanger Institute COVID-19 Surveillance Team
EPI_ISL_756661, EPI_ISL_756662, EPI_ISL_756663, EPI_ISL_756664	Lighthouse Lab in Alderley Park	Wellcome Sanger Institute for the COVID-19 Genomics UK (COG-UK) Consortium	Jacquelyn Wynn, Mairead Hyland, The Lighthouse Lab in Alderley Park and Alex Alderton, Roberto Amato, Sonia Goncalves, Ewan Harrison, David K. Jackson, Ian Johnston, Dominic Kwiatkowski, Cordelia Langford, John Sillitoe on behalf of the Wellcome Sanger Institute COVID-19 Surveillance Team
EPI_ISL_756665, EPI_ISL_756666, EPI_ISL_756667, EPI_ISL_756668	Lighthouse Lab in Cambridge	Wellcome Sanger Institute for the COVID-19 Genomics UK (COG-UK) Consortium	Rob Howes, The Lighthouse Lab in Cambridge and Alex Alderton, Roberto Amato, Sonia Goncalves, Ewan Harrison, David K. Jackson, Ian Johnston, Dominic Kwiatkowski, Cordelia Langford, John Sillitoe on behalf of the Wellcome Sanger Institute COVID-19 Surveillance Team
EPI_ISL_756669, EPI_ISL_756670	Lighthouse Lab in Alderley Park	Wellcome Sanger Institute for the COVID-19 Genomics UK	Jacquelyn Wynn, Mairead Hyland, The Lighthouse Lab in Alderley Park and Alex Alderton, Roberto Amato, Sonia Goncalves, Ewan Harrison, David K.

		(COG-UK) Consortium	Jackson, Ian Johnston, Dominic Kwiatkowski, Cordelia Langford, John Sillitoe on behalf of the Wellcome Sanger Institute COVID-19 Surveillance Team
EPI_ISL_756671, EPI_ISL_756672	Lighthouse Lab in Cambridge	Wellcome Sanger Institute for the COVID-19 Genomics UK (COG-UK) Consortium	Rob Howes, The Lighthouse Lab in Cambridge and Alex Alderton, Roberto Amato, Sonia Goncalves, Ewan Harrison, David K. Jackson, Ian Johnston, Dominic Kwiatkowski, Cordelia Langford, John Sillitoe on behalf of the Wellcome Sanger Institute COVID-19 Surveillance Team
EPI_ISL_756673	Lighthouse Lab in Alderley Park	Wellcome Sanger Institute for the COVID-19 Genomics UK (COG-UK) Consortium	Jacquelyn Wynn, Mairead Hyland, The Lighthouse Lab in Alderley Park and Alex Alderton, Roberto Amato, Sonia Goncalves, Ewan Harrison, David K. Jackson, Ian Johnston, Dominic Kwiatkowski, Cordelia Langford, John Sillitoe on behalf of the Wellcome Sanger Institute COVID-19 Surveillance Team
EPI_ISL_756674, EPI_ISL_756677, EPI_ISL_756678, EPI_ISL_756679, EPI_ISL_756680	Lighthouse Lab in Cambridge	Wellcome Sanger Institute for the COVID-19 Genomics UK (COG-UK) Consortium	Rob Howes, The Lighthouse Lab in Cambridge and Alex Alderton, Roberto Amato, Sonia Goncalves, Ewan Harrison, David K. Jackson, Ian Johnston, Dominic Kwiatkowski, Cordelia Langford, John Sillitoe on behalf of the Wellcome Sanger Institute COVID-19 Surveillance Team
EPI_ISL_756681, EPI_ISL_756682, EPI_ISL_756683, EPI_ISL_756685	Lighthouse Lab in Alderley Park	Wellcome Sanger Institute for the COVID-19 Genomics UK (COG-UK) Consortium	Jacquelyn Wynn, Mairead Hyland, The Lighthouse Lab in Alderley Park and Alex Alderton, Roberto Amato, Sonia Goncalves, Ewan Harrison, David K. Jackson, Ian Johnston, Dominic Kwiatkowski, Cordelia Langford, John Sillitoe on behalf of the Wellcome Sanger Institute COVID-19 Surveillance Team
EPI_ISL_756690	Lighthouse Lab in Glasgow	Wellcome Sanger Institute for the COVID-19 Genomics UK (COG-UK) Consortium	Harper VanSteenhouse, Yumi Kasai, David Gray, Carol Clugston, Anna Dominiczak and Alex Alderton, Roberto Amato, Sonia Goncalves, Ewan Harrison, David K. Jackson, Ian Johnston, Dominic Kwiatkowski, Cordelia Langford, John Sillitoe on behalf of the Wellcome Sanger Institute COVID-19 Surveillance Team
EPI_ISL_756691, EPI_ISL_756692	Lighthouse Lab in Milton Keynes	Wellcome Sanger Institute for the COVID-19 Genomics UK (COG-UK) Consortium	The Lighthouse Lab in Milton Keynes and Alex Alderton, Roberto Amato, Sonia Goncalves, Ewan Harrison, David K. Jackson, Ian Johnston, Dominic Kwiatkowski, Cordelia Langford, John Sillitoe on behalf of the Wellcome Sanger Institute COVID-19 Surveillance Team
EPI_ISL_756695	Lighthouse Lab in Glasgow	Wellcome Sanger Institute for the COVID-19 Genomics UK (COG-UK) Consortium	Harper VanSteenhouse, Yumi Kasai, David Gray, Carol Clugston, Anna Dominiczak and Alex Alderton, Roberto Amato, Sonia Goncalves, Ewan Harrison, David K. Jackson, Ian Johnston, Dominic Kwiatkowski, Cordelia Langford, John Sillitoe on behalf of the Wellcome Sanger Institute COVID-19 Surveillance Team
EPI_ISL_756698, EPI_ISL_756701	Lighthouse Lab in Milton Keynes	Wellcome Sanger Institute for the COVID-19 Genomics UK (COG-UK) Consortium	The Lighthouse Lab in Milton Keynes and Alex Alderton, Roberto Amato, Sonia Goncalves, Ewan Harrison, David K. Jackson, Ian Johnston, Dominic Kwiatkowski, Cordelia Langford, John Sillitoe on behalf of the Wellcome Sanger Institute COVID-19 Surveillance Team
EPI_ISL_756702	Lighthouse Lab in Glasgow	Wellcome Sanger Institute for the COVID-19 Genomics UK (COG-UK) Consortium	Harper VanSteenhouse, Yumi Kasai, David Gray, Carol Clugston, Anna Dominiczak and Alex Alderton, Roberto Amato, Sonia Goncalves, Ewan Harrison, David K. Jackson, Ian Johnston, Dominic Kwiatkowski, Cordelia Langford, John Sillitoe on behalf of the Wellcome Sanger Institute COVID-19 Surveillance Team
EPI_ISL_756703	Lighthouse Lab in Milton Keynes	Wellcome Sanger Institute for the COVID-19 Genomics UK (COG-UK) Consortium	The Lighthouse Lab in Milton Keynes and Alex Alderton, Roberto Amato, Sonia Goncalves, Ewan Harrison, David K. Jackson, Ian Johnston, Dominic Kwiatkowski, Cordelia Langford, John Sillitoe on behalf of the Wellcome Sanger Institute COVID-19 Surveillance Team
EPI_ISL_756705, EPI_ISL_756706	Lighthouse Lab in Glasgow	Wellcome Sanger Institute for the COVID-19 Genomics UK (COG-UK) Consortium	Harper VanSteenhouse, Yumi Kasai, David Gray, Carol Clugston, Anna Dominiczak and Alex Alderton, Roberto Amato, Sonia Goncalves, Ewan Harrison, David K. Jackson, Ian Johnston, Dominic Kwiatkowski, Cordelia Langford, John Sillitoe on behalf of the Wellcome Sanger Institute COVID-19 Surveillance Team
EPI_ISL_756707, EPI_ISL_756708	Lighthouse Lab in Milton Keynes	Wellcome Sanger Institute for the COVID-19 Genomics UK (COG-UK) Consortium	The Lighthouse Lab in Milton Keynes and Alex Alderton, Roberto Amato, Sonia Goncalves, Ewan Harrison, David K. Jackson, Ian Johnston, Dominic Kwiatkowski, Cordelia Langford, John Sillitoe on behalf of the Wellcome Sanger Institute COVID-19 Surveillance Team
EPI_ISL_756709	Lighthouse Lab in Glasgow	Wellcome Sanger Institute for the COVID-19 Genomics UK (COG-UK) Consortium	Harper VanSteenhouse, Yumi Kasai, David Gray, Carol Clugston, Anna Dominiczak and Alex Alderton, Roberto Amato, Sonia Goncalves, Ewan Harrison, David K. Jackson, Ian Johnston, Dominic Kwiatkowski, Cordelia Langford, John Sillitoe on behalf of the Wellcome Sanger Institute COVID-19 Surveillance Team
EPI_ISL_756710, EPI_ISL_756712, EPI_ISL_756713, EPI_ISL_756714, EPI_ISL_756715	Lighthouse Lab in Milton Keynes	Wellcome Sanger Institute for the COVID-19 Genomics UK (COG-UK) Consortium	The Lighthouse Lab in Milton Keynes and Alex Alderton, Roberto Amato, Sonia Goncalves, Ewan Harrison, David K. Jackson, Ian Johnston, Dominic Kwiatkowski, Cordelia Langford, John Sillitoe on behalf of the Wellcome Sanger Institute COVID-19 Surveillance Team
EPI_ISL_756716	Lighthouse Lab in Glasgow	Wellcome Sanger Institute for the COVID-19 Genomics UK (COG-UK) Consortium	Harper VanSteenhouse, Yumi Kasai, David Gray, Carol Clugston, Anna Dominiczak and Alex Alderton, Roberto Amato, Sonia Goncalves, Ewan Harrison, David K. Jackson, Ian Johnston, Dominic Kwiatkowski, Cordelia Langford, John Sillitoe on behalf of the Wellcome Sanger Institute COVID-19 Surveillance Team
EPI_ISL_756718, EPI_ISL_756722	Lighthouse Lab in Milton Keynes	Wellcome Sanger Institute for the COVID-19 Genomics UK (COG-UK) Consortium	The Lighthouse Lab in Milton Keynes and Alex Alderton, Roberto Amato, Sonia Goncalves, Ewan Harrison, David K. Jackson, Ian Johnston, Dominic Kwiatkowski, Cordelia Langford, John Sillitoe on behalf of the Wellcome Sanger Institute COVID-19 Surveillance Team
EPI_ISL_756723	Lighthouse Lab in Glasgow	Wellcome Sanger Institute for the COVID-19 Genomics UK (COG-UK) Consortium	Harper VanSteenhouse, Yumi Kasai, David Gray, Carol Clugston, Anna Dominiczak and Alex Alderton, Roberto Amato, Sonia Goncalves, Ewan Harrison, David K. Jackson, Ian Johnston, Dominic Kwiatkowski, Cordelia Langford, John Sillitoe on behalf of the Wellcome Sanger Institute COVID-19 Surveillance Team
EPI_ISL_756725, EPI_ISL_756726, EPI_ISL_756727, EPI_ISL_756728	Lighthouse Lab in Milton Keynes	Wellcome Sanger Institute for the COVID-19 Genomics UK (COG-UK) Consortium	The Lighthouse Lab in Milton Keynes and Alex Alderton, Roberto Amato, Sonia Goncalves, Ewan Harrison, David K. Jackson, Ian Johnston, Dominic Kwiatkowski, Cordelia Langford, John Sillitoe on behalf of the Wellcome Sanger Institute COVID-19 Surveillance Team
EPI_ISL_756729	Lighthouse Lab in Glasgow	Wellcome Sanger Institute for the COVID-19 Genomics UK (COG-UK) Consortium	Harper VanSteenhouse, Yumi Kasai, David Gray, Carol Clugston, Anna Dominiczak and Alex Alderton, Roberto Amato, Sonia Goncalves, Ewan Harrison, David K. Jackson, Ian Johnston, Dominic Kwiatkowski, Cordelia Langford, John Sillitoe on behalf of the Wellcome Sanger Institute COVID-19 Surveillance Team
EPI_ISL_756732, EPI_ISL_756733, EPI_ISL_756734, EPI_ISL_756735	Lighthouse Lab in Milton Keynes	Wellcome Sanger Institute for the COVID-19 Genomics UK (COG-UK) Consortium	The Lighthouse Lab in Milton Keynes and Alex Alderton, Roberto Amato, Sonia Goncalves, Ewan Harrison, David K. Jackson, Ian Johnston, Dominic Kwiatkowski, Cordelia Langford, John Sillitoe on behalf of the Wellcome Sanger Institute COVID-19 Surveillance Team
EPI_ISL_756737	Lighthouse Lab in Glasgow	Wellcome Sanger Institute for the COVID-19 Genomics UK (COG-UK) Consortium	Harper VanSteenhouse, Yumi Kasai, David Gray, Carol Clugston, Anna Dominiczak and Alex Alderton, Roberto Amato, Sonia Goncalves, Ewan Harrison, David K. Jackson, Ian Johnston, Dominic Kwiatkowski, Cordelia Langford, John Sillitoe on behalf of the Wellcome Sanger Institute COVID-19 Surveillance Team
EPI_ISL_756738, EPI_ISL_756739, EPI_ISL_756740, EPI_ISL_756741, EPI_ISL_756742, EPI_ISL_756743	Lighthouse Lab in Milton Keynes	Wellcome Sanger Institute for the COVID-19 Genomics UK (COG-UK) Consortium	The Lighthouse Lab in Milton Keynes and Alex Alderton, Roberto Amato, Sonia Goncalves, Ewan Harrison, David K. Jackson, Ian Johnston, Dominic Kwiatkowski, Cordelia Langford, John Sillitoe on behalf of the Wellcome Sanger Institute COVID-19 Surveillance Team
EPI_ISL_756744, EPI_ISL_756745	Lighthouse Lab in Glasgow	Wellcome Sanger Institute for the COVID-19 Genomics UK (COG-UK) Consortium	Harper VanSteenhouse, Yumi Kasai, David Gray, Carol Clugston, Anna Dominiczak and Alex Alderton, Roberto Amato, Sonia Goncalves, Ewan Harrison, David K. Jackson, Ian Johnston, Dominic Kwiatkowski, Cordelia Langford, John Sillitoe on behalf of the Wellcome Sanger Institute COVID-19 Surveillance Team
EPI_ISL_756746	Lighthouse Lab in Milton Keynes	Wellcome Sanger Institute for the COVID-19 Genomics UK (COG-UK) Consortium	The Lighthouse Lab in Milton Keynes and Alex Alderton, Roberto Amato, Sonia Goncalves, Ewan Harrison, David K. Jackson, Ian Johnston, Dominic Kwiatkowski, Cordelia Langford, John Sillitoe on behalf of the Wellcome Sanger Institute COVID-19 Surveillance Team
EPI_ISL_756748	Lighthouse Lab in Glasgow	Wellcome Sanger Institute for the COVID-19 Genomics UK (COG-UK) Consortium	Harper VanSteenhouse, Yumi Kasai, David Gray, Carol Clugston, Anna Dominiczak and Alex Alderton, Roberto Amato, Sonia Goncalves, Ewan Harrison, David K. Jackson, Ian Johnston, Dominic Kwiatkowski, Cordelia Langford, John Sillitoe on behalf of the Wellcome Sanger Institute COVID-19 Surveillance Team
EPI_ISL_756749, EPI_ISL_756751, EPI_ISL_756752, EPI_ISL_756753, EPI_ISL_756754, EPI_ISL_756755, EPI_ISL_756756, EPI_ISL_756757, EPI_ISL_756758, EPI_ISL_756759, EPI_ISL_756760			
see above	Lighthouse Lab in Milton Keynes	Wellcome Sanger Institute for the COVID-19 Genomics UK (COG-UK) Consortium	The Lighthouse Lab in Milton Keynes and Alex Alderton, Roberto Amato, Sonia Goncalves, Ewan Harrison, David K. Jackson, Ian Johnston, Dominic Kwiatkowski, Cordelia Langford, John Sillitoe on behalf of the Wellcome Sanger Institute COVID-19 Surveillance Team
EPI_ISL_756761	Lighthouse Lab in Glasgow	Wellcome Sanger Institute for the COVID-19 Genomics UK (COG-UK) Consortium	Harper VanSteenhouse, Yumi Kasai, David Gray, Carol Clugston, Anna Dominiczak and Alex Alderton, Roberto Amato, Sonia Goncalves, Ewan Harrison, David K. Jackson, Ian Johnston, Dominic Kwiatkowski, Cordelia Langford, John Sillitoe on behalf of the Wellcome Sanger Institute COVID-19 Surveillance Team
EPI_ISL_756762, EPI_ISL_756763, EPI_ISL_756765, EPI_ISL_756766,	Lighthouse Lab in Milton Keynes	Wellcome Sanger Institute for the COVID-19 Genomics UK (COG-UK) Consortium	The Lighthouse Lab in Milton Keynes and Alex Alderton, Roberto Amato, Sonia Goncalves, Ewan Harrison, David K. Jackson, Ian Johnston, Dominic Kwiatkowski, Cordelia Langford, John Sillitoe on behalf of the Wellcome Sanger Institute COVID-19 Surveillance Team

EPI_ISL_756767, EPI_ISL_756768, EPI_ISL_756770, EPI_ISL_756771, EPI_ISL_756774			
EPI_ISL_756775	Lighthouse Lab in Glasgow	Wellcome Sanger Institute for the COVID-19 Genomics UK (COG-UK) Consortium	Harper VanSteenhouse, Yumi Kasai, David Gray, Carol Clugston, Anna Dominiczak and Alex Alderton, Roberto Amato, Sonia Goncalves, Ewan Harrison, David K. Jackson, Ian Johnston, Dominic Kwiatkowski, Cordelia Langford, John Sillitoe on behalf of the Wellcome Sanger Institute COVID-19 Surveillance Team
EPI_ISL_756777, EPI_ISL_756778	Lighthouse Lab in Milton Keynes	Wellcome Sanger Institute for the COVID-19 Genomics UK (COG-UK) Consortium	The Lighthouse Lab in Milton Keynes and Alex Alderton, Roberto Amato, Sonia Goncalves, Ewan Harrison, David K. Jackson, Ian Johnston, Dominic Kwiatkowski, Cordelia Langford, John Sillitoe on behalf of the Wellcome Sanger Institute COVID-19 Surveillance Team
EPI_ISL_756780	Lighthouse Lab in Glasgow	Wellcome Sanger Institute for the COVID-19 Genomics UK (COG-UK) Consortium	Harper VanSteenhouse, Yumi Kasai, David Gray, Carol Clugston, Anna Dominiczak and Alex Alderton, Roberto Amato, Sonia Goncalves, Ewan Harrison, David K. Jackson, Ian Johnston, Dominic Kwiatkowski, Cordelia Langford, John Sillitoe on behalf of the Wellcome Sanger Institute COVID-19 Surveillance Team
EPI_ISL_756781, EPI_ISL_756782	Lighthouse Lab in Milton Keynes	Wellcome Sanger Institute for the COVID-19 Genomics UK (COG-UK) Consortium	The Lighthouse Lab in Milton Keynes and Alex Alderton, Roberto Amato, Sonia Goncalves, Ewan Harrison, David K. Jackson, Ian Johnston, Dominic Kwiatkowski, Cordelia Langford, John Sillitoe on behalf of the Wellcome Sanger Institute COVID-19 Surveillance Team
EPI_ISL_756784	Lighthouse Lab in Glasgow	Wellcome Sanger Institute for the COVID-19 Genomics UK (COG-UK) Consortium	Harper VanSteenhouse, Yumi Kasai, David Gray, Carol Clugston, Anna Dominiczak and Alex Alderton, Roberto Amato, Sonia Goncalves, Ewan Harrison, David K. Jackson, Ian Johnston, Dominic Kwiatkowski, Cordelia Langford, John Sillitoe on behalf of the Wellcome Sanger Institute COVID-19 Surveillance Team
EPI_ISL_756785, EPI_ISL_756786, EPI_ISL_756787, EPI_ISL_756791, EPI_ISL_756792	Lighthouse Lab in Milton Keynes	Wellcome Sanger Institute for the COVID-19 Genomics UK (COG-UK) Consortium	The Lighthouse Lab in Milton Keynes and Alex Alderton, Roberto Amato, Sonia Goncalves, Ewan Harrison, David K. Jackson, Ian Johnston, Dominic Kwiatkowski, Cordelia Langford, John Sillitoe on behalf of the Wellcome Sanger Institute COVID-19 Surveillance Team
EPI_ISL_756795	Lighthouse Lab in Glasgow	Wellcome Sanger Institute for the COVID-19 Genomics UK (COG-UK) Consortium	Harper VanSteenhouse, Yumi Kasai, David Gray, Carol Clugston, Anna Dominiczak and Alex Alderton, Roberto Amato, Sonia Goncalves, Ewan Harrison, David K. Jackson, Ian Johnston, Dominic Kwiatkowski, Cordelia Langford, John Sillitoe on behalf of the Wellcome Sanger Institute COVID-19 Surveillance Team
EPI_ISL_756796, EPI_ISL_756797, EPI_ISL_756798	Lighthouse Lab in Milton Keynes	Wellcome Sanger Institute for the COVID-19 Genomics UK (COG-UK) Consortium	The Lighthouse Lab in Milton Keynes and Alex Alderton, Roberto Amato, Sonia Goncalves, Ewan Harrison, David K. Jackson, Ian Johnston, Dominic Kwiatkowski, Cordelia Langford, John Sillitoe on behalf of the Wellcome Sanger Institute COVID-19 Surveillance Team
EPI_ISL_756800	Lighthouse Lab in Glasgow	Wellcome Sanger Institute for the COVID-19 Genomics UK (COG-UK) Consortium	Harper VanSteenhouse, Yumi Kasai, David Gray, Carol Clugston, Anna Dominiczak and Alex Alderton, Roberto Amato, Sonia Goncalves, Ewan Harrison, David K. Jackson, Ian Johnston, Dominic Kwiatkowski, Cordelia Langford, John Sillitoe on behalf of the Wellcome Sanger Institute COVID-19 Surveillance Team
EPI_ISL_756801, EPI_ISL_756802	Lighthouse Lab in Milton Keynes	Wellcome Sanger Institute for the COVID-19 Genomics UK (COG-UK) Consortium	The Lighthouse Lab in Milton Keynes and Alex Alderton, Roberto Amato, Sonia Goncalves, Ewan Harrison, David K. Jackson, Ian Johnston, Dominic Kwiatkowski, Cordelia Langford, John Sillitoe on behalf of the Wellcome Sanger Institute COVID-19 Surveillance Team
EPI_ISL_756803	Lighthouse Lab in Glasgow	Wellcome Sanger Institute for the COVID-19 Genomics UK (COG-UK) Consortium	Harper VanSteenhouse, Yumi Kasai, David Gray, Carol Clugston, Anna Dominiczak and Alex Alderton, Roberto Amato, Sonia Goncalves, Ewan Harrison, David K. Jackson, Ian Johnston, Dominic Kwiatkowski, Cordelia Langford, John Sillitoe on behalf of the Wellcome Sanger Institute COVID-19 Surveillance Team
EPI_ISL_756804	Lighthouse Lab in Milton Keynes	Wellcome Sanger Institute for the COVID-19 Genomics UK (COG-UK) Consortium	The Lighthouse Lab in Milton Keynes and Alex Alderton, Roberto Amato, Sonia Goncalves, Ewan Harrison, David K. Jackson, Ian Johnston, Dominic Kwiatkowski, Cordelia Langford, John Sillitoe on behalf of the Wellcome Sanger Institute COVID-19 Surveillance Team
EPI_ISL_756805	Lighthouse Lab in Glasgow	Wellcome Sanger Institute for the COVID-19 Genomics UK (COG-UK) Consortium	Harper VanSteenhouse, Yumi Kasai, David Gray, Carol Clugston, Anna Dominiczak and Alex Alderton, Roberto Amato, Sonia Goncalves, Ewan Harrison, David K. Jackson, Ian Johnston, Dominic Kwiatkowski, Cordelia Langford, John Sillitoe on behalf of the Wellcome Sanger Institute COVID-19 Surveillance Team
EPI_ISL_756806, EPI_ISL_756807, EPI_ISL_756808, EPI_ISL_756809, EPI_ISL_756810, EPI_ISL_756811, EPI_ISL_756812, EPI_ISL_756814, EPI_ISL_756815, EPI_ISL_756816, EPI_ISL_756817, EPI_ISL_756818, EPI_ISL_756819, EPI_ISL_756822, EPI_ISL_756825, EPI_ISL_756826, EPI_ISL_756828, EPI_ISL_756829, EPI_ISL_756830, EPI_ISL_756832, EPI_ISL_756833, EPI_ISL_756834			
see above	Lighthouse Lab in Milton Keynes	Wellcome Sanger Institute for the COVID-19 Genomics UK (COG-UK) Consortium	The Lighthouse Lab in Milton Keynes and Alex Alderton, Roberto Amato, Sonia Goncalves, Ewan Harrison, David K. Jackson, Ian Johnston, Dominic Kwiatkowski, Cordelia Langford, John Sillitoe on behalf of the Wellcome Sanger Institute COVID-19 Surveillance Team
EPI_ISL_756835	Lighthouse Lab in Glasgow	Wellcome Sanger Institute for the COVID-19 Genomics UK (COG-UK) Consortium	Harper VanSteenhouse, Yumi Kasai, David Gray, Carol Clugston, Anna Dominiczak and Alex Alderton, Roberto Amato, Sonia Goncalves, Ewan Harrison, David K. Jackson, Ian Johnston, Dominic Kwiatkowski, Cordelia Langford, John Sillitoe on behalf of the Wellcome Sanger Institute COVID-19 Surveillance Team
EPI_ISL_756837, EPI_ISL_756838, EPI_ISL_756839	Lighthouse Lab in Milton Keynes	Wellcome Sanger Institute for the COVID-19 Genomics UK (COG-UK) Consortium	The Lighthouse Lab in Milton Keynes and Alex Alderton, Roberto Amato, Sonia Goncalves, Ewan Harrison, David K. Jackson, Ian Johnston, Dominic Kwiatkowski, Cordelia Langford, John Sillitoe on behalf of the Wellcome Sanger Institute COVID-19 Surveillance Team
EPI_ISL_756840	Lighthouse Lab in Glasgow	Wellcome Sanger Institute for the COVID-19 Genomics UK (COG-UK) Consortium	Harper VanSteenhouse, Yumi Kasai, David Gray, Carol Clugston, Anna Dominiczak and Alex Alderton, Roberto Amato, Sonia Goncalves, Ewan Harrison, David K. Jackson, Ian Johnston, Dominic Kwiatkowski, Cordelia Langford, John Sillitoe on behalf of the Wellcome Sanger Institute COVID-19 Surveillance Team
EPI_ISL_756842, EPI_ISL_756843, EPI_ISL_756844, EPI_ISL_756845, EPI_ISL_756846, EPI_ISL_756848	Lighthouse Lab in Milton Keynes	Wellcome Sanger Institute for the COVID-19 Genomics UK (COG-UK) Consortium	The Lighthouse Lab in Milton Keynes and Alex Alderton, Roberto Amato, Sonia Goncalves, Ewan Harrison, David K. Jackson, Ian Johnston, Dominic Kwiatkowski, Cordelia Langford, John Sillitoe on behalf of the Wellcome Sanger Institute COVID-19 Surveillance Team
EPI_ISL_756849	Lighthouse Lab in Glasgow	Wellcome Sanger Institute for the COVID-19 Genomics UK (COG-UK) Consortium	Harper VanSteenhouse, Yumi Kasai, David Gray, Carol Clugston, Anna Dominiczak and Alex Alderton, Roberto Amato, Sonia Goncalves, Ewan Harrison, David K. Jackson, Ian Johnston, Dominic Kwiatkowski, Cordelia Langford, John Sillitoe on behalf of the Wellcome Sanger Institute COVID-19 Surveillance Team
EPI_ISL_756851	Lighthouse Lab in Milton Keynes	Wellcome Sanger Institute for the COVID-19 Genomics UK (COG-UK) Consortium	The Lighthouse Lab in Milton Keynes and Alex Alderton, Roberto Amato, Sonia Goncalves, Ewan Harrison, David K. Jackson, Ian Johnston, Dominic Kwiatkowski, Cordelia Langford, John Sillitoe on behalf of the Wellcome Sanger Institute COVID-19 Surveillance Team
EPI_ISL_756852	Lighthouse Lab in Glasgow	Wellcome Sanger Institute for the COVID-19 Genomics UK (COG-UK) Consortium	Harper VanSteenhouse, Yumi Kasai, David Gray, Carol Clugston, Anna Dominiczak and Alex Alderton, Roberto Amato, Sonia Goncalves, Ewan Harrison, David K. Jackson, Ian Johnston, Dominic Kwiatkowski, Cordelia Langford, John Sillitoe on behalf of the Wellcome Sanger Institute COVID-19 Surveillance Team
EPI_ISL_756853, EPI_ISL_756855, EPI_ISL_756857, EPI_ISL_756858, EPI_ISL_756860	Lighthouse Lab in Milton Keynes	Wellcome Sanger Institute for the COVID-19 Genomics UK (COG-UK) Consortium	The Lighthouse Lab in Milton Keynes and Alex Alderton, Roberto Amato, Sonia Goncalves, Ewan Harrison, David K. Jackson, Ian Johnston, Dominic Kwiatkowski, Cordelia Langford, John Sillitoe on behalf of the Wellcome Sanger Institute COVID-19 Surveillance Team
EPI_ISL_756861	Lighthouse Lab in Glasgow	Wellcome Sanger Institute for the COVID-19 Genomics UK (COG-UK) Consortium	Harper VanSteenhouse, Yumi Kasai, David Gray, Carol Clugston, Anna Dominiczak and Alex Alderton, Roberto Amato, Sonia Goncalves, Ewan Harrison, David K. Jackson, Ian Johnston, Dominic Kwiatkowski, Cordelia Langford, John Sillitoe on behalf of the Wellcome Sanger Institute COVID-19 Surveillance Team
EPI_ISL_756862, EPI_ISL_756864, EPI_ISL_756865, EPI_ISL_756866, EPI_ISL_756868, EPI_ISL_756871, EPI_ISL_756872, EPI_ISL_756873, EPI_ISL_756874, EPI_ISL_756876	Lighthouse Lab in Milton Keynes	Wellcome Sanger Institute for the COVID-19 Genomics UK (COG-UK) Consortium	The Lighthouse Lab in Milton Keynes and Alex Alderton, Roberto Amato, Sonia Goncalves, Ewan Harrison, David K. Jackson, Ian Johnston, Dominic Kwiatkowski, Cordelia Langford, John Sillitoe on behalf of the Wellcome Sanger Institute COVID-19 Surveillance Team
EPI_ISL_756877, EPI_ISL_756878	Lighthouse Lab in Glasgow	Wellcome Sanger Institute for the COVID-19 Genomics UK (COG-UK) Consortium	Harper VanSteenhouse, Yumi Kasai, David Gray, Carol Clugston, Anna Dominiczak and Alex Alderton, Roberto Amato, Sonia Goncalves, Ewan Harrison, David K. Jackson, Ian Johnston, Dominic Kwiatkowski, Cordelia Langford, John Sillitoe on behalf of the Wellcome Sanger Institute COVID-19 Surveillance Team

EPI_ISL_756880, EPI_ISL_756882, EPI_ISL_756883, EPI_ISL_756884	Lighthouse Lab in Milton Keynes	Wellcome Sanger Institute for the COVID-19 Genomics UK (COG-UK) Consortium	The Lighthouse Lab in Milton Keynes and Alex Alderton, Roberto Amato, Sonia Goncalves, Ewan Harrison, David K. Jackson, Ian Johnston, Dominic Kwiatkowski, Cordelia Langford, John Sillitoe on behalf of the Wellcome Sanger Institute COVID-19 Surveillance Team
EPI_ISL_756885, EPI_ISL_756886, EPI_ISL_756888, EPI_ISL_756889	Lighthouse Lab in Glasgow	Wellcome Sanger Institute for the COVID-19 Genomics UK (COG-UK) Consortium	Harper VanSteenhouse, Yumi Kasai, David Gray, Carol Clugston, Anna Dominiczak and Alex Alderton, Roberto Amato, Sonia Goncalves, Ewan Harrison, David K. Jackson, Ian Johnston, Dominic Kwiatkowski, Cordelia Langford, John Sillitoe on behalf of the Wellcome Sanger Institute COVID-19 Surveillance Team
EPI_ISL_756890, EPI_ISL_756891, EPI_ISL_756892	Lighthouse Lab in Milton Keynes	Wellcome Sanger Institute for the COVID-19 Genomics UK (COG-UK) Consortium	The Lighthouse Lab in Milton Keynes and Alex Alderton, Roberto Amato, Sonia Goncalves, Ewan Harrison, David K. Jackson, Ian Johnston, Dominic Kwiatkowski, Cordelia Langford, John Sillitoe on behalf of the Wellcome Sanger Institute COVID-19 Surveillance Team
EPI_ISL_756893, EPI_ISL_756894, EPI_ISL_756895	Lighthouse Lab in Glasgow	Wellcome Sanger Institute for the COVID-19 Genomics UK (COG-UK) Consortium	Harper VanSteenhouse, Yumi Kasai, David Gray, Carol Clugston, Anna Dominiczak and Alex Alderton, Roberto Amato, Sonia Goncalves, Ewan Harrison, David K. Jackson, Ian Johnston, Dominic Kwiatkowski, Cordelia Langford, John Sillitoe on behalf of the Wellcome Sanger Institute COVID-19 Surveillance Team
EPI_ISL_756896	Lighthouse Lab in Milton Keynes	Wellcome Sanger Institute for the COVID-19 Genomics UK (COG-UK) Consortium	The Lighthouse Lab in Milton Keynes and Alex Alderton, Roberto Amato, Sonia Goncalves, Ewan Harrison, David K. Jackson, Ian Johnston, Dominic Kwiatkowski, Cordelia Langford, John Sillitoe on behalf of the Wellcome Sanger Institute COVID-19 Surveillance Team
EPI_ISL_756897	Lighthouse Lab in Glasgow	Wellcome Sanger Institute for the COVID-19 Genomics UK (COG-UK) Consortium	Harper VanSteenhouse, Yumi Kasai, David Gray, Carol Clugston, Anna Dominiczak and Alex Alderton, Roberto Amato, Sonia Goncalves, Ewan Harrison, David K. Jackson, Ian Johnston, Dominic Kwiatkowski, Cordelia Langford, John Sillitoe on behalf of the Wellcome Sanger Institute COVID-19 Surveillance Team
EPI_ISL_756899	Lighthouse Lab in Milton Keynes	Wellcome Sanger Institute for the COVID-19 Genomics UK (COG-UK) Consortium	The Lighthouse Lab in Milton Keynes and Alex Alderton, Roberto Amato, Sonia Goncalves, Ewan Harrison, David K. Jackson, Ian Johnston, Dominic Kwiatkowski, Cordelia Langford, John Sillitoe on behalf of the Wellcome Sanger Institute COVID-19 Surveillance Team
EPI_ISL_756900	Lighthouse Lab in Glasgow	Wellcome Sanger Institute for the COVID-19 Genomics UK (COG-UK) Consortium	Harper VanSteenhouse, Yumi Kasai, David Gray, Carol Clugston, Anna Dominiczak and Alex Alderton, Roberto Amato, Sonia Goncalves, Ewan Harrison, David K. Jackson, Ian Johnston, Dominic Kwiatkowski, Cordelia Langford, John Sillitoe on behalf of the Wellcome Sanger Institute COVID-19 Surveillance Team
EPI_ISL_756901, EPI_ISL_756902, EPI_ISL_756903, EPI_ISL_756904, EPI_ISL_756908	Lighthouse Lab in Milton Keynes	Wellcome Sanger Institute for the COVID-19 Genomics UK (COG-UK) Consortium	The Lighthouse Lab in Milton Keynes and Alex Alderton, Roberto Amato, Sonia Goncalves, Ewan Harrison, David K. Jackson, Ian Johnston, Dominic Kwiatkowski, Cordelia Langford, John Sillitoe on behalf of the Wellcome Sanger Institute COVID-19 Surveillance Team
EPI_ISL_756909, EPI_ISL_756910	Lighthouse Lab in Glasgow	Wellcome Sanger Institute for the COVID-19 Genomics UK (COG-UK) Consortium	Harper VanSteenhouse, Yumi Kasai, David Gray, Carol Clugston, Anna Dominiczak and Alex Alderton, Roberto Amato, Sonia Goncalves, Ewan Harrison, David K. Jackson, Ian Johnston, Dominic Kwiatkowski, Cordelia Langford, John Sillitoe on behalf of the Wellcome Sanger Institute COVID-19 Surveillance Team
EPI_ISL_756911	Lighthouse Lab in Milton Keynes	Wellcome Sanger Institute for the COVID-19 Genomics UK (COG-UK) Consortium	The Lighthouse Lab in Milton Keynes and Alex Alderton, Roberto Amato, Sonia Goncalves, Ewan Harrison, David K. Jackson, Ian Johnston, Dominic Kwiatkowski, Cordelia Langford, John Sillitoe on behalf of the Wellcome Sanger Institute COVID-19 Surveillance Team
EPI_ISL_756912	Lighthouse Lab in Glasgow	Wellcome Sanger Institute for the COVID-19 Genomics UK (COG-UK) Consortium	Harper VanSteenhouse, Yumi Kasai, David Gray, Carol Clugston, Anna Dominiczak and Alex Alderton, Roberto Amato, Sonia Goncalves, Ewan Harrison, David K. Jackson, Ian Johnston, Dominic Kwiatkowski, Cordelia Langford, John Sillitoe on behalf of the Wellcome Sanger Institute COVID-19 Surveillance Team
EPI_ISL_756913, EPI_ISL_756914, EPI_ISL_756915, EPI_ISL_756916, EPI_ISL_756919	Lighthouse Lab in Milton Keynes	Wellcome Sanger Institute for the COVID-19 Genomics UK (COG-UK) Consortium	The Lighthouse Lab in Milton Keynes and Alex Alderton, Roberto Amato, Sonia Goncalves, Ewan Harrison, David K. Jackson, Ian Johnston, Dominic Kwiatkowski, Cordelia Langford, John Sillitoe on behalf of the Wellcome Sanger Institute COVID-19 Surveillance Team
EPI_ISL_756920	Lighthouse Lab in Glasgow	Wellcome Sanger Institute for the COVID-19 Genomics UK (COG-UK) Consortium	Harper VanSteenhouse, Yumi Kasai, David Gray, Carol Clugston, Anna Dominiczak and Alex Alderton, Roberto Amato, Sonia Goncalves, Ewan Harrison, David K. Jackson, Ian Johnston, Dominic Kwiatkowski, Cordelia Langford, John Sillitoe on behalf of the Wellcome Sanger Institute COVID-19 Surveillance Team
EPI_ISL_756921, EPI_ISL_756922	Lighthouse Lab in Milton Keynes	Wellcome Sanger Institute for the COVID-19 Genomics UK (COG-UK) Consortium	The Lighthouse Lab in Milton Keynes and Alex Alderton, Roberto Amato, Sonia Goncalves, Ewan Harrison, David K. Jackson, Ian Johnston, Dominic Kwiatkowski, Cordelia Langford, John Sillitoe on behalf of the Wellcome Sanger Institute COVID-19 Surveillance Team
EPI_ISL_756923	Lighthouse Lab in Glasgow	Wellcome Sanger Institute for the COVID-19 Genomics UK (COG-UK) Consortium	Harper VanSteenhouse, Yumi Kasai, David Gray, Carol Clugston, Anna Dominiczak and Alex Alderton, Roberto Amato, Sonia Goncalves, Ewan Harrison, David K. Jackson, Ian Johnston, Dominic Kwiatkowski, Cordelia Langford, John Sillitoe on behalf of the Wellcome Sanger Institute COVID-19 Surveillance Team
EPI_ISL_756925, EPI_ISL_756926, EPI_ISL_756929, EPI_ISL_756930, EPI_ISL_756932, EPI_ISL_756934, EPI_ISL_756936, EPI_ISL_756937	Lighthouse Lab in Milton Keynes	Wellcome Sanger Institute for the COVID-19 Genomics UK (COG-UK) Consortium	The Lighthouse Lab in Milton Keynes and Alex Alderton, Roberto Amato, Sonia Goncalves, Ewan Harrison, David K. Jackson, Ian Johnston, Dominic Kwiatkowski, Cordelia Langford, John Sillitoe on behalf of the Wellcome Sanger Institute COVID-19 Surveillance Team
EPI_ISL_756940	Lighthouse Lab in Glasgow	Wellcome Sanger Institute for the COVID-19 Genomics UK (COG-UK) Consortium	Harper VanSteenhouse, Yumi Kasai, David Gray, Carol Clugston, Anna Dominiczak and Alex Alderton, Roberto Amato, Sonia Goncalves, Ewan Harrison, David K. Jackson, Ian Johnston, Dominic Kwiatkowski, Cordelia Langford, John Sillitoe on behalf of the Wellcome Sanger Institute COVID-19 Surveillance Team
EPI_ISL_756941, EPI_ISL_756942, EPI_ISL_756944, EPI_ISL_756945, EPI_ISL_756946	Lighthouse Lab in Milton Keynes	Wellcome Sanger Institute for the COVID-19 Genomics UK (COG-UK) Consortium	The Lighthouse Lab in Milton Keynes and Alex Alderton, Roberto Amato, Sonia Goncalves, Ewan Harrison, David K. Jackson, Ian Johnston, Dominic Kwiatkowski, Cordelia Langford, John Sillitoe on behalf of the Wellcome Sanger Institute COVID-19 Surveillance Team
EPI_ISL_756947	Lighthouse Lab in Glasgow	Wellcome Sanger Institute for the COVID-19 Genomics UK (COG-UK) Consortium	Harper VanSteenhouse, Yumi Kasai, David Gray, Carol Clugston, Anna Dominiczak and Alex Alderton, Roberto Amato, Sonia Goncalves, Ewan Harrison, David K. Jackson, Ian Johnston, Dominic Kwiatkowski, Cordelia Langford, John Sillitoe on behalf of the Wellcome Sanger Institute COVID-19 Surveillance Team
EPI_ISL_756948, EPI_ISL_756949	Lighthouse Lab in Milton Keynes	Wellcome Sanger Institute for the COVID-19 Genomics UK (COG-UK) Consortium	The Lighthouse Lab in Milton Keynes and Alex Alderton, Roberto Amato, Sonia Goncalves, Ewan Harrison, David K. Jackson, Ian Johnston, Dominic Kwiatkowski, Cordelia Langford, John Sillitoe on behalf of the Wellcome Sanger Institute COVID-19 Surveillance Team
EPI_ISL_756951, EPI_ISL_756953	Lighthouse Lab in Glasgow	Wellcome Sanger Institute for the COVID-19 Genomics UK (COG-UK) Consortium	Harper VanSteenhouse, Yumi Kasai, David Gray, Carol Clugston, Anna Dominiczak and Alex Alderton, Roberto Amato, Sonia Goncalves, Ewan Harrison, David K. Jackson, Ian Johnston, Dominic Kwiatkowski, Cordelia Langford, John Sillitoe on behalf of the Wellcome Sanger Institute COVID-19 Surveillance Team
EPI_ISL_756957	Lighthouse Lab in Milton Keynes	Wellcome Sanger Institute for the COVID-19 Genomics UK (COG-UK) Consortium	The Lighthouse Lab in Milton Keynes and Alex Alderton, Roberto Amato, Sonia Goncalves, Ewan Harrison, David K. Jackson, Ian Johnston, Dominic Kwiatkowski, Cordelia Langford, John Sillitoe on behalf of the Wellcome Sanger Institute COVID-19 Surveillance Team
EPI_ISL_756959	Lighthouse Lab in Glasgow	Wellcome Sanger Institute for the COVID-19 Genomics UK (COG-UK) Consortium	Harper VanSteenhouse, Yumi Kasai, David Gray, Carol Clugston, Anna Dominiczak and Alex Alderton, Roberto Amato, Sonia Goncalves, Ewan Harrison, David K. Jackson, Ian Johnston, Dominic Kwiatkowski, Cordelia Langford, John Sillitoe on behalf of the Wellcome Sanger Institute COVID-19 Surveillance Team
EPI_ISL_756961, EPI_ISL_756963, EPI_ISL_756964, EPI_ISL_756965, EPI_ISL_756966, EPI_ISL_756967, EPI_ISL_756968	Lighthouse Lab in Milton Keynes	Wellcome Sanger Institute for the COVID-19 Genomics UK (COG-UK) Consortium	The Lighthouse Lab in Milton Keynes and Alex Alderton, Roberto Amato, Sonia Goncalves, Ewan Harrison, David K. Jackson, Ian Johnston, Dominic Kwiatkowski, Cordelia Langford, John Sillitoe on behalf of the Wellcome Sanger Institute COVID-19 Surveillance Team
EPI_ISL_756970, EPI_ISL_756971	Lighthouse Lab in Glasgow	Wellcome Sanger Institute for the COVID-19 Genomics UK (COG-UK) Consortium	Harper VanSteenhouse, Yumi Kasai, David Gray, Carol Clugston, Anna Dominiczak and Alex Alderton, Roberto Amato, Sonia Goncalves, Ewan Harrison, David K. Jackson, Ian Johnston, Dominic Kwiatkowski, Cordelia Langford, John Sillitoe on behalf of the Wellcome Sanger Institute COVID-19 Surveillance Team
EPI_ISL_756972	Lighthouse Lab in Milton Keynes	Wellcome Sanger Institute for the COVID-19 Genomics UK (COG-UK) Consortium	The Lighthouse Lab in Milton Keynes and Alex Alderton, Roberto Amato, Sonia Goncalves, Ewan Harrison, David K. Jackson, Ian Johnston, Dominic Kwiatkowski, Cordelia Langford, John Sillitoe on behalf of the Wellcome Sanger Institute COVID-19 Surveillance Team

EPI_ISL_756973	Lighthouse Lab in Glasgow	Wellcome Sanger Institute for the COVID-19 Genomics UK (COG-UK) Consortium	Harper VanSteenhouse, Yumi Kasai, David Gray, Carol Clugston, Anna Dominiczak and Alex Alderton, Roberto Amato, Sonia Goncalves, Ewan Harrison, David K. Jackson, Ian Johnston, Dominic Kwiatkowski, Cordelia Langford, John Sillitoe on behalf of the Wellcome Sanger Institute COVID-19 Surveillance Team
EPI_ISL_756975, EPI_ISL_756976	Lighthouse Lab in Milton Keynes	Wellcome Sanger Institute for the COVID-19 Genomics UK (COG-UK) Consortium	The Lighthouse Lab in Milton Keynes and Alex Alderton, Roberto Amato, Sonia Goncalves, Ewan Harrison, David K. Jackson, Ian Johnston, Dominic Kwiatkowski, Cordelia Langford, John Sillitoe on behalf of the Wellcome Sanger Institute COVID-19 Surveillance Team
EPI_ISL_756977	Lighthouse Lab in Glasgow	Wellcome Sanger Institute for the COVID-19 Genomics UK (COG-UK) Consortium	Harper VanSteenhouse, Yumi Kasai, David Gray, Carol Clugston, Anna Dominiczak and Alex Alderton, Roberto Amato, Sonia Goncalves, Ewan Harrison, David K. Jackson, Ian Johnston, Dominic Kwiatkowski, Cordelia Langford, John Sillitoe on behalf of the Wellcome Sanger Institute COVID-19 Surveillance Team
EPI_ISL_756979, EPI_ISL_756980, EPI_ISL_756981, EPI_ISL_756983, EPI_ISL_756984, EPI_ISL_756986, EPI_ISL_756987, EPI_ISL_756988, EPI_ISL_756990, EPI_ISL_756991, EPI_ISL_756992, EPI_ISL_756996, EPI_ISL_756997			
see above	Lighthouse Lab in Milton Keynes	Wellcome Sanger Institute for the COVID-19 Genomics UK (COG-UK) Consortium	The Lighthouse Lab in Milton Keynes and Alex Alderton, Roberto Amato, Sonia Goncalves, Ewan Harrison, David K. Jackson, Ian Johnston, Dominic Kwiatkowski, Cordelia Langford, John Sillitoe on behalf of the Wellcome Sanger Institute COVID-19 Surveillance Team
EPI_ISL_756998	Lighthouse Lab in Glasgow	Wellcome Sanger Institute for the COVID-19 Genomics UK (COG-UK) Consortium	Harper VanSteenhouse, Yumi Kasai, David Gray, Carol Clugston, Anna Dominiczak and Alex Alderton, Roberto Amato, Sonia Goncalves, Ewan Harrison, David K. Jackson, Ian Johnston, Dominic Kwiatkowski, Cordelia Langford, John Sillitoe on behalf of the Wellcome Sanger Institute COVID-19 Surveillance Team
EPI_ISL_757001, EPI_ISL_757003, EPI_ISL_757004	Lighthouse Lab in Milton Keynes	Wellcome Sanger Institute for the COVID-19 Genomics UK (COG-UK) Consortium	The Lighthouse Lab in Milton Keynes and Alex Alderton, Roberto Amato, Sonia Goncalves, Ewan Harrison, David K. Jackson, Ian Johnston, Dominic Kwiatkowski, Cordelia Langford, John Sillitoe on behalf of the Wellcome Sanger Institute COVID-19 Surveillance Team
EPI_ISL_757005	Lighthouse Lab in Glasgow	Wellcome Sanger Institute for the COVID-19 Genomics UK (COG-UK) Consortium	Harper VanSteenhouse, Yumi Kasai, David Gray, Carol Clugston, Anna Dominiczak and Alex Alderton, Roberto Amato, Sonia Goncalves, Ewan Harrison, David K. Jackson, Ian Johnston, Dominic Kwiatkowski, Cordelia Langford, John Sillitoe on behalf of the Wellcome Sanger Institute COVID-19 Surveillance Team
EPI_ISL_757006	Lighthouse Lab in Milton Keynes	Wellcome Sanger Institute for the COVID-19 Genomics UK (COG-UK) Consortium	The Lighthouse Lab in Milton Keynes and Alex Alderton, Roberto Amato, Sonia Goncalves, Ewan Harrison, David K. Jackson, Ian Johnston, Dominic Kwiatkowski, Cordelia Langford, John Sillitoe on behalf of the Wellcome Sanger Institute COVID-19 Surveillance Team
EPI_ISL_757008, EPI_ISL_757009	Lighthouse Lab in Glasgow	Wellcome Sanger Institute for the COVID-19 Genomics UK (COG-UK) Consortium	Harper VanSteenhouse, Yumi Kasai, David Gray, Carol Clugston, Anna Dominiczak and Alex Alderton, Roberto Amato, Sonia Goncalves, Ewan Harrison, David K. Jackson, Ian Johnston, Dominic Kwiatkowski, Cordelia Langford, John Sillitoe on behalf of the Wellcome Sanger Institute COVID-19 Surveillance Team
EPI_ISL_757010	Lighthouse Lab in Milton Keynes	Wellcome Sanger Institute for the COVID-19 Genomics UK (COG-UK) Consortium	The Lighthouse Lab in Milton Keynes and Alex Alderton, Roberto Amato, Sonia Goncalves, Ewan Harrison, David K. Jackson, Ian Johnston, Dominic Kwiatkowski, Cordelia Langford, John Sillitoe on behalf of the Wellcome Sanger Institute COVID-19 Surveillance Team
EPI_ISL_757011, EPI_ISL_757012	Lighthouse Lab in Glasgow	Wellcome Sanger Institute for the COVID-19 Genomics UK (COG-UK) Consortium	Harper VanSteenhouse, Yumi Kasai, David Gray, Carol Clugston, Anna Dominiczak and Alex Alderton, Roberto Amato, Sonia Goncalves, Ewan Harrison, David K. Jackson, Ian Johnston, Dominic Kwiatkowski, Cordelia Langford, John Sillitoe on behalf of the Wellcome Sanger Institute COVID-19 Surveillance Team
EPI_ISL_757014, EPI_ISL_757016, EPI_ISL_757019	Lighthouse Lab in Milton Keynes	Wellcome Sanger Institute for the COVID-19 Genomics UK (COG-UK) Consortium	The Lighthouse Lab in Milton Keynes and Alex Alderton, Roberto Amato, Sonia Goncalves, Ewan Harrison, David K. Jackson, Ian Johnston, Dominic Kwiatkowski, Cordelia Langford, John Sillitoe on behalf of the Wellcome Sanger Institute COVID-19 Surveillance Team
EPI_ISL_757021	Lighthouse Lab in Glasgow	Wellcome Sanger Institute for the COVID-19 Genomics UK (COG-UK) Consortium	Harper VanSteenhouse, Yumi Kasai, David Gray, Carol Clugston, Anna Dominiczak and Alex Alderton, Roberto Amato, Sonia Goncalves, Ewan Harrison, David K. Jackson, Ian Johnston, Dominic Kwiatkowski, Cordelia Langford, John Sillitoe on behalf of the Wellcome Sanger Institute COVID-19 Surveillance Team
EPI_ISL_757024, EPI_ISL_757025, EPI_ISL_757026	Lighthouse Lab in Milton Keynes	Wellcome Sanger Institute for the COVID-19 Genomics UK (COG-UK) Consortium	The Lighthouse Lab in Milton Keynes and Alex Alderton, Roberto Amato, Sonia Goncalves, Ewan Harrison, David K. Jackson, Ian Johnston, Dominic Kwiatkowski, Cordelia Langford, John Sillitoe on behalf of the Wellcome Sanger Institute COVID-19 Surveillance Team
EPI_ISL_757027, EPI_ISL_757028	Lighthouse Lab in Glasgow	Wellcome Sanger Institute for the COVID-19 Genomics UK (COG-UK) Consortium	Harper VanSteenhouse, Yumi Kasai, David Gray, Carol Clugston, Anna Dominiczak and Alex Alderton, Roberto Amato, Sonia Goncalves, Ewan Harrison, David K. Jackson, Ian Johnston, Dominic Kwiatkowski, Cordelia Langford, John Sillitoe on behalf of the Wellcome Sanger Institute COVID-19 Surveillance Team
EPI_ISL_757029, EPI_ISL_757030	Lighthouse Lab in Milton Keynes	Wellcome Sanger Institute for the COVID-19 Genomics UK (COG-UK) Consortium	The Lighthouse Lab in Milton Keynes and Alex Alderton, Roberto Amato, Sonia Goncalves, Ewan Harrison, David K. Jackson, Ian Johnston, Dominic Kwiatkowski, Cordelia Langford, John Sillitoe on behalf of the Wellcome Sanger Institute COVID-19 Surveillance Team
EPI_ISL_757032, EPI_ISL_757034, EPI_ISL_757036, EPI_ISL_757037, EPI_ISL_757040, EPI_ISL_757042, EPI_ISL_757043, EPI_ISL_757044, EPI_ISL_757045, EPI_ISL_757047, EPI_ISL_757048, EPI_ISL_757049, EPI_ISL_757050, EPI_ISL_757052, EPI_ISL_757053, EPI_ISL_757055, EPI_ISL_757057, EPI_ISL_757058, EPI_ISL_757059, EPI_ISL_757063, EPI_ISL_757064, EPI_ISL_757065, EPI_ISL_757066, EPI_ISL_757067, EPI_ISL_757069, EPI_ISL_757070, EPI_ISL_757073, EPI_ISL_757075, EPI_ISL_757077, EPI_ISL_757079, EPI_ISL_757082, EPI_ISL_757085, EPI_ISL_757091, EPI_ISL_757092, EPI_ISL_757093, EPI_ISL_757096, EPI_ISL_757099, EPI_ISL_757100, EPI_ISL_757102, EPI_ISL_757104, EPI_ISL_757105, EPI_ISL_757107, EPI_ISL_757108, EPI_ISL_757117, EPI_ISL_757118, EPI_ISL_757120, EPI_ISL_757121, EPI_ISL_757122, EPI_ISL_757123, EPI_ISL_757124, EPI_ISL_757126, EPI_ISL_757128, EPI_ISL_757129, EPI_ISL_757130, EPI_ISL_757132, EPI_ISL_757134, EPI_ISL_757135, EPI_ISL_757136, EPI_ISL_757137, EPI_ISL_757138, EPI_ISL_757139, EPI_ISL_757140, EPI_ISL_757141, EPI_ISL_757143, EPI_ISL_757144, EPI_ISL_757147, EPI_ISL_757148, EPI_ISL_757150, EPI_ISL_757152, EPI_ISL_757153, EPI_ISL_757154, EPI_ISL_757156, EPI_ISL_757157, EPI_ISL_757158, EPI_ISL_757159, EPI_ISL_757160, EPI_ISL_757161, EPI_ISL_757162, EPI_ISL_757163, EPI_ISL_757164, EPI_ISL_757169, EPI_ISL_757170, EPI_ISL_757171, EPI_ISL_757172, EPI_ISL_757173, EPI_ISL_757174, EPI_ISL_757175, EPI_ISL_757176, EPI_ISL_757177, EPI_ISL_757180, EPI_ISL_757181, EPI_ISL_757182, EPI_ISL_757184, EPI_ISL_757186, EPI_ISL_757225, EPI_ISL_757226, EPI_ISL_757227, EPI_ISL_757228, EPI_ISL_757230, EPI_ISL_757231, EPI_ISL_757232, EPI_ISL_757233, EPI_ISL_757234, EPI_ISL_757235, EPI_ISL_757236, EPI_ISL_757237, EPI_ISL_757238, EPI_ISL_757239, EPI_ISL_757241, EPI_ISL_757242, EPI_ISL_757243, EPI_ISL_757246, EPI_ISL_757247, EPI_ISL_757248, EPI_ISL_757249, EPI_ISL_757250, EPI_ISL_757251, EPI_ISL_757252, EPI_ISL_757254, EPI_ISL_757255, EPI_ISL_757256, EPI_ISL_757258, EPI_ISL_757259, EPI_ISL_757260, EPI_ISL_757261, EPI_ISL_757263, EPI_ISL_757265, EPI_ISL_757266, EPI_ISL_757267, EPI_ISL_757268, EPI_ISL_757269, EPI_ISL_757270, EPI_ISL_757271, EPI_ISL_757272			
see above	Lighthouse Lab in Glasgow	Wellcome Sanger Institute for the COVID-19 Genomics UK (COG-UK) Consortium	Harper VanSteenhouse, Yumi Kasai, David Gray, Carol Clugston, Anna Dominiczak and Alex Alderton, Roberto Amato, Sonia Goncalves, Ewan Harrison, David K. Jackson, Ian Johnston, Dominic Kwiatkowski, Cordelia Langford, John Sillitoe on behalf of the Wellcome Sanger Institute COVID-19 Surveillance Team
EPI_ISL_757285, EPI_ISL_757286	Department of Virology, Public Health Laboratories Division	Department of Virology, Public Health Laboratories Division	Massab Umair, Aamer Ikram, Muhammad Salman
EPI_ISL_757451, EPI_ISL_757452, EPI_ISL_757453, EPI_ISL_757454, EPI_ISL_757455, EPI_ISL_757456, EPI_ISL_757458, EPI_ISL_757459, EPI_ISL_757460, EPI_ISL_757461, EPI_ISL_757462, EPI_ISL_757465, EPI_ISL_757466, EPI_ISL_757467, EPI_ISL_757468, EPI_ISL_757469, EPI_ISL_757471, EPI_ISL_757472, EPI_ISL_757473, EPI_ISL_757474, EPI_ISL_757475, EPI_ISL_757476, EPI_ISL_757477, EPI_ISL_757478, EPI_ISL_757479, EPI_ISL_757480, EPI_ISL_757481, EPI_ISL_757482, EPI_ISL_757483, EPI_ISL_757484, EPI_ISL_757485, EPI_ISL_757486, EPI_ISL_757487, EPI_ISL_757488, EPI_ISL_757489, EPI_ISL_757490, EPI_ISL_757491, EPI_ISL_757494, EPI_ISL_757495, EPI_ISL_757510, EPI_ISL_757511, EPI_ISL_757512, EPI_ISL_757513, EPI_ISL_757514, EPI_ISL_757515, EPI_ISL_757516, EPI_ISL_757517, EPI_ISL_757518, EPI_ISL_757519, EPI_ISL_757520, EPI_ISL_757521, EPI_ISL_757522, EPI_ISL_757523, EPI_ISL_757524, EPI_ISL_757525, EPI_ISL_757526, EPI_ISL_757527, EPI_ISL_757528, EPI_ISL_757529, EPI_ISL_757530, EPI_ISL_757531, EPI_ISL_757532, EPI_ISL_757533, EPI_ISL_757534, EPI_ISL_757535, EPI_ISL_757536, EPI_ISL_757537, EPI_ISL_757538, EPI_ISL_757539, EPI_ISL_757540, EPI_ISL_757541, EPI_ISL_757542, EPI_ISL_757543, EPI_ISL_757544, EPI_ISL_757546, EPI_ISL_757547, EPI_ISL_757548, EPI_ISL_757549, EPI_ISL_757550, EPI_ISL_757551, EPI_ISL_757552, EPI_ISL_757553, EPI_ISL_757554, EPI_ISL_757555, EPI_ISL_757556, EPI_ISL_757557, EPI_ISL_757558, EPI_ISL_757559, EPI_ISL_757560, EPI_ISL_757561, EPI_ISL_757562, EPI_ISL_757563, EPI_ISL_757564, EPI_ISL_757566, EPI_ISL_757567, EPI_ISL_757568, EPI_ISL_757569, EPI_ISL_757570, EPI_ISL_757571, EPI_ISL_757572, EPI_ISL_757573, EPI_ISL_757574, EPI_ISL_757575, EPI_ISL_757576, EPI_ISL_757577, EPI_ISL_757578, EPI_ISL_757579, EPI_ISL_757581, EPI_ISL_757582, EPI_ISL_757583, EPI_ISL_757584, EPI_ISL_757585, EPI_ISL_757586, EPI_ISL_757587, EPI_ISL_757588, EPI_ISL_757589, EPI_ISL_757590, EPI_ISL_757591, EPI_ISL_757592, EPI_ISL_757593, EPI_ISL_757594, EPI_ISL_757595, EPI_ISL_757596, EPI_ISL_757597, EPI_ISL_757598, EPI_ISL_757599, EPI_ISL_757600, EPI_ISL_757601, EPI_ISL_757602, EPI_ISL_757603, EPI_ISL_757604, EPI_ISL_757605, EPI_ISL_757606, EPI_ISL_757607, EPI_ISL_757608, EPI_ISL_757609, EPI_ISL_757610, EPI_ISL_757611, EPI_ISL_757612, EPI_ISL_757613, EPI_ISL_757614, EPI_ISL_757615, EPI_ISL_757616, EPI_ISL_757617, EPI_ISL_757618, EPI_ISL_757619, EPI_ISL_757620, EPI_ISL_757622, EPI_ISL_757623, EPI_ISL_757624, EPI_ISL_757625, EPI_ISL_757626, EPI_ISL_757627, EPI_ISL_757628, EPI_ISL_757629, EPI_ISL_757630, EPI_ISL_757631, EPI_ISL_757632, EPI_ISL_757633, EPI_ISL_757634, EPI_ISL_757635, EPI_ISL_757636, EPI_ISL_757638, EPI_ISL_757639, EPI_ISL_757640, EPI_ISL_757641, EPI_ISL_757642, EPI_ISL_757643, EPI_ISL_757644, EPI_ISL_757645, EPI_ISL_757646, EPI_ISL_757647, EPI_ISL_757648, EPI_ISL_757649, EPI_ISL_757650, EPI_ISL_757651, EPI_ISL_757652, EPI_ISL_757653, EPI_ISL_757654, EPI_ISL_757655, EPI_ISL_757656, EPI_ISL_757657, EPI_ISL_757658, EPI_ISL_757659, EPI_ISL_757660, EPI_ISL_757661, EPI_ISL_757662, EPI_ISL_757663, EPI_ISL_757665, EPI_ISL_757666, EPI_ISL_757667, EPI_ISL_757668, EPI_ISL_757669, EPI_ISL_757670, EPI_ISL_757671, EPI_ISL_757672, EPI_ISL_757673, EPI_ISL_757674, EPI_ISL_757675, EPI_ISL_757676, EPI_ISL_757677, EPI_ISL_757678, EPI_ISL_757679, EPI_ISL_757680, EPI_ISL_757681, EPI_ISL_757682, EPI_ISL_757683, EPI_ISL_757684, EPI_ISL_757685, EPI_ISL_757686, EPI_ISL_757687, EPI_ISL_757688, EPI_ISL_757690, EPI_ISL_757691, EPI_ISL_757692, EPI_ISL_757693, EPI_ISL_757694, EPI_ISL_757695, EPI_ISL_757696, EPI_ISL_757697, EPI_ISL_757698, EPI_ISL_757877, EPI_ISL_757879, EPI_ISL_757880, EPI_ISL_757881, EPI_ISL_757882, EPI_ISL_757884, EPI_ISL_757885, EPI_ISL_757886, EPI_ISL_757887, EPI_ISL_757888, EPI_ISL_757889, EPI_ISL_757890, EPI_ISL_757892, EPI_ISL_757896, EPI_ISL_757897, EPI_ISL_757898, EPI_ISL_757899, EPI_ISL_757900, EPI_ISL_757902, EPI_ISL_757903, EPI_ISL_757904, EPI_ISL_757906, EPI_ISL_757908, EPI_ISL_757909, EPI_ISL_757910, EPI_ISL_757911, EPI_ISL_757912, EPI_ISL_757913, EPI_ISL_757914, EPI_ISL_757915, EPI_ISL_757916, EPI_ISL_757917, EPI_ISL_757918, EPI_ISL_757919, EPI_ISL_757920, EPI_ISL_757921, EPI_ISL_757922, EPI_ISL_757923, EPI_ISL_757924, EPI_ISL_757925, EPI_ISL_757926, EPI_ISL_757927, EPI_ISL_757928, EPI_ISL_757929, EPI_ISL_757930, EPI_ISL_757931, EPI_ISL_757932, EPI_ISL_757933, EPI_ISL_757934, EPI_ISL_757935, EPI_ISL_757936, EPI_ISL_757939, EPI_ISL_757940, EPI_ISL_757941, EPI_ISL_757942, EPI_ISL_757943, EPI_ISL_757944, EPI_ISL_757960, EPI_ISL_757961, EPI_ISL_757962, EPI_ISL_757963, EPI_ISL_757964, EPI_ISL_757965, EPI_ISL_757966, EPI_ISL_757967, EPI_ISL_757968, EPI_ISL_757969, EPI_ISL_757970, EPI_ISL_757971, EPI_ISL_757972, EPI_ISL_757973, EPI_ISL_757975, EPI_ISL_757977, EPI_ISL_757978, EPI_ISL_757980, EPI_ISL_757981, EPI_ISL_757982, EPI_ISL_757983, EPI_ISL_757984, EPI_ISL_757985, EPI_ISL_757986, EPI_ISL_757987, EPI_ISL_757988, EPI_ISL_757989, EPI_ISL_757991, EPI_ISL_757992, EPI_ISL_757994, EPI_ISL_757995, EPI_ISL_757996, EPI_ISL_757997, EPI_ISL_757998, EPI_ISL_757999, EPI_ISL_758000, EPI_ISL_758001, EPI_ISL_758002, EPI_ISL_758003, EPI_ISL_758004, EPI_ISL_758005, EPI_ISL_758006, EPI_ISL_758007, EPI_ISL_758008, EPI_ISL_758009, EPI_ISL_758010, EPI_ISL_758011, EPI_ISL_758012, EPI_ISL_758014, EPI_ISL_758015, EPI_ISL_758016, EPI_ISL_758017, EPI_ISL_758018, EPI_ISL_758019, EPI_ISL_758020, EPI_ISL_758021, EPI_ISL_758022, EPI_ISL_758023, EPI_ISL_758024, EPI_ISL_758025, EPI_ISL_758026, EPI_ISL_758027, EPI_ISL_758028, EPI_ISL_758029, EPI_ISL_758030, EPI_ISL_758031, EPI_ISL_758032, EPI_ISL_758033, EPI_ISL_758034, EPI_ISL_758035, EPI_ISL_758037, EPI_ISL_758039, EPI_ISL_758040, EPI_ISL_758041, EPI_ISL_758042, EPI_ISL_758043, EPI_ISL_758044, EPI_ISL_758045, EPI_ISL_758046, EPI_ISL_758047, EPI_ISL_758048, EPI_ISL_758049, EPI_ISL_758050, EPI_ISL_758051, EPI_ISL_758052, EPI_ISL_758053, EPI_ISL_758054, EPI_ISL_758055, EPI_ISL_758057, EPI_ISL_758058, EPI_ISL_758059, EPI_ISL_758060, EPI_ISL_758061, EPI_ISL_758062, EPI_ISL_758063, EPI_ISL_758064, EPI_ISL_758073, EPI_ISL_758074, EPI_ISL_758075, EPI_ISL_758076, EPI_ISL_758077, EPI_ISL_758078, EPI_ISL_758079, EPI_ISL_758080, EPI_ISL_758081, EPI_ISL_758082, EPI_ISL_758090, EPI_ISL_758091, EPI_ISL_758093, EPI_ISL_758103, EPI_ISL_758104, EPI_ISL_758128, EPI_ISL_758129, EPI_ISL_758130, EPI_ISL_758131, EPI_ISL_758132, EPI_ISL_758133, EPI_ISL_758134, EPI_ISL_758135, EPI_ISL_758136, EPI_ISL_758137, EPI_ISL_758138, EPI_ISL_758139, EPI_ISL_758140, EPI_ISL_758141, EPI_ISL_758143, EPI_ISL_758144, EPI_ISL_758145, EPI_ISL_758146, EPI_ISL_758147, EPI_ISL_758148, EPI_ISL_758149, EPI_ISL_758150, EPI_ISL_758151, EPI_ISL_758152, EPI_ISL_758153, EPI_ISL_758154, EPI_ISL_758155, EPI_ISL_758157, EPI_ISL_758158, EPI_ISL_758160, EPI_ISL_758161, EPI_ISL_758162, EPI_ISL_758163, EPI_ISL_758165, EPI_ISL_758166, EPI_ISL_758167, EPI_ISL_758168, EPI_ISL_758169, EPI_ISL_758170, EPI_ISL_758171, EPI_ISL_758172, EPI_ISL_758173, EPI_ISL_758174,			

EPI_ISL_758175, EPI_ISL_758176, EPI_ISL_758177, EPI_ISL_758178, EPI_ISL_758179, EPI_ISL_758181, EPI_ISL_758182, EPI_ISL_758183, EPI_ISL_758184, EPI_ISL_758185, EPI_ISL_758186, EPI_ISL_758187, EPI_ISL_758188, EPI_ISL_758189, EPI_ISL_758190, EPI_ISL_758191, EPI_ISL_758192, EPI_ISL_758193, EPI_ISL_758194, EPI_ISL_758195, EPI_ISL_758196, EPI_ISL_758197, EPI_ISL_758198, EPI_ISL_758199, EPI_ISL_758200, EPI_ISL_758201, EPI_ISL_758202, EPI_ISL_758203, EPI_ISL_758204, EPI_ISL_758205, EPI_ISL_758207, EPI_ISL_758208, EPI_ISL_758209, EPI_ISL_758210, EPI_ISL_758211, EPI_ISL_758212, EPI_ISL_758213, EPI_ISL_758214, EPI_ISL_758215, EPI_ISL_758216, EPI_ISL_758217, EPI_ISL_758218, EPI_ISL_758219, EPI_ISL_758220, EPI_ISL_758221, EPI_ISL_758222, EPI_ISL_758223, EPI_ISL_758224, EPI_ISL_758225, EPI_ISL_758226, EPI_ISL_758227, EPI_ISL_758228, EPI_ISL_758229, EPI_ISL_758230, EPI_ISL_758231, EPI_ISL_758232, EPI_ISL_758233, EPI_ISL_758234, EPI_ISL_758235, EPI_ISL_758236, EPI_ISL_758237, EPI_ISL_758238, EPI_ISL_758239, EPI_ISL_758240, EPI_ISL_758241, EPI_ISL_758242, EPI_ISL_758243, EPI_ISL_758244, EPI_ISL_758246, EPI_ISL_758248, EPI_ISL_758249, EPI_ISL_758250, EPI_ISL_758252, EPI_ISL_758253, EPI_ISL_758254, EPI_ISL_758255, EPI_ISL_758256, EPI_ISL_758257, EPI_ISL_758258, EPI_ISL_758259, EPI_ISL_758260, EPI_ISL_758261, EPI_ISL_758262, EPI_ISL_758263, EPI_ISL_758264, EPI_ISL_758265, EPI_ISL_758267, EPI_ISL_758268, EPI_ISL_758269, EPI_ISL_758270, EPI_ISL_758271, EPI_ISL_758272, EPI_ISL_758273, EPI_ISL_758274, EPI_ISL_758275, EPI_ISL_758276, EPI_ISL_758277, EPI_ISL_758278, EPI_ISL_758279, EPI_ISL_758280, EPI_ISL_758281, EPI_ISL_758282, EPI_ISL_758283, EPI_ISL_758284, EPI_ISL_758285, EPI_ISL_758286, EPI_ISL_758287, EPI_ISL_758288, EPI_ISL_758289, EPI_ISL_758291, EPI_ISL_758292, EPI_ISL_758294, EPI_ISL_758295, EPI_ISL_758296, EPI_ISL_758297, EPI_ISL_758299, EPI_ISL_758300, EPI_ISL_758301, EPI_ISL_758302, EPI_ISL_758303, EPI_ISL_758304, EPI_ISL_758305, EPI_ISL_758306, EPI_ISL_758307, EPI_ISL_758308, EPI_ISL_758309, EPI_ISL_758310, EPI_ISL_758311, EPI_ISL_758312, EPI_ISL_758313, EPI_ISL_758314, EPI_ISL_758317, EPI_ISL_758318, EPI_ISL_758319, EPI_ISL_758320, EPI_ISL_758321, EPI_ISL_758322, EPI_ISL_758323, EPI_ISL_758324, EPI_ISL_758325, EPI_ISL_758326, EPI_ISL_758327, EPI_ISL_758328, EPI_ISL_758329, EPI_ISL_758330, EPI_ISL_758331, EPI_ISL_758332, EPI_ISL_758333, EPI_ISL_758334, EPI_ISL_758335, EPI_ISL_758336, EPI_ISL_758337, EPI_ISL_758338, EPI_ISL_758339, EPI_ISL_758340, EPI_ISL_758341, EPI_ISL_758342, EPI_ISL_758343, EPI_ISL_758344, EPI_ISL_758345, EPI_ISL_758346, EPI_ISL_758347, EPI_ISL_758348, EPI_ISL_758349, EPI_ISL_758350, EPI_ISL_758351, EPI_ISL_758352, EPI_ISL_758353, EPI_ISL_758354, EPI_ISL_758355, EPI_ISL_758358, EPI_ISL_758359, EPI_ISL_758360, EPI_ISL_758361, EPI_ISL_758362, EPI_ISL_758363, EPI_ISL_758364, EPI_ISL_758365, EPI_ISL_758366, EPI_ISL_758367, EPI_ISL_758368, EPI_ISL_758369, EPI_ISL_758370, EPI_ISL_758371, EPI_ISL_758372, EPI_ISL_758373, EPI_ISL_758374, EPI_ISL_758375, EPI_ISL_758377, EPI_ISL_758378, EPI_ISL_758379, EPI_ISL_758380, EPI_ISL_758381, EPI_ISL_758382, EPI_ISL_758383, EPI_ISL_758384, EPI_ISL_758385, EPI_ISL_758387, EPI_ISL_758388, EPI_ISL_758389, EPI_ISL_758390, EPI_ISL_758391, EPI_ISL_758392, EPI_ISL_758393, EPI_ISL_758394, EPI_ISL_758395, EPI_ISL_758396, EPI_ISL_758397, EPI_ISL_758398, EPI_ISL_758399, EPI_ISL_758400, EPI_ISL_758401, EPI_ISL_758402, EPI_ISL_758403, EPI_ISL_758404, EPI_ISL_758405, EPI_ISL_758406, EPI_ISL_758407, EPI_ISL_758409, EPI_ISL_758411, EPI_ISL_758413, EPI_ISL_758414, EPI_ISL_758415, EPI_ISL_758416, EPI_ISL_758417, EPI_ISL_758418, EPI_ISL_758419, EPI_ISL_758420, EPI_ISL_758421, EPI_ISL_758422, EPI_ISL_758423, EPI_ISL_758425, EPI_ISL_758426, EPI_ISL_758427, EPI_ISL_758428, EPI_ISL_758429, EPI_ISL_758430, EPI_ISL_758431, EPI_ISL_758432, EPI_ISL_758433, EPI_ISL_758434, EPI_ISL_758435, EPI_ISL_758436, EPI_ISL_758437, EPI_ISL_758438, EPI_ISL_758439, EPI_ISL_758440, EPI_ISL_758441, EPI_ISL_758442, EPI_ISL_758443, EPI_ISL_758444, EPI_ISL_758445, EPI_ISL_758446, EPI_ISL_758447, EPI_ISL_758448, EPI_ISL_758449, EPI_ISL_758450, EPI_ISL_758451, EPI_ISL_758452, EPI_ISL_758453, EPI_ISL_758454, EPI_ISL_758455, EPI_ISL_758456, EPI_ISL_758457, EPI_ISL_758458, EPI_ISL_758459, EPI_ISL_758460, EPI_ISL_758461, EPI_ISL_758462, EPI_ISL_758463, EPI_ISL_758464, EPI_ISL_758465, EPI_ISL_758466, EPI_ISL_758467, EPI_ISL_758470, EPI_ISL_758471, EPI_ISL_758472, EPI_ISL_758473, EPI_ISL_758474, EPI_ISL_758475, EPI_ISL_758476, EPI_ISL_758477, EPI_ISL_758478, EPI_ISL_758479, EPI_ISL_758480, EPI_ISL_758481, EPI_ISL_758482, EPI_ISL_758483, EPI_ISL_758484, EPI_ISL_758485, EPI_ISL_758486, EPI_ISL_758487, EPI_ISL_758488, EPI_ISL_758489, EPI_ISL_758491, EPI_ISL_758492, EPI_ISL_758493, EPI_ISL_758494, EPI_ISL_758495, EPI_ISL_758496, EPI_ISL_758497, EPI_ISL_758498, EPI_ISL_758499, EPI_ISL_758500, EPI_ISL_758501, EPI_ISL_758502, EPI_ISL_758503, EPI_ISL_758504, EPI_ISL_758505, EPI_ISL_758506, EPI_ISL_758507, EPI_ISL_758508, EPI_ISL_758509, EPI_ISL_758510, EPI_ISL_758511, EPI_ISL_758512, EPI_ISL_758513, EPI_ISL_758514, EPI_ISL_758515, EPI_ISL_758516, EPI_ISL_758517, EPI_ISL_758518, EPI_ISL_758519, EPI_ISL_758520, EPI_ISL_758521, EPI_ISL_758522, EPI_ISL_758523, EPI_ISL_758524, EPI_ISL_758525, EPI_ISL_758526, EPI_ISL_758527, EPI_ISL_758528, EPI_ISL_758529, EPI_ISL_758530, EPI_ISL_758531, EPI_ISL_758532, EPI_ISL_758533, EPI_ISL_758534, EPI_ISL_758535, EPI_ISL_758536, EPI_ISL_758537, EPI_ISL_758538, EPI_ISL_758539, EPI_ISL_758540, EPI_ISL_758541, EPI_ISL_758542, EPI_ISL_758543, EPI_ISL_758545, EPI_ISL_758546, EPI_ISL_758547, EPI_ISL_758548, EPI_ISL_758549, EPI_ISL_758550, EPI_ISL_758551, EPI_ISL_758552, EPI_ISL_758553, EPI_ISL_758554, EPI_ISL_758555, EPI_ISL_758556, EPI_ISL_758557, EPI_ISL_758558, EPI_ISL_758559, EPI_ISL_758560, EPI_ISL_758561, EPI_ISL_758562, EPI_ISL_758563, EPI_ISL_758564, EPI_ISL_758565, EPI_ISL_758566, EPI_ISL_758567, EPI_ISL_758568, EPI_ISL_758570, EPI_ISL_758571, EPI_ISL_758572, EPI_ISL_758573, EPI_ISL_758574, EPI_ISL_758575, EPI_ISL_758576, EPI_ISL_758577, EPI_ISL_758578, EPI_ISL_758579, EPI_ISL_758580, EPI_ISL_758581, EPI_ISL_758582, EPI_ISL_758583, EPI_ISL_758585, EPI_ISL_758586, EPI_ISL_758587, EPI_ISL_758588, EPI_ISL_758589, EPI_ISL_758590, EPI_ISL_758591, EPI_ISL_758592, EPI_ISL_758593, EPI_ISL_758594, EPI_ISL_758595, EPI_ISL_758596, EPI_ISL_758597, EPI_ISL_758598, EPI_ISL_758599, EPI_ISL_758600, EPI_ISL_758602, EPI_ISL_758603, EPI_ISL_758604, EPI_ISL_758605, EPI_ISL_758606, EPI_ISL_758607, EPI_ISL_758608, EPI_ISL_758609, EPI_ISL_758610, EPI_ISL_758611, EPI_ISL_758612, EPI_ISL_758613, EPI_ISL_758614, EPI_ISL_758615, EPI_ISL_758616, EPI_ISL_758617, EPI_ISL_758618, EPI_ISL_758620, EPI_ISL_758622, EPI_ISL_758623, EPI_ISL_758624, EPI_ISL_758625, EPI_ISL_758626, EPI_ISL_758627, EPI_ISL_758629, EPI_ISL_758630, EPI_ISL_758631, EPI_ISL_758632, EPI_ISL_758633, EPI_ISL_758634, EPI_ISL_758635, EPI_ISL_758636, EPI_ISL_758637, EPI_ISL_758638, EPI_ISL_758639, EPI_ISL_758641, EPI_ISL_758642, EPI_ISL_758643, EPI_ISL_758644, EPI_ISL_758645, EPI_ISL_758646, EPI_ISL_758647, EPI_ISL_758648, EPI_ISL_758649, EPI_ISL_758650, EPI_ISL_758652, EPI_ISL_758653, EPI_ISL_758654, EPI_ISL_758655, EPI_ISL_758656, EPI_ISL_758657, EPI_ISL_758659, EPI_ISL_758660, EPI_ISL_758661, EPI_ISL_758663, EPI_ISL_758664, EPI_ISL_758665, EPI_ISL_758666, EPI_ISL_758667, EPI_ISL_758668, EPI_ISL_758669, EPI_ISL_758670, EPI_ISL_758671, EPI_ISL_758672, EPI_ISL_758673, EPI_ISL_758674, EPI_ISL_758675, EPI_ISL_758676, EPI_ISL_758677, EPI_ISL_758678, EPI_ISL_758679, EPI_ISL_758680, EPI_ISL_758681, EPI_ISL_758682, EPI_ISL_758684, EPI_ISL_758685, EPI_ISL_758686, EPI_ISL_758687, EPI_ISL_758688, EPI_ISL_758689, EPI_ISL_758690, EPI_ISL_758691, EPI_ISL_758692, EPI_ISL_758694, EPI_ISL_758695, EPI_ISL_758696, EPI_ISL_758697, EPI_ISL_758698, EPI_ISL_758699, EPI_ISL_758700, EPI_ISL_758869, EPI_ISL_758870, EPI_ISL_758871, EPI_ISL_758872, EPI_ISL_758873, EPI_ISL_758874, EPI_ISL_758875, EPI_ISL_758876, EPI_ISL_758877, EPI_ISL_758878, EPI_ISL_758879, EPI_ISL_758880, EPI_ISL_758881, EPI_ISL_758882, EPI_ISL_758884, EPI_ISL_758885, EPI_ISL_758886, EPI_ISL_758887, EPI_ISL_758888, EPI_ISL_758889, EPI_ISL_758890, EPI_ISL_758892, EPI_ISL_758893, EPI_ISL_758894, EPI_ISL_758895, EPI_ISL_758896, EPI_ISL_758897, EPI_ISL_758898, EPI_ISL_758899, EPI_ISL_758900, EPI_ISL_758901, EPI_ISL_758902, EPI_ISL_758903, EPI_ISL_758904, EPI_ISL_758905, EPI_ISL_758907, EPI_ISL_758908, EPI_ISL_758910, EPI_ISL_758911, EPI_ISL_758914, EPI_ISL_758915, EPI_ISL_758916, EPI_ISL_758917, EPI_ISL_758918, EPI_ISL_758919, EPI_ISL_758920, EPI_ISL_758921, EPI_ISL_758922, EPI_ISL_758923, EPI_ISL_758924, EPI_ISL_758925, EPI_ISL_758926, EPI_ISL_758927, EPI_ISL_758928, EPI_ISL_758929, EPI_ISL_758930, EPI_ISL_758931, EPI_ISL_758932, EPI_ISL_758933, EPI_ISL_758934, EPI_ISL_758935, EPI_ISL_758936, EPI_ISL_758938, EPI_ISL_758939, EPI_ISL_758940, EPI_ISL_758941, EPI_ISL_758942, EPI_ISL_758944, EPI_ISL_758970, EPI_ISL_758971, EPI_ISL_758972, EPI_ISL_758973, EPI_ISL_758974, EPI_ISL_758975, EPI_ISL_758976, EPI_ISL_758977, EPI_ISL_758978, EPI_ISL_758979, EPI_ISL_758980, EPI_ISL_758981, EPI_ISL_758982, EPI_ISL_758983, EPI_ISL_758984, EPI_ISL_758985, EPI_ISL_758986, EPI_ISL_758987, EPI_ISL_758988, EPI_ISL_758990, EPI_ISL_758991, EPI_ISL_758992, EPI_ISL_758993, EPI_ISL_758994, EPI_ISL_758995, EPI_ISL_758996, EPI_ISL_758997, EPI_ISL_758998, EPI_ISL_758999, EPI_ISL_759000, EPI_ISL_759001, EPI_ISL_759002, EPI_ISL_759003, EPI_ISL_759004, EPI_ISL_759005, EPI_ISL_759006, EPI_ISL_759007, EPI_ISL_759008, EPI_ISL_759009, EPI_ISL_759011, EPI_ISL_759012, EPI_ISL_759013, EPI_ISL_759014, EPI_ISL_759015, EPI_ISL_759016, EPI_ISL_759017, EPI_ISL_759018, EPI_ISL_759019, EPI_ISL_759020, EPI_ISL_759021, EPI_ISL_759022, EPI_ISL_759023, EPI_ISL_759024, EPI_ISL_759025, EPI_ISL_759026, EPI_ISL_759028, EPI_ISL_759029, EPI_ISL_759030, EPI_ISL_759031, EPI_ISL_759032, EPI_ISL_759033, EPI_ISL_759034, EPI_ISL_759035, EPI_ISL_759037, EPI_ISL_759038, EPI_ISL_759039, EPI_ISL_759040, EPI_ISL_759046, EPI_ISL_759047, EPI_ISL_759048, EPI_ISL_759049, EPI_ISL_759050, EPI_ISL_759051, EPI_ISL_759052, EPI_ISL_759053, EPI_ISL_759054, EPI_ISL_759055, EPI_ISL_759056, EPI_ISL_759057, EPI_ISL_759058, EPI_ISL_759059, EPI_ISL_759065, EPI_ISL_759075, EPI_ISL_759076, EPI_ISL_759104, EPI_ISL_759105, EPI_ISL_759106, EPI_ISL_759107, EPI_ISL_759108, EPI_ISL_759109, EPI_ISL_759110, EPI_ISL_759111, EPI_ISL_759112, EPI_ISL_759113, EPI_ISL_759114, EPI_ISL_759115, EPI_ISL_759116, EPI_ISL_759117, EPI_ISL_759118, EPI_ISL_759119, EPI_ISL_759120, EPI_ISL_759121, EPI_ISL_759122, EPI_ISL_759123, EPI_ISL_759124, EPI_ISL_759125, EPI_ISL_759126, EPI_ISL_759127, EPI_ISL_759128, EPI_ISL_759129, EPI_ISL_759130, EPI_ISL_759131, EPI_ISL_759132, EPI_ISL_759133, EPI_ISL_759134, EPI_ISL_759135, EPI_ISL_759136, EPI_ISL_759137, EPI_ISL_759141, EPI_ISL_759142, EPI_ISL_759143, EPI_ISL_759144, EPI_ISL_759145, EPI_ISL_759146, EPI_ISL_759147, EPI_ISL_759148, EPI_ISL_759149, EPI_ISL_759150, EPI_ISL_759151, EPI_ISL_759152, EPI_ISL_759153, EPI_ISL_759154, EPI_ISL_759155, EPI_ISL_759156, EPI_ISL_759157, EPI_ISL_759158, EPI_ISL_759159, EPI_ISL_759160, EPI_ISL_759161, EPI_ISL_759162, EPI_ISL_759164, EPI_ISL_759165, EPI_ISL_759166, EPI_ISL_759167, EPI_ISL_759168, EPI_ISL_759169, EPI_ISL_759171, EPI_ISL_759172, EPI_ISL_759173, EPI_ISL_759174, EPI_ISL_759175, EPI_ISL_759176, EPI_ISL_759177, EPI_ISL_759178, EPI_ISL_759179, EPI_ISL_759180, EPI_ISL_759181, EPI_ISL_759182, EPI_ISL_759183, EPI_ISL_759184, EPI_ISL_759185, EPI_ISL_759186, EPI_ISL_759187, EPI_ISL_759188, EPI_ISL_759189, EPI_ISL_759190, EPI_ISL_759192, EPI_ISL_759193, EPI_ISL_759194, EPI_ISL_759195, EPI_ISL_759196, EPI_ISL_759197, EPI_ISL_759198, EPI_ISL_759199, EPI_ISL_759200, EPI_ISL_759202, EPI_ISL_759203, EPI_ISL_759204, EPI_ISL_759205, EPI_ISL_759206, EPI_ISL_759207, EPI_ISL_759208, EPI_ISL_759209, EPI_ISL_759210, EPI_ISL_759211, EPI_ISL_759212, EPI_ISL_759213, EPI_ISL_759214, EPI_ISL_759215, EPI_ISL_759216, EPI_ISL_759217, EPI_ISL_759218, EPI_ISL_759219, EPI_ISL_759220, EPI_ISL_759221, EPI_ISL_759222, EPI_ISL_759223, EPI_ISL_759224, EPI_ISL_759225, EPI_ISL_759226, EPI_ISL_759227, EPI_ISL_759229, EPI_ISL_759230, EPI_ISL_759231, EPI_ISL_759232, EPI_ISL_759233, EPI_ISL_759234, EPI_ISL_759235, EPI_ISL_759236, EPI_ISL_759237, EPI_ISL_759238, EPI_ISL_759241, EPI_ISL_759242, EPI_ISL_759243, EPI_ISL_759244, EPI_ISL_759245, EPI_ISL_759246, EPI_ISL_759247, EPI_ISL_759248, EPI_ISL_759249, EPI_ISL_759250, EPI_ISL_759251, EPI_ISL_759252, EPI_ISL_759253, EPI_ISL_759254, EPI_ISL_759255, EPI_ISL_759256, EPI_ISL_759257, EPI_ISL_759258, EPI_ISL_759259, EPI_ISL_759260, EPI_ISL_759261, EPI_ISL_759262, EPI_ISL_759263, EPI_ISL_759265, EPI_ISL_759266, EPI_ISL_759267, EPI_ISL_759268, EPI_ISL_759269, EPI_ISL_759271, EPI_ISL_759273, EPI_ISL_759274, EPI_ISL_759275, EPI_ISL_759276, EPI_ISL_759278, EPI_ISL_759279, EPI_ISL_759281, EPI_ISL_759282, EPI_ISL_759283, EPI_ISL_759284, EPI_ISL_759286, EPI_ISL_759287, EPI_ISL_759288, EPI_ISL_759289, EPI_ISL_759290, EPI_ISL_759291, EPI_ISL_759292, EPI_ISL_759293, EPI_ISL_759294, EPI_ISL_759295, EPI_ISL_759296, EPI_ISL_759297, EPI_ISL_759298, EPI_ISL_759299, EPI_ISL_759300, EPI_ISL_759301, EPI_ISL_759302, EPI_ISL_759303, EPI_ISL_759304, EPI_ISL_759305, EPI_ISL_759306, EPI_ISL_759307, EPI_ISL_759308, EPI_ISL_759309, EPI_ISL_759310, EPI_ISL_759311, EPI_ISL_759312, EPI_ISL_759313, EPI_ISL_759315, EPI_ISL_759316, EPI_ISL_759317, EPI_ISL_759318, EPI_ISL_759319, EPI_ISL_759320, EPI_ISL_759321, EPI_ISL_759322, EPI_ISL_759323, EPI_ISL_759324, EPI_ISL_759325, EPI_ISL_759326, EPI_ISL_759328, EPI_ISL_759329, EPI_ISL_759330, EPI_ISL_759332, EPI_ISL_759333, EPI_ISL_759334, EPI_ISL_759335, EPI_ISL_759336, EPI_ISL_759337, EPI_ISL_759338, EPI_ISL_759339, EPI_ISL_759341, EPI_ISL_759342, EPI_ISL_759343, EPI_ISL_759345, EPI_ISL_759346, EPI_ISL_759347, EPI_ISL_759348, EPI_ISL_759349, EPI_ISL_759350, EPI_ISL_759351, EPI_ISL_759352, EPI_ISL_759353, EPI_ISL_759354, EPI_ISL_759355, EPI_ISL_759356, EPI_ISL_759357, EPI_ISL_759358, EPI_ISL_759359, EPI_ISL_759360, EPI_ISL_759362, EPI_ISL_759364, EPI_ISL_759366, EPI_ISL_759367, EPI_ISL_759368, EPI_ISL_759369, EPI_ISL_759371, EPI_ISL_759372, EPI_ISL_759373, EPI_ISL_759374, EPI_ISL_759375, EPI_ISL_759376, EPI_ISL_759377, EPI_ISL_759378, EPI_ISL_759379, EPI_ISL_759380, EPI_ISL_759381, EPI_ISL_759382, EPI_ISL_759383, EPI_ISL_759384, EPI_ISL_759386, EPI_ISL_759387, EPI_ISL_759388, EPI_ISL_759389, EPI_ISL_759390, EPI_ISL_759391, EPI_ISL_759393, EPI_ISL_759394, EPI_ISL_759395, EPI_ISL_759396, EPI_ISL_759397, EPI_ISL_759398, EPI_ISL_759399, EPI_ISL_759400, EPI_ISL_759401, EPI_ISL_759402, EPI_ISL_759403, EPI_ISL_759404, EPI_ISL_759405, EPI_ISL_759407, EPI_ISL_759408, EPI_ISL_759409, EPI_ISL_759410, EPI_ISL_759412, EPI_ISL_759413, EPI_ISL_759414, EPI_ISL_759415, EPI_ISL_759416, EPI_ISL_759417, EPI_ISL_759418, EPI_ISL_759419, EPI_ISL_759420, EPI_ISL_759421, EPI_ISL_759422, EPI_ISL_759423, EPI_ISL_759424, EPI_ISL_759425, EPI_ISL_759426, EPI_ISL_759427, EPI_ISL_759428, EPI_ISL_759429, EPI_ISL_759430, EPI_ISL_759431, EPI_ISL_759432, EPI_ISL_759433, EPI_ISL_759434, EPI_ISL_759435, EPI_ISL_759436, EPI_ISL_759437, EPI_ISL_759438, EPI_ISL_759440, EPI_ISL_759441, EPI_ISL_759442, EPI_ISL_759443, EPI_ISL_759444, EPI_ISL_759445, EPI_ISL_759446, EPI_ISL_759447, EPI_ISL_759448, EPI_ISL_759449, EPI_ISL_759450, EPI_ISL_759451, EPI_ISL_759452, EPI_ISL_759453, EPI_ISL_759454, EPI_ISL_759455, EPI_ISL_759456, EPI_ISL_759457, EPI_ISL_759458, EPI_ISL_759459, EPI_ISL_759461, EPI_ISL_759462, EPI_ISL_759463, EPI_ISL_759464, EPI_ISL_759465, EPI_ISL_759466, EPI_ISL_759467, EPI_ISL_759468, EPI_ISL_759469, EPI_ISL_759471, EPI_ISL_759472, EPI_ISL_759473, EPI_ISL_759475, EPI_ISL_759476, EPI_ISL_759477, EPI_ISL_759478, EPI_ISL_759479, EPI_ISL_759480, EPI_ISL_759481, EPI_ISL_759482, EPI_ISL_759483, EPI_ISL_759484, EPI_ISL_759485, EPI_ISL_759486, EPI_ISL_759487, EPI_ISL_759488, EPI_ISL_759489, EPI_ISL_759490, EPI_ISL_759491, EPI_ISL_759492, EPI_ISL_759493, EPI_ISL_759494, EPI_ISL_759496, EPI_ISL_759497, EPI_ISL_759499, EPI_ISL_759500, EPI_ISL_759501, EPI_ISL_759502, EPI_ISL_759503, EPI_ISL_759504, EPI_ISL_759505, EPI_ISL_759506, EPI_ISL_759507, EPI_ISL_759508, EPI_ISL_759509, EPI_ISL_759510, EPI_ISL_759511, EPI_ISL_759512, EPI_ISL_759514, EPI_ISL_759516, EPI_ISL_759517, EPI_ISL_759518, EPI_ISL_759519, EPI_ISL_759520, EPI_ISL_759521, EPI_ISL_759522, EPI_ISL_759523, EPI_ISL_759524, EPI_ISL_759525, EPI_ISL_759526, EPI_ISL_759527, EPI_ISL_759528, EPI_ISL_759529, EPI_ISL_759530, EPI_ISL_759531, EPI_ISL_759532, EPI_ISL_759536, EPI_ISL_759537, EPI_ISL_759538, EPI_ISL_759539, EPI_ISL_759540, EPI_ISL_759541, EPI_ISL_759542, EPI_ISL_759543, EPI_ISL_759544, EPI_ISL_759546, EPI_ISL_759547, EPI_ISL_759548, EPI_ISL_759549, EPI_ISL_759550, EPI_ISL_759551, EPI_ISL_759552, EPI_ISL_759553, EPI_ISL_759554, EPI_ISL_759555, EPI_ISL_759556, EPI_ISL_759557, EPI_ISL_759558, EPI_ISL_759559, EPI_ISL_759560, EPI_ISL_759561, EPI_ISL_759562, EPI_ISL_759563, EPI_ISL_759564, EPI_ISL_759565, EPI_ISL_759566, EPI_ISL_759567, EPI_ISL_759568, EPI_ISL_759569, EPI_ISL_759570, EPI_ISL_759571, EPI_ISL_759572, EPI_ISL_759573, EPI_ISL_759574, EPI_ISL_759575, EPI_ISL_759576, EPI_ISL_759577, EPI_ISL_759578, EPI_ISL_759580, EPI_ISL_759581, EPI_ISL_759582, EPI_ISL_759583, EPI_ISL_759584, EPI_ISL_759585, EPI_ISL_759586, EPI_ISL_759587, EPI_ISL_759588, EPI_ISL_759589, EPI_ISL_759590, EPI_ISL_759591, EPI_ISL_759592, EPI_ISL_759593, EPI_ISL_759594, EPI_ISL_759595, EPI_ISL_759596, EPI_ISL_759597, EPI_ISL_759598, EPI_ISL_759599, EPI_ISL_759601, EPI_ISL_759602, EPI_ISL_759603, EPI_ISL_759604, EPI_ISL_759605, EPI_ISL_759606, EPI_ISL_759607, EPI_ISL_759608, EPI_ISL_759609, EPI_ISL_759610, EPI_ISL_759611, EPI_ISL_759612, EPI_ISL_759613, EPI_ISL_759614, EPI_ISL_759615, EPI_ISL_759616, EPI_ISL_759617, EPI_ISL_759618, EPI_ISL_759619, EPI_ISL_759620, EPI_ISL_759621, EPI_ISL_759622, EPI_ISL_759623, EPI_ISL_759624, EPI_ISL_759625, EPI_ISL_759626, EPI_ISL_759627, EPI_ISL_759628, EPI_ISL_759629, EPI_ISL_759630, EPI_ISL_759631, EPI_ISL_759633, EPI_ISL_759634, EPI_ISL_759635, EPI_ISL_759636, EPI_ISL_759637, EPI_ISL_759638, EPI_ISL_759639, EPI_ISL_759640, EPI_ISL_759641, EPI_ISL_759642, EPI_ISL_759643, EPI_ISL_759644, EPI_ISL_759645, EPI_ISL_759646, EPI_ISL_759647, EPI_ISL_759648, EPI_ISL_759649, EPI_ISL_759650, EPI_ISL_759651, EPI_ISL_759652, EPI_ISL_759653, EPI_ISL_759654, EPI_ISL_759655, EPI_ISL_759656, EPI_ISL_759657, EPI_ISL_759658, EPI_ISL_759659, EPI_ISL_759660, EPI_ISL_759662, EPI_ISL_759663, EPI_ISL_759664, EPI_ISL_759665, EPI_ISL_759666, EPI_ISL_759667, EPI_ISL_759668, EPI_ISL_759669, EPI_ISL_759670, EPI_ISL_759671, EPI_ISL_759672, EPI_ISL_759673, EPI_ISL_759674, EPI_ISL_759675, EPI_ISL_759676, EPI_ISL_759677, EPI_ISL_759678, EPI_ISL_759681, EPI_ISL_759682, EPI_ISL_759683, EPI_ISL_759684, EPI_ISL_759686, EPI_ISL_759687, EPI_ISL_759688, EPI_ISL_759689, EPI_ISL_759690, EPI_ISL_759691, EPI_ISL_759692, EPI_ISL_759693, EPI_ISL_759694, EPI_ISL_759695, EPI_ISL_759696, EPI_ISL_759697, EPI_ISL_759699, EPI_ISL_759700

see above	Department of Virus and Microbiological Special Diagnostics, Statens Serum Institut, Copenhagen, Denmark	Albertsen Lab, Department of Chemistry and Bioscience, Aalborg University, Denmark	Danish Covid-19 Genome Consortium
EPI_ISL_759707, EPI_ISL_759708, EPI_ISL_759711, EPI_ISL_759716, EPI_ISL_759719	Instituto Nacional de Saude (INSA)	Instituto Nacional de Saude (INSA)	Borges et al

EPI_ISL_759725, EPI_ISL_759728, EPI_ISL_759730, EPI_ISL_759733, EPI_ISL_759734, EPI_ISL_759743, EPI_ISL_759744, EPI_ISL_759746, EPI_ISL_759747, EPI_ISL_759749, EPI_ISL_759750, EPI_ISL_759752, EPI_ISL_759758

see above	University of Wisconsin-Madison AIDS Vaccine Research Laboratories	University of Wisconsin-Madison AIDS Vaccine Research Laboratories	Gage Moreno, Katarina Braun, et al. AIDS Vaccine Research Laboratories
EPI_ISL_759893, EPI_ISL_759894	Temporary Specimen Collection Centre	Hong Kong Department of Health	Alan K.L. Tsang, Peter C.W. Yip, Edman T.K. Lam, Rickjason C.W. Chan, Dominic N.C. Tsang
EPI_ISL_759895	United Christian Hospital	Hong Kong Department of Health	Alan K.L. Tsang, Peter C.W. Yip, Edman T.K. Lam, Rickjason C.W. Chan, Dominic N.C. Tsang
EPI_ISL_759896	Queen Elizabeth Hospital	Hong Kong Department of Health	Alan K.L. Tsang, Peter C.W. Yip, Edman T.K. Lam, Rickjason C.W. Chan, Dominic N.C. Tsang
EPI_ISL_759897, EPI_ISL_759898	United Christian Hospital	Hong Kong Department of Health	Alan K.L. Tsang, Peter C.W. Yip, Edman T.K. Lam, Rickjason C.W. Chan, Dominic N.C. Tsang
EPI_ISL_759986	University Hospital of Northern Norway, Department for Microbiology and Infectious Disease Control	Norwegian Institute of Public Health, Department of Virology	Kathrine Stene-Johansen, Kamilla Heddeland Instefjord, Hilde Elshaug, Marie Paulsen Madsen, Rasmus Riis Kopperud, Hilde Vollan, Karoline Bragstad, Olav Hungnes
EPI_ISL_760249	Lighthouse Lab in Alderley Park	Wellcome Sanger Institute for the COVID-19 Genomics UK	Jacquelyn Wynn, Mairead Hyland, The Lighthouse Lab in Alderley Park and Alex Alderton, Roberto Amato, Sonia Goncalves, Ewan Harrison, David K.

		(COG-UK) Consortium	Jackson, Ian Johnston, Dominic Kwiatkowski, Cordelia Langford, John Sillitoe on behalf of the Wellcome Sanger Institute COVID-19 Surveillance Team
EPI_ISL_760250, EPI_ISL_760251, EPI_ISL_760252, EPI_ISL_760254, EPI_ISL_760255, EPI_ISL_760256, EPI_ISL_760257	Lighthouse Lab in Glasgow	Wellcome Sanger Institute for the COVID-19 Genomics UK (COG-UK) Consortium	Harper VanSteenhouse, Yumi Kasai, David Gray, Carol Clugston, Anna Dominiczak and Alex Alderton, Roberto Amato, Sonia Goncalves, Ewan Harrison, David K. Jackson, Ian Johnston, Dominic Kwiatkowski, Cordelia Langford, John Sillitoe on behalf of the Wellcome Sanger Institute COVID-19 Surveillance Team
EPI_ISL_760259	Lighthouse Lab in Alderley Park	Wellcome Sanger Institute for the COVID-19 Genomics UK (COG-UK) Consortium	Jacquelyn Wynn, Mairead Hyland, The Lighthouse Lab in Alderley Park and Alex Alderton, Roberto Amato, Sonia Goncalves, Ewan Harrison, David K. Jackson, Ian Johnston, Dominic Kwiatkowski, Cordelia Langford, John Sillitoe on behalf of the Wellcome Sanger Institute COVID-19 Surveillance Team
EPI_ISL_760260	Lighthouse Lab in Glasgow	Wellcome Sanger Institute for the COVID-19 Genomics UK (COG-UK) Consortium	Harper VanSteenhouse, Yumi Kasai, David Gray, Carol Clugston, Anna Dominiczak and Alex Alderton, Roberto Amato, Sonia Goncalves, Ewan Harrison, David K. Jackson, Ian Johnston, Dominic Kwiatkowski, Cordelia Langford, John Sillitoe on behalf of the Wellcome Sanger Institute COVID-19 Surveillance Team
EPI_ISL_760261	Lighthouse Lab in Alderley Park	Wellcome Sanger Institute for the COVID-19 Genomics UK (COG-UK) Consortium	Jacquelyn Wynn, Mairead Hyland, The Lighthouse Lab in Alderley Park and Alex Alderton, Roberto Amato, Sonia Goncalves, Ewan Harrison, David K. Jackson, Ian Johnston, Dominic Kwiatkowski, Cordelia Langford, John Sillitoe on behalf of the Wellcome Sanger Institute COVID-19 Surveillance Team
EPI_ISL_760262	Lighthouse Lab in Glasgow	Wellcome Sanger Institute for the COVID-19 Genomics UK (COG-UK) Consortium	Harper VanSteenhouse, Yumi Kasai, David Gray, Carol Clugston, Anna Dominiczak and Alex Alderton, Roberto Amato, Sonia Goncalves, Ewan Harrison, David K. Jackson, Ian Johnston, Dominic Kwiatkowski, Cordelia Langford, John Sillitoe on behalf of the Wellcome Sanger Institute COVID-19 Surveillance Team
EPI_ISL_760263	Lighthouse Lab in Alderley Park	Wellcome Sanger Institute for the COVID-19 Genomics UK (COG-UK) Consortium	Jacquelyn Wynn, Mairead Hyland, The Lighthouse Lab in Alderley Park and Alex Alderton, Roberto Amato, Sonia Goncalves, Ewan Harrison, David K. Jackson, Ian Johnston, Dominic Kwiatkowski, Cordelia Langford, John Sillitoe on behalf of the Wellcome Sanger Institute COVID-19 Surveillance Team
EPI_ISL_760264	Lighthouse Lab in Glasgow	Wellcome Sanger Institute for the COVID-19 Genomics UK (COG-UK) Consortium	Harper VanSteenhouse, Yumi Kasai, David Gray, Carol Clugston, Anna Dominiczak and Alex Alderton, Roberto Amato, Sonia Goncalves, Ewan Harrison, David K. Jackson, Ian Johnston, Dominic Kwiatkowski, Cordelia Langford, John Sillitoe on behalf of the Wellcome Sanger Institute COVID-19 Surveillance Team
EPI_ISL_760266, EPI_ISL_760267, EPI_ISL_760268	Lighthouse Lab in Alderley Park	Wellcome Sanger Institute for the COVID-19 Genomics UK (COG-UK) Consortium	Jacquelyn Wynn, Mairead Hyland, The Lighthouse Lab in Alderley Park and Alex Alderton, Roberto Amato, Sonia Goncalves, Ewan Harrison, David K. Jackson, Ian Johnston, Dominic Kwiatkowski, Cordelia Langford, John Sillitoe on behalf of the Wellcome Sanger Institute COVID-19 Surveillance Team
EPI_ISL_760269, EPI_ISL_760271, EPI_ISL_760272	Lighthouse Lab in Glasgow	Wellcome Sanger Institute for the COVID-19 Genomics UK (COG-UK) Consortium	Harper VanSteenhouse, Yumi Kasai, David Gray, Carol Clugston, Anna Dominiczak and Alex Alderton, Roberto Amato, Sonia Goncalves, Ewan Harrison, David K. Jackson, Ian Johnston, Dominic Kwiatkowski, Cordelia Langford, John Sillitoe on behalf of the Wellcome Sanger Institute COVID-19 Surveillance Team
EPI_ISL_760273	Lighthouse Lab in Alderley Park	Wellcome Sanger Institute for the COVID-19 Genomics UK (COG-UK) Consortium	Jacquelyn Wynn, Mairead Hyland, The Lighthouse Lab in Alderley Park and Alex Alderton, Roberto Amato, Sonia Goncalves, Ewan Harrison, David K. Jackson, Ian Johnston, Dominic Kwiatkowski, Cordelia Langford, John Sillitoe on behalf of the Wellcome Sanger Institute COVID-19 Surveillance Team
EPI_ISL_760274, EPI_ISL_760275	Lighthouse Lab in Glasgow	Wellcome Sanger Institute for the COVID-19 Genomics UK (COG-UK) Consortium	Harper VanSteenhouse, Yumi Kasai, David Gray, Carol Clugston, Anna Dominiczak and Alex Alderton, Roberto Amato, Sonia Goncalves, Ewan Harrison, David K. Jackson, Ian Johnston, Dominic Kwiatkowski, Cordelia Langford, John Sillitoe on behalf of the Wellcome Sanger Institute COVID-19 Surveillance Team
EPI_ISL_760276	Lighthouse Lab in Alderley Park	Wellcome Sanger Institute for the COVID-19 Genomics UK (COG-UK) Consortium	Jacquelyn Wynn, Mairead Hyland, The Lighthouse Lab in Alderley Park and Alex Alderton, Roberto Amato, Sonia Goncalves, Ewan Harrison, David K. Jackson, Ian Johnston, Dominic Kwiatkowski, Cordelia Langford, John Sillitoe on behalf of the Wellcome Sanger Institute COVID-19 Surveillance Team
EPI_ISL_760277, EPI_ISL_760279, EPI_ISL_760280	Lighthouse Lab in Glasgow	Wellcome Sanger Institute for the COVID-19 Genomics UK (COG-UK) Consortium	Harper VanSteenhouse, Yumi Kasai, David Gray, Carol Clugston, Anna Dominiczak and Alex Alderton, Roberto Amato, Sonia Goncalves, Ewan Harrison, David K. Jackson, Ian Johnston, Dominic Kwiatkowski, Cordelia Langford, John Sillitoe on behalf of the Wellcome Sanger Institute COVID-19 Surveillance Team
EPI_ISL_760281	Lighthouse Lab in Alderley Park	Wellcome Sanger Institute for the COVID-19 Genomics UK (COG-UK) Consortium	Jacquelyn Wynn, Mairead Hyland, The Lighthouse Lab in Alderley Park and Alex Alderton, Roberto Amato, Sonia Goncalves, Ewan Harrison, David K. Jackson, Ian Johnston, Dominic Kwiatkowski, Cordelia Langford, John Sillitoe on behalf of the Wellcome Sanger Institute COVID-19 Surveillance Team
EPI_ISL_760282, EPI_ISL_760283	Lighthouse Lab in Glasgow	Wellcome Sanger Institute for the COVID-19 Genomics UK (COG-UK) Consortium	Harper VanSteenhouse, Yumi Kasai, David Gray, Carol Clugston, Anna Dominiczak and Alex Alderton, Roberto Amato, Sonia Goncalves, Ewan Harrison, David K. Jackson, Ian Johnston, Dominic Kwiatkowski, Cordelia Langford, John Sillitoe on behalf of the Wellcome Sanger Institute COVID-19 Surveillance Team
EPI_ISL_760284, EPI_ISL_760286	Lighthouse Lab in Alderley Park	Wellcome Sanger Institute for the COVID-19 Genomics UK (COG-UK) Consortium	Jacquelyn Wynn, Mairead Hyland, The Lighthouse Lab in Alderley Park and Alex Alderton, Roberto Amato, Sonia Goncalves, Ewan Harrison, David K. Jackson, Ian Johnston, Dominic Kwiatkowski, Cordelia Langford, John Sillitoe on behalf of the Wellcome Sanger Institute COVID-19 Surveillance Team
EPI_ISL_760287, EPI_ISL_760289, EPI_ISL_760291, EPI_ISL_760294	Lighthouse Lab in Glasgow	Wellcome Sanger Institute for the COVID-19 Genomics UK (COG-UK) Consortium	Harper VanSteenhouse, Yumi Kasai, David Gray, Carol Clugston, Anna Dominiczak and Alex Alderton, Roberto Amato, Sonia Goncalves, Ewan Harrison, David K. Jackson, Ian Johnston, Dominic Kwiatkowski, Cordelia Langford, John Sillitoe on behalf of the Wellcome Sanger Institute COVID-19 Surveillance Team
EPI_ISL_760296	Lighthouse Lab in Alderley Park	Wellcome Sanger Institute for the COVID-19 Genomics UK (COG-UK) Consortium	Jacquelyn Wynn, Mairead Hyland, The Lighthouse Lab in Alderley Park and Alex Alderton, Roberto Amato, Sonia Goncalves, Ewan Harrison, David K. Jackson, Ian Johnston, Dominic Kwiatkowski, Cordelia Langford, John Sillitoe on behalf of the Wellcome Sanger Institute COVID-19 Surveillance Team
EPI_ISL_760300	Lighthouse Lab in Glasgow	Wellcome Sanger Institute for the COVID-19 Genomics UK (COG-UK) Consortium	Harper VanSteenhouse, Yumi Kasai, David Gray, Carol Clugston, Anna Dominiczak and Alex Alderton, Roberto Amato, Sonia Goncalves, Ewan Harrison, David K. Jackson, Ian Johnston, Dominic Kwiatkowski, Cordelia Langford, John Sillitoe on behalf of the Wellcome Sanger Institute COVID-19 Surveillance Team
EPI_ISL_760301	Lighthouse Lab in Alderley Park	Wellcome Sanger Institute for the COVID-19 Genomics UK (COG-UK) Consortium	Jacquelyn Wynn, Mairead Hyland, The Lighthouse Lab in Alderley Park and Alex Alderton, Roberto Amato, Sonia Goncalves, Ewan Harrison, David K. Jackson, Ian Johnston, Dominic Kwiatkowski, Cordelia Langford, John Sillitoe on behalf of the Wellcome Sanger Institute COVID-19 Surveillance Team
EPI_ISL_760302	Lighthouse Lab in Glasgow	Wellcome Sanger Institute for the COVID-19 Genomics UK (COG-UK) Consortium	Harper VanSteenhouse, Yumi Kasai, David Gray, Carol Clugston, Anna Dominiczak and Alex Alderton, Roberto Amato, Sonia Goncalves, Ewan Harrison, David K. Jackson, Ian Johnston, Dominic Kwiatkowski, Cordelia Langford, John Sillitoe on behalf of the Wellcome Sanger Institute COVID-19 Surveillance Team
EPI_ISL_760305, EPI_ISL_760306	Lighthouse Lab in Alderley Park	Wellcome Sanger Institute for the COVID-19 Genomics UK (COG-UK) Consortium	Jacquelyn Wynn, Mairead Hyland, The Lighthouse Lab in Alderley Park and Alex Alderton, Roberto Amato, Sonia Goncalves, Ewan Harrison, David K. Jackson, Ian Johnston, Dominic Kwiatkowski, Cordelia Langford, John Sillitoe on behalf of the Wellcome Sanger Institute COVID-19 Surveillance Team
EPI_ISL_760307, EPI_ISL_760308, EPI_ISL_760309, EPI_ISL_760315, EPI_ISL_760316, EPI_ISL_760317, EPI_ISL_760319, EPI_ISL_760320, EPI_ISL_760322, EPI_ISL_760324, EPI_ISL_760325			
see above	Lighthouse Lab in Glasgow	Wellcome Sanger Institute for the COVID-19 Genomics UK (COG-UK) Consortium	Harper VanSteenhouse, Yumi Kasai, David Gray, Carol Clugston, Anna Dominiczak and Alex Alderton, Roberto Amato, Sonia Goncalves, Ewan Harrison, David K. Jackson, Ian Johnston, Dominic Kwiatkowski, Cordelia Langford, John Sillitoe on behalf of the Wellcome Sanger Institute COVID-19 Surveillance Team
EPI_ISL_760326	Lighthouse Lab in Alderley Park	Wellcome Sanger Institute for the COVID-19 Genomics UK (COG-UK) Consortium	Jacquelyn Wynn, Mairead Hyland, The Lighthouse Lab in Alderley Park and Alex Alderton, Roberto Amato, Sonia Goncalves, Ewan Harrison, David K. Jackson, Ian Johnston, Dominic Kwiatkowski, Cordelia Langford, John Sillitoe on behalf of the Wellcome Sanger Institute COVID-19 Surveillance Team
EPI_ISL_760331, EPI_ISL_760334, EPI_ISL_760335, EPI_ISL_760336, EPI_ISL_760337, EPI_ISL_760338, EPI_ISL_760340	Lighthouse Lab in Glasgow	Wellcome Sanger Institute for the COVID-19 Genomics UK (COG-UK) Consortium	Harper VanSteenhouse, Yumi Kasai, David Gray, Carol Clugston, Anna Dominiczak and Alex Alderton, Roberto Amato, Sonia Goncalves, Ewan Harrison, David K. Jackson, Ian Johnston, Dominic Kwiatkowski, Cordelia Langford, John Sillitoe on behalf of the Wellcome Sanger Institute COVID-19 Surveillance Team
EPI_ISL_760341	Lighthouse Lab in Alderley Park	Wellcome Sanger Institute for the COVID-19 Genomics UK (COG-UK) Consortium	Jacquelyn Wynn, Mairead Hyland, The Lighthouse Lab in Alderley Park and Alex Alderton, Roberto Amato, Sonia Goncalves, Ewan Harrison, David K. Jackson, Ian Johnston, Dominic Kwiatkowski, Cordelia Langford, John Sillitoe on behalf of the Wellcome Sanger Institute COVID-19 Surveillance Team
EPI_ISL_760342, EPI_ISL_760343	Lighthouse Lab in Glasgow	Wellcome Sanger Institute for the COVID-19 Genomics UK (COG-UK) Consortium	Harper VanSteenhouse, Yumi Kasai, David Gray, Carol Clugston, Anna Dominiczak and Alex Alderton, Roberto Amato, Sonia Goncalves, Ewan Harrison, David K. Jackson, Ian Johnston, Dominic Kwiatkowski, Cordelia Langford, John Sillitoe on behalf of the Wellcome Sanger Institute COVID-19 Surveillance Team
EPI_ISL_760344	Lighthouse Lab in Alderley Park	Wellcome Sanger Institute for the COVID-19 Genomics UK	Jacquelyn Wynn, Mairead Hyland, The Lighthouse Lab in Alderley Park and Alex Alderton, Roberto Amato, Sonia Goncalves, Ewan Harrison, David K.

		(COG-UK) Consortium	Jackson, Ian Johnston, Dominic Kwiatkowski, Cordelia Langford, John Sillitoe on behalf of the Wellcome Sanger Institute COVID-19 Surveillance Team
EPI_ISL_760345	Lighthouse Lab in Glasgow	Wellcome Sanger Institute for the COVID-19 Genomics UK (COG-UK) Consortium	Harper VanSteenhouse, Yumi Kasai, David Gray, Carol Clugston, Anna Dominiczak and Alex Alderton, Roberto Amato, Sonia Goncalves, Ewan Harrison, David K. Jackson, Ian Johnston, Dominic Kwiatkowski, Cordelia Langford, John Sillitoe on behalf of the Wellcome Sanger Institute COVID-19 Surveillance Team
EPI_ISL_760346	Lighthouse Lab in Alderley Park	Wellcome Sanger Institute for the COVID-19 Genomics UK (COG-UK) Consortium	Jacquelyn Wynn, Mairead Hyland, The Lighthouse Lab in Alderley Park and Alex Alderton, Roberto Amato, Sonia Goncalves, Ewan Harrison, David K. Jackson, Ian Johnston, Dominic Kwiatkowski, Cordelia Langford, John Sillitoe on behalf of the Wellcome Sanger Institute COVID-19 Surveillance Team
EPI_ISL_760347, EPI_ISL_760349, EPI_ISL_760351, EPI_ISL_760352, EPI_ISL_760353, EPI_ISL_760356, EPI_ISL_760357	Lighthouse Lab in Glasgow	Wellcome Sanger Institute for the COVID-19 Genomics UK (COG-UK) Consortium	Harper VanSteenhouse, Yumi Kasai, David Gray, Carol Clugston, Anna Dominiczak and Alex Alderton, Roberto Amato, Sonia Goncalves, Ewan Harrison, David K. Jackson, Ian Johnston, Dominic Kwiatkowski, Cordelia Langford, John Sillitoe on behalf of the Wellcome Sanger Institute COVID-19 Surveillance Team
EPI_ISL_760358, EPI_ISL_760359, EPI_ISL_760360	Lighthouse Lab in Alderley Park	Wellcome Sanger Institute for the COVID-19 Genomics UK (COG-UK) Consortium	Jacquelyn Wynn, Mairead Hyland, The Lighthouse Lab in Alderley Park and Alex Alderton, Roberto Amato, Sonia Goncalves, Ewan Harrison, David K. Jackson, Ian Johnston, Dominic Kwiatkowski, Cordelia Langford, John Sillitoe on behalf of the Wellcome Sanger Institute COVID-19 Surveillance Team
EPI_ISL_760361, EPI_ISL_760362	Lighthouse Lab in Glasgow	Wellcome Sanger Institute for the COVID-19 Genomics UK (COG-UK) Consortium	Harper VanSteenhouse, Yumi Kasai, David Gray, Carol Clugston, Anna Dominiczak and Alex Alderton, Roberto Amato, Sonia Goncalves, Ewan Harrison, David K. Jackson, Ian Johnston, Dominic Kwiatkowski, Cordelia Langford, John Sillitoe on behalf of the Wellcome Sanger Institute COVID-19 Surveillance Team
EPI_ISL_760364	Lighthouse Lab in Alderley Park	Wellcome Sanger Institute for the COVID-19 Genomics UK (COG-UK) Consortium	Jacquelyn Wynn, Mairead Hyland, The Lighthouse Lab in Alderley Park and Alex Alderton, Roberto Amato, Sonia Goncalves, Ewan Harrison, David K. Jackson, Ian Johnston, Dominic Kwiatkowski, Cordelia Langford, John Sillitoe on behalf of the Wellcome Sanger Institute COVID-19 Surveillance Team
EPI_ISL_760365, EPI_ISL_760367, EPI_ISL_760368	Lighthouse Lab in Glasgow	Wellcome Sanger Institute for the COVID-19 Genomics UK (COG-UK) Consortium	Harper VanSteenhouse, Yumi Kasai, David Gray, Carol Clugston, Anna Dominiczak and Alex Alderton, Roberto Amato, Sonia Goncalves, Ewan Harrison, David K. Jackson, Ian Johnston, Dominic Kwiatkowski, Cordelia Langford, John Sillitoe on behalf of the Wellcome Sanger Institute COVID-19 Surveillance Team
EPI_ISL_760370, EPI_ISL_760371, EPI_ISL_760372	Lighthouse Lab in Alderley Park	Wellcome Sanger Institute for the COVID-19 Genomics UK (COG-UK) Consortium	Jacquelyn Wynn, Mairead Hyland, The Lighthouse Lab in Alderley Park and Alex Alderton, Roberto Amato, Sonia Goncalves, Ewan Harrison, David K. Jackson, Ian Johnston, Dominic Kwiatkowski, Cordelia Langford, John Sillitoe on behalf of the Wellcome Sanger Institute COVID-19 Surveillance Team
EPI_ISL_760373, EPI_ISL_760375, EPI_ISL_760376, EPI_ISL_760378, EPI_ISL_760380	Lighthouse Lab in Glasgow	Wellcome Sanger Institute for the COVID-19 Genomics UK (COG-UK) Consortium	Harper VanSteenhouse, Yumi Kasai, David Gray, Carol Clugston, Anna Dominiczak and Alex Alderton, Roberto Amato, Sonia Goncalves, Ewan Harrison, David K. Jackson, Ian Johnston, Dominic Kwiatkowski, Cordelia Langford, John Sillitoe on behalf of the Wellcome Sanger Institute COVID-19 Surveillance Team
EPI_ISL_760381	Lighthouse Lab in Alderley Park	Wellcome Sanger Institute for the COVID-19 Genomics UK (COG-UK) Consortium	Jacquelyn Wynn, Mairead Hyland, The Lighthouse Lab in Alderley Park and Alex Alderton, Roberto Amato, Sonia Goncalves, Ewan Harrison, David K. Jackson, Ian Johnston, Dominic Kwiatkowski, Cordelia Langford, John Sillitoe on behalf of the Wellcome Sanger Institute COVID-19 Surveillance Team
EPI_ISL_760383	Lighthouse Lab in Glasgow	Wellcome Sanger Institute for the COVID-19 Genomics UK (COG-UK) Consortium	Harper VanSteenhouse, Yumi Kasai, David Gray, Carol Clugston, Anna Dominiczak and Alex Alderton, Roberto Amato, Sonia Goncalves, Ewan Harrison, David K. Jackson, Ian Johnston, Dominic Kwiatkowski, Cordelia Langford, John Sillitoe on behalf of the Wellcome Sanger Institute COVID-19 Surveillance Team
EPI_ISL_760386, EPI_ISL_760387, EPI_ISL_760392	Lighthouse Lab in Alderley Park	Wellcome Sanger Institute for the COVID-19 Genomics UK (COG-UK) Consortium	Jacquelyn Wynn, Mairead Hyland, The Lighthouse Lab in Alderley Park and Alex Alderton, Roberto Amato, Sonia Goncalves, Ewan Harrison, David K. Jackson, Ian Johnston, Dominic Kwiatkowski, Cordelia Langford, John Sillitoe on behalf of the Wellcome Sanger Institute COVID-19 Surveillance Team
EPI_ISL_760393, EPI_ISL_760394, EPI_ISL_760395	Lighthouse Lab in Glasgow	Wellcome Sanger Institute for the COVID-19 Genomics UK (COG-UK) Consortium	Harper VanSteenhouse, Yumi Kasai, David Gray, Carol Clugston, Anna Dominiczak and Alex Alderton, Roberto Amato, Sonia Goncalves, Ewan Harrison, David K. Jackson, Ian Johnston, Dominic Kwiatkowski, Cordelia Langford, John Sillitoe on behalf of the Wellcome Sanger Institute COVID-19 Surveillance Team
EPI_ISL_760397	Lighthouse Lab in Alderley Park	Wellcome Sanger Institute for the COVID-19 Genomics UK (COG-UK) Consortium	Jacquelyn Wynn, Mairead Hyland, The Lighthouse Lab in Alderley Park and Alex Alderton, Roberto Amato, Sonia Goncalves, Ewan Harrison, David K. Jackson, Ian Johnston, Dominic Kwiatkowski, Cordelia Langford, John Sillitoe on behalf of the Wellcome Sanger Institute COVID-19 Surveillance Team
EPI_ISL_760401, EPI_ISL_760402, EPI_ISL_760403, EPI_ISL_760404	Lighthouse Lab in Glasgow	Wellcome Sanger Institute for the COVID-19 Genomics UK (COG-UK) Consortium	Harper VanSteenhouse, Yumi Kasai, David Gray, Carol Clugston, Anna Dominiczak and Alex Alderton, Roberto Amato, Sonia Goncalves, Ewan Harrison, David K. Jackson, Ian Johnston, Dominic Kwiatkowski, Cordelia Langford, John Sillitoe on behalf of the Wellcome Sanger Institute COVID-19 Surveillance Team
EPI_ISL_760405	Lighthouse Lab in Alderley Park	Wellcome Sanger Institute for the COVID-19 Genomics UK (COG-UK) Consortium	Jacquelyn Wynn, Mairead Hyland, The Lighthouse Lab in Alderley Park and Alex Alderton, Roberto Amato, Sonia Goncalves, Ewan Harrison, David K. Jackson, Ian Johnston, Dominic Kwiatkowski, Cordelia Langford, John Sillitoe on behalf of the Wellcome Sanger Institute COVID-19 Surveillance Team
EPI_ISL_760407	Lighthouse Lab in Glasgow	Wellcome Sanger Institute for the COVID-19 Genomics UK (COG-UK) Consortium	Harper VanSteenhouse, Yumi Kasai, David Gray, Carol Clugston, Anna Dominiczak and Alex Alderton, Roberto Amato, Sonia Goncalves, Ewan Harrison, David K. Jackson, Ian Johnston, Dominic Kwiatkowski, Cordelia Langford, John Sillitoe on behalf of the Wellcome Sanger Institute COVID-19 Surveillance Team
EPI_ISL_760408	Lighthouse Lab in Alderley Park	Wellcome Sanger Institute for the COVID-19 Genomics UK (COG-UK) Consortium	Jacquelyn Wynn, Mairead Hyland, The Lighthouse Lab in Alderley Park and Alex Alderton, Roberto Amato, Sonia Goncalves, Ewan Harrison, David K. Jackson, Ian Johnston, Dominic Kwiatkowski, Cordelia Langford, John Sillitoe on behalf of the Wellcome Sanger Institute COVID-19 Surveillance Team
EPI_ISL_760409, EPI_ISL_760410, EPI_ISL_760411, EPI_ISL_760413	Lighthouse Lab in Glasgow	Wellcome Sanger Institute for the COVID-19 Genomics UK (COG-UK) Consortium	Harper VanSteenhouse, Yumi Kasai, David Gray, Carol Clugston, Anna Dominiczak and Alex Alderton, Roberto Amato, Sonia Goncalves, Ewan Harrison, David K. Jackson, Ian Johnston, Dominic Kwiatkowski, Cordelia Langford, John Sillitoe on behalf of the Wellcome Sanger Institute COVID-19 Surveillance Team
EPI_ISL_760415	Lighthouse Lab in Alderley Park	Wellcome Sanger Institute for the COVID-19 Genomics UK (COG-UK) Consortium	Jacquelyn Wynn, Mairead Hyland, The Lighthouse Lab in Alderley Park and Alex Alderton, Roberto Amato, Sonia Goncalves, Ewan Harrison, David K. Jackson, Ian Johnston, Dominic Kwiatkowski, Cordelia Langford, John Sillitoe on behalf of the Wellcome Sanger Institute COVID-19 Surveillance Team
EPI_ISL_760417	Lighthouse Lab in Glasgow	Wellcome Sanger Institute for the COVID-19 Genomics UK (COG-UK) Consortium	Harper VanSteenhouse, Yumi Kasai, David Gray, Carol Clugston, Anna Dominiczak and Alex Alderton, Roberto Amato, Sonia Goncalves, Ewan Harrison, David K. Jackson, Ian Johnston, Dominic Kwiatkowski, Cordelia Langford, John Sillitoe on behalf of the Wellcome Sanger Institute COVID-19 Surveillance Team
EPI_ISL_760419, EPI_ISL_760421	Lighthouse Lab in Alderley Park	Wellcome Sanger Institute for the COVID-19 Genomics UK (COG-UK) Consortium	Jacquelyn Wynn, Mairead Hyland, The Lighthouse Lab in Alderley Park and Alex Alderton, Roberto Amato, Sonia Goncalves, Ewan Harrison, David K. Jackson, Ian Johnston, Dominic Kwiatkowski, Cordelia Langford, John Sillitoe on behalf of the Wellcome Sanger Institute COVID-19 Surveillance Team
EPI_ISL_760422, EPI_ISL_760423, EPI_ISL_760426, EPI_ISL_760427, EPI_ISL_760428, EPI_ISL_760429	Lighthouse Lab in Glasgow	Wellcome Sanger Institute for the COVID-19 Genomics UK (COG-UK) Consortium	Harper VanSteenhouse, Yumi Kasai, David Gray, Carol Clugston, Anna Dominiczak and Alex Alderton, Roberto Amato, Sonia Goncalves, Ewan Harrison, David K. Jackson, Ian Johnston, Dominic Kwiatkowski, Cordelia Langford, John Sillitoe on behalf of the Wellcome Sanger Institute COVID-19 Surveillance Team
EPI_ISL_760430	Lighthouse Lab in Alderley Park	Wellcome Sanger Institute for the COVID-19 Genomics UK (COG-UK) Consortium	Jacquelyn Wynn, Mairead Hyland, The Lighthouse Lab in Alderley Park and Alex Alderton, Roberto Amato, Sonia Goncalves, Ewan Harrison, David K. Jackson, Ian Johnston, Dominic Kwiatkowski, Cordelia Langford, John Sillitoe on behalf of the Wellcome Sanger Institute COVID-19 Surveillance Team
EPI_ISL_760433, EPI_ISL_760435	Lighthouse Lab in Glasgow	Wellcome Sanger Institute for the COVID-19 Genomics UK (COG-UK) Consortium	Harper VanSteenhouse, Yumi Kasai, David Gray, Carol Clugston, Anna Dominiczak and Alex Alderton, Roberto Amato, Sonia Goncalves, Ewan Harrison, David K. Jackson, Ian Johnston, Dominic Kwiatkowski, Cordelia Langford, John Sillitoe on behalf of the Wellcome Sanger Institute COVID-19 Surveillance Team
EPI_ISL_760436	Lighthouse Lab in Alderley Park	Wellcome Sanger Institute for the COVID-19 Genomics UK (COG-UK) Consortium	Jacquelyn Wynn, Mairead Hyland, The Lighthouse Lab in Alderley Park and Alex Alderton, Roberto Amato, Sonia Goncalves, Ewan Harrison, David K. Jackson, Ian Johnston, Dominic Kwiatkowski, Cordelia Langford, John Sillitoe on behalf of the Wellcome Sanger Institute COVID-19 Surveillance Team
EPI_ISL_760437, EPI_ISL_760438, EPI_ISL_760439, EPI_ISL_760440	Lighthouse Lab in Glasgow	Wellcome Sanger Institute for the COVID-19 Genomics UK (COG-UK) Consortium	Harper VanSteenhouse, Yumi Kasai, David Gray, Carol Clugston, Anna Dominiczak and Alex Alderton, Roberto Amato, Sonia Goncalves, Ewan Harrison, David K. Jackson, Ian Johnston, Dominic Kwiatkowski, Cordelia Langford, John Sillitoe on behalf of the Wellcome Sanger Institute COVID-19 Surveillance Team
EPI_ISL_760441, EPI_ISL_760442	Lighthouse Lab in Alderley Park	Wellcome Sanger Institute for the COVID-19 Genomics UK (COG-UK) Consortium	Jacquelyn Wynn, Mairead Hyland, The Lighthouse Lab in Alderley Park and Alex Alderton, Roberto Amato, Sonia Goncalves, Ewan Harrison, David K. Jackson, Ian Johnston, Dominic Kwiatkowski, Cordelia Langford, John Sillitoe on behalf of the Wellcome Sanger Institute COVID-19 Surveillance Team
EPI_ISL_760443, EPI_ISL_760444	Lighthouse Lab in Glasgow	Wellcome Sanger Institute for the COVID-19 Genomics UK	Harper VanSteenhouse, Yumi Kasai, David Gray, Carol Clugston, Anna Dominiczak and Alex Alderton, Roberto Amato, Sonia Goncalves, Ewan Harrison,

		(COG-UK) Consortium	David K. Jackson, Ian Johnston, Dominic Kwiatkowski, Cordelia Langford, John Sillitoe on behalf of the Wellcome Sanger Institute COVID-19 Surveillance Team
EPI_ISL_760445	Lighthouse Lab in Alderley Park	Wellcome Sanger Institute for the COVID-19 Genomics UK (COG-UK) Consortium	Jacquelyn Wynn, Mairead Hyland, The Lighthouse Lab in Alderley Park and Alex Alderton, Roberto Amato, Sonia Goncalves, Ewan Harrison, David K. Jackson, Ian Johnston, Dominic Kwiatkowski, Cordelia Langford, John Sillitoe on behalf of the Wellcome Sanger Institute COVID-19 Surveillance Team
EPI_ISL_760447	Lighthouse Lab in Glasgow	Wellcome Sanger Institute for the COVID-19 Genomics UK (COG-UK) Consortium	Harper VanSteenhouse, Yumi Kasai, David Gray, Carol Clugston, Anna Dominiczak and Alex Alderton, Roberto Amato, Sonia Goncalves, Ewan Harrison, David K. Jackson, Ian Johnston, Dominic Kwiatkowski, Cordelia Langford, John Sillitoe on behalf of the Wellcome Sanger Institute COVID-19 Surveillance Team
EPI_ISL_760448	Lighthouse Lab in Alderley Park	Wellcome Sanger Institute for the COVID-19 Genomics UK (COG-UK) Consortium	Jacquelyn Wynn, Mairead Hyland, The Lighthouse Lab in Alderley Park and Alex Alderton, Roberto Amato, Sonia Goncalves, Ewan Harrison, David K. Jackson, Ian Johnston, Dominic Kwiatkowski, Cordelia Langford, John Sillitoe on behalf of the Wellcome Sanger Institute COVID-19 Surveillance Team
EPI_ISL_760449	Lighthouse Lab in Glasgow	Wellcome Sanger Institute for the COVID-19 Genomics UK (COG-UK) Consortium	Harper VanSteenhouse, Yumi Kasai, David Gray, Carol Clugston, Anna Dominiczak and Alex Alderton, Roberto Amato, Sonia Goncalves, Ewan Harrison, David K. Jackson, Ian Johnston, Dominic Kwiatkowski, Cordelia Langford, John Sillitoe on behalf of the Wellcome Sanger Institute COVID-19 Surveillance Team
EPI_ISL_760450	Lighthouse Lab in Alderley Park	Wellcome Sanger Institute for the COVID-19 Genomics UK (COG-UK) Consortium	Jacquelyn Wynn, Mairead Hyland, The Lighthouse Lab in Alderley Park and Alex Alderton, Roberto Amato, Sonia Goncalves, Ewan Harrison, David K. Jackson, Ian Johnston, Dominic Kwiatkowski, Cordelia Langford, John Sillitoe on behalf of the Wellcome Sanger Institute COVID-19 Surveillance Team
EPI_ISL_760452, EPI_ISL_760453, EPI_ISL_760454, EPI_ISL_760455, EPI_ISL_760456, EPI_ISL_760458, EPI_ISL_760459	Lighthouse Lab in Glasgow	Wellcome Sanger Institute for the COVID-19 Genomics UK (COG-UK) Consortium	Harper VanSteenhouse, Yumi Kasai, David Gray, Carol Clugston, Anna Dominiczak and Alex Alderton, Roberto Amato, Sonia Goncalves, Ewan Harrison, David K. Jackson, Ian Johnston, Dominic Kwiatkowski, Cordelia Langford, John Sillitoe on behalf of the Wellcome Sanger Institute COVID-19 Surveillance Team
EPI_ISL_760460	Lighthouse Lab in Alderley Park	Wellcome Sanger Institute for the COVID-19 Genomics UK (COG-UK) Consortium	Jacquelyn Wynn, Mairead Hyland, The Lighthouse Lab in Alderley Park and Alex Alderton, Roberto Amato, Sonia Goncalves, Ewan Harrison, David K. Jackson, Ian Johnston, Dominic Kwiatkowski, Cordelia Langford, John Sillitoe on behalf of the Wellcome Sanger Institute COVID-19 Surveillance Team
EPI_ISL_760462, EPI_ISL_760463, EPI_ISL_760464, EPI_ISL_760465, EPI_ISL_760466, EPI_ISL_760467, EPI_ISL_760468, EPI_ISL_760469, EPI_ISL_760470	Lighthouse Lab in Glasgow	Wellcome Sanger Institute for the COVID-19 Genomics UK (COG-UK) Consortium	Harper VanSteenhouse, Yumi Kasai, David Gray, Carol Clugston, Anna Dominiczak and Alex Alderton, Roberto Amato, Sonia Goncalves, Ewan Harrison, David K. Jackson, Ian Johnston, Dominic Kwiatkowski, Cordelia Langford, John Sillitoe on behalf of the Wellcome Sanger Institute COVID-19 Surveillance Team
EPI_ISL_760471	Lighthouse Lab in Alderley Park	Wellcome Sanger Institute for the COVID-19 Genomics UK (COG-UK) Consortium	Jacquelyn Wynn, Mairead Hyland, The Lighthouse Lab in Alderley Park and Alex Alderton, Roberto Amato, Sonia Goncalves, Ewan Harrison, David K. Jackson, Ian Johnston, Dominic Kwiatkowski, Cordelia Langford, John Sillitoe on behalf of the Wellcome Sanger Institute COVID-19 Surveillance Team
EPI_ISL_760472, EPI_ISL_760473	Lighthouse Lab in Glasgow	Wellcome Sanger Institute for the COVID-19 Genomics UK (COG-UK) Consortium	Harper VanSteenhouse, Yumi Kasai, David Gray, Carol Clugston, Anna Dominiczak and Alex Alderton, Roberto Amato, Sonia Goncalves, Ewan Harrison, David K. Jackson, Ian Johnston, Dominic Kwiatkowski, Cordelia Langford, John Sillitoe on behalf of the Wellcome Sanger Institute COVID-19 Surveillance Team
EPI_ISL_760474, EPI_ISL_760475	Lighthouse Lab in Alderley Park	Wellcome Sanger Institute for the COVID-19 Genomics UK (COG-UK) Consortium	Jacquelyn Wynn, Mairead Hyland, The Lighthouse Lab in Alderley Park and Alex Alderton, Roberto Amato, Sonia Goncalves, Ewan Harrison, David K. Jackson, Ian Johnston, Dominic Kwiatkowski, Cordelia Langford, John Sillitoe on behalf of the Wellcome Sanger Institute COVID-19 Surveillance Team
EPI_ISL_760476, EPI_ISL_760477, EPI_ISL_760480, EPI_ISL_760481, EPI_ISL_760482, EPI_ISL_760483	Lighthouse Lab in Glasgow	Wellcome Sanger Institute for the COVID-19 Genomics UK (COG-UK) Consortium	Harper VanSteenhouse, Yumi Kasai, David Gray, Carol Clugston, Anna Dominiczak and Alex Alderton, Roberto Amato, Sonia Goncalves, Ewan Harrison, David K. Jackson, Ian Johnston, Dominic Kwiatkowski, Cordelia Langford, John Sillitoe on behalf of the Wellcome Sanger Institute COVID-19 Surveillance Team
EPI_ISL_760485, EPI_ISL_760486	Lighthouse Lab in Alderley Park	Wellcome Sanger Institute for the COVID-19 Genomics UK (COG-UK) Consortium	Jacquelyn Wynn, Mairead Hyland, The Lighthouse Lab in Alderley Park and Alex Alderton, Roberto Amato, Sonia Goncalves, Ewan Harrison, David K. Jackson, Ian Johnston, Dominic Kwiatkowski, Cordelia Langford, John Sillitoe on behalf of the Wellcome Sanger Institute COVID-19 Surveillance Team
EPI_ISL_760487, EPI_ISL_760488	Lighthouse Lab in Glasgow	Wellcome Sanger Institute for the COVID-19 Genomics UK (COG-UK) Consortium	Harper VanSteenhouse, Yumi Kasai, David Gray, Carol Clugston, Anna Dominiczak and Alex Alderton, Roberto Amato, Sonia Goncalves, Ewan Harrison, David K. Jackson, Ian Johnston, Dominic Kwiatkowski, Cordelia Langford, John Sillitoe on behalf of the Wellcome Sanger Institute COVID-19 Surveillance Team
EPI_ISL_760491	Lighthouse Lab in Alderley Park	Wellcome Sanger Institute for the COVID-19 Genomics UK (COG-UK) Consortium	Jacquelyn Wynn, Mairead Hyland, The Lighthouse Lab in Alderley Park and Alex Alderton, Roberto Amato, Sonia Goncalves, Ewan Harrison, David K. Jackson, Ian Johnston, Dominic Kwiatkowski, Cordelia Langford, John Sillitoe on behalf of the Wellcome Sanger Institute COVID-19 Surveillance Team
EPI_ISL_760494, EPI_ISL_760496	Lighthouse Lab in Glasgow	Wellcome Sanger Institute for the COVID-19 Genomics UK (COG-UK) Consortium	Harper VanSteenhouse, Yumi Kasai, David Gray, Carol Clugston, Anna Dominiczak and Alex Alderton, Roberto Amato, Sonia Goncalves, Ewan Harrison, David K. Jackson, Ian Johnston, Dominic Kwiatkowski, Cordelia Langford, John Sillitoe on behalf of the Wellcome Sanger Institute COVID-19 Surveillance Team
EPI_ISL_760498	Lighthouse Lab in Alderley Park	Wellcome Sanger Institute for the COVID-19 Genomics UK (COG-UK) Consortium	Jacquelyn Wynn, Mairead Hyland, The Lighthouse Lab in Alderley Park and Alex Alderton, Roberto Amato, Sonia Goncalves, Ewan Harrison, David K. Jackson, Ian Johnston, Dominic Kwiatkowski, Cordelia Langford, John Sillitoe on behalf of the Wellcome Sanger Institute COVID-19 Surveillance Team
EPI_ISL_760499	Lighthouse Lab in Glasgow	Wellcome Sanger Institute for the COVID-19 Genomics UK (COG-UK) Consortium	Harper VanSteenhouse, Yumi Kasai, David Gray, Carol Clugston, Anna Dominiczak and Alex Alderton, Roberto Amato, Sonia Goncalves, Ewan Harrison, David K. Jackson, Ian Johnston, Dominic Kwiatkowski, Cordelia Langford, John Sillitoe on behalf of the Wellcome Sanger Institute COVID-19 Surveillance Team
EPI_ISL_760500	Lighthouse Lab in Alderley Park	Wellcome Sanger Institute for the COVID-19 Genomics UK (COG-UK) Consortium	Jacquelyn Wynn, Mairead Hyland, The Lighthouse Lab in Alderley Park and Alex Alderton, Roberto Amato, Sonia Goncalves, Ewan Harrison, David K. Jackson, Ian Johnston, Dominic Kwiatkowski, Cordelia Langford, John Sillitoe on behalf of the Wellcome Sanger Institute COVID-19 Surveillance Team
EPI_ISL_760502, EPI_ISL_760506, EPI_ISL_760507, EPI_ISL_760511, EPI_ISL_760513, EPI_ISL_760515	Lighthouse Lab in Glasgow	Wellcome Sanger Institute for the COVID-19 Genomics UK (COG-UK) Consortium	Harper VanSteenhouse, Yumi Kasai, David Gray, Carol Clugston, Anna Dominiczak and Alex Alderton, Roberto Amato, Sonia Goncalves, Ewan Harrison, David K. Jackson, Ian Johnston, Dominic Kwiatkowski, Cordelia Langford, John Sillitoe on behalf of the Wellcome Sanger Institute COVID-19 Surveillance Team
EPI_ISL_760516	Lighthouse Lab in Alderley Park	Wellcome Sanger Institute for the COVID-19 Genomics UK (COG-UK) Consortium	Jacquelyn Wynn, Mairead Hyland, The Lighthouse Lab in Alderley Park and Alex Alderton, Roberto Amato, Sonia Goncalves, Ewan Harrison, David K. Jackson, Ian Johnston, Dominic Kwiatkowski, Cordelia Langford, John Sillitoe on behalf of the Wellcome Sanger Institute COVID-19 Surveillance Team
EPI_ISL_760517, EPI_ISL_760519, EPI_ISL_760520	Lighthouse Lab in Glasgow	Wellcome Sanger Institute for the COVID-19 Genomics UK (COG-UK) Consortium	Harper VanSteenhouse, Yumi Kasai, David Gray, Carol Clugston, Anna Dominiczak and Alex Alderton, Roberto Amato, Sonia Goncalves, Ewan Harrison, David K. Jackson, Ian Johnston, Dominic Kwiatkowski, Cordelia Langford, John Sillitoe on behalf of the Wellcome Sanger Institute COVID-19 Surveillance Team
EPI_ISL_760521	Lighthouse Lab in Alderley Park	Wellcome Sanger Institute for the COVID-19 Genomics UK (COG-UK) Consortium	Jacquelyn Wynn, Mairead Hyland, The Lighthouse Lab in Alderley Park and Alex Alderton, Roberto Amato, Sonia Goncalves, Ewan Harrison, David K. Jackson, Ian Johnston, Dominic Kwiatkowski, Cordelia Langford, John Sillitoe on behalf of the Wellcome Sanger Institute COVID-19 Surveillance Team
EPI_ISL_760522, EPI_ISL_760523	Lighthouse Lab in Glasgow	Wellcome Sanger Institute for the COVID-19 Genomics UK (COG-UK) Consortium	Harper VanSteenhouse, Yumi Kasai, David Gray, Carol Clugston, Anna Dominiczak and Alex Alderton, Roberto Amato, Sonia Goncalves, Ewan Harrison, David K. Jackson, Ian Johnston, Dominic Kwiatkowski, Cordelia Langford, John Sillitoe on behalf of the Wellcome Sanger Institute COVID-19 Surveillance Team
EPI_ISL_760524	Lighthouse Lab in Alderley Park	Wellcome Sanger Institute for the COVID-19 Genomics UK (COG-UK) Consortium	Jacquelyn Wynn, Mairead Hyland, The Lighthouse Lab in Alderley Park and Alex Alderton, Roberto Amato, Sonia Goncalves, Ewan Harrison, David K. Jackson, Ian Johnston, Dominic Kwiatkowski, Cordelia Langford, John Sillitoe on behalf of the Wellcome Sanger Institute COVID-19 Surveillance Team
EPI_ISL_760525, EPI_ISL_760526, EPI_ISL_760527, EPI_ISL_760529	Lighthouse Lab in Glasgow	Wellcome Sanger Institute for the COVID-19 Genomics UK (COG-UK) Consortium	Harper VanSteenhouse, Yumi Kasai, David Gray, Carol Clugston, Anna Dominiczak and Alex Alderton, Roberto Amato, Sonia Goncalves, Ewan Harrison, David K. Jackson, Ian Johnston, Dominic Kwiatkowski, Cordelia Langford, John Sillitoe on behalf of the Wellcome Sanger Institute COVID-19 Surveillance Team
EPI_ISL_760531	Lighthouse Lab in Alderley Park	Wellcome Sanger Institute for the COVID-19 Genomics UK (COG-UK) Consortium	Jacquelyn Wynn, Mairead Hyland, The Lighthouse Lab in Alderley Park and Alex Alderton, Roberto Amato, Sonia Goncalves, Ewan Harrison, David K. Jackson, Ian Johnston, Dominic Kwiatkowski, Cordelia Langford, John Sillitoe on behalf of the Wellcome Sanger Institute COVID-19 Surveillance Team
EPI_ISL_760533	Lighthouse Lab in Glasgow	Wellcome Sanger Institute for the COVID-19 Genomics UK	Harper VanSteenhouse, Yumi Kasai, David Gray, Carol Clugston, Anna Dominiczak and Alex Alderton, Roberto Amato, Sonia Goncalves, Ewan Harrison,

		(COG-UK) Consortium	David K. Jackson, Ian Johnston, Dominic Kwiatkowski, Cordelia Langford, John Sillitoe on behalf of the Wellcome Sanger Institute COVID-19 Surveillance Team
EPI_ISL_760534	Lighthouse Lab in Alderley Park	Wellcome Sanger Institute for the COVID-19 Genomics UK (COG-UK) Consortium	Jacquelyn Wynn, Mairead Hyland, The Lighthouse Lab in Alderley Park and Alex Alderton, Roberto Amato, Sonia Goncalves, Ewan Harrison, David K. Jackson, Ian Johnston, Dominic Kwiatkowski, Cordelia Langford, John Sillitoe on behalf of the Wellcome Sanger Institute COVID-19 Surveillance Team
EPI_ISL_760536, EPI_ISL_760537	Lighthouse Lab in Glasgow	Wellcome Sanger Institute for the COVID-19 Genomics UK (COG-UK) Consortium	Harper VanSteenhouse, Yumi Kasai, David Gray, Carol Clugston, Anna Dominiczak and Alex Alderton, Roberto Amato, Sonia Goncalves, Ewan Harrison, David K. Jackson, Ian Johnston, Dominic Kwiatkowski, Cordelia Langford, John Sillitoe on behalf of the Wellcome Sanger Institute COVID-19 Surveillance Team
EPI_ISL_760538	Lighthouse Lab in Alderley Park	Wellcome Sanger Institute for the COVID-19 Genomics UK (COG-UK) Consortium	Jacquelyn Wynn, Mairead Hyland, The Lighthouse Lab in Alderley Park and Alex Alderton, Roberto Amato, Sonia Goncalves, Ewan Harrison, David K. Jackson, Ian Johnston, Dominic Kwiatkowski, Cordelia Langford, John Sillitoe on behalf of the Wellcome Sanger Institute COVID-19 Surveillance Team
EPI_ISL_760539, EPI_ISL_760540, EPI_ISL_760541, EPI_ISL_760542	Lighthouse Lab in Glasgow	Wellcome Sanger Institute for the COVID-19 Genomics UK (COG-UK) Consortium	Harper VanSteenhouse, Yumi Kasai, David Gray, Carol Clugston, Anna Dominiczak and Alex Alderton, Roberto Amato, Sonia Goncalves, Ewan Harrison, David K. Jackson, Ian Johnston, Dominic Kwiatkowski, Cordelia Langford, John Sillitoe on behalf of the Wellcome Sanger Institute COVID-19 Surveillance Team
EPI_ISL_760543, EPI_ISL_760544, EPI_ISL_760546	Lighthouse Lab in Alderley Park	Wellcome Sanger Institute for the COVID-19 Genomics UK (COG-UK) Consortium	Jacquelyn Wynn, Mairead Hyland, The Lighthouse Lab in Alderley Park and Alex Alderton, Roberto Amato, Sonia Goncalves, Ewan Harrison, David K. Jackson, Ian Johnston, Dominic Kwiatkowski, Cordelia Langford, John Sillitoe on behalf of the Wellcome Sanger Institute COVID-19 Surveillance Team
EPI_ISL_760547	Lighthouse Lab in Glasgow	Wellcome Sanger Institute for the COVID-19 Genomics UK (COG-UK) Consortium	Harper VanSteenhouse, Yumi Kasai, David Gray, Carol Clugston, Anna Dominiczak and Alex Alderton, Roberto Amato, Sonia Goncalves, Ewan Harrison, David K. Jackson, Ian Johnston, Dominic Kwiatkowski, Cordelia Langford, John Sillitoe on behalf of the Wellcome Sanger Institute COVID-19 Surveillance Team
EPI_ISL_760549, EPI_ISL_760551, EPI_ISL_760552	Lighthouse Lab in Alderley Park	Wellcome Sanger Institute for the COVID-19 Genomics UK (COG-UK) Consortium	Jacquelyn Wynn, Mairead Hyland, The Lighthouse Lab in Alderley Park and Alex Alderton, Roberto Amato, Sonia Goncalves, Ewan Harrison, David K. Jackson, Ian Johnston, Dominic Kwiatkowski, Cordelia Langford, John Sillitoe on behalf of the Wellcome Sanger Institute COVID-19 Surveillance Team
EPI_ISL_760553	Lighthouse Lab in Glasgow	Wellcome Sanger Institute for the COVID-19 Genomics UK (COG-UK) Consortium	Harper VanSteenhouse, Yumi Kasai, David Gray, Carol Clugston, Anna Dominiczak and Alex Alderton, Roberto Amato, Sonia Goncalves, Ewan Harrison, David K. Jackson, Ian Johnston, Dominic Kwiatkowski, Cordelia Langford, John Sillitoe on behalf of the Wellcome Sanger Institute COVID-19 Surveillance Team
EPI_ISL_760555, EPI_ISL_760559, EPI_ISL_760561, EPI_ISL_760562	Lighthouse Lab in Alderley Park	Wellcome Sanger Institute for the COVID-19 Genomics UK (COG-UK) Consortium	Jacquelyn Wynn, Mairead Hyland, The Lighthouse Lab in Alderley Park and Alex Alderton, Roberto Amato, Sonia Goncalves, Ewan Harrison, David K. Jackson, Ian Johnston, Dominic Kwiatkowski, Cordelia Langford, John Sillitoe on behalf of the Wellcome Sanger Institute COVID-19 Surveillance Team
EPI_ISL_760563	Lighthouse Lab in Glasgow	Wellcome Sanger Institute for the COVID-19 Genomics UK (COG-UK) Consortium	Harper VanSteenhouse, Yumi Kasai, David Gray, Carol Clugston, Anna Dominiczak and Alex Alderton, Roberto Amato, Sonia Goncalves, Ewan Harrison, David K. Jackson, Ian Johnston, Dominic Kwiatkowski, Cordelia Langford, John Sillitoe on behalf of the Wellcome Sanger Institute COVID-19 Surveillance Team
EPI_ISL_760564, EPI_ISL_760565	Lighthouse Lab in Alderley Park	Wellcome Sanger Institute for the COVID-19 Genomics UK (COG-UK) Consortium	Jacquelyn Wynn, Mairead Hyland, The Lighthouse Lab in Alderley Park and Alex Alderton, Roberto Amato, Sonia Goncalves, Ewan Harrison, David K. Jackson, Ian Johnston, Dominic Kwiatkowski, Cordelia Langford, John Sillitoe on behalf of the Wellcome Sanger Institute COVID-19 Surveillance Team
EPI_ISL_760566	Lighthouse Lab in Glasgow	Wellcome Sanger Institute for the COVID-19 Genomics UK (COG-UK) Consortium	Harper VanSteenhouse, Yumi Kasai, David Gray, Carol Clugston, Anna Dominiczak and Alex Alderton, Roberto Amato, Sonia Goncalves, Ewan Harrison, David K. Jackson, Ian Johnston, Dominic Kwiatkowski, Cordelia Langford, John Sillitoe on behalf of the Wellcome Sanger Institute COVID-19 Surveillance Team
EPI_ISL_760567, EPI_ISL_760572, EPI_ISL_760574	Lighthouse Lab in Alderley Park	Wellcome Sanger Institute for the COVID-19 Genomics UK (COG-UK) Consortium	Jacquelyn Wynn, Mairead Hyland, The Lighthouse Lab in Alderley Park and Alex Alderton, Roberto Amato, Sonia Goncalves, Ewan Harrison, David K. Jackson, Ian Johnston, Dominic Kwiatkowski, Cordelia Langford, John Sillitoe on behalf of the Wellcome Sanger Institute COVID-19 Surveillance Team
EPI_ISL_760575, EPI_ISL_760576, EPI_ISL_760577, EPI_ISL_760578, EPI_ISL_760579, EPI_ISL_760580, EPI_ISL_760581	Lighthouse Lab in Glasgow	Wellcome Sanger Institute for the COVID-19 Genomics UK (COG-UK) Consortium	Harper VanSteenhouse, Yumi Kasai, David Gray, Carol Clugston, Anna Dominiczak and Alex Alderton, Roberto Amato, Sonia Goncalves, Ewan Harrison, David K. Jackson, Ian Johnston, Dominic Kwiatkowski, Cordelia Langford, John Sillitoe on behalf of the Wellcome Sanger Institute COVID-19 Surveillance Team
EPI_ISL_760582	Lighthouse Lab in Alderley Park	Wellcome Sanger Institute for the COVID-19 Genomics UK (COG-UK) Consortium	Jacquelyn Wynn, Mairead Hyland, The Lighthouse Lab in Alderley Park and Alex Alderton, Roberto Amato, Sonia Goncalves, Ewan Harrison, David K. Jackson, Ian Johnston, Dominic Kwiatkowski, Cordelia Langford, John Sillitoe on behalf of the Wellcome Sanger Institute COVID-19 Surveillance Team
EPI_ISL_760583, EPI_ISL_760584, EPI_ISL_760585	Lighthouse Lab in Glasgow	Wellcome Sanger Institute for the COVID-19 Genomics UK (COG-UK) Consortium	Harper VanSteenhouse, Yumi Kasai, David Gray, Carol Clugston, Anna Dominiczak and Alex Alderton, Roberto Amato, Sonia Goncalves, Ewan Harrison, David K. Jackson, Ian Johnston, Dominic Kwiatkowski, Cordelia Langford, John Sillitoe on behalf of the Wellcome Sanger Institute COVID-19 Surveillance Team
EPI_ISL_760586, EPI_ISL_760587, EPI_ISL_760588, EPI_ISL_760589	Lighthouse Lab in Alderley Park	Wellcome Sanger Institute for the COVID-19 Genomics UK (COG-UK) Consortium	Jacquelyn Wynn, Mairead Hyland, The Lighthouse Lab in Alderley Park and Alex Alderton, Roberto Amato, Sonia Goncalves, Ewan Harrison, David K. Jackson, Ian Johnston, Dominic Kwiatkowski, Cordelia Langford, John Sillitoe on behalf of the Wellcome Sanger Institute COVID-19 Surveillance Team
EPI_ISL_760590	Lighthouse Lab in Glasgow	Wellcome Sanger Institute for the COVID-19 Genomics UK (COG-UK) Consortium	Harper VanSteenhouse, Yumi Kasai, David Gray, Carol Clugston, Anna Dominiczak and Alex Alderton, Roberto Amato, Sonia Goncalves, Ewan Harrison, David K. Jackson, Ian Johnston, Dominic Kwiatkowski, Cordelia Langford, John Sillitoe on behalf of the Wellcome Sanger Institute COVID-19 Surveillance Team
EPI_ISL_760591	Lighthouse Lab in Alderley Park	Wellcome Sanger Institute for the COVID-19 Genomics UK (COG-UK) Consortium	Jacquelyn Wynn, Mairead Hyland, The Lighthouse Lab in Alderley Park and Alex Alderton, Roberto Amato, Sonia Goncalves, Ewan Harrison, David K. Jackson, Ian Johnston, Dominic Kwiatkowski, Cordelia Langford, John Sillitoe on behalf of the Wellcome Sanger Institute COVID-19 Surveillance Team
EPI_ISL_760592	Lighthouse Lab in Glasgow	Wellcome Sanger Institute for the COVID-19 Genomics UK (COG-UK) Consortium	Harper VanSteenhouse, Yumi Kasai, David Gray, Carol Clugston, Anna Dominiczak and Alex Alderton, Roberto Amato, Sonia Goncalves, Ewan Harrison, David K. Jackson, Ian Johnston, Dominic Kwiatkowski, Cordelia Langford, John Sillitoe on behalf of the Wellcome Sanger Institute COVID-19 Surveillance Team
EPI_ISL_760593, EPI_ISL_760596, EPI_ISL_760597	Lighthouse Lab in Alderley Park	Wellcome Sanger Institute for the COVID-19 Genomics UK (COG-UK) Consortium	Jacquelyn Wynn, Mairead Hyland, The Lighthouse Lab in Alderley Park and Alex Alderton, Roberto Amato, Sonia Goncalves, Ewan Harrison, David K. Jackson, Ian Johnston, Dominic Kwiatkowski, Cordelia Langford, John Sillitoe on behalf of the Wellcome Sanger Institute COVID-19 Surveillance Team
EPI_ISL_760598	Lighthouse Lab in Glasgow	Wellcome Sanger Institute for the COVID-19 Genomics UK (COG-UK) Consortium	Harper VanSteenhouse, Yumi Kasai, David Gray, Carol Clugston, Anna Dominiczak and Alex Alderton, Roberto Amato, Sonia Goncalves, Ewan Harrison, David K. Jackson, Ian Johnston, Dominic Kwiatkowski, Cordelia Langford, John Sillitoe on behalf of the Wellcome Sanger Institute COVID-19 Surveillance Team
EPI_ISL_760599, EPI_ISL_760600, EPI_ISL_760601	Lighthouse Lab in Alderley Park	Wellcome Sanger Institute for the COVID-19 Genomics UK (COG-UK) Consortium	Jacquelyn Wynn, Mairead Hyland, The Lighthouse Lab in Alderley Park and Alex Alderton, Roberto Amato, Sonia Goncalves, Ewan Harrison, David K. Jackson, Ian Johnston, Dominic Kwiatkowski, Cordelia Langford, John Sillitoe on behalf of the Wellcome Sanger Institute COVID-19 Surveillance Team
EPI_ISL_760602, EPI_ISL_760604	Lighthouse Lab in Glasgow	Wellcome Sanger Institute for the COVID-19 Genomics UK (COG-UK) Consortium	Harper VanSteenhouse, Yumi Kasai, David Gray, Carol Clugston, Anna Dominiczak and Alex Alderton, Roberto Amato, Sonia Goncalves, Ewan Harrison, David K. Jackson, Ian Johnston, Dominic Kwiatkowski, Cordelia Langford, John Sillitoe on behalf of the Wellcome Sanger Institute COVID-19 Surveillance Team
EPI_ISL_760608, EPI_ISL_760609	Lighthouse Lab in Alderley Park	Wellcome Sanger Institute for the COVID-19 Genomics UK (COG-UK) Consortium	Jacquelyn Wynn, Mairead Hyland, The Lighthouse Lab in Alderley Park and Alex Alderton, Roberto Amato, Sonia Goncalves, Ewan Harrison, David K. Jackson, Ian Johnston, Dominic Kwiatkowski, Cordelia Langford, John Sillitoe on behalf of the Wellcome Sanger Institute COVID-19 Surveillance Team
EPI_ISL_760610, EPI_ISL_760611	Lighthouse Lab in Glasgow	Wellcome Sanger Institute for the COVID-19 Genomics UK (COG-UK) Consortium	Harper VanSteenhouse, Yumi Kasai, David Gray, Carol Clugston, Anna Dominiczak and Alex Alderton, Roberto Amato, Sonia Goncalves, Ewan Harrison, David K. Jackson, Ian Johnston, Dominic Kwiatkowski, Cordelia Langford, John Sillitoe on behalf of the Wellcome Sanger Institute COVID-19 Surveillance Team
EPI_ISL_760612, EPI_ISL_760613	Lighthouse Lab in Alderley Park	Wellcome Sanger Institute for the COVID-19 Genomics UK (COG-UK) Consortium	Jacquelyn Wynn, Mairead Hyland, The Lighthouse Lab in Alderley Park and Alex Alderton, Roberto Amato, Sonia Goncalves, Ewan Harrison, David K. Jackson, Ian Johnston, Dominic Kwiatkowski, Cordelia Langford, John Sillitoe on behalf of the Wellcome Sanger Institute COVID-19 Surveillance Team
EPI_ISL_760614	Lighthouse Lab in Glasgow	Wellcome Sanger Institute for the COVID-19 Genomics UK (COG-UK) Consortium	Harper VanSteenhouse, Yumi Kasai, David Gray, Carol Clugston, Anna Dominiczak and Alex Alderton, Roberto Amato, Sonia Goncalves, Ewan Harrison, David K. Jackson, Ian Johnston, Dominic Kwiatkowski, Cordelia Langford, John Sillitoe on behalf of the Wellcome Sanger Institute COVID-19 Surveillance Team

EPI_ISL_760615	Lighthouse Lab in Alderley Park	Wellcome Sanger Institute for the COVID-19 Genomics UK (COG-UK) Consortium	Jacquelyn Wynn, Mairead Hyland, The Lighthouse Lab in Alderley Park and Alex Alderton, Roberto Amato, Sonia Goncalves, Ewan Harrison, David K. Jackson, Ian Johnston, Dominic Kwiatkowski, Cordelia Langford, John Sillitoe on behalf of the Wellcome Sanger Institute COVID-19 Surveillance Team
EPI_ISL_760616	Lighthouse Lab in Glasgow	Wellcome Sanger Institute for the COVID-19 Genomics UK (COG-UK) Consortium	Harper VanSteenhouse, Yumi Kasai, David Gray, Carol Clugston, Anna Dominiczak and Alex Alderton, Roberto Amato, Sonia Goncalves, Ewan Harrison, David K. Jackson, Ian Johnston, Dominic Kwiatkowski, Cordelia Langford, John Sillitoe on behalf of the Wellcome Sanger Institute COVID-19 Surveillance Team
EPI_ISL_760617	Lighthouse Lab in Alderley Park	Wellcome Sanger Institute for the COVID-19 Genomics UK (COG-UK) Consortium	Jacquelyn Wynn, Mairead Hyland, The Lighthouse Lab in Alderley Park and Alex Alderton, Roberto Amato, Sonia Goncalves, Ewan Harrison, David K. Jackson, Ian Johnston, Dominic Kwiatkowski, Cordelia Langford, John Sillitoe on behalf of the Wellcome Sanger Institute COVID-19 Surveillance Team
EPI_ISL_760618, EPI_ISL_760619, EPI_ISL_760621	Lighthouse Lab in Glasgow	Wellcome Sanger Institute for the COVID-19 Genomics UK (COG-UK) Consortium	Harper VanSteenhouse, Yumi Kasai, David Gray, Carol Clugston, Anna Dominiczak and Alex Alderton, Roberto Amato, Sonia Goncalves, Ewan Harrison, David K. Jackson, Ian Johnston, Dominic Kwiatkowski, Cordelia Langford, John Sillitoe on behalf of the Wellcome Sanger Institute COVID-19 Surveillance Team
EPI_ISL_760623, EPI_ISL_760624, EPI_ISL_760627	Lighthouse Lab in Alderley Park	Wellcome Sanger Institute for the COVID-19 Genomics UK (COG-UK) Consortium	Jacquelyn Wynn, Mairead Hyland, The Lighthouse Lab in Alderley Park and Alex Alderton, Roberto Amato, Sonia Goncalves, Ewan Harrison, David K. Jackson, Ian Johnston, Dominic Kwiatkowski, Cordelia Langford, John Sillitoe on behalf of the Wellcome Sanger Institute COVID-19 Surveillance Team
EPI_ISL_760634, EPI_ISL_760635, EPI_ISL_760636, EPI_ISL_760638	Lighthouse Lab in Glasgow	Wellcome Sanger Institute for the COVID-19 Genomics UK (COG-UK) Consortium	Harper VanSteenhouse, Yumi Kasai, David Gray, Carol Clugston, Anna Dominiczak and Alex Alderton, Roberto Amato, Sonia Goncalves, Ewan Harrison, David K. Jackson, Ian Johnston, Dominic Kwiatkowski, Cordelia Langford, John Sillitoe on behalf of the Wellcome Sanger Institute COVID-19 Surveillance Team
EPI_ISL_760640	Lighthouse Lab in Alderley Park	Wellcome Sanger Institute for the COVID-19 Genomics UK (COG-UK) Consortium	Jacquelyn Wynn, Mairead Hyland, The Lighthouse Lab in Alderley Park and Alex Alderton, Roberto Amato, Sonia Goncalves, Ewan Harrison, David K. Jackson, Ian Johnston, Dominic Kwiatkowski, Cordelia Langford, John Sillitoe on behalf of the Wellcome Sanger Institute COVID-19 Surveillance Team
EPI_ISL_760643	Lighthouse Lab in Glasgow	Wellcome Sanger Institute for the COVID-19 Genomics UK (COG-UK) Consortium	Harper VanSteenhouse, Yumi Kasai, David Gray, Carol Clugston, Anna Dominiczak and Alex Alderton, Roberto Amato, Sonia Goncalves, Ewan Harrison, David K. Jackson, Ian Johnston, Dominic Kwiatkowski, Cordelia Langford, John Sillitoe on behalf of the Wellcome Sanger Institute COVID-19 Surveillance Team
EPI_ISL_760644	Lighthouse Lab in Alderley Park	Wellcome Sanger Institute for the COVID-19 Genomics UK (COG-UK) Consortium	Jacquelyn Wynn, Mairead Hyland, The Lighthouse Lab in Alderley Park and Alex Alderton, Roberto Amato, Sonia Goncalves, Ewan Harrison, David K. Jackson, Ian Johnston, Dominic Kwiatkowski, Cordelia Langford, John Sillitoe on behalf of the Wellcome Sanger Institute COVID-19 Surveillance Team
EPI_ISL_760645, EPI_ISL_760646	Lighthouse Lab in Glasgow	Wellcome Sanger Institute for the COVID-19 Genomics UK (COG-UK) Consortium	Harper VanSteenhouse, Yumi Kasai, David Gray, Carol Clugston, Anna Dominiczak and Alex Alderton, Roberto Amato, Sonia Goncalves, Ewan Harrison, David K. Jackson, Ian Johnston, Dominic Kwiatkowski, Cordelia Langford, John Sillitoe on behalf of the Wellcome Sanger Institute COVID-19 Surveillance Team
EPI_ISL_760647	Lighthouse Lab in Alderley Park	Wellcome Sanger Institute for the COVID-19 Genomics UK (COG-UK) Consortium	Jacquelyn Wynn, Mairead Hyland, The Lighthouse Lab in Alderley Park and Alex Alderton, Roberto Amato, Sonia Goncalves, Ewan Harrison, David K. Jackson, Ian Johnston, Dominic Kwiatkowski, Cordelia Langford, John Sillitoe on behalf of the Wellcome Sanger Institute COVID-19 Surveillance Team
EPI_ISL_760648	Lighthouse Lab in Glasgow	Wellcome Sanger Institute for the COVID-19 Genomics UK (COG-UK) Consortium	Harper VanSteenhouse, Yumi Kasai, David Gray, Carol Clugston, Anna Dominiczak and Alex Alderton, Roberto Amato, Sonia Goncalves, Ewan Harrison, David K. Jackson, Ian Johnston, Dominic Kwiatkowski, Cordelia Langford, John Sillitoe on behalf of the Wellcome Sanger Institute COVID-19 Surveillance Team
EPI_ISL_760649, EPI_ISL_760650	Lighthouse Lab in Alderley Park	Wellcome Sanger Institute for the COVID-19 Genomics UK (COG-UK) Consortium	Jacquelyn Wynn, Mairead Hyland, The Lighthouse Lab in Alderley Park and Alex Alderton, Roberto Amato, Sonia Goncalves, Ewan Harrison, David K. Jackson, Ian Johnston, Dominic Kwiatkowski, Cordelia Langford, John Sillitoe on behalf of the Wellcome Sanger Institute COVID-19 Surveillance Team
EPI_ISL_760651	Lighthouse Lab in Glasgow	Wellcome Sanger Institute for the COVID-19 Genomics UK (COG-UK) Consortium	Harper VanSteenhouse, Yumi Kasai, David Gray, Carol Clugston, Anna Dominiczak and Alex Alderton, Roberto Amato, Sonia Goncalves, Ewan Harrison, David K. Jackson, Ian Johnston, Dominic Kwiatkowski, Cordelia Langford, John Sillitoe on behalf of the Wellcome Sanger Institute COVID-19 Surveillance Team
EPI_ISL_760652, EPI_ISL_760656	Lighthouse Lab in Alderley Park	Wellcome Sanger Institute for the COVID-19 Genomics UK (COG-UK) Consortium	Jacquelyn Wynn, Mairead Hyland, The Lighthouse Lab in Alderley Park and Alex Alderton, Roberto Amato, Sonia Goncalves, Ewan Harrison, David K. Jackson, Ian Johnston, Dominic Kwiatkowski, Cordelia Langford, John Sillitoe on behalf of the Wellcome Sanger Institute COVID-19 Surveillance Team
EPI_ISL_760657, EPI_ISL_760659	Lighthouse Lab in Glasgow	Wellcome Sanger Institute for the COVID-19 Genomics UK (COG-UK) Consortium	Harper VanSteenhouse, Yumi Kasai, David Gray, Carol Clugston, Anna Dominiczak and Alex Alderton, Roberto Amato, Sonia Goncalves, Ewan Harrison, David K. Jackson, Ian Johnston, Dominic Kwiatkowski, Cordelia Langford, John Sillitoe on behalf of the Wellcome Sanger Institute COVID-19 Surveillance Team
EPI_ISL_760660, EPI_ISL_760661	Lighthouse Lab in Alderley Park	Wellcome Sanger Institute for the COVID-19 Genomics UK (COG-UK) Consortium	Jacquelyn Wynn, Mairead Hyland, The Lighthouse Lab in Alderley Park and Alex Alderton, Roberto Amato, Sonia Goncalves, Ewan Harrison, David K. Jackson, Ian Johnston, Dominic Kwiatkowski, Cordelia Langford, John Sillitoe on behalf of the Wellcome Sanger Institute COVID-19 Surveillance Team
EPI_ISL_760662	Lighthouse Lab in Glasgow	Wellcome Sanger Institute for the COVID-19 Genomics UK (COG-UK) Consortium	Harper VanSteenhouse, Yumi Kasai, David Gray, Carol Clugston, Anna Dominiczak and Alex Alderton, Roberto Amato, Sonia Goncalves, Ewan Harrison, David K. Jackson, Ian Johnston, Dominic Kwiatkowski, Cordelia Langford, John Sillitoe on behalf of the Wellcome Sanger Institute COVID-19 Surveillance Team
EPI_ISL_760663, EPI_ISL_760664, EPI_ISL_760665, EPI_ISL_760666, EPI_ISL_760667	Lighthouse Lab in Alderley Park	Wellcome Sanger Institute for the COVID-19 Genomics UK (COG-UK) Consortium	Jacquelyn Wynn, Mairead Hyland, The Lighthouse Lab in Alderley Park and Alex Alderton, Roberto Amato, Sonia Goncalves, Ewan Harrison, David K. Jackson, Ian Johnston, Dominic Kwiatkowski, Cordelia Langford, John Sillitoe on behalf of the Wellcome Sanger Institute COVID-19 Surveillance Team
EPI_ISL_760668, EPI_ISL_760669	Lighthouse Lab in Glasgow	Wellcome Sanger Institute for the COVID-19 Genomics UK (COG-UK) Consortium	Harper VanSteenhouse, Yumi Kasai, David Gray, Carol Clugston, Anna Dominiczak and Alex Alderton, Roberto Amato, Sonia Goncalves, Ewan Harrison, David K. Jackson, Ian Johnston, Dominic Kwiatkowski, Cordelia Langford, John Sillitoe on behalf of the Wellcome Sanger Institute COVID-19 Surveillance Team
EPI_ISL_760670	Lighthouse Lab in Alderley Park	Wellcome Sanger Institute for the COVID-19 Genomics UK (COG-UK) Consortium	Jacquelyn Wynn, Mairead Hyland, The Lighthouse Lab in Alderley Park and Alex Alderton, Roberto Amato, Sonia Goncalves, Ewan Harrison, David K. Jackson, Ian Johnston, Dominic Kwiatkowski, Cordelia Langford, John Sillitoe on behalf of the Wellcome Sanger Institute COVID-19 Surveillance Team
EPI_ISL_760671, EPI_ISL_760673, EPI_ISL_760674, EPI_ISL_760675, EPI_ISL_760676	Lighthouse Lab in Glasgow	Wellcome Sanger Institute for the COVID-19 Genomics UK (COG-UK) Consortium	Harper VanSteenhouse, Yumi Kasai, David Gray, Carol Clugston, Anna Dominiczak and Alex Alderton, Roberto Amato, Sonia Goncalves, Ewan Harrison, David K. Jackson, Ian Johnston, Dominic Kwiatkowski, Cordelia Langford, John Sillitoe on behalf of the Wellcome Sanger Institute COVID-19 Surveillance Team
EPI_ISL_760677, EPI_ISL_760679	Lighthouse Lab in Alderley Park	Wellcome Sanger Institute for the COVID-19 Genomics UK (COG-UK) Consortium	Jacquelyn Wynn, Mairead Hyland, The Lighthouse Lab in Alderley Park and Alex Alderton, Roberto Amato, Sonia Goncalves, Ewan Harrison, David K. Jackson, Ian Johnston, Dominic Kwiatkowski, Cordelia Langford, John Sillitoe on behalf of the Wellcome Sanger Institute COVID-19 Surveillance Team
EPI_ISL_760682, EPI_ISL_760683	Lighthouse Lab in Glasgow	Wellcome Sanger Institute for the COVID-19 Genomics UK (COG-UK) Consortium	Harper VanSteenhouse, Yumi Kasai, David Gray, Carol Clugston, Anna Dominiczak and Alex Alderton, Roberto Amato, Sonia Goncalves, Ewan Harrison, David K. Jackson, Ian Johnston, Dominic Kwiatkowski, Cordelia Langford, John Sillitoe on behalf of the Wellcome Sanger Institute COVID-19 Surveillance Team
EPI_ISL_760684	Lighthouse Lab in Alderley Park	Wellcome Sanger Institute for the COVID-19 Genomics UK (COG-UK) Consortium	Jacquelyn Wynn, Mairead Hyland, The Lighthouse Lab in Alderley Park and Alex Alderton, Roberto Amato, Sonia Goncalves, Ewan Harrison, David K. Jackson, Ian Johnston, Dominic Kwiatkowski, Cordelia Langford, John Sillitoe on behalf of the Wellcome Sanger Institute COVID-19 Surveillance Team
EPI_ISL_760686, EPI_ISL_760687	Lighthouse Lab in Glasgow	Wellcome Sanger Institute for the COVID-19 Genomics UK (COG-UK) Consortium	Harper VanSteenhouse, Yumi Kasai, David Gray, Carol Clugston, Anna Dominiczak and Alex Alderton, Roberto Amato, Sonia Goncalves, Ewan Harrison, David K. Jackson, Ian Johnston, Dominic Kwiatkowski, Cordelia Langford, John Sillitoe on behalf of the Wellcome Sanger Institute COVID-19 Surveillance Team
EPI_ISL_760688	Lighthouse Lab in Alderley Park	Wellcome Sanger Institute for the COVID-19 Genomics UK (COG-UK) Consortium	Jacquelyn Wynn, Mairead Hyland, The Lighthouse Lab in Alderley Park and Alex Alderton, Roberto Amato, Sonia Goncalves, Ewan Harrison, David K. Jackson, Ian Johnston, Dominic Kwiatkowski, Cordelia Langford, John Sillitoe on behalf of the Wellcome Sanger Institute COVID-19 Surveillance Team
EPI_ISL_760689, EPI_ISL_760690	Lighthouse Lab in Glasgow	Wellcome Sanger Institute for the COVID-19 Genomics UK (COG-UK) Consortium	Harper VanSteenhouse, Yumi Kasai, David Gray, Carol Clugston, Anna Dominiczak and Alex Alderton, Roberto Amato, Sonia Goncalves, Ewan Harrison, David K. Jackson, Ian Johnston, Dominic Kwiatkowski, Cordelia Langford, John Sillitoe on behalf of the Wellcome Sanger Institute COVID-19 Surveillance Team
EPI_ISL_760691	Lighthouse Lab in Alderley Park	Wellcome Sanger Institute for the COVID-19 Genomics UK (COG-UK) Consortium	Jacquelyn Wynn, Mairead Hyland, The Lighthouse Lab in Alderley Park and Alex Alderton, Roberto Amato, Sonia Goncalves, Ewan Harrison, David K. Jackson, Ian Johnston, Dominic Kwiatkowski, Cordelia Langford, John Sillitoe on behalf of the Wellcome Sanger Institute COVID-19 Surveillance Team

EPI_ISL_760692	Lighthouse Lab in Glasgow	Wellcome Sanger Institute for the COVID-19 Genomics UK (COG-UK) Consortium	Harper VanSteenhouse, Yumi Kasai, David Gray, Carol Clugston, Anna Dominiczak and Alex Alderton, Roberto Amato, Sonia Goncalves, Ewan Harrison, David K. Jackson, Ian Johnston, Dominic Kwiatkowski, Cordelia Langford, John Sillitoe on behalf of the Wellcome Sanger Institute COVID-19 Surveillance Team
EPI_ISL_760693	Lighthouse Lab in Alderley Park	Wellcome Sanger Institute for the COVID-19 Genomics UK (COG-UK) Consortium	Jacquelyn Wynn, Mairead Hyland, The Lighthouse Lab in Alderley Park and Alex Alderton, Roberto Amato, Sonia Goncalves, Ewan Harrison, David K. Jackson, Ian Johnston, Dominic Kwiatkowski, Cordelia Langford, John Sillitoe on behalf of the Wellcome Sanger Institute COVID-19 Surveillance Team
EPI_ISL_760694, EPI_ISL_760695, EPI_ISL_760696	Lighthouse Lab in Glasgow	Wellcome Sanger Institute for the COVID-19 Genomics UK (COG-UK) Consortium	Harper VanSteenhouse, Yumi Kasai, David Gray, Carol Clugston, Anna Dominiczak and Alex Alderton, Roberto Amato, Sonia Goncalves, Ewan Harrison, David K. Jackson, Ian Johnston, Dominic Kwiatkowski, Cordelia Langford, John Sillitoe on behalf of the Wellcome Sanger Institute COVID-19 Surveillance Team
EPI_ISL_760698	Lighthouse Lab in Milton Keynes	Wellcome Sanger Institute for the COVID-19 Genomics UK (COG-UK) Consortium	The Lighthouse Lab in Milton Keynes and Alex Alderton, Roberto Amato, Sonia Goncalves, Ewan Harrison, David K. Jackson, Ian Johnston, Dominic Kwiatkowski, Cordelia Langford, John Sillitoe on behalf of the Wellcome Sanger Institute COVID-19 Surveillance Team
EPI_ISL_760699	Lighthouse Lab in Alderley Park	Wellcome Sanger Institute for the COVID-19 Genomics UK (COG-UK) Consortium	Jacquelyn Wynn, Mairead Hyland, The Lighthouse Lab in Alderley Park and Alex Alderton, Roberto Amato, Sonia Goncalves, Ewan Harrison, David K. Jackson, Ian Johnston, Dominic Kwiatkowski, Cordelia Langford, John Sillitoe on behalf of the Wellcome Sanger Institute COVID-19 Surveillance Team
EPI_ISL_760700	Lighthouse Lab in Glasgow	Wellcome Sanger Institute for the COVID-19 Genomics UK (COG-UK) Consortium	Harper VanSteenhouse, Yumi Kasai, David Gray, Carol Clugston, Anna Dominiczak and Alex Alderton, Roberto Amato, Sonia Goncalves, Ewan Harrison, David K. Jackson, Ian Johnston, Dominic Kwiatkowski, Cordelia Langford, John Sillitoe on behalf of the Wellcome Sanger Institute COVID-19 Surveillance Team
EPI_ISL_760701, EPI_ISL_760702	Lighthouse Lab in Alderley Park	Wellcome Sanger Institute for the COVID-19 Genomics UK (COG-UK) Consortium	Jacquelyn Wynn, Mairead Hyland, The Lighthouse Lab in Alderley Park and Alex Alderton, Roberto Amato, Sonia Goncalves, Ewan Harrison, David K. Jackson, Ian Johnston, Dominic Kwiatkowski, Cordelia Langford, John Sillitoe on behalf of the Wellcome Sanger Institute COVID-19 Surveillance Team
EPI_ISL_760704	Lighthouse Lab in Glasgow	Wellcome Sanger Institute for the COVID-19 Genomics UK (COG-UK) Consortium	Harper VanSteenhouse, Yumi Kasai, David Gray, Carol Clugston, Anna Dominiczak and Alex Alderton, Roberto Amato, Sonia Goncalves, Ewan Harrison, David K. Jackson, Ian Johnston, Dominic Kwiatkowski, Cordelia Langford, John Sillitoe on behalf of the Wellcome Sanger Institute COVID-19 Surveillance Team
EPI_ISL_760705	Lighthouse Lab in Alderley Park	Wellcome Sanger Institute for the COVID-19 Genomics UK (COG-UK) Consortium	Jacquelyn Wynn, Mairead Hyland, The Lighthouse Lab in Alderley Park and Alex Alderton, Roberto Amato, Sonia Goncalves, Ewan Harrison, David K. Jackson, Ian Johnston, Dominic Kwiatkowski, Cordelia Langford, John Sillitoe on behalf of the Wellcome Sanger Institute COVID-19 Surveillance Team
EPI_ISL_760706, EPI_ISL_760707	Lighthouse Lab in Glasgow	Wellcome Sanger Institute for the COVID-19 Genomics UK (COG-UK) Consortium	Harper VanSteenhouse, Yumi Kasai, David Gray, Carol Clugston, Anna Dominiczak and Alex Alderton, Roberto Amato, Sonia Goncalves, Ewan Harrison, David K. Jackson, Ian Johnston, Dominic Kwiatkowski, Cordelia Langford, John Sillitoe on behalf of the Wellcome Sanger Institute COVID-19 Surveillance Team
EPI_ISL_760708, EPI_ISL_760710, EPI_ISL_760711	Lighthouse Lab in Alderley Park	Wellcome Sanger Institute for the COVID-19 Genomics UK (COG-UK) Consortium	Jacquelyn Wynn, Mairead Hyland, The Lighthouse Lab in Alderley Park and Alex Alderton, Roberto Amato, Sonia Goncalves, Ewan Harrison, David K. Jackson, Ian Johnston, Dominic Kwiatkowski, Cordelia Langford, John Sillitoe on behalf of the Wellcome Sanger Institute COVID-19 Surveillance Team
EPI_ISL_760713	Lighthouse Lab in Glasgow	Wellcome Sanger Institute for the COVID-19 Genomics UK (COG-UK) Consortium	Harper VanSteenhouse, Yumi Kasai, David Gray, Carol Clugston, Anna Dominiczak and Alex Alderton, Roberto Amato, Sonia Goncalves, Ewan Harrison, David K. Jackson, Ian Johnston, Dominic Kwiatkowski, Cordelia Langford, John Sillitoe on behalf of the Wellcome Sanger Institute COVID-19 Surveillance Team
EPI_ISL_760715	Lighthouse Lab in Alderley Park	Wellcome Sanger Institute for the COVID-19 Genomics UK (COG-UK) Consortium	Jacquelyn Wynn, Mairead Hyland, The Lighthouse Lab in Alderley Park and Alex Alderton, Roberto Amato, Sonia Goncalves, Ewan Harrison, David K. Jackson, Ian Johnston, Dominic Kwiatkowski, Cordelia Langford, John Sillitoe on behalf of the Wellcome Sanger Institute COVID-19 Surveillance Team
EPI_ISL_760716, EPI_ISL_760717	Lighthouse Lab in Glasgow	Wellcome Sanger Institute for the COVID-19 Genomics UK (COG-UK) Consortium	Harper VanSteenhouse, Yumi Kasai, David Gray, Carol Clugston, Anna Dominiczak and Alex Alderton, Roberto Amato, Sonia Goncalves, Ewan Harrison, David K. Jackson, Ian Johnston, Dominic Kwiatkowski, Cordelia Langford, John Sillitoe on behalf of the Wellcome Sanger Institute COVID-19 Surveillance Team
EPI_ISL_760721, EPI_ISL_760722, EPI_ISL_760723, EPI_ISL_760726, EPI_ISL_760727, EPI_ISL_760728	Lighthouse Lab in Alderley Park	Wellcome Sanger Institute for the COVID-19 Genomics UK (COG-UK) Consortium	Jacquelyn Wynn, Mairead Hyland, The Lighthouse Lab in Alderley Park and Alex Alderton, Roberto Amato, Sonia Goncalves, Ewan Harrison, David K. Jackson, Ian Johnston, Dominic Kwiatkowski, Cordelia Langford, John Sillitoe on behalf of the Wellcome Sanger Institute COVID-19 Surveillance Team
EPI_ISL_760730, EPI_ISL_760732	Lighthouse Lab in Glasgow	Wellcome Sanger Institute for the COVID-19 Genomics UK (COG-UK) Consortium	Harper VanSteenhouse, Yumi Kasai, David Gray, Carol Clugston, Anna Dominiczak and Alex Alderton, Roberto Amato, Sonia Goncalves, Ewan Harrison, David K. Jackson, Ian Johnston, Dominic Kwiatkowski, Cordelia Langford, John Sillitoe on behalf of the Wellcome Sanger Institute COVID-19 Surveillance Team
EPI_ISL_760733, EPI_ISL_760734	Lighthouse Lab in Alderley Park	Wellcome Sanger Institute for the COVID-19 Genomics UK (COG-UK) Consortium	Jacquelyn Wynn, Mairead Hyland, The Lighthouse Lab in Alderley Park and Alex Alderton, Roberto Amato, Sonia Goncalves, Ewan Harrison, David K. Jackson, Ian Johnston, Dominic Kwiatkowski, Cordelia Langford, John Sillitoe on behalf of the Wellcome Sanger Institute COVID-19 Surveillance Team
EPI_ISL_760735	Lighthouse Lab in Glasgow	Wellcome Sanger Institute for the COVID-19 Genomics UK (COG-UK) Consortium	Harper VanSteenhouse, Yumi Kasai, David Gray, Carol Clugston, Anna Dominiczak and Alex Alderton, Roberto Amato, Sonia Goncalves, Ewan Harrison, David K. Jackson, Ian Johnston, Dominic Kwiatkowski, Cordelia Langford, John Sillitoe on behalf of the Wellcome Sanger Institute COVID-19 Surveillance Team
EPI_ISL_760736, EPI_ISL_760737, EPI_ISL_760738	Lighthouse Lab in Alderley Park	Wellcome Sanger Institute for the COVID-19 Genomics UK (COG-UK) Consortium	Jacquelyn Wynn, Mairead Hyland, The Lighthouse Lab in Alderley Park and Alex Alderton, Roberto Amato, Sonia Goncalves, Ewan Harrison, David K. Jackson, Ian Johnston, Dominic Kwiatkowski, Cordelia Langford, John Sillitoe on behalf of the Wellcome Sanger Institute COVID-19 Surveillance Team
EPI_ISL_760741, EPI_ISL_760742, EPI_ISL_760743, EPI_ISL_760744	Lighthouse Lab in Glasgow	Wellcome Sanger Institute for the COVID-19 Genomics UK (COG-UK) Consortium	Harper VanSteenhouse, Yumi Kasai, David Gray, Carol Clugston, Anna Dominiczak and Alex Alderton, Roberto Amato, Sonia Goncalves, Ewan Harrison, David K. Jackson, Ian Johnston, Dominic Kwiatkowski, Cordelia Langford, John Sillitoe on behalf of the Wellcome Sanger Institute COVID-19 Surveillance Team
EPI_ISL_760745	Lighthouse Lab in Alderley Park	Wellcome Sanger Institute for the COVID-19 Genomics UK (COG-UK) Consortium	Jacquelyn Wynn, Mairead Hyland, The Lighthouse Lab in Alderley Park and Alex Alderton, Roberto Amato, Sonia Goncalves, Ewan Harrison, David K. Jackson, Ian Johnston, Dominic Kwiatkowski, Cordelia Langford, John Sillitoe on behalf of the Wellcome Sanger Institute COVID-19 Surveillance Team
EPI_ISL_760746	Lighthouse Lab in Glasgow	Wellcome Sanger Institute for the COVID-19 Genomics UK (COG-UK) Consortium	Harper VanSteenhouse, Yumi Kasai, David Gray, Carol Clugston, Anna Dominiczak and Alex Alderton, Roberto Amato, Sonia Goncalves, Ewan Harrison, David K. Jackson, Ian Johnston, Dominic Kwiatkowski, Cordelia Langford, John Sillitoe on behalf of the Wellcome Sanger Institute COVID-19 Surveillance Team
EPI_ISL_760748	Lighthouse Lab in Alderley Park	Wellcome Sanger Institute for the COVID-19 Genomics UK (COG-UK) Consortium	Jacquelyn Wynn, Mairead Hyland, The Lighthouse Lab in Alderley Park and Alex Alderton, Roberto Amato, Sonia Goncalves, Ewan Harrison, David K. Jackson, Ian Johnston, Dominic Kwiatkowski, Cordelia Langford, John Sillitoe on behalf of the Wellcome Sanger Institute COVID-19 Surveillance Team
EPI_ISL_760749, EPI_ISL_760750, EPI_ISL_760751	Lighthouse Lab in Glasgow	Wellcome Sanger Institute for the COVID-19 Genomics UK (COG-UK) Consortium	Harper VanSteenhouse, Yumi Kasai, David Gray, Carol Clugston, Anna Dominiczak and Alex Alderton, Roberto Amato, Sonia Goncalves, Ewan Harrison, David K. Jackson, Ian Johnston, Dominic Kwiatkowski, Cordelia Langford, John Sillitoe on behalf of the Wellcome Sanger Institute COVID-19 Surveillance Team
EPI_ISL_760753, EPI_ISL_760755, EPI_ISL_760756	Lighthouse Lab in Alderley Park	Wellcome Sanger Institute for the COVID-19 Genomics UK (COG-UK) Consortium	Jacquelyn Wynn, Mairead Hyland, The Lighthouse Lab in Alderley Park and Alex Alderton, Roberto Amato, Sonia Goncalves, Ewan Harrison, David K. Jackson, Ian Johnston, Dominic Kwiatkowski, Cordelia Langford, John Sillitoe on behalf of the Wellcome Sanger Institute COVID-19 Surveillance Team
EPI_ISL_760757	Lighthouse Lab in Glasgow	Wellcome Sanger Institute for the COVID-19 Genomics UK (COG-UK) Consortium	Harper VanSteenhouse, Yumi Kasai, David Gray, Carol Clugston, Anna Dominiczak and Alex Alderton, Roberto Amato, Sonia Goncalves, Ewan Harrison, David K. Jackson, Ian Johnston, Dominic Kwiatkowski, Cordelia Langford, John Sillitoe on behalf of the Wellcome Sanger Institute COVID-19 Surveillance Team
EPI_ISL_760759, EPI_ISL_760761, EPI_ISL_760762	Lighthouse Lab in Alderley Park	Wellcome Sanger Institute for the COVID-19 Genomics UK (COG-UK) Consortium	Jacquelyn Wynn, Mairead Hyland, The Lighthouse Lab in Alderley Park and Alex Alderton, Roberto Amato, Sonia Goncalves, Ewan Harrison, David K. Jackson, Ian Johnston, Dominic Kwiatkowski, Cordelia Langford, John Sillitoe on behalf of the Wellcome Sanger Institute COVID-19 Surveillance Team
EPI_ISL_760764, EPI_ISL_760765, EPI_ISL_760766, EPI_ISL_760767, EPI_ISL_760768	Lighthouse Lab in Glasgow	Wellcome Sanger Institute for the COVID-19 Genomics UK (COG-UK) Consortium	Harper VanSteenhouse, Yumi Kasai, David Gray, Carol Clugston, Anna Dominiczak and Alex Alderton, Roberto Amato, Sonia Goncalves, Ewan Harrison, David K. Jackson, Ian Johnston, Dominic Kwiatkowski, Cordelia Langford, John Sillitoe on behalf of the Wellcome Sanger Institute COVID-19 Surveillance Team
EPI_ISL_760769	Lighthouse Lab in Alderley Park	Wellcome Sanger Institute for the COVID-19 Genomics UK (COG-UK) Consortium	Jacquelyn Wynn, Mairead Hyland, The Lighthouse Lab in Alderley Park and Alex Alderton, Roberto Amato, Sonia Goncalves, Ewan Harrison, David K. Jackson, Ian Johnston, Dominic Kwiatkowski, Cordelia Langford, John Sillitoe on behalf of the Wellcome Sanger Institute COVID-19 Surveillance Team

EPI_ISL_760772, EPI_ISL_760773, EPI_ISL_760774	Lighthouse Lab in Glasgow	Wellcome Sanger Institute for the COVID-19 Genomics UK (COG-UK) Consortium	Harper VanSteenhouse, Yumi Kasai, David Gray, Carol Clugston, Anna Dominiczak and Alex Alderton, Roberto Amato, Sonia Goncalves, Ewan Harrison, David K. Jackson, Ian Johnston, Dominic Kwiatkowski, Cordelia Langford, John Sillitoe on behalf of the Wellcome Sanger Institute COVID-19 Surveillance Team
EPI_ISL_760776	Lighthouse Lab in Alderley Park	Wellcome Sanger Institute for the COVID-19 Genomics UK (COG-UK) Consortium	Jacquelyn Wynn, Mairead Hyland, The Lighthouse Lab in Alderley Park and Alex Alderton, Roberto Amato, Sonia Goncalves, Ewan Harrison, David K. Jackson, Ian Johnston, Dominic Kwiatkowski, Cordelia Langford, John Sillitoe on behalf of the Wellcome Sanger Institute COVID-19 Surveillance Team
EPI_ISL_760779	Lighthouse Lab in Glasgow	Wellcome Sanger Institute for the COVID-19 Genomics UK (COG-UK) Consortium	Harper VanSteenhouse, Yumi Kasai, David Gray, Carol Clugston, Anna Dominiczak and Alex Alderton, Roberto Amato, Sonia Goncalves, Ewan Harrison, David K. Jackson, Ian Johnston, Dominic Kwiatkowski, Cordelia Langford, John Sillitoe on behalf of the Wellcome Sanger Institute COVID-19 Surveillance Team
EPI_ISL_760780, EPI_ISL_760783, EPI_ISL_760784, EPI_ISL_760785, EPI_ISL_760786, EPI_ISL_760788, EPI_ISL_760790	Lighthouse Lab in Alderley Park	Wellcome Sanger Institute for the COVID-19 Genomics UK (COG-UK) Consortium	Jacquelyn Wynn, Mairead Hyland, The Lighthouse Lab in Alderley Park and Alex Alderton, Roberto Amato, Sonia Goncalves, Ewan Harrison, David K. Jackson, Ian Johnston, Dominic Kwiatkowski, Cordelia Langford, John Sillitoe on behalf of the Wellcome Sanger Institute COVID-19 Surveillance Team
EPI_ISL_760791	Lighthouse Lab in Glasgow	Wellcome Sanger Institute for the COVID-19 Genomics UK (COG-UK) Consortium	Harper VanSteenhouse, Yumi Kasai, David Gray, Carol Clugston, Anna Dominiczak and Alex Alderton, Roberto Amato, Sonia Goncalves, Ewan Harrison, David K. Jackson, Ian Johnston, Dominic Kwiatkowski, Cordelia Langford, John Sillitoe on behalf of the Wellcome Sanger Institute COVID-19 Surveillance Team
EPI_ISL_760792	Lighthouse Lab in Alderley Park	Wellcome Sanger Institute for the COVID-19 Genomics UK (COG-UK) Consortium	Jacquelyn Wynn, Mairead Hyland, The Lighthouse Lab in Alderley Park and Alex Alderton, Roberto Amato, Sonia Goncalves, Ewan Harrison, David K. Jackson, Ian Johnston, Dominic Kwiatkowski, Cordelia Langford, John Sillitoe on behalf of the Wellcome Sanger Institute COVID-19 Surveillance Team
EPI_ISL_760793	Lighthouse Lab in Glasgow	Wellcome Sanger Institute for the COVID-19 Genomics UK (COG-UK) Consortium	Harper VanSteenhouse, Yumi Kasai, David Gray, Carol Clugston, Anna Dominiczak and Alex Alderton, Roberto Amato, Sonia Goncalves, Ewan Harrison, David K. Jackson, Ian Johnston, Dominic Kwiatkowski, Cordelia Langford, John Sillitoe on behalf of the Wellcome Sanger Institute COVID-19 Surveillance Team
EPI_ISL_760794, EPI_ISL_760795	Lighthouse Lab in Alderley Park	Wellcome Sanger Institute for the COVID-19 Genomics UK (COG-UK) Consortium	Jacquelyn Wynn, Mairead Hyland, The Lighthouse Lab in Alderley Park and Alex Alderton, Roberto Amato, Sonia Goncalves, Ewan Harrison, David K. Jackson, Ian Johnston, Dominic Kwiatkowski, Cordelia Langford, John Sillitoe on behalf of the Wellcome Sanger Institute COVID-19 Surveillance Team
EPI_ISL_760797, EPI_ISL_760798	Lighthouse Lab in Glasgow	Wellcome Sanger Institute for the COVID-19 Genomics UK (COG-UK) Consortium	Harper VanSteenhouse, Yumi Kasai, David Gray, Carol Clugston, Anna Dominiczak and Alex Alderton, Roberto Amato, Sonia Goncalves, Ewan Harrison, David K. Jackson, Ian Johnston, Dominic Kwiatkowski, Cordelia Langford, John Sillitoe on behalf of the Wellcome Sanger Institute COVID-19 Surveillance Team
EPI_ISL_760799	Lighthouse Lab in Alderley Park	Wellcome Sanger Institute for the COVID-19 Genomics UK (COG-UK) Consortium	Jacquelyn Wynn, Mairead Hyland, The Lighthouse Lab in Alderley Park and Alex Alderton, Roberto Amato, Sonia Goncalves, Ewan Harrison, David K. Jackson, Ian Johnston, Dominic Kwiatkowski, Cordelia Langford, John Sillitoe on behalf of the Wellcome Sanger Institute COVID-19 Surveillance Team
EPI_ISL_760800	Lighthouse Lab in Glasgow	Wellcome Sanger Institute for the COVID-19 Genomics UK (COG-UK) Consortium	Harper VanSteenhouse, Yumi Kasai, David Gray, Carol Clugston, Anna Dominiczak and Alex Alderton, Roberto Amato, Sonia Goncalves, Ewan Harrison, David K. Jackson, Ian Johnston, Dominic Kwiatkowski, Cordelia Langford, John Sillitoe on behalf of the Wellcome Sanger Institute COVID-19 Surveillance Team
EPI_ISL_760801	Lighthouse Lab in Milton Keynes	Wellcome Sanger Institute for the COVID-19 Genomics UK (COG-UK) Consortium	The Lighthouse Lab in Milton Keynes and Alex Alderton, Roberto Amato, Sonia Goncalves, Ewan Harrison, David K. Jackson, Ian Johnston, Dominic Kwiatkowski, Cordelia Langford, John Sillitoe on behalf of the Wellcome Sanger Institute COVID-19 Surveillance Team
EPI_ISL_760803	Lighthouse Lab in Glasgow	Wellcome Sanger Institute for the COVID-19 Genomics UK (COG-UK) Consortium	Harper VanSteenhouse, Yumi Kasai, David Gray, Carol Clugston, Anna Dominiczak and Alex Alderton, Roberto Amato, Sonia Goncalves, Ewan Harrison, David K. Jackson, Ian Johnston, Dominic Kwiatkowski, Cordelia Langford, John Sillitoe on behalf of the Wellcome Sanger Institute COVID-19 Surveillance Team
EPI_ISL_760804	Lighthouse Lab in Alderley Park	Wellcome Sanger Institute for the COVID-19 Genomics UK (COG-UK) Consortium	Jacquelyn Wynn, Mairead Hyland, The Lighthouse Lab in Alderley Park and Alex Alderton, Roberto Amato, Sonia Goncalves, Ewan Harrison, David K. Jackson, Ian Johnston, Dominic Kwiatkowski, Cordelia Langford, John Sillitoe on behalf of the Wellcome Sanger Institute COVID-19 Surveillance Team
EPI_ISL_760805	Lighthouse Lab in Glasgow	Wellcome Sanger Institute for the COVID-19 Genomics UK (COG-UK) Consortium	Harper VanSteenhouse, Yumi Kasai, David Gray, Carol Clugston, Anna Dominiczak and Alex Alderton, Roberto Amato, Sonia Goncalves, Ewan Harrison, David K. Jackson, Ian Johnston, Dominic Kwiatkowski, Cordelia Langford, John Sillitoe on behalf of the Wellcome Sanger Institute COVID-19 Surveillance Team
EPI_ISL_760806, EPI_ISL_760808	Lighthouse Lab in Alderley Park	Wellcome Sanger Institute for the COVID-19 Genomics UK (COG-UK) Consortium	Jacquelyn Wynn, Mairead Hyland, The Lighthouse Lab in Alderley Park and Alex Alderton, Roberto Amato, Sonia Goncalves, Ewan Harrison, David K. Jackson, Ian Johnston, Dominic Kwiatkowski, Cordelia Langford, John Sillitoe on behalf of the Wellcome Sanger Institute COVID-19 Surveillance Team
EPI_ISL_760809, EPI_ISL_760810	Lighthouse Lab in Milton Keynes	Wellcome Sanger Institute for the COVID-19 Genomics UK (COG-UK) Consortium	The Lighthouse Lab in Milton Keynes and Alex Alderton, Roberto Amato, Sonia Goncalves, Ewan Harrison, David K. Jackson, Ian Johnston, Dominic Kwiatkowski, Cordelia Langford, John Sillitoe on behalf of the Wellcome Sanger Institute COVID-19 Surveillance Team
EPI_ISL_760811	Lighthouse Lab in Glasgow	Wellcome Sanger Institute for the COVID-19 Genomics UK (COG-UK) Consortium	Harper VanSteenhouse, Yumi Kasai, David Gray, Carol Clugston, Anna Dominiczak and Alex Alderton, Roberto Amato, Sonia Goncalves, Ewan Harrison, David K. Jackson, Ian Johnston, Dominic Kwiatkowski, Cordelia Langford, John Sillitoe on behalf of the Wellcome Sanger Institute COVID-19 Surveillance Team
EPI_ISL_760813	Lighthouse Lab in Cambridge	Wellcome Sanger Institute for the COVID-19 Genomics UK (COG-UK) Consortium	Rob Howes, The Lighthouse Lab in Cambridge and Alex Alderton, Roberto Amato, Sonia Goncalves, Ewan Harrison, David K. Jackson, Ian Johnston, Dominic Kwiatkowski, Cordelia Langford, John Sillitoe on behalf of the Wellcome Sanger Institute COVID-19 Surveillance Team
EPI_ISL_760816	Lighthouse Lab in Glasgow	Wellcome Sanger Institute for the COVID-19 Genomics UK (COG-UK) Consortium	Harper VanSteenhouse, Yumi Kasai, David Gray, Carol Clugston, Anna Dominiczak and Alex Alderton, Roberto Amato, Sonia Goncalves, Ewan Harrison, David K. Jackson, Ian Johnston, Dominic Kwiatkowski, Cordelia Langford, John Sillitoe on behalf of the Wellcome Sanger Institute COVID-19 Surveillance Team
EPI_ISL_760817	Lighthouse Lab in Alderley Park	Wellcome Sanger Institute for the COVID-19 Genomics UK (COG-UK) Consortium	Jacquelyn Wynn, Mairead Hyland, The Lighthouse Lab in Alderley Park and Alex Alderton, Roberto Amato, Sonia Goncalves, Ewan Harrison, David K. Jackson, Ian Johnston, Dominic Kwiatkowski, Cordelia Langford, John Sillitoe on behalf of the Wellcome Sanger Institute COVID-19 Surveillance Team
EPI_ISL_760818, EPI_ISL_760819, EPI_ISL_760820	Lighthouse Lab in Glasgow	Wellcome Sanger Institute for the COVID-19 Genomics UK (COG-UK) Consortium	Harper VanSteenhouse, Yumi Kasai, David Gray, Carol Clugston, Anna Dominiczak and Alex Alderton, Roberto Amato, Sonia Goncalves, Ewan Harrison, David K. Jackson, Ian Johnston, Dominic Kwiatkowski, Cordelia Langford, John Sillitoe on behalf of the Wellcome Sanger Institute COVID-19 Surveillance Team
EPI_ISL_760822	Lighthouse Lab in Cambridge	Wellcome Sanger Institute for the COVID-19 Genomics UK (COG-UK) Consortium	Rob Howes, The Lighthouse Lab in Cambridge and Alex Alderton, Roberto Amato, Sonia Goncalves, Ewan Harrison, David K. Jackson, Ian Johnston, Dominic Kwiatkowski, Cordelia Langford, John Sillitoe on behalf of the Wellcome Sanger Institute COVID-19 Surveillance Team
EPI_ISL_760824	Lighthouse Lab in Glasgow	Wellcome Sanger Institute for the COVID-19 Genomics UK (COG-UK) Consortium	Harper VanSteenhouse, Yumi Kasai, David Gray, Carol Clugston, Anna Dominiczak and Alex Alderton, Roberto Amato, Sonia Goncalves, Ewan Harrison, David K. Jackson, Ian Johnston, Dominic Kwiatkowski, Cordelia Langford, John Sillitoe on behalf of the Wellcome Sanger Institute COVID-19 Surveillance Team
EPI_ISL_760825	Lighthouse Lab in Alderley Park	Wellcome Sanger Institute for the COVID-19 Genomics UK (COG-UK) Consortium	Jacquelyn Wynn, Mairead Hyland, The Lighthouse Lab in Alderley Park and Alex Alderton, Roberto Amato, Sonia Goncalves, Ewan Harrison, David K. Jackson, Ian Johnston, Dominic Kwiatkowski, Cordelia Langford, John Sillitoe on behalf of the Wellcome Sanger Institute COVID-19 Surveillance Team
EPI_ISL_760828, EPI_ISL_760829, EPI_ISL_760831	Lighthouse Lab in Glasgow	Wellcome Sanger Institute for the COVID-19 Genomics UK (COG-UK) Consortium	Harper VanSteenhouse, Yumi Kasai, David Gray, Carol Clugston, Anna Dominiczak and Alex Alderton, Roberto Amato, Sonia Goncalves, Ewan Harrison, David K. Jackson, Ian Johnston, Dominic Kwiatkowski, Cordelia Langford, John Sillitoe on behalf of the Wellcome Sanger Institute COVID-19 Surveillance Team
EPI_ISL_760832	Lighthouse Lab in Alderley Park	Wellcome Sanger Institute for the COVID-19 Genomics UK (COG-UK) Consortium	Jacquelyn Wynn, Mairead Hyland, The Lighthouse Lab in Alderley Park and Alex Alderton, Roberto Amato, Sonia Goncalves, Ewan Harrison, David K. Jackson, Ian Johnston, Dominic Kwiatkowski, Cordelia Langford, John Sillitoe on behalf of the Wellcome Sanger Institute COVID-19 Surveillance Team
EPI_ISL_760833	Lighthouse Lab in Cambridge	Wellcome Sanger Institute for the COVID-19 Genomics UK (COG-UK) Consortium	Rob Howes, The Lighthouse Lab in Cambridge and Alex Alderton, Roberto Amato, Sonia Goncalves, Ewan Harrison, David K. Jackson, Ian Johnston, Dominic Kwiatkowski, Cordelia Langford, John Sillitoe on behalf of the Wellcome Sanger Institute COVID-19 Surveillance Team
EPI_ISL_760834, EPI_ISL_760835, EPI_ISL_760836	Lighthouse Lab in Glasgow	Wellcome Sanger Institute for the COVID-19 Genomics UK (COG-UK) Consortium	Harper VanSteenhouse, Yumi Kasai, David Gray, Carol Clugston, Anna Dominiczak and Alex Alderton, Roberto Amato, Sonia Goncalves, Ewan Harrison, David K. Jackson, Ian Johnston, Dominic Kwiatkowski, Cordelia Langford, John Sillitoe on behalf of the Wellcome Sanger Institute COVID-19 Surveillance

			Team
EPI_ISL_760838	Lighthouse Lab in Alderley Park	Wellcome Sanger Institute for the COVID-19 Genomics UK (COG-UK) Consortium	Jacquelyn Wynn, Mairead Hyland, The Lighthouse Lab in Alderley Park and Alex Alderton, Roberto Amato, Sonia Goncalves, Ewan Harrison, David K. Jackson, Ian Johnston, Dominic Kwiatkowski, Cordelia Langford, John Sillitoe on behalf of the Wellcome Sanger Institute COVID-19 Surveillance Team
EPI_ISL_760840, EPI_ISL_760841	Lighthouse Lab in Glasgow	Wellcome Sanger Institute for the COVID-19 Genomics UK (COG-UK) Consortium	Harper VanSteenhouse, Yumi Kasai, David Gray, Carol Clugston, Anna Dominiczak and Alex Alderton, Roberto Amato, Sonia Goncalves, Ewan Harrison, David K. Jackson, Ian Johnston, Dominic Kwiatkowski, Cordelia Langford, John Sillitoe on behalf of the Wellcome Sanger Institute COVID-19 Surveillance Team
EPI_ISL_760842	Lighthouse Lab in Milton Keynes	Wellcome Sanger Institute for the COVID-19 Genomics UK (COG-UK) Consortium	The Lighthouse Lab in Milton Keynes and Alex Alderton, Roberto Amato, Sonia Goncalves, Ewan Harrison, David K. Jackson, Ian Johnston, Dominic Kwiatkowski, Cordelia Langford, John Sillitoe on behalf of the Wellcome Sanger Institute COVID-19 Surveillance Team
EPI_ISL_760843	Lighthouse Lab in Glasgow	Wellcome Sanger Institute for the COVID-19 Genomics UK (COG-UK) Consortium	Harper VanSteenhouse, Yumi Kasai, David Gray, Carol Clugston, Anna Dominiczak and Alex Alderton, Roberto Amato, Sonia Goncalves, Ewan Harrison, David K. Jackson, Ian Johnston, Dominic Kwiatkowski, Cordelia Langford, John Sillitoe on behalf of the Wellcome Sanger Institute COVID-19 Surveillance Team
EPI_ISL_760844	Lighthouse Lab in Milton Keynes	Wellcome Sanger Institute for the COVID-19 Genomics UK (COG-UK) Consortium	The Lighthouse Lab in Milton Keynes and Alex Alderton, Roberto Amato, Sonia Goncalves, Ewan Harrison, David K. Jackson, Ian Johnston, Dominic Kwiatkowski, Cordelia Langford, John Sillitoe on behalf of the Wellcome Sanger Institute COVID-19 Surveillance Team
EPI_ISL_760845, EPI_ISL_760847, EPI_ISL_760848, EPI_ISL_760849, EPI_ISL_760850, EPI_ISL_760851	Lighthouse Lab in Glasgow	Wellcome Sanger Institute for the COVID-19 Genomics UK (COG-UK) Consortium	Harper VanSteenhouse, Yumi Kasai, David Gray, Carol Clugston, Anna Dominiczak and Alex Alderton, Roberto Amato, Sonia Goncalves, Ewan Harrison, David K. Jackson, Ian Johnston, Dominic Kwiatkowski, Cordelia Langford, John Sillitoe on behalf of the Wellcome Sanger Institute COVID-19 Surveillance Team
EPI_ISL_760853	Lighthouse Lab in Alderley Park	Wellcome Sanger Institute for the COVID-19 Genomics UK (COG-UK) Consortium	Jacquelyn Wynn, Mairead Hyland, The Lighthouse Lab in Alderley Park and Alex Alderton, Roberto Amato, Sonia Goncalves, Ewan Harrison, David K. Jackson, Ian Johnston, Dominic Kwiatkowski, Cordelia Langford, John Sillitoe on behalf of the Wellcome Sanger Institute COVID-19 Surveillance Team
EPI_ISL_760854, EPI_ISL_760855, EPI_ISL_760856	Lighthouse Lab in Glasgow	Wellcome Sanger Institute for the COVID-19 Genomics UK (COG-UK) Consortium	Harper VanSteenhouse, Yumi Kasai, David Gray, Carol Clugston, Anna Dominiczak and Alex Alderton, Roberto Amato, Sonia Goncalves, Ewan Harrison, David K. Jackson, Ian Johnston, Dominic Kwiatkowski, Cordelia Langford, John Sillitoe on behalf of the Wellcome Sanger Institute COVID-19 Surveillance Team
EPI_ISL_760857	Lighthouse Lab in Alderley Park	Wellcome Sanger Institute for the COVID-19 Genomics UK (COG-UK) Consortium	Jacquelyn Wynn, Mairead Hyland, The Lighthouse Lab in Alderley Park and Alex Alderton, Roberto Amato, Sonia Goncalves, Ewan Harrison, David K. Jackson, Ian Johnston, Dominic Kwiatkowski, Cordelia Langford, John Sillitoe on behalf of the Wellcome Sanger Institute COVID-19 Surveillance Team
EPI_ISL_760858, EPI_ISL_760859	Lighthouse Lab in Cambridge	Wellcome Sanger Institute for the COVID-19 Genomics UK (COG-UK) Consortium	Rob Howes, The Lighthouse Lab in Cambridge and Alex Alderton, Roberto Amato, Sonia Goncalves, Ewan Harrison, David K. Jackson, Ian Johnston, Dominic Kwiatkowski, Cordelia Langford, John Sillitoe on behalf of the Wellcome Sanger Institute COVID-19 Surveillance Team
EPI_ISL_760861, EPI_ISL_760862	Lighthouse Lab in Alderley Park	Wellcome Sanger Institute for the COVID-19 Genomics UK (COG-UK) Consortium	Jacquelyn Wynn, Mairead Hyland, The Lighthouse Lab in Alderley Park and Alex Alderton, Roberto Amato, Sonia Goncalves, Ewan Harrison, David K. Jackson, Ian Johnston, Dominic Kwiatkowski, Cordelia Langford, John Sillitoe on behalf of the Wellcome Sanger Institute COVID-19 Surveillance Team
EPI_ISL_760863	Lighthouse Lab in Milton Keynes	Wellcome Sanger Institute for the COVID-19 Genomics UK (COG-UK) Consortium	The Lighthouse Lab in Milton Keynes and Alex Alderton, Roberto Amato, Sonia Goncalves, Ewan Harrison, David K. Jackson, Ian Johnston, Dominic Kwiatkowski, Cordelia Langford, John Sillitoe on behalf of the Wellcome Sanger Institute COVID-19 Surveillance Team
EPI_ISL_760865	Lighthouse Lab in Alderley Park	Wellcome Sanger Institute for the COVID-19 Genomics UK (COG-UK) Consortium	Jacquelyn Wynn, Mairead Hyland, The Lighthouse Lab in Alderley Park and Alex Alderton, Roberto Amato, Sonia Goncalves, Ewan Harrison, David K. Jackson, Ian Johnston, Dominic Kwiatkowski, Cordelia Langford, John Sillitoe on behalf of the Wellcome Sanger Institute COVID-19 Surveillance Team
EPI_ISL_760866	Lighthouse Lab in Glasgow	Wellcome Sanger Institute for the COVID-19 Genomics UK (COG-UK) Consortium	Harper VanSteenhouse, Yumi Kasai, David Gray, Carol Clugston, Anna Dominiczak and Alex Alderton, Roberto Amato, Sonia Goncalves, Ewan Harrison, David K. Jackson, Ian Johnston, Dominic Kwiatkowski, Cordelia Langford, John Sillitoe on behalf of the Wellcome Sanger Institute COVID-19 Surveillance Team
EPI_ISL_760867, EPI_ISL_760868	Lighthouse Lab in Alderley Park	Wellcome Sanger Institute for the COVID-19 Genomics UK (COG-UK) Consortium	Jacquelyn Wynn, Mairead Hyland, The Lighthouse Lab in Alderley Park and Alex Alderton, Roberto Amato, Sonia Goncalves, Ewan Harrison, David K. Jackson, Ian Johnston, Dominic Kwiatkowski, Cordelia Langford, John Sillitoe on behalf of the Wellcome Sanger Institute COVID-19 Surveillance Team
EPI_ISL_760869, EPI_ISL_760870, EPI_ISL_760872, EPI_ISL_760879, EPI_ISL_760880, EPI_ISL_760882	Lighthouse Lab in Glasgow	Wellcome Sanger Institute for the COVID-19 Genomics UK (COG-UK) Consortium	Harper VanSteenhouse, Yumi Kasai, David Gray, Carol Clugston, Anna Dominiczak and Alex Alderton, Roberto Amato, Sonia Goncalves, Ewan Harrison, David K. Jackson, Ian Johnston, Dominic Kwiatkowski, Cordelia Langford, John Sillitoe on behalf of the Wellcome Sanger Institute COVID-19 Surveillance Team
EPI_ISL_760884	Lighthouse Lab in Alderley Park	Wellcome Sanger Institute for the COVID-19 Genomics UK (COG-UK) Consortium	Jacquelyn Wynn, Mairead Hyland, The Lighthouse Lab in Alderley Park and Alex Alderton, Roberto Amato, Sonia Goncalves, Ewan Harrison, David K. Jackson, Ian Johnston, Dominic Kwiatkowski, Cordelia Langford, John Sillitoe on behalf of the Wellcome Sanger Institute COVID-19 Surveillance Team
EPI_ISL_760885, EPI_ISL_760886, EPI_ISL_760895	Lighthouse Lab in Glasgow	Wellcome Sanger Institute for the COVID-19 Genomics UK (COG-UK) Consortium	Harper VanSteenhouse, Yumi Kasai, David Gray, Carol Clugston, Anna Dominiczak and Alex Alderton, Roberto Amato, Sonia Goncalves, Ewan Harrison, David K. Jackson, Ian Johnston, Dominic Kwiatkowski, Cordelia Langford, John Sillitoe on behalf of the Wellcome Sanger Institute COVID-19 Surveillance Team
EPI_ISL_760896	Lighthouse Lab in Milton Keynes	Wellcome Sanger Institute for the COVID-19 Genomics UK (COG-UK) Consortium	The Lighthouse Lab in Milton Keynes and Alex Alderton, Roberto Amato, Sonia Goncalves, Ewan Harrison, David K. Jackson, Ian Johnston, Dominic Kwiatkowski, Cordelia Langford, John Sillitoe on behalf of the Wellcome Sanger Institute COVID-19 Surveillance Team
EPI_ISL_760897	Lighthouse Lab in Alderley Park	Wellcome Sanger Institute for the COVID-19 Genomics UK (COG-UK) Consortium	Jacquelyn Wynn, Mairead Hyland, The Lighthouse Lab in Alderley Park and Alex Alderton, Roberto Amato, Sonia Goncalves, Ewan Harrison, David K. Jackson, Ian Johnston, Dominic Kwiatkowski, Cordelia Langford, John Sillitoe on behalf of the Wellcome Sanger Institute COVID-19 Surveillance Team
EPI_ISL_760899, EPI_ISL_760901, EPI_ISL_760902	Lighthouse Lab in Glasgow	Wellcome Sanger Institute for the COVID-19 Genomics UK (COG-UK) Consortium	Harper VanSteenhouse, Yumi Kasai, David Gray, Carol Clugston, Anna Dominiczak and Alex Alderton, Roberto Amato, Sonia Goncalves, Ewan Harrison, David K. Jackson, Ian Johnston, Dominic Kwiatkowski, Cordelia Langford, John Sillitoe on behalf of the Wellcome Sanger Institute COVID-19 Surveillance Team
EPI_ISL_760905	Lighthouse Lab in Milton Keynes	Wellcome Sanger Institute for the COVID-19 Genomics UK (COG-UK) Consortium	The Lighthouse Lab in Milton Keynes and Alex Alderton, Roberto Amato, Sonia Goncalves, Ewan Harrison, David K. Jackson, Ian Johnston, Dominic Kwiatkowski, Cordelia Langford, John Sillitoe on behalf of the Wellcome Sanger Institute COVID-19 Surveillance Team
EPI_ISL_760906	Lighthouse Lab in Alderley Park	Wellcome Sanger Institute for the COVID-19 Genomics UK (COG-UK) Consortium	Jacquelyn Wynn, Mairead Hyland, The Lighthouse Lab in Alderley Park and Alex Alderton, Roberto Amato, Sonia Goncalves, Ewan Harrison, David K. Jackson, Ian Johnston, Dominic Kwiatkowski, Cordelia Langford, John Sillitoe on behalf of the Wellcome Sanger Institute COVID-19 Surveillance Team
EPI_ISL_760907, EPI_ISL_760909, EPI_ISL_760910, EPI_ISL_760911	Lighthouse Lab in Glasgow	Wellcome Sanger Institute for the COVID-19 Genomics UK (COG-UK) Consortium	Harper VanSteenhouse, Yumi Kasai, David Gray, Carol Clugston, Anna Dominiczak and Alex Alderton, Roberto Amato, Sonia Goncalves, Ewan Harrison, David K. Jackson, Ian Johnston, Dominic Kwiatkowski, Cordelia Langford, John Sillitoe on behalf of the Wellcome Sanger Institute COVID-19 Surveillance Team
EPI_ISL_760912	Lighthouse Lab in Alderley Park	Wellcome Sanger Institute for the COVID-19 Genomics UK (COG-UK) Consortium	Jacquelyn Wynn, Mairead Hyland, The Lighthouse Lab in Alderley Park and Alex Alderton, Roberto Amato, Sonia Goncalves, Ewan Harrison, David K. Jackson, Ian Johnston, Dominic Kwiatkowski, Cordelia Langford, John Sillitoe on behalf of the Wellcome Sanger Institute COVID-19 Surveillance Team
EPI_ISL_760913, EPI_ISL_760919	Lighthouse Lab in Glasgow	Wellcome Sanger Institute for the COVID-19 Genomics UK (COG-UK) Consortium	Harper VanSteenhouse, Yumi Kasai, David Gray, Carol Clugston, Anna Dominiczak and Alex Alderton, Roberto Amato, Sonia Goncalves, Ewan Harrison, David K. Jackson, Ian Johnston, Dominic Kwiatkowski, Cordelia Langford, John Sillitoe on behalf of the Wellcome Sanger Institute COVID-19 Surveillance Team
EPI_ISL_760921	Lighthouse Lab in Alderley Park	Wellcome Sanger Institute for the COVID-19 Genomics UK (COG-UK) Consortium	Jacquelyn Wynn, Mairead Hyland, The Lighthouse Lab in Alderley Park and Alex Alderton, Roberto Amato, Sonia Goncalves, Ewan Harrison, David K. Jackson, Ian Johnston, Dominic Kwiatkowski, Cordelia Langford, John Sillitoe on behalf of the Wellcome Sanger Institute COVID-19 Surveillance Team
EPI_ISL_760922	Lighthouse Lab in Cambridge	Wellcome Sanger Institute for the COVID-19 Genomics UK (COG-UK) Consortium	Rob Howes, The Lighthouse Lab in Cambridge and Alex Alderton, Roberto Amato, Sonia Goncalves, Ewan Harrison, David K. Jackson, Ian Johnston, Dominic Kwiatkowski, Cordelia Langford, John Sillitoe on behalf of the Wellcome Sanger Institute COVID-19 Surveillance Team
EPI_ISL_760925	Lighthouse Lab in Alderley Park	Wellcome Sanger Institute for the COVID-19 Genomics UK (COG-UK) Consortium	Jacquelyn Wynn, Mairead Hyland, The Lighthouse Lab in Alderley Park and Alex Alderton, Roberto Amato, Sonia Goncalves, Ewan Harrison, David K. Jackson, Ian Johnston, Dominic Kwiatkowski, Cordelia Langford, John Sillitoe on behalf of the Wellcome Sanger Institute COVID-19 Surveillance Team
EPI_ISL_760926, EPI_ISL_760927	Lighthouse Lab in Glasgow	Wellcome Sanger Institute for the COVID-19 Genomics UK (COG-UK) Consortium	Harper VanSteenhouse, Yumi Kasai, David Gray, Carol Clugston, Anna Dominiczak and Alex Alderton, Roberto Amato, Sonia Goncalves, Ewan Harrison, David K. Jackson, Ian Johnston, Dominic Kwiatkowski, Cordelia Langford, John Sillitoe on behalf of the Wellcome Sanger Institute COVID-19 Surveillance Team

EPI_ISL_760928, EPI_ISL_760929, EPI_ISL_760931	Lighthouse Lab in Alderley Park	Wellcome Sanger Institute for the COVID-19 Genomics UK (COG-UK) Consortium	Jacquelyn Wynn, Mairead Hyland, The Lighthouse Lab in Alderley Park and Alex Alderton, Roberto Amato, Sonia Goncalves, Ewan Harrison, David K. Jackson, Ian Johnston, Dominic Kwiatkowski, Cordelia Langford, John Sillitoe on behalf of the Wellcome Sanger Institute COVID-19 Surveillance Team
EPI_ISL_760932	Lighthouse Lab in Cambridge	Wellcome Sanger Institute for the COVID-19 Genomics UK (COG-UK) Consortium	Rob Howes, The Lighthouse Lab in Cambridge and Alex Alderton, Roberto Amato, Sonia Goncalves, Ewan Harrison, David K. Jackson, Ian Johnston, Dominic Kwiatkowski, Cordelia Langford, John Sillitoe on behalf of the Wellcome Sanger Institute COVID-19 Surveillance Team
EPI_ISL_760933	Lighthouse Lab in Alderley Park	Wellcome Sanger Institute for the COVID-19 Genomics UK (COG-UK) Consortium	Jacquelyn Wynn, Mairead Hyland, The Lighthouse Lab in Alderley Park and Alex Alderton, Roberto Amato, Sonia Goncalves, Ewan Harrison, David K. Jackson, Ian Johnston, Dominic Kwiatkowski, Cordelia Langford, John Sillitoe on behalf of the Wellcome Sanger Institute COVID-19 Surveillance Team
EPI_ISL_760934	Lighthouse Lab in Glasgow	Wellcome Sanger Institute for the COVID-19 Genomics UK (COG-UK) Consortium	Harper VanSteenhouse, Yumi Kasai, David Gray, Carol Clugston, Anna Dominiczak and Alex Alderton, Roberto Amato, Sonia Goncalves, Ewan Harrison, David K. Jackson, Ian Johnston, Dominic Kwiatkowski, Cordelia Langford, John Sillitoe on behalf of the Wellcome Sanger Institute COVID-19 Surveillance Team
EPI_ISL_760936	Lighthouse Lab in Cambridge	Wellcome Sanger Institute for the COVID-19 Genomics UK (COG-UK) Consortium	Rob Howes, The Lighthouse Lab in Cambridge and Alex Alderton, Roberto Amato, Sonia Goncalves, Ewan Harrison, David K. Jackson, Ian Johnston, Dominic Kwiatkowski, Cordelia Langford, John Sillitoe on behalf of the Wellcome Sanger Institute COVID-19 Surveillance Team
EPI_ISL_760937, EPI_ISL_760938, EPI_ISL_760942	Lighthouse Lab in Alderley Park	Wellcome Sanger Institute for the COVID-19 Genomics UK (COG-UK) Consortium	Jacquelyn Wynn, Mairead Hyland, The Lighthouse Lab in Alderley Park and Alex Alderton, Roberto Amato, Sonia Goncalves, Ewan Harrison, David K. Jackson, Ian Johnston, Dominic Kwiatkowski, Cordelia Langford, John Sillitoe on behalf of the Wellcome Sanger Institute COVID-19 Surveillance Team
EPI_ISL_760943	Lighthouse Lab in Milton Keynes	Wellcome Sanger Institute for the COVID-19 Genomics UK (COG-UK) Consortium	The Lighthouse Lab in Milton Keynes and Alex Alderton, Roberto Amato, Sonia Goncalves, Ewan Harrison, David K. Jackson, Ian Johnston, Dominic Kwiatkowski, Cordelia Langford, John Sillitoe on behalf of the Wellcome Sanger Institute COVID-19 Surveillance Team
EPI_ISL_760944	Lighthouse Lab in Glasgow	Wellcome Sanger Institute for the COVID-19 Genomics UK (COG-UK) Consortium	Harper VanSteenhouse, Yumi Kasai, David Gray, Carol Clugston, Anna Dominiczak and Alex Alderton, Roberto Amato, Sonia Goncalves, Ewan Harrison, David K. Jackson, Ian Johnston, Dominic Kwiatkowski, Cordelia Langford, John Sillitoe on behalf of the Wellcome Sanger Institute COVID-19 Surveillance Team
EPI_ISL_760945	Lighthouse Lab in Alderley Park	Wellcome Sanger Institute for the COVID-19 Genomics UK (COG-UK) Consortium	Jacquelyn Wynn, Mairead Hyland, The Lighthouse Lab in Alderley Park and Alex Alderton, Roberto Amato, Sonia Goncalves, Ewan Harrison, David K. Jackson, Ian Johnston, Dominic Kwiatkowski, Cordelia Langford, John Sillitoe on behalf of the Wellcome Sanger Institute COVID-19 Surveillance Team
EPI_ISL_760946, EPI_ISL_760947	Lighthouse Lab in Glasgow	Wellcome Sanger Institute for the COVID-19 Genomics UK (COG-UK) Consortium	Harper VanSteenhouse, Yumi Kasai, David Gray, Carol Clugston, Anna Dominiczak and Alex Alderton, Roberto Amato, Sonia Goncalves, Ewan Harrison, David K. Jackson, Ian Johnston, Dominic Kwiatkowski, Cordelia Langford, John Sillitoe on behalf of the Wellcome Sanger Institute COVID-19 Surveillance Team
EPI_ISL_760948, EPI_ISL_760950	Lighthouse Lab in Alderley Park	Wellcome Sanger Institute for the COVID-19 Genomics UK (COG-UK) Consortium	Jacquelyn Wynn, Mairead Hyland, The Lighthouse Lab in Alderley Park and Alex Alderton, Roberto Amato, Sonia Goncalves, Ewan Harrison, David K. Jackson, Ian Johnston, Dominic Kwiatkowski, Cordelia Langford, John Sillitoe on behalf of the Wellcome Sanger Institute COVID-19 Surveillance Team
EPI_ISL_760954, EPI_ISL_760959	Lighthouse Lab in Milton Keynes	Wellcome Sanger Institute for the COVID-19 Genomics UK (COG-UK) Consortium	The Lighthouse Lab in Milton Keynes and Alex Alderton, Roberto Amato, Sonia Goncalves, Ewan Harrison, David K. Jackson, Ian Johnston, Dominic Kwiatkowski, Cordelia Langford, John Sillitoe on behalf of the Wellcome Sanger Institute COVID-19 Surveillance Team
EPI_ISL_760960, EPI_ISL_760962	Lighthouse Lab in Alderley Park	Wellcome Sanger Institute for the COVID-19 Genomics UK (COG-UK) Consortium	Jacquelyn Wynn, Mairead Hyland, The Lighthouse Lab in Alderley Park and Alex Alderton, Roberto Amato, Sonia Goncalves, Ewan Harrison, David K. Jackson, Ian Johnston, Dominic Kwiatkowski, Cordelia Langford, John Sillitoe on behalf of the Wellcome Sanger Institute COVID-19 Surveillance Team
EPI_ISL_760963	Lighthouse Lab in Milton Keynes	Wellcome Sanger Institute for the COVID-19 Genomics UK (COG-UK) Consortium	The Lighthouse Lab in Milton Keynes and Alex Alderton, Roberto Amato, Sonia Goncalves, Ewan Harrison, David K. Jackson, Ian Johnston, Dominic Kwiatkowski, Cordelia Langford, John Sillitoe on behalf of the Wellcome Sanger Institute COVID-19 Surveillance Team
EPI_ISL_760964, EPI_ISL_760965, EPI_ISL_760966	Lighthouse Lab in Glasgow	Wellcome Sanger Institute for the COVID-19 Genomics UK (COG-UK) Consortium	Harper VanSteenhouse, Yumi Kasai, David Gray, Carol Clugston, Anna Dominiczak and Alex Alderton, Roberto Amato, Sonia Goncalves, Ewan Harrison, David K. Jackson, Ian Johnston, Dominic Kwiatkowski, Cordelia Langford, John Sillitoe on behalf of the Wellcome Sanger Institute COVID-19 Surveillance Team
EPI_ISL_760967, EPI_ISL_760968	Lighthouse Lab in Alderley Park	Wellcome Sanger Institute for the COVID-19 Genomics UK (COG-UK) Consortium	Jacquelyn Wynn, Mairead Hyland, The Lighthouse Lab in Alderley Park and Alex Alderton, Roberto Amato, Sonia Goncalves, Ewan Harrison, David K. Jackson, Ian Johnston, Dominic Kwiatkowski, Cordelia Langford, John Sillitoe on behalf of the Wellcome Sanger Institute COVID-19 Surveillance Team
EPI_ISL_760970	Lighthouse Lab in Glasgow	Wellcome Sanger Institute for the COVID-19 Genomics UK (COG-UK) Consortium	Harper VanSteenhouse, Yumi Kasai, David Gray, Carol Clugston, Anna Dominiczak and Alex Alderton, Roberto Amato, Sonia Goncalves, Ewan Harrison, David K. Jackson, Ian Johnston, Dominic Kwiatkowski, Cordelia Langford, John Sillitoe on behalf of the Wellcome Sanger Institute COVID-19 Surveillance Team
EPI_ISL_760971	Lighthouse Lab in Alderley Park	Wellcome Sanger Institute for the COVID-19 Genomics UK (COG-UK) Consortium	Jacquelyn Wynn, Mairead Hyland, The Lighthouse Lab in Alderley Park and Alex Alderton, Roberto Amato, Sonia Goncalves, Ewan Harrison, David K. Jackson, Ian Johnston, Dominic Kwiatkowski, Cordelia Langford, John Sillitoe on behalf of the Wellcome Sanger Institute COVID-19 Surveillance Team
EPI_ISL_760972, EPI_ISL_760973, EPI_ISL_760974	Lighthouse Lab in Glasgow	Wellcome Sanger Institute for the COVID-19 Genomics UK (COG-UK) Consortium	Harper VanSteenhouse, Yumi Kasai, David Gray, Carol Clugston, Anna Dominiczak and Alex Alderton, Roberto Amato, Sonia Goncalves, Ewan Harrison, David K. Jackson, Ian Johnston, Dominic Kwiatkowski, Cordelia Langford, John Sillitoe on behalf of the Wellcome Sanger Institute COVID-19 Surveillance Team
EPI_ISL_760976	Lighthouse Lab in Cambridge	Wellcome Sanger Institute for the COVID-19 Genomics UK (COG-UK) Consortium	Rob Howes, The Lighthouse Lab in Cambridge and Alex Alderton, Roberto Amato, Sonia Goncalves, Ewan Harrison, David K. Jackson, Ian Johnston, Dominic Kwiatkowski, Cordelia Langford, John Sillitoe on behalf of the Wellcome Sanger Institute COVID-19 Surveillance Team
EPI_ISL_760979	Lighthouse Lab in Alderley Park	Wellcome Sanger Institute for the COVID-19 Genomics UK (COG-UK) Consortium	Jacquelyn Wynn, Mairead Hyland, The Lighthouse Lab in Alderley Park and Alex Alderton, Roberto Amato, Sonia Goncalves, Ewan Harrison, David K. Jackson, Ian Johnston, Dominic Kwiatkowski, Cordelia Langford, John Sillitoe on behalf of the Wellcome Sanger Institute COVID-19 Surveillance Team
EPI_ISL_760981	Lighthouse Lab in Glasgow	Wellcome Sanger Institute for the COVID-19 Genomics UK (COG-UK) Consortium	Harper VanSteenhouse, Yumi Kasai, David Gray, Carol Clugston, Anna Dominiczak and Alex Alderton, Roberto Amato, Sonia Goncalves, Ewan Harrison, David K. Jackson, Ian Johnston, Dominic Kwiatkowski, Cordelia Langford, John Sillitoe on behalf of the Wellcome Sanger Institute COVID-19 Surveillance Team
EPI_ISL_760982	Lighthouse Lab in Alderley Park	Wellcome Sanger Institute for the COVID-19 Genomics UK (COG-UK) Consortium	Jacquelyn Wynn, Mairead Hyland, The Lighthouse Lab in Alderley Park and Alex Alderton, Roberto Amato, Sonia Goncalves, Ewan Harrison, David K. Jackson, Ian Johnston, Dominic Kwiatkowski, Cordelia Langford, John Sillitoe on behalf of the Wellcome Sanger Institute COVID-19 Surveillance Team
EPI_ISL_760983	Lighthouse Lab in Milton Keynes	Wellcome Sanger Institute for the COVID-19 Genomics UK (COG-UK) Consortium	The Lighthouse Lab in Milton Keynes and Alex Alderton, Roberto Amato, Sonia Goncalves, Ewan Harrison, David K. Jackson, Ian Johnston, Dominic Kwiatkowski, Cordelia Langford, John Sillitoe on behalf of the Wellcome Sanger Institute COVID-19 Surveillance Team
EPI_ISL_760984	Lighthouse Lab in Glasgow	Wellcome Sanger Institute for the COVID-19 Genomics UK (COG-UK) Consortium	Harper VanSteenhouse, Yumi Kasai, David Gray, Carol Clugston, Anna Dominiczak and Alex Alderton, Roberto Amato, Sonia Goncalves, Ewan Harrison, David K. Jackson, Ian Johnston, Dominic Kwiatkowski, Cordelia Langford, John Sillitoe on behalf of the Wellcome Sanger Institute COVID-19 Surveillance Team
EPI_ISL_760987	Lighthouse Lab in Milton Keynes	Wellcome Sanger Institute for the COVID-19 Genomics UK (COG-UK) Consortium	The Lighthouse Lab in Milton Keynes and Alex Alderton, Roberto Amato, Sonia Goncalves, Ewan Harrison, David K. Jackson, Ian Johnston, Dominic Kwiatkowski, Cordelia Langford, John Sillitoe on behalf of the Wellcome Sanger Institute COVID-19 Surveillance Team
EPI_ISL_760989	Lighthouse Lab in Alderley Park	Wellcome Sanger Institute for the COVID-19 Genomics UK (COG-UK) Consortium	Jacquelyn Wynn, Mairead Hyland, The Lighthouse Lab in Alderley Park and Alex Alderton, Roberto Amato, Sonia Goncalves, Ewan Harrison, David K. Jackson, Ian Johnston, Dominic Kwiatkowski, Cordelia Langford, John Sillitoe on behalf of the Wellcome Sanger Institute COVID-19 Surveillance Team
EPI_ISL_760990, EPI_ISL_760991, EPI_ISL_760992, EPI_ISL_760993	Lighthouse Lab in Glasgow	Wellcome Sanger Institute for the COVID-19 Genomics UK (COG-UK) Consortium	Harper VanSteenhouse, Yumi Kasai, David Gray, Carol Clugston, Anna Dominiczak and Alex Alderton, Roberto Amato, Sonia Goncalves, Ewan Harrison, David K. Jackson, Ian Johnston, Dominic Kwiatkowski, Cordelia Langford, John Sillitoe on behalf of the Wellcome Sanger Institute COVID-19 Surveillance Team
EPI_ISL_760994	Lighthouse Lab in Alderley Park	Wellcome Sanger Institute for the COVID-19 Genomics UK (COG-UK) Consortium	Jacquelyn Wynn, Mairead Hyland, The Lighthouse Lab in Alderley Park and Alex Alderton, Roberto Amato, Sonia Goncalves, Ewan Harrison, David K. Jackson, Ian Johnston, Dominic Kwiatkowski, Cordelia Langford, John Sillitoe on behalf of the Wellcome Sanger Institute COVID-19 Surveillance Team
EPI_ISL_760995, EPI_ISL_760996, EPI_ISL_760997, EPI_ISL_760999	Lighthouse Lab in Glasgow	Wellcome Sanger Institute for the COVID-19 Genomics UK (COG-UK) Consortium	Harper VanSteenhouse, Yumi Kasai, David Gray, Carol Clugston, Anna Dominiczak and Alex Alderton, Roberto Amato, Sonia Goncalves, Ewan Harrison, David K. Jackson, Ian Johnston, Dominic Kwiatkowski, Cordelia Langford, John Sillitoe on behalf of the Wellcome Sanger Institute COVID-19 Surveillance Team
EPI_ISL_761000, EPI_ISL_761001	Lighthouse Lab in Alderley Park	Wellcome Sanger Institute for the COVID-19 Genomics UK (COG-UK) Consortium	Jacquelyn Wynn, Mairead Hyland, The Lighthouse Lab in Alderley Park and Alex Alderton, Roberto Amato, Sonia Goncalves, Ewan Harrison, David K. Jackson, Ian Johnston, Dominic Kwiatkowski, Cordelia Langford, John Sillitoe on behalf of the Wellcome Sanger Institute COVID-19 Surveillance Team

EPI_ISL_761002	Lighthouse Lab in Milton Keynes	Wellcome Sanger Institute for the COVID-19 Genomics UK (COG-UK) Consortium	The Lighthouse Lab in Milton Keynes and Alex Alderton, Roberto Amato, Sonia Goncalves, Ewan Harrison, David K. Jackson, Ian Johnston, Dominic Kwiatkowski, Cordelia Langford, John Sillitoe on behalf of the Wellcome Sanger Institute COVID-19 Surveillance Team
EPI_ISL_761003, EPI_ISL_761004, EPI_ISL_761005, EPI_ISL_761006	Lighthouse Lab in Glasgow	Wellcome Sanger Institute for the COVID-19 Genomics UK (COG-UK) Consortium	Harper VanSteenhouse, Yumi Kasai, David Gray, Carol Clugston, Anna Dominiczak and Alex Alderton, Roberto Amato, Sonia Goncalves, Ewan Harrison, David K. Jackson, Ian Johnston, Dominic Kwiatkowski, Cordelia Langford, John Sillitoe on behalf of the Wellcome Sanger Institute COVID-19 Surveillance Team
EPI_ISL_761011	Lighthouse Lab in Alderley Park	Wellcome Sanger Institute for the COVID-19 Genomics UK (COG-UK) Consortium	Jacquelyn Wynn, Mairead Hyland, The Lighthouse Lab in Alderley Park and Alex Alderton, Roberto Amato, Sonia Goncalves, Ewan Harrison, David K. Jackson, Ian Johnston, Dominic Kwiatkowski, Cordelia Langford, John Sillitoe on behalf of the Wellcome Sanger Institute COVID-19 Surveillance Team
EPI_ISL_761012	Lighthouse Lab in Glasgow	Wellcome Sanger Institute for the COVID-19 Genomics UK (COG-UK) Consortium	Harper VanSteenhouse, Yumi Kasai, David Gray, Carol Clugston, Anna Dominiczak and Alex Alderton, Roberto Amato, Sonia Goncalves, Ewan Harrison, David K. Jackson, Ian Johnston, Dominic Kwiatkowski, Cordelia Langford, John Sillitoe on behalf of the Wellcome Sanger Institute COVID-19 Surveillance Team
EPI_ISL_761013	Lighthouse Lab in Alderley Park	Wellcome Sanger Institute for the COVID-19 Genomics UK (COG-UK) Consortium	Jacquelyn Wynn, Mairead Hyland, The Lighthouse Lab in Alderley Park and Alex Alderton, Roberto Amato, Sonia Goncalves, Ewan Harrison, David K. Jackson, Ian Johnston, Dominic Kwiatkowski, Cordelia Langford, John Sillitoe on behalf of the Wellcome Sanger Institute COVID-19 Surveillance Team
EPI_ISL_761014, EPI_ISL_761016	Lighthouse Lab in Glasgow	Wellcome Sanger Institute for the COVID-19 Genomics UK (COG-UK) Consortium	Harper VanSteenhouse, Yumi Kasai, David Gray, Carol Clugston, Anna Dominiczak and Alex Alderton, Roberto Amato, Sonia Goncalves, Ewan Harrison, David K. Jackson, Ian Johnston, Dominic Kwiatkowski, Cordelia Langford, John Sillitoe on behalf of the Wellcome Sanger Institute COVID-19 Surveillance Team
EPI_ISL_761017	Lighthouse Lab in Cambridge	Wellcome Sanger Institute for the COVID-19 Genomics UK (COG-UK) Consortium	Rob Howes, The Lighthouse Lab in Cambridge and Alex Alderton, Roberto Amato, Sonia Goncalves, Ewan Harrison, David K. Jackson, Ian Johnston, Dominic Kwiatkowski, Cordelia Langford, John Sillitoe on behalf of the Wellcome Sanger Institute COVID-19 Surveillance Team
EPI_ISL_761018, EPI_ISL_761019, EPI_ISL_761020	Lighthouse Lab in Glasgow	Wellcome Sanger Institute for the COVID-19 Genomics UK (COG-UK) Consortium	Harper VanSteenhouse, Yumi Kasai, David Gray, Carol Clugston, Anna Dominiczak and Alex Alderton, Roberto Amato, Sonia Goncalves, Ewan Harrison, David K. Jackson, Ian Johnston, Dominic Kwiatkowski, Cordelia Langford, John Sillitoe on behalf of the Wellcome Sanger Institute COVID-19 Surveillance Team
EPI_ISL_761021	Lighthouse Lab in Cambridge	Wellcome Sanger Institute for the COVID-19 Genomics UK (COG-UK) Consortium	Rob Howes, The Lighthouse Lab in Cambridge and Alex Alderton, Roberto Amato, Sonia Goncalves, Ewan Harrison, David K. Jackson, Ian Johnston, Dominic Kwiatkowski, Cordelia Langford, John Sillitoe on behalf of the Wellcome Sanger Institute COVID-19 Surveillance Team
EPI_ISL_761022	Lighthouse Lab in Alderley Park	Wellcome Sanger Institute for the COVID-19 Genomics UK (COG-UK) Consortium	Jacquelyn Wynn, Mairead Hyland, The Lighthouse Lab in Alderley Park and Alex Alderton, Roberto Amato, Sonia Goncalves, Ewan Harrison, David K. Jackson, Ian Johnston, Dominic Kwiatkowski, Cordelia Langford, John Sillitoe on behalf of the Wellcome Sanger Institute COVID-19 Surveillance Team
EPI_ISL_761023	Lighthouse Lab in Milton Keynes	Wellcome Sanger Institute for the COVID-19 Genomics UK (COG-UK) Consortium	The Lighthouse Lab in Milton Keynes and Alex Alderton, Roberto Amato, Sonia Goncalves, Ewan Harrison, David K. Jackson, Ian Johnston, Dominic Kwiatkowski, Cordelia Langford, John Sillitoe on behalf of the Wellcome Sanger Institute COVID-19 Surveillance Team
EPI_ISL_761024	Lighthouse Lab in Glasgow	Wellcome Sanger Institute for the COVID-19 Genomics UK (COG-UK) Consortium	Harper VanSteenhouse, Yumi Kasai, David Gray, Carol Clugston, Anna Dominiczak and Alex Alderton, Roberto Amato, Sonia Goncalves, Ewan Harrison, David K. Jackson, Ian Johnston, Dominic Kwiatkowski, Cordelia Langford, John Sillitoe on behalf of the Wellcome Sanger Institute COVID-19 Surveillance Team
EPI_ISL_761025, EPI_ISL_761027	Lighthouse Lab in Alderley Park	Wellcome Sanger Institute for the COVID-19 Genomics UK (COG-UK) Consortium	Jacquelyn Wynn, Mairead Hyland, The Lighthouse Lab in Alderley Park and Alex Alderton, Roberto Amato, Sonia Goncalves, Ewan Harrison, David K. Jackson, Ian Johnston, Dominic Kwiatkowski, Cordelia Langford, John Sillitoe on behalf of the Wellcome Sanger Institute COVID-19 Surveillance Team
EPI_ISL_761028	Lighthouse Lab in Glasgow	Wellcome Sanger Institute for the COVID-19 Genomics UK (COG-UK) Consortium	Harper VanSteenhouse, Yumi Kasai, David Gray, Carol Clugston, Anna Dominiczak and Alex Alderton, Roberto Amato, Sonia Goncalves, Ewan Harrison, David K. Jackson, Ian Johnston, Dominic Kwiatkowski, Cordelia Langford, John Sillitoe on behalf of the Wellcome Sanger Institute COVID-19 Surveillance Team
EPI_ISL_761029	Lighthouse Lab in Alderley Park	Wellcome Sanger Institute for the COVID-19 Genomics UK (COG-UK) Consortium	Jacquelyn Wynn, Mairead Hyland, The Lighthouse Lab in Alderley Park and Alex Alderton, Roberto Amato, Sonia Goncalves, Ewan Harrison, David K. Jackson, Ian Johnston, Dominic Kwiatkowski, Cordelia Langford, John Sillitoe on behalf of the Wellcome Sanger Institute COVID-19 Surveillance Team
EPI_ISL_761031, EPI_ISL_761034, EPI_ISL_761037, EPI_ISL_761038	Lighthouse Lab in Glasgow	Wellcome Sanger Institute for the COVID-19 Genomics UK (COG-UK) Consortium	Harper VanSteenhouse, Yumi Kasai, David Gray, Carol Clugston, Anna Dominiczak and Alex Alderton, Roberto Amato, Sonia Goncalves, Ewan Harrison, David K. Jackson, Ian Johnston, Dominic Kwiatkowski, Cordelia Langford, John Sillitoe on behalf of the Wellcome Sanger Institute COVID-19 Surveillance Team
EPI_ISL_761039	Lighthouse Lab in Milton Keynes	Wellcome Sanger Institute for the COVID-19 Genomics UK (COG-UK) Consortium	The Lighthouse Lab in Milton Keynes and Alex Alderton, Roberto Amato, Sonia Goncalves, Ewan Harrison, David K. Jackson, Ian Johnston, Dominic Kwiatkowski, Cordelia Langford, John Sillitoe on behalf of the Wellcome Sanger Institute COVID-19 Surveillance Team
EPI_ISL_761040	Lighthouse Lab in Alderley Park	Wellcome Sanger Institute for the COVID-19 Genomics UK (COG-UK) Consortium	Jacquelyn Wynn, Mairead Hyland, The Lighthouse Lab in Alderley Park and Alex Alderton, Roberto Amato, Sonia Goncalves, Ewan Harrison, David K. Jackson, Ian Johnston, Dominic Kwiatkowski, Cordelia Langford, John Sillitoe on behalf of the Wellcome Sanger Institute COVID-19 Surveillance Team
EPI_ISL_761041, EPI_ISL_761044, EPI_ISL_761045	Lighthouse Lab in Glasgow	Wellcome Sanger Institute for the COVID-19 Genomics UK (COG-UK) Consortium	Harper VanSteenhouse, Yumi Kasai, David Gray, Carol Clugston, Anna Dominiczak and Alex Alderton, Roberto Amato, Sonia Goncalves, Ewan Harrison, David K. Jackson, Ian Johnston, Dominic Kwiatkowski, Cordelia Langford, John Sillitoe on behalf of the Wellcome Sanger Institute COVID-19 Surveillance Team
EPI_ISL_761046	Lighthouse Lab in Alderley Park	Wellcome Sanger Institute for the COVID-19 Genomics UK (COG-UK) Consortium	Jacquelyn Wynn, Mairead Hyland, The Lighthouse Lab in Alderley Park and Alex Alderton, Roberto Amato, Sonia Goncalves, Ewan Harrison, David K. Jackson, Ian Johnston, Dominic Kwiatkowski, Cordelia Langford, John Sillitoe on behalf of the Wellcome Sanger Institute COVID-19 Surveillance Team
EPI_ISL_761047	Lighthouse Lab in Glasgow	Wellcome Sanger Institute for the COVID-19 Genomics UK (COG-UK) Consortium	Harper VanSteenhouse, Yumi Kasai, David Gray, Carol Clugston, Anna Dominiczak and Alex Alderton, Roberto Amato, Sonia Goncalves, Ewan Harrison, David K. Jackson, Ian Johnston, Dominic Kwiatkowski, Cordelia Langford, John Sillitoe on behalf of the Wellcome Sanger Institute COVID-19 Surveillance Team
EPI_ISL_761049	Lighthouse Lab in Alderley Park	Wellcome Sanger Institute for the COVID-19 Genomics UK (COG-UK) Consortium	Jacquelyn Wynn, Mairead Hyland, The Lighthouse Lab in Alderley Park and Alex Alderton, Roberto Amato, Sonia Goncalves, Ewan Harrison, David K. Jackson, Ian Johnston, Dominic Kwiatkowski, Cordelia Langford, John Sillitoe on behalf of the Wellcome Sanger Institute COVID-19 Surveillance Team
EPI_ISL_761050	Lighthouse Lab in Milton Keynes	Wellcome Sanger Institute for the COVID-19 Genomics UK (COG-UK) Consortium	The Lighthouse Lab in Milton Keynes and Alex Alderton, Roberto Amato, Sonia Goncalves, Ewan Harrison, David K. Jackson, Ian Johnston, Dominic Kwiatkowski, Cordelia Langford, John Sillitoe on behalf of the Wellcome Sanger Institute COVID-19 Surveillance Team
EPI_ISL_761051, EPI_ISL_761053	Lighthouse Lab in Glasgow	Wellcome Sanger Institute for the COVID-19 Genomics UK (COG-UK) Consortium	Harper VanSteenhouse, Yumi Kasai, David Gray, Carol Clugston, Anna Dominiczak and Alex Alderton, Roberto Amato, Sonia Goncalves, Ewan Harrison, David K. Jackson, Ian Johnston, Dominic Kwiatkowski, Cordelia Langford, John Sillitoe on behalf of the Wellcome Sanger Institute COVID-19 Surveillance Team
EPI_ISL_761054	Lighthouse Lab in Alderley Park	Wellcome Sanger Institute for the COVID-19 Genomics UK (COG-UK) Consortium	Jacquelyn Wynn, Mairead Hyland, The Lighthouse Lab in Alderley Park and Alex Alderton, Roberto Amato, Sonia Goncalves, Ewan Harrison, David K. Jackson, Ian Johnston, Dominic Kwiatkowski, Cordelia Langford, John Sillitoe on behalf of the Wellcome Sanger Institute COVID-19 Surveillance Team
EPI_ISL_761055	Lighthouse Lab in Glasgow	Wellcome Sanger Institute for the COVID-19 Genomics UK (COG-UK) Consortium	Harper VanSteenhouse, Yumi Kasai, David Gray, Carol Clugston, Anna Dominiczak and Alex Alderton, Roberto Amato, Sonia Goncalves, Ewan Harrison, David K. Jackson, Ian Johnston, Dominic Kwiatkowski, Cordelia Langford, John Sillitoe on behalf of the Wellcome Sanger Institute COVID-19 Surveillance Team
EPI_ISL_761056	Lighthouse Lab in Alderley Park	Wellcome Sanger Institute for the COVID-19 Genomics UK (COG-UK) Consortium	Jacquelyn Wynn, Mairead Hyland, The Lighthouse Lab in Alderley Park and Alex Alderton, Roberto Amato, Sonia Goncalves, Ewan Harrison, David K. Jackson, Ian Johnston, Dominic Kwiatkowski, Cordelia Langford, John Sillitoe on behalf of the Wellcome Sanger Institute COVID-19 Surveillance Team
EPI_ISL_761057	Lighthouse Lab in Cambridge	Wellcome Sanger Institute for the COVID-19 Genomics UK (COG-UK) Consortium	Rob Howes, The Lighthouse Lab in Cambridge and Alex Alderton, Roberto Amato, Sonia Goncalves, Ewan Harrison, David K. Jackson, Ian Johnston, Dominic Kwiatkowski, Cordelia Langford, John Sillitoe on behalf of the Wellcome Sanger Institute COVID-19 Surveillance Team
EPI_ISL_761060	Lighthouse Lab in Alderley Park	Wellcome Sanger Institute for the COVID-19 Genomics UK (COG-UK) Consortium	Jacquelyn Wynn, Mairead Hyland, The Lighthouse Lab in Alderley Park and Alex Alderton, Roberto Amato, Sonia Goncalves, Ewan Harrison, David K. Jackson, Ian Johnston, Dominic Kwiatkowski, Cordelia Langford, John Sillitoe on behalf of the Wellcome Sanger Institute COVID-19 Surveillance Team
EPI_ISL_761062, EPI_ISL_761063	Lighthouse Lab in Glasgow	Wellcome Sanger Institute for the COVID-19 Genomics UK (COG-UK) Consortium	Harper VanSteenhouse, Yumi Kasai, David Gray, Carol Clugston, Anna Dominiczak and Alex Alderton, Roberto Amato, Sonia Goncalves, Ewan Harrison, David K. Jackson, Ian Johnston, Dominic Kwiatkowski, Cordelia Langford, John Sillitoe on behalf of the Wellcome Sanger Institute COVID-19 Surveillance Team
EPI_ISL_761064, EPI_ISL_761065,	Lighthouse Lab in Alderley Park	Wellcome Sanger Institute for the COVID-19 Genomics UK	Jacquelyn Wynn, Mairead Hyland, The Lighthouse Lab in Alderley Park and Alex Alderton, Roberto Amato, Sonia Goncalves, Ewan Harrison, David K.

EPI_ISL_761066		(COG-UK) Consortium	Jackson, Ian Johnston, Dominic Kwiatkowski, Cordelia Langford, John Sillitoe on behalf of the Wellcome Sanger Institute COVID-19 Surveillance Team
EPI_ISL_761067, EPI_ISL_761070, EPI_ISL_761071, EPI_ISL_761072	Lighthouse Lab in Glasgow	Wellcome Sanger Institute for the COVID-19 Genomics UK (COG-UK) Consortium	Harper VanSteenhouse, Yumi Kasai, David Gray, Carol Clugston, Anna Dominiczak and Alex Alderton, Roberto Amato, Sonia Goncalves, Ewan Harrison, David K. Jackson, Ian Johnston, Dominic Kwiatkowski, Cordelia Langford, John Sillitoe on behalf of the Wellcome Sanger Institute COVID-19 Surveillance Team
EPI_ISL_761074, EPI_ISL_761075	Lighthouse Lab in Cambridge	Wellcome Sanger Institute for the COVID-19 Genomics UK (COG-UK) Consortium	Rob Howes, The Lighthouse Lab in Cambridge and Alex Alderton, Roberto Amato, Sonia Goncalves, Ewan Harrison, David K. Jackson, Ian Johnston, Dominic Kwiatkowski, Cordelia Langford, John Sillitoe on behalf of the Wellcome Sanger Institute COVID-19 Surveillance Team
EPI_ISL_761076, EPI_ISL_761077, EPI_ISL_761078	Lighthouse Lab in Glasgow	Wellcome Sanger Institute for the COVID-19 Genomics UK (COG-UK) Consortium	Harper VanSteenhouse, Yumi Kasai, David Gray, Carol Clugston, Anna Dominiczak and Alex Alderton, Roberto Amato, Sonia Goncalves, Ewan Harrison, David K. Jackson, Ian Johnston, Dominic Kwiatkowski, Cordelia Langford, John Sillitoe on behalf of the Wellcome Sanger Institute COVID-19 Surveillance Team
EPI_ISL_761079	Lighthouse Lab in Milton Keynes	Wellcome Sanger Institute for the COVID-19 Genomics UK (COG-UK) Consortium	The Lighthouse Lab in Milton Keynes and Alex Alderton, Roberto Amato, Sonia Goncalves, Ewan Harrison, David K. Jackson, Ian Johnston, Dominic Kwiatkowski, Cordelia Langford, John Sillitoe on behalf of the Wellcome Sanger Institute COVID-19 Surveillance Team
EPI_ISL_761080, EPI_ISL_761081, EPI_ISL_761082, EPI_ISL_761083, EPI_ISL_761084, EPI_ISL_761085, EPI_ISL_761086, EPI_ISL_761087, EPI_ISL_761090, EPI_ISL_761092	Lighthouse Lab in Alderley Park	Wellcome Sanger Institute for the COVID-19 Genomics UK (COG-UK) Consortium	Jacquelyn Wynn, Mairead Hyland, The Lighthouse Lab in Alderley Park and Alex Alderton, Roberto Amato, Sonia Goncalves, Ewan Harrison, David K. Jackson, Ian Johnston, Dominic Kwiatkowski, Cordelia Langford, John Sillitoe on behalf of the Wellcome Sanger Institute COVID-19 Surveillance Team
EPI_ISL_761094, EPI_ISL_761095	Lighthouse Lab in Glasgow	Wellcome Sanger Institute for the COVID-19 Genomics UK (COG-UK) Consortium	Harper VanSteenhouse, Yumi Kasai, David Gray, Carol Clugston, Anna Dominiczak and Alex Alderton, Roberto Amato, Sonia Goncalves, Ewan Harrison, David K. Jackson, Ian Johnston, Dominic Kwiatkowski, Cordelia Langford, John Sillitoe on behalf of the Wellcome Sanger Institute COVID-19 Surveillance Team
EPI_ISL_761096, EPI_ISL_761097, EPI_ISL_761098, EPI_ISL_761099, EPI_ISL_761100, EPI_ISL_761101, EPI_ISL_761102, EPI_ISL_761103, EPI_ISL_761106, EPI_ISL_761107, EPI_ISL_761110, EPI_ISL_761111			
see above	Lighthouse Lab in Alderley Park	Wellcome Sanger Institute for the COVID-19 Genomics UK (COG-UK) Consortium	Jacquelyn Wynn, Mairead Hyland, The Lighthouse Lab in Alderley Park and Alex Alderton, Roberto Amato, Sonia Goncalves, Ewan Harrison, David K. Jackson, Ian Johnston, Dominic Kwiatkowski, Cordelia Langford, John Sillitoe on behalf of the Wellcome Sanger Institute COVID-19 Surveillance Team
EPI_ISL_761114, EPI_ISL_761116	Lighthouse Lab in Glasgow	Wellcome Sanger Institute for the COVID-19 Genomics UK (COG-UK) Consortium	Harper VanSteenhouse, Yumi Kasai, David Gray, Carol Clugston, Anna Dominiczak and Alex Alderton, Roberto Amato, Sonia Goncalves, Ewan Harrison, David K. Jackson, Ian Johnston, Dominic Kwiatkowski, Cordelia Langford, John Sillitoe on behalf of the Wellcome Sanger Institute COVID-19 Surveillance Team
EPI_ISL_761117	Lighthouse Lab in Alderley Park	Wellcome Sanger Institute for the COVID-19 Genomics UK (COG-UK) Consortium	Jacquelyn Wynn, Mairead Hyland, The Lighthouse Lab in Alderley Park and Alex Alderton, Roberto Amato, Sonia Goncalves, Ewan Harrison, David K. Jackson, Ian Johnston, Dominic Kwiatkowski, Cordelia Langford, John Sillitoe on behalf of the Wellcome Sanger Institute COVID-19 Surveillance Team
EPI_ISL_761118	Lighthouse Lab in Glasgow	Wellcome Sanger Institute for the COVID-19 Genomics UK (COG-UK) Consortium	Harper VanSteenhouse, Yumi Kasai, David Gray, Carol Clugston, Anna Dominiczak and Alex Alderton, Roberto Amato, Sonia Goncalves, Ewan Harrison, David K. Jackson, Ian Johnston, Dominic Kwiatkowski, Cordelia Langford, John Sillitoe on behalf of the Wellcome Sanger Institute COVID-19 Surveillance Team
EPI_ISL_761119	Lighthouse Lab in Alderley Park	Wellcome Sanger Institute for the COVID-19 Genomics UK (COG-UK) Consortium	Jacquelyn Wynn, Mairead Hyland, The Lighthouse Lab in Alderley Park and Alex Alderton, Roberto Amato, Sonia Goncalves, Ewan Harrison, David K. Jackson, Ian Johnston, Dominic Kwiatkowski, Cordelia Langford, John Sillitoe on behalf of the Wellcome Sanger Institute COVID-19 Surveillance Team
EPI_ISL_761120	Lighthouse Lab in Glasgow	Wellcome Sanger Institute for the COVID-19 Genomics UK (COG-UK) Consortium	Harper VanSteenhouse, Yumi Kasai, David Gray, Carol Clugston, Anna Dominiczak and Alex Alderton, Roberto Amato, Sonia Goncalves, Ewan Harrison, David K. Jackson, Ian Johnston, Dominic Kwiatkowski, Cordelia Langford, John Sillitoe on behalf of the Wellcome Sanger Institute COVID-19 Surveillance Team
EPI_ISL_761124, EPI_ISL_761126, EPI_ISL_761127, EPI_ISL_761129, EPI_ISL_761130, EPI_ISL_761137, EPI_ISL_761138, EPI_ISL_761140, EPI_ISL_761141, EPI_ISL_761142, EPI_ISL_761144			
see above	Lighthouse Lab in Alderley Park	Wellcome Sanger Institute for the COVID-19 Genomics UK (COG-UK) Consortium	Jacquelyn Wynn, Mairead Hyland, The Lighthouse Lab in Alderley Park and Alex Alderton, Roberto Amato, Sonia Goncalves, Ewan Harrison, David K. Jackson, Ian Johnston, Dominic Kwiatkowski, Cordelia Langford, John Sillitoe on behalf of the Wellcome Sanger Institute COVID-19 Surveillance Team
EPI_ISL_761145	Lighthouse Lab in Glasgow	Wellcome Sanger Institute for the COVID-19 Genomics UK (COG-UK) Consortium	Harper VanSteenhouse, Yumi Kasai, David Gray, Carol Clugston, Anna Dominiczak and Alex Alderton, Roberto Amato, Sonia Goncalves, Ewan Harrison, David K. Jackson, Ian Johnston, Dominic Kwiatkowski, Cordelia Langford, John Sillitoe on behalf of the Wellcome Sanger Institute COVID-19 Surveillance Team
EPI_ISL_761147, EPI_ISL_761148, EPI_ISL_761149, EPI_ISL_761150, EPI_ISL_761151, EPI_ISL_761152, EPI_ISL_761153, EPI_ISL_761156	Lighthouse Lab in Alderley Park	Wellcome Sanger Institute for the COVID-19 Genomics UK (COG-UK) Consortium	Jacquelyn Wynn, Mairead Hyland, The Lighthouse Lab in Alderley Park and Alex Alderton, Roberto Amato, Sonia Goncalves, Ewan Harrison, David K. Jackson, Ian Johnston, Dominic Kwiatkowski, Cordelia Langford, John Sillitoe on behalf of the Wellcome Sanger Institute COVID-19 Surveillance Team
EPI_ISL_761157	Lighthouse Lab in Glasgow	Wellcome Sanger Institute for the COVID-19 Genomics UK (COG-UK) Consortium	Harper VanSteenhouse, Yumi Kasai, David Gray, Carol Clugston, Anna Dominiczak and Alex Alderton, Roberto Amato, Sonia Goncalves, Ewan Harrison, David K. Jackson, Ian Johnston, Dominic Kwiatkowski, Cordelia Langford, John Sillitoe on behalf of the Wellcome Sanger Institute COVID-19 Surveillance Team
EPI_ISL_761159, EPI_ISL_761160, EPI_ISL_761161, EPI_ISL_761164, EPI_ISL_761165, EPI_ISL_761167, EPI_ISL_761169, EPI_ISL_761171, EPI_ISL_761173, EPI_ISL_761174	Lighthouse Lab in Alderley Park	Wellcome Sanger Institute for the COVID-19 Genomics UK (COG-UK) Consortium	Jacquelyn Wynn, Mairead Hyland, The Lighthouse Lab in Alderley Park and Alex Alderton, Roberto Amato, Sonia Goncalves, Ewan Harrison, David K. Jackson, Ian Johnston, Dominic Kwiatkowski, Cordelia Langford, John Sillitoe on behalf of the Wellcome Sanger Institute COVID-19 Surveillance Team
EPI_ISL_761176	Lighthouse Lab in Glasgow	Wellcome Sanger Institute for the COVID-19 Genomics UK (COG-UK) Consortium	Harper VanSteenhouse, Yumi Kasai, David Gray, Carol Clugston, Anna Dominiczak and Alex Alderton, Roberto Amato, Sonia Goncalves, Ewan Harrison, David K. Jackson, Ian Johnston, Dominic Kwiatkowski, Cordelia Langford, John Sillitoe on behalf of the Wellcome Sanger Institute COVID-19 Surveillance Team
EPI_ISL_761177, EPI_ISL_761178, EPI_ISL_761179, EPI_ISL_761180, EPI_ISL_761181, EPI_ISL_761182, EPI_ISL_761183, EPI_ISL_761184, EPI_ISL_761185, EPI_ISL_761186	Lighthouse Lab in Alderley Park	Wellcome Sanger Institute for the COVID-19 Genomics UK (COG-UK) Consortium	Jacquelyn Wynn, Mairead Hyland, The Lighthouse Lab in Alderley Park and Alex Alderton, Roberto Amato, Sonia Goncalves, Ewan Harrison, David K. Jackson, Ian Johnston, Dominic Kwiatkowski, Cordelia Langford, John Sillitoe on behalf of the Wellcome Sanger Institute COVID-19 Surveillance Team
EPI_ISL_761187	Lighthouse Lab in Glasgow	Wellcome Sanger Institute for the COVID-19 Genomics UK (COG-UK) Consortium	Harper VanSteenhouse, Yumi Kasai, David Gray, Carol Clugston, Anna Dominiczak and Alex Alderton, Roberto Amato, Sonia Goncalves, Ewan Harrison, David K. Jackson, Ian Johnston, Dominic Kwiatkowski, Cordelia Langford, John Sillitoe on behalf of the Wellcome Sanger Institute COVID-19 Surveillance Team
EPI_ISL_761188, EPI_ISL_761189, EPI_ISL_761192	Lighthouse Lab in Alderley Park	Wellcome Sanger Institute for the COVID-19 Genomics UK (COG-UK) Consortium	Jacquelyn Wynn, Mairead Hyland, The Lighthouse Lab in Alderley Park and Alex Alderton, Roberto Amato, Sonia Goncalves, Ewan Harrison, David K. Jackson, Ian Johnston, Dominic Kwiatkowski, Cordelia Langford, John Sillitoe on behalf of the Wellcome Sanger Institute COVID-19 Surveillance Team
EPI_ISL_761193, EPI_ISL_761194	Lighthouse Lab in Glasgow	Wellcome Sanger Institute for the COVID-19 Genomics UK (COG-UK) Consortium	Harper VanSteenhouse, Yumi Kasai, David Gray, Carol Clugston, Anna Dominiczak and Alex Alderton, Roberto Amato, Sonia Goncalves, Ewan Harrison, David K. Jackson, Ian Johnston, Dominic Kwiatkowski, Cordelia Langford, John Sillitoe on behalf of the Wellcome Sanger Institute COVID-19 Surveillance Team
EPI_ISL_761198, EPI_ISL_761200, EPI_ISL_761201	Lighthouse Lab in Alderley Park	Wellcome Sanger Institute for the COVID-19 Genomics UK (COG-UK) Consortium	Jacquelyn Wynn, Mairead Hyland, The Lighthouse Lab in Alderley Park and Alex Alderton, Roberto Amato, Sonia Goncalves, Ewan Harrison, David K. Jackson, Ian Johnston, Dominic Kwiatkowski, Cordelia Langford, John Sillitoe on behalf of the Wellcome Sanger Institute COVID-19 Surveillance Team
EPI_ISL_761204	Lighthouse Lab in Glasgow	Wellcome Sanger Institute for the COVID-19 Genomics UK (COG-UK) Consortium	Harper VanSteenhouse, Yumi Kasai, David Gray, Carol Clugston, Anna Dominiczak and Alex Alderton, Roberto Amato, Sonia Goncalves, Ewan Harrison, David K. Jackson, Ian Johnston, Dominic Kwiatkowski, Cordelia Langford, John Sillitoe on behalf of the Wellcome Sanger Institute COVID-19 Surveillance Team

EPI_ISL_761205, EPI_ISL_761206	Lighthouse Lab in Alderley Park	Wellcome Sanger Institute for the COVID-19 Genomics UK (COG-UK) Consortium	Jacquelyn Wynn, Mairead Hyland, The Lighthouse Lab in Alderley Park and Alex Alderton, Roberto Amato, Sonia Goncalves, Ewan Harrison, David K. Jackson, Ian Johnston, Dominic Kwiatkowski, Cordelia Langford, John Sillitoe on behalf of the Wellcome Sanger Institute COVID-19 Surveillance Team
EPI_ISL_761207	Lighthouse Lab in Glasgow	Wellcome Sanger Institute for the COVID-19 Genomics UK (COG-UK) Consortium	Harper VanSteenhouse, Yumi Kasai, David Gray, Carol Clugston, Anna Dominiczak and Alex Alderton, Roberto Amato, Sonia Goncalves, Ewan Harrison, David K. Jackson, Ian Johnston, Dominic Kwiatkowski, Cordelia Langford, John Sillitoe on behalf of the Wellcome Sanger Institute COVID-19 Surveillance Team
EPI_ISL_761208	Lighthouse Lab in Alderley Park	Wellcome Sanger Institute for the COVID-19 Genomics UK (COG-UK) Consortium	Jacquelyn Wynn, Mairead Hyland, The Lighthouse Lab in Alderley Park and Alex Alderton, Roberto Amato, Sonia Goncalves, Ewan Harrison, David K. Jackson, Ian Johnston, Dominic Kwiatkowski, Cordelia Langford, John Sillitoe on behalf of the Wellcome Sanger Institute COVID-19 Surveillance Team
EPI_ISL_761209	Lighthouse Lab in Glasgow	Wellcome Sanger Institute for the COVID-19 Genomics UK (COG-UK) Consortium	Harper VanSteenhouse, Yumi Kasai, David Gray, Carol Clugston, Anna Dominiczak and Alex Alderton, Roberto Amato, Sonia Goncalves, Ewan Harrison, David K. Jackson, Ian Johnston, Dominic Kwiatkowski, Cordelia Langford, John Sillitoe on behalf of the Wellcome Sanger Institute COVID-19 Surveillance Team
EPI_ISL_761210, EPI_ISL_761213, EPI_ISL_761215, EPI_ISL_761217, EPI_ISL_761219, EPI_ISL_761220, EPI_ISL_761222, EPI_ISL_761223, EPI_ISL_761224	Lighthouse Lab in Alderley Park	Wellcome Sanger Institute for the COVID-19 Genomics UK (COG-UK) Consortium	Jacquelyn Wynn, Mairead Hyland, The Lighthouse Lab in Alderley Park and Alex Alderton, Roberto Amato, Sonia Goncalves, Ewan Harrison, David K. Jackson, Ian Johnston, Dominic Kwiatkowski, Cordelia Langford, John Sillitoe on behalf of the Wellcome Sanger Institute COVID-19 Surveillance Team
EPI_ISL_761225, EPI_ISL_761227, EPI_ISL_761229	Lighthouse Lab in Glasgow	Wellcome Sanger Institute for the COVID-19 Genomics UK (COG-UK) Consortium	Harper VanSteenhouse, Yumi Kasai, David Gray, Carol Clugston, Anna Dominiczak and Alex Alderton, Roberto Amato, Sonia Goncalves, Ewan Harrison, David K. Jackson, Ian Johnston, Dominic Kwiatkowski, Cordelia Langford, John Sillitoe on behalf of the Wellcome Sanger Institute COVID-19 Surveillance Team
EPI_ISL_761230, EPI_ISL_761231, EPI_ISL_761232, EPI_ISL_761234, EPI_ISL_761235, EPI_ISL_761236, EPI_ISL_761237, EPI_ISL_761238	Lighthouse Lab in Alderley Park	Wellcome Sanger Institute for the COVID-19 Genomics UK (COG-UK) Consortium	Jacquelyn Wynn, Mairead Hyland, The Lighthouse Lab in Alderley Park and Alex Alderton, Roberto Amato, Sonia Goncalves, Ewan Harrison, David K. Jackson, Ian Johnston, Dominic Kwiatkowski, Cordelia Langford, John Sillitoe on behalf of the Wellcome Sanger Institute COVID-19 Surveillance Team
EPI_ISL_761241	Lighthouse Lab in Glasgow	Wellcome Sanger Institute for the COVID-19 Genomics UK (COG-UK) Consortium	Harper VanSteenhouse, Yumi Kasai, David Gray, Carol Clugston, Anna Dominiczak and Alex Alderton, Roberto Amato, Sonia Goncalves, Ewan Harrison, David K. Jackson, Ian Johnston, Dominic Kwiatkowski, Cordelia Langford, John Sillitoe on behalf of the Wellcome Sanger Institute COVID-19 Surveillance Team
EPI_ISL_761243, EPI_ISL_761244, EPI_ISL_761245, EPI_ISL_761246, EPI_ISL_761247, EPI_ISL_761248, EPI_ISL_761249, EPI_ISL_761251	Lighthouse Lab in Alderley Park	Wellcome Sanger Institute for the COVID-19 Genomics UK (COG-UK) Consortium	Jacquelyn Wynn, Mairead Hyland, The Lighthouse Lab in Alderley Park and Alex Alderton, Roberto Amato, Sonia Goncalves, Ewan Harrison, David K. Jackson, Ian Johnston, Dominic Kwiatkowski, Cordelia Langford, John Sillitoe on behalf of the Wellcome Sanger Institute COVID-19 Surveillance Team
EPI_ISL_761252	Lighthouse Lab in Glasgow	Wellcome Sanger Institute for the COVID-19 Genomics UK (COG-UK) Consortium	Harper VanSteenhouse, Yumi Kasai, David Gray, Carol Clugston, Anna Dominiczak and Alex Alderton, Roberto Amato, Sonia Goncalves, Ewan Harrison, David K. Jackson, Ian Johnston, Dominic Kwiatkowski, Cordelia Langford, John Sillitoe on behalf of the Wellcome Sanger Institute COVID-19 Surveillance Team
EPI_ISL_761253, EPI_ISL_761254	Lighthouse Lab in Alderley Park	Wellcome Sanger Institute for the COVID-19 Genomics UK (COG-UK) Consortium	Jacquelyn Wynn, Mairead Hyland, The Lighthouse Lab in Alderley Park and Alex Alderton, Roberto Amato, Sonia Goncalves, Ewan Harrison, David K. Jackson, Ian Johnston, Dominic Kwiatkowski, Cordelia Langford, John Sillitoe on behalf of the Wellcome Sanger Institute COVID-19 Surveillance Team
EPI_ISL_761255, EPI_ISL_761259	Lighthouse Lab in Glasgow	Wellcome Sanger Institute for the COVID-19 Genomics UK (COG-UK) Consortium	Harper VanSteenhouse, Yumi Kasai, David Gray, Carol Clugston, Anna Dominiczak and Alex Alderton, Roberto Amato, Sonia Goncalves, Ewan Harrison, David K. Jackson, Ian Johnston, Dominic Kwiatkowski, Cordelia Langford, John Sillitoe on behalf of the Wellcome Sanger Institute COVID-19 Surveillance Team
EPI_ISL_761260, EPI_ISL_761262, EPI_ISL_761263, EPI_ISL_761264, EPI_ISL_761265	Lighthouse Lab in Alderley Park	Wellcome Sanger Institute for the COVID-19 Genomics UK (COG-UK) Consortium	Jacquelyn Wynn, Mairead Hyland, The Lighthouse Lab in Alderley Park and Alex Alderton, Roberto Amato, Sonia Goncalves, Ewan Harrison, David K. Jackson, Ian Johnston, Dominic Kwiatkowski, Cordelia Langford, John Sillitoe on behalf of the Wellcome Sanger Institute COVID-19 Surveillance Team
EPI_ISL_761266	Lighthouse Lab in Glasgow	Wellcome Sanger Institute for the COVID-19 Genomics UK (COG-UK) Consortium	Harper VanSteenhouse, Yumi Kasai, David Gray, Carol Clugston, Anna Dominiczak and Alex Alderton, Roberto Amato, Sonia Goncalves, Ewan Harrison, David K. Jackson, Ian Johnston, Dominic Kwiatkowski, Cordelia Langford, John Sillitoe on behalf of the Wellcome Sanger Institute COVID-19 Surveillance Team
EPI_ISL_761269, EPI_ISL_761271, EPI_ISL_761272, EPI_ISL_761274, EPI_ISL_761275	Lighthouse Lab in Alderley Park	Wellcome Sanger Institute for the COVID-19 Genomics UK (COG-UK) Consortium	Jacquelyn Wynn, Mairead Hyland, The Lighthouse Lab in Alderley Park and Alex Alderton, Roberto Amato, Sonia Goncalves, Ewan Harrison, David K. Jackson, Ian Johnston, Dominic Kwiatkowski, Cordelia Langford, John Sillitoe on behalf of the Wellcome Sanger Institute COVID-19 Surveillance Team
EPI_ISL_761276	Lighthouse Lab in Glasgow	Wellcome Sanger Institute for the COVID-19 Genomics UK (COG-UK) Consortium	Harper VanSteenhouse, Yumi Kasai, David Gray, Carol Clugston, Anna Dominiczak and Alex Alderton, Roberto Amato, Sonia Goncalves, Ewan Harrison, David K. Jackson, Ian Johnston, Dominic Kwiatkowski, Cordelia Langford, John Sillitoe on behalf of the Wellcome Sanger Institute COVID-19 Surveillance Team
EPI_ISL_761277	Lighthouse Lab in Alderley Park	Wellcome Sanger Institute for the COVID-19 Genomics UK (COG-UK) Consortium	Jacquelyn Wynn, Mairead Hyland, The Lighthouse Lab in Alderley Park and Alex Alderton, Roberto Amato, Sonia Goncalves, Ewan Harrison, David K. Jackson, Ian Johnston, Dominic Kwiatkowski, Cordelia Langford, John Sillitoe on behalf of the Wellcome Sanger Institute COVID-19 Surveillance Team
EPI_ISL_761278	Lighthouse Lab in Glasgow	Wellcome Sanger Institute for the COVID-19 Genomics UK (COG-UK) Consortium	Harper VanSteenhouse, Yumi Kasai, David Gray, Carol Clugston, Anna Dominiczak and Alex Alderton, Roberto Amato, Sonia Goncalves, Ewan Harrison, David K. Jackson, Ian Johnston, Dominic Kwiatkowski, Cordelia Langford, John Sillitoe on behalf of the Wellcome Sanger Institute COVID-19 Surveillance Team
EPI_ISL_761281, EPI_ISL_761282	Lighthouse Lab in Alderley Park	Wellcome Sanger Institute for the COVID-19 Genomics UK (COG-UK) Consortium	Jacquelyn Wynn, Mairead Hyland, The Lighthouse Lab in Alderley Park and Alex Alderton, Roberto Amato, Sonia Goncalves, Ewan Harrison, David K. Jackson, Ian Johnston, Dominic Kwiatkowski, Cordelia Langford, John Sillitoe on behalf of the Wellcome Sanger Institute COVID-19 Surveillance Team
EPI_ISL_761283	Lighthouse Lab in Glasgow	Wellcome Sanger Institute for the COVID-19 Genomics UK (COG-UK) Consortium	Harper VanSteenhouse, Yumi Kasai, David Gray, Carol Clugston, Anna Dominiczak and Alex Alderton, Roberto Amato, Sonia Goncalves, Ewan Harrison, David K. Jackson, Ian Johnston, Dominic Kwiatkowski, Cordelia Langford, John Sillitoe on behalf of the Wellcome Sanger Institute COVID-19 Surveillance Team
EPI_ISL_761284, EPI_ISL_761285, EPI_ISL_761286	Lighthouse Lab in Alderley Park	Wellcome Sanger Institute for the COVID-19 Genomics UK (COG-UK) Consortium	Jacquelyn Wynn, Mairead Hyland, The Lighthouse Lab in Alderley Park and Alex Alderton, Roberto Amato, Sonia Goncalves, Ewan Harrison, David K. Jackson, Ian Johnston, Dominic Kwiatkowski, Cordelia Langford, John Sillitoe on behalf of the Wellcome Sanger Institute COVID-19 Surveillance Team
EPI_ISL_761287	Lighthouse Lab in Glasgow	Wellcome Sanger Institute for the COVID-19 Genomics UK (COG-UK) Consortium	Harper VanSteenhouse, Yumi Kasai, David Gray, Carol Clugston, Anna Dominiczak and Alex Alderton, Roberto Amato, Sonia Goncalves, Ewan Harrison, David K. Jackson, Ian Johnston, Dominic Kwiatkowski, Cordelia Langford, John Sillitoe on behalf of the Wellcome Sanger Institute COVID-19 Surveillance Team
EPI_ISL_761288, EPI_ISL_761290, EPI_ISL_761291	Lighthouse Lab in Alderley Park	Wellcome Sanger Institute for the COVID-19 Genomics UK (COG-UK) Consortium	Jacquelyn Wynn, Mairead Hyland, The Lighthouse Lab in Alderley Park and Alex Alderton, Roberto Amato, Sonia Goncalves, Ewan Harrison, David K. Jackson, Ian Johnston, Dominic Kwiatkowski, Cordelia Langford, John Sillitoe on behalf of the Wellcome Sanger Institute COVID-19 Surveillance Team
EPI_ISL_761292, EPI_ISL_761293, EPI_ISL_761296	Lighthouse Lab in Glasgow	Wellcome Sanger Institute for the COVID-19 Genomics UK (COG-UK) Consortium	Harper VanSteenhouse, Yumi Kasai, David Gray, Carol Clugston, Anna Dominiczak and Alex Alderton, Roberto Amato, Sonia Goncalves, Ewan Harrison, David K. Jackson, Ian Johnston, Dominic Kwiatkowski, Cordelia Langford, John Sillitoe on behalf of the Wellcome Sanger Institute COVID-19 Surveillance Team
EPI_ISL_761297	Lighthouse Lab in Alderley Park	Wellcome Sanger Institute for the COVID-19 Genomics UK (COG-UK) Consortium	Jacquelyn Wynn, Mairead Hyland, The Lighthouse Lab in Alderley Park and Alex Alderton, Roberto Amato, Sonia Goncalves, Ewan Harrison, David K. Jackson, Ian Johnston, Dominic Kwiatkowski, Cordelia Langford, John Sillitoe on behalf of the Wellcome Sanger Institute COVID-19 Surveillance Team
EPI_ISL_761298	Lighthouse Lab in Glasgow	Wellcome Sanger Institute for the COVID-19 Genomics UK (COG-UK) Consortium	Harper VanSteenhouse, Yumi Kasai, David Gray, Carol Clugston, Anna Dominiczak and Alex Alderton, Roberto Amato, Sonia Goncalves, Ewan Harrison, David K. Jackson, Ian Johnston, Dominic Kwiatkowski, Cordelia Langford, John Sillitoe on behalf of the Wellcome Sanger Institute COVID-19 Surveillance Team

EPI_ISL_761299, EPI_ISL_761300, EPI_ISL_761301, EPI_ISL_761304, EPI_ISL_761305, EPI_ISL_761306	Lighthouse Lab in Alderley Park	Wellcome Sanger Institute for the COVID-19 Genomics UK (COG-UK) Consortium	Jacquelyn Wynn, Mairead Hyland, The Lighthouse Lab in Alderley Park and Alex Alderton, Roberto Amato, Sonia Goncalves, Ewan Harrison, David K. Jackson, Ian Johnston, Dominic Kwiatkowski, Cordelia Langford, John Sillitoe on behalf of the Wellcome Sanger Institute COVID-19 Surveillance Team
EPI_ISL_761307	Lighthouse Lab in Glasgow	Wellcome Sanger Institute for the COVID-19 Genomics UK (COG-UK) Consortium	Harper VanSteenhouse, Yumi Kasai, David Gray, Carol Clugston, Anna Dominiczak and Alex Alderton, Roberto Amato, Sonia Goncalves, Ewan Harrison, David K. Jackson, Ian Johnston, Dominic Kwiatkowski, Cordelia Langford, John Sillitoe on behalf of the Wellcome Sanger Institute COVID-19 Surveillance Team
EPI_ISL_761308, EPI_ISL_761309, EPI_ISL_761310, EPI_ISL_761316, EPI_ISL_761317, EPI_ISL_761319, EPI_ISL_761320, EPI_ISL_761321, EPI_ISL_761322, EPI_ISL_761323, EPI_ISL_761324, EPI_ISL_761325, EPI_ISL_761328, EPI_ISL_761329, EPI_ISL_761330, EPI_ISL_761332, EPI_ISL_761334, EPI_ISL_761335, EPI_ISL_761336			
see above	Lighthouse Lab in Alderley Park	Wellcome Sanger Institute for the COVID-19 Genomics UK (COG-UK) Consortium	Jacquelyn Wynn, Mairead Hyland, The Lighthouse Lab in Alderley Park and Alex Alderton, Roberto Amato, Sonia Goncalves, Ewan Harrison, David K. Jackson, Ian Johnston, Dominic Kwiatkowski, Cordelia Langford, John Sillitoe on behalf of the Wellcome Sanger Institute COVID-19 Surveillance Team
EPI_ISL_761338	Lighthouse Lab in Glasgow	Wellcome Sanger Institute for the COVID-19 Genomics UK (COG-UK) Consortium	Harper VanSteenhouse, Yumi Kasai, David Gray, Carol Clugston, Anna Dominiczak and Alex Alderton, Roberto Amato, Sonia Goncalves, Ewan Harrison, David K. Jackson, Ian Johnston, Dominic Kwiatkowski, Cordelia Langford, John Sillitoe on behalf of the Wellcome Sanger Institute COVID-19 Surveillance Team
EPI_ISL_761341, EPI_ISL_761342, EPI_ISL_761343, EPI_ISL_761344, EPI_ISL_761345, EPI_ISL_761346, EPI_ISL_761347, EPI_ISL_761349, EPI_ISL_761350	Lighthouse Lab in Alderley Park	Wellcome Sanger Institute for the COVID-19 Genomics UK (COG-UK) Consortium	Jacquelyn Wynn, Mairead Hyland, The Lighthouse Lab in Alderley Park and Alex Alderton, Roberto Amato, Sonia Goncalves, Ewan Harrison, David K. Jackson, Ian Johnston, Dominic Kwiatkowski, Cordelia Langford, John Sillitoe on behalf of the Wellcome Sanger Institute COVID-19 Surveillance Team
EPI_ISL_761351	Lighthouse Lab in Glasgow	Wellcome Sanger Institute for the COVID-19 Genomics UK (COG-UK) Consortium	Harper VanSteenhouse, Yumi Kasai, David Gray, Carol Clugston, Anna Dominiczak and Alex Alderton, Roberto Amato, Sonia Goncalves, Ewan Harrison, David K. Jackson, Ian Johnston, Dominic Kwiatkowski, Cordelia Langford, John Sillitoe on behalf of the Wellcome Sanger Institute COVID-19 Surveillance Team
EPI_ISL_761352	Lighthouse Lab in Alderley Park	Wellcome Sanger Institute for the COVID-19 Genomics UK (COG-UK) Consortium	Jacquelyn Wynn, Mairead Hyland, The Lighthouse Lab in Alderley Park and Alex Alderton, Roberto Amato, Sonia Goncalves, Ewan Harrison, David K. Jackson, Ian Johnston, Dominic Kwiatkowski, Cordelia Langford, John Sillitoe on behalf of the Wellcome Sanger Institute COVID-19 Surveillance Team
EPI_ISL_761353	Lighthouse Lab in Glasgow	Wellcome Sanger Institute for the COVID-19 Genomics UK (COG-UK) Consortium	Harper VanSteenhouse, Yumi Kasai, David Gray, Carol Clugston, Anna Dominiczak and Alex Alderton, Roberto Amato, Sonia Goncalves, Ewan Harrison, David K. Jackson, Ian Johnston, Dominic Kwiatkowski, Cordelia Langford, John Sillitoe on behalf of the Wellcome Sanger Institute COVID-19 Surveillance Team
EPI_ISL_761354, EPI_ISL_761356	Lighthouse Lab in Alderley Park	Wellcome Sanger Institute for the COVID-19 Genomics UK (COG-UK) Consortium	Jacquelyn Wynn, Mairead Hyland, The Lighthouse Lab in Alderley Park and Alex Alderton, Roberto Amato, Sonia Goncalves, Ewan Harrison, David K. Jackson, Ian Johnston, Dominic Kwiatkowski, Cordelia Langford, John Sillitoe on behalf of the Wellcome Sanger Institute COVID-19 Surveillance Team
EPI_ISL_761357, EPI_ISL_761358, EPI_ISL_761359, EPI_ISL_761360, EPI_ISL_761361, EPI_ISL_761362, EPI_ISL_761363, EPI_ISL_761364, EPI_ISL_761365, EPI_ISL_761366, EPI_ISL_761368, EPI_ISL_761369, EPI_ISL_761370, EPI_ISL_761371, EPI_ISL_761373, EPI_ISL_761374, EPI_ISL_761375, EPI_ISL_761376, EPI_ISL_761377, EPI_ISL_761378, EPI_ISL_761379, EPI_ISL_761382, EPI_ISL_761385, EPI_ISL_761386, EPI_ISL_761387, EPI_ISL_761388, EPI_ISL_761391, EPI_ISL_761392, EPI_ISL_761394, EPI_ISL_761395, EPI_ISL_761396, EPI_ISL_761397, EPI_ISL_761399, EPI_ISL_761400, EPI_ISL_761402, EPI_ISL_761403, EPI_ISL_761404, EPI_ISL_761405, EPI_ISL_761406, EPI_ISL_761407, EPI_ISL_761408, EPI_ISL_761409, EPI_ISL_761410, EPI_ISL_761411, EPI_ISL_761412, EPI_ISL_761413, EPI_ISL_761414, EPI_ISL_761415, EPI_ISL_761416, EPI_ISL_761417, EPI_ISL_761418, EPI_ISL_761419, EPI_ISL_761420, EPI_ISL_761421, EPI_ISL_761422, EPI_ISL_761423, EPI_ISL_761424, EPI_ISL_761425, EPI_ISL_761427, EPI_ISL_761428, EPI_ISL_761430, EPI_ISL_761431, EPI_ISL_761432, EPI_ISL_761433, EPI_ISL_761434, EPI_ISL_761435, EPI_ISL_761436, EPI_ISL_761437, EPI_ISL_761439, EPI_ISL_761440, EPI_ISL_761441, EPI_ISL_761442, EPI_ISL_761443, EPI_ISL_761444, EPI_ISL_761445, EPI_ISL_761446, EPI_ISL_761450, EPI_ISL_761451, EPI_ISL_761452, EPI_ISL_761453, EPI_ISL_761454, EPI_ISL_761455, EPI_ISL_761456, EPI_ISL_761457, EPI_ISL_761460, EPI_ISL_761461, EPI_ISL_761462, EPI_ISL_761463, EPI_ISL_761464, EPI_ISL_761465, EPI_ISL_761466, EPI_ISL_761467, EPI_ISL_761468, EPI_ISL_761469, EPI_ISL_761470, EPI_ISL_761471, EPI_ISL_761473, EPI_ISL_761474, EPI_ISL_761475, EPI_ISL_761476, EPI_ISL_761477, EPI_ISL_761478, EPI_ISL_761479, EPI_ISL_761482, EPI_ISL_761483, EPI_ISL_761484, EPI_ISL_761486, EPI_ISL_761487, EPI_ISL_761488, EPI_ISL_761489, EPI_ISL_761492, EPI_ISL_761493, EPI_ISL_761494, EPI_ISL_761495, EPI_ISL_761496, EPI_ISL_761497, EPI_ISL_761498, EPI_ISL_761499, EPI_ISL_761500, EPI_ISL_761501, EPI_ISL_761502, EPI_ISL_761503, EPI_ISL_761504, EPI_ISL_761506, EPI_ISL_761507, EPI_ISL_761508, EPI_ISL_761509, EPI_ISL_761510, EPI_ISL_761511, EPI_ISL_761513, EPI_ISL_761514, EPI_ISL_761518, EPI_ISL_761519, EPI_ISL_761520, EPI_ISL_761521, EPI_ISL_761523, EPI_ISL_761524, EPI_ISL_761525, EPI_ISL_761526, EPI_ISL_761527, EPI_ISL_761528, EPI_ISL_761529, EPI_ISL_761530, EPI_ISL_761531, EPI_ISL_761532, EPI_ISL_761533, EPI_ISL_761534, EPI_ISL_761535, EPI_ISL_761537, EPI_ISL_761538, EPI_ISL_761540, EPI_ISL_761541, EPI_ISL_761542, EPI_ISL_761543, EPI_ISL_761544, EPI_ISL_761545, EPI_ISL_761546, EPI_ISL_761547, EPI_ISL_761548, EPI_ISL_761550, EPI_ISL_761553, EPI_ISL_761554, EPI_ISL_761555, EPI_ISL_761556, EPI_ISL_761559, EPI_ISL_761560, EPI_ISL_761561, EPI_ISL_761563, EPI_ISL_761564, EPI_ISL_761565, EPI_ISL_761566, EPI_ISL_761567, EPI_ISL_761568, EPI_ISL_761569, EPI_ISL_761570, EPI_ISL_761571, EPI_ISL_761572, EPI_ISL_761573, EPI_ISL_761574, EPI_ISL_761575, EPI_ISL_761576, EPI_ISL_761577, EPI_ISL_761579, EPI_ISL_761580, EPI_ISL_761581, EPI_ISL_761582, EPI_ISL_761583, EPI_ISL_761584, EPI_ISL_761585, EPI_ISL_761586, EPI_ISL_761587, EPI_ISL_761588, EPI_ISL_761589, EPI_ISL_761590, EPI_ISL_761591, EPI_ISL_761592, EPI_ISL_761593, EPI_ISL_761594, EPI_ISL_761595, EPI_ISL_761597, EPI_ISL_761598, EPI_ISL_761599, EPI_ISL_761600, EPI_ISL_761602, EPI_ISL_761603, EPI_ISL_761605, EPI_ISL_761607, EPI_ISL_761608, EPI_ISL_761609, EPI_ISL_761611, EPI_ISL_761612, EPI_ISL_761613, EPI_ISL_761614, EPI_ISL_761615, EPI_ISL_761616, EPI_ISL_761617, EPI_ISL_761618, EPI_ISL_761619, EPI_ISL_761620, EPI_ISL_761621, EPI_ISL_761622, EPI_ISL_761623, EPI_ISL_761624, EPI_ISL_761626, EPI_ISL_761630, EPI_ISL_761631, EPI_ISL_761632, EPI_ISL_761634, EPI_ISL_761635, EPI_ISL_761636, EPI_ISL_761637, EPI_ISL_761639, EPI_ISL_761640, EPI_ISL_761642, EPI_ISL_761643, EPI_ISL_761644, EPI_ISL_761646, EPI_ISL_761647, EPI_ISL_761650, EPI_ISL_761651, EPI_ISL_761652, EPI_ISL_761655, EPI_ISL_761657, EPI_ISL_761658, EPI_ISL_761659, EPI_ISL_761660, EPI_ISL_761661, EPI_ISL_761662, EPI_ISL_761663, EPI_ISL_761664			
see above	Lighthouse Lab in Milton Keynes	Wellcome Sanger Institute for the COVID-19 Genomics UK (COG-UK) Consortium	The Lighthouse Lab in Milton Keynes and Alex Alderton, Roberto Amato, Sonia Goncalves, Ewan Harrison, David K. Jackson, Ian Johnston, Dominic Kwiatkowski, Cordelia Langford, John Sillitoe on behalf of the Wellcome Sanger Institute COVID-19 Surveillance Team
EPI_ISL_761665, EPI_ISL_761666, EPI_ISL_761667, EPI_ISL_761668, EPI_ISL_761669, EPI_ISL_761670, EPI_ISL_761671, EPI_ISL_761672, EPI_ISL_761673, EPI_ISL_761674, EPI_ISL_761675, EPI_ISL_761677, EPI_ISL_761678, EPI_ISL_761680, EPI_ISL_761681, EPI_ISL_761683, EPI_ISL_761686, EPI_ISL_761687, EPI_ISL_761688, EPI_ISL_761690, EPI_ISL_761691, EPI_ISL_761693, EPI_ISL_761695, EPI_ISL_761698, EPI_ISL_761700, EPI_ISL_761701, EPI_ISL_761705, EPI_ISL_761708, EPI_ISL_761709, EPI_ISL_761710, EPI_ISL_761711, EPI_ISL_761712, EPI_ISL_761713, EPI_ISL_761715, EPI_ISL_761718, EPI_ISL_761722, EPI_ISL_761724, EPI_ISL_761725, EPI_ISL_761726, EPI_ISL_761727, EPI_ISL_761728, EPI_ISL_761729, EPI_ISL_761730, EPI_ISL_761731, EPI_ISL_761732, EPI_ISL_761736, EPI_ISL_761737, EPI_ISL_761738, EPI_ISL_761740, EPI_ISL_761742, EPI_ISL_761743, EPI_ISL_761744, EPI_ISL_761745, EPI_ISL_761746, EPI_ISL_761747, EPI_ISL_761751, EPI_ISL_761752, EPI_ISL_761753, EPI_ISL_761756, EPI_ISL_761757, EPI_ISL_761758, EPI_ISL_761759, EPI_ISL_761760, EPI_ISL_761762, EPI_ISL_761763, EPI_ISL_761766, EPI_ISL_761768, EPI_ISL_761769, EPI_ISL_761774, EPI_ISL_761775, EPI_ISL_761777, EPI_ISL_761781, EPI_ISL_761782, EPI_ISL_761786, EPI_ISL_761787, EPI_ISL_761788, EPI_ISL_761789, EPI_ISL_761790, EPI_ISL_761791, EPI_ISL_761792, EPI_ISL_761794, EPI_ISL_761795, EPI_ISL_761796, EPI_ISL_761797, EPI_ISL_761798, EPI_ISL_761800, EPI_ISL_761802, EPI_ISL_761803, EPI_ISL_761805, EPI_ISL_761807, EPI_ISL_761808, EPI_ISL_761809, EPI_ISL_761810, EPI_ISL_761811, EPI_ISL_761812, EPI_ISL_761813, EPI_ISL_761815, EPI_ISL_761817, EPI_ISL_761818, EPI_ISL_761820, EPI_ISL_761821, EPI_ISL_761822, EPI_ISL_761824, EPI_ISL_761825, EPI_ISL_761826, EPI_ISL_761827, EPI_ISL_761829, EPI_ISL_761830, EPI_ISL_761831, EPI_ISL_761832, EPI_ISL_761835, EPI_ISL_761836, EPI_ISL_761837, EPI_ISL_761838, EPI_ISL_761839, EPI_ISL_761840, EPI_ISL_761841, EPI_ISL_761842, EPI_ISL_761843, EPI_ISL_761844, EPI_ISL_761845, EPI_ISL_761846, EPI_ISL_761849, EPI_ISL_761851, EPI_ISL_761852, EPI_ISL_761853, EPI_ISL_761854, EPI_ISL_761855, EPI_ISL_761858, EPI_ISL_761860, EPI_ISL_761861, EPI_ISL_761862, EPI_ISL_761867, EPI_ISL_761868, EPI_ISL_761869, EPI_ISL_761870, EPI_ISL_761871, EPI_ISL_761872, EPI_ISL_761873, EPI_ISL_761874, EPI_ISL_761875, EPI_ISL_761877, EPI_ISL_761878, EPI_ISL_761879, EPI_ISL_761880, EPI_ISL_761881, EPI_ISL_761885, EPI_ISL_761886, EPI_ISL_761887, EPI_ISL_761888, EPI_ISL_761889, EPI_ISL_761890, EPI_ISL_761891, EPI_ISL_761892, EPI_ISL_761894, EPI_ISL_761895, EPI_ISL_761899, EPI_ISL_761900, EPI_ISL_761901, EPI_ISL_761903, EPI_ISL_761904, EPI_ISL_761906, EPI_ISL_761907, EPI_ISL_761908, EPI_ISL_761909, EPI_ISL_761910, EPI_ISL_761912, EPI_ISL_761915, EPI_ISL_761916, EPI_ISL_761917, EPI_ISL_761919			
see above	Lighthouse Lab in Alderley Park	Wellcome Sanger Institute for the COVID-19 Genomics UK (COG-UK) Consortium	Jacquelyn Wynn, Mairead Hyland, The Lighthouse Lab in Alderley Park and Alex Alderton, Roberto Amato, Sonia Goncalves, Ewan Harrison, David K. Jackson, Ian Johnston, Dominic Kwiatkowski, Cordelia Langford, John Sillitoe on behalf of the Wellcome Sanger Institute COVID-19 Surveillance Team
EPI_ISL_761921, EPI_ISL_761923, EPI_ISL_761924, EPI_ISL_761925	Lighthouse Lab in Glasgow	Wellcome Sanger Institute for the COVID-19 Genomics UK (COG-UK) Consortium	Harper VanSteenhouse, Yumi Kasai, David Gray, Carol Clugston, Anna Dominiczak and Alex Alderton, Roberto Amato, Sonia Goncalves, Ewan Harrison, David K. Jackson, Ian Johnston, Dominic Kwiatkowski, Cordelia Langford, John Sillitoe on behalf of the Wellcome Sanger Institute COVID-19 Surveillance Team
EPI_ISL_761927	Lighthouse Lab in Cambridge	Wellcome Sanger Institute for the COVID-19 Genomics UK (COG-UK) Consortium	Rob Howes, The Lighthouse Lab in Cambridge and Alex Alderton, Roberto Amato, Sonia Goncalves, Ewan Harrison, David K. Jackson, Ian Johnston, Dominic Kwiatkowski, Cordelia Langford, John Sillitoe on behalf of the Wellcome Sanger Institute COVID-19 Surveillance Team
EPI_ISL_761928, EPI_ISL_761930, EPI_ISL_761931, EPI_ISL_761933	Lighthouse Lab in Glasgow	Wellcome Sanger Institute for the COVID-19 Genomics UK (COG-UK) Consortium	Harper VanSteenhouse, Yumi Kasai, David Gray, Carol Clugston, Anna Dominiczak and Alex Alderton, Roberto Amato, Sonia Goncalves, Ewan Harrison, David K. Jackson, Ian Johnston, Dominic Kwiatkowski, Cordelia Langford, John Sillitoe on behalf of the Wellcome Sanger Institute COVID-19 Surveillance Team
EPI_ISL_761935	Lighthouse Lab in Cambridge	Wellcome Sanger Institute for the COVID-19 Genomics UK (COG-UK) Consortium	Rob Howes, The Lighthouse Lab in Cambridge and Alex Alderton, Roberto Amato, Sonia Goncalves, Ewan Harrison, David K. Jackson, Ian Johnston, Dominic Kwiatkowski, Cordelia Langford, John Sillitoe on behalf of the Wellcome Sanger Institute COVID-19 Surveillance Team
EPI_ISL_761936, EPI_ISL_761938	Lighthouse Lab in Glasgow	Wellcome Sanger Institute for the COVID-19 Genomics UK (COG-UK) Consortium	Harper VanSteenhouse, Yumi Kasai, David Gray, Carol Clugston, Anna Dominiczak and Alex Alderton, Roberto Amato, Sonia Goncalves, Ewan Harrison, David K. Jackson, Ian Johnston, Dominic Kwiatkowski, Cordelia Langford, John Sillitoe on behalf of the Wellcome Sanger Institute COVID-19 Surveillance Team
EPI_ISL_761939	Lighthouse Lab in Cambridge	Wellcome Sanger Institute for the COVID-19 Genomics UK (COG-UK) Consortium	Rob Howes, The Lighthouse Lab in Cambridge and Alex Alderton, Roberto Amato, Sonia Goncalves, Ewan Harrison, David K. Jackson, Ian Johnston, Dominic Kwiatkowski, Cordelia Langford, John Sillitoe on behalf of the Wellcome Sanger Institute COVID-19 Surveillance Team
EPI_ISL_761940, EPI_ISL_761941, EPI_ISL_761942, EPI_ISL_761943, EPI_ISL_761944, EPI_ISL_761945	Lighthouse Lab in Glasgow	Wellcome Sanger Institute for the COVID-19 Genomics UK (COG-UK) Consortium	Harper VanSteenhouse, Yumi Kasai, David Gray, Carol Clugston, Anna Dominiczak and Alex Alderton, Roberto Amato, Sonia Goncalves, Ewan Harrison, David K. Jackson, Ian Johnston, Dominic Kwiatkowski, Cordelia Langford, John Sillitoe on behalf of the Wellcome Sanger Institute COVID-19 Surveillance Team
EPI_ISL_761946	Lighthouse Lab in Cambridge	Wellcome Sanger Institute for the COVID-19 Genomics UK (COG-UK) Consortium	Rob Howes, The Lighthouse Lab in Cambridge and Alex Alderton, Roberto Amato, Sonia Goncalves, Ewan Harrison, David K. Jackson, Ian Johnston, Dominic Kwiatkowski, Cordelia Langford, John Sillitoe on behalf of the Wellcome Sanger Institute COVID-19 Surveillance Team

EPI_ISL_761947, EPI_ISL_761948, EPI_ISL_761949, EPI_ISL_761950, EPI_ISL_761951	Lighthouse Lab in Glasgow	Wellcome Sanger Institute for the COVID-19 Genomics UK (COG-UK) Consortium	Harper VanSteenhouse, Yumi Kasai, David Gray, Carol Clugston, Anna Dominiczak and Alex Alderton, Roberto Amato, Sonia Goncalves, Ewan Harrison, David K. Jackson, Ian Johnston, Dominic Kwiatkowski, Cordelia Langford, John Sillitoe on behalf of the Wellcome Sanger Institute COVID-19 Surveillance Team
EPI_ISL_761952, EPI_ISL_761953	Lighthouse Lab in Cambridge	Wellcome Sanger Institute for the COVID-19 Genomics UK (COG-UK) Consortium	Rob Howes, The Lighthouse Lab in Cambridge and Alex Alderton, Roberto Amato, Sonia Goncalves, Ewan Harrison, David K. Jackson, Ian Johnston, Dominic Kwiatkowski, Cordelia Langford, John Sillitoe on behalf of the Wellcome Sanger Institute COVID-19 Surveillance Team
EPI_ISL_761957, EPI_ISL_761958, EPI_ISL_761959, EPI_ISL_761960	Lighthouse Lab in Glasgow	Wellcome Sanger Institute for the COVID-19 Genomics UK (COG-UK) Consortium	Harper VanSteenhouse, Yumi Kasai, David Gray, Carol Clugston, Anna Dominiczak and Alex Alderton, Roberto Amato, Sonia Goncalves, Ewan Harrison, David K. Jackson, Ian Johnston, Dominic Kwiatkowski, Cordelia Langford, John Sillitoe on behalf of the Wellcome Sanger Institute COVID-19 Surveillance Team
EPI_ISL_761961	Lighthouse Lab in Cambridge	Wellcome Sanger Institute for the COVID-19 Genomics UK (COG-UK) Consortium	Rob Howes, The Lighthouse Lab in Cambridge and Alex Alderton, Roberto Amato, Sonia Goncalves, Ewan Harrison, David K. Jackson, Ian Johnston, Dominic Kwiatkowski, Cordelia Langford, John Sillitoe on behalf of the Wellcome Sanger Institute COVID-19 Surveillance Team
EPI_ISL_761962, EPI_ISL_761964, EPI_ISL_761965, EPI_ISL_761966	Lighthouse Lab in Glasgow	Wellcome Sanger Institute for the COVID-19 Genomics UK (COG-UK) Consortium	Harper VanSteenhouse, Yumi Kasai, David Gray, Carol Clugston, Anna Dominiczak and Alex Alderton, Roberto Amato, Sonia Goncalves, Ewan Harrison, David K. Jackson, Ian Johnston, Dominic Kwiatkowski, Cordelia Langford, John Sillitoe on behalf of the Wellcome Sanger Institute COVID-19 Surveillance Team
EPI_ISL_761967, EPI_ISL_761968	Lighthouse Lab in Cambridge	Wellcome Sanger Institute for the COVID-19 Genomics UK (COG-UK) Consortium	Rob Howes, The Lighthouse Lab in Cambridge and Alex Alderton, Roberto Amato, Sonia Goncalves, Ewan Harrison, David K. Jackson, Ian Johnston, Dominic Kwiatkowski, Cordelia Langford, John Sillitoe on behalf of the Wellcome Sanger Institute COVID-19 Surveillance Team
EPI_ISL_761969	Lighthouse Lab in Glasgow	Wellcome Sanger Institute for the COVID-19 Genomics UK (COG-UK) Consortium	Harper VanSteenhouse, Yumi Kasai, David Gray, Carol Clugston, Anna Dominiczak and Alex Alderton, Roberto Amato, Sonia Goncalves, Ewan Harrison, David K. Jackson, Ian Johnston, Dominic Kwiatkowski, Cordelia Langford, John Sillitoe on behalf of the Wellcome Sanger Institute COVID-19 Surveillance Team
EPI_ISL_761970	Lighthouse Lab in Cambridge	Wellcome Sanger Institute for the COVID-19 Genomics UK (COG-UK) Consortium	Rob Howes, The Lighthouse Lab in Cambridge and Alex Alderton, Roberto Amato, Sonia Goncalves, Ewan Harrison, David K. Jackson, Ian Johnston, Dominic Kwiatkowski, Cordelia Langford, John Sillitoe on behalf of the Wellcome Sanger Institute COVID-19 Surveillance Team
EPI_ISL_761972, EPI_ISL_761974, EPI_ISL_761975, EPI_ISL_761978	Lighthouse Lab in Glasgow	Wellcome Sanger Institute for the COVID-19 Genomics UK (COG-UK) Consortium	Harper VanSteenhouse, Yumi Kasai, David Gray, Carol Clugston, Anna Dominiczak and Alex Alderton, Roberto Amato, Sonia Goncalves, Ewan Harrison, David K. Jackson, Ian Johnston, Dominic Kwiatkowski, Cordelia Langford, John Sillitoe on behalf of the Wellcome Sanger Institute COVID-19 Surveillance Team
EPI_ISL_761979, EPI_ISL_761981	Lighthouse Lab in Cambridge	Wellcome Sanger Institute for the COVID-19 Genomics UK (COG-UK) Consortium	Rob Howes, The Lighthouse Lab in Cambridge and Alex Alderton, Roberto Amato, Sonia Goncalves, Ewan Harrison, David K. Jackson, Ian Johnston, Dominic Kwiatkowski, Cordelia Langford, John Sillitoe on behalf of the Wellcome Sanger Institute COVID-19 Surveillance Team
EPI_ISL_761982, EPI_ISL_761983, EPI_ISL_761984, EPI_ISL_761986, EPI_ISL_761988, EPI_ISL_761989, EPI_ISL_761990, EPI_ISL_761991, EPI_ISL_761992, EPI_ISL_761993	Lighthouse Lab in Glasgow	Wellcome Sanger Institute for the COVID-19 Genomics UK (COG-UK) Consortium	Harper VanSteenhouse, Yumi Kasai, David Gray, Carol Clugston, Anna Dominiczak and Alex Alderton, Roberto Amato, Sonia Goncalves, Ewan Harrison, David K. Jackson, Ian Johnston, Dominic Kwiatkowski, Cordelia Langford, John Sillitoe on behalf of the Wellcome Sanger Institute COVID-19 Surveillance Team
EPI_ISL_761994	Lighthouse Lab in Cambridge	Wellcome Sanger Institute for the COVID-19 Genomics UK (COG-UK) Consortium	Rob Howes, The Lighthouse Lab in Cambridge and Alex Alderton, Roberto Amato, Sonia Goncalves, Ewan Harrison, David K. Jackson, Ian Johnston, Dominic Kwiatkowski, Cordelia Langford, John Sillitoe on behalf of the Wellcome Sanger Institute COVID-19 Surveillance Team
EPI_ISL_761996	Lighthouse Lab in Glasgow	Wellcome Sanger Institute for the COVID-19 Genomics UK (COG-UK) Consortium	Harper VanSteenhouse, Yumi Kasai, David Gray, Carol Clugston, Anna Dominiczak and Alex Alderton, Roberto Amato, Sonia Goncalves, Ewan Harrison, David K. Jackson, Ian Johnston, Dominic Kwiatkowski, Cordelia Langford, John Sillitoe on behalf of the Wellcome Sanger Institute COVID-19 Surveillance Team
EPI_ISL_761997	Lighthouse Lab in Cambridge	Wellcome Sanger Institute for the COVID-19 Genomics UK (COG-UK) Consortium	Rob Howes, The Lighthouse Lab in Cambridge and Alex Alderton, Roberto Amato, Sonia Goncalves, Ewan Harrison, David K. Jackson, Ian Johnston, Dominic Kwiatkowski, Cordelia Langford, John Sillitoe on behalf of the Wellcome Sanger Institute COVID-19 Surveillance Team
EPI_ISL_761998, EPI_ISL_762001, EPI_ISL_762004, EPI_ISL_762005, EPI_ISL_762006	Lighthouse Lab in Glasgow	Wellcome Sanger Institute for the COVID-19 Genomics UK (COG-UK) Consortium	Harper VanSteenhouse, Yumi Kasai, David Gray, Carol Clugston, Anna Dominiczak and Alex Alderton, Roberto Amato, Sonia Goncalves, Ewan Harrison, David K. Jackson, Ian Johnston, Dominic Kwiatkowski, Cordelia Langford, John Sillitoe on behalf of the Wellcome Sanger Institute COVID-19 Surveillance Team
EPI_ISL_762007, EPI_ISL_762008	Lighthouse Lab in Cambridge	Wellcome Sanger Institute for the COVID-19 Genomics UK (COG-UK) Consortium	Rob Howes, The Lighthouse Lab in Cambridge and Alex Alderton, Roberto Amato, Sonia Goncalves, Ewan Harrison, David K. Jackson, Ian Johnston, Dominic Kwiatkowski, Cordelia Langford, John Sillitoe on behalf of the Wellcome Sanger Institute COVID-19 Surveillance Team
EPI_ISL_762010, EPI_ISL_762011, EPI_ISL_762013	Lighthouse Lab in Glasgow	Wellcome Sanger Institute for the COVID-19 Genomics UK (COG-UK) Consortium	Harper VanSteenhouse, Yumi Kasai, David Gray, Carol Clugston, Anna Dominiczak and Alex Alderton, Roberto Amato, Sonia Goncalves, Ewan Harrison, David K. Jackson, Ian Johnston, Dominic Kwiatkowski, Cordelia Langford, John Sillitoe on behalf of the Wellcome Sanger Institute COVID-19 Surveillance Team
EPI_ISL_762014, EPI_ISL_762016, EPI_ISL_762018	Lighthouse Lab in Cambridge	Wellcome Sanger Institute for the COVID-19 Genomics UK (COG-UK) Consortium	Rob Howes, The Lighthouse Lab in Cambridge and Alex Alderton, Roberto Amato, Sonia Goncalves, Ewan Harrison, David K. Jackson, Ian Johnston, Dominic Kwiatkowski, Cordelia Langford, John Sillitoe on behalf of the Wellcome Sanger Institute COVID-19 Surveillance Team
EPI_ISL_762021	Lighthouse Lab in Glasgow	Wellcome Sanger Institute for the COVID-19 Genomics UK (COG-UK) Consortium	Harper VanSteenhouse, Yumi Kasai, David Gray, Carol Clugston, Anna Dominiczak and Alex Alderton, Roberto Amato, Sonia Goncalves, Ewan Harrison, David K. Jackson, Ian Johnston, Dominic Kwiatkowski, Cordelia Langford, John Sillitoe on behalf of the Wellcome Sanger Institute COVID-19 Surveillance Team
EPI_ISL_762024, EPI_ISL_762027, EPI_ISL_762029, EPI_ISL_762030	Lighthouse Lab in Cambridge	Wellcome Sanger Institute for the COVID-19 Genomics UK (COG-UK) Consortium	Rob Howes, The Lighthouse Lab in Cambridge and Alex Alderton, Roberto Amato, Sonia Goncalves, Ewan Harrison, David K. Jackson, Ian Johnston, Dominic Kwiatkowski, Cordelia Langford, John Sillitoe on behalf of the Wellcome Sanger Institute COVID-19 Surveillance Team
EPI_ISL_762031, EPI_ISL_762032, EPI_ISL_762034, EPI_ISL_762035	Lighthouse Lab in Glasgow	Wellcome Sanger Institute for the COVID-19 Genomics UK (COG-UK) Consortium	Harper VanSteenhouse, Yumi Kasai, David Gray, Carol Clugston, Anna Dominiczak and Alex Alderton, Roberto Amato, Sonia Goncalves, Ewan Harrison, David K. Jackson, Ian Johnston, Dominic Kwiatkowski, Cordelia Langford, John Sillitoe on behalf of the Wellcome Sanger Institute COVID-19 Surveillance Team
EPI_ISL_762036, EPI_ISL_762037	Lighthouse Lab in Cambridge	Wellcome Sanger Institute for the COVID-19 Genomics UK (COG-UK) Consortium	Rob Howes, The Lighthouse Lab in Cambridge and Alex Alderton, Roberto Amato, Sonia Goncalves, Ewan Harrison, David K. Jackson, Ian Johnston, Dominic Kwiatkowski, Cordelia Langford, John Sillitoe on behalf of the Wellcome Sanger Institute COVID-19 Surveillance Team
EPI_ISL_762039, EPI_ISL_762040	Lighthouse Lab in Glasgow	Wellcome Sanger Institute for the COVID-19 Genomics UK (COG-UK) Consortium	Harper VanSteenhouse, Yumi Kasai, David Gray, Carol Clugston, Anna Dominiczak and Alex Alderton, Roberto Amato, Sonia Goncalves, Ewan Harrison, David K. Jackson, Ian Johnston, Dominic Kwiatkowski, Cordelia Langford, John Sillitoe on behalf of the Wellcome Sanger Institute COVID-19 Surveillance Team
EPI_ISL_762042, EPI_ISL_762043	Lighthouse Lab in Cambridge	Wellcome Sanger Institute for the COVID-19 Genomics UK (COG-UK) Consortium	Rob Howes, The Lighthouse Lab in Cambridge and Alex Alderton, Roberto Amato, Sonia Goncalves, Ewan Harrison, David K. Jackson, Ian Johnston, Dominic Kwiatkowski, Cordelia Langford, John Sillitoe on behalf of the Wellcome Sanger Institute COVID-19 Surveillance Team
EPI_ISL_762044	Lighthouse Lab in Glasgow	Wellcome Sanger Institute for the COVID-19 Genomics UK (COG-UK) Consortium	Harper VanSteenhouse, Yumi Kasai, David Gray, Carol Clugston, Anna Dominiczak and Alex Alderton, Roberto Amato, Sonia Goncalves, Ewan Harrison, David K. Jackson, Ian Johnston, Dominic Kwiatkowski, Cordelia Langford, John Sillitoe on behalf of the Wellcome Sanger Institute COVID-19 Surveillance Team
EPI_ISL_762045	Lighthouse Lab in Cambridge	Wellcome Sanger Institute for the COVID-19 Genomics UK (COG-UK) Consortium	Rob Howes, The Lighthouse Lab in Cambridge and Alex Alderton, Roberto Amato, Sonia Goncalves, Ewan Harrison, David K. Jackson, Ian Johnston, Dominic Kwiatkowski, Cordelia Langford, John Sillitoe on behalf of the Wellcome Sanger Institute COVID-19 Surveillance Team
EPI_ISL_762046, EPI_ISL_762047, EPI_ISL_762048, EPI_ISL_762049	Lighthouse Lab in Glasgow	Wellcome Sanger Institute for the COVID-19 Genomics UK (COG-UK) Consortium	Harper VanSteenhouse, Yumi Kasai, David Gray, Carol Clugston, Anna Dominiczak and Alex Alderton, Roberto Amato, Sonia Goncalves, Ewan Harrison, David K. Jackson, Ian Johnston, Dominic Kwiatkowski, Cordelia Langford, John Sillitoe on behalf of the Wellcome Sanger Institute COVID-19 Surveillance Team
EPI_ISL_762050	Lighthouse Lab in Cambridge	Wellcome Sanger Institute for the COVID-19 Genomics UK (COG-UK) Consortium	Rob Howes, The Lighthouse Lab in Cambridge and Alex Alderton, Roberto Amato, Sonia Goncalves, Ewan Harrison, David K. Jackson, Ian Johnston, Dominic Kwiatkowski, Cordelia Langford, John Sillitoe on behalf of the Wellcome Sanger Institute COVID-19 Surveillance Team
EPI_ISL_762051, EPI_ISL_762052,	Lighthouse Lab in Glasgow	Wellcome Sanger Institute for the COVID-19 Genomics UK	Harper VanSteenhouse, Yumi Kasai, David Gray, Carol Clugston, Anna Dominiczak and Alex Alderton, Roberto Amato, Sonia Goncalves, Ewan Harrison,

EPI_ISL_762053, EPI_ISL_762055, EPI_ISL_762056		(COG-UK) Consortium	David K. Jackson, Ian Johnston, Dominic Kwiatkowski, Cordelia Langford, John Sillitoe on behalf of the Wellcome Sanger Institute COVID-19 Surveillance Team
EPI_ISL_762057	Lighthouse Lab in Cambridge	Wellcome Sanger Institute for the COVID-19 Genomics UK (COG-UK) Consortium	Rob Howes, The Lighthouse Lab in Cambridge and Alex Alderton, Roberto Amato, Sonia Goncalves, Ewan Harrison, David K. Jackson, Ian Johnston, Dominic Kwiatkowski, Cordelia Langford, John Sillitoe on behalf of the Wellcome Sanger Institute COVID-19 Surveillance Team
EPI_ISL_762058	Lighthouse Lab in Glasgow	Wellcome Sanger Institute for the COVID-19 Genomics UK (COG-UK) Consortium	Harper VanSteenhouse, Yumi Kasai, David Gray, Carol Clugston, Anna Dominiczak and Alex Alderton, Roberto Amato, Sonia Goncalves, Ewan Harrison, David K. Jackson, Ian Johnston, Dominic Kwiatkowski, Cordelia Langford, John Sillitoe on behalf of the Wellcome Sanger Institute COVID-19 Surveillance Team
EPI_ISL_762059, EPI_ISL_762060, EPI_ISL_762061, EPI_ISL_762063, EPI_ISL_762064, EPI_ISL_762065, EPI_ISL_762066, EPI_ISL_762067	Lighthouse Lab in Cambridge	Wellcome Sanger Institute for the COVID-19 Genomics UK (COG-UK) Consortium	Rob Howes, The Lighthouse Lab in Cambridge and Alex Alderton, Roberto Amato, Sonia Goncalves, Ewan Harrison, David K. Jackson, Ian Johnston, Dominic Kwiatkowski, Cordelia Langford, John Sillitoe on behalf of the Wellcome Sanger Institute COVID-19 Surveillance Team
EPI_ISL_762068, EPI_ISL_762071	Lighthouse Lab in Glasgow	Wellcome Sanger Institute for the COVID-19 Genomics UK (COG-UK) Consortium	Harper VanSteenhouse, Yumi Kasai, David Gray, Carol Clugston, Anna Dominiczak and Alex Alderton, Roberto Amato, Sonia Goncalves, Ewan Harrison, David K. Jackson, Ian Johnston, Dominic Kwiatkowski, Cordelia Langford, John Sillitoe on behalf of the Wellcome Sanger Institute COVID-19 Surveillance Team
EPI_ISL_762073, EPI_ISL_762074, EPI_ISL_762075	Lighthouse Lab in Cambridge	Wellcome Sanger Institute for the COVID-19 Genomics UK (COG-UK) Consortium	Rob Howes, The Lighthouse Lab in Cambridge and Alex Alderton, Roberto Amato, Sonia Goncalves, Ewan Harrison, David K. Jackson, Ian Johnston, Dominic Kwiatkowski, Cordelia Langford, John Sillitoe on behalf of the Wellcome Sanger Institute COVID-19 Surveillance Team
EPI_ISL_762076, EPI_ISL_762077, EPI_ISL_762080, EPI_ISL_762081, EPI_ISL_762082, EPI_ISL_762083, EPI_ISL_762084, EPI_ISL_762086	Lighthouse Lab in Glasgow	Wellcome Sanger Institute for the COVID-19 Genomics UK (COG-UK) Consortium	Harper VanSteenhouse, Yumi Kasai, David Gray, Carol Clugston, Anna Dominiczak and Alex Alderton, Roberto Amato, Sonia Goncalves, Ewan Harrison, David K. Jackson, Ian Johnston, Dominic Kwiatkowski, Cordelia Langford, John Sillitoe on behalf of the Wellcome Sanger Institute COVID-19 Surveillance Team
EPI_ISL_762087, EPI_ISL_762089	Lighthouse Lab in Cambridge	Wellcome Sanger Institute for the COVID-19 Genomics UK (COG-UK) Consortium	Rob Howes, The Lighthouse Lab in Cambridge and Alex Alderton, Roberto Amato, Sonia Goncalves, Ewan Harrison, David K. Jackson, Ian Johnston, Dominic Kwiatkowski, Cordelia Langford, John Sillitoe on behalf of the Wellcome Sanger Institute COVID-19 Surveillance Team
EPI_ISL_762092, EPI_ISL_762094, EPI_ISL_762095	Lighthouse Lab in Glasgow	Wellcome Sanger Institute for the COVID-19 Genomics UK (COG-UK) Consortium	Harper VanSteenhouse, Yumi Kasai, David Gray, Carol Clugston, Anna Dominiczak and Alex Alderton, Roberto Amato, Sonia Goncalves, Ewan Harrison, David K. Jackson, Ian Johnston, Dominic Kwiatkowski, Cordelia Langford, John Sillitoe on behalf of the Wellcome Sanger Institute COVID-19 Surveillance Team
EPI_ISL_762097	Lighthouse Lab in Cambridge	Wellcome Sanger Institute for the COVID-19 Genomics UK (COG-UK) Consortium	Rob Howes, The Lighthouse Lab in Cambridge and Alex Alderton, Roberto Amato, Sonia Goncalves, Ewan Harrison, David K. Jackson, Ian Johnston, Dominic Kwiatkowski, Cordelia Langford, John Sillitoe on behalf of the Wellcome Sanger Institute COVID-19 Surveillance Team
EPI_ISL_762100, EPI_ISL_762101, EPI_ISL_762102	Lighthouse Lab in Glasgow	Wellcome Sanger Institute for the COVID-19 Genomics UK (COG-UK) Consortium	Harper VanSteenhouse, Yumi Kasai, David Gray, Carol Clugston, Anna Dominiczak and Alex Alderton, Roberto Amato, Sonia Goncalves, Ewan Harrison, David K. Jackson, Ian Johnston, Dominic Kwiatkowski, Cordelia Langford, John Sillitoe on behalf of the Wellcome Sanger Institute COVID-19 Surveillance Team
EPI_ISL_762103	Lighthouse Lab in Cambridge	Wellcome Sanger Institute for the COVID-19 Genomics UK (COG-UK) Consortium	Rob Howes, The Lighthouse Lab in Cambridge and Alex Alderton, Roberto Amato, Sonia Goncalves, Ewan Harrison, David K. Jackson, Ian Johnston, Dominic Kwiatkowski, Cordelia Langford, John Sillitoe on behalf of the Wellcome Sanger Institute COVID-19 Surveillance Team
EPI_ISL_762105, EPI_ISL_762106, EPI_ISL_762107, EPI_ISL_762108	Lighthouse Lab in Glasgow	Wellcome Sanger Institute for the COVID-19 Genomics UK (COG-UK) Consortium	Harper VanSteenhouse, Yumi Kasai, David Gray, Carol Clugston, Anna Dominiczak and Alex Alderton, Roberto Amato, Sonia Goncalves, Ewan Harrison, David K. Jackson, Ian Johnston, Dominic Kwiatkowski, Cordelia Langford, John Sillitoe on behalf of the Wellcome Sanger Institute COVID-19 Surveillance Team
EPI_ISL_762109	Lighthouse Lab in Cambridge	Wellcome Sanger Institute for the COVID-19 Genomics UK (COG-UK) Consortium	Rob Howes, The Lighthouse Lab in Cambridge and Alex Alderton, Roberto Amato, Sonia Goncalves, Ewan Harrison, David K. Jackson, Ian Johnston, Dominic Kwiatkowski, Cordelia Langford, John Sillitoe on behalf of the Wellcome Sanger Institute COVID-19 Surveillance Team
EPI_ISL_762110, EPI_ISL_762111	Lighthouse Lab in Glasgow	Wellcome Sanger Institute for the COVID-19 Genomics UK (COG-UK) Consortium	Harper VanSteenhouse, Yumi Kasai, David Gray, Carol Clugston, Anna Dominiczak and Alex Alderton, Roberto Amato, Sonia Goncalves, Ewan Harrison, David K. Jackson, Ian Johnston, Dominic Kwiatkowski, Cordelia Langford, John Sillitoe on behalf of the Wellcome Sanger Institute COVID-19 Surveillance Team
EPI_ISL_762112, EPI_ISL_762113, EPI_ISL_762114	Lighthouse Lab in Cambridge	Wellcome Sanger Institute for the COVID-19 Genomics UK (COG-UK) Consortium	Rob Howes, The Lighthouse Lab in Cambridge and Alex Alderton, Roberto Amato, Sonia Goncalves, Ewan Harrison, David K. Jackson, Ian Johnston, Dominic Kwiatkowski, Cordelia Langford, John Sillitoe on behalf of the Wellcome Sanger Institute COVID-19 Surveillance Team
EPI_ISL_762115, EPI_ISL_762116	Lighthouse Lab in Glasgow	Wellcome Sanger Institute for the COVID-19 Genomics UK (COG-UK) Consortium	Harper VanSteenhouse, Yumi Kasai, David Gray, Carol Clugston, Anna Dominiczak and Alex Alderton, Roberto Amato, Sonia Goncalves, Ewan Harrison, David K. Jackson, Ian Johnston, Dominic Kwiatkowski, Cordelia Langford, John Sillitoe on behalf of the Wellcome Sanger Institute COVID-19 Surveillance Team
EPI_ISL_762118	Lighthouse Lab in Cambridge	Wellcome Sanger Institute for the COVID-19 Genomics UK (COG-UK) Consortium	Rob Howes, The Lighthouse Lab in Cambridge and Alex Alderton, Roberto Amato, Sonia Goncalves, Ewan Harrison, David K. Jackson, Ian Johnston, Dominic Kwiatkowski, Cordelia Langford, John Sillitoe on behalf of the Wellcome Sanger Institute COVID-19 Surveillance Team
EPI_ISL_762119, EPI_ISL_762121, EPI_ISL_762123	Lighthouse Lab in Glasgow	Wellcome Sanger Institute for the COVID-19 Genomics UK (COG-UK) Consortium	Harper VanSteenhouse, Yumi Kasai, David Gray, Carol Clugston, Anna Dominiczak and Alex Alderton, Roberto Amato, Sonia Goncalves, Ewan Harrison, David K. Jackson, Ian Johnston, Dominic Kwiatkowski, Cordelia Langford, John Sillitoe on behalf of the Wellcome Sanger Institute COVID-19 Surveillance Team
EPI_ISL_762124, EPI_ISL_762125, EPI_ISL_762126	Lighthouse Lab in Cambridge	Wellcome Sanger Institute for the COVID-19 Genomics UK (COG-UK) Consortium	Rob Howes, The Lighthouse Lab in Cambridge and Alex Alderton, Roberto Amato, Sonia Goncalves, Ewan Harrison, David K. Jackson, Ian Johnston, Dominic Kwiatkowski, Cordelia Langford, John Sillitoe on behalf of the Wellcome Sanger Institute COVID-19 Surveillance Team
EPI_ISL_762127, EPI_ISL_762129, EPI_ISL_762130, EPI_ISL_762132	Lighthouse Lab in Glasgow	Wellcome Sanger Institute for the COVID-19 Genomics UK (COG-UK) Consortium	Harper VanSteenhouse, Yumi Kasai, David Gray, Carol Clugston, Anna Dominiczak and Alex Alderton, Roberto Amato, Sonia Goncalves, Ewan Harrison, David K. Jackson, Ian Johnston, Dominic Kwiatkowski, Cordelia Langford, John Sillitoe on behalf of the Wellcome Sanger Institute COVID-19 Surveillance Team
EPI_ISL_762133	Lighthouse Lab in Cambridge	Wellcome Sanger Institute for the COVID-19 Genomics UK (COG-UK) Consortium	Rob Howes, The Lighthouse Lab in Cambridge and Alex Alderton, Roberto Amato, Sonia Goncalves, Ewan Harrison, David K. Jackson, Ian Johnston, Dominic Kwiatkowski, Cordelia Langford, John Sillitoe on behalf of the Wellcome Sanger Institute COVID-19 Surveillance Team
EPI_ISL_762134	Lighthouse Lab in Glasgow	Wellcome Sanger Institute for the COVID-19 Genomics UK (COG-UK) Consortium	Harper VanSteenhouse, Yumi Kasai, David Gray, Carol Clugston, Anna Dominiczak and Alex Alderton, Roberto Amato, Sonia Goncalves, Ewan Harrison, David K. Jackson, Ian Johnston, Dominic Kwiatkowski, Cordelia Langford, John Sillitoe on behalf of the Wellcome Sanger Institute COVID-19 Surveillance Team
EPI_ISL_762135, EPI_ISL_762136, EPI_ISL_762137, EPI_ISL_762138, EPI_ISL_762139	Lighthouse Lab in Cambridge	Wellcome Sanger Institute for the COVID-19 Genomics UK (COG-UK) Consortium	Rob Howes, The Lighthouse Lab in Cambridge and Alex Alderton, Roberto Amato, Sonia Goncalves, Ewan Harrison, David K. Jackson, Ian Johnston, Dominic Kwiatkowski, Cordelia Langford, John Sillitoe on behalf of the Wellcome Sanger Institute COVID-19 Surveillance Team
EPI_ISL_762141	Lighthouse Lab in Glasgow	Wellcome Sanger Institute for the COVID-19 Genomics UK (COG-UK) Consortium	Harper VanSteenhouse, Yumi Kasai, David Gray, Carol Clugston, Anna Dominiczak and Alex Alderton, Roberto Amato, Sonia Goncalves, Ewan Harrison, David K. Jackson, Ian Johnston, Dominic Kwiatkowski, Cordelia Langford, John Sillitoe on behalf of the Wellcome Sanger Institute COVID-19 Surveillance Team
EPI_ISL_762142	Lighthouse Lab in Cambridge	Wellcome Sanger Institute for the COVID-19 Genomics UK (COG-UK) Consortium	Rob Howes, The Lighthouse Lab in Cambridge and Alex Alderton, Roberto Amato, Sonia Goncalves, Ewan Harrison, David K. Jackson, Ian Johnston, Dominic Kwiatkowski, Cordelia Langford, John Sillitoe on behalf of the Wellcome Sanger Institute COVID-19 Surveillance Team
EPI_ISL_762144, EPI_ISL_762145	Lighthouse Lab in Glasgow	Wellcome Sanger Institute for the COVID-19 Genomics UK (COG-UK) Consortium	Harper VanSteenhouse, Yumi Kasai, David Gray, Carol Clugston, Anna Dominiczak and Alex Alderton, Roberto Amato, Sonia Goncalves, Ewan Harrison, David K. Jackson, Ian Johnston, Dominic Kwiatkowski, Cordelia Langford, John Sillitoe on behalf of the Wellcome Sanger Institute COVID-19 Surveillance Team
EPI_ISL_762146, EPI_ISL_762147, EPI_ISL_762148	Lighthouse Lab in Cambridge	Wellcome Sanger Institute for the COVID-19 Genomics UK (COG-UK) Consortium	Rob Howes, The Lighthouse Lab in Cambridge and Alex Alderton, Roberto Amato, Sonia Goncalves, Ewan Harrison, David K. Jackson, Ian Johnston, Dominic Kwiatkowski, Cordelia Langford, John Sillitoe on behalf of the Wellcome Sanger Institute COVID-19 Surveillance Team

EPI_ISL_762149, EPI_ISL_762150, EPI_ISL_762151	Lighthouse Lab in Glasgow	Wellcome Sanger Institute for the COVID-19 Genomics UK (COG-UK) Consortium	Harper VanSteenhouse, Yumi Kasai, David Gray, Carol Clugston, Anna Dominiczak and Alex Alderton, Roberto Amato, Sonia Goncalves, Ewan Harrison, David K. Jackson, Ian Johnston, Dominic Kwiatkowski, Cordelia Langford, John Sillitoe on behalf of the Wellcome Sanger Institute COVID-19 Surveillance Team
EPI_ISL_762153	Lighthouse Lab in Cambridge	Wellcome Sanger Institute for the COVID-19 Genomics UK (COG-UK) Consortium	Rob Howes, The Lighthouse Lab in Cambridge and Alex Alderton, Roberto Amato, Sonia Goncalves, Ewan Harrison, David K. Jackson, Ian Johnston, Dominic Kwiatkowski, Cordelia Langford, John Sillitoe on behalf of the Wellcome Sanger Institute COVID-19 Surveillance Team
EPI_ISL_762154, EPI_ISL_762156, EPI_ISL_762157, EPI_ISL_762158, EPI_ISL_762162	Lighthouse Lab in Glasgow	Wellcome Sanger Institute for the COVID-19 Genomics UK (COG-UK) Consortium	Harper VanSteenhouse, Yumi Kasai, David Gray, Carol Clugston, Anna Dominiczak and Alex Alderton, Roberto Amato, Sonia Goncalves, Ewan Harrison, David K. Jackson, Ian Johnston, Dominic Kwiatkowski, Cordelia Langford, John Sillitoe on behalf of the Wellcome Sanger Institute COVID-19 Surveillance Team
EPI_ISL_762163	Lighthouse Lab in Cambridge	Wellcome Sanger Institute for the COVID-19 Genomics UK (COG-UK) Consortium	Rob Howes, The Lighthouse Lab in Cambridge and Alex Alderton, Roberto Amato, Sonia Goncalves, Ewan Harrison, David K. Jackson, Ian Johnston, Dominic Kwiatkowski, Cordelia Langford, John Sillitoe on behalf of the Wellcome Sanger Institute COVID-19 Surveillance Team
EPI_ISL_762164, EPI_ISL_762165	Lighthouse Lab in Glasgow	Wellcome Sanger Institute for the COVID-19 Genomics UK (COG-UK) Consortium	Harper VanSteenhouse, Yumi Kasai, David Gray, Carol Clugston, Anna Dominiczak and Alex Alderton, Roberto Amato, Sonia Goncalves, Ewan Harrison, David K. Jackson, Ian Johnston, Dominic Kwiatkowski, Cordelia Langford, John Sillitoe on behalf of the Wellcome Sanger Institute COVID-19 Surveillance Team
EPI_ISL_762166	Lighthouse Lab in Cambridge	Wellcome Sanger Institute for the COVID-19 Genomics UK (COG-UK) Consortium	Rob Howes, The Lighthouse Lab in Cambridge and Alex Alderton, Roberto Amato, Sonia Goncalves, Ewan Harrison, David K. Jackson, Ian Johnston, Dominic Kwiatkowski, Cordelia Langford, John Sillitoe on behalf of the Wellcome Sanger Institute COVID-19 Surveillance Team
EPI_ISL_762167, EPI_ISL_762168, EPI_ISL_762169, EPI_ISL_762170, EPI_ISL_762171, EPI_ISL_762172	Lighthouse Lab in Glasgow	Wellcome Sanger Institute for the COVID-19 Genomics UK (COG-UK) Consortium	Harper VanSteenhouse, Yumi Kasai, David Gray, Carol Clugston, Anna Dominiczak and Alex Alderton, Roberto Amato, Sonia Goncalves, Ewan Harrison, David K. Jackson, Ian Johnston, Dominic Kwiatkowski, Cordelia Langford, John Sillitoe on behalf of the Wellcome Sanger Institute COVID-19 Surveillance Team
EPI_ISL_762173, EPI_ISL_762175	Lighthouse Lab in Cambridge	Wellcome Sanger Institute for the COVID-19 Genomics UK (COG-UK) Consortium	Rob Howes, The Lighthouse Lab in Cambridge and Alex Alderton, Roberto Amato, Sonia Goncalves, Ewan Harrison, David K. Jackson, Ian Johnston, Dominic Kwiatkowski, Cordelia Langford, John Sillitoe on behalf of the Wellcome Sanger Institute COVID-19 Surveillance Team
EPI_ISL_762176, EPI_ISL_762177, EPI_ISL_762179, EPI_ISL_762182	Lighthouse Lab in Glasgow	Wellcome Sanger Institute for the COVID-19 Genomics UK (COG-UK) Consortium	Harper VanSteenhouse, Yumi Kasai, David Gray, Carol Clugston, Anna Dominiczak and Alex Alderton, Roberto Amato, Sonia Goncalves, Ewan Harrison, David K. Jackson, Ian Johnston, Dominic Kwiatkowski, Cordelia Langford, John Sillitoe on behalf of the Wellcome Sanger Institute COVID-19 Surveillance Team
EPI_ISL_762183	Lighthouse Lab in Cambridge	Wellcome Sanger Institute for the COVID-19 Genomics UK (COG-UK) Consortium	Rob Howes, The Lighthouse Lab in Cambridge and Alex Alderton, Roberto Amato, Sonia Goncalves, Ewan Harrison, David K. Jackson, Ian Johnston, Dominic Kwiatkowski, Cordelia Langford, John Sillitoe on behalf of the Wellcome Sanger Institute COVID-19 Surveillance Team
EPI_ISL_762184	Lighthouse Lab in Glasgow	Wellcome Sanger Institute for the COVID-19 Genomics UK (COG-UK) Consortium	Harper VanSteenhouse, Yumi Kasai, David Gray, Carol Clugston, Anna Dominiczak and Alex Alderton, Roberto Amato, Sonia Goncalves, Ewan Harrison, David K. Jackson, Ian Johnston, Dominic Kwiatkowski, Cordelia Langford, John Sillitoe on behalf of the Wellcome Sanger Institute COVID-19 Surveillance Team
EPI_ISL_762186, EPI_ISL_762187, EPI_ISL_762189, EPI_ISL_762192, EPI_ISL_762193, EPI_ISL_762195	Lighthouse Lab in Cambridge	Wellcome Sanger Institute for the COVID-19 Genomics UK (COG-UK) Consortium	Rob Howes, The Lighthouse Lab in Cambridge and Alex Alderton, Roberto Amato, Sonia Goncalves, Ewan Harrison, David K. Jackson, Ian Johnston, Dominic Kwiatkowski, Cordelia Langford, John Sillitoe on behalf of the Wellcome Sanger Institute COVID-19 Surveillance Team
EPI_ISL_762196, EPI_ISL_762197	Lighthouse Lab in Glasgow	Wellcome Sanger Institute for the COVID-19 Genomics UK (COG-UK) Consortium	Harper VanSteenhouse, Yumi Kasai, David Gray, Carol Clugston, Anna Dominiczak and Alex Alderton, Roberto Amato, Sonia Goncalves, Ewan Harrison, David K. Jackson, Ian Johnston, Dominic Kwiatkowski, Cordelia Langford, John Sillitoe on behalf of the Wellcome Sanger Institute COVID-19 Surveillance Team
EPI_ISL_762199, EPI_ISL_762200, EPI_ISL_762201, EPI_ISL_762202, EPI_ISL_762203, EPI_ISL_762204, EPI_ISL_762205, EPI_ISL_762206, EPI_ISL_762207, EPI_ISL_762208, EPI_ISL_762209, EPI_ISL_762210, EPI_ISL_762211, EPI_ISL_762212, EPI_ISL_762213, EPI_ISL_762214, EPI_ISL_762215, EPI_ISL_762217, EPI_ISL_762218, EPI_ISL_762219, EPI_ISL_762221, EPI_ISL_762222, EPI_ISL_762223, EPI_ISL_762224, EPI_ISL_762225, EPI_ISL_762226, EPI_ISL_762228, EPI_ISL_762230, EPI_ISL_762231, EPI_ISL_762232, EPI_ISL_762234, EPI_ISL_762235, EPI_ISL_762236, EPI_ISL_762237, EPI_ISL_762242, EPI_ISL_762243, EPI_ISL_762244, EPI_ISL_762245, EPI_ISL_762247, EPI_ISL_762248, EPI_ISL_762250, EPI_ISL_762251, EPI_ISL_762254, EPI_ISL_762255, EPI_ISL_762256, EPI_ISL_762257, EPI_ISL_762258, EPI_ISL_762259, EPI_ISL_762260, EPI_ISL_762261, EPI_ISL_762262, EPI_ISL_762263, EPI_ISL_762264, EPI_ISL_762265, EPI_ISL_762266, EPI_ISL_762267, EPI_ISL_762269, EPI_ISL_762270, EPI_ISL_762271, EPI_ISL_762272, EPI_ISL_762273, EPI_ISL_762274, EPI_ISL_762275, EPI_ISL_762276, EPI_ISL_762277, EPI_ISL_762278, EPI_ISL_762281, EPI_ISL_762282, EPI_ISL_762283, EPI_ISL_762284, EPI_ISL_762287, EPI_ISL_762289, EPI_ISL_762290, EPI_ISL_762291, EPI_ISL_762292, EPI_ISL_762293, EPI_ISL_762294, EPI_ISL_762296, EPI_ISL_762297, EPI_ISL_762298, EPI_ISL_762299, EPI_ISL_762300, EPI_ISL_762301, EPI_ISL_762303, EPI_ISL_762304, EPI_ISL_762306, EPI_ISL_762307, EPI_ISL_762309, EPI_ISL_762311, EPI_ISL_762312, EPI_ISL_762313, EPI_ISL_762314, EPI_ISL_762315, EPI_ISL_762316, EPI_ISL_762317, EPI_ISL_762318, EPI_ISL_762319, EPI_ISL_762320, EPI_ISL_762321, EPI_ISL_762323, EPI_ISL_762324, EPI_ISL_762325, EPI_ISL_762326, EPI_ISL_762327, EPI_ISL_762328, EPI_ISL_762329, EPI_ISL_762330, EPI_ISL_762331, EPI_ISL_762332, EPI_ISL_762334, EPI_ISL_762335, EPI_ISL_762336, EPI_ISL_762337, EPI_ISL_762340, EPI_ISL_762342, EPI_ISL_762343, EPI_ISL_762347, EPI_ISL_762348, EPI_ISL_762349, EPI_ISL_762350, EPI_ISL_762351, EPI_ISL_762353, EPI_ISL_762354, EPI_ISL_762355, EPI_ISL_762356, EPI_ISL_762357, EPI_ISL_762358, EPI_ISL_762360, EPI_ISL_762361, EPI_ISL_762365, EPI_ISL_762366, EPI_ISL_762367, EPI_ISL_762369, EPI_ISL_762370, EPI_ISL_762371, EPI_ISL_762373, EPI_ISL_762374, EPI_ISL_762375, EPI_ISL_762376, EPI_ISL_762377, EPI_ISL_762378, EPI_ISL_762380, EPI_ISL_762382, EPI_ISL_762383, EPI_ISL_762384, EPI_ISL_762385, EPI_ISL_762386, EPI_ISL_762388, EPI_ISL_762391, EPI_ISL_762392, EPI_ISL_762393, EPI_ISL_762394, EPI_ISL_762396, EPI_ISL_762397, EPI_ISL_762399, EPI_ISL_762400, EPI_ISL_762401, EPI_ISL_762402, EPI_ISL_762403, EPI_ISL_762404, EPI_ISL_762405, EPI_ISL_762406, EPI_ISL_762407, EPI_ISL_762408, EPI_ISL_762409, EPI_ISL_762410, EPI_ISL_762411, EPI_ISL_762412, EPI_ISL_762413, EPI_ISL_762414, EPI_ISL_762415, EPI_ISL_762416, EPI_ISL_762417, EPI_ISL_762418, EPI_ISL_762419, EPI_ISL_762422, EPI_ISL_762429, EPI_ISL_762431, EPI_ISL_762433, EPI_ISL_762435, EPI_ISL_762436, EPI_ISL_762437, EPI_ISL_762438, EPI_ISL_762439, EPI_ISL_762440, EPI_ISL_762442, EPI_ISL_762444, EPI_ISL_762445, EPI_ISL_762447, EPI_ISL_762448, EPI_ISL_762450, EPI_ISL_762451, EPI_ISL_762453, EPI_ISL_762454, EPI_ISL_762455, EPI_ISL_762456, EPI_ISL_762457, EPI_ISL_762459, EPI_ISL_762460, EPI_ISL_762461, EPI_ISL_762463, EPI_ISL_762464, EPI_ISL_762465, EPI_ISL_762467, EPI_ISL_762470, EPI_ISL_762471, EPI_ISL_762472, EPI_ISL_762473, EPI_ISL_762474, EPI_ISL_762475, EPI_ISL_762476, EPI_ISL_762477, EPI_ISL_762478, EPI_ISL_762479, EPI_ISL_762480, EPI_ISL_762481, EPI_ISL_762482, EPI_ISL_762483, EPI_ISL_762484, EPI_ISL_762485, EPI_ISL_762486, EPI_ISL_762487, EPI_ISL_762489, EPI_ISL_762490, EPI_ISL_762491, EPI_ISL_762492, EPI_ISL_762493, EPI_ISL_762494, EPI_ISL_762496, EPI_ISL_762498, EPI_ISL_762501, EPI_ISL_762502, EPI_ISL_762503, EPI_ISL_762504, EPI_ISL_762505, EPI_ISL_762507, EPI_ISL_762508, EPI_ISL_762509, EPI_ISL_762510, EPI_ISL_762511, EPI_ISL_762512, EPI_ISL_762513, EPI_ISL_762514, EPI_ISL_762515, EPI_ISL_762516, EPI_ISL_762518, EPI_ISL_762520, EPI_ISL_762521, EPI_ISL_762522, EPI_ISL_762525, EPI_ISL_762526, EPI_ISL_762527, EPI_ISL_762529, EPI_ISL_762530, EPI_ISL_762531, EPI_ISL_762533, EPI_ISL_762534, EPI_ISL_762535, EPI_ISL_762537, EPI_ISL_762540, EPI_ISL_762541, EPI_ISL_762542, EPI_ISL_762543, EPI_ISL_762545			
see above	Lighthouse Lab in Milton Keynes	Wellcome Sanger Institute for the COVID-19 Genomics UK (COG-UK) Consortium	The Lighthouse Lab in Milton Keynes and Alex Alderton, Roberto Amato, Sonia Goncalves, Ewan Harrison, David K. Jackson, Ian Johnston, Dominic Kwiatkowski, Cordelia Langford, John Sillitoe on behalf of the Wellcome Sanger Institute COVID-19 Surveillance Team
EPI_ISL_762546	Lighthouse Lab in Cambridge	Wellcome Sanger Institute for the COVID-19 Genomics UK (COG-UK) Consortium	Rob Howes, The Lighthouse Lab in Cambridge and Alex Alderton, Roberto Amato, Sonia Goncalves, Ewan Harrison, David K. Jackson, Ian Johnston, Dominic Kwiatkowski, Cordelia Langford, John Sillitoe on behalf of the Wellcome Sanger Institute COVID-19 Surveillance Team
EPI_ISL_762547	Lighthouse Lab in Alderley Park	Wellcome Sanger Institute for the COVID-19 Genomics UK (COG-UK) Consortium	Jacquelyn Wynn, Mairead Hyland, The Lighthouse Lab in Alderley Park and Alex Alderton, Roberto Amato, Sonia Goncalves, Ewan Harrison, David K. Jackson, Ian Johnston, Dominic Kwiatkowski, Cordelia Langford, John Sillitoe on behalf of the Wellcome Sanger Institute COVID-19 Surveillance Team
EPI_ISL_762548	Lighthouse Lab in Glasgow	Wellcome Sanger Institute for the COVID-19 Genomics UK (COG-UK) Consortium	Harper VanSteenhouse, Yumi Kasai, David Gray, Carol Clugston, Anna Dominiczak and Alex Alderton, Roberto Amato, Sonia Goncalves, Ewan Harrison, David K. Jackson, Ian Johnston, Dominic Kwiatkowski, Cordelia Langford, John Sillitoe on behalf of the Wellcome Sanger Institute COVID-19 Surveillance Team
EPI_ISL_762551, EPI_ISL_762552, EPI_ISL_762553	Lighthouse Lab in Cambridge	Wellcome Sanger Institute for the COVID-19 Genomics UK (COG-UK) Consortium	Rob Howes, The Lighthouse Lab in Cambridge and Alex Alderton, Roberto Amato, Sonia Goncalves, Ewan Harrison, David K. Jackson, Ian Johnston, Dominic Kwiatkowski, Cordelia Langford, John Sillitoe on behalf of the Wellcome Sanger Institute COVID-19 Surveillance Team
EPI_ISL_762555, EPI_ISL_762556	Lighthouse Lab in Glasgow	Wellcome Sanger Institute for the COVID-19 Genomics UK (COG-UK) Consortium	Harper VanSteenhouse, Yumi Kasai, David Gray, Carol Clugston, Anna Dominiczak and Alex Alderton, Roberto Amato, Sonia Goncalves, Ewan Harrison, David K. Jackson, Ian Johnston, Dominic Kwiatkowski, Cordelia Langford, John Sillitoe on behalf of the Wellcome Sanger Institute COVID-19 Surveillance Team
EPI_ISL_762557, EPI_ISL_762558	Lighthouse Lab in Alderley Park	Wellcome Sanger Institute for the COVID-19 Genomics UK (COG-UK) Consortium	Jacquelyn Wynn, Mairead Hyland, The Lighthouse Lab in Alderley Park and Alex Alderton, Roberto Amato, Sonia Goncalves, Ewan Harrison, David K. Jackson, Ian Johnston, Dominic Kwiatkowski, Cordelia Langford, John Sillitoe on behalf of the Wellcome Sanger Institute COVID-19 Surveillance Team
EPI_ISL_762560, EPI_ISL_762561	Lighthouse Lab in Glasgow	Wellcome Sanger Institute for the COVID-19 Genomics UK (COG-UK) Consortium	Harper VanSteenhouse, Yumi Kasai, David Gray, Carol Clugston, Anna Dominiczak and Alex Alderton, Roberto Amato, Sonia Goncalves, Ewan Harrison, David K. Jackson, Ian Johnston, Dominic Kwiatkowski, Cordelia Langford, John Sillitoe on behalf of the Wellcome Sanger Institute COVID-19 Surveillance Team
EPI_ISL_762562	Lighthouse Lab in Alderley Park	Wellcome Sanger Institute for the COVID-19 Genomics UK (COG-UK) Consortium	Jacquelyn Wynn, Mairead Hyland, The Lighthouse Lab in Alderley Park and Alex Alderton, Roberto Amato, Sonia Goncalves, Ewan Harrison, David K. Jackson, Ian Johnston, Dominic Kwiatkowski, Cordelia Langford, John Sillitoe on behalf of the Wellcome Sanger Institute COVID-19 Surveillance Team
EPI_ISL_762563	Lighthouse Lab in Cambridge	Wellcome Sanger Institute for the COVID-19 Genomics UK (COG-UK) Consortium	Rob Howes, The Lighthouse Lab in Cambridge and Alex Alderton, Roberto Amato, Sonia Goncalves, Ewan Harrison, David K. Jackson, Ian Johnston, Dominic Kwiatkowski, Cordelia Langford, John Sillitoe on behalf of the Wellcome Sanger Institute COVID-19 Surveillance Team
EPI_ISL_762564	Lighthouse Lab in Alderley Park	Wellcome Sanger Institute for the COVID-19 Genomics UK (COG-UK) Consortium	Jacquelyn Wynn, Mairead Hyland, The Lighthouse Lab in Alderley Park and Alex Alderton, Roberto Amato, Sonia Goncalves, Ewan Harrison, David K. Jackson, Ian Johnston, Dominic Kwiatkowski, Cordelia Langford, John Sillitoe on behalf of the Wellcome Sanger Institute COVID-19 Surveillance Team
EPI_ISL_762565, EPI_ISL_762566	Lighthouse Lab in Cambridge	Wellcome Sanger Institute for the COVID-19 Genomics UK	Rob Howes, The Lighthouse Lab in Cambridge and Alex Alderton, Roberto Amato, Sonia Goncalves, Ewan Harrison, David K. Jackson, Ian Johnston,

		(COG-UK) Consortium	Dominic Kwiatkowski, Cordelia Langford, John Sillitoe on behalf of the Wellcome Sanger Institute COVID-19 Surveillance Team
EPI_ISL_762567	Lighthouse Lab in Glasgow	Wellcome Sanger Institute for the COVID-19 Genomics UK (COG-UK) Consortium	Harper VanSteenhouse, Yumi Kasai, David Gray, Carol Clugston, Anna Dominiczak and Alex Alderton, Roberto Amato, Sonia Goncalves, Ewan Harrison, David K. Jackson, Ian Johnston, Dominic Kwiatkowski, Cordelia Langford, John Sillitoe on behalf of the Wellcome Sanger Institute COVID-19 Surveillance Team
EPI_ISL_762570, EPI_ISL_762572, EPI_ISL_762575	Lighthouse Lab in Cambridge	Wellcome Sanger Institute for the COVID-19 Genomics UK (COG-UK) Consortium	Rob Howes, The Lighthouse Lab in Cambridge and Alex Alderton, Roberto Amato, Sonia Goncalves, Ewan Harrison, David K. Jackson, Ian Johnston, Dominic Kwiatkowski, Cordelia Langford, John Sillitoe on behalf of the Wellcome Sanger Institute COVID-19 Surveillance Team
EPI_ISL_762576	Lighthouse Lab in Glasgow	Wellcome Sanger Institute for the COVID-19 Genomics UK (COG-UK) Consortium	Harper VanSteenhouse, Yumi Kasai, David Gray, Carol Clugston, Anna Dominiczak and Alex Alderton, Roberto Amato, Sonia Goncalves, Ewan Harrison, David K. Jackson, Ian Johnston, Dominic Kwiatkowski, Cordelia Langford, John Sillitoe on behalf of the Wellcome Sanger Institute COVID-19 Surveillance Team
EPI_ISL_762577, EPI_ISL_762578, EPI_ISL_762580	Lighthouse Lab in Cambridge	Wellcome Sanger Institute for the COVID-19 Genomics UK (COG-UK) Consortium	Rob Howes, The Lighthouse Lab in Cambridge and Alex Alderton, Roberto Amato, Sonia Goncalves, Ewan Harrison, David K. Jackson, Ian Johnston, Dominic Kwiatkowski, Cordelia Langford, John Sillitoe on behalf of the Wellcome Sanger Institute COVID-19 Surveillance Team
EPI_ISL_762581	Lighthouse Lab in Alderley Park	Wellcome Sanger Institute for the COVID-19 Genomics UK (COG-UK) Consortium	Jacquelyn Wynn, Mairead Hyland, The Lighthouse Lab in Alderley Park and Alex Alderton, Roberto Amato, Sonia Goncalves, Ewan Harrison, David K. Jackson, Ian Johnston, Dominic Kwiatkowski, Cordelia Langford, John Sillitoe on behalf of the Wellcome Sanger Institute COVID-19 Surveillance Team
EPI_ISL_762583	Lighthouse Lab in Glasgow	Wellcome Sanger Institute for the COVID-19 Genomics UK (COG-UK) Consortium	Harper VanSteenhouse, Yumi Kasai, David Gray, Carol Clugston, Anna Dominiczak and Alex Alderton, Roberto Amato, Sonia Goncalves, Ewan Harrison, David K. Jackson, Ian Johnston, Dominic Kwiatkowski, Cordelia Langford, John Sillitoe on behalf of the Wellcome Sanger Institute COVID-19 Surveillance Team
EPI_ISL_762584	Lighthouse Lab in Cambridge	Wellcome Sanger Institute for the COVID-19 Genomics UK (COG-UK) Consortium	Rob Howes, The Lighthouse Lab in Cambridge and Alex Alderton, Roberto Amato, Sonia Goncalves, Ewan Harrison, David K. Jackson, Ian Johnston, Dominic Kwiatkowski, Cordelia Langford, John Sillitoe on behalf of the Wellcome Sanger Institute COVID-19 Surveillance Team
EPI_ISL_762586	Lighthouse Lab in Glasgow	Wellcome Sanger Institute for the COVID-19 Genomics UK (COG-UK) Consortium	Harper VanSteenhouse, Yumi Kasai, David Gray, Carol Clugston, Anna Dominiczak and Alex Alderton, Roberto Amato, Sonia Goncalves, Ewan Harrison, David K. Jackson, Ian Johnston, Dominic Kwiatkowski, Cordelia Langford, John Sillitoe on behalf of the Wellcome Sanger Institute COVID-19 Surveillance Team
EPI_ISL_762587	Lighthouse Lab in Cambridge	Wellcome Sanger Institute for the COVID-19 Genomics UK (COG-UK) Consortium	Rob Howes, The Lighthouse Lab in Cambridge and Alex Alderton, Roberto Amato, Sonia Goncalves, Ewan Harrison, David K. Jackson, Ian Johnston, Dominic Kwiatkowski, Cordelia Langford, John Sillitoe on behalf of the Wellcome Sanger Institute COVID-19 Surveillance Team
EPI_ISL_762588	Lighthouse Lab in Alderley Park	Wellcome Sanger Institute for the COVID-19 Genomics UK (COG-UK) Consortium	Jacquelyn Wynn, Mairead Hyland, The Lighthouse Lab in Alderley Park and Alex Alderton, Roberto Amato, Sonia Goncalves, Ewan Harrison, David K. Jackson, Ian Johnston, Dominic Kwiatkowski, Cordelia Langford, John Sillitoe on behalf of the Wellcome Sanger Institute COVID-19 Surveillance Team
EPI_ISL_762589	Lighthouse Lab in Glasgow	Wellcome Sanger Institute for the COVID-19 Genomics UK (COG-UK) Consortium	Harper VanSteenhouse, Yumi Kasai, David Gray, Carol Clugston, Anna Dominiczak and Alex Alderton, Roberto Amato, Sonia Goncalves, Ewan Harrison, David K. Jackson, Ian Johnston, Dominic Kwiatkowski, Cordelia Langford, John Sillitoe on behalf of the Wellcome Sanger Institute COVID-19 Surveillance Team
EPI_ISL_762590, EPI_ISL_762591, EPI_ISL_762593, EPI_ISL_762594, EPI_ISL_762595	Lighthouse Lab in Cambridge	Wellcome Sanger Institute for the COVID-19 Genomics UK (COG-UK) Consortium	Rob Howes, The Lighthouse Lab in Cambridge and Alex Alderton, Roberto Amato, Sonia Goncalves, Ewan Harrison, David K. Jackson, Ian Johnston, Dominic Kwiatkowski, Cordelia Langford, John Sillitoe on behalf of the Wellcome Sanger Institute COVID-19 Surveillance Team
EPI_ISL_762597	Lighthouse Lab in Glasgow	Wellcome Sanger Institute for the COVID-19 Genomics UK (COG-UK) Consortium	Harper VanSteenhouse, Yumi Kasai, David Gray, Carol Clugston, Anna Dominiczak and Alex Alderton, Roberto Amato, Sonia Goncalves, Ewan Harrison, David K. Jackson, Ian Johnston, Dominic Kwiatkowski, Cordelia Langford, John Sillitoe on behalf of the Wellcome Sanger Institute COVID-19 Surveillance Team
EPI_ISL_762599	Lighthouse Lab in Alderley Park	Wellcome Sanger Institute for the COVID-19 Genomics UK (COG-UK) Consortium	Jacquelyn Wynn, Mairead Hyland, The Lighthouse Lab in Alderley Park and Alex Alderton, Roberto Amato, Sonia Goncalves, Ewan Harrison, David K. Jackson, Ian Johnston, Dominic Kwiatkowski, Cordelia Langford, John Sillitoe on behalf of the Wellcome Sanger Institute COVID-19 Surveillance Team
EPI_ISL_762600, EPI_ISL_762601, EPI_ISL_762603	Lighthouse Lab in Cambridge	Wellcome Sanger Institute for the COVID-19 Genomics UK (COG-UK) Consortium	Rob Howes, The Lighthouse Lab in Cambridge and Alex Alderton, Roberto Amato, Sonia Goncalves, Ewan Harrison, David K. Jackson, Ian Johnston, Dominic Kwiatkowski, Cordelia Langford, John Sillitoe on behalf of the Wellcome Sanger Institute COVID-19 Surveillance Team
EPI_ISL_762604	Lighthouse Lab in Glasgow	Wellcome Sanger Institute for the COVID-19 Genomics UK (COG-UK) Consortium	Harper VanSteenhouse, Yumi Kasai, David Gray, Carol Clugston, Anna Dominiczak and Alex Alderton, Roberto Amato, Sonia Goncalves, Ewan Harrison, David K. Jackson, Ian Johnston, Dominic Kwiatkowski, Cordelia Langford, John Sillitoe on behalf of the Wellcome Sanger Institute COVID-19 Surveillance Team
EPI_ISL_762605, EPI_ISL_762606	Lighthouse Lab in Alderley Park	Wellcome Sanger Institute for the COVID-19 Genomics UK (COG-UK) Consortium	Jacquelyn Wynn, Mairead Hyland, The Lighthouse Lab in Alderley Park and Alex Alderton, Roberto Amato, Sonia Goncalves, Ewan Harrison, David K. Jackson, Ian Johnston, Dominic Kwiatkowski, Cordelia Langford, John Sillitoe on behalf of the Wellcome Sanger Institute COVID-19 Surveillance Team
EPI_ISL_762608, EPI_ISL_762611, EPI_ISL_762612, EPI_ISL_762613	Lighthouse Lab in Cambridge	Wellcome Sanger Institute for the COVID-19 Genomics UK (COG-UK) Consortium	Rob Howes, The Lighthouse Lab in Cambridge and Alex Alderton, Roberto Amato, Sonia Goncalves, Ewan Harrison, David K. Jackson, Ian Johnston, Dominic Kwiatkowski, Cordelia Langford, John Sillitoe on behalf of the Wellcome Sanger Institute COVID-19 Surveillance Team
EPI_ISL_762614	Lighthouse Lab in Glasgow	Wellcome Sanger Institute for the COVID-19 Genomics UK (COG-UK) Consortium	Harper VanSteenhouse, Yumi Kasai, David Gray, Carol Clugston, Anna Dominiczak and Alex Alderton, Roberto Amato, Sonia Goncalves, Ewan Harrison, David K. Jackson, Ian Johnston, Dominic Kwiatkowski, Cordelia Langford, John Sillitoe on behalf of the Wellcome Sanger Institute COVID-19 Surveillance Team
EPI_ISL_762615, EPI_ISL_762616	Lighthouse Lab in Cambridge	Wellcome Sanger Institute for the COVID-19 Genomics UK (COG-UK) Consortium	Rob Howes, The Lighthouse Lab in Cambridge and Alex Alderton, Roberto Amato, Sonia Goncalves, Ewan Harrison, David K. Jackson, Ian Johnston, Dominic Kwiatkowski, Cordelia Langford, John Sillitoe on behalf of the Wellcome Sanger Institute COVID-19 Surveillance Team
EPI_ISL_762617	Lighthouse Lab in Alderley Park	Wellcome Sanger Institute for the COVID-19 Genomics UK (COG-UK) Consortium	Jacquelyn Wynn, Mairead Hyland, The Lighthouse Lab in Alderley Park and Alex Alderton, Roberto Amato, Sonia Goncalves, Ewan Harrison, David K. Jackson, Ian Johnston, Dominic Kwiatkowski, Cordelia Langford, John Sillitoe on behalf of the Wellcome Sanger Institute COVID-19 Surveillance Team
EPI_ISL_762618	Lighthouse Lab in Glasgow	Wellcome Sanger Institute for the COVID-19 Genomics UK (COG-UK) Consortium	Harper VanSteenhouse, Yumi Kasai, David Gray, Carol Clugston, Anna Dominiczak and Alex Alderton, Roberto Amato, Sonia Goncalves, Ewan Harrison, David K. Jackson, Ian Johnston, Dominic Kwiatkowski, Cordelia Langford, John Sillitoe on behalf of the Wellcome Sanger Institute COVID-19 Surveillance Team
EPI_ISL_762619	Lighthouse Lab in Cambridge	Wellcome Sanger Institute for the COVID-19 Genomics UK (COG-UK) Consortium	Rob Howes, The Lighthouse Lab in Cambridge and Alex Alderton, Roberto Amato, Sonia Goncalves, Ewan Harrison, David K. Jackson, Ian Johnston, Dominic Kwiatkowski, Cordelia Langford, John Sillitoe on behalf of the Wellcome Sanger Institute COVID-19 Surveillance Team
EPI_ISL_762620	Lighthouse Lab in Alderley Park	Wellcome Sanger Institute for the COVID-19 Genomics UK (COG-UK) Consortium	Jacquelyn Wynn, Mairead Hyland, The Lighthouse Lab in Alderley Park and Alex Alderton, Roberto Amato, Sonia Goncalves, Ewan Harrison, David K. Jackson, Ian Johnston, Dominic Kwiatkowski, Cordelia Langford, John Sillitoe on behalf of the Wellcome Sanger Institute COVID-19 Surveillance Team
EPI_ISL_762622	Lighthouse Lab in Glasgow	Wellcome Sanger Institute for the COVID-19 Genomics UK (COG-UK) Consortium	Harper VanSteenhouse, Yumi Kasai, David Gray, Carol Clugston, Anna Dominiczak and Alex Alderton, Roberto Amato, Sonia Goncalves, Ewan Harrison, David K. Jackson, Ian Johnston, Dominic Kwiatkowski, Cordelia Langford, John Sillitoe on behalf of the Wellcome Sanger Institute COVID-19 Surveillance Team
EPI_ISL_762623, EPI_ISL_762624	Lighthouse Lab in Cambridge	Wellcome Sanger Institute for the COVID-19 Genomics UK (COG-UK) Consortium	Rob Howes, The Lighthouse Lab in Cambridge and Alex Alderton, Roberto Amato, Sonia Goncalves, Ewan Harrison, David K. Jackson, Ian Johnston, Dominic Kwiatkowski, Cordelia Langford, John Sillitoe on behalf of the Wellcome Sanger Institute COVID-19 Surveillance Team
EPI_ISL_762625	Lighthouse Lab in Alderley Park	Wellcome Sanger Institute for the COVID-19 Genomics UK (COG-UK) Consortium	Jacquelyn Wynn, Mairead Hyland, The Lighthouse Lab in Alderley Park and Alex Alderton, Roberto Amato, Sonia Goncalves, Ewan Harrison, David K. Jackson, Ian Johnston, Dominic Kwiatkowski, Cordelia Langford, John Sillitoe on behalf of the Wellcome Sanger Institute COVID-19 Surveillance Team
EPI_ISL_762626, EPI_ISL_762627	Lighthouse Lab in Cambridge	Wellcome Sanger Institute for the COVID-19 Genomics UK (COG-UK) Consortium	Rob Howes, The Lighthouse Lab in Cambridge and Alex Alderton, Roberto Amato, Sonia Goncalves, Ewan Harrison, David K. Jackson, Ian Johnston, Dominic Kwiatkowski, Cordelia Langford, John Sillitoe on behalf of the Wellcome Sanger Institute COVID-19 Surveillance Team
EPI_ISL_762630, EPI_ISL_762633	Lighthouse Lab in Glasgow	Wellcome Sanger Institute for the COVID-19 Genomics UK (COG-UK) Consortium	Harper VanSteenhouse, Yumi Kasai, David Gray, Carol Clugston, Anna Dominiczak and Alex Alderton, Roberto Amato, Sonia Goncalves, Ewan Harrison, David K. Jackson, Ian Johnston, Dominic Kwiatkowski, Cordelia Langford, John Sillitoe on behalf of the Wellcome Sanger Institute COVID-19 Surveillance Team
EPI_ISL_762637	Lighthouse Lab in Cambridge	Wellcome Sanger Institute for the COVID-19 Genomics UK (COG-UK) Consortium	Rob Howes, The Lighthouse Lab in Cambridge and Alex Alderton, Roberto Amato, Sonia Goncalves, Ewan Harrison, David K. Jackson, Ian Johnston, Dominic Kwiatkowski, Cordelia Langford, John Sillitoe on behalf of the Wellcome Sanger Institute COVID-19 Surveillance Team

EPI_ISL_762638	Lighthouse Lab in Alderley Park	Wellcome Sanger Institute for the COVID-19 Genomics UK (COG-UK) Consortium	Jacquelyn Wynn, Mairead Hyland, The Lighthouse Lab in Alderley Park and Alex Alderton, Roberto Amato, Sonia Goncalves, Ewan Harrison, David K. Jackson, Ian Johnston, Dominic Kwiatkowski, Cordelia Langford, John Sillitoe on behalf of the Wellcome Sanger Institute COVID-19 Surveillance Team
EPI_ISL_762639	Lighthouse Lab in Glasgow	Wellcome Sanger Institute for the COVID-19 Genomics UK (COG-UK) Consortium	Harper VanSteenhouse, Yumi Kasai, David Gray, Carol Clugston, Anna Dominiczak and Alex Alderton, Roberto Amato, Sonia Goncalves, Ewan Harrison, David K. Jackson, Ian Johnston, Dominic Kwiatkowski, Cordelia Langford, John Sillitoe on behalf of the Wellcome Sanger Institute COVID-19 Surveillance Team
EPI_ISL_762642, EPI_ISL_762643	Lighthouse Lab in Alderley Park	Wellcome Sanger Institute for the COVID-19 Genomics UK (COG-UK) Consortium	Jacquelyn Wynn, Mairead Hyland, The Lighthouse Lab in Alderley Park and Alex Alderton, Roberto Amato, Sonia Goncalves, Ewan Harrison, David K. Jackson, Ian Johnston, Dominic Kwiatkowski, Cordelia Langford, John Sillitoe on behalf of the Wellcome Sanger Institute COVID-19 Surveillance Team
EPI_ISL_762645	Lighthouse Lab in Cambridge	Wellcome Sanger Institute for the COVID-19 Genomics UK (COG-UK) Consortium	Rob Howes, The Lighthouse Lab in Cambridge and Alex Alderton, Roberto Amato, Sonia Goncalves, Ewan Harrison, David K. Jackson, Ian Johnston, Dominic Kwiatkowski, Cordelia Langford, John Sillitoe on behalf of the Wellcome Sanger Institute COVID-19 Surveillance Team
EPI_ISL_762646	Lighthouse Lab in Glasgow	Wellcome Sanger Institute for the COVID-19 Genomics UK (COG-UK) Consortium	Harper VanSteenhouse, Yumi Kasai, David Gray, Carol Clugston, Anna Dominiczak and Alex Alderton, Roberto Amato, Sonia Goncalves, Ewan Harrison, David K. Jackson, Ian Johnston, Dominic Kwiatkowski, Cordelia Langford, John Sillitoe on behalf of the Wellcome Sanger Institute COVID-19 Surveillance Team
EPI_ISL_762647	Lighthouse Lab in Alderley Park	Wellcome Sanger Institute for the COVID-19 Genomics UK (COG-UK) Consortium	Jacquelyn Wynn, Mairead Hyland, The Lighthouse Lab in Alderley Park and Alex Alderton, Roberto Amato, Sonia Goncalves, Ewan Harrison, David K. Jackson, Ian Johnston, Dominic Kwiatkowski, Cordelia Langford, John Sillitoe on behalf of the Wellcome Sanger Institute COVID-19 Surveillance Team
EPI_ISL_762648	Lighthouse Lab in Glasgow	Wellcome Sanger Institute for the COVID-19 Genomics UK (COG-UK) Consortium	Harper VanSteenhouse, Yumi Kasai, David Gray, Carol Clugston, Anna Dominiczak and Alex Alderton, Roberto Amato, Sonia Goncalves, Ewan Harrison, David K. Jackson, Ian Johnston, Dominic Kwiatkowski, Cordelia Langford, John Sillitoe on behalf of the Wellcome Sanger Institute COVID-19 Surveillance Team
EPI_ISL_762649, EPI_ISL_762650, EPI_ISL_762651	Lighthouse Lab in Cambridge	Wellcome Sanger Institute for the COVID-19 Genomics UK (COG-UK) Consortium	Rob Howes, The Lighthouse Lab in Cambridge and Alex Alderton, Roberto Amato, Sonia Goncalves, Ewan Harrison, David K. Jackson, Ian Johnston, Dominic Kwiatkowski, Cordelia Langford, John Sillitoe on behalf of the Wellcome Sanger Institute COVID-19 Surveillance Team
EPI_ISL_762653, EPI_ISL_762654	Lighthouse Lab in Glasgow	Wellcome Sanger Institute for the COVID-19 Genomics UK (COG-UK) Consortium	Harper VanSteenhouse, Yumi Kasai, David Gray, Carol Clugston, Anna Dominiczak and Alex Alderton, Roberto Amato, Sonia Goncalves, Ewan Harrison, David K. Jackson, Ian Johnston, Dominic Kwiatkowski, Cordelia Langford, John Sillitoe on behalf of the Wellcome Sanger Institute COVID-19 Surveillance Team
EPI_ISL_762655	Lighthouse Lab in Alderley Park	Wellcome Sanger Institute for the COVID-19 Genomics UK (COG-UK) Consortium	Jacquelyn Wynn, Mairead Hyland, The Lighthouse Lab in Alderley Park and Alex Alderton, Roberto Amato, Sonia Goncalves, Ewan Harrison, David K. Jackson, Ian Johnston, Dominic Kwiatkowski, Cordelia Langford, John Sillitoe on behalf of the Wellcome Sanger Institute COVID-19 Surveillance Team
EPI_ISL_762656, EPI_ISL_762657	Lighthouse Lab in Glasgow	Wellcome Sanger Institute for the COVID-19 Genomics UK (COG-UK) Consortium	Harper VanSteenhouse, Yumi Kasai, David Gray, Carol Clugston, Anna Dominiczak and Alex Alderton, Roberto Amato, Sonia Goncalves, Ewan Harrison, David K. Jackson, Ian Johnston, Dominic Kwiatkowski, Cordelia Langford, John Sillitoe on behalf of the Wellcome Sanger Institute COVID-19 Surveillance Team
EPI_ISL_762658	Lighthouse Lab in Cambridge	Wellcome Sanger Institute for the COVID-19 Genomics UK (COG-UK) Consortium	Rob Howes, The Lighthouse Lab in Cambridge and Alex Alderton, Roberto Amato, Sonia Goncalves, Ewan Harrison, David K. Jackson, Ian Johnston, Dominic Kwiatkowski, Cordelia Langford, John Sillitoe on behalf of the Wellcome Sanger Institute COVID-19 Surveillance Team
EPI_ISL_762659	Lighthouse Lab in Alderley Park	Wellcome Sanger Institute for the COVID-19 Genomics UK (COG-UK) Consortium	Jacquelyn Wynn, Mairead Hyland, The Lighthouse Lab in Alderley Park and Alex Alderton, Roberto Amato, Sonia Goncalves, Ewan Harrison, David K. Jackson, Ian Johnston, Dominic Kwiatkowski, Cordelia Langford, John Sillitoe on behalf of the Wellcome Sanger Institute COVID-19 Surveillance Team
EPI_ISL_762660	Lighthouse Lab in Cambridge	Wellcome Sanger Institute for the COVID-19 Genomics UK (COG-UK) Consortium	Rob Howes, The Lighthouse Lab in Cambridge and Alex Alderton, Roberto Amato, Sonia Goncalves, Ewan Harrison, David K. Jackson, Ian Johnston, Dominic Kwiatkowski, Cordelia Langford, John Sillitoe on behalf of the Wellcome Sanger Institute COVID-19 Surveillance Team
EPI_ISL_762661, EPI_ISL_762662, EPI_ISL_762663	Lighthouse Lab in Glasgow	Wellcome Sanger Institute for the COVID-19 Genomics UK (COG-UK) Consortium	Harper VanSteenhouse, Yumi Kasai, David Gray, Carol Clugston, Anna Dominiczak and Alex Alderton, Roberto Amato, Sonia Goncalves, Ewan Harrison, David K. Jackson, Ian Johnston, Dominic Kwiatkowski, Cordelia Langford, John Sillitoe on behalf of the Wellcome Sanger Institute COVID-19 Surveillance Team
EPI_ISL_762664, EPI_ISL_762665, EPI_ISL_762667, EPI_ISL_762670	Lighthouse Lab in Cambridge	Wellcome Sanger Institute for the COVID-19 Genomics UK (COG-UK) Consortium	Rob Howes, The Lighthouse Lab in Cambridge and Alex Alderton, Roberto Amato, Sonia Goncalves, Ewan Harrison, David K. Jackson, Ian Johnston, Dominic Kwiatkowski, Cordelia Langford, John Sillitoe on behalf of the Wellcome Sanger Institute COVID-19 Surveillance Team
EPI_ISL_762671, EPI_ISL_762672	Lighthouse Lab in Alderley Park	Wellcome Sanger Institute for the COVID-19 Genomics UK (COG-UK) Consortium	Jacquelyn Wynn, Mairead Hyland, The Lighthouse Lab in Alderley Park and Alex Alderton, Roberto Amato, Sonia Goncalves, Ewan Harrison, David K. Jackson, Ian Johnston, Dominic Kwiatkowski, Cordelia Langford, John Sillitoe on behalf of the Wellcome Sanger Institute COVID-19 Surveillance Team
EPI_ISL_762673	Lighthouse Lab in Cambridge	Wellcome Sanger Institute for the COVID-19 Genomics UK (COG-UK) Consortium	Rob Howes, The Lighthouse Lab in Cambridge and Alex Alderton, Roberto Amato, Sonia Goncalves, Ewan Harrison, David K. Jackson, Ian Johnston, Dominic Kwiatkowski, Cordelia Langford, John Sillitoe on behalf of the Wellcome Sanger Institute COVID-19 Surveillance Team
EPI_ISL_762676	Lighthouse Lab in Glasgow	Wellcome Sanger Institute for the COVID-19 Genomics UK (COG-UK) Consortium	Harper VanSteenhouse, Yumi Kasai, David Gray, Carol Clugston, Anna Dominiczak and Alex Alderton, Roberto Amato, Sonia Goncalves, Ewan Harrison, David K. Jackson, Ian Johnston, Dominic Kwiatkowski, Cordelia Langford, John Sillitoe on behalf of the Wellcome Sanger Institute COVID-19 Surveillance Team
EPI_ISL_762677	Lighthouse Lab in Cambridge	Wellcome Sanger Institute for the COVID-19 Genomics UK (COG-UK) Consortium	Rob Howes, The Lighthouse Lab in Cambridge and Alex Alderton, Roberto Amato, Sonia Goncalves, Ewan Harrison, David K. Jackson, Ian Johnston, Dominic Kwiatkowski, Cordelia Langford, John Sillitoe on behalf of the Wellcome Sanger Institute COVID-19 Surveillance Team
EPI_ISL_762678	Lighthouse Lab in Glasgow	Wellcome Sanger Institute for the COVID-19 Genomics UK (COG-UK) Consortium	Harper VanSteenhouse, Yumi Kasai, David Gray, Carol Clugston, Anna Dominiczak and Alex Alderton, Roberto Amato, Sonia Goncalves, Ewan Harrison, David K. Jackson, Ian Johnston, Dominic Kwiatkowski, Cordelia Langford, John Sillitoe on behalf of the Wellcome Sanger Institute COVID-19 Surveillance Team
EPI_ISL_762680	Lighthouse Lab in Cambridge	Wellcome Sanger Institute for the COVID-19 Genomics UK (COG-UK) Consortium	Rob Howes, The Lighthouse Lab in Cambridge and Alex Alderton, Roberto Amato, Sonia Goncalves, Ewan Harrison, David K. Jackson, Ian Johnston, Dominic Kwiatkowski, Cordelia Langford, John Sillitoe on behalf of the Wellcome Sanger Institute COVID-19 Surveillance Team
EPI_ISL_762683	Lighthouse Lab in Alderley Park	Wellcome Sanger Institute for the COVID-19 Genomics UK (COG-UK) Consortium	Jacquelyn Wynn, Mairead Hyland, The Lighthouse Lab in Alderley Park and Alex Alderton, Roberto Amato, Sonia Goncalves, Ewan Harrison, David K. Jackson, Ian Johnston, Dominic Kwiatkowski, Cordelia Langford, John Sillitoe on behalf of the Wellcome Sanger Institute COVID-19 Surveillance Team
EPI_ISL_762684, EPI_ISL_762685	Lighthouse Lab in Cambridge	Wellcome Sanger Institute for the COVID-19 Genomics UK (COG-UK) Consortium	Rob Howes, The Lighthouse Lab in Cambridge and Alex Alderton, Roberto Amato, Sonia Goncalves, Ewan Harrison, David K. Jackson, Ian Johnston, Dominic Kwiatkowski, Cordelia Langford, John Sillitoe on behalf of the Wellcome Sanger Institute COVID-19 Surveillance Team
EPI_ISL_762686	Lighthouse Lab in Alderley Park	Wellcome Sanger Institute for the COVID-19 Genomics UK (COG-UK) Consortium	Jacquelyn Wynn, Mairead Hyland, The Lighthouse Lab in Alderley Park and Alex Alderton, Roberto Amato, Sonia Goncalves, Ewan Harrison, David K. Jackson, Ian Johnston, Dominic Kwiatkowski, Cordelia Langford, John Sillitoe on behalf of the Wellcome Sanger Institute COVID-19 Surveillance Team
EPI_ISL_762687, EPI_ISL_762689	Lighthouse Lab in Cambridge	Wellcome Sanger Institute for the COVID-19 Genomics UK (COG-UK) Consortium	Rob Howes, The Lighthouse Lab in Cambridge and Alex Alderton, Roberto Amato, Sonia Goncalves, Ewan Harrison, David K. Jackson, Ian Johnston, Dominic Kwiatkowski, Cordelia Langford, John Sillitoe on behalf of the Wellcome Sanger Institute COVID-19 Surveillance Team
EPI_ISL_762690	Lighthouse Lab in Glasgow	Wellcome Sanger Institute for the COVID-19 Genomics UK (COG-UK) Consortium	Harper VanSteenhouse, Yumi Kasai, David Gray, Carol Clugston, Anna Dominiczak and Alex Alderton, Roberto Amato, Sonia Goncalves, Ewan Harrison, David K. Jackson, Ian Johnston, Dominic Kwiatkowski, Cordelia Langford, John Sillitoe on behalf of the Wellcome Sanger Institute COVID-19 Surveillance Team
EPI_ISL_762691, EPI_ISL_762693	Lighthouse Lab in Alderley Park	Wellcome Sanger Institute for the COVID-19 Genomics UK (COG-UK) Consortium	Jacquelyn Wynn, Mairead Hyland, The Lighthouse Lab in Alderley Park and Alex Alderton, Roberto Amato, Sonia Goncalves, Ewan Harrison, David K. Jackson, Ian Johnston, Dominic Kwiatkowski, Cordelia Langford, John Sillitoe on behalf of the Wellcome Sanger Institute COVID-19 Surveillance Team
EPI_ISL_762694	Lighthouse Lab in Glasgow	Wellcome Sanger Institute for the COVID-19 Genomics UK (COG-UK) Consortium	Harper VanSteenhouse, Yumi Kasai, David Gray, Carol Clugston, Anna Dominiczak and Alex Alderton, Roberto Amato, Sonia Goncalves, Ewan Harrison, David K. Jackson, Ian Johnston, Dominic Kwiatkowski, Cordelia Langford, John Sillitoe on behalf of the Wellcome Sanger Institute COVID-19 Surveillance Team
EPI_ISL_762695, EPI_ISL_762696, EPI_ISL_762699, EPI_ISL_762700	Lighthouse Lab in Cambridge	Wellcome Sanger Institute for the COVID-19 Genomics UK (COG-UK) Consortium	Rob Howes, The Lighthouse Lab in Cambridge and Alex Alderton, Roberto Amato, Sonia Goncalves, Ewan Harrison, David K. Jackson, Ian Johnston, Dominic Kwiatkowski, Cordelia Langford, John Sillitoe on behalf of the Wellcome Sanger Institute COVID-19 Surveillance Team
EPI_ISL_762701	Lighthouse Lab in Glasgow	Wellcome Sanger Institute for the COVID-19 Genomics UK (COG-UK) Consortium	Harper VanSteenhouse, Yumi Kasai, David Gray, Carol Clugston, Anna Dominiczak and Alex Alderton, Roberto Amato, Sonia Goncalves, Ewan Harrison, David K. Jackson, Ian Johnston, Dominic Kwiatkowski, Cordelia Langford, John Sillitoe on behalf of the Wellcome Sanger Institute COVID-19 Surveillance

			Team
EPI_ISL_762702, EPI_ISL_762703	Lighthouse Lab in Alderley Park	Wellcome Sanger Institute for the COVID-19 Genomics UK (COG-UK) Consortium	Jacquelyn Wynn, Mairead Hyland, The Lighthouse Lab in Alderley Park and Alex Alderton, Roberto Amato, Sonia Goncalves, Ewan Harrison, David K. Jackson, Ian Johnston, Dominic Kwiatkowski, Cordelia Langford, John Sillitoe on behalf of the Wellcome Sanger Institute COVID-19 Surveillance Team
EPI_ISL_762705	Lighthouse Lab in Cambridge	Wellcome Sanger Institute for the COVID-19 Genomics UK (COG-UK) Consortium	Rob Howes, The Lighthouse Lab in Cambridge and Alex Alderton, Roberto Amato, Sonia Goncalves, Ewan Harrison, David K. Jackson, Ian Johnston, Dominic Kwiatkowski, Cordelia Langford, John Sillitoe on behalf of the Wellcome Sanger Institute COVID-19 Surveillance Team
EPI_ISL_762706	Lighthouse Lab in Alderley Park	Wellcome Sanger Institute for the COVID-19 Genomics UK (COG-UK) Consortium	Jacquelyn Wynn, Mairead Hyland, The Lighthouse Lab in Alderley Park and Alex Alderton, Roberto Amato, Sonia Goncalves, Ewan Harrison, David K. Jackson, Ian Johnston, Dominic Kwiatkowski, Cordelia Langford, John Sillitoe on behalf of the Wellcome Sanger Institute COVID-19 Surveillance Team
EPI_ISL_762708	Lighthouse Lab in Glasgow	Wellcome Sanger Institute for the COVID-19 Genomics UK (COG-UK) Consortium	Harper VanSteenhouse, Yumi Kasai, David Gray, Carol Clugston, Anna Dominiczak and Alex Alderton, Roberto Amato, Sonia Goncalves, Ewan Harrison, David K. Jackson, Ian Johnston, Dominic Kwiatkowski, Cordelia Langford, John Sillitoe on behalf of the Wellcome Sanger Institute COVID-19 Surveillance Team
EPI_ISL_762709, EPI_ISL_762710, EPI_ISL_762712	Lighthouse Lab in Alderley Park	Wellcome Sanger Institute for the COVID-19 Genomics UK (COG-UK) Consortium	Jacquelyn Wynn, Mairead Hyland, The Lighthouse Lab in Alderley Park and Alex Alderton, Roberto Amato, Sonia Goncalves, Ewan Harrison, David K. Jackson, Ian Johnston, Dominic Kwiatkowski, Cordelia Langford, John Sillitoe on behalf of the Wellcome Sanger Institute COVID-19 Surveillance Team
EPI_ISL_762713	Lighthouse Lab in Cambridge	Wellcome Sanger Institute for the COVID-19 Genomics UK (COG-UK) Consortium	Rob Howes, The Lighthouse Lab in Cambridge and Alex Alderton, Roberto Amato, Sonia Goncalves, Ewan Harrison, David K. Jackson, Ian Johnston, Dominic Kwiatkowski, Cordelia Langford, John Sillitoe on behalf of the Wellcome Sanger Institute COVID-19 Surveillance Team
EPI_ISL_762714	Lighthouse Lab in Alderley Park	Wellcome Sanger Institute for the COVID-19 Genomics UK (COG-UK) Consortium	Jacquelyn Wynn, Mairead Hyland, The Lighthouse Lab in Alderley Park and Alex Alderton, Roberto Amato, Sonia Goncalves, Ewan Harrison, David K. Jackson, Ian Johnston, Dominic Kwiatkowski, Cordelia Langford, John Sillitoe on behalf of the Wellcome Sanger Institute COVID-19 Surveillance Team
EPI_ISL_762715, EPI_ISL_762718, EPI_ISL_762719, EPI_ISL_762721	Lighthouse Lab in Cambridge	Wellcome Sanger Institute for the COVID-19 Genomics UK (COG-UK) Consortium	Rob Howes, The Lighthouse Lab in Cambridge and Alex Alderton, Roberto Amato, Sonia Goncalves, Ewan Harrison, David K. Jackson, Ian Johnston, Dominic Kwiatkowski, Cordelia Langford, John Sillitoe on behalf of the Wellcome Sanger Institute COVID-19 Surveillance Team
EPI_ISL_762722, EPI_ISL_762724	Lighthouse Lab in Alderley Park	Wellcome Sanger Institute for the COVID-19 Genomics UK (COG-UK) Consortium	Jacquelyn Wynn, Mairead Hyland, The Lighthouse Lab in Alderley Park and Alex Alderton, Roberto Amato, Sonia Goncalves, Ewan Harrison, David K. Jackson, Ian Johnston, Dominic Kwiatkowski, Cordelia Langford, John Sillitoe on behalf of the Wellcome Sanger Institute COVID-19 Surveillance Team
EPI_ISL_762725, EPI_ISL_762727	Lighthouse Lab in Cambridge	Wellcome Sanger Institute for the COVID-19 Genomics UK (COG-UK) Consortium	Rob Howes, The Lighthouse Lab in Cambridge and Alex Alderton, Roberto Amato, Sonia Goncalves, Ewan Harrison, David K. Jackson, Ian Johnston, Dominic Kwiatkowski, Cordelia Langford, John Sillitoe on behalf of the Wellcome Sanger Institute COVID-19 Surveillance Team
EPI_ISL_762728	Lighthouse Lab in Glasgow	Wellcome Sanger Institute for the COVID-19 Genomics UK (COG-UK) Consortium	Harper VanSteenhouse, Yumi Kasai, David Gray, Carol Clugston, Anna Dominiczak and Alex Alderton, Roberto Amato, Sonia Goncalves, Ewan Harrison, David K. Jackson, Ian Johnston, Dominic Kwiatkowski, Cordelia Langford, John Sillitoe on behalf of the Wellcome Sanger Institute COVID-19 Surveillance Team
EPI_ISL_762729	Lighthouse Lab in Cambridge	Wellcome Sanger Institute for the COVID-19 Genomics UK (COG-UK) Consortium	Rob Howes, The Lighthouse Lab in Cambridge and Alex Alderton, Roberto Amato, Sonia Goncalves, Ewan Harrison, David K. Jackson, Ian Johnston, Dominic Kwiatkowski, Cordelia Langford, John Sillitoe on behalf of the Wellcome Sanger Institute COVID-19 Surveillance Team
EPI_ISL_762730	Lighthouse Lab in Alderley Park	Wellcome Sanger Institute for the COVID-19 Genomics UK (COG-UK) Consortium	Jacquelyn Wynn, Mairead Hyland, The Lighthouse Lab in Alderley Park and Alex Alderton, Roberto Amato, Sonia Goncalves, Ewan Harrison, David K. Jackson, Ian Johnston, Dominic Kwiatkowski, Cordelia Langford, John Sillitoe on behalf of the Wellcome Sanger Institute COVID-19 Surveillance Team
EPI_ISL_762731, EPI_ISL_762732	Lighthouse Lab in Glasgow	Wellcome Sanger Institute for the COVID-19 Genomics UK (COG-UK) Consortium	Harper VanSteenhouse, Yumi Kasai, David Gray, Carol Clugston, Anna Dominiczak and Alex Alderton, Roberto Amato, Sonia Goncalves, Ewan Harrison, David K. Jackson, Ian Johnston, Dominic Kwiatkowski, Cordelia Langford, John Sillitoe on behalf of the Wellcome Sanger Institute COVID-19 Surveillance Team
EPI_ISL_762735	Lighthouse Lab in Alderley Park	Wellcome Sanger Institute for the COVID-19 Genomics UK (COG-UK) Consortium	Jacquelyn Wynn, Mairead Hyland, The Lighthouse Lab in Alderley Park and Alex Alderton, Roberto Amato, Sonia Goncalves, Ewan Harrison, David K. Jackson, Ian Johnston, Dominic Kwiatkowski, Cordelia Langford, John Sillitoe on behalf of the Wellcome Sanger Institute COVID-19 Surveillance Team
EPI_ISL_762736	Lighthouse Lab in Cambridge	Wellcome Sanger Institute for the COVID-19 Genomics UK (COG-UK) Consortium	Rob Howes, The Lighthouse Lab in Cambridge and Alex Alderton, Roberto Amato, Sonia Goncalves, Ewan Harrison, David K. Jackson, Ian Johnston, Dominic Kwiatkowski, Cordelia Langford, John Sillitoe on behalf of the Wellcome Sanger Institute COVID-19 Surveillance Team
EPI_ISL_762739	Lighthouse Lab in Glasgow	Wellcome Sanger Institute for the COVID-19 Genomics UK (COG-UK) Consortium	Harper VanSteenhouse, Yumi Kasai, David Gray, Carol Clugston, Anna Dominiczak and Alex Alderton, Roberto Amato, Sonia Goncalves, Ewan Harrison, David K. Jackson, Ian Johnston, Dominic Kwiatkowski, Cordelia Langford, John Sillitoe on behalf of the Wellcome Sanger Institute COVID-19 Surveillance Team
EPI_ISL_762740, EPI_ISL_762741, EPI_ISL_762742	Lighthouse Lab in Cambridge	Wellcome Sanger Institute for the COVID-19 Genomics UK (COG-UK) Consortium	Rob Howes, The Lighthouse Lab in Cambridge and Alex Alderton, Roberto Amato, Sonia Goncalves, Ewan Harrison, David K. Jackson, Ian Johnston, Dominic Kwiatkowski, Cordelia Langford, John Sillitoe on behalf of the Wellcome Sanger Institute COVID-19 Surveillance Team
EPI_ISL_762743, EPI_ISL_762744	Lighthouse Lab in Glasgow	Wellcome Sanger Institute for the COVID-19 Genomics UK (COG-UK) Consortium	Harper VanSteenhouse, Yumi Kasai, David Gray, Carol Clugston, Anna Dominiczak and Alex Alderton, Roberto Amato, Sonia Goncalves, Ewan Harrison, David K. Jackson, Ian Johnston, Dominic Kwiatkowski, Cordelia Langford, John Sillitoe on behalf of the Wellcome Sanger Institute COVID-19 Surveillance Team
EPI_ISL_762745	Lighthouse Lab in Cambridge	Wellcome Sanger Institute for the COVID-19 Genomics UK (COG-UK) Consortium	Rob Howes, The Lighthouse Lab in Cambridge and Alex Alderton, Roberto Amato, Sonia Goncalves, Ewan Harrison, David K. Jackson, Ian Johnston, Dominic Kwiatkowski, Cordelia Langford, John Sillitoe on behalf of the Wellcome Sanger Institute COVID-19 Surveillance Team
EPI_ISL_762746	Lighthouse Lab in Glasgow	Wellcome Sanger Institute for the COVID-19 Genomics UK (COG-UK) Consortium	Harper VanSteenhouse, Yumi Kasai, David Gray, Carol Clugston, Anna Dominiczak and Alex Alderton, Roberto Amato, Sonia Goncalves, Ewan Harrison, David K. Jackson, Ian Johnston, Dominic Kwiatkowski, Cordelia Langford, John Sillitoe on behalf of the Wellcome Sanger Institute COVID-19 Surveillance Team
EPI_ISL_762747, EPI_ISL_762748	Lighthouse Lab in Cambridge	Wellcome Sanger Institute for the COVID-19 Genomics UK (COG-UK) Consortium	Rob Howes, The Lighthouse Lab in Cambridge and Alex Alderton, Roberto Amato, Sonia Goncalves, Ewan Harrison, David K. Jackson, Ian Johnston, Dominic Kwiatkowski, Cordelia Langford, John Sillitoe on behalf of the Wellcome Sanger Institute COVID-19 Surveillance Team
EPI_ISL_762749	Lighthouse Lab in Glasgow	Wellcome Sanger Institute for the COVID-19 Genomics UK (COG-UK) Consortium	Harper VanSteenhouse, Yumi Kasai, David Gray, Carol Clugston, Anna Dominiczak and Alex Alderton, Roberto Amato, Sonia Goncalves, Ewan Harrison, David K. Jackson, Ian Johnston, Dominic Kwiatkowski, Cordelia Langford, John Sillitoe on behalf of the Wellcome Sanger Institute COVID-19 Surveillance Team
EPI_ISL_762750	Lighthouse Lab in Alderley Park	Wellcome Sanger Institute for the COVID-19 Genomics UK (COG-UK) Consortium	Jacquelyn Wynn, Mairead Hyland, The Lighthouse Lab in Alderley Park and Alex Alderton, Roberto Amato, Sonia Goncalves, Ewan Harrison, David K. Jackson, Ian Johnston, Dominic Kwiatkowski, Cordelia Langford, John Sillitoe on behalf of the Wellcome Sanger Institute COVID-19 Surveillance Team
EPI_ISL_762751, EPI_ISL_762752, EPI_ISL_762753	Lighthouse Lab in Cambridge	Wellcome Sanger Institute for the COVID-19 Genomics UK (COG-UK) Consortium	Rob Howes, The Lighthouse Lab in Cambridge and Alex Alderton, Roberto Amato, Sonia Goncalves, Ewan Harrison, David K. Jackson, Ian Johnston, Dominic Kwiatkowski, Cordelia Langford, John Sillitoe on behalf of the Wellcome Sanger Institute COVID-19 Surveillance Team
EPI_ISL_762754	Lighthouse Lab in Alderley Park	Wellcome Sanger Institute for the COVID-19 Genomics UK (COG-UK) Consortium	Jacquelyn Wynn, Mairead Hyland, The Lighthouse Lab in Alderley Park and Alex Alderton, Roberto Amato, Sonia Goncalves, Ewan Harrison, David K. Jackson, Ian Johnston, Dominic Kwiatkowski, Cordelia Langford, John Sillitoe on behalf of the Wellcome Sanger Institute COVID-19 Surveillance Team
EPI_ISL_762757	Lighthouse Lab in Cambridge	Wellcome Sanger Institute for the COVID-19 Genomics UK (COG-UK) Consortium	Rob Howes, The Lighthouse Lab in Cambridge and Alex Alderton, Roberto Amato, Sonia Goncalves, Ewan Harrison, David K. Jackson, Ian Johnston, Dominic Kwiatkowski, Cordelia Langford, John Sillitoe on behalf of the Wellcome Sanger Institute COVID-19 Surveillance Team
EPI_ISL_762759, EPI_ISL_762760, EPI_ISL_762761	Lighthouse Lab in Glasgow	Wellcome Sanger Institute for the COVID-19 Genomics UK (COG-UK) Consortium	Harper VanSteenhouse, Yumi Kasai, David Gray, Carol Clugston, Anna Dominiczak and Alex Alderton, Roberto Amato, Sonia Goncalves, Ewan Harrison, David K. Jackson, Ian Johnston, Dominic Kwiatkowski, Cordelia Langford, John Sillitoe on behalf of the Wellcome Sanger Institute COVID-19 Surveillance Team
EPI_ISL_762762	Lighthouse Lab in Cambridge	Wellcome Sanger Institute for the COVID-19 Genomics UK (COG-UK) Consortium	Rob Howes, The Lighthouse Lab in Cambridge and Alex Alderton, Roberto Amato, Sonia Goncalves, Ewan Harrison, David K. Jackson, Ian Johnston, Dominic Kwiatkowski, Cordelia Langford, John Sillitoe on behalf of the Wellcome Sanger Institute COVID-19 Surveillance Team
EPI_ISL_762763, EPI_ISL_762764	Lighthouse Lab in Alderley Park	Wellcome Sanger Institute for the COVID-19 Genomics UK (COG-UK) Consortium	Jacquelyn Wynn, Mairead Hyland, The Lighthouse Lab in Alderley Park and Alex Alderton, Roberto Amato, Sonia Goncalves, Ewan Harrison, David K. Jackson, Ian Johnston, Dominic Kwiatkowski, Cordelia Langford, John Sillitoe on behalf of the Wellcome Sanger Institute COVID-19 Surveillance Team
EPI_ISL_762766	Lighthouse Lab in Glasgow	Wellcome Sanger Institute for the COVID-19 Genomics UK (COG-UK) Consortium	Harper VanSteenhouse, Yumi Kasai, David Gray, Carol Clugston, Anna Dominiczak and Alex Alderton, Roberto Amato, Sonia Goncalves, Ewan Harrison, David K. Jackson, Ian Johnston, Dominic Kwiatkowski, Cordelia Langford, John Sillitoe on behalf of the Wellcome Sanger Institute COVID-19 Surveillance Team

EPI_ISL_762767, EPI_ISL_762769	Lighthouse Lab in Cambridge	Wellcome Sanger Institute for the COVID-19 Genomics UK (COG-UK) Consortium	Rob Howes, The Lighthouse Lab in Cambridge and Alex Alderton, Roberto Amato, Sonia Goncalves, Ewan Harrison, David K. Jackson, Ian Johnston, Dominic Kwiatkowski, Cordelia Langford, John Sillitoe on behalf of the Wellcome Sanger Institute COVID-19 Surveillance Team
EPI_ISL_762770, EPI_ISL_762773	Lighthouse Lab in Glasgow	Wellcome Sanger Institute for the COVID-19 Genomics UK (COG-UK) Consortium	Harper VanSteenhouse, Yumi Kasai, David Gray, Carol Clugston, Anna Dominiczak and Alex Alderton, Roberto Amato, Sonia Goncalves, Ewan Harrison, David K. Jackson, Ian Johnston, Dominic Kwiatkowski, Cordelia Langford, John Sillitoe on behalf of the Wellcome Sanger Institute COVID-19 Surveillance Team
EPI_ISL_762774	Lighthouse Lab in Cambridge	Wellcome Sanger Institute for the COVID-19 Genomics UK (COG-UK) Consortium	Rob Howes, The Lighthouse Lab in Cambridge and Alex Alderton, Roberto Amato, Sonia Goncalves, Ewan Harrison, David K. Jackson, Ian Johnston, Dominic Kwiatkowski, Cordelia Langford, John Sillitoe on behalf of the Wellcome Sanger Institute COVID-19 Surveillance Team
EPI_ISL_762775	Lighthouse Lab in Glasgow	Wellcome Sanger Institute for the COVID-19 Genomics UK (COG-UK) Consortium	Harper VanSteenhouse, Yumi Kasai, David Gray, Carol Clugston, Anna Dominiczak and Alex Alderton, Roberto Amato, Sonia Goncalves, Ewan Harrison, David K. Jackson, Ian Johnston, Dominic Kwiatkowski, Cordelia Langford, John Sillitoe on behalf of the Wellcome Sanger Institute COVID-19 Surveillance Team
EPI_ISL_762777	Lighthouse Lab in Alderley Park	Wellcome Sanger Institute for the COVID-19 Genomics UK (COG-UK) Consortium	Jacquelyn Wynn, Mairead Hyland, The Lighthouse Lab in Alderley Park and Alex Alderton, Roberto Amato, Sonia Goncalves, Ewan Harrison, David K. Jackson, Ian Johnston, Dominic Kwiatkowski, Cordelia Langford, John Sillitoe on behalf of the Wellcome Sanger Institute COVID-19 Surveillance Team
EPI_ISL_762778, EPI_ISL_762780, EPI_ISL_762782, EPI_ISL_762783, EPI_ISL_762784	Lighthouse Lab in Cambridge	Wellcome Sanger Institute for the COVID-19 Genomics UK (COG-UK) Consortium	Rob Howes, The Lighthouse Lab in Cambridge and Alex Alderton, Roberto Amato, Sonia Goncalves, Ewan Harrison, David K. Jackson, Ian Johnston, Dominic Kwiatkowski, Cordelia Langford, John Sillitoe on behalf of the Wellcome Sanger Institute COVID-19 Surveillance Team
EPI_ISL_762785	Lighthouse Lab in Glasgow	Wellcome Sanger Institute for the COVID-19 Genomics UK (COG-UK) Consortium	Harper VanSteenhouse, Yumi Kasai, David Gray, Carol Clugston, Anna Dominiczak and Alex Alderton, Roberto Amato, Sonia Goncalves, Ewan Harrison, David K. Jackson, Ian Johnston, Dominic Kwiatkowski, Cordelia Langford, John Sillitoe on behalf of the Wellcome Sanger Institute COVID-19 Surveillance Team
EPI_ISL_762786, EPI_ISL_762789	Lighthouse Lab in Alderley Park	Wellcome Sanger Institute for the COVID-19 Genomics UK (COG-UK) Consortium	Jacquelyn Wynn, Mairead Hyland, The Lighthouse Lab in Alderley Park and Alex Alderton, Roberto Amato, Sonia Goncalves, Ewan Harrison, David K. Jackson, Ian Johnston, Dominic Kwiatkowski, Cordelia Langford, John Sillitoe on behalf of the Wellcome Sanger Institute COVID-19 Surveillance Team
EPI_ISL_762790	Lighthouse Lab in Cambridge	Wellcome Sanger Institute for the COVID-19 Genomics UK (COG-UK) Consortium	Rob Howes, The Lighthouse Lab in Cambridge and Alex Alderton, Roberto Amato, Sonia Goncalves, Ewan Harrison, David K. Jackson, Ian Johnston, Dominic Kwiatkowski, Cordelia Langford, John Sillitoe on behalf of the Wellcome Sanger Institute COVID-19 Surveillance Team
EPI_ISL_762791	Lighthouse Lab in Alderley Park	Wellcome Sanger Institute for the COVID-19 Genomics UK (COG-UK) Consortium	Jacquelyn Wynn, Mairead Hyland, The Lighthouse Lab in Alderley Park and Alex Alderton, Roberto Amato, Sonia Goncalves, Ewan Harrison, David K. Jackson, Ian Johnston, Dominic Kwiatkowski, Cordelia Langford, John Sillitoe on behalf of the Wellcome Sanger Institute COVID-19 Surveillance Team
EPI_ISL_762792	Lighthouse Lab in Cambridge	Wellcome Sanger Institute for the COVID-19 Genomics UK (COG-UK) Consortium	Rob Howes, The Lighthouse Lab in Cambridge and Alex Alderton, Roberto Amato, Sonia Goncalves, Ewan Harrison, David K. Jackson, Ian Johnston, Dominic Kwiatkowski, Cordelia Langford, John Sillitoe on behalf of the Wellcome Sanger Institute COVID-19 Surveillance Team
EPI_ISL_762793	Lighthouse Lab in Alderley Park	Wellcome Sanger Institute for the COVID-19 Genomics UK (COG-UK) Consortium	Jacquelyn Wynn, Mairead Hyland, The Lighthouse Lab in Alderley Park and Alex Alderton, Roberto Amato, Sonia Goncalves, Ewan Harrison, David K. Jackson, Ian Johnston, Dominic Kwiatkowski, Cordelia Langford, John Sillitoe on behalf of the Wellcome Sanger Institute COVID-19 Surveillance Team
EPI_ISL_762794	Lighthouse Lab in Glasgow	Wellcome Sanger Institute for the COVID-19 Genomics UK (COG-UK) Consortium	Harper VanSteenhouse, Yumi Kasai, David Gray, Carol Clugston, Anna Dominiczak and Alex Alderton, Roberto Amato, Sonia Goncalves, Ewan Harrison, David K. Jackson, Ian Johnston, Dominic Kwiatkowski, Cordelia Langford, John Sillitoe on behalf of the Wellcome Sanger Institute COVID-19 Surveillance Team
EPI_ISL_762795	Lighthouse Lab in Cambridge	Wellcome Sanger Institute for the COVID-19 Genomics UK (COG-UK) Consortium	Rob Howes, The Lighthouse Lab in Cambridge and Alex Alderton, Roberto Amato, Sonia Goncalves, Ewan Harrison, David K. Jackson, Ian Johnston, Dominic Kwiatkowski, Cordelia Langford, John Sillitoe on behalf of the Wellcome Sanger Institute COVID-19 Surveillance Team
EPI_ISL_762796, EPI_ISL_762798, EPI_ISL_762799	Lighthouse Lab in Alderley Park	Wellcome Sanger Institute for the COVID-19 Genomics UK (COG-UK) Consortium	Jacquelyn Wynn, Mairead Hyland, The Lighthouse Lab in Alderley Park and Alex Alderton, Roberto Amato, Sonia Goncalves, Ewan Harrison, David K. Jackson, Ian Johnston, Dominic Kwiatkowski, Cordelia Langford, John Sillitoe on behalf of the Wellcome Sanger Institute COVID-19 Surveillance Team
EPI_ISL_762801	Lighthouse Lab in Glasgow	Wellcome Sanger Institute for the COVID-19 Genomics UK (COG-UK) Consortium	Harper VanSteenhouse, Yumi Kasai, David Gray, Carol Clugston, Anna Dominiczak and Alex Alderton, Roberto Amato, Sonia Goncalves, Ewan Harrison, David K. Jackson, Ian Johnston, Dominic Kwiatkowski, Cordelia Langford, John Sillitoe on behalf of the Wellcome Sanger Institute COVID-19 Surveillance Team
EPI_ISL_762803, EPI_ISL_762804, EPI_ISL_762805, EPI_ISL_762806, EPI_ISL_762807, EPI_ISL_762808	Lighthouse Lab in Cambridge	Wellcome Sanger Institute for the COVID-19 Genomics UK (COG-UK) Consortium	Rob Howes, The Lighthouse Lab in Cambridge and Alex Alderton, Roberto Amato, Sonia Goncalves, Ewan Harrison, David K. Jackson, Ian Johnston, Dominic Kwiatkowski, Cordelia Langford, John Sillitoe on behalf of the Wellcome Sanger Institute COVID-19 Surveillance Team
EPI_ISL_762809	Lighthouse Lab in Glasgow	Wellcome Sanger Institute for the COVID-19 Genomics UK (COG-UK) Consortium	Harper VanSteenhouse, Yumi Kasai, David Gray, Carol Clugston, Anna Dominiczak and Alex Alderton, Roberto Amato, Sonia Goncalves, Ewan Harrison, David K. Jackson, Ian Johnston, Dominic Kwiatkowski, Cordelia Langford, John Sillitoe on behalf of the Wellcome Sanger Institute COVID-19 Surveillance Team
EPI_ISL_762811	Lighthouse Lab in Cambridge	Wellcome Sanger Institute for the COVID-19 Genomics UK (COG-UK) Consortium	Rob Howes, The Lighthouse Lab in Cambridge and Alex Alderton, Roberto Amato, Sonia Goncalves, Ewan Harrison, David K. Jackson, Ian Johnston, Dominic Kwiatkowski, Cordelia Langford, John Sillitoe on behalf of the Wellcome Sanger Institute COVID-19 Surveillance Team
EPI_ISL_762813	Lighthouse Lab in Glasgow	Wellcome Sanger Institute for the COVID-19 Genomics UK (COG-UK) Consortium	Harper VanSteenhouse, Yumi Kasai, David Gray, Carol Clugston, Anna Dominiczak and Alex Alderton, Roberto Amato, Sonia Goncalves, Ewan Harrison, David K. Jackson, Ian Johnston, Dominic Kwiatkowski, Cordelia Langford, John Sillitoe on behalf of the Wellcome Sanger Institute COVID-19 Surveillance Team
EPI_ISL_762815	Lighthouse Lab in Alderley Park	Wellcome Sanger Institute for the COVID-19 Genomics UK (COG-UK) Consortium	Jacquelyn Wynn, Mairead Hyland, The Lighthouse Lab in Alderley Park and Alex Alderton, Roberto Amato, Sonia Goncalves, Ewan Harrison, David K. Jackson, Ian Johnston, Dominic Kwiatkowski, Cordelia Langford, John Sillitoe on behalf of the Wellcome Sanger Institute COVID-19 Surveillance Team
EPI_ISL_762818	Lighthouse Lab in Cambridge	Wellcome Sanger Institute for the COVID-19 Genomics UK (COG-UK) Consortium	Rob Howes, The Lighthouse Lab in Cambridge and Alex Alderton, Roberto Amato, Sonia Goncalves, Ewan Harrison, David K. Jackson, Ian Johnston, Dominic Kwiatkowski, Cordelia Langford, John Sillitoe on behalf of the Wellcome Sanger Institute COVID-19 Surveillance Team
EPI_ISL_762819	Lighthouse Lab in Alderley Park	Wellcome Sanger Institute for the COVID-19 Genomics UK (COG-UK) Consortium	Jacquelyn Wynn, Mairead Hyland, The Lighthouse Lab in Alderley Park and Alex Alderton, Roberto Amato, Sonia Goncalves, Ewan Harrison, David K. Jackson, Ian Johnston, Dominic Kwiatkowski, Cordelia Langford, John Sillitoe on behalf of the Wellcome Sanger Institute COVID-19 Surveillance Team
EPI_ISL_762820	Lighthouse Lab in Cambridge	Wellcome Sanger Institute for the COVID-19 Genomics UK (COG-UK) Consortium	Rob Howes, The Lighthouse Lab in Cambridge and Alex Alderton, Roberto Amato, Sonia Goncalves, Ewan Harrison, David K. Jackson, Ian Johnston, Dominic Kwiatkowski, Cordelia Langford, John Sillitoe on behalf of the Wellcome Sanger Institute COVID-19 Surveillance Team
EPI_ISL_762821	Lighthouse Lab in Alderley Park	Wellcome Sanger Institute for the COVID-19 Genomics UK (COG-UK) Consortium	Jacquelyn Wynn, Mairead Hyland, The Lighthouse Lab in Alderley Park and Alex Alderton, Roberto Amato, Sonia Goncalves, Ewan Harrison, David K. Jackson, Ian Johnston, Dominic Kwiatkowski, Cordelia Langford, John Sillitoe on behalf of the Wellcome Sanger Institute COVID-19 Surveillance Team
EPI_ISL_762822	Lighthouse Lab in Cambridge	Wellcome Sanger Institute for the COVID-19 Genomics UK (COG-UK) Consortium	Rob Howes, The Lighthouse Lab in Cambridge and Alex Alderton, Roberto Amato, Sonia Goncalves, Ewan Harrison, David K. Jackson, Ian Johnston, Dominic Kwiatkowski, Cordelia Langford, John Sillitoe on behalf of the Wellcome Sanger Institute COVID-19 Surveillance Team
EPI_ISL_762823	Lighthouse Lab in Alderley Park	Wellcome Sanger Institute for the COVID-19 Genomics UK (COG-UK) Consortium	Jacquelyn Wynn, Mairead Hyland, The Lighthouse Lab in Alderley Park and Alex Alderton, Roberto Amato, Sonia Goncalves, Ewan Harrison, David K. Jackson, Ian Johnston, Dominic Kwiatkowski, Cordelia Langford, John Sillitoe on behalf of the Wellcome Sanger Institute COVID-19 Surveillance Team
EPI_ISL_762824	Lighthouse Lab in Glasgow	Wellcome Sanger Institute for the COVID-19 Genomics UK (COG-UK) Consortium	Harper VanSteenhouse, Yumi Kasai, David Gray, Carol Clugston, Anna Dominiczak and Alex Alderton, Roberto Amato, Sonia Goncalves, Ewan Harrison, David K. Jackson, Ian Johnston, Dominic Kwiatkowski, Cordelia Langford, John Sillitoe on behalf of the Wellcome Sanger Institute COVID-19 Surveillance Team
EPI_ISL_762825	Lighthouse Lab in Cambridge	Wellcome Sanger Institute for the COVID-19 Genomics UK (COG-UK) Consortium	Rob Howes, The Lighthouse Lab in Cambridge and Alex Alderton, Roberto Amato, Sonia Goncalves, Ewan Harrison, David K. Jackson, Ian Johnston, Dominic Kwiatkowski, Cordelia Langford, John Sillitoe on behalf of the Wellcome Sanger Institute COVID-19 Surveillance Team
EPI_ISL_762826	Lighthouse Lab in Glasgow	Wellcome Sanger Institute for the COVID-19 Genomics UK (COG-UK) Consortium	Harper VanSteenhouse, Yumi Kasai, David Gray, Carol Clugston, Anna Dominiczak and Alex Alderton, Roberto Amato, Sonia Goncalves, Ewan Harrison, David K. Jackson, Ian Johnston, Dominic Kwiatkowski, Cordelia Langford, John Sillitoe on behalf of the Wellcome Sanger Institute COVID-19 Surveillance Team
EPI_ISL_762827	Lighthouse Lab in Cambridge	Wellcome Sanger Institute for the COVID-19 Genomics UK (COG-UK) Consortium	Rob Howes, The Lighthouse Lab in Cambridge and Alex Alderton, Roberto Amato, Sonia Goncalves, Ewan Harrison, David K. Jackson, Ian Johnston, Dominic Kwiatkowski, Cordelia Langford, John Sillitoe on behalf of the Wellcome Sanger Institute COVID-19 Surveillance Team

EPI_ISL_762830	Lighthouse Lab in Alderley Park	Wellcome Sanger Institute for the COVID-19 Genomics UK (COG-UK) Consortium	Jacquelyn Wynn, Mairead Hyland, The Lighthouse Lab in Alderley Park and Alex Alderton, Roberto Amato, Sonia Goncalves, Ewan Harrison, David K. Jackson, Ian Johnston, Dominic Kwiatkowski, Cordelia Langford, John Sillitoe on behalf of the Wellcome Sanger Institute COVID-19 Surveillance Team
EPI_ISL_762831	Lighthouse Lab in Cambridge	Wellcome Sanger Institute for the COVID-19 Genomics UK (COG-UK) Consortium	Rob Howes, The Lighthouse Lab in Cambridge and Alex Alderton, Roberto Amato, Sonia Goncalves, Ewan Harrison, David K. Jackson, Ian Johnston, Dominic Kwiatkowski, Cordelia Langford, John Sillitoe on behalf of the Wellcome Sanger Institute COVID-19 Surveillance Team
EPI_ISL_762832	Lighthouse Lab in Alderley Park	Wellcome Sanger Institute for the COVID-19 Genomics UK (COG-UK) Consortium	Jacquelyn Wynn, Mairead Hyland, The Lighthouse Lab in Alderley Park and Alex Alderton, Roberto Amato, Sonia Goncalves, Ewan Harrison, David K. Jackson, Ian Johnston, Dominic Kwiatkowski, Cordelia Langford, John Sillitoe on behalf of the Wellcome Sanger Institute COVID-19 Surveillance Team
EPI_ISL_762833, EPI_ISL_762834, EPI_ISL_762836	Lighthouse Lab in Cambridge	Wellcome Sanger Institute for the COVID-19 Genomics UK (COG-UK) Consortium	Rob Howes, The Lighthouse Lab in Cambridge and Alex Alderton, Roberto Amato, Sonia Goncalves, Ewan Harrison, David K. Jackson, Ian Johnston, Dominic Kwiatkowski, Cordelia Langford, John Sillitoe on behalf of the Wellcome Sanger Institute COVID-19 Surveillance Team
EPI_ISL_762837	Lighthouse Lab in Glasgow	Wellcome Sanger Institute for the COVID-19 Genomics UK (COG-UK) Consortium	Harper VanSteenhouse, Yumi Kasai, David Gray, Carol Clugston, Anna Dominiczak and Alex Alderton, Roberto Amato, Sonia Goncalves, Ewan Harrison, David K. Jackson, Ian Johnston, Dominic Kwiatkowski, Cordelia Langford, John Sillitoe on behalf of the Wellcome Sanger Institute COVID-19 Surveillance Team
EPI_ISL_762838	Lighthouse Lab in Cambridge	Wellcome Sanger Institute for the COVID-19 Genomics UK (COG-UK) Consortium	Rob Howes, The Lighthouse Lab in Cambridge and Alex Alderton, Roberto Amato, Sonia Goncalves, Ewan Harrison, David K. Jackson, Ian Johnston, Dominic Kwiatkowski, Cordelia Langford, John Sillitoe on behalf of the Wellcome Sanger Institute COVID-19 Surveillance Team
EPI_ISL_762839, EPI_ISL_762841, EPI_ISL_762843, EPI_ISL_762844, EPI_ISL_762845	Lighthouse Lab in Alderley Park	Wellcome Sanger Institute for the COVID-19 Genomics UK (COG-UK) Consortium	Jacquelyn Wynn, Mairead Hyland, The Lighthouse Lab in Alderley Park and Alex Alderton, Roberto Amato, Sonia Goncalves, Ewan Harrison, David K. Jackson, Ian Johnston, Dominic Kwiatkowski, Cordelia Langford, John Sillitoe on behalf of the Wellcome Sanger Institute COVID-19 Surveillance Team
EPI_ISL_762846	Lighthouse Lab in Glasgow	Wellcome Sanger Institute for the COVID-19 Genomics UK (COG-UK) Consortium	Harper VanSteenhouse, Yumi Kasai, David Gray, Carol Clugston, Anna Dominiczak and Alex Alderton, Roberto Amato, Sonia Goncalves, Ewan Harrison, David K. Jackson, Ian Johnston, Dominic Kwiatkowski, Cordelia Langford, John Sillitoe on behalf of the Wellcome Sanger Institute COVID-19 Surveillance Team
EPI_ISL_762847	Lighthouse Lab in Alderley Park	Wellcome Sanger Institute for the COVID-19 Genomics UK (COG-UK) Consortium	Jacquelyn Wynn, Mairead Hyland, The Lighthouse Lab in Alderley Park and Alex Alderton, Roberto Amato, Sonia Goncalves, Ewan Harrison, David K. Jackson, Ian Johnston, Dominic Kwiatkowski, Cordelia Langford, John Sillitoe on behalf of the Wellcome Sanger Institute COVID-19 Surveillance Team
EPI_ISL_762848	Lighthouse Lab in Cambridge	Wellcome Sanger Institute for the COVID-19 Genomics UK (COG-UK) Consortium	Rob Howes, The Lighthouse Lab in Cambridge and Alex Alderton, Roberto Amato, Sonia Goncalves, Ewan Harrison, David K. Jackson, Ian Johnston, Dominic Kwiatkowski, Cordelia Langford, John Sillitoe on behalf of the Wellcome Sanger Institute COVID-19 Surveillance Team
EPI_ISL_762849	Lighthouse Lab in Alderley Park	Wellcome Sanger Institute for the COVID-19 Genomics UK (COG-UK) Consortium	Jacquelyn Wynn, Mairead Hyland, The Lighthouse Lab in Alderley Park and Alex Alderton, Roberto Amato, Sonia Goncalves, Ewan Harrison, David K. Jackson, Ian Johnston, Dominic Kwiatkowski, Cordelia Langford, John Sillitoe on behalf of the Wellcome Sanger Institute COVID-19 Surveillance Team
EPI_ISL_762850	Lighthouse Lab in Glasgow	Wellcome Sanger Institute for the COVID-19 Genomics UK (COG-UK) Consortium	Harper VanSteenhouse, Yumi Kasai, David Gray, Carol Clugston, Anna Dominiczak and Alex Alderton, Roberto Amato, Sonia Goncalves, Ewan Harrison, David K. Jackson, Ian Johnston, Dominic Kwiatkowski, Cordelia Langford, John Sillitoe on behalf of the Wellcome Sanger Institute COVID-19 Surveillance Team
EPI_ISL_762852	Lighthouse Lab in Cambridge	Wellcome Sanger Institute for the COVID-19 Genomics UK (COG-UK) Consortium	Rob Howes, The Lighthouse Lab in Cambridge and Alex Alderton, Roberto Amato, Sonia Goncalves, Ewan Harrison, David K. Jackson, Ian Johnston, Dominic Kwiatkowski, Cordelia Langford, John Sillitoe on behalf of the Wellcome Sanger Institute COVID-19 Surveillance Team
EPI_ISL_762853, EPI_ISL_762854	Lighthouse Lab in Glasgow	Wellcome Sanger Institute for the COVID-19 Genomics UK (COG-UK) Consortium	Harper VanSteenhouse, Yumi Kasai, David Gray, Carol Clugston, Anna Dominiczak and Alex Alderton, Roberto Amato, Sonia Goncalves, Ewan Harrison, David K. Jackson, Ian Johnston, Dominic Kwiatkowski, Cordelia Langford, John Sillitoe on behalf of the Wellcome Sanger Institute COVID-19 Surveillance Team
EPI_ISL_762855	Lighthouse Lab in Cambridge	Wellcome Sanger Institute for the COVID-19 Genomics UK (COG-UK) Consortium	Rob Howes, The Lighthouse Lab in Cambridge and Alex Alderton, Roberto Amato, Sonia Goncalves, Ewan Harrison, David K. Jackson, Ian Johnston, Dominic Kwiatkowski, Cordelia Langford, John Sillitoe on behalf of the Wellcome Sanger Institute COVID-19 Surveillance Team
EPI_ISL_762856	Lighthouse Lab in Glasgow	Wellcome Sanger Institute for the COVID-19 Genomics UK (COG-UK) Consortium	Harper VanSteenhouse, Yumi Kasai, David Gray, Carol Clugston, Anna Dominiczak and Alex Alderton, Roberto Amato, Sonia Goncalves, Ewan Harrison, David K. Jackson, Ian Johnston, Dominic Kwiatkowski, Cordelia Langford, John Sillitoe on behalf of the Wellcome Sanger Institute COVID-19 Surveillance Team
EPI_ISL_762858	Lighthouse Lab in Cambridge	Wellcome Sanger Institute for the COVID-19 Genomics UK (COG-UK) Consortium	Rob Howes, The Lighthouse Lab in Cambridge and Alex Alderton, Roberto Amato, Sonia Goncalves, Ewan Harrison, David K. Jackson, Ian Johnston, Dominic Kwiatkowski, Cordelia Langford, John Sillitoe on behalf of the Wellcome Sanger Institute COVID-19 Surveillance Team
EPI_ISL_762865	Lighthouse Lab in Glasgow	Wellcome Sanger Institute for the COVID-19 Genomics UK (COG-UK) Consortium	Harper VanSteenhouse, Yumi Kasai, David Gray, Carol Clugston, Anna Dominiczak and Alex Alderton, Roberto Amato, Sonia Goncalves, Ewan Harrison, David K. Jackson, Ian Johnston, Dominic Kwiatkowski, Cordelia Langford, John Sillitoe on behalf of the Wellcome Sanger Institute COVID-19 Surveillance Team
EPI_ISL_762867	Lighthouse Lab in Milton Keynes	Wellcome Sanger Institute for the COVID-19 Genomics UK (COG-UK) Consortium	The Lighthouse Lab in Milton Keynes and Alex Alderton, Roberto Amato, Sonia Goncalves, Ewan Harrison, David K. Jackson, Ian Johnston, Dominic Kwiatkowski, Cordelia Langford, John Sillitoe on behalf of the Wellcome Sanger Institute COVID-19 Surveillance Team
EPI_ISL_762869	Lighthouse Lab in Alderley Park	Wellcome Sanger Institute for the COVID-19 Genomics UK (COG-UK) Consortium	Jacquelyn Wynn, Mairead Hyland, The Lighthouse Lab in Alderley Park and Alex Alderton, Roberto Amato, Sonia Goncalves, Ewan Harrison, David K. Jackson, Ian Johnston, Dominic Kwiatkowski, Cordelia Langford, John Sillitoe on behalf of the Wellcome Sanger Institute COVID-19 Surveillance Team
EPI_ISL_762871, EPI_ISL_762872, EPI_ISL_762876, EPI_ISL_762877	Lighthouse Lab in Glasgow	Wellcome Sanger Institute for the COVID-19 Genomics UK (COG-UK) Consortium	Harper VanSteenhouse, Yumi Kasai, David Gray, Carol Clugston, Anna Dominiczak and Alex Alderton, Roberto Amato, Sonia Goncalves, Ewan Harrison, David K. Jackson, Ian Johnston, Dominic Kwiatkowski, Cordelia Langford, John Sillitoe on behalf of the Wellcome Sanger Institute COVID-19 Surveillance Team
EPI_ISL_762878, EPI_ISL_762879	Lighthouse Lab in Alderley Park	Wellcome Sanger Institute for the COVID-19 Genomics UK (COG-UK) Consortium	Jacquelyn Wynn, Mairead Hyland, The Lighthouse Lab in Alderley Park and Alex Alderton, Roberto Amato, Sonia Goncalves, Ewan Harrison, David K. Jackson, Ian Johnston, Dominic Kwiatkowski, Cordelia Langford, John Sillitoe on behalf of the Wellcome Sanger Institute COVID-19 Surveillance Team
EPI_ISL_762880	Lighthouse Lab in Milton Keynes	Wellcome Sanger Institute for the COVID-19 Genomics UK (COG-UK) Consortium	The Lighthouse Lab in Milton Keynes and Alex Alderton, Roberto Amato, Sonia Goncalves, Ewan Harrison, David K. Jackson, Ian Johnston, Dominic Kwiatkowski, Cordelia Langford, John Sillitoe on behalf of the Wellcome Sanger Institute COVID-19 Surveillance Team
EPI_ISL_762881	Lighthouse Lab in Glasgow	Wellcome Sanger Institute for the COVID-19 Genomics UK (COG-UK) Consortium	Harper VanSteenhouse, Yumi Kasai, David Gray, Carol Clugston, Anna Dominiczak and Alex Alderton, Roberto Amato, Sonia Goncalves, Ewan Harrison, David K. Jackson, Ian Johnston, Dominic Kwiatkowski, Cordelia Langford, John Sillitoe on behalf of the Wellcome Sanger Institute COVID-19 Surveillance Team
EPI_ISL_762883	Lighthouse Lab in Alderley Park	Wellcome Sanger Institute for the COVID-19 Genomics UK (COG-UK) Consortium	Jacquelyn Wynn, Mairead Hyland, The Lighthouse Lab in Alderley Park and Alex Alderton, Roberto Amato, Sonia Goncalves, Ewan Harrison, David K. Jackson, Ian Johnston, Dominic Kwiatkowski, Cordelia Langford, John Sillitoe on behalf of the Wellcome Sanger Institute COVID-19 Surveillance Team
EPI_ISL_762885	Lighthouse Lab in Glasgow	Wellcome Sanger Institute for the COVID-19 Genomics UK (COG-UK) Consortium	Harper VanSteenhouse, Yumi Kasai, David Gray, Carol Clugston, Anna Dominiczak and Alex Alderton, Roberto Amato, Sonia Goncalves, Ewan Harrison, David K. Jackson, Ian Johnston, Dominic Kwiatkowski, Cordelia Langford, John Sillitoe on behalf of the Wellcome Sanger Institute COVID-19 Surveillance Team
EPI_ISL_762890, EPI_ISL_762894	Lighthouse Lab in Alderley Park	Wellcome Sanger Institute for the COVID-19 Genomics UK (COG-UK) Consortium	Jacquelyn Wynn, Mairead Hyland, The Lighthouse Lab in Alderley Park and Alex Alderton, Roberto Amato, Sonia Goncalves, Ewan Harrison, David K. Jackson, Ian Johnston, Dominic Kwiatkowski, Cordelia Langford, John Sillitoe on behalf of the Wellcome Sanger Institute COVID-19 Surveillance Team
EPI_ISL_762896	Lighthouse Lab in Milton Keynes	Wellcome Sanger Institute for the COVID-19 Genomics UK (COG-UK) Consortium	The Lighthouse Lab in Milton Keynes and Alex Alderton, Roberto Amato, Sonia Goncalves, Ewan Harrison, David K. Jackson, Ian Johnston, Dominic Kwiatkowski, Cordelia Langford, John Sillitoe on behalf of the Wellcome Sanger Institute COVID-19 Surveillance Team
EPI_ISL_762897	Lighthouse Lab in Glasgow	Wellcome Sanger Institute for the COVID-19 Genomics UK (COG-UK) Consortium	Harper VanSteenhouse, Yumi Kasai, David Gray, Carol Clugston, Anna Dominiczak and Alex Alderton, Roberto Amato, Sonia Goncalves, Ewan Harrison, David K. Jackson, Ian Johnston, Dominic Kwiatkowski, Cordelia Langford, John Sillitoe on behalf of the Wellcome Sanger Institute COVID-19 Surveillance Team
EPI_ISL_762901	Lighthouse Lab in Alderley Park	Wellcome Sanger Institute for the COVID-19 Genomics UK (COG-UK) Consortium	Jacquelyn Wynn, Mairead Hyland, The Lighthouse Lab in Alderley Park and Alex Alderton, Roberto Amato, Sonia Goncalves, Ewan Harrison, David K. Jackson, Ian Johnston, Dominic Kwiatkowski, Cordelia Langford, John Sillitoe on behalf of the Wellcome Sanger Institute COVID-19 Surveillance Team
EPI_ISL_762903	Lighthouse Lab in Milton Keynes	Wellcome Sanger Institute for the COVID-19 Genomics UK	The Lighthouse Lab in Milton Keynes and Alex Alderton, Roberto Amato, Sonia Goncalves, Ewan Harrison, David K. Jackson, Ian Johnston, Dominic

		(COG-UK) Consortium	Kwiatkowski, Cordelia Langford, John Sillitoe on behalf of the Wellcome Sanger Institute COVID-19 Surveillance Team
EPI_ISL_762905	Lighthouse Lab in Glasgow	Wellcome Sanger Institute for the COVID-19 Genomics UK (COG-UK) Consortium	Harper VanSteenhouse, Yumi Kasai, David Gray, Carol Clugston, Anna Dominiczak and Alex Alderton, Roberto Amato, Sonia Goncalves, Ewan Harrison, David K. Jackson, Ian Johnston, Dominic Kwiatkowski, Cordelia Langford, John Sillitoe on behalf of the Wellcome Sanger Institute COVID-19 Surveillance Team
EPI_ISL_762908	Lighthouse Lab in Milton Keynes	Wellcome Sanger Institute for the COVID-19 Genomics UK (COG-UK) Consortium	The Lighthouse Lab in Milton Keynes and Alex Alderton, Roberto Amato, Sonia Goncalves, Ewan Harrison, David K. Jackson, Ian Johnston, Dominic Kwiatkowski, Cordelia Langford, John Sillitoe on behalf of the Wellcome Sanger Institute COVID-19 Surveillance Team
EPI_ISL_762909	Lighthouse Lab in Glasgow	Wellcome Sanger Institute for the COVID-19 Genomics UK (COG-UK) Consortium	Harper VanSteenhouse, Yumi Kasai, David Gray, Carol Clugston, Anna Dominiczak and Alex Alderton, Roberto Amato, Sonia Goncalves, Ewan Harrison, David K. Jackson, Ian Johnston, Dominic Kwiatkowski, Cordelia Langford, John Sillitoe on behalf of the Wellcome Sanger Institute COVID-19 Surveillance Team
EPI_ISL_762910, EPI_ISL_762913	Lighthouse Lab in Alderley Park	Wellcome Sanger Institute for the COVID-19 Genomics UK (COG-UK) Consortium	Jacquelyn Wynn, Mairead Hyland, The Lighthouse Lab in Alderley Park and Alex Alderton, Roberto Amato, Sonia Goncalves, Ewan Harrison, David K. Jackson, Ian Johnston, Dominic Kwiatkowski, Cordelia Langford, John Sillitoe on behalf of the Wellcome Sanger Institute COVID-19 Surveillance Team
EPI_ISL_762914	Lighthouse Lab in Milton Keynes	Wellcome Sanger Institute for the COVID-19 Genomics UK (COG-UK) Consortium	The Lighthouse Lab in Milton Keynes and Alex Alderton, Roberto Amato, Sonia Goncalves, Ewan Harrison, David K. Jackson, Ian Johnston, Dominic Kwiatkowski, Cordelia Langford, John Sillitoe on behalf of the Wellcome Sanger Institute COVID-19 Surveillance Team
EPI_ISL_762918	Lighthouse Lab in Glasgow	Wellcome Sanger Institute for the COVID-19 Genomics UK (COG-UK) Consortium	Harper VanSteenhouse, Yumi Kasai, David Gray, Carol Clugston, Anna Dominiczak and Alex Alderton, Roberto Amato, Sonia Goncalves, Ewan Harrison, David K. Jackson, Ian Johnston, Dominic Kwiatkowski, Cordelia Langford, John Sillitoe on behalf of the Wellcome Sanger Institute COVID-19 Surveillance Team
EPI_ISL_762919, EPI_ISL_762921	Lighthouse Lab in Alderley Park	Wellcome Sanger Institute for the COVID-19 Genomics UK (COG-UK) Consortium	Jacquelyn Wynn, Mairead Hyland, The Lighthouse Lab in Alderley Park and Alex Alderton, Roberto Amato, Sonia Goncalves, Ewan Harrison, David K. Jackson, Ian Johnston, Dominic Kwiatkowski, Cordelia Langford, John Sillitoe on behalf of the Wellcome Sanger Institute COVID-19 Surveillance Team
EPI_ISL_762924, EPI_ISL_762925, EPI_ISL_762927, EPI_ISL_762928, EPI_ISL_762929, EPI_ISL_762930, EPI_ISL_762934	Lighthouse Lab in Glasgow	Wellcome Sanger Institute for the COVID-19 Genomics UK (COG-UK) Consortium	Harper VanSteenhouse, Yumi Kasai, David Gray, Carol Clugston, Anna Dominiczak and Alex Alderton, Roberto Amato, Sonia Goncalves, Ewan Harrison, David K. Jackson, Ian Johnston, Dominic Kwiatkowski, Cordelia Langford, John Sillitoe on behalf of the Wellcome Sanger Institute COVID-19 Surveillance Team
EPI_ISL_762935	Lighthouse Lab in Alderley Park	Wellcome Sanger Institute for the COVID-19 Genomics UK (COG-UK) Consortium	Jacquelyn Wynn, Mairead Hyland, The Lighthouse Lab in Alderley Park and Alex Alderton, Roberto Amato, Sonia Goncalves, Ewan Harrison, David K. Jackson, Ian Johnston, Dominic Kwiatkowski, Cordelia Langford, John Sillitoe on behalf of the Wellcome Sanger Institute COVID-19 Surveillance Team
EPI_ISL_762936, EPI_ISL_762941	Lighthouse Lab in Glasgow	Wellcome Sanger Institute for the COVID-19 Genomics UK (COG-UK) Consortium	Harper VanSteenhouse, Yumi Kasai, David Gray, Carol Clugston, Anna Dominiczak and Alex Alderton, Roberto Amato, Sonia Goncalves, Ewan Harrison, David K. Jackson, Ian Johnston, Dominic Kwiatkowski, Cordelia Langford, John Sillitoe on behalf of the Wellcome Sanger Institute COVID-19 Surveillance Team
EPI_ISL_762945	Lighthouse Lab in Milton Keynes	Wellcome Sanger Institute for the COVID-19 Genomics UK (COG-UK) Consortium	The Lighthouse Lab in Milton Keynes and Alex Alderton, Roberto Amato, Sonia Goncalves, Ewan Harrison, David K. Jackson, Ian Johnston, Dominic Kwiatkowski, Cordelia Langford, John Sillitoe on behalf of the Wellcome Sanger Institute COVID-19 Surveillance Team
EPI_ISL_762952	Lighthouse Lab in Alderley Park	Wellcome Sanger Institute for the COVID-19 Genomics UK (COG-UK) Consortium	Jacquelyn Wynn, Mairead Hyland, The Lighthouse Lab in Alderley Park and Alex Alderton, Roberto Amato, Sonia Goncalves, Ewan Harrison, David K. Jackson, Ian Johnston, Dominic Kwiatkowski, Cordelia Langford, John Sillitoe on behalf of the Wellcome Sanger Institute COVID-19 Surveillance Team
EPI_ISL_762954	Lighthouse Lab in Glasgow	Wellcome Sanger Institute for the COVID-19 Genomics UK (COG-UK) Consortium	Harper VanSteenhouse, Yumi Kasai, David Gray, Carol Clugston, Anna Dominiczak and Alex Alderton, Roberto Amato, Sonia Goncalves, Ewan Harrison, David K. Jackson, Ian Johnston, Dominic Kwiatkowski, Cordelia Langford, John Sillitoe on behalf of the Wellcome Sanger Institute COVID-19 Surveillance Team
EPI_ISL_762955	Lighthouse Lab in Alderley Park	Wellcome Sanger Institute for the COVID-19 Genomics UK (COG-UK) Consortium	Jacquelyn Wynn, Mairead Hyland, The Lighthouse Lab in Alderley Park and Alex Alderton, Roberto Amato, Sonia Goncalves, Ewan Harrison, David K. Jackson, Ian Johnston, Dominic Kwiatkowski, Cordelia Langford, John Sillitoe on behalf of the Wellcome Sanger Institute COVID-19 Surveillance Team
EPI_ISL_762956	Lighthouse Lab in Glasgow	Wellcome Sanger Institute for the COVID-19 Genomics UK (COG-UK) Consortium	Harper VanSteenhouse, Yumi Kasai, David Gray, Carol Clugston, Anna Dominiczak and Alex Alderton, Roberto Amato, Sonia Goncalves, Ewan Harrison, David K. Jackson, Ian Johnston, Dominic Kwiatkowski, Cordelia Langford, John Sillitoe on behalf of the Wellcome Sanger Institute COVID-19 Surveillance Team
EPI_ISL_762957	Lighthouse Lab in Alderley Park	Wellcome Sanger Institute for the COVID-19 Genomics UK (COG-UK) Consortium	Jacquelyn Wynn, Mairead Hyland, The Lighthouse Lab in Alderley Park and Alex Alderton, Roberto Amato, Sonia Goncalves, Ewan Harrison, David K. Jackson, Ian Johnston, Dominic Kwiatkowski, Cordelia Langford, John Sillitoe on behalf of the Wellcome Sanger Institute COVID-19 Surveillance Team
EPI_ISL_762959, EPI_ISL_762960	Lighthouse Lab in Glasgow	Wellcome Sanger Institute for the COVID-19 Genomics UK (COG-UK) Consortium	Harper VanSteenhouse, Yumi Kasai, David Gray, Carol Clugston, Anna Dominiczak and Alex Alderton, Roberto Amato, Sonia Goncalves, Ewan Harrison, David K. Jackson, Ian Johnston, Dominic Kwiatkowski, Cordelia Langford, John Sillitoe on behalf of the Wellcome Sanger Institute COVID-19 Surveillance Team
EPI_ISL_762964	Lighthouse Lab in Alderley Park	Wellcome Sanger Institute for the COVID-19 Genomics UK (COG-UK) Consortium	Jacquelyn Wynn, Mairead Hyland, The Lighthouse Lab in Alderley Park and Alex Alderton, Roberto Amato, Sonia Goncalves, Ewan Harrison, David K. Jackson, Ian Johnston, Dominic Kwiatkowski, Cordelia Langford, John Sillitoe on behalf of the Wellcome Sanger Institute COVID-19 Surveillance Team
EPI_ISL_762966	Lighthouse Lab in Glasgow	Wellcome Sanger Institute for the COVID-19 Genomics UK (COG-UK) Consortium	Harper VanSteenhouse, Yumi Kasai, David Gray, Carol Clugston, Anna Dominiczak and Alex Alderton, Roberto Amato, Sonia Goncalves, Ewan Harrison, David K. Jackson, Ian Johnston, Dominic Kwiatkowski, Cordelia Langford, John Sillitoe on behalf of the Wellcome Sanger Institute COVID-19 Surveillance Team
EPI_ISL_762967	Lighthouse Lab in Milton Keynes	Wellcome Sanger Institute for the COVID-19 Genomics UK (COG-UK) Consortium	The Lighthouse Lab in Milton Keynes and Alex Alderton, Roberto Amato, Sonia Goncalves, Ewan Harrison, David K. Jackson, Ian Johnston, Dominic Kwiatkowski, Cordelia Langford, John Sillitoe on behalf of the Wellcome Sanger Institute COVID-19 Surveillance Team
EPI_ISL_762973, EPI_ISL_762974, EPI_ISL_762975	Lighthouse Lab in Alderley Park	Wellcome Sanger Institute for the COVID-19 Genomics UK (COG-UK) Consortium	Jacquelyn Wynn, Mairead Hyland, The Lighthouse Lab in Alderley Park and Alex Alderton, Roberto Amato, Sonia Goncalves, Ewan Harrison, David K. Jackson, Ian Johnston, Dominic Kwiatkowski, Cordelia Langford, John Sillitoe on behalf of the Wellcome Sanger Institute COVID-19 Surveillance Team
EPI_ISL_762976, EPI_ISL_762978	Lighthouse Lab in Milton Keynes	Wellcome Sanger Institute for the COVID-19 Genomics UK (COG-UK) Consortium	The Lighthouse Lab in Milton Keynes and Alex Alderton, Roberto Amato, Sonia Goncalves, Ewan Harrison, David K. Jackson, Ian Johnston, Dominic Kwiatkowski, Cordelia Langford, John Sillitoe on behalf of the Wellcome Sanger Institute COVID-19 Surveillance Team
EPI_ISL_762980	Lighthouse Lab in Glasgow	Wellcome Sanger Institute for the COVID-19 Genomics UK (COG-UK) Consortium	Harper VanSteenhouse, Yumi Kasai, David Gray, Carol Clugston, Anna Dominiczak and Alex Alderton, Roberto Amato, Sonia Goncalves, Ewan Harrison, David K. Jackson, Ian Johnston, Dominic Kwiatkowski, Cordelia Langford, John Sillitoe on behalf of the Wellcome Sanger Institute COVID-19 Surveillance Team
EPI_ISL_762981	Lighthouse Lab in Milton Keynes	Wellcome Sanger Institute for the COVID-19 Genomics UK (COG-UK) Consortium	The Lighthouse Lab in Milton Keynes and Alex Alderton, Roberto Amato, Sonia Goncalves, Ewan Harrison, David K. Jackson, Ian Johnston, Dominic Kwiatkowski, Cordelia Langford, John Sillitoe on behalf of the Wellcome Sanger Institute COVID-19 Surveillance Team
EPI_ISL_762984, EPI_ISL_762986, EPI_ISL_762987	Lighthouse Lab in Glasgow	Wellcome Sanger Institute for the COVID-19 Genomics UK (COG-UK) Consortium	Harper VanSteenhouse, Yumi Kasai, David Gray, Carol Clugston, Anna Dominiczak and Alex Alderton, Roberto Amato, Sonia Goncalves, Ewan Harrison, David K. Jackson, Ian Johnston, Dominic Kwiatkowski, Cordelia Langford, John Sillitoe on behalf of the Wellcome Sanger Institute COVID-19 Surveillance Team
EPI_ISL_763062	Department for Virology, Molecular Biology and Genome Research, R. G. Lugar Center for Public Health Research, National Center for Disease Control and Public Health (NCDC) of Georgia.	Department for Virology, Molecular Biology and Genome Research, R. G. Lugar Center for Public Health Research, National Center for Disease Control and Public Health (NCDC) of Georgia.	Giorgi Tomashvili, Gvantsa Brachveli, Meri Pantsulaia, Nino Berishvili, Tata Imnadze, Ana Papkiauri, Gvantsa Chanturia, Ann Machablishvili, Nato Kotaria, Marine Murtskhvaladze, Lela Sabadze, Mari Gavashelidze, Tamar Jashiashvili, Tea Tevdoradze, Ketevan Sidamonidze, Ekaterine Khmaladze, Ekaterine Zhghenti, Roena Sukhiashvili, Mariam Zakalashvili, Lela Urushadze, Magda Dgebuadze, Davit Tsaguria, Ekaterine Zangaladze, Adam Kotorashvili, Maia Alkhazashvili, Irma Burjanadze, Anna Kasradze, Khatuna Zakhashvili, Paata Imnadze, Amiran Gamkrelidze.
EPI_ISL_763074	Diagnosticos da America - DASA	Instituto Adolfo Lutz, Interdiciplinary Procedures Center, Strategic Laboratory	Claudio Tavares Sacchi, Claudia Regina Gonçalves, Erica Valessa Ramos Gomes, Karoline Rodrigues Campos

EPI_ISL_763126, EPI_ISL_763127, EPI_ISL_763174, EPI_ISL_763175, EPI_ISL_763176, EPI_ISL_763177, EPI_ISL_763178, EPI_ISL_763179, EPI_ISL_763189, EPI_ISL_763191, EPI_ISL_763192, EPI_ISL_763194, EPI_ISL_763198, EPI_ISL_763200, EPI_ISL_763202, EPI_ISL_763203, EPI_ISL_763204, EPI_ISL_763205, EPI_ISL_763206, EPI_ISL_763211, EPI_ISL_763214, EPI_ISL_763215, EPI_ISL_763216, EPI_ISL_763217, EPI_ISL_763218, EPI_ISL_763219, EPI_ISL_763221, EPI_ISL_763224, EPI_ISL_763225, EPI_ISL_763226, EPI_ISL_763229, EPI_ISL_763246, EPI_ISL_763247, EPI_ISL_763248, EPI_ISL_763250, EPI_ISL_763252, EPI_ISL_763254, EPI_ISL_763255, EPI_ISL_763256, EPI_ISL_763257, EPI_ISL_763262, EPI_ISL_763263, EPI_ISL_763264, EPI_ISL_763266, EPI_ISL_763267, EPI_ISL_763268, EPI_ISL_763269, EPI_ISL_763270, EPI_ISL_763272, EPI_ISL_763274, EPI_ISL_763279, EPI_ISL_763284, EPI_ISL_763285, EPI_ISL_763286, EPI_ISL_763287, EPI_ISL_763288, EPI_ISL_763291, EPI_ISL_763292, EPI_ISL_763296, EPI_ISL_763297, EPI_ISL_763298, EPI_ISL_763300, EPI_ISL_763303, EPI_ISL_763309, EPI_ISL_763310, EPI_ISL_763311, EPI_ISL_763314, EPI_ISL_763348, EPI_ISL_763356, EPI_ISL_763357, EPI_ISL_763358

see above	Dutch COVID-19 response team	Erasmus Medical Center	Bas Oude Munnink, Reina Sikkema, David Nieuwenhuijse, Irina Chestakova, Anne van der Linden, Marjan Boter, Emmanuelle Munger, Corine GeurtsvanKessel, Annemiek van der Eijk, Richard Molenkamp, Marion Koopmans, on behalf of the Dutch national COVID-19 response team.
EPI_ISL_763829, EPI_ISL_763835	Centre for Enzyme Innovation, University of Portsmouth / Translational Research Laboratory, Portsmouth Hospitals NHS Trust	COVID-19 Genomics UK (COG-UK) Consortium	Angela Beckett,Yann Bourgeois,Garry Scarlett,Sharon Glaysher,Scott Elliott,Kelly Bicknell,Robert Impey,Allyson Lloyd,Sarah Wyllie,Ethan Butcher,Anoop Chauhan,Samuel Robson
EPI_ISL_763841, EPI_ISL_763844, EPI_ISL_763845, EPI_ISL_763846	Oxford Viromics, NDM, University of Oxford; Oxford University Hospitals; Basingstoke and North Hampshire Hospital	COVID-19 Genomics UK (COG-UK) Consortium	Tanya Golubchik, David Bonsall, George Macintyre, Amy Trebes, Mariateresa de Cesare, Catrin Moore, Alex Mobbs, Anita Justice, Robert Shaw, Monique Andersson, Timothy Peto, Emma Wise, Nathan Moore, Jessica Lynch, Nick Cortes, Matilde Mori, Stephen Kidd, David Buck, John Todd, Christophe Fraser
EPI_ISL_763853	Quadram Institute Bioscience	COVID-19 Genomics UK (COG-UK) Consortium	Dave J. Baker, Gemma L. Kay, Alp Aydin, Thanh Le-Viet, Steven Rudder, Ana P. Tedim, Anastasia Kolyva, Maria Diaz, Leonardo de Oliveira Martins, Nabil-Fareed Alikhan, Lizzie Meadows, Rachael Stanley, Ngozi Elumogo, Muhammed Yasir, Nicholas M. Thomson, Alexander J Trotter, Rachel Gilroy, Samuel Bloomfield, Claire Stuart, Andrew Bell, Reenesh Prakash, Samir Dervisevic, Alison E. Mather, John Wain, Mark Webber, Andrew J. Page, Justin O'Grady
EPI_ISL_763855, EPI_ISL_763857	Queens Medical Centre, Clinical Microbiology Department / DeepSeq Nottingham	COVID-19 Genomics UK (COG-UK) Consortium	Gemma Clark, Wendy Smith, Manjinder Khakh, Vicki M Fleming, Michelle M Lister, Hannah Howson-Wells, Jonathan Ball, Patrick McClure, Joseph Chappell, Theocharis Tsoleridis, Nadine Holmes, Matthew Carlisle, Christopher Moore, Fei Sang, Johnny Debebe, Victoria Wright, Matthew Loose
EPI_ISL_763859, EPI_ISL_763860, EPI_ISL_763861, EPI_ISL_763862, EPI_ISL_763863	Oxford Viromics, NDM, University of Oxford; Oxford University Hospitals; Basingstoke and North Hampshire Hospital	COVID-19 Genomics UK (COG-UK) Consortium	Tanya Golubchik, David Bonsall, George Macintyre, Amy Trebes, Mariateresa de Cesare, Catrin Moore, Alex Mobbs, Anita Justice, Robert Shaw, Monique Andersson, Timothy Peto, Emma Wise, Nathan Moore, Jessica Lynch, Nick Cortes, Matilde Mori, Stephen Kidd, David Buck, John Todd, Christophe Fraser
EPI_ISL_763875, EPI_ISL_763876, EPI_ISL_763883, EPI_ISL_763886, EPI_ISL_763887, EPI_ISL_763888, EPI_ISL_763889	Department of Pathology, University of Cambridge	COVID-19 Genomics UK (COG-UK) Consortium	Aminu S. Jahun, Yasmin Chaudhry, Grant Hall, Iliana Georgana, Myra Hosmillo, Martin D. Curran, Malte Pinckert, Surendra Parmar, Ian Goodfellow
EPI_ISL_763892, EPI_ISL_763893, EPI_ISL_763894, EPI_ISL_763895, EPI_ISL_763896, EPI_ISL_763897, EPI_ISL_763898, EPI_ISL_763899, EPI_ISL_763900, EPI_ISL_763902, EPI_ISL_763903, EPI_ISL_763904, EPI_ISL_763905, EPI_ISL_763906			
see above	Virology Department, Royal Infirmary of Edinburgh, NHS Lothian / School of Biological Sciences, University of Edinburgh / Institute of Genetics and Molecular Medicine, University of Edinburgh	COVID-19 Genomics UK (COG-UK) Consortium	McHugh M, Dewar R, Rooke S, Gallagher M, Balcaza C, O'Toole Á, Scher E, Hill V, McCrone JT, Colquhoun R, Yu X, Jackson B, Rambaut A, Williams TC, Templeton K
EPI_ISL_763907	University of Exeter	COVID-19 Genomics UK (COG-UK) Consortium	Ben Temperton,Aaron Jeffries,Michelle Michelsen,Joanna Warwick-Dugdale,Audrey Farbos,Robyn Manley,Stephen Michell,Jane Masoli
EPI_ISL_763912, EPI_ISL_763914, EPI_ISL_763918, EPI_ISL_763920, EPI_ISL_763921, EPI_ISL_763922	Quadram Institute Bioscience	COVID-19 Genomics UK (COG-UK) Consortium	Dave J. Baker, Gemma L. Kay, Alp Aydin, Thanh Le-Viet, Steven Rudder, Ana P. Tedim, Anastasia Kolyva, Maria Diaz, Leonardo de Oliveira Martins, Nabil-Fareed Alikhan, Lizzie Meadows, Rachael Stanley, Ngozi Elumogo, Muhammed Yasir, Nicholas M. Thomson, Alexander J Trotter, Rachel Gilroy, Samuel Bloomfield, Claire Stuart, Andrew Bell, Reenesh Prakash, Samir Dervisevic, Alison E. Mather, John Wain, Mark Webber, Andrew J. Page, Justin O'Grady
EPI_ISL_763926	Queens Medical Centre, Clinical Microbiology Department / DeepSeq Nottingham	COVID-19 Genomics UK (COG-UK) Consortium	Gemma Clark, Wendy Smith, Manjinder Khakh, Vicki M Fleming, Michelle M Lister, Hannah Howson-Wells, Jonathan Ball, Patrick McClure, Joseph Chappell, Theocharis Tsoleridis, Nadine Holmes, Matthew Carlisle, Christopher Moore, Fei Sang, Johnny Debebe, Victoria Wright, Matthew Loose
EPI_ISL_763928, EPI_ISL_763929, EPI_ISL_763932, EPI_ISL_763933, EPI_ISL_763934, EPI_ISL_763935, EPI_ISL_763937, EPI_ISL_763938, EPI_ISL_763939, EPI_ISL_763940, EPI_ISL_763941, EPI_ISL_763943, EPI_ISL_763944, EPI_ISL_763945, EPI_ISL_763947, EPI_ISL_763948, EPI_ISL_763949, EPI_ISL_763950, EPI_ISL_763951, EPI_ISL_763952, EPI_ISL_763953, EPI_ISL_763954, EPI_ISL_763955, EPI_ISL_763956, EPI_ISL_763957, EPI_ISL_763958, EPI_ISL_763959, EPI_ISL_763960, EPI_ISL_763961, EPI_ISL_763962, EPI_ISL_763963, EPI_ISL_763964, EPI_ISL_763965, EPI_ISL_763966, EPI_ISL_763967, EPI_ISL_763968, EPI_ISL_763969, EPI_ISL_763970, EPI_ISL_763971, EPI_ISL_763972, EPI_ISL_763973, EPI_ISL_763974			
see above	Oxford Viromics, NDM, University of Oxford; Oxford University Hospitals; Basingstoke and North Hampshire Hospital	COVID-19 Genomics UK (COG-UK) Consortium	Tanya Golubchik, David Bonsall, George Macintyre, Amy Trebes, Mariateresa de Cesare, Catrin Moore, Alex Mobbs, Anita Justice, Robert Shaw, Monique Andersson, Timothy Peto, Emma Wise, Nathan Moore, Jessica Lynch, Nick Cortes, Matilde Mori, Stephen Kidd, David Buck, John Todd, Christophe Fraser
EPI_ISL_763980, EPI_ISL_763984, EPI_ISL_763985, EPI_ISL_763986, EPI_ISL_763987, EPI_ISL_763989, EPI_ISL_763990, EPI_ISL_763991, EPI_ISL_763994, EPI_ISL_763995, EPI_ISL_763998, EPI_ISL_763999, EPI_ISL_764003, EPI_ISL_764005			
see above	Wales Specialist Virology Centre Sequencing lab: Pathogen Genomics Unit	COVID-19 Genomics UK (COG-UK) Consortium	Catherine Moore, Johnathan Evans, Laura Gifford, Malorie Perry, Simon Cottrell, Angela Marchbank, Alec Birchley, Alexander Adams, Amy Gaskin, Bree Gatica-Wilcox, Jason Coombes, Joel Southgate, Lauren Gilbert, Lee Graham, Nicole Pacchiarini, Sara Kumziene-Summerhayes, Sarah Taylor, Sophie Jones, Sara Rey, Matthew Bull, Joanne Watkins, Sally Corden, Tom Connor
EPI_ISL_764009, EPI_ISL_764010, EPI_ISL_764011, EPI_ISL_764012, EPI_ISL_764013, EPI_ISL_764022	Centre for Enzyme Innovation, University of Portsmouth / Translational Research Laboratory, Portsmouth Hospitals NHS Trust	COVID-19 Genomics UK (COG-UK) Consortium	Angela Beckett,Yann Bourgeois,Garry Scarlett,Sharon Glaysher,Scott Elliott,Kelly Bicknell,Robert Impey,Allyson Lloyd,Sarah Wyllie,Ethan Butcher,Anoop Chauhan,Samuel Robson
EPI_ISL_764033	Virology Department, Sheffield Teaching Hospitals NHS Foundation Trust/Department of Infection, Immunity and Cardiovascular Disease, The Medical School, University of Sheffield	COVID-19 Genomics UK (COG-UK) Consortium	Thushan de Silva, Matthew Parker, Nikki Smith, Adri Angyal, Rebecca Brown, Luke Green, Rachel Tucker, Paul Parsons, Danielle Groves, Katie Johnson, Laura Carrilero, Alex Keeley, Dave Partridge, Matthew Wyles, Benjamin Lindsey, Mehmet Yavuz, Mohammad Raza, Cariad Evans
EPI_ISL_764069, EPI_ISL_764071, EPI_ISL_764072, EPI_ISL_764073, EPI_ISL_764074	Virology Department, Royal Infirmary of Edinburgh, NHS Lothian / School of Biological Sciences, University of Edinburgh / Institute of Genetics and Molecular Medicine, University of Edinburgh	COVID-19 Genomics UK (COG-UK) Consortium	McHugh M, Dewar R, Rooke S, Gallagher M, Balcaza C, O'Toole Á, Scher E, Hill V, McCrone JT, Colquhoun R, Yu X, Jackson B, Rambaut A, Williams TC, Templeton K
EPI_ISL_764077, EPI_ISL_764078, EPI_ISL_764079	Oxford Viromics, NDM, University of Oxford; Oxford University Hospitals; Basingstoke and North Hampshire Hospital	COVID-19 Genomics UK (COG-UK) Consortium	Tanya Golubchik, David Bonsall, George Macintyre, Amy Trebes, Mariateresa de Cesare, Catrin Moore, Alex Mobbs, Anita Justice, Robert Shaw, Monique Andersson, Timothy Peto, Emma Wise, Nathan Moore, Jessica Lynch, Nick Cortes, Matilde Mori, Stephen Kidd, David Buck, John Todd, Christophe Fraser
EPI_ISL_764083	Virology Department, Royal Infirmary of Edinburgh, NHS Lothian / School of Biological Sciences, University of Edinburgh / Institute of Genetics and Molecular Medicine, University of Edinburgh	COVID-19 Genomics UK (COG-UK) Consortium	McHugh M, Dewar R, Rooke S, Gallagher M, Balcaza C, O'Toole Á, Scher E, Hill V, McCrone JT, Colquhoun R, Yu X, Jackson B, Rambaut A, Williams TC, Templeton K
EPI_ISL_764085, EPI_ISL_764087, EPI_ISL_764088, EPI_ISL_764089, EPI_ISL_764090, EPI_ISL_764092, EPI_ISL_764093	Oxford Viromics, NDM, University of Oxford; Oxford University Hospitals; Basingstoke and North Hampshire Hospital	COVID-19 Genomics UK (COG-UK) Consortium	Tanya Golubchik, David Bonsall, George Macintyre, Amy Trebes, Mariateresa de Cesare, Catrin Moore, Alex Mobbs, Anita Justice, Robert Shaw, Monique Andersson, Timothy Peto, Emma Wise, Nathan Moore, Jessica Lynch, Nick Cortes, Matilde Mori, Stephen Kidd, David Buck, John Todd, Christophe Fraser
EPI_ISL_764105	Virology Department, Sheffield Teaching Hospitals NHS Foundation Trust/Department of Infection, Immunity and Cardiovascular Disease, The Medical School, University of Sheffield	COVID-19 Genomics UK (COG-UK) Consortium	Thushan de Silva, Matthew Parker, Nikki Smith, Adri Angyal, Rebecca Brown, Luke Green, Rachel Tucker, Paul Parsons, Danielle Groves, Katie Johnson, Laura Carrilero, Alex Keeley, Dave Partridge, Matthew Wyles, Benjamin Lindsey, Mehmet Yavuz, Mohammad Raza, Cariad Evans
EPI_ISL_764113, EPI_ISL_764115	Quadram Institute Bioscience	COVID-19 Genomics UK (COG-UK) Consortium	Dave J. Baker, Gemma L. Kay, Alp Aydin, Thanh Le-Viet, Steven Rudder, Ana P. Tedim, Anastasia Kolyva, Maria Diaz, Leonardo de Oliveira Martins, Nabil-Fareed Alikhan, Lizzie Meadows, Rachael Stanley, Ngozi Elumogo, Muhammed Yasir, Nicholas M. Thomson, Alexander J Trotter, Rachel Gilroy, Samuel Bloomfield, Claire Stuart, Andrew Bell, Reenesh Prakash, Samir Dervisevic, Alison E. Mather, John Wain, Mark Webber, Andrew J. Page, Justin O'Grady
EPI_ISL_764117	Oxford Viromics, NDM, University of Oxford; Oxford University Hospitals; Basingstoke and North Hampshire Hospital	COVID-19 Genomics UK (COG-UK) Consortium	Tanya Golubchik, David Bonsall, George Macintyre, Amy Trebes, Mariateresa de Cesare, Catrin Moore, Alex Mobbs, Anita Justice, Robert Shaw, Monique Andersson, Timothy Peto, Emma Wise, Nathan Moore, Jessica Lynch, Nick Cortes, Matilde Mori, Stephen Kidd, David Buck, John Todd, Christophe Fraser
EPI_ISL_764123	Centre for Enzyme Innovation, University of Portsmouth / Translational Research Laboratory, Portsmouth Hospitals NHS Trust	COVID-19 Genomics UK (COG-UK) Consortium	Angela Beckett,Yann Bourgeois,Garry Scarlett,Sharon Glaysher,Scott Elliott,Kelly Bicknell,Robert Impey,Allyson Lloyd,Sarah Wyllie,Ethan Butcher,Anoop Chauhan,Samuel Robson

EPI_ISL_764243	Oxford Viromics, NDM, University of Oxford; Oxford University Hospitals; Basingstoke and North Hampshire Hospital	COVID-19 Genomics UK (COG-UK) Consortium	Tanya Golubchik, David Bonsall, George Macintyre, Amy Trebes, Mariateresa de Cesare, Catrin Moore, Alex Mobbs, Anita Justice, Robert Shaw, Monique Andersson, Timothy Peto, Emma Wise, Nathan Moore, Jessica Lynch, Nick Cortes, Matilde Mori, Stephen Kidd, David Buck, John Todd, Christophe Fraser
EPI_ISL_764304, EPI_ISL_764316, EPI_ISL_764319, EPI_ISL_764322, EPI_ISL_764330, EPI_ISL_764345, EPI_ISL_764347, EPI_ISL_764352, EPI_ISL_764354	Department of Pathology, University of Cambridge	COVID-19 Genomics UK (COG-UK) Consortium	Aminu S. Jahun, Yasmin Chaudhry, Grant Hall, Iliana Georgana, Myra Hosmillo, Martin D. Curran, Malte Pinckert, Surendra Parmar, Ian Goodfellow
EPI_ISL_764358, EPI_ISL_764359, EPI_ISL_764361, EPI_ISL_764362, EPI_ISL_764364, EPI_ISL_764365, EPI_ISL_764369, EPI_ISL_764371, EPI_ISL_764372	Virology Department, Royal Infirmary of Edinburgh, NHS Lothian / School of Biological Sciences, University of Edinburgh / Institute of Genetics and Molecular Medicine, University of Edinburgh	COVID-19 Genomics UK (COG-UK) Consortium	McHugh M, Dewar R, Rooke S, Gallagher M, Balcaza C, O'Toole Á, Scher E, Hill V, McCrone JT, Colquhoun R, Yu X, Jackson B, Rambaut A, Williams TC, Templeton K
EPI_ISL_764373, EPI_ISL_764378, EPI_ISL_764382	University of Exeter	COVID-19 Genomics UK (COG-UK) Consortium	Ben Temperton,Aaron Jeffries,Michelle Michelsen,Joanna Warwick-Dugdale,Audrey Farbos,Robyn Manley,Stephen Michell,Jane Masoli
EPI_ISL_764447, EPI_ISL_764450, EPI_ISL_764454, EPI_ISL_764455, EPI_ISL_764457, EPI_ISL_764458, EPI_ISL_764459, EPI_ISL_764460, EPI_ISL_764466, EPI_ISL_764470, EPI_ISL_764473, EPI_ISL_764475, EPI_ISL_764480, EPI_ISL_764481, EPI_ISL_764483, EPI_ISL_764487			
see above	Quadram Institute Bioscience	COVID-19 Genomics UK (COG-UK) Consortium	Dave J. Baker, Gemma L. Kay, Alp Aydin, Thanh Le-Viet, Steven Rudder, Ana P. Tedim, Anastasia Kolyva, Maria Diaz, Leonardo de Oliveira Martins, Nabil-Fareed Alikhan, Lizzie Meadows, Rachael Stanley, Ngozi Elumogo, Muhammed Yasir, Nicholas M. Thomson, Alexander J Trotter, Rachel Gilroy, Samuel Bloomfield, Claire Stuart, Andrew Bell, Reenesh Prakash, Samir Dervisevic, Alison E. Mather, John Wain, Mark Webber, Andrew J. Page, Justin O'Grady
EPI_ISL_764494, EPI_ISL_764497, EPI_ISL_764498, EPI_ISL_764500, EPI_ISL_764504, EPI_ISL_764505, EPI_ISL_764510, EPI_ISL_764513, EPI_ISL_764514, EPI_ISL_764515, EPI_ISL_764516, EPI_ISL_764517, EPI_ISL_764518, EPI_ISL_764520, EPI_ISL_764522, EPI_ISL_764523			
see above	Queens Medical Centre, Clinical Microbiology Department / DeepSeq Nottingham	COVID-19 Genomics UK (COG-UK) Consortium	Gemma Clark, Wendy Smith, Manjinder Khakh, Vicki M Fleming, Michelle M Lister, Hannah Howson-Wells, Jonathan Ball, Patrick McClure, Joseph Chappell, Theocharis Tsoleridis, Nadine Holmes, Matthew Carlisle, Christopher Moore, Fei Sang, Johnny Debebe, Victoria Wright, Matthew Loose
EPI_ISL_764525, EPI_ISL_764526, EPI_ISL_764527, EPI_ISL_764528, EPI_ISL_764529, EPI_ISL_764530, EPI_ISL_764531, EPI_ISL_764532, EPI_ISL_764533, EPI_ISL_764534, EPI_ISL_764535, EPI_ISL_764536, EPI_ISL_764537, EPI_ISL_764538, EPI_ISL_764539, EPI_ISL_764540, EPI_ISL_764541, EPI_ISL_764542, EPI_ISL_764543, EPI_ISL_764544, EPI_ISL_764545, EPI_ISL_764546, EPI_ISL_764547, EPI_ISL_764548, EPI_ISL_764549, EPI_ISL_764550, EPI_ISL_764551, EPI_ISL_764552, EPI_ISL_764553, EPI_ISL_764554, EPI_ISL_764555, EPI_ISL_764556, EPI_ISL_764558, EPI_ISL_764559, EPI_ISL_764560, EPI_ISL_764561, EPI_ISL_764562, EPI_ISL_764563, EPI_ISL_764564, EPI_ISL_764565, EPI_ISL_764566, EPI_ISL_764567, EPI_ISL_764568, EPI_ISL_764569, EPI_ISL_764570, EPI_ISL_764571, EPI_ISL_764572			
see above	Oxford Viromics, NDM, University of Oxford; Oxford University Hospitals; Basingstoke and North Hampshire Hospital	COVID-19 Genomics UK (COG-UK) Consortium	Tanya Golubchik, David Bonsall, George Macintyre, Amy Trebes, Mariateresa de Cesare, Catrin Moore, Alex Mobbs, Anita Justice, Robert Shaw, Monique Andersson, Timothy Peto, Emma Wise, Nathan Moore, Jessica Lynch, Nick Cortes, Matilde Mori, Stephen Kidd, David Buck, John Todd, Christophe Fraser
EPI_ISL_764578, EPI_ISL_764611, EPI_ISL_764613, EPI_ISL_764616, EPI_ISL_764617, EPI_ISL_764628, EPI_ISL_764634, EPI_ISL_764635, EPI_ISL_764637, EPI_ISL_764641, EPI_ISL_764666, EPI_ISL_764694, EPI_ISL_764699, EPI_ISL_764711, EPI_ISL_764717, EPI_ISL_764729, EPI_ISL_764730, EPI_ISL_764751, EPI_ISL_764752, EPI_ISL_764753, EPI_ISL_764755, EPI_ISL_764757, EPI_ISL_764758, EPI_ISL_764761, EPI_ISL_764762, EPI_ISL_764764, EPI_ISL_764778, EPI_ISL_764788, EPI_ISL_764790, EPI_ISL_764804, EPI_ISL_764821, EPI_ISL_764827, EPI_ISL_764831, EPI_ISL_764844, EPI_ISL_764854, EPI_ISL_764855, EPI_ISL_764859, EPI_ISL_764863, EPI_ISL_764870, EPI_ISL_764872, EPI_ISL_764873, EPI_ISL_764874, EPI_ISL_764875, EPI_ISL_764880, EPI_ISL_764881, EPI_ISL_764883, EPI_ISL_764884, EPI_ISL_764887, EPI_ISL_764888, EPI_ISL_764893, EPI_ISL_764894, EPI_ISL_764899, EPI_ISL_764901, EPI_ISL_764902, EPI_ISL_764904, EPI_ISL_764905, EPI_ISL_764916, EPI_ISL_764917, EPI_ISL_764918, EPI_ISL_764920, EPI_ISL_764921, EPI_ISL_764926, EPI_ISL_764928, EPI_ISL_764929, EPI_ISL_764931, EPI_ISL_764933, EPI_ISL_764935, EPI_ISL_764936, EPI_ISL_764937, EPI_ISL_764943, EPI_ISL_764946, EPI_ISL_764947, EPI_ISL_764949, EPI_ISL_764950, EPI_ISL_764955, EPI_ISL_764956, EPI_ISL_764963, EPI_ISL_764978, EPI_ISL_764982, EPI_ISL_764985, EPI_ISL_764988, EPI_ISL_764991, EPI_ISL_764995, EPI_ISL_765005, EPI_ISL_765006, EPI_ISL_765007, EPI_ISL_765008, EPI_ISL_765009, EPI_ISL_765015, EPI_ISL_765016, EPI_ISL_765017, EPI_ISL_765025, EPI_ISL_765026, EPI_ISL_765028, EPI_ISL_765033, EPI_ISL_765034, EPI_ISL_765037, EPI_ISL_765042, EPI_ISL_765043, EPI_ISL_765047, EPI_ISL_765048, EPI_ISL_765049, EPI_ISL_765060, EPI_ISL_765061, EPI_ISL_765065, EPI_ISL_765068, EPI_ISL_765069, EPI_ISL_765072, EPI_ISL_765074, EPI_ISL_765076, EPI_ISL_765078, EPI_ISL_765081, EPI_ISL_765083, EPI_ISL_765107, EPI_ISL_765108, EPI_ISL_765109			
see above	Wales Specialist Virology Centre Sequencing lab: Pathogen Genomics Unit	COVID-19 Genomics UK (COG-UK) Consortium	Catherine Moore, Johnathan Evans, Laura Gifford, Malorie Perry, Simon Cottrell, Angela Marchbank, Alec Birchley, Alexander Adams, Amy Gaskin, Bree Gatica-Wilcox, Jason Coombes, Joel Southgate, Lauren Gilbert, Lee Graham, Nicole Pacchiarini, Sara Kumziene-Summerhayes, Sarah Taylor, Sophie Jones, Sara Rey, Matthew Bull, Joanne Watkins, Sally Corden, Tom Connor
EPI_ISL_765127, EPI_ISL_765135	Centre for Enzyme Innovation, University of Portsmouth / Translational Research Laboratory, Portsmouth Hospitals NHS Trust	COVID-19 Genomics UK (COG-UK) Consortium	Angela Beckett,Yann Bourgeois,Garry Scarlett,Sharon Glaysher,Scott Elliott,Kelly Bicknell,Robert Impey,Allyson Lloyd,Sarah Wyllie,Ethan Butcher,Anoop Chauhan,Samuel Robson
EPI_ISL_765155, EPI_ISL_765158, EPI_ISL_765165, EPI_ISL_765171, EPI_ISL_765174, EPI_ISL_765176, EPI_ISL_765183, EPI_ISL_765198, EPI_ISL_765208	Virology Department, Sheffield Teaching Hospitals NHS Foundation Trust/Department of Infection, Immunity and Cardiovascular Disease, The Medical School, University of Sheffield	COVID-19 Genomics UK (COG-UK) Consortium	Thushan de Silva, Matthew Parker, Nikki Smith, Adri Angyal, Rebecca Brown, Luke Green, Rachel Tucker, Paul Parsons, Danielle Groves, Katie Johnson, Laura Carrilero, Alex Keeley, Dave Partridge, Matthew Wyles, Benjamin Lindsey, Mehmet Yavuz, Mohammad Raza, Cariad Evans
EPI_ISL_765478	EHPAD	National Reference Center for Viruses of Respiratory Infections, Institut Pasteur, Paris	Marion Barbet, Sylvie Behillil, Méline Bizard, Angela Brisebarre, Camille Capel, Etienne Simon-Lorière, Vincent Enouf, Maud Vanpeene, Sylvie van der Werf
EPI_ISL_765517, EPI_ISL_765518, EPI_ISL_765519, EPI_ISL_765524, EPI_ISL_765525	SARATOGA HOSPITAL LABORATORY	Wadsworth Center, New York State Department.of Health	Kirsten St. George, Daryl M. Lamson, Alexis Russel, Matthew Shudt, Melissa A Leisner, Jonathan Plitnick, Navjot Singh, John Kelly, Sara Griesemer, Erasmus Schneider, Erica Lasek-Nesselquist
EPI_ISL_765533, EPI_ISL_765541, EPI_ISL_765544, EPI_ISL_765547, EPI_ISL_765555, EPI_ISL_765562, EPI_ISL_765563, EPI_ISL_765566	MONTEFIORE MEDICAL CENTER LABORATORIES	Wadsworth Center, New York State Department.of Health	Kirsten St. George, Daryl M. Lamson, Alexis Russel, Matthew Shudt, Melissa A Leisner, Jonathan Plitnick, Navjot Singh, John Kelly, Sara Griesemer, Erasmus Schneider, Erica Lasek-Nesselquist
EPI_ISL_765567	National Institute for Infectious Diseases, INMI, "L. Spallanzani" IRCCS	National Institute for Infectious Diseases, INMI, "L. Spallanzani" IRCCS	E Giombini, C.E.M Gruber, M Rueca, B Bartolini, O Butera, F Messina, A Di Caro, G Parisi, MR Capobianchi
EPI_ISL_765570	National Institute for Infectious Diseases, INMI, "L. Spallanzani" IRCCS	National Institute for Infectious Diseases, INMI, "L. Spallanzani" IRCCS	M. Rueca, C.E.M Gruber, B Bartolini, O Butera, F Messina, E Giombini, A Di Caro, MR Capobianchi
EPI_ISL_765572	National Institute for Infectious Diseases, INMI, "L. Spallanzani" IRCCS	National Institute for Infectious Diseases, INMI, "L. Spallanzani" IRCCS	C.E.M Gruber, M Rueca, B Bartolini, F Messina, E Giombini,O Butera, A Di Caro, MR Capobianchi
EPI_ISL_765573	National Institute for Infectious Diseases, INMI, "L. Spallanzani" IRCCS	National Institute for Infectious Diseases, INMI, "L. Spallanzani" IRCCS	F Messina, E Giombini, M Rueca, B Bartolini, C.E.M Gruber, MR Capobianchi, O Butera, A Di Caro
EPI_ISL_765819, EPI_ISL_765821, EPI_ISL_765823	North Shore Medical Center	Infectious Disease Program, Broad Institute of Harvard and MIT	Lemieux,J.E., Siddle,K.J., Shaw,B., Adams,G., Pierce,V., Turbett,S., Anahtar,M., Branda,J., Slater,D., Harris,J., Lin,A.E., Gladden-Young,A., Lagerborg,K., Rudy,M., DeRuff,K., Carter,A., Normandin,E., Bauer,M., Reilly,S., Tomkins-Tinch,C., Loreth,C., Chaluvadi,S., Neumann,A., Cusick,C., Chapman,S.B., Gnirke,A., Flowers,K., Cerrato,F., Birren,B.W., Gallagher,G., Smole,S., Park,D.J., MacInnis,B.L., Ryan,E., LaRocque,R., Rosenberg,E. and Sabeti,P.C.
EPI_ISL_765889	Massachusetts General Hospital	Infectious Disease Program, Broad Institute of Harvard and MIT	Lemieux,J.E., Siddle,K.J., Shaw,B., Adams,G., Pierce,V., Turbett,S., Anahtar,M., Branda,J., Slater,D., Harris,J., Lin,A.E., Gladden-Young,A., Lagerborg,K., Rudy,M., DeRuff,K., Carter,A., Normandin,E., Bauer,M., Reilly,S., Tomkins-Tinch,C., Loreth,C., Chaluvadi,S., Neumann,A., Cusick,C., Chapman,S.B., Gnirke,A., Flowers,K., Cerrato,F., Birren,B.W., Gallagher,G., Smole,S., Park,D.J., MacInnis,B.L., Ryan,E., LaRocque,R., Rosenberg,E. and Sabeti,P.C.
EPI_ISL_765961, EPI_ISL_765966, EPI_ISL_765967, EPI_ISL_765970, EPI_ISL_765972, EPI_ISL_765975, EPI_ISL_765976, EPI_ISL_765978	Wyoming Public Health Laboratory	Wyoming Public Health Laboratory	Noah Hull, Taylor Fearing, Channing Weber, Ashley Norberg, Bailey Bowcutt, and Wanda Manley
EPI_ISL_765987, EPI_ISL_765989, EPI_ISL_765992	UCLA Clinical Micro Lab	Los Angeles County PHL	P. Hemarajata et al.
EPI_ISL_765999, EPI_ISL_766004,	Los Angeles County PHL	Los Angeles County PHL	P. Hemarajata et al.

EPI_ISL_766006, EPI_ISL_766013			
EPI_ISL_766016, EPI_ISL_766017, EPI_ISL_766018, EPI_ISL_766019, EPI_ISL_766020, EPI_ISL_766021, EPI_ISL_766022, EPI_ISL_766023, EPI_ISL_766024, EPI_ISL_766025, EPI_ISL_766026, EPI_ISL_766027			
see above	Department of Virology and Immunology, University of Helsinki and Helsinki University Hospital, Huslab Finland	Department of Virology, Faculty of Medicine, University of Helsinki, Helsinki, Finland	Teemu Smura, Olli Vapalahti, Maija Lappalainen, Satu Kurkela
EPI_ISL_766120, EPI_ISL_766125, EPI_ISL_766127, EPI_ISL_766136, EPI_ISL_766137, EPI_ISL_766143, EPI_ISL_766147, EPI_ISL_766148, EPI_ISL_766150, EPI_ISL_766154, EPI_ISL_766156, EPI_ISL_766158, EPI_ISL_766159, EPI_ISL_766160, EPI_ISL_766161, EPI_ISL_766162, EPI_ISL_766165, EPI_ISL_766169, EPI_ISL_766170, EPI_ISL_766173, EPI_ISL_766176, EPI_ISL_766181, EPI_ISL_766182, EPI_ISL_766183, EPI_ISL_766184, EPI_ISL_766185, EPI_ISL_766195, EPI_ISL_766200, EPI_ISL_766201, EPI_ISL_766204, EPI_ISL_766207, EPI_ISL_766209, EPI_ISL_766214, EPI_ISL_766216, EPI_ISL_766218, EPI_ISL_766220, EPI_ISL_766226, EPI_ISL_766229, EPI_ISL_766248, EPI_ISL_766249, EPI_ISL_766258, EPI_ISL_766298, EPI_ISL_766301, EPI_ISL_766310, EPI_ISL_766312, EPI_ISL_766317, EPI_ISL_766395, EPI_ISL_766399, EPI_ISL_766401, EPI_ISL_766411, EPI_ISL_766412, EPI_ISL_766416, EPI_ISL_766418, EPI_ISL_766422, EPI_ISL_766424, EPI_ISL_766431, EPI_ISL_766433, EPI_ISL_766434, EPI_ISL_766437, EPI_ISL_766438, EPI_ISL_766443, EPI_ISL_766444, EPI_ISL_766446, EPI_ISL_766448, EPI_ISL_766449, EPI_ISL_766450, EPI_ISL_766453, EPI_ISL_766456, EPI_ISL_766457, EPI_ISL_766461, EPI_ISL_766475, EPI_ISL_766476, EPI_ISL_766478, EPI_ISL_766479, EPI_ISL_766483, EPI_ISL_766485, EPI_ISL_766492, EPI_ISL_766495, EPI_ISL_766496, EPI_ISL_766497, EPI_ISL_766498, EPI_ISL_766502, EPI_ISL_766504, EPI_ISL_766506, EPI_ISL_766507, EPI_ISL_766515, EPI_ISL_766555, EPI_ISL_766565, EPI_ISL_766567			
see above	Respiratory Virus Unit, National Infection Service, Public Health England	COVID-19 Genomics UK (COG-UK) Consortium	PHE Covid Sequencing Team
EPI_ISL_766569	Oman-National Influenza Center	Oman-National Influenza Center	Samiha Al-Kharusi, Laila Al-Balushi, Hamida Al-Barwani, Aisha Al-Busaidi, Intisar Al-Shukri, Samira Al-Mahruqi, Hanan Al-Kindi, Amina Al-Jardani
EPI_ISL_766574, EPI_ISL_766575, EPI_ISL_766576, EPI_ISL_766577, EPI_ISL_766578, EPI_ISL_766579	ULSS 8 Berica	Istituto Zooprofilattico Sperimentale delle Venezie	Adelaide Milani, Alessia Schivo, Annalisa Salviato, Erika Giorgia Quaranta, Ambra Pastori, Bianca Zecchin, Alice Fusaro, Isabella Monne, Calogero Terregino, Antonia Ricci
EPI_ISL_766580, EPI_ISL_766581	ULSS 2 Treviso	Istituto Zooprofilattico Sperimentale delle Venezie	Adelaide Milani, Alessia Schivo, Annalisa Salviato, Erika Giorgia Quaranta, Ambra Pastori, Bianca Zecchin, Alice Fusaro, Isabella Monne, Calogero Terregino, Antonia Ricci
EPI_ISL_766585	Triemli Hospital	Institute of Medical Virology, University of Zurich	Stefan Schmutz, Maryam Zaheri, Verena Kufner, Annette Audigé, Maria Grünberg, Kevin Steiner, Jon Huder, Cyril Shah, Riccarda Capaul, Jürg Böni, Michael Huber, Alexandra Trkola
EPI_ISL_766586	Limmattal Hospital	Institute of Medical Virology, University of Zurich	Stefan Schmutz, Maryam Zaheri, Verena Kufner, Annette Audigé, Maria Grünberg, Kevin Steiner, Jon Huder, Cyril Shah, Riccarda Capaul, Jürg Böni, Michael Huber, Alexandra Trkola
EPI_ISL_766590	Dr. Boubaker Karim laboratory	Institute of Medical Virology, University of Zurich	Stefan Schmutz, Maryam Zaheri, Verena Kufner, Annette Audigé, Maria Grünberg, Kevin Steiner, Jon Huder, Cyril Shah, Riccarda Capaul, Jürg Böni, Michael Huber, Alexandra Trkola
EPI_ISL_766594, EPI_ISL_766595, EPI_ISL_766596, EPI_ISL_766597, EPI_ISL_766598, EPI_ISL_766599, EPI_ISL_766600, EPI_ISL_766601, EPI_ISL_766602, EPI_ISL_766603, EPI_ISL_766604, EPI_ISL_766605, EPI_ISL_766606, EPI_ISL_766607			
see above	The Public Health Agency of Sweden	The Public Health Agency of Sweden	Department of Microbiology, The Public Health Agency of Sweden
EPI_ISL_766608, EPI_ISL_766609, EPI_ISL_766610, EPI_ISL_766622	Klinisk mikrobiologi	The Public Health Agency of Sweden	Department of Microbiology, The Public Health Agency of Sweden
EPI_ISL_766630, EPI_ISL_766631, EPI_ISL_766632	Klinisk mikrobiologi, Viruslab	The Public Health Agency of Sweden	Department of Microbiology, The Public Health Agency of Sweden
EPI_ISL_766633, EPI_ISL_766634, EPI_ISL_766635, EPI_ISL_766636, EPI_ISL_766637, EPI_ISL_766638, EPI_ISL_766639	Klinisk mikrobiologi	The Public Health Agency of Sweden	Department of Microbiology, The Public Health Agency of Sweden
EPI_ISL_766640, EPI_ISL_766641	Laboratoriemedicin, Klinisk mikrobiologi	The Public Health Agency of Sweden	Department of Microbiology, The Public Health Agency of Sweden
EPI_ISL_766644	Vardcentralen Brinken	The Public Health Agency of Sweden	Department of Microbiology, The Public Health Agency of Sweden
EPI_ISL_766692, EPI_ISL_766693	Klinisk mikrobiologi, Laboratoriemedicin	The Public Health Agency of Sweden	Department of Microbiology, The Public Health Agency of Sweden
EPI_ISL_766694	Klinisk mikrobiologi	The Public Health Agency of Sweden	Department of Microbiology, The Public Health Agency of Sweden
EPI_ISL_766695	A05 Biomedicum	The Public Health Agency of Sweden	Department of Microbiology, The Public Health Agency of Sweden
EPI_ISL_766696	Klinisk Mikrobiologi	The Public Health Agency of Sweden	Department of Microbiology, The Public Health Agency of Sweden
EPI_ISL_766697	Klinisk mikrobiologi, bakteriologi	The Public Health Agency of Sweden	Department of Microbiology, The Public Health Agency of Sweden
EPI_ISL_766698, EPI_ISL_766699	A05 Biomedicum	The Public Health Agency of Sweden	Department of Microbiology, The Public Health Agency of Sweden
EPI_ISL_766700, EPI_ISL_766701, EPI_ISL_766702, EPI_ISL_766703, EPI_ISL_766704, EPI_ISL_766705, EPI_ISL_766706	The Public Health Agency of Sweden	The Public Health Agency of Sweden	Department of Microbiology, The Public Health Agency of Sweden
EPI_ISL_766707	Klinisk mikrobiologi	The Public Health Agency of Sweden	Department of Microbiology, The Public Health Agency of Sweden
EPI_ISL_766708	A05 Biomedicum	The Public Health Agency of Sweden	Department of Microbiology, The Public Health Agency of Sweden
EPI_ISL_766710, EPI_ISL_766711, EPI_ISL_766712	Klinisk mikrobiologi	The Public Health Agency of Sweden	Department of Microbiology, The Public Health Agency of Sweden
EPI_ISL_766713	Klinisk Mikrobiologi	The Public Health Agency of Sweden	Department of Microbiology, The Public Health Agency of Sweden
EPI_ISL_766714	Klinisk mikrobiologi	The Public Health Agency of Sweden	Department of Microbiology, The Public Health Agency of Sweden
EPI_ISL_766715, EPI_ISL_766716, EPI_ISL_766717, EPI_ISL_766718, EPI_ISL_766719, EPI_ISL_766720, EPI_ISL_766721, EPI_ISL_766722, EPI_ISL_766723	A05 Biomedicum	The Public Health Agency of Sweden	Department of Microbiology, The Public Health Agency of Sweden
EPI_ISL_766724	TATAA Biocenter	The Public Health Agency of Sweden	Department of Microbiology, The Public Health Agency of Sweden
EPI_ISL_766725, EPI_ISL_766726, EPI_ISL_766727, EPI_ISL_766728, EPI_ISL_766729, EPI_ISL_766730	Klinisk mikrobiologi	The Public Health Agency of Sweden	Department of Microbiology, The Public Health Agency of Sweden
EPI_ISL_766777, EPI_ISL_766779, EPI_ISL_766785	Wyoming Public Health Laboratory	Wyoming Public Health Laboratory	Noah Hull, Taylor Fearing, Channing Weber, Ashley Norberg, Bailey Bowcutt, and Wanda Manley
EPI_ISL_766859	Respiratory Virus Unit, National Infection Service, Public Health England	COVID-19 Genomics UK (COG-UK) Consortium	PHE Covid Sequencing Team
EPI_ISL_766901	New Mexico Department of Health Scientific Laboratory	New Mexico Department of Health Scientific Laboratory	D'eldra Malone, Ellie Johnson, Anastacia Griego-Fisher
EPI_ISL_766997, EPI_ISL_767005, EPI_ISL_767010	Delaware Public Health Laboratory	Delaware Public Health Laboratory	Gregory Hovan
EPI_ISL_767013	University of Bari Biomedical Sciences and Human Oncology	University of Bari Biomedical Sciences and Human Oncology	Maria Chironna, Anna Sallustio, Daniela Loconsole, Marisa Accogli
EPI_ISL_767053, EPI_ISL_767054, EPI_ISL_767055, EPI_ISL_767056, EPI_ISL_767060, EPI_ISL_767061, EPI_ISL_767064, EPI_ISL_767065, EPI_ISL_767066, EPI_ISL_767068, EPI_ISL_767069, EPI_ISL_767073, EPI_ISL_767074, EPI_ISL_767075, EPI_ISL_767076, EPI_ISL_767077, EPI_ISL_767078, EPI_ISL_767080,			

EPI_ISL_767081, EPI_ISL_767082, EPI_ISL_767085			
see above	New Mexico Department of Health Scientific Laboratory	New Mexico Department of Health Scientific Laboratory	D'eldra Malone, Ellie Johnson, Anastacia Griego-Fisher
EPI_ISL_767091	Lighthouse Lab in Alderley Park	Wellcome Sanger Institute for the COVID-19 Genomics UK (COG-UK) Consortium	Jacquelyn Wynn, Mairead Hyland, The Lighthouse Lab in Alderley Park and Alex Alderton, Roberto Amato, Sonia Goncalves, Ewan Harrison, David K. Jackson, Ian Johnston, Dominic Kwiatkowski, Cordelia Langford, John Sillitoe on behalf of the Wellcome Sanger Institute COVID-19 Surveillance Team
EPI_ISL_767092	Lighthouse Lab in Glasgow	Wellcome Sanger Institute for the COVID-19 Genomics UK (COG-UK) Consortium	Harper VanSteenhouse, Yumi Kasai, David Gray, Carol Clugston, Anna Dominiczak and Alex Alderton, Roberto Amato, Sonia Goncalves, Ewan Harrison, David K. Jackson, Ian Johnston, Dominic Kwiatkowski, Cordelia Langford, John Sillitoe on behalf of the Wellcome Sanger Institute COVID-19 Surveillance Team
EPI_ISL_767093, EPI_ISL_767094	Lighthouse Lab in Alderley Park	Wellcome Sanger Institute for the COVID-19 Genomics UK (COG-UK) Consortium	Jacquelyn Wynn, Mairead Hyland, The Lighthouse Lab in Alderley Park and Alex Alderton, Roberto Amato, Sonia Goncalves, Ewan Harrison, David K. Jackson, Ian Johnston, Dominic Kwiatkowski, Cordelia Langford, John Sillitoe on behalf of the Wellcome Sanger Institute COVID-19 Surveillance Team
EPI_ISL_767096	Lighthouse Lab in Glasgow	Wellcome Sanger Institute for the COVID-19 Genomics UK (COG-UK) Consortium	Harper VanSteenhouse, Yumi Kasai, David Gray, Carol Clugston, Anna Dominiczak and Alex Alderton, Roberto Amato, Sonia Goncalves, Ewan Harrison, David K. Jackson, Ian Johnston, Dominic Kwiatkowski, Cordelia Langford, John Sillitoe on behalf of the Wellcome Sanger Institute COVID-19 Surveillance Team
EPI_ISL_767097	Lighthouse Lab in Alderley Park	Wellcome Sanger Institute for the COVID-19 Genomics UK (COG-UK) Consortium	Jacquelyn Wynn, Mairead Hyland, The Lighthouse Lab in Alderley Park and Alex Alderton, Roberto Amato, Sonia Goncalves, Ewan Harrison, David K. Jackson, Ian Johnston, Dominic Kwiatkowski, Cordelia Langford, John Sillitoe on behalf of the Wellcome Sanger Institute COVID-19 Surveillance Team
EPI_ISL_767099, EPI_ISL_767100, EPI_ISL_767101	Lighthouse Lab in Milton Keynes	Wellcome Sanger Institute for the COVID-19 Genomics UK (COG-UK) Consortium	The Lighthouse Lab in Milton Keynes and Alex Alderton, Roberto Amato, Sonia Goncalves, Ewan Harrison, David K. Jackson, Ian Johnston, Dominic Kwiatkowski, Cordelia Langford, John Sillitoe on behalf of the Wellcome Sanger Institute COVID-19 Surveillance Team
EPI_ISL_767103, EPI_ISL_767104, EPI_ISL_767105, EPI_ISL_767106	Lighthouse Lab in Alderley Park	Wellcome Sanger Institute for the COVID-19 Genomics UK (COG-UK) Consortium	Jacquelyn Wynn, Mairead Hyland, The Lighthouse Lab in Alderley Park and Alex Alderton, Roberto Amato, Sonia Goncalves, Ewan Harrison, David K. Jackson, Ian Johnston, Dominic Kwiatkowski, Cordelia Langford, John Sillitoe on behalf of the Wellcome Sanger Institute COVID-19 Surveillance Team
EPI_ISL_767107, EPI_ISL_767108, EPI_ISL_767111, EPI_ISL_767112	Lighthouse Lab in Milton Keynes	Wellcome Sanger Institute for the COVID-19 Genomics UK (COG-UK) Consortium	The Lighthouse Lab in Milton Keynes and Alex Alderton, Roberto Amato, Sonia Goncalves, Ewan Harrison, David K. Jackson, Ian Johnston, Dominic Kwiatkowski, Cordelia Langford, John Sillitoe on behalf of the Wellcome Sanger Institute COVID-19 Surveillance Team
EPI_ISL_767113, EPI_ISL_767114	Lighthouse Lab in Glasgow	Wellcome Sanger Institute for the COVID-19 Genomics UK (COG-UK) Consortium	Harper VanSteenhouse, Yumi Kasai, David Gray, Carol Clugston, Anna Dominiczak and Alex Alderton, Roberto Amato, Sonia Goncalves, Ewan Harrison, David K. Jackson, Ian Johnston, Dominic Kwiatkowski, Cordelia Langford, John Sillitoe on behalf of the Wellcome Sanger Institute COVID-19 Surveillance Team
EPI_ISL_767115	Lighthouse Lab in Cambridge	Wellcome Sanger Institute for the COVID-19 Genomics UK (COG-UK) Consortium	Rob Howes, The Lighthouse Lab in Cambridge and Alex Alderton, Roberto Amato, Sonia Goncalves, Ewan Harrison, David K. Jackson, Ian Johnston, Dominic Kwiatkowski, Cordelia Langford, John Sillitoe on behalf of the Wellcome Sanger Institute COVID-19 Surveillance Team
EPI_ISL_767116	Lighthouse Lab in Alderley Park	Wellcome Sanger Institute for the COVID-19 Genomics UK (COG-UK) Consortium	Jacquelyn Wynn, Mairead Hyland, The Lighthouse Lab in Alderley Park and Alex Alderton, Roberto Amato, Sonia Goncalves, Ewan Harrison, David K. Jackson, Ian Johnston, Dominic Kwiatkowski, Cordelia Langford, John Sillitoe on behalf of the Wellcome Sanger Institute COVID-19 Surveillance Team
EPI_ISL_767118	Lighthouse Lab in Glasgow	Wellcome Sanger Institute for the COVID-19 Genomics UK (COG-UK) Consortium	Harper VanSteenhouse, Yumi Kasai, David Gray, Carol Clugston, Anna Dominiczak and Alex Alderton, Roberto Amato, Sonia Goncalves, Ewan Harrison, David K. Jackson, Ian Johnston, Dominic Kwiatkowski, Cordelia Langford, John Sillitoe on behalf of the Wellcome Sanger Institute COVID-19 Surveillance Team
EPI_ISL_767120, EPI_ISL_767121, EPI_ISL_767122, EPI_ISL_767123, EPI_ISL_767124, EPI_ISL_767127, EPI_ISL_767129, EPI_ISL_767130, EPI_ISL_767131, EPI_ISL_767132, EPI_ISL_767133			
see above	Lighthouse Lab in Alderley Park	Wellcome Sanger Institute for the COVID-19 Genomics UK (COG-UK) Consortium	Jacquelyn Wynn, Mairead Hyland, The Lighthouse Lab in Alderley Park and Alex Alderton, Roberto Amato, Sonia Goncalves, Ewan Harrison, David K. Jackson, Ian Johnston, Dominic Kwiatkowski, Cordelia Langford, John Sillitoe on behalf of the Wellcome Sanger Institute COVID-19 Surveillance Team
EPI_ISL_767134	Lighthouse Lab in Milton Keynes	Wellcome Sanger Institute for the COVID-19 Genomics UK (COG-UK) Consortium	The Lighthouse Lab in Milton Keynes and Alex Alderton, Roberto Amato, Sonia Goncalves, Ewan Harrison, David K. Jackson, Ian Johnston, Dominic Kwiatkowski, Cordelia Langford, John Sillitoe on behalf of the Wellcome Sanger Institute COVID-19 Surveillance Team
EPI_ISL_767136	Lighthouse Lab in Alderley Park	Wellcome Sanger Institute for the COVID-19 Genomics UK (COG-UK) Consortium	Jacquelyn Wynn, Mairead Hyland, The Lighthouse Lab in Alderley Park and Alex Alderton, Roberto Amato, Sonia Goncalves, Ewan Harrison, David K. Jackson, Ian Johnston, Dominic Kwiatkowski, Cordelia Langford, John Sillitoe on behalf of the Wellcome Sanger Institute COVID-19 Surveillance Team
EPI_ISL_767138	Lighthouse Lab in Cambridge	Wellcome Sanger Institute for the COVID-19 Genomics UK (COG-UK) Consortium	Rob Howes, The Lighthouse Lab in Cambridge and Alex Alderton, Roberto Amato, Sonia Goncalves, Ewan Harrison, David K. Jackson, Ian Johnston, Dominic Kwiatkowski, Cordelia Langford, John Sillitoe on behalf of the Wellcome Sanger Institute COVID-19 Surveillance Team
EPI_ISL_767141, EPI_ISL_767146, EPI_ISL_767147, EPI_ISL_767148, EPI_ISL_767150, EPI_ISL_767151, EPI_ISL_767152, EPI_ISL_767153, EPI_ISL_767156, EPI_ISL_767157, EPI_ISL_767159, EPI_ISL_767160, EPI_ISL_767161, EPI_ISL_767163, EPI_ISL_767164, EPI_ISL_767165, EPI_ISL_767166, EPI_ISL_767168, EPI_ISL_767169, EPI_ISL_767170, EPI_ISL_767173, EPI_ISL_767174, EPI_ISL_767175, EPI_ISL_767176, EPI_ISL_767177, EPI_ISL_767178, EPI_ISL_767179, EPI_ISL_767180, EPI_ISL_767181, EPI_ISL_767182, EPI_ISL_767183, EPI_ISL_767184, EPI_ISL_767185, EPI_ISL_767186, EPI_ISL_767187, EPI_ISL_767188, EPI_ISL_767189, EPI_ISL_767190, EPI_ISL_767191, EPI_ISL_767192, EPI_ISL_767193, EPI_ISL_767194, EPI_ISL_767195, EPI_ISL_767196, EPI_ISL_767197, EPI_ISL_767198, EPI_ISL_767199, EPI_ISL_767200, EPI_ISL_767201, EPI_ISL_767204, EPI_ISL_767205, EPI_ISL_767206, EPI_ISL_767207, EPI_ISL_767208, EPI_ISL_767209, EPI_ISL_767210, EPI_ISL_767212, EPI_ISL_767213, EPI_ISL_767216, EPI_ISL_767217, EPI_ISL_767218, EPI_ISL_767220, EPI_ISL_767221, EPI_ISL_767222, EPI_ISL_767223, EPI_ISL_767224, EPI_ISL_767226, EPI_ISL_767227, EPI_ISL_767228, EPI_ISL_767232, EPI_ISL_767233, EPI_ISL_767240, EPI_ISL_767241, EPI_ISL_767243, EPI_ISL_767244, EPI_ISL_767245, EPI_ISL_767247, EPI_ISL_767248, EPI_ISL_767249, EPI_ISL_767250, EPI_ISL_767253, EPI_ISL_767256, EPI_ISL_767257, EPI_ISL_767258, EPI_ISL_767259, EPI_ISL_767261, EPI_ISL_767262, EPI_ISL_767263, EPI_ISL_767264, EPI_ISL_767265, EPI_ISL_767268, EPI_ISL_767269, EPI_ISL_767270, EPI_ISL_767272, EPI_ISL_767273, EPI_ISL_767274, EPI_ISL_767276, EPI_ISL_767277, EPI_ISL_767278, EPI_ISL_767279, EPI_ISL_767281, EPI_ISL_767282, EPI_ISL_767284, EPI_ISL_767286, EPI_ISL_767289, EPI_ISL_767290, EPI_ISL_767292, EPI_ISL_767293, EPI_ISL_767295, EPI_ISL_767296, EPI_ISL_767298, EPI_ISL_767301, EPI_ISL_767302, EPI_ISL_767303, EPI_ISL_767305, EPI_ISL_767306, EPI_ISL_767307, EPI_ISL_767309, EPI_ISL_767313, EPI_ISL_767314, EPI_ISL_767315, EPI_ISL_767316, EPI_ISL_767317, EPI_ISL_767318			
see above	Lighthouse Lab in Alderley Park	Wellcome Sanger Institute for the COVID-19 Genomics UK (COG-UK) Consortium	Jacquelyn Wynn, Mairead Hyland, The Lighthouse Lab in Alderley Park and Alex Alderton, Roberto Amato, Sonia Goncalves, Ewan Harrison, David K. Jackson, Ian Johnston, Dominic Kwiatkowski, Cordelia Langford, John Sillitoe on behalf of the Wellcome Sanger Institute COVID-19 Surveillance Team
EPI_ISL_767419	URMC LABS	Wadsworth Center, New York State Department.of Health	Kirsten St. George, Daryl M. Lamson, Alexis Russel, Matthew Shudt, Melissa A Leisner, Jonathan Plitnick, Navjot Singh, John Kelly, Sara Griesemer, Erasmus Schneider, Erica Lasek-Nesselquist
EPI_ISL_767422, EPI_ISL_767450, EPI_ISL_767453, EPI_ISL_767454, EPI_ISL_767457, EPI_ISL_767461, EPI_ISL_767463, EPI_ISL_767464, EPI_ISL_767466, EPI_ISL_767468, EPI_ISL_767469, EPI_ISL_767470, EPI_ISL_767471, EPI_ISL_767472, EPI_ISL_767476, EPI_ISL_767478, EPI_ISL_767479, EPI_ISL_767480, EPI_ISL_767484, EPI_ISL_767485, EPI_ISL_767486, EPI_ISL_767487, EPI_ISL_767489, EPI_ISL_767490, EPI_ISL_767491, EPI_ISL_767492, EPI_ISL_767493, EPI_ISL_767494, EPI_ISL_767495, EPI_ISL_767496, EPI_ISL_767500, EPI_ISL_767501, EPI_ISL_767502, EPI_ISL_767503, EPI_ISL_767509, EPI_ISL_767511, EPI_ISL_767512, EPI_ISL_767514, EPI_ISL_767518, EPI_ISL_767519, EPI_ISL_767521, EPI_ISL_767524, EPI_ISL_767525, EPI_ISL_767527, EPI_ISL_767528, EPI_ISL_767529, EPI_ISL_767530, EPI_ISL_767531, EPI_ISL_767537			
see above	Wadsworth Center, New York State Department.of Health	Wadsworth Center, New York State Department.of Health	Kirsten St. George, Daryl M. Lamson, Alexis Russel, Matthew Shudt, Melissa A Leisner, Jonathan Plitnick, Navjot Singh, John Kelly, Sara Griesemer, Erasmus Schneider, Erica Lasek-Nesselquist
EPI_ISL_767539, EPI_ISL_767542, EPI_ISL_767545, EPI_ISL_767547, EPI_ISL_767549, EPI_ISL_767554, EPI_ISL_767556, EPI_ISL_767557, EPI_ISL_767560, EPI_ISL_767561, EPI_ISL_767562, EPI_ISL_767564, EPI_ISL_767566, EPI_ISL_767568, EPI_ISL_767569, EPI_ISL_767570, EPI_ISL_767571, EPI_ISL_767573, EPI_ISL_767575			
see above	URMC LABS	Wadsworth Center, New York State Department.of Health	Kirsten St. George, Daryl M. Lamson, Alexis Russel, Matthew Shudt, Melissa A Leisner, Jonathan Plitnick, Navjot Singh, John Kelly, Sara Griesemer, Erasmus Schneider, Erica Lasek-Nesselquist
EPI_ISL_767594	SARATOGA HOSPITAL LABORATORY	Wadsworth Center, New York State Department.of Health	Kirsten St. George, Daryl M. Lamson, Alexis Russel, Matthew Shudt, Melissa A Leisner, Jonathan Plitnick, Navjot Singh, John Kelly, Sara Griesemer, Erasmus Schneider, Erica Lasek-Nesselquist
EPI_ISL_767596	MEMORIAL SLOAN KETTERING CANCER CENTER	Wadsworth Center, New York State Department.of Health	Kirsten St. George, Daryl M. Lamson, Alexis Russel, Matthew Shudt, Melissa A Leisner, Jonathan Plitnick, Navjot Singh, John Kelly, Sara Griesemer, Erasmus Schneider, Erica Lasek-Nesselquist
EPI_ISL_767599, EPI_ISL_767603, EPI_ISL_767606, EPI_ISL_767607, EPI_ISL_767608, EPI_ISL_767612	SARATOGA HOSPITAL LABORATORY	Wadsworth Center, New York State Department.of Health	Kirsten St. George, Daryl M. Lamson, Alexis Russel, Matthew Shudt, Melissa A Leisner, Jonathan Plitnick, Navjot Singh, John Kelly, Sara Griesemer, Erasmus Schneider, Erica Lasek-Nesselquist
EPI_ISL_767632, EPI_ISL_767635, EPI_ISL_767637, EPI_ISL_767639, EPI_ISL_767640, EPI_ISL_767643, EPI_ISL_767644, EPI_ISL_767659, EPI_ISL_767660, EPI_ISL_767661, EPI_ISL_767662, EPI_ISL_767663, EPI_ISL_767664			
see above	MEMORIAL SLOAN KETTERING CANCER CENTER	Wadsworth Center, New York State Department.of Health	Kirsten St. George, Daryl M. Lamson, Alexis Russel, Matthew Shudt, Melissa A Leisner, Jonathan Plitnick, Navjot Singh, John Kelly, Sara Griesemer, Erasmus Schneider, Erica Lasek-Nesselquist
EPI_ISL_767676	BIO-REFERENCE LABORATORIES	Wadsworth Center, New York State Department.of Health	Kirsten St. George, Daryl M. Lamson, Alexis Russel, Matthew Shudt, Melissa A Leisner, Jonathan Plitnick, Navjot Singh, John Kelly, Sara Griesemer, Erasmus Schneider, Erica Lasek-Nesselquist
EPI_ISL_767682, EPI_ISL_767683, EPI_ISL_767684, EPI_ISL_767685, EPI_ISL_767686, EPI_ISL_767687, EPI_ISL_767688, EPI_ISL_767689, EPI_ISL_767692, EPI_ISL_767693, EPI_ISL_767697, EPI_ISL_767698, EPI_ISL_767700, EPI_ISL_767704, EPI_ISL_767706, EPI_ISL_767707			

see above	MEMORIAL SLOAN KETTERING CANCER CENTER	Wadsworth Center, New York State Department.of Health	Kirsten St. George, Daryl M. Lamson, Alexis Russel, Matthew Shudt, Melissa A Leisner, Jonathan Plitnick, Navjot Singh, John Kelly, Sara Griesemer, Erasmus Schneider, Erica Lasek-Nesselquist
EPI_ISL_768361, EPI_ISL_768362, EPI_ISL_768363, EPI_ISL_768365, EPI_ISL_768366, EPI_ISL_768367, EPI_ISL_768368, EPI_ISL_768369, EPI_ISL_768370, EPI_ISL_768371, EPI_ISL_768372, EPI_ISL_768373, EPI_ISL_768374, EPI_ISL_768375, EPI_ISL_768376, EPI_ISL_768377, EPI_ISL_768378, EPI_ISL_768379, EPI_ISL_768380, EPI_ISL_768381, EPI_ISL_768382, EPI_ISL_768383, EPI_ISL_768384, EPI_ISL_768385, EPI_ISL_768386, EPI_ISL_768387, EPI_ISL_768388, EPI_ISL_768389, EPI_ISL_768390, EPI_ISL_768391, EPI_ISL_768392, EPI_ISL_768393, EPI_ISL_768396, EPI_ISL_768397, EPI_ISL_768398, EPI_ISL_768399, EPI_ISL_768400, EPI_ISL_768401, EPI_ISL_768402, EPI_ISL_768403, EPI_ISL_768404, EPI_ISL_768406, EPI_ISL_768407, EPI_ISL_768408, EPI_ISL_768409, EPI_ISL_768410, EPI_ISL_768411, EPI_ISL_768412, EPI_ISL_768413, EPI_ISL_768414, EPI_ISL_768415, EPI_ISL_768416, EPI_ISL_768417, EPI_ISL_768418, EPI_ISL_768419, EPI_ISL_768420, EPI_ISL_768421, EPI_ISL_768422, EPI_ISL_768423, EPI_ISL_768424, EPI_ISL_768425, EPI_ISL_768426, EPI_ISL_768427, EPI_ISL_768428, EPI_ISL_768429, EPI_ISL_768430, EPI_ISL_768431, EPI_ISL_768432, EPI_ISL_768433, EPI_ISL_768434, EPI_ISL_768435, EPI_ISL_768436, EPI_ISL_768437, EPI_ISL_768438, EPI_ISL_768439, EPI_ISL_768440, EPI_ISL_768441, EPI_ISL_768442, EPI_ISL_768443, EPI_ISL_768444, EPI_ISL_768445, EPI_ISL_768446, EPI_ISL_768447, EPI_ISL_768448, EPI_ISL_768449, EPI_ISL_768450, EPI_ISL_768451, EPI_ISL_768452, EPI_ISL_768453, EPI_ISL_768454, EPI_ISL_768455, EPI_ISL_768456, EPI_ISL_768457, EPI_ISL_768458, EPI_ISL_768459, EPI_ISL_768460, EPI_ISL_768461, EPI_ISL_768462, EPI_ISL_768463, EPI_ISL_768464, EPI_ISL_768465, EPI_ISL_768466, EPI_ISL_768467, EPI_ISL_768468, EPI_ISL_768469, EPI_ISL_768470, EPI_ISL_768471, EPI_ISL_768472, EPI_ISL_768475, EPI_ISL_768476, EPI_ISL_768477, EPI_ISL_768478, EPI_ISL_768479, EPI_ISL_768480, EPI_ISL_768481, EPI_ISL_768482, EPI_ISL_768483, EPI_ISL_768484, EPI_ISL_768485, EPI_ISL_768486, EPI_ISL_768487, EPI_ISL_768488, EPI_ISL_768489, EPI_ISL_768490, EPI_ISL_768493, EPI_ISL_768494, EPI_ISL_768495, EPI_ISL_768496			
see above	LSUHS Emerging Viral Threat Laboratory	Microbial Genome Sequencing Center	Jeremy P. Kamil, Jennifer L. Carroll, Camille F. Abshire, Maarten Van Diest, Andrew D. Yurochko, Martin J. Sapp, Rona S. Scott, Christopher G. Kevil, Daniel J. Snyder, Vaughn S. Cooper, John A. Vanchiere
EPI_ISL_768497, EPI_ISL_768498, EPI_ISL_768499, EPI_ISL_768500, EPI_ISL_768501, EPI_ISL_768502, EPI_ISL_768503, EPI_ISL_768504, EPI_ISL_768505, EPI_ISL_768506, EPI_ISL_768507, EPI_ISL_768508			
see above	LSUHS Emerging Viral Threat Laboratory	Microbial Genome Sequencing Center	Jennifer L. Carroll, Jeremy P. Kamil, Camille F. Abshire, Maarten Van Diest, Andrew D. Yurochko, Martin J. Sapp, Rona S. Scott, Christopher G. Kevil, Daniel J. Snyder, Vaughn S. Cooper, John A. Vanchiere
EPI_ISL_768509, EPI_ISL_768510, EPI_ISL_768511, EPI_ISL_768512, EPI_ISL_768513, EPI_ISL_768514, EPI_ISL_768515, EPI_ISL_768516, EPI_ISL_768518, EPI_ISL_768519, EPI_ISL_768520			
see above	LSUHS Emerging Viral Threat Laboratory	Microbial Genome Sequencing Center	Maarten Van Diest, Jeremy P. Kamil, Jennifer L. Carroll, Camille F. Abshire, Andrew D. Yurochko, Martin J. Sapp, Rona S. Scott, Christopher G. Kevil, Daniel J. Snyder, Vaughn S. Cooper, John A. Vanchiere
EPI_ISL_768523	Singburi Hospital	National Institute of Health, Department of Medical Sciences, Ministry of Public Health, Thailand	Pilailuk Okada; Siripaporn Phuygun; Sittiporn Parnmen; Ratana Tacharoenmuang; Pakorn Piromtong; Natchaya Khiadsang; Thanutsapa Thanadachakul; Warawan Wongboot; sirikanda wimol; Sunthareeya Waicharoen;
EPI_ISL_768525	LSUHS Emerging Viral Threat Laboratory	Microbial Genome Sequencing Center	Jeremy P. Kamil, Jennifer L. Carroll, Camille F. Abshire, Maarten Van Diest, Andrew D. Yurochko, Martin J. Sapp, Rona S. Scott, Christopher G. Kevil, Daniel J. Snyder, Vaughn S. Cooper, John A. Vanchiere
EPI_ISL_768533, EPI_ISL_768535, EPI_ISL_768540, EPI_ISL_768541	Regional Medical Sciences Center 5 Samut Songkhram	National Institute of Health, Department of Medical Sciences, Ministry of Public Health, Thailand	Pilailuk Okada; Siripaporn Phuygun; Sittiporn Parnmen; Ratana Tacharoenmuang; Pakorn Piromtong; Natchaya Khiadsang; Thanutsapa Thanadachakul; Warawan Wongboot; sirikanda wimol; Sunthareeya Waicharoen;
EPI_ISL_768618	National Public Health Laboratory, National Centre for Infectious Diseases	National Public Health Laboratory, National Centre for Infectious Diseases	Tze Minn Mak, Sophie Octavia, Zhenyang Zhou, Lin Cui, Raymond Tzer Pin Lin
EPI_ISL_768633	Jena University Hospital, Institute for Infectious Diseases and Infection Control	Institute of infectious medicine & hospital hygiene, CaSe-Group	Spott, Riccardo; Marquet, Mike; Pletz, Matthias W.; Brandt, Christian
EPI_ISL_768802	Respiratory Virus Unit, National Infection Service, Public Health England	COVID-19 Genomics UK (COG-UK) Consortium	PHE Covid Sequencing Team
EPI_ISL_768840	Laboratoire Biolife	Laboratoire de Biotechnologie	Mouna Ouadghiri, Tarik Aanniz, Mohammed Walid Chemao Elfihri, Mohamed Chenaoui, Hanae Dakka, Afaf Alaoui, Otmane Touzani, Amina Benouda, Bouchra Belfquih, Lahcen belyamani, Saaid Amzazi and Azeddine Ibrahimi
EPI_ISL_768842, EPI_ISL_768844	Lighthouse Lab in Alderley Park	Wellcome Sanger Institute for the COVID-19 Genomics UK (COG-UK) Consortium	Jacquelyn Wynn, Mairead Hyland, The Lighthouse Lab in Alderley Park and Alex Alderton, Roberto Amato, Sonia Goncalves, Ewan Harrison, David K. Jackson, Ian Johnston, Dominic Kwiatkowski, Cordelia Langford, John Sillitoe on behalf of the Wellcome Sanger Institute COVID-19 Surveillance Team
EPI_ISL_768847, EPI_ISL_768848, EPI_ISL_768849, EPI_ISL_768850, EPI_ISL_768852, EPI_ISL_768853, EPI_ISL_768854, EPI_ISL_768856, EPI_ISL_768858, EPI_ISL_768860, EPI_ISL_768861, EPI_ISL_768862, EPI_ISL_768864, EPI_ISL_768865, EPI_ISL_768866, EPI_ISL_768867, EPI_ISL_768870, EPI_ISL_768872, EPI_ISL_768873, EPI_ISL_768874, EPI_ISL_768875, EPI_ISL_768876, EPI_ISL_768877, EPI_ISL_768878, EPI_ISL_768879, EPI_ISL_768881, EPI_ISL_768882, EPI_ISL_768883, EPI_ISL_768884, EPI_ISL_768886, EPI_ISL_768887, EPI_ISL_768889, EPI_ISL_768891, EPI_ISL_768892, EPI_ISL_768893, EPI_ISL_768894, EPI_ISL_768895, EPI_ISL_768896, EPI_ISL_768897			
see above	Lighthouse Lab in Milton Keynes	Wellcome Sanger Institute for the COVID-19 Genomics UK (COG-UK) Consortium	The Lighthouse Lab in Milton Keynes and Alex Alderton, Roberto Amato, Sonia Goncalves, Ewan Harrison, David K. Jackson, Ian Johnston, Dominic Kwiatkowski, Cordelia Langford, John Sillitoe on behalf of the Wellcome Sanger Institute COVID-19 Surveillance Team
EPI_ISL_768899, EPI_ISL_768900	Lighthouse Lab in Alderley Park	Wellcome Sanger Institute for the COVID-19 Genomics UK (COG-UK) Consortium	Jacquelyn Wynn, Mairead Hyland, The Lighthouse Lab in Alderley Park and Alex Alderton, Roberto Amato, Sonia Goncalves, Ewan Harrison, David K. Jackson, Ian Johnston, Dominic Kwiatkowski, Cordelia Langford, John Sillitoe on behalf of the Wellcome Sanger Institute COVID-19 Surveillance Team
EPI_ISL_768901, EPI_ISL_768902, EPI_ISL_768903, EPI_ISL_768905, EPI_ISL_768906, EPI_ISL_768909, EPI_ISL_768910, EPI_ISL_768912, EPI_ISL_768913, EPI_ISL_768915, EPI_ISL_768916, EPI_ISL_768917, EPI_ISL_768918, EPI_ISL_768919, EPI_ISL_768920, EPI_ISL_768921, EPI_ISL_768922, EPI_ISL_768923, EPI_ISL_768925, EPI_ISL_768926, EPI_ISL_768927, EPI_ISL_768928, EPI_ISL_768929, EPI_ISL_768930, EPI_ISL_768933, EPI_ISL_768935, EPI_ISL_768936, EPI_ISL_768937, EPI_ISL_768938, EPI_ISL_768939, EPI_ISL_768940, EPI_ISL_768942, EPI_ISL_768943, EPI_ISL_768944, EPI_ISL_768946, EPI_ISL_768947, EPI_ISL_768948, EPI_ISL_768949, EPI_ISL_768950, EPI_ISL_768952, EPI_ISL_768953, EPI_ISL_768955, EPI_ISL_768956, EPI_ISL_768958, EPI_ISL_768959, EPI_ISL_768961, EPI_ISL_768962, EPI_ISL_768963, EPI_ISL_768964, EPI_ISL_768966, EPI_ISL_768968, EPI_ISL_768969, EPI_ISL_768970, EPI_ISL_768971, EPI_ISL_768973, EPI_ISL_768974, EPI_ISL_768975, EPI_ISL_768976, EPI_ISL_768978, EPI_ISL_768979, EPI_ISL_768980, EPI_ISL_768981, EPI_ISL_768982, EPI_ISL_768983, EPI_ISL_768984, EPI_ISL_768986, EPI_ISL_768987, EPI_ISL_768989, EPI_ISL_768990, EPI_ISL_768991, EPI_ISL_768993, EPI_ISL_768994, EPI_ISL_768996, EPI_ISL_768997, EPI_ISL_768998, EPI_ISL_768999, EPI_ISL_769000, EPI_ISL_769002, EPI_ISL_769003, EPI_ISL_769005, EPI_ISL_769006, EPI_ISL_769008, EPI_ISL_769009, EPI_ISL_769010, EPI_ISL_769012, EPI_ISL_769013, EPI_ISL_769016, EPI_ISL_769017, EPI_ISL_769018, EPI_ISL_769019, EPI_ISL_769020, EPI_ISL_769023, EPI_ISL_769024, EPI_ISL_769025, EPI_ISL_769026, EPI_ISL_769027, EPI_ISL_769030, EPI_ISL_769032, EPI_ISL_769033, EPI_ISL_769035, EPI_ISL_769036, EPI_ISL_769037, EPI_ISL_769039, EPI_ISL_769040, EPI_ISL_769042, EPI_ISL_769043, EPI_ISL_769045, EPI_ISL_769046, EPI_ISL_769047, EPI_ISL_769051, EPI_ISL_769052, EPI_ISL_769056, EPI_ISL_769057, EPI_ISL_769058, EPI_ISL_769059, EPI_ISL_769060, EPI_ISL_769062, EPI_ISL_769063, EPI_ISL_769064, EPI_ISL_769066, EPI_ISL_769067, EPI_ISL_769069, EPI_ISL_769070, EPI_ISL_769072, EPI_ISL_769073, EPI_ISL_769074, EPI_ISL_769075, EPI_ISL_769076, EPI_ISL_769078, EPI_ISL_769079, EPI_ISL_769080, EPI_ISL_769082, EPI_ISL_769083, EPI_ISL_769084, EPI_ISL_769086, EPI_ISL_769087, EPI_ISL_769088, EPI_ISL_769089, EPI_ISL_769091, EPI_ISL_769092, EPI_ISL_769093, EPI_ISL_769094, EPI_ISL_769095, EPI_ISL_769096, EPI_ISL_769097, EPI_ISL_769101, EPI_ISL_769102, EPI_ISL_769103, EPI_ISL_769105, EPI_ISL_769108, EPI_ISL_769109, EPI_ISL_769111, EPI_ISL_769112, EPI_ISL_769113, EPI_ISL_769115, EPI_ISL_769118, EPI_ISL_769120, EPI_ISL_769121, EPI_ISL_769122, EPI_ISL_769123, EPI_ISL_769124, EPI_ISL_769125, EPI_ISL_769126, EPI_ISL_769127, EPI_ISL_769128, EPI_ISL_769129, EPI_ISL_769130, EPI_ISL_769131, EPI_ISL_769132, EPI_ISL_769134, EPI_ISL_769135, EPI_ISL_769136, EPI_ISL_769138, EPI_ISL_769139, EPI_ISL_769141, EPI_ISL_769145, EPI_ISL_769147, EPI_ISL_769148, EPI_ISL_769149, EPI_ISL_769150, EPI_ISL_769153, EPI_ISL_769155, EPI_ISL_769156, EPI_ISL_769157, EPI_ISL_769158, EPI_ISL_769159, EPI_ISL_769162, EPI_ISL_769163, EPI_ISL_769164, EPI_ISL_769165, EPI_ISL_769167, EPI_ISL_769168, EPI_ISL_769169, EPI_ISL_769171, EPI_ISL_769173, EPI_ISL_769174, EPI_ISL_769175, EPI_ISL_769176, EPI_ISL_769177, EPI_ISL_769179, EPI_ISL_769181, EPI_ISL_769182, EPI_ISL_769183, EPI_ISL_769184, EPI_ISL_769188, EPI_ISL_769189, EPI_ISL_769190, EPI_ISL_769192, EPI_ISL_769193, EPI_ISL_769194, EPI_ISL_769195, EPI_ISL_769197, EPI_ISL_769198, EPI_ISL_769199, EPI_ISL_769200, EPI_ISL_769201, EPI_ISL_769203, EPI_ISL_769204, EPI_ISL_769208, EPI_ISL_769209, EPI_ISL_769210, EPI_ISL_769212, EPI_ISL_769213, EPI_ISL_769214, EPI_ISL_769215, EPI_ISL_769216, EPI_ISL_769217, EPI_ISL_769218, EPI_ISL_769219, EPI_ISL_769221			
see above	Lighthouse Lab in Cambridge	Wellcome Sanger Institute for the COVID-19 Genomics UK (COG-UK) Consortium	Rob Howes, The Lighthouse Lab in Cambridge and Alex Alderton, Roberto Amato, Sonia Goncalves, Ewan Harrison, David K. Jackson, Ian Johnston, Dominic Kwiatkowski, Cordelia Langford, John Sillitoe on behalf of the Wellcome Sanger Institute COVID-19 Surveillance Team
EPI_ISL_769223, EPI_ISL_769224	Lighthouse Lab in Glasgow	Wellcome Sanger Institute for the COVID-19 Genomics UK (COG-UK) Consortium	Harper VanSteenhouse, Yumi Kasai, David Gray, Carol Clugston, Anna Dominiczak and Alex Alderton, Roberto Amato, Sonia Goncalves, Ewan Harrison, David K. Jackson, Ian Johnston, Dominic Kwiatkowski, Cordelia Langford, John Sillitoe on behalf of the Wellcome Sanger Institute COVID-19 Surveillance Team
EPI_ISL_769226, EPI_ISL_769228, EPI_ISL_769230, EPI_ISL_769231, EPI_ISL_769232	Lighthouse Lab in Milton Keynes	Wellcome Sanger Institute for the COVID-19 Genomics UK (COG-UK) Consortium	The Lighthouse Lab in Milton Keynes and Alex Alderton, Roberto Amato, Sonia Goncalves, Ewan Harrison, David K. Jackson, Ian Johnston, Dominic Kwiatkowski, Cordelia Langford, John Sillitoe on behalf of the Wellcome Sanger Institute COVID-19 Surveillance Team
EPI_ISL_769233	Lighthouse Lab in Glasgow	Wellcome Sanger Institute for the COVID-19 Genomics UK (COG-UK) Consortium	Harper VanSteenhouse, Yumi Kasai, David Gray, Carol Clugston, Anna Dominiczak and Alex Alderton, Roberto Amato, Sonia Goncalves, Ewan Harrison, David K. Jackson, Ian Johnston, Dominic Kwiatkowski, Cordelia Langford, John Sillitoe on behalf of the Wellcome Sanger Institute COVID-19 Surveillance Team
EPI_ISL_769235	Lighthouse Lab in Milton Keynes	Wellcome Sanger Institute for the COVID-19 Genomics UK (COG-UK) Consortium	The Lighthouse Lab in Milton Keynes and Alex Alderton, Roberto Amato, Sonia Goncalves, Ewan Harrison, David K. Jackson, Ian Johnston, Dominic Kwiatkowski, Cordelia Langford, John Sillitoe on behalf of the Wellcome Sanger Institute COVID-19 Surveillance Team
EPI_ISL_769236	Lighthouse Lab in Glasgow	Wellcome Sanger Institute for the COVID-19 Genomics UK (COG-UK) Consortium	Harper VanSteenhouse, Yumi Kasai, David Gray, Carol Clugston, Anna Dominiczak and Alex Alderton, Roberto Amato, Sonia Goncalves, Ewan Harrison, David K. Jackson, Ian Johnston, Dominic Kwiatkowski, Cordelia Langford, John Sillitoe on behalf of the Wellcome Sanger Institute COVID-19 Surveillance Team
EPI_ISL_769237, EPI_ISL_769239, EPI_ISL_769242	Lighthouse Lab in Milton Keynes	Wellcome Sanger Institute for the COVID-19 Genomics UK (COG-UK) Consortium	The Lighthouse Lab in Milton Keynes and Alex Alderton, Roberto Amato, Sonia Goncalves, Ewan Harrison, David K. Jackson, Ian Johnston, Dominic Kwiatkowski, Cordelia Langford, John Sillitoe on behalf of the Wellcome Sanger Institute COVID-19 Surveillance Team
EPI_ISL_769243, EPI_ISL_769244	Lighthouse Lab in Glasgow	Wellcome Sanger Institute for the COVID-19 Genomics UK (COG-UK) Consortium	Harper VanSteenhouse, Yumi Kasai, David Gray, Carol Clugston, Anna Dominiczak and Alex Alderton, Roberto Amato, Sonia Goncalves, Ewan Harrison, David K. Jackson, Ian Johnston, Dominic Kwiatkowski, Cordelia Langford, John Sillitoe on behalf of the Wellcome Sanger Institute COVID-19 Surveillance

			Team
EPI_ISL_769247, EPI_ISL_769248	Lighthouse Lab in Milton Keynes	Wellcome Sanger Institute for the COVID-19 Genomics UK (COG-UK) Consortium	The Lighthouse Lab in Milton Keynes and Alex Alderton, Roberto Amato, Sonia Goncalves, Ewan Harrison, David K. Jackson, Ian Johnston, Dominic Kwiatkowski, Cordelia Langford, John Sillitoe on behalf of the Wellcome Sanger Institute COVID-19 Surveillance Team
EPI_ISL_769249, EPI_ISL_769250	Lighthouse Lab in Glasgow	Wellcome Sanger Institute for the COVID-19 Genomics UK (COG-UK) Consortium	Harper VanSteenhouse, Yumi Kasai, David Gray, Carol Clugston, Anna Dominiczak and Alex Alderton, Roberto Amato, Sonia Goncalves, Ewan Harrison, David K. Jackson, Ian Johnston, Dominic Kwiatkowski, Cordelia Langford, John Sillitoe on behalf of the Wellcome Sanger Institute COVID-19 Surveillance Team
EPI_ISL_769251	Lighthouse Lab in Milton Keynes	Wellcome Sanger Institute for the COVID-19 Genomics UK (COG-UK) Consortium	The Lighthouse Lab in Milton Keynes and Alex Alderton, Roberto Amato, Sonia Goncalves, Ewan Harrison, David K. Jackson, Ian Johnston, Dominic Kwiatkowski, Cordelia Langford, John Sillitoe on behalf of the Wellcome Sanger Institute COVID-19 Surveillance Team
EPI_ISL_769252	Lighthouse Lab in Glasgow	Wellcome Sanger Institute for the COVID-19 Genomics UK (COG-UK) Consortium	Harper VanSteenhouse, Yumi Kasai, David Gray, Carol Clugston, Anna Dominiczak and Alex Alderton, Roberto Amato, Sonia Goncalves, Ewan Harrison, David K. Jackson, Ian Johnston, Dominic Kwiatkowski, Cordelia Langford, John Sillitoe on behalf of the Wellcome Sanger Institute COVID-19 Surveillance Team
EPI_ISL_769253	Lighthouse Lab in Milton Keynes	Wellcome Sanger Institute for the COVID-19 Genomics UK (COG-UK) Consortium	The Lighthouse Lab in Milton Keynes and Alex Alderton, Roberto Amato, Sonia Goncalves, Ewan Harrison, David K. Jackson, Ian Johnston, Dominic Kwiatkowski, Cordelia Langford, John Sillitoe on behalf of the Wellcome Sanger Institute COVID-19 Surveillance Team
EPI_ISL_769254, EPI_ISL_769255	Lighthouse Lab in Glasgow	Wellcome Sanger Institute for the COVID-19 Genomics UK (COG-UK) Consortium	Harper VanSteenhouse, Yumi Kasai, David Gray, Carol Clugston, Anna Dominiczak and Alex Alderton, Roberto Amato, Sonia Goncalves, Ewan Harrison, David K. Jackson, Ian Johnston, Dominic Kwiatkowski, Cordelia Langford, John Sillitoe on behalf of the Wellcome Sanger Institute COVID-19 Surveillance Team
EPI_ISL_769258	Lighthouse Lab in Milton Keynes	Wellcome Sanger Institute for the COVID-19 Genomics UK (COG-UK) Consortium	The Lighthouse Lab in Milton Keynes and Alex Alderton, Roberto Amato, Sonia Goncalves, Ewan Harrison, David K. Jackson, Ian Johnston, Dominic Kwiatkowski, Cordelia Langford, John Sillitoe on behalf of the Wellcome Sanger Institute COVID-19 Surveillance Team
EPI_ISL_769259, EPI_ISL_769260, EPI_ISL_769261	Lighthouse Lab in Glasgow	Wellcome Sanger Institute for the COVID-19 Genomics UK (COG-UK) Consortium	Harper VanSteenhouse, Yumi Kasai, David Gray, Carol Clugston, Anna Dominiczak and Alex Alderton, Roberto Amato, Sonia Goncalves, Ewan Harrison, David K. Jackson, Ian Johnston, Dominic Kwiatkowski, Cordelia Langford, John Sillitoe on behalf of the Wellcome Sanger Institute COVID-19 Surveillance Team
EPI_ISL_769265	Lighthouse Lab in Milton Keynes	Wellcome Sanger Institute for the COVID-19 Genomics UK (COG-UK) Consortium	The Lighthouse Lab in Milton Keynes and Alex Alderton, Roberto Amato, Sonia Goncalves, Ewan Harrison, David K. Jackson, Ian Johnston, Dominic Kwiatkowski, Cordelia Langford, John Sillitoe on behalf of the Wellcome Sanger Institute COVID-19 Surveillance Team
EPI_ISL_769267	Lighthouse Lab in Glasgow	Wellcome Sanger Institute for the COVID-19 Genomics UK (COG-UK) Consortium	Harper VanSteenhouse, Yumi Kasai, David Gray, Carol Clugston, Anna Dominiczak and Alex Alderton, Roberto Amato, Sonia Goncalves, Ewan Harrison, David K. Jackson, Ian Johnston, Dominic Kwiatkowski, Cordelia Langford, John Sillitoe on behalf of the Wellcome Sanger Institute COVID-19 Surveillance Team
EPI_ISL_769272, EPI_ISL_769277, EPI_ISL_769279	Lighthouse Lab in Milton Keynes	Wellcome Sanger Institute for the COVID-19 Genomics UK (COG-UK) Consortium	The Lighthouse Lab in Milton Keynes and Alex Alderton, Roberto Amato, Sonia Goncalves, Ewan Harrison, David K. Jackson, Ian Johnston, Dominic Kwiatkowski, Cordelia Langford, John Sillitoe on behalf of the Wellcome Sanger Institute COVID-19 Surveillance Team
EPI_ISL_769281, EPI_ISL_769282	Lighthouse Lab in Glasgow	Wellcome Sanger Institute for the COVID-19 Genomics UK (COG-UK) Consortium	Harper VanSteenhouse, Yumi Kasai, David Gray, Carol Clugston, Anna Dominiczak and Alex Alderton, Roberto Amato, Sonia Goncalves, Ewan Harrison, David K. Jackson, Ian Johnston, Dominic Kwiatkowski, Cordelia Langford, John Sillitoe on behalf of the Wellcome Sanger Institute COVID-19 Surveillance Team
EPI_ISL_769283	Lighthouse Lab in Milton Keynes	Wellcome Sanger Institute for the COVID-19 Genomics UK (COG-UK) Consortium	The Lighthouse Lab in Milton Keynes and Alex Alderton, Roberto Amato, Sonia Goncalves, Ewan Harrison, David K. Jackson, Ian Johnston, Dominic Kwiatkowski, Cordelia Langford, John Sillitoe on behalf of the Wellcome Sanger Institute COVID-19 Surveillance Team
EPI_ISL_769284	Lighthouse Lab in Glasgow	Wellcome Sanger Institute for the COVID-19 Genomics UK (COG-UK) Consortium	Harper VanSteenhouse, Yumi Kasai, David Gray, Carol Clugston, Anna Dominiczak and Alex Alderton, Roberto Amato, Sonia Goncalves, Ewan Harrison, David K. Jackson, Ian Johnston, Dominic Kwiatkowski, Cordelia Langford, John Sillitoe on behalf of the Wellcome Sanger Institute COVID-19 Surveillance Team
EPI_ISL_769285	Lighthouse Lab in Milton Keynes	Wellcome Sanger Institute for the COVID-19 Genomics UK (COG-UK) Consortium	The Lighthouse Lab in Milton Keynes and Alex Alderton, Roberto Amato, Sonia Goncalves, Ewan Harrison, David K. Jackson, Ian Johnston, Dominic Kwiatkowski, Cordelia Langford, John Sillitoe on behalf of the Wellcome Sanger Institute COVID-19 Surveillance Team
EPI_ISL_769286, EPI_ISL_769288, EPI_ISL_769289, EPI_ISL_769291	Lighthouse Lab in Glasgow	Wellcome Sanger Institute for the COVID-19 Genomics UK (COG-UK) Consortium	Harper VanSteenhouse, Yumi Kasai, David Gray, Carol Clugston, Anna Dominiczak and Alex Alderton, Roberto Amato, Sonia Goncalves, Ewan Harrison, David K. Jackson, Ian Johnston, Dominic Kwiatkowski, Cordelia Langford, John Sillitoe on behalf of the Wellcome Sanger Institute COVID-19 Surveillance Team
EPI_ISL_769293	Lighthouse Lab in Milton Keynes	Wellcome Sanger Institute for the COVID-19 Genomics UK (COG-UK) Consortium	The Lighthouse Lab in Milton Keynes and Alex Alderton, Roberto Amato, Sonia Goncalves, Ewan Harrison, David K. Jackson, Ian Johnston, Dominic Kwiatkowski, Cordelia Langford, John Sillitoe on behalf of the Wellcome Sanger Institute COVID-19 Surveillance Team
EPI_ISL_769294	Lighthouse Lab in Glasgow	Wellcome Sanger Institute for the COVID-19 Genomics UK (COG-UK) Consortium	Harper VanSteenhouse, Yumi Kasai, David Gray, Carol Clugston, Anna Dominiczak and Alex Alderton, Roberto Amato, Sonia Goncalves, Ewan Harrison, David K. Jackson, Ian Johnston, Dominic Kwiatkowski, Cordelia Langford, John Sillitoe on behalf of the Wellcome Sanger Institute COVID-19 Surveillance Team
EPI_ISL_769295, EPI_ISL_769296, EPI_ISL_769299, EPI_ISL_769300, EPI_ISL_769301, EPI_ISL_769302, EPI_ISL_769303, EPI_ISL_769304, EPI_ISL_769305	Lighthouse Lab in Milton Keynes	Wellcome Sanger Institute for the COVID-19 Genomics UK (COG-UK) Consortium	The Lighthouse Lab in Milton Keynes and Alex Alderton, Roberto Amato, Sonia Goncalves, Ewan Harrison, David K. Jackson, Ian Johnston, Dominic Kwiatkowski, Cordelia Langford, John Sillitoe on behalf of the Wellcome Sanger Institute COVID-19 Surveillance Team
EPI_ISL_769306	Lighthouse Lab in Glasgow	Wellcome Sanger Institute for the COVID-19 Genomics UK (COG-UK) Consortium	Harper VanSteenhouse, Yumi Kasai, David Gray, Carol Clugston, Anna Dominiczak and Alex Alderton, Roberto Amato, Sonia Goncalves, Ewan Harrison, David K. Jackson, Ian Johnston, Dominic Kwiatkowski, Cordelia Langford, John Sillitoe on behalf of the Wellcome Sanger Institute COVID-19 Surveillance Team
EPI_ISL_769307	Lighthouse Lab in Milton Keynes	Wellcome Sanger Institute for the COVID-19 Genomics UK (COG-UK) Consortium	The Lighthouse Lab in Milton Keynes and Alex Alderton, Roberto Amato, Sonia Goncalves, Ewan Harrison, David K. Jackson, Ian Johnston, Dominic Kwiatkowski, Cordelia Langford, John Sillitoe on behalf of the Wellcome Sanger Institute COVID-19 Surveillance Team
EPI_ISL_769308, EPI_ISL_769310	Lighthouse Lab in Glasgow	Wellcome Sanger Institute for the COVID-19 Genomics UK (COG-UK) Consortium	Harper VanSteenhouse, Yumi Kasai, David Gray, Carol Clugston, Anna Dominiczak and Alex Alderton, Roberto Amato, Sonia Goncalves, Ewan Harrison, David K. Jackson, Ian Johnston, Dominic Kwiatkowski, Cordelia Langford, John Sillitoe on behalf of the Wellcome Sanger Institute COVID-19 Surveillance Team
EPI_ISL_769311	Lighthouse Lab in Milton Keynes	Wellcome Sanger Institute for the COVID-19 Genomics UK (COG-UK) Consortium	The Lighthouse Lab in Milton Keynes and Alex Alderton, Roberto Amato, Sonia Goncalves, Ewan Harrison, David K. Jackson, Ian Johnston, Dominic Kwiatkowski, Cordelia Langford, John Sillitoe on behalf of the Wellcome Sanger Institute COVID-19 Surveillance Team
EPI_ISL_769312	Lighthouse Lab in Glasgow	Wellcome Sanger Institute for the COVID-19 Genomics UK (COG-UK) Consortium	Harper VanSteenhouse, Yumi Kasai, David Gray, Carol Clugston, Anna Dominiczak and Alex Alderton, Roberto Amato, Sonia Goncalves, Ewan Harrison, David K. Jackson, Ian Johnston, Dominic Kwiatkowski, Cordelia Langford, John Sillitoe on behalf of the Wellcome Sanger Institute COVID-19 Surveillance Team
EPI_ISL_769313	Lighthouse Lab in Milton Keynes	Wellcome Sanger Institute for the COVID-19 Genomics UK (COG-UK) Consortium	The Lighthouse Lab in Milton Keynes and Alex Alderton, Roberto Amato, Sonia Goncalves, Ewan Harrison, David K. Jackson, Ian Johnston, Dominic Kwiatkowski, Cordelia Langford, John Sillitoe on behalf of the Wellcome Sanger Institute COVID-19 Surveillance Team
EPI_ISL_769314	Lighthouse Lab in Glasgow	Wellcome Sanger Institute for the COVID-19 Genomics UK (COG-UK) Consortium	Harper VanSteenhouse, Yumi Kasai, David Gray, Carol Clugston, Anna Dominiczak and Alex Alderton, Roberto Amato, Sonia Goncalves, Ewan Harrison, David K. Jackson, Ian Johnston, Dominic Kwiatkowski, Cordelia Langford, John Sillitoe on behalf of the Wellcome Sanger Institute COVID-19 Surveillance Team
EPI_ISL_769315, EPI_ISL_769317, EPI_ISL_769318	Lighthouse Lab in Milton Keynes	Wellcome Sanger Institute for the COVID-19 Genomics UK (COG-UK) Consortium	The Lighthouse Lab in Milton Keynes and Alex Alderton, Roberto Amato, Sonia Goncalves, Ewan Harrison, David K. Jackson, Ian Johnston, Dominic Kwiatkowski, Cordelia Langford, John Sillitoe on behalf of the Wellcome Sanger Institute COVID-19 Surveillance Team
EPI_ISL_769319, EPI_ISL_769320, EPI_ISL_769321, EPI_ISL_769322,	Lighthouse Lab in Glasgow	Wellcome Sanger Institute for the COVID-19 Genomics UK (COG-UK) Consortium	Harper VanSteenhouse, Yumi Kasai, David Gray, Carol Clugston, Anna Dominiczak and Alex Alderton, Roberto Amato, Sonia Goncalves, Ewan Harrison, David K. Jackson, Ian Johnston, Dominic Kwiatkowski, Cordelia Langford, John Sillitoe on behalf of the Wellcome Sanger Institute COVID-19 Surveillance

EPI_ISL_769323			Team
EPI_ISL_769324, EPI_ISL_769326	Lighthouse Lab in Milton Keynes	Wellcome Sanger Institute for the COVID-19 Genomics UK (COG-UK) Consortium	The Lighthouse Lab in Milton Keynes and Alex Alderton, Roberto Amato, Sonia Goncalves, Ewan Harrison, David K. Jackson, Ian Johnston, Dominic Kwiatkowski, Cordelia Langford, John Sillitoe on behalf of the Wellcome Sanger Institute COVID-19 Surveillance Team
EPI_ISL_769327, EPI_ISL_769330	Lighthouse Lab in Glasgow	Wellcome Sanger Institute for the COVID-19 Genomics UK (COG-UK) Consortium	Harper VanSteenhouse, Yumi Kasai, David Gray, Carol Clugston, Anna Dominiczak and Alex Alderton, Roberto Amato, Sonia Goncalves, Ewan Harrison, David K. Jackson, Ian Johnston, Dominic Kwiatkowski, Cordelia Langford, John Sillitoe on behalf of the Wellcome Sanger Institute COVID-19 Surveillance Team
EPI_ISL_769331, EPI_ISL_769332	Lighthouse Lab in Milton Keynes	Wellcome Sanger Institute for the COVID-19 Genomics UK (COG-UK) Consortium	The Lighthouse Lab in Milton Keynes and Alex Alderton, Roberto Amato, Sonia Goncalves, Ewan Harrison, David K. Jackson, Ian Johnston, Dominic Kwiatkowski, Cordelia Langford, John Sillitoe on behalf of the Wellcome Sanger Institute COVID-19 Surveillance Team
EPI_ISL_769334	Lighthouse Lab in Glasgow	Wellcome Sanger Institute for the COVID-19 Genomics UK (COG-UK) Consortium	Harper VanSteenhouse, Yumi Kasai, David Gray, Carol Clugston, Anna Dominiczak and Alex Alderton, Roberto Amato, Sonia Goncalves, Ewan Harrison, David K. Jackson, Ian Johnston, Dominic Kwiatkowski, Cordelia Langford, John Sillitoe on behalf of the Wellcome Sanger Institute COVID-19 Surveillance Team
EPI_ISL_769337, EPI_ISL_769338, EPI_ISL_769339, EPI_ISL_769340	Lighthouse Lab in Milton Keynes	Wellcome Sanger Institute for the COVID-19 Genomics UK (COG-UK) Consortium	The Lighthouse Lab in Milton Keynes and Alex Alderton, Roberto Amato, Sonia Goncalves, Ewan Harrison, David K. Jackson, Ian Johnston, Dominic Kwiatkowski, Cordelia Langford, John Sillitoe on behalf of the Wellcome Sanger Institute COVID-19 Surveillance Team
EPI_ISL_769341	Lighthouse Lab in Glasgow	Wellcome Sanger Institute for the COVID-19 Genomics UK (COG-UK) Consortium	Harper VanSteenhouse, Yumi Kasai, David Gray, Carol Clugston, Anna Dominiczak and Alex Alderton, Roberto Amato, Sonia Goncalves, Ewan Harrison, David K. Jackson, Ian Johnston, Dominic Kwiatkowski, Cordelia Langford, John Sillitoe on behalf of the Wellcome Sanger Institute COVID-19 Surveillance Team
EPI_ISL_769342	Lighthouse Lab in Milton Keynes	Wellcome Sanger Institute for the COVID-19 Genomics UK (COG-UK) Consortium	The Lighthouse Lab in Milton Keynes and Alex Alderton, Roberto Amato, Sonia Goncalves, Ewan Harrison, David K. Jackson, Ian Johnston, Dominic Kwiatkowski, Cordelia Langford, John Sillitoe on behalf of the Wellcome Sanger Institute COVID-19 Surveillance Team
EPI_ISL_769343	Lighthouse Lab in Glasgow	Wellcome Sanger Institute for the COVID-19 Genomics UK (COG-UK) Consortium	Harper VanSteenhouse, Yumi Kasai, David Gray, Carol Clugston, Anna Dominiczak and Alex Alderton, Roberto Amato, Sonia Goncalves, Ewan Harrison, David K. Jackson, Ian Johnston, Dominic Kwiatkowski, Cordelia Langford, John Sillitoe on behalf of the Wellcome Sanger Institute COVID-19 Surveillance Team
EPI_ISL_769344	Lighthouse Lab in Milton Keynes	Wellcome Sanger Institute for the COVID-19 Genomics UK (COG-UK) Consortium	The Lighthouse Lab in Milton Keynes and Alex Alderton, Roberto Amato, Sonia Goncalves, Ewan Harrison, David K. Jackson, Ian Johnston, Dominic Kwiatkowski, Cordelia Langford, John Sillitoe on behalf of the Wellcome Sanger Institute COVID-19 Surveillance Team
EPI_ISL_769345	Lighthouse Lab in Glasgow	Wellcome Sanger Institute for the COVID-19 Genomics UK (COG-UK) Consortium	Harper VanSteenhouse, Yumi Kasai, David Gray, Carol Clugston, Anna Dominiczak and Alex Alderton, Roberto Amato, Sonia Goncalves, Ewan Harrison, David K. Jackson, Ian Johnston, Dominic Kwiatkowski, Cordelia Langford, John Sillitoe on behalf of the Wellcome Sanger Institute COVID-19 Surveillance Team
EPI_ISL_769346, EPI_ISL_769347	Lighthouse Lab in Milton Keynes	Wellcome Sanger Institute for the COVID-19 Genomics UK (COG-UK) Consortium	The Lighthouse Lab in Milton Keynes and Alex Alderton, Roberto Amato, Sonia Goncalves, Ewan Harrison, David K. Jackson, Ian Johnston, Dominic Kwiatkowski, Cordelia Langford, John Sillitoe on behalf of the Wellcome Sanger Institute COVID-19 Surveillance Team
EPI_ISL_769348, EPI_ISL_769349	Lighthouse Lab in Glasgow	Wellcome Sanger Institute for the COVID-19 Genomics UK (COG-UK) Consortium	Harper VanSteenhouse, Yumi Kasai, David Gray, Carol Clugston, Anna Dominiczak and Alex Alderton, Roberto Amato, Sonia Goncalves, Ewan Harrison, David K. Jackson, Ian Johnston, Dominic Kwiatkowski, Cordelia Langford, John Sillitoe on behalf of the Wellcome Sanger Institute COVID-19 Surveillance Team
EPI_ISL_769350, EPI_ISL_769351	Lighthouse Lab in Milton Keynes	Wellcome Sanger Institute for the COVID-19 Genomics UK (COG-UK) Consortium	The Lighthouse Lab in Milton Keynes and Alex Alderton, Roberto Amato, Sonia Goncalves, Ewan Harrison, David K. Jackson, Ian Johnston, Dominic Kwiatkowski, Cordelia Langford, John Sillitoe on behalf of the Wellcome Sanger Institute COVID-19 Surveillance Team
EPI_ISL_769352, EPI_ISL_769353	Lighthouse Lab in Glasgow	Wellcome Sanger Institute for the COVID-19 Genomics UK (COG-UK) Consortium	Harper VanSteenhouse, Yumi Kasai, David Gray, Carol Clugston, Anna Dominiczak and Alex Alderton, Roberto Amato, Sonia Goncalves, Ewan Harrison, David K. Jackson, Ian Johnston, Dominic Kwiatkowski, Cordelia Langford, John Sillitoe on behalf of the Wellcome Sanger Institute COVID-19 Surveillance Team
EPI_ISL_769354	Lighthouse Lab in Milton Keynes	Wellcome Sanger Institute for the COVID-19 Genomics UK (COG-UK) Consortium	The Lighthouse Lab in Milton Keynes and Alex Alderton, Roberto Amato, Sonia Goncalves, Ewan Harrison, David K. Jackson, Ian Johnston, Dominic Kwiatkowski, Cordelia Langford, John Sillitoe on behalf of the Wellcome Sanger Institute COVID-19 Surveillance Team
EPI_ISL_769355	Lighthouse Lab in Glasgow	Wellcome Sanger Institute for the COVID-19 Genomics UK (COG-UK) Consortium	Harper VanSteenhouse, Yumi Kasai, David Gray, Carol Clugston, Anna Dominiczak and Alex Alderton, Roberto Amato, Sonia Goncalves, Ewan Harrison, David K. Jackson, Ian Johnston, Dominic Kwiatkowski, Cordelia Langford, John Sillitoe on behalf of the Wellcome Sanger Institute COVID-19 Surveillance Team
EPI_ISL_769356, EPI_ISL_769359	Lighthouse Lab in Milton Keynes	Wellcome Sanger Institute for the COVID-19 Genomics UK (COG-UK) Consortium	The Lighthouse Lab in Milton Keynes and Alex Alderton, Roberto Amato, Sonia Goncalves, Ewan Harrison, David K. Jackson, Ian Johnston, Dominic Kwiatkowski, Cordelia Langford, John Sillitoe on behalf of the Wellcome Sanger Institute COVID-19 Surveillance Team
EPI_ISL_769360, EPI_ISL_769361	Lighthouse Lab in Glasgow	Wellcome Sanger Institute for the COVID-19 Genomics UK (COG-UK) Consortium	Harper VanSteenhouse, Yumi Kasai, David Gray, Carol Clugston, Anna Dominiczak and Alex Alderton, Roberto Amato, Sonia Goncalves, Ewan Harrison, David K. Jackson, Ian Johnston, Dominic Kwiatkowski, Cordelia Langford, John Sillitoe on behalf of the Wellcome Sanger Institute COVID-19 Surveillance Team
EPI_ISL_769362, EPI_ISL_769364	Lighthouse Lab in Milton Keynes	Wellcome Sanger Institute for the COVID-19 Genomics UK (COG-UK) Consortium	The Lighthouse Lab in Milton Keynes and Alex Alderton, Roberto Amato, Sonia Goncalves, Ewan Harrison, David K. Jackson, Ian Johnston, Dominic Kwiatkowski, Cordelia Langford, John Sillitoe on behalf of the Wellcome Sanger Institute COVID-19 Surveillance Team
EPI_ISL_769365, EPI_ISL_769367	Lighthouse Lab in Glasgow	Wellcome Sanger Institute for the COVID-19 Genomics UK (COG-UK) Consortium	Harper VanSteenhouse, Yumi Kasai, David Gray, Carol Clugston, Anna Dominiczak and Alex Alderton, Roberto Amato, Sonia Goncalves, Ewan Harrison, David K. Jackson, Ian Johnston, Dominic Kwiatkowski, Cordelia Langford, John Sillitoe on behalf of the Wellcome Sanger Institute COVID-19 Surveillance Team
EPI_ISL_769368, EPI_ISL_769369	Lighthouse Lab in Milton Keynes	Wellcome Sanger Institute for the COVID-19 Genomics UK (COG-UK) Consortium	The Lighthouse Lab in Milton Keynes and Alex Alderton, Roberto Amato, Sonia Goncalves, Ewan Harrison, David K. Jackson, Ian Johnston, Dominic Kwiatkowski, Cordelia Langford, John Sillitoe on behalf of the Wellcome Sanger Institute COVID-19 Surveillance Team
EPI_ISL_769370	Lighthouse Lab in Glasgow	Wellcome Sanger Institute for the COVID-19 Genomics UK (COG-UK) Consortium	Harper VanSteenhouse, Yumi Kasai, David Gray, Carol Clugston, Anna Dominiczak and Alex Alderton, Roberto Amato, Sonia Goncalves, Ewan Harrison, David K. Jackson, Ian Johnston, Dominic Kwiatkowski, Cordelia Langford, John Sillitoe on behalf of the Wellcome Sanger Institute COVID-19 Surveillance Team
EPI_ISL_769372, EPI_ISL_769373	Lighthouse Lab in Milton Keynes	Wellcome Sanger Institute for the COVID-19 Genomics UK (COG-UK) Consortium	The Lighthouse Lab in Milton Keynes and Alex Alderton, Roberto Amato, Sonia Goncalves, Ewan Harrison, David K. Jackson, Ian Johnston, Dominic Kwiatkowski, Cordelia Langford, John Sillitoe on behalf of the Wellcome Sanger Institute COVID-19 Surveillance Team
EPI_ISL_769374	Lighthouse Lab in Glasgow	Wellcome Sanger Institute for the COVID-19 Genomics UK (COG-UK) Consortium	Harper VanSteenhouse, Yumi Kasai, David Gray, Carol Clugston, Anna Dominiczak and Alex Alderton, Roberto Amato, Sonia Goncalves, Ewan Harrison, David K. Jackson, Ian Johnston, Dominic Kwiatkowski, Cordelia Langford, John Sillitoe on behalf of the Wellcome Sanger Institute COVID-19 Surveillance Team
EPI_ISL_769376, EPI_ISL_769378, EPI_ISL_769379	Lighthouse Lab in Milton Keynes	Wellcome Sanger Institute for the COVID-19 Genomics UK (COG-UK) Consortium	The Lighthouse Lab in Milton Keynes and Alex Alderton, Roberto Amato, Sonia Goncalves, Ewan Harrison, David K. Jackson, Ian Johnston, Dominic Kwiatkowski, Cordelia Langford, John Sillitoe on behalf of the Wellcome Sanger Institute COVID-19 Surveillance Team
EPI_ISL_769380, EPI_ISL_769381	Lighthouse Lab in Glasgow	Wellcome Sanger Institute for the COVID-19 Genomics UK (COG-UK) Consortium	Harper VanSteenhouse, Yumi Kasai, David Gray, Carol Clugston, Anna Dominiczak and Alex Alderton, Roberto Amato, Sonia Goncalves, Ewan Harrison, David K. Jackson, Ian Johnston, Dominic Kwiatkowski, Cordelia Langford, John Sillitoe on behalf of the Wellcome Sanger Institute COVID-19 Surveillance Team
EPI_ISL_769382	Lighthouse Lab in Milton Keynes	Wellcome Sanger Institute for the COVID-19 Genomics UK (COG-UK) Consortium	The Lighthouse Lab in Milton Keynes and Alex Alderton, Roberto Amato, Sonia Goncalves, Ewan Harrison, David K. Jackson, Ian Johnston, Dominic Kwiatkowski, Cordelia Langford, John Sillitoe on behalf of the Wellcome Sanger Institute COVID-19 Surveillance Team
EPI_ISL_769384, EPI_ISL_769385, EPI_ISL_769388, EPI_ISL_769389, EPI_ISL_769391, EPI_ISL_769392	Lighthouse Lab in Glasgow	Wellcome Sanger Institute for the COVID-19 Genomics UK (COG-UK) Consortium	Harper VanSteenhouse, Yumi Kasai, David Gray, Carol Clugston, Anna Dominiczak and Alex Alderton, Roberto Amato, Sonia Goncalves, Ewan Harrison, David K. Jackson, Ian Johnston, Dominic Kwiatkowski, Cordelia Langford, John Sillitoe on behalf of the Wellcome Sanger Institute COVID-19 Surveillance Team
EPI_ISL_769393	Lighthouse Lab in Milton Keynes	Wellcome Sanger Institute for the COVID-19 Genomics UK (COG-UK) Consortium	The Lighthouse Lab in Milton Keynes and Alex Alderton, Roberto Amato, Sonia Goncalves, Ewan Harrison, David K. Jackson, Ian Johnston, Dominic Kwiatkowski, Cordelia Langford, John Sillitoe on behalf of the Wellcome Sanger Institute COVID-19 Surveillance Team

EPI_ISL_769394	Lighthouse Lab in Glasgow	Wellcome Sanger Institute for the COVID-19 Genomics UK (COG-UK) Consortium	Harper VanSteenhouse, Yumi Kasai, David Gray, Carol Clugston, Anna Dominiczak and Alex Alderton, Roberto Amato, Sonia Goncalves, Ewan Harrison, David K. Jackson, Ian Johnston, Dominic Kwiatkowski, Cordelia Langford, John Sillitoe on behalf of the Wellcome Sanger Institute COVID-19 Surveillance Team
EPI_ISL_769395, EPI_ISL_769397, EPI_ISL_769398	Lighthouse Lab in Milton Keynes	Wellcome Sanger Institute for the COVID-19 Genomics UK (COG-UK) Consortium	The Lighthouse Lab in Milton Keynes and Alex Alderton, Roberto Amato, Sonia Goncalves, Ewan Harrison, David K. Jackson, Ian Johnston, Dominic Kwiatkowski, Cordelia Langford, John Sillitoe on behalf of the Wellcome Sanger Institute COVID-19 Surveillance Team
EPI_ISL_769399	Lighthouse Lab in Glasgow	Wellcome Sanger Institute for the COVID-19 Genomics UK (COG-UK) Consortium	Harper VanSteenhouse, Yumi Kasai, David Gray, Carol Clugston, Anna Dominiczak and Alex Alderton, Roberto Amato, Sonia Goncalves, Ewan Harrison, David K. Jackson, Ian Johnston, Dominic Kwiatkowski, Cordelia Langford, John Sillitoe on behalf of the Wellcome Sanger Institute COVID-19 Surveillance Team
EPI_ISL_769400	Lighthouse Lab in Milton Keynes	Wellcome Sanger Institute for the COVID-19 Genomics UK (COG-UK) Consortium	The Lighthouse Lab in Milton Keynes and Alex Alderton, Roberto Amato, Sonia Goncalves, Ewan Harrison, David K. Jackson, Ian Johnston, Dominic Kwiatkowski, Cordelia Langford, John Sillitoe on behalf of the Wellcome Sanger Institute COVID-19 Surveillance Team
EPI_ISL_769401, EPI_ISL_769402	Lighthouse Lab in Glasgow	Wellcome Sanger Institute for the COVID-19 Genomics UK (COG-UK) Consortium	Harper VanSteenhouse, Yumi Kasai, David Gray, Carol Clugston, Anna Dominiczak and Alex Alderton, Roberto Amato, Sonia Goncalves, Ewan Harrison, David K. Jackson, Ian Johnston, Dominic Kwiatkowski, Cordelia Langford, John Sillitoe on behalf of the Wellcome Sanger Institute COVID-19 Surveillance Team
EPI_ISL_769403	Lighthouse Lab in Milton Keynes	Wellcome Sanger Institute for the COVID-19 Genomics UK (COG-UK) Consortium	The Lighthouse Lab in Milton Keynes and Alex Alderton, Roberto Amato, Sonia Goncalves, Ewan Harrison, David K. Jackson, Ian Johnston, Dominic Kwiatkowski, Cordelia Langford, John Sillitoe on behalf of the Wellcome Sanger Institute COVID-19 Surveillance Team
EPI_ISL_769404, EPI_ISL_769405, EPI_ISL_769406	Lighthouse Lab in Glasgow	Wellcome Sanger Institute for the COVID-19 Genomics UK (COG-UK) Consortium	Harper VanSteenhouse, Yumi Kasai, David Gray, Carol Clugston, Anna Dominiczak and Alex Alderton, Roberto Amato, Sonia Goncalves, Ewan Harrison, David K. Jackson, Ian Johnston, Dominic Kwiatkowski, Cordelia Langford, John Sillitoe on behalf of the Wellcome Sanger Institute COVID-19 Surveillance Team
EPI_ISL_769407, EPI_ISL_769409, EPI_ISL_769410, EPI_ISL_769411	Lighthouse Lab in Milton Keynes	Wellcome Sanger Institute for the COVID-19 Genomics UK (COG-UK) Consortium	The Lighthouse Lab in Milton Keynes and Alex Alderton, Roberto Amato, Sonia Goncalves, Ewan Harrison, David K. Jackson, Ian Johnston, Dominic Kwiatkowski, Cordelia Langford, John Sillitoe on behalf of the Wellcome Sanger Institute COVID-19 Surveillance Team
EPI_ISL_769412, EPI_ISL_769413	Lighthouse Lab in Glasgow	Wellcome Sanger Institute for the COVID-19 Genomics UK (COG-UK) Consortium	Harper VanSteenhouse, Yumi Kasai, David Gray, Carol Clugston, Anna Dominiczak and Alex Alderton, Roberto Amato, Sonia Goncalves, Ewan Harrison, David K. Jackson, Ian Johnston, Dominic Kwiatkowski, Cordelia Langford, John Sillitoe on behalf of the Wellcome Sanger Institute COVID-19 Surveillance Team
EPI_ISL_769415	Lighthouse Lab in Milton Keynes	Wellcome Sanger Institute for the COVID-19 Genomics UK (COG-UK) Consortium	The Lighthouse Lab in Milton Keynes and Alex Alderton, Roberto Amato, Sonia Goncalves, Ewan Harrison, David K. Jackson, Ian Johnston, Dominic Kwiatkowski, Cordelia Langford, John Sillitoe on behalf of the Wellcome Sanger Institute COVID-19 Surveillance Team
EPI_ISL_769416, EPI_ISL_769419, EPI_ISL_769420, EPI_ISL_769421, EPI_ISL_769422	Lighthouse Lab in Glasgow	Wellcome Sanger Institute for the COVID-19 Genomics UK (COG-UK) Consortium	Harper VanSteenhouse, Yumi Kasai, David Gray, Carol Clugston, Anna Dominiczak and Alex Alderton, Roberto Amato, Sonia Goncalves, Ewan Harrison, David K. Jackson, Ian Johnston, Dominic Kwiatkowski, Cordelia Langford, John Sillitoe on behalf of the Wellcome Sanger Institute COVID-19 Surveillance Team
EPI_ISL_769423, EPI_ISL_769424, EPI_ISL_769426	Lighthouse Lab in Milton Keynes	Wellcome Sanger Institute for the COVID-19 Genomics UK (COG-UK) Consortium	The Lighthouse Lab in Milton Keynes and Alex Alderton, Roberto Amato, Sonia Goncalves, Ewan Harrison, David K. Jackson, Ian Johnston, Dominic Kwiatkowski, Cordelia Langford, John Sillitoe on behalf of the Wellcome Sanger Institute COVID-19 Surveillance Team
EPI_ISL_769427, EPI_ISL_769428	Lighthouse Lab in Glasgow	Wellcome Sanger Institute for the COVID-19 Genomics UK (COG-UK) Consortium	Harper VanSteenhouse, Yumi Kasai, David Gray, Carol Clugston, Anna Dominiczak and Alex Alderton, Roberto Amato, Sonia Goncalves, Ewan Harrison, David K. Jackson, Ian Johnston, Dominic Kwiatkowski, Cordelia Langford, John Sillitoe on behalf of the Wellcome Sanger Institute COVID-19 Surveillance Team
EPI_ISL_769429	Lighthouse Lab in Milton Keynes	Wellcome Sanger Institute for the COVID-19 Genomics UK (COG-UK) Consortium	The Lighthouse Lab in Milton Keynes and Alex Alderton, Roberto Amato, Sonia Goncalves, Ewan Harrison, David K. Jackson, Ian Johnston, Dominic Kwiatkowski, Cordelia Langford, John Sillitoe on behalf of the Wellcome Sanger Institute COVID-19 Surveillance Team
EPI_ISL_769430, EPI_ISL_769431	Lighthouse Lab in Glasgow	Wellcome Sanger Institute for the COVID-19 Genomics UK (COG-UK) Consortium	Harper VanSteenhouse, Yumi Kasai, David Gray, Carol Clugston, Anna Dominiczak and Alex Alderton, Roberto Amato, Sonia Goncalves, Ewan Harrison, David K. Jackson, Ian Johnston, Dominic Kwiatkowski, Cordelia Langford, John Sillitoe on behalf of the Wellcome Sanger Institute COVID-19 Surveillance Team
EPI_ISL_769432	Lighthouse Lab in Milton Keynes	Wellcome Sanger Institute for the COVID-19 Genomics UK (COG-UK) Consortium	The Lighthouse Lab in Milton Keynes and Alex Alderton, Roberto Amato, Sonia Goncalves, Ewan Harrison, David K. Jackson, Ian Johnston, Dominic Kwiatkowski, Cordelia Langford, John Sillitoe on behalf of the Wellcome Sanger Institute COVID-19 Surveillance Team
EPI_ISL_769434	Lighthouse Lab in Glasgow	Wellcome Sanger Institute for the COVID-19 Genomics UK (COG-UK) Consortium	Harper VanSteenhouse, Yumi Kasai, David Gray, Carol Clugston, Anna Dominiczak and Alex Alderton, Roberto Amato, Sonia Goncalves, Ewan Harrison, David K. Jackson, Ian Johnston, Dominic Kwiatkowski, Cordelia Langford, John Sillitoe on behalf of the Wellcome Sanger Institute COVID-19 Surveillance Team
EPI_ISL_769436, EPI_ISL_769437, EPI_ISL_769438	Lighthouse Lab in Milton Keynes	Wellcome Sanger Institute for the COVID-19 Genomics UK (COG-UK) Consortium	The Lighthouse Lab in Milton Keynes and Alex Alderton, Roberto Amato, Sonia Goncalves, Ewan Harrison, David K. Jackson, Ian Johnston, Dominic Kwiatkowski, Cordelia Langford, John Sillitoe on behalf of the Wellcome Sanger Institute COVID-19 Surveillance Team
EPI_ISL_769440, EPI_ISL_769441	Lighthouse Lab in Glasgow	Wellcome Sanger Institute for the COVID-19 Genomics UK (COG-UK) Consortium	Harper VanSteenhouse, Yumi Kasai, David Gray, Carol Clugston, Anna Dominiczak and Alex Alderton, Roberto Amato, Sonia Goncalves, Ewan Harrison, David K. Jackson, Ian Johnston, Dominic Kwiatkowski, Cordelia Langford, John Sillitoe on behalf of the Wellcome Sanger Institute COVID-19 Surveillance Team
EPI_ISL_769443, EPI_ISL_769444, EPI_ISL_769447, EPI_ISL_769448	Lighthouse Lab in Milton Keynes	Wellcome Sanger Institute for the COVID-19 Genomics UK (COG-UK) Consortium	The Lighthouse Lab in Milton Keynes and Alex Alderton, Roberto Amato, Sonia Goncalves, Ewan Harrison, David K. Jackson, Ian Johnston, Dominic Kwiatkowski, Cordelia Langford, John Sillitoe on behalf of the Wellcome Sanger Institute COVID-19 Surveillance Team
EPI_ISL_769449, EPI_ISL_769450, EPI_ISL_769452	Lighthouse Lab in Glasgow	Wellcome Sanger Institute for the COVID-19 Genomics UK (COG-UK) Consortium	Harper VanSteenhouse, Yumi Kasai, David Gray, Carol Clugston, Anna Dominiczak and Alex Alderton, Roberto Amato, Sonia Goncalves, Ewan Harrison, David K. Jackson, Ian Johnston, Dominic Kwiatkowski, Cordelia Langford, John Sillitoe on behalf of the Wellcome Sanger Institute COVID-19 Surveillance Team
EPI_ISL_769453	Lighthouse Lab in Milton Keynes	Wellcome Sanger Institute for the COVID-19 Genomics UK (COG-UK) Consortium	The Lighthouse Lab in Milton Keynes and Alex Alderton, Roberto Amato, Sonia Goncalves, Ewan Harrison, David K. Jackson, Ian Johnston, Dominic Kwiatkowski, Cordelia Langford, John Sillitoe on behalf of the Wellcome Sanger Institute COVID-19 Surveillance Team
EPI_ISL_769454	Lighthouse Lab in Glasgow	Wellcome Sanger Institute for the COVID-19 Genomics UK (COG-UK) Consortium	Harper VanSteenhouse, Yumi Kasai, David Gray, Carol Clugston, Anna Dominiczak and Alex Alderton, Roberto Amato, Sonia Goncalves, Ewan Harrison, David K. Jackson, Ian Johnston, Dominic Kwiatkowski, Cordelia Langford, John Sillitoe on behalf of the Wellcome Sanger Institute COVID-19 Surveillance Team
EPI_ISL_769455, EPI_ISL_769456	Lighthouse Lab in Milton Keynes	Wellcome Sanger Institute for the COVID-19 Genomics UK (COG-UK) Consortium	The Lighthouse Lab in Milton Keynes and Alex Alderton, Roberto Amato, Sonia Goncalves, Ewan Harrison, David K. Jackson, Ian Johnston, Dominic Kwiatkowski, Cordelia Langford, John Sillitoe on behalf of the Wellcome Sanger Institute COVID-19 Surveillance Team
EPI_ISL_769457, EPI_ISL_769458, EPI_ISL_769459, EPI_ISL_769460	Lighthouse Lab in Glasgow	Wellcome Sanger Institute for the COVID-19 Genomics UK (COG-UK) Consortium	Harper VanSteenhouse, Yumi Kasai, David Gray, Carol Clugston, Anna Dominiczak and Alex Alderton, Roberto Amato, Sonia Goncalves, Ewan Harrison, David K. Jackson, Ian Johnston, Dominic Kwiatkowski, Cordelia Langford, John Sillitoe on behalf of the Wellcome Sanger Institute COVID-19 Surveillance Team
EPI_ISL_769461, EPI_ISL_769464	Lighthouse Lab in Milton Keynes	Wellcome Sanger Institute for the COVID-19 Genomics UK (COG-UK) Consortium	The Lighthouse Lab in Milton Keynes and Alex Alderton, Roberto Amato, Sonia Goncalves, Ewan Harrison, David K. Jackson, Ian Johnston, Dominic Kwiatkowski, Cordelia Langford, John Sillitoe on behalf of the Wellcome Sanger Institute COVID-19 Surveillance Team
EPI_ISL_769466	Lighthouse Lab in Glasgow	Wellcome Sanger Institute for the COVID-19 Genomics UK (COG-UK) Consortium	Harper VanSteenhouse, Yumi Kasai, David Gray, Carol Clugston, Anna Dominiczak and Alex Alderton, Roberto Amato, Sonia Goncalves, Ewan Harrison, David K. Jackson, Ian Johnston, Dominic Kwiatkowski, Cordelia Langford, John Sillitoe on behalf of the Wellcome Sanger Institute COVID-19 Surveillance Team
EPI_ISL_769467	Lighthouse Lab in Milton Keynes	Wellcome Sanger Institute for the COVID-19 Genomics UK (COG-UK) Consortium	The Lighthouse Lab in Milton Keynes and Alex Alderton, Roberto Amato, Sonia Goncalves, Ewan Harrison, David K. Jackson, Ian Johnston, Dominic Kwiatkowski, Cordelia Langford, John Sillitoe on behalf of the Wellcome Sanger Institute COVID-19 Surveillance Team
EPI_ISL_769470, EPI_ISL_769471	Lighthouse Lab in Glasgow	Wellcome Sanger Institute for the COVID-19 Genomics UK (COG-UK) Consortium	Harper VanSteenhouse, Yumi Kasai, David Gray, Carol Clugston, Anna Dominiczak and Alex Alderton, Roberto Amato, Sonia Goncalves, Ewan Harrison, David K. Jackson, Ian Johnston, Dominic Kwiatkowski, Cordelia Langford, John Sillitoe on behalf of the Wellcome Sanger Institute COVID-19 Surveillance Team

EPI_ISL_769472	Lighthouse Lab in Milton Keynes	Wellcome Sanger Institute for the COVID-19 Genomics UK (COG-UK) Consortium	The Lighthouse Lab in Milton Keynes and Alex Alderton, Roberto Amato, Sonia Goncalves, Ewan Harrison, David K. Jackson, Ian Johnston, Dominic Kwiatkowski, Cordelia Langford, John Sillitoe on behalf of the Wellcome Sanger Institute COVID-19 Surveillance Team
EPI_ISL_769474, EPI_ISL_769475, EPI_ISL_769476	Lighthouse Lab in Glasgow	Wellcome Sanger Institute for the COVID-19 Genomics UK (COG-UK) Consortium	Harper VanSteenhouse, Yumi Kasai, David Gray, Carol Clugston, Anna Dominiczak and Alex Alderton, Roberto Amato, Sonia Goncalves, Ewan Harrison, David K. Jackson, Ian Johnston, Dominic Kwiatkowski, Cordelia Langford, John Sillitoe on behalf of the Wellcome Sanger Institute COVID-19 Surveillance Team
EPI_ISL_769477, EPI_ISL_769478, EPI_ISL_769479, EPI_ISL_769480	Lighthouse Lab in Milton Keynes	Wellcome Sanger Institute for the COVID-19 Genomics UK (COG-UK) Consortium	The Lighthouse Lab in Milton Keynes and Alex Alderton, Roberto Amato, Sonia Goncalves, Ewan Harrison, David K. Jackson, Ian Johnston, Dominic Kwiatkowski, Cordelia Langford, John Sillitoe on behalf of the Wellcome Sanger Institute COVID-19 Surveillance Team
EPI_ISL_769481, EPI_ISL_769482	Lighthouse Lab in Glasgow	Wellcome Sanger Institute for the COVID-19 Genomics UK (COG-UK) Consortium	Harper VanSteenhouse, Yumi Kasai, David Gray, Carol Clugston, Anna Dominiczak and Alex Alderton, Roberto Amato, Sonia Goncalves, Ewan Harrison, David K. Jackson, Ian Johnston, Dominic Kwiatkowski, Cordelia Langford, John Sillitoe on behalf of the Wellcome Sanger Institute COVID-19 Surveillance Team
EPI_ISL_769484, EPI_ISL_769486, EPI_ISL_769490, EPI_ISL_769491, EPI_ISL_769492, EPI_ISL_769493, EPI_ISL_769495	Lighthouse Lab in Milton Keynes	Wellcome Sanger Institute for the COVID-19 Genomics UK (COG-UK) Consortium	The Lighthouse Lab in Milton Keynes and Alex Alderton, Roberto Amato, Sonia Goncalves, Ewan Harrison, David K. Jackson, Ian Johnston, Dominic Kwiatkowski, Cordelia Langford, John Sillitoe on behalf of the Wellcome Sanger Institute COVID-19 Surveillance Team
EPI_ISL_769497, EPI_ISL_769499, EPI_ISL_769501, EPI_ISL_769503, EPI_ISL_769506	Lighthouse Lab in Glasgow	Wellcome Sanger Institute for the COVID-19 Genomics UK (COG-UK) Consortium	Harper VanSteenhouse, Yumi Kasai, David Gray, Carol Clugston, Anna Dominiczak and Alex Alderton, Roberto Amato, Sonia Goncalves, Ewan Harrison, David K. Jackson, Ian Johnston, Dominic Kwiatkowski, Cordelia Langford, John Sillitoe on behalf of the Wellcome Sanger Institute COVID-19 Surveillance Team
EPI_ISL_769508, EPI_ISL_769509, EPI_ISL_769510	Lighthouse Lab in Milton Keynes	Wellcome Sanger Institute for the COVID-19 Genomics UK (COG-UK) Consortium	The Lighthouse Lab in Milton Keynes and Alex Alderton, Roberto Amato, Sonia Goncalves, Ewan Harrison, David K. Jackson, Ian Johnston, Dominic Kwiatkowski, Cordelia Langford, John Sillitoe on behalf of the Wellcome Sanger Institute COVID-19 Surveillance Team
EPI_ISL_769511, EPI_ISL_769513	Lighthouse Lab in Glasgow	Wellcome Sanger Institute for the COVID-19 Genomics UK (COG-UK) Consortium	Harper VanSteenhouse, Yumi Kasai, David Gray, Carol Clugston, Anna Dominiczak and Alex Alderton, Roberto Amato, Sonia Goncalves, Ewan Harrison, David K. Jackson, Ian Johnston, Dominic Kwiatkowski, Cordelia Langford, John Sillitoe on behalf of the Wellcome Sanger Institute COVID-19 Surveillance Team
EPI_ISL_769514	Lighthouse Lab in Milton Keynes	Wellcome Sanger Institute for the COVID-19 Genomics UK (COG-UK) Consortium	The Lighthouse Lab in Milton Keynes and Alex Alderton, Roberto Amato, Sonia Goncalves, Ewan Harrison, David K. Jackson, Ian Johnston, Dominic Kwiatkowski, Cordelia Langford, John Sillitoe on behalf of the Wellcome Sanger Institute COVID-19 Surveillance Team
EPI_ISL_769518, EPI_ISL_769519	Lighthouse Lab in Glasgow	Wellcome Sanger Institute for the COVID-19 Genomics UK (COG-UK) Consortium	Harper VanSteenhouse, Yumi Kasai, David Gray, Carol Clugston, Anna Dominiczak and Alex Alderton, Roberto Amato, Sonia Goncalves, Ewan Harrison, David K. Jackson, Ian Johnston, Dominic Kwiatkowski, Cordelia Langford, John Sillitoe on behalf of the Wellcome Sanger Institute COVID-19 Surveillance Team
EPI_ISL_769521	Lighthouse Lab in Milton Keynes	Wellcome Sanger Institute for the COVID-19 Genomics UK (COG-UK) Consortium	The Lighthouse Lab in Milton Keynes and Alex Alderton, Roberto Amato, Sonia Goncalves, Ewan Harrison, David K. Jackson, Ian Johnston, Dominic Kwiatkowski, Cordelia Langford, John Sillitoe on behalf of the Wellcome Sanger Institute COVID-19 Surveillance Team
EPI_ISL_769522, EPI_ISL_769524, EPI_ISL_769525	Lighthouse Lab in Glasgow	Wellcome Sanger Institute for the COVID-19 Genomics UK (COG-UK) Consortium	Harper VanSteenhouse, Yumi Kasai, David Gray, Carol Clugston, Anna Dominiczak and Alex Alderton, Roberto Amato, Sonia Goncalves, Ewan Harrison, David K. Jackson, Ian Johnston, Dominic Kwiatkowski, Cordelia Langford, John Sillitoe on behalf of the Wellcome Sanger Institute COVID-19 Surveillance Team
EPI_ISL_769527	Lighthouse Lab in Milton Keynes	Wellcome Sanger Institute for the COVID-19 Genomics UK (COG-UK) Consortium	The Lighthouse Lab in Milton Keynes and Alex Alderton, Roberto Amato, Sonia Goncalves, Ewan Harrison, David K. Jackson, Ian Johnston, Dominic Kwiatkowski, Cordelia Langford, John Sillitoe on behalf of the Wellcome Sanger Institute COVID-19 Surveillance Team
EPI_ISL_769528	Lighthouse Lab in Glasgow	Wellcome Sanger Institute for the COVID-19 Genomics UK (COG-UK) Consortium	Harper VanSteenhouse, Yumi Kasai, David Gray, Carol Clugston, Anna Dominiczak and Alex Alderton, Roberto Amato, Sonia Goncalves, Ewan Harrison, David K. Jackson, Ian Johnston, Dominic Kwiatkowski, Cordelia Langford, John Sillitoe on behalf of the Wellcome Sanger Institute COVID-19 Surveillance Team
EPI_ISL_769529	Lighthouse Lab in Milton Keynes	Wellcome Sanger Institute for the COVID-19 Genomics UK (COG-UK) Consortium	The Lighthouse Lab in Milton Keynes and Alex Alderton, Roberto Amato, Sonia Goncalves, Ewan Harrison, David K. Jackson, Ian Johnston, Dominic Kwiatkowski, Cordelia Langford, John Sillitoe on behalf of the Wellcome Sanger Institute COVID-19 Surveillance Team
EPI_ISL_769530, EPI_ISL_769533, EPI_ISL_769535	Lighthouse Lab in Glasgow	Wellcome Sanger Institute for the COVID-19 Genomics UK (COG-UK) Consortium	Harper VanSteenhouse, Yumi Kasai, David Gray, Carol Clugston, Anna Dominiczak and Alex Alderton, Roberto Amato, Sonia Goncalves, Ewan Harrison, David K. Jackson, Ian Johnston, Dominic Kwiatkowski, Cordelia Langford, John Sillitoe on behalf of the Wellcome Sanger Institute COVID-19 Surveillance Team
EPI_ISL_769536, EPI_ISL_769537	Lighthouse Lab in Milton Keynes	Wellcome Sanger Institute for the COVID-19 Genomics UK (COG-UK) Consortium	The Lighthouse Lab in Milton Keynes and Alex Alderton, Roberto Amato, Sonia Goncalves, Ewan Harrison, David K. Jackson, Ian Johnston, Dominic Kwiatkowski, Cordelia Langford, John Sillitoe on behalf of the Wellcome Sanger Institute COVID-19 Surveillance Team
EPI_ISL_769540, EPI_ISL_769543	Lighthouse Lab in Glasgow	Wellcome Sanger Institute for the COVID-19 Genomics UK (COG-UK) Consortium	Harper VanSteenhouse, Yumi Kasai, David Gray, Carol Clugston, Anna Dominiczak and Alex Alderton, Roberto Amato, Sonia Goncalves, Ewan Harrison, David K. Jackson, Ian Johnston, Dominic Kwiatkowski, Cordelia Langford, John Sillitoe on behalf of the Wellcome Sanger Institute COVID-19 Surveillance Team
EPI_ISL_769544, EPI_ISL_769545, EPI_ISL_769546, EPI_ISL_769547, EPI_ISL_769548, EPI_ISL_769549	Lighthouse Lab in Milton Keynes	Wellcome Sanger Institute for the COVID-19 Genomics UK (COG-UK) Consortium	The Lighthouse Lab in Milton Keynes and Alex Alderton, Roberto Amato, Sonia Goncalves, Ewan Harrison, David K. Jackson, Ian Johnston, Dominic Kwiatkowski, Cordelia Langford, John Sillitoe on behalf of the Wellcome Sanger Institute COVID-19 Surveillance Team
EPI_ISL_769551	Lighthouse Lab in Glasgow	Wellcome Sanger Institute for the COVID-19 Genomics UK (COG-UK) Consortium	Harper VanSteenhouse, Yumi Kasai, David Gray, Carol Clugston, Anna Dominiczak and Alex Alderton, Roberto Amato, Sonia Goncalves, Ewan Harrison, David K. Jackson, Ian Johnston, Dominic Kwiatkowski, Cordelia Langford, John Sillitoe on behalf of the Wellcome Sanger Institute COVID-19 Surveillance Team
EPI_ISL_769552, EPI_ISL_769554, EPI_ISL_769555	Lighthouse Lab in Milton Keynes	Wellcome Sanger Institute for the COVID-19 Genomics UK (COG-UK) Consortium	The Lighthouse Lab in Milton Keynes and Alex Alderton, Roberto Amato, Sonia Goncalves, Ewan Harrison, David K. Jackson, Ian Johnston, Dominic Kwiatkowski, Cordelia Langford, John Sillitoe on behalf of the Wellcome Sanger Institute COVID-19 Surveillance Team
EPI_ISL_769556	Lighthouse Lab in Glasgow	Wellcome Sanger Institute for the COVID-19 Genomics UK (COG-UK) Consortium	Harper VanSteenhouse, Yumi Kasai, David Gray, Carol Clugston, Anna Dominiczak and Alex Alderton, Roberto Amato, Sonia Goncalves, Ewan Harrison, David K. Jackson, Ian Johnston, Dominic Kwiatkowski, Cordelia Langford, John Sillitoe on behalf of the Wellcome Sanger Institute COVID-19 Surveillance Team

EPI_ISL_769559, EPI_ISL_769561, EPI_ISL_769562, EPI_ISL_769564, EPI_ISL_769566, EPI_ISL_769569, EPI_ISL_769570, EPI_ISL_769571, EPI_ISL_769572, EPI_ISL_769573, EPI_ISL_769574, EPI_ISL_769575

see above	Lighthouse Lab in Milton Keynes	Wellcome Sanger Institute for the COVID-19 Genomics UK (COG-UK) Consortium	The Lighthouse Lab in Milton Keynes and Alex Alderton, Roberto Amato, Sonia Goncalves, Ewan Harrison, David K. Jackson, Ian Johnston, Dominic Kwiatkowski, Cordelia Langford, John Sillitoe on behalf of the Wellcome Sanger Institute COVID-19 Surveillance Team
EPI_ISL_769577	Lighthouse Lab in Glasgow	Wellcome Sanger Institute for the COVID-19 Genomics UK (COG-UK) Consortium	Harper VanSteenhouse, Yumi Kasai, David Gray, Carol Clugston, Anna Dominiczak and Alex Alderton, Roberto Amato, Sonia Goncalves, Ewan Harrison, David K. Jackson, Ian Johnston, Dominic Kwiatkowski, Cordelia Langford, John Sillitoe on behalf of the Wellcome Sanger Institute COVID-19 Surveillance Team
EPI_ISL_769578, EPI_ISL_769579, EPI_ISL_769580, EPI_ISL_769582	Lighthouse Lab in Milton Keynes	Wellcome Sanger Institute for the COVID-19 Genomics UK (COG-UK) Consortium	The Lighthouse Lab in Milton Keynes and Alex Alderton, Roberto Amato, Sonia Goncalves, Ewan Harrison, David K. Jackson, Ian Johnston, Dominic Kwiatkowski, Cordelia Langford, John Sillitoe on behalf of the Wellcome Sanger Institute COVID-19 Surveillance Team
EPI_ISL_769583	Lighthouse Lab in Glasgow	Wellcome Sanger Institute for the COVID-19 Genomics UK (COG-UK) Consortium	Harper VanSteenhouse, Yumi Kasai, David Gray, Carol Clugston, Anna Dominiczak and Alex Alderton, Roberto Amato, Sonia Goncalves, Ewan Harrison, David K. Jackson, Ian Johnston, Dominic Kwiatkowski, Cordelia Langford, John Sillitoe on behalf of the Wellcome Sanger Institute COVID-19 Surveillance Team
EPI_ISL_769585, EPI_ISL_769586, EPI_ISL_769588	Lighthouse Lab in Milton Keynes	Wellcome Sanger Institute for the COVID-19 Genomics UK (COG-UK) Consortium	The Lighthouse Lab in Milton Keynes and Alex Alderton, Roberto Amato, Sonia Goncalves, Ewan Harrison, David K. Jackson, Ian Johnston, Dominic Kwiatkowski, Cordelia Langford, John Sillitoe on behalf of the Wellcome Sanger Institute COVID-19 Surveillance Team
EPI_ISL_769589	Lighthouse Lab in Glasgow	Wellcome Sanger Institute for the COVID-19 Genomics UK (COG-UK) Consortium	Harper VanSteenhouse, Yumi Kasai, David Gray, Carol Clugston, Anna Dominiczak and Alex Alderton, Roberto Amato, Sonia Goncalves, Ewan Harrison, David K. Jackson, Ian Johnston, Dominic Kwiatkowski, Cordelia Langford, John Sillitoe on behalf of the Wellcome Sanger Institute COVID-19 Surveillance

			Team
EPI_ISL_769590, EPI_ISL_769592	Lighthouse Lab in Milton Keynes	Wellcome Sanger Institute for the COVID-19 Genomics UK (COG-UK) Consortium	The Lighthouse Lab in Milton Keynes and Alex Alderton, Roberto Amato, Sonia Goncalves, Ewan Harrison, David K. Jackson, Ian Johnston, Dominic Kwiatkowski, Cordelia Langford, John Sillitoe on behalf of the Wellcome Sanger Institute COVID-19 Surveillance Team
EPI_ISL_769594	Lighthouse Lab in Glasgow	Wellcome Sanger Institute for the COVID-19 Genomics UK (COG-UK) Consortium	Harper VanSteenhouse, Yumi Kasai, David Gray, Carol Clugston, Anna Dominiczak and Alex Alderton, Roberto Amato, Sonia Goncalves, Ewan Harrison, David K. Jackson, Ian Johnston, Dominic Kwiatkowski, Cordelia Langford, John Sillitoe on behalf of the Wellcome Sanger Institute COVID-19 Surveillance Team
EPI_ISL_769595, EPI_ISL_769596, EPI_ISL_769598, EPI_ISL_769599, EPI_ISL_769601, EPI_ISL_769602, EPI_ISL_769603, EPI_ISL_769605	Lighthouse Lab in Milton Keynes	Wellcome Sanger Institute for the COVID-19 Genomics UK (COG-UK) Consortium	The Lighthouse Lab in Milton Keynes and Alex Alderton, Roberto Amato, Sonia Goncalves, Ewan Harrison, David K. Jackson, Ian Johnston, Dominic Kwiatkowski, Cordelia Langford, John Sillitoe on behalf of the Wellcome Sanger Institute COVID-19 Surveillance Team
EPI_ISL_769606, EPI_ISL_769607	Lighthouse Lab in Glasgow	Wellcome Sanger Institute for the COVID-19 Genomics UK (COG-UK) Consortium	Harper VanSteenhouse, Yumi Kasai, David Gray, Carol Clugston, Anna Dominiczak and Alex Alderton, Roberto Amato, Sonia Goncalves, Ewan Harrison, David K. Jackson, Ian Johnston, Dominic Kwiatkowski, Cordelia Langford, John Sillitoe on behalf of the Wellcome Sanger Institute COVID-19 Surveillance Team
EPI_ISL_769608	Lighthouse Lab in Milton Keynes	Wellcome Sanger Institute for the COVID-19 Genomics UK (COG-UK) Consortium	The Lighthouse Lab in Milton Keynes and Alex Alderton, Roberto Amato, Sonia Goncalves, Ewan Harrison, David K. Jackson, Ian Johnston, Dominic Kwiatkowski, Cordelia Langford, John Sillitoe on behalf of the Wellcome Sanger Institute COVID-19 Surveillance Team
EPI_ISL_769609, EPI_ISL_769611, EPI_ISL_769612, EPI_ISL_769613, EPI_ISL_769614	Lighthouse Lab in Glasgow	Wellcome Sanger Institute for the COVID-19 Genomics UK (COG-UK) Consortium	Harper VanSteenhouse, Yumi Kasai, David Gray, Carol Clugston, Anna Dominiczak and Alex Alderton, Roberto Amato, Sonia Goncalves, Ewan Harrison, David K. Jackson, Ian Johnston, Dominic Kwiatkowski, Cordelia Langford, John Sillitoe on behalf of the Wellcome Sanger Institute COVID-19 Surveillance Team
EPI_ISL_769615, EPI_ISL_769618, EPI_ISL_769620, EPI_ISL_769621, EPI_ISL_769622, EPI_ISL_769624	Lighthouse Lab in Milton Keynes	Wellcome Sanger Institute for the COVID-19 Genomics UK (COG-UK) Consortium	The Lighthouse Lab in Milton Keynes and Alex Alderton, Roberto Amato, Sonia Goncalves, Ewan Harrison, David K. Jackson, Ian Johnston, Dominic Kwiatkowski, Cordelia Langford, John Sillitoe on behalf of the Wellcome Sanger Institute COVID-19 Surveillance Team
EPI_ISL_769625	Lighthouse Lab in Glasgow	Wellcome Sanger Institute for the COVID-19 Genomics UK (COG-UK) Consortium	Harper VanSteenhouse, Yumi Kasai, David Gray, Carol Clugston, Anna Dominiczak and Alex Alderton, Roberto Amato, Sonia Goncalves, Ewan Harrison, David K. Jackson, Ian Johnston, Dominic Kwiatkowski, Cordelia Langford, John Sillitoe on behalf of the Wellcome Sanger Institute COVID-19 Surveillance Team
EPI_ISL_769626, EPI_ISL_769627, EPI_ISL_769628, EPI_ISL_769629, EPI_ISL_769631, EPI_ISL_769633, EPI_ISL_769634, EPI_ISL_769637, EPI_ISL_769638, EPI_ISL_769640	Lighthouse Lab in Milton Keynes	Wellcome Sanger Institute for the COVID-19 Genomics UK (COG-UK) Consortium	The Lighthouse Lab in Milton Keynes and Alex Alderton, Roberto Amato, Sonia Goncalves, Ewan Harrison, David K. Jackson, Ian Johnston, Dominic Kwiatkowski, Cordelia Langford, John Sillitoe on behalf of the Wellcome Sanger Institute COVID-19 Surveillance Team
EPI_ISL_769641	Lighthouse Lab in Glasgow	Wellcome Sanger Institute for the COVID-19 Genomics UK (COG-UK) Consortium	Harper VanSteenhouse, Yumi Kasai, David Gray, Carol Clugston, Anna Dominiczak and Alex Alderton, Roberto Amato, Sonia Goncalves, Ewan Harrison, David K. Jackson, Ian Johnston, Dominic Kwiatkowski, Cordelia Langford, John Sillitoe on behalf of the Wellcome Sanger Institute COVID-19 Surveillance Team
EPI_ISL_769642, EPI_ISL_769643, EPI_ISL_769645, EPI_ISL_769647, EPI_ISL_769649, EPI_ISL_769650, EPI_ISL_769651	Lighthouse Lab in Milton Keynes	Wellcome Sanger Institute for the COVID-19 Genomics UK (COG-UK) Consortium	The Lighthouse Lab in Milton Keynes and Alex Alderton, Roberto Amato, Sonia Goncalves, Ewan Harrison, David K. Jackson, Ian Johnston, Dominic Kwiatkowski, Cordelia Langford, John Sillitoe on behalf of the Wellcome Sanger Institute COVID-19 Surveillance Team
EPI_ISL_769652	Lighthouse Lab in Glasgow	Wellcome Sanger Institute for the COVID-19 Genomics UK (COG-UK) Consortium	Harper VanSteenhouse, Yumi Kasai, David Gray, Carol Clugston, Anna Dominiczak and Alex Alderton, Roberto Amato, Sonia Goncalves, Ewan Harrison, David K. Jackson, Ian Johnston, Dominic Kwiatkowski, Cordelia Langford, John Sillitoe on behalf of the Wellcome Sanger Institute COVID-19 Surveillance Team
EPI_ISL_769653, EPI_ISL_769654	Lighthouse Lab in Milton Keynes	Wellcome Sanger Institute for the COVID-19 Genomics UK (COG-UK) Consortium	The Lighthouse Lab in Milton Keynes and Alex Alderton, Roberto Amato, Sonia Goncalves, Ewan Harrison, David K. Jackson, Ian Johnston, Dominic Kwiatkowski, Cordelia Langford, John Sillitoe on behalf of the Wellcome Sanger Institute COVID-19 Surveillance Team
EPI_ISL_769655	Lighthouse Lab in Glasgow	Wellcome Sanger Institute for the COVID-19 Genomics UK (COG-UK) Consortium	Harper VanSteenhouse, Yumi Kasai, David Gray, Carol Clugston, Anna Dominiczak and Alex Alderton, Roberto Amato, Sonia Goncalves, Ewan Harrison, David K. Jackson, Ian Johnston, Dominic Kwiatkowski, Cordelia Langford, John Sillitoe on behalf of the Wellcome Sanger Institute COVID-19 Surveillance Team
EPI_ISL_769656, EPI_ISL_769657, EPI_ISL_769658, EPI_ISL_769659, EPI_ISL_769660	Lighthouse Lab in Milton Keynes	Wellcome Sanger Institute for the COVID-19 Genomics UK (COG-UK) Consortium	The Lighthouse Lab in Milton Keynes and Alex Alderton, Roberto Amato, Sonia Goncalves, Ewan Harrison, David K. Jackson, Ian Johnston, Dominic Kwiatkowski, Cordelia Langford, John Sillitoe on behalf of the Wellcome Sanger Institute COVID-19 Surveillance Team
EPI_ISL_769662	Lighthouse Lab in Glasgow	Wellcome Sanger Institute for the COVID-19 Genomics UK (COG-UK) Consortium	Harper VanSteenhouse, Yumi Kasai, David Gray, Carol Clugston, Anna Dominiczak and Alex Alderton, Roberto Amato, Sonia Goncalves, Ewan Harrison, David K. Jackson, Ian Johnston, Dominic Kwiatkowski, Cordelia Langford, John Sillitoe on behalf of the Wellcome Sanger Institute COVID-19 Surveillance Team
EPI_ISL_769663, EPI_ISL_769664, EPI_ISL_769665	Lighthouse Lab in Milton Keynes	Wellcome Sanger Institute for the COVID-19 Genomics UK (COG-UK) Consortium	The Lighthouse Lab in Milton Keynes and Alex Alderton, Roberto Amato, Sonia Goncalves, Ewan Harrison, David K. Jackson, Ian Johnston, Dominic Kwiatkowski, Cordelia Langford, John Sillitoe on behalf of the Wellcome Sanger Institute COVID-19 Surveillance Team
EPI_ISL_769667	Lighthouse Lab in Glasgow	Wellcome Sanger Institute for the COVID-19 Genomics UK (COG-UK) Consortium	Harper VanSteenhouse, Yumi Kasai, David Gray, Carol Clugston, Anna Dominiczak and Alex Alderton, Roberto Amato, Sonia Goncalves, Ewan Harrison, David K. Jackson, Ian Johnston, Dominic Kwiatkowski, Cordelia Langford, John Sillitoe on behalf of the Wellcome Sanger Institute COVID-19 Surveillance Team
EPI_ISL_769668, EPI_ISL_769669, EPI_ISL_769670, EPI_ISL_769671, EPI_ISL_769672, EPI_ISL_769674, EPI_ISL_769676	Lighthouse Lab in Milton Keynes	Wellcome Sanger Institute for the COVID-19 Genomics UK (COG-UK) Consortium	The Lighthouse Lab in Milton Keynes and Alex Alderton, Roberto Amato, Sonia Goncalves, Ewan Harrison, David K. Jackson, Ian Johnston, Dominic Kwiatkowski, Cordelia Langford, John Sillitoe on behalf of the Wellcome Sanger Institute COVID-19 Surveillance Team
EPI_ISL_769677	Lighthouse Lab in Glasgow	Wellcome Sanger Institute for the COVID-19 Genomics UK (COG-UK) Consortium	Harper VanSteenhouse, Yumi Kasai, David Gray, Carol Clugston, Anna Dominiczak and Alex Alderton, Roberto Amato, Sonia Goncalves, Ewan Harrison, David K. Jackson, Ian Johnston, Dominic Kwiatkowski, Cordelia Langford, John Sillitoe on behalf of the Wellcome Sanger Institute COVID-19 Surveillance Team
EPI_ISL_769679, EPI_ISL_769680, EPI_ISL_769682, EPI_ISL_769683, EPI_ISL_769684, EPI_ISL_769685	Lighthouse Lab in Milton Keynes	Wellcome Sanger Institute for the COVID-19 Genomics UK (COG-UK) Consortium	The Lighthouse Lab in Milton Keynes and Alex Alderton, Roberto Amato, Sonia Goncalves, Ewan Harrison, David K. Jackson, Ian Johnston, Dominic Kwiatkowski, Cordelia Langford, John Sillitoe on behalf of the Wellcome Sanger Institute COVID-19 Surveillance Team
EPI_ISL_769687	Lighthouse Lab in Glasgow	Wellcome Sanger Institute for the COVID-19 Genomics UK (COG-UK) Consortium	Harper VanSteenhouse, Yumi Kasai, David Gray, Carol Clugston, Anna Dominiczak and Alex Alderton, Roberto Amato, Sonia Goncalves, Ewan Harrison, David K. Jackson, Ian Johnston, Dominic Kwiatkowski, Cordelia Langford, John Sillitoe on behalf of the Wellcome Sanger Institute COVID-19 Surveillance Team
EPI_ISL_769688	Lighthouse Lab in Milton Keynes	Wellcome Sanger Institute for the COVID-19 Genomics UK (COG-UK) Consortium	The Lighthouse Lab in Milton Keynes and Alex Alderton, Roberto Amato, Sonia Goncalves, Ewan Harrison, David K. Jackson, Ian Johnston, Dominic Kwiatkowski, Cordelia Langford, John Sillitoe on behalf of the Wellcome Sanger Institute COVID-19 Surveillance Team
EPI_ISL_769689	Lighthouse Lab in Glasgow	Wellcome Sanger Institute for the COVID-19 Genomics UK (COG-UK) Consortium	Harper VanSteenhouse, Yumi Kasai, David Gray, Carol Clugston, Anna Dominiczak and Alex Alderton, Roberto Amato, Sonia Goncalves, Ewan Harrison, David K. Jackson, Ian Johnston, Dominic Kwiatkowski, Cordelia Langford, John Sillitoe on behalf of the Wellcome Sanger Institute COVID-19 Surveillance Team
EPI_ISL_769692, EPI_ISL_769694, EPI_ISL_769700, EPI_ISL_769701,	Lighthouse Lab in Milton Keynes	Wellcome Sanger Institute for the COVID-19 Genomics UK (COG-UK) Consortium	The Lighthouse Lab in Milton Keynes and Alex Alderton, Roberto Amato, Sonia Goncalves, Ewan Harrison, David K. Jackson, Ian Johnston, Dominic Kwiatkowski, Cordelia Langford, John Sillitoe on behalf of the Wellcome Sanger Institute COVID-19 Surveillance Team

EPI_ISL_769702, EPI_ISL_769704			
EPI_ISL_769705	Lighthouse Lab in Glasgow	Wellcome Sanger Institute for the COVID-19 Genomics UK (COG-UK) Consortium	Harper VanSteenhouse, Yumi Kasai, David Gray, Carol Clugston, Anna Dominiczak and Alex Alderton, Roberto Amato, Sonia Goncalves, Ewan Harrison, David K. Jackson, Ian Johnston, Dominic Kwiatkowski, Cordelia Langford, John Sillitoe on behalf of the Wellcome Sanger Institute COVID-19 Surveillance Team
EPI_ISL_769707, EPI_ISL_769709	Lighthouse Lab in Milton Keynes	Wellcome Sanger Institute for the COVID-19 Genomics UK (COG-UK) Consortium	The Lighthouse Lab in Milton Keynes and Alex Alderton, Roberto Amato, Sonia Goncalves, Ewan Harrison, David K. Jackson, Ian Johnston, Dominic Kwiatkowski, Cordelia Langford, John Sillitoe on behalf of the Wellcome Sanger Institute COVID-19 Surveillance Team
EPI_ISL_769711	Lighthouse Lab in Glasgow	Wellcome Sanger Institute for the COVID-19 Genomics UK (COG-UK) Consortium	Harper VanSteenhouse, Yumi Kasai, David Gray, Carol Clugston, Anna Dominiczak and Alex Alderton, Roberto Amato, Sonia Goncalves, Ewan Harrison, David K. Jackson, Ian Johnston, Dominic Kwiatkowski, Cordelia Langford, John Sillitoe on behalf of the Wellcome Sanger Institute COVID-19 Surveillance Team
EPI_ISL_769712	Lighthouse Lab in Milton Keynes	Wellcome Sanger Institute for the COVID-19 Genomics UK (COG-UK) Consortium	The Lighthouse Lab in Milton Keynes and Alex Alderton, Roberto Amato, Sonia Goncalves, Ewan Harrison, David K. Jackson, Ian Johnston, Dominic Kwiatkowski, Cordelia Langford, John Sillitoe on behalf of the Wellcome Sanger Institute COVID-19 Surveillance Team
EPI_ISL_769713, EPI_ISL_769714	Lighthouse Lab in Glasgow	Wellcome Sanger Institute for the COVID-19 Genomics UK (COG-UK) Consortium	Harper VanSteenhouse, Yumi Kasai, David Gray, Carol Clugston, Anna Dominiczak and Alex Alderton, Roberto Amato, Sonia Goncalves, Ewan Harrison, David K. Jackson, Ian Johnston, Dominic Kwiatkowski, Cordelia Langford, John Sillitoe on behalf of the Wellcome Sanger Institute COVID-19 Surveillance Team
EPI_ISL_769715	Lighthouse Lab in Milton Keynes	Wellcome Sanger Institute for the COVID-19 Genomics UK (COG-UK) Consortium	The Lighthouse Lab in Milton Keynes and Alex Alderton, Roberto Amato, Sonia Goncalves, Ewan Harrison, David K. Jackson, Ian Johnston, Dominic Kwiatkowski, Cordelia Langford, John Sillitoe on behalf of the Wellcome Sanger Institute COVID-19 Surveillance Team
EPI_ISL_769716	Lighthouse Lab in Glasgow	Wellcome Sanger Institute for the COVID-19 Genomics UK (COG-UK) Consortium	Harper VanSteenhouse, Yumi Kasai, David Gray, Carol Clugston, Anna Dominiczak and Alex Alderton, Roberto Amato, Sonia Goncalves, Ewan Harrison, David K. Jackson, Ian Johnston, Dominic Kwiatkowski, Cordelia Langford, John Sillitoe on behalf of the Wellcome Sanger Institute COVID-19 Surveillance Team
EPI_ISL_769717, EPI_ISL_769719	Lighthouse Lab in Milton Keynes	Wellcome Sanger Institute for the COVID-19 Genomics UK (COG-UK) Consortium	The Lighthouse Lab in Milton Keynes and Alex Alderton, Roberto Amato, Sonia Goncalves, Ewan Harrison, David K. Jackson, Ian Johnston, Dominic Kwiatkowski, Cordelia Langford, John Sillitoe on behalf of the Wellcome Sanger Institute COVID-19 Surveillance Team
EPI_ISL_769720	Lighthouse Lab in Glasgow	Wellcome Sanger Institute for the COVID-19 Genomics UK (COG-UK) Consortium	Harper VanSteenhouse, Yumi Kasai, David Gray, Carol Clugston, Anna Dominiczak and Alex Alderton, Roberto Amato, Sonia Goncalves, Ewan Harrison, David K. Jackson, Ian Johnston, Dominic Kwiatkowski, Cordelia Langford, John Sillitoe on behalf of the Wellcome Sanger Institute COVID-19 Surveillance Team
EPI_ISL_769721, EPI_ISL_769722, EPI_ISL_769723	Lighthouse Lab in Milton Keynes	Wellcome Sanger Institute for the COVID-19 Genomics UK (COG-UK) Consortium	The Lighthouse Lab in Milton Keynes and Alex Alderton, Roberto Amato, Sonia Goncalves, Ewan Harrison, David K. Jackson, Ian Johnston, Dominic Kwiatkowski, Cordelia Langford, John Sillitoe on behalf of the Wellcome Sanger Institute COVID-19 Surveillance Team
EPI_ISL_769724	Lighthouse Lab in Glasgow	Wellcome Sanger Institute for the COVID-19 Genomics UK (COG-UK) Consortium	Harper VanSteenhouse, Yumi Kasai, David Gray, Carol Clugston, Anna Dominiczak and Alex Alderton, Roberto Amato, Sonia Goncalves, Ewan Harrison, David K. Jackson, Ian Johnston, Dominic Kwiatkowski, Cordelia Langford, John Sillitoe on behalf of the Wellcome Sanger Institute COVID-19 Surveillance Team
EPI_ISL_769725, EPI_ISL_769726, EPI_ISL_769727, EPI_ISL_769729, EPI_ISL_769730, EPI_ISL_769731, EPI_ISL_769732, EPI_ISL_769733, EPI_ISL_769734	Lighthouse Lab in Milton Keynes	Wellcome Sanger Institute for the COVID-19 Genomics UK (COG-UK) Consortium	The Lighthouse Lab in Milton Keynes and Alex Alderton, Roberto Amato, Sonia Goncalves, Ewan Harrison, David K. Jackson, Ian Johnston, Dominic Kwiatkowski, Cordelia Langford, John Sillitoe on behalf of the Wellcome Sanger Institute COVID-19 Surveillance Team
EPI_ISL_769735	Lighthouse Lab in Glasgow	Wellcome Sanger Institute for the COVID-19 Genomics UK (COG-UK) Consortium	Harper VanSteenhouse, Yumi Kasai, David Gray, Carol Clugston, Anna Dominiczak and Alex Alderton, Roberto Amato, Sonia Goncalves, Ewan Harrison, David K. Jackson, Ian Johnston, Dominic Kwiatkowski, Cordelia Langford, John Sillitoe on behalf of the Wellcome Sanger Institute COVID-19 Surveillance Team
EPI_ISL_769737, EPI_ISL_769738, EPI_ISL_769739, EPI_ISL_769740, EPI_ISL_769742	Lighthouse Lab in Milton Keynes	Wellcome Sanger Institute for the COVID-19 Genomics UK (COG-UK) Consortium	The Lighthouse Lab in Milton Keynes and Alex Alderton, Roberto Amato, Sonia Goncalves, Ewan Harrison, David K. Jackson, Ian Johnston, Dominic Kwiatkowski, Cordelia Langford, John Sillitoe on behalf of the Wellcome Sanger Institute COVID-19 Surveillance Team
EPI_ISL_769743	Lighthouse Lab in Glasgow	Wellcome Sanger Institute for the COVID-19 Genomics UK (COG-UK) Consortium	Harper VanSteenhouse, Yumi Kasai, David Gray, Carol Clugston, Anna Dominiczak and Alex Alderton, Roberto Amato, Sonia Goncalves, Ewan Harrison, David K. Jackson, Ian Johnston, Dominic Kwiatkowski, Cordelia Langford, John Sillitoe on behalf of the Wellcome Sanger Institute COVID-19 Surveillance Team
EPI_ISL_769744, EPI_ISL_769746, EPI_ISL_769747, EPI_ISL_769748, EPI_ISL_769749, EPI_ISL_769750	Lighthouse Lab in Milton Keynes	Wellcome Sanger Institute for the COVID-19 Genomics UK (COG-UK) Consortium	The Lighthouse Lab in Milton Keynes and Alex Alderton, Roberto Amato, Sonia Goncalves, Ewan Harrison, David K. Jackson, Ian Johnston, Dominic Kwiatkowski, Cordelia Langford, John Sillitoe on behalf of the Wellcome Sanger Institute COVID-19 Surveillance Team
EPI_ISL_769751, EPI_ISL_769752, EPI_ISL_769753	Lighthouse Lab in Glasgow	Wellcome Sanger Institute for the COVID-19 Genomics UK (COG-UK) Consortium	Harper VanSteenhouse, Yumi Kasai, David Gray, Carol Clugston, Anna Dominiczak and Alex Alderton, Roberto Amato, Sonia Goncalves, Ewan Harrison, David K. Jackson, Ian Johnston, Dominic Kwiatkowski, Cordelia Langford, John Sillitoe on behalf of the Wellcome Sanger Institute COVID-19 Surveillance Team
EPI_ISL_769754, EPI_ISL_769755, EPI_ISL_769757	Lighthouse Lab in Milton Keynes	Wellcome Sanger Institute for the COVID-19 Genomics UK (COG-UK) Consortium	The Lighthouse Lab in Milton Keynes and Alex Alderton, Roberto Amato, Sonia Goncalves, Ewan Harrison, David K. Jackson, Ian Johnston, Dominic Kwiatkowski, Cordelia Langford, John Sillitoe on behalf of the Wellcome Sanger Institute COVID-19 Surveillance Team
EPI_ISL_769758, EPI_ISL_769759	Lighthouse Lab in Glasgow	Wellcome Sanger Institute for the COVID-19 Genomics UK (COG-UK) Consortium	Harper VanSteenhouse, Yumi Kasai, David Gray, Carol Clugston, Anna Dominiczak and Alex Alderton, Roberto Amato, Sonia Goncalves, Ewan Harrison, David K. Jackson, Ian Johnston, Dominic Kwiatkowski, Cordelia Langford, John Sillitoe on behalf of the Wellcome Sanger Institute COVID-19 Surveillance Team
EPI_ISL_769762, EPI_ISL_769763, EPI_ISL_769765, EPI_ISL_769766	Lighthouse Lab in Milton Keynes	Wellcome Sanger Institute for the COVID-19 Genomics UK (COG-UK) Consortium	The Lighthouse Lab in Milton Keynes and Alex Alderton, Roberto Amato, Sonia Goncalves, Ewan Harrison, David K. Jackson, Ian Johnston, Dominic Kwiatkowski, Cordelia Langford, John Sillitoe on behalf of the Wellcome Sanger Institute COVID-19 Surveillance Team
EPI_ISL_769768	Lighthouse Lab in Glasgow	Wellcome Sanger Institute for the COVID-19 Genomics UK (COG-UK) Consortium	Harper VanSteenhouse, Yumi Kasai, David Gray, Carol Clugston, Anna Dominiczak and Alex Alderton, Roberto Amato, Sonia Goncalves, Ewan Harrison, David K. Jackson, Ian Johnston, Dominic Kwiatkowski, Cordelia Langford, John Sillitoe on behalf of the Wellcome Sanger Institute COVID-19 Surveillance Team
EPI_ISL_769770, EPI_ISL_769772	Lighthouse Lab in Milton Keynes	Wellcome Sanger Institute for the COVID-19 Genomics UK (COG-UK) Consortium	The Lighthouse Lab in Milton Keynes and Alex Alderton, Roberto Amato, Sonia Goncalves, Ewan Harrison, David K. Jackson, Ian Johnston, Dominic Kwiatkowski, Cordelia Langford, John Sillitoe on behalf of the Wellcome Sanger Institute COVID-19 Surveillance Team
EPI_ISL_769773	Lighthouse Lab in Glasgow	Wellcome Sanger Institute for the COVID-19 Genomics UK (COG-UK) Consortium	Harper VanSteenhouse, Yumi Kasai, David Gray, Carol Clugston, Anna Dominiczak and Alex Alderton, Roberto Amato, Sonia Goncalves, Ewan Harrison, David K. Jackson, Ian Johnston, Dominic Kwiatkowski, Cordelia Langford, John Sillitoe on behalf of the Wellcome Sanger Institute COVID-19 Surveillance Team
EPI_ISL_769775, EPI_ISL_769776, EPI_ISL_769777, EPI_ISL_769778, EPI_ISL_769780, EPI_ISL_769781, EPI_ISL_769782, EPI_ISL_769783, EPI_ISL_769785, EPI_ISL_769786	Lighthouse Lab in Milton Keynes	Wellcome Sanger Institute for the COVID-19 Genomics UK (COG-UK) Consortium	The Lighthouse Lab in Milton Keynes and Alex Alderton, Roberto Amato, Sonia Goncalves, Ewan Harrison, David K. Jackson, Ian Johnston, Dominic Kwiatkowski, Cordelia Langford, John Sillitoe on behalf of the Wellcome Sanger Institute COVID-19 Surveillance Team
EPI_ISL_769787, EPI_ISL_769788	Lighthouse Lab in Glasgow	Wellcome Sanger Institute for the COVID-19 Genomics UK (COG-UK) Consortium	Harper VanSteenhouse, Yumi Kasai, David Gray, Carol Clugston, Anna Dominiczak and Alex Alderton, Roberto Amato, Sonia Goncalves, Ewan Harrison, David K. Jackson, Ian Johnston, Dominic Kwiatkowski, Cordelia Langford, John Sillitoe on behalf of the Wellcome Sanger Institute COVID-19 Surveillance Team
EPI_ISL_769789, EPI_ISL_769790, EPI_ISL_769793	Lighthouse Lab in Milton Keynes	Wellcome Sanger Institute for the COVID-19 Genomics UK (COG-UK) Consortium	The Lighthouse Lab in Milton Keynes and Alex Alderton, Roberto Amato, Sonia Goncalves, Ewan Harrison, David K. Jackson, Ian Johnston, Dominic Kwiatkowski, Cordelia Langford, John Sillitoe on behalf of the Wellcome Sanger Institute COVID-19 Surveillance Team

EPI_ISL_769794	Lighthouse Lab in Glasgow	Wellcome Sanger Institute for the COVID-19 Genomics UK (COG-UK) Consortium	Harper VanSteenhouse, Yumi Kasai, David Gray, Carol Clugston, Anna Dominiczak and Alex Alderton, Roberto Amato, Sonia Goncalves, Ewan Harrison, David K. Jackson, Ian Johnston, Dominic Kwiatkowski, Cordelia Langford, John Sillitoe on behalf of the Wellcome Sanger Institute COVID-19 Surveillance Team
EPI_ISL_769795, EPI_ISL_769797, EPI_ISL_769798, EPI_ISL_769799, EPI_ISL_769800, EPI_ISL_769801	Lighthouse Lab in Milton Keynes	Wellcome Sanger Institute for the COVID-19 Genomics UK (COG-UK) Consortium	The Lighthouse Lab in Milton Keynes and Alex Alderton, Roberto Amato, Sonia Goncalves, Ewan Harrison, David K. Jackson, Ian Johnston, Dominic Kwiatkowski, Cordelia Langford, John Sillitoe on behalf of the Wellcome Sanger Institute COVID-19 Surveillance Team
EPI_ISL_769802	Lighthouse Lab in Glasgow	Wellcome Sanger Institute for the COVID-19 Genomics UK (COG-UK) Consortium	Harper VanSteenhouse, Yumi Kasai, David Gray, Carol Clugston, Anna Dominiczak and Alex Alderton, Roberto Amato, Sonia Goncalves, Ewan Harrison, David K. Jackson, Ian Johnston, Dominic Kwiatkowski, Cordelia Langford, John Sillitoe on behalf of the Wellcome Sanger Institute COVID-19 Surveillance Team
EPI_ISL_769803, EPI_ISL_769805	Lighthouse Lab in Milton Keynes	Wellcome Sanger Institute for the COVID-19 Genomics UK (COG-UK) Consortium	The Lighthouse Lab in Milton Keynes and Alex Alderton, Roberto Amato, Sonia Goncalves, Ewan Harrison, David K. Jackson, Ian Johnston, Dominic Kwiatkowski, Cordelia Langford, John Sillitoe on behalf of the Wellcome Sanger Institute COVID-19 Surveillance Team
EPI_ISL_769806	Lighthouse Lab in Glasgow	Wellcome Sanger Institute for the COVID-19 Genomics UK (COG-UK) Consortium	Harper VanSteenhouse, Yumi Kasai, David Gray, Carol Clugston, Anna Dominiczak and Alex Alderton, Roberto Amato, Sonia Goncalves, Ewan Harrison, David K. Jackson, Ian Johnston, Dominic Kwiatkowski, Cordelia Langford, John Sillitoe on behalf of the Wellcome Sanger Institute COVID-19 Surveillance Team
EPI_ISL_769807, EPI_ISL_769808, EPI_ISL_769812	Lighthouse Lab in Milton Keynes	Wellcome Sanger Institute for the COVID-19 Genomics UK (COG-UK) Consortium	The Lighthouse Lab in Milton Keynes and Alex Alderton, Roberto Amato, Sonia Goncalves, Ewan Harrison, David K. Jackson, Ian Johnston, Dominic Kwiatkowski, Cordelia Langford, John Sillitoe on behalf of the Wellcome Sanger Institute COVID-19 Surveillance Team
EPI_ISL_769813	Lighthouse Lab in Glasgow	Wellcome Sanger Institute for the COVID-19 Genomics UK (COG-UK) Consortium	Harper VanSteenhouse, Yumi Kasai, David Gray, Carol Clugston, Anna Dominiczak and Alex Alderton, Roberto Amato, Sonia Goncalves, Ewan Harrison, David K. Jackson, Ian Johnston, Dominic Kwiatkowski, Cordelia Langford, John Sillitoe on behalf of the Wellcome Sanger Institute COVID-19 Surveillance Team
EPI_ISL_769814, EPI_ISL_769817, EPI_ISL_769820, EPI_ISL_769822, EPI_ISL_769823, EPI_ISL_769824, EPI_ISL_769825	Lighthouse Lab in Milton Keynes	Wellcome Sanger Institute for the COVID-19 Genomics UK (COG-UK) Consortium	The Lighthouse Lab in Milton Keynes and Alex Alderton, Roberto Amato, Sonia Goncalves, Ewan Harrison, David K. Jackson, Ian Johnston, Dominic Kwiatkowski, Cordelia Langford, John Sillitoe on behalf of the Wellcome Sanger Institute COVID-19 Surveillance Team
EPI_ISL_769826	Lighthouse Lab in Glasgow	Wellcome Sanger Institute for the COVID-19 Genomics UK (COG-UK) Consortium	Harper VanSteenhouse, Yumi Kasai, David Gray, Carol Clugston, Anna Dominiczak and Alex Alderton, Roberto Amato, Sonia Goncalves, Ewan Harrison, David K. Jackson, Ian Johnston, Dominic Kwiatkowski, Cordelia Langford, John Sillitoe on behalf of the Wellcome Sanger Institute COVID-19 Surveillance Team
EPI_ISL_769827, EPI_ISL_769828, EPI_ISL_769829, EPI_ISL_769830, EPI_ISL_769835	Lighthouse Lab in Milton Keynes	Wellcome Sanger Institute for the COVID-19 Genomics UK (COG-UK) Consortium	The Lighthouse Lab in Milton Keynes and Alex Alderton, Roberto Amato, Sonia Goncalves, Ewan Harrison, David K. Jackson, Ian Johnston, Dominic Kwiatkowski, Cordelia Langford, John Sillitoe on behalf of the Wellcome Sanger Institute COVID-19 Surveillance Team
EPI_ISL_769836	Lighthouse Lab in Glasgow	Wellcome Sanger Institute for the COVID-19 Genomics UK (COG-UK) Consortium	Harper VanSteenhouse, Yumi Kasai, David Gray, Carol Clugston, Anna Dominiczak and Alex Alderton, Roberto Amato, Sonia Goncalves, Ewan Harrison, David K. Jackson, Ian Johnston, Dominic Kwiatkowski, Cordelia Langford, John Sillitoe on behalf of the Wellcome Sanger Institute COVID-19 Surveillance Team
EPI_ISL_769838, EPI_ISL_769841, EPI_ISL_769843, EPI_ISL_769845, EPI_ISL_769846, EPI_ISL_769847, EPI_ISL_769848, EPI_ISL_769849	Lighthouse Lab in Milton Keynes	Wellcome Sanger Institute for the COVID-19 Genomics UK (COG-UK) Consortium	The Lighthouse Lab in Milton Keynes and Alex Alderton, Roberto Amato, Sonia Goncalves, Ewan Harrison, David K. Jackson, Ian Johnston, Dominic Kwiatkowski, Cordelia Langford, John Sillitoe on behalf of the Wellcome Sanger Institute COVID-19 Surveillance Team
EPI_ISL_769850	Lighthouse Lab in Glasgow	Wellcome Sanger Institute for the COVID-19 Genomics UK (COG-UK) Consortium	Harper VanSteenhouse, Yumi Kasai, David Gray, Carol Clugston, Anna Dominiczak and Alex Alderton, Roberto Amato, Sonia Goncalves, Ewan Harrison, David K. Jackson, Ian Johnston, Dominic Kwiatkowski, Cordelia Langford, John Sillitoe on behalf of the Wellcome Sanger Institute COVID-19 Surveillance Team
EPI_ISL_769851, EPI_ISL_769852, EPI_ISL_769853, EPI_ISL_769854, EPI_ISL_769855, EPI_ISL_769856, EPI_ISL_769857, EPI_ISL_769858, EPI_ISL_769859, EPI_ISL_769860	Lighthouse Lab in Milton Keynes	Wellcome Sanger Institute for the COVID-19 Genomics UK (COG-UK) Consortium	The Lighthouse Lab in Milton Keynes and Alex Alderton, Roberto Amato, Sonia Goncalves, Ewan Harrison, David K. Jackson, Ian Johnston, Dominic Kwiatkowski, Cordelia Langford, John Sillitoe on behalf of the Wellcome Sanger Institute COVID-19 Surveillance Team
EPI_ISL_769861	Lighthouse Lab in Glasgow	Wellcome Sanger Institute for the COVID-19 Genomics UK (COG-UK) Consortium	Harper VanSteenhouse, Yumi Kasai, David Gray, Carol Clugston, Anna Dominiczak and Alex Alderton, Roberto Amato, Sonia Goncalves, Ewan Harrison, David K. Jackson, Ian Johnston, Dominic Kwiatkowski, Cordelia Langford, John Sillitoe on behalf of the Wellcome Sanger Institute COVID-19 Surveillance Team
EPI_ISL_769863	Laboratoire Biolife	Laboratoire de Biotechnologie	Mouna Ouadghiri, Tarik Aanniz, Mohammed Walid Chemao Elfihri, Mohamed Chenaoui, Hanae Dakka, Afaf Alaoui, Otmane Touzani, Amina Benouda, Bouchra Belfquih, Lahcen belyamani, Saaid Amzazi and Azeddine Ibrahimi
EPI_ISL_769878	Respiratory Virus Unit, National Infection Service, Public Health England	COVID-19 Genomics UK (COG-UK) Consortium	PHE Covid Sequencing Team
EPI_ISL_769919, EPI_ISL_769920, EPI_ISL_769921, EPI_ISL_769922, EPI_ISL_769923, EPI_ISL_769924, EPI_ISL_769926, EPI_ISL_769927, EPI_ISL_769929, EPI_ISL_769930, EPI_ISL_769931, EPI_ISL_769932, EPI_ISL_769933, EPI_ISL_769934, EPI_ISL_769935, EPI_ISL_769936, EPI_ISL_769937, EPI_ISL_769939, EPI_ISL_769941, EPI_ISL_769944, EPI_ISL_769946, EPI_ISL_769947, EPI_ISL_769949, EPI_ISL_769952, EPI_ISL_769955, EPI_ISL_769956, EPI_ISL_769957, EPI_ISL_769961, EPI_ISL_769962, EPI_ISL_769963, EPI_ISL_769964, EPI_ISL_769965, EPI_ISL_769966, EPI_ISL_769967, EPI_ISL_769968, EPI_ISL_769969, EPI_ISL_769970, EPI_ISL_769971, EPI_ISL_769975, EPI_ISL_769976, EPI_ISL_769977, EPI_ISL_769979, EPI_ISL_769983, EPI_ISL_769984			
see above	Albany Medical Center Hospital Clinical Laboratories	Wadsworth Center, New York State Department.of Health	Kirsten St. George, Daryl M. Lamson, Alexis Russel, Matthew Shudt, Melissa A Leisner, Jonathan Plitnick, Navjot Singh, John Kelly, Sara Griesemer, Erasmus Schneider, Erica Lasek-Nesselquist
EPI_ISL_770500, EPI_ISL_770501, EPI_ISL_770502, EPI_ISL_770503, EPI_ISL_770504, EPI_ISL_770505, EPI_ISL_770506, EPI_ISL_770507, EPI_ISL_770508, EPI_ISL_770509, EPI_ISL_770510, EPI_ISL_770511, EPI_ISL_770512, EPI_ISL_770513, EPI_ISL_770514, EPI_ISL_770515, EPI_ISL_770516, EPI_ISL_770517, EPI_ISL_770518, EPI_ISL_770519, EPI_ISL_770520, EPI_ISL_770521, EPI_ISL_770522, EPI_ISL_770523, EPI_ISL_770524, EPI_ISL_770525, EPI_ISL_770526, EPI_ISL_770527, EPI_ISL_770529, EPI_ISL_770530, EPI_ISL_770531, EPI_ISL_770532			
see above	Lithuanian University of Health Sciences Hospital, Department of Laboratory Medicine	Lithuanian University of Health Sciences, Molecular cardiology lab.	Lukas Zemaitis, Ingrida Olendrait, Arnoldas Pautienius, Kamile Tamusauskaite, Dovydas Gecys, Laura Pareckaite, Vaiva Lesauskaite, Astra Vitkauskiene
EPI_ISL_770692, EPI_ISL_770693, EPI_ISL_770694, EPI_ISL_770698, EPI_ISL_770701, EPI_ISL_770704, EPI_ISL_770708, EPI_ISL_770710, EPI_ISL_770711, EPI_ISL_770713, EPI_ISL_770719			
see above	ZOTZ KLIMAS MVZ Düsseldorf-Centrum GbR ÜBAG für Labormedizin, Genetik, Zytologie, Pathologie	Center of Medical Microbiology, Virology, and Hospital Hygiene, University of Duesseldorf	Maximilian Damagnez, Alexander Dilthey, Ashley-Jane Duplessis, Patrick Finzer, Katrin Hoffmann, Torsten Houwaart, Lisanna Hülse, Malte Kohns Vasconcelos, Marek Korencak, Nadine Lübke, Jessica Nicolai, Klaus Pfeffer, Daniel Strelow, Jörg Timm, Andreas Walker, Tobias Wienemann, Rainer Zotz
EPI_ISL_770783, EPI_ISL_770784, EPI_ISL_770785, EPI_ISL_770786, EPI_ISL_770787, EPI_ISL_770788, EPI_ISL_770789, EPI_ISL_770791, EPI_ISL_770792, EPI_ISL_770795, EPI_ISL_770796, EPI_ISL_770798, EPI_ISL_770799			
see above	Minnesota Department of Health, Public Health Laboratory	Minnesota Department of Health, Public Health Laboratory	Alexandra Lorentz, Jacob Garfin, Matt Plumb, and Xiong Wang
EPI_ISL_770802, EPI_ISL_770804, EPI_ISL_770806	Vault Health	Minnesota Department of Health, Public Health Laboratory	Alexandra Lorentz, Jacob Garfin, Matt Plumb, and Xiong Wang
EPI_ISL_770816, EPI_ISL_770817	Minnesota Department of Health, Public Health Laboratory	Minnesota Department of Health, Public Health Laboratory	Alexandra Lorentz, Jacob Garfin, Matt Plumb, and Xiong Wang
EPI_ISL_770818	North Memorial	Minnesota Department of Health, Public Health Laboratory	Alexandra Lorentz, Jacob Garfin, Matt Plumb, and Xiong Wang
EPI_ISL_770819	Minnesota Department of Health, Public Health Laboratory	Minnesota Department of Health, Public Health Laboratory	Alexandra Lorentz, Jacob Garfin, Matt Plumb, and Xiong Wang
EPI_ISL_770878, EPI_ISL_770879, EPI_ISL_770880, EPI_ISL_770881, EPI_ISL_770882, EPI_ISL_770889, EPI_ISL_770892, EPI_ISL_770895, EPI_ISL_770898, EPI_ISL_770899, EPI_ISL_770900, EPI_ISL_770901, EPI_ISL_770903, EPI_ISL_770904, EPI_ISL_770905, EPI_ISL_770907, EPI_ISL_770932, EPI_ISL_770933, EPI_ISL_770934, EPI_ISL_770935, EPI_ISL_770936, EPI_ISL_770937, EPI_ISL_770943, EPI_ISL_770944, EPI_ISL_770945, EPI_ISL_770946, EPI_ISL_770948, EPI_ISL_770949, EPI_ISL_770951, EPI_ISL_770952, EPI_ISL_770953, EPI_ISL_770955, EPI_ISL_770976, EPI_ISL_770977, EPI_ISL_770978, EPI_ISL_770979, EPI_ISL_770980, EPI_ISL_770981, EPI_ISL_770982, EPI_ISL_770983, EPI_ISL_770986, EPI_ISL_770987, EPI_ISL_770988, EPI_ISL_770989, EPI_ISL_770990, EPI_ISL_770991, EPI_ISL_770992, EPI_ISL_770993, EPI_ISL_770994, EPI_ISL_770995, EPI_ISL_770996, EPI_ISL_770997, EPI_ISL_770998, EPI_ISL_770999,			

EPI_ISL_771000, EPI_ISL_771001, EPI_ISL_771002, EPI_ISL_771003, EPI_ISL_771004, EPI_ISL_771005, EPI_ISL_771006, EPI_ISL_771008, EPI_ISL_771010, EPI_ISL_771011, EPI_ISL_771012, EPI_ISL_771013, EPI_ISL_771014, EPI_ISL_771015, EPI_ISL_771016, EPI_ISL_771017, EPI_ISL_771018, EPI_ISL_771019, EPI_ISL_771021, EPI_ISL_771022, EPI_ISL_771023, EPI_ISL_771024, EPI_ISL_771025, EPI_ISL_771026, EPI_ISL_771027, EPI_ISL_771028, EPI_ISL_771030, EPI_ISL_771031, EPI_ISL_771032, EPI_ISL_771033, EPI_ISL_771034, EPI_ISL_771035, EPI_ISL_771036, EPI_ISL_771037, EPI_ISL_771038, EPI_ISL_771039, EPI_ISL_771040, EPI_ISL_771041, EPI_ISL_771042, EPI_ISL_771043, EPI_ISL_771044, EPI_ISL_771045, EPI_ISL_771046, EPI_ISL_771047, EPI_ISL_771048, EPI_ISL_771050, EPI_ISL_771051, EPI_ISL_771052, EPI_ISL_771055, EPI_ISL_771057, EPI_ISL_771058, EPI_ISL_771059, EPI_ISL_771060, EPI_ISL_771061, EPI_ISL_771062, EPI_ISL_771063, EPI_ISL_771064, EPI_ISL_771065, EPI_ISL_771066, EPI_ISL_771067, EPI_ISL_771070, EPI_ISL_771071, EPI_ISL_771072, EPI_ISL_771073, EPI_ISL_771074, EPI_ISL_771075, EPI_ISL_771076, EPI_ISL_771077, EPI_ISL_771078, EPI_ISL_771080, EPI_ISL_771081, EPI_ISL_771082, EPI_ISL_771083, EPI_ISL_771084, EPI_ISL_771085, EPI_ISL_771086, EPI_ISL_771087, EPI_ISL_771088, EPI_ISL_771089, EPI_ISL_771090, EPI_ISL_771091, EPI_ISL_771093, EPI_ISL_771094, EPI_ISL_771095, EPI_ISL_771096, EPI_ISL_771097, EPI_ISL_771098, EPI_ISL_771099, EPI_ISL_771100, EPI_ISL_771102, EPI_ISL_771103, EPI_ISL_771104, EPI_ISL_771105, EPI_ISL_771106, EPI_ISL_771107, EPI_ISL_771108, EPI_ISL_771109, EPI_ISL_771110, EPI_ISL_771111, EPI_ISL_771112, EPI_ISL_771113, EPI_ISL_771114, EPI_ISL_771115, EPI_ISL_771116, EPI_ISL_771117, EPI_ISL_771118, EPI_ISL_771119, EPI_ISL_771120, EPI_ISL_771121, EPI_ISL_771122, EPI_ISL_771123, EPI_ISL_771124, EPI_ISL_771126, EPI_ISL_771127, EPI_ISL_771128, EPI_ISL_771129, EPI_ISL_771130, EPI_ISL_771131, EPI_ISL_771132, EPI_ISL_771133, EPI_ISL_771134, EPI_ISL_771135, EPI_ISL_771136, EPI_ISL_771137, EPI_ISL_771138, EPI_ISL_771139, EPI_ISL_771140, EPI_ISL_771141, EPI_ISL_771142, EPI_ISL_771143, EPI_ISL_771144, EPI_ISL_771145, EPI_ISL_771146, EPI_ISL_771147, EPI_ISL_771148, EPI_ISL_771149, EPI_ISL_771150

see above	Laboratoire national de santé, Microbiology, Virology	Laboratoire national de santé, Microbiology, Microbial Genomics Platform	Anke Wienecke-Baldacchino, Catherine Ragimbeau,Jessica Tapp, Fatu Djabi, Lise Pignon, Raoul Salmon, Tamir Abdelrahman
EPI_ISL_774976	Gonoshasthya-RNA Molecular Diagnostic and Research Center	Gonoshasthya-RNA Molecular Diagnostic and Research Center	Nihad Adnan, Mohd. Raeed Jamiruddin, Md. Ahsanul Haq, Mohib Ullah Khondoker, Nafisa Azmuda, Firoz Ahmed, Shahana Sharmin, Salma Akter, Taslin Jahan Mou, Mahfuza Marzan, Sayeda Moriam Liza, Nowshin Jahan, Tamanna Ali, Maha Jamiruddin, Mousumi Chaity, Shahad Saif Khandker, Mumtarin Jannat Oishee
EPI_ISL_775019	Gonoshasthya-RNA Molecular Research Center	Gonoshasthya-RNA Molecular Research Center	Nihad Adnan, Mohd. Raeed Jamiruddin, Md. Ahsanul Haq, Mohib Ullah Khondoker, Nafisa Azmuda, Firoz Ahmed, Shahana Sharmin, Salma Akter, Taslin Jahan Mou, Mahfuza Marzan, Sayeda Moriam Liza, Nowshin Jahan, Tamanna Ali, Maha Jamiruddin, Mousumi Chaity, Shahad Saif Khandker, Mumtarin Jannat Oishee
EPI_ISL_775020	Gonoshasthya-RNA Molecular Research Center	Gonoshasthya-RNA Molecular Research Center	Mohd. Raeed Jamiruddin, Nihad Adnan, Md. Ahsanul Haq, Mohib Ullah Khondoker, Nafisa Azmuda, Firoz Ahmed, Shahana Sharmin, Salma Akter, Taslin Jahan Mou, Mahfuza Marzan, Sayeda Moriam Liza, Nowshin Jahan, Tamanna Ali, Shahad Saif Khandker, Maha Jamiruddin, Mousumi Chaity, Mumtarin Jannat Oishee
EPI_ISL_775213	Gonoshasthya-RNA Molecular Research Center	Gonoshasthya-RNA Molecular Research Center	Nihad Adnan, Mohd. Raeed Jamiruddin, Md. Ahsanul Haq, Mohib Ullah Khondoker, Nafisa Azmuda, Firoz Ahmed, Shahana Sharmin, Salma Akter, Taslin Jahan Mou, Mahfuza Marzan, Sayeda Moriam Liza, Nowshin Jahan, Tamanna Ali, Maha Jamiruddin, Mousumi Chaity, Shahad Saif Khandker, Mumtarin Jannat Oishee
EPI_ISL_775218	Gonoshasthya-RNA Molecular Research Center	Gonoshasthya-RNA Molecular Research Center	Mohd. Raeed Jamiruddin, Nihad Adnan, Md. Ahsanul Haq, Mohib Ullah Khondoker, Nafisa Azmuda, Firoz Ahmed, Shahana Sharmin, Salma Akter, Taslin Jahan Mou, Mahfuza Marzan, Sayeda Moriam Liza, Nowshin Jahan, Tamanna Ali, Shahad Saif Khandker, Maha Jamiruddin, Mousumi Chaity, Mumtarin Jannat Oishee
EPI_ISL_775220, EPI_ISL_775221, EPI_ISL_775222, EPI_ISL_775225, EPI_ISL_775256, EPI_ISL_775258, EPI_ISL_775260, EPI_ISL_775262, EPI_ISL_775263	Laboratoire Biolife	Laboratoire de Biotechnologie	Mouna Ouadghiri, Tarik Aanniz, Mohammed Walid Chemao Elfihri, Mohamed Chenaoui, Hanae Dakka, Afaf Alaoui, Otmane Touzani, Amina Benouda, Bouchra Belfquih, Lahcen belyamani, Saaid Amzazi and Azeddine Ibrahimi
EPI_ISL_775272	Dept. of Medical Microbiology, Stavanger University Hospital, Helse Stavanger HF	Norwegian Institute of Public Health, Department of Virology	Kathrine Stene-Johansen, Kamilla Heddeland Instefjord, Hilde Elshaug, Atiya R Ali,Marie Paulsen Madsen, Rasmus Riis Kopperud, Hilde Vollan, Karoline Bragstad, Olav Hungnes
EPI_ISL_775273	Furst Medical Laboratory	Norwegian Institute of Public Health, Department of Virology	Kathrine Stene-Johansen, Kamilla Heddeland Instefjord, Hilde Elshaug, Atiya R Ali,Marie Paulsen Madsen, Rasmus Riis Kopperud, Hilde Vollan, Karoline Bragstad, Olav Hungnes
EPI_ISL_775275	Akershus University Hospital, Department for Microbiology and Infectious Disease Control	Norwegian Institute of Public Health, Department of Virology	Kathrine Stene-Johansen, Kamilla Heddeland Instefjord, Hilde Elshaug, Atiya R Ali,Marie Paulsen Madsen, Rasmus Riis Kopperud, Hilde Vollan, Karoline Bragstad, Olav Hungnes
EPI_ISL_775277	Nordland Hospital - Bodo, Laboratory Department, Molecular Biology Unit	Norwegian Institute of Public Health, Department of Virology	Kathrine Stene-Johansen, Kamilla Heddeland Instefjord, Hilde Elshaug, Atiya R Ali,Marie Paulsen Madsen, Rasmus Riis Kopperud, Hilde Vollan, Karoline Bragstad, Olav Hungnes
EPI_ISL_775278	Oslo University Hospital, Department of Medical Microbiology	Norwegian Institute of Public Health, Department of Virology	Kathrine Stene-Johansen, Kamilla Heddeland Instefjord, Hilde Elshaug, Atiya R Ali,Marie Paulsen Madsen, Rasmus Riis Kopperud, Hilde Vollan, Karoline Bragstad, Olav Hungnes
EPI_ISL_775291, EPI_ISL_775292	Nordland Hospital - Bodo, Laboratory Department, Molecular Biology Unit	Norwegian Institute of Public Health, Department of Virology	Kathrine Stene-Johansen, Kamilla Heddeland Instefjord, Hilde Elshaug, Atiya R Ali,Marie Paulsen Madsen, Rasmus Riis Kopperud, Hilde Vollan, Karoline Bragstad, Olav Hungnes
EPI_ISL_775293	Oslo University Hospital, Department of Medical Microbiology	Norwegian Institute of Public Health, Department of Virology	Kathrine Stene-Johansen, Kamilla Heddeland Instefjord, Hilde Elshaug, Atiya R Ali,Marie Paulsen Madsen, Rasmus Riis Kopperud, Hilde Vollan, Karoline Bragstad, Olav Hungnes
EPI_ISL_775305	Unilabs Laboratory Medicine	Norwegian Institute of Public Health, Department of Virology	Kathrine Stene-Johansen, Kamilla Heddeland Instefjord, Hilde Elshaug, Atiya R Ali,Marie Paulsen Madsen, Rasmus Riis Kopperud, Hilde Vollan, Karoline Bragstad, Olav Hungnes
EPI_ISL_775386	Dept. of Medical Microbiology, Stavanger University Hospital, Helse Stavanger HF	Norwegian Institute of Public Health, Department of Virology	Kathrine Stene-Johansen, Kamilla Heddeland Instefjord, Hilde Elshaug, Atiya R Ali,Marie Paulsen Madsen, Rasmus Riis Kopperud, Hilde Vollan, Karoline Bragstad, Olav Hungnes
EPI_ISL_775391, EPI_ISL_775392	Oslo University Hospital, Department of Medical Microbiology	Norwegian Institute of Public Health, Department of Virology	Kathrine Stene-Johansen, Kamilla Heddeland Instefjord, Hilde Elshaug, Atiya R Ali,Marie Paulsen Madsen, Rasmus Riis Kopperud, Hilde Vollan, Karoline Bragstad, Olav Hungnes
EPI_ISL_775394, EPI_ISL_775395, EPI_ISL_775396, EPI_ISL_775397	Medical Microbiology Unit, Department for Laboratory Medicine, Drammen Hospital, Vestre Viken Health Trust,	Norwegian Institute of Public Health, Department of Virology	Kathrine Stene-Johansen, Kamilla Heddeland Instefjord, Hilde Elshaug, Atiya R Ali,Marie Paulsen Madsen, Rasmus Riis Kopperud, Hilde Vollan, Karoline Bragstad, Olav Hungnes
EPI_ISL_775399, EPI_ISL_775400, EPI_ISL_775401, EPI_ISL_775402, EPI_ISL_775403, EPI_ISL_775404	Nordland Hospital - Bodo, Laboratory Department, Molecular Biology Unit	Norwegian Institute of Public Health, Department of Virology	Kathrine Stene-Johansen, Kamilla Heddeland Instefjord, Hilde Elshaug, Atiya R Ali,Marie Paulsen Madsen, Rasmus Riis Kopperud, Hilde Vollan, Karoline Bragstad, Olav Hungnes
EPI_ISL_775406	Unilabs Laboratory Medicine	Norwegian Institute of Public Health, Department of Virology	Kathrine Stene-Johansen, Kamilla Heddeland Instefjord, Hilde Elshaug, Atiya R Ali,Marie Paulsen Madsen, Rasmus Riis Kopperud, Hilde Vollan, Karoline Bragstad, Olav Hungnes
EPI_ISL_775407, EPI_ISL_775408, EPI_ISL_775409	Haukeland University Hospital, Dept. of Microbiology	Norwegian Institute of Public Health, Department of Virology	Kathrine Stene-Johansen, Kamilla Heddeland Instefjord, Hilde Elshaug, Atiya R Ali,Marie Paulsen Madsen, Rasmus Riis Kopperud, Hilde Vollan, Karoline Bragstad, Olav Hungnes
EPI_ISL_775410, EPI_ISL_775411, EPI_ISL_775412, EPI_ISL_775413	Oslo University Hospital, Department of Medical Microbiology	Norwegian Institute of Public Health, Department of Virology	Kathrine Stene-Johansen, Kamilla Heddeland Instefjord, Hilde Elshaug, Atiya R Ali,Marie Paulsen Madsen, Rasmus Riis Kopperud, Hilde Vollan, Karoline Bragstad, Olav Hungnes
EPI_ISL_775414	Norwegian Institute of Public Health, Department of Virology	Norwegian Institute of Public Health, Department of Virology	Kathrine Stene-Johansen, Kamilla Heddeland Instefjord, Hilde Elshaug, Atiya R Ali,Marie Paulsen Madsen, Rasmus Riis Kopperud, Hilde Vollan, Karoline Bragstad, Olav Hungnes
EPI_ISL_775416, EPI_ISL_775421, EPI_ISL_775422, EPI_ISL_775423, EPI_ISL_775424, EPI_ISL_775426	Oslo University Hospital, Department of Medical Microbiology	Norwegian Institute of Public Health, Department of Virology	Kathrine Stene-Johansen, Kamilla Heddeland Instefjord, Hilde Elshaug, Atiya R Ali,Marie Paulsen Madsen, Rasmus Riis Kopperud, Hilde Vollan, Karoline Bragstad, Olav Hungnes
EPI_ISL_775427	Furst Medical Laboratory	Norwegian Institute of Public Health, Department of Virology	Kathrine Stene-Johansen, Kamilla Heddeland Instefjord, Hilde Elshaug, Atiya R Ali,Marie Paulsen Madsen, Rasmus Riis Kopperud, Hilde Vollan, Karoline Bragstad, Olav Hungnes
EPI_ISL_775428, EPI_ISL_775429, EPI_ISL_775430, EPI_ISL_775431, EPI_ISL_775432, EPI_ISL_775433, EPI_ISL_775435, EPI_ISL_775436, EPI_ISL_775437	Department of Medical Microbiology - section Molde, Molde Hospital	Norwegian Institute of Public Health, Department of Virology	Kathrine Stene-Johansen, Kamilla Heddeland Instefjord, Hilde Elshaug, Atiya R Ali,Marie Paulsen Madsen, Rasmus Riis Kopperud, Hilde Vollan, Karoline Bragstad, Olav Hungnes
EPI_ISL_775438, EPI_ISL_775439	Nordland Hospital - Bodo, Laboratory Department, Molecular	Norwegian Institute of Public Health, Department of Virology	Kathrine Stene-Johansen, Kamilla Heddeland Instefjord, Hilde Elshaug, Atiya R Ali,Marie Paulsen Madsen, Rasmus Riis Kopperud, Hilde Vollan, Karoline

	Biology Unit		Bragstad, Olav Hungnes
EPI_ISL_775442	Medical Microbiology Unit, Department for Laboratory Medicine, Drammen Hospital, Vestre Viken Health Trust,	Norwegian Institute of Public Health, Department of Virology	Kathrine Stene-Johansen, Kamilla Heddeland Instefjord, Hilde Elshaug, Atiya R Ali,Marie Paulsen Madsen, Rasmus Riis Kopperud, Hilde Vollan, Karoline Bragstad, Olav Hungnes
EPI_ISL_775443	Norwegian Institute of Public Health, Department of Virology	Norwegian Institute of Public Health, Department of Virology	Kathrine Stene-Johansen, Kamilla Heddeland Instefjord, Hilde Elshaug, Atiya R Ali,Marie Paulsen Madsen, Rasmus Riis Kopperud, Hilde Vollan, Karoline Bragstad, Olav Hungnes
EPI_ISL_775458	University Hospital of Northern Norway, Department for Microbiology and Infectious Disease Control	Norwegian Institute of Public Health, Department of Virology	Kathrine Stene-Johansen, Kamilla Heddeland Instefjord, Hilde Elshaug, Atiya R Ali,Marie Paulsen Madsen, Rasmus Riis Kopperud, Hilde Vollan, Karoline Bragstad, Olav Hungnes
EPI_ISL_775463	Unilabs Laboratory Medicine	Norwegian Institute of Public Health, Department of Virology	Kathrine Stene-Johansen, Kamilla Heddeland Instefjord, Hilde Elshaug, Atiya R Ali,Marie Paulsen Madsen, Rasmus Riis Kopperud, Hilde Vollan, Karoline Bragstad, Olav Hungnes
EPI_ISL_775496, EPI_ISL_775497	Furst Medical Laboratory	Norwegian Institute of Public Health, Department of Virology	Kathrine Stene-Johansen, Kamilla Heddeland Instefjord, Hilde Elshaug, Atiya R Ali,Marie Paulsen Madsen, Rasmus Riis Kopperud, Hilde Vollan, Karoline Bragstad, Olav Hungnes
EPI_ISL_775505, EPI_ISL_775506, EPI_ISL_775507, EPI_ISL_775508	Department of Medical Microbiology, St. Olavs hospital	Norwegian Institute of Public Health, Department of Virology	Kathrine Stene-Johansen, Kamilla Heddeland Instefjord, Hilde Elshaug, Atiya R Ali,Marie Paulsen Madsen, Rasmus Riis Kopperud, Hilde Vollan, Karoline Bragstad, Olav Hungnes
EPI_ISL_775516, EPI_ISL_775517	Unilabs Laboratory Medicine	Norwegian Institute of Public Health, Department of Virology	Kathrine Stene-Johansen, Kamilla Heddeland Instefjord, Hilde Elshaug, Atiya R Ali,Marie Paulsen Madsen, Rasmus Riis Kopperud, Hilde Vollan, Karoline Bragstad, Olav Hungnes
EPI_ISL_775518, EPI_ISL_775519, EPI_ISL_775520, EPI_ISL_775521, EPI_ISL_775522	Hospital of Southern Norway - Kristiansand, Department of Medical Microbiology	Norwegian Institute of Public Health, Department of Virology	Kathrine Stene-Johansen, Kamilla Heddeland Instefjord, Hilde Elshaug, Atiya R Ali,Marie Paulsen Madsen, Rasmus Riis Kopperud, Hilde Vollan, Karoline Bragstad, Olav Hungnes
EPI_ISL_775524, EPI_ISL_775525, EPI_ISL_775526, EPI_ISL_775527	Furst Medical Laboratory	Norwegian Institute of Public Health, Department of Virology	Kathrine Stene-Johansen, Kamilla Heddeland Instefjord, Hilde Elshaug, Atiya R Ali,Marie Paulsen Madsen, Rasmus Riis Kopperud, Hilde Vollan, Karoline Bragstad, Olav Hungnes
EPI_ISL_775528, EPI_ISL_775529, EPI_ISL_775530	Akershus University Hospital, Department for Microbiology and Infectious Disease Control	Norwegian Institute of Public Health, Department of Virology	Kathrine Stene-Johansen, Kamilla Heddeland Instefjord, Hilde Elshaug, Atiya R Ali,Marie Paulsen Madsen, Rasmus Riis Kopperud, Hilde Vollan, Karoline Bragstad, Olav Hungnes
EPI_ISL_775531	Klinisk mikrobiologi, Viruslab	The Public Health Agency of Sweden	Department of Microbiology, The Public Health Agency of Sweden
EPI_ISL_775533, EPI_ISL_775534, EPI_ISL_775535, EPI_ISL_775536, EPI_ISL_775537, EPI_ISL_775538, EPI_ISL_775539, EPI_ISL_775540, EPI_ISL_775541	ABC	The Public Health Agency of Sweden	Department of Microbiology, The Public Health Agency of Sweden
EPI_ISL_775542, EPI_ISL_775543, EPI_ISL_775544, EPI_ISL_775545	Klinisk mikrobiologi, Viruslab	The Public Health Agency of Sweden	Department of Microbiology, The Public Health Agency of Sweden
EPI_ISL_775546, EPI_ISL_775547, EPI_ISL_775548	Klinisk Mikrobiologi	The Public Health Agency of Sweden	Department of Microbiology, The Public Health Agency of Sweden
EPI_ISL_775549	Klinisk mikrobiologi	The Public Health Agency of Sweden	Department of Microbiology, The Public Health Agency of Sweden
EPI_ISL_775550, EPI_ISL_775551, EPI_ISL_775552, EPI_ISL_775553, EPI_ISL_775554, EPI_ISL_775555, EPI_ISL_775556, EPI_ISL_775557, EPI_ISL_775558, EPI_ISL_775559, EPI_ISL_775560, EPI_ISL_775561, EPI_ISL_775562, EPI_ISL_775564, EPI_ISL_775565, EPI_ISL_775566, EPI_ISL_775567, EPI_ISL_775568, EPI_ISL_775569, EPI_ISL_775570, EPI_ISL_775571			
see above	ABC	The Public Health Agency of Sweden	Department of Microbiology, The Public Health Agency of Sweden
EPI_ISL_775572, EPI_ISL_775573, EPI_ISL_775574	Klinisk mikrobiologi	The Public Health Agency of Sweden	Department of Microbiology, The Public Health Agency of Sweden
EPI_ISL_775575, EPI_ISL_775576, EPI_ISL_775577, EPI_ISL_775578, EPI_ISL_775579, EPI_ISL_775580, EPI_ISL_775581	ABC	The Public Health Agency of Sweden	Department of Microbiology, The Public Health Agency of Sweden
EPI_ISL_775583	Synlab Medilab, Mikrobiologi	The Public Health Agency of Sweden	Department of Microbiology, The Public Health Agency of Sweden
EPI_ISL_775585, EPI_ISL_775586	Unilabs AB	The Public Health Agency of Sweden	Department of Microbiology, The Public Health Agency of Sweden
EPI_ISL_775587	Synlab Medilab, Mikrobiologi	The Public Health Agency of Sweden	Department of Microbiology, The Public Health Agency of Sweden
EPI_ISL_775588	Klinisk mikrobiologi	The Public Health Agency of Sweden	Department of Microbiology, The Public Health Agency of Sweden
EPI_ISL_775589	Laboratorium for klinisk mikrobiologi	The Public Health Agency of Sweden	Department of Microbiology, The Public Health Agency of Sweden
EPI_ISL_775590, EPI_ISL_775591, EPI_ISL_775592	Klinisk mikrobiologi	The Public Health Agency of Sweden	Department of Microbiology, The Public Health Agency of Sweden
EPI_ISL_775593	TATAA Biocenter	The Public Health Agency of Sweden	Department of Microbiology, The Public Health Agency of Sweden
EPI_ISL_776592, EPI_ISL_776593, EPI_ISL_776594, EPI_ISL_776595, EPI_ISL_776598, EPI_ISL_776599, EPI_ISL_776600, EPI_ISL_776616, EPI_ISL_776621, EPI_ISL_776623, EPI_ISL_776624, EPI_ISL_776625, EPI_ISL_776628, EPI_ISL_776631, EPI_ISL_776632, EPI_ISL_776633, EPI_ISL_776639, EPI_ISL_776640, EPI_ISL_776642, EPI_ISL_776643, EPI_ISL_776645, EPI_ISL_776649			
see above	Israel Central Virology laboratory	Israel Central Virology laboratory	Neta Zuckerman, Efrat Dahan Bucris, Oran Erster, Michal Mandelboim, Orna Mor, Ella Mendelson
EPI_ISL_776768	Instituto Adolfo Lutz - Regional de Aracatuba	Instituto Adolfo Lutz, Interdiciplinary Procedures Center, Strategic Laboratory	Claudio Tavares Sacchi, Claudia Regina Gonçalves, Erica Valessa Ramos Gomes, Karoline Rodrigues Campos
EPI_ISL_776833, EPI_ISL_776837	Israel Central Virology laboratory	Israel Central Virology laboratory	Neta Zuckerman, Efrat Dahan Bucris, Oran Erster, Michal Mandelboim, Orna Mor, Ella Mendelson
EPI_ISL_777011	Lighthouse Lab in Alderley Park	Wellcome Sanger Institute for the COVID-19 Genomics UK (COG-UK) Consortium	Jacquelyn Wynn, Mairead Hyland, The Lighthouse Lab in Alderley Park and Alex Alderton, Roberto Amato, Sonia Goncalves, Ewan Harrison, David K. Jackson, Ian Johnston, Dominic Kwiatkowski, Cordelia Langford, John Sillitoe on behalf of the Wellcome Sanger Institute COVID-19 Surveillance Team
EPI_ISL_777013	Lighthouse Lab in Glasgow	Wellcome Sanger Institute for the COVID-19 Genomics UK (COG-UK) Consortium	Harper VanSteenhouse, Yumi Kasai, David Gray, Carol Clugston, Anna Dominiczak and Alex Alderton, Roberto Amato, Sonia Goncalves, Ewan Harrison, David K. Jackson, Ian Johnston, Dominic Kwiatkowski, Cordelia Langford, John Sillitoe on behalf of the Wellcome Sanger Institute COVID-19 Surveillance Team
EPI_ISL_777014	Lighthouse Lab in Alderley Park	Wellcome Sanger Institute for the COVID-19 Genomics UK (COG-UK) Consortium	Jacquelyn Wynn, Mairead Hyland, The Lighthouse Lab in Alderley Park and Alex Alderton, Roberto Amato, Sonia Goncalves, Ewan Harrison, David K. Jackson, Ian Johnston, Dominic Kwiatkowski, Cordelia Langford, John Sillitoe on behalf of the Wellcome Sanger Institute COVID-19 Surveillance Team
EPI_ISL_777016	Lighthouse Lab in Glasgow	Wellcome Sanger Institute for the COVID-19 Genomics UK (COG-UK) Consortium	Harper VanSteenhouse, Yumi Kasai, David Gray, Carol Clugston, Anna Dominiczak and Alex Alderton, Roberto Amato, Sonia Goncalves, Ewan Harrison, David K. Jackson, Ian Johnston, Dominic Kwiatkowski, Cordelia Langford, John Sillitoe on behalf of the Wellcome Sanger Institute COVID-19 Surveillance Team
EPI_ISL_777017, EPI_ISL_777018	Lighthouse Lab in Alderley Park	Wellcome Sanger Institute for the COVID-19 Genomics UK (COG-UK) Consortium	Jacquelyn Wynn, Mairead Hyland, The Lighthouse Lab in Alderley Park and Alex Alderton, Roberto Amato, Sonia Goncalves, Ewan Harrison, David K. Jackson, Ian Johnston, Dominic Kwiatkowski, Cordelia Langford, John Sillitoe on behalf of the Wellcome Sanger Institute COVID-19 Surveillance Team
EPI_ISL_777019	Lighthouse Lab in Glasgow	Wellcome Sanger Institute for the COVID-19 Genomics UK (COG-UK) Consortium	Harper VanSteenhouse, Yumi Kasai, David Gray, Carol Clugston, Anna Dominiczak and Alex Alderton, Roberto Amato, Sonia Goncalves, Ewan Harrison, David K. Jackson, Ian Johnston, Dominic Kwiatkowski, Cordelia Langford, John Sillitoe on behalf of the Wellcome Sanger Institute COVID-19 Surveillance

			Team
EPI_ISL_777020	Lighthouse Lab in Alderley Park	Wellcome Sanger Institute for the COVID-19 Genomics UK (COG-UK) Consortium	Jacquelyn Wynn, Mairead Hyland, The Lighthouse Lab in Alderley Park and Alex Alderton, Roberto Amato, Sonia Goncalves, Ewan Harrison, David K. Jackson, Ian Johnston, Dominic Kwiatkowski, Cordelia Langford, John Sillitoe on behalf of the Wellcome Sanger Institute COVID-19 Surveillance Team
EPI_ISL_777021, EPI_ISL_777023	Lighthouse Lab in Glasgow	Wellcome Sanger Institute for the COVID-19 Genomics UK (COG-UK) Consortium	Harper VanSteenhouse, Yumi Kasai, David Gray, Carol Clugston, Anna Dominiczak and Alex Alderton, Roberto Amato, Sonia Goncalves, Ewan Harrison, David K. Jackson, Ian Johnston, Dominic Kwiatkowski, Cordelia Langford, John Sillitoe on behalf of the Wellcome Sanger Institute COVID-19 Surveillance Team
EPI_ISL_777025, EPI_ISL_777026, EPI_ISL_777028, EPI_ISL_777030	Lighthouse Lab in Alderley Park	Wellcome Sanger Institute for the COVID-19 Genomics UK (COG-UK) Consortium	Jacquelyn Wynn, Mairead Hyland, The Lighthouse Lab in Alderley Park and Alex Alderton, Roberto Amato, Sonia Goncalves, Ewan Harrison, David K. Jackson, Ian Johnston, Dominic Kwiatkowski, Cordelia Langford, John Sillitoe on behalf of the Wellcome Sanger Institute COVID-19 Surveillance Team
EPI_ISL_777031	Lighthouse Lab in Glasgow	Wellcome Sanger Institute for the COVID-19 Genomics UK (COG-UK) Consortium	Harper VanSteenhouse, Yumi Kasai, David Gray, Carol Clugston, Anna Dominiczak and Alex Alderton, Roberto Amato, Sonia Goncalves, Ewan Harrison, David K. Jackson, Ian Johnston, Dominic Kwiatkowski, Cordelia Langford, John Sillitoe on behalf of the Wellcome Sanger Institute COVID-19 Surveillance Team
EPI_ISL_777033	Lighthouse Lab in Alderley Park	Wellcome Sanger Institute for the COVID-19 Genomics UK (COG-UK) Consortium	Jacquelyn Wynn, Mairead Hyland, The Lighthouse Lab in Alderley Park and Alex Alderton, Roberto Amato, Sonia Goncalves, Ewan Harrison, David K. Jackson, Ian Johnston, Dominic Kwiatkowski, Cordelia Langford, John Sillitoe on behalf of the Wellcome Sanger Institute COVID-19 Surveillance Team
EPI_ISL_777034	Lighthouse Lab in Glasgow	Wellcome Sanger Institute for the COVID-19 Genomics UK (COG-UK) Consortium	Harper VanSteenhouse, Yumi Kasai, David Gray, Carol Clugston, Anna Dominiczak and Alex Alderton, Roberto Amato, Sonia Goncalves, Ewan Harrison, David K. Jackson, Ian Johnston, Dominic Kwiatkowski, Cordelia Langford, John Sillitoe on behalf of the Wellcome Sanger Institute COVID-19 Surveillance Team
EPI_ISL_777035, EPI_ISL_777036	Lighthouse Lab in Alderley Park	Wellcome Sanger Institute for the COVID-19 Genomics UK (COG-UK) Consortium	Jacquelyn Wynn, Mairead Hyland, The Lighthouse Lab in Alderley Park and Alex Alderton, Roberto Amato, Sonia Goncalves, Ewan Harrison, David K. Jackson, Ian Johnston, Dominic Kwiatkowski, Cordelia Langford, John Sillitoe on behalf of the Wellcome Sanger Institute COVID-19 Surveillance Team
EPI_ISL_777039	Lighthouse Lab in Milton Keynes	Wellcome Sanger Institute for the COVID-19 Genomics UK (COG-UK) Consortium	The Lighthouse Lab in Milton Keynes and Alex Alderton, Roberto Amato, Sonia Goncalves, Ewan Harrison, David K. Jackson, Ian Johnston, Dominic Kwiatkowski, Cordelia Langford, John Sillitoe on behalf of the Wellcome Sanger Institute COVID-19 Surveillance Team
EPI_ISL_777043, EPI_ISL_777045	Lighthouse Lab in Alderley Park	Wellcome Sanger Institute for the COVID-19 Genomics UK (COG-UK) Consortium	Jacquelyn Wynn, Mairead Hyland, The Lighthouse Lab in Alderley Park and Alex Alderton, Roberto Amato, Sonia Goncalves, Ewan Harrison, David K. Jackson, Ian Johnston, Dominic Kwiatkowski, Cordelia Langford, John Sillitoe on behalf of the Wellcome Sanger Institute COVID-19 Surveillance Team
EPI_ISL_777047	Lighthouse Lab in Glasgow	Wellcome Sanger Institute for the COVID-19 Genomics UK (COG-UK) Consortium	Harper VanSteenhouse, Yumi Kasai, David Gray, Carol Clugston, Anna Dominiczak and Alex Alderton, Roberto Amato, Sonia Goncalves, Ewan Harrison, David K. Jackson, Ian Johnston, Dominic Kwiatkowski, Cordelia Langford, John Sillitoe on behalf of the Wellcome Sanger Institute COVID-19 Surveillance Team
EPI_ISL_777048, EPI_ISL_777050, EPI_ISL_777051, EPI_ISL_777052, EPI_ISL_777053	Lighthouse Lab in Alderley Park	Wellcome Sanger Institute for the COVID-19 Genomics UK (COG-UK) Consortium	Jacquelyn Wynn, Mairead Hyland, The Lighthouse Lab in Alderley Park and Alex Alderton, Roberto Amato, Sonia Goncalves, Ewan Harrison, David K. Jackson, Ian Johnston, Dominic Kwiatkowski, Cordelia Langford, John Sillitoe on behalf of the Wellcome Sanger Institute COVID-19 Surveillance Team
EPI_ISL_777055	Lighthouse Lab in Glasgow	Wellcome Sanger Institute for the COVID-19 Genomics UK (COG-UK) Consortium	Harper VanSteenhouse, Yumi Kasai, David Gray, Carol Clugston, Anna Dominiczak and Alex Alderton, Roberto Amato, Sonia Goncalves, Ewan Harrison, David K. Jackson, Ian Johnston, Dominic Kwiatkowski, Cordelia Langford, John Sillitoe on behalf of the Wellcome Sanger Institute COVID-19 Surveillance Team
EPI_ISL_777057	Lighthouse Lab in Alderley Park	Wellcome Sanger Institute for the COVID-19 Genomics UK (COG-UK) Consortium	Jacquelyn Wynn, Mairead Hyland, The Lighthouse Lab in Alderley Park and Alex Alderton, Roberto Amato, Sonia Goncalves, Ewan Harrison, David K. Jackson, Ian Johnston, Dominic Kwiatkowski, Cordelia Langford, John Sillitoe on behalf of the Wellcome Sanger Institute COVID-19 Surveillance Team
EPI_ISL_777058, EPI_ISL_777059	Lighthouse Lab in Glasgow	Wellcome Sanger Institute for the COVID-19 Genomics UK (COG-UK) Consortium	Harper VanSteenhouse, Yumi Kasai, David Gray, Carol Clugston, Anna Dominiczak and Alex Alderton, Roberto Amato, Sonia Goncalves, Ewan Harrison, David K. Jackson, Ian Johnston, Dominic Kwiatkowski, Cordelia Langford, John Sillitoe on behalf of the Wellcome Sanger Institute COVID-19 Surveillance Team
EPI_ISL_777060	Lighthouse Lab in Alderley Park	Wellcome Sanger Institute for the COVID-19 Genomics UK (COG-UK) Consortium	Jacquelyn Wynn, Mairead Hyland, The Lighthouse Lab in Alderley Park and Alex Alderton, Roberto Amato, Sonia Goncalves, Ewan Harrison, David K. Jackson, Ian Johnston, Dominic Kwiatkowski, Cordelia Langford, John Sillitoe on behalf of the Wellcome Sanger Institute COVID-19 Surveillance Team
EPI_ISL_777061, EPI_ISL_777062	Lighthouse Lab in Glasgow	Wellcome Sanger Institute for the COVID-19 Genomics UK (COG-UK) Consortium	Harper VanSteenhouse, Yumi Kasai, David Gray, Carol Clugston, Anna Dominiczak and Alex Alderton, Roberto Amato, Sonia Goncalves, Ewan Harrison, David K. Jackson, Ian Johnston, Dominic Kwiatkowski, Cordelia Langford, John Sillitoe on behalf of the Wellcome Sanger Institute COVID-19 Surveillance Team
EPI_ISL_777063	Lighthouse Lab in Alderley Park	Wellcome Sanger Institute for the COVID-19 Genomics UK (COG-UK) Consortium	Jacquelyn Wynn, Mairead Hyland, The Lighthouse Lab in Alderley Park and Alex Alderton, Roberto Amato, Sonia Goncalves, Ewan Harrison, David K. Jackson, Ian Johnston, Dominic Kwiatkowski, Cordelia Langford, John Sillitoe on behalf of the Wellcome Sanger Institute COVID-19 Surveillance Team
EPI_ISL_777064	Lighthouse Lab in Milton Keynes	Wellcome Sanger Institute for the COVID-19 Genomics UK (COG-UK) Consortium	The Lighthouse Lab in Milton Keynes and Alex Alderton, Roberto Amato, Sonia Goncalves, Ewan Harrison, David K. Jackson, Ian Johnston, Dominic Kwiatkowski, Cordelia Langford, John Sillitoe on behalf of the Wellcome Sanger Institute COVID-19 Surveillance Team
EPI_ISL_777065, EPI_ISL_777066	Lighthouse Lab in Glasgow	Wellcome Sanger Institute for the COVID-19 Genomics UK (COG-UK) Consortium	Harper VanSteenhouse, Yumi Kasai, David Gray, Carol Clugston, Anna Dominiczak and Alex Alderton, Roberto Amato, Sonia Goncalves, Ewan Harrison, David K. Jackson, Ian Johnston, Dominic Kwiatkowski, Cordelia Langford, John Sillitoe on behalf of the Wellcome Sanger Institute COVID-19 Surveillance Team
EPI_ISL_777067	Lighthouse Lab in Alderley Park	Wellcome Sanger Institute for the COVID-19 Genomics UK (COG-UK) Consortium	Jacquelyn Wynn, Mairead Hyland, The Lighthouse Lab in Alderley Park and Alex Alderton, Roberto Amato, Sonia Goncalves, Ewan Harrison, David K. Jackson, Ian Johnston, Dominic Kwiatkowski, Cordelia Langford, John Sillitoe on behalf of the Wellcome Sanger Institute COVID-19 Surveillance Team
EPI_ISL_777068	Lighthouse Lab in Glasgow	Wellcome Sanger Institute for the COVID-19 Genomics UK (COG-UK) Consortium	Harper VanSteenhouse, Yumi Kasai, David Gray, Carol Clugston, Anna Dominiczak and Alex Alderton, Roberto Amato, Sonia Goncalves, Ewan Harrison, David K. Jackson, Ian Johnston, Dominic Kwiatkowski, Cordelia Langford, John Sillitoe on behalf of the Wellcome Sanger Institute COVID-19 Surveillance Team
EPI_ISL_777069, EPI_ISL_777070, EPI_ISL_777071, EPI_ISL_777072	Lighthouse Lab in Alderley Park	Wellcome Sanger Institute for the COVID-19 Genomics UK (COG-UK) Consortium	Jacquelyn Wynn, Mairead Hyland, The Lighthouse Lab in Alderley Park and Alex Alderton, Roberto Amato, Sonia Goncalves, Ewan Harrison, David K. Jackson, Ian Johnston, Dominic Kwiatkowski, Cordelia Langford, John Sillitoe on behalf of the Wellcome Sanger Institute COVID-19 Surveillance Team
EPI_ISL_777073	Lighthouse Lab in Glasgow	Wellcome Sanger Institute for the COVID-19 Genomics UK (COG-UK) Consortium	Harper VanSteenhouse, Yumi Kasai, David Gray, Carol Clugston, Anna Dominiczak and Alex Alderton, Roberto Amato, Sonia Goncalves, Ewan Harrison, David K. Jackson, Ian Johnston, Dominic Kwiatkowski, Cordelia Langford, John Sillitoe on behalf of the Wellcome Sanger Institute COVID-19 Surveillance Team
EPI_ISL_777074, EPI_ISL_777075, EPI_ISL_777076, EPI_ISL_777077, EPI_ISL_777078, EPI_ISL_777079	Lighthouse Lab in Alderley Park	Wellcome Sanger Institute for the COVID-19 Genomics UK (COG-UK) Consortium	Jacquelyn Wynn, Mairead Hyland, The Lighthouse Lab in Alderley Park and Alex Alderton, Roberto Amato, Sonia Goncalves, Ewan Harrison, David K. Jackson, Ian Johnston, Dominic Kwiatkowski, Cordelia Langford, John Sillitoe on behalf of the Wellcome Sanger Institute COVID-19 Surveillance Team
EPI_ISL_777080	Lighthouse Lab in Milton Keynes	Wellcome Sanger Institute for the COVID-19 Genomics UK (COG-UK) Consortium	The Lighthouse Lab in Milton Keynes and Alex Alderton, Roberto Amato, Sonia Goncalves, Ewan Harrison, David K. Jackson, Ian Johnston, Dominic Kwiatkowski, Cordelia Langford, John Sillitoe on behalf of the Wellcome Sanger Institute COVID-19 Surveillance Team
EPI_ISL_777081	Lighthouse Lab in Glasgow	Wellcome Sanger Institute for the COVID-19 Genomics UK (COG-UK) Consortium	Harper VanSteenhouse, Yumi Kasai, David Gray, Carol Clugston, Anna Dominiczak and Alex Alderton, Roberto Amato, Sonia Goncalves, Ewan Harrison, David K. Jackson, Ian Johnston, Dominic Kwiatkowski, Cordelia Langford, John Sillitoe on behalf of the Wellcome Sanger Institute COVID-19 Surveillance Team
EPI_ISL_777083	Lighthouse Lab in Alderley Park	Wellcome Sanger Institute for the COVID-19 Genomics UK (COG-UK) Consortium	Jacquelyn Wynn, Mairead Hyland, The Lighthouse Lab in Alderley Park and Alex Alderton, Roberto Amato, Sonia Goncalves, Ewan Harrison, David K. Jackson, Ian Johnston, Dominic Kwiatkowski, Cordelia Langford, John Sillitoe on behalf of the Wellcome Sanger Institute COVID-19 Surveillance Team
EPI_ISL_777084, EPI_ISL_777085	Lighthouse Lab in Milton Keynes	Wellcome Sanger Institute for the COVID-19 Genomics UK (COG-UK) Consortium	The Lighthouse Lab in Milton Keynes and Alex Alderton, Roberto Amato, Sonia Goncalves, Ewan Harrison, David K. Jackson, Ian Johnston, Dominic Kwiatkowski, Cordelia Langford, John Sillitoe on behalf of the Wellcome Sanger Institute COVID-19 Surveillance Team
EPI_ISL_777087, EPI_ISL_777088	Lighthouse Lab in Alderley Park	Wellcome Sanger Institute for the COVID-19 Genomics UK (COG-UK) Consortium	Jacquelyn Wynn, Mairead Hyland, The Lighthouse Lab in Alderley Park and Alex Alderton, Roberto Amato, Sonia Goncalves, Ewan Harrison, David K. Jackson, Ian Johnston, Dominic Kwiatkowski, Cordelia Langford, John Sillitoe on behalf of the Wellcome Sanger Institute COVID-19 Surveillance Team
EPI_ISL_777089	Lighthouse Lab in Glasgow	Wellcome Sanger Institute for the COVID-19 Genomics UK	Harper VanSteenhouse, Yumi Kasai, David Gray, Carol Clugston, Anna Dominiczak and Alex Alderton, Roberto Amato, Sonia Goncalves, Ewan Harrison,

		(COG-UK) Consortium	David K. Jackson, Ian Johnston, Dominic Kwiatkowski, Cordelia Langford, John Sillitoe on behalf of the Wellcome Sanger Institute COVID-19 Surveillance Team
EPI_ISL_777092, EPI_ISL_777093, EPI_ISL_777094, EPI_ISL_777096	Lighthouse Lab in Alderley Park	Wellcome Sanger Institute for the COVID-19 Genomics UK (COG-UK) Consortium	Jacquelyn Wynn, Mairead Hyland, The Lighthouse Lab in Alderley Park and Alex Alderton, Roberto Amato, Sonia Goncalves, Ewan Harrison, David K. Jackson, Ian Johnston, Dominic Kwiatkowski, Cordelia Langford, John Sillitoe on behalf of the Wellcome Sanger Institute COVID-19 Surveillance Team
EPI_ISL_777098	Lighthouse Lab in Glasgow	Wellcome Sanger Institute for the COVID-19 Genomics UK (COG-UK) Consortium	Harper VanSteenhouse, Yumi Kasai, David Gray, Carol Clugston, Anna Dominiczak and Alex Alderton, Roberto Amato, Sonia Goncalves, Ewan Harrison, David K. Jackson, Ian Johnston, Dominic Kwiatkowski, Cordelia Langford, John Sillitoe on behalf of the Wellcome Sanger Institute COVID-19 Surveillance Team
EPI_ISL_777102, EPI_ISL_777104	Lighthouse Lab in Milton Keynes	Wellcome Sanger Institute for the COVID-19 Genomics UK (COG-UK) Consortium	The Lighthouse Lab in Milton Keynes and Alex Alderton, Roberto Amato, Sonia Goncalves, Ewan Harrison, David K. Jackson, Ian Johnston, Dominic Kwiatkowski, Cordelia Langford, John Sillitoe on behalf of the Wellcome Sanger Institute COVID-19 Surveillance Team
EPI_ISL_777109, EPI_ISL_777110, EPI_ISL_777111	Lighthouse Lab in Alderley Park	Wellcome Sanger Institute for the COVID-19 Genomics UK (COG-UK) Consortium	Jacquelyn Wynn, Mairead Hyland, The Lighthouse Lab in Alderley Park and Alex Alderton, Roberto Amato, Sonia Goncalves, Ewan Harrison, David K. Jackson, Ian Johnston, Dominic Kwiatkowski, Cordelia Langford, John Sillitoe on behalf of the Wellcome Sanger Institute COVID-19 Surveillance Team
EPI_ISL_777112	Lighthouse Lab in Glasgow	Wellcome Sanger Institute for the COVID-19 Genomics UK (COG-UK) Consortium	Harper VanSteenhouse, Yumi Kasai, David Gray, Carol Clugston, Anna Dominiczak and Alex Alderton, Roberto Amato, Sonia Goncalves, Ewan Harrison, David K. Jackson, Ian Johnston, Dominic Kwiatkowski, Cordelia Langford, John Sillitoe on behalf of the Wellcome Sanger Institute COVID-19 Surveillance Team
EPI_ISL_777113, EPI_ISL_777114	Lighthouse Lab in Alderley Park	Wellcome Sanger Institute for the COVID-19 Genomics UK (COG-UK) Consortium	Jacquelyn Wynn, Mairead Hyland, The Lighthouse Lab in Alderley Park and Alex Alderton, Roberto Amato, Sonia Goncalves, Ewan Harrison, David K. Jackson, Ian Johnston, Dominic Kwiatkowski, Cordelia Langford, John Sillitoe on behalf of the Wellcome Sanger Institute COVID-19 Surveillance Team
EPI_ISL_777115	Lighthouse Lab in Glasgow	Wellcome Sanger Institute for the COVID-19 Genomics UK (COG-UK) Consortium	Harper VanSteenhouse, Yumi Kasai, David Gray, Carol Clugston, Anna Dominiczak and Alex Alderton, Roberto Amato, Sonia Goncalves, Ewan Harrison, David K. Jackson, Ian Johnston, Dominic Kwiatkowski, Cordelia Langford, John Sillitoe on behalf of the Wellcome Sanger Institute COVID-19 Surveillance Team
EPI_ISL_777116	Lighthouse Lab in Alderley Park	Wellcome Sanger Institute for the COVID-19 Genomics UK (COG-UK) Consortium	Jacquelyn Wynn, Mairead Hyland, The Lighthouse Lab in Alderley Park and Alex Alderton, Roberto Amato, Sonia Goncalves, Ewan Harrison, David K. Jackson, Ian Johnston, Dominic Kwiatkowski, Cordelia Langford, John Sillitoe on behalf of the Wellcome Sanger Institute COVID-19 Surveillance Team
EPI_ISL_777117, EPI_ISL_777120, EPI_ISL_777121, EPI_ISL_777122	Lighthouse Lab in Glasgow	Wellcome Sanger Institute for the COVID-19 Genomics UK (COG-UK) Consortium	Harper VanSteenhouse, Yumi Kasai, David Gray, Carol Clugston, Anna Dominiczak and Alex Alderton, Roberto Amato, Sonia Goncalves, Ewan Harrison, David K. Jackson, Ian Johnston, Dominic Kwiatkowski, Cordelia Langford, John Sillitoe on behalf of the Wellcome Sanger Institute COVID-19 Surveillance Team
EPI_ISL_777123, EPI_ISL_777125, EPI_ISL_777126	Lighthouse Lab in Alderley Park	Wellcome Sanger Institute for the COVID-19 Genomics UK (COG-UK) Consortium	Jacquelyn Wynn, Mairead Hyland, The Lighthouse Lab in Alderley Park and Alex Alderton, Roberto Amato, Sonia Goncalves, Ewan Harrison, David K. Jackson, Ian Johnston, Dominic Kwiatkowski, Cordelia Langford, John Sillitoe on behalf of the Wellcome Sanger Institute COVID-19 Surveillance Team
EPI_ISL_777127	Lighthouse Lab in Glasgow	Wellcome Sanger Institute for the COVID-19 Genomics UK (COG-UK) Consortium	Harper VanSteenhouse, Yumi Kasai, David Gray, Carol Clugston, Anna Dominiczak and Alex Alderton, Roberto Amato, Sonia Goncalves, Ewan Harrison, David K. Jackson, Ian Johnston, Dominic Kwiatkowski, Cordelia Langford, John Sillitoe on behalf of the Wellcome Sanger Institute COVID-19 Surveillance Team
EPI_ISL_777129	Lighthouse Lab in Alderley Park	Wellcome Sanger Institute for the COVID-19 Genomics UK (COG-UK) Consortium	Jacquelyn Wynn, Mairead Hyland, The Lighthouse Lab in Alderley Park and Alex Alderton, Roberto Amato, Sonia Goncalves, Ewan Harrison, David K. Jackson, Ian Johnston, Dominic Kwiatkowski, Cordelia Langford, John Sillitoe on behalf of the Wellcome Sanger Institute COVID-19 Surveillance Team
EPI_ISL_777131	Lighthouse Lab in Glasgow	Wellcome Sanger Institute for the COVID-19 Genomics UK (COG-UK) Consortium	Harper VanSteenhouse, Yumi Kasai, David Gray, Carol Clugston, Anna Dominiczak and Alex Alderton, Roberto Amato, Sonia Goncalves, Ewan Harrison, David K. Jackson, Ian Johnston, Dominic Kwiatkowski, Cordelia Langford, John Sillitoe on behalf of the Wellcome Sanger Institute COVID-19 Surveillance Team
EPI_ISL_777133	Lighthouse Lab in Cambridge	Wellcome Sanger Institute for the COVID-19 Genomics UK (COG-UK) Consortium	Rob Howes, The Lighthouse Lab in Cambridge and Alex Alderton, Roberto Amato, Sonia Goncalves, Ewan Harrison, David K. Jackson, Ian Johnston, Dominic Kwiatkowski, Cordelia Langford, John Sillitoe on behalf of the Wellcome Sanger Institute COVID-19 Surveillance Team
EPI_ISL_777134, EPI_ISL_777136, EPI_ISL_777137, EPI_ISL_777138, EPI_ISL_777139	Lighthouse Lab in Alderley Park	Wellcome Sanger Institute for the COVID-19 Genomics UK (COG-UK) Consortium	Jacquelyn Wynn, Mairead Hyland, The Lighthouse Lab in Alderley Park and Alex Alderton, Roberto Amato, Sonia Goncalves, Ewan Harrison, David K. Jackson, Ian Johnston, Dominic Kwiatkowski, Cordelia Langford, John Sillitoe on behalf of the Wellcome Sanger Institute COVID-19 Surveillance Team
EPI_ISL_777140, EPI_ISL_777142	Lighthouse Lab in Glasgow	Wellcome Sanger Institute for the COVID-19 Genomics UK (COG-UK) Consortium	Harper VanSteenhouse, Yumi Kasai, David Gray, Carol Clugston, Anna Dominiczak and Alex Alderton, Roberto Amato, Sonia Goncalves, Ewan Harrison, David K. Jackson, Ian Johnston, Dominic Kwiatkowski, Cordelia Langford, John Sillitoe on behalf of the Wellcome Sanger Institute COVID-19 Surveillance Team
EPI_ISL_777143, EPI_ISL_777144, EPI_ISL_777147	Lighthouse Lab in Alderley Park	Wellcome Sanger Institute for the COVID-19 Genomics UK (COG-UK) Consortium	Jacquelyn Wynn, Mairead Hyland, The Lighthouse Lab in Alderley Park and Alex Alderton, Roberto Amato, Sonia Goncalves, Ewan Harrison, David K. Jackson, Ian Johnston, Dominic Kwiatkowski, Cordelia Langford, John Sillitoe on behalf of the Wellcome Sanger Institute COVID-19 Surveillance Team
EPI_ISL_777148, EPI_ISL_777149	Lighthouse Lab in Glasgow	Wellcome Sanger Institute for the COVID-19 Genomics UK (COG-UK) Consortium	Harper VanSteenhouse, Yumi Kasai, David Gray, Carol Clugston, Anna Dominiczak and Alex Alderton, Roberto Amato, Sonia Goncalves, Ewan Harrison, David K. Jackson, Ian Johnston, Dominic Kwiatkowski, Cordelia Langford, John Sillitoe on behalf of the Wellcome Sanger Institute COVID-19 Surveillance Team
EPI_ISL_777150	Lighthouse Lab in Alderley Park	Wellcome Sanger Institute for the COVID-19 Genomics UK (COG-UK) Consortium	Jacquelyn Wynn, Mairead Hyland, The Lighthouse Lab in Alderley Park and Alex Alderton, Roberto Amato, Sonia Goncalves, Ewan Harrison, David K. Jackson, Ian Johnston, Dominic Kwiatkowski, Cordelia Langford, John Sillitoe on behalf of the Wellcome Sanger Institute COVID-19 Surveillance Team
EPI_ISL_777151, EPI_ISL_777152, EPI_ISL_777154	Lighthouse Lab in Glasgow	Wellcome Sanger Institute for the COVID-19 Genomics UK (COG-UK) Consortium	Harper VanSteenhouse, Yumi Kasai, David Gray, Carol Clugston, Anna Dominiczak and Alex Alderton, Roberto Amato, Sonia Goncalves, Ewan Harrison, David K. Jackson, Ian Johnston, Dominic Kwiatkowski, Cordelia Langford, John Sillitoe on behalf of the Wellcome Sanger Institute COVID-19 Surveillance Team
EPI_ISL_777157	Lighthouse Lab in Alderley Park	Wellcome Sanger Institute for the COVID-19 Genomics UK (COG-UK) Consortium	Jacquelyn Wynn, Mairead Hyland, The Lighthouse Lab in Alderley Park and Alex Alderton, Roberto Amato, Sonia Goncalves, Ewan Harrison, David K. Jackson, Ian Johnston, Dominic Kwiatkowski, Cordelia Langford, John Sillitoe on behalf of the Wellcome Sanger Institute COVID-19 Surveillance Team
EPI_ISL_777158	Lighthouse Lab in Milton Keynes	Wellcome Sanger Institute for the COVID-19 Genomics UK (COG-UK) Consortium	The Lighthouse Lab in Milton Keynes and Alex Alderton, Roberto Amato, Sonia Goncalves, Ewan Harrison, David K. Jackson, Ian Johnston, Dominic Kwiatkowski, Cordelia Langford, John Sillitoe on behalf of the Wellcome Sanger Institute COVID-19 Surveillance Team
EPI_ISL_777159	Lighthouse Lab in Alderley Park	Wellcome Sanger Institute for the COVID-19 Genomics UK (COG-UK) Consortium	Jacquelyn Wynn, Mairead Hyland, The Lighthouse Lab in Alderley Park and Alex Alderton, Roberto Amato, Sonia Goncalves, Ewan Harrison, David K. Jackson, Ian Johnston, Dominic Kwiatkowski, Cordelia Langford, John Sillitoe on behalf of the Wellcome Sanger Institute COVID-19 Surveillance Team
EPI_ISL_777160, EPI_ISL_777161, EPI_ISL_777162	Lighthouse Lab in Glasgow	Wellcome Sanger Institute for the COVID-19 Genomics UK (COG-UK) Consortium	Harper VanSteenhouse, Yumi Kasai, David Gray, Carol Clugston, Anna Dominiczak and Alex Alderton, Roberto Amato, Sonia Goncalves, Ewan Harrison, David K. Jackson, Ian Johnston, Dominic Kwiatkowski, Cordelia Langford, John Sillitoe on behalf of the Wellcome Sanger Institute COVID-19 Surveillance Team
EPI_ISL_777163	Lighthouse Lab in Alderley Park	Wellcome Sanger Institute for the COVID-19 Genomics UK (COG-UK) Consortium	Jacquelyn Wynn, Mairead Hyland, The Lighthouse Lab in Alderley Park and Alex Alderton, Roberto Amato, Sonia Goncalves, Ewan Harrison, David K. Jackson, Ian Johnston, Dominic Kwiatkowski, Cordelia Langford, John Sillitoe on behalf of the Wellcome Sanger Institute COVID-19 Surveillance Team
EPI_ISL_777164	Lighthouse Lab in Glasgow	Wellcome Sanger Institute for the COVID-19 Genomics UK (COG-UK) Consortium	Harper VanSteenhouse, Yumi Kasai, David Gray, Carol Clugston, Anna Dominiczak and Alex Alderton, Roberto Amato, Sonia Goncalves, Ewan Harrison, David K. Jackson, Ian Johnston, Dominic Kwiatkowski, Cordelia Langford, John Sillitoe on behalf of the Wellcome Sanger Institute COVID-19 Surveillance Team
EPI_ISL_777166, EPI_ISL_777167, EPI_ISL_777168, EPI_ISL_777170, EPI_ISL_777171	Lighthouse Lab in Alderley Park	Wellcome Sanger Institute for the COVID-19 Genomics UK (COG-UK) Consortium	Jacquelyn Wynn, Mairead Hyland, The Lighthouse Lab in Alderley Park and Alex Alderton, Roberto Amato, Sonia Goncalves, Ewan Harrison, David K. Jackson, Ian Johnston, Dominic Kwiatkowski, Cordelia Langford, John Sillitoe on behalf of the Wellcome Sanger Institute COVID-19 Surveillance Team
EPI_ISL_777173	Lighthouse Lab in Cambridge	Wellcome Sanger Institute for the COVID-19 Genomics UK (COG-UK) Consortium	Rob Howes, The Lighthouse Lab in Cambridge and Alex Alderton, Roberto Amato, Sonia Goncalves, Ewan Harrison, David K. Jackson, Ian Johnston, Dominic Kwiatkowski, Cordelia Langford, John Sillitoe on behalf of the Wellcome Sanger Institute COVID-19 Surveillance Team
EPI_ISL_777174, EPI_ISL_777175	Lighthouse Lab in Alderley Park	Wellcome Sanger Institute for the COVID-19 Genomics UK (COG-UK) Consortium	Jacquelyn Wynn, Mairead Hyland, The Lighthouse Lab in Alderley Park and Alex Alderton, Roberto Amato, Sonia Goncalves, Ewan Harrison, David K. Jackson, Ian Johnston, Dominic Kwiatkowski, Cordelia Langford, John Sillitoe on behalf of the Wellcome Sanger Institute COVID-19 Surveillance Team

EPI_ISL_777176	Lighthouse Lab in Glasgow	Wellcome Sanger Institute for the COVID-19 Genomics UK (COG-UK) Consortium	Harper VanSteenhouse, Yumi Kasai, David Gray, Carol Clugston, Anna Dominiczak and Alex Alderton, Roberto Amato, Sonia Goncalves, Ewan Harrison, David K. Jackson, Ian Johnston, Dominic Kwiatkowski, Cordelia Langford, John Sillitoe on behalf of the Wellcome Sanger Institute COVID-19 Surveillance Team
EPI_ISL_777177, EPI_ISL_777178, EPI_ISL_777179, EPI_ISL_777183	Lighthouse Lab in Alderley Park	Wellcome Sanger Institute for the COVID-19 Genomics UK (COG-UK) Consortium	Jacquelyn Wynn, Mairead Hyland, The Lighthouse Lab in Alderley Park and Alex Alderton, Roberto Amato, Sonia Goncalves, Ewan Harrison, David K. Jackson, Ian Johnston, Dominic Kwiatkowski, Cordelia Langford, John Sillitoe on behalf of the Wellcome Sanger Institute COVID-19 Surveillance Team
EPI_ISL_777184, EPI_ISL_777187	Lighthouse Lab in Milton Keynes	Wellcome Sanger Institute for the COVID-19 Genomics UK (COG-UK) Consortium	The Lighthouse Lab in Milton Keynes and Alex Alderton, Roberto Amato, Sonia Goncalves, Ewan Harrison, David K. Jackson, Ian Johnston, Dominic Kwiatkowski, Cordelia Langford, John Sillitoe on behalf of the Wellcome Sanger Institute COVID-19 Surveillance Team
EPI_ISL_777188	Lighthouse Lab in Alderley Park	Wellcome Sanger Institute for the COVID-19 Genomics UK (COG-UK) Consortium	Jacquelyn Wynn, Mairead Hyland, The Lighthouse Lab in Alderley Park and Alex Alderton, Roberto Amato, Sonia Goncalves, Ewan Harrison, David K. Jackson, Ian Johnston, Dominic Kwiatkowski, Cordelia Langford, John Sillitoe on behalf of the Wellcome Sanger Institute COVID-19 Surveillance Team
EPI_ISL_777189, EPI_ISL_777191	Lighthouse Lab in Glasgow	Wellcome Sanger Institute for the COVID-19 Genomics UK (COG-UK) Consortium	Harper VanSteenhouse, Yumi Kasai, David Gray, Carol Clugston, Anna Dominiczak and Alex Alderton, Roberto Amato, Sonia Goncalves, Ewan Harrison, David K. Jackson, Ian Johnston, Dominic Kwiatkowski, Cordelia Langford, John Sillitoe on behalf of the Wellcome Sanger Institute COVID-19 Surveillance Team
EPI_ISL_777193, EPI_ISL_777195	Lighthouse Lab in Alderley Park	Wellcome Sanger Institute for the COVID-19 Genomics UK (COG-UK) Consortium	Jacquelyn Wynn, Mairead Hyland, The Lighthouse Lab in Alderley Park and Alex Alderton, Roberto Amato, Sonia Goncalves, Ewan Harrison, David K. Jackson, Ian Johnston, Dominic Kwiatkowski, Cordelia Langford, John Sillitoe on behalf of the Wellcome Sanger Institute COVID-19 Surveillance Team
EPI_ISL_777196	Lighthouse Lab in Glasgow	Wellcome Sanger Institute for the COVID-19 Genomics UK (COG-UK) Consortium	Harper VanSteenhouse, Yumi Kasai, David Gray, Carol Clugston, Anna Dominiczak and Alex Alderton, Roberto Amato, Sonia Goncalves, Ewan Harrison, David K. Jackson, Ian Johnston, Dominic Kwiatkowski, Cordelia Langford, John Sillitoe on behalf of the Wellcome Sanger Institute COVID-19 Surveillance Team
EPI_ISL_777197, EPI_ISL_777198	Lighthouse Lab in Alderley Park	Wellcome Sanger Institute for the COVID-19 Genomics UK (COG-UK) Consortium	Jacquelyn Wynn, Mairead Hyland, The Lighthouse Lab in Alderley Park and Alex Alderton, Roberto Amato, Sonia Goncalves, Ewan Harrison, David K. Jackson, Ian Johnston, Dominic Kwiatkowski, Cordelia Langford, John Sillitoe on behalf of the Wellcome Sanger Institute COVID-19 Surveillance Team
EPI_ISL_777200	Lighthouse Lab in Milton Keynes	Wellcome Sanger Institute for the COVID-19 Genomics UK (COG-UK) Consortium	The Lighthouse Lab in Milton Keynes and Alex Alderton, Roberto Amato, Sonia Goncalves, Ewan Harrison, David K. Jackson, Ian Johnston, Dominic Kwiatkowski, Cordelia Langford, John Sillitoe on behalf of the Wellcome Sanger Institute COVID-19 Surveillance Team
EPI_ISL_777201	Lighthouse Lab in Cambridge	Wellcome Sanger Institute for the COVID-19 Genomics UK (COG-UK) Consortium	Rob Howes, The Lighthouse Lab in Cambridge and Alex Alderton, Roberto Amato, Sonia Goncalves, Ewan Harrison, David K. Jackson, Ian Johnston, Dominic Kwiatkowski, Cordelia Langford, John Sillitoe on behalf of the Wellcome Sanger Institute COVID-19 Surveillance Team
EPI_ISL_777204	Lighthouse Lab in Alderley Park	Wellcome Sanger Institute for the COVID-19 Genomics UK (COG-UK) Consortium	Jacquelyn Wynn, Mairead Hyland, The Lighthouse Lab in Alderley Park and Alex Alderton, Roberto Amato, Sonia Goncalves, Ewan Harrison, David K. Jackson, Ian Johnston, Dominic Kwiatkowski, Cordelia Langford, John Sillitoe on behalf of the Wellcome Sanger Institute COVID-19 Surveillance Team
EPI_ISL_777205, EPI_ISL_777209, EPI_ISL_777215	Lighthouse Lab in Glasgow	Wellcome Sanger Institute for the COVID-19 Genomics UK (COG-UK) Consortium	Harper VanSteenhouse, Yumi Kasai, David Gray, Carol Clugston, Anna Dominiczak and Alex Alderton, Roberto Amato, Sonia Goncalves, Ewan Harrison, David K. Jackson, Ian Johnston, Dominic Kwiatkowski, Cordelia Langford, John Sillitoe on behalf of the Wellcome Sanger Institute COVID-19 Surveillance Team
EPI_ISL_777217	Lighthouse Lab in Alderley Park	Wellcome Sanger Institute for the COVID-19 Genomics UK (COG-UK) Consortium	Jacquelyn Wynn, Mairead Hyland, The Lighthouse Lab in Alderley Park and Alex Alderton, Roberto Amato, Sonia Goncalves, Ewan Harrison, David K. Jackson, Ian Johnston, Dominic Kwiatkowski, Cordelia Langford, John Sillitoe on behalf of the Wellcome Sanger Institute COVID-19 Surveillance Team
EPI_ISL_777218	Lighthouse Lab in Cambridge	Wellcome Sanger Institute for the COVID-19 Genomics UK (COG-UK) Consortium	Rob Howes, The Lighthouse Lab in Cambridge and Alex Alderton, Roberto Amato, Sonia Goncalves, Ewan Harrison, David K. Jackson, Ian Johnston, Dominic Kwiatkowski, Cordelia Langford, John Sillitoe on behalf of the Wellcome Sanger Institute COVID-19 Surveillance Team
EPI_ISL_777219	Lighthouse Lab in Glasgow	Wellcome Sanger Institute for the COVID-19 Genomics UK (COG-UK) Consortium	Harper VanSteenhouse, Yumi Kasai, David Gray, Carol Clugston, Anna Dominiczak and Alex Alderton, Roberto Amato, Sonia Goncalves, Ewan Harrison, David K. Jackson, Ian Johnston, Dominic Kwiatkowski, Cordelia Langford, John Sillitoe on behalf of the Wellcome Sanger Institute COVID-19 Surveillance Team
EPI_ISL_777220, EPI_ISL_777221	Lighthouse Lab in Alderley Park	Wellcome Sanger Institute for the COVID-19 Genomics UK (COG-UK) Consortium	Jacquelyn Wynn, Mairead Hyland, The Lighthouse Lab in Alderley Park and Alex Alderton, Roberto Amato, Sonia Goncalves, Ewan Harrison, David K. Jackson, Ian Johnston, Dominic Kwiatkowski, Cordelia Langford, John Sillitoe on behalf of the Wellcome Sanger Institute COVID-19 Surveillance Team
EPI_ISL_777222, EPI_ISL_777223	Lighthouse Lab in Glasgow	Wellcome Sanger Institute for the COVID-19 Genomics UK (COG-UK) Consortium	Harper VanSteenhouse, Yumi Kasai, David Gray, Carol Clugston, Anna Dominiczak and Alex Alderton, Roberto Amato, Sonia Goncalves, Ewan Harrison, David K. Jackson, Ian Johnston, Dominic Kwiatkowski, Cordelia Langford, John Sillitoe on behalf of the Wellcome Sanger Institute COVID-19 Surveillance Team
EPI_ISL_777224	Lighthouse Lab in Milton Keynes	Wellcome Sanger Institute for the COVID-19 Genomics UK (COG-UK) Consortium	The Lighthouse Lab in Milton Keynes and Alex Alderton, Roberto Amato, Sonia Goncalves, Ewan Harrison, David K. Jackson, Ian Johnston, Dominic Kwiatkowski, Cordelia Langford, John Sillitoe on behalf of the Wellcome Sanger Institute COVID-19 Surveillance Team
EPI_ISL_777225, EPI_ISL_777227	Lighthouse Lab in Glasgow	Wellcome Sanger Institute for the COVID-19 Genomics UK (COG-UK) Consortium	Harper VanSteenhouse, Yumi Kasai, David Gray, Carol Clugston, Anna Dominiczak and Alex Alderton, Roberto Amato, Sonia Goncalves, Ewan Harrison, David K. Jackson, Ian Johnston, Dominic Kwiatkowski, Cordelia Langford, John Sillitoe on behalf of the Wellcome Sanger Institute COVID-19 Surveillance Team
EPI_ISL_777228	Lighthouse Lab in Alderley Park	Wellcome Sanger Institute for the COVID-19 Genomics UK (COG-UK) Consortium	Jacquelyn Wynn, Mairead Hyland, The Lighthouse Lab in Alderley Park and Alex Alderton, Roberto Amato, Sonia Goncalves, Ewan Harrison, David K. Jackson, Ian Johnston, Dominic Kwiatkowski, Cordelia Langford, John Sillitoe on behalf of the Wellcome Sanger Institute COVID-19 Surveillance Team
EPI_ISL_777230	Lighthouse Lab in Glasgow	Wellcome Sanger Institute for the COVID-19 Genomics UK (COG-UK) Consortium	Harper VanSteenhouse, Yumi Kasai, David Gray, Carol Clugston, Anna Dominiczak and Alex Alderton, Roberto Amato, Sonia Goncalves, Ewan Harrison, David K. Jackson, Ian Johnston, Dominic Kwiatkowski, Cordelia Langford, John Sillitoe on behalf of the Wellcome Sanger Institute COVID-19 Surveillance Team
EPI_ISL_777231	Lighthouse Lab in Alderley Park	Wellcome Sanger Institute for the COVID-19 Genomics UK (COG-UK) Consortium	Jacquelyn Wynn, Mairead Hyland, The Lighthouse Lab in Alderley Park and Alex Alderton, Roberto Amato, Sonia Goncalves, Ewan Harrison, David K. Jackson, Ian Johnston, Dominic Kwiatkowski, Cordelia Langford, John Sillitoe on behalf of the Wellcome Sanger Institute COVID-19 Surveillance Team
EPI_ISL_777232	Lighthouse Lab in Glasgow	Wellcome Sanger Institute for the COVID-19 Genomics UK (COG-UK) Consortium	Harper VanSteenhouse, Yumi Kasai, David Gray, Carol Clugston, Anna Dominiczak and Alex Alderton, Roberto Amato, Sonia Goncalves, Ewan Harrison, David K. Jackson, Ian Johnston, Dominic Kwiatkowski, Cordelia Langford, John Sillitoe on behalf of the Wellcome Sanger Institute COVID-19 Surveillance Team
EPI_ISL_777234	Lighthouse Lab in Alderley Park	Wellcome Sanger Institute for the COVID-19 Genomics UK (COG-UK) Consortium	Jacquelyn Wynn, Mairead Hyland, The Lighthouse Lab in Alderley Park and Alex Alderton, Roberto Amato, Sonia Goncalves, Ewan Harrison, David K. Jackson, Ian Johnston, Dominic Kwiatkowski, Cordelia Langford, John Sillitoe on behalf of the Wellcome Sanger Institute COVID-19 Surveillance Team
EPI_ISL_777235, EPI_ISL_777236	Lighthouse Lab in Glasgow	Wellcome Sanger Institute for the COVID-19 Genomics UK (COG-UK) Consortium	Harper VanSteenhouse, Yumi Kasai, David Gray, Carol Clugston, Anna Dominiczak and Alex Alderton, Roberto Amato, Sonia Goncalves, Ewan Harrison, David K. Jackson, Ian Johnston, Dominic Kwiatkowski, Cordelia Langford, John Sillitoe on behalf of the Wellcome Sanger Institute COVID-19 Surveillance Team
EPI_ISL_777237	Lighthouse Lab in Alderley Park	Wellcome Sanger Institute for the COVID-19 Genomics UK (COG-UK) Consortium	Jacquelyn Wynn, Mairead Hyland, The Lighthouse Lab in Alderley Park and Alex Alderton, Roberto Amato, Sonia Goncalves, Ewan Harrison, David K. Jackson, Ian Johnston, Dominic Kwiatkowski, Cordelia Langford, John Sillitoe on behalf of the Wellcome Sanger Institute COVID-19 Surveillance Team
EPI_ISL_777238	Lighthouse Lab in Glasgow	Wellcome Sanger Institute for the COVID-19 Genomics UK (COG-UK) Consortium	Harper VanSteenhouse, Yumi Kasai, David Gray, Carol Clugston, Anna Dominiczak and Alex Alderton, Roberto Amato, Sonia Goncalves, Ewan Harrison, David K. Jackson, Ian Johnston, Dominic Kwiatkowski, Cordelia Langford, John Sillitoe on behalf of the Wellcome Sanger Institute COVID-19 Surveillance Team
EPI_ISL_777239, EPI_ISL_777240	Lighthouse Lab in Alderley Park	Wellcome Sanger Institute for the COVID-19 Genomics UK (COG-UK) Consortium	Jacquelyn Wynn, Mairead Hyland, The Lighthouse Lab in Alderley Park and Alex Alderton, Roberto Amato, Sonia Goncalves, Ewan Harrison, David K. Jackson, Ian Johnston, Dominic Kwiatkowski, Cordelia Langford, John Sillitoe on behalf of the Wellcome Sanger Institute COVID-19 Surveillance Team
EPI_ISL_777241, EPI_ISL_777242	Lighthouse Lab in Glasgow	Wellcome Sanger Institute for the COVID-19 Genomics UK (COG-UK) Consortium	Harper VanSteenhouse, Yumi Kasai, David Gray, Carol Clugston, Anna Dominiczak and Alex Alderton, Roberto Amato, Sonia Goncalves, Ewan Harrison, David K. Jackson, Ian Johnston, Dominic Kwiatkowski, Cordelia Langford, John Sillitoe on behalf of the Wellcome Sanger Institute COVID-19 Surveillance Team
EPI_ISL_777243	Lighthouse Lab in Alderley Park	Wellcome Sanger Institute for the COVID-19 Genomics UK (COG-UK) Consortium	Jacquelyn Wynn, Mairead Hyland, The Lighthouse Lab in Alderley Park and Alex Alderton, Roberto Amato, Sonia Goncalves, Ewan Harrison, David K. Jackson, Ian Johnston, Dominic Kwiatkowski, Cordelia Langford, John Sillitoe on behalf of the Wellcome Sanger Institute COVID-19 Surveillance Team
EPI_ISL_777245	Lighthouse Lab in Milton Keynes	Wellcome Sanger Institute for the COVID-19 Genomics UK	The Lighthouse Lab in Milton Keynes and Alex Alderton, Roberto Amato, Sonia Goncalves, Ewan Harrison, David K. Jackson, Ian Johnston, Dominic

		(COG-UK) Consortium	Kwiatkowski, Cordelia Langford, John Sillitoe on behalf of the Wellcome Sanger Institute COVID-19 Surveillance Team
EPI_ISL_777246	Lighthouse Lab in Glasgow	Wellcome Sanger Institute for the COVID-19 Genomics UK (COG-UK) Consortium	Harper VanSteenhouse, Yumi Kasai, David Gray, Carol Clugston, Anna Dominiczak and Alex Alderton, Roberto Amato, Sonia Goncalves, Ewan Harrison, David K. Jackson, Ian Johnston, Dominic Kwiatkowski, Cordelia Langford, John Sillitoe on behalf of the Wellcome Sanger Institute COVID-19 Surveillance Team
EPI_ISL_777247, EPI_ISL_777248, EPI_ISL_777249	Lighthouse Lab in Alderley Park	Wellcome Sanger Institute for the COVID-19 Genomics UK (COG-UK) Consortium	Jacquelyn Wynn, Mairead Hyland, The Lighthouse Lab in Alderley Park and Alex Alderton, Roberto Amato, Sonia Goncalves, Ewan Harrison, David K. Jackson, Ian Johnston, Dominic Kwiatkowski, Cordelia Langford, John Sillitoe on behalf of the Wellcome Sanger Institute COVID-19 Surveillance Team
EPI_ISL_777250, EPI_ISL_777253	Lighthouse Lab in Glasgow	Wellcome Sanger Institute for the COVID-19 Genomics UK (COG-UK) Consortium	Harper VanSteenhouse, Yumi Kasai, David Gray, Carol Clugston, Anna Dominiczak and Alex Alderton, Roberto Amato, Sonia Goncalves, Ewan Harrison, David K. Jackson, Ian Johnston, Dominic Kwiatkowski, Cordelia Langford, John Sillitoe on behalf of the Wellcome Sanger Institute COVID-19 Surveillance Team
EPI_ISL_777254	Lighthouse Lab in Alderley Park	Wellcome Sanger Institute for the COVID-19 Genomics UK (COG-UK) Consortium	Jacquelyn Wynn, Mairead Hyland, The Lighthouse Lab in Alderley Park and Alex Alderton, Roberto Amato, Sonia Goncalves, Ewan Harrison, David K. Jackson, Ian Johnston, Dominic Kwiatkowski, Cordelia Langford, John Sillitoe on behalf of the Wellcome Sanger Institute COVID-19 Surveillance Team
EPI_ISL_777255	Lighthouse Lab in Glasgow	Wellcome Sanger Institute for the COVID-19 Genomics UK (COG-UK) Consortium	Harper VanSteenhouse, Yumi Kasai, David Gray, Carol Clugston, Anna Dominiczak and Alex Alderton, Roberto Amato, Sonia Goncalves, Ewan Harrison, David K. Jackson, Ian Johnston, Dominic Kwiatkowski, Cordelia Langford, John Sillitoe on behalf of the Wellcome Sanger Institute COVID-19 Surveillance Team
EPI_ISL_777256, EPI_ISL_777257, EPI_ISL_777259, EPI_ISL_777260	Lighthouse Lab in Alderley Park	Wellcome Sanger Institute for the COVID-19 Genomics UK (COG-UK) Consortium	Jacquelyn Wynn, Mairead Hyland, The Lighthouse Lab in Alderley Park and Alex Alderton, Roberto Amato, Sonia Goncalves, Ewan Harrison, David K. Jackson, Ian Johnston, Dominic Kwiatkowski, Cordelia Langford, John Sillitoe on behalf of the Wellcome Sanger Institute COVID-19 Surveillance Team
EPI_ISL_777261	Lighthouse Lab in Glasgow	Wellcome Sanger Institute for the COVID-19 Genomics UK (COG-UK) Consortium	Harper VanSteenhouse, Yumi Kasai, David Gray, Carol Clugston, Anna Dominiczak and Alex Alderton, Roberto Amato, Sonia Goncalves, Ewan Harrison, David K. Jackson, Ian Johnston, Dominic Kwiatkowski, Cordelia Langford, John Sillitoe on behalf of the Wellcome Sanger Institute COVID-19 Surveillance Team
EPI_ISL_777262, EPI_ISL_777263, EPI_ISL_777264, EPI_ISL_777265, EPI_ISL_777271, EPI_ISL_777274	Lighthouse Lab in Alderley Park	Wellcome Sanger Institute for the COVID-19 Genomics UK (COG-UK) Consortium	Jacquelyn Wynn, Mairead Hyland, The Lighthouse Lab in Alderley Park and Alex Alderton, Roberto Amato, Sonia Goncalves, Ewan Harrison, David K. Jackson, Ian Johnston, Dominic Kwiatkowski, Cordelia Langford, John Sillitoe on behalf of the Wellcome Sanger Institute COVID-19 Surveillance Team
EPI_ISL_777275	Lighthouse Lab in Cambridge	Wellcome Sanger Institute for the COVID-19 Genomics UK (COG-UK) Consortium	Rob Howes, The Lighthouse Lab in Cambridge and Alex Alderton, Roberto Amato, Sonia Goncalves, Ewan Harrison, David K. Jackson, Ian Johnston, Dominic Kwiatkowski, Cordelia Langford, John Sillitoe on behalf of the Wellcome Sanger Institute COVID-19 Surveillance Team
EPI_ISL_777276, EPI_ISL_777277, EPI_ISL_777278, EPI_ISL_777280, EPI_ISL_777281	Lighthouse Lab in Alderley Park	Wellcome Sanger Institute for the COVID-19 Genomics UK (COG-UK) Consortium	Jacquelyn Wynn, Mairead Hyland, The Lighthouse Lab in Alderley Park and Alex Alderton, Roberto Amato, Sonia Goncalves, Ewan Harrison, David K. Jackson, Ian Johnston, Dominic Kwiatkowski, Cordelia Langford, John Sillitoe on behalf of the Wellcome Sanger Institute COVID-19 Surveillance Team
EPI_ISL_777283, EPI_ISL_777284	Lighthouse Lab in Glasgow	Wellcome Sanger Institute for the COVID-19 Genomics UK (COG-UK) Consortium	Harper VanSteenhouse, Yumi Kasai, David Gray, Carol Clugston, Anna Dominiczak and Alex Alderton, Roberto Amato, Sonia Goncalves, Ewan Harrison, David K. Jackson, Ian Johnston, Dominic Kwiatkowski, Cordelia Langford, John Sillitoe on behalf of the Wellcome Sanger Institute COVID-19 Surveillance Team
EPI_ISL_777285, EPI_ISL_777286, EPI_ISL_777287	Lighthouse Lab in Alderley Park	Wellcome Sanger Institute for the COVID-19 Genomics UK (COG-UK) Consortium	Jacquelyn Wynn, Mairead Hyland, The Lighthouse Lab in Alderley Park and Alex Alderton, Roberto Amato, Sonia Goncalves, Ewan Harrison, David K. Jackson, Ian Johnston, Dominic Kwiatkowski, Cordelia Langford, John Sillitoe on behalf of the Wellcome Sanger Institute COVID-19 Surveillance Team
EPI_ISL_777289	Lighthouse Lab in Cambridge	Wellcome Sanger Institute for the COVID-19 Genomics UK (COG-UK) Consortium	Rob Howes, The Lighthouse Lab in Cambridge and Alex Alderton, Roberto Amato, Sonia Goncalves, Ewan Harrison, David K. Jackson, Ian Johnston, Dominic Kwiatkowski, Cordelia Langford, John Sillitoe on behalf of the Wellcome Sanger Institute COVID-19 Surveillance Team
EPI_ISL_777290	Lighthouse Lab in Glasgow	Wellcome Sanger Institute for the COVID-19 Genomics UK (COG-UK) Consortium	Harper VanSteenhouse, Yumi Kasai, David Gray, Carol Clugston, Anna Dominiczak and Alex Alderton, Roberto Amato, Sonia Goncalves, Ewan Harrison, David K. Jackson, Ian Johnston, Dominic Kwiatkowski, Cordelia Langford, John Sillitoe on behalf of the Wellcome Sanger Institute COVID-19 Surveillance Team
EPI_ISL_777291	Lighthouse Lab in Alderley Park	Wellcome Sanger Institute for the COVID-19 Genomics UK (COG-UK) Consortium	Jacquelyn Wynn, Mairead Hyland, The Lighthouse Lab in Alderley Park and Alex Alderton, Roberto Amato, Sonia Goncalves, Ewan Harrison, David K. Jackson, Ian Johnston, Dominic Kwiatkowski, Cordelia Langford, John Sillitoe on behalf of the Wellcome Sanger Institute COVID-19 Surveillance Team
EPI_ISL_777293	Lighthouse Lab in Cambridge	Wellcome Sanger Institute for the COVID-19 Genomics UK (COG-UK) Consortium	Rob Howes, The Lighthouse Lab in Cambridge and Alex Alderton, Roberto Amato, Sonia Goncalves, Ewan Harrison, David K. Jackson, Ian Johnston, Dominic Kwiatkowski, Cordelia Langford, John Sillitoe on behalf of the Wellcome Sanger Institute COVID-19 Surveillance Team
EPI_ISL_777294	Lighthouse Lab in Alderley Park	Wellcome Sanger Institute for the COVID-19 Genomics UK (COG-UK) Consortium	Jacquelyn Wynn, Mairead Hyland, The Lighthouse Lab in Alderley Park and Alex Alderton, Roberto Amato, Sonia Goncalves, Ewan Harrison, David K. Jackson, Ian Johnston, Dominic Kwiatkowski, Cordelia Langford, John Sillitoe on behalf of the Wellcome Sanger Institute COVID-19 Surveillance Team
EPI_ISL_777295	Lighthouse Lab in Milton Keynes	Wellcome Sanger Institute for the COVID-19 Genomics UK (COG-UK) Consortium	The Lighthouse Lab in Milton Keynes and Alex Alderton, Roberto Amato, Sonia Goncalves, Ewan Harrison, David K. Jackson, Ian Johnston, Dominic Kwiatkowski, Cordelia Langford, John Sillitoe on behalf of the Wellcome Sanger Institute COVID-19 Surveillance Team
EPI_ISL_777296, EPI_ISL_777297	Lighthouse Lab in Alderley Park	Wellcome Sanger Institute for the COVID-19 Genomics UK (COG-UK) Consortium	Jacquelyn Wynn, Mairead Hyland, The Lighthouse Lab in Alderley Park and Alex Alderton, Roberto Amato, Sonia Goncalves, Ewan Harrison, David K. Jackson, Ian Johnston, Dominic Kwiatkowski, Cordelia Langford, John Sillitoe on behalf of the Wellcome Sanger Institute COVID-19 Surveillance Team
EPI_ISL_777298	Lighthouse Lab in Glasgow	Wellcome Sanger Institute for the COVID-19 Genomics UK (COG-UK) Consortium	Harper VanSteenhouse, Yumi Kasai, David Gray, Carol Clugston, Anna Dominiczak and Alex Alderton, Roberto Amato, Sonia Goncalves, Ewan Harrison, David K. Jackson, Ian Johnston, Dominic Kwiatkowski, Cordelia Langford, John Sillitoe on behalf of the Wellcome Sanger Institute COVID-19 Surveillance Team
EPI_ISL_777299	Lighthouse Lab in Alderley Park	Wellcome Sanger Institute for the COVID-19 Genomics UK (COG-UK) Consortium	Jacquelyn Wynn, Mairead Hyland, The Lighthouse Lab in Alderley Park and Alex Alderton, Roberto Amato, Sonia Goncalves, Ewan Harrison, David K. Jackson, Ian Johnston, Dominic Kwiatkowski, Cordelia Langford, John Sillitoe on behalf of the Wellcome Sanger Institute COVID-19 Surveillance Team
EPI_ISL_777302	Lighthouse Lab in Glasgow	Wellcome Sanger Institute for the COVID-19 Genomics UK (COG-UK) Consortium	Harper VanSteenhouse, Yumi Kasai, David Gray, Carol Clugston, Anna Dominiczak and Alex Alderton, Roberto Amato, Sonia Goncalves, Ewan Harrison, David K. Jackson, Ian Johnston, Dominic Kwiatkowski, Cordelia Langford, John Sillitoe on behalf of the Wellcome Sanger Institute COVID-19 Surveillance Team
EPI_ISL_777304	Lighthouse Lab in Milton Keynes	Wellcome Sanger Institute for the COVID-19 Genomics UK (COG-UK) Consortium	The Lighthouse Lab in Milton Keynes and Alex Alderton, Roberto Amato, Sonia Goncalves, Ewan Harrison, David K. Jackson, Ian Johnston, Dominic Kwiatkowski, Cordelia Langford, John Sillitoe on behalf of the Wellcome Sanger Institute COVID-19 Surveillance Team
EPI_ISL_777305, EPI_ISL_777306	Lighthouse Lab in Glasgow	Wellcome Sanger Institute for the COVID-19 Genomics UK (COG-UK) Consortium	Harper VanSteenhouse, Yumi Kasai, David Gray, Carol Clugston, Anna Dominiczak and Alex Alderton, Roberto Amato, Sonia Goncalves, Ewan Harrison, David K. Jackson, Ian Johnston, Dominic Kwiatkowski, Cordelia Langford, John Sillitoe on behalf of the Wellcome Sanger Institute COVID-19 Surveillance Team
EPI_ISL_777308, EPI_ISL_777309, EPI_ISL_777311	Lighthouse Lab in Alderley Park	Wellcome Sanger Institute for the COVID-19 Genomics UK (COG-UK) Consortium	Jacquelyn Wynn, Mairead Hyland, The Lighthouse Lab in Alderley Park and Alex Alderton, Roberto Amato, Sonia Goncalves, Ewan Harrison, David K. Jackson, Ian Johnston, Dominic Kwiatkowski, Cordelia Langford, John Sillitoe on behalf of the Wellcome Sanger Institute COVID-19 Surveillance Team
EPI_ISL_777313	Lighthouse Lab in Milton Keynes	Wellcome Sanger Institute for the COVID-19 Genomics UK (COG-UK) Consortium	The Lighthouse Lab in Milton Keynes and Alex Alderton, Roberto Amato, Sonia Goncalves, Ewan Harrison, David K. Jackson, Ian Johnston, Dominic Kwiatkowski, Cordelia Langford, John Sillitoe on behalf of the Wellcome Sanger Institute COVID-19 Surveillance Team
EPI_ISL_777314, EPI_ISL_777315	Lighthouse Lab in Alderley Park	Wellcome Sanger Institute for the COVID-19 Genomics UK (COG-UK) Consortium	Jacquelyn Wynn, Mairead Hyland, The Lighthouse Lab in Alderley Park and Alex Alderton, Roberto Amato, Sonia Goncalves, Ewan Harrison, David K. Jackson, Ian Johnston, Dominic Kwiatkowski, Cordelia Langford, John Sillitoe on behalf of the Wellcome Sanger Institute COVID-19 Surveillance Team
EPI_ISL_777316	Lighthouse Lab in Glasgow	Wellcome Sanger Institute for the COVID-19 Genomics UK (COG-UK) Consortium	Harper VanSteenhouse, Yumi Kasai, David Gray, Carol Clugston, Anna Dominiczak and Alex Alderton, Roberto Amato, Sonia Goncalves, Ewan Harrison, David K. Jackson, Ian Johnston, Dominic Kwiatkowski, Cordelia Langford, John Sillitoe on behalf of the Wellcome Sanger Institute COVID-19 Surveillance Team
EPI_ISL_777318, EPI_ISL_777319	Lighthouse Lab in Alderley Park	Wellcome Sanger Institute for the COVID-19 Genomics UK (COG-UK) Consortium	Jacquelyn Wynn, Mairead Hyland, The Lighthouse Lab in Alderley Park and Alex Alderton, Roberto Amato, Sonia Goncalves, Ewan Harrison, David K. Jackson, Ian Johnston, Dominic Kwiatkowski, Cordelia Langford, John Sillitoe on behalf of the Wellcome Sanger Institute COVID-19 Surveillance Team
EPI_ISL_777321, EPI_ISL_777322	Lighthouse Lab in Glasgow	Wellcome Sanger Institute for the COVID-19 Genomics UK (COG-UK) Consortium	Harper VanSteenhouse, Yumi Kasai, David Gray, Carol Clugston, Anna Dominiczak and Alex Alderton, Roberto Amato, Sonia Goncalves, Ewan Harrison, David K. Jackson, Ian Johnston, Dominic Kwiatkowski, Cordelia Langford, John Sillitoe on behalf of the Wellcome Sanger Institute COVID-19 Surveillance

			Team
EPI_ISL_777323	Lighthouse Lab in Cambridge	Wellcome Sanger Institute for the COVID-19 Genomics UK (COG-UK) Consortium	Rob Howes, The Lighthouse Lab in Cambridge and Alex Alderton, Roberto Amato, Sonia Goncalves, Ewan Harrison, David K. Jackson, Ian Johnston, Dominic Kwiatkowski, Cordelia Langford, John Sillitoe on behalf of the Wellcome Sanger Institute COVID-19 Surveillance Team
EPI_ISL_777324	Lighthouse Lab in Glasgow	Wellcome Sanger Institute for the COVID-19 Genomics UK (COG-UK) Consortium	Harper VanSteenhouse, Yumi Kasai, David Gray, Carol Clugston, Anna Dominiczak and Alex Alderton, Roberto Amato, Sonia Goncalves, Ewan Harrison, David K. Jackson, Ian Johnston, Dominic Kwiatkowski, Cordelia Langford, John Sillitoe on behalf of the Wellcome Sanger Institute COVID-19 Surveillance Team
EPI_ISL_777326, EPI_ISL_777327	Lighthouse Lab in Alderley Park	Wellcome Sanger Institute for the COVID-19 Genomics UK (COG-UK) Consortium	Jacquelyn Wynn, Mairead Hyland, The Lighthouse Lab in Alderley Park and Alex Alderton, Roberto Amato, Sonia Goncalves, Ewan Harrison, David K. Jackson, Ian Johnston, Dominic Kwiatkowski, Cordelia Langford, John Sillitoe on behalf of the Wellcome Sanger Institute COVID-19 Surveillance Team
EPI_ISL_777328, EPI_ISL_777330, EPI_ISL_777331, EPI_ISL_777332, EPI_ISL_777334	Lighthouse Lab in Cambridge	Wellcome Sanger Institute for the COVID-19 Genomics UK (COG-UK) Consortium	Rob Howes, The Lighthouse Lab in Cambridge and Alex Alderton, Roberto Amato, Sonia Goncalves, Ewan Harrison, David K. Jackson, Ian Johnston, Dominic Kwiatkowski, Cordelia Langford, John Sillitoe on behalf of the Wellcome Sanger Institute COVID-19 Surveillance Team
EPI_ISL_777338	Lighthouse Lab in Alderley Park	Wellcome Sanger Institute for the COVID-19 Genomics UK (COG-UK) Consortium	Jacquelyn Wynn, Mairead Hyland, The Lighthouse Lab in Alderley Park and Alex Alderton, Roberto Amato, Sonia Goncalves, Ewan Harrison, David K. Jackson, Ian Johnston, Dominic Kwiatkowski, Cordelia Langford, John Sillitoe on behalf of the Wellcome Sanger Institute COVID-19 Surveillance Team
EPI_ISL_777339	Lighthouse Lab in Cambridge	Wellcome Sanger Institute for the COVID-19 Genomics UK (COG-UK) Consortium	Rob Howes, The Lighthouse Lab in Cambridge and Alex Alderton, Roberto Amato, Sonia Goncalves, Ewan Harrison, David K. Jackson, Ian Johnston, Dominic Kwiatkowski, Cordelia Langford, John Sillitoe on behalf of the Wellcome Sanger Institute COVID-19 Surveillance Team
EPI_ISL_777340, EPI_ISL_777341	Lighthouse Lab in Glasgow	Wellcome Sanger Institute for the COVID-19 Genomics UK (COG-UK) Consortium	Harper VanSteenhouse, Yumi Kasai, David Gray, Carol Clugston, Anna Dominiczak and Alex Alderton, Roberto Amato, Sonia Goncalves, Ewan Harrison, David K. Jackson, Ian Johnston, Dominic Kwiatkowski, Cordelia Langford, John Sillitoe on behalf of the Wellcome Sanger Institute COVID-19 Surveillance Team
EPI_ISL_777342, EPI_ISL_777343, EPI_ISL_777344, EPI_ISL_777345	Lighthouse Lab in Cambridge	Wellcome Sanger Institute for the COVID-19 Genomics UK (COG-UK) Consortium	Rob Howes, The Lighthouse Lab in Cambridge and Alex Alderton, Roberto Amato, Sonia Goncalves, Ewan Harrison, David K. Jackson, Ian Johnston, Dominic Kwiatkowski, Cordelia Langford, John Sillitoe on behalf of the Wellcome Sanger Institute COVID-19 Surveillance Team
EPI_ISL_777346	Lighthouse Lab in Glasgow	Wellcome Sanger Institute for the COVID-19 Genomics UK (COG-UK) Consortium	Harper VanSteenhouse, Yumi Kasai, David Gray, Carol Clugston, Anna Dominiczak and Alex Alderton, Roberto Amato, Sonia Goncalves, Ewan Harrison, David K. Jackson, Ian Johnston, Dominic Kwiatkowski, Cordelia Langford, John Sillitoe on behalf of the Wellcome Sanger Institute COVID-19 Surveillance Team
EPI_ISL_777347, EPI_ISL_777348, EPI_ISL_777350, EPI_ISL_777351, EPI_ISL_777352	Lighthouse Lab in Cambridge	Wellcome Sanger Institute for the COVID-19 Genomics UK (COG-UK) Consortium	Rob Howes, The Lighthouse Lab in Cambridge and Alex Alderton, Roberto Amato, Sonia Goncalves, Ewan Harrison, David K. Jackson, Ian Johnston, Dominic Kwiatkowski, Cordelia Langford, John Sillitoe on behalf of the Wellcome Sanger Institute COVID-19 Surveillance Team
EPI_ISL_777353	Lighthouse Lab in Glasgow	Wellcome Sanger Institute for the COVID-19 Genomics UK (COG-UK) Consortium	Harper VanSteenhouse, Yumi Kasai, David Gray, Carol Clugston, Anna Dominiczak and Alex Alderton, Roberto Amato, Sonia Goncalves, Ewan Harrison, David K. Jackson, Ian Johnston, Dominic Kwiatkowski, Cordelia Langford, John Sillitoe on behalf of the Wellcome Sanger Institute COVID-19 Surveillance Team
EPI_ISL_777354, EPI_ISL_777355	Lighthouse Lab in Cambridge	Wellcome Sanger Institute for the COVID-19 Genomics UK (COG-UK) Consortium	Rob Howes, The Lighthouse Lab in Cambridge and Alex Alderton, Roberto Amato, Sonia Goncalves, Ewan Harrison, David K. Jackson, Ian Johnston, Dominic Kwiatkowski, Cordelia Langford, John Sillitoe on behalf of the Wellcome Sanger Institute COVID-19 Surveillance Team
EPI_ISL_777358	Lighthouse Lab in Glasgow	Wellcome Sanger Institute for the COVID-19 Genomics UK (COG-UK) Consortium	Harper VanSteenhouse, Yumi Kasai, David Gray, Carol Clugston, Anna Dominiczak and Alex Alderton, Roberto Amato, Sonia Goncalves, Ewan Harrison, David K. Jackson, Ian Johnston, Dominic Kwiatkowski, Cordelia Langford, John Sillitoe on behalf of the Wellcome Sanger Institute COVID-19 Surveillance Team
EPI_ISL_777359, EPI_ISL_777360	Lighthouse Lab in Cambridge	Wellcome Sanger Institute for the COVID-19 Genomics UK (COG-UK) Consortium	Rob Howes, The Lighthouse Lab in Cambridge and Alex Alderton, Roberto Amato, Sonia Goncalves, Ewan Harrison, David K. Jackson, Ian Johnston, Dominic Kwiatkowski, Cordelia Langford, John Sillitoe on behalf of the Wellcome Sanger Institute COVID-19 Surveillance Team
EPI_ISL_777361	Lighthouse Lab in Glasgow	Wellcome Sanger Institute for the COVID-19 Genomics UK (COG-UK) Consortium	Harper VanSteenhouse, Yumi Kasai, David Gray, Carol Clugston, Anna Dominiczak and Alex Alderton, Roberto Amato, Sonia Goncalves, Ewan Harrison, David K. Jackson, Ian Johnston, Dominic Kwiatkowski, Cordelia Langford, John Sillitoe on behalf of the Wellcome Sanger Institute COVID-19 Surveillance Team
EPI_ISL_777362	Lighthouse Lab in Cambridge	Wellcome Sanger Institute for the COVID-19 Genomics UK (COG-UK) Consortium	Rob Howes, The Lighthouse Lab in Cambridge and Alex Alderton, Roberto Amato, Sonia Goncalves, Ewan Harrison, David K. Jackson, Ian Johnston, Dominic Kwiatkowski, Cordelia Langford, John Sillitoe on behalf of the Wellcome Sanger Institute COVID-19 Surveillance Team
EPI_ISL_777363	Lighthouse Lab in Glasgow	Wellcome Sanger Institute for the COVID-19 Genomics UK (COG-UK) Consortium	Harper VanSteenhouse, Yumi Kasai, David Gray, Carol Clugston, Anna Dominiczak and Alex Alderton, Roberto Amato, Sonia Goncalves, Ewan Harrison, David K. Jackson, Ian Johnston, Dominic Kwiatkowski, Cordelia Langford, John Sillitoe on behalf of the Wellcome Sanger Institute COVID-19 Surveillance Team
EPI_ISL_777365, EPI_ISL_777366, EPI_ISL_777367, EPI_ISL_777368, EPI_ISL_777370, EPI_ISL_777371	Lighthouse Lab in Cambridge	Wellcome Sanger Institute for the COVID-19 Genomics UK (COG-UK) Consortium	Rob Howes, The Lighthouse Lab in Cambridge and Alex Alderton, Roberto Amato, Sonia Goncalves, Ewan Harrison, David K. Jackson, Ian Johnston, Dominic Kwiatkowski, Cordelia Langford, John Sillitoe on behalf of the Wellcome Sanger Institute COVID-19 Surveillance Team
EPI_ISL_777372	Lighthouse Lab in Glasgow	Wellcome Sanger Institute for the COVID-19 Genomics UK (COG-UK) Consortium	Harper VanSteenhouse, Yumi Kasai, David Gray, Carol Clugston, Anna Dominiczak and Alex Alderton, Roberto Amato, Sonia Goncalves, Ewan Harrison, David K. Jackson, Ian Johnston, Dominic Kwiatkowski, Cordelia Langford, John Sillitoe on behalf of the Wellcome Sanger Institute COVID-19 Surveillance Team
EPI_ISL_777375	Lighthouse Lab in Alderley Park	Wellcome Sanger Institute for the COVID-19 Genomics UK (COG-UK) Consortium	Jacquelyn Wynn, Mairead Hyland, The Lighthouse Lab in Alderley Park and Alex Alderton, Roberto Amato, Sonia Goncalves, Ewan Harrison, David K. Jackson, Ian Johnston, Dominic Kwiatkowski, Cordelia Langford, John Sillitoe on behalf of the Wellcome Sanger Institute COVID-19 Surveillance Team
EPI_ISL_777376, EPI_ISL_777377	Lighthouse Lab in Glasgow	Wellcome Sanger Institute for the COVID-19 Genomics UK (COG-UK) Consortium	Harper VanSteenhouse, Yumi Kasai, David Gray, Carol Clugston, Anna Dominiczak and Alex Alderton, Roberto Amato, Sonia Goncalves, Ewan Harrison, David K. Jackson, Ian Johnston, Dominic Kwiatkowski, Cordelia Langford, John Sillitoe on behalf of the Wellcome Sanger Institute COVID-19 Surveillance Team
EPI_ISL_777378, EPI_ISL_777379	Lighthouse Lab in Cambridge	Wellcome Sanger Institute for the COVID-19 Genomics UK (COG-UK) Consortium	Rob Howes, The Lighthouse Lab in Cambridge and Alex Alderton, Roberto Amato, Sonia Goncalves, Ewan Harrison, David K. Jackson, Ian Johnston, Dominic Kwiatkowski, Cordelia Langford, John Sillitoe on behalf of the Wellcome Sanger Institute COVID-19 Surveillance Team
EPI_ISL_777380	Lighthouse Lab in Alderley Park	Wellcome Sanger Institute for the COVID-19 Genomics UK (COG-UK) Consortium	Jacquelyn Wynn, Mairead Hyland, The Lighthouse Lab in Alderley Park and Alex Alderton, Roberto Amato, Sonia Goncalves, Ewan Harrison, David K. Jackson, Ian Johnston, Dominic Kwiatkowski, Cordelia Langford, John Sillitoe on behalf of the Wellcome Sanger Institute COVID-19 Surveillance Team
EPI_ISL_777381	Lighthouse Lab in Cambridge	Wellcome Sanger Institute for the COVID-19 Genomics UK (COG-UK) Consortium	Rob Howes, The Lighthouse Lab in Cambridge and Alex Alderton, Roberto Amato, Sonia Goncalves, Ewan Harrison, David K. Jackson, Ian Johnston, Dominic Kwiatkowski, Cordelia Langford, John Sillitoe on behalf of the Wellcome Sanger Institute COVID-19 Surveillance Team
EPI_ISL_777382	Lighthouse Lab in Glasgow	Wellcome Sanger Institute for the COVID-19 Genomics UK (COG-UK) Consortium	Harper VanSteenhouse, Yumi Kasai, David Gray, Carol Clugston, Anna Dominiczak and Alex Alderton, Roberto Amato, Sonia Goncalves, Ewan Harrison, David K. Jackson, Ian Johnston, Dominic Kwiatkowski, Cordelia Langford, John Sillitoe on behalf of the Wellcome Sanger Institute COVID-19 Surveillance Team
EPI_ISL_777384	Lighthouse Lab in Cambridge	Wellcome Sanger Institute for the COVID-19 Genomics UK (COG-UK) Consortium	Rob Howes, The Lighthouse Lab in Cambridge and Alex Alderton, Roberto Amato, Sonia Goncalves, Ewan Harrison, David K. Jackson, Ian Johnston, Dominic Kwiatkowski, Cordelia Langford, John Sillitoe on behalf of the Wellcome Sanger Institute COVID-19 Surveillance Team
EPI_ISL_777385	Lighthouse Lab in Glasgow	Wellcome Sanger Institute for the COVID-19 Genomics UK (COG-UK) Consortium	Harper VanSteenhouse, Yumi Kasai, David Gray, Carol Clugston, Anna Dominiczak and Alex Alderton, Roberto Amato, Sonia Goncalves, Ewan Harrison, David K. Jackson, Ian Johnston, Dominic Kwiatkowski, Cordelia Langford, John Sillitoe on behalf of the Wellcome Sanger Institute COVID-19 Surveillance Team
EPI_ISL_777386	Lighthouse Lab in Alderley Park	Wellcome Sanger Institute for the COVID-19 Genomics UK (COG-UK) Consortium	Jacquelyn Wynn, Mairead Hyland, The Lighthouse Lab in Alderley Park and Alex Alderton, Roberto Amato, Sonia Goncalves, Ewan Harrison, David K. Jackson, Ian Johnston, Dominic Kwiatkowski, Cordelia Langford, John Sillitoe on behalf of the Wellcome Sanger Institute COVID-19 Surveillance Team
EPI_ISL_777387, EPI_ISL_777388	Lighthouse Lab in Cambridge	Wellcome Sanger Institute for the COVID-19 Genomics UK (COG-UK) Consortium	Rob Howes, The Lighthouse Lab in Cambridge and Alex Alderton, Roberto Amato, Sonia Goncalves, Ewan Harrison, David K. Jackson, Ian Johnston, Dominic Kwiatkowski, Cordelia Langford, John Sillitoe on behalf of the Wellcome Sanger Institute COVID-19 Surveillance Team

EPI_ISL_777389	Lighthouse Lab in Alderley Park	Wellcome Sanger Institute for the COVID-19 Genomics UK (COG-UK) Consortium	Jacquelyn Wynn, Mairead Hyland, The Lighthouse Lab in Alderley Park and Alex Alderton, Roberto Amato, Sonia Goncalves, Ewan Harrison, David K. Jackson, Ian Johnston, Dominic Kwiatkowski, Cordelia Langford, John Sillitoe on behalf of the Wellcome Sanger Institute COVID-19 Surveillance Team
EPI_ISL_777390, EPI_ISL_777391	Lighthouse Lab in Cambridge	Wellcome Sanger Institute for the COVID-19 Genomics UK (COG-UK) Consortium	Rob Howes, The Lighthouse Lab in Cambridge and Alex Alderton, Roberto Amato, Sonia Goncalves, Ewan Harrison, David K. Jackson, Ian Johnston, Dominic Kwiatkowski, Cordelia Langford, John Sillitoe on behalf of the Wellcome Sanger Institute COVID-19 Surveillance Team
EPI_ISL_777392, EPI_ISL_777393, EPI_ISL_777395	Lighthouse Lab in Glasgow	Wellcome Sanger Institute for the COVID-19 Genomics UK (COG-UK) Consortium	Harper VanSteenhouse, Yumi Kasai, David Gray, Carol Clugston, Anna Dominiczak and Alex Alderton, Roberto Amato, Sonia Goncalves, Ewan Harrison, David K. Jackson, Ian Johnston, Dominic Kwiatkowski, Cordelia Langford, John Sillitoe on behalf of the Wellcome Sanger Institute COVID-19 Surveillance Team
EPI_ISL_777396	Lighthouse Lab in Cambridge	Wellcome Sanger Institute for the COVID-19 Genomics UK (COG-UK) Consortium	Rob Howes, The Lighthouse Lab in Cambridge and Alex Alderton, Roberto Amato, Sonia Goncalves, Ewan Harrison, David K. Jackson, Ian Johnston, Dominic Kwiatkowski, Cordelia Langford, John Sillitoe on behalf of the Wellcome Sanger Institute COVID-19 Surveillance Team

We gratefully acknowledge the following Authors from the Originating laboratories responsible for obtaining the specimens, as well as the Submitting laboratories where the genome data were generated and shared via GISAID, on which this research is based.

All Submitters of data may be contacted directly via www.gisaid.org

Authors are sorted alphabetically.

Accession ID	Originating Laboratory	Submitting Laboratory	Authors
EPI_ISL_777398	Lighthouse Lab in Cambridge	Wellcome Sanger Institute for the COVID-19 Genomics UK (COG-UK) Consortium	Rob Howes, The Lighthouse Lab in Cambridge and Alex Alderton, Roberto Amato, Sonia Goncalves, Ewan Harrison, David K. Jackson, Ian Johnston, Dominic Kwiatkowski, Cordelia Langford, John Sillitoe on behalf of the Wellcome Sanger Institute COVID-19 Surveillance Team
EPI_ISL_777399	Lighthouse Lab in Glasgow	Wellcome Sanger Institute for the COVID-19 Genomics UK (COG-UK) Consortium	Harper VanSteenhouse, Yumi Kasai, David Gray, Carol Clugston, Anna Dominiczak and Alex Alderton, Roberto Amato, Sonia Goncalves, Ewan Harrison, David K. Jackson, Ian Johnston, Dominic Kwiatkowski, Cordelia Langford, John Sillitoe on behalf of the Wellcome Sanger Institute COVID-19 Surveillance Team
EPI_ISL_777400, EPI_ISL_777401	Lighthouse Lab in Cambridge	Wellcome Sanger Institute for the COVID-19 Genomics UK (COG-UK) Consortium	Rob Howes, The Lighthouse Lab in Cambridge and Alex Alderton, Roberto Amato, Sonia Goncalves, Ewan Harrison, David K. Jackson, Ian Johnston, Dominic Kwiatkowski, Cordelia Langford, John Sillitoe on behalf of the Wellcome Sanger Institute COVID-19 Surveillance Team
EPI_ISL_777402	Lighthouse Lab in Glasgow	Wellcome Sanger Institute for the COVID-19 Genomics UK (COG-UK) Consortium	Harper VanSteenhouse, Yumi Kasai, David Gray, Carol Clugston, Anna Dominiczak and Alex Alderton, Roberto Amato, Sonia Goncalves, Ewan Harrison, David K. Jackson, Ian Johnston, Dominic Kwiatkowski, Cordelia Langford, John Sillitoe on behalf of the Wellcome Sanger Institute COVID-19 Surveillance Team
EPI_ISL_777406	Lighthouse Lab in Cambridge	Wellcome Sanger Institute for the COVID-19 Genomics UK (COG-UK) Consortium	Rob Howes, The Lighthouse Lab in Cambridge and Alex Alderton, Roberto Amato, Sonia Goncalves, Ewan Harrison, David K. Jackson, Ian Johnston, Dominic Kwiatkowski, Cordelia Langford, John Sillitoe on behalf of the Wellcome Sanger Institute COVID-19 Surveillance Team
EPI_ISL_777407	Lighthouse Lab in Glasgow	Wellcome Sanger Institute for the COVID-19 Genomics UK (COG-UK) Consortium	Harper VanSteenhouse, Yumi Kasai, David Gray, Carol Clugston, Anna Dominiczak and Alex Alderton, Roberto Amato, Sonia Goncalves, Ewan Harrison, David K. Jackson, Ian Johnston, Dominic Kwiatkowski, Cordelia Langford, John Sillitoe on behalf of the Wellcome Sanger Institute COVID-19 Surveillance Team
EPI_ISL_777409	Lighthouse Lab in Cambridge	Wellcome Sanger Institute for the COVID-19 Genomics UK (COG-UK) Consortium	Rob Howes, The Lighthouse Lab in Cambridge and Alex Alderton, Roberto Amato, Sonia Goncalves, Ewan Harrison, David K. Jackson, Ian Johnston, Dominic Kwiatkowski, Cordelia Langford, John Sillitoe on behalf of the Wellcome Sanger Institute COVID-19 Surveillance Team
EPI_ISL_777410	Lighthouse Lab in Glasgow	Wellcome Sanger Institute for the COVID-19 Genomics UK (COG-UK) Consortium	Harper VanSteenhouse, Yumi Kasai, David Gray, Carol Clugston, Anna Dominiczak and Alex Alderton, Roberto Amato, Sonia Goncalves, Ewan Harrison, David K. Jackson, Ian Johnston, Dominic Kwiatkowski, Cordelia Langford, John Sillitoe on behalf of the Wellcome Sanger Institute COVID-19 Surveillance Team
EPI_ISL_777412	Lighthouse Lab in Alderley Park	Wellcome Sanger Institute for the COVID-19 Genomics UK (COG-UK) Consortium	Jacquelyn Wynn, Mairead Hyland, The Lighthouse Lab in Alderley Park and Alex Alderton, Roberto Amato, Sonia Goncalves, Ewan Harrison, David K. Jackson, Ian Johnston, Dominic Kwiatkowski, Cordelia Langford, John Sillitoe on behalf of the Wellcome Sanger Institute COVID-19 Surveillance Team
EPI_ISL_777413, EPI_ISL_777414	Lighthouse Lab in Cambridge	Wellcome Sanger Institute for the COVID-19 Genomics UK (COG-UK) Consortium	Rob Howes, The Lighthouse Lab in Cambridge and Alex Alderton, Roberto Amato, Sonia Goncalves, Ewan Harrison, David K. Jackson, Ian Johnston, Dominic Kwiatkowski, Cordelia Langford, John Sillitoe on behalf of the Wellcome Sanger Institute COVID-19 Surveillance Team
EPI_ISL_777416	Lighthouse Lab in Alderley Park	Wellcome Sanger Institute for the COVID-19 Genomics UK (COG-UK) Consortium	Jacquelyn Wynn, Mairead Hyland, The Lighthouse Lab in Alderley Park and Alex Alderton, Roberto Amato, Sonia Goncalves, Ewan Harrison, David K. Jackson, Ian Johnston, Dominic Kwiatkowski, Cordelia Langford, John Sillitoe on behalf of the Wellcome Sanger Institute COVID-19 Surveillance Team
EPI_ISL_777417	Lighthouse Lab in Glasgow	Wellcome Sanger Institute for the COVID-19 Genomics UK (COG-UK) Consortium	Harper VanSteenhouse, Yumi Kasai, David Gray, Carol Clugston, Anna Dominiczak and Alex Alderton, Roberto Amato, Sonia Goncalves, Ewan Harrison, David K. Jackson, Ian Johnston, Dominic Kwiatkowski, Cordelia Langford, John Sillitoe on behalf of the Wellcome Sanger Institute COVID-19 Surveillance Team
EPI_ISL_777418, EPI_ISL_777419	Lighthouse Lab in Cambridge	Wellcome Sanger Institute for the COVID-19 Genomics UK (COG-UK) Consortium	Rob Howes, The Lighthouse Lab in Cambridge and Alex Alderton, Roberto Amato, Sonia Goncalves, Ewan Harrison, David K. Jackson, Ian Johnston, Dominic Kwiatkowski, Cordelia Langford, John Sillitoe on behalf of the Wellcome Sanger Institute COVID-19 Surveillance Team
EPI_ISL_777420	Lighthouse Lab in Glasgow	Wellcome Sanger Institute for the COVID-19 Genomics UK (COG-UK) Consortium	Harper VanSteenhouse, Yumi Kasai, David Gray, Carol Clugston, Anna Dominiczak and Alex Alderton, Roberto Amato, Sonia Goncalves, Ewan Harrison, David K. Jackson, Ian Johnston, Dominic Kwiatkowski, Cordelia Langford, John Sillitoe on behalf of the Wellcome Sanger Institute COVID-19 Surveillance Team
EPI_ISL_777422	Lighthouse Lab in Cambridge	Wellcome Sanger Institute for the COVID-19 Genomics UK (COG-UK) Consortium	Rob Howes, The Lighthouse Lab in Cambridge and Alex Alderton, Roberto Amato, Sonia Goncalves, Ewan Harrison, David K. Jackson, Ian Johnston, Dominic Kwiatkowski, Cordelia Langford, John Sillitoe on behalf of the Wellcome Sanger Institute COVID-19 Surveillance Team
EPI_ISL_777423	Lighthouse Lab in Glasgow	Wellcome Sanger Institute for the COVID-19 Genomics UK (COG-UK) Consortium	Harper VanSteenhouse, Yumi Kasai, David Gray, Carol Clugston, Anna Dominiczak and Alex Alderton, Roberto Amato, Sonia Goncalves, Ewan Harrison, David K. Jackson, Ian Johnston, Dominic Kwiatkowski, Cordelia Langford, John Sillitoe on behalf of the Wellcome Sanger Institute COVID-19 Surveillance Team
EPI_ISL_777424	Lighthouse Lab in Alderley Park	Wellcome Sanger Institute for the COVID-19 Genomics UK (COG-UK) Consortium	Jacquelyn Wynn, Mairead Hyland, The Lighthouse Lab in Alderley Park and Alex Alderton, Roberto Amato, Sonia Goncalves, Ewan Harrison, David K. Jackson, Ian Johnston, Dominic Kwiatkowski, Cordelia Langford, John Sillitoe on behalf of the Wellcome Sanger Institute COVID-19 Surveillance Team
EPI_ISL_777426, EPI_ISL_777428, EPI_ISL_777429	Lighthouse Lab in Cambridge	Wellcome Sanger Institute for the COVID-19 Genomics UK (COG-UK) Consortium	Rob Howes, The Lighthouse Lab in Cambridge and Alex Alderton, Roberto Amato, Sonia Goncalves, Ewan Harrison, David K. Jackson, Ian Johnston, Dominic Kwiatkowski, Cordelia Langford, John Sillitoe on behalf of the Wellcome Sanger Institute COVID-19 Surveillance Team
EPI_ISL_777430	Lighthouse Lab in Alderley Park	Wellcome Sanger Institute for the COVID-19 Genomics UK (COG-UK) Consortium	Jacquelyn Wynn, Mairead Hyland, The Lighthouse Lab in Alderley Park and Alex Alderton, Roberto Amato, Sonia Goncalves, Ewan Harrison, David K. Jackson, Ian Johnston, Dominic Kwiatkowski, Cordelia Langford, John Sillitoe on behalf of the Wellcome Sanger Institute COVID-19 Surveillance Team
EPI_ISL_777431	Lighthouse Lab in Cambridge	Wellcome Sanger Institute for the COVID-19 Genomics UK (COG-UK) Consortium	Rob Howes, The Lighthouse Lab in Cambridge and Alex Alderton, Roberto Amato, Sonia Goncalves, Ewan Harrison, David K. Jackson, Ian Johnston, Dominic Kwiatkowski, Cordelia Langford, John Sillitoe on behalf of the Wellcome Sanger Institute COVID-19 Surveillance Team
EPI_ISL_777432	Lighthouse Lab in Glasgow	Wellcome Sanger Institute for the COVID-19 Genomics UK (COG-UK) Consortium	Harper VanSteenhouse, Yumi Kasai, David Gray, Carol Clugston, Anna Dominiczak and Alex Alderton, Roberto Amato, Sonia Goncalves, Ewan Harrison, David K. Jackson, Ian Johnston, Dominic Kwiatkowski, Cordelia Langford, John Sillitoe on behalf of the Wellcome Sanger Institute COVID-19 Surveillance Team
EPI_ISL_777433, EPI_ISL_777434	Lighthouse Lab in Cambridge	Wellcome Sanger Institute for the COVID-19 Genomics UK (COG-UK) Consortium	Rob Howes, The Lighthouse Lab in Cambridge and Alex Alderton, Roberto Amato, Sonia Goncalves, Ewan Harrison, David K. Jackson, Ian Johnston, Dominic Kwiatkowski, Cordelia Langford, John Sillitoe on behalf of the Wellcome Sanger Institute COVID-19 Surveillance Team
EPI_ISL_777435	Lighthouse Lab in Alderley Park	Wellcome Sanger Institute for the COVID-19 Genomics UK (COG-UK) Consortium	Jacquelyn Wynn, Mairead Hyland, The Lighthouse Lab in Alderley Park and Alex Alderton, Roberto Amato, Sonia Goncalves, Ewan Harrison, David K. Jackson, Ian Johnston, Dominic Kwiatkowski, Cordelia Langford, John Sillitoe on behalf of the Wellcome Sanger Institute COVID-19 Surveillance Team
EPI_ISL_777436	Lighthouse Lab in Glasgow	Wellcome Sanger Institute for the COVID-19 Genomics UK (COG-UK) Consortium	Harper VanSteenhouse, Yumi Kasai, David Gray, Carol Clugston, Anna Dominiczak and Alex Alderton, Roberto Amato, Sonia Goncalves, Ewan Harrison, David K. Jackson, Ian Johnston, Dominic Kwiatkowski, Cordelia Langford, John Sillitoe on behalf of the Wellcome Sanger Institute COVID-19 Surveillance Team
EPI_ISL_777437	Lighthouse Lab in Alderley Park	Wellcome Sanger Institute for the COVID-19 Genomics UK (COG-UK) Consortium	Jacquelyn Wynn, Mairead Hyland, The Lighthouse Lab in Alderley Park and Alex Alderton, Roberto Amato, Sonia Goncalves, Ewan Harrison, David K. Jackson, Ian Johnston, Dominic Kwiatkowski, Cordelia Langford, John Sillitoe on behalf of the Wellcome Sanger Institute COVID-19 Surveillance Team
EPI_ISL_777438	Lighthouse Lab in Glasgow	Wellcome Sanger Institute for the COVID-19 Genomics UK (COG-UK) Consortium	Harper VanSteenhouse, Yumi Kasai, David Gray, Carol Clugston, Anna Dominiczak and Alex Alderton, Roberto Amato, Sonia Goncalves, Ewan Harrison, David K. Jackson, Ian Johnston, Dominic Kwiatkowski, Cordelia Langford, John Sillitoe on behalf of the Wellcome Sanger Institute COVID-19 Surveillance Team

EPI_ISL_777439	Lighthouse Lab in Cambridge	Wellcome Sanger Institute for the COVID-19 Genomics UK (COG-UK) Consortium	Rob Howes, The Lighthouse Lab in Cambridge and Alex Alderton, Roberto Amato, Sonia Goncalves, Ewan Harrison, David K. Jackson, Ian Johnston, Dominic Kwiatkowski, Cordelia Langford, John Sillitoe on behalf of the Wellcome Sanger Institute COVID-19 Surveillance Team
EPI_ISL_777440	Lighthouse Lab in Alderley Park	Wellcome Sanger Institute for the COVID-19 Genomics UK (COG-UK) Consortium	Jacquelyn Wynn, Mairead Hyland, The Lighthouse Lab in Alderley Park and Alex Alderton, Roberto Amato, Sonia Goncalves, Ewan Harrison, David K. Jackson, Ian Johnston, Dominic Kwiatkowski, Cordelia Langford, John Sillitoe on behalf of the Wellcome Sanger Institute COVID-19 Surveillance Team
EPI_ISL_777442	Lighthouse Lab in Cambridge	Wellcome Sanger Institute for the COVID-19 Genomics UK (COG-UK) Consortium	Rob Howes, The Lighthouse Lab in Cambridge and Alex Alderton, Roberto Amato, Sonia Goncalves, Ewan Harrison, David K. Jackson, Ian Johnston, Dominic Kwiatkowski, Cordelia Langford, John Sillitoe on behalf of the Wellcome Sanger Institute COVID-19 Surveillance Team
EPI_ISL_777443	Lighthouse Lab in Glasgow	Wellcome Sanger Institute for the COVID-19 Genomics UK (COG-UK) Consortium	Harper VanSteenhouse, Yumi Kasai, David Gray, Carol Clugston, Anna Dominiczak and Alex Alderton, Roberto Amato, Sonia Goncalves, Ewan Harrison, David K. Jackson, Ian Johnston, Dominic Kwiatkowski, Cordelia Langford, John Sillitoe on behalf of the Wellcome Sanger Institute COVID-19 Surveillance Team
EPI_ISL_777445, EPI_ISL_777446	Lighthouse Lab in Cambridge	Wellcome Sanger Institute for the COVID-19 Genomics UK (COG-UK) Consortium	Rob Howes, The Lighthouse Lab in Cambridge and Alex Alderton, Roberto Amato, Sonia Goncalves, Ewan Harrison, David K. Jackson, Ian Johnston, Dominic Kwiatkowski, Cordelia Langford, John Sillitoe on behalf of the Wellcome Sanger Institute COVID-19 Surveillance Team
EPI_ISL_777447	Lighthouse Lab in Glasgow	Wellcome Sanger Institute for the COVID-19 Genomics UK (COG-UK) Consortium	Harper VanSteenhouse, Yumi Kasai, David Gray, Carol Clugston, Anna Dominiczak and Alex Alderton, Roberto Amato, Sonia Goncalves, Ewan Harrison, David K. Jackson, Ian Johnston, Dominic Kwiatkowski, Cordelia Langford, John Sillitoe on behalf of the Wellcome Sanger Institute COVID-19 Surveillance Team
EPI_ISL_777448, EPI_ISL_777451	Lighthouse Lab in Alderley Park	Wellcome Sanger Institute for the COVID-19 Genomics UK (COG-UK) Consortium	Jacquelyn Wynn, Mairead Hyland, The Lighthouse Lab in Alderley Park and Alex Alderton, Roberto Amato, Sonia Goncalves, Ewan Harrison, David K. Jackson, Ian Johnston, Dominic Kwiatkowski, Cordelia Langford, John Sillitoe on behalf of the Wellcome Sanger Institute COVID-19 Surveillance Team
EPI_ISL_777452, EPI_ISL_777453, EPI_ISL_777454, EPI_ISL_777456, EPI_ISL_777457	Lighthouse Lab in Cambridge	Wellcome Sanger Institute for the COVID-19 Genomics UK (COG-UK) Consortium	Rob Howes, The Lighthouse Lab in Cambridge and Alex Alderton, Roberto Amato, Sonia Goncalves, Ewan Harrison, David K. Jackson, Ian Johnston, Dominic Kwiatkowski, Cordelia Langford, John Sillitoe on behalf of the Wellcome Sanger Institute COVID-19 Surveillance Team
EPI_ISL_777458	Lighthouse Lab in Alderley Park	Wellcome Sanger Institute for the COVID-19 Genomics UK (COG-UK) Consortium	Jacquelyn Wynn, Mairead Hyland, The Lighthouse Lab in Alderley Park and Alex Alderton, Roberto Amato, Sonia Goncalves, Ewan Harrison, David K. Jackson, Ian Johnston, Dominic Kwiatkowski, Cordelia Langford, John Sillitoe on behalf of the Wellcome Sanger Institute COVID-19 Surveillance Team
EPI_ISL_777460	Lighthouse Lab in Cambridge	Wellcome Sanger Institute for the COVID-19 Genomics UK (COG-UK) Consortium	Rob Howes, The Lighthouse Lab in Cambridge and Alex Alderton, Roberto Amato, Sonia Goncalves, Ewan Harrison, David K. Jackson, Ian Johnston, Dominic Kwiatkowski, Cordelia Langford, John Sillitoe on behalf of the Wellcome Sanger Institute COVID-19 Surveillance Team
EPI_ISL_777461, EPI_ISL_777462	Lighthouse Lab in Glasgow	Wellcome Sanger Institute for the COVID-19 Genomics UK (COG-UK) Consortium	Harper VanSteenhouse, Yumi Kasai, David Gray, Carol Clugston, Anna Dominiczak and Alex Alderton, Roberto Amato, Sonia Goncalves, Ewan Harrison, David K. Jackson, Ian Johnston, Dominic Kwiatkowski, Cordelia Langford, John Sillitoe on behalf of the Wellcome Sanger Institute COVID-19 Surveillance Team
EPI_ISL_777464	Lighthouse Lab in Cambridge	Wellcome Sanger Institute for the COVID-19 Genomics UK (COG-UK) Consortium	Rob Howes, The Lighthouse Lab in Cambridge and Alex Alderton, Roberto Amato, Sonia Goncalves, Ewan Harrison, David K. Jackson, Ian Johnston, Dominic Kwiatkowski, Cordelia Langford, John Sillitoe on behalf of the Wellcome Sanger Institute COVID-19 Surveillance Team
EPI_ISL_777465, EPI_ISL_777467, EPI_ISL_777470	Lighthouse Lab in Alderley Park	Wellcome Sanger Institute for the COVID-19 Genomics UK (COG-UK) Consortium	Jacquelyn Wynn, Mairead Hyland, The Lighthouse Lab in Alderley Park and Alex Alderton, Roberto Amato, Sonia Goncalves, Ewan Harrison, David K. Jackson, Ian Johnston, Dominic Kwiatkowski, Cordelia Langford, John Sillitoe on behalf of the Wellcome Sanger Institute COVID-19 Surveillance Team
EPI_ISL_777471	Lighthouse Lab in Glasgow	Wellcome Sanger Institute for the COVID-19 Genomics UK (COG-UK) Consortium	Harper VanSteenhouse, Yumi Kasai, David Gray, Carol Clugston, Anna Dominiczak and Alex Alderton, Roberto Amato, Sonia Goncalves, Ewan Harrison, David K. Jackson, Ian Johnston, Dominic Kwiatkowski, Cordelia Langford, John Sillitoe on behalf of the Wellcome Sanger Institute COVID-19 Surveillance Team
EPI_ISL_777473	Lighthouse Lab in Cambridge	Wellcome Sanger Institute for the COVID-19 Genomics UK (COG-UK) Consortium	Rob Howes, The Lighthouse Lab in Cambridge and Alex Alderton, Roberto Amato, Sonia Goncalves, Ewan Harrison, David K. Jackson, Ian Johnston, Dominic Kwiatkowski, Cordelia Langford, John Sillitoe on behalf of the Wellcome Sanger Institute COVID-19 Surveillance Team
EPI_ISL_777474	Lighthouse Lab in Glasgow	Wellcome Sanger Institute for the COVID-19 Genomics UK (COG-UK) Consortium	Harper VanSteenhouse, Yumi Kasai, David Gray, Carol Clugston, Anna Dominiczak and Alex Alderton, Roberto Amato, Sonia Goncalves, Ewan Harrison, David K. Jackson, Ian Johnston, Dominic Kwiatkowski, Cordelia Langford, John Sillitoe on behalf of the Wellcome Sanger Institute COVID-19 Surveillance Team
EPI_ISL_777476	Lighthouse Lab in Cambridge	Wellcome Sanger Institute for the COVID-19 Genomics UK (COG-UK) Consortium	Rob Howes, The Lighthouse Lab in Cambridge and Alex Alderton, Roberto Amato, Sonia Goncalves, Ewan Harrison, David K. Jackson, Ian Johnston, Dominic Kwiatkowski, Cordelia Langford, John Sillitoe on behalf of the Wellcome Sanger Institute COVID-19 Surveillance Team
EPI_ISL_777477	Lighthouse Lab in Alderley Park	Wellcome Sanger Institute for the COVID-19 Genomics UK (COG-UK) Consortium	Jacquelyn Wynn, Mairead Hyland, The Lighthouse Lab in Alderley Park and Alex Alderton, Roberto Amato, Sonia Goncalves, Ewan Harrison, David K. Jackson, Ian Johnston, Dominic Kwiatkowski, Cordelia Langford, John Sillitoe on behalf of the Wellcome Sanger Institute COVID-19 Surveillance Team
EPI_ISL_777478	Lighthouse Lab in Cambridge	Wellcome Sanger Institute for the COVID-19 Genomics UK (COG-UK) Consortium	Rob Howes, The Lighthouse Lab in Cambridge and Alex Alderton, Roberto Amato, Sonia Goncalves, Ewan Harrison, David K. Jackson, Ian Johnston, Dominic Kwiatkowski, Cordelia Langford, John Sillitoe on behalf of the Wellcome Sanger Institute COVID-19 Surveillance Team
EPI_ISL_777479	Lighthouse Lab in Alderley Park	Wellcome Sanger Institute for the COVID-19 Genomics UK (COG-UK) Consortium	Jacquelyn Wynn, Mairead Hyland, The Lighthouse Lab in Alderley Park and Alex Alderton, Roberto Amato, Sonia Goncalves, Ewan Harrison, David K. Jackson, Ian Johnston, Dominic Kwiatkowski, Cordelia Langford, John Sillitoe on behalf of the Wellcome Sanger Institute COVID-19 Surveillance Team
EPI_ISL_777480	Lighthouse Lab in Cambridge	Wellcome Sanger Institute for the COVID-19 Genomics UK (COG-UK) Consortium	Rob Howes, The Lighthouse Lab in Cambridge and Alex Alderton, Roberto Amato, Sonia Goncalves, Ewan Harrison, David K. Jackson, Ian Johnston, Dominic Kwiatkowski, Cordelia Langford, John Sillitoe on behalf of the Wellcome Sanger Institute COVID-19 Surveillance Team
EPI_ISL_777481	Lighthouse Lab in Alderley Park	Wellcome Sanger Institute for the COVID-19 Genomics UK (COG-UK) Consortium	Jacquelyn Wynn, Mairead Hyland, The Lighthouse Lab in Alderley Park and Alex Alderton, Roberto Amato, Sonia Goncalves, Ewan Harrison, David K. Jackson, Ian Johnston, Dominic Kwiatkowski, Cordelia Langford, John Sillitoe on behalf of the Wellcome Sanger Institute COVID-19 Surveillance Team
EPI_ISL_777482	Lighthouse Lab in Cambridge	Wellcome Sanger Institute for the COVID-19 Genomics UK (COG-UK) Consortium	Rob Howes, The Lighthouse Lab in Cambridge and Alex Alderton, Roberto Amato, Sonia Goncalves, Ewan Harrison, David K. Jackson, Ian Johnston, Dominic Kwiatkowski, Cordelia Langford, John Sillitoe on behalf of the Wellcome Sanger Institute COVID-19 Surveillance Team
EPI_ISL_777484	Lighthouse Lab in Glasgow	Wellcome Sanger Institute for the COVID-19 Genomics UK (COG-UK) Consortium	Harper VanSteenhouse, Yumi Kasai, David Gray, Carol Clugston, Anna Dominiczak and Alex Alderton, Roberto Amato, Sonia Goncalves, Ewan Harrison, David K. Jackson, Ian Johnston, Dominic Kwiatkowski, Cordelia Langford, John Sillitoe on behalf of the Wellcome Sanger Institute COVID-19 Surveillance Team
EPI_ISL_777486	Lighthouse Lab in Cambridge	Wellcome Sanger Institute for the COVID-19 Genomics UK (COG-UK) Consortium	Rob Howes, The Lighthouse Lab in Cambridge and Alex Alderton, Roberto Amato, Sonia Goncalves, Ewan Harrison, David K. Jackson, Ian Johnston, Dominic Kwiatkowski, Cordelia Langford, John Sillitoe on behalf of the Wellcome Sanger Institute COVID-19 Surveillance Team
EPI_ISL_777489, EPI_ISL_777491	Lighthouse Lab in Alderley Park	Wellcome Sanger Institute for the COVID-19 Genomics UK (COG-UK) Consortium	Jacquelyn Wynn, Mairead Hyland, The Lighthouse Lab in Alderley Park and Alex Alderton, Roberto Amato, Sonia Goncalves, Ewan Harrison, David K. Jackson, Ian Johnston, Dominic Kwiatkowski, Cordelia Langford, John Sillitoe on behalf of the Wellcome Sanger Institute COVID-19 Surveillance Team
EPI_ISL_777492	Lighthouse Lab in Cambridge	Wellcome Sanger Institute for the COVID-19 Genomics UK (COG-UK) Consortium	Rob Howes, The Lighthouse Lab in Cambridge and Alex Alderton, Roberto Amato, Sonia Goncalves, Ewan Harrison, David K. Jackson, Ian Johnston, Dominic Kwiatkowski, Cordelia Langford, John Sillitoe on behalf of the Wellcome Sanger Institute COVID-19 Surveillance Team
EPI_ISL_777493	Lighthouse Lab in Glasgow	Wellcome Sanger Institute for the COVID-19 Genomics UK (COG-UK) Consortium	Harper VanSteenhouse, Yumi Kasai, David Gray, Carol Clugston, Anna Dominiczak and Alex Alderton, Roberto Amato, Sonia Goncalves, Ewan Harrison, David K. Jackson, Ian Johnston, Dominic Kwiatkowski, Cordelia Langford, John Sillitoe on behalf of the Wellcome Sanger Institute COVID-19 Surveillance Team
EPI_ISL_777494	Lighthouse Lab in Cambridge	Wellcome Sanger Institute for the COVID-19 Genomics UK (COG-UK) Consortium	Rob Howes, The Lighthouse Lab in Cambridge and Alex Alderton, Roberto Amato, Sonia Goncalves, Ewan Harrison, David K. Jackson, Ian Johnston, Dominic Kwiatkowski, Cordelia Langford, John Sillitoe on behalf of the Wellcome Sanger Institute COVID-19 Surveillance Team
EPI_ISL_777496, EPI_ISL_777497, EPI_ISL_777498	Lighthouse Lab in Glasgow	Wellcome Sanger Institute for the COVID-19 Genomics UK (COG-UK) Consortium	Harper VanSteenhouse, Yumi Kasai, David Gray, Carol Clugston, Anna Dominiczak and Alex Alderton, Roberto Amato, Sonia Goncalves, Ewan Harrison, David K. Jackson, Ian Johnston, Dominic Kwiatkowski, Cordelia Langford, John Sillitoe on behalf of the Wellcome Sanger Institute COVID-19 Surveillance Team
EPI_ISL_777499, EPI_ISL_777500	Lighthouse Lab in Cambridge	Wellcome Sanger Institute for the COVID-19 Genomics UK (COG-UK) Consortium	Rob Howes, The Lighthouse Lab in Cambridge and Alex Alderton, Roberto Amato, Sonia Goncalves, Ewan Harrison, David K. Jackson, Ian Johnston, Dominic Kwiatkowski, Cordelia Langford, John Sillitoe on behalf of the Wellcome Sanger Institute COVID-19 Surveillance Team
EPI_ISL_777502	Lighthouse Lab in Alderley Park	Wellcome Sanger Institute for the COVID-19 Genomics UK	Jacquelyn Wynn, Mairead Hyland, The Lighthouse Lab in Alderley Park and Alex Alderton, Roberto Amato, Sonia Goncalves, Ewan Harrison, David K.

		(COG-UK) Consortium	Jackson, Ian Johnston, Dominic Kwiatkowski, Cordelia Langford, John Sillitoe on behalf of the Wellcome Sanger Institute COVID-19 Surveillance Team
EPI_ISL_777503	Lighthouse Lab in Cambridge	Wellcome Sanger Institute for the COVID-19 Genomics UK (COG-UK) Consortium	Rob Howes, The Lighthouse Lab in Cambridge and Alex Alderton, Roberto Amato, Sonia Goncalves, Ewan Harrison, David K. Jackson, Ian Johnston, Dominic Kwiatkowski, Cordelia Langford, John Sillitoe on behalf of the Wellcome Sanger Institute COVID-19 Surveillance Team
EPI_ISL_777504, EPI_ISL_777505, EPI_ISL_777506	Lighthouse Lab in Glasgow	Wellcome Sanger Institute for the COVID-19 Genomics UK (COG-UK) Consortium	Harper VanSteenhouse, Yumi Kasai, David Gray, Carol Clugston, Anna Dominiczak and Alex Alderton, Roberto Amato, Sonia Goncalves, Ewan Harrison, David K. Jackson, Ian Johnston, Dominic Kwiatkowski, Cordelia Langford, John Sillitoe on behalf of the Wellcome Sanger Institute COVID-19 Surveillance Team
EPI_ISL_777507, EPI_ISL_777508	Lighthouse Lab in Cambridge	Wellcome Sanger Institute for the COVID-19 Genomics UK (COG-UK) Consortium	Rob Howes, The Lighthouse Lab in Cambridge and Alex Alderton, Roberto Amato, Sonia Goncalves, Ewan Harrison, David K. Jackson, Ian Johnston, Dominic Kwiatkowski, Cordelia Langford, John Sillitoe on behalf of the Wellcome Sanger Institute COVID-19 Surveillance Team
EPI_ISL_777510	Lighthouse Lab in Glasgow	Wellcome Sanger Institute for the COVID-19 Genomics UK (COG-UK) Consortium	Harper VanSteenhouse, Yumi Kasai, David Gray, Carol Clugston, Anna Dominiczak and Alex Alderton, Roberto Amato, Sonia Goncalves, Ewan Harrison, David K. Jackson, Ian Johnston, Dominic Kwiatkowski, Cordelia Langford, John Sillitoe on behalf of the Wellcome Sanger Institute COVID-19 Surveillance Team
EPI_ISL_777511, EPI_ISL_777512, EPI_ISL_777513	Lighthouse Lab in Cambridge	Wellcome Sanger Institute for the COVID-19 Genomics UK (COG-UK) Consortium	Rob Howes, The Lighthouse Lab in Cambridge and Alex Alderton, Roberto Amato, Sonia Goncalves, Ewan Harrison, David K. Jackson, Ian Johnston, Dominic Kwiatkowski, Cordelia Langford, John Sillitoe on behalf of the Wellcome Sanger Institute COVID-19 Surveillance Team
EPI_ISL_777514	Lighthouse Lab in Glasgow	Wellcome Sanger Institute for the COVID-19 Genomics UK (COG-UK) Consortium	Harper VanSteenhouse, Yumi Kasai, David Gray, Carol Clugston, Anna Dominiczak and Alex Alderton, Roberto Amato, Sonia Goncalves, Ewan Harrison, David K. Jackson, Ian Johnston, Dominic Kwiatkowski, Cordelia Langford, John Sillitoe on behalf of the Wellcome Sanger Institute COVID-19 Surveillance Team
EPI_ISL_777516, EPI_ISL_777517, EPI_ISL_777519, EPI_ISL_777521	Lighthouse Lab in Cambridge	Wellcome Sanger Institute for the COVID-19 Genomics UK (COG-UK) Consortium	Rob Howes, The Lighthouse Lab in Cambridge and Alex Alderton, Roberto Amato, Sonia Goncalves, Ewan Harrison, David K. Jackson, Ian Johnston, Dominic Kwiatkowski, Cordelia Langford, John Sillitoe on behalf of the Wellcome Sanger Institute COVID-19 Surveillance Team
EPI_ISL_777523	Lighthouse Lab in Glasgow	Wellcome Sanger Institute for the COVID-19 Genomics UK (COG-UK) Consortium	Harper VanSteenhouse, Yumi Kasai, David Gray, Carol Clugston, Anna Dominiczak and Alex Alderton, Roberto Amato, Sonia Goncalves, Ewan Harrison, David K. Jackson, Ian Johnston, Dominic Kwiatkowski, Cordelia Langford, John Sillitoe on behalf of the Wellcome Sanger Institute COVID-19 Surveillance Team
EPI_ISL_777524	Lighthouse Lab in Alderley Park	Wellcome Sanger Institute for the COVID-19 Genomics UK (COG-UK) Consortium	Jacquelyn Wynn, Mairead Hyland, The Lighthouse Lab in Alderley Park and Alex Alderton, Roberto Amato, Sonia Goncalves, Ewan Harrison, David K. Jackson, Ian Johnston, Dominic Kwiatkowski, Cordelia Langford, John Sillitoe on behalf of the Wellcome Sanger Institute COVID-19 Surveillance Team
EPI_ISL_777526	Lighthouse Lab in Glasgow	Wellcome Sanger Institute for the COVID-19 Genomics UK (COG-UK) Consortium	Harper VanSteenhouse, Yumi Kasai, David Gray, Carol Clugston, Anna Dominiczak and Alex Alderton, Roberto Amato, Sonia Goncalves, Ewan Harrison, David K. Jackson, Ian Johnston, Dominic Kwiatkowski, Cordelia Langford, John Sillitoe on behalf of the Wellcome Sanger Institute COVID-19 Surveillance Team
EPI_ISL_777528	Lighthouse Lab in Cambridge	Wellcome Sanger Institute for the COVID-19 Genomics UK (COG-UK) Consortium	Rob Howes, The Lighthouse Lab in Cambridge and Alex Alderton, Roberto Amato, Sonia Goncalves, Ewan Harrison, David K. Jackson, Ian Johnston, Dominic Kwiatkowski, Cordelia Langford, John Sillitoe on behalf of the Wellcome Sanger Institute COVID-19 Surveillance Team
EPI_ISL_777530	Lighthouse Lab in Alderley Park	Wellcome Sanger Institute for the COVID-19 Genomics UK (COG-UK) Consortium	Jacquelyn Wynn, Mairead Hyland, The Lighthouse Lab in Alderley Park and Alex Alderton, Roberto Amato, Sonia Goncalves, Ewan Harrison, David K. Jackson, Ian Johnston, Dominic Kwiatkowski, Cordelia Langford, John Sillitoe on behalf of the Wellcome Sanger Institute COVID-19 Surveillance Team
EPI_ISL_777531	Lighthouse Lab in Glasgow	Wellcome Sanger Institute for the COVID-19 Genomics UK (COG-UK) Consortium	Harper VanSteenhouse, Yumi Kasai, David Gray, Carol Clugston, Anna Dominiczak and Alex Alderton, Roberto Amato, Sonia Goncalves, Ewan Harrison, David K. Jackson, Ian Johnston, Dominic Kwiatkowski, Cordelia Langford, John Sillitoe on behalf of the Wellcome Sanger Institute COVID-19 Surveillance Team
EPI_ISL_777532	Lighthouse Lab in Alderley Park	Wellcome Sanger Institute for the COVID-19 Genomics UK (COG-UK) Consortium	Jacquelyn Wynn, Mairead Hyland, The Lighthouse Lab in Alderley Park and Alex Alderton, Roberto Amato, Sonia Goncalves, Ewan Harrison, David K. Jackson, Ian Johnston, Dominic Kwiatkowski, Cordelia Langford, John Sillitoe on behalf of the Wellcome Sanger Institute COVID-19 Surveillance Team
EPI_ISL_777533	Lighthouse Lab in Glasgow	Wellcome Sanger Institute for the COVID-19 Genomics UK (COG-UK) Consortium	Harper VanSteenhouse, Yumi Kasai, David Gray, Carol Clugston, Anna Dominiczak and Alex Alderton, Roberto Amato, Sonia Goncalves, Ewan Harrison, David K. Jackson, Ian Johnston, Dominic Kwiatkowski, Cordelia Langford, John Sillitoe on behalf of the Wellcome Sanger Institute COVID-19 Surveillance Team
EPI_ISL_777534, EPI_ISL_777535	Lighthouse Lab in Alderley Park	Wellcome Sanger Institute for the COVID-19 Genomics UK (COG-UK) Consortium	Jacquelyn Wynn, Mairead Hyland, The Lighthouse Lab in Alderley Park and Alex Alderton, Roberto Amato, Sonia Goncalves, Ewan Harrison, David K. Jackson, Ian Johnston, Dominic Kwiatkowski, Cordelia Langford, John Sillitoe on behalf of the Wellcome Sanger Institute COVID-19 Surveillance Team
EPI_ISL_777536	Lighthouse Lab in Cambridge	Wellcome Sanger Institute for the COVID-19 Genomics UK (COG-UK) Consortium	Rob Howes, The Lighthouse Lab in Cambridge and Alex Alderton, Roberto Amato, Sonia Goncalves, Ewan Harrison, David K. Jackson, Ian Johnston, Dominic Kwiatkowski, Cordelia Langford, John Sillitoe on behalf of the Wellcome Sanger Institute COVID-19 Surveillance Team
EPI_ISL_777538, EPI_ISL_777539	Lighthouse Lab in Alderley Park	Wellcome Sanger Institute for the COVID-19 Genomics UK (COG-UK) Consortium	Jacquelyn Wynn, Mairead Hyland, The Lighthouse Lab in Alderley Park and Alex Alderton, Roberto Amato, Sonia Goncalves, Ewan Harrison, David K. Jackson, Ian Johnston, Dominic Kwiatkowski, Cordelia Langford, John Sillitoe on behalf of the Wellcome Sanger Institute COVID-19 Surveillance Team
EPI_ISL_777541, EPI_ISL_777542, EPI_ISL_777545, EPI_ISL_777546, EPI_ISL_777547	Lighthouse Lab in Cambridge	Wellcome Sanger Institute for the COVID-19 Genomics UK (COG-UK) Consortium	Rob Howes, The Lighthouse Lab in Cambridge and Alex Alderton, Roberto Amato, Sonia Goncalves, Ewan Harrison, David K. Jackson, Ian Johnston, Dominic Kwiatkowski, Cordelia Langford, John Sillitoe on behalf of the Wellcome Sanger Institute COVID-19 Surveillance Team
EPI_ISL_777548	Lighthouse Lab in Alderley Park	Wellcome Sanger Institute for the COVID-19 Genomics UK (COG-UK) Consortium	Jacquelyn Wynn, Mairead Hyland, The Lighthouse Lab in Alderley Park and Alex Alderton, Roberto Amato, Sonia Goncalves, Ewan Harrison, David K. Jackson, Ian Johnston, Dominic Kwiatkowski, Cordelia Langford, John Sillitoe on behalf of the Wellcome Sanger Institute COVID-19 Surveillance Team
EPI_ISL_777549, EPI_ISL_777551	Lighthouse Lab in Cambridge	Wellcome Sanger Institute for the COVID-19 Genomics UK (COG-UK) Consortium	Rob Howes, The Lighthouse Lab in Cambridge and Alex Alderton, Roberto Amato, Sonia Goncalves, Ewan Harrison, David K. Jackson, Ian Johnston, Dominic Kwiatkowski, Cordelia Langford, John Sillitoe on behalf of the Wellcome Sanger Institute COVID-19 Surveillance Team
EPI_ISL_777552	Lighthouse Lab in Alderley Park	Wellcome Sanger Institute for the COVID-19 Genomics UK (COG-UK) Consortium	Jacquelyn Wynn, Mairead Hyland, The Lighthouse Lab in Alderley Park and Alex Alderton, Roberto Amato, Sonia Goncalves, Ewan Harrison, David K. Jackson, Ian Johnston, Dominic Kwiatkowski, Cordelia Langford, John Sillitoe on behalf of the Wellcome Sanger Institute COVID-19 Surveillance Team
EPI_ISL_777553	Lighthouse Lab in Cambridge	Wellcome Sanger Institute for the COVID-19 Genomics UK (COG-UK) Consortium	Rob Howes, The Lighthouse Lab in Cambridge and Alex Alderton, Roberto Amato, Sonia Goncalves, Ewan Harrison, David K. Jackson, Ian Johnston, Dominic Kwiatkowski, Cordelia Langford, John Sillitoe on behalf of the Wellcome Sanger Institute COVID-19 Surveillance Team
EPI_ISL_777554, EPI_ISL_777557	Lighthouse Lab in Glasgow	Wellcome Sanger Institute for the COVID-19 Genomics UK (COG-UK) Consortium	Harper VanSteenhouse, Yumi Kasai, David Gray, Carol Clugston, Anna Dominiczak and Alex Alderton, Roberto Amato, Sonia Goncalves, Ewan Harrison, David K. Jackson, Ian Johnston, Dominic Kwiatkowski, Cordelia Langford, John Sillitoe on behalf of the Wellcome Sanger Institute COVID-19 Surveillance Team
EPI_ISL_777558	Lighthouse Lab in Alderley Park	Wellcome Sanger Institute for the COVID-19 Genomics UK (COG-UK) Consortium	Jacquelyn Wynn, Mairead Hyland, The Lighthouse Lab in Alderley Park and Alex Alderton, Roberto Amato, Sonia Goncalves, Ewan Harrison, David K. Jackson, Ian Johnston, Dominic Kwiatkowski, Cordelia Langford, John Sillitoe on behalf of the Wellcome Sanger Institute COVID-19 Surveillance Team
EPI_ISL_777559	Lighthouse Lab in Cambridge	Wellcome Sanger Institute for the COVID-19 Genomics UK (COG-UK) Consortium	Rob Howes, The Lighthouse Lab in Cambridge and Alex Alderton, Roberto Amato, Sonia Goncalves, Ewan Harrison, David K. Jackson, Ian Johnston, Dominic Kwiatkowski, Cordelia Langford, John Sillitoe on behalf of the Wellcome Sanger Institute COVID-19 Surveillance Team
EPI_ISL_777560	Lighthouse Lab in Glasgow	Wellcome Sanger Institute for the COVID-19 Genomics UK (COG-UK) Consortium	Harper VanSteenhouse, Yumi Kasai, David Gray, Carol Clugston, Anna Dominiczak and Alex Alderton, Roberto Amato, Sonia Goncalves, Ewan Harrison, David K. Jackson, Ian Johnston, Dominic Kwiatkowski, Cordelia Langford, John Sillitoe on behalf of the Wellcome Sanger Institute COVID-19 Surveillance Team
EPI_ISL_777561	Lighthouse Lab in Alderley Park	Wellcome Sanger Institute for the COVID-19 Genomics UK (COG-UK) Consortium	Jacquelyn Wynn, Mairead Hyland, The Lighthouse Lab in Alderley Park and Alex Alderton, Roberto Amato, Sonia Goncalves, Ewan Harrison, David K. Jackson, Ian Johnston, Dominic Kwiatkowski, Cordelia Langford, John Sillitoe on behalf of the Wellcome Sanger Institute COVID-19 Surveillance Team
EPI_ISL_777562	Lighthouse Lab in Glasgow	Wellcome Sanger Institute for the COVID-19 Genomics UK (COG-UK) Consortium	Harper VanSteenhouse, Yumi Kasai, David Gray, Carol Clugston, Anna Dominiczak and Alex Alderton, Roberto Amato, Sonia Goncalves, Ewan Harrison, David K. Jackson, Ian Johnston, Dominic Kwiatkowski, Cordelia Langford, John Sillitoe on behalf of the Wellcome Sanger Institute COVID-19 Surveillance Team
EPI_ISL_777563	Lighthouse Lab in Alderley Park	Wellcome Sanger Institute for the COVID-19 Genomics UK (COG-UK) Consortium	Jacquelyn Wynn, Mairead Hyland, The Lighthouse Lab in Alderley Park and Alex Alderton, Roberto Amato, Sonia Goncalves, Ewan Harrison, David K. Jackson, Ian Johnston, Dominic Kwiatkowski, Cordelia Langford, John Sillitoe on behalf of the Wellcome Sanger Institute COVID-19 Surveillance Team

EPI_ISL_777565, EPI_ISL_777566	Lighthouse Lab in Cambridge	Wellcome Sanger Institute for the COVID-19 Genomics UK (COG-UK) Consortium	Rob Howes, The Lighthouse Lab in Cambridge and Alex Alderton, Roberto Amato, Sonia Goncalves, Ewan Harrison, David K. Jackson, Ian Johnston, Dominic Kwiatkowski, Cordelia Langford, John Sillitoe on behalf of the Wellcome Sanger Institute COVID-19 Surveillance Team
EPI_ISL_777567, EPI_ISL_777568	Lighthouse Lab in Alderley Park	Wellcome Sanger Institute for the COVID-19 Genomics UK (COG-UK) Consortium	Jacquelyn Wynn, Mairead Hyland, The Lighthouse Lab in Alderley Park and Alex Alderton, Roberto Amato, Sonia Goncalves, Ewan Harrison, David K. Jackson, Ian Johnston, Dominic Kwiatkowski, Cordelia Langford, John Sillitoe on behalf of the Wellcome Sanger Institute COVID-19 Surveillance Team
EPI_ISL_777570, EPI_ISL_777571	Lighthouse Lab in Cambridge	Wellcome Sanger Institute for the COVID-19 Genomics UK (COG-UK) Consortium	Rob Howes, The Lighthouse Lab in Cambridge and Alex Alderton, Roberto Amato, Sonia Goncalves, Ewan Harrison, David K. Jackson, Ian Johnston, Dominic Kwiatkowski, Cordelia Langford, John Sillitoe on behalf of the Wellcome Sanger Institute COVID-19 Surveillance Team
EPI_ISL_777573	Lighthouse Lab in Alderley Park	Wellcome Sanger Institute for the COVID-19 Genomics UK (COG-UK) Consortium	Jacquelyn Wynn, Mairead Hyland, The Lighthouse Lab in Alderley Park and Alex Alderton, Roberto Amato, Sonia Goncalves, Ewan Harrison, David K. Jackson, Ian Johnston, Dominic Kwiatkowski, Cordelia Langford, John Sillitoe on behalf of the Wellcome Sanger Institute COVID-19 Surveillance Team
EPI_ISL_777575	Lighthouse Lab in Cambridge	Wellcome Sanger Institute for the COVID-19 Genomics UK (COG-UK) Consortium	Rob Howes, The Lighthouse Lab in Cambridge and Alex Alderton, Roberto Amato, Sonia Goncalves, Ewan Harrison, David K. Jackson, Ian Johnston, Dominic Kwiatkowski, Cordelia Langford, John Sillitoe on behalf of the Wellcome Sanger Institute COVID-19 Surveillance Team
EPI_ISL_777576	Lighthouse Lab in Alderley Park	Wellcome Sanger Institute for the COVID-19 Genomics UK (COG-UK) Consortium	Jacquelyn Wynn, Mairead Hyland, The Lighthouse Lab in Alderley Park and Alex Alderton, Roberto Amato, Sonia Goncalves, Ewan Harrison, David K. Jackson, Ian Johnston, Dominic Kwiatkowski, Cordelia Langford, John Sillitoe on behalf of the Wellcome Sanger Institute COVID-19 Surveillance Team
EPI_ISL_777577, EPI_ISL_777578	Lighthouse Lab in Cambridge	Wellcome Sanger Institute for the COVID-19 Genomics UK (COG-UK) Consortium	Rob Howes, The Lighthouse Lab in Cambridge and Alex Alderton, Roberto Amato, Sonia Goncalves, Ewan Harrison, David K. Jackson, Ian Johnston, Dominic Kwiatkowski, Cordelia Langford, John Sillitoe on behalf of the Wellcome Sanger Institute COVID-19 Surveillance Team
EPI_ISL_777580	Lighthouse Lab in Alderley Park	Wellcome Sanger Institute for the COVID-19 Genomics UK (COG-UK) Consortium	Jacquelyn Wynn, Mairead Hyland, The Lighthouse Lab in Alderley Park and Alex Alderton, Roberto Amato, Sonia Goncalves, Ewan Harrison, David K. Jackson, Ian Johnston, Dominic Kwiatkowski, Cordelia Langford, John Sillitoe on behalf of the Wellcome Sanger Institute COVID-19 Surveillance Team
EPI_ISL_777581	Lighthouse Lab in Cambridge	Wellcome Sanger Institute for the COVID-19 Genomics UK (COG-UK) Consortium	Rob Howes, The Lighthouse Lab in Cambridge and Alex Alderton, Roberto Amato, Sonia Goncalves, Ewan Harrison, David K. Jackson, Ian Johnston, Dominic Kwiatkowski, Cordelia Langford, John Sillitoe on behalf of the Wellcome Sanger Institute COVID-19 Surveillance Team
EPI_ISL_777582	Lighthouse Lab in Alderley Park	Wellcome Sanger Institute for the COVID-19 Genomics UK (COG-UK) Consortium	Jacquelyn Wynn, Mairead Hyland, The Lighthouse Lab in Alderley Park and Alex Alderton, Roberto Amato, Sonia Goncalves, Ewan Harrison, David K. Jackson, Ian Johnston, Dominic Kwiatkowski, Cordelia Langford, John Sillitoe on behalf of the Wellcome Sanger Institute COVID-19 Surveillance Team
EPI_ISL_777583	Lighthouse Lab in Glasgow	Wellcome Sanger Institute for the COVID-19 Genomics UK (COG-UK) Consortium	Harper VanSteenhouse, Yumi Kasai, David Gray, Carol Clugston, Anna Dominiczak and Alex Alderton, Roberto Amato, Sonia Goncalves, Ewan Harrison, David K. Jackson, Ian Johnston, Dominic Kwiatkowski, Cordelia Langford, John Sillitoe on behalf of the Wellcome Sanger Institute COVID-19 Surveillance Team
EPI_ISL_777584	Lighthouse Lab in Alderley Park	Wellcome Sanger Institute for the COVID-19 Genomics UK (COG-UK) Consortium	Jacquelyn Wynn, Mairead Hyland, The Lighthouse Lab in Alderley Park and Alex Alderton, Roberto Amato, Sonia Goncalves, Ewan Harrison, David K. Jackson, Ian Johnston, Dominic Kwiatkowski, Cordelia Langford, John Sillitoe on behalf of the Wellcome Sanger Institute COVID-19 Surveillance Team
EPI_ISL_777585, EPI_ISL_777586	Lighthouse Lab in Cambridge	Wellcome Sanger Institute for the COVID-19 Genomics UK (COG-UK) Consortium	Rob Howes, The Lighthouse Lab in Cambridge and Alex Alderton, Roberto Amato, Sonia Goncalves, Ewan Harrison, David K. Jackson, Ian Johnston, Dominic Kwiatkowski, Cordelia Langford, John Sillitoe on behalf of the Wellcome Sanger Institute COVID-19 Surveillance Team
EPI_ISL_777587	Lighthouse Lab in Alderley Park	Wellcome Sanger Institute for the COVID-19 Genomics UK (COG-UK) Consortium	Jacquelyn Wynn, Mairead Hyland, The Lighthouse Lab in Alderley Park and Alex Alderton, Roberto Amato, Sonia Goncalves, Ewan Harrison, David K. Jackson, Ian Johnston, Dominic Kwiatkowski, Cordelia Langford, John Sillitoe on behalf of the Wellcome Sanger Institute COVID-19 Surveillance Team
EPI_ISL_777590	Lighthouse Lab in Cambridge	Wellcome Sanger Institute for the COVID-19 Genomics UK (COG-UK) Consortium	Rob Howes, The Lighthouse Lab in Cambridge and Alex Alderton, Roberto Amato, Sonia Goncalves, Ewan Harrison, David K. Jackson, Ian Johnston, Dominic Kwiatkowski, Cordelia Langford, John Sillitoe on behalf of the Wellcome Sanger Institute COVID-19 Surveillance Team
EPI_ISL_777592	Lighthouse Lab in Alderley Park	Wellcome Sanger Institute for the COVID-19 Genomics UK (COG-UK) Consortium	Jacquelyn Wynn, Mairead Hyland, The Lighthouse Lab in Alderley Park and Alex Alderton, Roberto Amato, Sonia Goncalves, Ewan Harrison, David K. Jackson, Ian Johnston, Dominic Kwiatkowski, Cordelia Langford, John Sillitoe on behalf of the Wellcome Sanger Institute COVID-19 Surveillance Team
EPI_ISL_777594, EPI_ISL_777596	Lighthouse Lab in Cambridge	Wellcome Sanger Institute for the COVID-19 Genomics UK (COG-UK) Consortium	Rob Howes, The Lighthouse Lab in Cambridge and Alex Alderton, Roberto Amato, Sonia Goncalves, Ewan Harrison, David K. Jackson, Ian Johnston, Dominic Kwiatkowski, Cordelia Langford, John Sillitoe on behalf of the Wellcome Sanger Institute COVID-19 Surveillance Team
EPI_ISL_777597	Lighthouse Lab in Alderley Park	Wellcome Sanger Institute for the COVID-19 Genomics UK (COG-UK) Consortium	Jacquelyn Wynn, Mairead Hyland, The Lighthouse Lab in Alderley Park and Alex Alderton, Roberto Amato, Sonia Goncalves, Ewan Harrison, David K. Jackson, Ian Johnston, Dominic Kwiatkowski, Cordelia Langford, John Sillitoe on behalf of the Wellcome Sanger Institute COVID-19 Surveillance Team
EPI_ISL_777598	Lighthouse Lab in Cambridge	Wellcome Sanger Institute for the COVID-19 Genomics UK (COG-UK) Consortium	Rob Howes, The Lighthouse Lab in Cambridge and Alex Alderton, Roberto Amato, Sonia Goncalves, Ewan Harrison, David K. Jackson, Ian Johnston, Dominic Kwiatkowski, Cordelia Langford, John Sillitoe on behalf of the Wellcome Sanger Institute COVID-19 Surveillance Team
EPI_ISL_777601	Lighthouse Lab in Glasgow	Wellcome Sanger Institute for the COVID-19 Genomics UK (COG-UK) Consortium	Harper VanSteenhouse, Yumi Kasai, David Gray, Carol Clugston, Anna Dominiczak and Alex Alderton, Roberto Amato, Sonia Goncalves, Ewan Harrison, David K. Jackson, Ian Johnston, Dominic Kwiatkowski, Cordelia Langford, John Sillitoe on behalf of the Wellcome Sanger Institute COVID-19 Surveillance Team
EPI_ISL_777603, EPI_ISL_777604	Lighthouse Lab in Cambridge	Wellcome Sanger Institute for the COVID-19 Genomics UK (COG-UK) Consortium	Rob Howes, The Lighthouse Lab in Cambridge and Alex Alderton, Roberto Amato, Sonia Goncalves, Ewan Harrison, David K. Jackson, Ian Johnston, Dominic Kwiatkowski, Cordelia Langford, John Sillitoe on behalf of the Wellcome Sanger Institute COVID-19 Surveillance Team
EPI_ISL_777605	Lighthouse Lab in Alderley Park	Wellcome Sanger Institute for the COVID-19 Genomics UK (COG-UK) Consortium	Jacquelyn Wynn, Mairead Hyland, The Lighthouse Lab in Alderley Park and Alex Alderton, Roberto Amato, Sonia Goncalves, Ewan Harrison, David K. Jackson, Ian Johnston, Dominic Kwiatkowski, Cordelia Langford, John Sillitoe on behalf of the Wellcome Sanger Institute COVID-19 Surveillance Team
EPI_ISL_777606, EPI_ISL_777607, EPI_ISL_777608, EPI_ISL_777609	Lighthouse Lab in Cambridge	Wellcome Sanger Institute for the COVID-19 Genomics UK (COG-UK) Consortium	Rob Howes, The Lighthouse Lab in Cambridge and Alex Alderton, Roberto Amato, Sonia Goncalves, Ewan Harrison, David K. Jackson, Ian Johnston, Dominic Kwiatkowski, Cordelia Langford, John Sillitoe on behalf of the Wellcome Sanger Institute COVID-19 Surveillance Team
EPI_ISL_777610, EPI_ISL_777612	Lighthouse Lab in Glasgow	Wellcome Sanger Institute for the COVID-19 Genomics UK (COG-UK) Consortium	Harper VanSteenhouse, Yumi Kasai, David Gray, Carol Clugston, Anna Dominiczak and Alex Alderton, Roberto Amato, Sonia Goncalves, Ewan Harrison, David K. Jackson, Ian Johnston, Dominic Kwiatkowski, Cordelia Langford, John Sillitoe on behalf of the Wellcome Sanger Institute COVID-19 Surveillance Team
EPI_ISL_777615, EPI_ISL_777616, EPI_ISL_777617, EPI_ISL_777619, EPI_ISL_777620	Lighthouse Lab in Cambridge	Wellcome Sanger Institute for the COVID-19 Genomics UK (COG-UK) Consortium	Rob Howes, The Lighthouse Lab in Cambridge and Alex Alderton, Roberto Amato, Sonia Goncalves, Ewan Harrison, David K. Jackson, Ian Johnston, Dominic Kwiatkowski, Cordelia Langford, John Sillitoe on behalf of the Wellcome Sanger Institute COVID-19 Surveillance Team
EPI_ISL_777621	Lighthouse Lab in Alderley Park	Wellcome Sanger Institute for the COVID-19 Genomics UK (COG-UK) Consortium	Jacquelyn Wynn, Mairead Hyland, The Lighthouse Lab in Alderley Park and Alex Alderton, Roberto Amato, Sonia Goncalves, Ewan Harrison, David K. Jackson, Ian Johnston, Dominic Kwiatkowski, Cordelia Langford, John Sillitoe on behalf of the Wellcome Sanger Institute COVID-19 Surveillance Team
EPI_ISL_777622	Lighthouse Lab in Glasgow	Wellcome Sanger Institute for the COVID-19 Genomics UK (COG-UK) Consortium	Harper VanSteenhouse, Yumi Kasai, David Gray, Carol Clugston, Anna Dominiczak and Alex Alderton, Roberto Amato, Sonia Goncalves, Ewan Harrison, David K. Jackson, Ian Johnston, Dominic Kwiatkowski, Cordelia Langford, John Sillitoe on behalf of the Wellcome Sanger Institute COVID-19 Surveillance Team
EPI_ISL_777624	Lighthouse Lab in Cambridge	Wellcome Sanger Institute for the COVID-19 Genomics UK (COG-UK) Consortium	Rob Howes, The Lighthouse Lab in Cambridge and Alex Alderton, Roberto Amato, Sonia Goncalves, Ewan Harrison, David K. Jackson, Ian Johnston, Dominic Kwiatkowski, Cordelia Langford, John Sillitoe on behalf of the Wellcome Sanger Institute COVID-19 Surveillance Team
EPI_ISL_777627	Lighthouse Lab in Alderley Park	Wellcome Sanger Institute for the COVID-19 Genomics UK (COG-UK) Consortium	Jacquelyn Wynn, Mairead Hyland, The Lighthouse Lab in Alderley Park and Alex Alderton, Roberto Amato, Sonia Goncalves, Ewan Harrison, David K. Jackson, Ian Johnston, Dominic Kwiatkowski, Cordelia Langford, John Sillitoe on behalf of the Wellcome Sanger Institute COVID-19 Surveillance Team
EPI_ISL_777628	Lighthouse Lab in Glasgow	Wellcome Sanger Institute for the COVID-19 Genomics UK (COG-UK) Consortium	Harper VanSteenhouse, Yumi Kasai, David Gray, Carol Clugston, Anna Dominiczak and Alex Alderton, Roberto Amato, Sonia Goncalves, Ewan Harrison, David K. Jackson, Ian Johnston, Dominic Kwiatkowski, Cordelia Langford, John Sillitoe on behalf of the Wellcome Sanger Institute COVID-19 Surveillance Team
EPI_ISL_777629	Lighthouse Lab in Alderley Park	Wellcome Sanger Institute for the COVID-19 Genomics UK (COG-UK) Consortium	Jacquelyn Wynn, Mairead Hyland, The Lighthouse Lab in Alderley Park and Alex Alderton, Roberto Amato, Sonia Goncalves, Ewan Harrison, David K. Jackson, Ian Johnston, Dominic Kwiatkowski, Cordelia Langford, John Sillitoe on behalf of the Wellcome Sanger Institute COVID-19 Surveillance Team
EPI_ISL_777630	Lighthouse Lab in Glasgow	Wellcome Sanger Institute for the COVID-19 Genomics UK (COG-UK) Consortium	Harper VanSteenhouse, Yumi Kasai, David Gray, Carol Clugston, Anna Dominiczak and Alex Alderton, Roberto Amato, Sonia Goncalves, Ewan Harrison, David K. Jackson, Ian Johnston, Dominic Kwiatkowski, Cordelia Langford, John Sillitoe on behalf of the Wellcome Sanger Institute COVID-19 Surveillance Team
EPI_ISL_777632	Lighthouse Lab in Cambridge	Wellcome Sanger Institute for the COVID-19 Genomics UK	Rob Howes, The Lighthouse Lab in Cambridge and Alex Alderton, Roberto Amato, Sonia Goncalves, Ewan Harrison, David K. Jackson, Ian Johnston,

		(COG-UK) Consortium	Dominic Kwiatkowski, Cordelia Langford, John Sillitoe on behalf of the Wellcome Sanger Institute COVID-19 Surveillance Team
EPI_ISL_777633, EPI_ISL_777634	Lighthouse Lab in Alderley Park	Wellcome Sanger Institute for the COVID-19 Genomics UK (COG-UK) Consortium	Jacquelyn Wynn, Mairead Hyland, The Lighthouse Lab in Alderley Park and Alex Alderton, Roberto Amato, Sonia Goncalves, Ewan Harrison, David K. Jackson, Ian Johnston, Dominic Kwiatkowski, Cordelia Langford, John Sillitoe on behalf of the Wellcome Sanger Institute COVID-19 Surveillance Team
EPI_ISL_777636, EPI_ISL_777637	Lighthouse Lab in Cambridge	Wellcome Sanger Institute for the COVID-19 Genomics UK (COG-UK) Consortium	Rob Howes, The Lighthouse Lab in Cambridge and Alex Alderton, Roberto Amato, Sonia Goncalves, Ewan Harrison, David K. Jackson, Ian Johnston, Dominic Kwiatkowski, Cordelia Langford, John Sillitoe on behalf of the Wellcome Sanger Institute COVID-19 Surveillance Team
EPI_ISL_777638, EPI_ISL_777639	Lighthouse Lab in Alderley Park	Wellcome Sanger Institute for the COVID-19 Genomics UK (COG-UK) Consortium	Jacquelyn Wynn, Mairead Hyland, The Lighthouse Lab in Alderley Park and Alex Alderton, Roberto Amato, Sonia Goncalves, Ewan Harrison, David K. Jackson, Ian Johnston, Dominic Kwiatkowski, Cordelia Langford, John Sillitoe on behalf of the Wellcome Sanger Institute COVID-19 Surveillance Team
EPI_ISL_777641	Lighthouse Lab in Glasgow	Wellcome Sanger Institute for the COVID-19 Genomics UK (COG-UK) Consortium	Harper VanSteenhouse, Yumi Kasai, David Gray, Carol Clugston, Anna Dominiczak and Alex Alderton, Roberto Amato, Sonia Goncalves, Ewan Harrison, David K. Jackson, Ian Johnston, Dominic Kwiatkowski, Cordelia Langford, John Sillitoe on behalf of the Wellcome Sanger Institute COVID-19 Surveillance Team
EPI_ISL_777642, EPI_ISL_777643	Lighthouse Lab in Cambridge	Wellcome Sanger Institute for the COVID-19 Genomics UK (COG-UK) Consortium	Rob Howes, The Lighthouse Lab in Cambridge and Alex Alderton, Roberto Amato, Sonia Goncalves, Ewan Harrison, David K. Jackson, Ian Johnston, Dominic Kwiatkowski, Cordelia Langford, John Sillitoe on behalf of the Wellcome Sanger Institute COVID-19 Surveillance Team
EPI_ISL_777644	Lighthouse Lab in Alderley Park	Wellcome Sanger Institute for the COVID-19 Genomics UK (COG-UK) Consortium	Jacquelyn Wynn, Mairead Hyland, The Lighthouse Lab in Alderley Park and Alex Alderton, Roberto Amato, Sonia Goncalves, Ewan Harrison, David K. Jackson, Ian Johnston, Dominic Kwiatkowski, Cordelia Langford, John Sillitoe on behalf of the Wellcome Sanger Institute COVID-19 Surveillance Team
EPI_ISL_777645	Lighthouse Lab in Cambridge	Wellcome Sanger Institute for the COVID-19 Genomics UK (COG-UK) Consortium	Rob Howes, The Lighthouse Lab in Cambridge and Alex Alderton, Roberto Amato, Sonia Goncalves, Ewan Harrison, David K. Jackson, Ian Johnston, Dominic Kwiatkowski, Cordelia Langford, John Sillitoe on behalf of the Wellcome Sanger Institute COVID-19 Surveillance Team
EPI_ISL_777646	Lighthouse Lab in Glasgow	Wellcome Sanger Institute for the COVID-19 Genomics UK (COG-UK) Consortium	Harper VanSteenhouse, Yumi Kasai, David Gray, Carol Clugston, Anna Dominiczak and Alex Alderton, Roberto Amato, Sonia Goncalves, Ewan Harrison, David K. Jackson, Ian Johnston, Dominic Kwiatkowski, Cordelia Langford, John Sillitoe on behalf of the Wellcome Sanger Institute COVID-19 Surveillance Team
EPI_ISL_777647	Lighthouse Lab in Cambridge	Wellcome Sanger Institute for the COVID-19 Genomics UK (COG-UK) Consortium	Rob Howes, The Lighthouse Lab in Cambridge and Alex Alderton, Roberto Amato, Sonia Goncalves, Ewan Harrison, David K. Jackson, Ian Johnston, Dominic Kwiatkowski, Cordelia Langford, John Sillitoe on behalf of the Wellcome Sanger Institute COVID-19 Surveillance Team
EPI_ISL_777649, EPI_ISL_777650, EPI_ISL_777651	Lighthouse Lab in Alderley Park	Wellcome Sanger Institute for the COVID-19 Genomics UK (COG-UK) Consortium	Jacquelyn Wynn, Mairead Hyland, The Lighthouse Lab in Alderley Park and Alex Alderton, Roberto Amato, Sonia Goncalves, Ewan Harrison, David K. Jackson, Ian Johnston, Dominic Kwiatkowski, Cordelia Langford, John Sillitoe on behalf of the Wellcome Sanger Institute COVID-19 Surveillance Team
EPI_ISL_777652	Lighthouse Lab in Cambridge	Wellcome Sanger Institute for the COVID-19 Genomics UK (COG-UK) Consortium	Rob Howes, The Lighthouse Lab in Cambridge and Alex Alderton, Roberto Amato, Sonia Goncalves, Ewan Harrison, David K. Jackson, Ian Johnston, Dominic Kwiatkowski, Cordelia Langford, John Sillitoe on behalf of the Wellcome Sanger Institute COVID-19 Surveillance Team
EPI_ISL_777653	Lighthouse Lab in Glasgow	Wellcome Sanger Institute for the COVID-19 Genomics UK (COG-UK) Consortium	Harper VanSteenhouse, Yumi Kasai, David Gray, Carol Clugston, Anna Dominiczak and Alex Alderton, Roberto Amato, Sonia Goncalves, Ewan Harrison, David K. Jackson, Ian Johnston, Dominic Kwiatkowski, Cordelia Langford, John Sillitoe on behalf of the Wellcome Sanger Institute COVID-19 Surveillance Team
EPI_ISL_777655	Lighthouse Lab in Alderley Park	Wellcome Sanger Institute for the COVID-19 Genomics UK (COG-UK) Consortium	Jacquelyn Wynn, Mairead Hyland, The Lighthouse Lab in Alderley Park and Alex Alderton, Roberto Amato, Sonia Goncalves, Ewan Harrison, David K. Jackson, Ian Johnston, Dominic Kwiatkowski, Cordelia Langford, John Sillitoe on behalf of the Wellcome Sanger Institute COVID-19 Surveillance Team
EPI_ISL_777656	Lighthouse Lab in Cambridge	Wellcome Sanger Institute for the COVID-19 Genomics UK (COG-UK) Consortium	Rob Howes, The Lighthouse Lab in Cambridge and Alex Alderton, Roberto Amato, Sonia Goncalves, Ewan Harrison, David K. Jackson, Ian Johnston, Dominic Kwiatkowski, Cordelia Langford, John Sillitoe on behalf of the Wellcome Sanger Institute COVID-19 Surveillance Team
EPI_ISL_777658	Lighthouse Lab in Glasgow	Wellcome Sanger Institute for the COVID-19 Genomics UK (COG-UK) Consortium	Harper VanSteenhouse, Yumi Kasai, David Gray, Carol Clugston, Anna Dominiczak and Alex Alderton, Roberto Amato, Sonia Goncalves, Ewan Harrison, David K. Jackson, Ian Johnston, Dominic Kwiatkowski, Cordelia Langford, John Sillitoe on behalf of the Wellcome Sanger Institute COVID-19 Surveillance Team
EPI_ISL_777659	Lighthouse Lab in Alderley Park	Wellcome Sanger Institute for the COVID-19 Genomics UK (COG-UK) Consortium	Jacquelyn Wynn, Mairead Hyland, The Lighthouse Lab in Alderley Park and Alex Alderton, Roberto Amato, Sonia Goncalves, Ewan Harrison, David K. Jackson, Ian Johnston, Dominic Kwiatkowski, Cordelia Langford, John Sillitoe on behalf of the Wellcome Sanger Institute COVID-19 Surveillance Team
EPI_ISL_777660	Lighthouse Lab in Glasgow	Wellcome Sanger Institute for the COVID-19 Genomics UK (COG-UK) Consortium	Harper VanSteenhouse, Yumi Kasai, David Gray, Carol Clugston, Anna Dominiczak and Alex Alderton, Roberto Amato, Sonia Goncalves, Ewan Harrison, David K. Jackson, Ian Johnston, Dominic Kwiatkowski, Cordelia Langford, John Sillitoe on behalf of the Wellcome Sanger Institute COVID-19 Surveillance Team
EPI_ISL_777661	Lighthouse Lab in Cambridge	Wellcome Sanger Institute for the COVID-19 Genomics UK (COG-UK) Consortium	Rob Howes, The Lighthouse Lab in Cambridge and Alex Alderton, Roberto Amato, Sonia Goncalves, Ewan Harrison, David K. Jackson, Ian Johnston, Dominic Kwiatkowski, Cordelia Langford, John Sillitoe on behalf of the Wellcome Sanger Institute COVID-19 Surveillance Team
EPI_ISL_777662, EPI_ISL_777663	Lighthouse Lab in Glasgow	Wellcome Sanger Institute for the COVID-19 Genomics UK (COG-UK) Consortium	Harper VanSteenhouse, Yumi Kasai, David Gray, Carol Clugston, Anna Dominiczak and Alex Alderton, Roberto Amato, Sonia Goncalves, Ewan Harrison, David K. Jackson, Ian Johnston, Dominic Kwiatkowski, Cordelia Langford, John Sillitoe on behalf of the Wellcome Sanger Institute COVID-19 Surveillance Team
EPI_ISL_777664, EPI_ISL_777665, EPI_ISL_777666	Lighthouse Lab in Cambridge	Wellcome Sanger Institute for the COVID-19 Genomics UK (COG-UK) Consortium	Rob Howes, The Lighthouse Lab in Cambridge and Alex Alderton, Roberto Amato, Sonia Goncalves, Ewan Harrison, David K. Jackson, Ian Johnston, Dominic Kwiatkowski, Cordelia Langford, John Sillitoe on behalf of the Wellcome Sanger Institute COVID-19 Surveillance Team
EPI_ISL_777667	Lighthouse Lab in Glasgow	Wellcome Sanger Institute for the COVID-19 Genomics UK (COG-UK) Consortium	Harper VanSteenhouse, Yumi Kasai, David Gray, Carol Clugston, Anna Dominiczak and Alex Alderton, Roberto Amato, Sonia Goncalves, Ewan Harrison, David K. Jackson, Ian Johnston, Dominic Kwiatkowski, Cordelia Langford, John Sillitoe on behalf of the Wellcome Sanger Institute COVID-19 Surveillance Team
EPI_ISL_777668	Lighthouse Lab in Cambridge	Wellcome Sanger Institute for the COVID-19 Genomics UK (COG-UK) Consortium	Rob Howes, The Lighthouse Lab in Cambridge and Alex Alderton, Roberto Amato, Sonia Goncalves, Ewan Harrison, David K. Jackson, Ian Johnston, Dominic Kwiatkowski, Cordelia Langford, John Sillitoe on behalf of the Wellcome Sanger Institute COVID-19 Surveillance Team
EPI_ISL_777669, EPI_ISL_777672	Lighthouse Lab in Alderley Park	Wellcome Sanger Institute for the COVID-19 Genomics UK (COG-UK) Consortium	Jacquelyn Wynn, Mairead Hyland, The Lighthouse Lab in Alderley Park and Alex Alderton, Roberto Amato, Sonia Goncalves, Ewan Harrison, David K. Jackson, Ian Johnston, Dominic Kwiatkowski, Cordelia Langford, John Sillitoe on behalf of the Wellcome Sanger Institute COVID-19 Surveillance Team
EPI_ISL_777673, EPI_ISL_777674, EPI_ISL_777675, EPI_ISL_777677, EPI_ISL_777678, EPI_ISL_777679, EPI_ISL_777680, EPI_ISL_777682, EPI_ISL_777683, EPI_ISL_777684, EPI_ISL_777686, EPI_ISL_777687, EPI_ISL_777688, EPI_ISL_777691, EPI_ISL_777692, EPI_ISL_777693, EPI_ISL_777694, EPI_ISL_777696, EPI_ISL_777697, EPI_ISL_777698, EPI_ISL_777699, EPI_ISL_777700, EPI_ISL_777702, EPI_ISL_777703, EPI_ISL_777704, EPI_ISL_777707, EPI_ISL_777708, EPI_ISL_777709, EPI_ISL_777710, EPI_ISL_777712, EPI_ISL_777713, EPI_ISL_777714, EPI_ISL_777716, EPI_ISL_777717, EPI_ISL_777718, EPI_ISL_777720, EPI_ISL_777722, EPI_ISL_777725, EPI_ISL_777726, EPI_ISL_777727, EPI_ISL_777728, EPI_ISL_777729, EPI_ISL_777730, EPI_ISL_777731, EPI_ISL_777732, EPI_ISL_777733, EPI_ISL_777734, EPI_ISL_777735, EPI_ISL_777736, EPI_ISL_777738, EPI_ISL_777740, EPI_ISL_777743, EPI_ISL_777744, EPI_ISL_777745, EPI_ISL_777748, EPI_ISL_777749, EPI_ISL_777751, EPI_ISL_777752, EPI_ISL_777753, EPI_ISL_777754, EPI_ISL_777755, EPI_ISL_777757, EPI_ISL_777758, EPI_ISL_777759, EPI_ISL_777760, EPI_ISL_777761, EPI_ISL_777762, EPI_ISL_777763, EPI_ISL_777765, EPI_ISL_777767, EPI_ISL_777769, EPI_ISL_777770, EPI_ISL_777775, EPI_ISL_777777, EPI_ISL_777781, EPI_ISL_777782, EPI_ISL_777783, EPI_ISL_777784, EPI_ISL_777785, EPI_ISL_777786, EPI_ISL_777787, EPI_ISL_777789, EPI_ISL_777792, EPI_ISL_777795, EPI_ISL_777796, EPI_ISL_777797, EPI_ISL_777798, EPI_ISL_777799, EPI_ISL_777800, EPI_ISL_777801, EPI_ISL_777803, EPI_ISL_777805, EPI_ISL_777807, EPI_ISL_777810, EPI_ISL_777811, EPI_ISL_777813, EPI_ISL_777814, EPI_ISL_777815, EPI_ISL_777817, EPI_ISL_777821, EPI_ISL_777822, EPI_ISL_777823, EPI_ISL_777824, EPI_ISL_777825, EPI_ISL_777826, EPI_ISL_777828, EPI_ISL_777829, EPI_ISL_777830, EPI_ISL_777831, EPI_ISL_777832, EPI_ISL_777834, EPI_ISL_777835, EPI_ISL_777836, EPI_ISL_777837, EPI_ISL_777838, EPI_ISL_777840, EPI_ISL_777841, EPI_ISL_777842, EPI_ISL_777845, EPI_ISL_777846, EPI_ISL_777847, EPI_ISL_777849, EPI_ISL_777851, EPI_ISL_777852, EPI_ISL_777853, EPI_ISL_777854, EPI_ISL_777857, EPI_ISL_777858, EPI_ISL_777859, EPI_ISL_777860, EPI_ISL_777861, EPI_ISL_777863, EPI_ISL_777866, EPI_ISL_777867, EPI_ISL_777868, EPI_ISL_777869, EPI_ISL_777870, EPI_ISL_777872, EPI_ISL_777874, EPI_ISL_777876, EPI_ISL_777877, EPI_ISL_777878, EPI_ISL_777879, EPI_ISL_777881, EPI_ISL_777882, EPI_ISL_777883, EPI_ISL_777885, EPI_ISL_777886, EPI_ISL_777888, EPI_ISL_777889, EPI_ISL_777890, EPI_ISL_777891, EPI_ISL_777894, EPI_ISL_777895, EPI_ISL_777896, EPI_ISL_777897, EPI_ISL_777898, EPI_ISL_777899, EPI_ISL_777900, EPI_ISL_777901, EPI_ISL_777903, EPI_ISL_777904, EPI_ISL_777906, EPI_ISL_777907, EPI_ISL_777908, EPI_ISL_777909, EPI_ISL_777910, EPI_ISL_777911, EPI_ISL_777913, EPI_ISL_777914, EPI_ISL_777916, EPI_ISL_777919, EPI_ISL_777920, EPI_ISL_777922, EPI_ISL_777923, EPI_ISL_777927, EPI_ISL_777928, EPI_ISL_777929, EPI_ISL_777930, EPI_ISL_777931, EPI_ISL_777933, EPI_ISL_777934, EPI_ISL_777936, EPI_ISL_777937, EPI_ISL_777940, EPI_ISL_777942, EPI_ISL_777943, EPI_ISL_777944, EPI_ISL_777945, EPI_ISL_777946, EPI_ISL_777947, EPI_ISL_777949, EPI_ISL_777950, EPI_ISL_777951, EPI_ISL_777952, EPI_ISL_777953, EPI_ISL_777954, EPI_ISL_777956, EPI_ISL_777958, EPI_ISL_777961, EPI_ISL_777963, EPI_ISL_777964, EPI_ISL_777965, EPI_ISL_777966, EPI_ISL_777967, EPI_ISL_777968, EPI_ISL_777969, EPI_ISL_777971, EPI_ISL_777972, EPI_ISL_777974, EPI_ISL_777975, EPI_ISL_777976, EPI_ISL_777977, EPI_ISL_777978, EPI_ISL_777979, EPI_ISL_777981, EPI_ISL_777983, EPI_ISL_777985, EPI_ISL_777986, EPI_ISL_777987, EPI_ISL_777990, EPI_ISL_777993, EPI_ISL_777994, EPI_ISL_777995, EPI_ISL_777996, EPI_ISL_777997, EPI_ISL_777998, EPI_ISL_777999, EPI_ISL_778002			
see above	Lighthouse Lab in Cambridge	Wellcome Sanger Institute for the COVID-19 Genomics UK (COG-UK) Consortium	Rob Howes, The Lighthouse Lab in Cambridge and Alex Alderton, Roberto Amato, Sonia Goncalves, Ewan Harrison, David K. Jackson, Ian Johnston, Dominic Kwiatkowski, Cordelia Langford, John Sillitoe on behalf of the Wellcome Sanger Institute COVID-19 Surveillance Team
EPI_ISL_778003, EPI_ISL_778004	Lighthouse Lab in Glasgow	Wellcome Sanger Institute for the COVID-19 Genomics UK (COG-UK) Consortium	Harper VanSteenhouse, Yumi Kasai, David Gray, Carol Clugston, Anna Dominiczak and Alex Alderton, Roberto Amato, Sonia Goncalves, Ewan Harrison, David K. Jackson, Ian Johnston, Dominic Kwiatkowski, Cordelia Langford, John Sillitoe on behalf of the Wellcome Sanger Institute COVID-19 Surveillance Team
EPI_ISL_778005, EPI_ISL_778006	Lighthouse Lab in Cambridge	Wellcome Sanger Institute for the COVID-19 Genomics UK (COG-UK) Consortium	Rob Howes, The Lighthouse Lab in Cambridge and Alex Alderton, Roberto Amato, Sonia Goncalves, Ewan Harrison, David K. Jackson, Ian Johnston, Dominic Kwiatkowski, Cordelia Langford, John Sillitoe on behalf of the Wellcome Sanger Institute COVID-19 Surveillance Team

EPI_ISL_778007, EPI_ISL_778009	Lighthouse Lab in Glasgow	Wellcome Sanger Institute for the COVID-19 Genomics UK (COG-UK) Consortium	Harper VanSteenhouse, Yumi Kasai, David Gray, Carol Clugston, Anna Dominiczak and Alex Alderton, Roberto Amato, Sonia Goncalves, Ewan Harrison, David K. Jackson, Ian Johnston, Dominic Kwiatkowski, Cordelia Langford, John Sillitoe on behalf of the Wellcome Sanger Institute COVID-19 Surveillance Team
EPI_ISL_778011, EPI_ISL_778013	Lighthouse Lab in Cambridge	Wellcome Sanger Institute for the COVID-19 Genomics UK (COG-UK) Consortium	Rob Howes, The Lighthouse Lab in Cambridge and Alex Alderton, Roberto Amato, Sonia Goncalves, Ewan Harrison, David K. Jackson, Ian Johnston, Dominic Kwiatkowski, Cordelia Langford, John Sillitoe on behalf of the Wellcome Sanger Institute COVID-19 Surveillance Team
EPI_ISL_778014	Lighthouse Lab in Glasgow	Wellcome Sanger Institute for the COVID-19 Genomics UK (COG-UK) Consortium	Harper VanSteenhouse, Yumi Kasai, David Gray, Carol Clugston, Anna Dominiczak and Alex Alderton, Roberto Amato, Sonia Goncalves, Ewan Harrison, David K. Jackson, Ian Johnston, Dominic Kwiatkowski, Cordelia Langford, John Sillitoe on behalf of the Wellcome Sanger Institute COVID-19 Surveillance Team
EPI_ISL_778015, EPI_ISL_778017	Lighthouse Lab in Cambridge	Wellcome Sanger Institute for the COVID-19 Genomics UK (COG-UK) Consortium	Rob Howes, The Lighthouse Lab in Cambridge and Alex Alderton, Roberto Amato, Sonia Goncalves, Ewan Harrison, David K. Jackson, Ian Johnston, Dominic Kwiatkowski, Cordelia Langford, John Sillitoe on behalf of the Wellcome Sanger Institute COVID-19 Surveillance Team
EPI_ISL_778018	Lighthouse Lab in Alderley Park	Wellcome Sanger Institute for the COVID-19 Genomics UK (COG-UK) Consortium	Jacquelyn Wynn, Mairead Hyland, The Lighthouse Lab in Alderley Park and Alex Alderton, Roberto Amato, Sonia Goncalves, Ewan Harrison, David K. Jackson, Ian Johnston, Dominic Kwiatkowski, Cordelia Langford, John Sillitoe on behalf of the Wellcome Sanger Institute COVID-19 Surveillance Team
EPI_ISL_778019, EPI_ISL_778020	Lighthouse Lab in Cambridge	Wellcome Sanger Institute for the COVID-19 Genomics UK (COG-UK) Consortium	Rob Howes, The Lighthouse Lab in Cambridge and Alex Alderton, Roberto Amato, Sonia Goncalves, Ewan Harrison, David K. Jackson, Ian Johnston, Dominic Kwiatkowski, Cordelia Langford, John Sillitoe on behalf of the Wellcome Sanger Institute COVID-19 Surveillance Team
EPI_ISL_778021	Lighthouse Lab in Alderley Park	Wellcome Sanger Institute for the COVID-19 Genomics UK (COG-UK) Consortium	Jacquelyn Wynn, Mairead Hyland, The Lighthouse Lab in Alderley Park and Alex Alderton, Roberto Amato, Sonia Goncalves, Ewan Harrison, David K. Jackson, Ian Johnston, Dominic Kwiatkowski, Cordelia Langford, John Sillitoe on behalf of the Wellcome Sanger Institute COVID-19 Surveillance Team
EPI_ISL_778022, EPI_ISL_778023	Lighthouse Lab in Cambridge	Wellcome Sanger Institute for the COVID-19 Genomics UK (COG-UK) Consortium	Rob Howes, The Lighthouse Lab in Cambridge and Alex Alderton, Roberto Amato, Sonia Goncalves, Ewan Harrison, David K. Jackson, Ian Johnston, Dominic Kwiatkowski, Cordelia Langford, John Sillitoe on behalf of the Wellcome Sanger Institute COVID-19 Surveillance Team
EPI_ISL_778024	Lighthouse Lab in Glasgow	Wellcome Sanger Institute for the COVID-19 Genomics UK (COG-UK) Consortium	Harper VanSteenhouse, Yumi Kasai, David Gray, Carol Clugston, Anna Dominiczak and Alex Alderton, Roberto Amato, Sonia Goncalves, Ewan Harrison, David K. Jackson, Ian Johnston, Dominic Kwiatkowski, Cordelia Langford, John Sillitoe on behalf of the Wellcome Sanger Institute COVID-19 Surveillance Team
EPI_ISL_778025	Lighthouse Lab in Cambridge	Wellcome Sanger Institute for the COVID-19 Genomics UK (COG-UK) Consortium	Rob Howes, The Lighthouse Lab in Cambridge and Alex Alderton, Roberto Amato, Sonia Goncalves, Ewan Harrison, David K. Jackson, Ian Johnston, Dominic Kwiatkowski, Cordelia Langford, John Sillitoe on behalf of the Wellcome Sanger Institute COVID-19 Surveillance Team
EPI_ISL_778026	Lighthouse Lab in Glasgow	Wellcome Sanger Institute for the COVID-19 Genomics UK (COG-UK) Consortium	Harper VanSteenhouse, Yumi Kasai, David Gray, Carol Clugston, Anna Dominiczak and Alex Alderton, Roberto Amato, Sonia Goncalves, Ewan Harrison, David K. Jackson, Ian Johnston, Dominic Kwiatkowski, Cordelia Langford, John Sillitoe on behalf of the Wellcome Sanger Institute COVID-19 Surveillance Team
EPI_ISL_778027, EPI_ISL_778028	Lighthouse Lab in Cambridge	Wellcome Sanger Institute for the COVID-19 Genomics UK (COG-UK) Consortium	Rob Howes, The Lighthouse Lab in Cambridge and Alex Alderton, Roberto Amato, Sonia Goncalves, Ewan Harrison, David K. Jackson, Ian Johnston, Dominic Kwiatkowski, Cordelia Langford, John Sillitoe on behalf of the Wellcome Sanger Institute COVID-19 Surveillance Team
EPI_ISL_778029	Lighthouse Lab in Alderley Park	Wellcome Sanger Institute for the COVID-19 Genomics UK (COG-UK) Consortium	Jacquelyn Wynn, Mairead Hyland, The Lighthouse Lab in Alderley Park and Alex Alderton, Roberto Amato, Sonia Goncalves, Ewan Harrison, David K. Jackson, Ian Johnston, Dominic Kwiatkowski, Cordelia Langford, John Sillitoe on behalf of the Wellcome Sanger Institute COVID-19 Surveillance Team
EPI_ISL_778030, EPI_ISL_778032	Lighthouse Lab in Cambridge	Wellcome Sanger Institute for the COVID-19 Genomics UK (COG-UK) Consortium	Rob Howes, The Lighthouse Lab in Cambridge and Alex Alderton, Roberto Amato, Sonia Goncalves, Ewan Harrison, David K. Jackson, Ian Johnston, Dominic Kwiatkowski, Cordelia Langford, John Sillitoe on behalf of the Wellcome Sanger Institute COVID-19 Surveillance Team
EPI_ISL_778033	Lighthouse Lab in Alderley Park	Wellcome Sanger Institute for the COVID-19 Genomics UK (COG-UK) Consortium	Jacquelyn Wynn, Mairead Hyland, The Lighthouse Lab in Alderley Park and Alex Alderton, Roberto Amato, Sonia Goncalves, Ewan Harrison, David K. Jackson, Ian Johnston, Dominic Kwiatkowski, Cordelia Langford, John Sillitoe on behalf of the Wellcome Sanger Institute COVID-19 Surveillance Team
EPI_ISL_778034	Lighthouse Lab in Glasgow	Wellcome Sanger Institute for the COVID-19 Genomics UK (COG-UK) Consortium	Harper VanSteenhouse, Yumi Kasai, David Gray, Carol Clugston, Anna Dominiczak and Alex Alderton, Roberto Amato, Sonia Goncalves, Ewan Harrison, David K. Jackson, Ian Johnston, Dominic Kwiatkowski, Cordelia Langford, John Sillitoe on behalf of the Wellcome Sanger Institute COVID-19 Surveillance Team
EPI_ISL_778035	Lighthouse Lab in Alderley Park	Wellcome Sanger Institute for the COVID-19 Genomics UK (COG-UK) Consortium	Jacquelyn Wynn, Mairead Hyland, The Lighthouse Lab in Alderley Park and Alex Alderton, Roberto Amato, Sonia Goncalves, Ewan Harrison, David K. Jackson, Ian Johnston, Dominic Kwiatkowski, Cordelia Langford, John Sillitoe on behalf of the Wellcome Sanger Institute COVID-19 Surveillance Team
EPI_ISL_778038, EPI_ISL_778039	Lighthouse Lab in Cambridge	Wellcome Sanger Institute for the COVID-19 Genomics UK (COG-UK) Consortium	Rob Howes, The Lighthouse Lab in Cambridge and Alex Alderton, Roberto Amato, Sonia Goncalves, Ewan Harrison, David K. Jackson, Ian Johnston, Dominic Kwiatkowski, Cordelia Langford, John Sillitoe on behalf of the Wellcome Sanger Institute COVID-19 Surveillance Team
EPI_ISL_778040	Lighthouse Lab in Alderley Park	Wellcome Sanger Institute for the COVID-19 Genomics UK (COG-UK) Consortium	Jacquelyn Wynn, Mairead Hyland, The Lighthouse Lab in Alderley Park and Alex Alderton, Roberto Amato, Sonia Goncalves, Ewan Harrison, David K. Jackson, Ian Johnston, Dominic Kwiatkowski, Cordelia Langford, John Sillitoe on behalf of the Wellcome Sanger Institute COVID-19 Surveillance Team
EPI_ISL_778041	Lighthouse Lab in Cambridge	Wellcome Sanger Institute for the COVID-19 Genomics UK (COG-UK) Consortium	Rob Howes, The Lighthouse Lab in Cambridge and Alex Alderton, Roberto Amato, Sonia Goncalves, Ewan Harrison, David K. Jackson, Ian Johnston, Dominic Kwiatkowski, Cordelia Langford, John Sillitoe on behalf of the Wellcome Sanger Institute COVID-19 Surveillance Team
EPI_ISL_778044, EPI_ISL_778050, EPI_ISL_778051	Lighthouse Lab in Alderley Park	Wellcome Sanger Institute for the COVID-19 Genomics UK (COG-UK) Consortium	Jacquelyn Wynn, Mairead Hyland, The Lighthouse Lab in Alderley Park and Alex Alderton, Roberto Amato, Sonia Goncalves, Ewan Harrison, David K. Jackson, Ian Johnston, Dominic Kwiatkowski, Cordelia Langford, John Sillitoe on behalf of the Wellcome Sanger Institute COVID-19 Surveillance Team
EPI_ISL_778053	Lighthouse Lab in Glasgow	Wellcome Sanger Institute for the COVID-19 Genomics UK (COG-UK) Consortium	Harper VanSteenhouse, Yumi Kasai, David Gray, Carol Clugston, Anna Dominiczak and Alex Alderton, Roberto Amato, Sonia Goncalves, Ewan Harrison, David K. Jackson, Ian Johnston, Dominic Kwiatkowski, Cordelia Langford, John Sillitoe on behalf of the Wellcome Sanger Institute COVID-19 Surveillance Team
EPI_ISL_778055	Lighthouse Lab in Alderley Park	Wellcome Sanger Institute for the COVID-19 Genomics UK (COG-UK) Consortium	Jacquelyn Wynn, Mairead Hyland, The Lighthouse Lab in Alderley Park and Alex Alderton, Roberto Amato, Sonia Goncalves, Ewan Harrison, David K. Jackson, Ian Johnston, Dominic Kwiatkowski, Cordelia Langford, John Sillitoe on behalf of the Wellcome Sanger Institute COVID-19 Surveillance Team
EPI_ISL_778056	Lighthouse Lab in Cambridge	Wellcome Sanger Institute for the COVID-19 Genomics UK (COG-UK) Consortium	Rob Howes, The Lighthouse Lab in Cambridge and Alex Alderton, Roberto Amato, Sonia Goncalves, Ewan Harrison, David K. Jackson, Ian Johnston, Dominic Kwiatkowski, Cordelia Langford, John Sillitoe on behalf of the Wellcome Sanger Institute COVID-19 Surveillance Team
EPI_ISL_778058	Lighthouse Lab in Glasgow	Wellcome Sanger Institute for the COVID-19 Genomics UK (COG-UK) Consortium	Harper VanSteenhouse, Yumi Kasai, David Gray, Carol Clugston, Anna Dominiczak and Alex Alderton, Roberto Amato, Sonia Goncalves, Ewan Harrison, David K. Jackson, Ian Johnston, Dominic Kwiatkowski, Cordelia Langford, John Sillitoe on behalf of the Wellcome Sanger Institute COVID-19 Surveillance Team
EPI_ISL_778059	Lighthouse Lab in Alderley Park	Wellcome Sanger Institute for the COVID-19 Genomics UK (COG-UK) Consortium	Jacquelyn Wynn, Mairead Hyland, The Lighthouse Lab in Alderley Park and Alex Alderton, Roberto Amato, Sonia Goncalves, Ewan Harrison, David K. Jackson, Ian Johnston, Dominic Kwiatkowski, Cordelia Langford, John Sillitoe on behalf of the Wellcome Sanger Institute COVID-19 Surveillance Team
EPI_ISL_778060	Lighthouse Lab in Glasgow	Wellcome Sanger Institute for the COVID-19 Genomics UK (COG-UK) Consortium	Harper VanSteenhouse, Yumi Kasai, David Gray, Carol Clugston, Anna Dominiczak and Alex Alderton, Roberto Amato, Sonia Goncalves, Ewan Harrison, David K. Jackson, Ian Johnston, Dominic Kwiatkowski, Cordelia Langford, John Sillitoe on behalf of the Wellcome Sanger Institute COVID-19 Surveillance Team
EPI_ISL_778064, EPI_ISL_778065	Lighthouse Lab in Alderley Park	Wellcome Sanger Institute for the COVID-19 Genomics UK (COG-UK) Consortium	Jacquelyn Wynn, Mairead Hyland, The Lighthouse Lab in Alderley Park and Alex Alderton, Roberto Amato, Sonia Goncalves, Ewan Harrison, David K. Jackson, Ian Johnston, Dominic Kwiatkowski, Cordelia Langford, John Sillitoe on behalf of the Wellcome Sanger Institute COVID-19 Surveillance Team
EPI_ISL_778066, EPI_ISL_778067	Lighthouse Lab in Cambridge	Wellcome Sanger Institute for the COVID-19 Genomics UK (COG-UK) Consortium	Rob Howes, The Lighthouse Lab in Cambridge and Alex Alderton, Roberto Amato, Sonia Goncalves, Ewan Harrison, David K. Jackson, Ian Johnston, Dominic Kwiatkowski, Cordelia Langford, John Sillitoe on behalf of the Wellcome Sanger Institute COVID-19 Surveillance Team
EPI_ISL_778068	Lighthouse Lab in Glasgow	Wellcome Sanger Institute for the COVID-19 Genomics UK (COG-UK) Consortium	Harper VanSteenhouse, Yumi Kasai, David Gray, Carol Clugston, Anna Dominiczak and Alex Alderton, Roberto Amato, Sonia Goncalves, Ewan Harrison, David K. Jackson, Ian Johnston, Dominic Kwiatkowski, Cordelia Langford, John Sillitoe on behalf of the Wellcome Sanger Institute COVID-19 Surveillance Team
EPI_ISL_778069, EPI_ISL_778070	Lighthouse Lab in Alderley Park	Wellcome Sanger Institute for the COVID-19 Genomics UK (COG-UK) Consortium	Jacquelyn Wynn, Mairead Hyland, The Lighthouse Lab in Alderley Park and Alex Alderton, Roberto Amato, Sonia Goncalves, Ewan Harrison, David K. Jackson, Ian Johnston, Dominic Kwiatkowski, Cordelia Langford, John Sillitoe on behalf of the Wellcome Sanger Institute COVID-19 Surveillance Team
EPI_ISL_778072	Lighthouse Lab in Cambridge	Wellcome Sanger Institute for the COVID-19 Genomics UK	Rob Howes, The Lighthouse Lab in Cambridge and Alex Alderton, Roberto Amato, Sonia Goncalves, Ewan Harrison, David K. Jackson, Ian Johnston,

		(COG-UK) Consortium	Dominic Kwiatkowski, Cordelia Langford, John Sillitoe on behalf of the Wellcome Sanger Institute COVID-19 Surveillance Team
EPI_ISL_778073	Lighthouse Lab in Glasgow	Wellcome Sanger Institute for the COVID-19 Genomics UK (COG-UK) Consortium	Harper VanSteenhouse, Yumi Kasai, David Gray, Carol Clugston, Anna Dominiczak and Alex Alderton, Roberto Amato, Sonia Goncalves, Ewan Harrison, David K. Jackson, Ian Johnston, Dominic Kwiatkowski, Cordelia Langford, John Sillitoe on behalf of the Wellcome Sanger Institute COVID-19 Surveillance Team
EPI_ISL_778075	Lighthouse Lab in Cambridge	Wellcome Sanger Institute for the COVID-19 Genomics UK (COG-UK) Consortium	Rob Howes, The Lighthouse Lab in Cambridge and Alex Alderton, Roberto Amato, Sonia Goncalves, Ewan Harrison, David K. Jackson, Ian Johnston, Dominic Kwiatkowski, Cordelia Langford, John Sillitoe on behalf of the Wellcome Sanger Institute COVID-19 Surveillance Team
EPI_ISL_778076	Lighthouse Lab in Glasgow	Wellcome Sanger Institute for the COVID-19 Genomics UK (COG-UK) Consortium	Harper VanSteenhouse, Yumi Kasai, David Gray, Carol Clugston, Anna Dominiczak and Alex Alderton, Roberto Amato, Sonia Goncalves, Ewan Harrison, David K. Jackson, Ian Johnston, Dominic Kwiatkowski, Cordelia Langford, John Sillitoe on behalf of the Wellcome Sanger Institute COVID-19 Surveillance Team
EPI_ISL_778079	Lighthouse Lab in Cambridge	Wellcome Sanger Institute for the COVID-19 Genomics UK (COG-UK) Consortium	Rob Howes, The Lighthouse Lab in Cambridge and Alex Alderton, Roberto Amato, Sonia Goncalves, Ewan Harrison, David K. Jackson, Ian Johnston, Dominic Kwiatkowski, Cordelia Langford, John Sillitoe on behalf of the Wellcome Sanger Institute COVID-19 Surveillance Team
EPI_ISL_778082	Lighthouse Lab in Glasgow	Wellcome Sanger Institute for the COVID-19 Genomics UK (COG-UK) Consortium	Harper VanSteenhouse, Yumi Kasai, David Gray, Carol Clugston, Anna Dominiczak and Alex Alderton, Roberto Amato, Sonia Goncalves, Ewan Harrison, David K. Jackson, Ian Johnston, Dominic Kwiatkowski, Cordelia Langford, John Sillitoe on behalf of the Wellcome Sanger Institute COVID-19 Surveillance Team
EPI_ISL_778083	Lighthouse Lab in Alderley Park	Wellcome Sanger Institute for the COVID-19 Genomics UK (COG-UK) Consortium	Jacquelyn Wynn, Mairead Hyland, The Lighthouse Lab in Alderley Park and Alex Alderton, Roberto Amato, Sonia Goncalves, Ewan Harrison, David K. Jackson, Ian Johnston, Dominic Kwiatkowski, Cordelia Langford, John Sillitoe on behalf of the Wellcome Sanger Institute COVID-19 Surveillance Team
EPI_ISL_778084, EPI_ISL_778086, EPI_ISL_778087	Lighthouse Lab in Cambridge	Wellcome Sanger Institute for the COVID-19 Genomics UK (COG-UK) Consortium	Rob Howes, The Lighthouse Lab in Cambridge and Alex Alderton, Roberto Amato, Sonia Goncalves, Ewan Harrison, David K. Jackson, Ian Johnston, Dominic Kwiatkowski, Cordelia Langford, John Sillitoe on behalf of the Wellcome Sanger Institute COVID-19 Surveillance Team
EPI_ISL_778090, EPI_ISL_778093	Lighthouse Lab in Glasgow	Wellcome Sanger Institute for the COVID-19 Genomics UK (COG-UK) Consortium	Harper VanSteenhouse, Yumi Kasai, David Gray, Carol Clugston, Anna Dominiczak and Alex Alderton, Roberto Amato, Sonia Goncalves, Ewan Harrison, David K. Jackson, Ian Johnston, Dominic Kwiatkowski, Cordelia Langford, John Sillitoe on behalf of the Wellcome Sanger Institute COVID-19 Surveillance Team
EPI_ISL_778094, EPI_ISL_778095, EPI_ISL_778096	Lighthouse Lab in Cambridge	Wellcome Sanger Institute for the COVID-19 Genomics UK (COG-UK) Consortium	Rob Howes, The Lighthouse Lab in Cambridge and Alex Alderton, Roberto Amato, Sonia Goncalves, Ewan Harrison, David K. Jackson, Ian Johnston, Dominic Kwiatkowski, Cordelia Langford, John Sillitoe on behalf of the Wellcome Sanger Institute COVID-19 Surveillance Team
EPI_ISL_778098	Lighthouse Lab in Glasgow	Wellcome Sanger Institute for the COVID-19 Genomics UK (COG-UK) Consortium	Harper VanSteenhouse, Yumi Kasai, David Gray, Carol Clugston, Anna Dominiczak and Alex Alderton, Roberto Amato, Sonia Goncalves, Ewan Harrison, David K. Jackson, Ian Johnston, Dominic Kwiatkowski, Cordelia Langford, John Sillitoe on behalf of the Wellcome Sanger Institute COVID-19 Surveillance Team
EPI_ISL_778099, EPI_ISL_778100	Lighthouse Lab in Cambridge	Wellcome Sanger Institute for the COVID-19 Genomics UK (COG-UK) Consortium	Rob Howes, The Lighthouse Lab in Cambridge and Alex Alderton, Roberto Amato, Sonia Goncalves, Ewan Harrison, David K. Jackson, Ian Johnston, Dominic Kwiatkowski, Cordelia Langford, John Sillitoe on behalf of the Wellcome Sanger Institute COVID-19 Surveillance Team
EPI_ISL_778101	Lighthouse Lab in Alderley Park	Wellcome Sanger Institute for the COVID-19 Genomics UK (COG-UK) Consortium	Jacquelyn Wynn, Mairead Hyland, The Lighthouse Lab in Alderley Park and Alex Alderton, Roberto Amato, Sonia Goncalves, Ewan Harrison, David K. Jackson, Ian Johnston, Dominic Kwiatkowski, Cordelia Langford, John Sillitoe on behalf of the Wellcome Sanger Institute COVID-19 Surveillance Team
EPI_ISL_778103	Lighthouse Lab in Cambridge	Wellcome Sanger Institute for the COVID-19 Genomics UK (COG-UK) Consortium	Rob Howes, The Lighthouse Lab in Cambridge and Alex Alderton, Roberto Amato, Sonia Goncalves, Ewan Harrison, David K. Jackson, Ian Johnston, Dominic Kwiatkowski, Cordelia Langford, John Sillitoe on behalf of the Wellcome Sanger Institute COVID-19 Surveillance Team
EPI_ISL_778104	Lighthouse Lab in Alderley Park	Wellcome Sanger Institute for the COVID-19 Genomics UK (COG-UK) Consortium	Jacquelyn Wynn, Mairead Hyland, The Lighthouse Lab in Alderley Park and Alex Alderton, Roberto Amato, Sonia Goncalves, Ewan Harrison, David K. Jackson, Ian Johnston, Dominic Kwiatkowski, Cordelia Langford, John Sillitoe on behalf of the Wellcome Sanger Institute COVID-19 Surveillance Team
EPI_ISL_778106, EPI_ISL_778107	Lighthouse Lab in Glasgow	Wellcome Sanger Institute for the COVID-19 Genomics UK (COG-UK) Consortium	Harper VanSteenhouse, Yumi Kasai, David Gray, Carol Clugston, Anna Dominiczak and Alex Alderton, Roberto Amato, Sonia Goncalves, Ewan Harrison, David K. Jackson, Ian Johnston, Dominic Kwiatkowski, Cordelia Langford, John Sillitoe on behalf of the Wellcome Sanger Institute COVID-19 Surveillance Team
EPI_ISL_778108	Lighthouse Lab in Alderley Park	Wellcome Sanger Institute for the COVID-19 Genomics UK (COG-UK) Consortium	Jacquelyn Wynn, Mairead Hyland, The Lighthouse Lab in Alderley Park and Alex Alderton, Roberto Amato, Sonia Goncalves, Ewan Harrison, David K. Jackson, Ian Johnston, Dominic Kwiatkowski, Cordelia Langford, John Sillitoe on behalf of the Wellcome Sanger Institute COVID-19 Surveillance Team
EPI_ISL_778110	Lighthouse Lab in Cambridge	Wellcome Sanger Institute for the COVID-19 Genomics UK (COG-UK) Consortium	Rob Howes, The Lighthouse Lab in Cambridge and Alex Alderton, Roberto Amato, Sonia Goncalves, Ewan Harrison, David K. Jackson, Ian Johnston, Dominic Kwiatkowski, Cordelia Langford, John Sillitoe on behalf of the Wellcome Sanger Institute COVID-19 Surveillance Team
EPI_ISL_778111	Lighthouse Lab in Glasgow	Wellcome Sanger Institute for the COVID-19 Genomics UK (COG-UK) Consortium	Harper VanSteenhouse, Yumi Kasai, David Gray, Carol Clugston, Anna Dominiczak and Alex Alderton, Roberto Amato, Sonia Goncalves, Ewan Harrison, David K. Jackson, Ian Johnston, Dominic Kwiatkowski, Cordelia Langford, John Sillitoe on behalf of the Wellcome Sanger Institute COVID-19 Surveillance Team
EPI_ISL_778113	Lighthouse Lab in Alderley Park	Wellcome Sanger Institute for the COVID-19 Genomics UK (COG-UK) Consortium	Jacquelyn Wynn, Mairead Hyland, The Lighthouse Lab in Alderley Park and Alex Alderton, Roberto Amato, Sonia Goncalves, Ewan Harrison, David K. Jackson, Ian Johnston, Dominic Kwiatkowski, Cordelia Langford, John Sillitoe on behalf of the Wellcome Sanger Institute COVID-19 Surveillance Team
EPI_ISL_778114, EPI_ISL_778115	Lighthouse Lab in Cambridge	Wellcome Sanger Institute for the COVID-19 Genomics UK (COG-UK) Consortium	Rob Howes, The Lighthouse Lab in Cambridge and Alex Alderton, Roberto Amato, Sonia Goncalves, Ewan Harrison, David K. Jackson, Ian Johnston, Dominic Kwiatkowski, Cordelia Langford, John Sillitoe on behalf of the Wellcome Sanger Institute COVID-19 Surveillance Team
EPI_ISL_778116	Lighthouse Lab in Alderley Park	Wellcome Sanger Institute for the COVID-19 Genomics UK (COG-UK) Consortium	Jacquelyn Wynn, Mairead Hyland, The Lighthouse Lab in Alderley Park and Alex Alderton, Roberto Amato, Sonia Goncalves, Ewan Harrison, David K. Jackson, Ian Johnston, Dominic Kwiatkowski, Cordelia Langford, John Sillitoe on behalf of the Wellcome Sanger Institute COVID-19 Surveillance Team
EPI_ISL_778117	Lighthouse Lab in Glasgow	Wellcome Sanger Institute for the COVID-19 Genomics UK (COG-UK) Consortium	Harper VanSteenhouse, Yumi Kasai, David Gray, Carol Clugston, Anna Dominiczak and Alex Alderton, Roberto Amato, Sonia Goncalves, Ewan Harrison, David K. Jackson, Ian Johnston, Dominic Kwiatkowski, Cordelia Langford, John Sillitoe on behalf of the Wellcome Sanger Institute COVID-19 Surveillance Team
EPI_ISL_778120	Lighthouse Lab in Cambridge	Wellcome Sanger Institute for the COVID-19 Genomics UK (COG-UK) Consortium	Rob Howes, The Lighthouse Lab in Cambridge and Alex Alderton, Roberto Amato, Sonia Goncalves, Ewan Harrison, David K. Jackson, Ian Johnston, Dominic Kwiatkowski, Cordelia Langford, John Sillitoe on behalf of the Wellcome Sanger Institute COVID-19 Surveillance Team
EPI_ISL_778121	Lighthouse Lab in Alderley Park	Wellcome Sanger Institute for the COVID-19 Genomics UK (COG-UK) Consortium	Jacquelyn Wynn, Mairead Hyland, The Lighthouse Lab in Alderley Park and Alex Alderton, Roberto Amato, Sonia Goncalves, Ewan Harrison, David K. Jackson, Ian Johnston, Dominic Kwiatkowski, Cordelia Langford, John Sillitoe on behalf of the Wellcome Sanger Institute COVID-19 Surveillance Team
EPI_ISL_778122	Lighthouse Lab in Glasgow	Wellcome Sanger Institute for the COVID-19 Genomics UK (COG-UK) Consortium	Harper VanSteenhouse, Yumi Kasai, David Gray, Carol Clugston, Anna Dominiczak and Alex Alderton, Roberto Amato, Sonia Goncalves, Ewan Harrison, David K. Jackson, Ian Johnston, Dominic Kwiatkowski, Cordelia Langford, John Sillitoe on behalf of the Wellcome Sanger Institute COVID-19 Surveillance Team
EPI_ISL_778123, EPI_ISL_778126	Lighthouse Lab in Cambridge	Wellcome Sanger Institute for the COVID-19 Genomics UK (COG-UK) Consortium	Rob Howes, The Lighthouse Lab in Cambridge and Alex Alderton, Roberto Amato, Sonia Goncalves, Ewan Harrison, David K. Jackson, Ian Johnston, Dominic Kwiatkowski, Cordelia Langford, John Sillitoe on behalf of the Wellcome Sanger Institute COVID-19 Surveillance Team
EPI_ISL_778128	Lighthouse Lab in Alderley Park	Wellcome Sanger Institute for the COVID-19 Genomics UK (COG-UK) Consortium	Jacquelyn Wynn, Mairead Hyland, The Lighthouse Lab in Alderley Park and Alex Alderton, Roberto Amato, Sonia Goncalves, Ewan Harrison, David K. Jackson, Ian Johnston, Dominic Kwiatkowski, Cordelia Langford, John Sillitoe on behalf of the Wellcome Sanger Institute COVID-19 Surveillance Team
EPI_ISL_778130	Lighthouse Lab in Cambridge	Wellcome Sanger Institute for the COVID-19 Genomics UK (COG-UK) Consortium	Rob Howes, The Lighthouse Lab in Cambridge and Alex Alderton, Roberto Amato, Sonia Goncalves, Ewan Harrison, David K. Jackson, Ian Johnston, Dominic Kwiatkowski, Cordelia Langford, John Sillitoe on behalf of the Wellcome Sanger Institute COVID-19 Surveillance Team
EPI_ISL_778131	Lighthouse Lab in Glasgow	Wellcome Sanger Institute for the COVID-19 Genomics UK (COG-UK) Consortium	Harper VanSteenhouse, Yumi Kasai, David Gray, Carol Clugston, Anna Dominiczak and Alex Alderton, Roberto Amato, Sonia Goncalves, Ewan Harrison, David K. Jackson, Ian Johnston, Dominic Kwiatkowski, Cordelia Langford, John Sillitoe on behalf of the Wellcome Sanger Institute COVID-19 Surveillance Team
EPI_ISL_778132	Lighthouse Lab in Alderley Park	Wellcome Sanger Institute for the COVID-19 Genomics UK (COG-UK) Consortium	Jacquelyn Wynn, Mairead Hyland, The Lighthouse Lab in Alderley Park and Alex Alderton, Roberto Amato, Sonia Goncalves, Ewan Harrison, David K. Jackson, Ian Johnston, Dominic Kwiatkowski, Cordelia Langford, John Sillitoe on behalf of the Wellcome Sanger Institute COVID-19 Surveillance Team
EPI_ISL_778133	Lighthouse Lab in Glasgow	Wellcome Sanger Institute for the COVID-19 Genomics UK	Harper VanSteenhouse, Yumi Kasai, David Gray, Carol Clugston, Anna Dominiczak and Alex Alderton, Roberto Amato, Sonia Goncalves, Ewan Harrison,

		(COG-UK) Consortium	David K. Jackson, Ian Johnston, Dominic Kwiatkowski, Cordelia Langford, John Sillitoe on behalf of the Wellcome Sanger Institute COVID-19 Surveillance Team
EPI_ISL_778135, EPI_ISL_778137, EPI_ISL_778138, EPI_ISL_778139, EPI_ISL_778141, EPI_ISL_778142, EPI_ISL_778143	Lighthouse Lab in Cambridge	Wellcome Sanger Institute for the COVID-19 Genomics UK (COG-UK) Consortium	Rob Howes, The Lighthouse Lab in Cambridge and Alex Alderton, Roberto Amato, Sonia Goncalves, Ewan Harrison, David K. Jackson, Ian Johnston, Dominic Kwiatkowski, Cordelia Langford, John Sillitoe on behalf of the Wellcome Sanger Institute COVID-19 Surveillance Team
EPI_ISL_778149, EPI_ISL_778153, EPI_ISL_778156	Lighthouse Lab in Glasgow	Wellcome Sanger Institute for the COVID-19 Genomics UK (COG-UK) Consortium	Harper VanSteenhouse, Yumi Kasai, David Gray, Carol Clugston, Anna Dominiczak and Alex Alderton, Roberto Amato, Sonia Goncalves, Ewan Harrison, David K. Jackson, Ian Johnston, Dominic Kwiatkowski, Cordelia Langford, John Sillitoe on behalf of the Wellcome Sanger Institute COVID-19 Surveillance Team
EPI_ISL_778157, EPI_ISL_778160, EPI_ISL_778161, EPI_ISL_778162	Lighthouse Lab in Cambridge	Wellcome Sanger Institute for the COVID-19 Genomics UK (COG-UK) Consortium	Rob Howes, The Lighthouse Lab in Cambridge and Alex Alderton, Roberto Amato, Sonia Goncalves, Ewan Harrison, David K. Jackson, Ian Johnston, Dominic Kwiatkowski, Cordelia Langford, John Sillitoe on behalf of the Wellcome Sanger Institute COVID-19 Surveillance Team
EPI_ISL_778163, EPI_ISL_778164, EPI_ISL_778165, EPI_ISL_778166	Lighthouse Lab in Alderley Park	Wellcome Sanger Institute for the COVID-19 Genomics UK (COG-UK) Consortium	Jacquelyn Wynn, Mairead Hyland, The Lighthouse Lab in Alderley Park and Alex Alderton, Roberto Amato, Sonia Goncalves, Ewan Harrison, David K. Jackson, Ian Johnston, Dominic Kwiatkowski, Cordelia Langford, John Sillitoe on behalf of the Wellcome Sanger Institute COVID-19 Surveillance Team
EPI_ISL_778167	Lighthouse Lab in Glasgow	Wellcome Sanger Institute for the COVID-19 Genomics UK (COG-UK) Consortium	Harper VanSteenhouse, Yumi Kasai, David Gray, Carol Clugston, Anna Dominiczak and Alex Alderton, Roberto Amato, Sonia Goncalves, Ewan Harrison, David K. Jackson, Ian Johnston, Dominic Kwiatkowski, Cordelia Langford, John Sillitoe on behalf of the Wellcome Sanger Institute COVID-19 Surveillance Team
EPI_ISL_778169, EPI_ISL_778170, EPI_ISL_778172, EPI_ISL_778173, EPI_ISL_778174, EPI_ISL_778175	Lighthouse Lab in Cambridge	Wellcome Sanger Institute for the COVID-19 Genomics UK (COG-UK) Consortium	Rob Howes, The Lighthouse Lab in Cambridge and Alex Alderton, Roberto Amato, Sonia Goncalves, Ewan Harrison, David K. Jackson, Ian Johnston, Dominic Kwiatkowski, Cordelia Langford, John Sillitoe on behalf of the Wellcome Sanger Institute COVID-19 Surveillance Team
EPI_ISL_778176	Lighthouse Lab in Glasgow	Wellcome Sanger Institute for the COVID-19 Genomics UK (COG-UK) Consortium	Harper VanSteenhouse, Yumi Kasai, David Gray, Carol Clugston, Anna Dominiczak and Alex Alderton, Roberto Amato, Sonia Goncalves, Ewan Harrison, David K. Jackson, Ian Johnston, Dominic Kwiatkowski, Cordelia Langford, John Sillitoe on behalf of the Wellcome Sanger Institute COVID-19 Surveillance Team
EPI_ISL_778177, EPI_ISL_778180	Lighthouse Lab in Cambridge	Wellcome Sanger Institute for the COVID-19 Genomics UK (COG-UK) Consortium	Rob Howes, The Lighthouse Lab in Cambridge and Alex Alderton, Roberto Amato, Sonia Goncalves, Ewan Harrison, David K. Jackson, Ian Johnston, Dominic Kwiatkowski, Cordelia Langford, John Sillitoe on behalf of the Wellcome Sanger Institute COVID-19 Surveillance Team
EPI_ISL_778187	Lighthouse Lab in Alderley Park	Wellcome Sanger Institute for the COVID-19 Genomics UK (COG-UK) Consortium	Jacquelyn Wynn, Mairead Hyland, The Lighthouse Lab in Alderley Park and Alex Alderton, Roberto Amato, Sonia Goncalves, Ewan Harrison, David K. Jackson, Ian Johnston, Dominic Kwiatkowski, Cordelia Langford, John Sillitoe on behalf of the Wellcome Sanger Institute COVID-19 Surveillance Team
EPI_ISL_778188, EPI_ISL_778189	Lighthouse Lab in Cambridge	Wellcome Sanger Institute for the COVID-19 Genomics UK (COG-UK) Consortium	Rob Howes, The Lighthouse Lab in Cambridge and Alex Alderton, Roberto Amato, Sonia Goncalves, Ewan Harrison, David K. Jackson, Ian Johnston, Dominic Kwiatkowski, Cordelia Langford, John Sillitoe on behalf of the Wellcome Sanger Institute COVID-19 Surveillance Team
EPI_ISL_778191	Lighthouse Lab in Alderley Park	Wellcome Sanger Institute for the COVID-19 Genomics UK (COG-UK) Consortium	Jacquelyn Wynn, Mairead Hyland, The Lighthouse Lab in Alderley Park and Alex Alderton, Roberto Amato, Sonia Goncalves, Ewan Harrison, David K. Jackson, Ian Johnston, Dominic Kwiatkowski, Cordelia Langford, John Sillitoe on behalf of the Wellcome Sanger Institute COVID-19 Surveillance Team
EPI_ISL_778192	Lighthouse Lab in Glasgow	Wellcome Sanger Institute for the COVID-19 Genomics UK (COG-UK) Consortium	Harper VanSteenhouse, Yumi Kasai, David Gray, Carol Clugston, Anna Dominiczak and Alex Alderton, Roberto Amato, Sonia Goncalves, Ewan Harrison, David K. Jackson, Ian Johnston, Dominic Kwiatkowski, Cordelia Langford, John Sillitoe on behalf of the Wellcome Sanger Institute COVID-19 Surveillance Team
EPI_ISL_778194	Lighthouse Lab in Cambridge	Wellcome Sanger Institute for the COVID-19 Genomics UK (COG-UK) Consortium	Rob Howes, The Lighthouse Lab in Cambridge and Alex Alderton, Roberto Amato, Sonia Goncalves, Ewan Harrison, David K. Jackson, Ian Johnston, Dominic Kwiatkowski, Cordelia Langford, John Sillitoe on behalf of the Wellcome Sanger Institute COVID-19 Surveillance Team
EPI_ISL_778196	Lighthouse Lab in Alderley Park	Wellcome Sanger Institute for the COVID-19 Genomics UK (COG-UK) Consortium	Jacquelyn Wynn, Mairead Hyland, The Lighthouse Lab in Alderley Park and Alex Alderton, Roberto Amato, Sonia Goncalves, Ewan Harrison, David K. Jackson, Ian Johnston, Dominic Kwiatkowski, Cordelia Langford, John Sillitoe on behalf of the Wellcome Sanger Institute COVID-19 Surveillance Team
EPI_ISL_778197, EPI_ISL_778198, EPI_ISL_778199	Lighthouse Lab in Cambridge	Wellcome Sanger Institute for the COVID-19 Genomics UK (COG-UK) Consortium	Rob Howes, The Lighthouse Lab in Cambridge and Alex Alderton, Roberto Amato, Sonia Goncalves, Ewan Harrison, David K. Jackson, Ian Johnston, Dominic Kwiatkowski, Cordelia Langford, John Sillitoe on behalf of the Wellcome Sanger Institute COVID-19 Surveillance Team
EPI_ISL_778201	Lighthouse Lab in Alderley Park	Wellcome Sanger Institute for the COVID-19 Genomics UK (COG-UK) Consortium	Jacquelyn Wynn, Mairead Hyland, The Lighthouse Lab in Alderley Park and Alex Alderton, Roberto Amato, Sonia Goncalves, Ewan Harrison, David K. Jackson, Ian Johnston, Dominic Kwiatkowski, Cordelia Langford, John Sillitoe on behalf of the Wellcome Sanger Institute COVID-19 Surveillance Team
EPI_ISL_778203	Lighthouse Lab in Glasgow	Wellcome Sanger Institute for the COVID-19 Genomics UK (COG-UK) Consortium	Harper VanSteenhouse, Yumi Kasai, David Gray, Carol Clugston, Anna Dominiczak and Alex Alderton, Roberto Amato, Sonia Goncalves, Ewan Harrison, David K. Jackson, Ian Johnston, Dominic Kwiatkowski, Cordelia Langford, John Sillitoe on behalf of the Wellcome Sanger Institute COVID-19 Surveillance Team
EPI_ISL_778204, EPI_ISL_778205, EPI_ISL_778206	Lighthouse Lab in Cambridge	Wellcome Sanger Institute for the COVID-19 Genomics UK (COG-UK) Consortium	Rob Howes, The Lighthouse Lab in Cambridge and Alex Alderton, Roberto Amato, Sonia Goncalves, Ewan Harrison, David K. Jackson, Ian Johnston, Dominic Kwiatkowski, Cordelia Langford, John Sillitoe on behalf of the Wellcome Sanger Institute COVID-19 Surveillance Team
EPI_ISL_778207	Lighthouse Lab in Alderley Park	Wellcome Sanger Institute for the COVID-19 Genomics UK (COG-UK) Consortium	Jacquelyn Wynn, Mairead Hyland, The Lighthouse Lab in Alderley Park and Alex Alderton, Roberto Amato, Sonia Goncalves, Ewan Harrison, David K. Jackson, Ian Johnston, Dominic Kwiatkowski, Cordelia Langford, John Sillitoe on behalf of the Wellcome Sanger Institute COVID-19 Surveillance Team
EPI_ISL_778208, EPI_ISL_778210, EPI_ISL_778211	Lighthouse Lab in Cambridge	Wellcome Sanger Institute for the COVID-19 Genomics UK (COG-UK) Consortium	Rob Howes, The Lighthouse Lab in Cambridge and Alex Alderton, Roberto Amato, Sonia Goncalves, Ewan Harrison, David K. Jackson, Ian Johnston, Dominic Kwiatkowski, Cordelia Langford, John Sillitoe on behalf of the Wellcome Sanger Institute COVID-19 Surveillance Team
EPI_ISL_778212	Lighthouse Lab in Alderley Park	Wellcome Sanger Institute for the COVID-19 Genomics UK (COG-UK) Consortium	Jacquelyn Wynn, Mairead Hyland, The Lighthouse Lab in Alderley Park and Alex Alderton, Roberto Amato, Sonia Goncalves, Ewan Harrison, David K. Jackson, Ian Johnston, Dominic Kwiatkowski, Cordelia Langford, John Sillitoe on behalf of the Wellcome Sanger Institute COVID-19 Surveillance Team
EPI_ISL_778213, EPI_ISL_778214	Lighthouse Lab in Cambridge	Wellcome Sanger Institute for the COVID-19 Genomics UK (COG-UK) Consortium	Rob Howes, The Lighthouse Lab in Cambridge and Alex Alderton, Roberto Amato, Sonia Goncalves, Ewan Harrison, David K. Jackson, Ian Johnston, Dominic Kwiatkowski, Cordelia Langford, John Sillitoe on behalf of the Wellcome Sanger Institute COVID-19 Surveillance Team
EPI_ISL_778215, EPI_ISL_778216	Lighthouse Lab in Glasgow	Wellcome Sanger Institute for the COVID-19 Genomics UK (COG-UK) Consortium	Harper VanSteenhouse, Yumi Kasai, David Gray, Carol Clugston, Anna Dominiczak and Alex Alderton, Roberto Amato, Sonia Goncalves, Ewan Harrison, David K. Jackson, Ian Johnston, Dominic Kwiatkowski, Cordelia Langford, John Sillitoe on behalf of the Wellcome Sanger Institute COVID-19 Surveillance Team
EPI_ISL_778217, EPI_ISL_778218, EPI_ISL_778221, EPI_ISL_778223, EPI_ISL_778224	Lighthouse Lab in Cambridge	Wellcome Sanger Institute for the COVID-19 Genomics UK (COG-UK) Consortium	Rob Howes, The Lighthouse Lab in Cambridge and Alex Alderton, Roberto Amato, Sonia Goncalves, Ewan Harrison, David K. Jackson, Ian Johnston, Dominic Kwiatkowski, Cordelia Langford, John Sillitoe on behalf of the Wellcome Sanger Institute COVID-19 Surveillance Team
EPI_ISL_778225	Lighthouse Lab in Alderley Park	Wellcome Sanger Institute for the COVID-19 Genomics UK (COG-UK) Consortium	Jacquelyn Wynn, Mairead Hyland, The Lighthouse Lab in Alderley Park and Alex Alderton, Roberto Amato, Sonia Goncalves, Ewan Harrison, David K. Jackson, Ian Johnston, Dominic Kwiatkowski, Cordelia Langford, John Sillitoe on behalf of the Wellcome Sanger Institute COVID-19 Surveillance Team
EPI_ISL_778226	Lighthouse Lab in Cambridge	Wellcome Sanger Institute for the COVID-19 Genomics UK (COG-UK) Consortium	Rob Howes, The Lighthouse Lab in Cambridge and Alex Alderton, Roberto Amato, Sonia Goncalves, Ewan Harrison, David K. Jackson, Ian Johnston, Dominic Kwiatkowski, Cordelia Langford, John Sillitoe on behalf of the Wellcome Sanger Institute COVID-19 Surveillance Team
EPI_ISL_778228	Lighthouse Lab in Alderley Park	Wellcome Sanger Institute for the COVID-19 Genomics UK (COG-UK) Consortium	Jacquelyn Wynn, Mairead Hyland, The Lighthouse Lab in Alderley Park and Alex Alderton, Roberto Amato, Sonia Goncalves, Ewan Harrison, David K. Jackson, Ian Johnston, Dominic Kwiatkowski, Cordelia Langford, John Sillitoe on behalf of the Wellcome Sanger Institute COVID-19 Surveillance Team
EPI_ISL_778229	Lighthouse Lab in Glasgow	Wellcome Sanger Institute for the COVID-19 Genomics UK (COG-UK) Consortium	Harper VanSteenhouse, Yumi Kasai, David Gray, Carol Clugston, Anna Dominiczak and Alex Alderton, Roberto Amato, Sonia Goncalves, Ewan Harrison, David K. Jackson, Ian Johnston, Dominic Kwiatkowski, Cordelia Langford, John Sillitoe on behalf of the Wellcome Sanger Institute COVID-19 Surveillance Team
EPI_ISL_778230	Lighthouse Lab in Alderley Park	Wellcome Sanger Institute for the COVID-19 Genomics UK (COG-UK) Consortium	Jacquelyn Wynn, Mairead Hyland, The Lighthouse Lab in Alderley Park and Alex Alderton, Roberto Amato, Sonia Goncalves, Ewan Harrison, David K. Jackson, Ian Johnston, Dominic Kwiatkowski, Cordelia Langford, John Sillitoe on behalf of the Wellcome Sanger Institute COVID-19 Surveillance Team
EPI_ISL_778232	Lighthouse Lab in Cambridge	Wellcome Sanger Institute for the COVID-19 Genomics UK (COG-UK) Consortium	Rob Howes, The Lighthouse Lab in Cambridge and Alex Alderton, Roberto Amato, Sonia Goncalves, Ewan Harrison, David K. Jackson, Ian Johnston, Dominic Kwiatkowski, Cordelia Langford, John Sillitoe on behalf of the Wellcome Sanger Institute COVID-19 Surveillance Team

EPI_ISL_778233, EPI_ISL_778234	Lighthouse Lab in Alderley Park	Wellcome Sanger Institute for the COVID-19 Genomics UK (COG-UK) Consortium	Jacquelyn Wynn, Mairead Hyland, The Lighthouse Lab in Alderley Park and Alex Alderton, Roberto Amato, Sonia Goncalves, Ewan Harrison, David K. Jackson, Ian Johnston, Dominic Kwiatkowski, Cordelia Langford, John Sillitoe on behalf of the Wellcome Sanger Institute COVID-19 Surveillance Team
EPI_ISL_778235	Lighthouse Lab in Cambridge	Wellcome Sanger Institute for the COVID-19 Genomics UK (COG-UK) Consortium	Rob Howes, The Lighthouse Lab in Cambridge and Alex Alderton, Roberto Amato, Sonia Goncalves, Ewan Harrison, David K. Jackson, Ian Johnston, Dominic Kwiatkowski, Cordelia Langford, John Sillitoe on behalf of the Wellcome Sanger Institute COVID-19 Surveillance Team
EPI_ISL_778236	Lighthouse Lab in Glasgow	Wellcome Sanger Institute for the COVID-19 Genomics UK (COG-UK) Consortium	Harper VanSteenhouse, Yumi Kasai, David Gray, Carol Clugston, Anna Dominiczak and Alex Alderton, Roberto Amato, Sonia Goncalves, Ewan Harrison, David K. Jackson, Ian Johnston, Dominic Kwiatkowski, Cordelia Langford, John Sillitoe on behalf of the Wellcome Sanger Institute COVID-19 Surveillance Team
EPI_ISL_778237	Lighthouse Lab in Cambridge	Wellcome Sanger Institute for the COVID-19 Genomics UK (COG-UK) Consortium	Rob Howes, The Lighthouse Lab in Cambridge and Alex Alderton, Roberto Amato, Sonia Goncalves, Ewan Harrison, David K. Jackson, Ian Johnston, Dominic Kwiatkowski, Cordelia Langford, John Sillitoe on behalf of the Wellcome Sanger Institute COVID-19 Surveillance Team
EPI_ISL_778238, EPI_ISL_778239, EPI_ISL_778240	Lighthouse Lab in Alderley Park	Wellcome Sanger Institute for the COVID-19 Genomics UK (COG-UK) Consortium	Jacquelyn Wynn, Mairead Hyland, The Lighthouse Lab in Alderley Park and Alex Alderton, Roberto Amato, Sonia Goncalves, Ewan Harrison, David K. Jackson, Ian Johnston, Dominic Kwiatkowski, Cordelia Langford, John Sillitoe on behalf of the Wellcome Sanger Institute COVID-19 Surveillance Team
EPI_ISL_778241, EPI_ISL_778242, EPI_ISL_778243	Lighthouse Lab in Cambridge	Wellcome Sanger Institute for the COVID-19 Genomics UK (COG-UK) Consortium	Rob Howes, The Lighthouse Lab in Cambridge and Alex Alderton, Roberto Amato, Sonia Goncalves, Ewan Harrison, David K. Jackson, Ian Johnston, Dominic Kwiatkowski, Cordelia Langford, John Sillitoe on behalf of the Wellcome Sanger Institute COVID-19 Surveillance Team
EPI_ISL_778244	Lighthouse Lab in Glasgow	Wellcome Sanger Institute for the COVID-19 Genomics UK (COG-UK) Consortium	Harper VanSteenhouse, Yumi Kasai, David Gray, Carol Clugston, Anna Dominiczak and Alex Alderton, Roberto Amato, Sonia Goncalves, Ewan Harrison, David K. Jackson, Ian Johnston, Dominic Kwiatkowski, Cordelia Langford, John Sillitoe on behalf of the Wellcome Sanger Institute COVID-19 Surveillance Team
EPI_ISL_778246	Lighthouse Lab in Alderley Park	Wellcome Sanger Institute for the COVID-19 Genomics UK (COG-UK) Consortium	Jacquelyn Wynn, Mairead Hyland, The Lighthouse Lab in Alderley Park and Alex Alderton, Roberto Amato, Sonia Goncalves, Ewan Harrison, David K. Jackson, Ian Johnston, Dominic Kwiatkowski, Cordelia Langford, John Sillitoe on behalf of the Wellcome Sanger Institute COVID-19 Surveillance Team
EPI_ISL_778247, EPI_ISL_778248	Lighthouse Lab in Glasgow	Wellcome Sanger Institute for the COVID-19 Genomics UK (COG-UK) Consortium	Harper VanSteenhouse, Yumi Kasai, David Gray, Carol Clugston, Anna Dominiczak and Alex Alderton, Roberto Amato, Sonia Goncalves, Ewan Harrison, David K. Jackson, Ian Johnston, Dominic Kwiatkowski, Cordelia Langford, John Sillitoe on behalf of the Wellcome Sanger Institute COVID-19 Surveillance Team
EPI_ISL_778249	Lighthouse Lab in Alderley Park	Wellcome Sanger Institute for the COVID-19 Genomics UK (COG-UK) Consortium	Jacquelyn Wynn, Mairead Hyland, The Lighthouse Lab in Alderley Park and Alex Alderton, Roberto Amato, Sonia Goncalves, Ewan Harrison, David K. Jackson, Ian Johnston, Dominic Kwiatkowski, Cordelia Langford, John Sillitoe on behalf of the Wellcome Sanger Institute COVID-19 Surveillance Team
EPI_ISL_778250	Lighthouse Lab in Glasgow	Wellcome Sanger Institute for the COVID-19 Genomics UK (COG-UK) Consortium	Harper VanSteenhouse, Yumi Kasai, David Gray, Carol Clugston, Anna Dominiczak and Alex Alderton, Roberto Amato, Sonia Goncalves, Ewan Harrison, David K. Jackson, Ian Johnston, Dominic Kwiatkowski, Cordelia Langford, John Sillitoe on behalf of the Wellcome Sanger Institute COVID-19 Surveillance Team
EPI_ISL_778254, EPI_ISL_778255	Lighthouse Lab in Cambridge	Wellcome Sanger Institute for the COVID-19 Genomics UK (COG-UK) Consortium	Rob Howes, The Lighthouse Lab in Cambridge and Alex Alderton, Roberto Amato, Sonia Goncalves, Ewan Harrison, David K. Jackson, Ian Johnston, Dominic Kwiatkowski, Cordelia Langford, John Sillitoe on behalf of the Wellcome Sanger Institute COVID-19 Surveillance Team
EPI_ISL_778256	Lighthouse Lab in Alderley Park	Wellcome Sanger Institute for the COVID-19 Genomics UK (COG-UK) Consortium	Jacquelyn Wynn, Mairead Hyland, The Lighthouse Lab in Alderley Park and Alex Alderton, Roberto Amato, Sonia Goncalves, Ewan Harrison, David K. Jackson, Ian Johnston, Dominic Kwiatkowski, Cordelia Langford, John Sillitoe on behalf of the Wellcome Sanger Institute COVID-19 Surveillance Team
EPI_ISL_778257	Lighthouse Lab in Cambridge	Wellcome Sanger Institute for the COVID-19 Genomics UK (COG-UK) Consortium	Rob Howes, The Lighthouse Lab in Cambridge and Alex Alderton, Roberto Amato, Sonia Goncalves, Ewan Harrison, David K. Jackson, Ian Johnston, Dominic Kwiatkowski, Cordelia Langford, John Sillitoe on behalf of the Wellcome Sanger Institute COVID-19 Surveillance Team
EPI_ISL_778258	Lighthouse Lab in Glasgow	Wellcome Sanger Institute for the COVID-19 Genomics UK (COG-UK) Consortium	Harper VanSteenhouse, Yumi Kasai, David Gray, Carol Clugston, Anna Dominiczak and Alex Alderton, Roberto Amato, Sonia Goncalves, Ewan Harrison, David K. Jackson, Ian Johnston, Dominic Kwiatkowski, Cordelia Langford, John Sillitoe on behalf of the Wellcome Sanger Institute COVID-19 Surveillance Team
EPI_ISL_778259	Lighthouse Lab in Alderley Park	Wellcome Sanger Institute for the COVID-19 Genomics UK (COG-UK) Consortium	Jacquelyn Wynn, Mairead Hyland, The Lighthouse Lab in Alderley Park and Alex Alderton, Roberto Amato, Sonia Goncalves, Ewan Harrison, David K. Jackson, Ian Johnston, Dominic Kwiatkowski, Cordelia Langford, John Sillitoe on behalf of the Wellcome Sanger Institute COVID-19 Surveillance Team
EPI_ISL_778260	Lighthouse Lab in Glasgow	Wellcome Sanger Institute for the COVID-19 Genomics UK (COG-UK) Consortium	Harper VanSteenhouse, Yumi Kasai, David Gray, Carol Clugston, Anna Dominiczak and Alex Alderton, Roberto Amato, Sonia Goncalves, Ewan Harrison, David K. Jackson, Ian Johnston, Dominic Kwiatkowski, Cordelia Langford, John Sillitoe on behalf of the Wellcome Sanger Institute COVID-19 Surveillance Team
EPI_ISL_778261	Lighthouse Lab in Alderley Park	Wellcome Sanger Institute for the COVID-19 Genomics UK (COG-UK) Consortium	Jacquelyn Wynn, Mairead Hyland, The Lighthouse Lab in Alderley Park and Alex Alderton, Roberto Amato, Sonia Goncalves, Ewan Harrison, David K. Jackson, Ian Johnston, Dominic Kwiatkowski, Cordelia Langford, John Sillitoe on behalf of the Wellcome Sanger Institute COVID-19 Surveillance Team
EPI_ISL_778262	Lighthouse Lab in Cambridge	Wellcome Sanger Institute for the COVID-19 Genomics UK (COG-UK) Consortium	Rob Howes, The Lighthouse Lab in Cambridge and Alex Alderton, Roberto Amato, Sonia Goncalves, Ewan Harrison, David K. Jackson, Ian Johnston, Dominic Kwiatkowski, Cordelia Langford, John Sillitoe on behalf of the Wellcome Sanger Institute COVID-19 Surveillance Team
EPI_ISL_778263	Lighthouse Lab in Glasgow	Wellcome Sanger Institute for the COVID-19 Genomics UK (COG-UK) Consortium	Harper VanSteenhouse, Yumi Kasai, David Gray, Carol Clugston, Anna Dominiczak and Alex Alderton, Roberto Amato, Sonia Goncalves, Ewan Harrison, David K. Jackson, Ian Johnston, Dominic Kwiatkowski, Cordelia Langford, John Sillitoe on behalf of the Wellcome Sanger Institute COVID-19 Surveillance Team
EPI_ISL_778264, EPI_ISL_778266	Lighthouse Lab in Alderley Park	Wellcome Sanger Institute for the COVID-19 Genomics UK (COG-UK) Consortium	Jacquelyn Wynn, Mairead Hyland, The Lighthouse Lab in Alderley Park and Alex Alderton, Roberto Amato, Sonia Goncalves, Ewan Harrison, David K. Jackson, Ian Johnston, Dominic Kwiatkowski, Cordelia Langford, John Sillitoe on behalf of the Wellcome Sanger Institute COVID-19 Surveillance Team
EPI_ISL_778271, EPI_ISL_778273, EPI_ISL_778277, EPI_ISL_778278	Lighthouse Lab in Cambridge	Wellcome Sanger Institute for the COVID-19 Genomics UK (COG-UK) Consortium	Rob Howes, The Lighthouse Lab in Cambridge and Alex Alderton, Roberto Amato, Sonia Goncalves, Ewan Harrison, David K. Jackson, Ian Johnston, Dominic Kwiatkowski, Cordelia Langford, John Sillitoe on behalf of the Wellcome Sanger Institute COVID-19 Surveillance Team
EPI_ISL_778281	Lighthouse Lab in Glasgow	Wellcome Sanger Institute for the COVID-19 Genomics UK (COG-UK) Consortium	Harper VanSteenhouse, Yumi Kasai, David Gray, Carol Clugston, Anna Dominiczak and Alex Alderton, Roberto Amato, Sonia Goncalves, Ewan Harrison, David K. Jackson, Ian Johnston, Dominic Kwiatkowski, Cordelia Langford, John Sillitoe on behalf of the Wellcome Sanger Institute COVID-19 Surveillance Team
EPI_ISL_778282	Lighthouse Lab in Alderley Park	Wellcome Sanger Institute for the COVID-19 Genomics UK (COG-UK) Consortium	Jacquelyn Wynn, Mairead Hyland, The Lighthouse Lab in Alderley Park and Alex Alderton, Roberto Amato, Sonia Goncalves, Ewan Harrison, David K. Jackson, Ian Johnston, Dominic Kwiatkowski, Cordelia Langford, John Sillitoe on behalf of the Wellcome Sanger Institute COVID-19 Surveillance Team
EPI_ISL_778284, EPI_ISL_778285	Lighthouse Lab in Cambridge	Wellcome Sanger Institute for the COVID-19 Genomics UK (COG-UK) Consortium	Rob Howes, The Lighthouse Lab in Cambridge and Alex Alderton, Roberto Amato, Sonia Goncalves, Ewan Harrison, David K. Jackson, Ian Johnston, Dominic Kwiatkowski, Cordelia Langford, John Sillitoe on behalf of the Wellcome Sanger Institute COVID-19 Surveillance Team
EPI_ISL_778287, EPI_ISL_778288	Lighthouse Lab in Alderley Park	Wellcome Sanger Institute for the COVID-19 Genomics UK (COG-UK) Consortium	Jacquelyn Wynn, Mairead Hyland, The Lighthouse Lab in Alderley Park and Alex Alderton, Roberto Amato, Sonia Goncalves, Ewan Harrison, David K. Jackson, Ian Johnston, Dominic Kwiatkowski, Cordelia Langford, John Sillitoe on behalf of the Wellcome Sanger Institute COVID-19 Surveillance Team
EPI_ISL_778289	Lighthouse Lab in Glasgow	Wellcome Sanger Institute for the COVID-19 Genomics UK (COG-UK) Consortium	Harper VanSteenhouse, Yumi Kasai, David Gray, Carol Clugston, Anna Dominiczak and Alex Alderton, Roberto Amato, Sonia Goncalves, Ewan Harrison, David K. Jackson, Ian Johnston, Dominic Kwiatkowski, Cordelia Langford, John Sillitoe on behalf of the Wellcome Sanger Institute COVID-19 Surveillance Team
EPI_ISL_778292	Lighthouse Lab in Alderley Park	Wellcome Sanger Institute for the COVID-19 Genomics UK (COG-UK) Consortium	Jacquelyn Wynn, Mairead Hyland, The Lighthouse Lab in Alderley Park and Alex Alderton, Roberto Amato, Sonia Goncalves, Ewan Harrison, David K. Jackson, Ian Johnston, Dominic Kwiatkowski, Cordelia Langford, John Sillitoe on behalf of the Wellcome Sanger Institute COVID-19 Surveillance Team
EPI_ISL_778293, EPI_ISL_778294	Lighthouse Lab in Cambridge	Wellcome Sanger Institute for the COVID-19 Genomics UK (COG-UK) Consortium	Rob Howes, The Lighthouse Lab in Cambridge and Alex Alderton, Roberto Amato, Sonia Goncalves, Ewan Harrison, David K. Jackson, Ian Johnston, Dominic Kwiatkowski, Cordelia Langford, John Sillitoe on behalf of the Wellcome Sanger Institute COVID-19 Surveillance Team
EPI_ISL_778295	Lighthouse Lab in Alderley Park	Wellcome Sanger Institute for the COVID-19 Genomics UK (COG-UK) Consortium	Jacquelyn Wynn, Mairead Hyland, The Lighthouse Lab in Alderley Park and Alex Alderton, Roberto Amato, Sonia Goncalves, Ewan Harrison, David K. Jackson, Ian Johnston, Dominic Kwiatkowski, Cordelia Langford, John Sillitoe on behalf of the Wellcome Sanger Institute COVID-19 Surveillance Team
EPI_ISL_778296	Lighthouse Lab in Cambridge	Wellcome Sanger Institute for the COVID-19 Genomics UK (COG-UK) Consortium	Rob Howes, The Lighthouse Lab in Cambridge and Alex Alderton, Roberto Amato, Sonia Goncalves, Ewan Harrison, David K. Jackson, Ian Johnston, Dominic Kwiatkowski, Cordelia Langford, John Sillitoe on behalf of the Wellcome Sanger Institute COVID-19 Surveillance Team
EPI_ISL_778297	Lighthouse Lab in Glasgow	Wellcome Sanger Institute for the COVID-19 Genomics UK	Harper VanSteenhouse, Yumi Kasai, David Gray, Carol Clugston, Anna Dominiczak and Alex Alderton, Roberto Amato, Sonia Goncalves, Ewan Harrison,

		(COG-UK) Consortium	David K. Jackson, Ian Johnston, Dominic Kwiatkowski, Cordelia Langford, John Sillitoe on behalf of the Wellcome Sanger Institute COVID-19 Surveillance Team
EPI_ISL_778298	Lighthouse Lab in Milton Keynes	Wellcome Sanger Institute for the COVID-19 Genomics UK (COG-UK) Consortium	The Lighthouse Lab in Milton Keynes and Alex Alderton, Roberto Amato, Sonia Goncalves, Ewan Harrison, David K. Jackson, Ian Johnston, Dominic Kwiatkowski, Cordelia Langford, John Sillitoe on behalf of the Wellcome Sanger Institute COVID-19 Surveillance Team
EPI_ISL_778299, EPI_ISL_778301, EPI_ISL_778302, EPI_ISL_778303	Lighthouse Lab in Glasgow	Wellcome Sanger Institute for the COVID-19 Genomics UK (COG-UK) Consortium	Harper VanSteenhouse, Yumi Kasai, David Gray, Carol Clugston, Anna Dominiczak and Alex Alderton, Roberto Amato, Sonia Goncalves, Ewan Harrison, David K. Jackson, Ian Johnston, Dominic Kwiatkowski, Cordelia Langford, John Sillitoe on behalf of the Wellcome Sanger Institute COVID-19 Surveillance Team
EPI_ISL_778304, EPI_ISL_778307, EPI_ISL_778308, EPI_ISL_778310	Lighthouse Lab in Milton Keynes	Wellcome Sanger Institute for the COVID-19 Genomics UK (COG-UK) Consortium	The Lighthouse Lab in Milton Keynes and Alex Alderton, Roberto Amato, Sonia Goncalves, Ewan Harrison, David K. Jackson, Ian Johnston, Dominic Kwiatkowski, Cordelia Langford, John Sillitoe on behalf of the Wellcome Sanger Institute COVID-19 Surveillance Team
EPI_ISL_778311	Lighthouse Lab in Alderley Park	Wellcome Sanger Institute for the COVID-19 Genomics UK (COG-UK) Consortium	Jacquelyn Wynn, Mairead Hyland, The Lighthouse Lab in Alderley Park and Alex Alderton, Roberto Amato, Sonia Goncalves, Ewan Harrison, David K. Jackson, Ian Johnston, Dominic Kwiatkowski, Cordelia Langford, John Sillitoe on behalf of the Wellcome Sanger Institute COVID-19 Surveillance Team
EPI_ISL_778313, EPI_ISL_778314, EPI_ISL_778316, EPI_ISL_778317, EPI_ISL_778318, EPI_ISL_778319, EPI_ISL_778320, EPI_ISL_778321	Lighthouse Lab in Milton Keynes	Wellcome Sanger Institute for the COVID-19 Genomics UK (COG-UK) Consortium	The Lighthouse Lab in Milton Keynes and Alex Alderton, Roberto Amato, Sonia Goncalves, Ewan Harrison, David K. Jackson, Ian Johnston, Dominic Kwiatkowski, Cordelia Langford, John Sillitoe on behalf of the Wellcome Sanger Institute COVID-19 Surveillance Team
EPI_ISL_778322, EPI_ISL_778325	Lighthouse Lab in Glasgow	Wellcome Sanger Institute for the COVID-19 Genomics UK (COG-UK) Consortium	Harper VanSteenhouse, Yumi Kasai, David Gray, Carol Clugston, Anna Dominiczak and Alex Alderton, Roberto Amato, Sonia Goncalves, Ewan Harrison, David K. Jackson, Ian Johnston, Dominic Kwiatkowski, Cordelia Langford, John Sillitoe on behalf of the Wellcome Sanger Institute COVID-19 Surveillance Team
EPI_ISL_778326	Lighthouse Lab in Milton Keynes	Wellcome Sanger Institute for the COVID-19 Genomics UK (COG-UK) Consortium	The Lighthouse Lab in Milton Keynes and Alex Alderton, Roberto Amato, Sonia Goncalves, Ewan Harrison, David K. Jackson, Ian Johnston, Dominic Kwiatkowski, Cordelia Langford, John Sillitoe on behalf of the Wellcome Sanger Institute COVID-19 Surveillance Team
EPI_ISL_778327, EPI_ISL_778328	Lighthouse Lab in Alderley Park	Wellcome Sanger Institute for the COVID-19 Genomics UK (COG-UK) Consortium	Jacquelyn Wynn, Mairead Hyland, The Lighthouse Lab in Alderley Park and Alex Alderton, Roberto Amato, Sonia Goncalves, Ewan Harrison, David K. Jackson, Ian Johnston, Dominic Kwiatkowski, Cordelia Langford, John Sillitoe on behalf of the Wellcome Sanger Institute COVID-19 Surveillance Team
EPI_ISL_778329	Lighthouse Lab in Milton Keynes	Wellcome Sanger Institute for the COVID-19 Genomics UK (COG-UK) Consortium	The Lighthouse Lab in Milton Keynes and Alex Alderton, Roberto Amato, Sonia Goncalves, Ewan Harrison, David K. Jackson, Ian Johnston, Dominic Kwiatkowski, Cordelia Langford, John Sillitoe on behalf of the Wellcome Sanger Institute COVID-19 Surveillance Team
EPI_ISL_778331	Lighthouse Lab in Alderley Park	Wellcome Sanger Institute for the COVID-19 Genomics UK (COG-UK) Consortium	Jacquelyn Wynn, Mairead Hyland, The Lighthouse Lab in Alderley Park and Alex Alderton, Roberto Amato, Sonia Goncalves, Ewan Harrison, David K. Jackson, Ian Johnston, Dominic Kwiatkowski, Cordelia Langford, John Sillitoe on behalf of the Wellcome Sanger Institute COVID-19 Surveillance Team
EPI_ISL_778332	Lighthouse Lab in Glasgow	Wellcome Sanger Institute for the COVID-19 Genomics UK (COG-UK) Consortium	Harper VanSteenhouse, Yumi Kasai, David Gray, Carol Clugston, Anna Dominiczak and Alex Alderton, Roberto Amato, Sonia Goncalves, Ewan Harrison, David K. Jackson, Ian Johnston, Dominic Kwiatkowski, Cordelia Langford, John Sillitoe on behalf of the Wellcome Sanger Institute COVID-19 Surveillance Team
EPI_ISL_778333	Lighthouse Lab in Alderley Park	Wellcome Sanger Institute for the COVID-19 Genomics UK (COG-UK) Consortium	Jacquelyn Wynn, Mairead Hyland, The Lighthouse Lab in Alderley Park and Alex Alderton, Roberto Amato, Sonia Goncalves, Ewan Harrison, David K. Jackson, Ian Johnston, Dominic Kwiatkowski, Cordelia Langford, John Sillitoe on behalf of the Wellcome Sanger Institute COVID-19 Surveillance Team
EPI_ISL_778334	Lighthouse Lab in Glasgow	Wellcome Sanger Institute for the COVID-19 Genomics UK (COG-UK) Consortium	Harper VanSteenhouse, Yumi Kasai, David Gray, Carol Clugston, Anna Dominiczak and Alex Alderton, Roberto Amato, Sonia Goncalves, Ewan Harrison, David K. Jackson, Ian Johnston, Dominic Kwiatkowski, Cordelia Langford, John Sillitoe on behalf of the Wellcome Sanger Institute COVID-19 Surveillance Team
EPI_ISL_778335	Lighthouse Lab in Alderley Park	Wellcome Sanger Institute for the COVID-19 Genomics UK (COG-UK) Consortium	Jacquelyn Wynn, Mairead Hyland, The Lighthouse Lab in Alderley Park and Alex Alderton, Roberto Amato, Sonia Goncalves, Ewan Harrison, David K. Jackson, Ian Johnston, Dominic Kwiatkowski, Cordelia Langford, John Sillitoe on behalf of the Wellcome Sanger Institute COVID-19 Surveillance Team
EPI_ISL_778337	Lighthouse Lab in Glasgow	Wellcome Sanger Institute for the COVID-19 Genomics UK (COG-UK) Consortium	Harper VanSteenhouse, Yumi Kasai, David Gray, Carol Clugston, Anna Dominiczak and Alex Alderton, Roberto Amato, Sonia Goncalves, Ewan Harrison, David K. Jackson, Ian Johnston, Dominic Kwiatkowski, Cordelia Langford, John Sillitoe on behalf of the Wellcome Sanger Institute COVID-19 Surveillance Team
EPI_ISL_778339, EPI_ISL_778341, EPI_ISL_778344	Lighthouse Lab in Milton Keynes	Wellcome Sanger Institute for the COVID-19 Genomics UK (COG-UK) Consortium	The Lighthouse Lab in Milton Keynes and Alex Alderton, Roberto Amato, Sonia Goncalves, Ewan Harrison, David K. Jackson, Ian Johnston, Dominic Kwiatkowski, Cordelia Langford, John Sillitoe on behalf of the Wellcome Sanger Institute COVID-19 Surveillance Team
EPI_ISL_778345	Lighthouse Lab in Glasgow	Wellcome Sanger Institute for the COVID-19 Genomics UK (COG-UK) Consortium	Harper VanSteenhouse, Yumi Kasai, David Gray, Carol Clugston, Anna Dominiczak and Alex Alderton, Roberto Amato, Sonia Goncalves, Ewan Harrison, David K. Jackson, Ian Johnston, Dominic Kwiatkowski, Cordelia Langford, John Sillitoe on behalf of the Wellcome Sanger Institute COVID-19 Surveillance Team
[truncated: 7,612,901 more chars]
