# Supplementary material for: Strategy to Develop and Evaluate a Multiplex RT-ddPCR in Response to SARS-CoV-2 Genomic Evolution
Source: Curr Issues Mol Biol. 2021 Nov 6;43(3):1937–49. doi: 10.3390/cimb43030134 (PMC8928932; doi:10.3390/cimb43030134)
Supplement: Supplementary file 1 [file cimb-43-00134-s001.zip › Supplementary File S4.pdf]

Supplementary File S4: Table with SARS-*CoV*-2 *in silico* analysis and investigation in clinical samples and wastewater samples including Cq values from previous test and the material and methods used to determine the Cq.

Supplementary Table S1: Inclusivity results of ORF1a and RdRP_IP4 assays over time

	January – March 2020 [1]	June – December 2020 [2]	November 2020 – February 2021
**ORF1a**	100%	99.89%	99.87%
**RdRP_IP4**	100%	99.95%	99.92%

Supplementary Table S2: Number of false negatives of the ORF1a and RdRP_IP4 assays related to the B.1.1.7 and B.1.351 lineages over time.

	B.1.1.7			B.1.351		
	ORF1a	RdRP_IP4	Total	ORF1a	RdRP_IP4	Total
**November 2020**	0	0	18	/	/	0
**December 2020**	1	1	103	0	0	21
**January 2021**	6	22	7291	0	1	138
**February 2021**	7	21	26 199	0	0	134

Supplementary Table S3: Table with SARS-CoV-2 investigation in clinical samples and wastewater samples including Cq values from previous test and the material and methods used to determine the Cq.

Sample	RT-ddPCR		RT-qPCR		
	SARS-CoV-2 (ORF1a)	SARS-CoV-2 (RdRp_IP4)	N1	N2	E
**Wastewater sample 1**	+ (2.48 copies/µL)	+ (1.93 copies/µL)	+ (Cq=32.49)	+ (Cq=32.41)	+ (Cq=33.03)
**Wastewater sample 2**	+ (6.33 copies/µL)	+ (2.20 copies/µL)	+ (Cq=30.05)	+ (Cq=30.07)	+ (Cq=30.15)
**Wastewater sample 3**	+ (29.43 copies/µL)	+ (36.29 copies/µL)	+ (Cq=26.62)	+ (Cq=26.75)	+ (Cq=27.16)
**Clinical sample 1**	+ (2.75 copies/µL)	+ (2.75 copies/µL)	+ (Cq=37.12)		
**Clinical sample 2**	+ (26.13 copies/µL)	+ (32.18 copies/µL)	+ (Cq=31.73)		

**Clinical sample 3**	+ (88440 copies/µL)	+ (91080 copies/µL)	+ (Cq=21.4)		
**Clinical sample 4**	-	-	-	-	-
**Clinical sample 5**	-	-	-	-	-

The sample name and the kind of sample are given in addition to the results of the detection of SARS-CoV-2 using the ORF1a assay and the RdRp_IP4 assay. The presence or absence of PCR amplification is symbolized by "+" or "-" respectively. For each RT-ddPCR, the observed copies/µL was given between brackets.

Material and methods (wastewater samples)

Wastewater samples were collected as 24-hour composite samples at the inlet of three Belgian wastewater treatment plants (WWTP). The samples were transported to the lab at 4°C and analyzed within 24 hours upon arrival. The samples were processed as described by Medema et al [3]. Briefly, debris was pelleted from a homogenized subsample of 50 ml and the supernatant was concentrated using the centrifugal filter Centricon Plus-70 100 KDa (Merck, https://www.merckmillipore.com) to a volume ranging from 400 µL to 1500 µL. RNA was extracted from 200µL wastewater concentrate by using the Maxwell® RSC PureFood GMO and Authentication Kit (Promega, https://be.promega.com), according to the manufacturer's instructions and the Maxwell® Rapid Sample Concentrator (RSC) instrument. RNA was screened by three reverse transcription quantitative PCR (RT-qPCR) assays targeting the SARS-CoV-2 nucleocapsid (N) gene (N1 & N2) [4] and the envelope (E) gene [5]. No false positive results for other coronaviruses are expected for the primers and probes targeting the N1 and N2 genes based on the results of the CDC [4] and Gand et al [1]. The primers and probes that target the E gene can also detect other members of the Sarbecovirus subgenus. This target can be used for a first screening, but other targets using more specific primers and probe, in this case N1 and N2, should also be included to confirm the presence of SARS-CoV-2. All RT-qPCR assays were performed in a singleplex reaction using 5µL of RNA on an AriaMx (Agilent, https://www.agilent.com) using the TaqMan™ Fast Virus 1-Step Master Mix.

Material and methods (clinical samples)

The clinical samples were obtained from colleagues who have been infected or not by the virus and tested within Sciensano. They have given their consent/ethical approval for the samples to be used for research purposes. RNA is extracted from nasopharyngeal swabs. According the IndiMag® Pathogen kit handbook, 200µl of each sample was inactivated. All the components of the kit were kept at room temperature except the resuspended carrier RNA which is thawed just before the inactivation buffer preparation. 200µl of sample was mixed with 100µl VXL buffer, 400µl ACB buffer, 25µl magnetic beads and 5µl carrier RNA. This mix, that contains inactivated virus, was incubated for 5 minutes at room temperature and then transferred to extraction blocks. 20µl of proteinase K was added to the mix and the extraction was performed using the IndiMag48 instrument using the “Pathogen” protocol. The purified RNA was eluted in 50µl AVE buffer and tested for the presence of SARS-CoV-2 genome using the AgPath-ID™ One-Step RT-PCR Reagents kit (ThermoFisher). For SARS-CoV-2 detection, one set of primers/probe was used from available RT-qPCR assays, namely 2019-nCoV CDC EUA Authorized qPCR Probe Assay primer/probe mix from Integrated DNA Technologies (Supplementary Table S4). No false positive results for other coronaviruses are expected for the primers and probe targeting the N1 genes based on the results of the CDC [4] and Gand et al [1]. As an endogenous internal control, the gene encoding for Ribonuclease-P (RNASE_P) is amplified using a set of primers/probe developed in-house.

Supplementary Table S4: Primer and probe sets included in the multiplex RT-PCR diagnostic test

Primer/Probe	5’ → 3’ Sequence	Target	Concentration
**2019-nCoV_N1-F**	GACCCCAAAATCAGCGAAAT	Gene N	0.5 µM
**2019-nCoV_N1-R**	TCTGGTTACTGCCAGTTGAATCTG	Gene N	0.5 µM
**2019-nCoV_N1-P**	FAM-ACCCCGCATTACGTTTGGTGGACC-BHQ1	Gene N	0.125 µM
**2019-nCoV_N1-P**	FAM-ACCCCGCAT/ZEN/TACGTTTGGTGGACC-3IABkFQ	Gene N	0.125 µM
**RNASE_P-F**	AGATTTGGACCTGCGAGCG	Human endogenous Rnase_P	0.110 µM

**RNASE_P-R**	GAGCGGCTGTCTCCACAAGT	Human endogenous Rnase_P	0.110 µM
**RNASE_P-P**	HEX–TTCTGACCTGAAGGCTCTGCGCG–BHQ	Human endogenous Rnase_P	0.075 µM

For the 2019-nCoV EUA Authorized qPCR Probe Assay primer/probe mix, TaqMan® probes are labeled at the 5′-end with the reporter molecule 6-carboxyfluorescein (FAM) and with the quencher, Black Hole Quencher 1 (BHQ-1) (Biosearch Technologies, Inc., Novato, CA) at the 3′-end. TaqMan® probes can also be labeled at the 5′-end with the reporter molecule 6-carboxyfluorescein (FAM) and with a double quencher, ZEN™ Internal Quencher positioned between the ninth (9th) and tenth (10th) nucleotide base in the oligonucleotide sequence and Iowa Black® FQ (3IABkFQ) located at the 3'-end (Integrated DNA Technologies, Coralville, IA). A semi-quantitative reporting of the RT-qPCR results has been proposed by Sciensano [6] and Clinical Sample 1 (high Ct), Clinical Sample 2 (moderate Ct) and Clinical Sample 3 (low Ct) were considered weakly positive ($<10^3$ RNA copies/mL), positive (10^3-10^5 RNA copies/mL) and strongly positive (10^5-10^7 RNA copies/mL).

References

1. Gand, M.; Vanneste, K.; Thomas, I.; Van Gucht, S.; Capron, A.; Herman, P.; Roosens, N.H.C.; De Keersmaecker, S.C.J. Use of Whole Genome Sequencing Data for a First in Silico Specificity Evaluation of the RT-QPCR Assays Used for SARS-CoV-2 Detection. *International Journal of Molecular Sciences* **2020**, *21*, 5585–5585, doi:10.3390/ijms21155585.
2. Gand, M.; Vanneste, K.; Thomas, I.; Van Gucht, S.; Capron, A.; Herman, P.; Roosens, N.H.C.; De Keersmaecker, S.C.J. Deepening of In Silico Evaluation of SARS-CoV-2 Detection RT-QPCR Assays in the Context of New Variants. *Genes* **2021**, *12*, 565–565, doi:10.3390/genes12040565.
3. Medema, G.; Heijnen, L.; Elsinga, G.; Italiaander, R.; Brouwer, A. Presence of SARS-Coronavirus-2 RNA in Sewage and Correlation with Reported COVID-19 Prevalence in the Early Stage of the Epidemic in The Netherlands. *Environmental Science & Technology Letters* **2020**, *7*, 511–516, doi:10.1021/acs.estlett.0c00357.
4. Centers for Disease Control and Prevention (CDC) *CDC 2019-Novel Coronavirus (2019-NCoV) Real-Time RT-PCR Diagnostic Panel*; 2020;
5. Corman, V.M.; Landt, O.; Kaiser, M.; Molenkamp, R.; Meijer, A.; Chu, D.K.; Bleicker, T.; Brünink, S.; Schneider, J.; Schmidt, M.L.; et al. Detection of 2019 Novel Coronavirus (2019-NCoV) by Real-Time RT-PCR. *Eurosurveillance* **2020**, *25*, doi:10.2807/1560-7917.ES.2020.25.3.2000045.
6. Sciensano *RAG INTERPRETATION AND REPORTING OF SARS-COV-2 PCR RESULTS*; 2020;
