# Supplementary material for: Strategy to Develop and Evaluate a Multiplex RT-ddPCR in Response to SARS-CoV-2 Genomic Evolution
Source: Curr Issues Mol Biol. 2021 Nov 6;43(3):1937–49. doi: 10.3390/cimb43030134 (PMC8928932; doi:10.3390/cimb43030134)

**Supplementary File S5: Calculation of $LOD_{95\%}$ on the POD curve for the sensitivity of the developed RT-ddPCR method for the ORF1a assay (A) and RdRp_IP4 assay (B).** The results of **Error! Reference source not found.** were used for the calculations using the Quodata web application [57].

## A. ORF1a assay

### Results

#### Plausibility Check

The plausibility check indicates no irregularities.

#### POD curve and $LOD_{95\%}$

The $LOD_{95\%}$ is 4.570 with a 95 % confidence interval of [2.746, 7.612].

The figure below summarises the results. The blue diamonds characterise the laboratory-specific RODs. The blue curve denotes the mean POD curve along with the corresponding 95 % confidence range highlighted as the grey band. The POD curve under ideal conditions is displayed as the black dashed curve.

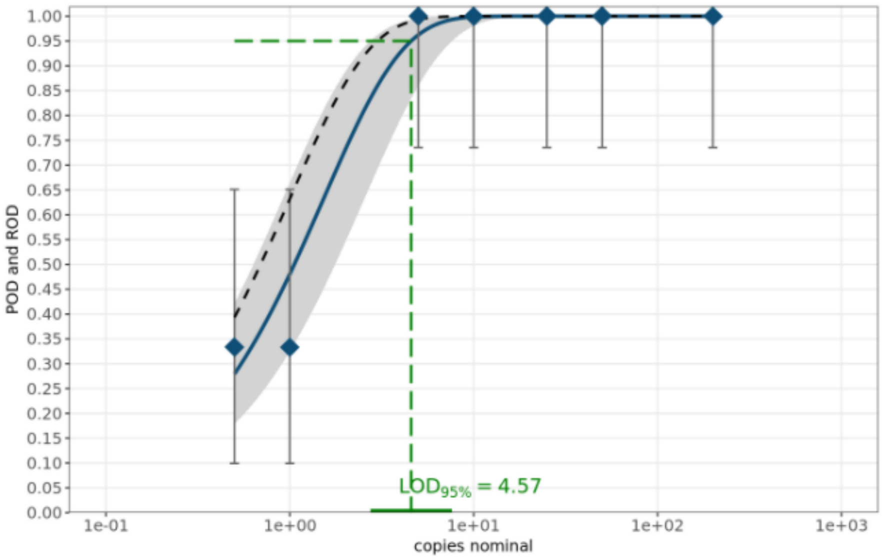

B. RdRp_IP4 assay

### Results

#### Plausibility Check

The calculated POD curve indicates sensitivity better than achievable according to the theoretical POD curve. It is most likely that the given nominal copies are incorrect. Please check it again.

Furthermore, the number of positive test results is significantly higher than expected at nominal copies of **0.5**. Please check the correctness of the serial dilution.

#### POD curve and $LOD_{95\%}$

The $LOD_{95\%}$ is 1.591 with a 95 % confidence interval of [0.950, 2.665].

The figure below summarises the results. The blue diamonds characterise the laboratory-specific RODs. The blue curve denotes the mean POD curve along with the corresponding 95 % confidence range highlighted as the grey band. The POD curve under ideal conditions is displayed as the black dashed curve.

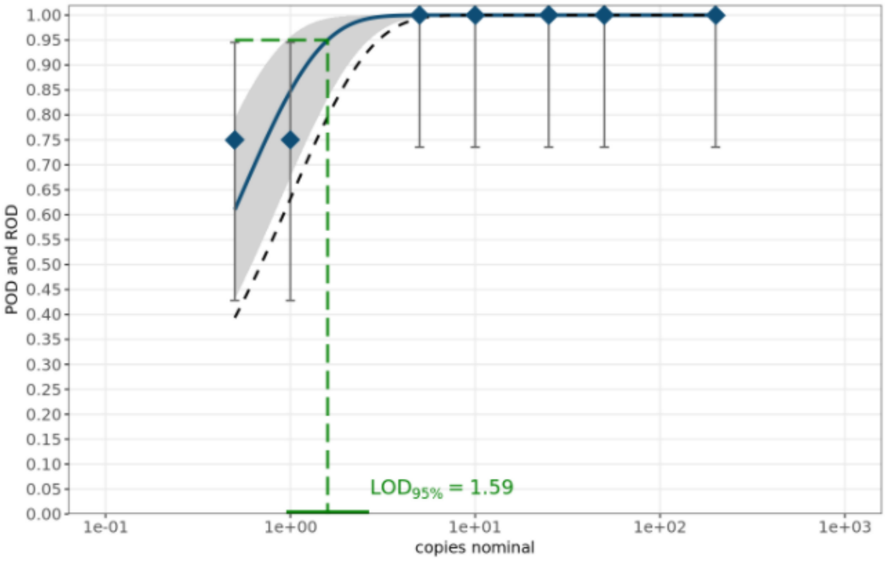

Supplement: Supplementary file 1 [file cimb-43-00134-s001.zip › Supplementary File S5.pdf]
