# Supplementary material for: Strategy to Develop and Evaluate a Multiplex RT-ddPCR in Response to SARS-CoV-2 Genomic Evolution
Source: Curr Issues Mol Biol. 2021 Nov 6;43(3):1937–49. doi: 10.3390/cimb43030134 (PMC8928932; doi:10.3390/cimb43030134)

Supplementary File S6: RT-ddPCR plots of the sensitivity assessment. The positive droplets (blue for ORF1a assay (A) and green for RdRp_IP4 assay (B)) are above the threshold (pink line) while the negative droplets (grey) are below the line.

A. ORF1a assay

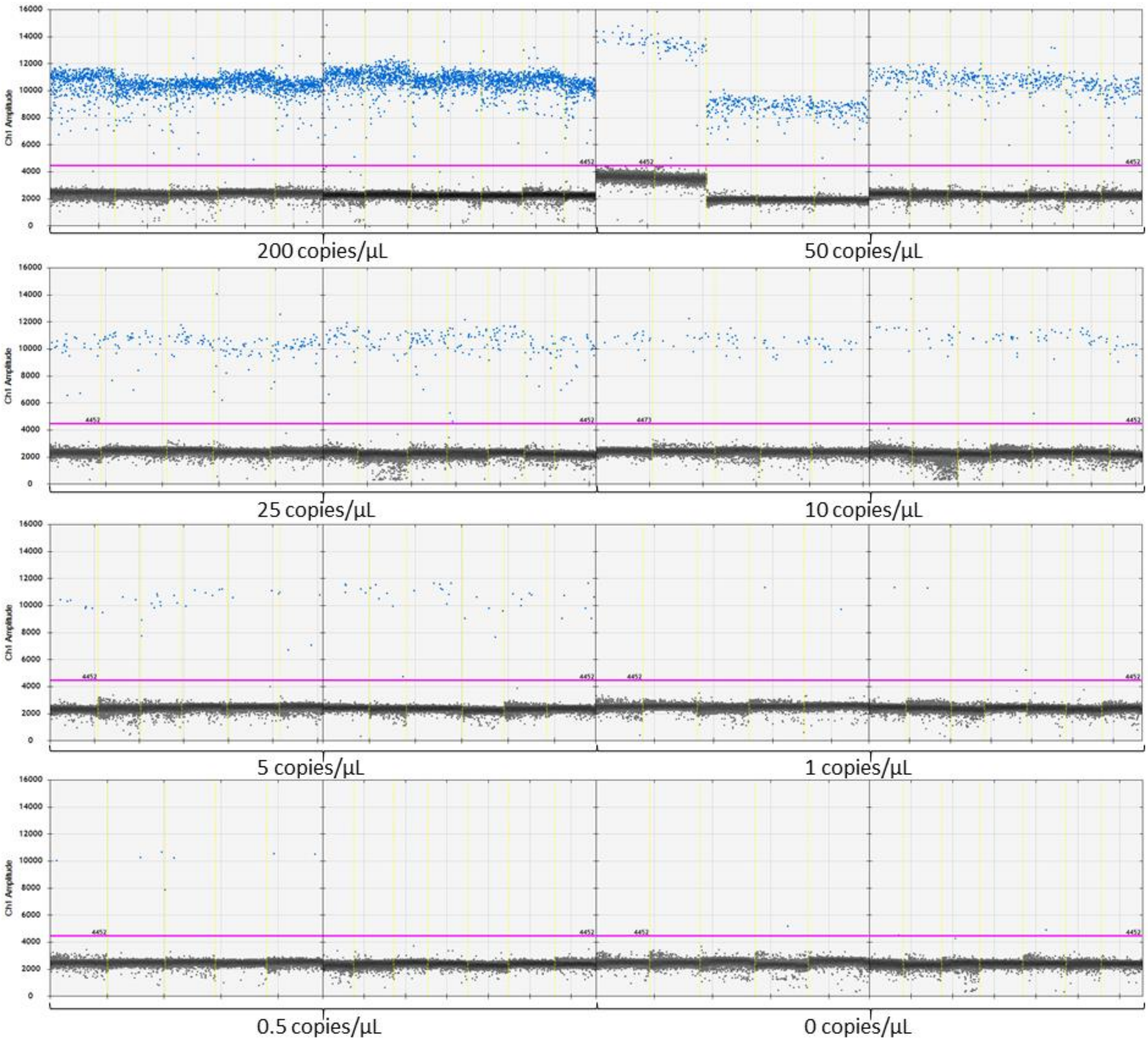

B. RdRp_IP4-assay

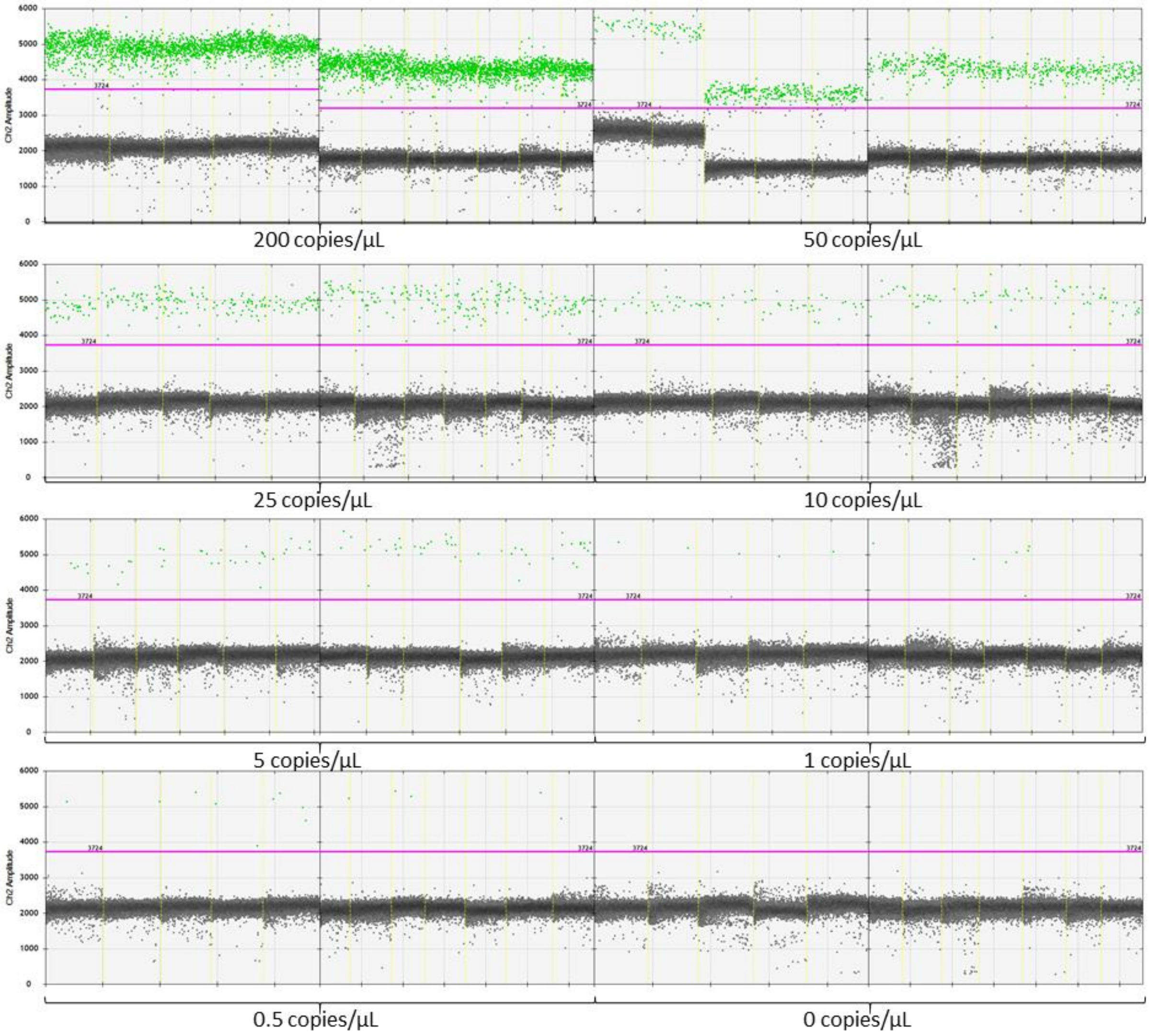

Supplement: Supplementary file 1 [file cimb-43-00134-s001.zip › Supplementary File S6.pdf]
